# Lung transcriptome of a COVID-19 patient and systems biology predictions suggest impaired surfactant production which may be druggable by surfactant therapy

**DOI:** 10.1038/s41598-020-76404-8

**Published:** 2020-11-10

**Authors:** Abul Bashar Mir Md. Khademul Islam, Md. Abdullah-Al-Kamran Khan

**Affiliations:** 1 Department of Genetic Engineering and Biotechnology, University of Dhaka, Dhaka, 1000 Bangladesh; 2 Department of Mathematics and Natural Sciences, BRAC University, Dhaka, Bangladesh

**Keywords:** Computational biology and bioinformatics, Microbiology, Systems biology

## Abstract

An incomplete understanding of the molecular mechanisms behind impairment of lung pathobiology by COVID-19 complicates its clinical management. In this study, we analyzed the gene expression pattern of cells obtained from biopsies of COVID-19-affected patient and compared to the effects observed in typical SARS-CoV-2 and SARS-CoV-infected cell-lines. We then compared gene expression patterns of COVID-19-affected lung tissues and SARS-CoV-2-infected cell-lines and mapped those to known lung-related molecular networks, including hypoxia induced responses, lung development, respiratory processes, cholesterol biosynthesis and surfactant metabolism; all of which are suspected to be downregulated following SARS-CoV-2 infection based on the observed symptomatic impairments. Network analyses suggest that SARS-CoV-2 infection might lead to acute lung injury in COVID-19 by affecting surfactant proteins and their regulators SPD, SPC, and TTF1 through NSP5 and NSP12; thrombosis regulators PLAT, and EGR1 by ORF8 and NSP12; and mitochondrial NDUFA10, NDUFAF5, and SAMM50 through NSP12. Furthermore, hypoxia response through HIF-1 signaling might also be targeted by SARS-CoV-2 proteins. Drug enrichment analysis of dysregulated genes has allowed us to propose novel therapies, including lung surfactants, respiratory stimulants, sargramostim, and oseltamivir. Our study presents a distinct mechanism of probable virus induced lung damage apart from cytokine storm.

## Introduction

The recent Coronavirus Disease (COVID-19) pandemic has affected approximately 30 million people across 212 countries, and territories and the number of active cases is still on the rise (on 18 September, 2020)^1^. At the time of this article, approximately 4% of the infected population has suffered death^1^, and the fatality rate is continuously increasing due to the lack of detailed knowledge of the molecular mechanism of Severe Acute Respiratory Syndrome Coronavirus 2 (SARS-CoV-2) infection and proper-targeted therapeutic approaches against it.

SARS-CoV-2 is an enveloped RNA virus, which contains single-stranded positive-sense RNA and belongs to the betacoronavirus genus of coronavirus^2^. It has 11 protein coding genes encompassing its ~ 29.9 Kb genome^3^. About 90% genomic identity was observed between SARS-CoV-2 and bat-derived SARS-like coronavirus, while SARS-CoV-2 genome is ~ 79% and ~ 50% identical with that of Severe Acute Respiratory Syndrome Coronavirus (SARS-CoV) and Middle East Respiratory Syndrome related Coronavirus (MERS-CoV), respectively^2,4,5^. Lu et al.^2^ showed the considerable differences between SARS-CoV-2 and SARS-CoV genomes, including 380 amino acids substitution, ORF8a deletion, ORF8b elongation, and ORF3b truncation^2^. Despite their identical genomic features, SARS-CoV-2 presents unique clinical and pathophysiological features, such as prolonged incubation period^6^, and latency inside the host^7^; which complicate its clinical management.

Based on the clinical exhibitions of COVID-19, most of the mild to critically affected patients show respiratory complications including moderate to severe pneumonia, which can further progress into acute respiratory distress syndrome (ARDS), sepsis, and multiple organ dysfunction (MOD) in severely ill patients^8^. Most of these clinical symptoms are associated with respiratory system, specifically the lungs^9^ resulting in the depleted lung functionality. Complications in other systems such as the cardiovascular and nervous systems were also reported^10,11^. Recently, cases of pulmonary embolism in the lungs of COVID-19 patients have been reported^12^.

In SARS-CoV and MERS-CoV infections, increased level of pro-inflammatory cytokines were evident^13^, which in turn increased the activation and recruitment of inflammatory cells into the lung tissues, facilitating acute lung injury^14^. Similarly, increased levels of many pro-inflammatory cytokines were also detected in moderate-to-critically affected COVID-19 patients^15^, leading to respiratory failure from ARDS. However, the complex interplays between pro-inflammatory and anti-inflammatory cytokines have not been completely illustrated. Apart from the cytokine storm, other factors such as host innate immunity, autoimmunity against the pulmonary epithelial and endothelial cells, and host genetic and epigenetic factors also play important roles in the pathogenesis of SARS-CoV infection^16,17^. Moreover, the multifaceted host-virus interactions are also found to be a key player in the pathogenesis reported for other coronavirus infections^18^.

Previously, transcriptional responses in COVID-19 were experimentally recorded using various in vitro and in vivo approaches such as cell lines, animal models, and SARS-CoV-2-infected lungs^19^, Nasopharyngeal (NP) swabs^20^, and bronchoalveolar lavage fluid of COVID-19 patients^21^. However, SARS-CoV-2-mediated deregulation of the lung transcriptome and its potential implications in pathogenesis of acute lung failure remained elusive. Therefore, designing of prospective therapeutics for the clinical management of the COVID-19 is still in its infancy. To this end, we analyzed publicly available transcriptome data from the lung biopsy of a COVID-19 patient and summarized the probably altered pathways in COVID-19. Furthermore, the probable roles of SARS-CoV-2 in these dysregulations, and resulting acute lung damage, were also investigated.

## Results

### Antiviral immune responses and organ specific functions are dysregulated in lungs

During respiratory virus infections, many host pathways are fine-tuned to battle against the invading pathogen; on the other side, the infecting viruses also try to hijack and modulate host pathways for immune evasion and survival inside the host^22^. These complex interactions lead to the disease complexity, causing several critical pathophysiological conditions in host’s respiratory system^22^. To explore which particular biological processes/pathways are dysregulated in SARS-CoV-2 infection, we first identified the dysregulated-genes in both SARS-CoV and SARS-CoV-2 infections and performed comparative functional enrichment analyses^23^.

We detected 3031 (2408 upregulated and 623 downregulated), 142 (91 upregulated and 51 downregulated, and 6714 (2476 upregulated and 4238 downregulated) dysregulated genes from SARS-CoV infected 2B4 cells, SARS-CoV-2-infected NHBE cells, and lung biopsy of COVID-19 patient, respectively (Supplementary Fig. 1). We observed a wide array of differentially expressed genes in SARS-CoV-2-infected lung whose expression profiles differed from those recorded in SARS-CoV-2-infected NHBE cells and SARS-CoV-infected 2B4 cells (Supplementary Fig. 1).

**Figure 1:**
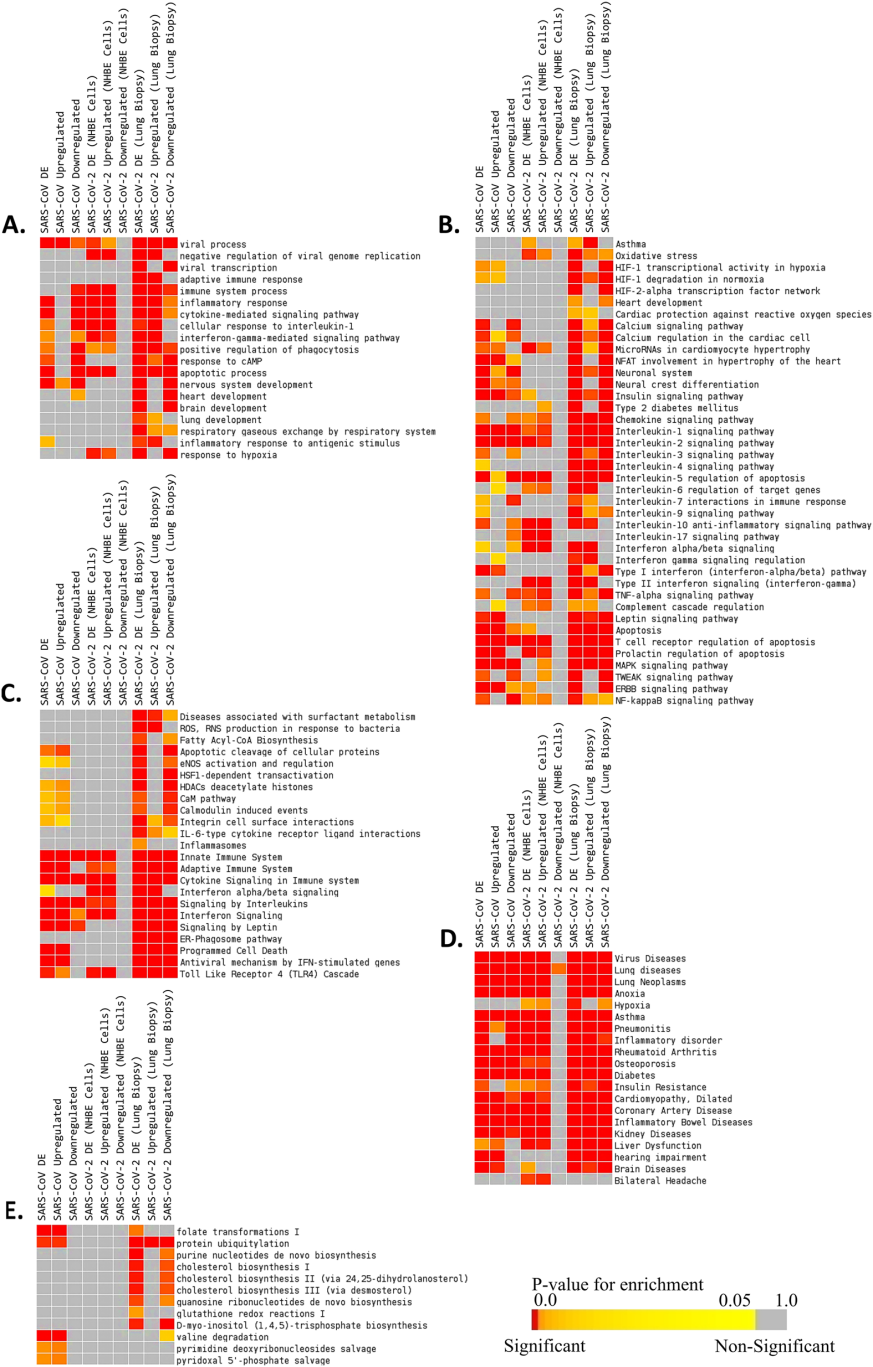
Enrichment analysis and comparison between dysregulated genes in SARS-CoV, SARS-CoV-2 (NHBE cells) and SARS-CoV-2 (Lung biopsy) infections using (**A**) GOBP^41^ module, (**B**) Bioplanet pathway^24^ module, (**C)** Reactome pathway^28^ module, (**D)** DisGeNet^25^ module, (**E)** HumanCyc^108^ module. Selected significant terms are represented in heatmap. Significance of enrichment in terms of adjusted *p*-value (< 0.05) is represented in color-coded P-value scale for all heatmaps. Color towards red indicates higher significance and color towards yellow indicates less significance, while grey means non-significant. The column headers are indicating the dysregulation status of the genes used for the enrichment analysis (DE: differentially expressed/Upregulated/Downregulated), while the row labels are pointing the enriched terms.

As expected, enrichment analyses revealed biological processes related to antiviral inflammatory responses, and viral processes were overrepresented in all three infection models (Fig. 1A). Several biological processes, such as negative regulation of viral replication, immune system process, response to hypoxia, and heart development were only enriched in the SARS-CoV-2 infection models (Fig. 1A). However, few pivotal processes were uniquely enriched for the dysregulated genes from COVID-19-affected lung, namely viral transcription, adaptive immune response, brain development, lung development, and respiratory gaseous exchange by respiratory system (Fig. 1A).

Similarly, enrichment using the ‘Bioplanet’^24^ module suggests host antiviral immune responses through various inflammatory cytokine signaling pathways, apoptosis, and interferon-I signaling in all of these infection models (Fig. 1B,C). While, HIF-1 signaling, heart development, asthma, and type-II interferon signaling pathways were found in both SARS-CoV-2 infection data (Fig. 1B, Supplementary Fig. 2). Intriguingly, some pathways, such as disease associated with surfactant metabolism, ROS/RNS production, fatty acyl-CoA biosynthesis, ER-phagosome pathway, and inflammasomes, were only found in the SARS-CoV-2-infected lung (Fig. 1C, Supplementary Fig. 2). Functional enrichment using the ‘DisGeNet’^25^ module revealed that the dysregulated genes of SARS-CoV and SARS-CoV-2 infections are also involved in other diseases, namely viral disease, lung diseases, asthma, pneumonitis, and hypoxia (Fig. 1D, Supplementary Fig. 2). Interestingly, several cholesterol biosynthesis pathways were found to be dysregulated only in the lung biopsy of COVID-19 patient (Fig. 1E, Supplementary Fig. 2). As cholesterol in lung plays important roles in maintaining normal lung physiology and protection against many diseases^26^, downregulation of these indicates possible association with lung-related comorbidities of COVID-19 patients.

**Figure 2:**
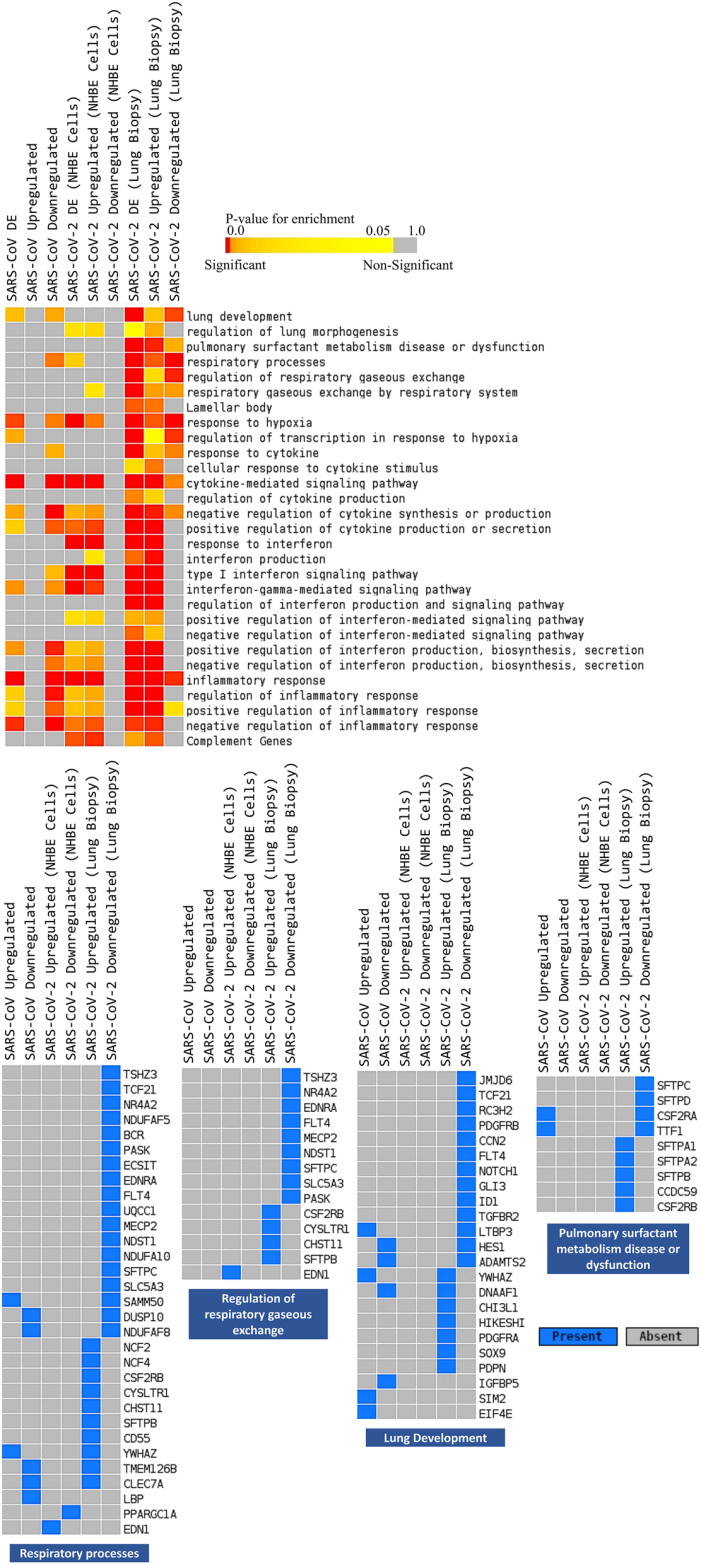
Enrichment analysis and comparison between dysregulated genes and the genes of some selected processes in SARS-CoV, SARS-CoV-2 (NHBE cells) and SARS-CoV-2 (Lung biopsy) infections using combined module. Selected significant terms are represented in heatmap in upper panel. Color schemes are similar as Fig. 1. Lower panel heatmaps presents enriched genes for some selected terms from upper panel enrichment analysis. For individual processes, blue means presence (significantly differentially expressed gene) while grey means absence (not significantly differentially expressed genes for this module for this experimental condition). The column headers are indicating the dysregulation status of the genes used for the enrichment analysis (DE: differentially expressed, Upregulated and Downregulated), while the row labels are pointing the enriched terms.

These enrichment analyses suggest that several pathways related to lung’s function are likely dysregulated directly or indirectly by the infecting SARS-CoV-2 virus. While hunting for more definitive clues on which particular processes are being modulated during SARS-CoV-2 infection, we again performed enrichment analysis with our in-house combined module (Supplementary file 4). This enrichment analysis revealed several important lung-function-related processes only for the dysregulated genes from the COVID-19 patient’s lung. These includued, lung development, pulmonary surfactant metabolism disease or dysfunction, respiratory processes, regulation of respiratory gaseous exchange, and some antiviral responses (Fig. 2, Supplementary Fig. 3). We also exported a list of significantly enriched terms in color-coded heatmaps. Intriguingly, while equating the expression of these enriched genes, we discovered that genes of these key lung-related processes are significantly altered in SARS-CoV-2-infected lungs compared to the SARS-CoV-2-infected NHBE cells and SARS-CoV infection model (Supplementary Fig. 4).

### Genes in lung surfactant metabolism pathway are dysregulated in COVID-19 patient’s lung

Pulmonary surfactant proteins play an important role in maintaining the surface tension necessary for efficient gaseous exchange at the air–liquid interface in the alveoli, and can also modulate functions of lung’s innate immune cells to eliminate pathogens^27^. Additionally, they have been shown to impede inflammatory responses and the clearance of apoptotic cells in lungs following viral infection^27^. Taking cue from the results of previous section, it was apparent that surfactant metabolism could be a potential target of viral modulation. Therefore, we sought to decipher the probable routes of SARS-CoV-2-induced dysregulations of this pathway and its downstream signaling. To attain this, we mapped the differentially expressed genes into this pathway using the Reactome pathway browser^28^. Next, we scrutinized the mechanisms of this pathway to elucidate the probable alterations happening in COVID-19-affected lung.

**Figure 3:**
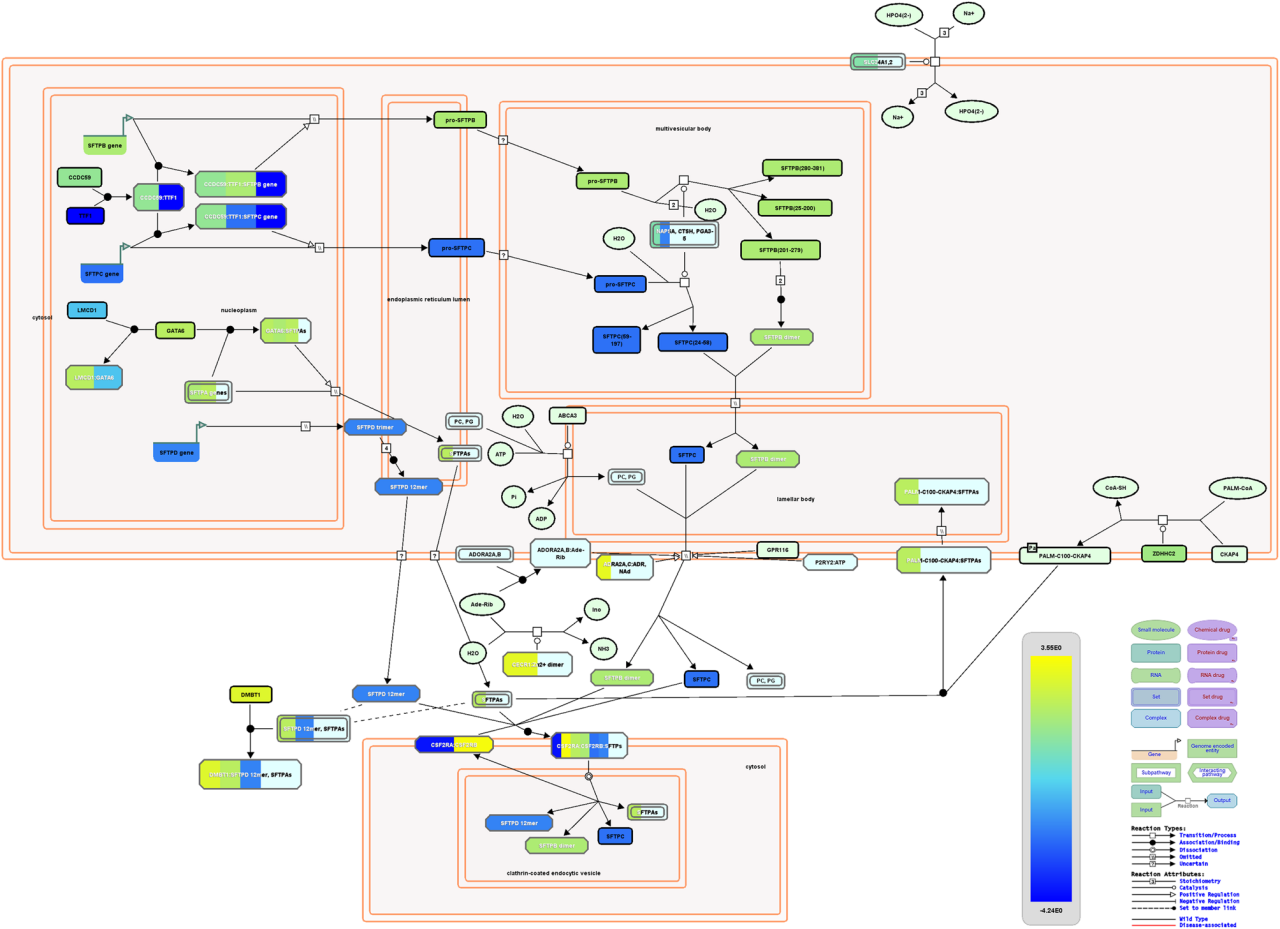
Schematic representation of lung surfactant metabolism pathway from Reactome pathway database^28^. Color towards yellow indicates upregulation and while blue indicates downregulation.

In normal lung, TTF1-CCDC59 complex can transactivate *SFTPB* and *SFTPC* gene expression, which play an important role by regulating the alveolar surface tension^29^. But in COVID-19-affected lung, *TTF1* and *SFTPC* genes were found to be downregulated, whereas *SFTPB* was upregulated (Fig. 3). GATA6 transcription factor promotes the transcription of *SFTPA* gene^30^ which is involved in immune and inflammatory responses, and lowers the surface tension in the alveoli^31^. Both *GATA6* and *SFTPA* genes were upregulated in SARS-CoV-2-infected lung, while the GATA6 antagonist *LMCD1* was downregulated (Fig. 3). The CSF2RA-CSF2RB complex can bind GM-CSF to induce activation of macrophages^32^ and helps in the degradation of STFPs in the alveolar macrophages^33^. We found *CFSF2RA* downregulated and *CSF2RB* upregulated in the lung of the COVID-19-affected patient (Fig. 3). Pro-SFTPB and Pro-SFTPC are cleaved by NAPSA, CTSH, and PGA3-5 to produce active SFTPB and SFTPC^34,35^. Surprisingly, NAPSA was dysregulated in the COVID-19-affected lung (Fig. 3). Overall, the transcription of surfactant genes, production of active surfactant proteins, and their turnover may be dysregulated in the COVID-19 patient’s lung, which could have resulted in the severely lethal disease complications. As these mechanisms are found to be altered upon SARS-CoV-2 infections, the virus may be positively facilitating these anomalies.

### Host proteins which interact with the virus are involved in different respiratory-function-related pathways and diseases

In previously reported human coronavirus infections, SARS and MERS coronaviruses are often found to commandeer host machineries, suppressing host immune responses and other important biological processes for their continued existence within the infected cells^36^. We performed functional enrichment analyses using previously reported SARS-CoV and SARS-CoV-2 host factor proteins^37–39^ to illuminate the pathways which may be targeted by viral proteins, causing the lung injuries in COVID-19 patients.

**Figure 4:**
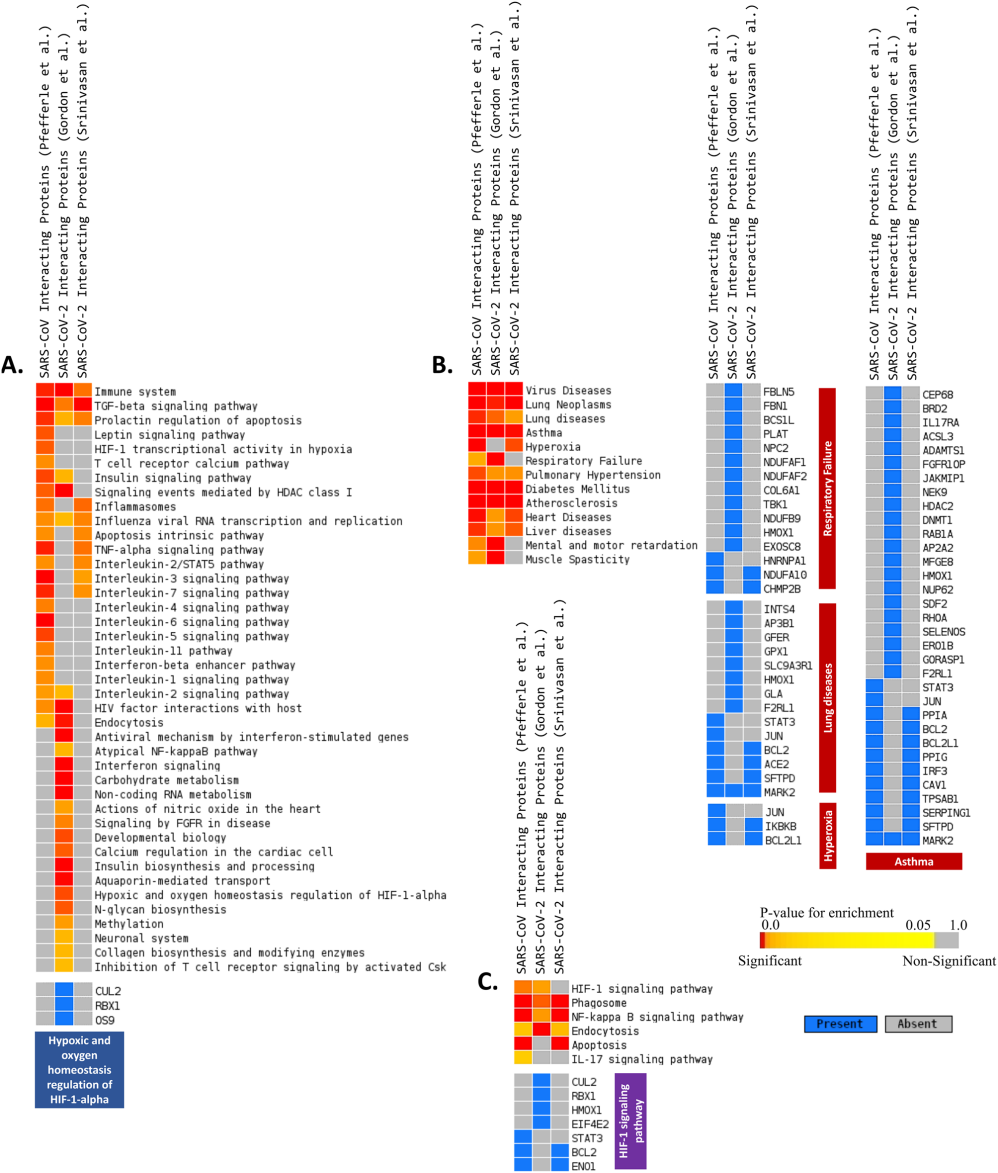
Enrichment analysis and comparison between host proteins interacting SARS-CoV proteins (Pfefferle et al.^38^), SARS-CoV-2 proteins (Srinivasan et al.^39^) and SARS-CoV-2 proteins (Gordon et al.^37^) and the genes of some selected processes using (**A**) Bioplanet pathway^24^ module, (**B**) DisGeNet^25^ module, (**C**) KEGG pathway^109^ module. Selected significant terms are represented in heatmap. Color schemes are similar as Figs. 1 and 2.

As anticipated, the enrichment analyses showed association of both SARS-CoV and SARS-CoV-2 infections with several immune signaling pathways, such as interleukin signaling, interferon signaling, apoptosis, and inflammasomes (Fig. 4A,C). However, this approach also revealed several vital pathways for respiratory function such as HIF-1 signaling, and hypoxic and oxygen homeostasis regulation of HIF-1 alpha (Fig. 4A,C). Using the DisGenNet module^25^, lung disease, asthma, hyperoxia, respiratory failure, and pulmonary hypertension were found to be enriched (Fig. 4B). These results enlightened that SARS-CoV-2 is likely utilizing its proteins to modulate normal lung’s physiological and immune responses, which we further explored by linking these viral-host protein–protein interactions (PPI) to our previously identified essential lung processes.

### SARS-CoV-2 proteins and host epigenetic regulators can modulate the functions of lung and other respiratory processes

Results from the previous sections rationalized that both the dysregulated genes in COVID-19-affected lung and SARS-CoV-2 interacting proteins are involved in several respiratory functions. Hence, we produced several functional networks of dysregulated genes, viral protein-host protein interactions, and host epigenetic regulators involved in those processes to gain insight on virus-mediated deregulations and the resulting pathophysiological effects of COVID-19. We have mainly addressed four broad biological processes that can significantly affect COVID-19 patients: response to hypoxia, lung development, respiratory processes, and surfactant metabolism.

**Figure 5:**
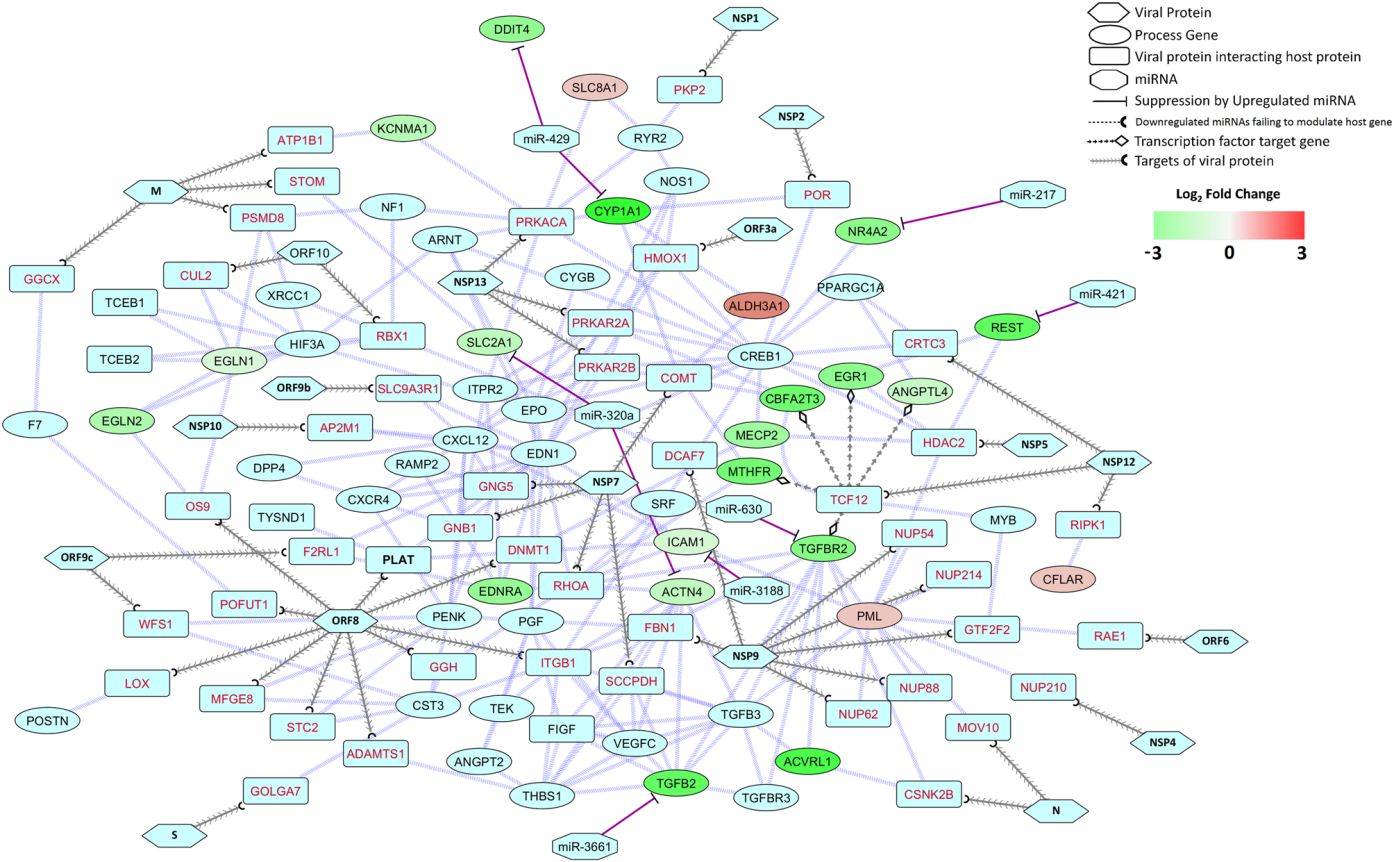
Network representing the interactions between proteins in response to hypoxia process (combined module) along with SARS-CoV-2 proteins (Gordon et al.^37^), and host miRNAs. Hexagon, ellipse, rounded rectangle, octagon represents viral proteins, process related genes, proteins that interacts viral proteins and host miRNAs, respectively. Blunted arrow indicates suppression by miRNAs, dotted arrow pointed with open half-circle indicates downregulated miRNAs failing to modulate host gene, arrowed line pointed with open half-circle indicates targets of viral proteins, and arrowed line pointed with open diamond indicates transcription factors of a gene.

Numerous genes in hypoxia response and HIF-1 alpha signaling were abruptly dysregulated in the SARS-CoV-2-infected lung (Supplementary Fig. 5). This included PLAT, a tissue plasminogen activator with profound roles in lung homeostasis, whose aberrant regulation can lead to many lung injuries^40^. In the PPI map of differentially-expressed genes of the GOBP^41^ module ‘response to hypoxia’ and SARS-CoV-2 target genes^37^, this PLAT protein was found to be directly targeted by viral protein ORF8 (Fig. 5). Moreover, several indirect responses from the viral protein interactions were also revealed (Fig. 5). SARS-CoV-2 M protein can target STOM which interacts with *SLC2A1*. *SLC2A1* can also be targeted by host miRNA miR-320a (Fig. 5). *SLC2A1* encoding GLUT1 protein is upregulated by hypoxic responses in alveolar epithelial cells^42^.

Viral ORF8 interaction with OS9 can modulate EGLN1 and EGLN2, which might disrupt the functions of the EGLN-HIF oxygen sensing system^43^. KCNMA1 was found to interact with host proteins ATP1B1 and PRKACA that interact with viral M and NSP13 proteins, respectively (Fig. 5). ORF9c may modulate EDNRA indirectly through F2RL1 (Fig. 5) and can alter the vasoconstrictor effect resulting from EDNRA^44^. PML functions might be altered through the viral N and MOV10 proteins’ interaction. Functions of SLCBA1 may be modulated by NSP13 through PRKACA (Fig. 5). NSP7 can regulate ALDH3A1 which can affect CYP1A1 and NR4A2 (Fig. 5). NSP5 interacting HDAC2 can curb PML and REST which have hypoxia responsive functions^45,46^ (Fig. 5). NSP12 can modulate a wide range of hypoxia functions related proteins, namely TGFBR2, MECP2, MTHFR, CBFA2T3, EGR1, and ANGPTL4 by interacting with the transcription factor TCF12 (Fig. 5). NSP12 through RIPK1 interactions can dampen the apoptosis regulating function of CFLAR^47^ (Fig. 5). Apart from this, several host miRNAs could possibly downregulate the expression of some genes, namely- miR-320a, miR-3188, miR-3661, miR-217, miR-421 and miR-429 (Fig. 5). These viral mediated deviations found in the hypoxia responses might be a decisive factor in the lung injury found in COVID-19 patients^48^.

**Figure 6:**
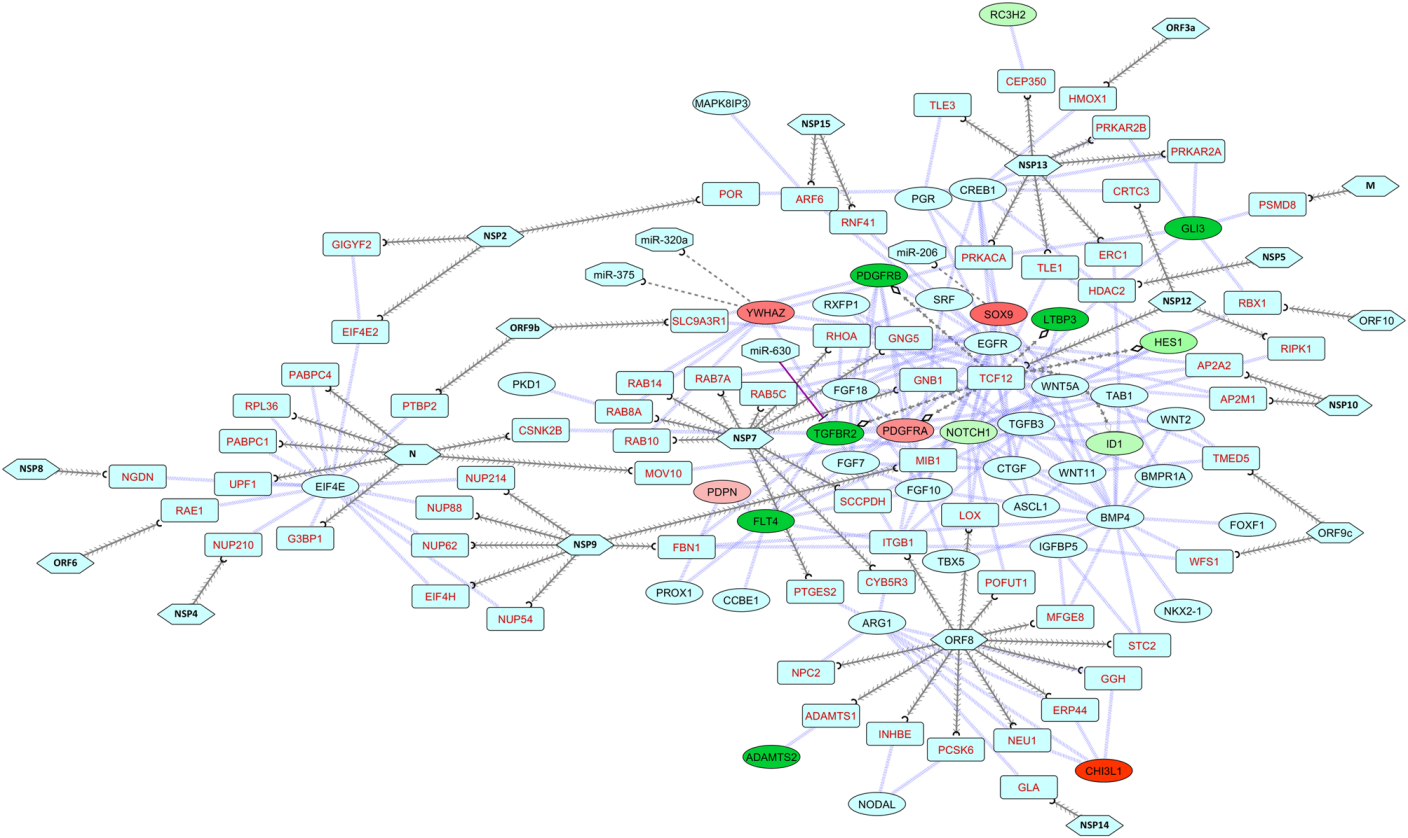
Network representing the interactions between proteins in lung development process (combined module) along with SARS-CoV-2 proteins (Gordon et al.^37^), and host miRNAs. Legends are similar as Fig. 5.

In the lung development network, viral protein NSP13 is found to indirectly target RC3H2, SOX9, GLI3 through CEP350, PRKACA, PRKAR2A proteins (Fig. 6). While GLI3 can also be modulated through several viral-host interactions, for instance ORF10-RBX1, NSP5-HDAC2, and the M-PSMD8 interactions (Fig. 6). ORF8 can indirectly modulate ADAMTS2, CHI3L1, NOTCH1, TCF12, and FLT4 (Fig. 6). Transcription factor TCF12 is targeted by viral NSP12 protein which can in turn affect transcriptions of *PDGFRA*, *PDGFRB*, *TGFBR2*, *ID1*, *HES1*, and *LTBP3* genes (Fig. 6). NSP5 can modulate NOTCH1 through HDAC2 (Fig. 6). Moreover, host miRNAs such as miR-630 can downregulate TGFBR2; while miR-206, miR-320a and miR-375 are downregulated and so SOX9, and WYHAZ are overexpressed (Fig. 6). Virus could hamper all these components of different growth factor signaling pathways which are crucial for various lung injury repair mechanisms^49,50^.

**Figure 7:**
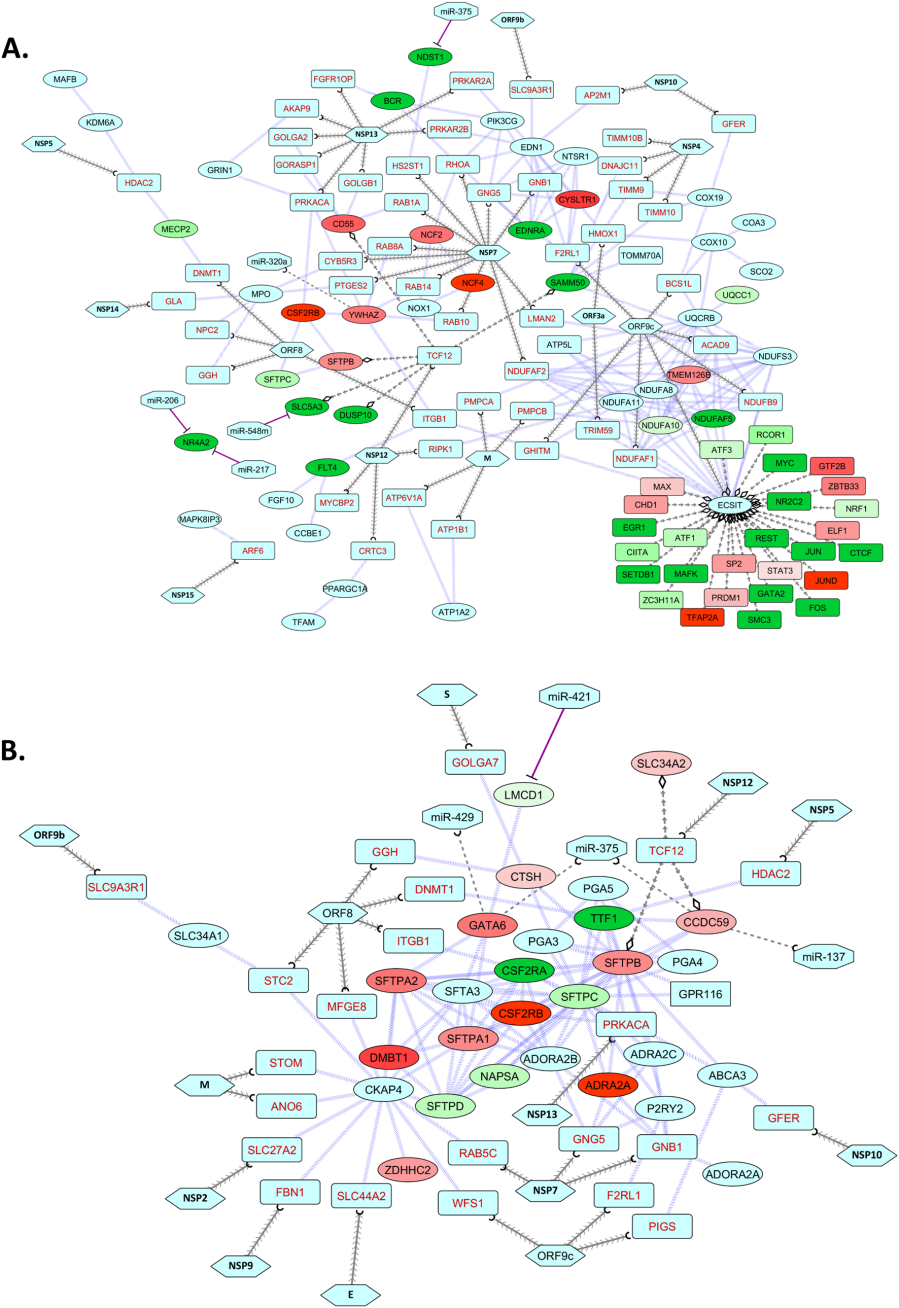
Network representing the interactions between proteins in (**A**) respiratory processes (combined module), (**B**) surfactant metabolism along with SARS-CoV-2 proteins (Gordon et al.^37^), and host miRNAs. Legends are similar as Fig. 5.

From the respiratory process network, we can delineate that several transcription factors are dysregulated. Many of these are associated with ECSIT that can directly interact with viral ORF9c and indirectly by viral ORF8, and NSP7 (Fig. 7A). Moreover, ECSIT itself is directly targeted by ORF9c (Fig. 7A). NSP12 can modulate SFTPB, SFTPC, SLC5A3, DUSP10, and SAMM50 by targeting TCF12 (Fig. 7A). Also, in this network we have observed suppressive actions of miRNAs- miR-206, miR-217, miR-375 on NR4A2 and NDST1; as well as upregulation of YWHAZ due to the probable downregulation of miR-320a (Fig. 7A). Virus might be dampening the host immune response in the lung by targeting ECSIT^51^ and by diminishing, respiratory gaseous exchange by negatively modulating the surfactant proteins^52^.

Surfactant metabolism is found to be modulated not only by viral proteins, but also through irregular host responses (Fig. 7B). Viral proteins NSP12 and NSP5 can target transcription factor TCF12 and epigenetic regulator HDAC2, respectively, which in turn modulate important members of surfactant metabolism process, namely- TTF1, CCDC59, SFTPB, SFTPC, CSF2RA, CSF2RB, NAPSA, SFTPD, and DMBT1 (Fig. 7B). These can also be modulated through CKAP4 which is observed to interact with viral M, NSP2, NSP9, E, and ORF8 proteins (Fig. 7B). Furthermore, we have observed that viral M and S proteins can interact with proteins of this process both directly and indirectly (Supplementary Fig. 6). Host miRNA miR-421 was found to downregulate *LMCD1*; while miR-137, miR-375, and miR-429 fail to modulate *CCDC59* and *GATA6* because of their probable inactivation/suppression by differential expression of the regulating TFs (Fig. 7B). As *SFTPD* and *SFTPC* are downregulated along with several regulatory partners, their primary function of immunomodulation and efficient air exchange in lung^53,54^ might be seriously hindered by the viral proteins; which could further lead to pathogenic lung injury^55^.

SARS-CoV-2 proteins can target several epigenetic factors, such as HDAC2, DNMT1, CUL2, MOV10, RBX1 and TLE1, to alter the above-mentioned processes. Epigenetic factors play a key role in balancing normal lung pathobiology, and anomalies in their regulation can lead to many lung diseases^56^. For instance, HDAC2 and DNMT1 have significant roles in chronic obstructive pulmonary disease (COPD) progression^57,58^.

### Drug enrichment suggests lung surfactant replacement therapy as a potential treatment option

**Figure 8:**
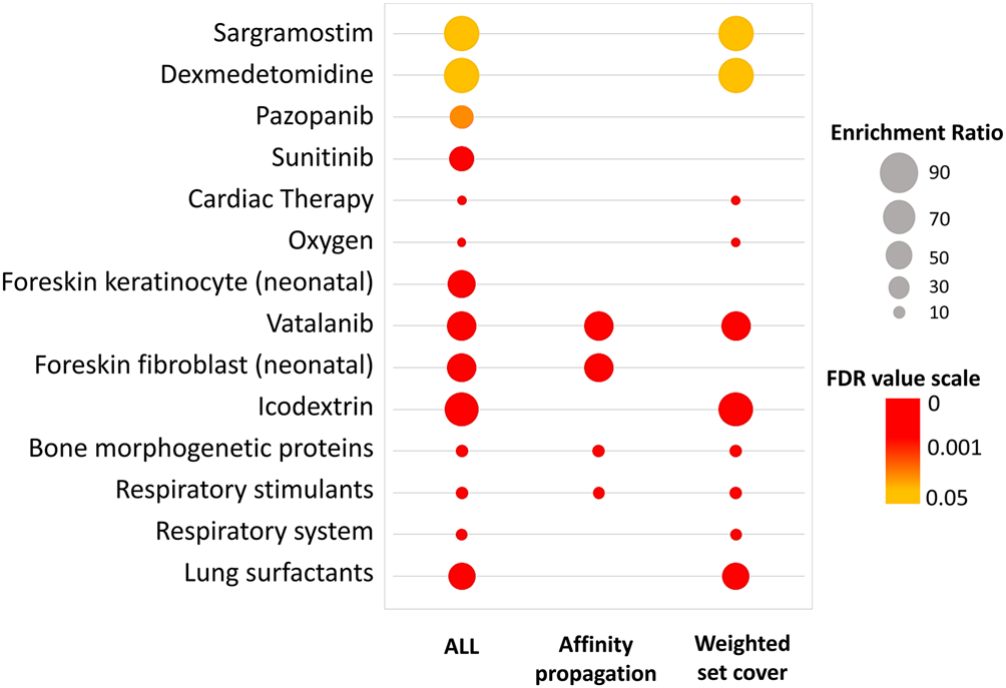
Bubble plot of drug enrichment results using WebGestalt tool^59^. Color towards red indicates higher significance and color towards yellow indicates less significance. Bubble size indicates the enrichment ratio.

Our results pointed towards the probable SARS-CoV-2 directed dysregulation in several important respiratory processes and surfactant metabolism pathway in COVID-19-affected lung. We then sought to dig out possible therapies/drugs for the improvement of the lung and respiratory conditions of COVID-19-affected patients. In this regard, we performed drug enrichment analysis for differentially expressed genes present in the combined module terms ‘response to hypoxia’, ‘lung development’, ‘respiratory process’ and ‘surfactant metabolism pathway’ using the WebGestalt functional enrichment tool^59^. We have found significant enrichment scores for lung surfactants, respiratory stimulants, sargramostim, and oseltamivir (Fig. 8, Supplementary Fig. 7).

## Discussion

Though there are a wide variety of symptoms and clinical features seen in COVID-19 patients, almost every mild-to-critically affected patient showed respiratory and breathing complications, ranging from pneumonia to acute lung injury^60^. Severely ill patients are greatly supported by artificial ventilation, as no therapeutic drugs for mitigating these complications have been discovered. This is due to the lack of understanding on the molecular aspects of lung-related abnormalities in COVID-19. We have identified dysregulated genes related to respiratory and lung-related processes upon the SARS-CoV-2-infection. We have prioritized these pathways and searched for potential therapies/drugs for the treatment which could lessen the resultant effects of gene dysregulation.

Several SARS-CoV-2 entry receptor (ACE2)/entry associated proteins (TMPRSS2, BSG, CTSL, and DPP4) have been previously described^61^. All of these viral entry associated factors are readily expressed in the lung, except ACE2 (Supplementary Fig. 8A). While analyzing the expression profiling in SARS-CoV-2-infected lung, we discovered a quite unexpected scenario. Astoundingly, in COVID-19-affected lung, *ACE2* was upregulated while the genes of the entry associated proteins were downregulated (Supplementary Fig. 8B). More COVID-19 patient data must be analyzed to validate this striking finding.

“Cytokine storm” is a much discussed phenomenon in previously reported pathogenic human coronavirus infections that can be lethal for the host due to the destruction of its own respiratory systems^62^. Similar responses can also occur following SARS-CoV-2 infection^63^. We observed that several inflammatory and antiviral responses are readily dysregulated in the SARS-CoV-2-infected lung that could lead to the abnormalities in overall respiratory functions.

Apart from this, our study identified dysregulation in several lung function related vital processes, namely- response to hypoxia, lung development, respiratory process, and surfactant metabolism; alterations in these pathways lead to abnormal lung pathobiology in COVID-19. Networks generated from combining the viral-host protein interactions suggest that viral proteins might be actively involved in these dysregulations along with several host factors, which might also be decontrolled due to the viral infections.

Hypoxic conditions are common in respiratory infections due to reduced oxygenation of the blood^64^. Several genes involved in hypoxia-induced responses were found to be dysregulated (Fig. 5) in the COVID-19-affected lung. ALDH3A1 can protect airway epithelial cells from destruction^65^. Though it is upregulated, its functions can be impeded through viral proteins (Fig. 5). CFLAR functions in shutting down apoptotic responses by interacting with RIPK1^47^, but viral interactions with RIPK1 might prevent this (Fig. 5). SARS-CoV-2 can induce the transcription of *ANGPTL4* by utilizing TCF12 transcription factor (Fig. 5) which in turn could cause pulmonary tissue damage^66^. Viral proteins could promote the activity of EGLN1 and EGLN2 (Fig. 5) to suppress the transcription of HIF-induced genes in hypoxia^67^; on the other hand the regulation through these proteins might be hampered due to viral interactions. Moreover, the constant overexpression of HIF-mediated inflammatory genes could also occur, which could lead to inflammation-induced lung damage. Severe hypoxia-induced responses can occur through the downregulation of MECP2^68^ in SARS-CoV-2 patients (Fig. 5). In hypoxia, REST is induced and can act as a negative regulator of gene expression to maintain a balance between different processes^45^, that were found to be downregulated and can be targeted through miR-421 in COVID-19 (Fig. 5). GLUT1 (*SLC2A1* gene) promotes increased glucose transport into hypoxic cells for its prolonged adaptation during this condition^69^, but it was found to be downregulated in lung of COVID-19 patient and this can occur through miR-320a (Fig. 5). During hypoxia, TGFβ signaling regulates inflammation and vascular responses^70^; but attenuated TGFβ expression might lead to increased disease severity (Fig. 5). EGR1 transcription factor can be modulated through viral proteins (Fig. 5) that might prevent the hypoxia induced EGR1 activation of HIF-1 alpha^71^. This probable route of viral-mediated HIF-1 signaling inhibition could curb the whole hypoxia induced survival responses. Moreover, defective hypoxia response can occur due to the overactivity of EGR1 that can result in anomalous thrombosis^72^. Also, PLAT is a crucial factor in splitting down clots^73^; this function might be directly targeted by SARS-CoV-2 ORF8 (Fig. 5). Many COVID-19 patients were reported to have pulmonary embolism and thrombosis^74^, which could be the effect of altered hypoxic responses.

Similarly, several lung development and respiratory process genes/proteins were found to be dysregulated in the COVID-19 patient’s lung (Fig. 6, 7). SOX9, an important regulator for the recovery from acute lung injury^75^, can be down-modulated by the virus (Fig. 6). Though the host miRNAs cannot target the YWHAZ pro-survival protein^76^, they can induce expression surfactant protein A2^77^, which might be indirectly modulated through viral protein NSP7 (Fig. 6). Likewise, viral protein ORF8 can indirectly modulate CHI3L1 (Fig. 6) that normally suppresses lung epithelium injury^78^. Virus can block TCF12 and inhibit the expression of PDGFRA and PDGFRB (Fig. 6) which could play a role in lung maturation and injury response^79^. GLI3, which exerts essential role in developing the lung and regulating the innate immune cells^80^, is downregulated in COVID-19 lungs (Fig. 6). LTBP3 can promote lung alveolarization^81^, but can be modulated by SARS-CoV-2 protein NSP12 (Fig. 6). Abnormal NOTCH signaling contributes significantly in various lung diseases^82^, and in SARS-CoV-2 infection *NOTCH1* and *HES1* are found downregulated (Fig. 6). CD55, a member of the complement system which plays crucial role in host defense in airway epithelium^83^, can be modulated by viral protein NSP12 (Fig. 7A). *CYSLTR1* is upregulated in SARS-CoV-2 infection (Fig. 7A), which is correlated to COPD^84^. This protein can be modulated by viral ORF9c and ORF3a (Fig. 7A). ECSIT was found to directly interact with viral ORF9c (Fig. 7A) which might stop ECSIT-mediated antiviral innate immune response^51^. Several mitochondrial genes, for instance *NDUFA10*, *NDUFAF5*, and *SAMM50* were downregulated in COVID-19-affected lung (Fig. 7A). As mitochondria plays important role in cellular respiration and lung diseases^85^, aberrant expression of these genes might also lead to lung-related complications. DUSP10 can regulate unusual inflammatory responses upon viral infections^86^, but in SARS-CoV-2-infected lung this gene was found downregulated, possibly through NSP12-TCF12 interactions (Fig. 7A).

Pulmonary surfactant proteins are lipoproteins that mainly function to lower the alveolar surface tension^87^ and can elicit immune stimulatory roles against some respiratory pathogens^88^. Among the surfactant proteins, SP-A and SP-D mainly evoke immune responses, while SP-B and SP-D play roles in maintaining efficient respiratory gas exchange^54^. Several lung diseases including asthma, acute respiratory distress syndrome (ARDS), COPD are reportedly associated with aberration in the function of surfactant proteins^55,89^. In SARS-CoV-2-affected lung, production of the surfactant proteins is found to be dysregulated (Fig. 7B). Viral protein NSP5 can recruit HDAC2 and downregulate the expression of *TTF1* which is needed for the expression of SP-B and SP-C (Fig. 7B). SP-A and SP-D can be targeted indirectly by several viral proteins (Fig. 7B) which might dampen the production of surfactant proteins in lungs, thus complicating the disease condition. The CSF2RA-CSF2RB complex modulate the surfactant recycling that maintains the overall balance of surfactant content^33^. We found that *CSF2RA* is dysregulated in SARS-CoV-2-infected lung, which could lead to abnormal surfactant recycling (Fig. 7B). While performing enrichment analysis, we witnessed a significant downregulation of several cholesterol biosynthesis pathways in COVID-19 patient’s lung cells (Fig. 1E) that could lead to low accumulation of phospholipids in lungs. As phosphatidylcholine (PC) and phosphatidylglycerol (PG) are the principal phospholipids of surfactant proteins^90^, disrupted production of lipids might make surfactant proteins non-functional.

Considering all these, lung surfactants might be useful in the treatment of COVID-19 patients, as lung surfactant therapies were previously reported to be successful in other respiratory infections and acute lung injury to reduce the lung damage of the patients^87, 91,92^. Also, other drugs including respiratory stimulants for COPD^93^, sargramostim for treating pulmonary alveolar proteinosis^94^, and oseltamivir in curing influenza-related lower respiratory tract complications^95^ showed potential for improving the lung’s and respiratory system’s overall condition.

From our results, we can suggest that surfactant protein production along with other respiratory responses in lung could be dysregulated in COVID-19. However, further experimental proteomic analyses of this dysregulation are required for the functional implications of this study. Along with the antiviral drugs to mitigate the viral responses, drugs that can improve lung conditions in COVID-19 could also be considered as a treatment option for the patients. Our generated results can be useful for obtaining greater insight on the probable dampened surfactant production upon SARS-CoV-2 infection, and the potential implications of surfactant therapy as a therapeutic agent for COVID-19 treatment.

## Methods

### Analysis of microarray expression data

Microarray expression data from both SARS-CoV-infected 2B4 cells and uninfected controls (both maintained for 24 h) obtained from Gene Expression Omnibus (GEO) (https://www.ncbi.nlm.nih.gov/geo)^96^, accession: GSE17400. Raw Affymatrix CEL files were background corrected, normalized using Bioconductor package “affy v1.28.1” using 'rma' algorithm. Quality of microarray experiment (data not shown) was verified by Bioconductor package “arrayQualityMetrics v3.44.0”^97^. Differentially expressed (DE) between two experimental conditions were called using Bioconductor package Limma^98^. Probe annotations were converted to genes using in-house python script basing the Ensembl gene model (Biomart 99)^99^. The highest absolute expression value was considered for the probes that were annotated to the same gene. We have considered the genes to be differentially expressed, which have FDR^100^ *p*-value ≤ 0.05 and Log2 fold change value ≥ 0.25 (Supplementary file 1).

### Analysis of RNA-seq expression data

Illumina sequenced RNA-seq raw FastQ reads were extracted from GEO database^96^, accession: GSE147507. This data includes independent biological triplicates of primary human lung epithelium (NHBE) cell lines which were mock treated or infected with SARS-CoV-2 for 24hrs. This data also contains two technical replicate of post-mortem lung biopsy sample of a deceased COVID-19 patient, along with lung biopsy samples of two different healthy persons as control. We have checked the raw sequence quality using FastQC program (v0.11.9)^101^, and found that the "Per base sequence quality" and "Per sequence quality scores" were high over the threshold for all sequences (data not shown). Mapping of reads was done with TopHat (tophat v2.1.1 with Bowtie v2.4.1)^102^. Short reads were uniquely aligned allowing at best two mismatches to the human reference genome (GRCh38) as downloaded from UCSC database^103^. Sequences matched exactly more than once with equal quality were discarded to avoid bias^104^. The reads that were not mapped to the genome were utilized to map against the transcriptome (junctions mapping). Ensembl gene model^105^ (version 99, as extracted from UCSC) was used for this mapping. After mapping, we used SubRead package featureCount (v2.21)^106^ to calculate absolute read abundance (read count, rc) for each transcript/gene associated to the Ensembl genes. For differential expression (DE) analysis we used DESeq2 (v1.26.0) with R (v3.6.2; 2019-07-05)^107^ that uses a model based on the negative binomial distribution. To avoid false positives, we considered only those transcripts where at least 10 reads are annotated in at least one of the samples used in this study and also applied a minimum Log2 fold change of 0.5 for to be differentially expressed transcripts apart from adjusted p-value cut-off of ≤ 0.05 by FDR. Raw read counts of this experiment are provided in supplementary file 2. To assess the fidelity of the RNA-seq data used in this study and normalization method applied here, we checked the normalized Log2 expression data quality using R/Bioconductor package “arrayQualityMetrics (v3.44.0)”^97^. From this analysis, in our data no outlier was detected by “Distance between arrays”, “Boxplots”, and “MA plots” methods and replicate samples were clustered together (Supplementary file 3).

### Retrieval of the host proteins that interact with SARS-CoV and SARS-CoV-2 proteins

We have obtained the list of human proteins that form high confidence interactions with SARS-CoV and SARS-CoV-2 proteins from previously conducted studies^37–39^ and processed their provided protein names into the associated HGNC official gene symbol.

### Functional enrichment analysis

We utilized Gitools (v1.8.4) for enrichment analysis and heatmap generation^23^. We have utilized the Gene Ontology Biological Processes (GOBP)^41^, Reactome pathway^28^, Bioplanet pathways^24^, HumanCyc database^108^, DisGeNet^25^, KEGG pathway^109^ modules, and a custom in-house built combined module (Supplementary file 4) for the overrepresentation analysis. This combined module was generated from related modules with few genes to a parent term/process which otherwise would have been left out from analysis due to statistical stringency cutoff (module with at least 10 genes are selected) during enrichment analysis. Resulting p-values were adjusted for multiple testing using the Benjamin and Hochberg's method of False Discovery Rate (FDR)^100^.

### Mapping of the differentially expressed genes from SARS-CoV-2-infected lung onto biological pathways

We utilized the Reactome pathway browser^28^ for the mapping of dysregulated genes of SARS-CoV-2 infection onto different biological pathways. We then focused on the pathways which were found to be enriched for lung-related functions.

### Obtaining the transcription factors which can modulate the differential gene expression

We obtained the transcription factors (TFs) which bind to a given differentially expressed gene using a custom TFs module created using ENCODE^110^, TRRUST^111^, and ChEA^112^ databases.

### Obtaining human miRNAs target genes

We extracted the experimentally validated target genes of human miRNAs from miRTarBase database^113^.

### Extraction of transcription factors modulate human miRNA expression

We downloaded experimentally validated TFs which bind to miRNA promoters and modules from TransmiR (v2.0) database which provides regulatory relations between TFs and miRNAs^114^. Because miRNAs play roles in transcriptional regulation, we considered TFs that are expressed (upregulated) and can ‘activate’ or ‘regulate’ miRNAs, or in the absence of TFs (downregulation), those could otherwise ‘suppress’ miRNAs.

### Identification of the host epigenetic factors genes

We used the EpiFactors database^115^ to find human genes related to epigenetic activity.

### Construction of biological networks

Construction, visualization and analysis of biological networks with differentially expressed genes, their associated transcription factors, associated human miRNAs, and interacting viral proteins were executed in the Cytoscape software (v3.8.0)^116^. We used the STRING^117^ database to extract only the highest confidences (0.9) edges for protein–protein interactions to reduce any false positive connection.

### Drug enrichment analysis

We used the WebGestalt tool^59^ for predicting potential drugs targeting the given differentially expressed genes. We selected the drugs based on FDR (BH) ≤ 0.05^100^, using both DrugBank^118^ and GLAD4U^119^ drug database combined.

## Supplementary information

Supplementary Information.

Supplementary Legend.

## Data Availability

Publicly available data were utilized. Analyses generated data are deposited as supplementary files.
