# Supplementary material for: Lung transcriptome of a COVID-19 patient and systems biology predictions suggest impaired surfactant production which may be druggable by surfactant therapy
Source: Sci Rep. 2020 Nov 10;10:19395. doi: 10.1038/s41598-020-76404-8 (PMC7656460; doi:10.1038/s41598-020-76404-8)
Supplement: Supplementary file 2 — Supplementary Legend. [file 41598_2020_76404_MOESM2_ESM.docx]

**Supplementary Figure legends**

**Supplementary Figure 1:** Venn diagrams for comparing the differences between **A.** Dysregulated genes in SARS-CoV, SARS-CoV-2 (NHBE cells), and SARS-CoV-2 (lung biopsy) infections, **B.** upregulated genes in SARS-CoV, downregulated genes in SARS-CoV-2 (NHBE cells), and upregulated genes in SARS-CoV-2 (lung biopsy) infections, **C.** upregulated genes in SARS-CoV, upregulated genes in SARS-CoV-2 (NHBE cells), and downregulated genes in SARS-CoV-2 (lung biopsy) infections, **D.** upregulated genes in SARS-CoV, downregulated genes in SARS-CoV-2 (NHBE cells), and downregulated genes in SARS-CoV-2 (lung biopsy) infections, **E.** upregulated genes in SARS-CoV, upregulated genes in SARS-CoV-2 (NHBE cells), and upregulated genes in SARS-CoV-2 (lung biopsy) infections, **F.** downregulated genes in SARS-CoV, upregulated genes in SARS-CoV-2 (NHBE cells), and upregulated genes in SARS-CoV-2 (lung biopsy) infections, **G.** downregulated genes in SARS-CoV, downregulated genes in SARS-CoV-2 (NHBE cells), and upregulated genes in SARS-CoV-2 (lung biopsy) infections, **H.** downregulated genes in SARS-CoV, upregulated genes in SARS-CoV-2 (NHBE cells), and downregulated genes in SARS-CoV-2 (lung biopsy) infections, **I.** downregulated genes in SARS-CoV, SARS-CoV-2 (NHBE cells), and SARS-CoV-2 (lung biopsy) infections.

**Supplementary Figure 2:** Dysregulated genes of selected terms from Figure 1 in SARS-CoV, SARS-CoV-2 (NHBE cells) and SARS-CoV-2 (Lung biopsy) infections. Genes of selected significant terms are represented here. For individual processes, blue means presence (differentially expressed gene of the module term) while grey means absence (not differentially expressed in the experimental condition in that module term). Processes in orange, green, purple, red, cyan color background represent Bioplanet^[24](#_ENREF_24)^, HumanCyc^[108](#_ENREF_108)^, GOBP[^41^](#_ENREF_41), Reactome^[28](#_ENREF_28)^, DisGeNet^[25](#_ENREF_25)^ enriched terms, respectively.

**Supplementary Figure 3:** Dysregulated genes of additional terms from combined module-based enrichment in SARS-CoV, SARS-CoV-2 (NHBE cells) and SARS-CoV-2 (Lung biopsy) infections. Genes of selected significant terms are represented here. For individual processes, blue means presence while grey means absence (color code as in Supplementary Figure 2).

**Supplementary Figure 4:** Expression profiles of genes in different lung associated processes obtained from enrichment analysis with combined module. Color towards red indicates more differential upregulation while color towards green indicates more differential downregulation; yellow color suggesting less expression change compared to control.

**Supplementary Figure 5:** Schematic representation of hypoxia response pathway from Reactome pathway database[^28^](#_ENREF_28). Color towards yellow indicates upregulation and while blue indicates downregulation.

**Supplementary Figure 6:** Network representing the interactions between genes in surfactant metabolism along with SARS-CoV-2 proteins (Srinivasan et al.[^39^](#_ENREF_39)), and host miRNAs. Legends are similar as Figure 5.

**Supplementary Figure 7:** Bubble plot of drug enrichment results of **A.** Surfactant metabolism genes, **B.** Lung development genes using WebGestalt tool[^59^](#_ENREF_59). Color towards red indicates higher significance and color towards yellow indicates less significance. Bubble size indicates the enrichment ratio.

**Supplementary Figure 8:** Expression profiles of SARS-CoV-2 receptor/associated proteins for entry into the target cell in **A.** Different organs of normal human, **B.** SARS-CoV-2, SARS-CoV infections. For FPKM scale, color towards blue means higher expression while color towards grey indicates lower level of expression. For Log_2_ fold change scale, color towards red indicates upregulation while color towards green indicates downregulation; yellow color suggesting no significant expression change.

**List of supplementary files**

**Supplementary file 1:** Log_2_ Normalized expression data of control and SARS-CoV infection (GEO accession: GSE17400).

**Supplementary file 2:** Raw read counts expression of RNA-seq data (GEO accession: GSE147507) of control and SARS-CoV-2 infection**.**

**Supplementary file 3:** Quality analysis results of processed normalized RNA-seq data using “arrayQualityMetrics”.

**Supplementary file 4:** List of the combined modules along with the genes and their associated original term and sources.

**Supplementary file 5:** List of host proteins which interact with SARS-CoV, SARS-CoV-2 proteins.
