# Supplementary material for: Lung transcriptome of a COVID-19 patient and systems biology predictions suggest impaired surfactant production which may be druggable by surfactant therapy
Source: Sci Rep. 2020 Nov 10;10:19395. doi: 10.1038/s41598-020-76404-8 (PMC7656460; doi:10.1038/s41598-020-76404-8)
Supplement: Supplementary file 1 — Supplementary Information. [file 41598_2020_76404_MOESM1_ESM.pdf]

Lung transcriptome of a COVID-19 patient and systems biology predictions suggest impaired surfactant production which may be druggable by surfactant therapy

Abul Bashar Mir Md. Khademul Islam[1*] and Md. Abdullah-Al-Kamran Khan[2]

[1]Department of Genetic Engineering & Biotechnology, University of Dhaka, Dhaka, Bangladesh.

[2]Department of Mathematics and Natural Sciences, BRAC University, Dhaka, Bangladesh.

*** Correspondence:**

Dr. Abul Bashar Mir Md. Khademul Islam
Associate Professor
Department of Genetic Engineering and Biotechnology
University of Dhaka
Dhaka 1000, Bangladesh.
Email: khademul@du.ac.bd

Supplementary Figure 1

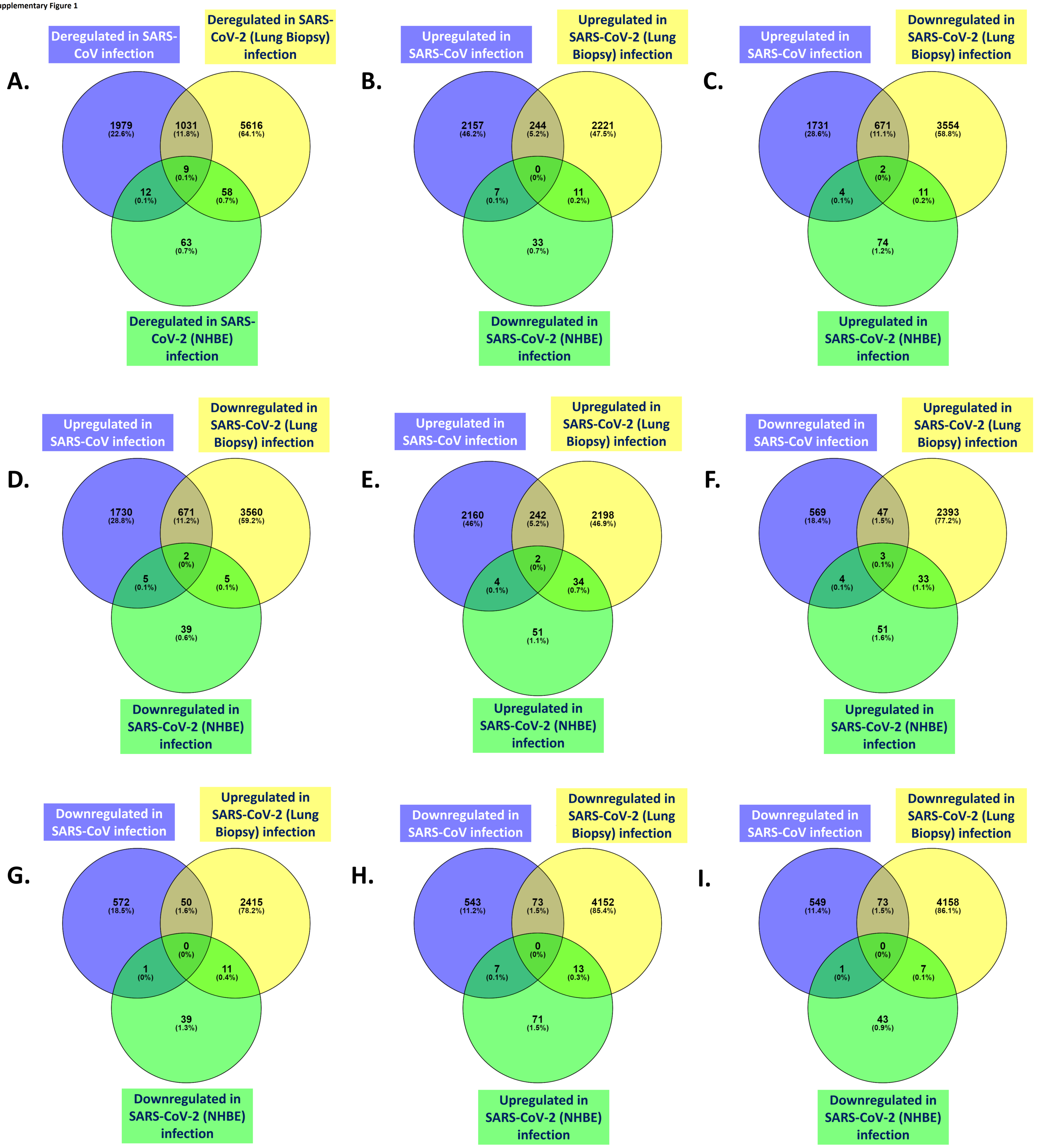

Supplementary Figure 2

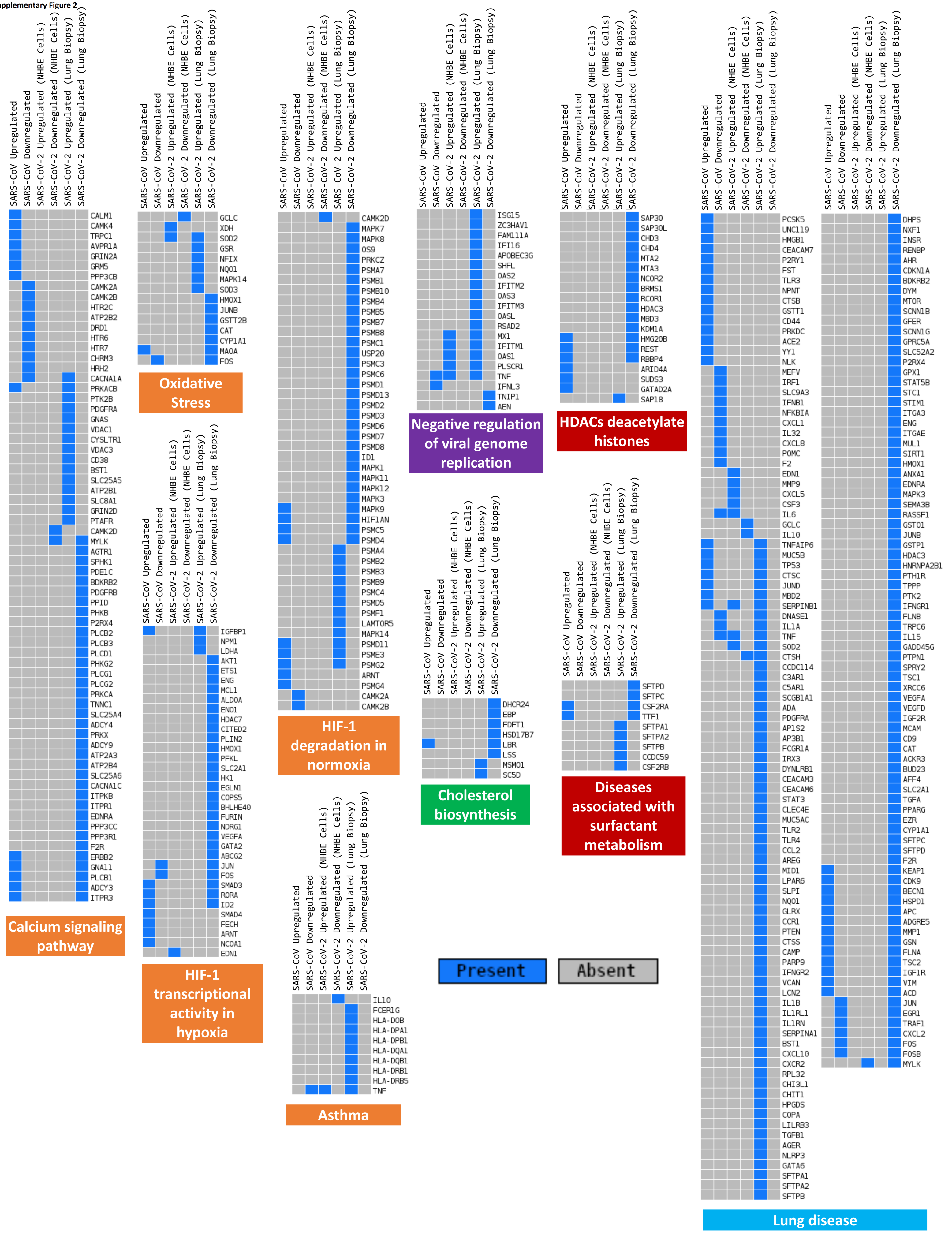

Supplementary Figure 3

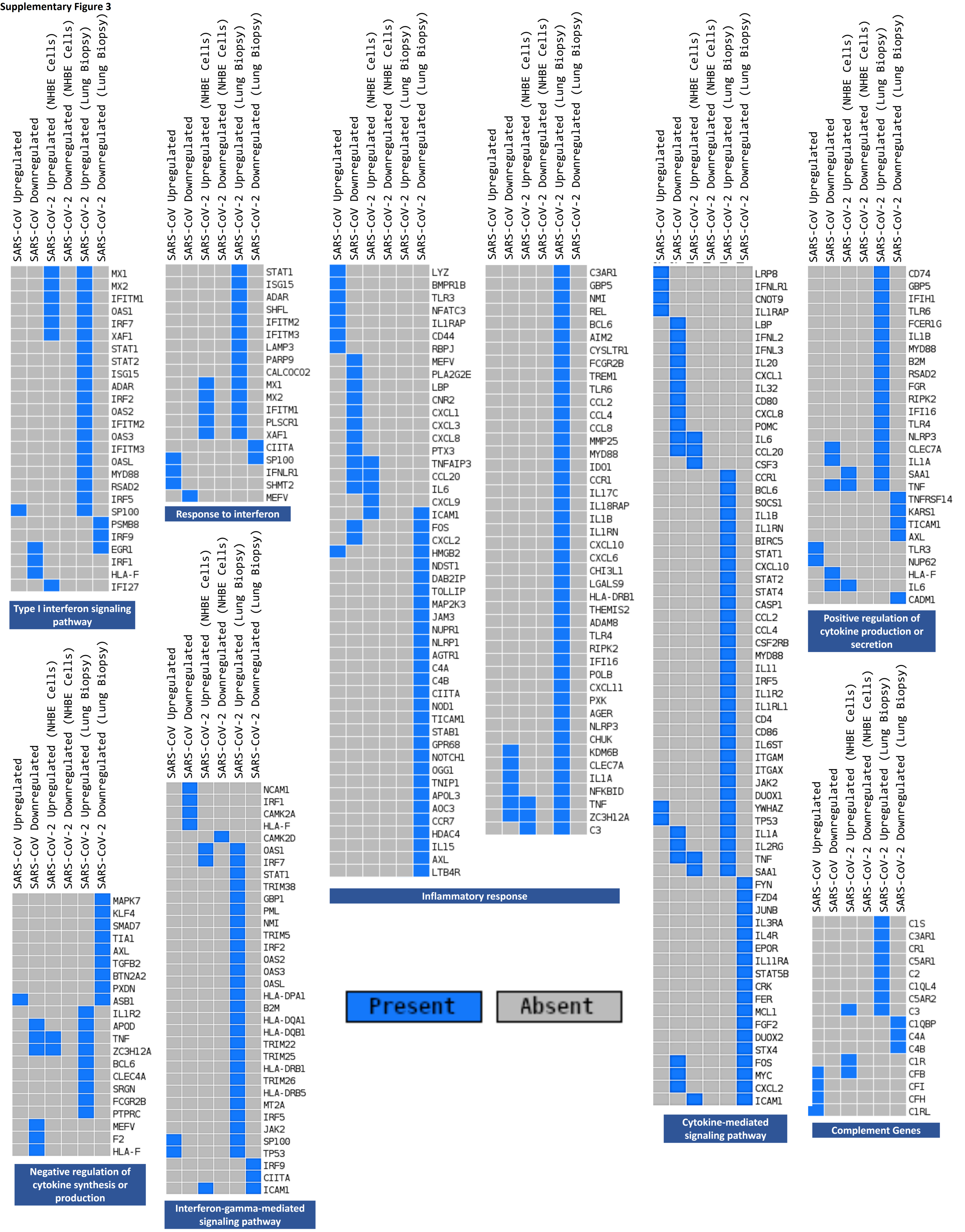

Supplementary Figure 4

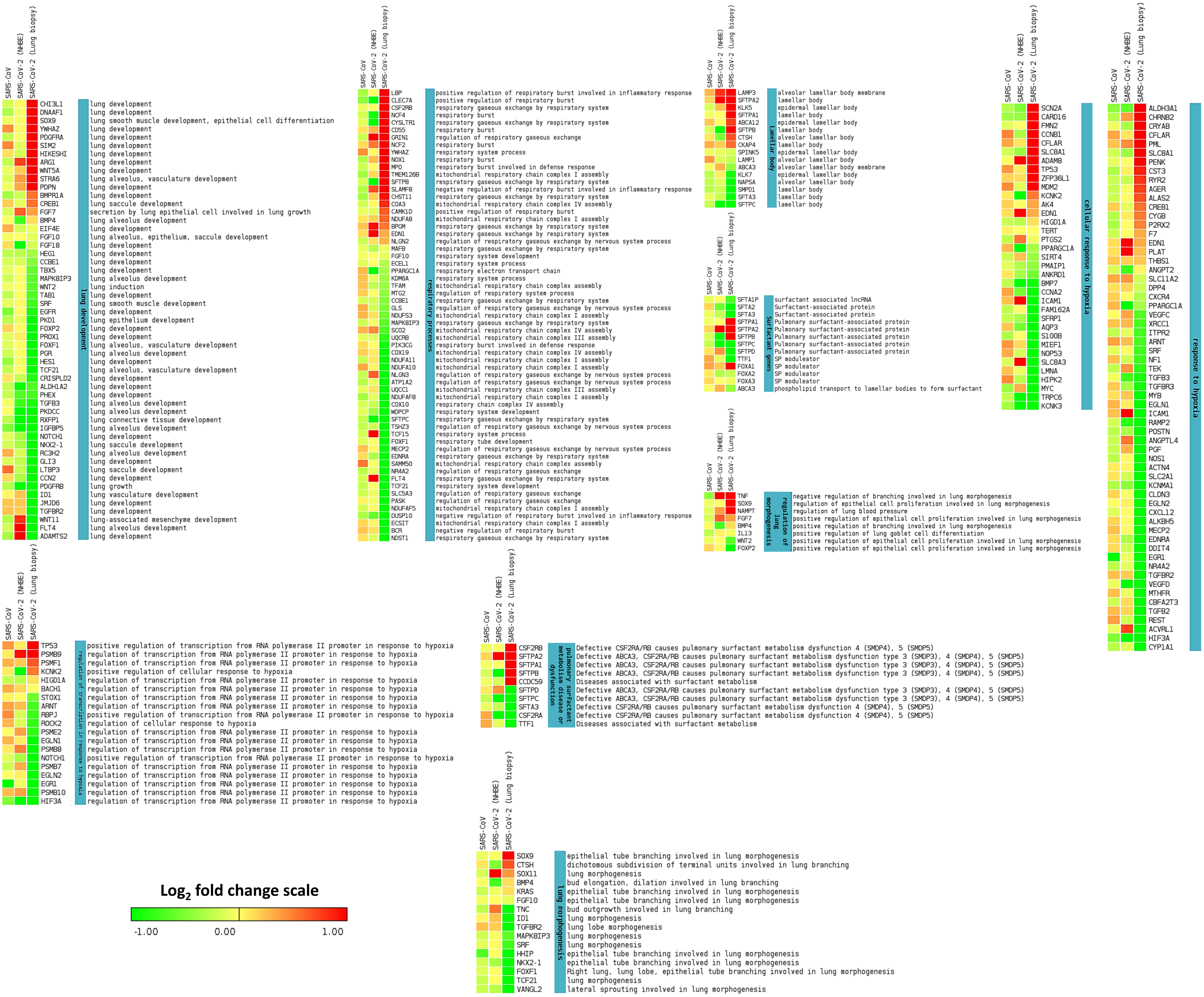

Supplementary Figure 5

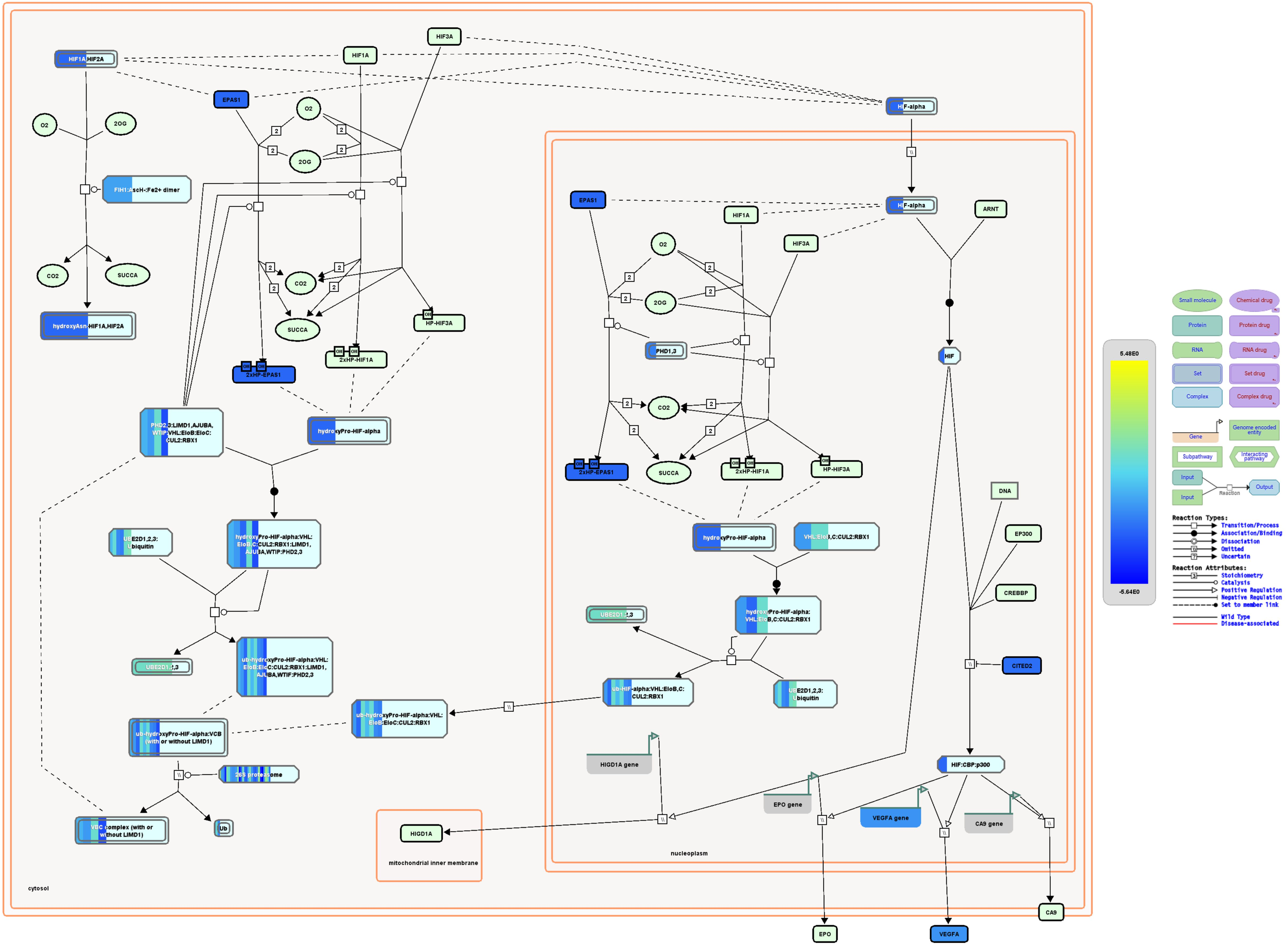

Supplementary Figure 6

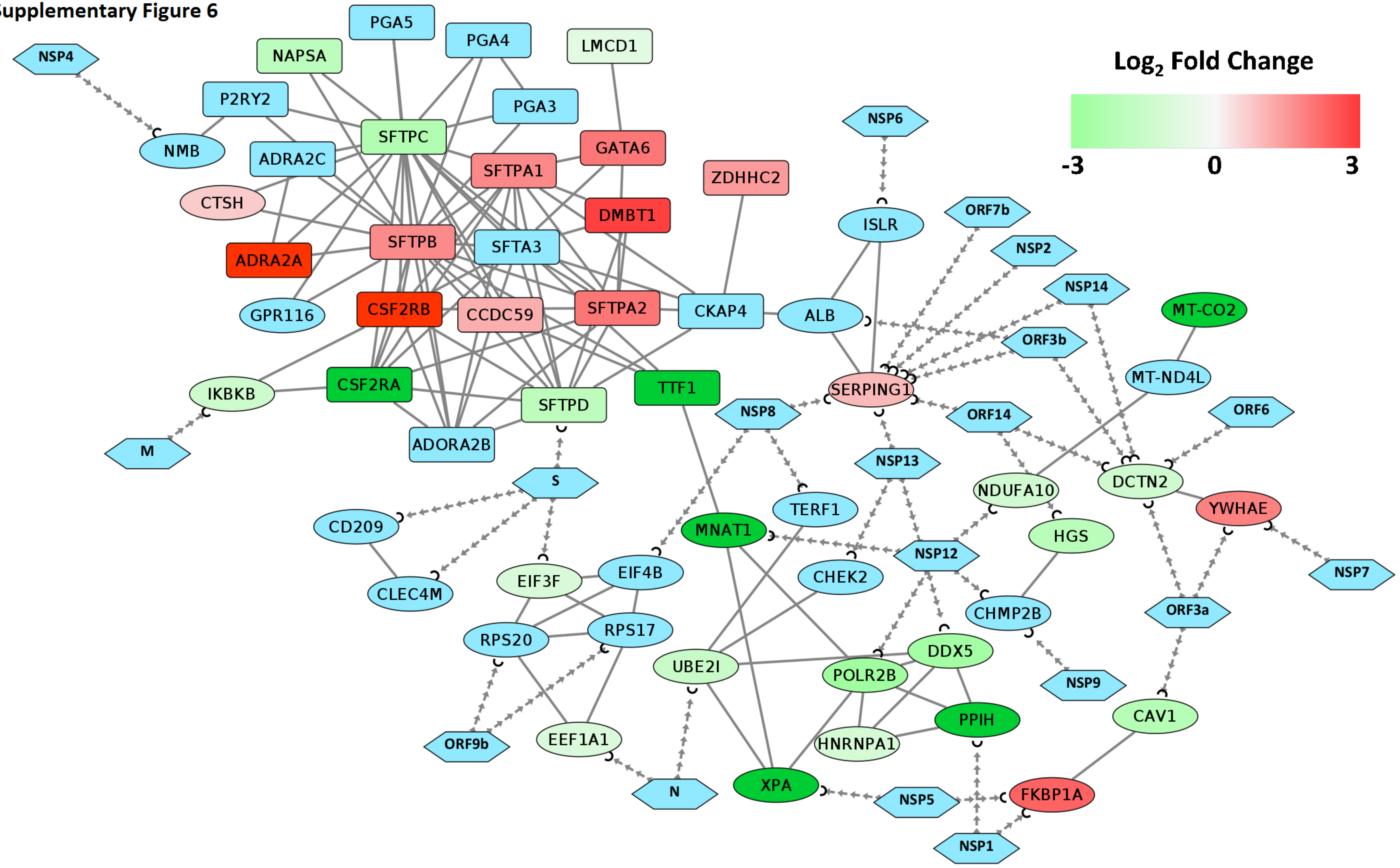

Supplementary Figure 7

A.

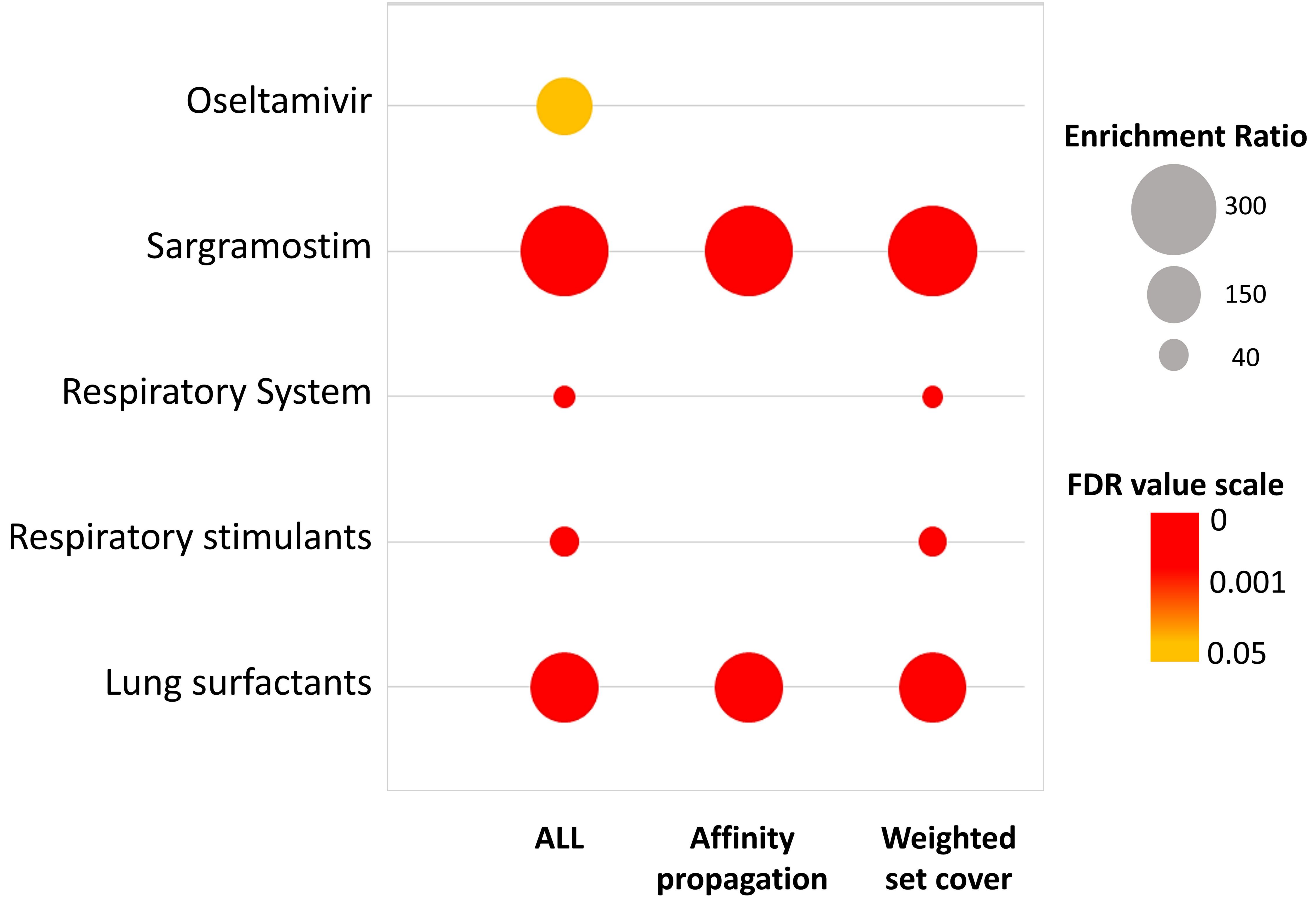

B.

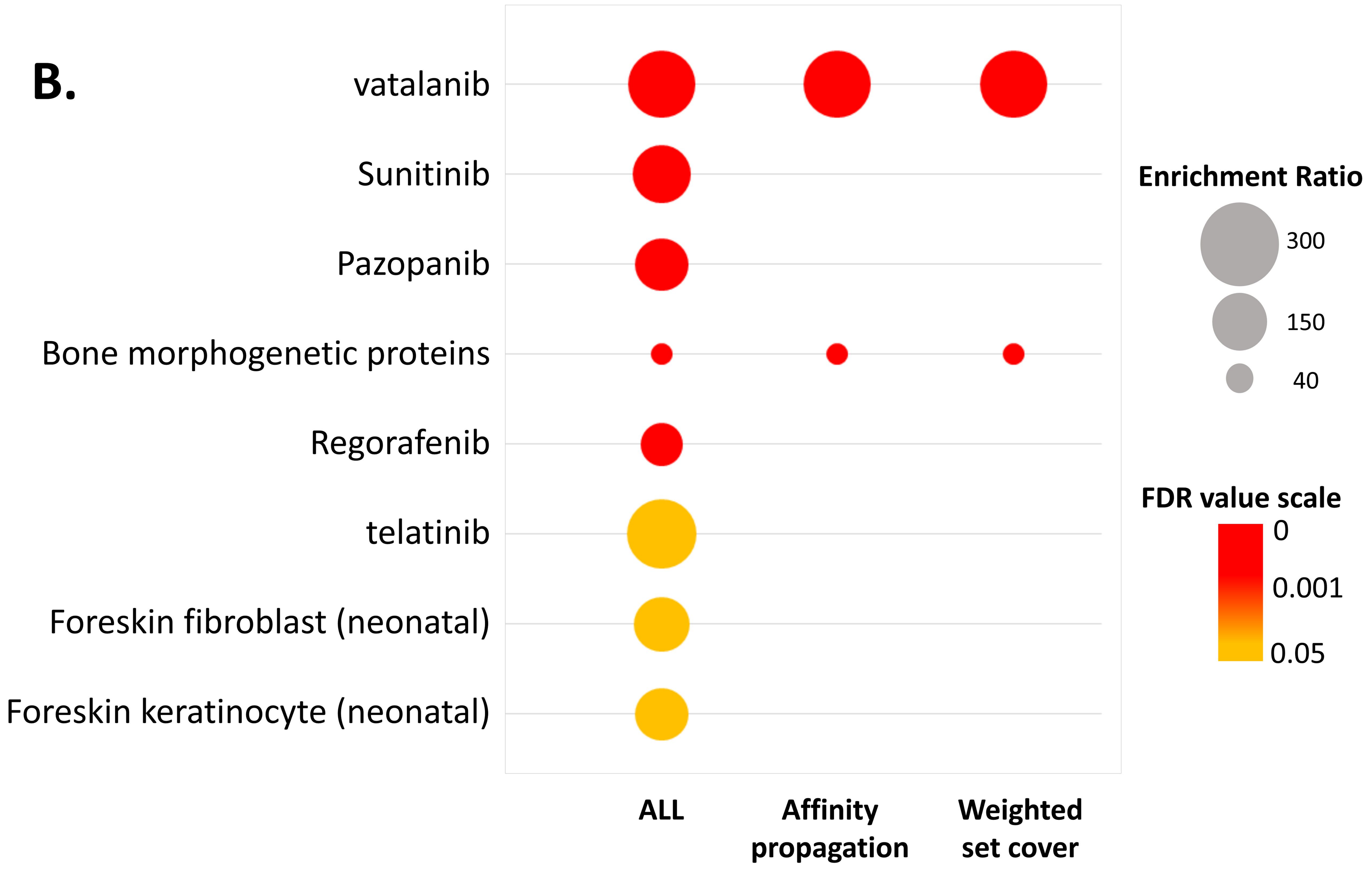

Supplementary Figure 8

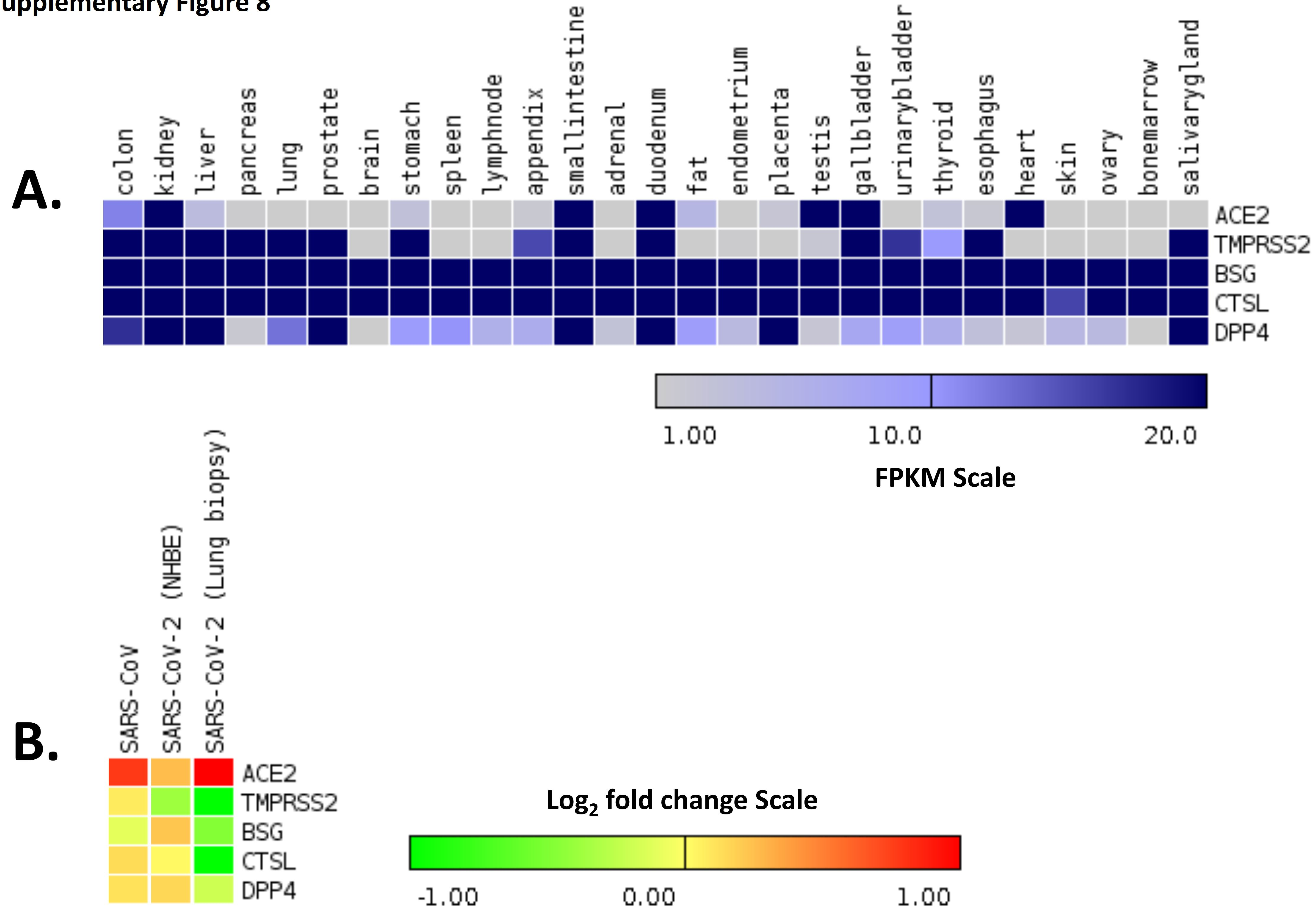

Supplementary file 1: Log2 Normalized expression data of SARS-CoV infection (GSE17400)

SAMPLE ID	GSM432033	GSM432034	GSM432209	GSM432332	GSM432333	GSM432360
Sample	WT	WT	WT	SARS-CoV	SARS-CoV	SARS-CoV
Genes						
TULP1	7.308	7.296	7.364	7.617	7.095	7.290
ATRX	10.203	10.109	10.160	9.958	9.930	10.079
AC093422.2	4.814	4.656	4.816	5.050	5.024	4.753
TCOF1	9.517	9.643	9.715	9.505	9.281	9.536
NSRP1	8.686	8.644	8.692	8.148	8.359	8.427
SPPL3	11.180	11.196	11.359	11.237	11.137	11.191
AF001548.2	6.645	6.735	6.687	6.662	6.825	6.701
OPA1	10.032	10.108	10.077	9.751	9.828	9.828
SLC66A1	8.071	8.141	8.213	8.213	8.378	8.258
SLC66A2	8.750	8.778	8.894	8.545	8.517	8.370
AC011008.1	5.590	5.514	5.621	5.656	5.789	5.739
AL359853.2	7.534	7.176	7.442	7.785	7.531	7.463
ITGA8	7.528	7.505	7.962	8.135	7.950	7.977
ITGA9	6.848	6.979	7.029	6.953	6.892	6.914
ITGA1	7.486	7.795	7.665	6.956	7.392	7.205
ITGA2	10.146	10.207	10.184	9.895	10.149	9.808
ITGA4	7.792	7.307	7.782	7.863	7.606	7.723
ITGA5	8.344	8.375	8.390	8.401	8.496	8.481
ITGA6	11.576	11.626	11.489	11.290	11.390	11.593
ITGA7	7.753	7.961	8.149	7.800	7.903	7.911
TRHR	6.641	6.567	6.416	6.526	6.481	6.547
UFSP1	6.957	6.962	6.973	6.799	6.994	6.962
UFSP2	8.601	8.714	8.851	8.602	8.580	8.623
TEX28P2	5.842	5.912	6.019	6.408	6.090	6.168
TEX28P1	5.842	5.912	6.019	6.408	6.090	6.168
CHST9	8.774	8.919	9.031	8.728	8.609	8.845
CHST8	7.513	7.484	7.701	7.575	7.546	7.580
CHST1	6.936	6.889	6.823	6.886	6.991	6.786
CHST3	8.787	8.879	8.925	8.730	8.828	8.890
CHST2	5.260	5.187	5.448	5.539	5.386	5.319
CHST5	7.790	7.766	7.754	7.990	7.730	7.666
CHST4	11.553	11.621	11.692	11.542	11.601	11.486
CHST7	10.336	10.391	10.553	10.221	10.286	10.309
CHST6	7.089	7.074	7.208	6.993	7.136	7.179
ITGAX	7.951	7.998	8.003	8.370	8.060	8.098
RPS4XP3	6.247	6.312	6.387	6.168	6.461	6.439
BCL2A1	4.369	4.307	4.293	4.471	4.659	4.315
MTFR1	9.571	9.708	9.593	9.321	9.547	9.536
RPS4XP9	6.077	6.110	5.895	6.081	6.080	6.209
ITGAV	10.658	10.676	10.640	10.361	10.294	10.587
SNRPCP6	8.196	8.262	8.287	8.740	8.301	8.304
ITGAL	6.611	6.625	6.662	6.719	6.678	6.751
ITGAM	6.416	6.323	6.475	6.570	6.598	6.580
ITGAD	7.271	7.473	7.388	7.218	7.365	7.474
ITGAE	8.483	8.483	8.580	8.457	8.372	8.465
IGF2R	10.811	10.838	10.877	10.725	10.549	10.795
SLC36A4	8.087	8.167	8.244	7.970	8.017	8.177
CKS1B	11.113	11.134	11.460	11.121	11.202	11.148
SLC36A1	8.575	8.780	8.742	8.360	8.542	8.520
AC133540.1	6.661	6.928	7.015	6.998	7.009	6.884
ARTN	8.132	8.062	8.084	8.055	7.823	8.100

ART1	7.898	7.911	7.956	7.854	7.937	7.887
ART3	4.699	4.849	4.767	4.850	4.789	4.867
ART5	8.762	8.655	8.699	8.743	8.966	8.883
AC106801.1	8.360	8.495	8.033	8.434	8.411	8.345
AC129492.7	7.156	6.944	7.162	7.156	7.223	7.145
CRTAM	4.516	4.476	4.748	4.418	4.599	4.499
CADPS2	8.518	8.550	8.629	8.461	8.272	8.485
RLF	8.092	8.027	8.178	8.387	8.173	8.270
CRTAP	10.805	10.836	10.800	10.790	10.886	10.855
ADPGK	10.937	10.993	11.162	10.967	10.887	11.001
HPYR1	7.888	8.096	8.038	8.061	8.003	8.082
AL139039.4	6.774	6.673	6.348	6.365	6.894	6.831
CPEB4	9.672	9.732	9.886	9.621	9.870	9.488
CPEB1	8.538	8.670	8.632	8.366	8.529	8.228
CPEB3	5.454	5.582	5.692	5.497	5.349	5.619
AL139039.3	6.774	6.673	6.348	6.365	6.894	6.831
SRBD1	8.300	8.445	8.515	8.137	8.302	8.205
ABAT	9.290	9.146	9.156	8.651	8.531	8.726
POU5F1B	8.619	8.606	8.654	8.646	8.777	8.730
BX255925.1	6.510	6.569	6.282	6.435	6.555	6.627
BX255925.3	9.143	9.192	9.371	9.222	9.152	9.225
AC009053.2	4.156	4.012	4.009	4.011	4.093	4.087
AL358394.2	7.055	7.154	7.088	6.784	6.754	6.953
PHOX2A	7.500	7.755	7.806	7.893	7.698	7.575
PHOX2B	6.978	6.852	6.723	6.827	6.957	7.003
UPK2	7.557	7.701	7.842	7.705	7.767	7.803
CFH	8.274	8.178	8.217	8.142	8.003	8.163
CFI	9.005	8.920	9.083	8.478	8.519	8.657
AL354692.1	4.466	4.136	4.384	4.447	4.408	4.351
ANKFY1	9.649	9.614	9.696	9.418	9.472	9.577
CFB	10.634	10.645	10.647	10.363	10.369	10.287
TPT1-AS1	7.121	7.311	7.258	7.429	7.431	7.561
CFP	5.718	5.789	5.998	6.051	6.019	5.960
OPA3	8.798	8.779	8.896	8.833	8.791	8.900
AAAS	9.064	8.999	9.089	9.031	9.140	9.071
SIDT1	4.912	4.690	5.069	4.797	4.828	4.905
SIDT2	8.842	8.869	8.880	8.862	8.824	8.846
SAGE1	5.310	5.243	5.192	5.365	5.361	5.343
UFD1	10.990	11.069	11.163	11.083	11.032	11.067
AC093802.2	6.745	6.928	6.889	6.743	6.925	6.824
AAGAB	9.806	9.924	9.958	9.614	9.682	9.808
C4orf19	9.773	9.807	9.955	9.826	9.845	9.858
AC093519.1	7.149	7.263	7.119	7.268	7.249	7.221
C4orf17	7.589	7.121	7.049	7.556	7.569	7.506
AL591806.4	12.136	12.242	12.354	12.061	11.964	12.018
NBEAP1	3.919	4.047	4.084	4.176	3.941	4.007
SLC66A3	9.693	9.795	9.857	9.699	9.479	9.612
ABCE1	10.094	10.213	10.182	10.294	10.154	10.278
TEN1-CDK3	8.180	8.232	8.437	8.065	8.159	8.133
AF287957.2	7.941	7.858	7.809	7.996	7.967	7.920
AF287957.1	7.941	7.858	7.809	7.996	7.967	7.920
ATP5IF1	9.611	9.748	10.017	9.571	9.418	9.674
NCBP2-AS1	9.116	9.115	9.333	9.064	9.083	8.941
ZNF197	7.317	7.317	7.303	7.411	7.453	7.421
ST20-AS1	8.453	8.265	8.359	8.051	8.120	8.301
TBC1D8-AS1	5.689	5.715	5.359	5.810	5.713	5.569

ZNF117	7.925	7.866	7.854	8.031	8.013	7.878
ZNF114	6.126	6.082	6.163	6.016	6.217	6.087
ZNF112	5.949	6.034	5.875	5.617	5.656	5.835
SEZ6L	8.160	8.119	8.159	8.457	8.269	8.319
AC008742.1	6.548	6.843	6.959	7.215	6.923	6.794
PRKAR2A	10.809	11.023	11.046	10.817	11.013	10.735
AL136298.3	8.221	7.998	8.020	8.058	8.096	7.989
MAGT1	11.668	11.803	11.926	11.906	11.840	11.925
TMBIM6	12.987	13.056	13.173	12.784	12.970	12.829
TMEM200B	6.421	6.372	6.582	6.462	6.439	6.392
ITGA2B	7.386	7.383	7.632	7.867	7.587	7.595
AC067852.2	9.608	9.764	9.914	9.567	9.595	9.573
PHACTR4	10.365	10.424	10.392	10.241	10.390	10.267
NLGN2	9.255	9.327	9.411	9.470	9.609	9.522
NLGN3	7.841	7.986	8.110	8.296	8.136	8.045
PHACTR3	9.868	9.875	9.831	9.531	9.390	9.528
NUP107	10.306	10.333	10.462	10.157	10.081	10.032
KRTAP2-3	7.077	7.055	7.139	6.990	7.126	6.993
KRTAP2-2	5.043	4.814	4.886	5.034	5.252	5.107
KRTAP2-1	6.093	5.855	5.651	6.092	6.076	6.043
KRTAP2-4	5.871	5.920	5.873	5.964	5.972	5.990
MIR4700	10.591	10.681	10.676	10.395	10.363	10.525
U62317.3	8.438	8.440	8.175	8.622	8.502	8.578
AC095038.4	4.610	4.371	4.389	4.243	4.379	4.354
VMP1	11.119	11.132	11.193	11.197	11.142	11.284
EPN2-AS1	5.545	5.447	5.471	5.359	5.454	5.485
AC046130.2	6.347	6.068	6.521	6.430	6.276	6.095
NR1D1	11.339	11.416	11.398	11.392	11.527	11.729
NR1D2	8.305	8.355	8.587	8.387	8.347	8.121
AC129926.1	5.727	5.773	5.661	5.881	5.817	5.869
PLPBP	12.402	12.431	12.509	12.246	12.507	12.239
AL513218.1	7.854	7.675	7.752	8.090	8.095	8.016
AL513218.2	10.193	10.193	10.245	10.181	10.182	10.196
OGFR	10.048	10.079	10.203	10.071	10.055	10.042
RPS26P47	12.821	12.916	13.024	12.923	12.903	12.978
SEPTIN14P21	7.159	6.862	6.635	6.551	6.694	6.987
AC235097.1	4.895	4.833	4.855	5.407	5.304	5.222
PTCH2	8.122	8.130	8.022	8.128	8.228	8.116
AL122058.1	4.420	4.492	4.371	4.599	4.650	4.604
PTCH1	6.407	6.557	6.775	6.964	6.945	6.646
TIMM44	8.391	8.622	8.638	8.571	8.325	8.394
RAD9B	4.446	4.448	4.599	4.295	4.280	4.541
AC006065.3	6.275	5.927	6.395	6.381	6.523	6.452
ERGIC2	10.804	10.796	10.980	10.884	10.830	10.649
AC006065.4	6.275	5.927	6.395	6.381	6.523	6.452
ERGIC1	11.564	11.803	12.248	12.204	11.765	11.419
B3GAT3	9.042	9.113	9.150	9.001	9.145	9.125
B3GAT2	6.152	6.060	6.155	6.397	6.053	6.181
B3GAT1	7.825	8.144	8.008	8.131	8.129	8.092
CALM2	13.685	13.715	13.759	13.763	13.800	13.812
CALM3	11.368	11.428	11.660	11.499	11.461	11.309
SPG21	11.665	11.718	11.919	11.714	11.655	11.639
CALM1	12.474	12.541	12.644	12.482	12.457	12.308
GSDMB	11.939	11.899	11.875	11.856	11.782	11.834
GSDMC	7.892	7.943	7.945	7.906	8.117	8.033
GSDMD	10.266	10.256	10.524	10.054	10.014	10.006

FBXL13	4.627	4.574	4.815	4.631	4.626	4.677
UPK1B	6.920	6.832	6.787	7.193	7.112	6.916
AC099522.2	5.775	5.986	5.807	6.252	6.085	6.082
DLGAP2	7.600	7.754	7.411	7.762	7.971	7.809
DLGAP3	7.600	7.569	7.734	7.603	7.722	7.660
DLGAP1	8.023	8.143	8.111	8.221	8.050	8.212
DLGAP4	8.675	8.619	9.006	8.909	9.036	8.645
DLGAP5	9.659	9.708	9.851	9.045	9.184	9.155
CYP27B1	8.361	8.378	8.381	7.885	8.235	8.120
OIP5	8.782	8.801	9.001	8.229	8.532	8.397
AC004160.1	6.635	6.481	6.588	6.762	6.704	6.892
FBXL19	8.780	8.804	9.031	9.011	8.909	8.972
INO80B-WBP1	10.130	10.206	10.336	10.332	10.107	10.248
PTPN18	9.100	9.040	9.076	8.819	8.876	8.929
PTPN13	6.978	6.951	6.861	6.461	6.778	6.754
PTPN12	9.432	9.459	9.594	9.380	9.356	9.354
PTPN11	11.646	11.718	11.671	11.549	11.458	11.618
PTPN14	9.609	9.485	9.412	9.284	9.150	9.332
LYAR	8.258	8.170	8.365	8.227	8.336	7.961
AC090227.2	10.814	10.879	10.926	10.776	10.627	10.786
AC138035.1	9.349	9.203	9.195	9.170	9.223	9.503
AC138035.2	4.095	4.251	4.353	3.909	4.106	4.174
AC090227.1	6.271	6.333	6.470	6.193	6.295	6.507
AC106754.1	4.624	4.664	4.647	4.679	4.837	4.730
AL353588.1	6.454	6.752	6.483	6.530	6.595	6.743
C2orf81	8.796	8.692	8.549	8.781	8.566	8.877
C2orf80	5.109	5.347	5.098	5.263	5.240	5.142
C2orf83	6.535	6.446	6.607	6.907	6.453	6.485
C2orf88	7.946	8.066	8.111	8.110	8.242	8.244
THSD8	9.746	9.690	10.001	9.410	9.390	9.375
TRAPPC6A	9.877	10.120	10.361	9.870	9.685	9.848
MZB1	7.949	7.937	7.990	8.144	8.285	8.074
TRAPPC6B	8.692	8.857	8.778	8.390	8.270	8.602
HLA-DQB1	9.549	9.669	9.869	9.486	9.577	9.600
MSMB	4.685	4.546	4.762	4.571	4.610	4.646
HLA-DQB2	6.482	6.443	6.506	6.587	6.666	6.730
THSD4	9.808	9.708	9.631	9.798	9.922	9.642
AL355916.3	8.707	8.703	8.644	8.732	8.699	8.704
ATP2B1-AS1	7.567	7.592	7.560	7.206	7.513	7.307
AC093536.2	5.489	5.725	5.641	5.595	5.561	5.576
AC093536.1	10.426	10.382	10.538	10.404	10.407	10.293
FOXS1	6.501	6.603	6.472	6.610	6.455	6.487
LPAR2	9.283	9.402	9.414	9.203	9.222	9.115
CDK9	8.695	8.543	8.784	8.310	8.461	8.355
THSD1	6.981	6.869	7.047	6.961	6.911	6.714
AC243742.3	8.099	7.982	8.231	7.620	7.926	7.816
CAMKK2	10.763	10.808	11.003	10.412	10.607	10.464
CAMKK1	8.226	8.203	8.425	8.394	8.435	8.496
MYDGF	10.711	10.682	10.964	10.773	10.832	10.798
LINC00158	4.815	4.981	5.154	5.153	5.114	5.142
AL031710.1	6.016	5.969	5.679	6.189	6.030	6.032
CHMP3	11.930	11.678	11.663	11.593	11.371	11.803
RPL36AL	12.035	12.122	12.258	12.102	11.963	12.024
CHMP7	9.404	9.406	9.591	9.409	9.369	9.379
CHMP6	8.736	8.813	8.812	8.741	8.733	8.674
CHMP5	10.966	11.119	11.044	11.084	10.964	11.154

AC015845.2	7.641	7.607	7.642	7.825	7.731	7.763
AC015845.1	7.310	7.306	7.551	7.663	7.456	7.616
LY6G6C	8.062	8.039	7.826	8.106	8.144	8.100
HOXA13	7.573	7.642	7.606	7.802	7.834	7.865
LY6G6E	5.900	5.855	5.953	5.764	5.876	6.020
HOXA11	8.057	8.088	8.212	8.020	8.069	8.007
HOXA10	9.474	9.477	9.571	9.329	9.514	9.304
AC104021.1	6.155	6.313	6.270	6.351	6.344	6.234
GALNTL5	4.510	4.553	4.466	4.458	4.507	4.482
RPS4XP2	12.748	12.864	12.961	12.845	12.882	13.010
GALNTL6	5.461	5.477	5.690	5.668	5.536	5.439
LINC02019	7.117	7.127	7.190	7.045	7.150	7.196
LINC02011	4.506	4.228	4.576	4.517	4.509	4.584
LINC02012	7.427	7.349	7.589	7.515	7.376	7.412
SPECC1	7.930	8.074	8.002	7.746	7.824	7.947
LINC02016	4.723	4.805	4.924	4.944	4.967	5.109
TDH	6.996	7.286	7.192	6.823	7.086	7.075
AC136621.1	7.926	7.925	8.054	8.204	8.104	7.865
KRTAP11-1	8.077	8.167	8.044	8.290	8.313	8.154
TDG	9.470	9.641	9.619	9.587	9.545	9.654
SCAMP1-AS1	9.072	8.873	9.004	8.829	8.789	8.756
CRPPA-AS1	4.627	4.845	4.853	4.799	4.839	4.864
ENTPD6	9.979	10.081	10.189	9.822	9.885	9.868
AL157700.1	5.208	5.488	5.425	5.359	5.208	5.336
LOXHD1	6.579	6.486	6.541	6.751	6.719	6.654
LRRC25	8.979	8.549	8.343	9.182	9.053	8.925
KIAA1522	11.049	11.069	11.177	11.037	10.929	11.064
MPDU1	9.934	10.065	10.190	9.865	9.953	9.866
LRRC20	8.872	8.876	9.076	8.857	8.782	8.693
AC026422.1	4.053	4.008	3.967	4.309	3.788	3.921
LRRC29	7.369	7.578	7.747	7.650	7.579	7.582
LRRC28	9.756	9.620	9.779	9.649	9.663	9.657
AC024560.1	8.765	8.604	8.668	8.538	8.791	8.736
GCNT4	6.686	6.509	6.383	6.935	6.726	6.771
AC024560.3	7.003	6.663	6.671	7.053	7.192	7.025
AC020663.5	8.346	8.389	8.363	8.574	8.442	8.548
AC024560.4	9.390	9.411	9.316	9.060	9.420	9.381
GCNT3	11.021	11.078	11.204	11.060	11.022	11.029
GCNT2	8.646	8.786	8.808	8.423	8.567	8.640
MBD3L2B	6.806	6.936	7.361	6.869	6.923	7.036
LPCAT4	7.682	7.698	7.818	7.537	7.638	7.781
LPCAT2	6.529	6.381	6.435	6.540	6.370	6.606
LPCAT3	8.566	8.615	8.558	8.303	8.460	8.410
LPCAT1	9.420	9.554	9.617	9.353	9.155	9.544
AL391832.2	6.545	6.577	6.632	6.558	6.952	6.831
AP005205.1	5.601	5.896	5.739	5.995	6.038	6.116
Z97989.1	5.565	5.484	5.644	6.077	5.621	5.710
TAGLN	8.456	8.361	8.450	8.499	8.629	8.569
CCDC178	8.048	7.765	7.633	7.825	8.192	8.233
SNAPC3	7.931	8.044	8.016	7.939	7.780	7.951
ALPP	8.175	8.176	8.223	8.123	8.205	8.047
CCDC170	6.573	6.631	6.264	6.594	6.763	6.529
CCDC171	5.575	5.336	5.737	5.513	5.667	5.459
CCDC172	6.575	6.804	6.385	6.764	6.607	6.800
CCDC173	5.484	5.395	5.372	5.630	5.505	5.570
CCDC174	8.749	8.645	8.700	8.514	8.739	8.590

CCDC177	6.909	6.795	6.705	6.818	6.939	6.778
PDK1	9.438	9.526	9.562	9.064	9.037	9.240
PDK3	9.408	9.472	9.468	9.021	9.246	9.132
PDK2	8.650	8.852	8.835	8.417	8.646	8.627
ALPG	8.175	8.176	8.223	8.123	8.205	8.047
PDK4	7.479	7.481	7.624	7.209	7.447	7.432
LNPEP	10.189	10.334	10.230	9.990	10.181	9.817
ALPI	6.100	5.981	6.298	6.256	6.455	6.257
SEC61A1	12.022	12.188	12.266	12.133	12.235	12.038
SEC61A2	6.893	7.198	6.966	7.083	7.072	7.071
ALPL	7.637	7.497	7.698	7.974	7.919	7.743
PECR	9.845	9.834	10.028	9.835	9.985	9.795
PKP4P1	10.228	10.310	10.380	10.226	10.097	10.215
PLA2G12AP1	10.193	10.193	10.245	10.181	10.182	10.196
RN7SKP150	6.707	6.575	6.364	6.485	6.292	6.649
LINC01091	5.386	5.752	5.401	5.576	5.726	5.655
LINC01093	4.433	4.566	4.443	4.355	4.496	4.382
LINC01095	5.339	5.359	5.544	5.541	5.645	5.831
LINC01094	4.394	4.273	4.222	4.552	4.251	4.415
LINC01097	4.946	5.002	4.761	5.039	4.779	5.010
LINC01096	5.636	5.747	5.615	5.811	5.654	5.828
LINC01098	4.092	4.308	4.437	4.437	4.374	4.458
CTAGE11P	5.576	5.499	5.349	5.691	5.724	5.752
HMGN2P17	12.569	12.690	12.791	12.495	12.553	12.522
DDIT3	10.350	10.161	10.485	10.437	10.348	10.271
DDIT4	12.396	12.388	12.594	12.338	12.332	12.284
RIIAD1	7.893	7.785	8.012	8.108	8.030	7.846
AC022137.1	4.235	4.271	4.237	4.410	4.594	4.484
MRAP-AS1	6.884	6.832	6.712	6.789	6.799	6.967
GOLGA8T	8.279	8.162	8.137	8.002	8.055	8.233
GOLGA8Q	7.048	6.865	6.859	7.005	7.107	7.121
GOLGA8S	8.279	8.162	8.137	8.002	8.055	8.233
GOLGA8O	8.279	8.162	8.137	8.002	8.055	8.233
GOLGA8N	8.279	8.162	8.137	8.002	8.055	8.233
GOLGA8J	8.279	8.162	8.137	8.002	8.055	8.233
GOLGA8G	6.446	6.294	6.356	6.148	6.123	6.590
GOLGA8F	6.446	6.294	6.356	6.148	6.123	6.590
GOLGA8A	8.958	8.764	8.446	8.529	8.355	8.894
GOLGA8B	8.958	8.764	8.446	8.529	8.355	8.894
CT45A9	5.629	5.540	5.552	5.723	5.686	5.660
CT45A8	5.629	5.540	5.552	5.723	5.686	5.660
CT45A1	5.629	5.540	5.552	5.723	5.686	5.660
CT45A3	5.629	5.540	5.552	5.723	5.686	5.660
CT45A2	5.629	5.540	5.552	5.723	5.686	5.660
CT45A5	5.629	5.540	5.552	5.723	5.686	5.660
CT45A7	5.629	5.540	5.552	5.723	5.686	5.660
CT45A6	5.629	5.540	5.552	5.723	5.686	5.660
FTCDNL1	5.440	5.623	5.629	5.711	5.820	5.406
VMO1	6.881	6.854	6.936	7.204	6.969	6.940
AC017071.1	5.279	5.154	5.061	5.202	5.151	5.264
AC011369.2	4.176	4.085	4.012	4.093	4.124	4.018
NPC2	12.289	12.484	12.553	12.350	12.170	12.295
NPC1	9.659	9.690	9.845	9.659	9.641	9.652
GATA3-AS1	6.716	6.524	6.481	6.872	6.717	6.622
LINC00324	6.504	6.442	6.528	6.639	6.762	6.861
LINC00326	10.595	10.647	10.883	10.696	10.848	10.797

LINC00327	5.489	5.528	5.609	5.784	5.763	5.710
RAB3GAP2	9.900	10.220	10.274	10.172	10.159	9.766
RAB3GAP1	11.466	11.617	11.742	11.484	11.633	11.306
C3orf52	8.116	7.971	8.260	7.936	7.970	7.935
C3orf56	5.096	5.192	5.203	5.450	5.128	5.156
DAG1	11.031	11.014	11.101	10.871	10.746	10.789
ETHE1	10.382	10.508	10.734	10.429	10.467	10.457
SGSM3	8.411	8.576	8.656	8.407	8.580	8.498
KDM1A	9.666	9.704	9.873	9.690	9.627	9.575
KDM1B	6.656	7.209	7.144	6.872	7.017	7.198
CIAO2A	11.794	11.866	11.937	11.810	11.834	11.802
CIAO2B	11.228	11.291	11.506	11.334	11.364	11.278
PRKCQ	8.032	8.024	8.135	7.996	8.261	8.278
AC012313.3	6.625	6.663	6.769	6.609	6.870	6.676
AC012313.5	6.580	6.404	6.521	6.542	6.597	6.757
AC012313.6	6.957	6.959	6.935	6.939	6.956	6.894
AC012313.9	8.014	8.032	8.060	7.996	8.040	7.876
FCER1G	7.545	7.556	7.300	7.574	7.536	7.582
BTG4	6.698	6.621	6.469	6.637	6.508	6.717
BTG3	10.214	10.359	10.478	10.019	10.013	9.917
BTG2	7.842	7.858	7.785	7.869	7.985	8.060
BTG1	12.865	12.887	13.212	12.870	12.907	12.489
SRD5A3	11.286	11.463	11.505	11.313	11.282	11.309
SRD5A2	7.314	7.353	7.336	7.417	7.516	7.249
SRD5A1	8.867	8.829	8.777	8.559	8.629	8.718
AC009963.2	4.276	4.375	4.369	4.157	4.349	4.491
ARMT1	9.628	9.730	9.753	9.477	9.498	9.475
AC069224.2	5.984	5.922	5.736	5.771	6.161	5.896
AL359881.3	7.746	7.516	7.332	7.547	7.559	7.623
GGA1	11.043	11.103	11.062	10.795	10.758	10.920
GGA3	9.021	8.984	9.009	9.030	9.064	8.862
GGA2	9.759	9.863	9.875	9.815	9.861	9.729
FHOD1	8.409	8.455	8.496	8.519	8.341	8.305
FHOD3	6.324	6.245	6.239	6.381	6.223	6.206
GJC2	8.152	8.297	8.393	8.355	8.457	8.320
C4orf3	11.253	11.358	11.544	11.362	11.382	11.333
GJC1	9.470	9.473	9.581	9.153	9.386	9.383
PSMB9	10.074	10.078	10.223	10.079	10.184	9.902
PSMB8	10.440	10.484	10.580	10.414	10.488	10.383
PSMB7	11.360	11.406	11.547	11.275	11.244	11.284
PSMB6	11.144	11.234	11.385	11.076	10.939	10.989
PSMB5	11.419	11.472	11.610	11.276	11.247	11.234
C14orf119	10.384	10.400	10.540	10.593	10.417	10.638
PSMB3	13.034	13.114	13.211	13.094	12.978	13.111
PSMB2	12.376	12.378	12.519	12.369	12.262	12.304
PSMB1	11.221	11.305	11.444	11.270	11.155	11.301
LMBR1L	8.442	8.394	8.351	8.682	8.590	8.610
TRAV23DV6	4.471	4.056	4.379	4.459	4.449	4.607
TDGF1	7.201	7.200	7.349	7.310	7.139	7.101
AC132192.2	6.542	6.724	6.882	6.929	6.633	6.952
ART4	6.076	6.129	6.096	6.496	6.182	6.145
SETP4	11.533	11.382	11.645	11.005	11.381	10.837
LTN1	8.400	8.426	8.231	8.130	8.115	8.237
TRIML2	6.148	6.508	6.726	6.445	6.671	6.606
TRIML1	5.872	5.282	5.923	5.952	5.863	5.968
NACC2	8.539	8.584	8.653	8.233	8.226	8.326

VPS13D	8.882	8.978	8.948	8.778	8.717	8.789
NACC1	9.260	9.118	9.278	9.214	9.449	9.169
VPS13A	8.059	8.029	8.036	7.746	7.592	7.903
SH3KBP1	12.345	12.308	12.412	12.271	12.494	12.335
VPS13C	10.983	10.951	10.873	10.497	10.433	10.680
VPS13B	7.910	7.714	7.863	7.983	7.797	7.759
BEX5	8.805	8.823	8.915	8.695	8.679	8.698
BEX4	4.222	4.388	4.386	4.656	4.397	4.397
BEX3	12.920	12.957	13.050	12.865	12.863	12.837
BEX2	9.098	9.008	9.062	8.766	8.647	8.655
BEX1	9.131	9.210	9.280	8.783	8.825	8.857
TSR2	9.611	9.513	9.643	9.440	9.462	9.265
TMEM160	9.366	9.400	9.745	8.960	9.013	8.945
TMEM163	6.810	7.004	7.110	6.837	6.958	7.098
TMEM165	11.030	11.114	11.152	11.358	11.309	11.232
TMEM164	10.223	10.231	10.316	10.201	10.115	10.178
EVI2A	4.699	4.646	4.640	4.833	4.674	4.784
TMEM168	8.555	8.295	8.522	8.117	8.334	8.257
EVI2B	5.306	5.158	5.526	5.198	5.277	5.226
FOXN1	7.286	7.202	7.157	7.000	7.204	7.073
AC079780.1	8.338	8.471	8.402	8.305	8.379	8.291
FOXN3	10.296	9.913	9.383	10.165	10.181	10.140
NICN1	8.618	8.583	8.676	8.278	8.456	8.463
COL14A1	7.221	7.099	7.160	7.106	7.397	7.133
MEMO1P1	10.241	10.250	10.344	10.169	10.153	10.173
AC093627.21	7.823	7.854	7.896	7.871	7.945	7.743
AC093627.27	5.629	5.637	5.650	5.415	5.501	5.815
IFFO1	10.057	10.101	10.049	10.135	10.005	10.042
IFFO2	7.573	7.436	7.559	7.522	7.492	7.584
AC109466.1	7.158	7.280	7.321	7.577	7.347	7.666
AL590064.1	6.125	6.168	6.328	5.885	6.084	5.980
SNAP91	5.867	5.752	5.541	5.570	5.653	5.810
AC090559.2	4.628	4.592	4.511	4.654	4.478	4.566
AC138827.2	6.226	5.977	5.916	5.792	6.012	5.967
RPL15P3	12.772	12.801	12.947	12.791	12.625	12.834
RPL15P2	12.772	12.801	12.947	12.791	12.625	12.834
AL121762.3	6.036	6.161	6.015	6.233	6.245	6.182
SRP14	12.333	12.360	12.551	12.397	12.399	12.353
AC140481.4	5.675	5.822	5.573	6.014	5.852	5.828
MACROD1	8.512	8.406	8.685	8.200	8.315	8.319
AL672207.1	8.207	8.302	8.302	8.274	8.306	8.209
PPIL1	9.896	10.002	10.113	10.094	10.098	10.099
NALT1	7.653	7.867	7.910	7.650	7.791	7.927
AC217785.3	9.004	9.090	9.056	8.792	8.907	8.821
AC239618.2	9.332	9.504	9.629	9.337	9.422	9.489
AC013486.1	7.568	7.527	7.462	7.781	7.759	7.609
KRR1	9.693	9.595	9.796	9.453	9.632	9.414
NNT-AS1	8.450	8.509	8.768	8.300	8.393	8.414
STRN	8.406	8.344	8.572	8.157	8.357	8.205
AC091564.5	7.324	7.318	7.510	7.402	7.552	7.513
AC091564.7	12.304	12.367	12.561	12.612	12.530	12.410
AC091564.1	4.346	4.366	4.235	4.409	4.413	4.405
MTCL1	11.684	11.688	11.677	11.335	11.208	11.443
LINC01213	6.032	5.959	5.810	5.959	6.039	6.056
HMGN1P3	11.925	11.994	12.146	11.893	11.885	11.984
UGT2A1	4.611	4.675	4.703	4.711	4.782	4.727

AL117339.5	8.133	7.945	7.853	8.197	8.033	8.146
AL117339.4	6.983	7.024	6.923	7.051	7.128	7.081
TP53I3	9.437	9.510	9.592	9.393	9.282	9.444
AC009136.1	7.341	7.520	7.356	7.793	7.580	7.467
MB	6.829	6.799	7.040	6.913	6.790	6.892
DUX4L7	8.052	7.820	8.258	8.487	8.319	8.298
DUX4L6	8.052	7.820	8.258	8.487	8.319	8.298
DUX4L5	8.052	7.820	8.258	8.487	8.319	8.298
DUX4L4	8.052	7.820	8.258	8.487	8.319	8.298
DUX4L3	8.052	7.820	8.258	8.487	8.319	8.298
DUX4L2	8.052	7.820	8.258	8.487	8.319	8.298
DUX4L1	8.052	7.820	8.258	8.487	8.319	8.298
SLC18A3	7.650	7.391	7.282	7.509	7.587	7.608
SLC18A2	6.850	6.987	6.980	6.609	6.817	6.864
SLC18A1	5.744	5.879	5.580	6.026	5.733	5.927
DUX4L9	8.052	7.820	8.258	8.487	8.319	8.298
DUX4L8	8.052	7.820	8.258	8.487	8.319	8.298
CU634019.2	7.923	7.782	7.902	7.606	7.461	7.712
CU634019.6	7.457	7.458	7.194	7.008	7.081	7.575
RALBP1	10.881	10.881	10.994	10.572	10.622	10.602
CU634019.4	4.107	4.171	4.060	4.133	4.131	4.177
B3GALT5-AS1	6.600	6.669	6.899	6.826	6.779	6.773
ZNF831	5.937	5.762	5.653	5.901	5.883	5.816
ZNF830	9.094	9.090	9.257	9.146	9.174	9.010
ZNF835	7.410	7.598	7.492	7.541	7.718	7.625
ZNF837	7.634	7.695	7.749	7.902	7.927	7.828
ZNF836	8.918	8.882	8.807	8.710	8.711	8.867
ZNF839	8.010	8.027	8.089	7.950	8.105	8.027
AC004949.1	4.608	4.687	4.720	4.574	4.608	4.758
AC037198.1	7.132	7.187	6.945	7.201	7.137	7.078
OXCT2P1	7.449	7.489	7.485	7.502	7.464	7.555
FAM91A1	11.082	11.129	11.138	10.938	10.962	11.106
CLYBL-AS2	4.597	4.576	4.169	4.504	4.261	4.465
ZNF574	9.100	9.118	9.300	9.277	9.241	9.182
TUNAR	6.229	6.215	6.242	6.076	6.192	6.340
SUGCT	6.979	6.918	6.790	7.272	6.918	6.918
TSPY8	8.003	7.967	8.011	8.092	8.024	8.064
CPEB2	7.762	7.930	7.746	8.067	8.167	8.162
TSPY3	8.003	7.967	8.011	8.092	8.024	8.064
TSPY2	8.003	7.967	8.011	8.092	8.024	8.064
TSPY1	4.547	4.525	4.638	4.840	4.849	4.586
RAB33A	5.673	5.859	5.732	5.651	5.956	5.827
SLC17A9	9.401	9.275	9.467	9.437	9.460	9.337
RAB33B	7.859	7.773	7.828	7.626	7.829	7.744
SLC17A4	8.014	7.823	7.723	7.725	7.822	7.799
SLC17A5	9.693	9.828	9.830	9.869	9.959	9.733
SLC17A6	4.854	5.103	5.014	4.980	5.157	5.188
SLC17A7	8.169	8.338	8.099	8.249	8.307	8.303
SLC17A1	7.675	7.743	7.643	8.020	8.080	8.078
SLC17A2	7.039	6.837	6.990	7.259	7.386	7.183
SLC17A3	6.509	6.391	6.361	6.595	6.784	6.592
C16orf58	10.445	10.423	10.562	10.229	10.269	10.247
TMEFF1	4.923	5.117	5.248	4.928	4.937	5.062
WASIR1	4.455	4.473	4.535	4.391	4.421	4.784
C16orf54	3.914	3.855	3.748	3.880	3.911	3.751
AP5B1	7.273	7.253	7.254	7.123	7.215	7.064

CHMP4A	10.032	10.162	10.219	9.727	9.880	9.921
CHMP4B	12.143	12.308	12.349	12.086	11.968	11.931
CHMP4C	9.792	9.876	9.963	9.644	9.747	9.663
COBLL1	9.590	9.595	9.680	9.091	9.232	9.173
AC006008.1	5.698	6.085	5.712	5.740	5.944	6.027
TMEM255B	7.125	7.021	7.116	7.509	7.038	7.197
TMEM255A	3.773	4.032	3.693	3.858	3.775	3.805
ST7	9.122	9.031	9.079	8.886	9.213	9.139
GU182345.2	8.835	8.689	8.488	8.744	8.793	8.879
PRKAR1AP1	7.563	7.546	7.499	7.580	7.881	7.810
STH	7.468	7.869	7.843	7.685	7.483	7.734
ZNF302	7.890	7.747	8.000	7.572	7.763	7.790
ZNF300	7.596	7.511	7.486	7.334	7.032	7.554
STS	9.568	9.628	9.749	9.401	9.419	9.544
HOXB13	7.820	7.593	7.890	7.844	7.772	7.899
TP53TG1	8.383	8.438	8.538	8.539	8.515	8.583
FBXO17	9.219	8.907	9.153	9.183	8.859	9.200
PPP3CA	10.215	10.148	10.321	10.102	10.244	10.099
PPP3CB	8.881	8.825	8.987	8.778	8.585	8.895
PPP3CC	7.267	7.491	7.328	7.177	7.283	7.160
LIF	11.486	11.481	11.560	11.545	11.381	11.347
GPATCH8	9.091	9.130	9.120	9.023	9.088	9.064
RPL13	14.391	14.386	14.416	14.405	14.340	14.364
NPY6R	7.891	7.919	7.677	7.986	7.927	8.142
RPL18	13.855	13.899	13.977	13.859	13.838	13.887
AC079140.6	6.727	6.475	6.204	6.663	6.730	6.589
ADRA2A	8.916	9.003	9.217	8.833	9.044	8.998
ADRA2B	7.837	7.723	7.760	7.696	7.945	7.900
ADRA2C	7.919	7.947	8.057	8.311	8.059	8.011
TEX26	5.231	5.312	5.107	5.348	5.429	5.288
TEX22	8.392	8.364	8.348	8.345	8.321	8.426
TEX28	5.842	5.912	6.019	6.408	6.090	6.168
TEX29	6.787	6.781	6.831	6.716	6.630	6.692
AC024022.1	6.384	6.233	6.095	6.570	6.429	6.496
SYT8	7.100	7.122	7.450	7.334	7.415	7.508
LINC01252	6.287	6.317	6.265	6.194	6.296	6.370
LINC01257	4.873	4.993	4.851	5.174	4.954	5.011
MTCH2	10.966	11.082	11.157	10.857	10.789	10.876
MTCH1	12.585	12.674	12.764	12.580	12.477	12.551
LINC01254	4.389	4.531	4.514	4.329	4.453	4.561
SYT1	6.604	6.504	6.579	6.319	6.703	6.915
SYT2	9.066	8.917	8.795	9.082	9.016	8.889
SYT3	7.109	7.206	7.106	7.421	7.183	7.258
SYT4	6.506	6.717	6.421	6.310	6.433	6.440
SYT5	7.396	7.124	7.210	7.377	7.468	7.336
SYT6	6.911	7.002	6.831	7.080	7.067	6.994
SYT7	8.143	8.140	8.157	8.372	8.113	8.130
AL359757.2	6.013	5.977	5.881	6.306	6.274	6.177
TIAL1	10.433	10.411	10.605	10.507	10.378	10.470
FUBP3	9.124	9.296	9.165	8.932	8.975	9.248
FUBP1	9.802	9.826	9.880	9.773	9.630	9.760
IGKV1-17	6.685	6.554	6.707	6.553	6.594	6.673
SCHIP1	8.267	8.417	8.572	8.161	8.496	8.409
AC073367.1	5.339	5.384	5.384	5.363	5.404	5.459
AC097537.1	5.784	5.606	5.438	5.569	5.772	5.821
MARCO	6.323	6.413	6.465	6.673	6.389	6.631

AC004223.3	7.997	8.075	7.967	7.969	8.117	8.028
NBL1	11.811	11.865	12.049	11.714	11.822	11.734
HSFX2	6.429	6.476	6.702	6.417	6.226	6.277
HSFX3	6.481	6.562	6.345	6.278	6.650	6.533
HSFX1	6.429	6.476	6.702	6.417	6.226	6.277
HSFX4	6.481	6.562	6.345	6.278	6.650	6.533
DRC7	7.451	7.376	7.558	7.458	7.618	7.847
DRC3	6.763	6.875	6.606	6.840	6.897	6.877
DRC1	8.083	8.298	8.016	8.115	8.167	8.212
C1orf53	8.568	8.536	8.712	7.996	8.431	8.218
C1orf52	9.705	9.627	9.724	9.667	9.704	9.708
C1orf50	8.265	8.335	8.466	8.039	8.329	8.224
C1orf56	8.708	8.581	8.758	8.565	8.608	8.569
C1orf54	7.029	7.359	7.105	7.108	7.096	7.325
MARC1	9.713	9.585	9.645	9.574	9.564	9.643
MARC2	8.656	8.742	8.798	8.642	8.570	8.682
RPGRIP1	7.230	7.114	7.195	7.279	7.353	7.392
SMPD4	9.394	9.406	9.580	9.048	8.942	9.120
SMPD1	9.357	9.428	9.587	9.248	9.301	9.266
SMPD3	7.240	7.335	7.521	7.429	7.417	7.257
SMPD2	8.581	8.602	8.747	8.419	8.495	8.613
BRIP1	8.325	8.483	8.665	7.827	8.042	8.026
PDE11A	7.050	6.967	6.886	7.162	7.142	6.823
TTC28	7.961	7.892	7.805	7.555	7.647	7.853
TTC29	4.375	4.161	4.161	4.563	4.466	4.580
TTC25	5.625	5.514	5.542	5.588	5.615	5.594
TTC26	7.365	7.549	7.684	7.241	7.220	7.432
TTC27	8.875	8.964	9.080	8.812	8.794	8.738
PATL2	6.001	6.227	6.157	6.129	6.198	5.868
TTC22	9.566	9.491	9.667	9.392	9.586	9.461
PATL1	10.276	10.356	10.233	10.196	10.231	10.075
AL031119.1	4.087	4.058	3.815	4.000	4.081	3.998
DAAM2-AS1	5.974	5.981	6.153	6.190	6.145	6.076
ENO1	13.380	13.427	13.534	13.379	13.305	13.389
ENO2	9.368	9.536	9.613	9.357	9.298	9.397
ENO3	6.647	6.729	6.722	6.611	6.696	6.697
AC005224.3	7.086	7.003	6.773	7.307	7.107	7.110
OIP5-AS1	9.870	9.936	9.892	9.694	9.612	9.810
CFD	7.478	7.302	7.670	7.330	7.462	7.289
OBSCN	8.638	8.652	8.792	8.660	8.486	8.774
Z82195.2	5.709	5.871	5.546	5.726	5.730	5.776
SRRD	8.955	8.975	8.983	8.842	8.768	8.885
MCL1	11.992	12.041	12.124	12.015	12.007	12.071
SORBS1	8.579	8.520	8.274	8.743	8.447	8.621
SORBS3	9.793	9.754	10.111	9.893	9.803	9.773
SORBS2	9.723	9.870	10.007	9.728	9.670	9.523
ODC1	11.425	11.507	11.681	11.495	11.456	11.485
IL18RAP	6.901	6.994	7.182	7.247	7.216	7.048
EOMES	7.479	7.448	7.584	7.564	7.411	7.447
BCDIN3D-AS1	6.311	6.358	6.124	6.282	6.421	6.474
AP006285.3	7.447	7.425	7.491	7.568	7.606	7.459
AL772307.1	8.174	8.070	8.261	8.185	8.174	8.173
AP006285.1	7.447	7.425	7.491	7.568	7.606	7.459
CGB1	7.556	7.653	7.596	8.049	7.638	7.796
PDSS1	8.324	8.348	8.528	7.996	8.094	8.024
AL691432.2	7.039	7.081	6.964	6.837	7.109	7.127

FKBP14	8.143	8.136	8.263	8.011	8.245	8.097
FKBP15	9.358	9.276	9.324	8.964	9.185	9.175
UBE2J1	8.964	9.094	9.101	9.019	9.018	9.046
FKBP10	7.619	7.696	7.810	7.499	7.725	7.700
FKBP11	8.333	8.464	8.585	8.424	8.465	8.336
FBXO10	7.801	7.893	7.849	7.755	7.926	8.027
FBXO11	9.418	9.420	9.416	9.098	9.321	9.480
FBXO16	10.119	10.242	10.446	10.127	10.442	9.782
AC068870.1	7.189	7.230	6.988	7.503	7.424	7.338
FBXO15	7.287	7.460	7.260	7.467	7.458	7.358
SFTA3	4.229	4.140	4.229	4.208	4.335	4.296
AC010247.1	6.527	6.635	6.563	6.876	6.235	6.728
AC010247.2	7.838	7.956	7.847	7.978	8.216	8.138
CNGB1	6.886	7.026	7.184	7.392	7.110	7.119
CNGB3	6.380	6.166	6.028	6.466	6.526	6.346
BCLAF1	11.209	11.254	11.241	11.094	10.995	11.093
BCLAF3	7.155	7.351	7.313	7.114	7.122	7.185
CTXND1	7.435	7.555	7.700	8.046	7.697	7.639
PLA2G7	5.093	4.935	5.231	5.046	4.919	5.120
PLA2G5	7.723	7.928	7.838	7.813	8.034	7.975
PLA2G3	8.443	8.204	8.126	8.424	8.344	8.431
GDPGP1	5.934	6.064	5.649	5.820	5.975	5.994
CCDC77	7.298	7.286	7.365	7.008	6.748	6.950
KRTAP1-3	7.890	8.220	8.283	8.190	8.472	8.212
KRTAP1-5	6.977	6.956	6.965	6.904	7.014	6.971
CCDC70	6.619	6.498	6.600	6.942	6.928	6.752
AC016831.6	7.675	7.498	7.462	7.894	7.905	7.759
AC016831.5	7.216	7.238	7.228	7.050	7.269	7.285
AC016831.4	6.884	6.960	6.496	6.984	7.153	7.102
AC016831.1	7.675	7.498	7.462	7.894	7.905	7.759
GHITM	11.424	11.486	11.556	11.429	11.348	11.404
CSHL1	7.785	7.620	7.627	7.744	7.836	7.768
FKBP1B	8.364	8.176	8.377	8.191	8.165	8.244
FKBP1C	12.103	12.236	12.339	12.120	12.137	12.132
LINC02193	4.095	4.251	4.353	3.909	4.106	4.174
FKBP1A	12.103	12.236	12.339	12.120	12.137	12.132
LINC02199	6.761	6.976	6.751	6.772	6.817	7.258
LINC02223	5.973	5.965	5.846	5.936	5.997	6.077
LINC02221	4.286	4.275	4.387	4.901	4.494	4.356
LINC02226	7.524	7.584	7.464	7.524	7.682	7.701
LINC02225	4.352	4.194	4.401	4.550	4.235	4.458
MAP11	8.331	8.397	8.397	8.451	8.524	8.405
ELFN1-AS1	8.053	7.992	8.040	8.028	8.175	8.167
UNC50	9.755	9.758	9.904	9.775	9.893	9.852
NKAIN4	7.221	7.114	7.340	7.373	7.303	7.367
NKAIN3	5.870	5.786	5.959	5.917	5.917	5.755
NKAIN2	5.887	6.151	5.680	6.002	6.143	6.099
NKAIN1	7.266	7.163	7.213	7.312	7.631	7.410
AL356740.2	7.024	6.967	6.943	7.028	7.233	7.205
AL356740.3	7.024	6.967	6.943	7.028	7.233	7.205
MAP1B	7.244	7.308	7.331	7.208	7.389	7.231
IGSF9B	6.192	6.149	5.918	6.349	6.199	6.347
AL133482.1	7.146	7.354	7.289	7.343	7.575	7.497
UNC5B	8.759	8.661	8.729	8.742	8.848	8.812
UNC5C	6.616	6.823	6.935	6.991	6.866	6.977
AC013652.1	5.852	5.814	5.941	5.883	5.906	5.930

UNC5A	9.800	9.819	9.872	9.533	9.481	9.507
UNC5D	5.006	4.797	4.786	4.987	4.865	4.901
MAP1S	8.660	8.554	8.755	8.502	8.709	8.482
CCP110	8.337	8.280	8.269	8.011	8.073	8.189
ANKRD19P	7.512	7.495	7.668	7.549	7.815	7.555
ERLNC1	8.919	8.981	8.817	8.751	8.984	8.919
RPL18A	13.186	13.284	13.431	13.346	13.253	13.366
VDAC1P1	11.673	11.745	11.859	11.695	11.598	11.659
VDAC1P3	11.673	11.745	11.859	11.695	11.598	11.659
VDAC1P6	11.673	11.745	11.859	11.695	11.598	11.659
CLEC18A	5.972	6.568	6.142	6.006	6.214	6.333
CEND1	8.459	8.523	8.559	8.495	8.474	8.461
CLEC18B	5.972	6.568	6.142	6.006	6.214	6.333
BORCS7	9.066	8.972	9.074	8.295	8.433	8.571
BORCS6	8.473	8.516	8.624	8.732	8.760	8.529
BORCS5	7.593	7.861	7.948	7.657	7.820	7.695
TARDBPP1	4.997	5.137	5.272	5.295	5.378	5.268
BORCS8	8.906	9.056	9.003	8.617	8.750	8.590
SDR42E1	8.504	8.638	8.617	8.481	8.260	8.392
LINC01753	7.406	7.730	7.526	7.597	7.961	7.562
LINC01750	5.731	5.730	5.716	5.711	5.930	5.804
LINC01751	7.479	7.648	7.473	7.504	7.729	7.700
LINC01756	7.440	7.410	7.369	7.168	7.451	7.449
EPB41L1	10.033	10.183	10.105	10.062	10.049	9.873
PPCDC	8.759	8.868	9.080	8.407	8.594	8.520
EPB41L3	5.558	5.301	5.439	5.529	5.322	5.490
EPB41L2	9.490	9.474	9.568	9.234	9.306	9.322
EPB41L5	9.343	9.237	9.414	9.073	9.268	9.228
ACSS3	6.585	6.871	6.630	6.499	6.742	6.665
ACSS2	10.099	10.146	10.285	10.139	10.103	10.070
ACSS1	7.735	7.713	7.771	7.921	7.587	7.878
ABCA12	6.913	6.687	6.815	6.741	6.548	6.479
PUDP	10.436	10.484	10.571	10.418	10.398	10.349
PCOLCE2	7.342	7.518	7.589	6.952	7.204	7.116
MAGEH1	5.403	5.366	5.664	5.511	5.555	5.539
WNT3	8.154	8.032	7.996	8.002	8.110	8.040
WNT2	6.814	6.677	6.613	6.790	6.648	6.841
WNT1	6.668	6.674	6.559	7.050	6.914	6.885
LRRN4	6.203	6.624	6.506	6.642	6.484	6.499
AGXT	7.314	7.400	7.484	7.483	7.604	7.513
LRRN2	7.014	7.167	7.051	7.061	7.270	7.276
LRRN1	5.070	5.285	5.345	5.308	5.226	5.356
WNT4	7.715	7.736	7.569	7.743	7.944	7.857
AL139327.1	4.603	4.747	4.715	4.750	4.596	4.829
LAMA5-AS1	7.070	6.985	7.068	7.195	6.893	7.017
AC098850.3	8.663	8.479	8.503	8.310	8.228	8.678
AL049869.3	6.277	6.190	6.231	6.441	6.321	6.371
AL049869.1	9.129	9.158	9.232	9.007	8.933	9.090
HOXA11-AS	6.399	6.341	6.348	6.633	6.531	6.496
RNF175	5.885	5.931	5.916	5.768	5.906	5.907
RNF170	8.802	8.936	9.058	8.735	8.602	8.726
AC243734.1	6.743	6.757	6.617	6.803	6.702	6.551
ASAP1-IT2	4.949	4.896	4.832	4.883	4.852	4.695
CHROMR	6.948	6.995	6.850	6.684	6.812	6.547
PCNA	11.185	11.276	11.360	11.192	11.106	11.143
ZBTB7A	9.660	9.823	9.879	9.939	9.972	9.883

ZBTB7C	7.484	7.414	7.518	7.315	7.541	7.508
ZBTB7B	10.022	9.992	10.161	10.217	10.274	10.136
PCNP	10.472	10.420	10.538	10.396	10.282	10.427
PCNT	9.225	9.126	9.214	9.010	8.915	9.022
STIMATE	8.327	8.412	8.411	8.618	8.463	8.412
UTY	8.316	8.325	8.338	8.224	8.451	8.367
PCAT1	4.500	4.470	4.461	4.399	4.556	4.502
PACRG	7.124	6.874	7.171	7.300	7.137	7.263
PCAT4	8.271	8.229	7.949	8.096	8.351	8.378
AC009022.1	7.559	7.552	7.405	7.429	7.405	7.683
PCAT6	7.166	7.419	7.431	7.386	7.227	7.474
DNPH1	9.503	9.590	9.755	9.445	9.487	9.300
AC093627.4	5.629	5.637	5.650	5.415	5.501	5.815
AC093627.9	7.823	7.854	7.896	7.871	7.945	7.743
ILF3	11.018	11.053	11.057	10.767	10.736	10.890
ILF2	11.027	11.156	11.307	11.011	11.041	11.052
GOLGA2P10	8.875	8.968	8.987	8.777	8.722	8.806
AC024267.5	4.286	4.319	4.405	4.513	4.330	4.336
BOK	10.091	10.165	10.371	9.944	9.886	9.984
BOC	8.085	8.231	8.269	8.244	8.420	8.266
DENND2B	8.726	8.795	8.889	8.572	8.742	8.674
DENND2C	7.531	7.671	7.663	7.830	7.930	7.554
DENND2A	6.922	6.908	6.903	6.709	6.931	6.975
DENND2D	8.952	9.018	9.027	8.870	9.081	8.932
GAGE1	7.444	7.371	7.139	7.545	7.560	7.476
PPT1	10.590	10.763	10.830	10.587	10.572	10.541
PPT2	9.420	9.348	9.490	9.387	9.471	9.391
INPP4B	9.748	9.798	9.845	9.535	9.436	9.467
INPP4A	7.998	7.968	8.113	7.596	7.687	7.735
SAFB	9.007	9.113	9.292	9.110	8.937	8.978
TRNP1	11.990	12.168	12.244	12.047	12.080	12.072
IGHV5-78	4.456	4.724	4.497	4.383	4.525	4.513
AC245884.12	6.623	6.464	6.514	6.632	6.752	6.406
PPP1R26	8.564	8.661	8.597	8.450	8.543	8.455
PPP1R27	8.537	8.490	8.553	8.495	8.691	8.638
PPP1R21	8.162	8.221	8.209	7.797	7.822	7.933
NGLY1	8.888	9.010	9.036	8.654	8.722	8.677
MACO1	8.366	8.355	8.406	8.061	7.808	8.189
TRDN-AS1	5.334	5.329	5.178	5.567	5.601	5.628
LMX1A	6.394	6.168	6.070	6.606	6.340	6.569
ZCCHC24	7.201	7.615	7.549	7.398	7.651	7.426
LMX1B	7.195	7.387	7.291	7.504	7.556	7.553
MRGPRF	6.023	6.023	6.170	6.198	6.070	6.146
CEP83-DT	6.807	6.935	7.078	7.143	6.929	6.783
PPP1R2B	9.453	9.606	9.656	9.156	8.958	9.225
PPP1R2C	6.778	6.685	6.725	6.963	7.225	6.763
KCP	7.873	8.067	7.854	8.032	7.866	7.895
AC092053.3	7.413	6.974	7.260	7.782	7.604	7.341
AC092053.4	8.916	8.655	8.841	8.888	8.980	8.914
CNR1	7.416	7.463	7.246	7.237	7.206	7.340
GBX2	6.840	6.560	6.700	6.877	6.935	6.843
GBX1	7.849	7.792	7.958	7.979	7.870	8.008
CNR2	8.142	8.105	7.991	8.396	8.364	8.364
GNL3L	8.222	8.298	8.278	8.091	7.997	8.119
JARID2-AS1	7.078	7.327	7.297	7.414	7.378	7.352
PIGCP1	8.988	8.963	8.927	8.793	8.858	8.807

AP001282.2	8.875	8.960	8.994	8.306	8.827	8.368
AL355315.1	8.867	9.075	8.924	8.982	9.105	9.022
OTULIN	8.679	8.875	8.742	8.698	8.637	8.773
TGS1	8.494	8.578	8.562	8.455	8.437	8.410
ANKS1B	5.958	6.117	5.877	6.035	5.998	6.035
ANKS1A	9.372	9.378	9.321	9.039	9.060	9.135
BMT2	7.250	7.316	7.336	7.336	7.418	7.159
AC006206.1	8.261	8.148	8.137	8.285	8.277	8.327
CT476828.21	8.052	7.820	8.258	8.487	8.319	8.298
ADAM10	12.413	12.490	12.527	12.374	12.273	12.401
ADAM11	8.117	8.158	8.255	8.223	8.322	8.212
ADAM12	7.944	8.068	8.111	8.006	8.108	8.102
ADAM15	9.369	9.545	9.672	9.416	9.381	9.436
ADAM17	9.649	9.656	9.677	9.625	9.738	9.652
ADAM18	6.343	6.348	6.324	6.602	6.399	6.457
ADAM19	7.745	7.705	7.733	7.940	7.819	8.051
MEPE	8.033	8.074	8.120	8.117	8.217	8.164
HAGH	8.980	9.015	9.138	8.858	8.846	8.840
GLYAT	7.301	7.342	7.121	7.169	7.444	7.418
OPALIN	7.372	7.289	7.314	7.570	7.572	7.642
NEPRO	9.344	9.364	9.502	9.049	9.223	9.138
U2AF1L4	7.945	7.864	7.872	7.936	7.978	7.948
U2AF1L5	11.314	11.348	11.538	11.066	11.070	11.140
NDEL1	8.699	8.685	8.879	8.632	8.588	8.603
AC002398.2	7.992	8.039	8.260	7.791	7.776	7.858
SHANK3	5.960	5.927	6.230	6.517	6.240	6.459
SHANK2	10.069	10.161	10.218	9.840	9.908	9.855
SHANK1	7.207	7.286	7.318	7.552	7.250	7.301
ADAM1A	6.238	6.095	6.062	5.545	5.611	6.028
PLAC8L1	7.508	7.370	7.467	7.391	7.673	7.648
UCP2	9.671	9.688	9.839	9.476	9.539	9.480
PCSK9	10.318	10.385	10.565	10.209	10.155	10.315
LINC02210-CRHF	7.826	7.845	8.021	7.935	7.905	7.824
PCSK2	6.734	6.659	6.971	7.132	6.724	6.819
PCSK1	4.339	4.516	4.260	4.336	4.308	4.391
PCSK6	7.438	7.326	7.481	7.611	7.478	7.614
PCSK7	9.010	9.090	9.087	9.121	9.063	9.047
PCSK4	8.122	8.223	8.451	8.519	8.582	8.362
PCSK5	8.196	8.050	8.149	7.370	7.783	7.718
MAEA	9.916	10.062	10.065	9.646	9.597	9.835
TC2N	9.118	9.292	9.222	8.655	8.909	8.957
PLTP	6.515	6.647	6.645	6.833	6.752	6.735
FKBP9P1	11.203	11.183	11.321	11.147	11.146	11.053
DPYSL4	6.967	6.993	7.102	7.001	7.213	7.309
MIR6812	10.396	10.440	10.546	10.273	10.133	10.152
SETBP1	7.248	7.344	7.592	7.339	7.284	7.183
SAMSN1	6.289	6.137	5.985	6.258	6.255	6.196
AC135776.3	6.809	6.865	6.701	7.148	7.106	6.941
AC135776.1	6.809	6.865	6.701	7.148	7.106	6.941
FAM151A	7.537	7.610	7.629	7.593	7.525	7.603
TAAR3P	7.012	6.860	6.475	6.858	6.712	6.873
AL365400.2	4.670	4.678	4.965	4.948	4.801	4.839
PAXIP1	8.069	8.007	8.215	7.806	7.875	7.934
LDLRAD4-AS1	6.231	6.220	5.973	6.018	6.171	6.279
RPL13AP7	14.076	14.099	14.141	14.125	14.125	14.139
RPL13AP6	14.076	14.099	14.141	14.125	14.125	14.139

RPL13AP5	14.424	14.395	14.422	14.370	14.350	14.418
LINC02424	4.025	4.253	4.020	4.131	3.905	3.970
LINC02426	4.966	4.740	4.969	5.078	5.184	4.832
LINC02421	4.811	4.636	4.677	4.528	4.689	4.749
GPAT2P2	6.417	6.586	6.446	6.526	6.489	6.524
USE1	8.170	8.206	8.315	8.004	7.995	7.981
CNRIP1	7.230	7.313	7.371	7.557	7.493	7.485
Z97205.2	7.550	7.442	7.448	7.535	7.703	7.687
AL035417.1	5.205	5.197	4.914	5.127	4.904	5.076
SNORA63C	7.506	7.364	7.485	7.373	7.356	7.367
AL592148.3	6.559	6.622	6.694	6.663	6.578	6.750
AF274858.1	6.530	6.557	6.457	6.708	6.751	6.593
DTX1	7.508	7.635	7.653	7.701	7.685	7.720
CYP1B1-AS1	5.124	5.243	5.153	5.051	5.509	5.242
AC009021.2	7.156	7.159	6.911	7.124	6.867	7.203
ARMC10P1	9.444	9.534	9.588	9.472	9.496	9.373
CDC34	9.198	9.233	9.489	9.180	9.073	9.064
CDC37	10.734	10.744	10.921	10.687	10.478	10.660
OR2T35	6.846	6.574	6.640	6.713	6.771	6.677
AC027575.3	7.248	7.304	7.239	7.326	7.373	7.281
PROSER2-AS1	7.342	7.303	7.658	7.834	7.556	7.806
SPIN2A	7.577	7.355	7.865	7.625	7.587	7.439
SPIN2B	7.577	7.355	7.865	7.625	7.587	7.439
PGLS	10.872	10.927	11.103	10.727	10.813	10.745
LINC01198	5.352	5.471	5.265	5.284	5.424	5.506
LINC01197	6.080	6.412	6.107	5.847	6.114	6.085
LINC01191	6.950	6.794	6.823	6.849	6.943	6.982
AC009549.1	6.542	6.512	6.510	6.494	6.700	6.454
LINC01193	7.312	7.506	7.442	7.325	7.376	7.443
WDR7	9.751	9.769	9.763	9.287	9.283	9.219
CDC42-IT1	4.756	4.662	4.702	4.658	4.781	4.638
AC026403.1	14.563	14.550	14.541	14.502	14.503	14.523
CAPN10-DT	6.344	6.344	6.286	6.479	6.466	6.622
CCNA2	9.093	9.110	9.302	8.929	8.942	8.819
WDR1	11.731	11.788	11.857	11.714	11.592	11.698
CCNA1	6.302	6.436	6.219	6.339	6.324	6.302
DYNC1I1	7.419	7.297	7.190	7.635	7.651	7.565
VPS28	10.764	10.845	11.061	10.784	10.680	10.781
DYNC1I2	11.005	11.015	11.111	10.996	10.922	10.866
SKA1	6.754	6.403	6.891	6.019	6.307	6.107
SKA3	8.627	8.950	8.746	8.581	8.524	8.423
SKA2	10.330	10.216	10.293	9.870	10.099	9.872
SLC26A4-AS1	4.292	4.246	4.325	4.314	4.252	4.454
RN7SKP54	6.043	6.140	5.869	5.732	6.119	5.868
PCDH20	4.046	4.248	4.137	4.187	4.020	3.974
AC138915.2	5.861	5.785	5.791	6.273	6.001	6.099
AC090015.1	5.989	6.271	6.052	6.671	6.216	6.303
RNU6-703P	5.727	5.562	5.635	5.731	5.850	5.585
C6orf89	9.480	9.548	9.488	9.489	9.644	9.534
PPP1R13B	7.834	7.933	8.134	7.743	7.853	7.862
PPP1R13L	10.453	10.544	10.688	10.447	10.490	10.358
TMC5	11.635	11.878	11.969	11.929	12.028	11.123
AC007099.1	6.773	6.830	6.663	6.954	6.895	6.939
AC064805.1	9.976	10.060	10.013	10.037	10.084	10.050
AL357054.4	4.486	4.413	4.532	4.420	4.393	4.384
WNT10A	6.795	7.013	6.993	6.862	7.131	7.088

WNT10B	8.149	7.948	7.908	8.273	8.282	8.050
AC022414.1	7.116	7.308	7.458	7.083	7.233	7.238
AC104451.2	7.508	7.509	7.604	7.698	7.576	7.716
HOGA1	9.103	9.230	9.240	8.851	8.860	8.849
LPP-AS2	6.259	6.147	6.130	5.703	5.943	5.733
PRKAR2B	5.676	5.768	5.886	5.648	5.763	5.729
TM4SF18	7.768	7.906	7.642	7.848	7.697	7.803
FRG2EP	4.631	4.456	4.334	4.623	4.651	4.476
TMEM35B	9.802	9.869	9.954	9.753	9.826	9.864
TMEM35A	5.996	5.848	5.871	5.750	6.098	5.976
LAMB1	9.297	9.422	9.392	9.089	8.968	9.093
AC144831.1	7.559	7.638	7.729	7.812	7.686	7.628
LAMB2	9.227	9.167	9.325	8.689	8.720	8.909
ATP7A	8.410	8.404	8.655	8.574	8.606	8.315
LAMB4	6.820	6.570	6.773	6.979	6.887	6.929
ATP7B	8.837	8.840	8.718	8.139	8.426	8.395
MPHOSPH9	8.662	9.057	8.893	8.392	8.759	8.662
MPHOSPH8	8.603	8.510	8.616	8.225	8.454	8.401
AL606752.1	5.801	5.454	5.523	5.952	5.862	5.792
MPHOSPH6	9.970	10.110	10.064	10.139	10.017	10.044
AL109766.1	11.661	11.743	11.787	11.620	11.573	11.609
ASTN2	6.466	6.497	6.423	6.614	6.365	6.475
AL023775.1	8.693	8.759	8.829	8.743	8.940	8.716
MIR935	6.184	5.796	5.962	6.575	6.284	6.549
MIR939	10.331	10.274	10.468	10.286	9.990	10.270
MGA	8.128	8.029	8.266	7.771	8.184	7.947
MGP	5.966	5.906	6.120	6.310	6.187	6.162
RSPH3	8.697	8.693	8.823	8.389	8.528	8.551
SAMD4A	8.197	8.204	8.090	7.979	8.040	8.181
ADSL	10.779	10.928	11.052	10.878	10.712	10.839
SAMD4B	10.095	9.952	10.200	10.021	10.156	10.200
GAS2L1	8.823	8.819	9.014	8.630	8.562	8.681
ACVR1C	6.822	7.096	7.032	7.011	7.097	7.109
GAS2L3	10.503	10.324	10.417	10.374	10.456	10.311
GAS2L2	7.544	7.672	7.572	7.572	7.606	7.798
FAM53C	9.779	9.832	9.940	9.761	9.841	9.872
AC011374.1	4.914	4.759	4.607	4.458	4.862	4.608
ZW10	9.376	9.428	9.472	9.258	9.256	9.292
RSRC1	9.121	8.787	9.053	8.863	9.077	8.612
RSRC2	10.454	10.437	10.513	10.607	10.490	10.765
CPN2	7.060	6.676	6.662	6.953	6.932	6.964
CPN1	7.025	7.245	7.180	7.262	7.198	7.197
TP73-AS1	8.171	8.205	8.103	7.828	7.944	7.854
PYY2	8.197	8.005	8.214	8.682	8.557	8.351
AC090643.1	5.864	5.841	5.897	5.893	5.857	5.934
CDADC1	6.615	6.494	6.481	6.509	6.374	6.686
PHACTR1	7.654	7.445	7.251	7.515	7.527	7.588
OR2K2	5.205	5.292	5.272	5.329	5.495	5.399
ARHGAP44	8.163	8.058	8.148	8.036	8.188	8.135
ZNF733P	6.759	6.711	6.644	6.939	6.943	6.850
CASQ1	5.559	5.457	5.593	5.716	5.402	5.478
ARHGAP42	7.003	7.017	7.086	6.789	6.665	6.653
PHACTR2	9.065	9.126	8.991	8.940	9.078	8.998
PCBP1-AS1	7.028	7.116	6.991	6.938	7.049	6.981
AGAP12P	9.081	9.018	9.114	9.210	9.131	9.065
TNNT1	9.127	9.188	9.494	8.980	9.117	9.046

CDC42EP3	8.471	8.626	8.651	8.584	8.636	8.546
CDC42EP2	9.665	9.675	9.820	9.829	9.909	9.760
CDC42EP1	10.767	10.855	11.072	10.950	10.901	10.831
CDC42EP5	7.558	7.672	7.920	7.202	7.432	7.404
CDC42EP4	10.009	10.000	10.083	9.724	9.964	9.940
HEIH	10.311	10.328	10.396	10.254	10.220	10.336
GPRC6A	7.451	7.461	7.482	7.486	7.435	7.492
TBKBP1	8.019	7.961	8.283	8.522	8.253	8.177
ABI2	9.570	9.543	9.609	9.595	9.661	9.636
ABI3	8.190	7.983	7.891	8.071	8.103	8.148
OTC	6.482	6.411	6.425	6.596	6.719	6.766
TESC	12.570	12.534	12.846	12.379	12.229	12.358
OTP	6.552	6.747	6.802	7.097	6.856	6.872
AC008969.1	6.104	6.249	6.281	6.203	6.088	6.203
PITPNC1	9.034	9.032	9.155	8.873	9.065	8.920
CACNA1G-AS1	7.085	7.286	7.353	7.502	7.496	7.524
ZBED5	9.889	9.793	9.879	9.746	9.620	9.939
KRT31	6.615	6.930	6.585	6.692	6.674	6.779
KRT32	7.524	7.535	7.346	7.431	7.434	7.388
KRT35	5.985	5.738	5.899	6.087	6.072	6.319
KRT34	5.941	5.958	5.887	5.879	5.992	6.096
KRT37	7.145	7.207	7.017	7.274	7.221	6.924
KRT36	6.025	5.725	5.771	5.949	6.009	6.065
IL21	7.067	7.042	6.709	7.106	7.090	7.141
IL20	6.365	6.601	6.335	6.871	6.776	6.919
IL22	7.474	7.570	7.462	7.243	7.460	7.555
IL25	7.251	7.478	7.272	7.277	7.312	7.440
IL24	6.360	6.418	6.352	6.330	6.374	6.260
IL27	8.026	8.137	8.136	8.170	8.099	8.127
IL26	3.860	3.954	4.025	4.117	3.895	4.118
TIMP1	12.097	12.211	12.336	12.148	12.110	12.179
CLINT1	11.885	11.893	11.995	11.933	11.881	11.806
C4B	8.508	8.681	8.617	8.611	8.632	8.722
AHRR	6.262	6.310	6.229	6.235	6.359	6.461
ADCY4	9.570	9.598	9.405	9.657	9.631	9.540
ADCY5	8.016	8.014	7.886	7.925	7.935	8.067
ADCY6	9.535	9.526	9.536	9.379	9.372	9.442
ADCY7	9.036	9.092	9.131	8.925	9.006	9.140
ADCY1	7.834	7.970	7.738	8.004	8.083	8.010
ADCY2	7.341	7.579	7.515	7.469	7.754	7.646
ADCY3	8.327	8.506	8.413	8.410	8.206	8.211
ADCY8	6.080	6.318	6.509	6.846	6.410	6.464
ADCY9	7.718	7.598	7.759	7.617	7.722	7.635
CARNMT1-AS1	6.067	6.225	6.132	6.388	6.193	6.370
AC068134.3	7.890	7.894	7.762	7.726	7.828	7.682
NUCKS1	12.146	12.125	12.270	12.042	12.278	11.858
DDI2	8.496	8.414	8.445	8.117	8.102	8.187
DDI1	5.048	5.316	5.231	5.172	5.139	5.151
XRCC1	9.408	9.434	9.590	9.276	9.283	9.180
HOXB-AS3	7.074	7.067	7.098	7.021	7.148	6.938
XRCC3	8.110	8.125	8.373	8.088	8.182	8.067
HOXB-AS1	7.048	7.149	7.160	7.097	7.260	7.240
XRCC5	12.140	12.208	12.337	12.098	11.935	12.039
XRCC4	9.491	9.617	9.688	9.537	9.497	9.399
XRCC6	12.523	12.581	12.785	12.590	12.405	12.449
MEIS3	8.644	8.839	8.783	8.825	8.644	8.687

MEIS2	10.129	10.257	10.204	9.978	10.078	9.741
MEIS1	7.084	7.147	7.146	6.913	7.059	7.400
PCDHAC2	10.289	10.323	10.367	10.104	10.324	10.100
PCDHAC1	10.289	10.323	10.367	10.104	10.324	10.100
CTLA4	7.635	7.666	7.470	7.780	7.743	7.799
ESPNL	8.567	8.504	8.568	8.442	8.748	8.649
USP32P1	8.663	8.479	8.503	8.310	8.228	8.678
USP32P2	8.816	8.726	8.703	8.342	8.439	8.740
AC104041.1	3.796	3.986	3.800	3.952	3.855	3.921
DDX60	9.597	9.714	9.589	9.264	9.343	9.245
AC018523.2	11.955	11.935	11.995	12.035	12.000	12.010
GAU1	8.353	8.236	8.156	8.428	8.401	8.336
AL137127.1	10.399	10.397	10.335	10.152	10.050	10.221
ADCY10	5.522	5.775	5.518	5.725	5.767	5.594
KCNA6	7.347	7.434	7.299	7.499	7.478	7.536
KCNA7	6.351	6.384	6.515	6.738	6.458	6.565
KCNA4	5.804	5.821	5.841	5.831	5.911	5.831
KCNA5	7.157	6.827	6.288	6.973	7.027	7.250
OR10D1P	7.401	7.725	7.442	7.592	7.529	7.764
KCNA3	6.205	6.002	6.048	6.103	6.219	6.295
KCNA1	6.268	6.295	6.417	6.339	6.410	6.288
OPTN	9.467	9.489	9.565	9.369	9.355	9.429
BHMT	7.233	7.252	7.088	7.209	7.254	7.289
OPTC	7.539	7.634	7.771	7.965	7.806	7.628
FTL	14.413	14.458	14.478	14.437	14.424	14.436
AC093283.1	6.689	6.839	6.635	6.872	6.717	6.866
FTO	9.747	9.737	9.804	9.481	9.486	9.527
ZDHHC1	7.495	7.482	7.580	7.420	7.137	7.501
ACACA	11.754	11.781	11.789	11.816	11.544	11.731
ACACB	10.096	9.891	9.875	10.216	10.287	10.340
ZDHHC3	9.331	9.429	9.525	9.382	9.383	9.391
PLA2G2A	8.021	8.174	7.928	8.108	8.122	8.070
PLA2G2E	7.555	7.452	7.376	7.784	7.702	7.849
PLA2G2D	8.691	8.586	8.564	8.612	8.609	8.802
PLA2G2F	5.970	6.059	5.941	5.870	6.095	5.993
AC127391.1	9.106	9.330	9.270	9.257	9.359	9.374
AC018462.1	5.049	4.818	4.907	4.851	4.876	4.944
CYFIP1	10.259	10.281	10.278	10.236	10.158	10.131
CYFIP2	9.383	9.458	9.431	9.148	9.158	9.324
GUSBP5	4.826	5.227	4.897	4.843	4.857	5.150
GUSBP1	6.276	6.340	6.209	6.266	6.370	6.288
GUSBP3	5.859	5.735	5.446	5.456	5.312	5.507
GUSBP2	6.208	6.243	5.920	6.406	6.416	6.188
AC107622.1	3.804	3.858	3.917	3.897	3.777	4.025
AC051649.3	8.109	8.099	8.208	8.273	8.408	8.309
LHPP	8.821	8.846	8.967	8.819	8.842	8.766
NUP188	8.659	8.715	8.755	8.686	8.568	8.786
SETMAR	8.291	8.446	8.259	8.436	8.515	8.280
GLT8D2	7.880	7.912	7.721	7.974	7.887	7.996
GLT8D1	9.942	9.946	10.007	9.636	9.728	9.767
AL354836.1	7.952	7.926	7.997	7.706	7.741	8.081
AL844892.1	5.620	5.568	5.605	5.779	5.854	5.729
OR2C3	4.490	4.387	4.404	4.455	4.493	4.418
OR2C1	7.172	7.389	7.196	7.293	7.396	7.420
CU633904.2	7.923	7.782	7.902	7.606	7.461	7.712
REG1A	6.930	6.989	6.942	6.899	7.057	7.048

REG1B	6.384	6.646	6.532	6.504	6.550	6.543
MRGPRE	6.756	6.557	6.724	6.896	6.860	7.078
OSR2	6.446	6.431	6.744	6.381	6.329	6.448
TTC39B	6.825	6.625	6.594	6.369	6.478	6.747
TTC39A	9.222	9.367	9.376	9.202	9.052	9.240
BPNT1	8.429	8.434	8.479	8.276	8.482	8.308
ABHD14A-ACY1	10.008	10.064	10.115	9.872	9.784	9.806
RNU7-1	11.391	11.444	11.677	11.240	11.213	11.453
BCL9	7.744	7.781	7.894	7.635	7.802	7.863
AC009060.1	4.773	5.006	4.923	4.941	5.193	5.050
BCL6	9.324	9.373	9.460	9.409	9.251	9.308
BCL3	8.479	8.351	8.586	8.966	8.785	9.007
BCL2	7.833	7.969	7.684	7.892	7.964	7.890
AC008267.1	5.676	5.724	5.684	5.684	5.764	5.598
AC008267.5	7.216	7.357	7.427	7.378	7.334	7.447
NPEPL1	8.644	8.723	8.917	8.981	8.801	8.830
AC008267.8	5.674	5.585	5.624	5.593	5.751	5.647
CLMN	10.036	10.015	10.158	9.821	9.797	9.878
CLMP	6.480	6.147	6.296	6.662	6.489	6.495
AC004556.4	8.109	8.099	8.208	8.273	8.408	8.309
AC004556.1	8.109	8.099	8.208	8.273	8.408	8.309
AC004556.3	10.767	10.843	11.072	10.720	10.660	10.651
ACTBP9	10.907	11.040	11.009	10.707	10.734	10.803
AC015982.2	6.740	6.642	6.884	7.128	6.533	6.949
MTM1	7.188	7.136	7.253	6.940	7.094	7.101
SERPINB9	9.220	9.035	9.193	8.832	9.215	8.923
CSNK1G3	9.837	9.734	9.713	9.717	9.592	9.744
CSNK1G1	9.595	9.269	9.339	8.959	9.061	9.190
ALOX12-AS1	7.513	7.606	7.246	7.550	7.518	7.731
SERPINB1	10.307	10.539	10.583	10.243	10.323	10.395
SERPINB3	7.512	7.591	7.620	7.692	7.773	7.683
SERPINB2	7.230	7.369	7.273	7.332	7.543	7.541
SERPINB5	3.898	3.985	4.179	4.112	4.131	4.276
SERPINB4	7.479	7.470	7.597	7.858	7.477	7.604
SERPINB7	7.667	7.761	7.917	7.635	7.827	7.951
SERPINB6	10.076	10.182	10.394	10.041	9.851	9.934
FAM76B	7.902	7.865	7.896	7.485	7.522	7.528
FAM76A	6.946	7.132	7.069	6.927	7.024	7.019
DDX59-AS1	5.051	5.151	5.248	5.499	5.365	5.292
EXD3	7.605	7.633	7.739	8.130	7.739	7.672
EXD2	9.352	9.432	9.429	9.373	9.411	9.512
EXD1	5.052	4.939	4.900	4.816	4.952	4.785
AC006116.1	6.860	6.531	6.826	7.113	7.178	7.029
ELP6	8.731	8.806	8.941	8.661	8.750	8.861
ELP5	9.008	9.071	9.150	8.966	9.034	8.998
ELP4	9.082	9.100	9.287	9.209	8.977	9.085
ELP3	9.686	9.663	9.706	9.612	9.540	9.599
ELP2	8.616	8.664	8.646	8.419	8.356	8.588
ELP1	9.032	8.922	8.938	8.747	8.783	8.863
AC006116.9	6.016	6.102	5.989	6.461	6.207	6.281
SPRYD3	8.524	8.420	8.614	8.419	8.457	8.478
SPRYD4	8.962	9.019	9.124	9.038	8.998	9.008
AC098859.1	10.409	10.430	10.647	10.285	10.293	10.153
KLHDC9	8.056	8.157	8.176	8.127	8.189	8.246
RAC2	10.293	10.385	10.633	10.333	10.434	10.313
KLHDC4	8.799	8.792	8.680	8.663	8.826	8.673

KLHDC2	10.279	10.436	10.533	10.090	10.138	10.214
KLHDC3	10.283	10.334	10.432	10.314	10.431	10.291
KLHDC1	4.376	4.282	4.338	4.531	4.567	4.352
ASXL1	10.082	10.170	10.301	9.927	10.010	10.104
TRIM51	7.241	6.882	7.097	7.513	7.235	7.041
ASXL3	6.860	6.857	6.855	7.130	6.992	7.022
ASXL2	10.888	11.058	11.029	11.022	10.857	10.950
HDDC2	9.595	9.622	9.775	9.663	9.793	9.617
HDDC3	9.494	9.736	9.910	9.556	9.585	9.579
ZNF252P-AS1	5.671	5.670	5.587	5.671	5.745	5.633
DONSON	8.646	8.653	8.712	8.320	8.459	8.396
SPATA13	9.023	8.986	9.109	8.826	8.746	8.905
AC025272.1	8.957	8.926	8.848	8.986	9.059	8.961
SPATA17	6.108	6.254	6.254	6.133	6.290	6.080
SPATA16	6.813	7.078	7.006	7.104	7.155	7.184
VCL	12.287	12.485	12.553	12.262	12.316	11.937
COL18A1-AS1	5.902	6.297	6.126	6.059	6.166	6.166
SPATA18	7.612	7.545	7.343	7.727	7.814	7.617
AC011473.4	7.568	7.468	7.631	7.919	7.733	7.697
AC010343.1	13.214	13.233	13.392	13.147	13.175	13.114
AC011752.1	6.037	6.197	6.033	6.228	6.311	6.222
CEP170P1	7.445	7.429	7.486	7.410	7.557	7.412
ZNF214	6.698	6.612	6.650	6.979	7.011	6.702
VCX	7.570	7.633	7.681	7.804	7.550	7.687
VCY	8.470	8.155	8.014	8.567	8.399	8.342
HFE	8.877	9.168	9.117	8.874	9.028	8.986
PBX4	7.031	7.139	7.191	7.129	7.037	7.029
GPR146	5.860	5.856	6.021	5.875	5.849	5.868
ZNF213	8.474	8.545	8.675	8.572	8.499	8.370
GPR143	6.953	6.901	6.886	6.984	6.974	6.785
ZNF212	8.716	8.756	8.762	8.771	8.517	8.597
GPR149	4.567	4.685	4.484	5.010	4.689	4.709
SLC7A6	9.071	8.955	9.099	8.899	8.651	9.017
KNL1	7.324	7.352	7.254	7.252	7.286	7.395
HLA-B	13.705	13.724	13.757	13.585	13.558	13.528
CITED2	8.647	8.653	8.904	8.581	8.655	8.780
CITED1	7.880	7.797	7.914	7.909	7.971	7.923
SLC7A4	7.500	7.660	7.689	7.682	7.806	7.522
CITED4	9.515	9.551	9.790	9.487	9.454	9.390
DAGLA	8.680	8.706	8.715	8.533	8.563	8.577
DAGLB	8.808	8.915	8.990	8.902	8.814	8.865
SLC7A2	8.951	8.835	8.811	8.679	8.592	8.710
AL162456.1	4.982	5.161	5.076	4.829	4.692	5.295
HLA-F	11.803	11.835	11.902	11.793	11.754	11.779
ADGRF5-AS1	6.485	6.530	6.100	6.351	6.508	6.539
HIRA	8.745	8.500	8.640	8.484	8.521	8.485
RIN1	8.793	8.992	9.119	9.154	9.017	8.951
RIN2	9.100	9.018	9.030	8.756	8.941	8.684
WWOX	8.968	8.979	8.995	8.876	8.978	8.841
AC021087.3	5.088	5.046	4.803	5.074	5.133	4.901
AC021087.4	6.770	6.853	6.884	7.243	6.961	7.150
AC021087.5	10.434	10.522	10.701	10.473	10.250	10.447
RIPK1	8.500	8.607	8.525	8.340	8.490	8.525
RIPK3	7.820	7.903	8.028	7.790	7.862	7.790
RIPK2	10.215	10.170	10.339	10.258	10.245	10.335
PDCL	8.219	8.211	8.330	8.138	8.290	8.073

IGFBP7-AS1	3.869	4.101	4.038	4.040	3.873	4.104
SRCIN1	7.283	7.239	7.199	7.518	7.212	7.292
TUBG1	11.397	11.448	11.592	11.117	11.033	11.072
C2orf27A	7.972	7.912	8.183	8.328	8.259	8.126
CRABP1	7.039	6.945	7.141	6.795	6.867	6.791
FAM149A	10.269	10.111	10.283	9.644	9.600	9.871
C2orf27B	7.972	7.912	8.183	8.328	8.259	8.126
C7orf77	5.719	5.947	5.706	5.890	5.927	6.093
ZNF419	9.284	9.409	9.414	9.177	9.235	9.210
ZNF418	6.965	6.712	6.790	7.092	7.072	6.993
ZNF417	3.817	3.797	3.908	3.775	3.706	3.781
ZNF416	7.709	7.686	7.665	7.638	7.774	7.556
ZNF415	5.112	5.135	4.928	5.102	5.193	5.167
ZNF414	7.748	7.772	8.096	7.418	7.845	7.629
ZNF410	10.324	10.307	10.396	10.393	10.354	10.526
GLIPR2	7.578	7.548	7.750	7.931	7.694	7.889
LOXL1-AS1	6.939	7.172	7.251	6.196	6.703	6.563
E2F6P3	9.209	9.151	9.184	9.181	9.090	9.233
E2F6P2	9.209	9.151	9.184	9.181	9.090	9.233
E2F6P1	9.209	9.151	9.184	9.181	9.090	9.233
POU4F1	7.555	7.659	7.632	7.334	7.633	7.580
POU4F2	5.456	5.521	5.514	5.548	5.408	5.448
POU4F3	5.794	5.864	5.884	5.779	5.971	5.704
LINC01547	8.164	8.032	8.145	8.296	8.227	8.192
LINC01545	6.861	6.677	6.841	6.955	6.991	6.915
FBF1	7.644	7.666	7.471	7.728	7.845	7.738
RASA4	7.614	7.679	7.735	7.914	7.765	7.740
LINC01549	4.427	4.513	4.453	4.450	4.717	4.658
RASA1	12.332	12.369	12.424	12.451	12.401	12.532
RASA2	9.598	9.560	9.525	9.467	9.672	9.497
RASA3	7.690	7.728	7.583	7.813	8.011	7.670
ACTN3	7.198	7.203	7.095	7.108	7.209	7.345
ACTN2	8.115	8.119	7.896	8.073	8.296	8.262
ACTN1	12.048	12.078	12.240	12.078	12.105	11.984
ACTN4	12.815	12.947	13.071	12.823	13.012	12.779
EZR	12.884	13.030	13.113	12.890	12.766	12.862
AL359198.1	7.610	7.362	7.387	7.756	7.514	7.504
N6AMT1	6.606	6.643	6.764	6.672	6.701	6.691
RAD9A	10.131	10.302	10.184	10.099	10.241	10.360
OR5J2	4.791	5.007	4.783	5.144	5.126	4.957
ERGIC3	12.085	12.298	12.499	12.413	12.409	12.346
ABLIM1	11.178	11.259	11.293	10.984	10.654	10.961
ABLIM3	9.477	9.511	9.579	9.329	9.356	9.427
ABLIM2	7.664	7.581	7.508	7.807	7.563	7.606
FAM172A	9.044	9.192	9.202	8.849	9.170	9.078
LINC00630	4.400	4.356	4.353	4.300	4.572	4.255
LINC00636	5.205	5.059	5.211	5.164	5.305	5.197
LINC00638	8.716	8.830	8.790	8.671	8.921	8.898
LINC00639	4.632	4.772	4.583	4.641	4.774	4.768
RNU4-82P	6.321	6.015	6.276	6.324	6.505	6.279
BIVM-ERCC5	7.712	7.756	7.841	7.683	7.351	7.765
RAB6D	11.383	11.523	11.428	11.371	11.347	11.483
GSTZ1	8.894	9.046	9.004	8.686	8.724	8.747
RAB6A	11.383	11.523	11.428	11.371	11.347	11.483
RAB6B	8.587	8.587	8.578	8.846	8.819	8.801
RAB6C	11.383	11.523	11.428	11.371	11.347	11.483

AC034228.3	5.286	5.356	5.123	5.393	5.460	5.294
DLGAP4-AS1	7.383	7.312	7.344	7.533	7.369	7.463
IRF1-AS1	7.725	7.664	7.392	7.597	7.660	7.697
DAOA	6.356	6.744	6.624	6.305	6.533	6.657
ERVV-1	5.619	5.714	5.722	5.766	5.832	5.683
AC027458.1	7.887	7.795	7.885	7.884	7.898	7.959
KRTAP5-AS1	6.644	6.772	6.554	6.570	6.812	6.783
GDI2	12.046	12.154	12.215	12.203	12.001	12.086
GDI1	11.461	11.525	11.519	11.413	11.285	11.439
TTC30A	7.519	7.838	7.957	7.481	7.333	7.547
MMP25-AS1	8.791	8.717	8.568	8.698	8.572	8.842
TTC30B	6.859	7.024	7.200	6.711	6.827	6.822
TFAP4	8.787	8.776	8.770	8.940	8.966	8.865
SPDL1	8.362	8.468	8.468	8.135	8.231	8.335
ST13P18	12.367	12.411	12.560	12.394	12.296	12.369
ST13P13	12.367	12.411	12.560	12.394	12.296	12.369
NAPEPLD	7.791	7.828	7.907	7.844	8.161	7.495
GSDME	9.246	9.326	9.351	8.940	9.096	9.065
SPTA1	5.546	5.759	5.647	5.448	5.710	5.509
HGSNAT	9.642	9.641	9.628	9.472	9.561	9.443
AC104695.4	4.549	4.612	4.535	4.764	4.717	4.779
AC104695.2	4.950	4.797	4.849	4.850	4.894	4.916
ANKRD20A17P	3.767	3.749	3.901	3.720	3.792	3.690
CRYZL1	8.253	8.333	8.375	8.043	8.320	8.285
AC242376.2	5.411	5.319	5.530	5.572	5.595	5.507
SERF2	12.250	12.325	12.464	12.351	12.356	12.378
NAV2-AS2	6.996	6.976	7.168	7.032	7.033	7.113
NAV2-AS4	8.254	8.267	8.033	8.142	8.379	8.303
NAV2-AS5	6.107	6.237	6.116	6.356	6.479	6.453
PGM5P4-AS1	7.383	7.470	7.517	7.591	7.447	7.467
NAT9	9.299	9.229	9.372	9.146	9.254	9.100
NAT8	6.898	6.791	6.847	6.773	6.742	6.452
NAT2	7.404	7.521	7.458	7.765	7.469	7.483
NAT1	8.466	8.632	8.643	8.539	8.522	8.510
ADGRL4	3.685	3.759	3.800	3.736	3.874	3.656
ADGRL2	9.739	9.906	9.874	9.795	9.733	9.829
ADGRL3	5.811	5.476	5.782	6.069	5.731	5.877
ADGRL1	8.762	8.658	8.729	8.524	8.593	8.845
AL592490.1	4.046	4.248	4.137	4.187	4.020	3.974
FAIM2	7.367	7.490	7.559	7.537	7.439	7.508
AL031315.1	6.995	7.032	7.208	7.360	7.072	7.267
AC008280.3	4.753	4.962	5.063	5.119	5.069	5.017
CR388205.3	9.231	9.374	9.436	9.462	9.438	9.339
PARG	8.531	8.495	8.391	8.456	8.288	8.609
AC105760.1	4.207	4.263	4.145	4.274	4.197	4.246
AP000311.1	11.448	11.482	11.710	11.497	11.351	11.428
COX5A	12.767	12.808	12.922	12.755	12.726	12.722
COX5B	11.579	11.721	11.854	11.723	11.765	11.693
TMEM258	12.483	12.610	12.714	12.518	12.575	12.534
TMEM256	9.364	9.321	9.605	9.350	9.574	9.480
TMEM254	8.449	8.629	8.546	8.490	8.531	8.454
TMEM252	6.474	6.515	6.613	6.705	6.664	6.604
TMEM251	9.649	9.762	9.902	9.608	9.647	9.659
TMEM250	8.815	8.941	9.062	8.794	8.807	8.862
AC023590.1	3.916	4.117	3.890	3.901	4.088	4.038
AL391560.2	5.776	5.777	5.502	5.772	5.859	5.842

SIPA1L1	8.575	8.516	8.582	8.482	8.709	8.485
SIPA1L3	9.541	9.490	9.613	9.571	9.566	9.579
SIPA1L2	7.641	7.745	7.839	7.865	7.827	7.834
PPFIA4	6.000	5.979	6.077	6.093	6.134	6.114
AC079776.2	8.584	8.611	8.486	8.387	8.523	8.668
SLC35F5	10.250	10.232	10.242	10.121	10.034	10.222
SLC35F4	5.269	5.126	5.322	5.169	5.152	5.138
SLC35F6	10.028	9.964	10.107	9.982	9.996	9.755
SLC35F1	7.742	7.786	7.819	7.705	7.849	7.815
SLC35F3	5.257	5.086	5.156	5.041	5.078	5.324
SLC35F2	9.379	9.393	9.242	9.226	9.241	9.259
AC091959.3	9.169	9.184	9.199	9.151	9.523	9.072
SUPV3L1	8.568	8.696	8.638	8.608	8.650	8.550
LMAN1L	7.935	7.903	7.876	7.966	7.894	8.052
TRMT112	11.806	11.794	11.970	11.815	11.799	11.811
MPC2	11.679	11.735	11.894	11.601	11.609	11.606
MPC1	10.113	10.174	10.374	10.234	10.288	10.054
TSPAN10	8.171	8.191	8.303	8.322	8.308	8.221
TSPAN11	8.168	8.311	8.219	7.990	8.330	8.413
TSPAN12	8.997	9.123	9.044	8.680	8.676	8.795
TSPAN13	12.631	12.684	12.727	12.735	12.683	12.673
TSPAN14	9.406	9.359	9.510	9.454	9.516	9.271
TSPAN15	10.169	10.249	10.341	10.186	10.030	10.062
TSPAN16	5.817	5.908	5.911	6.190	5.918	6.045
TSPAN17	10.200	10.353	10.408	10.315	10.320	10.245
AC092687.4	7.757	7.776	7.786	7.441	7.702	7.849
MT-ND1	13.966	14.006	13.987	14.087	14.178	14.074
MT-ND2	13.966	14.006	13.987	14.087	14.178	14.074
MT-ND3	14.545	14.551	14.548	14.608	14.629	14.551
MT-ND4	14.647	14.647	14.633	14.660	14.699	14.664
MT-ND5	13.861	13.915	13.820	13.987	13.988	13.823
MT-ND6	9.881	9.831	10.136	10.496	11.142	10.154
EAF1	8.010	8.174	8.232	8.098	8.232	8.125
AL096803.1	6.151	6.209	6.112	6.191	6.183	6.132
FOXF1	6.469	6.201	6.202	6.400	6.392	6.459
FOXF2	5.565	5.289	5.116	5.897	5.778	5.784
RBPJL	7.486	7.703	7.491	7.632	7.673	7.585
AC027796.1	6.849	7.001	6.975	6.997	6.860	6.994
TMUB2	9.314	9.312	9.453	9.220	9.149	9.347
SCGB1C1	5.564	5.715	5.701	5.949	5.905	5.855
SCGB1C2	5.564	5.715	5.701	5.949	5.905	5.855
SYNCRIP	11.713	11.782	11.815	11.659	11.938	11.209
MMAA	6.985	7.020	7.204	7.078	6.986	7.120
MMAB	10.137	10.281	10.388	10.190	10.028	10.189
TYR	7.414	7.507	7.163	7.663	7.703	7.699
ALG8	10.591	10.525	10.568	10.507	10.489	10.589
PDXK	11.067	11.162	11.338	10.890	10.829	10.849
ALG2	10.051	10.162	10.256	10.048	10.073	10.169
ALG3	11.157	11.230	11.360	11.277	11.333	11.180
ALG1	8.642	8.787	8.948	8.382	8.713	8.503
ALG6	9.604	9.723	9.630	9.585	9.595	9.731
ALG5	10.728	10.865	11.000	10.761	10.691	10.795
WIF1	5.118	5.031	5.209	5.382	5.131	5.233
PDXP	9.407	9.516	9.600	9.091	9.282	9.250
RNGTT	8.304	8.249	8.090	8.143	8.256	8.215
KIAA1324L	6.882	7.041	7.024	7.077	6.961	6.828

AC110771.1	5.622	5.516	5.495	5.607	5.536	5.657
INSL5	6.115	5.660	5.792	6.180	6.117	6.175
INSL4	6.738	6.611	6.760	6.656	6.750	6.793
HAR1A	4.756	4.719	4.578	4.736	4.750	4.750
INSL6	5.795	6.177	6.100	6.066	6.263	6.006
MAGEL2	5.867	6.017	5.864	6.052	5.962	5.999
INSL3	8.585	8.742	8.823	8.636	8.683	8.741
ZBTB44-DT	5.602	5.603	5.524	5.668	5.908	5.725
HGS	10.862	10.911	11.074	10.784	10.801	10.724
RDM1	6.877	7.025	7.076	6.843	7.028	6.814
AC022101.1	7.378	7.469	7.336	7.440	7.556	7.326
CLUL1	6.115	6.308	6.076	6.447	6.051	6.238
PDX1	6.911	7.063	6.981	7.401	7.078	7.038
TNFRSF14-AS1	8.624	8.766	8.777	8.746	8.719	8.793
AC244216.3	7.406	7.569	7.636	7.983	7.893	7.730
INMT	5.756	5.741	5.812	5.814	5.858	5.747
FECH	8.534	8.684	8.663	8.420	8.353	8.454
AC103760.1	6.874	6.678	6.850	6.431	6.376	6.493
FCSK	9.191	9.210	9.382	8.986	9.135	9.064
ADAP1	12.194	12.308	12.487	12.130	12.165	12.133
ADAP2	7.707	7.546	7.556	7.680	7.797	7.767
KLHL10	5.998	5.767	6.045	6.011	6.001	6.166
KLHL11	10.394	10.351	10.343	10.328	10.204	10.336
KLHL12	10.034	10.027	10.085	9.811	9.782	9.868
KLHL13	4.236	4.349	4.106	4.435	4.262	4.249
KLHL14	8.224	8.320	8.422	8.434	8.473	8.327
KLHL15	7.592	7.558	7.738	7.250	7.383	7.320
KLHL17	7.834	7.978	7.890	7.727	7.652	7.712
KLHL18	8.329	8.235	8.111	8.266	8.326	8.327
AC099850.2	5.811	5.840	5.790	5.623	5.647	5.943
RNU6-73P	6.763	6.811	6.676	6.758	6.721	6.724
AC099489.3	6.617	6.632	6.418	6.703	6.751	6.754
AC099489.1	7.839	8.083	8.004	8.199	8.167	8.018
Z84466.1	5.718	5.789	5.998	6.051	6.019	5.960
AC005921.1	4.269	4.508	4.243	4.376	4.373	4.389
DMRTB1	6.999	7.001	6.879	7.159	7.219	7.122
C7orf61	7.508	7.599	7.598	7.714	7.570	7.802
GABPB2	8.090	7.946	7.937	7.810	7.785	7.964
IGIP	7.381	7.023	7.169	7.319	7.215	7.334
ZNF529-AS1	5.520	5.547	5.594	5.675	5.497	5.741
ZNF200	8.487	8.497	8.529	8.311	8.425	8.424
THAP12P5	4.964	4.990	5.244	5.026	5.005	4.954
SLC16A12-AS1	5.567	5.740	5.479	5.714	5.829	5.699
RTEL1-TNFRSF6	8.539	8.492	8.553	8.544	8.586	8.557
ZNF462	9.017	8.869	8.947	8.676	8.823	8.689
SLN	6.972	7.285	6.984	7.016	7.249	7.153
SLK	9.686	9.745	9.669	9.093	9.335	9.293
BTG3-AS1	5.172	5.399	5.651	5.402	5.473	5.406
SLA	7.871	7.843	7.659	7.934	7.855	7.969
PHYH	9.148	9.170	9.355	9.065	9.036	9.253
ZNF461	4.982	5.004	4.930	4.788	4.887	4.874
NOTCH4	7.602	7.441	7.549	7.639	7.548	7.602
MEAF6	10.475	10.536	10.657	10.454	10.379	10.426
AZIN1	9.981	10.103	10.177	10.007	9.936	9.985
NOTCH1	7.221	7.313	7.413	7.438	7.321	7.337
NOTCH2	10.676	10.579	10.553	10.333	10.394	10.462

AZIN2	7.729	7.907	8.049	8.022	7.890	7.827
ZNF658	4.490	4.455	4.496	4.536	4.486	4.462
ZNF655	7.571	7.552	7.208	7.600	7.455	7.604
TCTEX1D2	9.028	9.183	9.287	9.182	9.213	9.194
AC247039.1	4.232	4.165	4.506	4.480	4.299	4.279
ZNF653	6.703	6.730	6.972	6.545	6.610	6.832
ZNF652	9.342	9.242	9.268	9.207	9.045	9.174
N4BP2L1	7.753	7.721	7.742	7.846	7.885	7.763
RAPGEF5	10.269	10.254	10.209	9.995	9.952	9.889
RAPGEF6	9.260	9.091	8.995	9.133	8.986	9.215
N4BP2L2	7.988	7.741	7.954	7.656	7.578	7.658
RAPGEF1	9.313	9.294	9.440	9.168	9.219	9.200
RAPGEF2	8.554	8.468	8.448	8.262	8.406	8.383
RAPGEF3	8.228	8.154	8.201	8.396	8.049	8.321
ZNF384	10.488	10.315	10.420	10.489	10.388	10.422
ZNF383	6.197	6.249	6.191	6.159	6.257	6.325
POC1A	8.788	8.804	8.884	8.569	8.552	8.616
POC1B	9.242	9.193	9.080	9.202	9.092	9.256
AC020929.1	8.615	8.439	8.623	8.777	8.560	8.589
AC005592.1	5.943	5.660	5.713	5.925	6.077	6.011
KRTAP3-2	6.923	7.046	7.179	7.024	7.213	7.286
KRTAP3-3	4.472	4.583	4.298	4.515	4.495	4.593
KRTAP3-1	7.758	8.118	7.812	8.143	8.204	8.126
AC011773.1	7.577	7.459	7.400	7.608	7.478	7.512
CLEC10A	6.803	6.622	6.836	6.926	6.847	6.671
SNRPD3	12.008	12.085	12.242	11.941	11.781	11.833
SNRPD2	12.375	12.424	12.644	12.641	12.523	12.574
SNRPD1	10.702	10.698	10.866	10.555	10.518	10.539
NMBR	5.069	5.335	4.887	4.802	5.292	5.206
RBMY1HP	6.498	6.612	6.640	6.865	6.762	6.723
PKP3	10.874	11.044	11.105	10.943	10.835	10.830
RBM26-AS1	7.725	7.787	7.825	7.534	7.848	7.688
PKP2	9.302	9.365	9.275	9.122	9.183	9.124
IFT43	9.437	9.438	9.590	9.333	9.348	9.412
IFT46	8.903	8.899	9.206	9.036	9.136	9.067
RFX6	4.256	4.150	4.284	4.173	4.407	4.379
NOP14-AS1	6.791	7.045	6.943	7.209	7.267	7.076
GU182355.3	8.835	8.689	8.488	8.744	8.793	8.879
CRIM1-DT	8.357	8.459	8.594	8.277	8.328	8.069
GLDN	7.671	7.652	7.767	7.497	7.628	7.493
GLDC	8.335	8.200	8.236	8.123	7.991	8.152
AC134026.2	4.567	4.685	4.484	5.010	4.689	4.709
PC	8.883	8.906	8.899	8.798	8.784	8.835
AC069528.1	4.790	4.728	4.670	4.773	4.763	4.796
LINC01834	8.365	8.524	8.438	8.520	8.722	8.644
LINC01833	7.897	8.006	7.951	8.030	8.213	8.056
LINC01831	5.893	5.801	5.601	6.200	5.961	5.953
DHRS11	9.739	9.737	9.823	9.278	9.483	9.491
DHRS12	7.904	7.925	7.946	7.811	7.887	7.800
DHRS13	9.629	9.711	9.911	9.665	9.582	9.612
LINC01838	8.741	8.435	8.137	8.543	8.611	8.519
GPR89B	10.031	10.035	10.006	9.992	10.000	9.944
AC004801.3	5.132	4.697	4.839	4.908	5.136	4.969
GPR89A	10.031	10.035	10.006	9.992	10.000	9.944
MAP1LC3B2	11.378	11.467	11.558	11.459	11.477	11.410
LGMN	10.282	10.306	10.378	9.961	10.022	10.049

NBDY	11.984	11.949	12.133	11.832	11.690	11.874
C12orf76	8.106	8.223	8.374	8.072	8.220	8.184
AC020659.1	8.535	8.569	8.589	8.507	8.774	8.538
CAPS2	4.805	4.832	5.055	4.856	4.930	4.930
EHMT1	8.329	8.154	8.207	7.916	8.013	8.098
EHMT2	8.721	8.708	8.853	8.681	8.872	8.717
ADIPOR2	11.097	10.987	11.108	10.879	10.791	10.880
AL137244.1	7.226	7.179	7.462	7.408	7.451	7.374
ADIPOR1	11.307	11.300	11.470	11.199	11.228	11.222
NBPF26	11.550	11.483	11.377	11.343	11.231	11.508
AC068234.1	6.556	6.632	6.543	6.616	6.790	6.495
NBPF20	11.550	11.483	11.377	11.343	11.231	11.508
AC068234.2	5.601	5.891	5.955	5.995	5.898	6.024
MARK4	10.093	10.137	10.282	9.953	10.054	10.003
STOX1	5.965	5.967	6.038	5.784	5.977	6.070
MARK1	8.266	8.147	8.383	8.314	8.252	7.958
MARK2	8.233	7.511	7.685	7.985	7.747	8.293
MARK3	9.161	9.161	9.420	9.108	8.958	9.147
IGBP1	10.883	10.962	11.112	10.817	10.848	10.761
AC079305.2	6.504	6.583	6.600	6.416	6.400	6.572
TPD52L2	11.879	11.995	12.142	11.905	11.769	11.893
TPD52L3	8.108	8.068	7.570	8.248	8.127	8.104
TPD52L1	11.380	11.397	11.631	11.240	11.168	11.165
EFCAB11	9.208	9.224	9.399	9.126	8.981	8.910
EFCAB13	6.949	6.938	6.879	7.097	6.955	7.107
EFCAB12	5.371	5.431	5.104	5.195	5.249	5.261
EFCAB14	11.601	11.587	11.651	11.429	11.406	11.393
OR7E47P	8.595	8.578	8.492	8.458	8.660	8.477
CAPSL	6.053	5.973	6.145	6.373	6.110	6.164
ACTR3C	7.477	7.538	7.580	7.581	7.467	7.410
ACTR3B	7.683	7.787	7.919	7.476	7.672	7.883
WDR13	10.280	10.383	10.361	10.084	9.932	10.053
WDR12	10.561	10.627	10.758	10.404	10.475	10.428
WDR11	8.573	8.463	8.526	8.339	8.360	8.526
WDR17	4.894	4.996	5.255	4.736	5.087	4.905
WDR19	7.662	7.661	7.540	7.455	7.366	7.482
WDR18	9.320	9.333	9.539	8.905	9.083	9.077
STK24-AS1	4.683	4.483	4.551	4.609	4.384	4.595
LINC00879	6.733	7.212	6.869	6.939	7.078	7.054
ATP11A-AS1	4.991	4.928	5.014	5.207	5.239	5.129
LINC00877	5.855	5.912	5.783	5.870	5.992	6.065
LINC00870	6.278	6.361	6.186	6.537	6.534	6.599
VN1R105P	4.623	4.658	4.664	4.620	4.660	4.680
USP9X	11.381	11.463	11.440	11.280	11.186	11.297
USP9Y	5.643	5.572	5.379	5.529	5.724	5.878
ACAD10	8.289	8.302	8.425	8.165	8.107	8.241
NLRP2B	8.443	8.275	8.398	8.219	8.165	8.231
AP000781.2	10.723	10.797	10.846	10.610	10.503	10.527
KRT33B	7.604	7.581	7.383	7.740	7.881	7.712
CGGBP1	10.349	10.346	10.459	10.232	10.190	10.229
ZFP41	8.739	8.791	8.858	8.421	8.726	8.657
ZFP42	5.383	5.605	5.463	5.515	5.390	5.564
CD244	6.774	6.772	6.774	6.901	6.788	6.974
AL078622.1	5.137	5.385	5.209	5.319	5.468	5.228
MYADML2	6.331	6.522	6.510	6.802	6.698	6.826
LINC00265-3P	10.722	10.564	10.478	10.430	10.307	10.610

AL159163.1	12.540	12.644	12.842	12.555	12.522	12.654
IPP	8.127	7.903	8.145	7.809	7.817	7.915
PLOD3	10.174	10.288	10.422	10.235	10.217	10.351
ESRRAP1	9.534	9.735	9.816	9.729	9.734	9.654
LDLRAD3	8.397	8.409	8.384	8.070	8.006	8.036
HPCA	6.566	6.624	6.557	6.682	6.883	6.456
CGAS	7.136	7.265	7.384	7.223	6.969	7.092
AC022149.1	12.677	12.851	12.862	12.813	12.633	12.690
APEH	10.229	10.229	10.390	10.055	9.986	9.981
DEFB132	4.644	4.624	4.597	4.722	4.737	4.729
LINC02119	4.115	4.446	4.353	4.370	4.292	4.320
LINC02114	5.155	5.107	5.099	5.217	5.052	4.965
LINC02116	5.963	5.972	5.920	5.925	5.990	6.178
AL445493.1	9.978	10.140	10.173	10.139	10.066	10.158
LINC02112	5.409	5.216	5.260	5.186	5.379	5.333
DPY19L2P4	4.777	4.763	4.662	4.758	4.617	4.757
KCTD12	8.092	7.927	8.082	8.002	8.161	7.862
CRCP	9.509	9.553	9.556	9.622	9.602	9.386
KCTD10	9.772	9.785	9.815	9.569	9.499	9.615
ANKUB1	6.272	6.153	6.239	6.375	6.088	6.173
DPY19L2P1	4.777	4.763	4.662	4.758	4.617	4.757
DPY19L2P2	5.314	5.488	5.033	5.148	5.331	5.554
DPY19L2P3	5.686	5.486	5.660	5.468	5.384	5.550
KCTD19	7.038	7.164	7.071	7.132	7.219	7.296
KCTD18	8.121	8.136	8.161	7.904	7.879	8.039
AL035666.1	6.844	6.705	6.457	6.700	6.637	6.466
AC104458.1	4.655	4.476	4.649	4.545	4.556	4.533
H6PD	8.643	8.593	8.729	8.507	8.674	8.595
SLC25A35	9.077	8.978	9.027	8.989	8.823	9.048
SLC25A34	8.132	8.193	8.143	8.042	7.936	8.151
SLC25A37	9.299	9.234	9.064	9.269	8.940	9.257
SLC25A36	10.939	10.769	10.958	10.652	10.984	10.728
SLC25A31	3.882	3.877	3.787	3.840	3.781	4.011
SLC25A30	9.102	9.043	9.077	8.616	8.841	8.850
SLC25A33	8.877	9.046	9.108	9.050	9.163	9.088
GPBAR1	7.980	8.061	7.892	7.984	8.210	8.062
SLC25A39	12.063	12.182	12.306	12.120	12.127	12.071
SLC25A38	9.572	9.715	9.914	9.554	9.543	9.550
TUBA1C	14.194	14.203	14.197	14.128	14.136	14.119
TUBA1B	13.856	13.887	13.923	13.767	13.791	13.823
TUBA1A	13.856	13.887	13.923	13.767	13.791	13.823
FAR2P1	5.675	5.822	5.573	6.014	5.852	5.828
FAR2P3	5.900	5.639	5.389	5.648	5.896	5.903
FAR2P2	5.900	5.822	5.573	6.014	5.896	5.903
FAM102B	8.762	8.662	8.818	8.351	8.457	8.491
FAM102A	10.457	10.637	10.688	10.374	10.218	10.223
RIMKLA	8.819	8.909	9.199	8.985	9.006	8.944
RIMKLB	8.984	8.916	8.883	9.278	9.212	9.325
PCID2	8.735	8.931	9.034	8.936	8.918	8.954
POMT1	7.334	7.191	7.306	7.333	7.168	7.337
POMT2	8.487	8.502	8.706	8.432	8.574	8.533
AIF1	8.546	8.573	8.472	8.623	8.572	8.645
AC098582.1	7.966	7.813	7.854	7.928	7.865	7.830
AGPS	9.652	9.736	9.702	9.697	9.471	9.583
LINC02809	7.728	7.876	7.883	8.030	8.086	8.034
LINC02806	7.855	7.714	7.795	7.658	7.857	7.820

LINC02805	6.444	6.325	6.294	6.583	6.449	6.548
LINC02802	7.347	7.553	7.386	7.650	7.400	7.673
LINC02800	5.624	5.765	5.941	6.064	6.049	5.924
STOM	10.258	10.263	10.273	9.958	10.017	9.860
EFCC1	7.550	7.282	7.182	7.511	7.553	7.396
PRAMEF28P	7.406	7.569	7.636	7.983	7.893	7.730
NPLOC4	10.508	10.579	10.728	10.457	10.519	10.425
TMEM86A	8.968	8.901	8.926	8.956	8.849	8.908
TMEM86B	7.007	6.930	7.112	7.097	7.013	7.136
LGALS13	5.492	5.600	5.529	5.604	5.547	5.688
LGALS12	7.390	7.395	7.361	7.392	7.423	7.375
LGALS14	6.929	7.022	6.993	7.247	7.171	7.366
SSBP4	8.967	9.156	9.231	9.055	8.980	8.936
TBX18-AS1	5.064	4.884	4.861	5.008	4.964	4.926
AC044860.1	8.456	8.424	8.630	8.777	8.518	8.587
PBLD	7.027	7.074	7.099	6.469	6.494	6.769
OR5K1	4.068	4.153	4.034	4.223	4.301	4.275
CNN3P1	5.749	5.620	5.811	5.803	5.929	6.032
AC103853.2	5.179	5.331	5.497	5.224	5.360	5.347
PGPEP1	9.272	9.306	9.455	9.153	9.130	9.309
SMU1	9.237	9.000	9.139	8.950	8.900	8.943
AC009237.3	7.980	7.984	8.120	7.953	7.974	8.081
SSRP1	10.849	10.826	10.799	10.707	10.639	10.713
IRAK1BP1	6.288	6.531	6.801	6.289	6.763	6.258
MIR770	6.949	6.858	7.029	7.206	7.166	7.098
AC131971.1	8.192	8.393	8.294	8.448	8.466	8.384
C1orf174	8.510	8.660	8.667	8.189	8.530	8.459
LINC00408	6.588	6.633	6.470	6.519	6.640	6.579
RBBP8NL	8.089	8.118	8.455	8.501	8.371	8.409
LINC00605	7.231	7.216	7.082	6.982	7.220	7.401
GPC1	9.756	9.829	9.929	9.556	9.513	9.605
GPC2	7.095	7.217	7.359	7.621	7.299	7.107
AC092647.5	10.629	10.703	10.894	10.627	10.639	10.605
GPC4	9.634	9.504	9.761	9.191	9.038	9.199
GPC5	4.496	4.558	4.314	4.574	4.549	4.336
GPC6	6.366	6.370	6.396	6.412	6.497	6.505
MPHOSPH10	9.856	9.878	9.877	9.761	9.770	9.539
AC034111.1	7.125	7.155	7.119	7.165	7.402	7.169
AC015712.6	7.950	7.805	7.816	7.846	7.795	7.730
ANKDD1A	6.865	7.131	6.917	6.882	6.789	7.152
NYX	7.728	7.564	7.677	8.211	7.829	7.694
AL136295.15	10.845	10.861	11.040	10.826	10.739	10.727
AL136295.11	9.786	9.800	9.901	9.508	9.462	9.477
AL136295.10	11.340	11.371	11.352	11.303	11.317	11.334
AL136295.13	6.938	7.101	7.036	7.140	7.285	7.004
AL136295.12	10.845	10.861	11.040	10.826	10.739	10.727
GSC2	4.904	5.014	4.981	4.988	5.282	5.024
JMJD1C-AS1	7.056	7.186	7.176	7.436	7.137	7.140
AL136984.1	5.883	5.823	5.757	5.930	5.869	5.768
ORM2	5.813	5.899	5.602	5.708	5.759	5.900
POGLUT3	9.531	9.212	9.367	9.078	9.228	9.179
CYCSP33	7.900	7.894	7.906	7.985	7.974	7.954
NXF2B	6.784	6.838	6.902	6.852	7.061	6.978
ALDH3A2	9.973	9.961	9.783	9.913	9.943	9.868
ALDH3A1	6.594	6.657	7.047	7.281	7.209	7.143
EDC3	9.896	9.960	10.161	10.050	9.949	9.941

EDC4	9.079	9.178	9.367	8.895	8.914	8.951
ATXN3L	5.690	5.481	5.687	6.100	5.647	5.837
AL359182.1	5.602	5.625	5.523	5.600	5.688	5.518
AL049747.1	8.337	8.191	8.030	8.700	8.431	8.720
PAICSP4	10.643	10.648	10.688	10.439	10.364	10.463
ANAPC1	9.207	9.103	9.217	9.018	9.021	8.942
ANAPC2	8.337	8.492	8.559	8.357	8.387	8.489
ANAPC5	11.583	11.665	11.808	11.588	11.468	11.528
ANAPC4	8.814	8.742	8.879	8.483	8.683	8.608
ANAPC7	10.289	10.392	10.473	10.458	10.250	10.261
AMZ1	8.171	8.332	8.295	8.337	8.283	8.325
PSMD14	11.873	11.860	11.992	11.954	11.853	11.839
PSMD11	11.102	11.333	11.467	11.275	11.282	11.093
PSMD10	10.778	10.785	10.795	10.736	10.747	10.876
PSMD13	9.980	10.133	10.240	9.976	9.841	9.934
PSMD12	10.930	10.968	11.091	10.962	10.955	10.896
AC026471.3	7.553	7.570	7.439	7.673	7.700	7.728
AC026471.4	6.726	7.027	6.722	6.843	6.897	6.811
MYO10	10.403	10.423	10.314	10.185	10.076	10.262
AC007613.1	4.755	4.866	4.738	5.059	4.871	4.894
AC114490.1	7.261	7.555	7.506	7.391	7.355	7.416
AC114490.3	7.261	7.555	7.506	7.391	7.355	7.416
AC114490.2	9.802	9.869	9.954	9.753	9.826	9.864
AC138409.2	6.208	6.243	5.920	6.406	6.416	6.188
RPS3AP6	13.488	13.531	13.648	13.817	13.801	13.836
MOBP	7.071	7.147	7.105	7.244	7.219	7.054
RPS3AP5	13.488	13.531	13.648	13.817	13.801	13.836
MYLK4	6.379	6.467	6.275	6.188	6.312	6.324
MYLK2	8.424	8.688	8.538	8.729	8.537	8.591
CNOT6LP1	5.603	5.684	5.875	5.635	5.559	5.627
TRMT44	7.965	8.088	8.114	7.944	7.842	8.078
MOB2	9.038	9.015	9.234	8.801	8.652	8.840
RNU6-1083P	8.782	8.890	8.861	8.891	8.885	9.080
MOB4	10.163	10.202	10.280	10.268	10.275	10.270
LRRC27	7.820	7.975	7.768	7.683	7.974	7.987
LRRC23	8.045	8.143	8.203	8.176	8.260	8.102
AL513188.1	5.134	4.794	4.782	4.904	4.921	4.888
AC009041.3	8.268	8.417	8.423	8.336	8.487	8.414
HMGB3	10.324	10.364	10.392	10.055	9.997	9.938
EEF1A1	14.499	14.538	14.562	14.577	14.462	14.579
EEF1A2	13.291	13.319	13.469	13.156	13.110	13.186
CHP1	10.707	10.808	10.862	10.682	10.646	10.810
CFAP126	6.003	6.050	5.974	6.034	6.093	6.159
CHP2	7.875	8.027	7.819	7.809	7.899	7.956
TCAF1P1	9.720	9.517	9.413	9.339	9.518	9.644
GCHFR	9.199	9.259	9.492	9.147	9.205	9.109
AC079416.3	5.053	4.890	4.969	4.902	4.730	4.716
AC079416.2	5.117	5.123	5.038	5.123	5.204	5.108
MNDA	4.803	4.396	4.827	5.390	5.020	4.975
MND1	7.208	7.071	7.294	6.609	6.915	6.882
GCNT7	4.940	4.901	4.891	5.208	5.219	4.922
HIKESHI	9.690	9.804	9.891	9.798	9.817	9.836
FGFR1OP	8.748	8.558	8.614	8.614	8.607	8.584
GCNT1	7.952	8.105	8.251	8.151	7.904	7.943
TPSAB1	7.692	7.866	7.807	8.093	8.088	7.961
CALY	5.774	5.839	5.953	6.043	5.866	5.700

CALR	11.111	11.317	11.327	11.587	12.007	11.411
ASAH2B	6.963	7.110	7.180	6.986	6.897	7.166
CHPF	10.770	10.867	11.069	10.954	10.846	10.844
COL23A1	6.763	6.747	6.751	6.858	6.895	7.023
AC084036.1	8.270	8.465	8.283	8.251	8.124	8.333
AL607035.1	7.096	6.651	6.702	6.865	6.557	6.666
RIBC2	6.755	6.702	6.953	6.651	6.861	6.710
RIBC1	7.294	7.411	7.493	7.448	7.485	7.358
APOC4-APOC2	8.568	8.617	8.592	8.694	8.621	8.673
NUTF2P2	10.828	10.703	10.997	10.559	10.661	10.303
C5orf49	5.450	5.030	5.214	5.379	5.551	5.392
CLEC1B	6.944	7.099	6.980	7.308	7.150	7.363
HTN3	4.489	4.487	4.626	4.574	4.549	4.632
C5orf46	7.404	7.373	7.444	7.401	7.377	7.550
AC018766.1	6.559	6.493	6.624	6.573	6.853	6.692
RFFL	9.325	9.558	9.479	9.166	9.317	9.363
ZFHX3	8.161	8.245	8.332	7.858	7.937	8.157
ZFHX2	6.638	6.560	6.618	6.730	6.753	6.948
RAB27A	10.089	10.150	10.168	9.991	9.938	9.846
RAB27B	11.187	11.192	11.135	11.087	10.953	11.066
LSAMP	7.551	7.563	7.526	7.567	7.694	7.528
C10orf71-AS1	6.465	6.459	6.469	6.582	6.639	6.623
FAM122A	8.151	8.234	8.415	7.891	8.150	8.073
FAM122B	9.392	9.532	9.435	8.902	8.904	9.207
FAM122C	7.165	7.315	7.128	7.467	7.348	7.304
MIR6891	13.705	13.724	13.757	13.585	13.558	13.528
AC090281.1	9.965	9.887	10.017	9.798	9.880	9.902
AC096541.1	7.653	7.781	7.744	7.660	7.876	7.884
AC011504.1	7.026	6.958	6.869	6.756	6.924	7.054
CYP2D6	8.206	8.266	8.340	8.286	8.325	8.436
CYP2D7	8.206	8.266	8.340	8.286	8.325	8.436
LNCARSR	7.306	7.507	7.232	7.419	7.420	7.621
AC096642.1	3.955	3.689	3.993	4.123	3.907	3.960
AC105339.5	8.264	8.257	8.119	8.123	8.228	8.058
ATXN7L2	6.018	6.088	6.124	6.043	5.986	6.045
CBX1	10.501	10.556	10.606	10.226	10.233	10.316
C10orf55	5.007	4.880	4.796	4.856	4.785	4.761
C10orf53	7.452	7.463	7.536	7.735	7.663	7.624
AC105339.1	7.590	7.761	7.674	8.150	7.848	8.077
SPRR4	9.453	9.421	9.365	9.377	9.684	9.586
SPRR3	9.245	9.551	9.505	9.398	9.529	9.429
DHX40	8.609	8.689	8.890	8.479	8.598	8.503
TRIM9	6.275	6.449	6.329	6.269	6.137	6.259
PFN1	13.168	13.264	13.408	13.209	13.298	13.043
PFN2	12.836	12.964	12.992	12.992	12.990	13.019
PFN4	7.293	7.208	7.017	7.190	7.211	7.224
AL121890.1	6.257	6.158	6.418	6.744	6.313	6.421
TRIM3	7.676	7.606	7.786	7.569	7.529	7.774
TRIM2	9.604	9.529	9.713	9.486	9.629	9.361
TRIM5	8.703	8.811	8.730	8.412	8.821	8.672
TRIM4	10.063	10.099	9.931	9.915	10.188	10.209
TRIM7	7.958	8.058	8.217	7.796	7.975	7.885
TRIM6	6.122	6.319	6.183	5.908	6.079	6.019
AC104590.1	5.422	5.842	5.727	5.800	5.967	6.227
PPA2	10.124	10.218	10.406	10.236	10.212	10.255
PPA1	11.683	11.804	11.955	11.914	11.855	11.817

AL031651.1	3.944	3.797	3.777	3.798	3.797	3.767
NUP62	10.932	10.905	11.125	10.716	10.576	10.566
TMCO2	6.628	6.973	7.034	7.214	7.168	7.017
CSF1R	5.871	6.026	6.006	5.984	5.842	6.007
SIPA1	8.029	8.031	8.122	8.030	7.993	8.001
RMND1	8.741	8.806	8.680	8.700	8.854	8.761
EIF3FP3	12.521	12.615	12.767	12.462	12.387	12.432
SSX2IP	9.173	9.257	9.132	8.718	9.032	9.070
ATXN8OS	7.213	7.430	7.371	7.438	7.356	7.326
LRAT	5.630	5.338	5.785	5.423	5.449	5.428
LINC01114	6.575	6.912	6.840	6.750	6.861	6.875
LINC01115	6.357	6.335	6.151	6.515	6.539	6.513
LINC01116	8.386	8.366	8.378	8.133	8.056	8.116
LINC01111	6.863	6.772	6.629	6.942	6.866	6.949
PPAN	9.059	8.957	9.093	9.138	9.012	8.925
LINC01118	7.790	7.684	7.727	7.841	7.801	7.811
LINC01119	7.687	7.878	7.791	7.790	7.906	7.932
PPAT	9.591	9.542	9.658	9.623	9.617	9.494
IDH1-AS1	7.458	7.478	7.684	7.384	7.441	7.246
AC138649.1	6.905	6.937	6.849	7.091	7.161	7.066
DCXR	10.560	10.622	10.862	10.611	10.465	10.551
NFYC	9.870	9.972	9.910	9.625	9.704	9.706
NFYB	9.046	9.079	9.097	8.790	8.832	8.870
NFYA	7.975	7.962	8.182	7.880	8.020	7.823
SMIM30	7.766	7.811	7.730	7.892	7.790	7.789
TST	10.677	10.694	10.934	10.435	10.513	10.470
DTD1-AS1	5.809	6.151	5.949	6.115	5.876	6.078
AL008638.2	7.272	7.615	7.364	7.679	7.460	7.763
AL512347.1	5.560	5.444	5.327	5.776	5.579	5.532
CES1P1	6.929	7.137	6.962	6.981	7.063	6.947
EBP	12.093	12.172	12.338	12.181	12.123	12.200
RAB1B	11.070	11.180	11.392	11.200	11.032	11.086
AC006077.2	4.950	4.909	4.952	4.796	5.304	5.087
NLE1	8.697	8.981	9.062	8.657	8.654	8.591
SKIL	10.154	10.102	9.956	9.999	9.819	10.043
CYTH1	8.722	8.836	8.868	8.843	8.879	8.841
GCGR	8.186	8.185	8.197	8.153	8.205	8.265
CYTH3	9.809	9.814	9.962	9.767	9.673	9.726
CYTH2	9.132	9.081	9.212	8.998	8.944	9.193
CYTH4	8.153	8.086	8.167	8.027	8.205	8.182
AL355140.1	8.098	8.106	8.034	7.727	8.064	8.007
DPH1	10.288	10.311	10.506	10.323	10.279	10.298
DPH2	9.043	9.109	9.171	9.029	9.059	8.970
DPH3	8.518	8.707	8.691	8.555	8.822	8.713
MYH7B	6.126	6.036	6.270	6.269	6.211	6.324
DPH5	9.474	9.499	9.516	9.609	9.538	9.526
DPH6	7.334	7.644	7.758	7.246	7.491	7.253
DPH7	8.016	8.074	8.171	8.213	8.203	8.336
LINC00242	6.169	6.411	6.479	6.284	6.484	6.365
LINC00240	4.748	4.726	4.430	4.773	4.692	4.815
KCNQ1DN	8.253	8.333	8.379	8.743	8.655	8.252
AC090229.1	5.056	5.191	5.192	5.036	5.027	5.120
PAH	7.246	6.961	7.054	6.437	6.380	6.613
PAM	10.544	10.574	10.801	10.471	10.320	10.560
DCN	8.044	8.252	7.993	8.127	8.127	8.229
DCK	8.229	8.398	8.456	7.902	8.040	8.035

DCD	6.330	6.418	6.440	6.807	6.361	6.648
THRB	9.069	9.184	9.251	8.807	8.966	8.883
DCC	6.872	6.758	6.530	6.977	6.927	6.948
THRA	9.020	8.972	9.150	8.966	8.998	9.018
AL021707.2	5.848	5.655	5.839	5.819	5.648	5.931
TAF4B	8.092	7.973	8.018	7.683	7.806	7.910
AL021707.6	7.128	7.035	6.935	7.143	7.073	6.985
DCX	6.403	6.453	6.662	6.722	6.333	6.515
C20orf173	6.393	6.644	6.526	6.895	6.682	6.652
AL139082.2	6.981	6.869	7.047	6.961	6.911	6.714
AL139082.1	8.012	7.896	7.716	8.128	7.926	7.957
CDH20	6.133	6.093	6.057	6.102	6.103	5.968
CDH22	9.129	9.044	8.900	9.082	9.058	9.171
CDH23	7.542	7.515	7.486	7.542	7.590	7.759
CDH24	8.998	8.951	8.997	8.936	9.113	8.939
KCNMB3P1	7.578	7.737	7.714	7.448	7.450	7.692
CDH26	7.241	7.354	7.328	7.331	7.381	7.243
FLRT3	9.196	9.153	9.239	9.132	9.406	9.127
FLRT2	6.929	6.765	6.624	6.998	7.006	6.850
AL157931.1	4.734	4.899	4.502	4.801	4.731	4.700
TRPT1	9.942	9.928	10.096	9.798	9.884	9.823
PXYLP1	8.238	8.276	8.243	8.111	8.096	8.202
ARCN1	10.685	10.794	10.755	10.574	10.581	10.730
LINC00939	5.992	6.029	6.054	5.889	5.629	6.053
AL590705.3	7.997	7.899	8.031	7.885	7.953	7.916
LINC00930	7.748	7.636	7.533	7.733	7.928	7.938
AC134879.2	8.398	8.520	8.424	8.478	8.571	8.522
LINC00934	5.872	5.989	5.813	5.811	6.169	6.062
LINC00937	6.760	6.529	6.864	6.842	6.754	6.930
AEN	9.015	9.154	9.225	9.196	9.222	9.052
AC063943.1	9.845	9.804	10.030	9.776	9.740	9.737
AC244167.1	4.136	4.329	4.011	4.241	4.217	4.217
PPP6C	10.276	10.286	10.316	10.288	10.189	10.253
SLC39A13	10.122	10.141	10.100	10.006	10.160	10.154
HSPA4L	8.185	8.210	8.362	8.393	8.267	8.286
PRAC2	6.921	7.079	6.981	7.375	7.112	7.090
PRAC1	4.857	5.121	4.699	5.186	4.906	4.821
MOS	6.257	6.348	6.139	6.206	6.208	6.297
SUZ12P1	9.402	9.196	9.277	8.975	9.347	8.904
MOK	9.667	9.664	9.722	9.453	9.411	9.593
EIF4EBP1	12.115	12.149	12.352	12.093	12.066	11.995
EIF4EBP3	8.747	8.747	8.902	8.630	8.674	8.756
ASPRV1	7.457	7.670	7.646	7.580	7.676	7.702
MOG	6.519	6.610	6.288	6.598	6.839	6.774
AC139256.2	8.029	7.870	7.644	7.214	7.397	7.841
AC139256.3	11.306	11.174	11.094	11.070	11.186	11.250
SH2D1B	6.527	6.504	6.292	6.359	6.170	6.512
AL035555.2	4.712	4.651	4.572	4.768	4.627	4.712
SH2D1A	6.824	6.808	6.771	6.960	6.991	6.812
LRRC37A2	7.316	7.496	7.592	7.563	7.519	7.583
LRRC37A3	7.316	7.496	7.592	7.563	7.519	7.583
PRR13P5	11.530	11.550	11.675	11.374	11.322	11.443
CCDC18-AS1	8.928	8.778	8.573	8.742	8.832	8.929
CSDC2	7.812	7.811	7.945	7.824	7.816	7.672
NDUFV2-AS1	6.611	6.538	6.768	6.980	6.811	6.858
AC004825.2	5.907	5.914	6.122	5.903	6.053	5.977

PDHA2	6.549	6.854	6.792	6.926	6.831	7.006
PDHA1	11.117	11.221	11.308	11.094	10.920	10.976
ERCC6	7.563	7.526	7.469	7.577	7.628	7.525
GZMA	5.642	5.780	5.711	5.691	5.477	5.734
STAU2-AS1	7.764	7.853	7.353	7.836	7.881	7.948
PCNAP3	4.083	4.259	4.393	3.968	3.926	4.224
RPL12P38	12.569	12.690	12.791	12.495	12.553	12.522
EEF1A1P13	14.499	14.538	14.562	14.577	14.462	14.579
CD247	5.962	6.112	5.807	6.046	5.957	6.232
RILPL1	8.607	8.853	8.956	8.885	8.805	8.945
RILPL2	10.790	11.084	11.280	10.964	11.019	10.900
DPH6-DT	4.178	4.276	4.304	4.519	4.597	4.600
EEF1A1P11	14.280	14.345	14.328	14.354	14.421	14.437
AL355994.2	7.755	7.704	7.729	7.806	7.881	7.816
FMR1	10.087	10.130	10.053	9.717	9.794	9.906
LYPD6	7.098	7.056	6.970	6.806	7.242	7.074
SEL1L2	5.679	5.861	5.594	5.886	5.926	5.808
LYPD4	7.174	7.206	7.094	7.248	7.421	7.223
LYPD5	6.966	7.123	7.119	7.039	7.442	7.076
LYPD3	8.453	8.459	8.484	8.305	8.375	8.375
LYPD1	8.180	8.283	8.280	7.930	8.080	7.882
CALCOCO1	8.754	8.844	8.870	8.601	8.764	8.867
CALCOCO2	11.077	11.112	11.176	10.958	10.988	10.951
LYPD8	7.089	7.222	7.067	7.041	6.937	6.994
LINC02303	4.625	4.528	4.485	4.528	4.697	4.533
ANKRD30A	4.496	4.616	4.478	4.574	4.602	4.692
RGS5	8.364	8.594	8.458	8.570	8.576	8.621
AC020661.1	10.211	10.212	10.269	10.262	10.426	10.364
AL445235.1	7.249	6.756	6.748	7.000	7.122	7.015
RGS1	4.070	4.054	4.149	4.194	4.252	4.142
RGS2	11.904	11.832	11.968	11.838	11.681	11.690
RGS3	9.477	9.558	9.649	9.370	9.301	9.376
RGS8	7.514	7.520	7.376	7.531	7.533	7.553
RGS9	6.493	6.357	6.729	6.369	6.597	6.528
AL359313.1	6.731	6.732	6.426	6.702	6.776	6.513
AL391834.1	4.377	4.377	4.381	4.425	4.463	4.501
ANKRD20A8P	3.767	3.749	3.901	3.720	3.792	3.690
FSD1L	5.984	5.900	5.627	6.077	5.917	6.076
AC073316.1	7.054	7.153	6.851	7.387	6.965	7.171
AL445433.2	4.335	4.119	4.282	4.659	4.237	4.295
FP671120.3	8.286	7.924	8.206	8.188	8.172	8.003
FP671120.2	6.476	6.270	6.787	6.705	6.102	6.706
FP671120.1	8.286	7.924	8.206	8.188	8.172	8.003
FP671120.7	10.511	10.453	10.731	11.036	10.643	10.881
DM1-AS	7.339	7.453	7.251	7.631	7.363	7.587
FP671120.5	4.239	4.005	4.058	4.351	4.227	3.969
FP671120.8	10.511	10.453	10.731	11.036	10.643	10.881
AC107214.1	6.728	6.843	6.878	6.858	6.995	6.951
CDK2AP1	12.147	12.177	12.205	12.100	12.154	12.144
CDK2AP2	9.628	9.645	9.864	9.577	9.496	9.603
LINC01657	5.084	5.353	5.183	5.133	5.235	5.294
LINC01656	6.827	6.882	6.731	6.924	6.869	6.814
LINC01654	5.414	5.563	5.337	5.494	5.391	5.468
LINC01653	6.403	6.431	5.958	6.200	6.405	6.437
MLYCD	9.468	9.573	9.699	9.548	9.558	9.400
CD28	6.391	6.364	6.366	6.546	6.462	6.493

MRPS9	9.111	9.116	9.231	8.902	8.892	8.899
MRPS7	10.291	10.373	10.645	10.484	10.378	10.451
MRPS6	10.167	10.285	10.406	10.224	10.136	10.295
MRPS5	10.473	10.483	10.706	10.411	10.264	10.422
IFI35	11.089	11.137	11.316	11.060	11.120	10.890
CD27	7.379	7.056	7.056	7.133	7.191	7.219
MRPS2	9.243	9.425	9.526	9.250	9.184	9.153
IFI30	10.026	10.127	10.117	10.085	10.164	10.065
CD24	13.975	14.040	14.019	14.031	13.962	14.052
AC008993.2	5.019	5.179	5.401	5.395	5.216	5.137
AC063926.2	8.044	8.395	8.133	8.259	8.237	8.179
NKIRAS2	10.204	10.238	10.504	10.154	10.026	10.139
NKIRAS1	7.599	7.629	7.747	7.557	7.673	7.722
FAT3	5.234	5.209	5.148	5.274	5.286	5.207
FAT2	6.765	6.888	7.018	6.901	6.854	6.819
FAT1	10.909	10.869	10.834	10.624	10.742	10.787
FAT4	7.071	7.057	7.131	6.729	6.976	6.983
PSG6	7.609	7.750	7.755	8.002	7.779	7.654
PSG7	8.212	8.133	7.864	8.079	8.062	8.018
PSG4	8.388	8.333	8.203	8.201	8.140	8.154
PSG5	7.976	7.702	7.538	7.655	7.867	7.714
PSG2	6.902	6.951	6.887	6.865	7.069	6.962
PSG3	8.617	8.629	8.598	8.547	8.671	8.623
PSG1	9.180	9.177	9.187	9.195	9.290	9.265
TMEM248	10.120	10.216	10.360	10.079	10.108	10.093
MYRF-AS1	8.270	8.452	8.399	8.351	8.386	8.367
AL121845.2	7.705	7.946	8.090	7.997	7.924	7.980
AL121845.3	8.435	8.378	8.528	8.112	8.208	8.295
AC124016.2	4.524	4.473	4.538	4.409	4.524	4.620
AC124016.3	5.842	5.971	6.061	5.788	5.908	5.830
AL596276.2	7.098	6.895	6.942	7.665	7.441	7.457
HMBS	9.619	9.611	9.771	9.438	9.409	9.424
TMEM241	9.629	9.762	9.684	9.604	9.685	9.666
TMEM242	8.865	9.018	9.127	8.840	8.890	8.842
AC127024.8	6.336	6.306	6.048	6.162	6.099	6.297
AKR7A2	9.897	9.985	10.145	9.770	9.942	9.849
AKR7A3	9.220	9.268	9.374	9.137	9.077	9.156
SOCAR	5.629	5.374	5.700	5.650	5.538	5.584
AC126763.1	7.182	7.381	7.328	7.421	7.457	7.500
DDA1	9.813	9.966	10.057	9.715	9.652	9.749
SOST	5.358	5.328	4.900	5.417	5.796	5.318
OR4F29	7.745	7.729	7.361	7.729	7.847	7.903
U73169.1	7.309	7.393	7.433	7.394	7.612	7.865
OR4F21	7.745	7.729	7.361	7.729	7.847	7.903
AL450469.1	6.263	6.102	5.825	6.164	6.325	6.277
SSTR5-AS1	7.987	8.036	7.945	8.007	8.080	8.104
TRBC1	7.126	7.105	7.169	7.322	7.278	7.266
TRBC2	7.126	7.091	7.122	7.322	7.278	7.266
PTPMT1	9.047	9.119	9.270	9.095	9.078	9.068
AL034369.1	3.903	3.908	4.029	4.191	3.960	4.043
CTAGE8	10.096	10.073	10.117	10.027	10.081	10.109
BMS1P22	4.384	4.179	4.266	4.012	4.127	4.194
CTRB2	7.771	7.821	7.820	7.688	7.974	7.794
PLCD4	6.989	6.775	6.938	6.732	7.040	7.132
CTRB1	6.442	6.495	6.767	6.824	6.661	6.516
PLCD3	8.935	8.895	8.991	8.859	8.942	8.873

PLCD1	9.358	9.318	9.159	9.397	9.423	9.334
OR8B8	7.427	7.701	7.665	7.511	7.717	7.684
SOS2	8.799	8.703	8.725	8.470	8.597	8.657
SOS1	8.786	8.669	8.653	8.486	8.489	8.595
AL512634.1	4.948	5.010	4.903	4.956	5.106	4.875
OR4F2P	7.745	7.729	7.361	7.729	7.847	7.903
INSYN1	7.639	7.830	7.704	7.628	7.764	7.924
GNMT	6.814	6.865	6.898	6.841	6.792	6.841
CAB39	10.750	10.763	10.937	10.704	10.606	10.656
CKMT1A	7.201	7.173	7.233	7.197	7.363	7.132
CKMT1B	7.201	7.173	7.233	7.197	7.363	7.132
DEFA1B	6.338	6.348	6.335	6.003	6.148	6.540
BCL2L15	8.338	8.407	8.289	8.484	8.371	8.365
BCL2L14	8.175	8.256	8.106	8.152	8.271	8.089
BCL2L11	9.717	9.675	9.485	9.720	9.715	9.686
BCL2L10	5.899	6.061	6.176	6.358	6.170	6.057
BCL2L13	9.676	9.846	9.798	9.568	9.508	9.688
NTMT1	9.095	9.206	9.264	9.057	9.160	9.176
FAM27C	8.133	8.243	8.349	8.600	8.309	8.374
FAM27B	7.218	7.453	7.410	7.437	7.268	7.258
C22orf15	8.108	8.120	8.141	8.164	8.183	8.116
HSD11B2	7.254	7.186	7.377	7.241	7.196	7.169
HSD11B1	6.793	6.966	6.947	6.599	7.008	6.753
ISYNA1	9.445	9.517	9.690	9.269	9.256	9.391
GTF2H3	9.619	9.234	9.162	8.940	8.885	9.033
GTF2H2	8.310	8.418	8.490	8.307	8.382	8.244
GTF2H1	8.922	8.948	8.949	8.810	9.035	8.699
GTF2H5	9.607	9.921	10.039	10.093	10.050	9.907
GTF2H4	7.897	7.741	8.031	7.471	7.439	7.562
ERVH-1	10.973	10.983	10.648	11.124	11.199	11.048
ALDH1B1	8.178	8.281	8.294	8.002	8.112	8.158
COX7A2L	11.504	11.499	11.702	11.666	11.658	11.693
AC112907.3	4.212	4.263	4.196	4.452	4.297	4.386
AC087071.1	6.522	6.825	6.805	6.966	7.008	6.782
SMARCC2	9.862	9.915	10.055	9.805	9.812	9.773
SMARCC1	10.459	10.581	10.546	10.304	10.753	10.209
RYK	10.654	10.686	10.809	10.687	10.469	10.651
CU638689.4	7.829	7.863	7.877	7.856	7.805	7.700
AC098487.1	6.126	6.454	6.259	6.332	6.370	6.498
IFNW1	5.811	5.838	5.918	5.969	5.780	5.898
BLOC1S6	9.703	9.666	9.702	9.508	9.633	9.463
BLOC1S5	9.019	8.999	8.979	8.806	8.840	9.008
BLOC1S4	9.608	9.651	9.803	9.394	9.497	9.574
LHX4	6.558	6.716	6.914	6.796	6.834	6.993
BLOC1S2	10.295	10.377	10.461	10.151	10.164	10.222
BLOC1S1	10.050	10.072	10.332	10.293	10.235	10.110
LHX8	3.892	3.899	3.780	3.879	3.842	3.829
LHX9	6.421	6.510	6.756	6.619	6.431	6.836
MAMDC2-AS1	5.127	5.288	5.288	5.447	5.225	5.303
LINC00323	3.948	4.026	4.197	4.401	4.093	4.103
NADK2	8.954	8.890	8.952	8.621	8.652	8.341
SLC38A5	6.885	6.934	7.109	7.086	7.049	7.057
SLC38A4	4.962	5.234	4.874	5.552	5.162	5.144
SLC38A7	9.278	9.296	9.351	8.942	9.318	9.345
SLC38A6	7.289	7.335	7.495	7.270	7.305	7.364
SLC38A1	12.088	12.156	12.244	11.980	11.875	11.976

AL353796.1	6.066	6.004	6.036	6.099	6.168	6.172
SLC38A3	6.731	6.802	7.030	6.760	6.990	6.832
SLC38A2	11.801	11.759	11.754	11.702	11.527	11.784
BX005432.1	6.517	6.543	6.395	6.499	6.649	6.716
SLC38A9	8.415	8.402	8.310	8.048	8.055	8.236
OCA2	9.003	9.152	9.061	9.001	8.994	9.058
Z97180.1	10.036	9.985	9.986	9.848	9.900	9.878
MIR3945HG	7.091	7.153	7.028	7.132	7.101	7.257
AP002990.1	14.111	14.146	14.160	14.135	14.090	14.165
AP000560.1	5.470	5.292	5.273	5.275	5.358	5.235
AC083973.1	8.153	8.342	8.351	8.312	8.365	8.489
YBX1	13.393	13.414	13.539	13.442	13.322	13.412
YBX3	12.273	12.313	12.408	12.150	12.125	11.997
YBX2	9.460	9.339	9.248	9.417	9.839	9.655
AP000880.1	5.896	5.913	6.010	5.969	6.088	6.354
AL138976.2	4.723	4.807	5.130	4.735	4.926	4.790
STIP1	11.081	11.191	11.337	11.127	10.987	11.151
CHIC2	9.302	9.344	9.538	9.168	9.136	9.256
CHIC1	7.728	7.786	7.822	7.478	7.491	7.637
POM121L4P	7.776	7.889	7.804	7.722	8.075	7.864
AL161668.1	4.969	4.927	4.629	5.122	4.957	4.850
AL161668.3	6.901	6.937	6.993	7.207	7.313	7.179
TRBV21-1	5.949	6.335	6.236	6.095	6.348	6.350
PCF11-AS1	7.497	7.389	7.244	7.356	7.580	7.283
AC106038.1	5.724	5.757	5.978	5.999	5.901	5.951
EHMT2-AS1	5.620	5.286	5.539	5.721	5.604	5.547
TYRL	7.414	7.507	7.163	7.663	7.703	7.699
VSNL1	7.700	7.526	7.345	7.717	7.796	7.707
SDCCAG8	7.658	7.608	7.632	7.394	7.253	7.588
CDIPT	11.677	11.688	11.881	11.538	11.581	11.558
NCOA2	8.699	8.639	8.785	8.180	8.327	8.594
NCOA3	10.563	10.473	10.506	10.326	10.764	10.203
NCOA1	9.441	9.592	9.630	9.510	9.708	9.427
NCOA6	9.570	9.694	9.778	9.569	9.455	9.691
NCOA7	11.314	11.348	11.310	11.357	11.338	11.265
NCOA4	11.694	11.775	11.853	11.717	11.634	11.747
NCOA5	9.052	9.191	9.342	9.152	9.058	9.178
ZFHX4-AS1	8.614	8.171	8.161	8.776	8.699	8.679
AC232271.1	7.702	7.589	7.600	7.843	7.792	7.766
CISH	8.308	8.315	8.229	8.219	8.256	8.387
PURPL	3.733	3.761	3.895	3.738	3.717	3.695
AP003471.1	5.959	6.227	6.110	6.104	5.991	6.117
RIF1	9.048	9.006	8.961	8.453	8.777	8.669
STARD3NL	11.295	11.380	11.412	11.268	11.256	11.352
SREK1	9.838	9.764	9.791	9.662	9.557	9.817
ERO1B	6.639	6.642	6.481	6.630	6.433	6.518
ERO1A	11.595	11.704	11.713	11.498	11.454	11.526
TMSB15B-AS1	5.924	6.147	5.702	5.768	5.645	6.029
MPO	6.708	6.756	6.733	6.818	6.859	6.812
CDIP1	8.259	8.213	8.368	8.215	8.277	8.265
GUCY1B2	6.282	6.344	6.177	6.400	6.490	6.577
WT1-AS	7.917	7.702	7.673	7.865	7.964	7.912
AC008074.2	5.337	5.516	5.319	5.402	5.391	5.323
ALKAL2	5.350	5.357	5.367	5.174	5.302	5.192
ZNF497	5.638	5.705	5.712	5.713	5.750	5.842
ZNF496	9.156	9.182	9.417	9.093	9.186	9.117

ERF	9.717	9.929	10.079	9.842	9.867	9.846
ERG	6.319	6.593	6.611	6.484	6.512	6.593
ZNF493	6.373	6.647	6.700	6.776	6.734	6.773
HAND1	7.651	7.685	7.654	7.787	7.825	7.749
HAND2	6.492	6.364	6.393	6.595	6.544	6.515
ZNF490	7.326	7.388	7.414	7.382	7.419	7.477
ERH	12.521	12.666	12.736	12.729	12.749	12.652
AC092070.2	7.456	7.369	7.447	7.515	7.336	7.472
AC092070.4	5.688	5.713	5.630	5.580	5.656	5.832
NLGN4Y	6.516	6.497	6.403	6.446	6.481	6.440
NLGN4X	5.654	5.558	5.719	5.724	5.797	5.710
FBN2	5.993	5.772	5.863	5.990	6.199	6.084
NCR2	8.571	8.650	8.734	8.844	8.795	8.683
NCR1	7.716	7.884	7.662	7.998	7.979	7.906
FBN1	6.324	6.227	6.477	6.794	6.605	6.798
AC046158.3	6.135	6.143	6.176	6.476	6.267	6.290
AC046158.4	6.224	6.402	6.466	6.425	6.424	6.491
AOAH	7.049	6.905	6.911	6.973	7.094	6.820
MPZ	7.490	7.437	7.355	7.606	7.611	7.621
SCN1B	8.397	8.477	8.642	8.754	8.676	8.751
SCN1A	5.572	5.340	5.217	5.625	5.778	5.734
EEPD1	10.255	10.269	10.320	10.147	10.047	10.066
FRYL	9.416	9.461	9.477	9.309	9.291	9.441
DEXI	9.491	9.546	9.662	9.560	9.496	9.492
FUOM	8.603	8.662	8.678	8.356	8.443	8.314
IDI1	12.071	12.140	12.267	12.269	12.104	12.201
IDI2	7.168	7.296	7.339	7.466	7.214	7.356
IPMK	6.669	6.500	6.670	6.474	6.449	6.402
AC090531.1	5.616	5.697	5.791	5.824	5.705	5.652
TMEM185B	9.978	10.070	10.028	9.716	9.772	9.809
TMEM185A	9.289	9.329	9.281	9.132	9.130	9.134
UBXN10	8.199	8.160	8.116	8.519	8.399	8.253
UBXN11	9.417	9.372	9.419	9.344	9.608	9.371
LINC02670	4.644	4.575	4.605	4.663	5.045	4.825
LINC02673	8.051	8.063	7.935	8.044	8.023	8.016
SNORD12C	10.936	11.054	11.301	11.263	11.275	11.341
RNU6-507P	5.970	6.047	5.829	6.033	5.895	6.053
POTEM	7.172	7.412	7.335	7.491	7.419	7.383
AC079142.1	6.899	7.056	7.008	6.982	7.147	6.978
RAB42P1	8.135	8.157	8.246	8.131	8.303	8.226
SFTA1P	6.680	6.659	6.920	6.690	6.989	6.873
FCHSD1	7.714	7.705	7.779	7.467	7.481	7.477
FCHSD2	8.048	7.904	8.054	7.711	7.890	8.075
IGHEP1	7.276	7.223	7.125	7.050	7.170	7.144
HIGD2A	11.415	11.480	11.661	11.405	11.423	11.326
AC103702.2	8.098	7.978	7.990	8.131	8.026	8.071
AC103702.1	7.451	7.270	7.292	7.498	7.274	7.502
AC091965.4	5.108	4.746	4.879	5.001	5.017	4.964
UBA52	12.962	13.084	13.150	12.954	12.859	12.945
DND1	9.778	9.851	9.856	9.219	9.772	9.656
AC091965.1	5.108	4.746	4.879	5.001	5.017	4.964
NOBOX	8.501	8.508	8.567	8.560	8.719	8.598
AL845464.1	7.787	7.777	7.897	8.212	8.057	7.746
IBSP	6.456	6.428	6.641	6.998	6.538	6.742
AC044839.1	5.867	5.764	5.698	5.847	5.770	5.577
AL583835.2	6.350	6.477	6.338	6.353	6.173	6.488

NID2	5.229	5.333	5.252	5.321	5.270	5.274
NID1	7.254	7.009	7.235	7.011	7.241	7.115
DLGAP1-AS1	7.917	7.949	8.056	8.034	7.971	8.214
DLGAP1-AS2	7.365	7.368	7.300	7.127	7.168	7.232
DLGAP1-AS3	5.502	5.587	5.323	5.374	5.417	5.400
FCER1A	4.335	4.420	4.327	4.302	4.311	4.365
SOSTDC1	8.023	8.269	8.472	7.810	7.879	7.922
ELOB	12.393	12.505	12.658	12.648	12.545	12.527
PAXIP1-AS1	8.076	7.784	7.968	7.549	7.559	7.662
PAXIP1-AS2	6.977	7.246	7.116	7.086	7.014	7.055
ELOA	10.098	9.905	9.985	10.040	10.087	9.900
OSTM1	7.531	7.620	7.642	7.395	7.675	7.740
AL031666.1	5.114	4.757	4.760	5.185	5.071	5.080
DAW1	5.388	5.373	5.229	5.606	5.541	5.370
AL031666.3	9.183	9.150	9.037	9.250	8.970	9.182
AL031666.2	5.077	5.036	5.240	4.975	5.163	5.201
OR4D1	7.855	7.740	7.725	7.756	7.947	8.016
OR4D2	7.350	7.308	6.960	7.425	7.321	7.415
AC245128.25	7.716	7.884	7.662	7.998	7.979	7.906
FANCG	9.440	9.429	9.471	8.965	9.190	9.214
CFLAR	10.894	10.911	10.931	10.759	10.850	10.785
FANCE	8.057	8.300	8.403	8.096	8.009	8.129
FANCC	8.350	8.398	8.410	8.527	8.415	8.405
FANCB	6.028	5.925	5.852	6.046	5.871	5.874
FANCA	8.480	8.676	8.774	8.210	8.364	8.496
HNRNPAB	12.549	12.543	12.630	12.336	12.312	12.320
FANCM	7.547	7.433	7.524	7.765	7.612	7.577
FANCL	9.256	9.205	9.237	8.871	8.882	8.997
FANCI	10.136	10.053	10.198	9.472	9.532	9.662
CA5B	8.189	8.278	8.288	8.297	8.492	8.614
ENTPD3-AS1	4.487	4.648	4.612	4.638	4.585	4.555
F13B	4.381	4.410	4.352	4.334	4.421	4.486
HNRNPA3	11.051	11.098	11.160	10.846	10.830	10.950
HNRNPA0	10.571	10.716	10.981	10.652	10.582	10.341
HNRNPA1	13.482	13.508	13.526	13.344	13.317	13.319
ADGRD2	7.671	7.428	7.569	7.959	7.839	7.704
ADGRD1	5.102	5.253	5.185	5.305	5.220	5.261
AL121894.2	5.282	5.464	5.425	5.333	5.208	5.513
HOXA7	5.769	5.838	5.989	5.943	5.917	5.913
HOXA6	7.609	7.443	7.654	8.052	7.640	7.704
HOXA5	8.613	8.553	8.720	8.518	8.549	8.573
HOXA4	7.051	7.236	7.286	7.219	7.218	7.138
HOXA3	7.711	7.974	7.940	7.661	7.741	7.633
HOXA2	6.009	6.070	5.953	5.892	6.205	6.078
HOXA1	6.727	6.735	6.478	6.819	6.564	6.730
IGLL1	5.806	5.779	5.849	5.808	5.913	5.914
HOXA9	6.677	6.700	6.874	7.312	6.958	6.944
CEP57	9.490	9.648	9.781	9.628	9.503	9.541
PRSS3P2	10.172	10.309	10.434	10.312	10.298	10.353
CEP55	10.188	10.146	10.198	9.598	9.765	9.718
AL121759.1	4.871	5.121	5.269	5.045	5.057	4.975
HSPD1	13.826	13.845	13.862	13.917	13.851	13.843
AC087672.2	5.831	6.091	5.751	5.959	6.002	5.847
MTX3	8.822	8.762	8.920	8.430	8.488	8.565
MTX2	9.847	9.971	9.962	9.928	9.916	9.774
MTX1	10.838	10.813	11.024	10.681	10.718	10.575

AC010533.1	7.512	7.761	7.741	7.553	7.759	7.835
PNMT	8.311	8.328	8.554	8.464	8.543	8.517
SLC25A16	6.844	6.854	7.097	6.669	6.579	6.606
LIAS	7.736	7.710	7.901	7.188	7.295	7.473
ATP10A	8.268	8.480	8.534	8.486	8.402	8.599
ATP10B	8.156	8.347	8.058	8.166	8.370	8.183
ATP10D	8.199	8.191	8.231	8.076	8.066	8.218
KRIT1	8.402	8.514	8.443	7.512	7.834	7.423
TRABD2A	7.720	7.588	7.855	7.947	7.907	7.904
TRABD2B	7.107	7.414	7.431	7.538	7.314	7.403
CDPF1	8.091	7.968	8.009	8.116	8.158	8.069
SLC6A20	8.456	8.469	8.468	8.501	8.451	8.550
SCRIB	9.598	9.674	9.835	9.458	9.445	9.527
TYMS	10.929	10.939	11.053	10.715	10.753	10.740
AC270239.1	7.048	6.865	6.859	7.005	7.107	7.121
WWP2	9.202	9.027	9.087	8.775	8.775	8.835
WWP1	11.058	11.110	11.041	10.985	11.111	10.768
DAPL1	9.849	9.926	10.029	9.810	9.880	9.911
TPI1	13.500	13.534	13.621	13.465	13.430	13.505
AC007255.1	6.793	6.632	6.583	6.909	6.790	7.003
LINC01122	6.896	7.143	7.054	6.856	6.983	6.943
MFSD14B	10.050	10.048	10.024	9.801	9.761	9.726
MFSD14C	9.390	9.558	9.403	9.220	9.414	9.178
PRTN3	5.189	5.493	5.141	5.360	5.316	5.219
MFSD14A	9.937	9.964	10.062	9.824	9.715	9.786
CTSH	12.734	12.803	12.913	12.753	12.704	12.705
CTSK	8.035	7.988	7.834	7.881	7.901	8.104
CTSL	9.086	8.929	9.190	8.897	8.951	8.947
CTSO	9.631	9.589	9.737	9.381	9.604	9.104
CTSA	11.819	11.805	11.944	11.622	11.495	11.748
CTSB	11.522	11.560	11.685	11.465	11.346	11.369
CTSC	12.886	12.926	13.029	12.772	12.735	12.779
CTSD	11.873	11.946	12.075	11.958	11.893	11.876
CTSE	10.090	10.224	10.175	10.056	10.298	10.105
CTSF	7.860	7.867	7.915	7.931	8.038	8.002
CTSG	8.196	8.393	8.082	8.263	8.182	8.258
CTSZ	8.532	8.965	9.410	10.381	9.245	8.298
CTSS	10.526	10.656	10.685	10.468	10.595	10.425
CTSV	9.146	9.212	9.428	8.796	8.896	8.983
CTSW	7.827	7.953	7.852	7.897	7.966	8.056
AC004130.2	8.961	8.955	9.096	8.899	8.890	8.860
HMGB1P35	6.166	6.394	6.396	6.136	6.194	6.205
AMMECR1	9.974	9.965	9.862	9.533	9.803	9.903
SLC25A10	9.028	8.985	9.386	8.953	8.949	8.957
EMD	9.328	9.675	9.889	9.744	9.471	9.365
EMB	10.329	10.293	10.375	10.062	10.059	10.086
MTND5P11	13.359	13.463	13.397	13.398	13.334	13.231
AC012368.1	7.232	7.342	7.101	7.280	7.293	7.310
ZNF826P	5.996	6.222	5.997	6.075	6.330	6.148
NUFIP1P1	8.079	8.069	8.209	8.190	8.453	7.961
AC009487.2	6.638	6.997	6.805	6.622	6.897	6.989
AFF4	9.527	9.561	9.483	9.229	9.313	9.299
AFF3	6.931	6.917	6.729	6.991	6.757	6.914
AFF2	7.155	7.271	6.969	7.032	7.389	7.134
PDPN	7.040	7.155	7.007	7.121	7.035	7.172
PDPR	10.074	9.925	9.956	9.553	9.743	9.753

ROR1-AS1	4.162	4.187	4.143	4.069	4.125	4.187
AC009506.2	6.433	6.426	6.409	6.445	6.496	6.491
STK40	8.794	8.695	8.830	8.670	8.831	8.779
UBA6	9.254	9.257	9.204	9.080	9.095	9.211
PDP1	8.908	9.071	8.881	8.671	8.838	8.813
PDP2	8.019	8.052	7.972	7.923	7.784	8.080
UBA5	10.056	10.180	10.059	9.895	9.890	10.039
UBA2	10.929	11.039	11.181	10.944	10.887	11.004
UBA3	8.870	8.807	8.804	8.757	8.656	8.714
UBA1	12.056	12.078	12.204	11.897	11.846	11.954
PHF24	7.132	7.538	7.301	7.450	7.377	7.401
PWP1	11.732	11.767	11.820	11.749	11.832	11.499
PHF20	9.372	9.217	9.338	9.106	9.414	8.932
PHF23	9.761	9.720	9.822	9.761	9.722	9.563
PSMB4	11.832	11.900	12.100	11.814	11.700	11.853
TAGAP	7.963	8.132	8.152	7.981	8.172	8.221
LINC02535	7.035	6.776	6.937	6.871	6.645	6.642
RPAIN	8.863	8.875	9.072	8.904	8.876	8.772
LINC02533	5.623	5.383	5.209	5.395	5.519	5.498
LINC02532	8.437	8.413	8.589	8.519	8.376	8.301
AC015819.3	3.887	3.813	3.849	3.919	4.116	3.951
RPRML	6.224	6.132	6.134	6.272	6.128	6.188
AL160153.1	4.050	3.981	3.947	4.004	4.139	4.050
CPXM1	6.491	6.731	6.845	6.832	7.018	6.789
PNO1	9.897	9.907	10.139	10.013	10.173	10.012
CPXM2	8.265	8.285	8.222	8.280	8.231	8.332
PENK	6.573	6.465	6.231	6.583	6.322	6.142
ZIM2	7.820	7.817	7.555	8.107	7.755	7.926
ZIM3	4.474	4.540	4.400	4.424	4.565	4.668
DTNB-AS1	4.969	4.913	4.857	5.037	5.211	4.936
GPR65	5.968	5.871	5.985	6.078	6.001	5.838
GPR62	8.057	8.172	8.308	8.245	8.341	8.229
GPR63	5.804	5.924	6.305	6.755	6.235	6.386
GPR61	6.374	6.555	6.615	6.410	6.409	6.621
PNOC	7.088	7.105	6.965	7.112	7.027	7.081
INE1	7.798	7.954	8.007	8.124	7.985	8.210
GPR68	7.911	7.774	7.846	7.903	7.755	7.955
AC012100.3	6.091	5.766	5.670	5.900	5.993	5.773
AL133318.1	8.880	9.154	9.428	9.179	9.418	8.737
RPL6P27	13.671	13.713	13.720	13.661	13.586	13.672
AC009562.1	7.044	6.867	6.670	6.959	6.964	6.951
GNS	10.842	10.786	10.796	10.384	10.421	10.568
AC008622.2	9.144	8.982	9.399	8.927	8.966	8.945
INSM2	7.249	6.953	6.979	7.319	7.293	7.362
JARID2	8.848	8.858	8.754	8.578	8.675	8.727
GNE	9.433	9.411	9.447	9.088	9.150	9.258
AL359258.1	4.272	4.199	4.364	4.408	4.339	4.313
AL359258.3	8.009	7.993	8.037	7.969	8.005	8.026
AL359258.2	8.009	7.993	8.037	7.969	8.005	8.026
AC255461.2	4.957	5.051	4.936	4.868	4.936	5.007
TMEM37	9.217	9.247	9.081	9.018	9.199	9.208
TMEM31	6.433	6.287	6.215	6.583	6.479	6.475
TMEM33	9.689	9.791	9.643	9.667	9.500	9.580
RMI2	10.775	10.780	10.924	10.500	10.492	10.657
NT5C1A	7.501	7.608	7.648	7.402	7.720	7.785
CPEB2-DT	5.129	5.158	5.347	5.413	5.221	5.220

OR14J1	7.193	7.490	7.673	7.245	7.376	7.343
SCD5	9.556	9.524	9.782	9.419	9.549	9.468
POGK	10.466	10.475	10.532	10.376	10.426	10.429
SDS	7.519	7.378	7.346	7.370	7.330	7.497
ANO10	9.358	9.379	9.394	9.374	9.398	9.306
POGZ	10.172	10.235	10.165	10.102	9.975	10.145
AC012354.6	6.427	6.497	6.457	6.360	6.573	6.393
AC012354.4	8.631	8.739	8.589	8.681	8.825	8.706
PRUNE2	6.223	6.313	6.239	6.490	6.516	6.236
PRUNE1	9.154	9.347	9.373	8.978	9.078	9.144
AC012354.9	6.427	6.497	6.457	6.360	6.573	6.393
APCDD1L	7.955	7.959	7.872	8.134	8.038	8.058
NRCAM	7.427	7.464	7.423	7.410	7.518	7.462
PNLIP	6.520	6.464	6.358	6.603	6.568	6.638
AC242842.2	7.865	7.879	8.165	7.721	8.167	7.693
AC242842.3	11.550	11.483	11.377	11.343	11.231	11.508
AC242842.1	7.865	7.879	8.165	7.721	8.167	7.814
CXorf58	6.234	6.312	6.115	6.198	6.361	6.207
CXorf56	7.669	7.568	7.621	7.553	7.661	7.560
LYZ	11.552	11.618	11.740	11.751	11.973	11.753
LYN	9.320	9.446	9.637	9.348	9.650	9.196
PRH2	7.199	7.315	7.449	7.834	7.457	7.479
PRH1	7.199	7.315	7.449	7.834	7.457	7.479
LY9	6.926	6.867	7.025	7.268	7.413	7.208
IP6K3	5.944	5.672	5.777	6.453	6.159	6.027
IP6K2	9.899	9.910	9.981	9.614	9.761	9.889
IP6K1	9.093	9.165	9.156	9.103	9.123	9.120
AC008498.2	5.786	5.953	5.852	5.909	5.843	5.987
MADD-AS1	4.571	4.649	4.859	4.709	4.745	4.814
MYO15B	8.134	8.196	8.232	8.090	8.184	8.347
MYO15A	7.030	7.227	7.084	7.179	7.096	7.340
AL121796.1	7.980	8.050	7.924	8.007	7.669	7.964
KATNBL1	6.101	6.155	6.116	6.029	6.196	6.228
NFKBIB	8.891	9.059	9.140	8.943	8.970	9.043
NFKBIA	9.816	9.814	9.920	11.671	11.559	11.516
NFKBID	8.310	8.504	8.264	8.286	8.296	8.497
NFKBIE	8.497	8.448	8.571	8.715	8.660	8.648
NFKBIZ	10.634	10.515	10.688	10.954	10.897	10.533
PLAC1	7.768	8.089	7.773	7.765	7.801	7.918
GOLGA8DP	6.446	6.294	6.356	6.148	6.123	6.590
TP53RK	9.415	9.454	9.541	9.509	9.566	9.478
PLAC4	9.471	9.501	9.218	9.326	9.529	9.527
AL096829.1	11.046	11.161	11.341	11.209	11.057	11.137
PLAC9	8.133	8.267	8.226	8.382	8.380	8.328
PLAC8	9.113	9.066	9.107	8.689	8.851	8.732
PRSS33	8.746	8.840	8.891	8.793	8.820	8.856
PRSS37	7.425	7.386	7.398	7.439	7.563	7.803
NR5A2	7.265	6.979	7.097	6.928	7.007	7.148
NR5A1	6.262	6.495	6.648	6.657	6.614	6.716
DLX4	7.120	7.132	6.897	6.717	6.831	7.039
DLX5	6.305	6.349	6.280	6.384	6.383	6.182
DLX6	7.217	6.930	6.971	7.280	6.834	7.062
DLX1	7.779	7.831	7.689	7.668	7.767	7.736
DLX2	7.863	7.632	7.168	8.285	7.962	7.883
DLX3	7.477	7.381	7.357	7.758	7.684	7.498
NACA	13.769	13.810	13.830	13.734	13.690	13.742

SMIM34B	8.540	8.492	8.438	8.629	8.751	8.728
LINP1	9.473	9.451	9.572	9.291	9.202	9.200
ACTB	14.766	14.803	14.756	14.737	14.719	14.751
VAV1	6.497	6.467	6.477	6.279	6.348	6.401
VAV3	9.469	9.433	9.480	9.285	9.226	9.412
VAV2	9.794	9.759	9.763	9.895	9.793	9.632
NACA4P	12.273	12.283	12.329	12.167	12.190	12.294
AL929602.1	4.376	4.773	4.668	4.763	4.535	4.427
FOXN4	7.982	8.138	7.967	8.067	8.080	8.142
STARD13	7.331	7.252	7.433	7.428	7.317	7.348
TP63	7.653	7.450	7.298	7.908	7.724	7.831
NR6A1	8.600	8.728	8.717	8.669	8.625	8.776
MARS1	11.094	11.117	11.223	11.055	11.052	10.956
MARS2	8.737	8.716	8.671	8.651	8.613	8.661
SMAD5-AS1	7.144	7.229	6.997	7.286	7.452	7.378
RNU6-322P	5.294	5.670	5.724	5.580	5.593	5.702
MIR4435-2HG	11.086	11.192	11.341	11.191	11.247	11.349
PYGO1	6.340	5.914	6.224	6.522	6.484	6.376
PYGO2	9.578	9.455	9.468	9.354	9.446	9.448
AL590143.1	4.098	4.130	4.250	3.870	4.014	3.938
CRHBP	6.172	6.119	5.973	6.252	6.061	6.182
FERMT2	9.305	9.356	9.471	9.208	9.164	9.197
FERMT3	7.759	7.695	7.811	7.820	7.834	7.909
RPL3P4	13.670	13.651	13.720	13.644	13.478	13.579
CRNKL1	6.756	7.097	6.954	6.634	6.800	6.949
AC068397.2	6.637	6.650	6.916	7.201	6.974	6.850
HCG18	9.211	9.261	9.340	9.042	9.201	9.174
CCM2L	7.276	7.366	7.253	7.483	7.448	7.453
DPM3	8.934	9.021	9.238	8.990	9.110	9.158
SYNGR3	6.917	6.733	6.858	6.762	6.699	6.699
SYNGR2	11.942	12.044	12.178	11.917	11.893	11.939
WDR97	6.190	5.997	6.182	6.014	6.370	6.332
HCG11	7.179	7.388	7.323	6.898	7.075	6.981
PRPH2	7.068	7.408	7.064	7.093	7.410	7.365
AL713998.1	6.603	6.739	6.577	6.804	6.561	6.668
AL592078.2	6.843	6.696	6.672	6.784	6.878	6.940
C9orf64	9.521	9.540	9.424	9.137	9.264	9.280
USP6NL	7.680	7.737	8.004	7.894	7.680	7.818
AACSP1	5.166	5.331	5.440	5.593	5.311	5.363
KLHDC8A	8.272	8.506	8.219	8.368	8.351	8.440
USP12	8.323	8.476	8.458	8.334	8.212	8.389
USP13	10.823	10.924	11.022	10.473	10.764	10.507
USP10	10.616	10.685	10.866	10.824	10.702	10.511
USP11	11.258	11.213	11.295	10.976	11.126	11.025
USP16	9.415	9.473	9.523	9.263	9.295	9.421
USP14	11.515	11.563	11.543	11.272	11.370	11.328
USP15	8.884	9.070	8.831	9.008	8.813	9.158
USP18	9.620	9.633	9.591	9.534	9.791	9.460
USP19	8.630	8.494	8.485	8.581	8.500	8.527
AC023301.1	5.614	5.660	5.706	5.793	5.803	6.004
INPP1	10.460	10.516	10.637	10.515	10.450	10.529
RPL23AP45	7.667	7.929	8.164	7.639	7.672	7.716
RPL23AP42	14.341	14.360	14.394	14.370	14.332	14.374
RPL23AP49	7.667	7.929	8.164	7.639	7.672	7.716
SIRPAP1	9.145	8.986	9.164	8.719	9.083	8.897
TAP1	9.362	9.414	9.458	9.383	9.503	9.325

CRKL	10.758	10.769	10.913	10.593	10.515	10.697
FAM86FP	9.708	9.675	9.729	9.656	9.678	9.650
TAP2	10.208	10.197	10.137	10.032	10.040	9.957
PSME2P2	11.833	11.858	11.931	11.903	11.876	11.874
AC007495.1	4.981	4.959	4.883	5.107	4.930	4.937
UBAC1	8.666	8.798	8.792	8.516	8.566	8.593
AC246793.1	5.461	5.418	5.406	5.359	5.455	5.416
CYMP-AS1	8.124	8.266	8.200	8.036	8.271	8.272
AL161909.1	13.598	13.601	13.717	13.679	13.480	13.759
SUPT5H	10.324	10.404	10.550	10.245	10.126	10.216
RETN	6.463	6.400	6.284	6.339	6.310	6.240
RPTN	5.807	5.621	5.213	5.760	5.823	5.652
SLC35E2B	10.590	10.532	10.665	10.204	10.150	10.351
SLC35E2A	10.590	10.532	10.665	10.204	10.150	10.351
AL035078.1	6.801	6.715	6.600	6.814	6.827	6.834
FXR1	12.030	12.100	12.247	12.068	11.902	11.983
FXR2	9.379	9.411	9.581	9.538	9.551	9.450
CYP4X1	4.437	4.235	4.491	4.547	4.420	4.337
AC126474.2	7.867	7.921	7.941	7.604	7.596	7.756
AC126474.1	6.162	6.290	6.198	6.132	6.257	6.308
RNASEH1-AS1	7.568	7.640	7.707	7.410	7.748	7.599
RHBDD2	10.414	10.419	10.503	10.118	10.102	10.157
RHBDD3	8.831	9.022	9.138	8.522	8.631	8.662
RHBDD1	8.692	8.761	8.663	8.522	8.467	8.381
KLC1	8.859	8.964	9.006	8.645	8.433	8.454
KLC2	9.260	9.367	9.327	9.080	9.155	9.206
KLC3	10.196	10.076	10.076	10.288	10.469	10.173
KLC4	8.755	8.907	8.921	8.793	8.689	8.784
KRT18P38	7.631	7.847	7.781	7.694	7.993	7.792
AL035411.1	11.071	11.101	11.237	11.072	11.041	11.124
CHCHD2	13.337	13.441	13.505	13.444	13.451	13.475
CHCHD3	14.111	14.146	14.160	14.135	14.090	14.165
CHCHD1	9.135	9.054	9.360	9.104	8.997	9.044
CHCHD6	8.224	8.401	8.551	8.170	8.258	8.304
CHCHD7	9.111	9.057	9.117	8.786	8.616	8.761
CHCHD4	8.957	8.905	8.994	8.513	8.695	8.652
CHCHD5	8.623	8.597	8.886	8.584	8.629	8.643
AP002518.1	5.835	5.788	5.734	5.647	5.906	5.854
OVCA2	10.288	10.311	10.506	10.323	10.279	10.298
TMTC4	8.120	8.087	7.905	7.858	7.803	7.937
ZNF512	9.172	9.384	9.253	9.198	9.268	9.252
TMTC3	9.395	9.410	9.289	9.203	9.260	9.297
ZNF510	7.582	7.573	7.689	7.825	7.697	7.911
ZNF511	8.643	8.555	8.689	8.622	8.706	8.579
ZNF516	7.360	7.298	7.319	7.468	7.234	7.388
HNMT	10.603	10.712	10.803	10.676	10.660	10.781
ZNF514	7.526	7.537	7.532	6.887	7.017	7.499
TMTC2	10.137	10.276	10.290	10.213	10.443	10.274
TSPYL4	8.639	8.643	8.595	8.323	8.445	8.458
TSPYL5	4.894	4.757	4.715	4.701	4.898	4.832
TSPYL6	4.378	4.372	4.216	4.614	4.209	4.383
AC091153.3	5.254	4.996	5.309	5.243	5.182	5.275
AC139491.7	6.574	6.430	6.216	6.413	6.536	6.651
TSPYL2	9.904	9.804	9.878	9.939	9.948	10.002
LINC01424	4.525	4.653	4.690	4.669	4.683	4.655
LINC01425	6.792	6.685	6.592	6.761	6.788	6.780

LINC01426	4.380	4.281	4.366	4.416	4.308	4.246
AC138869.3	3.826	3.885	3.772	3.930	3.832	3.885
LINC01428	6.985	6.953	7.208	7.119	7.317	7.189
XIRP1	8.083	8.244	8.042	8.232	8.048	8.301
XIRP2	4.717	4.659	4.529	4.577	4.713	4.739
AC241585.2	10.040	10.032	10.324	9.749	9.958	9.735
AC022075.1	7.732	7.748	7.948	8.008	7.874	7.860
VWF	6.975	6.963	6.999	7.150	6.985	6.976
CDH11	7.512	7.761	7.741	7.553	7.759	7.835
KIAA0355	8.613	8.508	8.753	8.241	8.336	8.448
ZNF22	8.891	8.777	8.952	8.843	8.958	8.898
ZFY-AS1	6.303	6.185	5.936	6.018	6.128	6.181
CDH17	7.508	7.409	7.492	7.353	7.385	7.523
AP002336.5	8.509	8.581	8.686	8.512	8.548	8.479
AC010207.1	6.270	6.507	6.457	6.296	6.216	6.498
LINC02731	5.528	5.665	5.581	5.774	5.887	5.890
TESMIN	6.698	6.557	6.822	6.660	6.679	6.638
AC068338.2	7.661	7.740	7.777	7.777	7.766	7.638
LINC01281	7.419	7.509	7.651	7.756	7.457	7.662
LINC01282	5.985	6.257	6.350	6.441	6.389	6.441
LINC01289	5.740	5.570	5.696	5.840	5.859	5.756
AC013726.3	5.767	5.658	5.555	5.785	5.589	5.294
AC013726.2	5.767	5.658	5.555	5.785	5.589	5.294
ARHGAP39	8.304	8.224	8.286	8.256	8.163	8.156
NTN1	6.705	6.817	6.927	6.911	6.711	6.581
NTN3	6.383	6.581	6.716	6.710	6.712	6.668
NTN4	9.551	9.557	9.441	9.371	9.406	9.532
NTN5	7.059	6.912	6.908	6.916	6.938	7.067
SI	3.910	3.666	3.780	3.840	4.026	3.809
IFNA21	6.704	6.678	6.687	6.650	6.818	6.698
ZBED5-AS1	10.407	10.405	10.475	10.259	10.198	10.241
B4GALNT4	6.110	5.735	6.194	6.207	6.062	6.361
B4GALNT1	7.949	7.951	7.940	7.731	8.008	7.926
B4GALNT2	6.715	6.877	6.748	6.691	6.728	6.656
B4GALNT3	8.148	8.179	8.319	8.466	8.305	8.217
MIR3654	14.111	14.146	14.160	14.135	14.090	14.165
LINC00488	4.588	4.464	4.506	4.517	4.615	4.722
MIR3650	7.595	7.527	7.665	7.397	7.727	7.487
NDE1	9.811	9.801	9.823	9.330	9.673	9.665
LINC00487	4.152	4.234	4.238	4.332	4.437	4.202
LINC00486	4.725	4.721	4.727	4.759	4.780	4.833
LINC00482	7.679	7.626	7.902	7.921	7.828	7.727
AC244517.2	4.795	4.750	4.735	4.977	5.042	5.139
NUDC	10.917	11.003	11.124	10.864	10.682	10.882
DEFA6	7.816	7.747	7.802	7.851	7.981	7.981
DEFA5	7.105	7.101	7.025	7.239	7.263	7.402
DEFA4	7.221	7.123	7.269	7.436	7.316	7.251
DEFA3	6.338	6.348	6.335	6.003	6.148	6.540
DEFA1	6.338	6.348	6.335	6.003	6.148	6.540
NUMBL	9.810	9.783	9.723	9.341	9.567	9.489
NUP210P1	6.434	6.724	6.688	6.682	6.606	6.771
VAC14-AS1	4.123	4.129	3.931	4.155	4.162	4.284
SEPSECS-AS1	4.678	4.510	4.663	4.733	4.573	4.552
NRP1	9.752	9.780	9.819	9.636	9.624	9.646
NRP2	8.366	8.516	8.349	8.576	8.480	8.482
AF131216.4	4.739	4.553	4.759	4.748	4.673	4.634

C12orf60	5.638	5.742	5.592	5.809	5.912	5.884
C12orf66	8.290	8.387	8.368	8.004	8.308	8.223
AF131216.3	8.614	8.403	8.312	8.684	8.728	8.558
DNAJB8-AS1	5.509	5.627	5.805	5.727	5.644	5.747
SPHKAP	6.389	6.304	6.206	6.586	6.495	6.342
AC234775.3	7.214	7.410	7.278	7.423	7.374	7.389
MAGED4B	7.555	7.615	7.676	7.555	7.742	7.929
MACROD2	10.899	10.873	10.967	10.708	10.464	10.742
GSTA4	7.646	7.709	7.544	7.758	7.629	7.631
GABPB1-AS1	8.601	8.584	8.413	8.201	8.448	8.330
GSTA1	4.922	5.051	4.758	4.963	5.025	4.880
GSTA3	5.685	5.773	5.500	5.990	5.879	6.068
DYNLT1	11.217	11.238	11.403	11.465	11.402	11.381
DYNLT3	11.360	11.301	11.472	11.291	11.073	11.221
STRAP	11.905	12.092	12.181	12.146	11.973	12.050
UBL4B	8.927	8.918	8.825	8.917	9.009	9.023
C1orf87	5.847	5.561	5.416	6.013	5.689	5.716
RNF208	9.142	9.072	9.287	9.282	9.388	9.224
RNF207	8.617	8.542	8.490	8.371	8.285	8.497
AC016597.2	5.203	5.295	5.096	4.992	5.326	5.330
AC015969.1	4.324	4.407	4.250	4.438	4.386	4.391
STRA6	8.065	8.084	8.411	8.471	8.327	8.197
HGC6.3	7.333	7.443	7.533	7.905	7.719	7.534
SIGLEC16	6.452	6.331	6.420	6.621	6.595	6.659
SIGLEC15	8.282	8.314	8.248	7.955	8.313	8.098
SIGLEC10	6.873	6.970	6.969	7.128	7.153	7.238
SIGLEC11	6.395	6.548	6.398	6.554	6.580	6.591
KIFC2	9.661	9.712	9.772	9.403	9.455	9.721
KIFC3	8.295	8.290	8.654	8.512	8.229	8.329
GPR176-DT	5.955	5.768	5.782	5.919	5.964	5.931
AC016727.1	5.214	5.273	5.054	5.299	5.186	5.414
NLGN1	4.375	4.663	4.454	4.391	4.385	4.422
SYNPO2	7.940	7.748	7.773	8.020	8.092	7.934
MIR6777	10.301	10.384	10.361	9.952	9.843	9.919
DMRT1	4.846	4.689	4.691	4.795	4.809	4.505
DMRT2	6.035	6.148	6.249	6.002	6.164	6.141
DMRT3	4.967	5.014	4.869	4.960	4.950	4.918
PROS1	6.235	6.140	6.359	6.098	6.222	5.991
CRADD	8.628	8.711	8.800	8.696	8.634	8.760
NCKAP5L	8.139	8.231	8.216	8.117	8.186	8.114
PLA2G12B	7.457	7.365	7.500	7.536	7.678	7.629
PLA2G12A	10.193	10.193	10.245	10.181	10.182	10.196
AC097375.3	5.766	5.678	5.225	5.765	5.746	5.437
HSBP1L1	7.686	7.494	7.702	7.353	7.062	7.435
LYRM2	9.572	9.628	9.612	9.659	9.534	9.701
IQCA1	7.169	7.282	7.283	7.309	7.555	7.373
MRPS31P5	6.981	6.869	7.047	6.961	6.911	6.714
TNKS2	9.333	9.433	9.385	9.235	9.319	9.186
LYRM1	9.397	9.540	9.666	9.662	9.662	9.615
AC087721.2	10.540	10.438	10.613	9.838	10.070	9.986
U73166.1	7.706	7.630	7.628	7.476	7.663	7.806
EPOR	11.299	11.232	11.219	11.366	11.604	11.507
EPOP	9.306	9.192	9.411	9.488	9.308	9.332
HS6ST1	9.840	9.970	10.016	9.808	9.836	9.923
HS6ST2	10.537	10.735	10.682	10.438	10.376	10.462
HS6ST3	6.487	6.490	6.513	6.733	6.725	6.546

TBILA	6.155	6.101	5.755	5.676	5.870	5.956
SNORA4	8.631	8.462	8.315	8.343	7.903	8.597
AC117489.1	6.867	6.766	6.948	7.082	6.981	7.173
TCERG1	9.668	9.618	9.641	9.370	9.232	9.624
CNBP	11.990	12.168	12.208	12.133	11.998	12.139
INTS13	9.778	9.919	9.926	9.757	9.668	9.815
INTS12	7.836	7.743	7.976	7.621	7.597	7.823
INTS11	9.760	9.808	9.900	9.449	9.429	9.589
INTS10	9.193	9.336	9.472	9.250	9.254	9.321
INTS14	10.474	10.410	10.568	10.383	10.383	10.384
KPNA2P3	11.882	11.907	11.917	11.581	11.775	11.682
TXN2	10.488	10.550	10.738	10.471	10.776	10.317
AGAP1	10.627	10.640	10.680	10.629	10.553	10.341
AGAP2	8.264	8.238	8.377	8.541	8.348	8.438
AGAP3	8.906	9.072	9.117	8.874	8.900	8.850
AGAP4	9.081	9.018	9.114	9.210	9.131	9.065
AGAP5	9.081	9.018	9.114	9.210	9.131	9.065
AGAP6	9.081	9.018	9.114	9.210	9.131	9.065
FXNP2	6.265	5.934	5.972	5.837	5.743	6.033
AGAP9	9.081	9.018	9.114	9.210	9.131	9.065
AC009879.3	5.600	5.684	5.378	5.800	5.467	5.674
AC009879.2	9.489	9.466	9.527	9.461	9.237	9.372
GDPD2	8.554	8.733	8.630	8.558	8.705	8.557
GDPD3	7.923	7.655	7.860	7.823	7.831	7.849
SLC23A3	8.135	8.024	7.957	8.176	8.121	8.191
GDPD1	7.025	6.774	6.767	7.094	7.114	7.138
EXTL3-AS1	5.345	5.386	5.346	5.536	5.841	5.365
GDPD5	8.602	8.746	8.799	8.575	8.623	8.620
OSGEPL1	8.223	8.123	8.029	8.032	8.049	8.212
GBP1P1	10.306	10.279	10.378	10.496	10.842	10.100
GBAP1	9.177	9.195	9.333	9.033	9.013	9.060
YWHAQ	12.583	12.753	12.831	12.645	12.647	12.735
YWHAH	10.888	11.015	11.050	10.819	10.706	10.664
AC090740.1	5.992	6.008	5.912	5.937	5.927	6.103
CNTNAP2-AS1	4.883	5.007	5.125	4.714	4.865	4.892
YWHAB	13.527	13.629	13.660	13.521	13.598	13.485
FCRL1	5.854	5.684	5.358	5.526	5.875	5.933
FCRL2	6.951	7.005	7.162	7.014	7.143	7.046
FCRL3	7.220	7.327	7.337	7.246	7.311	7.308
FCRL4	5.250	5.380	5.255	5.181	5.149	5.499
FCRL5	7.470	7.493	7.495	7.556	7.486	7.663
YWHAE	11.711	11.724	12.009	12.230	12.408	12.200
FCRLA	7.599	7.469	7.264	7.602	7.649	7.791
FCRLB	8.317	8.276	8.318	8.319	8.210	8.237
AL078601.3	5.382	5.300	5.416	5.353	5.461	5.374
CKAP2	9.416	9.337	9.364	9.023	9.139	9.086
MIR22HG	8.471	8.409	8.500	8.369	8.251	8.497
PDCD1LG2	7.427	7.621	7.478	7.693	7.649	7.630
CHST11	6.772	6.937	6.741	7.242	7.061	7.200
CHST10	9.163	9.267	9.399	9.175	9.196	9.184
CHST13	6.163	6.174	6.096	6.089	6.191	6.116
CHST12	9.305	9.160	9.467	9.056	8.974	9.129
CHST15	8.376	8.240	8.236	8.339	8.231	8.441
CHST14	9.196	9.325	9.323	9.150	9.284	9.221
ROCK1	10.599	10.495	10.565	10.515	10.284	10.438
AC017116.2	8.264	8.087	7.851	7.974	8.050	8.043

IGSF11-AS1	4.064	3.931	4.067	4.332	4.225	3.966
AGER	7.512	7.666	7.644	7.863	7.780	7.804
AL138955.1	4.063	4.161	4.074	4.161	4.395	4.540
LRRC4	7.412	7.533	7.575	7.710	7.527	7.784
WNK3	6.934	6.948	7.089	7.120	7.213	7.224
LRRC6	6.894	7.037	6.925	7.154	7.080	7.168
LRRC7	4.237	4.282	4.374	4.444	4.362	4.378
LRRC1	8.547	8.633	8.608	8.530	8.435	8.514
DRAIC	7.376	7.235	7.201	7.327	7.466	7.616
LRRC3	7.175	7.278	7.454	7.259	7.349	7.364
HAND2-AS1	5.682	6.161	5.929	5.990	6.079	6.012
RFNG	8.667	8.717	8.898	8.556	8.659	8.582
MORN5	6.798	6.715	6.842	6.935	6.818	6.702
MORN4	8.202	8.136	8.083	8.165	8.037	8.266
PLD6	7.904	7.888	8.007	7.663	7.663	7.806
MORN1	8.345	8.297	8.529	8.596	8.630	8.578
PLD1	9.074	9.001	9.045	8.886	8.935	8.945
MORN3	7.859	7.859	7.888	7.593	7.661	7.753
MORN2	8.699	8.823	9.010	8.953	8.971	8.932
ST20	8.626	8.639	8.662	8.691	8.774	8.847
TREML3P	6.436	6.584	6.320	6.482	6.500	6.598
RRP1	9.141	9.099	9.366	9.075	8.991	8.985
DOCK8	6.295	6.346	6.146	6.515	6.761	6.631
DOCK9	7.619	7.533	7.667	7.494	7.547	7.522
GRIA2	5.528	5.466	5.720	5.456	5.639	5.670
GRIA3	6.679	6.804	6.933	6.829	6.750	6.823
GRIA1	5.731	5.389	5.662	5.855	5.849	5.847
DOCK6	9.010	9.156	9.156	9.045	9.059	9.154
DOCK7	9.375	9.420	9.333	9.068	9.014	9.383
DOCK4	5.913	6.058	6.040	6.183	6.026	6.158
DOCK5	9.282	9.081	9.244	8.872	8.862	9.019
ACSBG1	6.786	6.535	6.561	6.729	6.566	6.592
ACSBG2	7.229	7.111	7.226	7.182	7.295	7.300
HOTAIRM1	5.042	5.186	5.169	5.001	5.403	5.070
TNFRSF12A	12.451	12.499	12.650	12.505	12.463	12.373
AC008708.2	5.848	6.031	5.970	6.025	6.172	6.031
C8orf49	3.810	3.973	4.077	4.104	3.925	4.224
C8orf48	4.362	4.178	4.137	4.506	4.460	4.408
SCNN1G	4.241	4.453	4.392	4.515	4.390	4.353
CTBP1-DT	8.362	8.490	8.709	8.229	8.416	8.397
ARHGAP22-IT1	7.628	7.687	7.423	7.605	7.685	7.770
SCNN1D	6.647	6.513	6.643	6.837	6.831	6.687
SCNN1B	6.425	6.728	6.867	6.898	6.756	6.674
SCNN1A	10.780	10.880	10.965	10.719	10.676	10.723
FAM98C	10.043	10.247	10.499	9.977	10.096	9.945
FAM98B	9.019	8.901	9.104	8.884	8.893	8.637
FAM98A	9.807	9.857	9.794	9.578	9.423	9.778
RNF11P2	4.710	4.605	4.667	4.654	4.674	4.569
SCUBE2	6.337	6.551	6.324	6.453	6.473	6.536
SCUBE3	8.026	7.987	7.832	8.010	8.207	8.174
SCUBE1	7.499	7.587	7.659	7.769	7.845	7.873
RPSAP54	13.432	13.517	13.661	13.465	13.519	13.574
ZNF750	3.954	4.042	3.751	3.698	3.778	3.791
RPSAP58	13.432	13.517	13.661	13.465	13.519	13.574
SMIM13	8.649	8.682	8.692	8.617	8.582	8.598
SMIM12	10.134	10.217	10.271	10.093	10.057	10.053

COX10-AS1	7.443	7.377	7.143	7.291	7.267	7.563
SMIM10	6.081	6.223	6.242	5.779	6.009	5.874
SMIM17	3.959	4.087	4.366	4.210	4.098	4.178
SMIM15	10.946	11.030	11.061	10.896	10.886	10.834
SMIM14	9.141	9.030	9.133	8.856	9.214	9.184
SMIM19	10.631	10.627	10.800	10.507	10.588	10.496
CHIAP2	4.165	4.023	4.109	4.265	4.391	4.491
BCORL1	7.960	7.836	8.030	7.834	8.165	7.916
HACE1	7.304	7.426	7.634	7.029	7.182	7.239
AC015936.1	6.961	7.173	7.052	7.147	6.914	7.093
CU633906.7	5.722	5.682	5.825	5.850	5.767	5.793
CU633906.4	7.829	7.863	7.877	7.856	7.805	7.700
CU633906.2	7.923	7.782	7.902	7.606	7.461	7.712
AC083849.5	4.332	4.346	4.365	4.407	4.401	4.389
NT5DC4	6.864	6.985	7.144	7.086	6.715	6.852
NT5DC2	9.831	9.896	10.047	9.772	9.687	9.765
NT5DC3	8.803	8.660	8.798	8.601	8.674	8.660
NT5DC1	9.721	9.744	9.699	9.294	9.476	9.210
RUSC2	8.387	8.418	8.293	8.301	8.133	8.403
AC073539.6	7.272	7.378	7.356	7.323	7.572	7.462
RUSC1	10.404	10.266	10.303	10.181	10.126	10.285
AC073539.1	7.272	7.378	7.356	7.323	7.572	7.462
FOXD1-AS1	6.748	6.675	6.883	7.053	6.891	6.802
PPIG	10.321	10.204	10.318	10.220	10.472	9.794
PPIF	9.945	9.954	10.171	10.030	9.966	9.910
PPIE	9.062	9.119	9.357	8.990	9.033	9.062
PPID	10.415	10.514	10.559	10.391	10.473	10.681
PPIC	10.605	10.554	10.665	10.657	10.535	10.571
PPIB	12.754	12.877	13.018	12.958	12.982	12.835
PPIA	14.423	14.465	14.415	14.484	14.467	14.475
IGLL3P	6.390	6.066	6.649	6.405	6.522	6.479
DCPS	9.351	9.267	9.403	8.949	8.876	9.025
MAP4K3-DT	7.217	7.169	7.277	6.929	7.377	6.864
PTHLH	5.266	5.282	5.345	5.417	5.316	5.348
THEMIS2	7.334	7.460	7.649	7.144	7.467	7.459
LLPH	9.685	9.677	9.754	9.586	9.624	9.645
DHCR24-DT	5.860	5.803	5.820	5.847	6.043	5.926
DCP2	9.419	9.380	9.299	9.211	9.082	9.361
AC107068.1	6.628	6.501	6.793	6.634	6.457	6.923
AP001425.1	4.507	4.609	4.513	4.486	4.512	4.483
LZIC	8.154	8.149	8.296	8.123	8.047	8.090
NAA50	10.239	10.285	10.351	10.037	9.804	10.138
CFDP1	10.612	10.678	10.803	10.538	10.411	10.494
AL356108.1	5.796	5.989	5.984	5.795	5.900	6.116
ATF6B	8.923	8.954	9.085	9.221	9.069	8.862
TDP1	8.782	8.773	8.810	8.491	8.468	8.525
PDE7A	7.279	7.318	7.176	7.077	7.274	7.213
TPPP3	7.693	7.671	7.661	7.661	7.638	7.631
PDE7B	6.822	6.921	6.930	6.933	6.874	6.984
TDP2	9.956	9.960	9.983	9.856	9.856	9.867
PIP	8.098	8.314	8.167	8.128	8.339	8.311
FAM53B-AS1	6.799	6.945	6.953	7.170	7.407	7.330
PI3	8.237	8.323	8.159	8.060	8.257	8.283
ANKRD52	8.683	8.793	8.889	8.674	8.852	8.690
ANKRD53	9.063	9.231	9.035	9.293	9.252	9.101
ANKRD50	8.557	8.515	8.333	8.128	7.877	8.090

ANKRD54	9.016	9.126	9.086	8.927	9.014	8.958
ANKRD55	6.940	7.069	6.797	6.882	6.951	6.966
AC009088.2	7.472	7.653	7.486	7.147	7.587	7.637
TMEM238L	8.076	8.141	8.045	8.163	8.307	8.180
KBTBD7	7.266	7.505	7.422	6.979	7.153	7.069
AMH	6.561	6.451	6.474	6.561	6.514	6.617
KBTBD4	8.782	9.040	8.872	8.625	8.487	8.649
KBTBD3	5.369	5.500	5.355	5.644	5.728	5.633
KBTBD2	9.623	9.820	9.747	9.609	9.660	9.578
AMN	8.425	8.385	8.663	8.826	8.611	8.573
AC015660.4	5.803	5.821	5.919	5.898	6.033	5.903
KBTBD8	5.475	5.753	5.761	5.673	5.565	5.623
AMT	7.582	7.683	7.604	7.665	7.707	7.723
RASL11B	6.905	6.858	6.815	7.008	6.956	6.986
RASL11A	7.117	7.143	7.272	7.289	7.269	7.278
LRIF1	9.619	9.572	9.630	9.722	9.710	9.790
BICRA	8.713	8.654	8.786	8.627	8.554	8.582
GLRB	8.459	8.494	8.426	8.123	8.350	8.408
AL603750.1	7.381	7.395	7.345	7.169	7.168	7.304
DUX4L15	8.052	7.820	8.258	8.487	8.319	8.298
DUX4L14	8.052	7.820	8.258	8.487	8.319	8.298
DUX4L13	8.052	7.820	8.258	8.487	8.319	8.298
DUX4L12	8.052	7.820	8.258	8.487	8.319	8.298
AC243651.1	8.029	7.870	7.644	7.214	7.397	7.841
DUX4L10	8.052	7.820	8.258	8.487	8.319	8.298
INKA2-AS1	7.508	7.472	7.519	7.511	7.718	7.521
PHAX	9.209	9.160	9.306	9.166	9.299	9.151
AL023973.1	7.859	8.033	8.002	7.839	8.036	7.955
TMEM14DP	11.497	11.554	11.614	11.559	11.704	11.507
RBM26	8.220	8.242	8.381	7.852	7.956	7.913
RBM27	9.561	9.639	9.848	9.699	9.691	9.479
RBM24	4.341	4.274	4.616	4.351	4.451	4.508
RBM25	9.756	9.705	9.778	9.939	9.986	9.560
RBM22	10.528	10.490	10.481	10.310	10.372	10.301
RBM23	9.544	9.575	9.661	9.625	9.614	9.670
RBM20	4.307	4.417	4.278	4.476	4.191	4.483
FMO9P	4.735	4.790	4.754	5.255	4.849	4.784
RBM28	8.401	8.395	8.426	8.200	8.313	8.383
EPHA8	8.382	8.285	8.152	8.414	8.409	8.620
EPHA7	7.287	7.062	7.008	6.762	6.829	6.854
EPHA6	5.282	5.269	5.141	5.241	5.277	5.082
EPHA5	4.998	5.252	5.249	5.225	4.886	5.007
EPHA4	7.705	7.672	7.755	7.842	7.804	7.754
EPHA3	8.893	8.902	8.978	8.578	8.620	8.770
EPHA2	10.900	10.940	10.945	10.792	10.574	10.751
EPHA1	9.081	9.179	9.238	9.177	9.143	9.159
AC010326.1	6.828	6.926	6.773	6.921	6.903	6.745
AL136304.1	8.492	8.528	8.746	8.798	8.834	8.838
LSMEM1	6.844	7.256	7.237	7.135	7.300	7.438
LSMEM2	6.731	6.767	6.838	6.716	6.792	7.053
ACAT1	10.217	10.278	10.433	10.094	9.987	10.129
AC021945.1	5.656	5.489	5.561	5.829	5.748	5.447
AL138478.1	7.602	7.224	7.078	7.729	7.649	7.462
ATOH1	7.371	7.407	7.576	7.579	7.554	7.626
AL669914.4	9.231	9.374	9.436	9.462	9.438	9.339
ZGLP1	8.598	8.548	8.677	8.515	8.525	8.580

COL9A2	7.793	7.895	7.841	7.864	7.974	7.895
COL9A3	6.605	6.632	6.608	7.006	6.653	6.424
LINC02530	5.501	5.512	5.321	5.469	5.619	5.537
FAM225A	6.635	6.627	6.702	6.618	6.724	6.463
LIMD2	6.870	6.987	7.098	7.376	7.197	7.112
GLB1L2	9.822	9.754	9.984	9.837	9.852	9.734
GLB1L3	6.808	6.790	6.542	6.956	7.054	6.949
AC005740.1	11.591	11.717	11.704	11.477	11.450	11.542
AC139795.2	7.666	7.765	7.875	7.760	7.774	7.727
AC139795.3	6.013	5.820	6.100	5.860	6.050	5.921
AC139795.1	8.229	8.336	8.426	8.293	8.428	8.440
ARL9	6.998	7.258	6.979	6.672	7.133	7.083
ARL1	10.263	10.250	10.454	10.441	10.479	10.402
ARL3	8.584	8.627	8.876	8.489	8.556	8.516
ARL2	9.971	9.980	10.232	10.057	10.054	9.974
ARL6	5.512	5.427	5.634	5.327	5.201	5.548
DHFR2	8.417	8.364	8.394	7.983	8.010	8.070
LINC02380	7.257	7.535	7.112	7.427	7.438	7.207
RRM2	12.111	12.120	12.285	11.709	11.761	11.676
RRM1	11.301	11.308	11.399	10.693	10.860	10.874
ZC3H12A	8.978	8.889	8.831	9.235	9.243	9.270
ZC3H12B	6.729	6.469	6.510	6.580	6.559	6.648
ZC3H12C	6.948	6.969	7.120	6.657	6.755	7.024
LINC02388	6.685	6.753	6.688	6.790	6.762	6.784
TSR1	9.339	9.452	9.350	9.088	9.021	9.079
TSR3	9.756	9.638	9.871	9.641	9.524	9.414
SBDSP1	9.294	9.369	9.337	9.362	9.125	9.280
RAD51-AS1	6.769	6.762	6.874	6.678	6.819	6.915
TH	6.580	6.637	6.483	6.491	6.568	6.487
TF	7.643	7.260	7.489	7.646	7.584	7.495
SNORD63B	5.958	5.897	5.797	5.779	6.072	6.076
MMP8	5.925	6.040	6.079	5.922	5.980	6.101
MLXIPL	8.011	8.235	8.250	8.483	8.292	8.294
TXNL1	11.223	11.330	11.365	11.403	11.399	11.313
CYP3A4	8.669	8.606	8.437	8.452	8.393	8.582
TAPBPL	8.310	8.305	8.422	7.833	7.996	7.981
MIRLET7A1HG	7.706	7.417	7.408	7.756	7.767	7.756
AL512604.4	8.100	7.933	7.696	8.123	8.070	7.955
CYP4A11	8.527	8.553	8.463	8.571	8.779	8.754
SRSF11	10.826	10.761	10.803	10.551	10.643	10.845
SRSF10	10.305	10.546	10.562	10.478	10.455	10.294
SRSF12	5.957	5.929	5.639	5.696	5.699	5.677
AL031009.1	7.204	7.119	7.102	7.242	7.025	7.201
TSPY4	8.003	7.967	8.011	8.092	8.024	8.064
ZPBP	6.243	6.441	6.413	6.182	6.301	6.563
AC112184.1	6.242	6.293	6.285	6.160	6.251	6.356
ZFYVE21	10.201	10.186	10.368	10.154	10.096	10.230
SULT1E1	6.374	6.433	6.560	6.187	6.493	6.494
SLC17A8	3.792	3.722	3.864	3.929	3.804	3.765
ZFYVE26	8.366	8.451	8.478	8.536	8.395	8.469
PIDD1	8.878	8.857	8.953	8.682	8.818	8.817
BBOX1	5.102	4.950	4.899	5.533	5.355	5.362
RASA4B	7.614	7.679	7.735	7.914	7.765	7.740
CRYBA2	5.231	5.438	5.427	5.459	5.543	5.593
CRYBA1	4.782	4.887	4.792	5.000	4.935	5.163
CRYBA4	6.055	6.366	6.256	6.209	6.373	6.367

RWDD2A	7.596	7.523	7.434	7.519	7.601	7.560
RWDD2B	9.123	9.018	9.003	9.119	9.015	8.991
RNU2-22P	4.207	4.092	4.133	4.142	4.053	4.260
LINC00526	6.831	7.071	7.182	6.685	6.780	6.766
LINC00525	6.093	6.076	5.829	6.102	6.076	6.243
LINC00523	8.324	8.412	8.496	8.441	8.363	8.357
LINC00520	11.046	10.800	10.145	11.087	11.114	11.189
LINC00528	4.557	4.454	4.812	4.870	4.653	4.606
LINC00529	4.591	4.289	4.195	4.022	4.356	4.442
ARL13B	7.502	7.379	7.347	6.951	6.848	6.949
TICRR	8.304	8.438	8.398	8.089	8.244	8.252
MC3R	6.323	6.384	6.535	6.624	6.383	6.603
MIR2052HG	7.308	7.314	7.287	7.365	7.364	7.386
UBALD1	9.638	9.616	9.907	9.770	9.916	9.588
UBALD2	9.759	9.792	9.924	9.526	9.448	9.454
GFM1	10.242	10.264	10.333	10.185	10.083	10.114
GFM2	9.939	9.966	10.090	10.020	9.868	9.937
RIPPLY1	6.357	6.525	6.249	6.417	6.456	6.369
RIPPLY3	4.850	4.959	4.994	5.201	4.801	4.755
RIPPLY2	6.549	6.481	6.562	6.686	6.812	6.679
WASIR2	4.455	4.473	4.535	4.391	4.421	4.784
SLC4A5	7.020	7.269	7.310	7.177	7.299	7.325
SLC4A4	9.110	8.945	9.161	8.716	8.953	8.868
SLC4A7	7.446	7.392	7.693	7.656	7.631	7.315
SLC4A1	8.623	8.544	8.644	8.728	8.636	8.647
RASA4DP	7.614	7.679	7.735	7.914	7.765	7.740
SLC4A3	7.438	7.744	7.724	7.667	7.738	7.718
SLC4A2	10.084	10.009	10.125	9.942	10.009	10.027
DCTN2	11.487	11.493	11.564	11.429	11.364	11.376
DCTN3	10.684	10.682	10.906	10.725	10.685	10.668
DCTN1	10.525	10.602	10.631	10.485	10.482	10.405
DCTN6	10.875	11.027	11.062	10.967	10.971	10.906
SLC4A8	8.859	8.747	8.596	8.843	8.853	8.961
DCTN4	10.425	10.523	10.465	10.217	10.167	10.160
DCTN5	10.515	10.528	10.681	10.460	10.398	10.605
KCMF1	11.216	11.294	11.341	11.304	11.335	11.338
RPS4Y1	8.071	8.235	8.268	8.284	8.215	8.193
ITCH	12.454	12.526	12.685	12.494	12.468	12.459
AC135050.2	11.750	11.798	12.028	11.675	11.577	11.660
KNTC1	8.856	8.764	8.648	8.258	8.189	8.350
AC135050.6	8.792	8.862	8.850	8.153	8.361	8.605
BROX	10.412	10.403	10.450	10.106	9.992	10.242
PLCL2	8.300	8.423	8.492	8.064	8.020	8.117
PLCL1	4.771	4.749	4.676	4.798	5.122	4.785
MXRA5	6.601	6.766	6.532	6.622	6.670	6.657
MXRA7	9.327	9.393	9.480	9.240	9.269	9.230
MXRA8	7.982	8.005	7.883	8.029	8.051	8.104
INSYN1-AS1	6.928	6.587	6.934	7.088	6.875	7.052
HHATL	6.471	6.761	6.834	6.355	6.621	6.562
AD000813.1	5.638	5.697	5.375	5.782	5.611	5.565
AL031313.1	6.411	6.496	6.453	6.394	6.539	6.397
IGSF8	8.433	8.500	8.751	8.517	8.727	8.432
KLRD1	7.337	7.598	7.423	7.448	7.751	7.660
TRAK2	9.288	9.317	9.552	9.064	9.410	9.019
IGSF5	6.432	6.333	6.182	6.471	6.439	6.329
IGSF6	4.444	4.319	4.400	4.352	4.594	4.531

OR2S2	7.805	7.940	7.740	7.796	8.034	7.746
IGSF3	9.184	9.012	9.153	8.804	9.092	8.942
CICP18	4.095	4.251	4.353	3.909	4.106	4.174
MOB1A	11.149	11.075	11.076	10.886	10.755	10.974
MOB1B	7.685	7.635	7.928	7.566	7.389	7.578
CLDN10-AS1	5.120	5.155	5.050	4.974	5.141	5.140
TRAK1	10.350	10.332	10.347	10.310	10.238	10.414
ILVBL	10.191	10.176	10.409	9.879	10.014	9.988
ITGB1	13.485	13.561	13.515	13.504	13.349	13.553
ITGB3	8.044	8.026	7.930	8.020	8.131	8.292
ITGB2	7.629	7.661	7.626	7.834	7.761	7.594
ITGB5	11.639	11.687	11.777	11.500	11.395	11.458
ITGB4	10.810	10.934	11.042	10.786	10.717	10.692
ITGB7	8.149	8.018	8.146	8.422	8.205	8.273
ITGB6	11.601	11.747	11.799	11.589	11.295	11.406
ITGB8	10.698	10.741	10.652	10.495	10.463	10.735
SLC37A1	8.578	8.537	8.575	8.585	8.576	8.570
KRT19P2	7.038	7.238	7.469	7.419	6.940	7.172
AL513165.2	9.842	9.874	9.936	9.842	9.697	9.874
AC091544.4	6.593	6.633	6.193	6.625	6.637	6.637
AC091544.2	7.209	7.260	6.995	7.175	7.252	7.275
KCNA10	6.735	6.897	6.934	6.997	6.888	6.829
AICDA	5.761	5.912	5.545	5.649	5.722	5.823
MAT1A	7.939	8.144	8.094	8.183	8.148	8.084
AC130650.2	6.002	5.729	5.745	6.061	5.640	5.967
CFAP69	7.467	7.382	7.344	7.363	7.522	7.508
RAN	12.806	12.875	13.026	12.910	12.802	12.842
CFAP61	6.933	6.847	6.868	6.902	7.167	6.932
CFAP65	7.756	7.860	7.820	7.836	8.030	7.993
FGR	7.110	7.060	6.917	7.165	7.168	7.213
USP35	7.250	7.225	7.141	7.109	7.121	7.105
FGG	5.383	5.332	5.605	5.480	5.454	5.763
FGA	7.735	7.807	7.873	7.813	8.023	7.765
RAX	8.049	8.135	8.110	8.302	8.261	8.288
ZAN	7.507	7.523	7.378	7.220	7.407	7.569
EXT2	9.612	9.726	9.823	9.642	9.644	9.730
EXT1	9.796	9.808	9.942	9.710	9.879	9.584
RNA5SP244	7.424	7.558	7.557	7.247	7.772	7.267
AC007192.1	10.931	10.862	11.123	10.697	10.663	10.683
LY96	4.719	4.919	4.855	4.650	5.025	4.809
SUMO2P3	12.617	12.628	12.692	12.617	12.655	12.614
AC022424.1	4.915	5.008	5.040	4.956	4.857	4.990
AL133297.2	5.923	5.660	5.918	6.019	5.766	6.010
ALDH5A1	8.642	8.745	8.834	8.141	8.274	8.291
OSBP	10.647	10.634	10.684	10.485	10.287	10.411
TCTA	10.097	10.266	10.311	10.049	10.037	10.081
AC011389.1	7.662	7.919	7.772	7.834	7.867	7.830
AC007846.1	6.623	6.550	6.444	6.557	6.698	6.677
OCM	6.868	6.848	6.885	6.618	6.658	6.865
USP3-AS1	4.972	4.886	4.613	4.915	4.838	4.693
TEFM	8.930	8.890	8.988	9.035	8.973	8.985
NUS1P1	7.922	7.857	7.833	7.800	7.854	7.895
RPL23AP65	14.341	14.360	14.394	14.370	14.332	14.374
NUS1P3	7.176	7.473	7.437	7.197	7.275	7.399
NUS1P2	7.176	7.473	7.437	7.197	7.275	7.399
PPP1R26-AS1	7.249	7.407	7.092	7.512	7.285	7.309

CASD1	8.088	8.155	8.162	7.685	7.674	7.826
RBMS1P1	10.452	10.271	10.361	10.325	10.323	10.527
AC099730.1	4.654	4.822	4.460	4.919	4.839	4.737
APOBEC2	7.560	7.595	7.572	7.668	7.585	7.578
RPS13P2	13.793	13.874	13.905	13.979	13.935	13.958
CEL	6.177	6.200	6.186	6.477	6.266	6.362
TMED7	10.269	10.340	10.292	10.182	10.251	10.341
BFSP2	6.081	6.218	6.132	6.262	6.470	6.350
TMED5	11.309	11.312	11.217	11.323	11.257	11.368
TSIX	6.734	6.917	6.839	7.041	6.912	6.918
TMED4	11.481	11.526	11.941	11.256	11.277	10.929
UHRF1BP1L	7.906	7.995	7.945	7.797	7.747	7.894
SERPIND1	5.599	5.803	5.875	5.923	5.684	5.781
RNASEH2A	9.746	9.690	10.001	9.410	9.390	9.375
RNASEH2C	8.681	8.650	8.849	8.447	8.466	8.502
RNASEH2B	8.529	8.720	8.324	8.659	8.696	8.743
RBIS	9.201	9.335	9.409	9.195	9.090	9.110
MAOB	11.489	11.502	11.564	11.303	11.194	11.300
MAOA	8.591	8.629	8.695	8.360	8.292	8.445
NEIL1	7.937	7.765	8.080	8.087	8.069	7.851
NEIL3	7.636	7.661	7.895	7.193	7.391	7.246
NEIL2	7.892	7.753	8.048	7.641	7.598	7.896
HNRNPR	11.463	11.395	11.525	11.257	11.225	11.206
CLYBL	7.689	7.752	7.710	7.909	7.814	7.653
AHI1	7.649	7.585	7.548	7.784	7.740	7.704
AC074008.1	5.956	5.947	5.617	5.791	5.789	5.687
AP001062.3	8.263	8.148	8.401	8.563	8.468	8.367
AC243965.2	5.155	5.181	5.251	5.153	5.208	5.076
RNU6-64P	6.350	6.102	6.376	6.347	6.762	6.693
MESP1	9.690	9.642	9.875	9.440	9.448	9.456
MESP2	8.972	8.980	8.983	9.015	9.066	9.039
MMP27	6.042	5.968	5.983	6.184	5.977	6.082
SNCAIP	6.704	6.657	6.472	6.734	6.753	6.698
OLMALINC	7.989	8.093	8.179	7.801	7.759	7.856
C1QBP	10.656	10.852	10.900	10.776	10.734	10.803
AC100774.1	4.952	5.148	4.826	5.045	5.010	5.001
HNRNPL	10.857	10.599	10.789	10.493	10.515	10.503
TAAR5	7.575	7.453	7.321	7.707	7.804	7.562
AP002472.1	9.540	9.637	9.529	9.430	9.522	9.486
TAAR2	6.610	6.656	6.734	6.654	6.697	6.805
TAAR9	6.413	6.453	6.315	6.408	6.434	6.582
TAAR8	6.601	6.906	6.424	6.668	6.488	6.710
ADSS2	10.639	10.766	10.691	10.544	10.457	10.435
AC079160.1	6.124	6.084	6.109	6.146	6.353	6.287
ADSS1	7.631	7.516	7.885	7.417	7.494	7.507
RCE1	8.852	9.030	9.166	9.227	9.111	9.020
UVSSA	9.129	9.026	9.002	8.821	9.099	8.921
SPANXA1	7.833	7.992	8.035	8.055	8.070	8.173
SPANXA2	7.833	7.992	8.035	8.055	8.070	8.173
SCN9A	6.365	6.177	6.543	6.280	6.200	6.464
IGHV3-33	7.842	8.095	8.219	8.243	8.177	8.357
IGHV3-30	7.842	8.095	8.219	8.243	8.177	8.357
VSTM4	7.476	7.515	7.633	7.963	7.697	7.618
PATJ	8.507	8.444	8.383	7.994	8.088	8.334
VSTM1	4.743	4.855	5.033	4.777	5.065	4.947
TSPY13P	5.699	6.018	5.845	6.396	6.072	5.960

RAD54B	7.477	7.532	7.528	7.400	7.446	7.287
RAD54L	8.206	8.180	8.116	8.001	8.052	8.034
ABCB1	7.960	7.975	8.156	7.767	7.756	7.845
ABCB7	7.915	7.882	7.954	7.745	7.838	7.793
ABCB6	7.867	8.029	7.982	8.165	8.229	8.071
ABCB5	6.844	6.841	6.799	6.848	6.878	6.975
ABCB4	7.657	7.619	7.624	7.130	7.335	7.321
ABCB9	9.363	9.415	9.617	9.208	9.175	9.023
ABCB8	7.698	7.834	7.884	7.678	7.747	7.820
HMGB1P3	6.355	6.147	6.014	5.932	6.226	6.139
HMGB1P4	13.082	13.095	13.157	12.934	12.997	12.733
HMGB1P5	13.218	13.387	13.396	13.059	12.997	12.987
EEF1A1P9	14.499	14.538	14.562	14.577	14.462	14.579
EEF1A1P6	14.499	14.538	14.562	14.577	14.462	14.579
EEF1A1P5	14.499	14.538	14.562	14.577	14.462	14.579
AP002381.2	6.790	6.925	7.018	6.888	6.938	6.935
TRIM43CP	6.793	6.730	6.758	6.858	6.755	6.999
GOLGB1	8.890	8.732	8.779	8.547	8.630	8.777
SLF2	7.656	7.778	7.848	7.596	7.680	7.618
SLF1	7.893	7.761	7.891	7.566	7.621	7.652
DNLZ	8.283	8.320	8.508	7.905	8.035	8.031
PMAIP1	10.118	10.079	10.168	10.194	10.428	10.075
SLC9A9	7.759	7.857	7.915	8.036	8.084	7.994
IST1	11.150	11.224	11.386	11.113	11.047	11.093
AL162384.1	6.236	6.444	6.313	6.218	6.576	6.541
AC104825.1	6.895	6.857	7.093	6.787	6.825	6.708
ERICH3-AS1	6.418	6.664	6.647	6.984	6.638	6.648
AL020997.5	7.209	7.414	7.479	7.194	7.393	7.465
SNRNP200	10.622	10.673	10.735	10.297	10.269	10.403
GADD45A	9.871	9.828	10.091	10.049	9.814	9.753
GADD45B	9.000	8.992	8.983	9.115	9.070	9.030
GADD45G	6.922	6.929	7.061	6.891	7.011	7.050
ZYG11B	9.104	8.983	9.042	8.836	8.834	9.086
ZYG11A	7.508	7.429	7.284	7.650	7.574	7.616
SPEF1	7.031	6.992	7.031	7.370	7.199	7.165
SPEF2	8.120	7.976	7.943	7.871	8.176	7.975
ZNF273	6.410	6.571	6.499	6.484	6.668	6.446
AC027228.3	6.885	6.583	6.517	6.888	7.137	6.827
ZNF276	8.052	8.071	8.050	8.046	8.028	8.025
ZNF275	9.342	9.380	9.332	9.462	9.089	9.119
ZNF274	9.560	9.405	9.594	9.569	9.569	9.676
KCNV2	4.772	4.625	4.661	4.755	4.741	4.813
KCNV1	5.999	6.053	5.840	5.779	5.873	5.918
PLCB1	8.523	8.415	8.152	8.540	8.581	8.589
NBPF25P	11.550	11.483	11.377	11.343	11.231	11.508
ITGA10	8.053	7.870	7.819	8.077	8.075	7.909
ITGA11	6.890	7.387	7.370	7.426	7.212	7.299
CTXN1	8.225	8.214	8.194	8.310	8.326	8.179
CTXN3	6.178	6.228	6.277	6.222	6.171	6.392
CCKBR	7.136	6.993	7.137	7.072	7.172	7.087
MAML2	9.224	9.295	9.191	8.954	9.032	9.176
MAML3	7.266	7.286	7.177	7.241	7.281	7.209
MAML1	9.391	9.298	9.392	9.240	9.260	9.287
AL035460.1	8.276	8.239	8.393	8.594	8.485	8.403
AC124283.5	9.164	9.213	9.179	8.990	8.986	9.119
AC012213.3	6.477	6.274	6.110	6.096	6.172	6.509

AC012213.5	8.747	8.805	8.804	8.547	8.667	8.562
AC124283.1	7.084	7.063	7.281	7.132	7.215	7.233
AC124283.3	6.491	6.469	6.130	6.339	6.514	6.512
AL022323.3	6.734	6.662	6.601	6.794	6.558	6.752
ERVMER61-1	4.325	4.116	4.079	4.226	4.158	4.411
HERC1	8.346	8.369	8.277	8.040	7.905	8.181
HERC3	7.966	7.813	7.854	7.928	7.865	7.830
HERC2	7.487	7.092	7.372	7.270	7.525	7.599
HERC5	8.972	8.901	8.832	8.796	8.934	8.669
HERC4	8.454	8.373	8.322	8.083	8.374	8.357
HERC6	9.258	9.291	9.229	8.862	8.995	8.979
AC025031.4	5.602	5.716	5.650	5.676	5.525	5.608
AC025031.2	9.097	9.152	9.039	9.651	9.706	9.651
PEX5L	6.424	6.311	6.279	6.591	6.633	6.739
C17orf58	9.500	9.613	9.592	9.104	9.358	9.326
SLCO2B1	7.972	7.797	7.683	7.683	7.997	8.089
PRR30	7.821	7.911	7.960	7.992	8.001	8.097
C17orf50	6.393	6.783	6.768	6.802	6.899	6.809
PRR32	6.509	6.705	6.575	6.694	6.667	6.769
AC008525.1	5.274	5.317	5.214	5.248	5.247	5.309
PRR34	8.129	8.333	8.288	8.175	8.384	8.180
PRR35	7.691	7.792	7.907	7.853	7.927	7.974
PRR36	8.613	8.654	8.582	8.731	8.666	8.623
MKNK2	11.633	11.491	11.754	11.348	11.352	11.468
LCLAT1	11.295	11.207	11.251	10.977	10.944	11.048
GLYCTK-AS1	6.765	7.057	6.936	6.828	7.104	7.068
MKNK1	9.997	10.079	10.113	9.977	9.932	10.080
LINC01905	7.079	6.786	6.638	6.822	6.752	6.903
LINC01904	6.750	6.714	6.561	6.597	6.647	6.827
AC078795.2	4.022	3.846	4.067	3.900	3.846	3.909
AC078795.3	4.076	3.908	4.033	4.079	3.999	4.035
AC078795.1	6.242	6.326	6.415	6.441	6.478	6.600
TRBV7-3	6.912	6.927	6.859	6.911	6.877	6.955
AC005176.1	6.473	6.601	6.511	6.658	6.768	6.742
LINC00052	7.098	6.971	6.929	6.859	7.084	6.967
IFNB1	6.649	6.725	6.638	7.100	7.254	7.146
ABCC6P1	7.536	7.684	7.757	7.553	7.471	7.421
ABCC6P2	7.536	7.684	7.757	7.553	7.471	7.421
BEAN1	5.294	5.668	5.179	5.657	5.316	5.311
AL137246.1	4.685	4.844	4.479	4.736	4.619	4.663
AAR2	10.195	10.304	10.327	10.132	10.116	10.093
OSBP2	8.032	8.037	8.245	8.121	8.137	8.090
LRP2	6.098	6.076	5.857	6.155	6.158	6.087
LRP3	7.864	7.993	8.252	8.360	8.449	8.096
LRP1	9.156	9.079	9.276	9.281	9.245	9.271
LRP6	9.610	9.578	9.697	9.503	9.715	9.532
LRP4	6.346	6.410	6.228	6.422	6.470	6.471
AL121928.1	8.024	8.119	8.090	7.972	7.966	8.128
LRP8	10.570	10.816	10.965	10.585	10.779	10.261
HLA-DOA	8.146	8.109	8.000	8.173	8.403	8.343
HLA-DOB	7.208	6.909	6.524	7.159	7.109	7.189
CALR3	5.153	5.104	5.056	5.307	5.114	5.160
STIM1	9.177	9.291	9.361	9.391	9.306	9.334
STIM2	8.494	8.573	8.468	8.546	8.462	8.497
ANTXR1	8.244	8.111	8.188	8.212	8.085	8.203
ANTXR2	10.664	10.772	10.877	10.466	10.366	10.400

SUCLA2-AS1	5.851	6.134	5.999	5.946	5.913	6.001
MAP2	7.253	7.250	7.368	7.420	7.400	7.414
C8A	6.546	6.269	6.433	6.471	6.509	6.520
C8B	7.898	7.828	7.941	7.896	7.912	7.851
MAP6	8.753	8.949	8.810	8.666	8.823	8.773
MAP7	9.675	9.736	9.617	9.309	9.427	9.474
MAP4	10.648	10.731	10.803	10.444	10.381	10.555
C8G	6.614	7.003	6.897	6.545	6.819	6.515
TEAD3	8.074	7.935	8.092	7.906	7.806	7.811
TEAD2	8.199	7.965	7.843	8.304	8.052	8.086
TEAD1	10.822	10.853	10.884	10.599	10.845	10.559
MAP9	7.721	7.532	7.755	7.595	7.672	7.683
UGT1A6	11.432	11.431	11.462	11.446	11.328	11.440
TEAD4	9.359	9.489	9.614	9.426	9.488	9.422
PTPN22	5.082	5.017	5.046	5.089	5.054	5.044
PTPN23	8.769	8.833	8.935	8.681	8.519	8.743
PTPN20	5.158	5.443	5.752	5.196	5.426	5.659
ANTKMT	9.161	9.198	9.447	8.940	8.907	8.921
LRRC37A17P	7.316	7.496	7.592	7.563	7.519	7.583
PLEKHF2	8.635	8.587	8.578	8.498	8.587	8.612
CHIT1	7.143	7.353	7.202	7.302	7.476	7.194
AC134682.1	7.635	7.391	7.589	7.563	7.454	7.569
HLA-DRB3	9.281	9.398	9.504	9.163	9.287	9.157
SERPINI1	6.274	6.322	6.626	6.326	6.727	6.742
SERPINI2	6.107	6.168	6.061	6.080	6.069	6.138
FANK1	6.507	6.408	6.436	6.736	6.360	6.384
MTPN	11.772	11.795	11.785	11.704	11.645	11.724
AC116456.2	6.978	6.712	6.656	6.863	6.975	6.817
EPHA10	8.068	8.120	8.223	8.163	8.087	8.030
DMRTA2	6.791	6.897	6.751	6.856	7.066	6.850
DMRTA1	5.583	5.532	5.455	5.815	5.472	5.767
EBPL	10.426	10.382	10.538	10.404	10.407	10.293
YY1AP1	9.568	9.526	9.618	9.420	9.283	9.408
AL713999.1	11.470	11.629	11.742	11.552	11.606	11.450
TOM1L2	9.034	8.967	9.175	9.045	8.807	8.959
MAPT	8.078	8.239	8.015	8.209	8.136	8.185
HLA-DQA1	6.499	6.530	6.428	7.021	6.768	6.385
HLA-DQA2	6.499	6.484	6.428	6.449	6.452	6.203
UTS2B	5.482	5.414	5.586	5.530	5.360	5.683
C14orf28	7.701	7.637	7.840	7.747	7.979	7.857
AC004264.1	6.774	6.797	6.785	6.789	6.952	6.965
UTS2R	9.948	10.106	10.072	10.075	10.097	10.240
MSR1	6.396	6.462	6.643	6.532	6.675	6.728
AVIL	8.782	8.890	8.861	8.891	8.885	9.080
ANKMY1	6.775	6.942	6.855	6.691	6.898	6.616
ANKMY2	10.081	10.029	10.196	9.934	9.957	9.948
MYCBP2	8.747	8.690	8.455	8.647	8.630	8.718
MYMX	7.863	7.755	7.829	8.090	7.969	8.105
AP000428.1	4.468	4.583	4.223	4.530	4.450	4.495
SYT9	6.338	6.448	6.190	6.491	6.612	6.454
HK2	10.909	10.977	11.038	10.942	10.918	10.865
HK3	8.282	8.430	8.217	8.417	8.243	8.446
HK1	10.399	10.385	10.532	10.350	10.140	10.274
LAX1	6.818	6.752	6.669	6.604	6.787	6.726
LINC01255	5.482	5.473	5.281	5.464	5.688	5.414
TBX3	7.617	7.446	7.685	7.728	7.651	7.854

MSRA	9.143	9.277	9.269	9.028	9.163	9.181
SUPT4H1	9.944	9.998	10.089	10.078	10.054	10.060
TBX1	7.651	7.524	7.402	7.679	7.834	7.678
DUS4L-BCAP29	8.047	8.045	8.181	7.750	7.991	7.869
EED	8.515	8.507	8.571	8.137	8.308	8.268
MAPKAPK5	9.757	9.781	9.864	9.689	9.577	9.639
MAPKAPK3	9.927	10.101	10.165	9.972	9.935	9.939
MAPKAPK2	10.207	10.296	10.449	10.209	10.193	10.198
ZNF815P	7.130	7.281	7.396	7.334	7.337	7.303
PDHX	9.935	10.010	10.063	9.910	9.803	9.994
LINC02023	5.300	5.311	5.140	5.336	5.331	5.396
LINC02026	6.503	6.226	6.130	6.459	6.239	6.589
LINC02029	6.681	6.855	6.568	6.661	6.777	6.523
LINC02028	5.814	5.825	5.875	5.767	5.748	5.985
SAMD5	7.260	7.127	7.147	7.319	7.526	7.358
SAMD1	9.381	9.305	9.474	9.304	9.506	9.391
LINC00888	9.446	9.474	9.580	9.345	9.419	9.370
ITGB1-DT	7.999	8.026	8.090	8.007	8.056	7.936
AC019117.4	5.405	5.563	5.397	5.786	5.331	5.583
SAMD8	6.740	6.848	6.627	6.246	6.347	6.472
SAMD9	10.833	10.843	10.797	10.752	10.868	10.697
TMEM38A	8.430	8.487	8.488	8.394	8.479	8.533
AL606834.2	5.515	5.735	5.711	5.410	5.632	5.589
SDCBP2-AS1	6.524	6.977	6.605	6.896	6.843	6.890
SBSPON	6.431	6.548	6.457	6.448	6.422	6.547
ZNF530	6.698	6.657	6.767	6.753	6.708	6.870
AL606834.1	5.268	5.312	5.372	5.441	5.375	5.265
BX088647.3	9.231	9.374	9.436	9.462	9.438	9.339
LRRC18	5.866	5.721	5.675	5.682	5.795	5.866
LRRC19	7.932	7.843	8.010	7.387	7.475	7.324
LRRC17	5.307	4.934	5.069	5.675	5.408	5.319
LRRC14	8.062	7.954	8.160	7.840	7.791	8.047
LRRC15	7.294	7.183	7.311	7.343	7.422	7.363
FAM111B	8.100	8.201	8.188	7.954	8.146	7.831
MIR194-2HG	8.516	8.399	8.578	8.583	8.641	8.734
FAM111A	10.877	10.871	10.993	10.830	10.644	10.659
DPPA5P4	6.464	6.666	6.786	6.499	6.744	6.918
SVOP	6.625	6.659	6.657	6.670	6.574	6.809
FAHD2B	9.431	9.641	9.714	9.478	9.585	9.534
CCDC103	7.430	7.493	7.577	7.352	7.646	7.518
DROSHA	9.057	9.003	9.153	8.853	8.866	8.956
CCDC105	7.237	7.373	6.984	7.099	7.239	7.343
CCDC107	8.426	8.466	8.619	8.548	8.484	8.486
CCDC106	7.349	7.450	7.654	6.824	6.746	7.044
CAVIN4	4.326	3.954	4.170	4.075	4.229	4.216
LINC01592	5.731	5.877	5.789	6.189	6.119	6.003
AC073415.1	5.119	5.145	5.015	5.070	5.333	5.174
LINC01597	6.413	6.550	6.764	6.116	6.277	6.365
AC092653.2	7.932	7.957	7.752	7.905	8.159	7.955
CR786580.1	6.139	6.096	6.076	6.214	6.293	6.282
CHRNA4	7.050	7.017	7.533	7.465	7.321	7.409
AP001347.1	5.989	6.133	5.974	6.265	6.130	6.105
CHRNA6	6.847	7.060	7.130	7.200	7.236	6.827
CHRNA7	6.743	6.757	6.617	6.803	6.702	6.551
CHRNA1	6.854	6.703	6.581	6.775	6.780	6.698
CHRNA2	8.238	8.000	8.281	8.489	8.330	8.350

CHRNA3	8.776	8.891	8.873	8.813	9.082	9.034
AC137056.2	4.906	4.815	4.813	5.001	4.771	4.646
AC093673.1	9.052	9.062	9.151	9.030	9.014	9.032
AC213033.2	7.981	7.880	7.994	8.183	7.956	7.961
AC010306.1	7.019	6.963	7.119	6.930	6.758	6.812
AL024498.1	5.982	6.199	6.173	6.304	6.454	6.206
LINC01049	6.832	6.729	6.634	6.685	6.854	6.821
ITPKB-IT1	7.753	7.646	7.860	7.993	7.888	7.908
LENEP	7.387	7.505	7.259	7.485	7.478	7.533
FSHB	6.813	6.798	7.092	6.892	7.115	6.958
FLG-AS1	4.277	4.509	4.150	4.158	4.353	4.481
AQP12B	9.887	9.844	9.778	9.715	9.826	9.873
AQP12A	9.887	9.844	9.778	9.715	9.826	9.873
FSHR	5.649	5.521	5.596	5.668	5.655	5.666
PEF1	10.602	10.628	10.766	10.510	10.376	10.492
AC093426.1	6.612	6.597	6.409	6.761	6.818	6.900
AP5Z1	8.306	8.116	8.238	7.957	7.915	8.166
SAP30	8.748	8.719	8.840	8.377	8.457	8.483
CWH43	6.111	5.685	5.934	6.331	6.112	5.985
SPATA5	6.775	6.631	6.629	6.383	6.401	6.458
LINC00311	6.141	6.373	6.216	6.071	6.022	6.175
LINC00310	6.247	6.247	6.093	6.795	6.475	6.275
LINC00313	7.319	7.219	7.358	7.196	7.171	7.432
LINC00315	7.894	7.843	7.825	7.732	7.815	7.949
LINC00314	4.087	3.826	4.015	4.017	4.020	4.081
LINC00317	6.539	6.665	6.695	6.707	6.790	6.757
C3orf80	6.603	6.561	6.856	5.920	6.219	6.208
FGFR1OP2	8.245	8.286	8.439	8.159	8.056	8.233
C3orf85	4.230	3.987	4.056	4.228	4.160	4.211
LAPTM5	8.371	8.588	8.287	8.387	8.263	8.520
ARFRP1	9.405	9.474	9.581	9.484	9.406	9.206
AC092140.2	4.718	4.347	4.601	4.690	4.636	4.685
COX6CP1	7.671	7.726	7.872	7.647	7.653	7.449
COX6CP2	6.888	6.848	7.067	7.001	7.065	7.322
VRTN	7.729	7.505	7.634	7.675	7.666	7.722
KDM2A	10.769	10.743	10.719	10.824	10.710	10.615
KDM2B	9.646	9.781	9.785	9.694	9.768	9.789
SIRPG	6.841	7.011	6.940	7.418	7.342	7.361
PPHLN1	9.889	9.885	9.968	9.669	9.717	9.724
SIRPD	7.250	7.201	7.319	7.376	7.638	7.406
SIRPA	9.145	8.986	9.164	8.719	9.083	8.897
LCE2B	6.003	6.183	6.120	6.053	5.995	6.041
AC000093.1	8.106	8.035	8.345	8.541	8.182	8.204
TRAPPC9	10.550	10.639	10.697	10.502	10.604	10.676
TRAPPC8	10.247	10.216	10.346	10.241	10.438	9.954
TRAPPC1	9.801	9.791	9.903	9.835	9.829	9.752
TRAPPC3	10.128	10.168	10.191	10.014	10.158	10.106
TRAPPC2	8.465	8.524	8.878	8.444	8.490	8.579
TRAPPC5	9.973	10.007	10.291	9.954	9.872	9.987
TRAPPC4	9.805	9.829	9.897	9.635	9.468	9.645
PRPH	8.584	8.636	8.454	8.725	8.561	8.758
GABRQ	7.080	6.972	6.797	6.890	7.150	7.213
WASH4P	9.341	9.202	9.345	9.278	9.301	9.395
HNRNPUL2-BSCl	9.989	10.037	10.120	9.921	9.898	9.914
AL162724.1	6.005	6.004	6.349	6.323	6.385	6.507
MTCP1	6.551	6.640	6.744	6.483	6.633	6.558

AC270258.1	4.396	4.571	4.221	4.462	4.509	4.397
PSMG4	8.611	8.590	8.740	8.292	8.280	8.303
OXT	7.660	7.492	7.610	7.583	7.844	7.783
ABCA17P	4.826	4.981	5.036	5.044	5.076	4.872
PSMG1	9.991	10.136	10.176	9.967	10.044	10.041
PSMG2	11.068	11.121	11.197	10.822	10.920	10.796
PSMG3	9.655	9.694	9.940	9.775	9.699	9.688
RRN3P3	9.409	9.632	9.458	9.533	9.370	9.493
RRN3P2	9.409	9.632	9.458	9.533	9.370	9.493
RRN3P1	9.409	9.632	9.458	9.533	9.370	9.493
AC093627.16	5.629	5.637	5.650	5.415	5.501	5.815
AC004151.1	7.846	8.015	8.180	8.088	7.814	7.983
PABPC1P3	13.179	13.076	13.119	13.096	13.248	13.081
AL133268.1	9.129	9.158	9.232	9.007	8.933	9.090
C9orf116	7.952	7.849	8.197	8.081	7.906	8.003
CD44-AS1	6.199	5.752	5.837	6.131	5.757	6.039
FOXM1	9.858	9.815	9.900	9.277	9.394	9.365
DLX6-AS1	6.091	5.909	5.930	6.089	5.873	5.980
GDAP2	7.720	7.764	7.745	7.573	7.707	7.503
GDAP1	7.862	7.904	8.032	7.507	7.392	7.583
FAHD1	10.944	11.049	11.137	10.773	10.804	10.813
CUTALP	8.922	8.900	8.681	8.759	8.596	8.846
TUG1	11.720	11.733	11.669	11.569	11.558	11.711
ILDR2	5.814	5.926	5.766	5.776	5.752	5.646
ILDR1	9.063	9.050	9.139	8.880	9.049	9.038
GLTP	11.040	11.082	11.218	11.079	11.035	11.058
AC083864.3	3.956	4.020	4.031	4.179	3.924	4.149
SUGT1P1	3.739	3.748	3.745	3.869	3.756	3.810
SUGT1P3	4.739	4.782	4.805	4.336	4.708	4.623
IL3RA	7.604	7.606	7.655	7.917	7.844	7.828
AC009065.7	5.879	5.787	5.901	6.237	5.907	6.061
GGTLC4P	10.427	10.413	10.580	10.351	10.206	10.300
NADSYN1	9.511	9.565	9.639	9.419	9.239	9.487
NPY4R2	6.188	6.259	6.188	5.996	6.132	6.303
UBTF	9.305	9.384	9.323	9.255	9.207	9.236
AC009812.4	5.416	5.148	5.337	5.358	5.298	5.425
CDCA2	9.100	9.013	9.240	8.591	8.932	8.623
CDCA3	10.304	10.208	10.441	9.832	10.101	9.838
CDCA7	10.171	10.149	10.259	9.976	9.902	9.979
CDCA4	9.534	9.697	9.770	9.354	9.493	9.398
CDCA5	10.017	10.023	10.101	9.624	9.609	9.692
NDUFA4L2	7.187	7.275	7.371	7.501	7.255	7.144
TRIM60P18	3.811	3.743	3.849	3.885	3.574	3.780
CRB1	6.344	6.418	6.439	6.372	6.624	6.479
RSF1	10.157	9.899	9.977	9.741	10.344	9.642
AC131160.1	10.848	10.902	11.037	10.861	10.759	10.791
UBE2V1P9	7.889	7.650	7.839	8.062	7.987	7.681
CRB2	5.861	5.962	6.040	5.949	6.071	5.994
UBE2V1P3	7.889	7.650	7.839	8.062	7.987	7.681
AC004522.3	7.951	8.090	7.687	7.814	8.046	7.996
MAPT-AS1	8.365	8.519	8.612	8.576	8.402	8.437
FAS	6.780	6.766	6.762	6.825	6.823	6.918
FAP	5.692	5.871	5.764	5.859	5.963	5.886
AC012085.2	6.026	6.160	6.288	6.678	6.611	6.487
SPRED1	8.076	8.270	8.192	7.793	7.863	8.161
SPRED2	10.116	10.093	10.059	9.750	9.699	9.812

SPRED3	8.895	8.805	8.955	9.225	9.093	9.063
RELT	8.446	8.576	8.593	8.470	8.604	8.472
CTNNA2-AS1	4.558	4.598	4.642	4.563	4.402	4.585
RELN	7.373	7.402	7.381	7.601	7.759	7.409
RELB	8.384	8.418	8.497	8.514	8.641	8.599
RELA	10.755	10.861	10.984	10.799	10.760	10.770
HDGF	11.783	11.841	11.966	11.702	11.743	11.658
CRISPLD2	7.863	7.967	7.919	8.046	7.981	8.006
CRISPLD1	4.624	4.612	4.531	4.534	4.595	4.700
ARHGAP26-AS1	5.934	6.170	6.133	6.266	6.198	6.404
PTH2R	5.953	6.375	5.912	6.284	6.161	6.454
UBE2R2	12.169	12.155	12.303	12.132	12.298	12.073
KLK1	8.305	8.342	8.414	8.416	8.321	8.474
KLK2	7.844	7.819	7.922	7.992	7.971	7.869
KLK3	7.813	7.648	7.590	7.722	7.676	7.616
KLK4	9.541	9.460	9.436	9.394	9.496	9.548
KLK5	6.765	6.912	6.884	7.199	6.938	6.840
KLK6	12.618	12.719	12.823	12.730	12.738	12.732
KLK7	7.959	8.026	8.049	7.998	7.988	8.088
KLK8	7.568	7.468	7.631	7.919	7.733	7.697
KLK9	7.568	7.468	7.631	7.919	7.733	7.697
AC116913.1	6.101	6.276	6.163	6.326	6.127	6.102
ZNF843	9.996	10.017	9.693	10.092	10.330	10.305
RHBDL2	7.389	7.384	7.487	7.419	7.764	7.575
RHBDL3	7.596	7.581	7.258	7.582	7.626	7.556
AC270153.2	5.087	5.195	5.205	5.304	5.341	5.302
RHBDL1	6.307	6.467	6.308	6.182	6.342	6.310
AL355297.3	9.125	9.413	9.580	9.394	9.416	9.509
TRIM6-TRIM34	9.012	8.999	9.043	8.865	8.983	8.951
STUM	7.569	7.715	7.665	7.802	7.975	7.869
AC078980.1	4.408	4.329	4.205	4.395	4.275	4.373
COPS8	10.998	11.010	11.150	10.979	10.799	10.904
COPS9	10.702	10.707	10.903	10.744	10.776	10.766
COPS2	11.798	11.835	11.938	11.887	11.782	11.855
COPS3	9.491	9.526	9.736	9.405	9.264	9.409
COPS6	10.501	10.527	10.764	10.327	10.126	10.256
COPS4	10.019	10.130	10.126	9.980	10.067	10.126
COPS5	9.998	10.065	10.205	10.091	10.068	9.989
ZNF592	9.540	9.637	9.540	9.541	9.522	9.589
ZNF593	8.900	8.960	9.156	8.923	8.889	8.835
ZNF596	4.886	4.989	4.914	5.115	5.359	4.952
MDGA1	7.942	7.938	7.905	8.063	8.168	7.982
MDGA2	4.521	4.693	4.964	4.969	4.615	4.770
ZNF598	9.586	9.745	9.861	9.510	9.572	9.555
ZNF599	6.793	6.766	6.906	6.786	6.584	6.790
ZSWIM8-AS1	8.219	8.131	8.033	8.249	8.238	8.260
TMC3-AS1	6.864	6.449	6.635	6.907	6.812	6.742
RCC1L	9.473	9.422	9.574	9.329	9.360	9.240
ZFR2	6.988	7.072	7.193	7.121	7.174	7.042
SHLD2	9.066	9.085	9.014	8.765	8.627	8.890
FP325330.1	7.089	6.877	6.589	7.103	6.935	7.029
FAM135B	7.409	7.547	7.514	7.686	7.535	7.542
SHLD1	7.497	7.530	7.600	7.557	7.748	7.807
IGF2	7.019	6.935	6.993	7.196	7.062	7.018
IGF1	7.668	7.792	7.801	7.987	7.897	7.945
AC023421.1	3.916	4.038	3.803	4.015	4.025	4.060

AC244230.2	7.596	7.530	7.413	7.661	7.558	7.777
ATP5F1D	10.471	10.451	10.679	10.166	10.208	10.114
ATP5F1E	12.564	12.720	12.754	12.697	12.695	12.740
ATP5F1A	12.824	12.964	13.008	12.891	12.561	12.804
ATP5F1B	13.353	13.447	13.539	13.363	13.177	13.322
ATP5F1C	12.529	12.563	12.677	12.620	12.642	12.569
CYP27A1	8.559	8.529	8.466	8.438	8.496	8.584
ELOVL2-AS1	5.014	4.978	4.808	4.983	4.989	4.996
DDRGK1	9.123	9.125	9.179	8.793	9.023	9.016
FBXL20	10.167	10.148	10.266	9.867	9.953	9.845
FBXL22	5.360	5.466	5.288	5.554	5.514	5.410
SSB	10.359	10.334	10.398	10.044	10.346	10.161
ZNF337	7.955	7.949	7.744	7.784	7.837	7.846
ZNF334	5.730	5.421	5.696	5.732	5.781	5.740
ZNF335	8.704	8.724	8.860	8.788	8.762	8.823
FAM168B	10.922	10.915	10.981	10.678	10.516	10.703
ZNF333	7.067	6.891	6.883	7.002	7.095	7.128
ZNF330	8.834	8.881	8.935	8.608	8.544	8.480
FAM168A	8.963	8.808	8.895	8.777	8.776	8.706
TEX15	6.601	6.748	6.675	6.870	6.782	6.955
TEX14	6.070	5.965	6.088	6.200	6.111	6.212
TEX11	7.192	7.314	7.396	7.583	7.407	7.422
TEX10	8.975	9.116	9.092	8.911	8.946	8.945
TEX12	4.090	4.060	4.111	3.989	3.912	4.121
RACGAP1P	4.866	4.751	4.819	4.729	5.124	4.572
AC063923.2	4.458	4.353	4.370	4.376	4.603	4.399
THYN1	9.176	9.111	9.302	9.160	9.114	9.170
NTF3	7.411	7.122	7.068	7.285	7.288	7.495
NTF4	6.453	6.384	6.438	6.408	6.119	6.234
UGDH	10.235	10.310	10.200	10.167	10.272	10.198
NCAPH2	8.917	8.966	9.036	8.940	8.960	8.965
SLCO1A2	7.047	7.197	6.890	6.865	7.110	6.976
TSEN54	10.145	10.187	10.287	10.098	10.040	10.048
AL357093.2	6.963	7.077	6.992	7.264	7.077	6.942
CXADR	11.475	11.809	11.915	11.644	11.619	11.355
CYP24A1	10.580	10.706	10.676	10.440	10.454	10.502
GTF3C2	11.073	11.061	11.133	10.896	10.966	10.993
ZNF33B	7.689	7.554	7.692	7.832	7.805	7.697
ZNF33A	7.246	7.470	7.425	7.001	6.928	7.176
TTC23	8.519	8.610	8.711	8.283	8.406	8.418
SNRNP48	8.025	7.867	7.966	7.820	7.841	7.976
MAD2L2	9.123	9.189	9.396	8.921	9.035	9.112
MAD2L1	10.158	10.069	10.315	9.736	9.722	9.831
AC125232.2	8.228	8.464	8.401	7.869	8.135	8.215
AC125232.1	8.228	8.464	8.401	7.869	8.135	8.215
ARHGEF35-AS1	7.162	7.203	7.256	7.373	7.284	7.256
BMPER	4.289	4.321	4.204	4.358	4.381	4.371
SYNJ2BP-COX16	10.254	10.342	10.450	10.298	10.293	10.245
LRP2BP	5.571	5.993	6.030	6.026	5.947	5.745
LINC01208	8.260	8.245	8.236	8.431	8.276	8.143
LINC01209	5.384	5.378	5.105	5.669	5.464	5.479
AC127029.3	7.738	7.861	7.945	7.774	7.988	7.983
LINC01204	7.205	7.439	7.361	7.536	7.397	7.309
LINC01206	6.639	6.726	6.776	7.015	6.855	6.867
AC021092.1	4.496	4.298	4.494	4.790	4.592	4.506
AC021092.2	8.121	7.888	7.678	7.629	7.591	8.026

AC011346.1	6.667	6.671	6.607	6.601	6.694	6.748
GALNS	9.234	9.193	9.238	8.910	8.790	9.031
CR759964.1	8.439	8.433	8.212	8.323	8.555	8.407
SNORA74A	9.302	9.334	9.367	9.326	9.456	9.432
AC012314.8	10.357	10.410	10.540	10.053	10.003	10.044
AC012314.3	10.357	10.410	10.540	10.053	10.003	10.044
AC012314.7	11.667	11.674	11.756	11.512	11.455	11.474
NPY2R	6.358	6.468	6.300	6.414	6.378	6.415
AC012314.5	11.667	11.674	11.756	11.512	11.455	11.474
AC012314.4	10.357	10.410	10.540	10.053	10.003	10.044
CDRT15L2	6.349	6.431	6.479	6.564	6.364	6.309
DYNLL1	12.838	12.854	12.986	12.883	12.901	12.888
DYNLL2	9.334	9.546	9.502	9.427	9.429	9.345
PRDM5	6.459	6.383	6.406	6.852	6.538	6.392
KHDC1	7.870	7.930	8.052	7.727	7.868	7.918
MIR15A	4.301	4.067	4.388	4.416	4.221	4.428
GPS1	9.241	9.209	9.446	9.144	8.860	9.014
GPS2	9.973	10.101	10.384	10.139	10.405	9.924
OTUD6B-AS1	9.025	8.892	8.917	8.804	8.813	8.903
AL512631.1	7.711	7.754	7.543	7.764	7.772	7.797
AL512631.2	7.426	7.288	7.480	7.390	7.540	7.575
GLO1	12.105	12.170	12.246	12.084	12.020	11.997
AC236040.1	7.736	7.709	7.860	8.013	7.863	7.976
PRDM7	5.397	5.375	5.334	5.309	5.333	5.603
CARHSP1	10.278	10.321	10.674	10.031	10.087	10.236
MIR3671	4.685	4.495	4.869	4.535	4.767	4.694
MIR155	4.376	4.236	4.373	4.654	4.631	4.462
NPY1R	9.093	8.988	9.091	8.769	8.674	8.925
TTC14	8.484	8.482	8.480	8.221	8.256	8.498
TTC17	10.287	10.321	10.394	10.152	10.153	10.148
TTC16	6.824	6.908	7.035	7.084	6.951	6.969
TTC13	7.596	7.798	7.720	7.369	7.649	7.608
TTC12	7.672	7.622	7.684	7.679	7.818	7.796
TTC19	10.646	10.763	10.737	10.475	10.359	10.374
AC092115.2	13.204	13.254	13.298	13.171	13.113	13.111
GTSE1-DT	6.328	6.132	6.263	6.209	6.085	6.076
AC093899.2	5.530	5.469	5.731	5.122	5.611	5.030
SLC31A1	9.417	9.475	9.429	9.402	9.425	9.280
SLC31A2	7.617	7.852	7.789	7.710	7.696	7.727
ZFP92	8.883	8.689	8.553	9.174	8.917	8.831
ZFP90	9.250	9.296	9.333	8.821	9.070	8.977
ZFP91	10.804	10.800	10.828	10.634	10.557	10.673
RICTOR	9.860	10.051	9.990	9.919	10.021	9.724
AC073136.1	10.848	10.876	10.865	10.631	10.750	10.741
AC073136.2	9.946	9.847	9.861	9.522	9.661	9.692
BX088556.1	7.344	7.403	7.456	7.273	7.478	7.510
EGR4	8.010	7.990	8.043	8.664	8.613	8.760
AC069213.1	4.021	3.907	3.712	3.955	3.979	3.896
DALRD3	9.958	10.122	10.207	9.804	10.013	9.809
AC069444.2	5.896	5.887	5.827	5.989	6.079	5.890
AC078860.3	4.842	4.995	4.565	4.761	4.928	4.927
PCDHA10	10.289	10.323	10.367	10.104	10.324	10.100
PCDHA11	10.289	10.323	10.367	10.104	10.324	10.100
PCDHA12	10.289	10.323	10.367	10.104	10.324	10.100
PCDHA13	10.289	10.323	10.367	10.104	10.324	10.100
PROK1	7.700	7.667	7.616	7.860	7.790	7.659

PROK2	4.244	4.140	3.992	4.095	4.088	4.165
KLF3-AS1	6.280	6.247	6.260	6.053	6.407	6.567
EPG5	8.451	8.510	8.485	8.431	8.206	8.324
PTDSS1	11.382	11.539	11.628	11.271	11.085	11.170
PTDSS2	7.810	8.080	8.186	8.071	7.868	8.004
MLIP	6.971	6.987	6.675	7.014	7.080	7.006
CHCHD10	12.202	12.315	12.511	12.237	12.296	12.152
CNTD1	7.941	7.947	8.082	8.258	8.257	8.049
CNTD2	8.837	8.991	8.805	9.040	9.160	9.036
ZEB1	6.872	6.803	7.004	7.083	7.068	7.060
GTF2A1L	5.509	5.630	5.622	5.696	5.713	5.733
OBSL1	10.313	10.284	10.633	9.938	9.909	9.989
FBXO45	9.305	9.185	9.230	8.996	9.348	9.000
KIF16B	6.655	6.689	6.658	6.438	6.655	6.770
FBXO46	9.546	9.563	9.619	9.407	9.516	9.665
FBXO41	8.546	8.554	8.406	8.258	8.142	8.355
FBXO40	7.075	6.951	7.064	7.282	7.097	7.217
FBXO43	6.231	6.330	5.937	6.286	6.391	6.294
FBXO42	8.946	8.752	8.813	8.962	8.823	8.923
AC211486.6	8.737	8.598	8.642	8.446	8.495	8.529
DCDC2B	7.156	7.219	7.421	7.439	7.284	7.246
AC211486.1	7.812	7.823	7.422	7.957	8.059	8.029
SFRP2	6.102	6.247	6.351	6.606	6.324	6.384
SFRP1	7.216	7.123	6.758	7.300	7.226	7.243
SFRP4	6.625	6.379	6.368	6.636	6.638	6.815
SFRP5	7.794	7.722	7.978	8.198	7.963	7.962
TP53I11	8.704	8.632	8.705	8.584	8.855	8.352
COL1A2	7.617	7.729	7.504	7.690	7.640	7.696
COL1A1	9.308	9.418	9.453	9.227	9.196	9.464
KIAA1217	8.525	8.550	8.375	8.288	8.617	8.590
LSP1P4	7.347	7.553	7.386	7.650	7.400	7.673
AP005432.2	5.992	5.868	5.819	6.085	5.900	5.815
DPY19L2	6.344	6.368	6.226	6.281	6.173	6.372
CEMIP	9.310	9.437	9.308	9.133	9.218	9.268
LACRT	6.798	6.951	6.644	6.950	6.769	6.871
AL391840.1	5.382	5.300	5.416	5.353	5.461	5.374
AL391840.2	5.765	5.534	5.339	5.466	5.359	5.323
AL391840.3	4.364	4.557	4.551	4.523	4.473	4.543
EIF1B	10.749	10.765	10.833	10.573	10.599	10.675
AC107464.1	6.403	6.390	6.186	6.520	6.448	6.603
MCM8-AS1	4.685	4.600	4.556	4.658	4.729	4.617
AL032819.2	7.772	7.930	8.147	8.096	8.120	8.078
AP001189.1	4.825	5.094	5.100	5.239	5.183	5.043
AP001189.5	5.814	6.021	5.876	5.829	5.786	6.129
AADAT	8.154	8.087	8.135	8.130	8.173	7.995
AADAC	6.380	6.357	6.370	6.412	6.494	6.456
C19orf33	11.740	11.806	12.051	11.924	11.678	11.929
EPCAM	12.982	13.066	13.139	13.056	13.110	13.141
LINC02270	6.153	6.149	6.016	6.224	6.173	6.158
DPPA3P2	4.039	4.108	4.021	3.947	4.005	4.017
LINC02272	4.573	4.753	4.568	4.466	4.627	4.796
LINC02273	7.964	7.924	7.826	8.026	7.951	8.019
LINC02274	4.143	4.434	4.228	4.645	4.540	4.412
COL6A1	8.812	8.792	8.995	8.841	8.761	8.942
WBP2	10.946	11.179	11.191	11.023	11.091	10.954
COL6A3	7.000	6.892	6.737	6.880	6.890	6.945

COL6A2	7.936	7.929	8.052	8.137	8.005	8.008
COL6A5	5.735	5.784	5.964	5.855	6.037	5.954
COL6A6	5.872	5.669	5.545	5.635	5.684	5.744
AC105206.1	6.152	6.278	5.799	6.233	6.098	6.160
KLK12	8.904	9.005	9.051	9.052	9.044	8.959
KLK13	7.042	7.093	7.241	7.270	7.406	7.344
KLK10	10.224	10.345	10.350	10.290	10.434	10.226
KLK11	8.028	8.102	8.211	8.147	8.280	8.243
KLK14	8.020	8.018	8.132	8.171	8.112	8.197
KLK15	7.252	6.987	7.291	7.651	7.398	7.319
AC010168.2	6.809	6.916	6.663	6.908	6.916	6.939
AL049647.1	7.180	6.926	6.730	7.121	7.129	7.138
PLLP	9.299	9.365	9.560	9.165	9.095	9.210
AL121956.1	5.593	5.634	5.296	5.973	5.730	5.869
AL121956.2	5.593	5.634	5.296	5.973	5.730	5.869
MORF4	12.008	12.128	12.125	11.953	11.974	11.999
AC105230.1	7.508	7.512	7.706	7.582	7.731	7.605
GCC2-AS1	7.473	7.518	7.652	7.448	7.729	7.395
TMA16	6.202	6.490	6.690	5.943	6.220	6.309
AC016026.1	7.863	7.910	7.894	7.624	7.751	7.741
PCM1	10.301	10.292	10.291	10.020	9.919	10.050
AC004623.1	4.214	4.064	4.248	4.255	4.061	4.068
LRRK1	7.850	8.039	8.068	7.888	7.871	7.814
LRRK2	4.784	4.951	4.813	4.763	4.825	4.827
PRELID3B	10.996	11.154	11.106	11.090	11.029	11.053
PRELID3A	7.539	7.489	7.479	7.605	7.478	7.378
MRPL10	11.041	11.118	11.324	10.904	10.889	10.899
MRPL11	10.460	10.569	10.739	10.504	10.640	10.517
MRPL12	9.992	10.057	10.225	9.966	9.955	9.987
MRPL13	9.438	9.398	9.562	9.629	9.479	9.544
MRPL14	9.823	9.883	10.026	10.014	9.895	9.929
MRPL15	10.552	10.604	10.725	10.625	10.532	10.571
MRPL16	9.803	9.838	10.027	9.692	9.610	9.669
MRPL17	10.491	10.581	10.884	10.596	10.475	10.480
MRPL18	11.165	11.252	11.379	11.289	11.237	11.222
MRPL19	8.781	8.820	8.887	8.561	8.435	8.587
AC145098.2	6.168	6.111	6.109	6.287	6.290	6.208
PLA1A	6.675	6.881	6.737	6.441	6.849	6.653
TMEM71	4.419	4.493	4.369	4.371	4.336	4.371
KDSR	10.497	10.539	10.571	10.098	10.271	10.033
AC019257.8	8.561	8.656	8.669	8.574	8.406	8.503
ANKRD20A2	8.236	8.156	8.141	8.146	8.143	8.231
ANKRD20A3	8.236	8.156	8.141	8.146	8.143	8.231
RNU6-29P	10.837	10.833	11.041	10.739	10.687	10.776
ANKRD20A4	8.236	8.156	8.141	8.146	8.143	8.231
SNCG	6.799	6.905	7.025	7.237	7.016	7.034
SNCB	7.678	7.852	8.064	7.775	7.864	7.923
RNF128	11.930	12.042	12.087	11.964	12.018	12.094
RNF126	9.234	9.396	9.402	9.274	9.065	9.156
RNF125	6.072	6.174	6.253	6.388	6.078	6.107
RNF123	9.096	9.372	9.355	9.172	9.210	9.261
RNF122	8.635	8.608	8.667	8.681	8.842	8.814
RNF121	8.384	8.416	8.538	8.357	8.339	8.277
Z84488.2	6.662	6.579	6.497	6.531	6.634	6.570
OR11A1	5.790	5.929	5.886	6.044	5.881	5.883
PHBP19	5.765	5.988	6.124	5.630	5.742	5.802

MIR103A2	7.740	7.830	7.776	7.526	7.737	7.757
SACS	7.312	7.092	7.042	6.981	6.769	7.181
NIBAN1	8.574	8.474	8.648	8.234	8.507	8.353
NIBAN2	10.703	10.948	11.051	10.868	10.839	10.815
NIBAN3	7.212	7.339	7.279	7.379	7.346	7.280
GPN3	9.731	9.854	9.929	9.695	9.728	9.808
GPN2	9.016	9.047	9.116	8.909	8.959	8.880
GPN1	10.063	10.083	10.065	10.050	10.090	10.011
NFKB1	9.845	9.844	10.043	10.293	10.219	10.033
NFKB2	8.545	8.463	8.552	8.442	8.685	8.533
DNPEP	10.781	10.864	10.930	10.747	10.716	10.736
RAB40AL	6.107	6.246	6.359	6.217	6.082	6.220
SIAH1	10.456	10.416	10.566	10.335	10.368	10.284
SIAH2	10.219	10.345	10.402	10.205	10.280	10.217
SIAH3	4.057	4.290	4.029	4.052	4.087	4.170
NSA2	11.970	12.017	12.172	12.019	11.858	11.933
AC131206.1	8.380	8.341	8.491	7.936	8.281	7.917
PDZRN4	4.235	4.530	4.445	4.405	4.379	4.365
DDX27	11.100	11.069	11.204	10.901	10.844	10.950
PHLPP1	8.998	9.002	9.198	8.975	8.960	9.117
PDZRN3	5.129	5.171	5.095	5.147	5.181	5.323
EMILIN3	8.551	8.534	8.616	8.455	8.617	8.596
EMILIN2	8.643	8.756	8.905	8.594	8.603	8.679
EMILIN1	6.759	6.816	6.960	7.381	6.874	6.879
MIR924HG	5.554	5.819	5.660	5.163	5.538	5.429
AL133477.2	6.985	6.686	7.009	7.197	7.102	7.111
ZNF155	5.308	5.203	4.758	5.379	5.359	5.179
DDX12P	10.054	10.138	10.088	9.791	9.917	9.889
TWSG1	8.558	8.561	8.650	8.305	8.482	8.527
P2RX1	7.552	7.350	7.475	7.555	7.545	7.594
P2RX3	6.300	5.751	6.019	5.810	6.113	6.320
P2RX2	8.197	8.361	8.343	8.463	8.417	8.369
P2RX5	8.510	8.442	8.495	8.458	8.378	8.489
P2RX4	10.528	10.632	10.706	10.639	10.582	10.441
P2RX7	5.731	5.873	5.747	5.810	6.007	6.008
P2RX6	8.359	8.326	8.351	8.179	8.358	8.319
AC112198.2	6.494	6.494	6.035	6.502	6.642	6.610
FAM27E3	8.253	8.221	8.282	8.628	8.425	8.403
DENND11	9.167	9.029	9.060	8.954	9.054	8.917
DENND10	9.433	9.625	9.751	9.441	9.458	9.192
RMDN2	5.687	5.980	5.944	5.981	5.968	5.918
RMDN3	9.703	9.679	9.733	9.560	9.527	9.515
RMDN1	8.424	8.524	8.584	8.118	8.355	8.310
LYNX1-SLURP2	8.594	8.658	8.599	8.625	8.724	8.666
DENND1C	7.849	7.859	7.494	8.178	7.841	8.063
DENND1B	9.590	9.725	9.748	9.528	9.621	9.697
DENND1A	8.594	8.714	8.857	8.604	8.519	8.625
DIS3	8.090	8.229	8.167	7.791	7.874	7.895
BAP1	9.248	9.384	9.397	8.936	9.107	9.186
SHARPIN	9.711	9.726	10.001	9.713	9.653	9.683
ZCCHC13	5.827	5.956	5.840	5.741	5.963	6.218
ERRFI1	10.435	10.303	10.430	10.411	10.389	10.450
ZCCHC10	7.862	7.901	7.880	7.633	7.707	7.793
ZCCHC17	9.960	9.868	10.069	9.759	9.897	9.741
ZCCHC14	9.382	9.420	9.379	9.299	9.257	9.372
ZCCHC18	6.466	6.467	6.457	6.456	6.365	6.578

CDRT4	7.664	7.761	7.932	7.617	7.538	7.768
CDRT1	7.433	7.584	7.533	7.694	7.652	7.656
ANKS4B	7.781	7.830	7.831	7.530	7.652	7.584
CR382287.1	7.972	7.912	8.183	8.328	8.259	8.126
UBE2J2	8.837	8.823	8.985	8.748	8.827	8.824
VPS35L	8.767	8.822	8.980	8.612	8.606	8.734
SLC45A4	9.000	9.068	8.968	8.820	8.695	8.717
PICART1	7.286	7.315	7.012	7.344	7.117	7.250
MYBL2	10.053	10.166	10.289	9.768	9.817	9.774
PTGR1	8.497	8.495	8.571	8.599	8.545	8.603
SLC45A3	10.005	10.168	10.187	10.200	10.233	10.128
SLC45A2	6.905	6.622	6.720	6.642	6.711	6.739
MTUS1	11.619	11.714	11.741	11.662	11.513	11.673
MTUS2	8.133	8.387	8.345	8.213	8.186	8.187
IFRD1	7.144	7.242	7.264	7.334	7.207	7.250
IFRD2	10.345	10.339	10.641	10.368	10.289	10.320
ALS2CL	9.311	9.402	9.335	9.506	9.302	9.542
PIK3IP1-AS1	5.211	5.337	5.422	5.553	5.454	5.421
VPS9D1-AS1	8.834	8.908	9.106	9.127	9.107	8.994
INTS4P1	4.969	5.286	5.084	4.833	5.173	5.127
TRAV5	5.523	5.409	5.610	5.724	5.715	5.426
ADARB2	7.431	7.250	7.394	7.443	7.574	7.555
AC007244.1	7.157	6.959	7.254	7.696	7.232	7.169
IMPAD1	10.830	10.785	10.713	10.656	10.458	10.534
SFTA2	10.942	11.008	11.121	10.829	10.900	10.869
FZD10	5.126	5.274	5.210	5.052	5.211	5.076
ADARB1	7.655	7.589	7.875	8.347	7.949	7.943
CHKA	10.542	10.555	10.694	10.621	10.596	10.536
CHKB	8.976	8.966	9.050	8.658	8.853	8.811
BMS1	8.702	8.858	8.866	8.856	8.826	8.647
AC009108.4	6.386	6.399	6.276	6.325	6.441	6.334
AC009108.5	5.919	5.724	6.123	6.286	5.880	6.208
AC239809.3	4.232	4.169	4.235	4.298	4.540	4.412
MRI1	8.817	8.835	8.803	8.856	8.757	8.833
MAPK1IP1L	12.249	12.243	12.291	11.952	12.237	11.799
DST	10.438	10.418	10.349	9.944	9.853	10.106
DSP	10.717	10.785	10.720	10.383	10.308	10.555
AC093726.2	7.187	7.149	7.041	7.226	7.252	7.134
AC093726.1	6.798	6.716	6.869	6.629	6.766	6.603
THBD	6.914	6.872	6.847	6.956	7.087	7.047
DSE	8.609	8.748	8.637	8.303	8.412	8.473
PDAP1	10.044	10.361	10.453	10.175	10.099	10.010
CENPBD1	9.337	9.379	9.358	9.170	9.192	9.232
AL136982.1	9.081	9.018	9.114	9.210	9.131	9.065
AL136982.4	9.081	9.018	9.114	9.210	9.131	9.065
AP001063.1	7.879	8.066	7.931	8.021	7.958	8.061
SNORD46	13.214	13.233	13.392	13.147	13.175	13.114
AC012379.2	7.005	6.877	6.789	6.903	6.827	6.928
PLA2G6	9.058	9.094	9.042	9.022	9.106	9.205
PARGP1	7.822	7.672	7.790	7.876	7.791	7.855
SLC22A23	8.671	8.645	8.613	8.362	8.614	8.631
AC090001.1	7.540	7.585	7.706	7.931	7.761	7.649
ELOCP28	5.517	5.621	5.540	5.611	5.389	5.682
SLC22A24	5.967	6.216	5.882	6.270	6.172	6.090
TBX2-AS1	7.167	7.236	7.323	7.323	7.222	7.276
RAB11B-AS1	6.696	6.571	6.888	6.952	6.552	6.659

ZNF83	3.864	3.804	3.872	3.879	3.842	4.124
AUH	8.257	8.142	8.338	7.837	7.894	8.078
ZNF81	7.369	7.493	7.308	7.310	7.430	7.379
ZNF80	3.578	3.697	3.828	3.795	3.833	3.764
ZNF85	6.497	6.675	6.506	6.622	6.733	6.809
ZNF84	8.405	8.490	8.377	8.170	8.170	8.249
AC099786.3	4.087	3.832	3.861	4.060	4.004	3.916
AC099786.1	5.987	6.013	5.920	6.255	6.040	6.253
CAPZA3	4.616	4.584	4.537	4.823	4.552	4.499
CAPZA2	10.909	11.091	11.079	11.102	11.191	11.182
CAPZA1	11.486	11.531	11.591	11.480	11.447	11.484
SCARF2	7.049	7.251	7.518	7.383	7.335	7.314
SCARF1	7.096	7.189	7.214	7.335	7.297	7.363
SKA2P1	8.112	8.108	8.264	7.802	7.785	7.796
RPL7AP10	8.960	9.146	9.342	9.021	8.953	9.004
TRPC4AP	9.682	9.678	9.854	9.690	9.634	9.695
KRTAP1-1	8.149	8.313	8.371	8.208	8.495	8.254
C10orf88	8.475	8.474	8.476	8.606	8.339	8.509
CER1	7.163	7.218	7.103	7.247	7.275	7.382
C10orf82	7.127	7.072	7.134	7.385	7.380	7.531
DOCK2	5.809	5.885	5.913	5.823	5.869	5.919
ZNF793-AS1	4.879	4.895	4.820	4.901	5.045	4.922
GRIN3A	7.167	7.366	7.118	7.283	7.310	7.429
GRIN3B	7.296	7.514	7.450	7.324	7.518	7.503
MSL2	9.411	9.378	9.570	9.562	9.599	9.487
DOCK3	5.662	5.500	5.758	5.647	5.478	5.749
AC092757.2	5.401	5.491	5.410	5.318	5.339	5.514
AC104417.2	7.192	7.197	7.115	6.998	6.831	7.073
NCF4-AS1	7.187	7.263	7.184	7.245	7.351	7.327
AL135901.1	8.771	8.749	8.537	8.470	8.629	8.783
CCDC78	5.868	5.503	5.578	5.495	5.629	5.642
AP001198.1	5.459	5.433	5.302	5.974	5.456	5.433
MIR6829	6.201	6.238	6.269	6.136	6.076	6.245
MIR6821	10.410	10.494	10.697	10.742	10.641	10.625
CERK	9.222	9.203	9.251	9.039	8.919	9.250
RGCC	5.944	6.320	6.417	5.990	6.158	6.020
AL606760.1	6.877	6.892	6.846	6.609	6.779	6.537
LIN7A	5.263	5.308	5.213	5.463	5.562	5.357
LIN7C	10.572	10.565	10.654	10.390	10.441	10.531
LIN7B	7.713	7.915	7.967	8.098	8.036	8.068
IMMP2L	8.271	8.481	8.544	8.225	8.412	8.510
MOSMO	10.172	10.266	10.323	10.207	10.272	10.064
LINC02475	5.500	5.469	5.742	5.559	5.684	5.486
LINC02478	6.884	6.840	6.813	6.873	6.891	6.932
POFUT2	8.878	8.784	8.643	8.816	9.018	8.860
FP565260.1	8.752	8.742	8.867	8.463	8.537	8.502
FP565260.2	10.399	10.354	10.596	10.369	10.330	10.402
FP565260.3	8.562	8.689	8.776	8.901	9.049	8.853
FP565260.4	6.492	6.705	6.462	6.459	6.519	6.734
FP565260.6	7.743	7.885	7.833	7.766	7.702	7.720
MTCO2P12	14.445	14.476	14.457	14.504	14.599	14.502
AC069503.2	10.783	10.818	11.070	10.578	10.571	10.708
AC069503.1	8.094	8.241	8.280	7.811	8.001	8.007
AC016734.1	11.793	11.889	12.020	11.857	11.790	11.760
NFATC1	8.188	8.216	8.306	8.581	8.289	8.312
NFATC2	8.919	8.712	8.712	8.931	8.392	8.966

RPL37A	14.454	14.486	14.543	14.493	14.459	14.496
NFATC4	8.305	8.403	8.406	8.281	8.305	8.316
TNFAIP8L2	7.800	7.603	7.665	7.838	7.836	7.782
TNFAIP8L1	7.633	7.647	7.550	7.361	7.602	7.448
AP2B1	11.576	11.535	11.602	11.461	11.278	11.436
AC006030.1	9.556	9.577	9.639	9.377	9.310	9.253
NANOGP8	5.840	5.952	5.690	5.962	6.031	5.876
AC110619.1	5.511	5.248	5.090	5.795	5.501	5.273
CYP26B1	7.126	7.251	7.216	7.182	7.155	7.224
AL845331.3	8.464	8.364	8.135	8.347	8.305	8.294
AL845331.2	8.108	8.089	8.007	7.989	8.099	8.301
RRBP1	9.359	9.406	9.467	9.196	9.185	9.317
PPP1R14B	10.594	10.616	10.868	10.408	10.337	10.381
PPP1R14C	8.070	7.895	8.037	8.046	7.919	7.951
PPP1R14A	4.975	5.253	5.105	5.417	5.196	5.204
SSC5D	7.634	7.712	7.666	7.949	7.785	7.540
LINC02192	4.593	4.622	4.630	4.675	4.450	4.648
SRRT	10.572	10.678	10.639	10.348	10.324	10.449
AC092723.2	7.786	7.961	7.876	7.849	7.978	7.970
PCDH12	7.837	7.741	7.942	7.857	7.816	7.801
PCDH10	6.317	6.478	6.209	6.386	6.440	6.427
PCDH17	6.900	6.993	6.960	6.878	7.011	6.991
ALKBH8	7.610	7.592	7.563	7.580	7.570	7.576
PCDH15	4.272	4.283	4.241	4.392	4.448	4.268
ALKBH6	9.328	9.212	9.293	8.868	8.802	8.967
ALKBH7	9.842	9.922	10.213	9.995	9.962	9.893
PCDH18	7.382	7.518	7.346	7.400	7.304	7.398
PCDH19	6.165	6.202	5.787	5.999	6.037	6.011
ALKBH2	9.552	9.675	9.785	9.518	9.622	9.526
ALKBH3	8.792	8.826	9.027	8.558	8.701	8.581
GARS1	12.818	12.813	12.871	12.476	12.633	12.409
MIR29C	7.181	7.193	7.221	7.143	6.856	7.309
AL392172.1	7.283	7.312	7.548	7.375	7.401	7.375
PVR	10.079	10.293	10.315	10.043	10.085	10.110
AC025279.1	4.600	4.598	4.396	4.581	4.518	4.361
RNY4P34	5.441	5.577	5.280	5.362	5.380	5.177
KISS1	6.468	6.580	6.591	6.826	6.728	6.816
TMED7-TICAM2	9.261	9.374	9.195	9.258	9.302	9.297
THAP3	7.792	7.629	7.951	7.836	7.822	7.916
OTUD4P1	7.316	7.082	7.160	7.330	7.384	7.245
COL17A1	7.458	7.601	7.328	7.771	7.583	7.461
LIME1	7.705	7.946	8.090	7.997	7.924	7.980
BISPR	7.690	7.794	7.603	7.825	7.889	7.701
FAM219B	9.336	9.308	9.479	9.049	9.060	9.148
FAM219A	9.410	9.164	9.065	8.968	9.141	9.104
PMCHL2	7.343	7.418	7.447	7.293	7.719	7.459
GNL3LP1	6.570	6.571	6.604	6.695	6.676	6.500
PMCHL1	7.343	7.418	7.447	7.293	7.719	7.459
AP002991.1	8.806	8.828	8.546	8.848	8.827	8.808
AC012409.1	6.964	6.994	6.810	6.977	6.977	6.929
DHH	6.394	6.049	6.220	6.276	6.551	6.459
AC112497.2	5.260	5.190	5.141	5.314	5.186	5.519
CDK20	8.501	8.479	8.524	8.393	8.487	8.438
AC215522.2	6.282	6.406	6.358	6.633	6.414	6.078
NRIP1	10.105	10.226	10.350	9.988	10.139	9.828
NRIP2	6.898	6.953	6.856	7.072	7.087	6.837

NRIP3	7.577	7.599	7.611	7.684	7.824	7.786
VTI1A	8.043	8.093	8.177	8.079	8.212	8.041
VTI1B	10.497	10.557	10.733	10.617	10.537	10.607
CDKN2AIP	8.529	8.560	8.636	8.539	8.641	8.292
CHSY3	7.054	7.180	7.121	7.221	7.226	7.152
CHSY1	9.382	9.367	9.461	9.122	9.169	9.305
OCIAD2	11.847	11.878	12.069	11.921	11.839	11.910
OCIAD1	11.411	11.475	11.555	11.574	11.507	11.507
AC024588.1	4.738	4.929	4.802	5.150	4.938	5.049
ALKBH3-AS1	6.739	6.803	6.615	6.593	6.801	6.974
TRMU	8.213	8.159	8.347	8.312	8.246	8.308
DOCK4-AS1	5.278	4.997	5.115	5.454	5.578	5.229
SH3GLB1	10.642	10.800	10.858	10.798	10.796	10.795
SH3GLB2	8.776	9.011	9.216	9.019	8.871	8.765
AL035696.4	5.835	5.673	5.749	5.459	5.571	5.655
CHERP	10.557	10.587	10.618	10.263	10.239	10.396
AL035696.3	6.183	6.307	6.219	6.206	6.209	6.317
CR759960.3	9.231	9.374	9.436	9.462	9.438	9.339
ZNF619	7.897	7.851	7.891	7.701	8.175	7.956
TRMO	8.624	8.348	8.593	8.598	8.606	8.378
TCL6	8.581	8.356	8.455	8.561	8.549	8.682
RNU6-1306P	6.384	6.587	6.585	6.980	6.788	6.758
IGKV1-13	8.513	8.519	8.493	8.474	8.731	8.605
SLCO4A1-AS1	7.971	8.069	8.018	7.859	8.108	8.010
CHI3L2	7.406	7.107	7.074	7.351	7.403	7.476
HELQ	7.516	7.594	7.481	7.404	7.618	7.543
RCBTB1	7.325	7.422	7.286	6.910	6.745	7.236
MAP1A	8.072	8.279	8.167	8.141	8.228	8.241
HELZ	9.650	9.688	9.791	9.597	9.659	9.604
RN7SL731P	7.566	7.643	7.755	7.509	7.626	7.477
MYADM	12.679	12.810	12.855	12.706	12.564	12.705
RBAK	8.675	8.722	8.949	8.587	8.619	8.669
SMG1P6	8.386	8.425	8.147	7.850	8.127	8.311
SMG1P7	8.386	8.425	8.147	7.850	8.127	8.311
SMG1P4	8.386	8.425	8.147	7.850	8.127	8.311
SMG1P5	10.644	10.766	10.844	10.772	10.875	10.874
SMG1P2	8.660	8.768	8.817	8.906	8.925	8.853
SMG1P3	8.386	8.425	8.147	7.850	8.127	8.311
SMG1P1	8.386	8.425	8.147	7.850	8.127	8.311
TEKT1	6.884	6.811	6.869	6.775	6.864	6.963
TEKT3	4.833	5.039	5.025	4.972	5.106	4.971
TEKT2	6.678	6.748	6.790	6.580	6.755	6.852
TEKT5	6.586	6.639	6.558	6.557	6.688	6.807
ODAPH	5.307	5.156	5.251	5.316	5.339	5.139
CLUH	9.651	9.626	9.751	9.530	9.384	9.529
HMCES	9.577	9.699	9.748	9.439	9.427	9.390
FOS	8.425	8.392	8.460	9.811	9.739	9.875
KIAA0753	8.640	8.624	8.701	8.577	8.418	8.672
AC136285.1	7.007	6.903	6.417	6.855	6.902	6.952
SAFB2	9.145	9.020	9.195	8.732	8.637	8.958
SEC14L3	6.844	6.846	6.971	6.961	6.926	6.904
SEC14L2	7.446	7.569	7.504	7.465	7.448	7.572
SEC14L1	10.665	10.503	10.650	10.733	10.601	10.578
SEC14L5	7.153	7.369	7.109	6.899	7.219	7.128
SEC14L4	7.247	7.263	7.348	7.507	7.324	7.353
UPF1	9.325	9.345	9.584	9.305	9.372	9.358

UPF2	8.468	8.389	8.502	8.290	8.046	8.243
SNED1	8.191	7.848	7.757	8.181	8.155	8.137
IL1A	6.270	5.964	6.137	7.237	7.278	7.449
IL1B	8.337	8.396	8.468	8.391	8.512	8.570
KRT6B	7.370	7.683	7.509	7.554	7.428	7.561
KRT6A	8.028	7.976	7.969	8.424	8.291	8.229
COMP	5.676	5.973	5.997	6.511	5.985	6.034
AL160313.2	6.549	6.750	6.854	6.991	6.771	6.836
COMT	11.965	12.038	12.258	11.942	11.779	11.801
ZNF154	5.701	5.786	5.748	5.855	5.815	5.922
BACE1-AS	8.384	8.412	8.198	8.401	8.418	8.480
AL353608.3	6.037	5.681	5.593	5.953	5.932	5.915
IL18	8.922	8.998	8.991	8.675	8.660	8.782
IL19	7.323	7.508	7.429	7.387	7.706	7.537
IL10	5.150	5.394	5.326	5.184	5.252	5.161
IL11	8.739	8.691	8.781	8.873	8.910	8.831
IL13	7.590	7.202	7.535	7.834	7.800	7.438
IL15	6.854	7.332	6.987	6.925	7.356	7.346
IL16	8.191	7.960	7.926	8.326	8.327	8.282
AC008494.3	4.760	4.737	4.939	5.118	4.743	4.907
EFHD2	9.344	9.419	9.525	9.385	9.475	9.202
EFHD1	8.279	8.272	7.980	8.321	8.381	8.204
PTCD3	9.576	9.637	9.672	9.585	9.451	9.553
PINX1	7.865	8.024	8.212	8.089	7.945	7.944
CADM3-AS1	7.382	7.515	7.235	7.393	7.447	7.432
AEBP1	7.975	7.751	7.808	7.700	7.888	7.812
AEBP2	9.536	9.571	9.463	9.175	9.324	9.291
LTA4H	11.990	11.976	12.245	11.905	11.788	11.836
AC104035.1	4.415	4.213	4.246	4.244	4.153	4.256
HMGN3-AS1	5.880	5.962	5.701	5.795	5.822	5.896
LRRTM4-AS1	5.477	5.674	5.604	5.487	5.781	5.731
DOCK9-DT	7.153	6.946	7.113	7.196	7.256	7.156
ABHD12B	4.123	4.217	4.156	4.253	4.098	4.305
ZDHHC20	11.818	11.778	11.786	11.456	11.206	11.398
ZDHHC21	7.366	7.528	7.503	7.312	7.225	7.428
ZDHHC22	7.510	7.482	7.830	7.695	7.847	7.670
ZDHHC23	7.698	7.583	7.637	7.522	7.210	7.547
ZDHHC24	9.876	9.952	10.129	9.952	9.917	9.931
BPY2B	6.451	6.630	6.565	6.511	6.789	6.752
BPY2C	6.451	6.630	6.565	6.511	6.789	6.752
MED15P9	6.668	6.616	6.630	6.487	6.686	6.616
AL109925.1	5.727	5.877	5.651	5.716	6.026	5.813
ISL1	8.222	8.096	8.184	7.957	8.133	8.098
AL109925.3	5.727	5.877	5.651	5.716	6.026	5.813
AL109925.2	5.727	5.877	5.651	5.716	6.026	5.813
AL109925.5	5.727	5.877	5.651	5.716	6.026	5.813
AL109925.4	5.727	5.877	5.651	5.716	6.026	5.813
AL109925.6	5.727	5.877	5.651	5.716	6.026	5.813
MROH9	4.301	4.267	4.419	4.369	4.586	4.467
MROH8	7.454	7.728	7.556	7.484	7.467	7.748
MS4A8	8.180	8.322	8.280	8.262	8.385	8.307
MS4A7	7.835	8.038	7.874	7.897	8.078	8.243
MROH7	6.896	6.734	6.897	6.710	7.060	7.018
MROH6	10.713	10.764	10.926	10.783	10.711	10.628
MROH1	9.105	9.247	9.413	9.111	8.988	9.156
MS4A3	4.701	4.584	4.821	4.834	4.762	4.724

MS4A1	6.976	6.915	6.663	6.895	7.005	7.093
ZNF141	5.230	5.272	5.188	5.359	5.389	5.445
ZNF140	7.883	7.997	8.082	8.137	8.192	8.053
ZNF143	7.864	8.120	7.989	7.661	7.728	7.879
ZNF142	8.378	8.528	8.560	8.406	8.547	8.587
DNTT	6.476	6.436	6.451	6.449	6.296	6.565
ZNF146	10.596	10.711	10.753	10.342	10.392	10.554
ZNF148	10.361	10.272	10.360	10.218	10.449	10.063
DSC2	9.154	9.217	9.237	8.969	9.023	9.168
DSC3	7.519	7.506	7.692	7.217	7.527	7.486
DSC1	4.900	5.025	5.112	4.964	4.952	4.989
MAGOH	9.550	9.699	9.784	9.349	9.477	9.473
DDX3X	11.946	12.026	12.076	11.971	11.699	12.016
C20orf27	9.066	9.042	9.278	8.835	9.020	8.987
VWA8	8.876	8.696	8.774	8.610	8.766	8.770
SOD1	12.310	12.344	12.527	12.351	12.329	12.298
VWA7	7.701	7.845	7.823	7.815	7.798	7.603
RANGAP1	10.687	10.799	10.919	10.652	10.731	10.532
VWA1	9.190	9.276	9.520	9.492	9.463	9.278
KCNN4	10.595	10.696	11.036	10.766	10.779	10.694
KCNN3	7.431	7.202	7.238	7.799	7.557	7.399
KCNN2	4.649	4.683	4.652	4.738	4.711	4.700
KCNN1	8.598	8.602	8.567	8.776	8.638	8.837
DSG2-AS1	8.142	8.073	8.082	8.034	8.174	7.982
AC006449.7	7.679	7.706	8.093	7.994	8.021	7.990
AL096870.4	10.032	10.162	10.219	9.727	9.880	9.921
AC006449.5	6.353	6.422	6.407	6.554	6.293	6.358
AL096870.2	10.032	10.162	10.219	9.727	9.880	9.921
ISLR	6.777	7.157	6.842	6.721	7.076	6.863
PKIG	8.222	8.293	8.402	7.967	8.094	8.157
PKIA	7.061	6.913	6.687	6.912	6.671	6.765
PKIB	10.469	10.583	10.608	10.532	10.249	10.547
UBAC2-AS1	6.714	6.756	6.473	6.777	6.671	6.899
NIT2	9.537	9.536	9.738	9.651	9.584	9.607
NIT1	10.152	10.253	10.420	10.217	10.119	10.140
HCFC1R1	10.352	10.424	10.678	10.395	10.438	10.292
MIR4775	8.380	8.420	8.416	8.119	8.073	8.155
TYSND1	8.025	8.119	8.188	8.116	8.050	8.099
INO80	9.811	9.716	9.767	9.771	9.810	9.868
VDAC1P8	5.676	5.599	5.995	5.901	5.916	5.724
CLEC18C	5.972	6.568	6.142	6.006	6.214	6.333
ZNF667-AS1	5.912	5.840	5.969	6.328	5.894	5.861
KANSL1L	7.275	7.403	7.356	7.055	7.296	6.923
AC003958.2	4.781	4.804	4.706	4.736	4.730	4.974
AC003958.5	4.781	4.804	4.706	4.736	4.730	4.974
FZD4-DT	6.698	6.896	6.791	6.893	6.846	6.840
AC244517.11	5.173	4.976	4.681	4.855	5.304	4.728
CYHR1	9.801	9.830	9.970	9.665	9.745	9.684
TCEANC2	8.531	8.634	8.628	8.703	8.536	8.804
HLA-F-AS1	9.575	9.758	9.859	9.538	9.695	10.000
CR1L	6.026	6.177	6.117	6.536	6.391	6.154
NUP153	10.068	10.131	10.209	9.835	9.763	9.883
AC040169.1	6.571	6.672	6.937	6.558	6.573	6.522
OR2F1	7.229	7.437	7.459	7.271	7.201	7.160
NUP155	8.273	8.486	8.470	7.924	8.063	8.204
REX1BD	9.806	9.907	10.186	9.742	9.688	9.774

SORCS1	6.745	6.568	6.605	7.019	6.835	6.785
SORCS2	6.094	6.208	6.104	6.028	6.235	6.321
SORCS3	5.513	5.574	5.269	5.746	5.929	5.685
CARS2	7.935	7.962	8.049	7.964	7.880	8.129
CARS1	11.335	11.293	11.391	11.066	10.994	10.999
GPKOW	9.469	9.516	9.692	9.315	9.185	9.221
AC078925.3	6.091	6.155	6.068	5.972	6.194	6.125
NANOG	7.397	7.265	7.419	7.443	7.355	7.209
AC078925.4	6.953	6.959	6.936	6.948	7.008	6.918
RRH	6.493	6.828	6.670	6.948	6.842	6.845
MAPK3	10.778	10.837	10.995	10.817	10.821	10.752
AC104581.4	8.531	8.730	8.607	8.369	8.581	8.509
MAPK6	10.815	10.912	10.868	10.881	10.789	10.833
BCO1	7.278	7.292	7.306	7.118	7.216	7.345
BCO2	6.844	6.736	6.639	6.438	6.782	6.758
MAPK8	8.931	8.842	8.714	8.574	8.593	8.588
MAPK9	9.628	9.748	9.640	9.044	9.545	9.423
CUZD1	6.385	6.784	6.656	6.591	6.656	6.762
LYL1	7.905	7.785	7.904	8.167	8.003	7.900
AL360295.1	5.801	5.847	5.862	6.189	6.050	5.948
BCOR	8.554	8.675	8.604	8.342	8.340	8.580
AC002524.1	11.697	11.741	11.783	11.421	11.391	11.509
VWA3B	6.886	7.047	6.801	7.035	7.025	7.042
THNSL2	7.331	7.383	7.471	7.196	7.509	7.439
THNSL1	7.543	7.659	7.633	7.496	7.556	7.696
VWA3A	10.518	10.165	9.611	10.445	10.541	10.383
CCL11	6.688	6.399	6.249	6.846	6.666	6.757
CCL13	7.038	7.079	7.083	6.953	7.232	6.971
CCL14	7.686	7.980	7.709	8.014	8.015	7.939
CCL15	5.162	5.050	4.985	4.875	5.162	5.123
GPBP1L1	10.358	10.367	10.586	10.344	10.276	10.244
CCL17	6.893	6.823	6.846	7.107	7.187	6.971
CCL18	7.227	7.269	7.493	7.505	7.423	7.493
CCL19	7.677	7.752	7.617	7.775	7.952	7.803
AC114781.1	6.291	6.094	6.385	6.685	6.255	6.126
LINC01981	4.605	4.587	4.636	4.756	4.742	4.732
LINC01986	4.601	4.599	4.505	4.683	4.632	4.571
LINC01988	8.791	8.883	9.194	9.079	8.939	9.046
RAC1P2	12.898	12.736	12.938	12.928	12.718	12.858
AL359396.2	5.245	5.046	4.928	5.276	5.106	5.068
PLEKHN1	7.889	7.866	8.056	8.101	7.899	7.925
KCNT1	7.447	7.608	7.475	7.813	7.761	7.644
SERPINA2	5.615	5.794	6.013	6.019	5.923	6.039
SERPINA3	13.952	14.012	14.082	13.968	13.892	13.975
SERPINA4	8.753	8.718	8.917	8.782	8.851	8.849
SERPINA5	9.280	9.250	9.497	9.220	9.208	9.282
SERPINA6	8.972	9.001	9.061	8.742	8.738	8.797
SERPINA7	6.609	6.769	6.320	6.618	6.525	6.408
SERPINA9	8.492	8.523	8.417	8.509	8.539	8.633
ZC3HC1	9.159	9.217	9.330	9.050	9.117	9.044
FOXRED2	9.330	9.507	9.544	9.311	9.555	9.208
FOXRED1	9.048	9.139	9.268	8.833	8.819	8.808
KCNT2	4.948	5.149	5.045	4.906	4.960	4.993
AC122134.1	4.791	4.747	4.866	4.850	4.795	4.610
SNORA32	7.030	6.890	6.650	6.616	6.727	6.999
SNORA31	14.237	14.309	14.306	14.348	14.379	14.439

SNORA37	6.243	6.223	6.310	6.605	6.127	6.214
KIDINS220	9.553	9.602	9.532	9.284	9.183	9.244
KCNMA1-AS1	8.302	8.381	8.284	8.304	8.179	8.403
GAGE10	5.775	5.709	5.645	5.667	5.597	5.710
GAGE13	7.444	7.371	7.139	7.545	7.560	7.476
SYNPO	8.706	8.501	8.703	8.849	8.899	8.740
CTC1	8.482	8.449	8.461	8.384	8.281	8.547
ANP32D	5.923	5.998	5.863	6.421	6.235	5.946
ANP32E	11.042	11.023	11.036	10.750	10.652	10.751
ANP32B	12.309	12.403	12.536	12.213	12.129	12.132
ANP32C	7.491	7.564	7.560	7.627	7.725	7.661
ANP32A	12.581	12.548	12.601	12.335	12.335	12.308
SPATA45	5.264	5.213	5.272	5.674	5.295	5.324
SPATA46	7.592	7.630	7.561	7.930	8.037	7.971
HPSE2	7.930	7.728	7.731	8.109	8.045	7.792
SPATA41	7.149	7.375	6.881	7.260	7.644	7.306
SPATA42	4.484	4.657	4.439	4.601	4.425	4.540
TAT	6.663	6.829	6.873	6.934	6.796	6.987
SLC43A3	10.723	10.797	10.846	10.610	10.503	10.527
ALMS1	8.533	8.637	8.444	8.369	8.382	8.540
TAZ	8.817	8.770	8.802	8.717	8.838	8.827
SLC43A2	8.224	8.055	8.425	8.478	8.298	8.337
ATRNL1	7.331	7.518	7.317	7.337	7.324	7.341
PRIM1	9.850	9.813	9.928	9.516	9.501	9.485
SLC43A1	8.842	8.870	8.860	8.419	8.640	8.567
PRIM2	8.034	8.129	8.110	7.831	7.767	7.879
ALOXE3	7.513	7.578	7.575	7.581	7.609	7.757
MUC19	5.790	6.032	5.923	5.906	5.919	6.078
MUC17	6.043	6.140	5.869	5.732	6.119	5.868
MUC16	5.568	5.625	5.554	5.683	5.895	5.597
MUC15	4.384	4.372	4.103	4.399	4.427	4.502
MUC13	9.686	9.819	9.704	9.712	9.701	9.732
MUC12	7.022	6.966	7.215	7.162	6.898	7.070
SYNE3	7.424	7.272	7.204	7.459	7.520	7.375
MBTD1	8.937	8.989	9.042	8.810	8.785	8.931
SYAP1	11.093	11.162	11.338	10.913	10.966	10.875
MROCKI	4.980	4.791	4.763	4.864	4.828	4.947
CTCF	10.003	9.993	10.101	9.803	9.681	9.905
RASGRP2	6.996	6.889	7.338	7.516	6.933	7.185
RASGRP3	6.981	7.010	6.714	7.023	7.252	7.058
RASGRP1	7.024	7.024	7.273	7.312	7.099	6.933
HMGN1P36	11.925	11.994	12.146	11.893	11.885	11.984
RASGRP4	6.205	6.426	6.568	6.458	6.503	6.446
LRRN3	6.378	6.643	6.132	6.613	6.617	6.599
WNT6	6.906	7.022	7.060	7.218	7.168	7.026
LIPE	8.738	8.973	9.308	9.281	9.214	8.982
AC108463.3	3.578	3.535	3.656	3.505	3.604	3.506
COX7B2	5.484	5.689	5.267	5.552	5.462	5.558
AP002784.2	12.178	12.366	12.490	12.429	12.479	12.468
AP002784.1	8.750	8.828	8.740	8.569	8.650	8.747
AC020922.1	7.662	7.571	7.855	7.878	7.689	7.786
TWNK	8.174	8.319	8.356	8.126	8.192	7.931
HCK	6.647	6.535	6.605	6.845	6.729	6.677
C7orf25	8.659	8.600	8.753	8.630	8.646	8.652
C7orf26	9.874	9.991	10.208	9.833	10.013	9.967
ZNF426	8.529	8.458	8.515	8.472	8.408	8.426

ZNF425	6.039	6.119	6.103	6.113	6.135	5.917
ZNF423	5.213	5.539	5.447	5.253	5.684	5.441
ZNF420	8.138	8.094	8.197	8.067	8.085	7.862
ZNF428	9.084	9.167	9.343	9.176	9.194	8.996
ZNF429	6.981	7.024	7.110	7.007	6.997	7.099
FO082796.1	7.159	6.862	6.635	6.551	6.694	6.987
FO082796.2	7.745	7.729	7.361	7.729	7.847	7.903
CCDC189	7.872	7.918	7.795	7.687	7.845	7.916
CCDC181	6.391	6.483	6.437	6.541	6.588	6.428
LINC01511	6.995	7.002	6.972	6.742	6.956	7.083
CCDC182	7.644	7.660	7.789	7.847	7.813	7.770
LINC01514	4.346	4.285	4.154	4.429	4.396	4.469
CCDC184	6.860	6.972	6.847	6.925	6.909	6.900
LINC01516	5.862	5.841	5.684	5.856	5.618	5.932
LINC01517	4.247	4.163	4.139	4.187	4.098	4.364
NEDD8	11.477	11.617	11.773	11.593	11.533	11.636
NEDD9	8.301	8.137	8.231	8.431	8.318	8.297
P2RY14	3.802	3.737	3.733	3.750	3.752	3.724
NEDD1	9.548	9.369	9.359	9.094	9.141	9.287
NEDD4	9.211	9.147	9.121	9.091	8.981	9.077
P2RY11	7.156	7.365	7.517	7.520	7.316	7.344
P2RY12	4.991	5.175	4.819	5.244	4.930	5.013
P2RY13	6.718	6.832	6.493	6.708	6.846	6.704
GARNL3	6.947	6.719	6.633	6.832	6.997	6.727
AC092683.1	7.457	7.393	7.354	7.151	7.137	7.249
SFR1	7.958	7.959	7.885	7.603	7.675	7.713
AC244090.1	10.825	10.879	10.996	10.822	10.870	10.919
AC244090.3	6.493	6.302	6.206	6.594	6.671	6.542
HEBP2	10.840	10.809	11.011	10.641	10.724	10.693
LMOD1	7.378	7.455	7.337	7.571	7.706	7.618
LMOD3	5.506	5.238	5.302	5.521	5.571	5.421
LMOD2	5.767	5.717	5.791	6.269	5.868	5.959
AP005230.1	3.914	3.998	3.795	3.930	3.989	4.039
AC116407.2	6.264	6.299	6.308	5.962	6.508	6.348
GPRC5D-AS1	5.208	4.981	4.857	5.276	5.184	5.271
FAM13A-AS1	7.209	7.302	7.173	7.514	7.490	7.402
AL357558.2	4.961	4.943	4.924	5.232	5.279	4.800
LINC00396	5.645	5.627	5.729	5.578	5.756	5.707
SLX1A-SULT1A3	10.684	10.799	10.996	10.631	10.700	10.565
AC061975.1	9.142	8.967	8.658	9.008	9.180	9.462
AL096828.2	7.419	7.209	7.181	7.370	7.440	7.336
AC024051.12	7.882	7.717	7.633	8.115	8.061	7.935
AC024051.10	7.882	7.717	7.633	8.115	8.061	7.935
AC024051.11	7.882	7.717	7.633	8.115	8.061	7.935
AC009091.1	7.623	7.706	7.631	7.631	7.621	7.671
AC010145.1	6.927	7.173	6.767	7.206	7.132	7.080
AGXT2	7.602	7.739	7.319	7.777	7.698	7.635
DAB2	9.457	9.631	9.560	9.350	9.494	9.242
DAB1	8.933	9.048	9.081	9.041	9.000	9.093
RBMY1D	6.498	6.612	6.640	6.865	6.762	6.723
C1RL-AS1	8.432	8.170	8.046	8.357	8.524	8.454
AADACP1	5.274	5.253	5.080	5.235	5.071	5.235
RAB5A	11.084	11.081	11.125	10.988	11.044	11.056
RAB28	8.379	8.313	8.606	7.880	8.249	7.758
RAB5C	12.003	12.049	12.313	12.138	12.070	11.920
RAB5B	9.948	9.937	10.100	9.822	9.846	9.848

AL135818.2	6.473	6.418	6.642	6.507	6.847	6.831
RAB29	10.077	9.944	10.083	9.614	9.546	9.708
AC068888.1	7.852	7.880	7.969	8.048	7.955	7.955
TCL1B	7.671	7.817	7.671	7.423	7.636	7.802
AL355987.4	4.362	4.422	4.630	4.378	4.457	4.536
WNT7A	8.024	7.946	7.948	8.029	7.956	7.846
AL355987.1	7.711	7.708	7.771	7.725	7.823	7.681
AC244250.4	7.431	7.554	7.493	7.569	7.741	7.624
EIF2S3B	10.719	10.666	10.597	9.405	9.673	10.408
FAHD2CP	9.302	9.481	9.533	8.971	9.256	9.222
OSCAR	5.623	5.608	5.407	5.683	5.748	5.677
CSRNP1	8.880	9.109	9.107	9.489	9.590	9.474
CSRNP2	8.125	8.107	8.027	7.922	8.191	8.153
CSRNP3	7.198	7.035	7.015	7.455	7.468	7.354
COX6C	12.072	12.103	12.204	11.978	11.942	11.972
CR753509.1	6.517	6.543	6.395	6.499	6.649	6.716
AL132822.1	7.590	7.487	7.235	7.585	7.687	7.688
SHISA9	7.049	6.509	6.600	6.834	7.122	6.897
BUB1	8.851	8.779	8.837	8.220	8.506	8.310
BUB3	10.930	10.963	11.042	10.707	10.742	10.768
NUPR1	7.879	7.925	8.012	7.875	7.979	7.988
NASP	10.591	10.553	10.647	10.209	10.120	10.224
TMEM130	6.642	6.524	6.549	6.609	6.708	6.532
TMEM131	9.064	9.138	9.130	8.835	8.720	8.921
TMEM134	9.441	9.484	9.701	9.219	9.362	9.397
TMEM135	9.129	9.204	9.236	8.829	8.660	8.806
TMEM138	9.159	9.222	9.258	9.128	9.176	9.135
TMEM139	10.227	10.246	10.309	10.142	9.999	10.169
FOXE1	8.135	7.894	8.255	7.662	7.972	7.979
FOXE3	6.065	6.080	5.967	5.929	5.989	5.958
CT45A10	5.629	5.540	5.552	5.723	5.686	5.660
SLC35C2	9.613	9.790	9.865	9.659	9.851	9.620
SLC35C1	10.124	10.069	10.156	9.989	10.020	10.004
XK	9.763	9.879	9.944	9.559	9.730	9.508
GNRHR2	6.365	6.372	6.516	6.392	6.493	6.497
XG	5.465	5.445	5.218	5.660	5.464	5.715
C11orf88	6.034	5.748	5.746	5.822	5.823	5.790
C11orf86	8.189	8.377	8.467	8.666	8.531	8.396
C11orf87	4.186	4.329	4.363	4.225	4.208	4.174
C11orf80	7.960	8.115	8.260	7.805	7.733	7.958
PCAT7	7.993	7.884	7.891	8.138	8.142	7.892
MUCL1	7.521	7.729	7.326	7.646	7.824	7.832
MUCL3	6.740	6.804	6.770	6.763	6.802	6.677
AC100861.1	7.766	7.793	7.568	7.444	7.639	7.685
AC068896.3	6.938	6.986	7.095	6.919	6.876	7.164
CRHR2	6.760	6.853	6.758	6.899	6.885	6.745
CRHR1	7.826	7.845	8.021	7.935	7.905	7.824
AC099677.3	5.957	6.181	6.028	6.078	6.243	6.123
AC099677.1	5.112	5.060	5.091	5.135	5.155	5.190
AP001011.1	6.652	6.621	6.491	6.501	6.701	6.676
C1D	9.358	9.532	9.441	9.302	9.362	9.478
SCGB1D1	4.680	5.010	4.663	4.875	4.728	4.900
AC011474.1	7.914	8.033	7.815	7.996	8.159	8.092
SCGB1D2	5.664	5.633	5.574	5.759	6.000	5.829
CDYL	9.129	9.035	9.096	8.993	9.003	9.031
AC104823.1	5.071	5.085	4.819	5.326	5.088	5.130

HCLS1	5.826	5.896	5.531	5.957	5.858	5.791
TCP10L	6.759	6.677	6.662	6.850	6.903	6.860
TBC1D3P2	10.803	10.689	10.641	10.716	10.587	10.876
KRI1	8.214	8.036	8.079	8.144	7.822	8.109
TECPR1	8.707	8.699	8.857	8.668	8.830	8.616
TECPR2	8.272	8.434	8.508	8.285	8.296	8.277
CDY1	7.045	7.164	6.836	7.174	7.246	7.298
COQ5	9.845	9.804	10.030	9.776	9.740	9.737
COQ4	8.750	8.926	8.957	8.429	8.554	8.681
AL008721.2	5.814	5.547	5.647	5.476	5.539	5.587
AC010655.3	4.806	4.827	4.624	4.715	4.847	4.648
AC010655.2	4.806	4.827	4.624	4.715	4.847	4.648
FCHO2	9.989	10.096	10.160	10.109	10.045	10.026
FCHO1	7.327	7.493	7.195	7.382	7.349	7.343
AC091152.2	4.748	4.752	4.701	5.080	4.728	4.862
AC091152.4	6.274	6.066	6.255	6.014	6.133	6.344
HCG4	5.097	5.198	5.203	5.259	5.348	5.414
DYNLL1P3	6.845	6.969	6.913	6.786	7.041	7.146
HCG9	7.210	7.374	7.374	7.625	7.425	7.297
AUP1	9.848	10.016	10.184	9.857	9.736	9.825
AC134980.3	5.651	5.789	5.544	5.480	5.658	5.651
AC134980.2	5.651	5.789	5.544	5.480	5.658	5.651
AP001596.2	7.821	7.889	7.642	7.799	7.899	7.919
PNPO	10.795	10.878	11.019	10.630	10.655	10.579
C8orf17	6.044	6.134	6.290	6.506	6.242	5.991
APBB1IP	8.202	8.051	8.182	8.196	7.936	8.041
SKI	9.454	9.670	9.736	9.452	9.583	9.426
C16orf89	6.740	6.632	6.744	6.579	6.695	6.921
LIMCH1	9.674	9.533	9.504	9.374	9.272	9.457
C16orf82	7.287	7.338	7.326	7.611	7.582	7.465
CLDND1	8.795	8.881	8.988	9.053	9.129	9.222
C16orf87	8.078	8.219	8.205	7.955	8.230	8.033
CLDND2	6.676	6.543	6.609	6.941	6.649	6.827
ZFAS1	12.314	12.430	12.617	12.598	12.565	12.582
GEM	7.349	7.613	7.528	8.404	8.126	8.075
RIC8B	9.310	9.395	9.341	9.185	9.052	9.271
RIC8A	10.931	10.889	10.919	10.849	10.756	10.858
H2AC8	6.482	6.829	6.884	7.068	6.980	7.196
H2AC7	7.447	7.364	7.916	7.833	7.685	7.781
H2AC6	9.610	9.683	9.890	9.935	9.886	9.802
BAHCC1	8.449	8.691	8.357	8.346	8.685	8.527
H2AC4	7.140	7.002	7.132	6.937	7.130	7.224
AC036176.1	6.122	6.287	6.268	6.716	6.534	6.287
PCDHGA9	11.092	11.102	11.188	11.070	11.043	11.048
NSMCE1-DT	4.962	4.991	5.046	5.067	5.127	4.968
PCDHGA2	11.092	11.102	11.188	11.070	11.043	11.048
PCDHGA3	11.092	11.102	11.188	11.070	11.043	11.048
PCDHGA1	11.092	11.102	11.188	11.070	11.043	11.048
PCDHGA6	11.092	11.102	11.188	11.070	11.043	11.048
PCDHGA7	11.092	11.102	11.188	11.070	11.043	11.048
PCDHGA4	11.092	11.102	11.188	11.070	11.043	11.048
PCDHGA5	11.092	11.102	11.188	11.070	11.043	11.048
AC004847.1	4.272	4.127	4.115	4.325	4.175	4.391
PHF8	8.253	8.176	8.180	8.094	7.980	8.113
PHF6	10.447	10.590	10.677	10.275	10.188	10.276
PHF7	7.508	7.684	7.642	7.594	7.737	7.778

PHF2	7.930	8.048	7.915	7.698	7.767	7.902
RABAC1	11.546	11.648	11.839	11.561	11.487	11.680
PHF1	9.536	9.652	9.717	9.516	9.554	9.582
C2CD4A	9.631	9.750	9.737	9.288	9.391	9.462
C2CD4B	8.459	8.298	8.527	8.486	8.479	8.454
C2CD4C	8.060	7.941	8.092	8.153	8.141	8.108
SH2D3C	6.306	6.371	6.362	6.299	6.495	6.420
AC024293.1	13.767	13.833	13.946	13.964	13.837	13.963
AC006372.4	6.308	6.023	6.082	6.280	6.176	6.487
AC006372.2	7.591	7.629	7.816	7.983	7.807	7.636
AC006372.3	6.759	6.791	6.806	6.924	7.016	6.906
RPL29P7	7.637	7.801	7.719	7.517	7.398	7.549
VARS2	8.614	8.670	8.743	8.558	8.568	8.605
VARS1	9.336	9.443	9.487	9.478	9.537	9.379
CCDC162P	5.407	5.439	5.121	5.384	5.373	5.234
RPS26	12.821	12.916	13.024	12.923	12.903	12.978
RPS27	13.947	14.022	14.018	14.057	14.036	14.057
RPS24	13.710	13.749	13.758	13.838	13.747	13.824
RPS25	12.267	12.428	12.443	12.421	12.337	12.473
RPS23	13.870	13.918	13.938	13.940	13.916	13.923
RPS20	13.772	13.812	13.879	13.778	13.633	13.771
RPS21	13.585	13.583	13.742	13.792	13.714	13.898
RPS28	13.528	13.573	13.649	13.437	13.393	13.460
RPS29	13.736	13.753	13.842	13.881	13.899	13.910
OR1E2	7.021	7.117	6.877	7.210	7.052	7.072
OR1E3	7.263	7.350	7.366	7.373	7.392	7.376
OR1E1	7.021	7.117	6.963	7.210	7.052	7.112
CHMP1B	10.825	10.762	10.820	10.714	10.709	10.736
CHMP1A	10.474	10.521	10.696	10.328	10.241	10.327
PREB	9.761	9.730	9.877	9.597	9.756	9.737
SNRPCP8	8.196	8.262	8.287	8.740	8.301	8.304
GOLGA6L5P	7.449	6.960	6.903	6.742	6.653	7.251
PREP	10.293	10.261	10.385	10.326	10.295	10.340
SNRPCP5	8.196	8.262	8.287	8.740	8.301	8.304
CU467612.1	4.623	4.658	4.664	4.620	4.660	4.680
AL390038.1	4.272	4.199	4.364	4.408	4.339	4.313
ITPK1-AS1	4.651	4.224	4.219	4.544	4.721	4.647
MIEN1	14.104	14.124	14.156	14.134	14.120	14.107
AC114271.1	6.686	6.640	6.760	6.360	6.648	6.691
AC110792.2	3.831	3.924	3.773	3.894	3.641	3.954
AC001226.2	7.374	7.613	7.484	7.130	7.420	7.291
NBAS	8.925	8.887	8.969	8.844	8.783	8.796
LBX1-AS1	6.933	6.745	6.945	6.892	6.876	6.883
SEC22B2P	8.653	8.456	8.743	8.348	8.393	8.317
NIF3L1	10.069	10.156	10.314	10.024	10.038	10.103
IGKV1OR10-1	8.398	8.520	8.424	8.478	8.571	8.522
AC007877.1	7.040	7.198	7.053	7.387	7.040	6.817
TMBIM4	12.030	12.141	12.216	12.162	12.212	11.977
TMBIM1	11.961	12.158	12.302	12.043	11.968	12.034
U62317.2	7.631	7.829	7.828	7.652	7.946	7.759
AC023908.3	4.742	4.830	4.912	4.938	4.964	4.934
U62317.1	7.120	6.999	7.162	7.149	7.250	7.250
DRD4	6.767	6.600	6.872	6.717	6.931	6.742
DRD5	7.498	7.416	7.253	7.443	7.632	7.492
DRD2	7.920	8.149	7.993	7.948	7.939	7.994
DRD3	6.366	6.385	6.179	6.271	6.334	6.372

DRD1	5.407	5.201	4.918	6.077	5.693	5.922
AC117457.1	7.345	7.615	7.284	7.523	7.656	7.553
CAMP	7.926	8.187	8.286	8.374	8.308	8.299
MGMT	7.923	8.155	8.361	7.788	7.834	7.956
SLC25A3P1	6.972	6.844	6.773	7.221	6.922	6.967
GLG1	11.847	11.796	11.846	11.796	11.979	11.331
BX088651.4	8.597	8.585	8.594	8.520	8.633	8.531
BX088651.2	4.141	4.060	4.251	4.180	4.213	4.069
TTC9C	8.875	8.816	8.968	8.830	8.941	8.908
TTC9B	7.984	7.939	8.107	8.258	8.205	8.229
NOSIP	9.675	9.726	9.995	9.374	9.181	9.463
ST7-AS1	6.288	6.197	6.185	6.126	6.242	6.377
ST7-AS2	5.266	5.122	5.176	5.224	5.232	5.096
IGHV3-30-2	6.356	6.471	6.499	6.503	6.500	6.426
AL157756.1	7.229	7.396	7.235	7.304	7.397	7.241
ENTPD7	8.295	8.259	8.482	8.219	8.340	8.370
GOLGA8IP	7.048	6.865	6.859	7.005	7.107	7.121
ENTPD5	8.228	8.439	8.223	8.093	8.010	8.062
ENTPD4	10.205	10.091	10.178	9.837	9.654	9.762
ENTPD3	8.983	8.999	9.000	8.877	8.751	8.779
ENTPD2	7.595	7.821	8.003	7.827	7.792	7.891
ENTPD1	7.492	7.345	7.428	7.475	7.502	7.484
DLEC1	8.253	8.277	8.313	8.359	8.295	8.281
SNAPC2	8.568	8.413	8.674	8.912	8.678	8.577
SNAPC1	8.648	8.997	9.250	9.278	9.274	8.853
SNAPC5	10.696	10.750	10.956	10.656	10.830	10.644
SNAPC4	9.402	9.340	9.389	9.344	9.426	9.369
RSPH10B	9.485	9.466	9.613	9.501	9.346	9.296
XIAP	10.861	10.889	10.788	10.603	10.606	10.766
RN7SL818P	6.210	6.139	6.179	6.290	6.315	6.341
HAGLROS	6.850	6.668	6.826	6.711	6.779	6.806
HESX1	6.689	6.977	6.686	6.801	6.796	6.726
AL049714.1	5.495	5.712	5.526	5.603	5.504	5.788
ZFP14	4.897	4.878	4.883	4.809	4.807	4.919
CAGE1	4.288	4.173	4.122	4.195	4.138	4.369
PROCR	7.759	7.667	7.920	7.519	7.638	7.513
ODF3	8.065	8.013	7.876	8.193	8.299	8.217
ODF2	7.798	7.976	7.960	7.564	7.604	7.616
ODF1	7.377	7.336	6.977	7.376	7.497	7.393
RNF8	8.057	8.123	8.073	7.896	7.998	7.957
ODF4	8.409	8.121	8.298	8.309	8.396	8.371
LINC00845	6.902	7.040	6.787	6.893	7.257	7.074
LINC00844	4.480	4.366	4.237	4.375	4.434	4.246
LINC00847	8.157	8.292	8.522	8.152	8.138	8.245
LINC00841	8.365	8.397	8.108	8.381	8.391	8.387
LINC00840	4.351	4.330	4.497	4.487	4.485	4.376
LINC00842	6.585	6.629	6.487	6.536	6.519	6.678
KIF3B	10.972	11.168	11.213	10.954	11.042	10.814
KIF3C	9.264	9.503	9.374	9.265	9.287	9.343
KIF3A	8.392	8.300	8.268	8.219	8.182	8.249
YAE1	9.669	9.759	9.904	9.870	9.916	9.894
RNF2	7.582	7.459	7.625	7.764	7.798	7.495
TMEFF2	4.875	4.930	4.983	5.181	4.883	4.939
FAM186A	4.607	4.853	4.440	4.540	4.546	4.691
AC011477.8	7.683	7.698	7.630	7.582	7.862	7.801
CLMAT3	6.357	6.388	5.916	6.316	6.605	6.465

AC011477.1	4.561	4.689	4.762	4.750	4.852	4.546
STAMBP	9.740	9.835	9.944	9.819	9.959	9.836
RBBP4P4	5.629	5.708	5.820	6.267	6.046	6.135
MUTYH	9.324	9.250	9.263	9.165	9.112	9.078
AATBC	5.251	5.538	5.302	5.422	5.495	5.217
SUCNR1	6.601	6.589	6.432	6.442	6.429	6.556
FAM209B	7.007	6.985	6.953	6.899	6.983	7.079
TBXA2R	8.468	8.681	8.790	8.511	8.634	8.530
SEL1L	10.489	10.497	10.594	10.443	10.536	10.462
CGB8	7.556	7.653	7.596	8.049	7.638	7.796
CGB5	7.556	7.653	7.596	8.049	7.638	7.796
CGB7	7.556	7.653	7.596	8.049	7.638	7.796
AL353704.1	6.629	6.767	6.421	6.662	6.833	6.744
PDSS2	8.365	8.466	8.520	8.154	8.290	8.290
CGB2	7.556	7.653	7.596	8.049	7.638	7.796
CGB3	7.556	7.653	7.596	8.049	7.638	7.796
RBMY2AP	5.549	5.417	5.468	5.650	5.551	5.647
KIAA2013	10.412	10.613	10.670	10.443	10.511	10.502
KIAA2012	8.229	8.270	8.283	8.336	8.376	8.260
CNTLN	6.854	7.035	6.887	7.144	7.042	7.073
CENPVL2	5.912	6.029	6.150	5.979	5.780	5.978
TMEM191A	8.320	8.443	8.592	8.648	8.459	8.560
EMP2	10.484	10.477	10.614	10.251	10.219	10.240
EMP3	7.266	7.345	7.523	7.447	7.355	7.465
EMP1	8.589	8.850	8.770	8.490	8.543	8.679
TMSB4X	13.840	13.910	13.927	13.935	13.998	13.997
TMSB4Y	7.401	7.739	7.637	7.689	7.884	7.733
CCDC22	8.959	9.129	9.181	8.804	8.818	8.909
CCDC25	9.497	9.622	9.641	9.330	9.296	9.223
CCDC24	8.551	8.564	8.788	8.510	8.575	8.676
CCDC27	6.668	6.544	6.796	6.649	6.631	6.757
CCDC26	4.226	4.232	4.061	4.207	4.140	4.112
VHL	8.417	8.345	8.208	8.412	8.546	8.423
FIBIN	6.946	6.960	6.714	6.739	6.938	6.990
LINC02140	7.289	7.320	7.172	7.132	7.432	7.365
AC093627.7	7.823	7.854	7.896	7.871	7.945	7.743
LINC02145	6.531	6.678	6.562	6.840	6.445	6.756
CDKN3	9.898	9.794	10.022	9.627	9.750	9.627
LINC02148	4.211	4.351	4.296	4.424	4.255	4.248
LINC02149	4.205	4.053	3.983	4.273	4.209	4.323
UBE2G2	9.660	9.467	9.428	9.412	9.375	9.320
UBE2G1	10.138	10.243	10.283	9.977	10.065	10.042
KCTD20	8.461	8.661	8.609	8.632	8.627	8.562
KCTD21	7.610	7.560	7.710	7.665	7.425	7.629
FAM207BP	6.844	6.748	6.928	6.911	6.706	6.745
NEDD8-MDP1	8.338	8.289	8.624	8.297	8.232	8.353
CYP20A1	7.628	7.652	7.728	7.524	7.634	7.554
AL449043.1	5.236	4.952	5.099	5.354	5.191	5.427
RANBP3L	5.383	5.044	5.258	5.427	5.371	5.262
AMYP1	5.089	5.094	5.291	5.046	4.927	5.253
AL442224.1	7.107	7.232	7.150	6.907	7.176	7.122
AL353784.1	3.916	3.918	3.911	3.780	3.934	3.935
AL356488.3	6.899	6.897	6.762	6.890	6.834	6.846
AL356488.2	7.544	7.153	7.252	7.258	7.315	7.360
SGPL1	9.631	9.687	9.656	9.468	9.430	9.626
APBB1	7.551	7.675	7.951	7.957	7.944	7.796

APBB2	7.692	7.707	7.720	7.792	8.110	7.932
APBB3	8.294	8.178	8.220	8.425	8.415	8.182
LINC01494	5.622	5.706	5.677	5.641	5.785	5.841
LINC01490	5.051	4.924	4.855	4.868	5.034	4.735
LINC01492	3.859	3.912	4.093	4.135	4.298	4.203
UNKL	8.015	7.799	7.889	7.867	7.786	7.876
WDR6	9.118	9.137	9.304	9.032	9.000	9.018
WDR5	8.702	8.844	8.731	8.622	8.736	8.583
WDR4	8.357	8.247	8.250	8.083	8.268	8.260
WDR3	9.737	9.741	9.854	9.673	9.579	9.705
DIAPH1	11.051	11.140	11.105	10.765	10.488	10.739
DIAPH2	8.284	8.196	8.387	8.032	7.927	8.173
DIAPH3	8.717	8.677	8.829	8.449	8.580	8.518
SMR3B	6.978	6.939	6.787	7.186	7.182	7.100
SMR3A	5.535	5.799	5.596	5.629	5.743	5.745
IGLVIVOR22-2	6.000	6.023	6.010	5.884	6.273	6.346
IGLVIVOR22-1	6.384	6.470	6.266	6.430	6.342	6.413
COX6A1	13.007	13.124	13.178	13.155	13.129	13.142
COX6A2	7.394	7.294	7.289	7.228	7.623	7.413
POU2AF1	5.535	5.628	5.549	5.861	5.572	5.847
LINC01723	8.400	8.394	8.312	8.276	8.399	8.644
LINC01722	5.393	5.468	5.141	5.870	5.727	5.701
LINC01720	4.362	4.310	3.863	4.403	4.116	4.319
LINC01725	4.199	4.155	4.190	4.215	4.136	4.064
RNU6-1016P	6.990	6.911	6.948	6.614	7.017	6.872
AL357079.3	6.546	6.637	6.617	6.687	6.566	6.404
AL357079.1	7.368	7.333	7.287	7.347	7.426	7.383
XRRA1	7.443	7.446	7.561	7.529	7.519	7.457
NDNF	7.671	7.623	7.681	7.621	7.883	7.820
MAGEE2	4.751	4.939	4.831	5.029	4.899	4.981
MAGEE1	7.989	8.039	8.032	7.821	8.135	8.095
XIST	8.345	8.363	8.080	8.360	8.346	8.383
RAMAC	10.440	10.548	10.560	10.603	10.486	10.555
SNX31	4.570	4.630	4.618	4.574	4.462	4.626
SNX30	7.548	7.619	7.499	7.131	7.316	7.363
SNX33	9.192	9.278	9.365	9.220	9.259	9.236
SNX32	7.879	8.021	7.984	8.058	8.178	8.126
MAP3K5-AS1	6.682	6.494	6.494	6.767	6.899	6.881
IRGQ	9.799	9.831	9.899	9.672	9.605	9.689
CHRAC1	9.550	9.502	9.550	9.122	9.180	9.169
IRGC	6.044	5.963	5.872	6.149	5.964	6.352
IRGM	4.056	4.001	3.960	4.015	4.142	4.286
AC093890.1	8.011	8.142	8.053	7.556	7.811	7.950
APELA	4.092	4.155	4.014	4.026	4.216	4.198
AP000552.6	7.049	7.195	7.322	7.016	7.141	7.207
LINC00458	5.641	5.873	5.475	5.803	5.673	5.346
LINC00459	6.178	6.205	6.270	6.585	6.549	6.662
LINC00452	7.633	7.797	7.600	7.497	7.689	7.625
LINC00454	7.344	7.328	7.406	7.648	7.584	7.469
GABRR1	5.525	5.556	5.514	5.664	5.860	5.734
GABRR2	7.941	7.877	8.012	8.252	8.053	7.772
GABRR3	4.565	4.522	4.560	4.767	4.663	4.506
TRIM59	8.848	8.813	8.969	8.612	8.836	8.627
TRIM58	3.858	3.746	3.737	4.063	3.981	3.636
TRIM55	5.030	4.708	5.107	5.277	4.961	4.941
TRIM54	6.453	6.143	6.383	6.519	6.334	6.054

TRIM56	9.052	9.287	9.251	8.990	9.070	8.721
RNASET2	12.540	12.644	12.842	12.555	12.522	12.654
TRIM50	7.610	7.693	7.531	7.778	7.798	7.911
TRIM52	7.506	7.361	7.503	7.278	7.565	7.476
CCNDBP1	9.053	9.101	9.244	8.885	8.996	9.089
RNU6-125P	6.416	6.393	6.187	6.326	6.444	6.523
BX664727.3	8.236	8.156	8.141	8.146	8.143	8.231
NUS1	9.759	9.843	9.852	9.546	9.530	9.638
NREP	8.480	8.471	8.544	8.397	8.379	8.426
AC207056.1	5.941	5.958	5.887	5.879	5.992	6.096
MISP3	7.502	7.475	7.513	7.822	7.834	7.730
ATP6V1E1	10.934	11.054	11.154	10.973	10.845	10.925
ATP6V1E2	7.667	7.647	7.879	7.480	7.640	7.626
KRTCAP2	11.470	11.629	11.742	11.552	11.606	11.450
KRTCAP3	9.149	9.225	9.457	9.152	9.086	9.176
AC092296.2	5.564	5.850	5.755	5.988	5.720	5.794
AC090061.1	5.288	5.383	5.411	5.392	5.726	5.646
AC005343.4	8.022	8.031	8.090	8.120	8.248	8.408
AC005343.3	8.022	8.031	8.090	8.120	8.248	8.408
SUPT16HP1	11.009	10.992	11.063	10.776	10.776	10.826
SOX9-AS1	6.663	6.728	6.590	6.546	7.057	6.831
TRGV5P	6.394	6.578	6.473	6.655	6.610	6.714
MAB21L4	7.528	7.465	7.498	7.397	7.425	7.601
MYOG	8.056	7.974	7.883	8.431	8.158	8.167
GNG5P2	12.530	12.641	12.776	12.587	12.551	12.483
MAB21L1	5.543	5.550	5.166	5.442	5.327	5.721
MAB21L2	6.364	6.232	6.496	6.181	6.321	6.131
MAB21L3	4.011	4.145	4.180	4.096	4.147	4.139
HSD17B8	9.844	10.010	10.150	9.994	9.990	9.919
HMGCL	10.104	10.160	10.234	10.047	10.029	10.110
HSD17B1	6.072	5.927	6.070	5.879	6.213	6.033
HSD17B2	10.041	10.297	10.376	9.848	9.774	9.860
HSD17B3	7.097	7.302	6.939	7.346	7.425	7.316
HSD17B4	10.263	10.299	10.406	10.220	10.106	10.220
HSD17B6	6.217	6.172	5.945	6.478	6.457	6.340
HSD17B7	10.477	10.548	10.659	10.659	10.664	10.429
ALDH3B2	6.795	6.961	6.981	6.911	6.833	6.925
ALDH3B1	11.063	11.145	11.211	10.964	10.940	10.951
ZNF219	8.267	8.269	8.505	8.233	8.493	8.248
PARD3	9.893	9.761	9.891	9.815	9.924	9.738
KCNA2	7.047	6.896	7.128	7.574	7.135	7.051
PSKH2	7.303	7.402	7.325	7.490	7.138	7.418
PSKH1	9.489	9.510	9.736	9.232	9.452	9.476
IL1RL1	8.453	8.383	8.248	8.430	8.459	8.423
IL1RL2	7.039	7.191	7.035	7.305	7.343	7.121
AC126323.6	4.810	4.857	4.955	5.108	4.774	4.845
AC126323.2	4.806	5.033	5.169	4.888	4.922	4.999
STRIT1	3.853	3.857	3.835	3.930	3.887	3.855
PEBP1	11.892	12.097	12.223	12.083	12.078	11.861
PEBP4	6.324	6.443	6.629	6.561	6.443	6.565
CEP192	7.236	7.399	7.347	6.829	6.930	7.348
UTP14A	9.508	9.462	9.532	9.413	9.358	9.337
TNFRSF21	12.591	12.573	12.615	12.328	12.209	12.357
UTP14C	9.579	9.473	9.557	9.413	9.427	9.365
TNFRSF25	8.028	8.015	8.169	8.585	8.400	8.263
CNOT2	10.650	10.671	10.837	10.587	10.573	10.591

PROSER1	8.624	8.609	8.696	8.580	8.642	8.572
PROSER2	8.900	8.742	8.811	8.482	8.769	8.743
PROSER3	8.413	8.535	8.241	8.300	8.568	8.480
HTATIP2	11.268	11.368	11.406	11.235	11.159	11.229
AC098818.2	5.111	4.938	4.823	4.919	4.776	4.896
SLX4IP	7.878	7.859	7.824	7.887	7.667	7.737
MAMLD1	7.789	7.620	7.512	7.757	7.899	7.863
RNMT	9.188	9.189	9.395	9.042	8.971	9.162
HUS1B	5.234	5.151	5.093	5.373	5.351	5.229
AC092810.3	6.441	6.371	6.396	6.371	6.406	6.694
PPP1R12BP1	3.648	3.833	3.920	3.826	3.760	3.869
AC092155.1	6.503	6.293	6.487	6.329	6.225	6.345
SLC29A4	8.115	8.124	8.405	8.312	8.413	8.406
SLC29A3	8.549	8.600	8.399	8.632	8.641	8.608
SLC29A2	9.415	9.346	9.480	9.235	9.219	9.254
SLC29A1	9.429	9.351	9.532	9.258	9.291	9.163
RSC1A1	5.510	5.974	5.639	5.308	5.410	5.346
C6orf201	4.983	4.772	4.911	4.922	4.821	4.903
CFAP157	10.155	10.250	10.139	10.126	10.245	10.107
MRAS	7.979	8.089	7.889	7.957	8.141	8.230
MRAP	7.128	7.231	7.327	7.159	7.350	7.244
AC006504.7	4.819	4.827	4.588	4.829	4.813	4.739
AC006504.5	9.096	9.024	9.116	8.973	9.098	9.140
SARM1	7.642	7.729	7.741	7.861	7.908	7.804
YARS1	11.390	11.441	11.586	11.400	11.391	11.424
AL136226.1	11.288	11.357	11.417	11.362	11.258	11.308
RFC5	11.193	11.173	11.405	10.991	11.230	10.955
RFC4	10.524	10.696	10.762	10.560	10.501	10.561
HTN1	6.065	6.200	5.954	6.003	6.158	6.222
RFC1	8.816	8.866	8.790	8.369	8.169	8.547
RFC3	10.089	10.111	10.283	9.794	9.915	9.893
RFC2	9.054	9.049	9.174	8.409	8.404	8.640
CBX8	7.867	7.964	8.148	8.051	7.940	7.803
CBX7	8.827	8.808	9.075	8.518	8.602	8.592
CBX6	12.358	12.291	12.452	12.210	12.196	12.301
CBX5	11.998	12.085	12.167	11.808	11.989	11.692
CBX4	10.840	10.895	11.067	10.806	11.129	10.748
CBX3	12.582	12.656	12.675	12.555	12.407	12.614
CBX2	8.494	8.437	8.289	8.275	8.398	8.578
LRRC52-AS1	7.353	7.176	7.327	7.365	7.107	7.390
HNRNPA1P4	13.482	13.508	13.526	13.344	13.317	13.319
HNRNPA1P7	13.482	13.508	13.526	13.344	13.317	13.319
HNRNPA1P3	9.643	9.648	9.606	9.459	9.373	9.474
AL355312.6	9.802	9.947	9.902	9.645	9.688	9.718
AP002807.1	8.838	8.908	8.985	8.612	8.597	8.764
CLASRP	9.924	9.845	10.013	9.881	9.834	9.940
OFD1P5Y	5.534	5.482	5.365	5.619	5.244	5.157
GNAQ	11.223	11.319	11.299	10.999	11.198	11.018
CKS2	10.305	10.316	10.544	10.521	10.711	10.563
ACADSB	7.096	7.202	7.126	7.213	7.074	7.276
LINC00954	4.450	4.675	4.552	4.855	4.857	4.713
AL359555.2	3.864	4.065	4.026	4.068	4.172	4.039
AL359555.4	7.346	7.501	7.354	7.757	7.666	7.554
AC134312.5	4.383	4.410	4.282	4.238	4.458	4.442
AC134312.2	5.418	5.370	5.364	5.430	5.373	5.400
SHOC1	5.402	5.463	5.245	5.534	5.538	5.598

SHOC2	9.076	9.074	9.106	8.973	8.844	8.961
ERCC8	8.135	8.162	8.186	8.049	7.824	8.179
ERCC1	9.921	10.083	10.173	9.847	9.851	9.894
ERCC2	9.430	9.444	9.616	9.489	9.472	9.438
ERCC3	9.045	9.029	9.084	8.851	8.731	8.885
ERCC4	8.851	9.072	8.964	8.692	8.877	9.060
ERCC5	7.712	7.756	7.841	7.683	7.351	7.765
KDF1	8.353	8.501	8.615	8.326	8.343	8.179
MRPS17	10.629	10.703	10.894	10.627	10.639	10.605
MRPS16	9.647	9.643	9.902	9.559	9.514	9.524
MRPS15	12.090	12.127	12.206	12.286	12.202	12.226
MRPS14	10.417	10.468	10.665	10.458	10.400	10.272
MRPS12	11.000	11.209	11.411	11.118	11.115	10.818
MRPS11	10.595	10.535	10.741	10.483	10.496	10.481
MRPS10	9.771	9.829	9.803	9.875	10.147	9.751
CD248	7.928	7.718	7.884	7.942	8.016	7.990
TDRD10	7.606	7.947	7.677	7.715	7.663	7.860
PFKP	11.648	11.727	11.931	11.547	11.540	11.598
PFKM	9.784	9.860	9.888	9.776	9.601	9.696
PFKL	10.117	10.147	10.406	10.005	9.957	9.956
PRIMPOL	8.353	8.511	8.458	8.298	8.268	8.535
SNURF	7.598	7.489	7.410	7.452	7.482	7.398
THOP1	8.937	8.780	8.941	8.443	8.388	8.580
AL662884.4	7.823	7.886	7.823	8.160	7.880	7.696
CLEC4M	6.854	6.746	6.798	6.865	6.922	6.834
AL662884.1	8.289	8.473	8.563	8.593	8.450	8.206
AL662884.3	7.823	7.886	7.823	8.160	7.880	7.696
AL662884.2	7.442	7.707	7.796	7.792	7.633	7.179
CLEC4G	5.049	4.880	5.074	4.827	5.034	5.177
CLEC4F	7.013	7.084	7.024	6.924	7.005	7.019
CLEC4E	6.843	6.947	7.017	7.265	7.151	7.023
CLEC4D	7.262	7.246	7.130	7.423	7.335	7.551
CLEC4C	6.021	6.263	5.895	6.331	6.212	6.222
CLEC4A	6.030	6.122	6.269	6.311	6.370	6.339
SSR1	11.228	11.274	11.338	11.206	11.178	11.165
SSR2	11.603	11.617	11.792	11.623	11.563	11.683
SSR3	12.106	12.317	12.339	12.417	12.429	12.464
SSR4	13.075	13.139	13.204	13.147	13.040	13.159
CTTNBP2NL	8.729	8.796	8.772	8.386	8.449	8.426
CASP8AP2	8.304	8.450	8.331	7.946	7.721	8.027
APOC4	7.876	7.787	8.209	8.098	8.027	8.036
APOC2	8.568	8.617	8.592	8.694	8.621	8.673
APOC3	5.723	5.929	5.879	6.204	5.892	5.915
APOC1	8.667	8.545	8.805	8.806	8.698	8.691
XDH	8.515	8.752	8.443	8.729	8.663	8.790
USB1	9.682	9.758	9.733	9.656	9.652	9.503
AL020995.1	4.223	4.225	4.297	4.141	4.272	4.149
POU6F2	5.937	5.905	5.842	6.382	6.172	6.229
ZFPM2	5.274	5.080	5.099	5.285	5.201	5.220
POU6F1	7.220	7.093	7.115	7.232	7.335	7.265
KLHDC10	9.488	9.539	9.614	9.114	9.227	9.384
ZFPM1	8.726	8.846	9.026	8.981	9.008	9.052
AL121916.1	8.542	8.609	8.531	8.542	8.546	8.631
PPM1K-DT	7.172	6.963	6.913	7.136	7.225	7.290
AL590762.1	10.182	10.115	10.383	10.275	10.363	10.031
UBE2O	8.608	8.566	8.747	8.743	8.877	8.683

AC007318.1	11.560	11.653	11.787	11.596	11.450	11.551
AMYH02020865.1	8.464	8.364	8.135	8.347	8.305	8.294
FASTK	10.017	10.193	10.276	10.134	10.246	10.112
PAGE4	6.721	6.923	6.778	7.047	6.916	7.041
PAGE5	7.072	7.075	7.168	7.121	7.030	7.018
PAGE2	3.577	3.806	3.762	3.805	3.853	3.676
B9D2	7.930	7.994	8.101	8.033	8.185	7.997
B9D1	7.742	7.936	8.015	7.849	7.865	7.782
PAGE1	5.272	5.443	5.565	5.784	5.616	5.539
LINC01128	8.662	8.811	8.567	8.651	8.496	8.538
LINC01127	5.271	5.447	5.238	5.167	5.132	5.146
LINC01126	6.508	6.291	6.151	6.025	6.322	6.251
LINC01121	7.393	7.330	7.233	7.563	7.485	7.444
LINC01120	6.037	6.338	6.129	6.212	6.113	6.088
LINC01123	7.940	7.954	8.122	7.953	7.938	7.880
EGF	4.783	5.111	4.979	5.153	4.805	4.964
PGK2	6.487	6.384	6.670	6.535	6.772	6.756
PGK1	13.724	13.814	13.837	13.814	13.831	13.868
BECN1	11.271	11.185	11.261	10.866	10.791	10.862
AL590556.3	7.097	7.309	7.376	7.264	7.341	7.309
RPP25L	10.326	10.321	10.401	10.276	10.108	10.258
DEUP1	4.900	4.767	4.903	5.014	5.043	5.000
MIR4647	11.212	11.317	11.420	11.307	11.284	11.228
MIR4648	8.914	8.941	9.131	8.950	8.647	8.874
AC064836.3	7.876	7.851	7.525	7.784	7.938	7.694
FAAP100	8.674	8.621	8.849	8.464	8.514	8.551
OXA1L	11.324	11.334	11.481	11.129	11.065	11.132
SCRN3	8.652	8.848	8.753	8.423	8.469	8.444
C6orf52	7.903	7.731	7.904	7.875	7.728	7.776
DOCK10	4.990	4.910	4.841	4.969	4.961	4.876
DOCK11	5.807	5.719	5.526	5.705	5.736	5.784
C6orf58	7.385	7.572	7.242	7.811	7.763	7.624
LPAL2	6.211	6.206	6.281	6.678	6.384	6.229
LINC00298	4.266	4.169	4.030	4.264	4.279	4.240
LINC00299	6.427	6.382	6.502	6.415	6.505	6.549
AC226593.1	4.701	4.584	4.821	4.834	4.762	4.724
LINC00294	9.037	9.078	8.932	8.964	8.874	9.024
LINC00290	4.204	3.953	4.227	4.389	4.117	4.121
DPM2	9.059	9.171	9.357	9.075	8.994	9.045
DPM1	11.378	11.443	11.599	11.632	11.560	11.513
LINC00293	7.103	7.170	7.151	7.345	7.134	7.107
IGKV2D-28	6.483	6.636	6.681	6.760	6.476	6.601
RABGEF1	7.985	8.194	8.101	7.820	7.947	7.949
VN1R10P	5.634	5.629	5.281	5.657	5.656	5.851
IGKV1D-8	5.518	5.477	5.557	5.532	5.367	5.547
MIR210	8.775	8.730	9.028	8.559	8.528	8.875
CHURC1	9.735	9.762	9.793	9.623	9.693	9.539
MIR214	6.025	6.238	6.060	6.047	6.375	6.366
TBC1D9B	10.773	10.807	10.886	10.848	10.683	10.783
ALOX12B	6.271	6.053	6.365	6.520	6.179	6.291
AC013733.1	5.750	6.014	5.609	6.024	6.064	6.076
GU182347.5	8.835	8.689	8.488	8.744	8.793	8.879
CDH10	6.176	6.181	5.837	6.366	6.338	6.409
GU182347.7	7.241	7.134	7.110	7.315	7.321	7.394
CDH12	4.743	4.770	4.608	4.898	4.759	4.833
CDH15	7.431	7.533	7.445	7.523	7.513	7.503

CREG1	11.239	11.354	11.420	11.177	11.244	11.148
CREG2	6.212	6.496	6.241	6.393	6.399	6.622
CDH16	6.464	6.532	6.434	6.296	6.497	6.402
CDH19	7.172	7.396	6.968	7.733	7.539	7.521
CDH18	6.844	6.660	6.738	6.810	7.001	6.625
GU182347.9	8.835	8.689	8.488	8.744	8.793	8.879
AL117348.2	7.438	7.393	7.358	7.385	7.635	7.457
AL645922.1	10.634	10.645	10.647	10.363	10.369	10.287
AC012158.1	6.902	6.890	7.000	6.558	6.816	6.869
TCF25	11.491	11.544	11.672	11.276	11.182	11.330
NIPBL	9.103	9.011	9.063	9.016	8.853	8.832
MBP	11.889	11.889	12.079	11.794	12.104	11.393
AC022784.3	4.329	4.422	4.340	4.600	4.542	4.403
AC022784.1	8.020	8.145	8.297	8.088	8.003	8.078
NPBWR1	7.683	7.867	7.909	7.865	7.986	7.827
AC011979.1	12.617	12.677	12.802	12.784	12.769	12.841
MIR646HG	7.379	7.622	7.460	7.273	7.430	7.424
OR7E12P	9.849	9.926	10.029	9.810	9.880	9.911
TAF7L	5.314	5.358	5.385	5.332	5.417	5.405
OR4A1P	6.755	6.945	6.757	6.806	7.176	6.943
COMMD10	9.489	9.555	9.709	9.823	9.869	9.867
HTR1D	6.666	6.931	6.556	6.750	6.759	6.846
HTR1E	7.218	7.453	7.168	7.070	7.228	7.449
HTR1F	6.624	6.636	6.385	6.587	6.913	6.473
HTR1A	8.023	7.948	7.997	8.149	8.212	8.174
HTR1B	7.102	7.133	7.180	7.239	7.310	7.386
CKAP5	10.146	10.283	10.342	10.154	10.160	10.070
BCL2L1	12.251	12.409	12.448	12.273	12.298	12.256
BCL2L2	9.167	9.257	9.349	9.112	9.124	9.097
MIR3198-2	10.502	10.538	10.611	10.315	10.120	10.332
ATP23	7.751	7.920	8.093	7.883	7.908	7.762
AC142391.1	6.204	6.467	6.150	6.306	6.431	6.397
AC142391.2	6.204	6.467	6.150	6.306	6.431	6.397
AL359955.1	4.338	4.409	4.292	4.455	4.467	4.436
ITSN1	8.775	8.718	8.545	8.586	8.593	8.598
ITSN2	8.808	8.690	8.662	8.744	8.594	8.740
PRAF2	9.182	9.324	9.342	9.068	9.185	9.195
AL358113.1	11.164	11.127	11.175	10.908	10.875	10.870
NEGR1	6.034	6.212	6.265	5.940	6.083	6.185
FAM193A	8.705	8.674	8.670	8.706	8.695	8.695
FAM193B	8.707	8.735	8.688	8.712	8.501	8.868
TREH	5.540	5.572	5.704	5.562	5.637	5.720
AC090337.1	4.185	4.130	4.147	4.300	4.351	4.308
AC005306.1	7.930	7.863	7.950	8.258	7.842	7.883
AC016582.1	6.252	6.359	6.051	6.329	6.684	6.334
MEFV	7.867	7.925	8.029	8.170	8.303	8.226
AC016582.3	5.443	5.591	5.755	5.733	5.432	5.648
AC006449.11	7.679	7.706	8.093	7.994	8.021	7.990
AFAP1L1	7.642	7.574	7.677	7.594	7.790	7.620
AFAP1L2	5.989	5.831	5.686	6.167	5.932	5.977
PCMT1	10.255	10.339	10.532	10.371	10.300	10.410
AC018638.4	9.349	9.203	9.195	9.170	9.223	9.503
AC018638.5	10.722	10.564	10.478	10.430	10.307	10.610
SLAIN2	8.972	9.052	9.035	9.004	9.056	8.906
SLAIN1	5.625	6.121	6.375	5.472	5.576	5.684
CPA2	7.208	7.290	7.321	7.150	7.197	7.256

CPA3	7.187	7.186	7.069	7.258	7.324	7.448
CPA1	4.182	4.274	4.202	4.114	4.145	4.274
CPA6	4.329	4.514	4.395	4.433	4.372	4.468
CPA5	6.933	6.838	7.130	7.226	7.211	7.069
OSM	5.731	5.868	5.905	6.005	5.839	5.726
OS9	12.062	12.198	12.248	12.007	11.996	11.999
SH2D4A	9.530	9.517	9.516	9.568	9.701	9.625
SH2D4B	5.675	5.690	5.595	5.543	5.969	5.661
NPHS2	8.073	8.177	7.981	7.979	8.143	8.311
NPHS1	7.138	6.954	7.012	7.082	6.958	6.984
TMEM44-AS1	7.600	7.463	7.503	7.431	7.567	7.520
ARG1	6.040	5.656	5.608	6.050	5.948	6.012
ARG2	8.493	8.543	8.683	8.569	8.687	8.717
TMEM50B	9.451	9.539	9.611	9.646	9.566	9.546
TMEM50A	11.156	11.159	11.223	11.140	11.212	11.128
AP003068.3	5.707	5.729	6.102	5.963	6.031	5.965
ZNF355P	4.338	4.409	4.292	4.455	4.467	4.436
LINC02339	5.943	6.106	5.960	5.901	6.206	5.941
AC100839.1	7.031	7.227	7.189	7.156	7.165	7.308
GON4L	8.298	8.318	8.485	8.298	8.377	8.242
AC011389.3	5.855	5.944	6.037	5.748	6.043	5.870
ABL1	9.485	9.521	9.547	9.261	9.217	9.190
ABL2	9.553	9.634	9.635	9.728	9.592	9.540
SNX29P1	6.534	6.276	6.309	6.768	6.854	6.558
TMED6	5.707	5.633	5.687	5.605	5.660	5.720
IL9R	7.336	7.420	7.085	7.221	7.337	7.436
SNX29P2	6.534	6.276	6.309	6.768	6.854	6.558
TMED3	10.850	11.009	11.166	10.692	10.630	10.815
TMED2	12.821	12.940	13.010	13.041	12.950	12.981
TMED1	8.842	9.066	9.196	8.960	8.800	8.981
TMEM201	9.584	9.329	9.316	9.264	9.171	9.155
BAIAP2L2	9.184	9.145	9.384	9.152	9.083	8.970
BAIAP2L1	11.005	11.073	11.019	10.821	10.915	10.838
HNRNPU	12.288	12.388	12.454	12.238	12.203	12.156
TMED9	12.145	12.161	12.251	11.623	11.661	11.771
TMED8	9.331	9.355	9.434	9.009	9.095	9.148
FSIP2-AS2	3.914	3.951	3.597	3.827	3.769	3.882
HNRNPK	12.826	12.956	12.948	12.901	12.847	12.836
FASN	12.459	12.473	12.570	12.675	12.467	12.483
HNRNPM	11.440	11.466	11.441	11.080	10.922	11.120
HNRNPC	11.610	11.805	11.829	11.883	11.823	11.810
HNRNPD	12.186	12.246	12.295	11.927	11.772	11.953
HNRNPF	10.179	10.312	10.468	10.477	10.267	10.302
TMEM208	10.383	10.416	10.656	10.369	10.157	10.318
EGFEM1P	4.395	4.496	4.255	4.409	4.368	4.283
TMEM209	8.781	8.631	8.664	8.049	8.119	8.392
TNNI3K	5.802	5.688	5.584	6.198	5.891	5.874
PLGRKT	9.894	10.006	9.947	9.744	9.850	9.795
PIMREG	9.034	9.009	9.256	8.628	8.945	8.791
ANKRD33B	7.488	7.516	7.279	7.551	7.651	7.469
TADA1	7.397	7.687	7.719	7.507	7.541	7.581
TADA3	9.829	9.883	10.001	9.810	9.789	9.776
CD52	7.048	7.022	6.895	7.376	6.984	7.279
CD53	8.829	8.945	8.762	8.672	8.871	8.891
CD55	10.350	10.585	10.587	10.452	10.442	10.426
CD58	9.040	9.009	9.096	9.304	9.335	9.303

CD59	13.074	13.223	13.273	13.081	13.040	13.083
SRP54	10.013	9.978	9.975	9.958	9.787	9.892
AP000265.1	4.787	4.855	4.925	5.239	4.933	4.726
AC016065.1	6.618	6.645	6.920	6.658	6.921	6.753
AC140134.1	4.546	4.452	4.404	4.410	4.521	4.445
CCDC120	7.846	7.878	7.808	7.830	7.805	7.792
SPATA9	4.680	4.665	5.065	4.647	4.620	4.740
SPATA8	7.691	7.805	7.711	7.731	7.698	7.726
SRA1	9.457	9.447	9.662	9.493	9.387	9.384
SPATA3	6.681	6.865	6.801	7.016	6.831	6.891
SPATA2	8.977	8.945	9.073	9.113	9.186	9.075
SPATA1	3.865	3.957	3.874	3.703	3.924	4.042
SPATA6	6.955	7.107	6.943	6.865	6.882	6.777
CD5L	5.894	6.111	5.972	6.304	6.192	6.122
SPATA4	8.071	8.186	7.874	8.146	8.204	8.151
AC090498.1	14.596	14.604	14.644	14.671	14.651	14.656
LURAP1L-AS1	4.117	4.115	4.020	4.078	4.230	4.073
HKDC1	10.526	10.542	10.510	10.212	10.135	10.251
AC007009.1	8.123	8.172	8.247	8.058	8.163	8.043
AC026336.2	5.909	5.753	5.895	5.966	6.046	6.365
KCNF1	7.372	7.202	7.351	7.326	7.407	7.189
AC107909.1	6.612	6.704	6.334	6.366	6.693	6.491
AC126283.2	10.193	10.193	10.245	10.181	10.182	10.196
PTRH2	9.598	9.627	9.780	9.694	9.608	9.796
PTRH1	8.408	8.480	8.601	8.282	8.378	8.268
AC027290.1	9.319	9.452	9.388	8.886	9.031	9.277
CTBP1	11.016	10.973	11.127	10.720	10.656	10.818
CTBP2	11.703	11.772	11.933	11.732	11.874	11.467
MFSD11	8.570	8.598	8.792	8.616	8.583	8.471
MFSD10	9.476	9.449	9.729	9.447	9.458	9.517
GTF3C1	10.399	10.455	10.522	10.165	10.199	10.331
MFSD12	9.179	9.282	9.358	9.254	9.287	9.206
GTF3C6	10.952	11.038	11.036	10.953	10.934	10.876
GTF3C5	9.416	9.534	9.745	9.267	9.390	9.330
GTF3C4	8.986	8.951	8.889	8.778	8.832	8.815
CD99L2	8.641	8.747	8.694	8.341	8.490	8.423
LEMD1	6.366	6.160	6.231	6.111	6.193	6.104
AL391001.1	4.289	4.244	4.232	4.075	4.264	4.173
GAR1	9.046	8.956	9.280	8.858	9.039	8.935
EPS15L1	8.751	8.666	8.681	8.828	8.949	8.978
GART	9.732	9.705	9.809	9.847	9.672	9.755
ATAD5	7.501	7.465	7.443	7.643	7.454	7.445
TP53I13	8.402	8.504	8.668	8.468	8.512	8.316
ATAD1	9.868	9.889	9.993	9.728	9.750	9.827
ATAD2	10.361	10.340	10.308	9.786	9.659	9.919
TCIRG1	8.838	8.908	8.985	8.612	8.597	8.764
FRAT1	7.031	7.235	7.155	6.895	6.921	7.004
FRAT2	7.903	8.018	7.982	7.431	7.395	7.526
AC107464.3	7.572	7.460	7.242	7.590	7.541	7.352
SIGIRR	10.433	10.464	10.601	10.281	10.251	10.309
C22orf23	7.011	7.131	7.288	7.171	7.119	7.148
C22orf24	6.928	7.019	6.904	6.957	6.923	6.988
AC131056.2	5.971	5.771	6.090	5.970	6.216	5.995
SC5D	10.537	10.577	10.613	10.611	10.494	10.496
MTIF3	8.451	8.626	8.676	8.519	8.688	8.560
MTIF2	9.697	9.714	9.716	9.309	9.247	9.391

AC007382.1	7.829	7.861	7.717	7.441	7.658	7.881
ELMSAN1	10.521	10.488	10.552	10.168	10.232	10.309
AL391807.1	4.545	4.588	4.834	4.759	4.659	4.691
TUBB2B	11.493	11.695	11.838	11.596	11.479	11.392
NAPB	7.702	7.655	7.625	7.329	7.464	7.334
SCN11A	5.391	5.181	5.215	5.442	5.250	5.306
CALB2	11.516	11.527	11.652	11.384	11.443	11.450
CALB1	5.723	5.744	5.944	5.738	5.881	5.769
AKNAD1	5.517	5.564	5.611	5.706	5.606	5.621
GFER	8.362	8.490	8.470	8.491	8.748	8.359
AC083862.2	7.284	7.361	7.292	7.534	7.675	7.281
FAM20A	6.423	6.503	6.355	6.482	6.694	6.691
FAM20B	9.718	9.789	9.756	9.570	9.733	9.537
FAM20C	7.266	7.237	7.382	7.339	7.332	7.348
AGGF1	9.243	9.302	9.370	9.069	9.231	9.240
PYURF	10.573	10.546	10.713	10.423	10.508	10.524
AL021920.1	5.070	5.139	5.257	5.557	5.241	5.115
AL021920.3	6.719	6.805	6.764	6.834	6.732	6.855
EEA1	9.329	9.224	9.233	9.046	8.959	9.118
CA5AP1	7.250	7.199	7.397	7.262	7.320	7.248
AL353622.1	4.658	4.545	4.641	4.209	4.056	4.650
AL353622.4	4.658	4.545	4.641	4.209	4.056	4.650
FXN	7.576	7.701	7.650	7.295	7.442	7.323
GTF2IP13	8.218	8.224	8.224	8.266	8.245	8.273
NIPAL1	8.028	8.235	8.168	7.698	8.086	7.864
NIPAL2	9.059	9.153	8.975	9.045	9.115	9.175
NIPAL3	9.127	9.221	9.439	9.014	9.036	8.989
NIPAL4	8.575	8.494	8.589	8.566	8.766	8.494
YRDC	9.175	9.198	9.218	9.203	9.281	9.230
PRYP3	7.337	7.057	7.188	7.293	7.443	7.388
PRYP4	7.337	7.057	7.188	7.293	7.443	7.388
CHID1	9.245	9.388	9.618	9.237	9.175	9.171
EWSR1	11.503	11.695	11.653	11.066	11.213	11.240
AC018706.1	5.420	5.587	5.749	5.941	5.777	5.628
TNK2-AS1	6.742	6.761	6.592	6.517	6.437	6.621
AHCTF1	8.131	8.203	8.321	8.115	8.056	8.171
AC008966.1	5.756	5.826	5.961	5.446	5.835	5.579
AC008966.2	6.509	6.374	6.642	6.646	6.614	6.823
ARL2BP	10.407	10.443	10.573	10.598	10.597	10.405
SLC47A2	6.667	6.883	6.545	6.831	6.886	6.971
SLC47A1	6.430	6.524	6.256	6.059	6.515	6.020
SLC16A10	6.406	6.488	6.251	6.515	6.519	6.241
CHM	10.097	10.185	10.120	9.803	9.680	10.063
RINT1	6.185	6.212	6.178	6.119	6.395	6.236
SH3BGRL	11.102	11.152	11.200	10.881	10.877	10.957
AC136469.2	5.585	5.406	5.400	5.506	5.468	5.568
AC129507.8	6.888	6.846	6.941	7.070	7.063	7.099
AC129507.5	5.819	5.934	5.964	6.321	6.162	6.363
AC129507.6	5.819	5.934	5.964	6.321	6.162	6.363
AC129507.7	6.888	6.846	6.941	7.070	7.063	7.099
POM121L9P	7.431	7.554	7.493	7.569	7.741	7.624
AC129507.1	5.819	5.934	5.964	6.321	6.162	6.363
AC129507.2	6.888	6.846	6.941	7.070	7.063	7.099
AC129507.3	6.888	6.846	6.941	7.070	7.063	7.099
HMHB1	5.131	5.007	4.990	5.170	5.470	5.179
ZNRD2-AS1	6.479	6.328	5.977	6.337	6.467	6.417

SERINC5	10.167	10.099	10.140	9.858	9.916	9.978
SERINC1	11.148	11.297	11.250	11.122	11.060	11.234
SERINC3	12.841	12.943	13.131	12.879	12.920	12.525
SERINC2	9.832	9.900	9.991	9.943	9.899	9.886
HPX	9.126	9.160	8.918	9.048	9.162	9.122
PINK1	9.069	9.178	9.286	9.150	9.116	9.223
MLH1	10.315	10.273	10.485	10.063	10.051	10.130
PIN1P1	6.553	6.804	6.834	6.975	6.904	6.786
PKDREJ	7.421	7.362	7.448	7.596	7.517	7.505
CAMTA2	8.903	8.935	8.895	8.615	8.722	8.791
CAMTA1	10.308	10.296	10.503	10.196	10.211	10.240
KXD1	9.778	9.766	9.889	9.728	9.703	9.697
SDF2L1	9.447	9.464	9.683	9.419	9.413	9.384
NPFFR2	6.534	6.176	6.045	6.915	6.702	6.528
NPFFR1	7.258	7.249	7.359	7.320	7.638	7.449
AL078590.2	6.754	7.033	6.989	7.001	6.772	6.930
ABTB1	8.934	9.116	9.187	8.756	8.910	8.942
ABTB2	9.351	9.428	9.537	9.571	9.560	9.607
AL591623.1	6.801	6.547	6.558	6.668	6.735	6.794
CIPC	7.943	8.143	8.138	7.618	7.442	7.761
C2CD4D-AS1	8.084	8.104	8.251	8.106	8.156	8.080
AC016571.1	8.175	8.149	8.135	8.252	8.292	8.345
RPS6KB1	8.628	8.569	8.689	8.195	8.076	8.482
TRADD	9.719	9.871	9.953	9.730	9.712	9.772
RPS6KB2	8.932	9.012	9.295	9.006	8.883	8.864
FAM177B	5.952	6.121	5.854	5.934	6.004	5.882
WWTR1-AS1	6.563	6.835	6.252	6.793	6.371	6.994
AC127070.4	6.882	6.824	6.839	6.953	6.814	6.829
BUD13	7.969	7.994	8.224	7.779	7.977	7.789
LINC02642	6.222	6.210	6.028	6.289	6.267	6.331
ISY1	9.971	9.972	10.120	9.685	9.926	9.680
F2RL2	4.595	4.660	4.741	4.798	4.962	4.864
F2RL1	12.502	12.626	12.602	12.454	12.484	12.429
XAB2	7.837	7.686	8.186	7.917	8.383	7.732
ST6GAL2	4.726	4.869	4.670	4.722	4.998	4.993
ST6GAL1	8.661	8.557	8.895	8.475	8.501	8.524
LINC00643	4.924	4.830	4.916	4.762	4.942	5.024
LINC00642	8.937	8.912	8.968	9.062	8.860	8.962
LINC00641	6.698	6.805	6.851	6.495	6.842	7.009
AL133499.1	6.450	6.217	6.525	6.821	7.187	6.714
LINC00644	4.204	4.312	4.467	4.519	4.386	4.407
LINC00648	4.904	4.836	4.733	4.915	5.077	5.031
OTUB1	11.302	11.362	11.511	11.228	11.297	11.259
FERD3L	7.465	7.720	7.625	7.597	7.805	7.630
OTUB2	8.618	8.649	8.631	8.364	8.533	8.573
ZNF663P	5.228	5.259	5.254	5.053	5.316	5.395
NPTN	12.915	12.980	13.019	12.980	12.905	13.004
ZNF252P	8.911	8.999	8.881	8.837	8.842	8.857
SULT2A1	5.501	5.525	5.494	5.530	5.642	5.501
AL049796.1	4.118	4.030	4.117	4.377	4.179	4.318
VEZT	10.789	10.827	10.895	10.658	10.645	10.701
DCAF1	9.348	9.250	9.274	9.433	9.231	9.210
DCAF7	10.684	10.634	10.618	10.447	10.414	10.503
DCAF6	9.664	9.593	9.621	9.373	9.232	9.433
DCAF5	9.499	9.478	9.692	9.339	9.455	9.282
DCAF4	9.115	9.109	9.128	8.924	8.858	9.047

DCAF8	8.940	8.845	9.062	9.073	9.134	8.991
POLR3F	6.057	6.150	6.224	6.147	6.306	6.206
POLR3G	7.490	7.634	7.790	7.559	7.543	7.662
POLR3D	8.832	9.119	9.200	8.949	9.045	9.047
POLR3E	9.962	9.988	10.091	9.886	9.790	9.883
POLR3B	8.967	8.938	8.928	8.714	8.699	8.776
POLR3C	8.427	8.466	8.565	8.325	8.418	8.386
POLR3A	8.736	8.366	8.311	8.591	8.263	8.852
POLR3K	9.701	9.755	9.886	9.776	9.668	9.661
POLR3H	9.104	9.102	9.252	9.002	9.003	9.070
DNMT1	9.774	9.612	9.734	9.320	9.308	9.397
AL161912.2	6.589	6.425	6.401	6.694	6.422	6.605
HMGB3P30	5.139	5.205	5.450	5.588	5.362	5.217
HOXD8	6.094	5.985	5.930	6.166	6.348	6.128
HOXD9	6.941	6.891	6.922	6.889	6.800	7.049
HOXD4	6.388	6.561	6.310	6.400	6.412	6.587
HOXD1	7.949	7.970	7.758	7.940	8.025	8.083
HOXD3	5.988	5.984	5.989	6.575	6.139	6.391
MTPAP	9.010	8.947	8.931	8.889	8.935	8.861
PCCA-DT	7.327	7.444	7.536	7.384	7.526	7.319
AC138470.1	3.925	4.101	3.854	4.180	4.011	4.174
GLT1D1	8.268	7.944	7.729	8.377	8.471	8.223
HSPG2	11.302	11.382	11.422	11.090	10.848	11.242
TMEM127	9.811	9.896	9.993	9.602	9.638	9.748
AC009365.1	8.409	8.500	8.552	8.367	8.606	8.639
LAIR1	7.598	7.517	7.548	7.696	7.853	7.673
LAIR2	8.524	8.182	8.167	8.718	8.514	8.475
TDRD12	7.359	7.283	7.127	7.293	7.326	7.317
AC092117.1	8.671	8.550	8.530	8.452	8.641	8.678
HSPE1-MOB4	11.689	11.785	11.891	11.930	11.936	11.995
RRP7BP	9.257	9.285	9.456	9.276	9.473	9.192
C6orf15	8.157	8.238	8.140	8.124	8.118	8.143
USP27X-AS1	7.118	7.131	7.012	6.974	6.991	6.955
CDK11A	8.763	8.734	8.898	8.918	8.703	8.710
CDK11B	8.763	8.734	8.898	8.918	8.703	8.710
AL359538.1	4.487	4.387	4.312	4.496	4.484	4.219
BRD7P2	10.625	10.648	10.622	10.604	11.069	10.353
LRRC8C-DT	6.294	6.132	6.442	5.953	6.106	6.252
MIR135A2	6.465	6.382	6.553	6.753	6.624	6.679
SCRN2	8.597	8.646	8.785	8.507	8.440	8.397
SCRN1	12.302	12.331	12.510	12.279	12.316	11.984
TNP2	5.916	5.959	5.796	6.008	6.067	5.990
TNP1	6.560	6.394	6.658	6.622	6.735	6.844
NPBWR2	5.556	5.657	5.545	5.905	5.443	5.351
RBCK1	11.653	11.671	11.899	11.611	11.621	11.557
ZNF503-AS1	8.218	8.159	8.217	8.790	8.413	8.384
ZNF503-AS2	8.515	8.497	8.551	8.818	8.645	8.943
SLC52A1	6.711	6.838	6.941	6.851	7.065	6.762
SLC52A3	8.673	8.739	8.912	9.220	8.914	8.941
SLC52A2	11.167	11.334	11.462	11.345	11.322	11.144
ARPP19	12.073	12.135	12.237	12.085	11.970	11.962
SLC6A19	8.048	8.291	8.324	8.193	8.293	8.284
SLC6A18	6.810	7.191	7.297	7.038	7.077	7.281
TMEM204	8.563	8.483	8.537	8.619	8.701	8.632
TMEM205	11.808	11.913	12.120	11.881	11.955	11.811
TMEM207	7.919	7.780	7.743	7.790	7.563	7.640

SLC6A13	8.153	8.302	8.314	8.426	8.404	8.408
SLC6A12	7.389	7.532	7.287	7.515	7.685	7.718
SLC6A11	6.353	6.440	6.569	6.716	6.628	6.583
SLC6A17	8.771	8.855	8.814	8.964	8.904	9.073
SLC6A16	6.699	6.818	6.565	6.729	7.075	6.738
SLC6A15	6.158	5.983	6.104	6.436	6.311	6.179
SLC6A14	7.916	7.971	7.910	7.547	7.705	7.670
HMGCS1	9.872	10.027	10.005	10.023	9.878	9.976
HMGCS2	7.081	6.994	6.707	7.277	7.204	7.076
KIAA1324	7.179	7.298	7.339	7.316	7.395	7.382
KIAA1328	7.772	7.854	7.853	8.072	7.805	7.994
AC090578.1	4.246	4.283	4.169	4.448	4.084	4.295
AC027290.3	7.384	7.276	7.037	7.414	7.324	7.150
C14orf93	7.845	7.872	7.986	7.893	7.915	7.756
ANKRA2	8.483	8.388	8.481	7.998	7.916	8.120
DEFB4B	8.356	8.232	8.207	8.393	8.386	8.369
DEFB4A	8.356	8.232	8.207	8.393	8.386	8.369
TAC1	6.907	7.045	7.135	6.255	6.549	6.835
TAC3	8.951	9.230	8.940	8.973	9.152	9.202
TAC4	6.131	5.973	5.871	6.191	5.762	6.013
PYCARD-AS1	8.235	8.256	8.365	8.537	8.496	8.271
CALHM5	7.465	7.365	7.087	7.762	7.544	7.506
MPND	8.713	8.832	9.014	8.508	8.493	8.561
AC138696.1	7.384	7.322	7.304	7.282	7.283	7.239
CALHM6	6.662	6.579	6.497	6.531	6.634	6.570
CALHM1	6.260	6.192	6.087	6.208	6.108	6.230
CALHM3	6.024	5.920	5.842	5.858	5.968	5.844
CALHM2	7.350	7.536	7.589	7.661	7.526	7.461
HPR	6.418	6.405	6.589	6.460	6.661	6.447
AC104083.1	8.693	8.550	8.587	8.234	8.262	8.362
HPN	8.397	8.477	8.642	8.754	8.676	8.751
HPD	6.492	6.405	6.528	6.544	6.403	6.556
C2orf50	5.920	6.045	5.926	6.177	6.127	6.206
GABARAPL3	6.480	6.181	6.123	6.373	6.518	6.446
AL845439.1	9.231	9.374	9.436	9.462	9.438	9.339
MIR373	7.282	7.149	7.230	7.410	7.454	7.037
OAZ2	10.967	11.046	11.215	10.928	11.002	11.037
OAZ3	7.439	7.060	7.428	7.217	7.349	7.181
OAZ1	13.392	13.441	13.487	13.337	13.316	13.330
Z97205.1	6.204	6.164	6.101	6.122	6.326	6.096
FBP1	7.313	7.534	7.554	7.763	7.544	7.561
FBP2	7.125	7.254	7.279	7.127	7.105	7.205
AC022413.2	7.360	7.120	7.384	7.397	7.520	7.405
KDM4C	7.916	7.726	7.817	7.596	7.487	7.748
KDM4B	9.131	9.359	9.409	9.078	9.353	8.956
URI1	10.270	10.333	10.359	10.150	9.951	10.152
KDM4D	6.694	6.997	6.784	6.658	6.922	6.817
KRAS	11.001	11.087	11.287	11.241	11.434	10.642
ADAM6	7.202	7.492	7.345	7.570	7.424	7.400
ADAM7	6.859	6.804	6.387	6.874	6.887	6.738
ADAM5	6.603	6.600	6.388	6.986	6.762	6.748
ADAM2	4.300	4.411	4.360	4.532	4.493	4.194
EEF1G	14.111	14.146	14.160	14.135	14.090	14.165
EEF1D	13.398	13.443	13.616	13.285	13.258	13.338
ADAM8	7.801	7.960	8.100	8.072	7.847	8.002
ADAM9	12.378	12.424	12.442	12.307	12.149	12.453

NKX1-1	8.534	8.429	8.566	8.592	8.460	8.603
SPO11	4.211	4.070	4.137	4.284	4.202	4.187
IL20RA	7.961	7.830	8.077	7.857	7.881	7.929
IL20RB	10.025	10.114	10.191	10.312	10.275	10.326
RPL8	14.026	14.047	14.096	14.092	13.969	14.080
RPL9	13.154	13.225	13.361	13.496	13.391	13.505
RPL6	13.671	13.713	13.720	13.661	13.586	13.672
RPL7	13.823	13.851	13.913	13.938	13.843	13.938
RPL4	13.722	13.735	13.799	13.673	13.424	13.650
OLFML3	7.136	7.131	7.095	7.079	7.253	7.048
KCNQ1OT1	8.861	8.768	8.603	8.999	9.056	9.056
RPL3	13.670	13.651	13.720	13.644	13.478	13.579
FEN1	11.048	10.997	11.060	10.828	10.765	10.733
FAM87B	7.847	8.047	7.302	7.735	7.893	7.763
FAM87A	7.847	8.047	7.302	7.735	7.893	7.763
LUC7L	8.524	8.519	8.553	8.493	8.339	8.453
AC095055.1	6.950	6.790	6.811	6.820	6.882	6.632
TERF1P1	8.934	8.971	9.168	8.832	8.933	8.613
TERF1P5	8.934	8.971	9.168	8.832	8.933	8.613
TERF1P4	8.934	8.971	9.168	8.832	8.933	8.613
SCD	13.053	13.131	13.123	13.048	12.815	13.115
NKX1-2	4.566	4.579	4.485	4.888	4.882	4.649
ARMCX5-GPRAS	8.174	8.273	8.175	8.117	8.094	8.255
ERMAP	6.421	6.432	6.462	6.191	6.341	6.014
SCT	5.789	5.547	6.001	6.125	5.602	5.812
TROAP	8.879	8.839	9.008	8.664	8.879	8.838
WASH6P	9.341	9.202	9.345	9.278	9.301	9.395
WFDC9	7.628	7.585	7.441	7.419	7.798	7.635
WFDC8	7.254	7.480	7.189	7.284	7.311	7.377
GOLGA6D	7.189	7.300	7.215	7.148	7.273	7.257
GOLGA6C	7.189	7.300	7.215	7.148	7.273	7.257
GOLGA6B	7.189	7.300	7.215	7.148	7.273	7.257
GOLGA6A	7.189	7.300	7.215	7.148	7.273	7.257
ACO1	9.568	9.628	9.708	9.546	9.442	9.620
WFDC3	7.309	7.580	7.327	7.470	7.393	7.573
ACO2	10.923	10.992	11.121	10.722	10.450	10.685
WFDC5	5.189	5.183	5.344	5.402	5.341	5.319
WFDC6	6.517	6.558	6.465	6.424	6.506	6.713
INHA	6.670	6.688	6.882	7.008	6.938	6.944
TMEM65	9.282	9.160	9.210	8.918	8.816	8.957
TMEM64	9.742	9.686	9.807	9.566	9.488	9.596
TMEM62	8.815	9.066	8.990	8.807	8.833	9.089
TMEM61	7.180	7.244	7.346	6.926	7.246	7.224
TMEM60	9.772	9.850	9.832	9.665	9.680	9.732
TMEM69	10.169	10.203	10.091	9.865	9.895	9.901
TMEM68	7.744	7.843	7.902	7.490	7.513	7.622
ITM2C	9.642	9.700	9.780	9.507	9.578	9.477
ITM2B	12.800	12.878	13.007	12.884	12.813	12.833
ITM2A	10.634	10.578	10.947	10.759	10.979	10.481
KLHL40	6.454	6.343	6.367	6.337	6.475	6.633
AL807752.5	4.599	4.628	4.386	4.615	4.739	4.646
AL807752.6	9.947	10.021	10.203	9.851	9.822	9.934
AL807752.7	8.661	8.681	8.585	8.582	8.744	8.750
FJX1	9.429	9.514	9.599	9.236	9.340	9.297
UGT8	9.257	9.111	9.203	8.970	9.202	9.150
PAXBP1	8.598	8.646	8.598	8.455	8.678	8.710

ZNF628	8.268	8.125	8.429	8.050	8.127	8.011
ZNF629	9.726	9.710	9.904	9.763	9.699	9.735
ZNF624	5.156	5.189	5.348	5.349	5.069	5.111
ZNF625	6.058	6.001	5.831	6.002	5.949	5.912
ZNF626	6.930	6.887	6.914	6.901	6.959	7.098
ZNF627	8.171	8.241	8.326	8.606	8.644	8.694
ZNF621	7.165	7.109	7.198	7.170	7.293	6.945
ZNF622	8.984	9.017	9.145	9.062	8.952	9.024
ZNF623	7.543	7.325	7.486	6.702	6.931	7.255
UGT2B15	7.634	7.560	7.574	7.466	7.681	7.478
UGT2B17	4.528	4.395	4.160	4.694	4.503	4.696
UGT2B11	8.916	8.824	8.983	8.796	8.862	8.989
OLFML1	4.645	4.815	4.573	4.662	4.654	4.649
APAF1	8.632	8.792	8.748	8.419	8.471	8.846
RPL5	13.409	13.548	13.615	13.535	13.599	13.631
FSCB	5.139	5.051	5.218	5.366	5.289	5.546
GML	7.344	7.180	6.760	7.406	7.303	7.351
PDZK1P1	7.425	7.530	7.493	6.845	7.025	6.955
AC211476.11	9.313	9.354	9.149	9.020	9.274	9.164
AC211476.10	9.531	9.523	9.413	9.362	9.364	9.406
S100A4	8.230	8.474	8.498	8.540	8.607	8.432
S100A6	13.546	13.681	13.718	13.757	13.738	13.686
IGFALS	8.450	8.292	8.526	8.877	8.673	8.483
PRM1	7.299	7.266	7.380	7.324	7.499	7.553
PRM3	6.749	6.741	6.555	6.873	6.974	6.759
PRM2	5.815	5.851	5.850	6.012	6.158	5.866
ACVR1	11.230	11.309	11.421	11.050	10.949	11.048
AC009318.3	7.786	7.629	7.281	7.392	7.469	7.692
SWSAP1	7.572	7.744	7.815	7.485	7.725	7.652
AC009318.1	6.508	6.235	6.297	6.400	6.314	6.424
THAP11	9.844	9.883	10.116	9.737	9.885	9.802
LIFR-AS1	5.677	5.849	5.502	5.997	5.800	6.035
HOOK2	9.770	9.807	9.891	9.565	9.473	9.576
HOOK3	8.148	8.168	8.213	7.845	7.734	8.125
HOOK1	10.042	10.079	10.081	9.701	9.684	9.745
KCNE1B	5.578	5.850	5.893	5.684	5.596	5.738
SEBOX	7.741	7.949	7.748	7.850	7.979	7.814
AC008443.3	7.877	7.947	7.630	8.039	8.084	8.058
AC008443.1	8.043	7.961	8.033	7.590	7.816	7.848
AC096677.2	7.237	7.470	7.375	7.086	7.122	7.291
MIEF1	10.573	10.624	10.663	10.581	10.630	10.344
MIEF2	8.216	8.260	8.455	8.020	8.110	8.088
DKC1	11.133	11.208	11.242	11.040	10.874	10.978
TBC1D10C	7.254	7.243	7.364	7.403	7.402	7.908
SLC4A1AP	9.231	9.138	9.239	8.926	9.010	9.104
TBC1D10A	9.703	9.848	9.906	9.881	9.822	9.856
IGHG4	7.283	7.344	7.278	7.289	7.467	7.351
IGHG1	7.283	7.344	7.278	7.289	7.467	7.351
IGHG2	7.283	7.344	7.278	7.289	7.467	7.351
IGHG3	7.283	7.344	7.278	7.289	7.467	7.351
BPIFB2	7.485	7.450	7.361	7.491	7.538	7.736
BPIFB1	7.467	7.363	7.563	7.361	7.377	7.356
BPIFB6	6.815	6.900	6.692	6.762	6.820	6.884
BPIFB4	7.531	7.492	7.604	7.756	7.829	7.825
OARD1	8.925	9.111	9.117	8.638	8.536	8.701
LINS1	8.856	8.823	8.819	8.854	8.781	8.730

CAMK2D	10.546	10.589	10.654	10.367	10.418	10.512
CAMK2G	9.183	9.304	9.287	9.130	9.158	9.193
CAMK2A	8.004	7.998	7.909	8.217	8.393	8.266
CAMK2B	8.364	8.438	8.487	8.365	8.493	8.431
AC078880.2	5.043	5.259	5.188	5.107	5.028	5.146
KAZALD1	6.854	6.890	7.035	7.154	7.057	6.962
RNASE11	5.025	4.965	5.201	5.131	5.159	4.559
IGHGP	7.283	7.344	7.278	7.289	7.467	7.351
LINC01864	5.742	5.279	5.345	5.486	5.553	5.498
ZSCAN18	9.451	9.498	9.578	9.180	9.312	9.396
LINC01863	6.402	6.290	6.251	6.434	6.297	6.367
ZSCAN10	7.066	7.268	7.294	7.246	7.364	7.233
ZSCAN12	7.534	7.356	7.385	7.409	7.391	7.307
LINC01869	8.146	8.258	8.429	8.352	8.276	8.143
ZSCAN16	7.150	6.937	7.042	7.181	7.096	7.028
ENOX1	5.803	5.895	6.028	5.947	6.092	5.889
ENOX2	7.898	7.854	7.806	7.720	7.610	7.650
C9orf50	7.102	7.343	7.269	7.463	7.482	7.440
C9orf57	4.851	4.762	4.612	5.045	4.834	4.937
WDR41	8.528	8.623	8.745	8.465	8.485	8.540
HCG27	6.958	7.180	7.201	7.158	7.252	7.404
WDR43	8.396	8.289	8.554	8.039	8.126	8.161
WDR44	8.202	8.194	8.209	7.951	8.080	7.904
WDR45	9.913	9.931	10.090	9.628	9.593	9.597
WDR46	8.643	8.638	8.594	8.313	8.071	8.459
HCG22	8.439	8.433	8.212	8.323	8.555	8.407
WDR48	9.896	9.926	9.951	9.720	9.921	9.789
WDR49	6.369	6.338	6.099	6.225	6.411	6.411
JAK2	6.815	6.630	6.817	6.682	6.885	6.681
JAK3	8.629	8.576	8.697	8.577	8.553	8.590
JAK1	10.627	10.647	10.680	10.432	10.351	10.278
KIFC1	9.531	9.706	9.932	9.548	9.572	9.473
SNX18P3	5.624	5.551	5.579	5.667	5.364	5.778
NMRAL1	9.908	9.983	10.235	9.688	9.764	9.804
OTOAP1	6.880	6.877	6.878	6.815	6.879	6.936
ANKRD24	7.147	7.125	7.354	7.190	7.213	7.145
LINC01192	6.357	6.358	6.267	6.679	6.468	6.405
AC079316.2	6.358	6.311	6.249	6.609	6.455	6.503
PRKDC	9.558	9.642	9.560	9.019	8.990	9.188
MKRN1	10.497	10.641	10.669	10.373	10.368	10.289
WFDC1	7.176	7.209	7.168	7.040	7.314	7.390
MKRN3	6.131	6.214	5.907	6.171	5.939	6.149
MKRN2	8.240	8.245	8.241	8.081	8.153	8.151
AC087501.1	4.412	4.806	4.829	5.023	4.601	5.001
LHFPL4	8.042	8.092	7.907	8.230	8.168	8.298
LHFPL5	5.834	5.968	6.054	5.947	5.991	5.992
LHFPL6	5.266	4.936	5.063	5.374	5.217	4.960
LINC00920	6.739	6.743	6.510	6.618	6.885	6.786
LHFPL2	9.243	9.258	9.438	9.369	9.287	9.297
LHFPL3	5.786	5.944	5.573	6.080	5.873	6.003
TLL1	4.362	4.447	4.288	4.303	4.369	4.319
TLL2	7.325	7.064	7.308	7.209	7.338	7.266
WFDC2	10.382	10.444	10.561	10.388	10.531	10.505
ANKRD29	5.530	5.366	5.363	5.405	5.456	5.454
RIMS4	7.610	7.658	7.511	7.636	7.637	7.735
KIF21A	8.240	8.233	8.415	8.108	8.109	8.158

RIMS1	6.876	6.817	6.963	7.029	7.098	6.992
RIMS2	13.912	13.956	14.032	14.042	14.027	14.131
KIF21B	8.506	8.467	8.397	8.439	8.530	8.539
AC107959.1	5.802	5.865	5.819	5.994	5.863	5.843
TMEM67	6.776	6.546	6.378	6.414	6.390	6.609
AC107959.2	6.513	6.559	6.249	6.445	6.384	6.534
AC107959.4	8.299	8.018	8.316	8.452	8.290	8.336
MCAM	8.859	8.836	8.956	8.936	9.042	9.090
MCAT	8.905	8.879	9.032	8.615	8.640	8.756
HCRT	6.425	6.420	6.422	6.758	6.639	6.822
FAM201A	5.510	5.675	5.825	5.162	5.407	5.354
TAGLN3	7.697	7.725	7.726	7.913	7.857	7.914
IL21R	6.695	6.653	6.656	7.026	6.874	6.637
EMX2	5.646	5.656	5.679	5.879	5.700	5.895
EMX1	6.703	6.880	6.780	7.153	7.039	6.945
AC018816.1	7.369	7.364	7.108	7.404	7.465	7.469
PRKD1	7.949	8.091	8.091	7.621	7.725	7.882
PRKD2	10.503	10.565	10.676	10.394	10.444	10.430
PRKD3	8.367	8.497	8.389	8.065	8.220	8.291
SLC7A7	8.336	8.294	8.269	8.227	8.338	8.284
AC022748.2	5.920	5.683	6.001	5.810	5.872	5.766
LRRC37A4P	7.316	7.496	7.592	7.563	7.519	7.583
KLHL42	8.729	8.612	8.731	8.407	8.383	8.586
DND1P1	9.778	9.851	9.856	9.219	9.772	9.656
OVCH1-AS1	9.226	9.347	9.230	9.187	9.205	8.871
KLHL41	7.390	7.468	7.379	7.511	7.541	7.566
KLF7	9.266	9.214	8.967	8.891	9.208	9.187
KLF6	10.559	10.504	10.557	10.574	10.522	10.294
KLF5	11.894	11.648	11.805	11.856	11.968	11.654
KLF4	9.281	9.232	9.288	9.264	9.284	9.251
KLF3	10.635	10.627	10.572	10.368	10.408	10.447
KLF2	8.856	8.812	8.906	8.756	8.602	8.744
KLF1	6.568	6.613	6.596	6.377	6.843	6.705
KLF9	7.220	7.279	7.389	7.520	7.364	7.294
KLF8	7.339	7.320	7.282	7.576	7.315	7.268
MCTS1	10.877	10.989	11.059	10.874	11.058	11.007
REST	9.747	9.581	9.242	9.696	9.755	9.662
SFSWAP	9.393	9.215	9.394	9.692	9.399	9.418
POU1F1	3.927	3.985	3.735	4.204	3.960	3.981
PHF21A	7.971	7.919	8.104	7.874	7.920	8.023
AL135903.1	9.971	10.078	10.189	10.282	10.341	10.161
VPS25	10.937	11.040	11.200	11.004	10.980	10.917
TACO1	8.413	8.409	8.621	8.349	8.359	8.288
MAP3K10	8.605	8.595	8.718	8.978	8.830	8.890
MAP3K11	9.938	9.909	10.061	9.835	9.955	9.922
MAP3K12	8.820	8.793	9.221	9.169	8.797	8.729
MAP3K13	11.915	12.125	12.178	12.117	12.393	11.967
ADNP2	9.244	9.285	9.530	9.300	9.258	9.145
ZNF540	6.534	6.439	6.420	6.316	6.335	6.456
KLRA1P	4.506	4.618	4.444	4.584	4.551	4.557
MAP3K19	9.542	9.503	9.260	9.583	9.715	9.649
ZNF549	5.021	5.199	4.889	4.988	5.027	5.191
STXBP5L	7.297	7.126	7.290	7.299	7.376	7.176
VPS29	11.574	11.714	11.835	11.680	11.643	11.604
HSD11B1-AS1	7.250	7.395	7.122	7.304	7.405	7.285
LINC01419	3.878	3.835	4.009	3.893	4.026	4.190

LINC01416	6.798	6.687	6.804	7.090	7.082	6.863
LINC01410	6.139	6.096	6.076	6.214	6.293	6.282
RARA	10.207	10.242	10.403	10.451	10.597	10.327
TAS2R50	5.055	4.923	4.940	4.747	4.900	4.909
AC004951.1	6.748	6.456	6.428	6.759	6.520	6.635
RARB	9.176	9.170	9.221	9.030	9.072	9.144
FAM163B	7.284	7.263	7.320	7.503	7.391	7.324
RARG	9.272	9.395	9.465	9.510	9.426	9.457
AC004951.4	9.289	9.336	9.445	9.220	9.197	9.291
RABIF	9.335	9.244	9.305	9.226	9.245	9.269
SLC7A1	11.377	11.354	11.375	11.127	11.074	11.205
JAKMIP3	5.873	5.972	5.909	6.197	5.823	6.070
JAKMIP2	6.042	6.255	5.917	6.180	5.948	6.042
JAKMIP1	6.106	5.946	5.965	6.007	6.298	6.352
PCYT2	8.941	9.079	9.225	8.873	8.976	8.862
NMT2	6.659	6.621	6.718	6.481	6.421	6.709
NMT1	11.035	11.103	11.200	11.001	10.826	10.899
SDE2	9.864	9.926	9.905	9.820	9.777	9.878
PRKRA	9.695	9.711	9.802	9.310	9.356	9.502
PTGES3	13.425	13.484	13.520	13.485	13.478	13.553
PTGES2	9.579	9.809	9.941	9.837	9.700	9.742
SNORA17B	7.492	7.288	7.305	7.420	7.454	7.385
SYF2	9.503	9.546	9.562	9.261	9.190	9.253
AC092656.1	4.718	4.527	4.508	4.671	4.716	4.744
MIR1825	8.898	8.795	8.985	8.752	8.757	8.677
MCM8	7.619	7.688	7.791	7.263	7.385	7.459
CCAR1	9.671	9.322	9.361	9.068	9.486	9.203
CCAR2	9.711	9.855	9.741	9.510	9.391	9.654
ZBTB8A	8.474	8.701	8.797	8.424	8.606	8.556
ZBTB8B	5.686	5.710	5.671	5.704	5.502	5.687
RNF139-AS1	6.549	6.569	6.616	6.654	6.384	6.841
LINC02850	3.937	3.945	4.018	3.664	3.960	3.859
LINC02853	6.701	7.038	6.683	7.020	6.936	7.074
AL591643.1	4.005	3.982	3.776	3.962	4.228	4.092
F12	8.835	8.906	9.071	8.808	8.988	8.990
F10	6.268	6.509	6.448	6.358	6.477	6.626
F11	7.059	7.057	7.273	7.553	7.448	7.250
HDGFL1	9.407	9.455	9.344	9.393	9.431	9.490
HDGFL3	10.471	10.481	10.656	10.516	10.513	10.478
HDGFL2	8.578	8.651	8.828	8.673	8.576	8.555
AC092660.1	7.843	7.983	7.881	7.756	7.970	7.869
RPL27AP	6.954	7.063	7.313	7.290	7.119	7.108
SEC24B-AS1	5.774	5.711	5.586	5.657	5.946	5.581
NODAL	8.225	8.172	8.037	8.208	8.392	8.404
RFPL1	4.204	4.049	4.081	4.179	4.348	4.114
RCOR2	8.252	8.291	8.364	8.418	8.576	8.409
RCOR1	8.935	9.041	8.997	8.599	8.744	8.823
RFPL2	7.588	7.714	7.746	7.721	7.894	7.952
TIGD5	9.012	9.324	9.465	9.223	9.139	8.998
TIGD4	5.062	5.135	4.970	4.952	5.150	5.101
TIGD7	8.094	8.089	8.251	7.934	8.260	8.158
TIGD6	4.353	4.271	4.433	4.637	4.295	4.285
AL669830.2	8.614	8.670	8.743	8.558	8.568	8.605
PTPRCAP	6.876	6.817	6.640	6.635	7.018	6.932
KIAA1958	8.637	8.702	8.810	8.679	8.893	8.807
OXSM	8.834	8.913	8.939	8.865	9.032	8.978

AC010680.2	6.784	6.601	6.757	6.741	6.895	6.628
AC010680.1	3.533	3.537	3.615	3.493	3.617	3.553
RAB43P1	8.719	8.813	8.783	8.875	9.033	8.729
AC010680.4	5.843	5.541	5.662	5.722	5.453	5.788
MIR186	4.272	4.316	4.222	4.293	4.233	4.336
MRPS18A	10.026	10.100	10.306	10.046	10.178	10.081
MRPS18B	10.301	10.407	10.450	10.162	10.376	10.177
MRPS18C	9.250	9.331	9.490	9.363	9.409	9.302
AC093614.1	5.629	5.637	5.650	5.415	5.501	5.815
AC005586.1	7.486	7.318	7.346	7.741	7.605	7.425
AC005586.2	7.609	7.632	7.657	7.590	7.821	7.683
NRARP	9.207	9.245	9.267	9.161	9.242	9.046
EXOC3L4	7.102	7.194	7.372	7.320	7.377	7.142
AC009234.1	4.191	4.138	4.179	4.378	4.356	4.439
NOP14	9.026	9.104	9.155	8.951	8.881	8.765
AMOTL1	9.124	9.190	9.268	9.022	9.006	9.026
LCN6	7.300	7.524	7.362	7.423	7.424	7.399
LCN1	6.701	6.792	6.922	7.036	6.796	6.893
RXYLT1	11.092	11.259	11.510	11.371	11.396	10.812
LCN2	14.252	14.259	14.307	14.288	14.300	14.301
BPI	7.551	7.561	7.730	7.673	7.825	7.924
VPS52	10.633	10.676	10.627	10.617	10.645	10.650
LCN8	7.096	6.981	7.023	7.315	7.349	7.337
ASLP1	6.765	6.541	6.855	7.101	6.677	6.896
AC021074.3	6.229	6.219	5.830	6.162	6.425	6.302
DDHD1	7.548	7.507	7.459	7.440	7.497	7.240
CYCSP44	5.276	5.193	5.555	5.416	5.376	5.331
DDHD2	9.496	9.445	9.592	9.012	8.856	9.058
DNAJC16	9.260	9.353	9.456	9.373	9.507	9.035
DNAJC17	7.333	7.255	7.588	7.191	7.403	7.265
DNAJC14	9.399	9.468	9.574	9.306	9.233	9.321
RTBDN	7.913	8.092	7.899	8.170	8.132	8.173
DNAJC12	6.925	6.853	6.787	6.846	6.885	6.737
DNAJC13	10.627	10.801	10.644	10.400	10.391	10.557
DNAJC10	10.774	10.969	10.912	10.748	10.756	10.894
DNAJC11	10.062	10.012	10.116	10.007	9.944	9.822
AL591135.1	8.642	8.710	8.568	8.301	8.199	8.581
DNAJC18	7.830	8.092	7.813	7.777	7.993	7.879
DNAJC19	10.445	10.482	10.612	10.404	10.367	10.439
EDF1	10.798	10.814	10.973	10.652	10.472	10.481
VPS50	10.176	10.249	10.216	10.168	10.139	10.240
SUPT16H	11.009	10.992	11.063	10.776	10.776	10.826
AC002553.1	5.779	5.928	5.653	5.899	5.990	6.067
SCML2	8.819	8.844	8.909	8.863	8.981	8.865
SCML1	8.710	8.646	8.598	8.495	8.625	8.711
SCML4	6.740	6.750	6.856	6.947	6.728	6.788
NRK	4.859	4.747	4.817	4.877	4.974	5.082
NRM	10.614	10.607	10.686	10.372	10.366	10.371
NRL	7.840	7.892	7.925	7.934	8.022	7.987
AC010627.1	5.563	5.506	5.551	5.404	5.542	5.553
ZFAND6P1	11.160	11.120	11.194	11.199	11.142	11.293
AP000525.1	8.071	7.941	7.789	8.159	7.815	8.096
TUSC3	6.724	6.638	6.507	6.649	6.652	6.779
IER3IP1	10.566	10.682	10.767	10.432	10.402	10.485
AL158206.1	7.925	8.058	8.093	7.857	7.912	7.971
TPSG1	8.200	8.306	8.303	8.344	8.237	8.223

NUP214	8.919	8.993	9.030	8.851	8.694	8.867
TPGS2	10.754	10.878	11.026	10.580	10.655	10.565
TPGS1	7.481	7.578	7.866	7.193	7.301	7.366
NUP210	9.798	9.803	9.741	9.618	9.730	9.551
AL022313.1	7.107	7.392	7.372	7.274	7.481	7.338
VPS54	8.690	8.560	8.616	8.180	8.330	8.412
KIAA0319L	10.253	10.453	10.613	10.328	10.368	10.170
EVX1	7.522	7.453	7.802	7.859	7.808	7.706
FHDC1	8.406	8.464	8.477	8.365	8.316	8.494
MIA-RAB4B	8.846	8.922	9.077	8.907	8.815	8.883
TRPM6	8.413	8.439	8.005	8.469	8.401	8.416
PRAMEF14	7.406	7.569	7.636	7.983	7.893	7.730
PRAMEF15	7.847	8.084	8.166	8.407	8.226	8.207
PRAMEF12	7.166	7.277	7.060	7.040	7.252	7.318
PRAMEF13	7.406	7.569	7.636	7.983	7.893	7.730
PRAMEF10	7.104	7.179	7.162	7.393	7.499	7.346
PRAMEF11	7.847	8.084	8.166	8.407	8.226	8.207
EME1	9.008	9.014	9.304	8.981	9.348	9.015
EME2	8.464	8.497	8.579	8.612	8.536	8.599
DUOX1	6.817	6.645	6.702	6.920	6.851	6.631
HGH1	10.628	10.720	10.795	10.612	10.605	10.524
DUOX2	6.564	6.384	6.441	6.742	6.648	6.629
ATP6V1FNB	5.625	5.634	5.284	5.332	5.654	5.654
MCHR2-AS1	6.673	6.524	6.658	7.086	6.847	6.624
AC002467.1	6.650	6.445	6.547	6.554	6.824	6.698
SULT1C4	5.070	5.137	5.008	5.029	5.139	5.138
ECH1	11.404	11.364	11.624	11.280	11.130	11.278
AC237221.1	8.351	8.199	8.184	8.398	8.498	8.461
AP002026.1	6.515	6.345	6.531	6.791	6.636	6.600
AL449403.3	4.316	4.358	4.198	4.258	4.200	4.306
CDHR1	6.113	6.034	6.407	6.275	6.093	6.138
MOGS	9.476	9.420	9.618	9.509	9.211	9.341
CDHR3	6.010	5.835	5.763	5.814	5.933	5.887
CDHR5	7.243	7.194	7.281	7.498	7.282	7.245
ACTR3P2	4.616	4.580	4.608	4.537	4.482	4.458
AC079411.1	6.773	6.597	6.696	6.850	7.027	6.766
AC026124.1	5.229	5.122	4.751	4.843	4.975	5.062
C19orf84	9.162	9.023	8.719	9.097	9.139	9.281
RAB22A	9.751	9.800	9.822	9.428	9.280	9.463
C19orf81	5.500	5.734	6.164	5.632	5.750	5.699
CST13P	5.970	6.054	6.023	6.005	6.244	6.100
AC009052.1	11.165	11.162	11.353	11.208	11.149	11.186
SALL4	8.389	8.387	8.350	8.331	8.380	8.442
SALL1	4.026	4.097	4.137	4.160	4.516	4.087
SALL3	6.535	6.491	6.533	6.620	6.677	6.584
SALL2	8.437	8.429	8.455	8.348	8.563	8.412
CYP1A1	7.953	7.735	7.883	8.005	8.101	8.266
CYP1A2	8.814	8.813	8.737	8.614	8.653	8.795
MIR147B	9.319	9.467	9.486	9.545	9.710	9.765
C5orf34	6.732	6.813	6.795	6.448	6.585	6.384
C5orf30	8.777	8.878	8.884	8.639	8.572	8.670
HYAL6P	4.417	4.333	4.278	4.593	4.560	4.403
FIS1	9.560	9.696	9.866	9.527	9.545	9.510
ACTR3BP1	6.931	6.977	6.989	6.912	6.997	6.878
ZMIZ2	11.142	11.186	11.175	11.067	10.989	11.086
ACTR3BP3	7.623	7.706	7.631	7.631	7.621	7.671

ACTR3BP2	7.623	7.706	7.631	7.631	7.621	7.671
ACTR3BP5	7.623	7.706	7.631	7.631	7.621	7.671
MELTF	8.144	8.109	8.088	7.746	7.675	7.955
ACTR3BP6	7.623	7.706	7.631	7.631	7.621	7.671
PPDPFL	5.810	5.990	5.922	5.986	6.086	6.057
AL583836.1	7.130	7.115	6.984	7.057	7.128	7.188
RIMBP3B	8.535	8.490	8.448	8.513	8.691	8.374
OR5H15	4.250	4.300	4.076	4.222	4.293	4.306
OR7E2P	4.131	4.207	4.340	4.264	4.394	4.223
WARS2-AS1	7.250	7.063	7.164	6.888	6.911	6.771
BX927229.2	9.231	9.374	9.436	9.462	9.438	9.339
AL121839.6	8.205	8.166	7.937	7.864	7.936	8.008
CYP2A7	8.125	8.301	8.394	7.997	8.081	8.010
CYP2A6	8.682	8.678	8.812	9.018	8.896	8.835
AL121839.2	8.205	8.166	7.937	7.864	7.936	8.008
OR7E24	6.629	6.495	6.558	6.379	6.517	6.666
PCP4	6.422	6.363	6.251	6.377	6.383	6.580
RNFT2	9.678	9.739	9.865	9.474	9.538	9.518
RNFT1	7.036	7.224	7.048	7.160	7.204	7.353
HBP1	8.879	9.004	9.125	9.072	9.187	9.072
SEC24C	9.494	9.653	9.629	9.585	9.537	9.583
ELOF1	9.888	10.005	10.169	9.875	9.870	9.838
MFHAS1	10.530	10.638	10.785	10.604	10.590	10.305
DHX16	9.298	9.276	9.333	8.968	8.871	8.985
DHX15	11.788	11.834	11.797	11.636	11.621	11.679
LEPROT	12.070	12.205	12.277	11.965	12.156	11.953
DGCR9	7.705	7.753	7.593	7.609	7.860	7.679
DGCR8	8.690	8.622	8.739	8.495	8.501	8.549
FTH1P20	13.980	13.991	14.082	13.979	13.810	13.985
DGCR2	9.818	9.923	10.028	9.660	9.844	9.780
DGCR5	7.903	7.897	8.002	8.124	7.921	7.975
DGCR6	10.503	10.564	10.866	10.550	10.504	10.580
ZNF783	8.582	8.550	8.608	8.506	8.488	8.568
ZNF782	5.550	5.276	5.197	5.360	5.494	5.249
ZNF781	4.693	4.707	4.580	4.745	4.717	4.876
ZNF787	8.731	8.761	8.999	8.759	8.942	8.537
ZNF786	7.024	6.834	7.250	6.919	7.015	6.807
ZNF785	8.802	8.582	8.420	8.672	9.001	8.582
ZNF784	8.860	8.946	9.144	8.916	8.908	8.883
ZNF789	7.306	7.461	7.335	7.237	7.357	7.348
RPP40	8.874	8.919	8.865	8.786	9.016	8.834
LLCFC1	6.420	6.308	6.311	6.539	6.610	6.467
PYY	5.590	5.420	5.596	5.503	5.655	5.837
SMIM29	8.604	8.603	8.853	8.822	8.712	8.606
SMIM22	10.020	10.149	10.354	10.249	10.188	10.160
SMIM20	9.505	9.701	9.819	9.553	9.390	9.550
SMIM21	6.860	6.977	6.982	6.795	6.852	7.003
SMIM26	9.097	9.201	9.366	8.851	8.819	8.924
SMIM27	8.330	8.549	8.547	8.200	8.321	8.164
SMIM24	8.054	8.100	8.232	8.240	8.270	8.102
SMIM25	7.528	7.489	7.387	7.298	7.617	7.623
HGFAC	7.472	7.414	7.719	7.421	7.355	7.518
ADAMDEC1	4.513	4.407	4.310	4.645	4.596	4.727
RBMY2DP	5.549	5.417	5.468	5.650	5.551	5.647
AC025278.2	7.655	7.697	7.518	7.524	7.752	7.734
AC025278.1	4.200	4.166	4.252	4.612	4.645	4.347

RHOT1	9.091	9.142	9.182	9.166	9.264	9.139
CDSN	8.501	8.511	8.430	8.407	8.507	8.673
DCLK1	4.843	4.731	4.688	4.618	4.803	4.896
DCLK3	4.601	4.652	4.737	4.841	4.663	4.708
DCLK2	7.425	7.428	7.370	7.378	7.634	7.467
NOX1	7.663	7.891	7.712	7.951	8.092	7.937
NOX3	5.008	5.278	5.260	5.421	5.557	5.417
NOX5	8.704	8.793	8.724	9.119	8.922	8.766
NOX4	5.017	5.218	5.125	5.376	5.086	4.988
PGS1	8.216	8.270	8.365	8.075	7.994	8.123
AC008870.3	4.922	4.772	4.415	4.704	4.509	4.593
UBE3B	9.850	9.970	9.990	9.737	9.796	9.799
UBE3C	9.094	9.159	9.194	8.823	9.001	8.851
UBE3A	8.689	8.834	8.668	8.520	8.519	8.525
UBE3D	5.690	5.608	5.982	5.349	5.556	5.645
AC007744.1	5.674	5.707	5.852	5.645	5.743	5.717
MTRF1	7.809	7.742	7.787	7.634	7.730	7.727
MIGA2	8.150	8.239	8.319	8.328	8.115	8.107
MIGA1	8.455	8.515	8.559	8.132	8.305	8.265
SNORD32A	14.076	14.099	14.141	14.125	14.125	14.139
AL391095.2	7.480	7.446	7.296	7.319	7.467	7.403
GET1	10.183	10.276	10.258	9.930	9.835	10.025
MTFR2	8.659	8.673	8.770	8.338	8.424	8.266
GET3	9.634	9.671	9.862	9.669	9.872	9.750
GET4	10.458	10.487	10.651	10.320	10.113	10.313
GLOD5	7.508	7.537	7.563	7.304	7.517	7.447
GLOD4	9.685	9.803	9.950	9.588	9.557	9.586
GCH1	8.672	8.754	8.865	8.463	8.365	8.640
LINC00504	4.373	4.437	4.370	4.395	4.597	4.594
STIMATE-MUSTN	8.327	8.412	8.411	8.618	8.463	8.412
LINC00507	4.133	4.255	4.448	4.411	4.430	4.217
NUDT4P2	9.971	10.062	10.046	9.918	9.987	10.114
AC074035.1	4.653	4.635	4.541	4.507	4.747	4.563
NAA20	9.067	9.185	9.286	9.177	9.078	9.140
LINC00210	4.623	4.523	4.344	4.603	4.505	4.496
NAA25	9.814	10.175	10.271	10.283	10.372	9.654
CNKSR1	8.711	8.775	8.731	8.661	8.706	8.558
CNKSR3	7.479	7.566	7.700	7.763	7.593	7.648
CNKSR2	7.342	7.461	7.350	7.295	7.082	7.270
AC078927.1	12.030	12.141	12.216	12.162	12.212	11.977
AL391988.1	4.970	4.854	4.987	4.877	4.911	4.775
QDPR	10.402	10.494	10.635	10.243	10.196	10.241
AC007780.1	7.285	7.304	7.300	7.353	7.312	7.371
TBC1D19	7.146	7.347	7.264	7.208	7.453	7.250
TBC1D12	7.446	7.574	7.625	7.273	6.931	7.225
TBC1D13	8.450	8.500	8.488	8.346	8.524	8.510
TBC1D16	8.733	8.654	8.856	8.604	8.597	8.532
TBC1D17	9.002	9.259	9.254	8.827	8.968	8.939
TBC1D14	9.950	9.968	10.073	9.890	9.871	10.018
TBC1D15	9.886	9.923	9.915	9.674	9.642	9.556
AUTS2	6.427	6.158	6.175	6.327	6.188	6.295
TENT2	8.693	8.799	8.899	8.748	8.554	8.700
ZBED1	9.021	9.038	9.136	8.916	8.782	8.935
AC006064.6	10.057	10.101	10.049	10.135	10.005	10.042
AC006064.2	4.444	4.414	4.445	4.572	4.448	4.614
HSPA1B	12.492	12.592	12.692	12.633	12.502	12.672

HSPA1A	13.146	13.229	13.208	13.291	13.366	13.384
PF4	8.644	8.710	8.552	8.627	8.672	8.591
ZBED4	8.850	9.050	9.036	8.748	8.669	8.707
CACTIN	8.903	8.876	8.980	8.842	8.894	8.902
LAMB3	10.975	11.106	11.214	10.847	10.723	10.935
MIR101-1	4.685	4.495	4.869	4.535	4.767	4.694
HSPA1L	7.574	7.440	7.386	7.607	7.690	7.502
CYSLTR2	7.633	7.610	7.581	7.609	7.882	7.714
CYSLTR1	4.848	4.962	4.668	4.890	4.910	4.959
PTGIS	5.584	5.817	5.822	5.780	5.835	5.778
PTGIR	8.723	8.780	8.854	8.810	8.755	8.915
LINC00967	6.423	6.229	6.031	6.313	6.396	6.403
LINC00964	4.368	4.382	4.329	4.336	4.347	4.253
LINC00963	7.082	7.196	7.356	7.112	7.222	7.096
LINC00960	7.103	7.195	7.091	6.846	7.148	7.370
CLTCL1	8.144	8.148	8.037	8.289	8.163	8.325
ANKRD60	6.422	6.283	6.358	6.812	6.470	6.343
LINC00968	8.000	8.051	7.844	8.123	8.301	8.101
HSPA13	8.940	8.852	9.102	9.091	9.109	9.132
HSPA14	8.783	8.762	8.895	8.830	8.776	8.812
MXI1	9.875	9.948	10.015	9.784	9.895	9.644
PDE2A	7.500	7.595	7.574	7.555	7.745	7.381
DXO	8.356	8.443	8.435	8.367	8.422	8.434
AC002464.1	5.507	5.665	5.511	5.832	5.764	5.465
SCFD1	9.805	9.897	10.000	9.817	9.792	9.705
SCFD2	9.720	9.866	10.004	9.853	9.929	9.720
MYPOP	9.252	9.370	9.373	9.337	9.307	9.359
FAM222A	7.601	7.562	7.643	7.498	7.657	7.787
GCOM2	9.036	8.971	9.207	8.986	9.194	8.944
GCOM1	9.541	9.570	9.535	9.536	9.571	9.433
FAM222B	10.762	10.855	10.750	10.642	10.795	10.581
MEN1	9.631	9.671	9.781	9.641	9.735	9.554
BRD3OS	8.879	8.866	9.062	8.986	9.065	8.790
KRT222	5.586	5.619	5.768	5.576	5.512	5.746
CSPG4P2Y	6.957	6.925	6.968	7.021	7.153	7.161
Z84488.1	5.083	5.134	5.186	5.196	5.410	5.195
OR2L2	4.954	4.912	4.878	4.982	4.982	4.868
OTUD7A	7.046	7.177	7.470	7.256	7.198	7.213
CTAGE9	10.096	10.073	10.117	10.027	10.081	10.109
RNU4-46P	10.741	10.510	10.494	9.897	10.117	10.099
OTUD7B	8.547	8.631	8.496	8.476	8.420	8.565
CTAGE1	4.544	4.676	4.482	4.595	4.568	4.625
RFPL1S	6.178	6.203	6.106	6.107	6.168	6.311
CTAGE4	10.096	10.073	10.117	10.027	10.081	10.109
CTAGE6	10.096	10.073	10.117	10.027	10.081	10.109
OR6W1P	7.638	7.706	7.773	7.887	7.986	7.757
PORCN	9.000	9.086	9.242	9.015	9.030	9.027
CRTAC1	7.209	7.386	7.239	7.612	7.372	7.467
STAMBPL1	7.988	8.166	8.273	7.623	7.875	7.664
LYPLA2P2	10.457	10.582	10.686	10.281	10.365	10.305
TTI1	8.880	8.973	8.852	8.403	8.655	8.689
MOCS1	7.502	7.247	7.522	7.549	7.601	7.584
AHR	11.232	11.165	11.144	11.305	11.253	11.379
RPL34-AS1	4.539	4.583	4.455	4.467	4.797	4.536
STK38L	8.791	8.833	8.788	8.605	8.690	8.817
QSOX2	8.654	8.752	8.941	8.695	8.556	8.724

QSOX1	11.820	11.823	12.020	11.693	11.586	11.651
GALNT18	8.822	9.047	9.220	8.749	8.726	8.758
PKHD1L1	6.105	6.148	6.146	6.068	6.296	6.250
TADA2A	8.629	8.706	8.633	8.364	8.385	8.391
TADA2B	9.557	9.644	9.620	9.609	9.656	9.722
NR2C2AP	8.735	8.829	9.037	8.831	8.848	8.843
AC073111.4	7.594	7.806	7.850	7.857	7.702	7.836
AC073111.3	7.543	7.588	7.804	7.736	7.702	7.825
KYAT1	7.601	7.622	7.471	7.585	7.524	7.599
KYAT3	9.952	9.988	9.952	9.769	9.660	9.761
DYNC2LI1	8.923	8.930	8.939	8.554	8.539	8.706
AC008014.1	7.207	7.327	7.419	7.263	7.373	7.327
KIF9	8.914	8.609	8.576	8.826	8.913	8.688
KIF6	7.300	7.201	7.212	7.137	7.348	7.201
KIF7	7.925	7.810	7.870	7.928	8.018	7.974
MIR29B2	7.181	7.193	7.221	7.143	6.856	7.309
MIR934	6.648	6.639	6.479	6.704	6.659	6.636
AP3M1	9.374	9.352	9.392	9.204	9.207	9.187
AP3M2	8.711	8.652	8.796	8.359	8.403	8.507
TRIM43B	6.793	6.730	6.758	6.858	6.755	6.999
S100Z	6.597	6.614	6.522	6.552	6.686	6.698
S100P	12.443	12.600	12.733	12.876	12.933	12.928
TNKS1BP1	8.603	8.565	8.719	8.276	8.558	8.463
AC093724.3	7.972	7.912	8.183	8.328	8.259	8.126
LINC01607	5.976	5.887	6.038	6.110	6.197	6.004
LINC01600	6.398	6.310	6.390	6.634	6.394	6.243
ABT1	8.945	9.004	9.106	8.910	9.066	9.012
AC099791.1	7.985	7.988	8.029	8.231	8.259	8.166
LAPTM4A	12.231	12.341	12.334	12.218	12.229	12.244
LAPTM4B	12.358	12.447	12.519	12.228	12.091	12.143
AC112176.1	5.552	5.563	5.568	5.684	5.563	5.755
SPATA31E1	5.822	6.118	5.790	5.878	5.997	5.838
AARSD1	9.374	9.330	9.487	9.271	9.228	9.172
ESYT1	10.645	10.725	10.870	10.640	10.553	10.633
ESYT2	10.048	10.231	10.221	9.932	9.808	9.935
ESYT3	5.600	5.485	5.288	5.930	5.643	5.702
CXCR3	8.098	8.222	8.061	8.331	8.271	8.362
CXCR2	4.556	4.549	4.786	5.119	4.968	4.578
CXCR1	8.637	8.911	8.908	8.742	8.910	8.800
CXCR6	7.264	7.371	7.117	7.364	7.490	7.406
CXCR5	7.653	7.616	7.558	7.470	7.747	7.619
CXCR4	9.030	9.059	9.113	9.032	9.150	9.117
CLRN3	7.842	7.757	7.937	7.444	7.569	7.666
CLRN1	8.891	8.613	8.703	8.869	8.941	8.895
AC093690.1	6.275	6.233	6.221	6.049	6.386	6.073
AL136171.2	5.085	5.467	5.006	5.153	5.285	5.230
CRYBB2	7.727	7.657	7.822	7.543	7.478	7.646
CRYBB3	7.764	7.723	7.884	8.156	7.865	7.859
CRYBB1	7.159	7.283	7.143	7.403	7.266	7.198
DEFB107B	4.217	3.957	4.044	4.390	4.598	4.193
DEFB107A	4.217	3.957	4.044	4.390	4.598	4.193
MAPKBP1	8.049	8.037	7.843	8.130	7.879	8.036
LINC00559	5.963	5.536	5.794	6.030	5.839	6.053
LINC00558	4.188	3.949	4.100	3.932	3.993	4.343
LINC00557	6.735	6.702	6.514	6.760	6.870	6.888
LINC00556	6.276	6.261	6.198	6.206	6.281	6.173

LINC00555	6.481	6.419	6.493	6.588	6.493	6.451
IFI44	10.677	10.615	10.605	10.721	10.598	10.610
LINC00551	3.713	3.757	3.856	3.739	3.640	3.696
LINC00550	3.834	3.996	3.730	3.803	3.829	3.803
EVA1A	7.038	7.154	6.821	7.050	6.786	7.199
EVA1B	8.239	8.126	8.328	8.120	8.227	8.228
EVA1C	5.200	5.334	5.362	5.316	5.611	5.400
C5AR2	5.187	5.235	5.386	5.091	5.350	5.248
ACSM3	9.196	9.257	9.324	9.075	8.929	9.016
AC022144.1	4.824	4.788	4.781	4.794	4.875	4.728
HAGHL	8.129	8.086	8.386	8.189	8.125	8.269
PALM	8.280	8.328	8.174	8.385	8.187	8.392
PABPC4L	4.707	4.804	4.808	4.710	4.991	4.740
PPY	5.848	5.996	6.050	6.066	6.044	6.238
AC139834.1	4.546	4.452	4.404	4.410	4.521	4.445
SLC9B1	5.861	5.785	5.791	6.273	6.001	6.099
SLC9B2	8.897	8.909	8.869	8.622	8.716	8.623
YTHDF2P1	11.486	11.478	11.615	11.351	11.379	11.337
CR774181.1	8.529	8.524	8.065	8.442	8.567	8.533
SP2-AS1	7.681	7.711	7.842	7.615	7.682	7.404
LAYN	7.333	7.303	7.118	7.322	7.117	7.332
LUM	4.708	4.709	4.698	4.592	4.984	4.677
CLDN23	11.203	11.270	11.331	11.157	11.154	11.068
KRBOX1-AS1	7.739	7.801	7.450	7.832	7.815	7.684
AC096531.2	4.354	4.120	4.348	4.395	4.680	4.341
VNN2	8.632	8.558	8.787	8.681	8.602	8.689
VNN3	7.151	7.081	6.756	7.353	7.028	7.160
LINC01647	6.382	6.476	6.470	6.591	6.621	6.547
KLRG1	7.351	7.433	7.282	7.381	7.423	7.495
RETNLB	7.197	7.289	7.071	6.971	7.151	7.224
TBC1D10B	10.634	10.755	10.721	10.619	10.782	10.539
C18orf32	10.592	10.629	10.839	10.575	10.579	10.577
WDYHV1	9.358	9.596	9.655	9.412	9.346	9.426
AC092881.1	5.581	5.621	5.344	5.875	5.929	5.782
MIRLET7BHG	7.569	7.654	7.574	7.552	7.590	7.376
NOP9	8.371	8.233	8.344	8.251	8.175	8.217
GPBP1	10.065	10.091	10.194	10.179	10.180	10.190
AC099654.2	6.605	6.723	6.687	6.753	6.698	6.645
AC010132.3	12.535	12.649	12.727	12.589	12.551	12.615
FO681491.1	5.821	6.035	5.659	6.080	5.739	5.988
DPP10-AS1	5.068	5.437	5.500	5.711	5.343	5.473
DPP10-AS3	6.205	6.218	6.123	6.352	6.509	6.395
CEP250	8.781	8.743	8.932	8.793	8.677	8.813
AC079781.4	6.451	6.418	6.257	6.244	6.283	6.307
AC079781.5	10.043	9.984	10.015	9.568	9.652	9.804
LGALS8-AS1	7.836	7.735	7.873	7.934	7.878	8.096
UHRF1	9.726	9.736	9.849	9.472	9.440	9.514
UHRF2	9.978	9.960	9.927	9.996	10.012	10.043
HHLA2	7.363	7.309	7.387	7.439	7.190	7.332
HHLA3	9.097	9.051	9.250	9.234	9.380	9.286
HHLA1	8.698	8.858	8.726	8.813	8.729	8.849
AL133410.1	5.720	5.436	5.243	5.080	4.880	5.216
AC008738.7	7.864	7.993	8.252	8.360	8.449	8.096
AL133410.3	10.920	11.009	11.206	10.897	10.871	10.720
DSCC1	7.869	7.810	8.110	7.944	7.624	7.794
AL672167.1	6.517	6.543	6.395	6.499	6.649	6.716

DNM1P41	6.110	6.335	6.440	6.542	6.496	6.649
DNM1P46	6.984	6.706	6.787	6.460	6.712	6.827
PRSS1	10.553	10.631	10.729	10.714	10.691	10.778
PRSS3	10.896	10.976	11.115	11.007	10.960	10.937
PRSS2	11.058	11.116	11.226	11.170	11.181	11.077
TSLP	4.086	3.898	3.968	4.076	3.869	3.933
PRSS8	11.380	11.564	11.667	11.392	11.453	11.307
AL137013.1	8.934	8.971	9.168	8.832	8.933	8.613
RB1	9.858	9.888	9.888	9.421	9.602	9.681
MC4R	6.670	6.821	6.543	6.533	7.173	6.876
AC093802.3	6.745	6.928	6.889	6.743	6.925	6.824
VIRMA	9.039	8.979	9.061	9.004	8.729	8.904
SPATA31A6	6.077	6.174	6.080	6.325	6.400	6.411
PPT2-EGFL8	7.787	7.777	7.897	8.212	8.057	7.746
MAT2A	10.669	10.696	10.714	10.499	10.358	10.617
HEG1	6.932	7.189	7.183	7.461	7.213	7.434
CFAP298	9.815	9.779	9.947	9.643	9.806	9.477
MAT2B	11.636	11.670	11.752	11.695	11.625	11.597
CASC3	12.410	12.459	12.487	12.171	12.032	12.085
CASC2	5.685	5.770	5.635	5.598	5.796	5.728
CASC1	4.405	4.371	4.434	4.525	4.376	4.442
ARR3	7.786	7.840	7.912	7.930	7.935	7.785
CASC6	5.763	6.024	5.837	5.872	6.091	6.104
CASC4	11.369	11.371	11.450	11.145	11.045	11.371
PXMP2	10.098	10.032	10.305	9.861	9.765	9.915
PXMP4	10.419	10.556	10.542	10.368	10.314	10.382
NUDT13	4.880	4.639	4.759	4.818	4.779	4.883
WDR47	7.449	7.478	7.663	7.132	6.784	7.332
NUDT11	7.377	7.358	7.319	7.400	7.303	7.519
CYP2C19	7.130	7.115	6.984	7.057	7.128	7.188
CYP2C18	9.386	9.494	9.465	9.090	9.201	9.298
NUDT16	9.337	9.382	9.387	9.290	9.328	9.239
TCIM	4.211	4.076	4.102	4.107	4.030	3.986
NUTM1	8.650	8.677	8.757	8.830	8.677	8.850
FAM160A2	9.139	9.103	9.136	8.949	8.970	9.111
NAALADL2	7.722	7.915	7.898	7.165	7.432	7.581
NAALADL1	7.599	7.549	7.557	7.631	7.752	7.647
KNCN	8.526	8.443	8.028	8.258	8.423	8.507
CYMP	6.637	6.991	7.004	7.063	6.973	6.908
FAM50B	8.880	8.994	9.117	8.760	8.834	8.782
FAM50A	10.922	10.935	11.079	10.774	10.725	10.732
AC034102.1	11.043	11.060	11.192	10.961	10.928	10.915
AC015871.3	8.072	8.048	8.053	7.825	7.977	8.186
AC015871.5	6.628	6.578	6.745	6.919	6.831	6.827
AQP6	8.802	8.884	8.729	8.902	8.975	8.841
AQP7	8.108	8.089	8.007	7.989	8.099	8.301
AQP4	6.087	6.188	6.082	6.118	6.385	6.050
AQP5	10.060	10.263	10.434	10.165	10.311	10.137
AQP2	8.603	8.772	8.661	8.703	8.685	8.673
AQP3	8.980	8.916	9.037	9.087	9.129	9.076
AQP1	6.868	7.166	6.951	6.978	7.013	7.173
AQP8	6.446	6.461	6.393	6.536	6.767	6.600
AQP9	7.607	7.700	7.427	7.734	7.828	7.850
SNX18P7	5.624	5.551	5.579	5.667	5.364	5.778
MPV17L	10.706	10.742	10.648	10.637	10.664	10.731
AL645949.2	7.049	7.208	7.348	7.217	7.089	7.354

AL645949.1	3.871	3.876	3.786	3.863	3.946	3.952
GSC	6.717	6.970	6.786	6.905	6.857	7.035
ETNPPL	4.743	4.612	4.467	4.645	4.759	4.463
SNHG5	10.756	10.859	11.059	10.752	10.584	10.774
SNHG4	9.302	9.334	9.367	9.326	9.456	9.432
SNHG7	9.003	9.179	9.324	8.937	9.073	8.986
SNHG6	9.957	9.912	10.137	9.926	9.708	9.985
SNHG1	9.959	9.803	9.788	9.844	9.766	9.997
GUCY1B1	9.363	9.315	9.364	9.397	9.320	9.417
POM121L12	10.822	10.894	10.978	11.151	10.990	11.072
RGPD4-AS1	4.506	4.559	4.670	4.604	4.449	4.454
SNHG9	8.530	8.516	8.536	8.482	8.518	8.624
SNHG8	8.553	8.568	8.746	8.367	8.345	8.366
ADORA1	7.565	7.891	7.848	8.317	7.734	7.916
ADORA3	7.576	7.604	7.312	7.806	7.534	7.771
PNRC2P1	8.987	8.930	9.059	8.990	8.923	9.042
POM121	10.486	10.539	10.542	10.343	10.291	10.297
AL162731.1	8.133	8.243	8.349	8.600	8.309	8.374
BTNL9	9.077	8.930	8.814	9.074	9.089	9.121
BTNL8	8.609	8.336	8.274	8.795	8.655	8.736
IGHV3-47	5.720	6.024	5.961	6.260	6.210	6.080
BTNL3	8.204	8.233	8.132	8.429	8.324	8.316
BTNL2	6.768	6.598	6.540	6.777	6.848	6.929
AL731533.2	5.982	6.139	6.261	6.402	6.280	6.172
AAMDC	8.530	8.375	8.535	8.446	8.333	8.429
ACTC1	8.377	8.300	8.526	8.295	8.437	8.434
DSN1	8.469	8.550	8.658	8.016	8.529	8.193
SPANXC	7.833	7.992	8.035	8.055	8.070	8.173
FHL2	11.990	12.077	12.140	12.019	12.022	11.959
FHL3	8.350	8.377	8.408	8.437	8.455	8.561
FHL1	7.155	7.155	7.147	7.124	7.265	7.298
FHL5	5.185	5.219	5.083	5.501	5.245	5.324
FTH1	13.980	13.991	14.082	13.979	13.810	13.985
RPL22P1	13.111	13.257	13.313	13.229	13.072	13.208
ZNF248	6.970	6.797	6.879	6.816	6.899	6.840
BCLAF1P2	10.477	10.240	10.238	10.103	10.442	10.215
BCLAF1P1	8.654	8.726	8.555	8.212	8.600	8.278
AL591895.1	9.114	9.061	9.136	8.853	8.856	8.765
PAWR	11.556	11.550	11.527	11.315	11.307	11.469
RAD51D	7.495	7.636	7.606	7.331	7.780	7.598
RAD51C	9.126	9.188	9.217	8.938	8.958	8.965
RAD51B	7.257	7.344	7.311	7.204	7.466	7.462
GATAD2B	9.665	9.604	9.608	9.582	9.787	9.672
GATAD2A	10.952	10.969	11.081	10.886	10.850	10.825
ARL4A	9.945	9.938	9.983	10.057	10.090	9.903
DNA2	8.129	7.993	7.692	7.760	7.576	7.557
TYMSOS	6.381	6.258	6.498	6.647	6.430	6.332
KCNS1	9.040	8.811	8.782	8.951	9.102	8.925
KCNS2	7.617	7.663	7.336	7.743	7.916	7.776
PKIA-AS1	3.946	3.816	3.766	3.761	3.894	3.875
C17orf64	6.651	6.730	6.502	6.771	6.883	6.845
C17orf67	8.225	8.093	8.146	8.543	8.440	8.595
SPOP	10.720	10.597	10.815	10.799	11.034	10.481
AC138150.1	8.454	8.881	9.023	8.626	8.942	8.545
SLCO2A1	7.625	7.815	7.676	7.845	7.857	7.893
SNORA50C	7.575	7.482	7.794	7.711	7.576	7.622

TP53BP1	8.908	8.912	9.040	9.071	8.807	9.054
TP53BP2	8.737	8.944	8.901	9.082	8.934	9.030
IPPK	8.782	8.800	8.940	8.923	8.949	9.041
AC026523.1	4.808	4.683	4.709	5.013	4.758	4.763
AC026523.2	5.648	5.615	5.713	5.876	5.809	5.813
THAP5P1	4.199	4.271	4.503	4.404	4.279	4.568
RAMP2-AS1	5.351	5.396	5.266	5.328	5.505	5.444
CASQ2	7.801	7.737	7.501	7.661	7.805	7.850
AC007879.3	5.378	5.289	5.300	5.492	5.451	5.508
AC092332.1	5.477	5.618	5.319	5.392	5.493	5.408
GLRX5	11.936	11.918	12.035	11.816	11.776	11.783
NPIPP1	11.306	11.174	11.094	11.070	11.186	11.250
GLRX2	9.757	9.904	9.867	9.850	9.682	9.781
GLRX3	10.566	10.650	10.760	10.591	10.508	10.524
PIH1D2	4.774	5.013	5.141	4.953	5.061	4.901
PIH1D3	5.811	5.941	5.710	6.080	6.135	6.093
PIH1D1	10.801	10.926	11.096	10.612	10.644	10.580
S100A11	12.659	12.769	12.800	12.842	12.841	12.833
S100A10	13.046	13.096	13.145	13.183	13.125	13.076
S100A13	10.315	10.474	10.681	10.373	10.415	10.418
S100A12	5.861	6.083	5.805	6.050	6.220	5.998
S100A14	11.178	11.273	11.440	11.329	11.500	11.486
PHYHIP	7.183	6.960	7.289	7.519	7.361	7.461
S100A16	12.823	12.967	13.064	13.027	13.008	12.995
AC084876.2	5.108	5.071	5.018	5.108	4.940	4.974
AC090518.1	5.356	5.464	5.371	5.525	5.892	5.556
CACNA2D1	7.988	7.915	7.998	7.744	7.819	7.704
GRK6	10.030	10.200	10.080	9.978	10.055	10.119
GRK7	4.558	4.621	4.396	4.375	4.589	4.393
GRK4	6.523	6.668	6.475	6.432	6.591	6.596
GRK5	8.411	8.585	8.481	8.327	8.619	8.483
GRK2	10.724	10.633	10.624	10.696	10.820	10.670
GRK3	8.260	8.120	8.350	8.185	8.239	8.234
GRK1	7.341	7.306	7.499	7.836	7.499	7.570
IPO5P1	6.761	6.854	6.773	6.555	6.705	6.456
RSPH9	5.391	5.578	5.717	5.593	5.737	5.705
CTAG1A	6.815	6.772	6.893	7.072	6.972	6.767
CTAG1B	6.815	6.772	6.893	7.072	6.972	6.767
AC125807.2	8.147	8.220	8.371	8.150	8.294	8.066
KLHL6	6.910	6.877	7.073	6.920	6.983	7.129
KLHL7	9.797	9.896	10.007	9.901	9.963	9.982
KLHL4	6.513	6.720	6.610	6.532	6.609	6.652
KLHL5	10.175	10.296	10.273	10.255	10.288	10.205
KLHL2	8.676	8.707	8.829	8.410	8.506	8.485
KLHL3	7.951	7.968	8.012	7.812	7.719	7.975
LINC01957	5.275	5.322	5.387	5.471	5.437	5.483
KMT5AP1	8.975	8.923	9.166	9.006	8.941	8.856
KLHL8	8.830	8.938	8.875	8.797	8.837	8.713
KLHL9	10.649	10.614	10.673	10.566	10.418	10.515
GABPAP	6.440	6.417	6.517	6.267	6.255	6.422
AC007161.3	6.561	6.464	6.655	6.781	6.714	6.707
AP003066.1	6.029	5.935	5.967	6.078	5.949	5.906
RSPH10B2	9.485	9.466	9.613	9.501	9.346	9.296
LINC00028	7.509	7.624	7.618	7.742	7.563	7.996
ZDHHC11B	7.209	7.101	6.882	7.539	7.023	7.215
MATN1-AS1	5.922	5.806	5.651	5.846	5.806	5.538

MAMDC2	4.219	4.043	4.183	4.245	4.287	4.340
AP000695.3	4.442	4.536	4.754	4.720	4.873	4.695
LYG1	7.367	7.536	7.513	7.573	7.525	7.506
LYG2	5.770	6.084	5.907	5.969	6.096	5.994
TCTN2	9.649	9.666	9.996	9.780	10.084	9.924
TCTN3	9.913	10.038	10.106	9.921	9.816	9.867
TCTN1	9.709	9.729	9.853	9.838	9.804	9.766
TNXB	8.923	8.954	9.136	9.221	9.166	9.008
LHFPL1	5.264	5.244	5.373	5.461	5.596	5.301
FATE1	7.301	7.495	7.453	7.592	7.379	7.486
AL929472.3	6.920	6.879	6.827	6.990	7.266	7.175
FAM133DP	9.535	9.496	9.496	9.402	9.565	9.304
XRN2	8.579	8.628	8.795	8.528	8.584	8.521
SERPINB11	4.983	5.317	5.324	5.311	5.311	5.332
SERPINB10	7.468	7.462	7.147	7.574	7.455	7.481
SERPINB13	6.670	6.928	6.948	6.997	6.932	6.910
SERPINB12	7.559	7.433	7.632	7.375	7.553	7.805
AC136443.1	9.154	9.228	9.331	8.950	9.052	9.023
AP002478.1	5.228	5.484	5.336	5.547	5.480	5.625
AL109615.3	6.087	6.184	6.274	6.171	6.294	6.002
AL109615.2	7.922	7.811	7.775	8.412	8.115	8.044
AFG3L1P	7.830	7.831	7.904	7.839	7.834	8.099
LRMP	8.218	8.242	8.140	8.335	8.341	8.490
BSCL2	9.989	10.037	10.120	9.921	9.898	9.914
AC135279.4	6.190	6.296	6.159	6.270	6.090	6.287
AC147651.4	7.783	7.866	7.894	7.956	7.918	8.062
RDM1P5	7.241	7.190	7.232	7.454	7.247	7.401
GSTT2B	7.413	7.486	7.589	7.314	7.369	7.502
ADGRA1	6.571	6.748	6.716	6.838	6.809	6.913
ADGRA3	8.698	8.727	8.801	8.600	8.570	8.572
ADGRA2	8.908	8.688	8.241	8.786	8.845	8.632
BPIFB9P	6.740	6.840	6.630	6.673	6.864	6.897
LACTB2-AS1	4.068	4.079	4.245	4.229	4.448	4.554
ISCA1P6	7.976	7.964	8.114	7.950	8.005	8.007
SLC32A1	5.969	5.938	5.901	6.008	6.207	6.201
TOP3BP1	7.548	7.380	7.762	7.392	7.440	7.365
ISCA1P1	7.976	7.964	8.114	7.950	8.005	8.007
TAS2R64P	4.416	4.272	4.122	4.192	4.103	4.420
PABPC1	13.739	13.741	13.741	13.595	13.779	13.647
PABPC3	13.089	13.047	13.074	12.968	13.081	13.023
DDB2	8.277	8.409	8.382	8.202	8.015	8.127
MPV17L2	8.291	8.376	8.378	8.178	8.301	8.380
FAM187A	7.430	7.493	7.577	7.352	7.646	7.518
SYNE1	8.426	8.475	8.472	8.058	8.181	7.994
SYNE2	9.806	9.769	9.826	9.538	9.869	9.327
FAM187B	7.425	7.274	7.441	7.380	7.413	7.469
SYNE4	7.900	8.141	7.853	8.041	7.958	7.966
CAMKMT	9.321	9.263	9.173	9.130	9.154	9.339
AL669918.1	7.208	6.909	6.524	7.159	7.109	7.189
RIMS3	7.215	7.231	7.261	7.086	7.306	7.373
FAM238B	7.309	7.204	7.127	7.181	7.333	7.337
FAM238C	6.840	6.912	6.982	7.072	6.900	7.215
ABI1	10.232	10.271	10.305	10.097	10.131	10.231
AVL9	10.327	10.369	10.435	10.206	10.230	10.368
UBQLN2	10.156	10.238	10.171	9.852	10.160	10.007
UBQLN3	5.971	5.751	5.818	6.027	6.146	5.999

UBQLN1	10.919	11.064	11.005	10.774	10.729	10.727
UBQLN4	9.190	9.118	9.244	9.113	8.946	9.171
AL356118.1	4.658	4.563	4.628	4.696	4.561	4.525
DAPK3	8.515	8.691	8.755	8.459	8.528	8.486
DAPK2	7.921	7.639	7.775	7.851	7.938	7.785
DAPK1	11.054	11.015	11.121	10.796	10.792	10.968
ULK2	7.250	7.124	6.951	7.132	7.134	7.154
SNTA1	8.753	8.796	9.084	8.627	8.676	8.539
ULK1	7.909	7.591	7.854	7.768	7.607	7.689
ULK4	7.034	7.249	7.249	7.448	7.267	7.488
TNR	7.436	7.458	7.435	7.701	7.638	7.617
ZFPL1	9.767	9.779	9.786	9.723	9.738	9.595
TNF	7.704	7.745	7.757	8.120	8.219	8.144
MYNN	9.339	9.371	9.405	9.648	9.587	9.753
TNN	5.491	5.553	5.601	5.584	5.631	5.512
RNLS	9.385	9.525	9.503	9.199	9.434	9.372
DHDDS-AS1	6.954	7.095	6.918	6.996	6.971	7.055
UBL7	9.642	9.642	9.781	9.546	9.462	9.508
GOLPH3	11.457	11.533	11.607	11.529	11.441	11.567
UBL3	9.459	9.584	9.446	9.145	9.006	9.223
KRT38	7.818	7.915	7.554	7.944	7.907	7.902
STK10	7.856	8.111	8.002	7.751	7.812	7.926
STK11	9.773	9.809	9.961	9.671	9.694	9.749
STK16	8.740	8.854	9.077	8.916	8.898	8.795
JSRP1	7.686	7.459	7.706	8.172	7.925	7.757
LINC02560	7.479	7.506	7.524	7.513	7.604	7.438
LINC02562	6.005	6.057	6.105	6.025	5.902	5.774
SV2A	8.249	7.980	8.212	8.236	8.323	8.441
SV2C	7.324	7.426	7.267	7.551	7.546	7.520
SV2B	7.644	8.026	7.527	7.647	7.747	7.934
REXO1L1P	5.953	6.007	5.944	5.911	6.010	5.917
SNORD3B-1	7.100	7.324	7.207	7.239	7.288	7.394
FNDC10	8.271	8.205	8.409	8.217	8.276	8.144
FNDC11	6.900	7.138	7.264	7.003	7.049	7.252
EHF	12.435	12.447	12.518	12.218	12.188	12.339
SNORD3B-2	7.100	7.324	7.207	7.239	7.288	7.394
GRB10	10.514	10.493	10.509	10.395	10.334	10.352
GRB14	8.273	8.151	8.341	7.992	8.153	8.397
AC009533.1	10.054	10.138	10.088	9.791	9.917	9.889
AC004590.1	6.615	6.672	6.548	7.130	6.882	6.854
HSD17B12	12.504	12.565	12.649	12.651	12.596	12.469
GPR15	6.818	6.764	6.769	6.802	6.824	6.747
HSD17B11	10.916	11.040	11.140	10.827	10.885	10.789
GPR12	7.143	7.343	6.893	7.199	7.527	7.224
HSD17B14	7.754	7.684	7.853	8.070	7.866	7.786
GPR19	7.876	7.770	7.777	7.871	7.935	7.908
GPR18	4.443	4.376	4.325	4.530	4.689	4.446
AC012485.1	6.871	6.833	6.822	7.009	6.994	6.750
ARNTL	8.905	8.824	8.626	8.733	8.923	8.767
KCNMA1	7.392	7.446	7.515	7.983	7.630	7.646
PCYOX1L	9.380	9.396	9.513	9.187	9.052	9.112
HEATR5A	8.795	8.807	8.739	8.613	8.599	8.947
HEATR5B	8.145	8.128	8.114	7.824	7.807	7.781
ARNT2	6.658	6.394	6.480	6.515	6.617	6.578
PLXNC1	5.850	6.162	5.999	5.577	6.069	5.701
ZNRF4	7.951	8.151	8.068	8.061	7.961	7.926

ZNRF1	9.334	9.387	9.505	9.266	9.511	9.258
ZNRF3	9.230	9.396	9.385	8.879	9.029	9.140
ZNRF2	7.915	8.072	8.205	7.715	8.038	7.964
MYBBP1A	8.099	8.253	8.408	8.092	8.159	8.200
EFCAB5	6.275	6.317	6.224	6.523	6.287	6.609
FLNA	12.059	12.151	12.270	11.999	11.783	11.963
FLNB	9.311	9.283	9.230	9.116	8.961	9.222
FLNC	8.196	8.283	8.386	8.242	8.450	8.285
EFCAB1	7.081	7.054	6.842	7.096	7.233	7.142
EFCAB3	4.806	4.827	4.624	4.715	4.847	4.648
EFCAB2	7.073	6.945	7.162	7.014	7.034	6.909
MIR34C	6.004	5.936	6.084	6.233	6.379	6.234
MIR34B	6.004	5.936	6.084	6.233	6.379	6.234
LETM1	8.863	8.740	8.907	8.859	8.640	8.633
GRTP1	7.275	7.225	7.394	6.625	6.951	6.987
LETM2	9.699	9.703	9.764	9.649	9.800	9.541
PRKCSH	11.207	11.241	11.308	11.204	11.203	11.166
RAD51AP1	9.667	9.489	9.616	9.015	8.940	8.991
SAP30BP	9.157	9.175	9.248	8.925	9.040	8.958
AC004510.1	12.228	12.251	12.384	12.278	12.266	12.208
SIT1	7.624	7.781	7.747	8.064	7.958	7.697
PHEX-AS1	6.470	6.636	6.581	6.839	6.792	6.871
CXorf21	5.971	5.871	5.983	6.172	5.621	5.882
PSMD9	10.783	10.818	11.070	10.578	10.571	10.708
PSMD8	11.648	11.758	11.839	11.682	11.616	11.644
PSMD5	10.031	10.090	10.137	9.863	9.862	9.789
PSMD4	11.481	11.542	11.690	11.319	11.096	11.287
PSMD7	11.284	11.325	11.379	11.064	11.119	11.016
CYB5AP4	4.957	4.647	4.954	4.833	4.964	5.014
PSMD1	10.992	11.079	11.231	11.042	11.244	10.957
PSMD3	13.914	13.985	14.028	13.944	13.927	13.969
PSMD2	11.634	11.680	11.739	11.521	11.340	11.480
TWF1P1	9.622	9.941	9.741	9.834	9.891	9.660
AC006026.3	8.213	8.179	8.242	7.928	7.974	8.025
SLC51A	7.117	6.846	6.702	6.827	7.073	6.868
DHDDS	8.564	8.638	8.591	8.572	8.573	8.516
RPGRIP1L	7.052	7.123	6.884	7.001	6.694	6.661
TIMP4	7.067	7.106	6.689	6.952	6.968	7.084
COPS7B	9.106	9.187	9.194	9.064	9.146	8.993
COPS7A	10.221	10.329	10.391	10.161	10.159	10.099
NAF1	8.411	8.210	8.416	8.054	8.112	8.380
AC087521.1	7.139	7.380	7.339	7.229	7.294	7.241
AL109920.3	5.500	5.406	5.581	5.473	5.610	5.359
OR5E1P	7.025	7.020	7.003	6.997	6.950	7.198
DKK4	6.245	6.479	6.356	6.412	6.409	6.217
SPIRE1	10.102	10.104	10.213	9.958	9.817	9.935
DKK3	8.423	8.386	8.546	8.008	7.941	8.125
SPIRE2	8.239	8.337	8.398	8.147	8.388	8.183
RABGGTB	10.576	10.563	10.649	10.407	10.434	10.556
RABGGTA	8.939	9.002	9.177	8.672	8.846	8.777
KDM7A	7.461	7.345	7.407	7.282	7.273	7.292
AC027020.2	4.512	4.745	4.800	4.789	4.848	4.983
EBLN2	7.629	7.660	7.632	7.694	7.656	7.603
AC012651.1	7.120	7.136	7.288	7.300	7.122	7.328
ATP8B5P	11.160	11.120	11.194	11.199	11.142	11.293
AL137782.1	10.187	9.960	9.981	9.764	10.038	9.712

EIF4EP2	10.849	10.892	10.958	10.870	10.800	10.938
PLBD2	10.229	10.101	9.912	9.834	9.820	9.962
PLBD1	11.592	11.634	11.815	11.570	11.398	11.447
PTAR1	9.861	9.860	9.927	9.461	9.467	9.688
CEP350	9.406	9.471	9.488	9.164	9.514	9.109
YEATS2	9.134	9.128	9.276	9.019	9.119	9.081
DHRS4L1	9.786	9.800	9.901	9.508	9.462	9.477
DHRS4L2	9.786	9.800	9.901	9.508	9.462	9.477
CTAGE15	10.096	10.073	10.117	10.027	10.081	10.109
IL10RB-DT	6.397	6.218	6.335	6.490	6.433	6.411
TCEANC	6.749	6.807	6.709	6.792	6.777	6.769
VPS13B-DT	6.596	6.384	6.259	6.807	6.539	6.718
SERBP1P1	12.050	12.085	12.112	11.907	11.806	11.884
C11orf58	11.661	11.743	11.787	11.620	11.573	11.609
SERBP1P5	12.050	12.085	12.112	11.907	11.806	11.884
OPRPN	6.659	6.368	6.563	7.019	6.942	6.911
AC007595.1	7.018	6.714	6.855	6.859	6.607	6.864
C11orf53	8.323	8.342	8.529	8.340	8.352	8.365
C11orf52	8.371	8.462	8.565	8.135	8.220	8.315
CNPPD1	9.814	9.799	9.946	9.711	9.649	9.747
RAD54L2	8.512	8.573	8.555	8.568	8.512	8.600
TUBB	13.665	13.649	13.663	13.496	13.381	13.448
HDAC1	11.804	11.923	12.024	11.692	11.612	11.692
HDAC3	9.895	9.936	9.979	9.741	9.762	9.792
HDAC2	10.356	10.283	10.469	10.214	10.299	10.102
HDAC5	8.552	8.593	8.674	8.857	8.689	8.678
HDAC4	7.499	7.482	7.486	7.528	7.682	7.714
HDAC7	8.887	8.809	9.061	9.050	9.010	8.809
HDAC6	10.012	9.946	9.923	9.900	9.822	9.941
HDAC9	10.667	10.793	10.862	10.978	10.890	10.948
HDAC8	8.505	8.463	8.742	8.614	8.425	8.336
TMEM161A	9.673	9.775	9.833	9.588	9.751	9.809
TMEM161B	9.532	9.670	9.705	9.433	9.585	9.594
KIAA1549L	8.067	8.081	8.093	7.762	8.163	8.065
AC123912.2	6.677	6.898	6.878	6.750	6.800	6.900
FILIP1	6.413	6.460	6.626	6.711	6.472	6.643
UPF3AP2	8.166	8.131	8.061	7.564	7.605	7.719
CDT1	10.753	10.548	10.528	10.568	10.726	10.536
POLD2	10.688	10.753	10.969	10.570	10.332	10.679
CST8	7.642	7.672	7.625	7.987	7.882	7.971
CST9	6.484	6.437	6.591	6.594	6.646	6.557
TRAPPC11	8.208	8.177	8.188	7.909	7.960	8.089
TRAPPC10	7.743	7.885	7.833	7.766	7.702	7.720
TRAPPC13	6.758	6.806	6.814	6.742	6.812	7.163
TRAPPC12	9.324	9.477	9.619	9.142	9.122	9.118
CST2	7.342	7.597	7.597	7.487	7.498	7.462
LINC01226	7.267	7.323	7.256	7.112	7.311	7.150
CST1	5.540	5.580	5.857	5.579	5.766	5.692
CST6	9.905	9.993	10.232	10.025	9.830	9.980
CST7	6.828	6.863	6.844	6.969	6.929	6.986
CST4	7.062	7.288	7.209	7.017	7.054	7.033
MGAT4EP	4.219	4.169	4.282	4.379	4.388	4.157
PLA2R1	5.494	5.495	5.789	6.060	5.717	5.550
CSTA	5.907	5.927	5.738	5.749	6.069	5.966
SUMO1P2	5.575	5.512	5.425	5.475	5.239	5.628
SUMO1P3	11.662	11.660	11.761	11.772	11.659	11.651

CMC1	9.182	9.108	9.466	9.236	9.404	9.275
CMC2	10.492	10.467	10.611	10.283	10.315	10.424
CMC4	8.885	8.883	9.033	8.878	9.052	8.876
MIR210HG	8.775	8.730	9.028	8.559	8.528	8.875
AMIGO1	7.660	7.655	7.513	7.370	7.667	7.637
CDC20B	7.361	7.577	7.587	7.379	7.418	7.422
AMIGO2	11.006	11.190	11.190	11.128	10.883	11.064
SRCAP	8.983	8.894	9.033	8.981	8.938	8.829
FAM86C1	9.708	9.675	9.729	9.656	9.678	9.650
TSKU	10.619	10.693	10.812	10.758	10.760	10.613
MFF-DT	5.921	6.153	5.904	6.032	5.941	5.946
CEBPB	12.428	12.406	12.602	12.288	12.167	12.237
CEBPA	7.953	7.956	8.029	8.064	7.883	7.985
XRCC2	7.874	7.876	7.789	7.858	7.847	7.877
CEBPG	9.342	9.298	9.479	8.967	9.155	8.998
CEBPE	7.043	7.345	7.334	7.757	7.318	7.399
CEBPD	9.846	9.812	10.090	9.941	10.048	9.914
OSTM1-AS1	4.545	4.574	4.686	4.663	4.583	4.617
ZNF547	6.320	6.398	6.129	6.326	6.685	6.382
PHKB	9.379	9.497	9.514	9.451	9.369	9.336
PPIEL	8.294	8.260	8.456	8.414	8.542	8.536
CECR9	4.974	4.902	4.747	4.934	4.743	4.831
CEBPZ	9.156	9.174	9.285	9.243	9.000	9.117
SLC14A2-AS1	5.885	6.074	5.768	6.140	6.025	5.829
EMSY	8.325	7.895	8.042	8.134	7.986	7.923
EML2-AS1	8.103	7.963	8.188	8.398	8.291	8.026
CRNN	7.011	6.723	6.971	6.797	6.995	7.012
AC093642.2	4.136	4.329	4.011	4.241	4.217	4.217
CTNNA1	12.558	12.576	12.692	12.500	12.366	12.545
CTNNA2	5.569	5.631	5.694	5.953	5.657	5.766
CTNNA3	8.265	8.246	8.243	8.297	8.389	8.324
ZNF548	10.917	10.786	10.341	10.950	11.038	10.900
JPX	6.618	6.297	6.449	6.153	6.382	5.663
AL392089.1	8.707	8.817	8.517	8.598	8.728	8.852
UBAP1L	7.471	7.540	7.796	7.822	7.661	7.880
TOMM40	10.392	10.458	10.581	10.352	10.493	10.299
TM7SF3	11.323	11.438	11.565	11.122	11.205	11.233
TM7SF2	10.197	10.331	10.579	10.215	10.238	10.303
EPB41L4B	8.858	8.917	9.050	8.589	8.660	8.695
EPB41L4A	10.100	10.086	10.180	9.798	9.772	9.721
FIRRE	6.766	6.639	6.617	6.687	6.900	6.795
ATP2C2	10.322	10.405	10.415	10.215	10.191	10.229
PTENP1	9.252	9.370	9.491	9.290	9.165	9.268
ATP2C1	10.515	10.693	10.726	10.606	10.643	10.707
CERS6	11.552	11.620	11.768	11.565	11.573	11.301
CERS4	7.570	7.669	7.845	7.613	7.820	7.760
CERS5	9.317	9.439	9.526	9.287	9.407	9.369
CERS2	11.982	12.028	12.131	11.956	11.933	11.984
CERS3	7.981	7.857	8.062	7.893	7.953	7.874
CERS1	7.718	7.798	7.840	7.674	7.611	7.721
TFB2M	9.309	9.408	9.373	9.214	9.250	9.201
AC005562.1	6.328	6.287	6.244	6.332	6.438	6.541
GP9	7.891	8.102	8.173	8.096	7.995	8.096
GP5	6.533	6.508	6.556	6.742	6.637	6.653
GP6	5.046	4.997	4.787	4.829	4.922	4.863
MAPK8IP3	8.991	8.921	9.082	9.058	8.907	9.064

GP2	7.692	7.546	7.442	7.590	7.564	7.693
VXN	6.483	6.805	6.645	6.614	6.622	6.847
GPI	13.038	13.085	13.177	13.082	13.002	13.065
EXTL1	6.851	6.754	6.602	6.860	6.987	6.831
EXTL2	9.431	9.389	9.272	9.321	9.408	9.381
EXTL3	9.092	9.398	9.302	9.062	9.368	9.012
RGSL1	6.421	6.520	6.405	6.382	6.469	6.452
SNORD83A	13.636	13.622	13.676	13.581	13.420	13.530
SNORD83B	13.607	13.636	13.696	13.542	13.313	13.485
MAN1A1	7.954	7.976	7.907	7.314	7.517	7.630
MAN1A2	9.134	9.039	9.078	8.994	8.876	9.033
PRDM4	8.950	8.926	9.021	8.788	8.833	8.926
ATIC	11.411	11.433	11.493	11.310	11.164	11.294
KCNQ1-AS1	6.573	6.470	6.287	6.317	6.571	6.523
LINC02762	8.123	8.159	8.103	8.141	8.242	8.272
LINC02763	3.966	4.035	4.055	4.148	4.002	4.020
HDAC11	8.586	8.636	8.669	8.647	8.575	8.612
HDAC10	8.228	8.219	8.393	8.439	8.602	8.408
LRTM2	6.903	7.018	6.868	7.372	6.930	7.005
FCGR1B	6.966	6.873	7.018	6.966	6.957	6.852
SNORA71B	10.954	10.723	9.744	10.986	11.001	10.785
SNORA71A	7.939	7.966	7.915	8.264	8.025	8.064
SYNM	7.967	7.834	7.883	7.776	7.627	7.752
SETDB1	9.685	9.732	9.818	9.660	9.781	9.638
AP4B1	8.676	8.770	9.010	8.765	8.683	8.840
ZBP1	7.021	7.079	7.137	7.212	7.072	7.008
AL589182.1	7.031	6.937	6.926	7.183	7.137	7.205
AL589182.3	4.376	4.773	4.668	4.763	4.535	4.427
STMP1	10.477	10.630	10.566	10.515	10.593	10.298
PIK3IP1	8.940	9.134	9.158	9.057	9.136	8.983
ZFAND2B	8.960	8.932	9.163	8.670	8.722	8.728
THADA	9.147	9.164	9.180	9.076	9.107	9.124
ZFAND2A	9.386	9.352	9.431	9.548	9.496	9.562
AC004951.3	8.237	8.211	8.107	8.168	8.183	8.138
ACSF2	9.324	9.281	9.434	9.221	9.142	9.235
ACSF3	7.962	7.991	8.025	8.103	7.880	7.974
MAN2A2	9.450	9.331	9.384	9.394	9.236	9.324
MAN2A1	10.991	11.059	11.187	10.996	11.038	10.526
AL512598.2	5.862	5.841	5.684	5.856	5.618	5.932
EDA2R	7.564	7.687	7.581	7.588	7.779	7.811
LCT	6.892	7.004	6.971	7.114	7.089	7.179
RBFOX3	6.448	6.319	6.353	6.467	6.348	6.461
FAM163A	6.224	6.337	6.341	6.372	6.388	6.369
RBFOX1	8.682	8.346	8.325	8.672	8.651	8.458
SLURP2	8.594	8.658	8.599	8.625	8.724	8.666
SLURP1	6.908	6.922	6.959	7.207	6.852	7.109
LINC01231	7.595	7.780	7.463	7.486	7.706	7.681
LINC01233	4.183	4.208	4.340	4.316	4.259	4.398
RSL1D1	11.185	11.286	11.358	11.229	11.124	11.190
POLE3	9.951	10.115	10.251	9.623	9.947	9.874
CFAP221	7.601	7.638	7.635	7.577	7.594	7.724
NXNL1	8.403	8.526	8.459	8.432	8.510	8.636
LINC01238	6.106	6.241	6.122	6.253	6.481	6.399
NXNL2	4.204	4.035	4.261	3.937	4.233	4.260
SYN2	7.524	7.293	7.108	7.465	7.685	7.562
LCK	7.111	7.361	7.115	7.183	7.127	7.202

SYN1	8.367	8.382	8.323	8.677	8.500	8.544
LCNL1	8.396	8.515	8.374	8.510	8.286	8.439
LINC00184	6.803	6.720	6.715	6.742	6.716	6.955
LINC00189	4.643	4.584	4.597	4.591	4.609	4.665
C12orf10	9.464	9.510	9.612	9.303	9.377	9.260
AL592429.2	5.632	5.959	5.784	5.879	5.966	5.919
AC008555.3	7.486	7.502	7.534	7.564	7.527	7.456
AC008555.1	5.327	5.482	5.308	5.219	5.283	5.366
NPY4R	6.188	6.259	6.188	5.996	6.132	6.303
FLVCR1-DT	8.057	7.974	8.163	7.964	7.814	7.747
STARD5	7.421	7.649	7.620	7.431	7.574	7.452
AL845472.2	8.069	8.342	8.384	8.466	8.460	8.437
RORA-AS1	5.906	5.787	5.883	5.902	5.819	5.827
AC113192.3	7.040	6.985	7.317	7.027	7.258	7.280
GABRB1	4.935	5.051	4.684	5.059	5.217	5.235
GABRB2	7.239	7.297	7.130	7.333	7.340	7.326
GABRB3	5.976	5.883	5.759	6.102	6.137	6.149
KRT223P	9.393	9.443	9.529	9.529	9.495	9.464
AC004918.2	6.847	6.762	7.006	6.884	7.015	7.095
AC004918.1	9.167	9.029	9.060	8.954	9.054	8.917
RBAK-RBAKDN	7.693	7.648	7.832	8.060	7.926	7.866
AC004918.4	9.167	9.029	9.060	8.954	9.054	8.917
AC006840.1	6.538	6.373	6.275	6.396	6.504	6.390
NLRC4	5.336	5.383	5.393	5.439	5.484	5.475
NLRC5	8.030	7.921	7.909	7.912	8.044	7.877
NLRC3	6.081	6.252	6.013	6.053	6.047	6.026
DYDC2	6.018	6.111	6.006	6.040	6.004	6.107
DYDC1	4.061	4.054	4.133	4.102	3.876	4.009
AL157395.1	6.874	7.169	7.092	7.277	7.197	6.967
RAP1GAP	11.238	11.346	11.382	11.054	11.001	10.976
AC022415.2	6.058	6.001	5.831	5.782	5.949	5.666
AC022415.3	6.389	6.624	6.470	6.533	6.576	6.635
AL133243.4	4.429	4.362	4.582	4.459	4.326	4.554
TPRG1	5.479	5.621	5.617	5.946	5.713	5.687
MALT1	8.176	8.110	8.183	8.017	8.204	8.116
NKAIN3-IT1	4.122	3.849	3.770	3.992	3.748	3.994
COL18A1	9.621	9.721	9.827	9.303	9.381	9.320
FP236315.2	4.107	4.171	4.060	4.133	4.131	4.177
MIR6727	9.760	9.808	9.900	9.449	9.429	9.589
Z97353.1	9.651	9.696	9.774	9.594	9.729	9.776
AL133260.1	13.951	13.948	14.015	14.017	14.031	14.037
OPRK1	6.515	6.492	6.324	6.693	6.540	6.690
AL138828.1	4.668	4.644	4.445	4.737	4.733	4.659
DNAH14	6.933	7.069	7.154	6.778	6.719	6.807
DNAH17	6.575	6.689	6.575	6.565	6.859	6.757
DNAH10	7.536	7.442	7.236	7.446	7.491	7.611
PHGDH	10.448	10.498	10.705	10.258	10.219	10.245
DNAH12	7.072	7.186	7.212	6.875	7.111	7.180
AC018755.1	4.100	4.196	4.183	4.433	4.113	4.233
AC018755.2	7.649	7.739	7.938	7.604	7.621	7.653
PGM5-AS1	7.383	7.470	7.517	7.591	7.447	7.467
GLI2	6.820	7.068	6.832	6.623	6.904	6.885
AC011298.1	7.499	7.573	7.379	7.706	7.625	7.635
PART1	7.744	7.705	7.698	7.965	7.686	7.740
EDN1	9.618	9.646	9.820	9.543	9.568	9.590
EDN3	7.457	7.486	7.373	7.552	7.503	7.538

EDN2	7.415	7.279	7.310	7.316	7.454	7.480
PTTG1IP	12.270	12.281	12.458	12.160	12.084	12.157
RSPRY1	10.529	10.521	10.715	10.536	10.563	10.404
DKKL1	7.676	7.478	7.754	7.884	7.761	7.862
FP885919.1	8.108	8.089	8.007	7.989	8.099	8.301
TMEM259	9.728	9.755	9.941	9.508	9.754	9.674
UBE2L6	10.855	10.938	11.070	11.066	11.248	10.974
OPN1MW	6.498	6.624	6.478	6.739	6.566	6.784
CR388415.2	8.529	8.524	8.065	8.442	8.567	8.533
PDGFC	11.253	11.320	11.411	11.116	11.006	11.241
UBE2L3	12.031	12.165	12.312	12.106	12.010	12.060
TGFA	11.230	11.358	11.356	11.238	11.234	11.196
PREX2	5.459	5.354	5.163	5.466	5.472	5.536
PREX1	7.282	7.233	7.354	7.309	7.313	7.274
MFSD4B	7.836	7.749	7.902	7.678	7.949	7.821
POTEH-AS1	5.279	5.017	5.088	5.269	5.142	5.236
MFSD4A	7.901	7.908	7.949	8.209	8.126	8.014
SLC14A2	7.697	7.630	7.627	7.906	7.886	7.829
SLC14A1	5.435	5.201	5.237	5.359	5.215	5.262
EVPL	9.221	9.236	9.318	8.979	9.048	9.022
STT3A	9.668	9.569	9.726	9.525	9.447	9.464
AC002070.1	7.443	7.666	7.725	7.560	7.764	7.610
FAM8A1	9.762	9.788	9.823	9.618	9.582	9.642
AC111200.2	10.130	10.206	10.336	10.332	10.107	10.248
AZGP1P1	7.964	7.926	8.049	8.136	7.778	8.007
TH2LCRR	5.575	5.388	5.297	5.578	5.662	5.589
TUBA8	7.089	6.956	6.854	7.265	7.172	7.028
AC091185.1	5.792	5.932	5.916	5.637	6.055	5.615
DYRK3-AS1	6.025	5.608	5.404	5.557	5.655	5.657
AL159987.1	8.856	9.093	8.775	8.540	8.825	9.021
LINC02249	6.872	6.818	7.024	7.154	6.849	6.969
NUTM2A-AS1	6.207	6.332	6.262	6.119	6.207	6.348
LINC02241	5.983	5.924	5.756	5.885	6.188	6.009
LINC02240	5.861	6.094	5.839	5.837	6.119	6.228
LINC02243	5.850	6.279	5.862	6.080	6.083	6.006
LINC02245	4.615	5.010	4.985	4.990	4.987	4.942
AL078602.1	6.086	6.103	6.361	6.198	6.329	6.344
SLC13A1	3.816	3.882	3.804	4.115	3.833	3.866
SLC13A2	7.032	7.242	7.178	7.346	7.267	7.270
SLC13A3	8.197	8.279	8.124	8.391	8.324	8.422
SLC13A4	7.632	7.398	7.621	7.508	7.768	7.688
SLC13A5	7.403	7.383	7.371	7.298	7.585	7.320
RGS16	7.373	7.676	7.548	7.781	7.654	7.605
RGS17	7.272	7.364	7.278	7.582	7.551	7.512
RGS14	7.114	7.333	7.449	7.384	7.563	7.286
RGS12	7.632	7.747	7.651	7.789	7.818	7.674
RGS13	3.899	3.794	3.852	3.833	3.866	3.964
RGS10	8.232	8.319	8.582	8.342	8.377	8.385
RGS11	7.565	7.583	7.755	7.655	7.558	7.608
RGS18	4.363	4.423	4.367	4.303	4.508	4.591
RGS19	9.329	9.328	9.510	9.319	9.389	9.226
EMX2OS	8.363	8.116	8.451	8.291	8.157	8.347
UTP6	9.339	9.312	9.442	9.304	9.105	9.338
UTP4	11.103	11.070	11.171	10.968	10.921	10.920
UTP3	9.847	9.618	9.725	9.438	9.442	9.451
CLEC16A	8.649	8.727	8.678	8.556	8.528	8.510

VANGL1	9.119	9.211	9.387	9.154	9.134	9.121
ANKAR	5.462	5.437	5.356	5.455	5.576	5.683
BTN2A2	7.635	7.781	7.782	7.433	7.620	7.604
BTN2A1	8.615	8.710	8.710	8.612	8.722	8.669
VANGL2	6.397	6.281	6.300	6.629	6.421	6.567
PDE4DIPP1	7.467	7.590	7.618	7.576	7.832	7.792
TTLL11	7.752	7.777	7.472	7.771	7.830	7.837
TTLL10	8.223	8.257	8.069	8.215	8.193	8.234
TTLL12	10.081	10.226	10.256	10.123	10.093	10.162
ODF2L	7.262	7.248	7.171	7.126	7.072	7.105
DNAH17-AS1	7.458	7.508	7.374	7.365	7.587	7.520
PLAU	12.351	12.479	12.511	12.453	12.366	12.379
PLAT	8.158	8.396	8.434	8.159	8.308	8.158
C1orf198	11.292	11.249	11.370	11.148	11.042	11.159
OR10J1	7.472	7.755	7.597	7.726	7.812	7.801
PLAA	8.773	8.939	8.938	8.631	8.717	8.603
C1orf194	7.383	7.500	7.378	7.685	7.597	7.711
AC094103.1	9.194	9.291	9.371	8.855	8.965	9.128
AC112219.2	7.996	8.134	8.172	8.050	8.236	8.250
DUOXA1	7.499	7.765	7.673	7.735	7.565	7.624
GTF2IP1	10.885	10.859	10.911	10.785	10.745	10.628
GTF2IP4	10.885	10.859	10.911	10.785	10.745	10.628
GTF2IP7	5.977	5.800	6.037	5.958	5.995	5.987
MZT2A	11.059	11.149	11.485	11.009	10.842	10.996
MZT2B	11.072	11.101	11.346	11.058	11.088	11.018
PMM2	9.329	9.406	9.412	9.301	9.157	9.217
PMM1	8.733	8.712	9.068	8.432	8.534	8.496
ZNF709	6.587	6.862	6.969	6.702	7.123	6.785
ZNF708	5.177	4.990	5.174	4.861	4.992	5.015
ZNF703	8.037	7.723	8.025	8.009	8.039	7.929
ZNF701	6.498	6.469	6.466	6.193	6.461	6.545
ZNF700	6.592	6.643	6.584	6.419	6.579	6.830
ZNF707	7.790	7.746	7.770	7.773	7.754	7.694
ZNF706	10.611	10.700	10.759	10.627	10.646	10.594
ZNF704	8.080	7.748	7.810	7.854	7.807	7.812
ZC3H10	14.596	14.604	14.644	14.671	14.651	14.656
RNF114	10.558	10.560	10.596	10.460	10.568	10.396
RNF115	9.588	9.705	9.776	9.616	9.697	9.484
RNF112	6.763	6.824	6.712	6.784	6.796	6.844
ZC3H14	9.841	9.841	9.814	9.875	9.925	9.772
RNF111	9.848	9.954	9.960	9.845	9.732	9.941
ZC3H18	8.902	9.030	9.072	8.800	9.035	8.800
GRIN1	8.377	8.342	8.528	8.441	8.561	8.483
DHX8	8.940	8.951	9.110	8.766	8.692	8.743
DHX9	10.119	10.161	10.255	10.167	10.088	10.186
NUP98	9.489	9.429	9.536	9.374	9.362	9.408
XPC	9.240	9.350	9.414	9.152	9.150	9.198
GRINA	10.588	10.591	10.714	10.905	10.994	10.770
NUP93	9.872	9.952	9.958	9.773	9.800	9.773
RAB40C	9.000	9.043	9.152	8.831	8.783	8.926
AC092916.2	10.587	10.543	10.739	10.633	10.579	10.541
RAB40A	6.107	6.246	6.359	6.217	6.082	6.220
GTSE1	9.607	9.481	9.588	9.409	9.305	9.425
GRID1-AS1	5.672	5.517	5.653	5.653	5.782	6.047
MYO3A	6.536	6.669	6.104	6.671	6.754	6.621
MYO3B	7.419	7.319	7.613	7.215	7.264	6.993

NOP2	10.057	10.101	10.049	10.135	10.005	10.042
DENND4A	8.666	8.629	8.737	8.714	8.525	8.643
DENND4B	9.446	9.392	9.311	9.340	9.369	9.280
CT55	6.905	7.001	6.959	7.251	7.235	7.328
SWAP70	8.412	8.492	8.495	8.006	8.170	8.010
PHLDA1	12.133	12.271	12.332	12.143	12.178	12.168
PHLDA3	8.914	9.163	9.305	9.072	9.032	9.108
PHLDA2	12.032	12.146	12.195	11.780	11.965	11.903
AC245123.1	6.155	6.313	6.270	6.351	6.344	6.234
PAMR1	8.166	8.180	7.902	8.057	8.219	8.311
TIPRL	9.160	9.148	9.313	9.147	9.340	8.952
GHDC	9.773	9.885	10.093	9.745	9.803	9.696
AC027243.1	6.656	6.334	6.113	6.385	6.448	6.228
H19	7.037	7.110	6.829	6.886	7.066	7.028
AL024509.3	4.857	5.059	5.148	4.786	4.861	5.049
AL024509.2	8.501	8.428	8.574	8.497	8.147	8.408
KIRREL3-AS2	4.939	4.890	4.788	5.135	5.248	5.001
KIRREL3-AS3	7.046	7.044	7.040	7.170	7.180	6.920
SSX8P	7.301	7.374	7.153	7.302	7.279	7.432
NOG	5.106	5.306	5.501	5.363	5.289	5.185
SMAD9	7.213	7.082	7.243	7.286	7.374	7.216
TRERF1	6.866	7.150	7.026	7.147	6.948	7.088
LRPAP1	9.770	9.680	9.948	9.677	9.423	9.607
SMAD5	10.114	10.126	10.070	9.913	10.006	10.046
SMAD6	8.724	8.675	8.826	8.474	8.296	8.507
SMAD7	7.569	7.448	7.698	7.534	7.419	7.541
GOLPH3L	10.970	11.112	11.187	11.183	11.123	10.992
SMAD1	9.119	9.061	9.065	8.529	8.865	9.115
SMAD2	10.046	10.189	10.197	10.061	9.960	10.164
SMAD3	10.779	10.848	10.781	10.536	10.434	10.540
MUS81	9.738	9.712	9.921	9.517	9.675	9.680
AMMECR1L	8.926	8.971	9.008	8.695	8.395	8.870
RAD21L1	4.096	4.045	4.034	4.067	4.349	4.252
PLRG1	9.887	9.820	9.858	9.563	9.688	9.756
AC058822.1	5.352	5.252	5.660	5.091	5.229	5.288
ERICH6	6.977	7.134	6.888	7.221	7.113	7.062
ERICH5	7.686	7.771	7.749	7.122	7.423	7.356
ERICH4	6.715	6.534	6.645	6.683	6.553	6.660
ERICH3	4.675	4.664	4.510	4.592	4.752	4.679
ERICH1	9.093	9.041	9.176	8.925	9.082	9.008
AL049872.1	13.049	13.066	13.213	12.923	13.203	12.740
HERC2P2	7.950	7.699	7.727	7.957	7.613	8.029
HERC2P5	6.831	6.888	7.012	6.858	7.207	6.953
HERC2P9	7.950	7.699	7.727	7.957	7.613	8.029
HERC2P8	6.831	6.888	7.012	6.858	7.207	6.953
TCEA2	7.850	7.914	8.133	7.722	7.825	8.038
TCEA3	7.452	7.404	7.490	7.577	7.549	7.597
TCEA1	11.480	11.515	11.666	11.474	11.401	11.357
BFAR	10.725	10.806	10.900	10.480	10.711	10.416
TMLHE-AS1	4.529	4.576	4.480	4.526	4.567	4.684
TDRKH-AS1	8.446	8.566	8.466	8.542	8.804	8.657
ABCD2	4.348	4.240	4.051	4.559	4.421	4.199
PANTR1	7.811	7.965	7.612	7.797	7.945	7.705
LEO1	9.107	9.053	8.991	8.673	8.725	8.797
MGRN1	10.270	10.301	10.397	10.042	10.067	10.195
AC009061.2	7.984	7.973	7.616	7.872	8.023	7.961

DPT	6.913	6.995	7.059	7.097	7.107	7.003
CUEDC1	9.433	9.347	9.654	9.240	9.309	9.290
CUEDC2	9.919	9.880	10.165	9.777	9.698	9.708
CTSLP8	7.216	7.281	7.235	7.567	7.208	7.375
RBMXL1	9.889	10.081	10.060	9.756	9.976	9.746
RBMXL2	6.644	6.256	6.066	6.559	6.626	6.603
RHEBL1	8.053	8.207	8.207	8.310	8.346	8.255
PTPRD-AS1	3.629	3.894	3.797	4.130	3.895	3.767
CNTF	6.224	6.313	6.090	6.408	6.476	6.443
AL031282.2	8.763	8.734	8.898	8.918	8.703	8.710
PNP	10.673	10.627	10.793	10.774	10.612	10.758
AC107081.5	7.754	7.985	7.892	8.033	8.227	8.153
MTND4LP30	14.452	14.479	14.426	14.490	14.644	14.603
SMKR1	5.009	5.017	4.918	4.997	5.122	5.085
MRLN	6.521	6.638	6.297	6.524	6.539	6.742
PNN	9.262	9.187	9.232	8.923	8.905	9.002
AC026469.1	7.787	7.854	7.827	8.068	7.942	7.935
HAMP	6.248	6.418	6.333	6.452	6.294	6.388
ADCY10P1	7.055	7.216	7.289	7.416	7.345	7.498
MR1	8.731	8.681	8.612	8.501	8.577	8.537
CNPY4	8.119	8.385	8.291	8.241	8.203	8.256
CNPY3	8.701	8.715	8.740	8.653	8.750	8.729
CNPY2	11.901	11.943	12.096	11.852	11.862	11.834
CNPY1	4.581	4.689	4.584	4.912	4.681	4.817
ANKRD30BL	5.821	6.035	5.659	6.080	5.739	5.988
RPL7AP28	7.070	6.987	7.140	6.858	6.558	6.981
SHOX2	8.072	8.205	8.208	8.059	8.050	8.249
CHFR	9.007	9.166	9.355	9.021	9.018	9.195
SMDT1	11.540	11.521	11.763	11.506	11.482	11.414
RCOR3	8.457	8.458	8.524	8.086	8.066	8.240
NUDT9P1	5.008	5.059	4.980	5.439	4.963	4.957
SYPL2	8.062	7.977	7.751	8.106	8.101	8.053
AC010342.1	9.706	9.674	9.796	9.356	9.306	9.441
RFX4	5.132	5.035	5.149	5.300	5.305	5.429
RFX5	9.854	9.892	9.798	9.706	9.679	9.680
APP	13.167	13.168	13.249	13.218	13.109	13.151
RFX7	9.271	9.280	9.268	8.979	9.014	9.063
RFX1	8.923	8.815	9.026	8.987	9.186	8.845
RFX2	7.970	8.011	8.069	8.032	8.139	8.073
RFX3	7.265	7.152	7.201	6.995	7.209	7.078
YDJC	9.573	9.536	9.708	9.496	9.427	9.461
IBA57	8.108	7.961	7.933	7.801	7.881	7.903
APC	8.352	8.156	8.125	8.132	8.047	8.274
TMEM179B	10.985	10.923	11.145	11.014	10.861	10.954
PDZK1	7.425	7.530	7.493	6.845	7.025	6.955
ADNP	10.754	10.847	10.872	10.621	10.710	10.674
ZNF57	7.305	7.359	7.527	7.252	7.252	7.272
BAK1	8.563	8.736	8.641	8.635	8.717	8.597
AC011462.1	10.322	10.438	10.621	10.377	10.286	10.318
AC011462.4	6.500	6.557	6.522	6.577	6.651	6.570
TARS1	10.215	10.259	10.331	9.924	9.895	9.866
TARS3	7.224	7.011	7.387	7.378	7.178	7.280
TARS2	9.168	9.262	9.322	9.111	9.129	9.098
AC244669.3	4.232	4.165	4.506	4.480	4.299	4.279
AC244669.2	4.486	4.626	4.587	4.711	4.614	4.633
AC244669.1	7.865	7.879	8.165	7.721	8.167	7.814

OSR1	5.201	5.282	5.433	5.284	5.238	5.307
IGFBP2	6.479	6.609	6.628	6.321	6.672	6.637
IGFBP3	11.771	11.810	12.012	11.723	11.717	11.715
IGFBP1	11.099	11.074	11.271	10.644	10.857	10.868
HBE1	7.090	7.254	7.203	7.126	7.235	7.126
IGFBP7	10.394	10.518	10.690	10.405	10.191	10.366
IGFBP4	9.677	9.730	9.994	9.694	9.720	9.521
IGFBP5	7.681	7.865	7.990	8.134	7.892	7.854
CYP2R1	8.562	8.487	8.542	8.556	8.556	8.633
CKLF	11.156	11.428	11.428	11.266	11.398	11.369
PMS2P9	9.313	9.354	9.149	9.020	9.274	9.164
APLNR	7.232	7.131	7.158	7.319	7.380	7.409
CYP8B1	6.392	6.147	6.410	6.467	6.564	6.433
PMS2P2	8.737	8.598	8.642	8.446	8.495	8.529
PMS2P3	9.354	9.354	9.385	9.020	9.274	9.164
ARL2-SNX15	8.680	8.823	8.965	8.818	8.841	8.780
PMS2P6	9.531	9.523	9.413	9.362	9.364	9.406
PMS2P7	8.737	8.598	8.642	8.446	8.495	8.529
AL031121.2	7.763	7.695	7.648	7.815	7.891	7.932
TXNIP	8.719	8.812	9.096	8.786	9.167	9.081
FAM157A	10.722	10.564	10.478	10.430	10.307	10.610
FAM157B	7.688	7.417	7.266	7.457	7.674	7.621
FAM157C	7.688	7.417	7.266	7.457	7.674	7.621
MECOM	10.573	10.511	10.776	10.893	10.670	10.694
SDR9C7	5.163	5.301	5.350	5.632	5.381	5.265
KIAA1614-AS1	7.372	7.487	7.577	7.379	7.545	7.477
CLIC6	7.584	7.744	7.753	7.537	7.603	7.578
CLIC4	9.483	9.473	9.494	9.260	9.287	9.272
CLIC5	7.596	7.495	7.486	7.468	7.744	7.438
CLIC2	4.786	4.656	4.552	4.756	4.833	4.684
AL691482.4	11.429	11.527	11.722	11.742	11.567	11.676
LMO7DN	5.480	5.383	5.178	5.172	5.326	5.485
LINC01686	6.667	7.043	6.726	6.934	6.837	6.833
LINC01687	4.707	4.461	4.493	4.744	4.549	4.667
HOXD-AS2	7.171	7.343	7.332	7.362	7.555	7.350
LINC01689	4.296	4.137	4.485	4.268	4.366	4.394
RRS1-AS1	4.534	4.443	4.506	4.313	4.625	4.393
SOWAHA	6.188	6.338	6.167	6.294	6.169	5.997
SECISBP2L	9.315	9.371	9.471	9.240	9.421	9.258
SOWAHC	9.902	9.959	9.961	9.705	9.674	9.795
LINC02483	6.390	6.630	6.279	6.675	6.627	6.502
LINC02482	8.122	8.206	8.072	8.191	8.187	8.225
AL513323.1	4.600	4.600	4.706	4.812	4.613	4.904
LINC02487	8.199	8.239	8.139	8.307	8.354	8.375
PKD1P5	7.215	7.288	7.487	7.403	7.143	7.216
PKD1P6	11.008	10.803	10.771	10.815	10.988	10.947
AL031281.2	6.909	7.060	7.184	6.796	6.975	7.155
Z98259.1	7.570	7.532	7.150	7.822	7.805	7.785
ZNF7	7.912	7.881	8.026	7.617	7.687	7.620
ZNF2	7.913	7.962	7.825	7.836	8.053	7.925
ZNF3	8.153	7.937	8.022	8.103	7.858	8.038
CTCFL	5.576	5.537	5.414	5.974	5.614	5.546
ZNF8	7.929	7.912	7.959	7.827	7.755	8.024
RAMACL	10.440	10.548	10.560	10.603	10.486	10.555
ATP5MC2P1	7.449	7.469	7.729	7.532	7.715	7.361
CDC5L	9.708	9.913	10.105	9.918	10.377	10.055

SBDS	9.294	9.369	9.337	9.362	9.125	9.280
AP2A1	9.685	9.656	9.851	10.061	10.083	9.831
AC083904.1	11.625	11.741	11.768	11.634	11.544	11.667
AP2A2	10.517	10.571	10.665	10.357	10.280	10.440
NIM1K	7.083	7.253	7.067	7.446	7.072	7.071
CLC	6.633	6.496	6.279	6.638	6.629	6.573
NSUN3	8.185	8.203	8.253	8.284	8.095	8.185
NSUN2	8.884	8.908	8.967	8.842	8.824	8.857
NSUN5	9.809	9.780	9.862	9.586	9.679	9.665
NSUN4	9.152	9.080	8.982	8.681	8.919	8.987
NSUN7	7.300	7.251	7.374	6.997	7.042	7.113
NSUN6	7.754	7.622	7.769	7.760	7.705	7.778
AL353804.4	6.017	5.927	5.794	5.957	6.098	5.919
PAGR1	9.870	9.936	10.043	9.909	9.829	9.846
RRP7A	9.257	9.285	9.456	9.276	9.473	9.192
CDR2L	9.176	9.119	9.304	9.010	9.203	9.037
AC090983.2	6.315	6.278	6.488	6.432	6.383	6.201
AC083899.1	9.207	9.103	9.217	9.018	9.021	8.942
AC034229.2	6.189	6.164	6.091	6.179	6.291	6.107
AC034229.3	6.189	6.164	6.091	6.179	6.291	6.107
ERLEC1P1	5.989	6.133	5.974	6.265	6.130	6.105
BRDT	4.954	4.760	4.731	5.062	4.827	5.188
IL9RP3	7.336	7.420	7.085	7.221	7.337	7.436
BRD8	9.028	9.098	9.103	8.816	8.899	8.970
BRD9	9.184	9.227	9.325	9.153	9.186	9.188
BRD4	10.291	10.057	10.216	10.205	10.016	10.123
AL357033.1	6.650	6.627	6.726	6.962	6.835	6.795
SPAAR	9.238	9.283	9.122	9.431	9.317	9.271
BRD7	10.625	10.648	10.622	10.604	11.069	10.353
BRD1	8.645	8.708	8.666	8.579	8.421	8.755
BRD2	11.017	10.992	11.285	11.380	11.398	10.976
BRD3	10.416	10.313	10.523	10.189	10.228	10.174
AC079341.1	6.132	6.209	6.136	6.366	6.276	6.384
CH25H	5.858	5.648	5.831	5.888	6.265	6.138
CSF2RB	6.460	6.495	6.420	6.690	6.418	6.470
CSF2RA	8.096	8.242	8.329	8.189	8.212	8.098
DMD	7.181	7.109	7.245	6.974	7.088	7.075
SEH1L	9.578	9.697	9.750	9.236	9.345	9.368
GAB4	8.738	8.862	8.790	8.962	9.106	9.003
AC105020.1	7.369	7.061	7.305	7.545	7.361	7.417
GAB2	8.884	8.927	9.061	8.749	8.785	8.739
GAB3	7.444	7.483	7.532	7.463	7.694	7.589
GAB1	8.386	8.466	8.311	8.347	8.244	8.285
ZSCAN9	7.858	7.774	8.014	7.788	7.669	7.926
ZSCAN4	5.032	5.060	4.995	5.133	5.172	5.149
ZSCAN1	7.105	7.178	7.279	7.165	7.435	7.400
ZSCAN2	8.089	7.965	8.144	7.848	7.888	7.948
GLIS3-AS1	7.185	7.167	7.237	7.159	7.203	7.225
DNAJB13	8.106	7.957	8.100	8.170	8.324	8.316
DNAJB12	9.926	9.939	9.997	9.698	9.641	9.789
DNAJB11	11.618	11.736	11.872	11.770	11.790	11.640
DNAJB14	8.743	8.805	8.923	8.618	8.954	8.660
FAM230C	4.238	4.363	4.213	4.424	4.649	4.524
SUPT20H	8.566	8.719	8.765	8.657	8.630	8.501
VILL	9.778	9.900	9.951	9.598	9.679	9.732
AC026369.8	7.380	7.159	7.174	7.110	7.418	7.413

AC026369.7	6.022	6.132	6.102	6.188	6.175	6.097
AC026369.1	6.022	6.132	6.102	6.188	6.175	6.097
AC090844.2	4.678	4.713	4.825	4.610	4.394	4.730
PSD	6.996	6.907	7.141	7.486	7.320	7.052
ARHGAP5-AS1	8.279	8.218	8.387	8.177	8.152	8.245
AC004803.1	6.569	6.587	6.667	6.502	6.672	6.520
AMOTL2	11.238	11.210	11.303	11.167	11.252	11.085
PRDM16-DT	7.796	8.239	8.098	8.053	8.102	8.124
AC010531.4	7.318	7.476	7.427	7.317	7.540	7.449
ZNF674-AS1	5.241	5.542	5.450	5.465	5.388	5.345
AC010531.1	8.793	8.867	9.029	8.966	9.193	9.177
PPIAL4A	5.156	5.042	5.248	5.210	5.376	5.205
KRTAP19-3	5.758	5.991	5.403	5.821	5.628	5.760
PPIAL4C	5.156	5.042	5.248	5.210	5.376	5.205
KRTAP19-1	5.191	5.248	4.832	5.104	5.044	5.193
PPIAL4E	5.156	5.042	5.248	5.210	5.376	5.205
PPIAL4G	5.156	5.042	5.248	5.210	5.376	5.205
PPIAL4F	5.156	5.042	5.248	5.210	5.376	5.205
PPIAL4H	5.156	5.042	5.248	5.210	5.376	5.205
ATAT1	7.603	7.706	7.519	7.661	7.511	7.454
FAM214A	9.492	9.493	9.480	9.401	9.314	9.512
FAM214B	9.898	9.966	9.878	9.967	10.039	9.870
CFAP36	8.356	8.306	8.446	8.123	8.230	8.337
AL139193.2	7.962	7.841	7.682	8.046	8.160	8.074
RNU4-78P	7.994	8.070	7.883	7.863	8.040	8.133
AKIRIN2	9.935	9.851	9.938	9.828	9.844	9.872
AKIRIN1	9.981	9.997	10.026	10.151	10.340	9.973
EREG	12.236	12.307	12.273	12.382	12.373	12.477
AVPI1	9.851	9.941	9.930	9.940	9.919	9.924
AC004982.1	5.761	5.911	5.943	5.886	5.880	5.879
ASAP1	10.644	10.501	10.511	10.259	10.267	10.466
ASAP2	10.346	10.327	10.280	10.016	10.005	10.244
ASAP3	7.570	7.377	7.343	7.455	7.534	7.551
ELL2P3	6.584	6.488	6.451	6.756	6.626	6.660
ELL2P1	8.752	9.000	8.495	8.969	9.008	9.069
AP004245.1	12.150	12.230	12.258	12.092	12.057	12.085
HILPDA	9.057	9.246	9.171	9.114	9.209	9.153
BANCR	6.597	6.822	6.687	7.046	6.850	6.714
C14orf132	7.140	7.208	7.249	7.223	7.391	7.283
INAVA	11.999	12.069	12.192	11.815	11.689	11.910
CALCRL	8.092	7.988	7.815	7.827	7.864	7.912
TMEM72-AS1	7.996	8.003	8.217	8.352	8.090	8.343
ANKLE1	5.373	5.204	4.822	5.005	5.079	4.920
CSNK1G2	9.472	9.376	9.463	9.296	9.197	9.457
ANKLE2	9.238	9.354	9.359	9.350	9.373	9.373
RHPN1	8.490	8.339	8.465	8.590	8.629	8.566
INPPL1	9.429	9.473	9.601	9.232	9.271	9.372
OLR1	3.892	4.023	4.051	3.826	4.163	4.157
RHPN2	10.629	10.612	10.632	10.265	10.352	10.584
SRRM1	10.691	10.609	10.764	10.534	10.509	10.405
SRRM3	7.567	7.558	7.648	7.719	7.740	7.758
SRRM2	10.838	10.801	10.818	10.864	10.701	10.804
SRRM5	6.404	6.389	6.100	6.389	6.391	6.397
SRRM4	7.626	7.516	7.441	7.673	7.765	7.690
H3C2	6.429	6.572	7.077	6.415	6.731	6.297
H3C3	7.098	7.053	7.521	7.148	7.352	7.067

H3C1	5.583	5.632	5.495	5.960	5.900	5.651
H3C6	7.801	7.994	7.636	7.719	7.871	8.170
H3C7	4.456	4.523	4.849	4.668	4.765	4.602
H3C4	6.517	6.511	6.779	6.863	7.152	7.146
H3C8	8.450	8.621	8.834	8.314	8.339	8.428
TM2D1	10.380	10.391	10.430	10.247	10.378	10.390
TM2D2	11.473	11.579	11.618	11.680	11.610	11.640
TM2D3	10.610	10.679	10.774	10.251	10.395	10.353
AGAP14P	9.081	9.018	9.114	9.210	9.131	9.065
OGFRL1	8.587	8.462	8.370	7.997	8.030	8.274
ADCK1	8.355	8.276	8.373	8.414	8.341	8.264
AL390755.2	5.837	5.692	5.734	5.576	5.746	5.741
RSBN1	9.109	9.059	9.101	8.862	9.084	9.025
ARHGAP23	10.959	11.078	11.134	11.016	10.977	10.878
ARHGAP22	5.683	5.468	5.869	5.536	5.622	5.629
ARHGAP21	8.642	8.710	8.568	8.301	8.199	8.581
ARHGAP20	7.523	7.671	7.301	7.586	7.585	7.552
ARHGAP27	9.346	9.421	9.598	9.535	9.422	9.558
ARHGAP26	9.171	9.291	9.259	9.010	9.248	9.117
ARHGAP25	7.650	7.612	7.706	7.656	7.562	7.741
ARHGAP24	8.159	8.116	8.104	7.906	8.101	8.086
ARHGAP29	11.174	11.277	11.350	11.292	11.053	11.203
ARHGAP28	6.532	6.755	6.476	6.713	6.830	6.797
AC005498.2	5.898	5.984	5.838	5.972	5.934	5.962
AC005498.3	8.048	8.024	7.815	7.897	7.941	7.918
AC009779.4	10.050	10.072	10.332	10.293	10.235	10.110
RBFA	8.754	8.921	9.041	8.734	8.681	8.356
AC244207.3	4.803	4.674	4.358	4.674	4.730	4.714
H4C1	5.583	5.632	5.260	5.567	5.900	5.571
H4C3	10.681	10.507	10.887	10.796	10.876	10.188
H4C2	5.872	6.021	5.805	6.266	6.414	6.005
H4C5	7.444	7.321	7.564	7.749	7.579	7.522
H4C4	5.946	6.313	6.434	6.306	6.600	6.508
H4C7	4.932	5.014	5.107	5.089	5.165	5.059
H4C6	5.087	5.205	5.090	5.344	5.481	5.465
H4C9	7.855	7.905	8.039	8.049	8.097	7.942
H4C8	6.908	7.354	7.179	7.449	7.323	7.673
DBIL5P	6.751	6.819	6.880	6.815	6.742	7.069
RSAD2	10.396	10.580	10.515	10.568	10.779	10.312
RSAD1	9.690	9.858	9.862	9.616	9.615	9.610
OR2H1	7.973	8.007	8.114	7.967	8.083	7.982
AC137932.1	5.624	5.664	5.773	5.735	5.786	5.880
AL133216.4	10.722	10.564	10.478	10.430	10.307	10.610
AC012555.1	6.818	6.760	6.767	6.630	6.461	6.674
GALK1	7.921	8.283	8.312	8.047	8.083	8.020
IL4R	9.447	9.601	9.604	9.541	9.588	9.517
DYNC1LI1	9.159	9.307	9.353	9.081	9.157	9.116
DYNC1LI2	11.513	11.348	11.467	11.376	11.328	11.273
EFHC2	7.112	7.150	7.305	7.121	7.278	6.963
EFHC1	7.792	7.856	7.754	7.669	7.621	7.765
ABCG1	7.885	7.592	7.755	7.886	7.881	7.731
ABCG2	6.814	7.288	6.982	7.159	7.127	7.037
ABCG4	6.886	6.950	6.972	7.039	7.046	7.171
ABCG5	7.009	7.133	7.276	7.086	7.234	6.913
PLSCR1	13.049	13.176	13.217	13.262	13.311	13.196
ABCG8	7.761	7.797	7.821	7.852	7.844	7.766

INSYN2A	3.777	3.983	3.975	3.855	3.963	3.879
INSYN2B	4.832	4.802	4.694	4.818	4.637	4.736
AL356583.1	4.381	4.370	4.321	4.274	4.226	4.410
PHKA1-AS1	5.224	5.172	5.179	5.353	5.233	5.315
GADL1	5.188	5.313	5.192	5.068	5.221	5.385
AKR1B10	11.021	11.080	11.058	10.688	10.573	10.556
SLA2	7.843	7.708	7.639	7.893	8.011	7.922
C4orf36	4.426	4.567	4.671	4.366	4.613	4.492
C4orf33	7.368	7.473	7.552	7.421	7.440	7.406
AC183084.2	10.910	10.920	10.896	10.979	11.199	10.847
NTRK3-AS1	7.427	7.561	7.485	7.687	7.537	7.559
C15orf48	9.319	9.467	9.486	9.545	9.710	9.765
AL050403.1	5.443	5.370	5.310	5.275	5.683	5.148
AL050403.2	6.528	6.688	6.841	6.775	7.228	6.826
C15orf40	8.975	8.997	9.104	8.545	8.618	8.644
C15orf41	8.895	8.877	8.897	8.288	8.643	8.714
AP002001.1	6.022	6.239	6.076	6.154	6.400	6.253
AC010616.1	11.546	11.648	11.839	11.561	11.487	11.680
PKD2	9.291	9.346	9.415	9.195	9.160	9.160
PKD1	8.217	8.227	8.301	8.711	8.454	8.244
AL445305.1	11.514	11.538	11.646	11.521	11.500	11.458
ZNF138	7.080	7.057	7.021	7.168	7.168	7.154
PRKACG	7.025	7.069	7.056	7.379	7.293	7.204
SQOR	11.034	11.152	11.269	11.051	10.943	11.027
PRKACA	10.678	10.585	10.768	10.557	10.566	10.487
PRKACB	11.232	11.281	11.377	11.105	10.954	11.052
ZNF131	9.017	8.753	8.831	8.859	8.826	8.745
ZNF132	5.534	5.458	5.578	5.744	5.742	5.607
ZNF133	7.221	7.203	7.369	7.357	7.089	7.267
ZNF134	8.297	8.227	8.431	8.308	8.301	8.341
ZNF135	4.728	4.888	4.759	4.953	5.050	4.875
ZNF136	7.666	7.829	7.798	7.552	7.724	7.636
C20orf78	7.227	7.298	7.158	7.257	7.183	7.316
SPG7	10.192	10.155	10.208	9.913	9.757	10.065
SPRYD7	6.773	6.756	6.656	6.564	6.629	6.580
AC016405.2	7.149	7.012	6.971	7.170	7.209	7.059
ALMS1-IT1	5.458	5.451	5.209	5.049	4.969	5.056
AL353583.1	5.996	5.930	5.970	6.145	6.036	6.256
KCNK4	6.793	6.946	6.963	7.121	7.244	7.065
KCNK5	9.156	9.248	9.270	9.209	9.069	9.360
KCNK6	7.006	6.811	6.753	6.485	6.804	6.812
KCNK7	7.988	7.979	7.710	7.758	8.010	7.935
KCNK1	10.676	10.790	10.911	10.642	10.740	10.613
KCNK2	5.564	5.654	5.529	5.487	5.627	5.408
KCNK3	8.477	8.515	8.662	8.502	8.549	8.582
MIR4724	7.663	7.346	7.272	6.779	7.024	7.267
MIR4722	10.218	10.222	10.352	10.155	9.930	10.080
MIR4723	9.845	9.822	9.881	9.829	9.964	9.932
PPAN-P2RY11	7.156	7.365	7.517	7.520	7.316	7.344
MIR4721	12.227	12.368	12.507	12.265	12.113	12.173
NUP160	8.157	8.142	8.095	7.561	7.822	7.937
AC093849.1	4.050	3.982	4.234	3.937	3.985	4.193
AC093849.2	6.754	6.685	6.509	6.789	6.873	6.859
PRMT5-AS1	6.077	6.189	6.123	6.088	6.281	6.219
VWDE	5.627	5.571	5.226	5.269	5.078	5.633
GNA14	7.530	7.488	7.292	7.577	7.761	7.578

GNA15	7.360	7.309	7.788	7.453	7.421	7.397
GNA12	10.794	10.879	10.923	10.762	10.785	10.612
GNA13	10.255	10.299	10.318	10.187	10.186	10.232
GNA11	10.178	10.304	10.355	9.817	9.939	9.975
AL450306.2	6.193	5.819	6.006	5.592	5.913	5.957
AL450306.1	6.193	6.072	6.006	5.856	5.913	5.957
AOX3P	5.779	5.992	5.888	5.872	5.880	6.066
MFGE8	8.578	8.745	8.759	8.484	8.592	8.579
SH2B1	8.644	8.738	8.808	8.701	8.472	8.674
FRRS1L	6.668	6.831	6.475	6.831	6.795	6.745
METTL14-DT	5.463	5.385	4.966	5.499	5.638	5.461
OGDH	10.208	10.221	10.346	10.068	10.023	10.121
SEMG1	4.460	4.433	4.311	4.553	4.349	4.499
SEMG2	4.150	3.794	4.126	4.031	4.042	4.250
RPL13AP25	14.322	14.274	14.333	14.309	14.244	14.333
RPL13AP20	14.424	14.395	14.422	14.370	14.350	14.418
DAZL	4.541	4.542	4.619	4.478	4.445	4.780
DAZ3	6.729	6.748	6.698	6.803	6.882	6.810
DAZ2	6.729	6.748	6.698	6.803	6.882	6.810
DAZ1	6.729	6.748	6.698	6.803	6.882	6.810
DAZ4	6.729	6.748	6.698	6.803	6.882	6.810
C1orf131	9.488	9.560	9.647	9.401	9.468	9.516
TTTY1	7.020	6.651	6.774	7.138	6.895	6.895
ZBTB33	10.216	10.272	10.247	10.156	10.031	10.132
GTF2F1	10.101	9.960	10.154	9.528	10.026	9.733
GTF2F2	7.681	7.748	7.812	7.323	7.319	7.353
AC104078.1	4.196	4.430	4.630	4.416	4.394	4.397
TMEM132E	6.373	6.518	6.323	6.810	6.730	6.459
TMEM132D	6.106	6.027	6.037	6.088	6.149	6.015
TMEM132A	8.953	9.050	9.187	8.964	8.902	8.933
KRT17P7	7.637	7.713	7.510	7.717	7.778	7.694
KRT17P4	7.637	7.713	7.510	7.717	7.778	7.694
KRT17P5	7.637	7.713	7.510	7.717	7.778	7.694
LINC01234	4.934	4.740	4.937	5.088	4.778	4.775
NAIF1	7.584	7.403	7.655	7.471	7.575	7.558
CCL28	7.106	7.449	7.475	7.625	7.490	7.400
CCL21	7.292	7.326	7.478	7.581	7.428	7.299
CCL20	8.253	7.761	7.656	9.032	8.889	8.982
CCL23	5.150	5.245	5.371	4.914	5.119	5.225
CCL22	8.199	8.246	7.868	8.332	8.314	8.338
CCL24	6.985	6.880	7.083	6.955	6.928	7.140
CCL27	6.385	6.453	6.717	7.014	7.249	6.838
CCL26	7.241	7.242	7.419	7.573	7.488	7.255
ALPK1	8.286	8.306	8.351	7.833	7.970	8.129
ALPK3	6.928	6.755	6.898	6.964	6.925	6.922
ALPK2	6.569	6.756	6.398	6.858	6.576	6.629
CUX1	9.462	9.508	9.578	9.326	9.199	9.401
CUX2	5.999	6.043	6.084	6.144	6.260	6.297
AC096586.1	5.080	5.032	4.951	5.176	5.280	5.085
SGTA	9.115	9.177	9.252	9.034	9.006	9.088
AC005165.1	6.328	6.500	6.555	6.611	6.804	6.556
LINC02250	3.962	3.697	3.840	3.853	3.891	3.874
CSNK1A1	11.688	11.634	11.844	11.621	11.544	11.686
GLYATL2	5.826	5.775	5.583	5.619	5.719	5.912
GLYATL1	7.422	7.436	7.335	7.604	7.645	7.492
YWHAZP3	11.821	11.873	11.907	11.936	11.811	11.952

TRIM49	5.312	5.102	5.249	5.279	5.179	5.099
RAP1B	11.673	11.706	11.864	11.695	11.674	11.766
RAP1A	9.871	9.968	9.978	9.811	9.828	9.823
FAM230D	5.796	5.943	5.381	5.595	5.788	5.869
FAM230E	5.796	5.943	5.381	5.595	5.788	5.869
FAM230B	5.796	5.943	5.381	5.595	5.788	5.869
BX927132.1	8.529	8.524	8.065	8.442	8.567	8.533
FAM230A	5.796	5.943	5.381	5.595	5.788	5.869
SNORA64	14.545	14.566	14.561	14.591	14.532	14.585
SNORA65	12.766	12.886	12.934	12.834	12.722	12.796
SNORA66	13.001	13.062	13.190	13.066	13.122	13.074
SNORA67	13.137	13.230	13.247	13.064	13.071	13.089
FAM230J	5.796	5.943	5.381	5.595	5.788	5.869
SNORA61	8.077	7.423	7.713	7.735	7.554	7.718
FAM230H	5.796	5.943	5.381	5.595	5.788	5.869
SNORA63	8.631	8.462	8.315	8.343	7.903	8.597
AL450405.1	12.688	12.701	12.918	12.751	12.591	12.750
ADCY6-DT	6.005	6.041	6.073	5.819	6.322	6.136
TRIM41	8.534	8.587	8.578	8.472	8.302	8.485
CTF1	8.159	8.217	8.220	8.538	8.325	8.180
AL158163.1	4.275	4.400	4.267	4.419	4.386	4.226
AL158163.2	5.520	5.617	5.541	5.345	5.621	5.551
AL022313.4	6.928	6.961	6.974	6.952	7.013	7.131
LDLR	12.009	11.963	12.038	11.926	11.659	11.893
AC007601.1	6.014	5.973	5.948	6.338	6.257	6.134
UBFD1	10.504	10.495	10.640	10.337	10.384	10.408
ACRV1	7.373	7.398	7.269	7.407	7.602	7.581
AF254983.1	4.289	4.521	4.101	4.378	4.376	4.379
MYF6	6.504	6.326	6.210	6.314	6.488	6.197
PGLYRP4	8.084	7.915	7.811	8.020	8.166	8.209
ZNF205	7.714	7.695	8.033	8.065	7.908	7.824
ELOA3B	5.658	5.566	5.464	5.809	5.745	5.944
ELOA3C	5.658	5.566	5.464	5.809	5.745	5.944
ELOA3D	5.658	5.566	5.464	5.809	5.745	5.944
MZF1-AS1	5.894	5.866	6.079	5.879	5.976	5.982
GFI1B	7.668	7.432	7.468	7.564	7.567	7.550
MYOD1	7.375	7.272	7.234	7.732	7.541	7.386
TOB2	10.104	10.068	10.308	9.735	10.008	9.685
SKP1P1	12.764	12.952	13.000	12.813	12.829	12.952
AP001605.1	5.145	5.195	5.189	5.073	5.178	5.279
SULF2	9.976	9.969	10.055	9.879	9.870	9.943
SULF1	6.688	6.894	6.631	6.687	6.938	6.654
IL6-AS1	7.117	7.274	7.264	7.483	7.356	7.421
AASDH	8.081	7.914	8.093	8.161	8.202	7.858
THSD4-AS1	6.787	6.768	6.634	6.882	7.056	6.891
LINC02078	5.362	5.407	5.306	5.202	5.544	5.374
LINC02076	6.011	5.996	5.905	6.070	6.005	6.013
LINC02070	4.016	4.144	4.191	4.378	4.392	4.294
LINC02071	6.467	6.338	6.369	6.509	6.605	6.520
TFG	12.002	11.955	12.089	12.017	11.981	11.921
HVCN1	9.229	9.404	9.367	9.359	9.336	9.401
RND1	8.380	8.297	8.461	8.700	8.630	8.528
RND2	6.592	6.593	6.275	6.533	6.620	6.455
RND3	11.779	11.746	11.836	11.705	11.654	11.627
CTNNBIP1	8.874	9.060	9.039	9.005	8.787	8.850
AL135905.1	5.987	6.069	6.345	5.810	5.880	5.764

AL135905.2	10.567	10.759	10.690	10.802	10.876	10.728
KIAA1549	6.542	6.517	6.551	6.649	6.399	6.571
LRRC41	9.537	9.714	9.716	9.660	9.641	9.692
LRRC40	8.739	8.970	8.981	8.613	8.768	8.605
LRRC43	10.190	10.415	10.316	10.099	10.279	10.395
LRRC42	8.890	8.932	9.245	8.945	8.904	8.901
LRRC45	8.206	8.057	8.207	7.910	7.748	7.808
LRRC47	10.718	10.810	10.915	10.471	10.468	10.485
LRRC46	8.020	7.941	8.202	7.824	7.952	8.072
LRRC49	6.954	7.044	7.123	6.950	7.067	7.256
FNDC8	6.930	6.689	6.900	7.011	7.021	6.808
FNDC9	6.155	6.203	6.151	6.230	6.450	6.359
FNDC4	9.024	9.114	9.157	9.235	9.157	9.247
FNDC5	7.972	8.203	7.980	7.823	8.143	8.091
FNDC7	6.452	6.696	6.601	6.897	6.929	6.778
AC124045.1	6.441	6.359	6.207	6.307	6.072	6.301
PWWP2B	8.632	8.752	8.756	8.572	8.695	8.742
PWWP2A	8.481	8.432	8.471	8.296	8.355	8.304
AC018716.1	7.026	7.004	6.993	7.218	7.279	7.205
AC092046.1	6.441	6.359	6.207	6.307	6.072	6.301
AC080112.2	10.854	11.220	11.393	10.839	10.999	10.636
LINC02693	7.797	7.547	7.669	8.019	7.647	7.717
CCDC152	9.479	9.485	9.437	9.267	9.386	9.321
CCDC153	6.009	6.140	6.339	6.052	5.972	6.205
CCDC150	6.533	6.694	6.475	6.733	6.651	6.656
CCDC151	7.945	7.928	7.846	8.344	8.068	8.092
CCDC157	8.857	8.875	8.834	8.755	8.948	9.001
CCDC154	7.870	7.703	7.866	7.755	7.709	7.678
CCDC155	6.307	6.187	6.470	6.630	6.362	6.551
CCDC158	6.004	6.178	6.117	5.981	6.059	6.164
CCDC159	7.785	7.785	7.914	7.988	7.828	7.988
LINC01521	6.748	6.795	6.780	6.609	6.545	6.557
LINC01520	7.003	7.200	6.766	7.257	7.094	7.160
LINC01523	6.747	6.910	6.945	7.084	7.073	7.100
LRRC4B	5.342	5.331	5.382	5.690	5.419	5.486
LINC01525	7.513	7.613	7.502	7.495	7.552	7.633
LINC01527	3.717	3.719	3.761	3.662	3.852	3.780
LINC01526	5.890	6.153	5.860	6.050	5.978	6.046
TASP1	7.876	7.707	7.731	7.588	7.559	7.594
PCED1A	8.571	8.494	8.593	8.368	8.450	8.492
PCED1B	7.714	7.583	7.623	7.843	7.650	7.712
AL354674.1	8.480	8.556	8.803	8.970	8.816	8.848
RN7SKP176	4.659	4.612	4.465	4.757	4.578	4.702
AL031681.3	6.596	6.558	6.499	6.493	6.321	6.488
SPZ1	4.423	4.246	4.396	4.550	4.504	4.634
AC139677.3	7.451	7.498	7.457	7.197	7.176	7.373
AC139677.2	7.316	7.496	7.592	7.563	7.519	7.583
AC006213.2	7.990	7.916	7.970	7.928	7.774	8.116
NES	7.537	7.578	7.686	7.506	7.761	7.502
CCZ1B	11.738	11.824	11.875	11.801	11.716	11.768
PALMD	7.898	7.881	8.315	7.528	7.878	7.769
PALM3	8.357	8.273	8.380	7.787	7.798	8.059
MANSC1	11.569	11.629	11.761	11.454	11.628	11.624
MAGEA2B	4.642	4.389	4.736	4.435	4.816	4.636
ICMT	10.721	10.758	10.812	10.676	10.565	10.604
FDCSP	12.213	12.365	12.315	12.427	12.480	12.411

AL451123.1	4.337	4.394	4.183	4.403	4.235	4.569
FCF1	9.842	10.017	10.035	9.520	9.552	9.620
ATP6V0A4	6.621	6.822	6.791	6.687	6.812	6.819
LINC00691	4.036	4.222	4.001	4.348	4.198	4.325
LINC00692	4.180	4.256	4.485	4.657	4.571	4.437
ATP6V0A1	10.963	11.128	11.155	10.926	10.829	10.939
ATP6V0A2	8.857	8.801	8.833	8.568	8.575	8.785
AC092666.1	7.066	7.185	7.400	7.255	7.358	7.430
MIR612	7.470	7.762	7.649	7.752	7.843	7.849
ACOXL	6.985	6.960	7.122	7.022	7.031	6.989
RPL36A-HNRNPH	13.408	13.485	13.653	13.701	13.471	13.603
ITGBL1	6.459	6.479	6.237	6.472	6.438	6.497
LINC00347	4.419	4.375	4.495	4.527	4.458	4.535
LINC00342	7.457	7.393	7.354	7.151	7.137	7.249
LINC00343	4.313	4.270	4.528	4.171	4.344	4.427
LINC00348	4.400	4.298	4.580	4.319	4.360	4.439
PODXL2	8.457	8.323	8.731	8.106	8.160	8.270
NPAT	8.657	8.465	8.497	8.147	8.398	8.162
USP30-AS1	6.948	6.873	6.917	7.118	7.040	7.050
LLGL2	8.873	8.912	9.008	8.768	8.929	8.750
LLGL1	10.093	10.178	9.765	10.141	10.152	10.228
ACOX2	5.997	6.272	6.243	6.153	6.048	6.246
ACOX3	8.247	8.169	8.160	8.024	8.107	8.056
ACOX1	9.318	9.291	9.308	8.998	9.154	9.157
AC140125.2	7.941	8.046	8.013	7.997	8.065	8.231
RABGAP1	9.884	9.806	9.903	9.667	9.815	9.832
RCSD1	6.942	6.980	6.674	7.201	6.768	6.989
GAL3ST1	7.622	7.704	8.061	7.780	7.847	7.883
GAL3ST3	7.592	7.617	7.427	7.674	7.574	7.681
GAL3ST2	7.030	7.196	7.299	7.261	7.188	7.255
GAL3ST4	7.875	7.927	7.823	7.968	7.909	7.959
AL356273.6	5.788	5.674	5.614	5.496	5.734	5.643
C3orf33	5.946	5.867	5.775	4.962	5.269	5.509
AC087343.1	12.617	12.677	12.802	12.784	12.769	12.841
NANP	7.586	7.602	7.600	7.704	7.649	7.601
NANS	10.156	10.268	10.345	9.886	9.838	10.020
C3orf38	8.135	8.200	8.240	8.330	8.290	8.416
MATN1	7.309	7.255	7.366	7.392	7.356	7.380
MATN3	4.674	4.496	4.614	4.269	4.490	4.386
MATN2	6.065	6.115	5.988	6.063	6.014	6.073
MATN4	7.823	7.769	7.914	7.953	7.812	7.943
PCGEM1	4.140	4.221	4.337	4.195	4.342	4.429
RPL12P4	13.555	13.621	13.669	13.572	13.470	13.564
ENDOG	8.569	8.542	8.861	8.398	8.326	8.540
EBF4	7.320	7.310	7.424	7.732	7.521	7.612
EBF3	5.669	5.760	5.654	5.735	5.741	5.862
EBF2	5.815	5.661	5.923	5.981	5.874	6.043
EBF1	6.890	6.670	6.383	6.948	6.702	6.679
SNAPC5P1	5.730	5.758	5.920	5.810	5.837	5.928
LMNA	11.679	11.838	12.043	11.749	11.719	11.563
BDH2	8.897	8.909	8.869	8.622	8.716	8.623
ENDOV	8.547	8.754	8.883	8.698	8.706	8.713
ENDOU	5.274	5.304	5.429	5.319	5.587	5.557
BDH1	9.392	9.564	9.656	9.508	9.529	9.423
SPG11	9.311	9.233	9.324	9.127	9.099	9.126
AC046134.2	8.195	8.348	8.298	8.298	8.483	8.231

GULP1	11.959	12.057	11.978	12.176	11.980	12.072
IGDCC4	5.843	5.829	5.754	5.911	5.628	5.491
FLVCR2	8.155	8.105	7.989	8.304	8.282	8.215
FLVCR1	9.533	9.677	9.605	8.962	9.225	9.428
TASOR2	9.227	9.205	9.071	8.775	8.893	8.932
AC104574.1	6.675	6.881	6.730	6.635	6.936	6.728
GGPS1	9.626	9.621	9.645	9.524	9.546	9.607
AC093297.2	7.000	6.877	6.817	6.476	6.592	6.699
NACA2	4.808	4.864	4.953	4.883	4.983	4.857
C9orf163	8.152	8.083	8.071	8.104	8.297	8.239
MARCKS	11.385	11.439	11.454	11.392	11.491	11.555
AC256309.1	4.824	4.788	4.781	4.794	4.875	4.728
CGRRF1	8.383	8.211	8.522	8.579	8.576	8.470
TMEM109	10.184	10.178	10.188	10.173	10.081	9.963
TMEM108	6.769	6.746	6.822	6.915	6.763	6.913
NACAD	6.819	6.967	6.863	6.985	6.793	7.060
TMEM102	6.834	7.035	7.337	7.076	6.924	7.084
TMEM101	8.718	8.837	8.965	8.598	8.716	8.398
FOXH1	7.647	7.457	7.748	7.793	7.669	7.732
TMEM107	9.171	9.037	9.260	8.717	8.863	8.622
TMEM104	8.651	8.582	8.892	8.911	8.677	8.544
AC036222.3	9.616	9.703	9.637	9.570	9.653	9.874
AC131009.4	8.053	7.959	7.722	7.719	7.737	7.935
IGHV7-81	5.323	5.273	5.103	5.100	5.386	5.278
COIL	8.415	8.296	8.398	8.369	8.704	8.126
RTL10	8.798	8.924	8.876	8.680	8.791	8.879
AC009163.3	7.790	7.766	7.754	7.990	7.730	7.666
FRZB	5.959	5.790	5.832	6.080	5.842	5.994
DGLUCY	9.488	9.460	9.573	9.567	9.710	9.623
PDCL2	3.686	3.787	3.869	3.884	3.960	3.941
AC006328.2	6.582	6.672	6.703	6.718	6.700	6.647
EIPR1	9.310	9.472	9.540	9.104	9.273	9.137
LRRC37BP1	8.061	8.058	8.021	7.915	7.840	7.926
CCM2	9.602	9.675	9.789	9.548	9.471	9.551
AL161457.2	7.042	7.041	7.069	6.914	6.923	6.807
AL662857.1	6.517	6.543	6.395	6.499	6.649	6.716
AL662857.3	7.145	7.246	7.000	7.072	7.096	7.194
Z84485.1	5.846	5.814	5.875	5.972	5.932	5.854
AF111169.1	6.335	6.287	6.320	5.878	6.328	6.287
HLTF	10.867	10.919	10.965	10.619	10.582	10.706
VOPP1	11.584	11.753	11.958	11.648	11.489	11.621
KRT3	6.496	6.728	6.841	6.704	6.649	6.823
KRT2	7.126	7.148	7.073	7.138	7.130	7.127
KRT1	7.233	7.279	7.026	7.286	7.403	7.452
KRT7	13.090	13.253	13.305	13.053	12.872	13.036
UBE2E2-AS1	5.640	5.797	5.995	6.171	5.990	6.067
KRT5	7.461	7.557	7.549	7.724	7.905	7.899
KRT4	8.268	8.374	8.348	8.007	8.135	8.211
KRT9	8.092	8.137	8.085	8.321	8.152	8.228
KRT8	13.948	14.005	14.050	14.066	14.014	14.045
DACT1	4.924	5.225	5.323	5.359	5.433	5.288
DACT3	7.588	7.836	7.416	7.526	7.563	7.628
DACT2	7.116	7.254	7.017	7.164	7.286	7.221
TMCC1	9.470	9.501	9.592	9.463	9.533	9.609
TMCC2	7.913	7.996	7.864	8.023	7.908	8.149
TMCC3	6.080	6.165	5.971	6.203	6.334	6.278

MACROH2A1	12.379	12.365	12.541	12.318	12.197	12.366
MACROH2A2	9.975	9.905	10.085	9.901	9.924	9.918
NDUFV2P1	11.142	11.224	11.372	11.121	10.999	11.129
AC016737.1	4.729	4.580	4.537	4.766	4.519	4.571
CDHR2	8.411	8.521	8.610	8.532	8.430	8.428
RIPK4	10.047	10.201	10.282	10.109	9.919	9.999
AC026954.6	9.973	10.101	10.384	10.139	10.405	9.924
INSC	5.314	5.106	5.133	5.742	5.408	5.467
AC233282.1	6.855	6.640	6.656	6.808	6.641	6.810
INSR	8.396	8.236	8.501	7.839	8.180	8.226
RUNX1T1	7.856	7.894	7.823	7.613	7.852	7.777
SNORD116-21	6.639	6.757	6.724	6.961	6.931	6.798
JHY	7.065	7.335	7.136	6.983	7.264	7.333
AC026954.2	9.973	10.101	10.384	10.139	10.405	9.924
SPTBN5	7.561	7.683	7.478	7.602	7.642	7.561
SPTBN2	8.011	8.010	8.211	8.195	8.268	8.142
SPTBN1	13.585	13.626	13.539	13.507	13.273	13.552
MRPL33	11.047	10.947	11.072	11.124	11.116	11.196
ARPC4-TTLL3	9.630	9.670	9.849	9.652	9.724	9.669
TFAP2A	10.110	10.108	10.264	9.900	9.834	9.937
TFAP2C	8.567	8.494	8.650	8.268	8.362	8.500
TFAP2B	4.996	4.949	5.058	5.139	5.203	5.206
TFAP2E	8.041	8.173	7.808	7.894	7.936	8.028
TFAP2D	6.590	6.692	6.575	6.638	6.608	6.589
AC002064.2	6.162	6.032	5.844	6.185	6.027	6.435
AC002064.4	4.498	4.438	4.578	4.701	4.700	4.718
CYYR1	6.763	6.698	6.147	6.789	6.684	6.617
AC245060.7	8.268	8.068	8.195	8.569	8.602	8.402
SAMHD1	10.309	10.130	10.121	9.973	10.491	9.867
CRABP2	9.272	9.324	9.604	9.665	9.755	9.624
RPA4	4.893	5.160	5.212	5.157	5.201	5.369
RPA1	10.145	10.088	10.209	9.820	9.594	9.827
RPA3	11.202	11.340	11.555	11.285	11.207	11.218
RPA2	10.002	10.060	10.210	9.837	9.989	9.847
REC8	9.081	8.964	9.029	9.085	8.935	9.014
AC025183.1	4.717	4.710	4.532	4.754	4.762	4.749
PCDHGB3	11.092	11.102	11.188	11.070	11.043	11.048
PCDHGB2	11.092	11.102	11.188	11.070	11.043	11.048
PCDHGB1	11.092	11.102	11.188	11.070	11.043	11.048
CHMP2A	10.809	10.819	11.061	10.798	10.631	10.735
AL138688.1	4.968	4.946	4.979	4.801	4.855	4.843
PCDHGB6	11.092	11.102	11.188	11.070	11.043	11.048
PCDHGB5	11.092	11.102	11.188	11.070	11.043	11.048
PCDHGB4	11.092	11.102	11.188	11.070	11.043	11.048
RREB1	9.843	9.696	9.871	9.509	9.448	9.583
TP53AIP1	6.980	7.108	7.224	7.327	7.191	7.261
PHC1	8.829	8.816	8.734	8.559	8.611	8.730
PHC3	10.274	10.229	10.240	10.004	9.985	10.122
PHC2	10.590	10.677	10.828	10.572	10.688	10.598
PIANP	6.871	6.939	7.047	7.203	7.038	7.282
AL132780.2	7.623	7.643	7.641	7.592	7.618	7.616
AL132780.3	9.686	9.780	9.996	9.792	9.808	9.696
STK4-AS1	7.999	7.936	7.813	7.741	7.968	7.714
ZNF362	10.324	10.402	10.548	10.293	10.242	10.189
ZNF365	6.584	6.840	6.625	6.833	6.917	6.835
AL132780.4	7.725	7.778	7.678	7.781	8.004	7.860

AL132780.5	4.412	4.316	4.333	4.439	4.382	4.333
PRF1	7.814	8.065	7.770	7.935	7.990	7.938
AL031673.1	6.995	6.648	6.797	6.962	6.974	6.723
TMEM189-UBE2V	12.225	12.293	12.363	12.065	12.065	12.079
NCAPG2	9.031	8.959	8.986	8.354	8.418	8.412
DPEP1	7.865	8.220	7.975	8.343	8.283	8.174
DPEP2	6.035	6.085	6.208	5.958	6.107	6.003
DPEP3	6.865	6.903	6.886	7.328	7.255	7.134
NMD3	11.078	11.136	11.176	11.109	11.033	11.084
GLIPR1	9.143	9.117	9.128	9.108	9.332	8.956
TEX43	5.005	5.088	4.817	4.998	5.098	4.847
TEX41	6.934	6.824	6.789	6.962	7.059	7.044
LSAMP-AS1	7.093	7.164	7.189	6.964	7.253	7.196
TEX47	6.239	6.359	6.111	6.103	6.384	6.225
TEX44	7.501	7.539	7.709	7.480	7.635	7.545
TEX45	8.129	8.263	8.271	8.149	8.176	8.169
TEX48	8.695	8.404	7.662	8.969	8.860	8.512
TEX49	5.389	5.210	5.274	5.403	5.241	5.357
LINC01541	4.077	3.784	3.745	3.955	3.975	4.050
BAIAP3	8.138	8.293	8.278	8.357	8.323	8.175
BAIAP2	8.570	8.714	8.755	8.613	8.630	8.703
NUP88	9.284	9.356	9.537	9.179	9.176	9.209
VAX2	6.629	6.933	6.843	7.318	6.912	7.008
AC018529.3	7.849	7.895	7.691	7.631	7.815	7.872
SNORC	9.260	9.258	9.264	9.245	9.097	9.160
AC068790.8	5.135	5.175	5.060	5.038	5.260	5.319
AC068790.9	9.578	9.647	9.245	9.393	9.684	9.638
AC068790.5	7.366	7.382	7.090	7.381	7.284	7.391
AC068790.2	3.952	3.890	3.802	4.096	3.821	3.801
MSL1	12.738	12.712	12.883	12.689	12.489	12.698
FOXP4	8.485	8.515	8.531	8.719	8.799	8.520
POLDIP3	10.862	10.818	11.000	10.823	10.816	10.760
POLDIP2	10.633	10.637	10.694	10.566	10.578	10.567
GP1BA	8.159	7.878	7.914	7.727	7.935	7.930
GP1BB	6.629	6.776	6.702	6.785	6.939	6.795
MIR3682	5.181	5.282	4.934	5.027	4.990	5.330
MIR3685	7.613	7.563	7.396	7.583	7.338	7.678
ALDH18A1	9.992	10.083	10.102	9.777	9.842	9.915
NUP85	9.716	9.668	9.912	9.457	9.386	9.436
C1orf74	7.855	7.729	7.934	7.925	8.074	7.916
YTHDF3	10.038	10.198	10.234	9.936	9.932	10.001
YTHDF2	11.486	11.478	11.615	11.351	11.379	11.337
LINC00102	3.746	3.746	3.588	3.697	3.696	3.696
AL358473.1	7.834	8.072	8.195	7.717	8.128	7.956
LINC00106	8.219	8.289	7.998	8.193	8.409	8.178
NPIPB13	11.306	11.174	11.094	11.070	11.186	11.250
AC019171.1	4.365	4.471	4.397	4.595	4.382	4.495
CFAP99	6.530	6.825	6.529	6.952	6.775	6.875
HS3ST3A1	4.204	4.260	4.255	4.519	4.217	4.571
RAB4B-EGLN2	9.863	9.905	10.153	9.962	9.978	9.954
PYCARD	9.060	9.064	9.322	8.796	8.987	8.980
MIR302D	6.683	6.626	6.864	7.044	7.444	6.745
PARD6G-AS1	7.756	7.737	7.777	7.871	7.824	7.968
CSNK1A1P1	5.356	5.175	5.241	5.254	5.213	5.070
AL133467.6	5.897	6.011	5.568	5.812	6.060	5.801
AL133467.3	7.671	7.817	7.671	7.423	7.636	7.802

AL049629.2	12.839	12.963	13.024	12.872	12.810	12.813
DUSP28	9.040	9.039	9.144	8.818	8.840	8.811
AC117382.2	6.779	6.654	6.510	6.647	6.870	6.764
DUSP21	6.037	6.010	5.944	6.154	6.480	6.083
DUSP22	8.613	8.705	8.663	8.512	8.551	8.724
DUSP23	8.862	8.833	9.161	8.854	8.896	8.857
DUSP26	6.469	6.656	6.659	6.654	6.724	6.669
DUSP27	5.237	4.935	5.035	4.962	5.214	5.208
TBC1D22A-AS1	6.144	6.168	6.174	6.110	6.074	6.146
FENDRR	7.503	7.533	7.852	7.814	7.851	7.613
YAF2	7.147	6.958	7.009	7.290	7.194	7.266
HSD17B7P2	10.477	10.548	10.659	10.659	10.664	10.429
USP46-AS1	5.231	5.180	5.100	5.338	5.257	5.187
ODAM	11.167	11.133	11.424	11.208	11.110	11.226
EDIL3	5.789	5.741	5.781	5.935	5.882	5.583
ZGPAT	8.435	8.378	8.528	8.112	8.208	8.295
EIF5A2	8.141	8.439	8.251	8.143	8.050	7.996
H2AC5P	6.891	7.136	7.222	7.180	6.977	7.116
UBE2D4	10.750	10.808	10.809	10.689	10.817	10.757
UBE2D2	11.100	11.215	11.357	11.121	11.004	11.103
UBE2D3	12.321	12.437	12.444	12.457	12.397	12.456
UBE2D1	9.107	9.062	9.215	8.935	8.951	9.147
SNORD65	11.440	11.575	11.802	11.951	12.180	11.982
SNORD60	8.531	8.481	8.588	8.491	8.308	8.552
EDNRA	7.257	7.323	7.022	7.151	7.409	7.290
EDNRB	5.953	6.051	6.266	5.971	6.148	6.088
SNORD68	13.941	13.952	14.003	13.975	13.935	13.896
SAP130	9.805	9.806	9.847	9.585	9.535	9.620
APOF	4.936	4.745	4.736	4.766	4.797	4.998
APOD	7.646	7.870	7.594	7.975	8.017	8.024
AC010501.2	6.925	6.838	6.889	6.833	6.991	6.809
APOB	6.140	6.143	6.137	6.189	6.087	5.898
AC109454.4	3.841	3.825	3.795	4.073	3.826	4.042
APOO	9.564	9.689	9.792	9.711	9.679	9.556
APOM	7.969	8.101	8.030	7.929	7.799	8.048
APOH	7.420	7.253	7.204	7.305	7.459	7.376
FAM104A	9.342	9.466	9.450	9.254	9.188	9.252
FAM104B	7.412	7.488	7.642	7.416	7.590	7.386
CCDC50	10.796	10.748	10.842	10.797	10.724	10.809
CCDC51	9.332	9.466	9.556	9.352	9.174	9.138
AC136618.1	7.256	7.004	7.129	7.152	6.993	7.140
CCDC54	5.747	6.050	5.872	5.776	5.999	5.838
CCDC57	8.060	8.079	8.248	8.161	8.244	8.098
CCDC58	8.500	8.545	8.740	8.603	8.435	8.560
CCDC59	10.311	10.227	10.380	10.348	10.594	10.144
AL390239.1	6.092	5.964	5.950	5.939	5.977	5.876
AC138028.6	7.084	7.105	7.248	7.355	7.369	7.401
NUFIP2	10.344	10.292	10.411	10.310	10.316	10.036
ZMIZ1	8.895	8.891	8.810	8.782	8.830	8.902
NUFIP1	8.079	8.069	8.209	8.190	8.453	7.961
PREPL	10.534	10.612	10.682	10.678	10.620	10.688
CABP7	6.328	6.394	6.327	6.201	6.355	6.522
CABP5	6.680	6.663	6.491	6.907	6.843	6.832
CABP4	9.983	9.929	10.021	9.985	10.069	9.990
CABP2	6.906	6.889	6.837	6.942	7.038	6.842
CABP1	7.185	7.016	7.009	7.407	7.078	7.143

FBXO34	9.884	9.870	10.001	9.775	9.693	9.770
FBXO36	6.752	6.660	6.713	6.599	6.571	6.973
FBXO30	8.064	8.181	8.143	7.966	8.093	8.080
FBXO31	8.595	8.558	8.591	8.387	8.484	8.477
FBXO32	8.638	8.640	8.630	8.552	8.552	8.570
FBXO33	8.816	9.150	9.131	8.548	9.036	8.426
FBXO38	9.510	9.480	9.484	9.525	9.486	9.525
FBXO39	6.057	5.930	5.730	5.863	6.050	6.048
AC096887.2	7.731	7.747	7.852	7.413	7.687	7.522
MBOAT2	9.392	9.280	9.376	9.172	9.298	9.234
MBOAT1	8.574	8.748	8.779	8.572	8.705	8.593
MBOAT7	12.523	12.504	12.511	12.478	12.382	12.550
SCOC-AS1	5.070	5.368	5.598	5.729	5.461	5.159
SLC25A53	5.125	5.148	5.090	5.060	4.848	5.059
SLC25A52	7.246	7.409	7.518	7.686	7.515	7.591
SLC25A51	7.246	7.409	7.518	7.686	7.515	7.591
UNC79	7.258	7.223	7.043	7.364	7.504	7.342
AL136169.1	4.844	4.802	4.899	4.537	4.533	4.775
ALAS1	10.415	10.570	10.651	10.312	10.186	10.349
ALAS2	7.501	7.359	7.509	7.630	7.597	7.726
TRAV20	6.789	6.932	6.780	6.843	6.928	7.082
FICD	9.002	8.974	9.106	8.785	8.944	8.673
KIAA0513	8.120	7.976	8.122	7.886	7.904	8.039
AC073321.1	7.071	7.173	7.381	7.001	7.240	7.169
CDCA7L	10.571	10.731	10.733	10.024	10.095	10.172
APBA1	8.098	8.106	8.034	7.727	8.064	8.007
APBA3	7.977	7.920	8.125	7.580	7.688	7.617
APBA2	8.218	8.366	8.214	8.313	8.406	8.280
AC015961.1	4.109	4.188	4.339	4.069	4.089	4.371
LINC00266-1	4.095	4.251	4.353	3.909	4.106	4.174
MORC2	8.991	8.840	8.798	8.817	8.758	8.877
MORC3	7.698	7.851	7.601	7.500	7.330	7.642
AC072039.2	3.978	4.004	3.974	4.028	3.995	4.065
MORC4	9.223	9.008	9.274	8.893	8.913	8.946
PTPRZ1	4.375	4.221	4.194	4.329	4.355	4.346
GPATCH2L	7.772	7.908	7.730	7.749	7.813	7.752
SSUH2	7.267	7.226	7.104	7.293	7.131	7.282
LINC01770	7.515	7.761	7.538	7.740	7.661	7.742
RAI1	10.595	10.704	10.811	10.461	10.654	10.403
RAI2	5.537	5.469	5.342	5.365	5.297	5.280
COL28A1	5.315	5.398	5.408	5.559	5.429	5.652
LINC01777	6.043	5.784	6.128	5.960	6.242	5.963
C1orf112	7.738	7.867	7.984	7.511	7.642	7.549
CBWD5	11.277	11.323	11.404	11.049	11.073	11.051
AC105233.4	6.451	6.418	6.257	6.244	6.283	6.307
C1orf116	10.601	10.601	10.767	10.586	10.718	10.530
C1orf115	8.884	8.995	9.159	8.708	8.702	8.659
AL645933.3	7.718	7.863	7.785	7.728	7.866	7.900
LGALS3BP	12.655	12.671	12.747	12.531	12.547	12.478
TTBK2	6.428	6.351	6.312	6.220	6.267	6.421
TTBK1	8.193	8.213	8.168	8.274	8.490	8.518
ZNFX1	10.966	11.011	10.965	11.031	10.974	11.030
AL359924.1	6.196	6.243	6.055	6.086	6.331	6.259
MAGEF1	10.247	10.317	10.420	10.018	10.030	10.168
ACBD3	9.948	9.857	10.005	9.698	10.058	9.710
ACBD6	9.012	9.187	9.330	8.756	8.901	8.751

ACBD5	9.492	9.575	9.646	9.427	9.384	9.446
ACBD4	8.879	8.777	8.897	8.889	8.829	8.895
HYLS1	7.450	7.378	7.498	7.531	7.620	7.389
TIGIT	5.662	5.903	5.763	5.792	5.891	5.825
AGAP7P	9.081	9.018	9.114	9.210	9.131	9.065
AC008115.4	7.804	7.600	7.691	7.646	7.694	7.768
AC092747.4	7.386	7.365	7.417	7.696	7.648	7.491
LINC00632	4.402	4.305	4.454	4.728	4.454	4.614
DFFA	9.762	9.810	9.874	9.843	10.031	9.752
DFFB	6.278	6.106	6.311	5.808	6.134	6.144
REV3L	8.422	8.438	8.319	8.049	8.163	8.286
RNF19B	8.930	8.973	9.013	9.042	9.285	9.018
DNAJC15	10.637	10.738	10.946	10.692	10.951	10.595
RNF19A	9.543	9.523	9.583	9.429	9.510	9.493
PLA2G4E-AS1	4.293	4.265	4.378	4.191	4.378	4.162
MIR429	8.288	8.312	8.319	8.400	8.250	8.307
MRPL43	9.676	9.816	9.974	9.770	9.690	9.718
MRPL42	10.490	10.524	10.679	10.615	10.541	10.541
AC092552.1	6.569	6.877	6.805	6.950	6.848	7.143
MRPL40	10.291	10.393	10.595	10.278	10.284	10.212
MRPL47	11.785	11.891	12.000	11.755	11.688	11.762
MRPL46	10.134	10.089	10.234	9.940	9.887	9.951
MRPL45	11.779	11.821	12.013	11.776	11.753	11.730
MRPL44	9.430	9.411	9.562	9.249	9.292	9.389
TRIP6	10.281	10.341	10.507	10.314	10.128	10.103
MRPL49	11.488	11.535	11.728	11.552	11.570	11.476
MRPL48	9.588	9.622	9.769	9.610	9.583	9.651
NGF	6.447	6.427	6.640	6.964	6.721	6.586
POM121B	10.031	10.002	10.057	9.989	10.211	9.782
NGB	8.077	8.328	8.202	7.798	8.220	8.210
NAV2-IT1	6.292	6.752	6.572	6.583	6.667	6.747
FHAD1	9.225	9.162	9.116	9.157	9.202	9.252
UNC93B8	9.103	9.078	9.349	9.121	9.095	8.982
AL450468.2	8.026	8.160	8.244	8.089	8.046	8.207
GOLGA2P3Y	6.210	6.139	6.179	6.290	6.315	6.341
AC012560.2	6.702	6.677	6.599	6.962	6.764	6.780
UNC93B1	10.288	10.450	10.501	10.242	10.390	10.156
UNC93B3	9.103	9.078	9.349	9.121	9.095	8.982
UNC93B2	9.103	9.078	9.349	9.121	9.095	8.982
SPOUT1	8.249	8.325	8.400	8.266	8.256	8.222
NEK6	9.703	9.845	9.888	9.751	9.761	9.807
NEK7	10.525	10.583	10.530	10.461	10.492	10.615
NEK4	8.300	8.439	8.514	8.071	8.134	8.170
NEK5	5.269	5.427	5.433	5.507	5.371	5.428
NEK2	9.731	9.705	9.946	9.224	9.535	9.289
NEK3	6.619	6.698	6.559	6.422	6.232	6.315
CCSAP	7.793	7.902	7.724	7.347	7.403	7.377
AL033529.1	8.474	8.701	8.797	8.424	8.606	8.556
AL807761.2	10.132	10.169	10.215	10.237	10.132	10.185
NEK8	7.865	7.798	7.811	7.459	7.723	7.667
NEK9	9.850	9.792	9.834	9.717	9.739	9.610
BMX	6.604	6.762	6.775	7.013	6.960	7.002
AC010201.2	5.746	5.621	5.345	5.660	5.388	5.701
G0S2	8.401	8.284	8.451	8.370	8.282	8.517
AC010201.1	5.157	5.106	5.075	5.389	4.956	5.030
CCDC3	8.769	8.897	8.822	9.026	9.067	8.922

CCDC6	11.582	11.664	11.675	11.498	11.341	11.485
CCDC7	7.823	7.810	7.613	8.305	7.922	7.887
CCDC8	7.804	7.495	7.741	7.666	7.770	7.699
CCDC9	8.775	8.773	8.820	8.730	8.672	8.777
PCDHB18P	8.067	7.879	7.992	8.354	8.130	8.180
AL163051.1	5.363	5.366	5.385	5.473	5.465	5.390
NSD1	8.017	8.109	7.985	8.238	8.209	8.108
NSD3	11.351	11.356	11.494	11.481	11.457	10.989
NSD2	9.427	9.546	9.586	9.360	9.437	9.298
AL356776.2	7.202	7.538	7.281	7.055	7.232	7.413
CENPVL1	5.912	6.029	6.150	5.979	5.780	5.978
STBD1	7.942	8.123	8.364	8.165	8.011	8.113
EID2B	6.359	6.400	6.543	5.807	5.881	5.852
STAM-AS1	4.272	4.052	4.218	4.426	4.362	4.218
WDHD1	7.439	7.230	7.520	7.235	7.030	7.083
AS3MT	5.589	5.504	5.610	5.521	5.693	5.756
CFAP161	4.817	4.852	4.931	5.376	5.086	5.012
CIB3	6.615	6.476	6.368	6.523	6.623	6.614
AL133464.1	7.294	7.264	7.203	7.315	7.324	7.271
TSPOAP1-AS1	7.311	7.361	7.292	7.765	7.529	7.430
EPYC	4.121	4.167	4.266	4.333	4.218	4.252
AC009309.1	6.501	6.695	6.324	6.690	6.523	6.557
AL139384.1	6.739	6.744	6.636	7.125	6.941	6.859
AC009309.2	5.837	5.905	6.123	6.269	6.031	5.988
KEL	8.550	8.420	8.657	8.667	8.653	8.562
LAGE3	9.772	9.799	10.101	9.760	9.890	9.831
STAB1	8.181	8.265	8.360	8.209	8.454	8.368
STAB2	7.831	7.508	7.474	7.644	7.718	7.590
PYHIN1	7.141	7.009	7.202	7.182	7.106	7.116
PCP2	6.885	6.934	7.151	7.000	7.025	6.870
MTMR14	9.276	9.398	9.482	9.126	9.140	9.162
TRMT2A	8.642	8.763	8.931	8.604	8.778	8.651
ECE2	7.777	7.742	7.712	8.026	7.901	7.868
ECE1	9.293	9.344	9.524	9.483	9.212	9.318
TRMT2B	8.083	7.924	7.630	7.648	7.920	7.853
AC025181.2	7.825	7.880	8.103	7.723	7.738	7.653
IMPACT	8.093	8.334	8.307	7.885	8.131	7.972
EVC2	6.311	6.591	6.442	6.442	6.318	6.593
AURKA	9.703	9.650	9.904	9.324	9.507	9.453
AURKB	9.005	9.230	9.176	8.709	8.748	8.752
AURKC	7.246	7.069	6.944	7.280	7.305	7.161
MAP3K2-DT	5.694	5.669	5.464	5.734	5.888	5.395
WFIKKN2	7.075	7.105	7.157	7.108	7.173	7.189
WFIKKN1	9.000	9.043	9.152	8.831	8.783	8.926
TCAF2C	7.650	7.730	7.688	7.716	7.904	7.828
ACADVL	10.722	10.753	10.837	10.505	10.375	10.552
NCR3LG1	8.124	8.161	8.237	7.990	8.004	7.964
PPIAP11	14.423	14.465	14.415	14.484	14.467	14.475
AL358216.1	7.574	7.524	7.364	7.837	7.710	7.503
CHN2	8.252	8.297	8.476	8.390	8.260	8.317
CHN1	8.168	8.311	8.083	8.222	8.022	8.406
MAGOH2P	7.703	7.732	7.828	7.407	8.002	7.915
CAV2	11.049	11.159	11.152	10.872	10.917	11.116
CAV3	8.278	8.349	8.131	8.405	8.407	8.366
AXL	11.104	11.095	11.310	11.010	10.961	10.940
CAV1	10.278	10.346	10.429	10.055	10.279	9.864

GAS1RR	6.452	6.629	6.703	6.447	6.792	6.739
AC055717.1	7.906	7.908	7.896	7.687	7.952	7.984
AL356966.1	6.728	7.027	6.868	6.716	6.872	6.766
AP003486.1	7.955	7.932	7.794	7.979	7.790	8.008
AL157838.1	5.713	5.756	5.954	5.826	5.791	5.713
AC004009.3	4.946	4.967	5.117	5.020	5.062	5.026
AC004009.2	5.718	5.763	5.496	5.755	5.683	5.902
SARNP	9.594	9.536	9.553	9.254	9.088	9.455
WASF4P	8.640	8.849	8.952	8.827	9.170	8.494
SOX2-OT	3.738	3.784	3.837	3.815	3.879	3.730
ZACN	7.127	7.270	7.118	7.352	7.364	7.648
SKAP2	11.117	11.035	10.997	11.146	10.836	11.156
SKAP1	9.347	9.394	9.596	9.435	9.325	9.400
LRRC36	7.695	7.580	7.690	7.765	7.778	7.850
PITPNA	9.648	9.576	9.748	9.706	9.533	9.554
PITPNB	11.666	11.651	11.761	11.645	11.504	11.517
DCAF12L2	5.881	5.805	5.783	5.685	5.925	5.924
DCAF12L1	5.790	5.684	5.828	5.947	5.881	5.787
CLEC7A	7.756	7.726	7.462	7.582	7.654	7.770
AC090627.1	5.990	6.069	6.018	5.897	5.940	5.969
AC009121.3	6.161	6.365	6.511	6.231	6.463	6.404
AC009121.2	6.749	6.741	6.555	6.873	6.974	6.759
OFD1P6Y	5.534	5.482	5.365	5.619	5.244	5.157
NEBL-AS1	5.573	5.865	5.760	5.620	5.464	5.771
AMN1	7.895	7.811	7.805	7.630	7.837	7.805
KIR3DL2	7.337	7.221	7.177	7.296	7.440	7.287
KIR3DL3	7.540	7.832	7.397	7.753	7.783	7.762
RPARP-AS1	7.387	7.320	7.455	7.681	7.633	7.622
LMNB2	9.826	9.829	9.970	9.557	9.370	9.578
IL13RA2	5.116	5.276	4.893	5.265	5.039	5.151
IL13RA1	10.484	10.560	10.646	10.338	10.345	10.353
LMNB1	9.976	10.003	10.082	9.845	9.891	9.630
MRPS28	8.486	8.496	8.795	8.429	8.256	8.420
MRPS26	8.886	9.053	9.185	8.979	8.862	8.890
CD274	8.324	8.332	8.253	8.404	8.372	8.326
MRPS24	12.186	12.208	12.390	12.175	12.176	12.134
CD276	10.616	10.719	10.817	10.574	10.552	10.532
MRPS22	10.154	10.336	10.453	10.424	10.341	10.284
MRPS23	8.781	8.998	9.207	9.036	9.070	9.028
MRPS21	10.231	10.233	10.392	10.272	9.810	10.108
CCDC88B	8.816	8.882	8.987	8.925	8.872	8.858
RXFP2	5.184	5.081	4.819	5.151	5.062	4.996
AL360270.2	4.319	4.347	4.324	4.319	4.441	4.443
AC239804.1	7.195	6.888	6.932	7.044	7.164	7.014
AC091132.1	7.661	7.826	7.758	7.458	7.267	7.420
AC091132.3	4.803	4.674	4.358	4.674	4.730	4.714
AC091132.4	6.669	6.541	6.555	6.856	6.841	6.802
JMJD6	9.200	9.143	9.368	9.176	9.126	9.223
JMJD7	8.026	8.076	8.316	7.955	7.839	8.004
JMJD4	9.073	9.217	9.510	8.862	8.946	8.998
DNAJC9-AS1	8.591	8.515	8.441	8.527	8.585	8.531
ZHX2	9.793	9.847	9.841	9.647	9.620	9.557
ZHX3	7.994	7.982	8.006	7.794	7.909	7.816
JMJD8	9.522	9.593	9.764	9.581	9.523	9.538
ZHX1	9.165	9.296	9.432	9.187	9.335	9.111
RPL41P5	14.596	14.604	14.644	14.671	14.651	14.656

AC008393.3	4.153	4.296	4.264	3.931	4.149	3.906
RPL41P1	14.596	14.604	14.644	14.671	14.651	14.656
RPL41P2	14.596	14.604	14.644	14.671	14.651	14.656
AC244489.3	11.306	11.174	11.094	11.070	11.186	11.250
AC244489.1	11.306	11.174	11.094	11.070	11.186	11.250
APPL1	9.035	8.975	8.939	8.731	9.145	8.620
APPL2	9.304	9.482	9.403	9.357	9.317	9.352
PINK1-AS	11.452	11.447	11.550	11.192	11.321	11.238
MEMO1	10.241	10.250	10.344	10.169	10.153	10.173
LINC02402	6.100	5.973	5.820	6.113	6.003	6.117
LINC02401	7.127	7.205	6.935	7.142	7.120	7.239
LINC02400	6.994	7.054	6.994	7.253	7.230	7.192
AP000462.2	6.858	7.000	6.876	7.000	6.961	6.940
LINC02405	5.550	5.849	5.690	5.516	5.533	5.736
HBBP1	6.388	6.699	6.393	6.490	6.454	6.513
SIGMAR1	11.254	11.321	11.410	11.237	11.438	11.085
HS3ST6	5.865	5.626	6.029	5.970	6.111	5.848
HS3ST5	7.028	7.306	7.469	7.286	7.296	7.164
HS3ST4	7.732	7.472	7.519	7.829	7.788	7.832
HS3ST2	7.134	7.123	7.017	6.977	7.070	6.958
HS3ST1	10.872	11.043	11.098	10.945	11.077	10.729
SVIP	9.746	9.775	9.905	9.570	9.902	9.644
NOCT	7.974	7.767	7.651	7.843	7.925	7.906
AC145343.1	7.906	7.561	7.982	7.689	7.765	7.717
PHOSPHO2	8.144	8.146	8.130	7.848	7.930	7.872
PHOSPHO1	6.313	6.572	6.358	6.471	6.539	6.367
ZNHIT3	9.682	9.712	9.904	9.601	9.518	9.561
ZNHIT2	8.098	8.192	8.497	8.104	8.198	7.975
ZNHIT1	9.134	9.252	9.461	9.174	9.082	9.372
ZNHIT6	10.216	10.249	10.243	10.105	9.977	9.922
AC091978.1	6.121	6.108	6.209	6.082	6.370	6.288
PLEKHM1P1	8.639	8.656	8.719	8.735	8.695	8.544
NFATC2IP	9.799	9.777	9.628	9.378	9.464	9.390
AC132008.2	6.231	5.833	6.061	5.942	5.799	6.065
OTX2-AS1	4.601	4.601	4.588	4.934	4.771	4.794
BLID	6.100	6.335	5.790	6.285	6.074	5.886
FAAH2	8.468	8.399	8.354	8.333	8.359	8.427
ZP1	5.846	6.439	6.297	6.427	6.342	6.442
ZP3	8.569	8.674	8.851	8.413	8.585	8.498
ZP2	6.462	6.688	6.363	6.486	6.313	6.385
ZP4	6.679	7.030	6.673	6.591	6.645	6.729
PMEL	6.821	6.930	6.870	6.853	7.044	6.908
TTYH2	6.966	6.920	6.829	7.014	6.948	6.945
TTYH3	11.735	11.745	11.880	11.717	11.737	11.663
STK11IP	8.681	8.568	8.712	8.301	8.425	8.347
SPAG11B	7.015	7.000	7.105	7.081	7.216	7.303
SPAG11A	7.015	7.000	7.105	7.081	7.216	7.303
MIR7106	9.220	9.299	9.495	9.240	9.193	9.152
PMF1-BGLAP	7.433	7.630	7.597	7.392	7.658	7.514
C6orf62	11.135	11.161	11.301	11.128	11.234	11.022
NFS1	9.570	9.508	9.635	9.348	9.481	9.446
LIPE-AS1	8.001	7.944	7.907	8.121	8.214	8.124
FBXL3	8.814	8.727	8.493	8.816	8.445	8.670
KIF5C-AS1	5.185	5.147	4.874	5.288	5.645	5.393
FBXL2	7.426	7.334	7.297	7.611	7.509	7.590
FBXL5	9.488	9.549	9.655	9.315	9.401	9.527

DES	7.965	7.879	8.096	8.332	8.215	8.029
ROBO4	7.242	7.333	7.380	7.625	7.544	7.486
DEK	11.641	11.640	11.704	11.408	11.517	11.493
SLC25A5-AS1	7.867	8.183	7.948	8.082	8.374	8.370
MANEA	7.904	8.112	8.163	7.763	7.736	7.855
LRP5L	6.274	6.436	6.214	6.287	6.343	6.293
MIR223	6.963	7.042	6.757	7.390	7.136	6.999
MIR221	6.003	5.820	5.745	5.855	5.663	6.085
FAM241B	8.678	8.380	8.705	8.645	8.582	8.579
FAM241A	8.224	8.302	8.188	7.878	8.132	8.009
HTR2A	5.570	5.540	5.468	5.383	5.529	5.496
HTR2C	4.460	4.507	4.382	4.645	4.689	4.601
HTR2B	4.992	5.034	4.920	5.057	5.109	5.045
GBA2	9.240	9.257	9.326	9.084	8.979	9.064
GBA3	7.002	6.997	6.949	7.055	6.952	6.954
EPHA1-AS1	6.953	6.708	7.323	7.583	7.057	7.124
KIAA0895	7.258	7.502	7.441	7.108	7.322	7.430
AC106712.2	4.745	4.637	4.630	5.006	4.751	4.882
TBPL1	9.592	9.644	9.715	9.650	9.749	9.703
AC073439.1	8.554	8.553	8.616	8.516	8.636	8.710
AC096576.6	5.791	5.566	5.445	5.831	5.866	5.797
ESAM	7.312	7.236	7.264	7.156	7.335	7.211
AC011228.1	6.101	6.095	6.026	6.233	6.018	6.087
ERMN	5.238	5.379	5.281	5.385	5.542	5.486
ACE	6.888	6.868	7.060	7.021	7.090	7.162
ACD	9.939	10.038	10.161	9.705	9.620	9.657
FAM222A-AS1	5.384	5.357	5.443	5.843	5.547	5.458
ACR	6.489	6.368	6.478	6.771	6.803	6.615
ME1	10.139	10.222	10.200	10.257	10.138	10.039
ME3	8.513	8.485	8.715	8.512	8.336	8.545
ME2	9.932	9.998	9.907	9.914	9.771	9.924
MPZL2	8.935	9.048	9.031	8.845	8.872	9.006
MPZL3	7.383	7.613	7.634	7.393	7.429	7.330
AC064799.1	13.304	13.343	13.490	13.360	13.298	13.417
MPZL1	10.794	10.906	10.931	10.826	10.865	10.837
UBDP1	3.785	3.884	3.928	3.845	3.770	3.782
ST6GALNAC6	8.316	8.125	8.364	8.417	8.464	8.306
CYP7B1	5.161	5.279	4.987	5.215	5.353	5.138
AL451069.3	8.786	9.099	8.818	8.810	8.913	8.977
AL451069.1	5.819	5.916	6.023	5.971	6.189	5.953
MET	11.634	11.701	11.688	11.578	11.488	11.593
CLPX	8.645	8.654	8.724	8.602	8.712	8.702
AC126118.1	6.731	6.440	6.325	6.588	6.623	6.598
LALBA	6.797	6.938	7.000	6.592	6.807	6.870
CLPP	9.665	9.688	9.872	9.635	9.610	9.559
CLPS	6.973	6.976	7.063	7.115	7.224	7.053
FOXD2-AS1	6.399	6.376	6.656	6.802	6.482	6.528
CLPB	7.576	7.723	7.774	7.636	7.677	7.583
AP000892.4	8.456	8.361	8.450	8.499	8.629	8.569
FEZF2	5.279	5.433	5.224	5.456	5.308	5.260
SLC9A3R2	5.915	5.725	5.949	6.066	6.311	5.574
SLC9A3R1	12.135	12.130	12.277	12.038	11.987	12.041
AP003115.1	4.149	4.352	4.270	4.563	4.245	4.396
AC254788.1	7.736	7.709	7.860	8.013	7.863	7.976
RBPMS-AS1	8.498	8.495	8.608	8.544	8.540	8.519
AC092279.1	6.890	6.813	6.658	6.960	6.969	6.797

AC092279.2	6.309	6.340	6.263	6.241	6.150	6.203
SLC40A1	11.381	11.474	11.601	11.471	11.565	11.495
CLP1	9.509	9.570	9.637	9.563	9.561	9.490
LILRP2	5.465	5.240	5.208	5.596	5.463	5.357
TRBV24-1	6.362	6.368	6.397	6.671	6.528	6.497
DTWD2	8.622	8.765	8.667	8.540	8.623	8.740
DTWD1	8.640	8.502	8.382	8.570	8.547	8.618
CPM	10.266	10.215	10.186	9.978	10.178	9.826
CPO	6.378	6.033	6.030	6.494	6.284	6.369
CPD	10.607	10.688	10.787	10.352	10.264	10.442
CPE	7.283	7.408	7.510	7.298	7.297	7.147
CPZ	7.935	8.175	8.140	8.413	7.842	8.229
CPQ	7.223	7.127	7.264	7.449	7.378	7.463
CASS4	5.015	5.089	4.934	5.309	5.367	5.298
CYP46A1	6.734	6.802	6.416	6.529	6.696	6.600
LINC02363	7.285	7.373	7.327	7.420	7.427	7.634
BANK1	8.219	8.233	8.208	8.106	8.557	8.006
LINC02361	7.543	7.707	7.634	7.343	7.545	7.395
LINC02366	5.838	6.086	5.912	5.854	5.917	6.021
LINC02367	4.744	4.859	4.504	4.755	4.863	4.806
DOC2A	8.086	7.946	8.082	8.098	8.092	8.068
AC107398.4	5.559	5.570	5.508	5.789	5.745	5.455
DOC2B	8.164	8.156	8.287	8.165	8.319	8.132
PWRN1	6.315	6.278	6.488	6.432	6.383	6.201
PWRN2	7.093	6.766	6.608	7.097	7.064	7.293
UQCRQ	12.600	12.638	12.725	12.690	12.594	12.729
LMTK3	6.102	5.897	5.871	5.935	6.092	5.852
LMTK2	9.409	9.391	9.570	9.048	9.151	9.103
HHIP	5.471	5.521	5.503	5.577	6.372	6.032
AC112229.4	7.471	7.500	7.686	7.180	7.427	7.531
AL139275.1	5.635	5.602	5.807	5.683	5.747	5.796
AC112229.1	7.564	7.798	7.581	7.620	7.841	7.801
AC112229.3	7.564	7.798	7.581	7.620	7.841	7.801
IGLV7-43	6.307	6.174	6.231	6.569	6.428	6.509
PJVK	6.195	6.095	6.143	6.010	6.276	6.229
PSAP	12.618	12.651	12.770	12.392	12.088	12.449
GABRP	13.673	13.703	13.682	13.642	13.536	13.605
KRT17	10.165	10.153	10.404	10.185	10.086	10.156
KRT16	6.680	6.645	6.842	6.913	6.816	6.787
KRT15	8.360	8.418	8.535	8.239	8.236	8.212
KRT14	5.630	5.722	5.438	5.759	5.576	5.772
KRT13	5.482	5.608	5.954	5.552	5.726	5.641
KRT12	8.351	8.334	8.378	8.178	8.089	8.211
KRT10	12.809	12.785	12.882	12.867	12.948	12.934
GABRE	9.370	9.159	9.055	9.289	8.922	9.525
GABRD	7.844	8.020	8.009	8.051	8.006	7.949
KRT19	14.098	14.139	14.162	14.007	13.875	14.013
KRT18	14.436	14.402	14.453	14.358	14.332	14.348
ABHD17A	10.190	10.287	10.526	10.113	10.130	10.051
ABHD17B	9.120	9.086	9.086	8.595	8.813	8.894
ABHD17C	11.499	11.630	11.689	11.554	11.583	11.465
SLC26A6	7.586	7.494	7.604	7.385	7.663	7.510
NBPF9	11.550	11.483	11.377	11.343	11.231	11.508
NBPF8	11.550	11.483	11.377	11.343	11.231	11.508
PAG1	6.427	6.653	6.882	6.725	6.761	6.273
NBPF4	6.013	6.253	5.960	6.011	6.055	6.109

NBPF3	11.550	11.483	11.377	11.343	11.185	11.508
NBPF1	11.009	11.042	10.885	10.932	10.739	11.001
WFDC21P	7.397	7.460	7.511	7.556	7.652	7.657
IQCH-AS1	8.820	8.816	8.763	8.781	8.930	8.624
NPIPB14P	7.559	7.552	7.405	7.429	7.405	7.683
ZFP69B	5.064	5.033	4.918	4.937	5.096	5.236
AC022306.2	5.644	5.564	5.568	5.516	5.972	5.839
PKLR	7.730	7.822	7.740	7.816	7.897	7.846
AC005829.1	6.624	6.679	7.071	6.635	6.748	6.671
AC005829.2	9.778	9.851	9.856	9.219	9.772	9.656
AL160314.2	6.315	6.325	6.178	6.027	6.218	6.181
TM9SF4	10.456	10.501	10.652	10.387	10.356	10.386
TM9SF1	10.845	10.861	11.040	10.826	10.739	10.727
TM9SF3	12.342	12.482	12.453	12.395	12.277	12.381
TM9SF2	11.552	11.620	11.547	11.471	11.314	11.558
ADD3-AS1	6.087	5.915	6.048	6.117	6.115	6.180
RPL29P11	13.159	13.195	13.371	13.208	13.111	13.211
SPATS2L	13.017	13.145	13.161	13.042	12.982	13.023
AC097104.1	6.893	6.749	6.852	7.061	6.945	7.041
MEPCE	10.018	10.061	10.209	9.902	9.990	9.988
KCNC4	7.791	7.832	7.740	7.917	7.861	7.760
KCNC1	7.822	7.906	7.735	8.037	7.761	7.888
KCNC2	5.597	5.672	5.762	5.892	5.773	5.737
KCNC3	8.071	8.037	8.259	8.412	8.253	8.205
AF127577.6	7.580	7.710	7.518	7.711	7.767	7.733
AC021713.1	5.318	5.618	5.474	5.318	5.294	5.388
MFSD6L	7.156	7.298	7.280	6.956	7.164	7.095
NGFR	6.961	7.027	6.773	6.839	6.921	6.827
G2E3-AS1	6.351	6.382	6.409	6.417	6.308	6.425
ACAA2	10.837	10.833	11.041	10.739	10.687	10.776
ACAA1	9.278	9.381	9.559	9.340	9.269	9.253
AC068860.1	5.736	6.018	5.755	5.776	5.755	5.610
OR3A2	6.192	6.224	6.239	6.224	6.527	5.976
OR3A3	7.343	7.540	7.589	7.626	7.786	7.505
ZFP91-CNTF	9.122	9.151	9.458	9.554	9.616	8.669
AC012254.2	8.840	8.972	8.960	8.579	8.528	8.710
AC068580.4	8.648	8.766	8.802	8.613	8.596	8.698
ARIH2	8.687	8.608	8.700	8.476	8.333	8.480
AC022336.2	5.781	5.712	5.713	5.839	5.971	5.797
SUFU	8.495	8.529	8.675	8.496	8.380	8.555
PLA2G4C	6.333	6.250	6.367	6.622	6.388	6.510
PLA2G4B	9.660	9.771	9.686	9.544	9.800	9.749
PLA2G4A	8.379	8.471	8.553	8.218	8.454	8.290
TLK2P1	8.869	8.897	9.012	8.849	8.889	8.833
PLA2G4F	7.871	7.881	8.116	8.191	7.976	7.886
CMTM8	9.614	9.802	9.786	9.711	9.695	9.554
CMTM2	6.713	6.754	6.645	6.550	6.631	6.822
CMTM3	9.562	9.669	9.639	9.341	9.393	9.441
CMTM1	7.392	7.252	7.580	7.374	7.370	7.346
CMTM6	12.447	12.436	12.639	12.575	12.429	12.568
CMTM7	11.567	11.600	11.813	11.481	11.340	11.475
CMTM4	11.797	12.049	12.118	11.995	12.112	11.602
CMTM5	7.549	7.667	7.538	7.723	7.627	7.568
COL11A1	5.353	5.379	4.885	5.440	5.467	5.304
COL11A2	8.157	8.346	8.396	8.335	8.284	8.532
AC104389.1	8.275	8.513	8.286	8.334	8.389	8.578

NCBP2AS2	9.116	9.115	9.333	9.064	9.083	8.941
AC104389.5	7.191	7.300	7.218	7.328	7.503	7.422
AC104389.7	8.275	8.513	8.286	8.334	8.389	8.578
AC104389.6	7.191	7.300	7.218	7.328	7.503	7.422
LAD1	12.078	12.173	12.262	12.177	12.189	12.088
NDUFA11	10.984	11.008	11.353	11.144	11.081	11.075
NDUFA12	12.252	12.328	12.447	12.339	12.260	12.278
NDUFA13	11.039	11.066	11.299	11.049	10.948	11.107
AC008083.2	6.628	6.779	6.746	6.439	6.694	6.727
AC007773.1	6.652	6.797	6.881	6.742	6.807	6.853
ALDH1L2	6.730	6.855	6.751	6.902	6.720	6.851
ALDH1L1	9.305	9.191	9.411	8.926	8.988	8.905
EFL1	9.364	9.458	9.469	9.305	9.193	9.262
GNG7	7.842	8.158	8.055	8.115	7.996	8.039
GNG4	8.288	7.845	8.103	7.796	7.916	7.868
GNG5	12.530	12.641	12.776	12.587	12.551	12.483
GNG2	7.158	7.386	7.304	7.132	7.262	7.180
GNG3	7.661	7.780	7.678	7.738	7.639	7.746
ENTPD1-AS1	7.905	7.731	7.980	8.014	7.786	7.763
GNG8	6.205	6.218	6.178	6.209	6.229	6.336
MAFG-DT	8.376	8.529	8.520	8.214	8.433	8.312
FUS	10.343	10.176	10.345	9.752	9.678	9.786
UGT3A1	6.186	6.435	6.286	6.209	6.346	6.430
FUZ	8.056	7.984	8.207	8.046	8.113	8.021
AC010197.2	4.100	4.126	4.340	4.286	4.275	4.443
TTLL6	7.620	7.864	7.734	7.691	7.756	7.593
SMARCE1	12.485	12.494	12.596	12.412	12.412	12.601
AL353748.3	5.097	5.450	5.400	5.219	5.355	5.246
RASGEF1C	6.655	6.740	6.500	6.510	6.605	6.502
RASGEF1B	5.977	6.251	5.926	6.023	6.016	6.146
RASGEF1A	6.320	6.336	6.417	6.063	5.846	6.226
TTLL9	7.956	8.034	7.719	7.930	8.080	7.928
AC237191.1	4.136	4.329	4.011	4.241	4.217	4.217
PLEKHS1	6.217	6.303	6.532	6.076	6.210	6.198
SMYD4	8.429	8.288	8.642	8.326	8.627	8.332
SMYD5	8.797	8.756	8.765	8.862	8.823	8.727
TRDMT1	6.617	6.711	6.423	7.065	6.660	6.634
SMYD1	7.499	7.561	7.583	7.625	7.583	7.685
SMYD2	10.177	10.322	10.430	10.048	10.204	10.126
SMYD3	9.744	9.716	9.859	9.550	9.540	9.589
AP000769.3	9.898	9.354	9.697	8.855	9.269	9.399
AP000769.5	13.287	12.735	12.628	12.932	13.268	12.707
FAM78A	7.057	6.961	6.833	7.355	7.071	7.105
FAM78B	7.834	7.632	7.549	7.563	7.766	7.820
LY75	10.795	10.820	10.817	10.543	10.552	10.680
CLK1	7.699	7.668	7.779	7.751	7.378	7.962
CLK2	9.559	9.464	9.612	9.468	9.439	9.517
CLK3	9.478	9.500	9.673	9.449	9.518	9.412
CLK4	7.623	7.532	7.676	7.897	7.824	7.754
ONECUT3	7.170	7.136	7.382	7.207	7.394	7.410
ONECUT2	11.833	11.901	11.993	11.894	11.644	11.632
ONECUT1	6.843	6.875	6.735	6.641	6.776	7.035
AC027237.5	7.305	7.368	7.174	7.216	7.239	7.296
GTF2IP20	9.349	9.203	9.195	9.170	9.223	9.503
AC116345.4	5.113	5.131	5.314	5.497	5.097	5.375
TMEM150A	7.712	7.786	7.980	7.937	8.066	7.672

TMEM150C	6.771	6.887	6.845	6.760	6.866	6.691
TMEM150B	7.931	8.051	7.996	7.865	7.914	8.167
MTO1	8.717	8.880	8.829	8.636	8.616	8.769
LYSMD3	9.136	9.220	9.241	9.043	9.140	9.233
ACVR2A	9.560	9.714	9.554	9.642	9.605	9.759
ACVR2B	7.786	7.775	7.690	7.483	7.440	7.484
RRAS2	10.080	10.209	10.274	10.116	10.214	10.037
LYSMD4	7.658	7.615	7.716	7.572	7.723	7.687
SPATA33	8.143	8.290	8.353	8.196	8.152	8.116
SPATA32	10.515	10.511	10.477	10.785	10.737	10.712
MTOR	7.328	7.448	7.670	7.303	7.208	7.344
MUC5AC	10.777	10.698	10.491	10.443	10.487	10.502
TMEM225B	6.145	6.372	6.280	6.224	6.309	6.533
OMG	7.712	7.653	7.616	7.710	7.889	7.760
OMD	5.316	5.501	5.411	5.891	5.908	5.736
AC002094.1	9.334	9.495	9.540	9.250	9.186	9.228
AC002094.3	7.741	7.949	7.748	7.850	7.979	7.814
OMP	6.380	6.249	6.354	6.400	6.354	6.783
CNBD2	8.101	7.932	7.743	8.052	8.019	8.198
CNBD1	5.538	4.897	5.272	5.770	5.611	5.678
C2orf69	9.176	9.198	9.037	8.911	8.952	9.010
C2orf68	8.583	8.591	8.695	8.018	8.448	8.323
AC012181.2	7.271	7.201	7.412	7.238	7.255	7.317
AC012181.1	7.271	7.201	7.412	7.238	7.255	7.317
AC104958.1	4.924	4.913	4.910	5.118	4.847	4.973
OR52D1	7.458	7.428	7.243	7.388	7.408	7.628
THRB-IT1	5.855	5.994	6.044	6.141	6.230	5.967
HIP1	8.047	8.105	8.058	8.187	8.129	8.004
SNHG15	9.609	9.652	9.768	9.718	9.632	9.723
SNHG14	6.694	6.757	6.724	6.961	6.931	6.798
SNHG17	9.596	9.562	9.719	9.469	9.487	9.569
SNHG16	11.941	11.926	12.101	11.800	11.724	11.844
SNHG11	9.380	9.392	9.455	9.006	9.186	9.212
SNHG10	7.342	7.631	7.261	7.496	7.552	7.684
SNHG12	8.556	8.432	8.314	8.383	8.220	8.657
SNHG19	8.531	8.481	8.588	8.491	8.308	8.552
SNHG18	7.141	7.295	7.373	7.472	7.170	7.274
AL161908.2	5.884	5.801	5.855	6.018	5.955	6.064
C9orf135-DT	6.247	6.106	6.097	6.657	6.209	6.225
IGHV1-69	7.655	7.838	7.869	7.825	7.775	7.867
THEM6	10.912	11.052	11.212	10.913	10.948	10.921
CYBB	6.986	7.193	7.066	7.253	7.186	7.199
CYBA	13.018	13.051	13.269	12.885	12.756	12.900
CEACAM19	10.079	10.293	10.315	10.043	10.085	10.110
AC005606.2	5.608	5.723	5.988	5.749	5.706	5.703
CIDEB	8.144	8.239	8.160	7.903	8.230	8.142
CIDEC	8.174	8.301	8.440	8.182	8.309	8.267
PGM5P3-AS1	7.383	7.470	7.517	7.591	7.447	7.467
CIDEA	7.588	7.572	7.522	7.816	7.544	7.568
FAM205BP	8.654	8.825	8.721	8.924	8.885	8.798
HSF1	10.457	10.467	10.638	10.394	10.288	10.246
TSPAN18	8.584	8.554	8.229	8.684	8.788	8.777
AL158032.1	4.291	4.101	4.144	4.315	4.267	4.252
HSF5	4.969	4.853	5.066	5.037	5.155	5.184
HSF4	8.086	8.023	8.167	8.356	8.291	8.296
RNPS1P1	11.871	11.908	11.941	11.543	11.535	11.525

PRPF4B	9.537	9.626	9.606	9.517	9.421	9.606
C7orf57	3.668	3.834	3.701	3.620	3.750	3.666
C7orf50	9.952	10.044	10.178	9.786	9.833	9.854
AC106886.6	9.488	9.608	9.790	9.768	9.895	9.826
AC040977.2	7.403	7.147	7.087	7.225	7.569	7.440
AC040977.1	7.690	7.773	7.482	7.350	7.474	7.540
RORC	8.547	8.622	8.449	8.592	8.493	8.622
RORB	6.315	6.613	6.456	6.596	6.850	6.716
RORA	7.732	7.583	7.703	7.599	7.551	7.504
ZNF479	6.759	6.711	6.644	6.939	6.943	6.850
CHEK2	8.082	8.173	8.316	8.066	8.082	8.180
MST1P2	8.186	8.305	8.122	8.250	8.252	8.202
ZNF471	4.533	4.793	4.440	4.707	4.710	4.643
ZNF470	4.820	4.957	4.663	5.185	4.817	4.942
ZNF473	8.602	8.591	8.846	8.576	8.595	8.740
ZNF474	6.684	6.663	6.669	6.830	6.544	6.744
FBH1	8.083	8.143	8.175	8.042	8.145	8.060
ROR1	10.203	10.501	10.579	10.378	10.355	9.872
SLTM	9.553	9.520	9.484	9.253	9.199	9.452
SFI1	7.775	7.999	8.055	7.850	7.919	7.920
MIR5196	7.177	7.142	7.305	6.759	7.047	6.945
KPNA6	11.218	11.394	11.338	11.082	11.262	10.945
KPNA5	7.267	7.436	7.570	7.190	7.002	7.463
KPNA4	11.684	11.607	11.679	11.573	11.623	11.690
KPNA3	9.917	9.901	9.933	9.848	9.777	9.910
KPNA2	11.882	11.907	11.917	11.581	11.775	11.682
KPNA1	9.364	9.249	9.200	9.498	9.431	9.335
AL137802.1	7.978	7.875	7.780	7.680	7.972	7.899
AL137802.2	6.719	6.805	6.764	6.834	6.732	6.855
AL137802.3	7.978	7.875	7.780	7.680	7.972	7.899
UNC119B	10.591	10.681	10.676	10.395	10.363	10.525
FRS3	7.710	7.497	7.689	7.699	7.645	7.720
FRS2	7.082	7.330	7.133	7.115	7.355	7.243
GOLT1B	9.656	9.718	9.746	9.914	9.846	9.914
GOLT1A	6.965	6.759	7.089	7.128	7.161	7.141
AC104640.1	3.852	3.853	3.810	3.849	3.865	3.815
LINC02610	7.506	7.536	7.642	7.267	7.480	7.267
LINC02616	6.210	6.227	6.165	6.179	6.220	6.087
LINC02615	5.851	5.883	5.838	5.717	5.731	5.754
B4GALT1	10.797	10.639	10.809	10.678	10.650	10.866
B4GALT3	9.836	9.860	9.839	9.657	9.746	9.748
B4GALT2	9.288	9.327	9.437	9.292	9.175	9.334
B4GALT5	10.997	11.033	11.108	11.009	11.046	11.078
B4GALT4	9.820	9.902	9.967	9.470	9.727	9.784
B4GALT7	8.647	8.803	8.914	8.588	8.663	8.612
B4GALT6	8.070	8.090	8.123	7.984	7.798	7.925
FCN3	6.495	6.325	6.539	6.838	6.886	6.543
FCN2	5.988	5.813	5.504	5.898	5.937	5.515
FCN1	6.427	6.498	6.298	6.520	6.470	6.759
OR5L2	7.050	7.161	6.860	7.097	7.336	7.335
TRRAP	7.824	7.827	7.769	7.584	7.775	7.852
LINC00612	5.333	5.374	5.331	5.418	5.221	5.300
LINC00615	5.210	5.197	5.040	5.254	5.467	5.236
LINC00616	4.732	4.857	4.565	4.868	4.832	4.785
FAM174C	8.996	8.966	9.063	8.751	8.807	8.828
FAM174B	8.321	8.377	8.538	8.155	8.336	8.340

FAM174A	9.212	9.182	9.219	9.190	9.014	9.060
CYP19A1	6.353	6.581	6.194	6.367	6.385	6.378
AC243922.3	7.508	7.289	7.527	7.633	7.611	7.536
SULT2B1	7.840	7.843	7.857	8.036	8.092	7.935
SVBP	8.470	8.575	8.728	8.197	8.263	8.360
SOGA3	6.707	6.826	6.941	7.042	6.836	7.004
SOGA1	8.360	8.028	8.355	8.322	8.358	8.228
COX17P1	9.418	9.379	9.661	9.406	9.471	9.510
DOHH	8.426	8.624	8.840	8.845	8.607	8.604
CRYGEP	7.681	7.550	7.632	7.803	7.628	7.544
EAF2	6.940	6.821	6.695	6.844	7.161	6.935
AC103796.1	6.622	6.463	6.475	6.526	6.637	6.583
AP001273.2	8.730	8.877	8.887	8.573	8.523	8.675
AF064858.3	6.715	6.818	6.926	6.877	6.933	6.653
AL590648.2	8.821	8.902	8.544	8.668	8.799	8.777
AL035665.1	6.986	6.863	6.944	7.092	7.158	7.214
AL121809.2	7.060	7.134	7.350	7.497	7.314	7.271
AC012339.1	4.236	4.270	4.052	4.016	4.240	4.222
FAM83H	11.966	11.964	12.079	11.870	11.826	11.838
PPP2R1A	11.542	11.635	11.729	11.553	11.474	11.431
PPP2R1B	8.802	8.960	8.987	8.663	8.677	8.759
ZMYND15	6.815	6.736	6.924	6.891	6.912	6.828
ZMYND12	6.048	6.304	6.097	6.325	6.205	6.241
ZMYND10	6.751	7.021	6.935	7.061	7.137	6.804
ZMYND11	10.871	10.813	10.872	10.653	10.636	10.533
TMUB1	9.191	9.480	9.675	9.562	9.492	9.517
SPSB1	7.306	7.298	7.192	7.194	7.316	7.283
ZMYND19	8.884	8.834	9.209	8.863	8.681	8.777
AC144573.1	5.665	5.647	5.531	5.601	5.643	5.647
FAM83F	8.333	8.348	8.447	8.162	8.306	8.163
HSPB6	8.263	8.007	8.136	8.642	8.537	8.553
HSPB7	7.676	7.447	6.978	7.503	7.586	7.311
URGCP-MRPS24	12.186	12.208	12.390	12.175	12.176	12.134
HSPB2	7.051	6.976	6.797	7.116	7.187	7.036
HSPB3	7.249	7.095	7.070	7.232	7.214	7.215
HSPB1	10.869	10.983	11.100	10.991	10.823	10.984
AL117190.4	6.393	6.679	6.768	6.991	6.907	6.783
HSPB8	8.154	8.214	8.231	8.257	8.112	7.987
HSPB9	6.764	6.910	6.795	7.085	6.794	6.828
DNM3OS	6.025	6.238	6.060	6.047	6.375	6.366
AC007560.1	10.524	10.657	10.675	10.471	10.494	10.472
AP006216.2	7.489	7.328	7.457	7.428	7.301	7.456
TMEM270	5.337	5.452	5.253	5.571	5.651	5.286
TMEM273	6.507	6.432	5.945	6.296	6.379	6.372
AP006216.1	6.097	6.096	6.223	6.292	6.275	5.988
PSME3IP1	10.860	10.884	10.989	10.887	10.877	10.935
AP000802.1	4.659	4.555	4.662	4.732	4.707	4.931
AC097374.1	5.843	5.956	5.645	5.694	5.997	6.062
TTTY6B	7.055	7.071	6.914	6.968	6.991	6.898
BMS1P17	4.384	4.179	4.266	4.012	4.127	4.194
LIG4	6.522	6.577	6.498	6.406	6.593	6.639
NAV3	6.817	6.871	6.841	7.033	6.790	6.880
NAV2	9.000	8.907	8.874	8.529	8.707	8.675
LIG1	9.296	9.338	9.556	8.780	8.730	8.929
BDP1	8.519	8.543	8.411	8.016	7.853	8.187
IREB2	9.893	9.849	9.839	9.580	9.623	9.829

BOLL	7.017	7.310	7.263	7.175	7.183	7.151
ATP5MG	11.170	11.231	11.358	11.098	11.090	11.206
ATP5MD	12.211	12.277	12.348	12.273	12.220	12.288
BARHL1	8.417	8.458	8.496	8.286	8.506	8.566
CSPG5	9.165	8.954	9.147	9.106	8.970	9.061
SCGB2A1	5.072	5.098	5.435	5.242	5.222	5.206
SCGB2A2	4.396	4.190	4.144	4.051	4.363	4.087
ZKSCAN1	9.894	9.880	9.941	9.601	9.400	9.755
ZKSCAN3	8.511	8.416	8.427	8.450	8.408	8.406
ZKSCAN2	8.297	8.315	8.326	8.213	8.362	8.321
ZKSCAN5	8.277	8.177	8.281	8.242	8.215	8.190
ZKSCAN4	7.995	8.055	8.048	8.031	8.085	8.085
ZKSCAN7	6.804	6.809	6.752	6.857	6.981	7.001
ZKSCAN8	8.526	8.736	8.776	8.611	8.554	8.655
AL160274.1	6.719	6.693	6.596	6.628	6.910	6.803
AJAP1	6.497	6.656	6.628	6.811	6.672	6.742
TMEM182	7.742	7.899	7.755	7.600	7.737	7.783
TMEM181	11.271	11.282	11.361	11.386	11.402	11.159
TMEM187	8.589	8.572	8.801	8.563	8.151	8.567
TMEM186	8.582	8.655	8.827	8.643	8.653	8.566
TMEM189	10.506	10.723	10.763	10.565	10.599	10.612
HCAR3	6.641	6.540	6.473	6.478	6.695	6.707
HCAR2	6.641	6.540	6.473	6.478	6.695	6.707
HCAR1	6.210	6.154	6.367	5.939	5.980	6.275
SCGB1A1	6.315	6.500	6.588	6.556	6.589	6.754
KCNH7-AS1	4.007	3.931	4.069	4.107	4.100	4.152
TAF6	10.097	10.215	10.229	10.195	10.079	10.062
TAF5	7.294	7.363	7.265	7.051	7.023	7.121
PRR23D1	5.370	5.394	5.407	5.751	5.892	5.738
TAF3	7.518	7.439	7.502	7.543	7.472	7.415
TAF2	8.910	8.982	8.768	9.018	9.065	9.013
TAF1	8.694	8.699	8.699	8.536	8.456	8.404
AC113554.1	6.830	6.645	6.817	6.989	7.024	7.162
HMOX1	7.511	7.373	7.661	7.641	7.402	7.602
TAF9	10.479	10.481	10.626	10.495	10.363	10.488
HMOX2	10.809	10.914	11.068	10.857	10.822	10.729
TSPAN32	7.362	7.518	7.495	7.689	7.704	7.584
TSPAN33	8.618	8.813	8.907	8.728	8.614	8.728
TSPAN31	10.442	10.579	10.632	9.890	10.401	10.433
ALAD	8.408	8.600	8.794	8.345	8.192	8.324
FOSB	7.634	7.679	7.445	8.365	8.224	8.482
ALG9	7.896	7.902	8.074	7.660	8.062	7.824
LRRCC1	6.464	6.473	6.260	6.370	6.399	6.385
FNIP1	8.715	8.670	8.822	8.525	8.475	8.593
FNIP2	10.664	10.729	10.787	10.470	10.741	10.576
BPTFP1	10.076	9.970	10.059	9.739	10.090	9.672
AKAP8L	8.437	8.423	8.650	8.682	8.616	8.549
DKKL1P1	7.064	7.050	7.266	7.186	7.496	7.182
ANKRD65	6.854	6.835	7.100	6.938	7.023	7.090
PDCD6	10.434	10.522	10.701	10.473	10.250	10.447
PSMA6	11.735	11.823	11.915	11.911	11.825	11.883
PDCD4	9.175	9.057	9.177	8.676	8.892	8.760
PDCD5	8.469	8.704	8.754	8.528	8.542	8.420
PDCD2	7.451	7.615	7.559	7.467	7.527	7.409
TCF21	6.787	6.688	6.653	6.918	7.029	6.851
PDCD1	7.744	7.736	7.823	7.792	7.737	7.862

AC067930.7	5.843	5.695	5.660	5.631	5.819	5.754
AC084782.1	4.558	4.482	4.477	4.627	4.437	4.664
AC067930.5	8.644	8.760	9.280	9.043	9.825	8.772
AC067930.4	5.843	5.695	5.660	5.631	5.819	5.754
AC067930.1	8.644	8.760	9.280	9.043	9.825	8.772
RN7SL724P	6.097	5.920	6.070	6.240	6.129	6.081
ARFGEF1	10.472	10.592	10.489	10.506	10.320	10.438
ARFGEF2	10.475	10.440	10.450	10.162	10.338	10.373
ARFGEF3	9.125	8.910	8.859	8.630	8.732	8.922
PLEKHA8P1	7.797	7.949	7.883	7.724	7.952	7.630
GVQW2	4.470	4.670	4.552	4.602	4.511	4.642
GVQW3	6.055	6.168	6.153	6.168	6.200	6.177
AC011467.7	6.981	6.965	6.836	6.957	6.886	6.903
DIAPH3-AS1	7.330	7.238	7.054	7.269	7.388	7.430
AL589863.1	5.647	5.865	5.767	5.946	5.538	5.729
AC207582.2	8.854	8.915	9.056	8.940	8.755	8.769
RAB39B	4.626	4.622	4.615	4.494	4.446	4.506
RAB39A	7.665	7.607	7.653	7.669	7.579	7.735
IL12B	4.293	4.200	4.226	4.253	4.230	4.093
IL12A	5.600	5.376	5.588	5.447	5.522	5.498
BORCS7-ASMT	5.589	5.504	5.610	5.521	5.693	5.756
FIGLA	5.148	5.496	5.407	5.365	5.487	5.683
RPIA	8.925	8.984	9.076	8.839	8.936	8.926
SEC22C	8.976	9.136	9.036	9.067	9.036	9.150
SEC22B	8.664	8.834	8.790	8.348	8.536	8.740
SEC22A	6.295	6.307	6.637	6.513	6.368	6.391
ELANE	6.038	6.073	6.214	6.254	6.151	6.064
CELA2A	5.422	5.273	5.486	5.415	5.501	5.429
RUBCN	9.274	9.217	9.177	8.879	8.944	9.084
CELA2B	5.504	5.563	5.792	6.231	5.779	5.754
PDCD10	12.167	12.188	12.235	12.336	12.168	12.391
PDCD11	8.823	8.833	8.856	8.635	8.560	8.668
IGKC	10.503	10.522	10.214	10.445	10.536	10.678
AP001048.1	6.763	6.792	6.676	6.977	7.028	6.713
PRNT	5.687	5.933	5.957	6.228	6.112	6.246
AP003119.3	7.336	7.340	7.375	7.325	7.432	7.351
AP003119.2	6.938	6.888	7.040	6.648	7.076	6.880
PRNP	10.622	10.759	10.769	10.290	10.422	10.504
TMEM59	12.025	12.101	12.269	12.201	12.150	12.146
PRND	6.204	6.320	6.078	6.214	6.358	6.356
TMEM54	11.131	11.201	11.483	11.151	11.101	11.083
SCLY	9.386	9.443	9.524	9.306	9.310	9.456
TMEM52	7.192	7.221	7.183	7.104	7.377	7.505
TMEM53	9.244	9.419	9.534	9.108	9.147	9.329
TMEM51	10.125	10.125	10.289	10.052	10.009	10.087
GOLGA6L22	8.692	8.361	8.050	8.620	8.552	8.728
AL136964.1	4.828	5.056	4.640	5.389	5.496	5.823
ZNF677	6.198	6.113	6.236	7.037	6.353	6.436
ZNF675	3.991	4.144	4.183	4.272	4.182	4.128
ZNF674	6.319	6.110	6.251	5.997	6.096	6.329
ZNF672	9.496	9.593	9.685	9.642	9.485	9.575
ZNF671	7.473	7.532	7.478	7.677	7.492	7.617
ZNF670	7.567	7.649	7.247	7.564	7.394	7.633
SNN	9.307	9.371	9.444	9.202	9.244	9.185
AC092910.3	6.425	6.168	6.162	6.254	6.192	6.455
KTI12	8.407	8.337	8.586	8.377	8.292	8.155

ZNF678	7.098	6.914	6.935	6.755	6.716	6.742
ACLY	12.535	12.663	12.700	12.601	12.488	12.679
MGST2	9.729	9.792	9.911	9.694	9.720	9.863
MGST3	11.435	11.633	11.793	11.575	11.617	11.590
MGST1	13.281	13.345	13.442	13.315	13.216	13.326
AC008894.3	7.568	7.675	7.580	7.500	7.442	7.724
GMDS	10.955	10.961	11.151	10.629	10.618	10.445
LSR	12.945	13.042	13.213	12.922	12.929	12.855
LSS	10.941	10.984	11.061	10.861	10.773	10.753
AGPAT3	10.487	10.602	10.690	10.662	10.685	10.376
PRSS55	6.290	6.338	6.378	6.385	6.368	6.428
PRSS54	5.821	6.083	6.146	6.086	6.226	6.076
PRSS50	7.029	6.991	6.979	7.194	7.444	7.205
PRSS53	8.303	8.433	8.407	8.293	8.257	8.339
MPV17	11.073	11.061	11.133	10.896	10.966	10.993
PRSS58	7.146	7.228	7.251	7.099	7.137	7.212
LIN28A	6.000	5.998	5.933	5.999	6.046	6.134
SNRPB2	9.934	10.032	10.089	10.011	10.011	10.077
SH3D19	8.591	8.614	8.759	8.674	8.721	8.694
TMEM198B	8.322	8.270	8.303	8.277	8.387	8.327
VAPB	10.969	11.037	11.117	10.884	10.880	10.856
VAPA	12.085	12.082	12.251	12.147	12.085	12.041
MAFIP	10.047	10.053	10.201	10.055	10.078	10.039
AL022100.2	8.809	8.749	8.552	8.615	8.786	8.730
TMEM154	9.520	9.387	9.457	9.255	9.475	9.046
BPIFA2	7.624	7.569	7.574	7.705	7.660	7.493
BPIFA3	8.757	8.813	8.694	8.823	8.922	9.014
BPIFA1	8.190	8.164	7.732	8.359	8.321	8.220
TMEM156	9.220	9.355	9.476	9.198	9.344	9.266
CLSTN2-AS1	6.029	5.874	5.816	5.971	6.061	6.145
AL353611.2	6.063	5.967	6.150	6.018	6.267	6.165
MAGI1-IT1	6.882	6.817	6.716	6.818	6.792	6.943
IFIH1	9.388	9.270	9.207	9.223	9.268	9.129
ACTR10	10.569	10.631	10.687	10.504	10.566	10.527
AC080038.2	6.661	6.557	6.738	6.778	6.761	6.947
ARL11	4.062	4.242	4.625	4.635	4.523	4.270
ARL10	11.415	11.480	11.661	11.405	11.423	11.326
ARL15	8.565	8.552	8.507	8.330	8.539	8.446
ARL14	11.846	11.943	11.894	11.821	11.613	11.796
ARL16	8.351	8.294	8.440	8.069	8.252	8.266
AL357568.2	4.954	5.117	4.982	5.157	5.210	5.325
WDR45B	12.021	12.135	12.197	11.946	11.956	11.992
AC104109.4	7.186	7.113	6.816	7.302	7.173	7.207
KIF4A	9.223	9.214	9.360	8.694	8.783	8.806
TMEM159	9.110	8.988	9.456	8.887	9.102	8.559
AL357874.3	6.078	6.209	6.278	6.152	6.017	6.349
LINC01858	4.873	4.920	5.112	5.231	5.053	5.294
AL357874.1	6.654	6.602	6.629	6.854	6.918	6.750
LINC01854	5.681	5.620	5.667	5.603	5.803	5.685
LINC01857	5.243	5.213	5.298	5.724	5.162	5.398
SOS1-IT1	4.676	4.875	4.670	4.897	4.736	4.784
LINC01852	6.586	6.580	6.787	6.622	6.669	6.723
DHRS7B	8.636	8.667	8.792	8.405	8.685	8.587
DHRS7C	6.556	6.370	5.969	6.637	6.590	6.282
DMTN	8.822	8.949	9.176	8.728	8.936	8.966
MANBAL	11.048	11.050	11.155	10.721	10.756	10.809

EIF4E3	8.796	8.880	8.941	8.706	8.772	8.819
EIF4E2	9.547	9.598	9.749	9.515	9.450	9.503
AL365205.1	11.796	11.909	12.049	12.073	12.009	12.047
ALG14-AS1	5.511	5.572	5.221	5.770	5.748	5.653
AC099343.3	4.270	4.480	4.502	4.476	4.289	4.304
WDR78	6.983	6.766	6.931	7.173	7.000	7.046
ACTR1B	10.019	10.087	10.179	10.057	9.913	9.953
ACTR1A	9.849	9.875	9.973	9.782	9.681	9.730
WDR70	8.529	8.553	8.617	8.595	8.534	8.515
WDR73	9.946	9.884	9.970	9.808	9.811	9.793
WDR72	7.980	7.965	8.119	7.740	7.781	7.957
WDR75	8.770	8.827	8.802	8.947	8.719	8.808
WDR74	9.778	9.894	10.093	9.710	9.862	9.830
WDR77	9.398	9.456	9.470	9.233	9.157	9.257
AC113935.1	13.304	13.343	13.490	13.360	13.298	13.417
LINC00892	5.228	5.341	5.153	5.091	5.198	5.247
LINC00893	7.600	7.504	7.088	7.595	7.505	7.519
TRIM67	8.042	8.127	7.986	8.067	8.014	8.198
LINC00896	7.235	7.280	7.361	7.423	7.625	7.591
LINC00894	7.600	7.504	7.088	7.595	7.505	7.519
MCF2	5.103	5.237	5.075	5.215	5.269	5.201
LINC00899	6.766	6.777	6.505	6.846	6.792	6.664
CDO1	6.513	6.668	6.047	6.383	6.502	6.593
PRPF8	10.501	10.573	10.633	10.342	10.151	10.360
RAPGEF4-AS1	5.679	5.514	5.295	5.497	5.297	5.727
PRPF3	8.574	8.629	8.696	8.489	8.275	8.315
PRPF4	9.769	9.810	9.945	9.736	9.993	9.754
PRPF6	10.439	10.529	10.652	10.400	10.219	10.367
Z93022.1	5.813	5.850	5.795	5.751	5.934	5.772
CD177	7.576	7.490	7.338	7.412	7.517	7.406
PCF11	8.106	8.042	8.169	8.086	7.831	8.100
SERPINB9P1	7.125	7.253	7.070	7.110	7.174	7.118
ZFP69	4.797	5.090	4.855	4.898	4.769	4.657
BTF3P11	4.167	4.261	4.204	4.237	4.711	4.303
BTF3P13	5.142	5.051	5.274	5.227	5.264	5.261
BTF3P12	7.305	7.297	7.318	7.238	7.228	7.289
PIP4K2A	9.061	9.008	9.061	8.915	8.962	8.920
ZFP62	8.571	8.667	8.570	8.361	8.353	8.424
PIP4K2C	10.066	10.157	10.177	9.963	9.773	10.033
PIP4K2B	10.029	9.951	10.112	9.954	9.926	9.916
ARL14EP	7.854	8.053	8.130	7.812	7.808	7.820
ZWINT	11.686	11.640	11.798	11.270	11.237	11.194
EGOT	6.627	6.453	6.510	6.745	6.692	6.647
AC100800.1	6.580	6.528	6.521	7.012	6.600	6.568
TTI2	9.478	9.634	9.751	9.582	9.628	9.564
CSAD	6.571	6.312	6.451	6.316	6.252	6.350
AC270149.1	3.830	4.000	4.066	3.900	3.760	4.082
CDON	7.743	8.016	7.730	7.549	7.725	7.882
KHDRBS2-OT	7.022	7.099	6.737	7.213	7.421	7.117
RPRM	7.587	7.872	7.830	7.796	7.658	7.767
DEFB118	8.053	8.171	7.873	8.107	8.156	8.270
DEFB119	5.651	5.417	5.458	5.773	5.775	5.797
DMBT1	8.030	8.305	8.053	8.074	7.999	8.209
PRKG1	7.397	7.452	7.177	7.346	7.601	7.610
PRKG2	7.667	7.745	8.159	8.083	7.924	7.758
MRE11	8.262	8.363	8.406	7.970	8.023	7.947

PMEPA1	13.119	13.147	13.211	13.085	13.178	13.074
LINC02135	4.425	4.370	4.438	4.699	4.596	4.339
LINC02139	5.535	5.621	5.580	5.720	5.905	5.615
CALML3	7.930	7.888	7.702	8.076	8.094	7.802
CALML6	6.494	6.547	6.564	6.723	6.629	6.701
CALML5	5.917	5.907	6.133	6.299	6.229	6.232
CALML4	8.960	8.988	9.172	8.634	8.748	8.702
CREM	7.938	8.169	8.171	8.010	8.229	8.127
PDGFRB	7.244	7.369	7.289	7.815	7.507	7.396
ANKRD13D	7.876	7.812	7.891	8.028	8.030	8.020
ANKRD13B	7.379	7.378	7.530	7.701	7.339	7.885
ANKRD13C	8.754	8.870	8.874	8.769	8.822	8.745
ANKRD13A	10.807	10.813	10.916	10.458	10.327	10.447
AIDA	10.721	10.779	10.847	10.685	10.605	10.682
PDGFRL	6.886	7.173	7.222	6.864	6.831	6.953
TMSB10	13.221	13.316	13.420	13.342	13.205	13.431
AL031123.5	6.383	6.250	6.078	6.509	6.225	6.364
MCTP1	9.236	9.166	9.288	8.872	8.933	9.057
MCTP2	9.467	9.498	9.295	9.375	9.260	9.405
AC129915.1	5.333	5.109	5.248	5.341	5.560	5.520
AC129915.3	5.333	5.109	5.248	5.341	5.560	5.520
RPL21P16	12.617	12.677	12.802	12.784	12.769	12.841
AC243967.3	3.986	3.824	3.810	3.988	3.886	3.935
TPM3P8	13.164	13.220	13.254	13.064	13.001	13.092
LINC02798	3.799	3.932	3.824	3.879	3.929	3.866
AL121761.1	7.314	7.278	7.352	7.482	7.458	7.286
LINC02796	5.920	6.005	5.915	6.069	6.126	6.030
TPM3P4	4.906	5.017	4.895	5.144	4.786	4.931
IER5L	9.054	9.119	9.054	9.045	9.115	9.182
LINC02790	7.678	7.537	7.631	7.601	7.678	7.665
PCIF1	9.674	9.617	9.821	9.629	9.735	9.514
LINC01442	5.223	5.418	5.427	5.483	5.462	5.632
NDOR1	8.145	8.175	8.306	8.209	8.314	8.383
AC007012.2	5.470	5.660	5.669	5.689	5.610	5.787
LINC01449	4.740	4.819	4.725	4.762	4.704	4.930
BABAM1	9.683	9.803	9.921	9.520	9.628	9.591
AC040174.1	3.763	3.790	3.829	3.827	3.612	3.746
BABAM2	10.476	10.516	10.570	10.507	10.319	10.357
RNU6-913P	5.467	5.248	5.351	5.277	5.316	5.440
SNORD88B	10.753	10.776	10.988	10.545	10.601	10.548
LINC02864	7.428	7.583	7.452	7.739	7.560	7.715
LINC02861	3.803	3.628	3.751	3.800	4.064	3.932
LINC02860	6.272	6.045	5.860	5.936	6.111	6.041
AGRP	7.244	7.309	7.488	7.172	7.338	7.423
AGRN	12.096	12.054	12.172	12.171	12.144	12.056
RABL6	10.556	10.606	11.043	10.775	10.922	10.269
AC145207.5	7.429	7.586	7.477	7.339	7.108	7.366
RABL3	8.146	7.995	8.061	8.014	8.097	7.976
AC145207.7	7.919	7.889	7.789	7.849	7.877	7.856
SYCN	7.190	6.948	6.762	7.149	7.322	7.249
MIR93	10.120	10.184	10.253	9.752	9.700	9.810
PACRGL	8.125	8.118	8.029	7.778	7.769	7.885
XAGE1A	6.866	6.915	7.116	7.241	6.974	7.277
XAGE1B	6.866	6.915	7.116	7.241	6.974	7.277
RUFY1	9.730	9.683	9.821	9.443	9.612	9.494
RUFY3	7.814	7.764	7.838	7.806	7.633	7.763

RUFY2	6.334	6.421	6.347	6.444	6.550	6.589
AGR2	13.924	13.969	13.976	14.000	14.136	13.974
AGR3	6.324	6.309	6.664	6.656	6.726	7.084
AC005674.2	6.459	6.269	6.253	6.418	6.471	6.362
TIGAR	8.671	8.717	8.787	8.666	8.611	8.617
NRG1-IT1	6.209	6.156	6.093	6.061	6.243	6.128
AC124947.2	6.118	6.227	6.072	6.015	5.708	5.891
GPAM	7.967	7.756	7.880	7.759	8.052	7.435
ACSM1	5.574	5.528	5.713	5.840	5.764	5.613
AC112206.2	6.831	6.890	6.698	6.936	6.932	6.869
ACSM5	7.486	7.584	7.558	7.514	7.563	7.495
NDC1	10.504	10.543	10.599	10.311	10.260	10.363
LINC00424	8.424	8.450	8.596	8.281	8.411	8.577
AC089998.1	4.689	4.733	4.713	4.616	4.815	4.887
EIF4ENIF1	8.623	8.710	8.794	8.584	8.432	8.707
AC012494.2	6.112	6.206	6.184	6.144	6.295	6.332
TRIM28	12.082	12.083	12.360	12.096	12.057	11.958
TRIM29	8.246	8.471	8.118	8.219	8.149	8.220
TRIM24	8.469	8.430	8.612	8.667	8.228	8.453
TRIM25	11.500	11.484	11.608	11.521	11.542	11.369
TRIM26	9.943	9.986	10.002	10.039	9.975	9.939
TRIM27	9.659	9.823	9.847	9.637	9.708	9.632
TRIM21	9.772	9.786	9.750	9.489	9.900	9.680
TRIM22	8.724	8.739	8.599	8.629	8.837	8.624
TRIM23	7.422	7.411	7.223	7.297	7.703	7.336
GNPAT	9.909	9.997	9.987	10.003	9.823	9.897
F2R	9.161	9.341	9.316	9.122	9.366	9.242
IHH	8.822	8.727	8.805	8.794	8.733	8.936
PPDPF	11.984	12.009	12.087	12.239	12.168	12.020
DNAJC28	5.185	5.030	5.264	5.353	5.264	5.158
DNAJC27	6.358	6.441	6.578	6.497	6.622	6.588
DNAJC25	7.202	7.147	7.110	7.137	7.236	7.112
DNAJC24	8.691	8.618	8.687	8.665	8.709	8.731
NTSR2	7.101	7.161	6.899	6.970	7.391	7.282
DNAJC22	9.267	9.387	9.632	9.116	9.443	8.646
DNAJC21	8.842	8.847	8.751	8.609	8.626	8.680
NTSR1	7.587	7.464	7.709	7.577	7.558	7.702
TRBV10-2	6.884	6.894	6.916	7.011	6.948	6.884
DTD1	9.181	9.213	9.430	9.167	9.128	9.038
DTD2	8.571	8.589	8.534	8.443	8.508	8.439
GSAP	8.525	8.466	8.527	8.211	8.053	8.328
AC009292.1	7.849	7.952	7.698	7.883	7.876	7.949
AC241401.2	8.943	8.649	8.679	8.388	8.372	8.812
HEMGN	5.766	5.380	5.836	6.035	5.785	5.726
AL158801.4	4.797	5.031	5.345	5.074	5.199	4.953
AL158801.6	12.031	12.165	12.312	12.106	12.010	12.060
CYCSP19	4.277	4.357	4.347	4.580	4.404	4.332
B4GAT1	9.294	9.268	9.479	9.337	9.276	9.345
BAG2	9.149	9.266	9.248	9.129	9.217	9.234
BAG3	10.451	10.448	10.502	10.429	10.539	10.523
BAG1	9.775	9.819	9.923	9.610	9.461	9.637
BAG6	11.565	11.631	11.792	11.522	11.346	11.444
BAG4	10.594	10.680	10.752	10.722	10.837	10.783
BAG5	10.350	10.415	10.462	10.136	10.070	10.196
KMO	6.017	5.718	5.597	6.146	6.035	6.104
ATXN1L	10.443	10.396	10.328	10.410	10.302	10.288

ZNF385C	6.985	6.817	6.760	7.085	6.990	6.932
ZNF385B	6.579	6.339	6.396	6.904	6.668	6.526
ZNF385A	8.219	7.985	8.048	8.090	8.358	8.171
ZNF385D	6.669	6.911	6.634	6.789	6.717	6.708
ATXN10	12.306	12.351	12.354	12.193	12.055	12.222
MYL12B	13.090	13.142	13.168	13.215	13.264	13.284
MYL12A	12.397	12.503	12.594	12.552	12.533	12.568
TMPO	11.199	11.275	11.265	10.822	10.800	10.737
ECM2	7.691	7.812	7.744	7.967	7.824	7.886
ECM1	7.502	7.580	7.436	7.475	7.491	7.553
AC087516.1	3.770	3.864	3.744	3.857	3.835	3.844
RAB3IL1	9.545	9.366	9.462	9.575	9.753	9.584
NAP1L1	12.485	12.687	12.749	12.676	12.531	12.499
NAP1L2	3.999	3.971	4.014	4.060	4.109	3.973
NAP1L3	4.226	4.260	4.190	4.202	3.997	4.031
NAP1L4	11.247	11.370	11.367	11.123	10.994	10.974
NAP1L5	4.675	4.724	4.924	5.082	4.905	4.985
AP003717.1	7.762	7.737	7.626	7.626	7.805	7.738
DYNC1I2P1	11.005	11.015	11.111	10.996	10.922	10.866
PEAK1	10.340	10.316	10.398	10.085	10.131	10.191
AL049649.1	5.180	5.225	4.744	5.075	5.281	5.355
IL1RAP	11.732	11.888	11.820	11.564	11.662	11.655
KIAA1143	8.916	9.053	9.079	8.681	8.630	8.794
KANK4	6.691	6.963	6.900	6.858	6.919	6.699
KANK2	8.900	8.755	8.893	8.576	8.599	8.548
KANK3	6.703	6.773	6.934	6.981	6.974	6.845
KANK1	8.707	8.817	8.669	8.598	8.728	8.852
AC114546.3	5.922	6.158	5.799	6.189	6.232	6.228
PDCL3P6	9.319	9.461	9.550	9.171	9.293	9.245
PDCL3P5	9.319	9.461	9.550	9.171	9.293	9.245
PDCL3P4	9.319	9.461	9.550	9.171	9.293	9.245
PDCL3P2	6.309	6.453	6.306	6.262	6.401	6.121
CANX	13.297	13.377	13.372	13.404	13.330	13.392
S100A11P1	12.364	12.506	12.544	12.546	12.621	12.488
AC012557.1	4.625	4.584	4.603	4.788	4.349	4.614
AL135908.1	5.666	5.529	5.512	5.714	5.613	5.431
ABHD8	7.741	7.755	7.912	7.718	7.787	7.690
MYLIP	8.557	8.594	8.621	8.829	8.553	8.608
ABHD2	12.020	11.987	11.978	11.787	11.735	11.597
ABHD3	10.780	10.782	10.778	10.770	10.772	10.782
ABHD1	7.262	7.323	6.990	7.398	7.545	7.347
ABHD6	7.651	7.545	7.688	7.692	7.716	7.617
AC011498.4	7.039	7.124	7.240	7.161	7.068	7.374
ABHD4	9.390	9.500	9.587	9.435	9.587	9.555
ABHD5	7.604	7.724	7.889	7.520	7.569	7.587
TWF1	10.423	10.582	10.647	10.488	10.413	10.579
TWF2	8.613	8.801	8.998	8.725	8.634	8.655
CFAP100	7.226	7.104	7.005	6.965	7.090	7.110
AC068594.1	6.248	6.279	5.888	6.054	6.167	6.048
KMT2E-AS1	6.052	6.197	6.523	6.537	6.534	6.329
AC068594.2	6.248	6.279	5.888	6.054	6.167	6.048
BCL2L2-PABPN1	11.770	11.818	12.043	11.535	11.668	11.517
AC243964.4	9.975	10.056	10.034	9.999	9.906	10.026
AC006517.6	6.360	6.376	6.591	6.594	6.723	6.582
SSU72	10.348	10.403	10.514	10.274	10.230	10.308
C5orf66	8.279	8.294	8.188	8.366	8.503	8.533

C5orf64	6.600	6.565	6.420	6.684	6.799	6.667
C5orf63	6.456	6.333	6.408	6.424	6.404	6.355
AP000223.1	4.969	4.510	4.643	4.757	4.645	4.736
AC105036.3	9.324	9.428	9.596	9.390	9.337	9.426
CBR3-AS1	7.841	8.021	8.040	7.716	8.011	8.054
RPL39P3	14.178	14.223	14.242	14.319	14.368	14.265
FAM47C	6.704	6.703	6.743	6.709	6.837	6.746
CCL3L3	5.971	5.771	6.090	5.970	6.216	5.995
FAM47A	4.757	4.706	4.798	4.962	4.681	4.861
FAM47E	4.457	4.302	4.442	4.315	4.325	4.217
GTF3A	11.338	11.399	11.623	11.420	11.434	11.378
CCL3L1	5.971	5.771	6.090	5.970	6.216	5.995
BATF2	8.311	8.364	8.276	8.390	8.607	8.204
AGO4	8.395	8.423	8.514	7.880	8.083	8.167
VPS72	10.244	10.356	10.472	10.282	10.308	10.322
AGO1	9.086	9.161	9.178	8.852	8.873	8.952
AGO3	7.995	7.947	8.067	8.024	8.159	7.758
AGO2	10.627	10.623	10.838	10.647	10.830	10.388
AC118553.2	9.937	9.964	10.062	9.824	9.715	9.786
FAM120B	8.203	8.276	8.214	8.309	8.353	8.376
FAM120C	8.180	8.204	8.042	7.919	7.967	7.908
FAM120A	12.170	12.201	12.264	12.187	12.276	11.880
MAPK14	9.763	9.839	9.848	9.808	9.735	9.811
MAPK15	8.050	8.160	8.096	8.368	8.303	8.255
MAPK10	6.515	6.602	6.504	6.393	6.650	6.447
MAPK11	8.779	8.846	8.907	8.736	8.566	8.706
MAPK12	9.305	9.211	9.272	9.624	9.468	9.533
MAPK13	9.282	9.481	9.405	9.306	9.195	9.298
SHISAL1	8.120	8.108	8.328	8.499	8.394	8.361
FZD1	7.606	7.697	7.760	7.740	7.636	7.520
FZD2	9.121	9.016	9.329	8.642	8.814	8.691
FZD3	8.047	7.923	7.777	8.119	8.023	8.038
FZD4	8.402	8.512	8.497	8.265	8.568	8.574
FZD5	9.336	9.301	9.356	9.031	8.943	9.100
FZD6	9.561	9.739	9.746	9.661	9.660	9.654
IQUB	4.896	4.766	4.922	4.966	5.113	4.990
SNORD114-3	4.569	4.356	4.343	4.452	4.229	4.350
HUS1	9.661	9.725	9.703	9.617	9.683	9.768
CYP2B6	11.859	11.821	11.840	11.319	11.236	11.239
C10orf71	6.604	6.558	6.829	7.049	6.620	6.781
ASH1L	10.149	9.791	9.852	9.844	9.964	9.881
UVRAG-DT	6.849	6.847	6.600	6.843	7.089	6.801
AMFR	10.338	10.245	10.216	9.919	10.009	9.999
USO1	10.279	9.968	9.982	9.885	9.961	10.022
SRARP	7.386	7.217	7.165	7.575	7.330	7.464
AC092651.1	8.228	8.464	8.401	7.869	8.135	8.215
PICK1	7.987	8.021	8.102	8.083	8.177	8.113
MRTO4	9.598	9.573	9.796	9.504	9.564	9.287
LPXN	8.523	8.643	8.477	8.488	8.639	8.450
AC093671.1	6.310	6.196	5.948	6.206	6.360	6.172
PLCG1-AS1	4.204	4.080	4.387	4.174	4.230	4.188
NOTCH3	8.895	8.872	9.159	8.426	8.641	8.782
STX18-AS1	5.807	5.520	5.549	5.772	5.645	5.520
AC103810.7	6.399	6.374	5.988	6.386	6.286	6.508
AC099684.1	6.024	5.886	5.792	6.444	5.959	5.955
AC103810.2	6.669	6.541	6.555	6.856	6.841	6.802

TMIGD3	5.820	5.511	5.632	5.924	5.749	5.861
ESCO2	7.643	7.667	7.701	7.534	7.634	7.561
ESCO1	9.291	9.323	9.331	9.332	9.281	9.432
NUP42	9.087	9.109	9.147	9.096	9.027	9.290
NUP43	10.102	10.110	10.252	10.042	9.982	9.938
AL353581.1	8.487	8.307	8.151	8.605	8.382	8.509
ZNF654	8.707	8.756	8.655	8.418	8.875	8.589
TCTEX1D1	5.518	5.591	5.470	5.780	5.551	5.454
AL162391.2	6.515	6.512	6.228	6.700	6.711	6.527
DTX2P1-UPK3BP	10.800	10.883	10.960	10.854	11.053	11.082
TARBP2	8.520	8.553	8.735	8.549	8.590	8.525
TARBP1	9.126	9.198	9.113	9.262	9.078	9.358
PPCS	9.965	10.001	10.141	9.720	9.848	9.940
PSPC1	9.331	9.306	9.290	8.902	8.999	8.784
MLLT10P1	4.785	4.916	4.823	4.938	4.891	5.068
AL035461.4	8.893	8.928	8.956	8.819	8.652	8.752
AC109811.1	5.718	5.853	5.689	5.855	5.827	6.052
SMIM10L2B-AS1	6.191	6.245	6.013	6.049	6.158	6.072
CHL1-AS2	7.636	7.642	7.938	8.076	7.918	7.862
CHL1-AS1	5.407	5.416	5.307	5.410	5.467	5.534
AC022257.1	4.409	4.464	4.159	4.454	4.334	4.605
AC104653.1	5.417	5.244	5.044	5.741	5.517	5.658
AC104653.2	5.417	5.244	5.044	5.741	5.517	5.658
COX20P1	9.167	9.249	9.282	8.939	9.168	9.223
CSF3R	7.984	7.965	7.899	7.988	8.092	8.010
AC010524.1	7.620	7.566	7.583	7.847	7.804	7.642
RAPGEF4	3.902	3.929	3.851	4.075	3.816	4.063
UBE4A	10.551	10.562	10.566	10.233	10.132	10.191
LINC00226	4.391	4.496	4.462	4.568	4.340	4.503
LINC00221	5.438	5.630	5.470	5.935	5.215	5.531
AC018892.3	6.962	6.796	6.949	6.813	6.961	6.895
MTREX	9.326	9.486	9.440	9.175	9.075	9.264
KRT18P44	6.325	6.467	6.546	6.014	6.062	6.321
PTGDR	6.415	6.120	6.343	6.334	6.332	6.371
PTGDS	8.661	8.681	8.585	8.582	8.744	8.750
TBC1D23	8.970	8.996	9.047	8.966	8.982	9.084
TBC1D21	7.258	7.461	7.726	7.554	7.547	7.474
TBC1D20	8.783	8.956	9.059	8.815	8.851	8.876
TBC1D26	7.638	7.937	7.866	7.949	7.941	8.171
ATR	9.595	9.652	9.546	9.509	9.328	9.472
LCMT1-AS2	7.972	7.943	8.100	8.332	8.215	8.143
TBC1D28	5.992	5.926	6.008	6.433	5.980	6.067
FUNDC2	10.153	10.226	10.319	10.438	10.375	10.360
TBC1D2B	10.336	10.468	10.390	10.074	9.853	10.024
FUNDC1	9.590	9.697	9.723	9.460	9.434	9.553
AC004023.1	5.262	5.369	5.133	5.307	5.490	5.322
RSU1P2	4.831	4.791	4.727	4.903	4.836	5.065
AC016596.1	6.860	6.777	6.627	6.671	6.806	6.688
TYRO3	8.951	8.832	8.792	8.735	8.653	8.633
ZNF382	4.694	4.682	4.691	4.796	4.920	4.763
CEP170B	10.991	11.051	11.042	10.888	10.830	11.037
ZNF823	6.865	6.840	7.007	6.867	6.790	6.515
WDFY4	6.861	7.009	6.869	7.296	7.249	7.323
WDFY3	9.003	8.927	8.900	8.936	8.866	9.031
WDFY2	8.069	8.031	8.277	8.363	8.164	8.281
WDFY1	9.541	9.736	9.649	9.377	9.315	9.320

CYP21A1P	7.212	7.474	7.435	7.470	7.513	7.440
TPI1P1	13.486	13.482	13.621	13.465	13.419	13.505
RN7SL832P	7.757	7.776	7.786	7.441	7.702	7.849
TMPRSS15	8.329	8.414	8.359	8.236	8.436	8.449
ANKRD35	7.189	7.326	7.521	7.557	7.620	7.475
ANKRD36	8.504	8.360	8.059	8.305	8.402	8.272
ANKRD37	8.135	8.197	8.242	8.306	8.267	8.331
ANKRD31	5.861	5.915	5.164	5.970	5.834	5.818
LINC00919	5.604	5.574	5.605	5.849	5.883	5.659
ANKRD33	6.607	6.635	6.605	6.943	6.852	6.811
LINC00917	6.473	6.728	6.826	6.748	6.719	6.567
ANKRD39	8.385	8.486	8.484	8.239	8.358	8.221
LINC00911	6.575	6.705	6.520	6.665	6.720	6.812
LINC00910	5.465	5.391	5.438	5.447	5.550	5.471
PRAME	7.299	7.228	7.336	7.238	7.574	7.211
AC270291.5	4.329	4.422	4.340	4.600	4.542	4.403
AC270291.4	8.020	8.145	8.297	8.088	8.003	8.078
AC097636.1	5.580	5.791	5.982	6.385	5.830	5.985
AC097636.2	6.337	6.162	6.285	6.385	6.353	6.341
MECR	9.120	9.188	9.350	9.086	9.101	9.187
TMEM121B	7.610	7.609	7.578	7.402	7.520	7.794
ZNF236-DT	6.082	5.983	5.798	5.911	6.056	5.775
SLC27A5	7.616	7.630	7.720	7.786	7.844	7.791
SLC27A4	8.500	8.663	8.636	8.540	8.548	8.669
SLC27A6	4.998	5.125	4.919	5.080	5.158	5.013
PRAM1	6.988	6.713	6.973	6.970	7.226	6.981
TXLNGY	7.050	6.937	6.889	6.977	7.138	7.066
SLC27A3	10.114	10.129	10.174	10.117	10.068	10.079
SLC27A2	9.689	9.869	9.959	9.534	9.445	9.634
AK9	6.814	6.815	6.571	6.937	7.042	6.690
AK8	8.344	8.087	8.220	8.439	8.439	8.267
TBCK	8.509	8.526	8.563	8.261	8.263	8.535
AK3	10.224	10.265	10.309	9.847	9.840	10.028
AK2	11.071	11.101	11.237	11.072	11.041	11.124
AK1	7.963	7.981	8.202	8.120	8.048	7.882
TBCD	9.065	9.107	9.270	8.975	9.010	8.876
AK7	8.902	8.965	8.963	8.834	9.046	8.812
AK6	8.862	8.837	8.915	8.815	8.817	8.876
AK5	7.208	7.122	7.101	7.202	7.368	7.388
AK4	11.529	11.637	11.831	11.799	11.904	11.498
WDR87	7.100	7.086	7.250	7.392	7.388	7.393
RAC1	13.587	13.570	13.616	13.492	13.357	13.508
MME	8.832	9.125	8.911	8.722	8.731	8.866
MMD	9.374	9.306	9.329	9.267	9.228	9.276
SH2D3A	8.566	8.579	8.677	8.274	8.315	8.306
MIR1238	8.231	8.214	8.264	8.028	8.292	8.270
LZTS1-AS1	6.955	7.194	7.044	7.366	7.106	7.378
PRR15L	10.368	10.353	10.587	10.252	10.245	10.288
AC080128.3	7.423	7.158	7.232	7.189	7.312	7.239
LIN9	5.604	5.549	5.587	5.681	5.590	5.562
SLC24A4	6.040	6.055	6.189	6.195	6.222	6.284
MDC1	8.228	8.276	8.325	8.245	8.147	8.282
SLC24A2	7.716	7.679	7.731	7.671	7.690	7.761
SLC24A3	6.438	6.321	5.983	6.377	6.172	6.159
SLC24A1	7.697	7.441	7.739	7.824	7.639	7.669
RPL12P16	13.555	13.621	13.669	13.572	13.470	13.564

SERTAD4-AS1	6.291	6.292	6.519	6.364	6.180	6.378
RPL12P11	7.561	7.622	7.564	7.549	7.565	7.718
C3AR1	5.886	5.533	5.645	5.646	5.962	5.876
ITLN1	6.121	6.328	6.309	6.197	6.072	6.200
SLC7A11-AS1	7.009	6.729	7.058	6.871	7.022	7.042
UHMK1	11.952	12.019	11.978	11.718	11.592	11.853
ZXDB	8.135	8.070	7.996	7.804	7.677	8.000
ZXDC	9.600	9.637	9.700	9.637	9.464	9.665
ZXDA	7.250	7.558	7.189	7.417	7.348	7.386
ZNF702P	4.391	4.648	4.534	4.502	4.500	4.696
ANKRD36C	4.861	5.007	4.897	4.904	5.162	4.815
ANKRD36B	8.504	8.360	8.059	8.305	8.402	8.272
OR2AD1P	6.725	6.829	6.512	6.765	6.816	6.963
AL031280.1	6.346	6.520	6.199	6.287	6.445	6.731
AC096656.1	6.671	6.519	6.253	6.959	6.585	6.652
Z97055.2	5.893	5.990	5.774	6.055	6.271	6.186
AC023830.1	6.809	6.865	6.797	6.831	6.886	6.756
AL589743.5	4.207	4.391	4.467	4.328	4.430	4.391
AC006205.2	6.516	6.144	6.176	6.549	6.629	6.552
AL589743.2	4.207	4.391	4.467	4.328	4.430	4.391
AL589743.1	8.071	7.941	7.789	8.159	7.815	8.096
EXOSC8	8.288	8.411	8.593	8.305	8.094	8.194
EXOSC9	9.058	9.102	9.217	8.948	9.199	8.884
BCKDK	9.587	9.653	9.831	9.680	9.600	9.635
EXOSC2	9.239	9.421	9.377	9.195	9.178	9.128
EXOSC3	7.978	8.016	8.158	7.984	7.893	8.081
EXOSC1	9.052	8.983	9.234	8.793	8.749	8.925
EXOSC6	8.856	8.708	8.837	8.488	8.527	8.627
EXOSC7	9.386	9.515	9.529	9.244	9.201	9.236
EXOSC4	10.472	10.581	10.785	10.512	10.425	10.389
EXOSC5	9.548	9.708	9.822	9.530	9.480	9.523
DEFB108B	4.396	4.571	4.221	4.462	4.509	4.397
DEFB108C	4.396	4.571	4.221	4.462	4.509	4.397
DEFB108A	4.396	4.571	4.221	4.462	4.509	4.397
DEFB108F	4.396	4.571	4.221	4.462	4.509	4.397
TULP4	9.750	9.663	10.115	9.246	9.959	9.129
LINC01635	6.039	5.962	5.939	5.976	5.946	6.009
DISC1	7.451	7.709	7.625	7.535	7.505	7.916
LINC01638	5.083	5.517	5.241	5.295	5.237	5.011
AC092652.2	5.325	5.002	5.066	5.228	5.080	5.205
INCENP	8.075	8.038	8.070	7.885	7.976	8.143
AC073861.1	14.097	14.100	14.202	14.276	14.229	14.242
ATP13A4-AS1	4.560	4.450	4.213	4.485	4.349	4.483
AC108676.1	6.900	6.992	6.935	6.886	6.905	6.890
AC024580.3	5.450	5.406	5.332	5.813	5.576	5.670
PSMB10	9.803	9.864	10.151	9.775	9.690	9.589
PAOX	7.699	7.894	7.895	7.974	7.862	7.906
SKP2	8.753	8.649	8.818	8.534	8.444	8.464
INS-IGF2	7.019	6.935	6.993	7.196	7.062	7.018
SKP1	12.764	12.952	13.000	12.813	12.829	12.952
NOC2L	9.534	9.586	9.727	9.610	9.519	9.559
CRYBG1	11.113	11.264	11.277	11.001	10.995	11.018
CRYBG3	8.543	8.622	8.472	8.470	8.356	8.372
CRYBG2	7.652	7.914	7.902	7.778	7.839	7.791
ZNF528-AS1	7.336	7.285	7.093	7.403	7.482	7.280
IFI16	10.535	10.605	10.609	10.320	10.425	10.400

TMEM30A	11.348	11.390	11.448	11.183	11.276	11.260
MC5R	7.841	8.066	7.560	7.887	7.924	7.935
RACK1	14.028	14.026	14.120	14.068	14.074	13.969
MIR503	8.618	8.474	8.711	8.715	8.546	8.939
GCSAML-AS1	4.490	4.387	4.404	4.455	4.493	4.418
TMEM30B	12.001	12.098	12.114	11.875	11.782	11.821
LRRFIP1	12.165	12.046	11.955	11.829	11.603	11.994
LRRFIP2	8.195	8.426	8.320	8.140	8.069	8.194
MARCHF10-DT	7.485	7.738	7.695	7.600	7.785	7.726
TFIP11	8.727	8.744	8.979	8.714	8.600	8.717
AP001324.1	13.691	13.753	13.856	13.823	13.660	13.829
LPAR3	9.173	9.337	9.552	9.336	9.388	9.118
AP001324.3	7.525	7.689	7.885	7.913	7.670	7.814
LPAR1	8.640	8.584	8.611	8.605	8.567	8.567
LPAR6	7.773	7.669	7.768	7.796	7.831	7.869
LPAR4	4.535	4.281	4.535	4.334	4.436	4.244
LPAR5	7.343	7.217	7.579	7.397	7.257	7.449
AC109326.1	6.308	6.150	6.257	6.553	6.508	6.681
HNRNPCP2	11.610	11.805	11.829	11.883	11.823	11.810
LIMS2	7.994	7.875	7.821	7.822	7.872	7.978
LIMS3	9.861	9.849	9.879	9.591	9.649	9.744
LIMS1	10.437	10.254	10.470	10.295	10.462	10.052
LIMS4	9.861	9.849	9.879	9.591	9.649	9.744
ST13P4	12.367	12.411	12.560	12.394	12.296	12.369
AC020917.4	8.161	8.104	8.282	8.258	8.185	8.233
SUN2	9.254	9.289	9.398	9.182	9.268	9.280
SUN3	4.157	4.107	4.096	4.180	4.308	4.068
SUN1	10.458	10.611	10.651	10.320	10.136	10.462
SUN5	8.857	9.050	8.928	8.848	8.974	8.999
AC105046.2	7.882	7.869	8.055	8.109	7.882	8.130
AC105046.1	7.060	6.947	6.866	6.851	7.120	6.997
AC142525.1	6.226	5.977	5.916	5.792	6.012	5.967
MRTFA-AS1	5.366	5.337	5.533	5.735	5.668	5.498
RBM14-RBM4	10.358	10.473	10.607	10.302	10.352	10.322
AC092587.1	9.968	9.978	10.093	10.085	10.117	9.975
SEC11A	12.573	12.586	12.787	12.563	12.485	12.518
SEC11C	9.837	10.057	10.140	10.275	10.340	10.205
ACKR4	5.949	5.951	6.022	5.795	6.376	5.837
ACKR1	7.005	7.140	7.116	7.121	6.895	7.131
ACKR2	7.862	7.994	7.545	7.850	7.873	7.894
ACKR3	4.270	4.634	4.721	4.766	4.398	4.620
CERT1	9.958	10.066	10.349	9.879	10.058	9.709
GPER1	7.238	7.397	7.355	7.619	7.400	7.565
QPCT	7.205	7.090	7.346	6.926	7.135	7.105
AC010834.1	9.046	9.024	9.183	9.146	9.218	9.194
SRP68	11.140	11.262	11.278	11.145	11.000	11.069
SEPTIN6	6.504	6.543	6.494	6.537	6.732	6.738
OMA1	8.933	9.048	9.081	9.041	9.000	9.093
PCYT1A	9.311	9.307	9.429	9.130	9.133	9.118
PCYT1B	6.416	6.553	6.517	6.768	6.628	6.612
AL162739.1	4.731	4.644	4.753	4.969	4.720	4.586
C5AR1	6.154	6.224	5.994	5.744	5.919	6.284
TRAV36DV7	5.115	4.979	5.136	5.170	5.424	5.032
PRRX1	7.164	7.142	6.933	7.212	7.040	7.147
PRRX2	8.311	8.368	8.493	8.753	8.704	8.550
RGR	7.020	6.869	6.730	7.055	7.348	7.226

USP29	6.347	6.499	6.515	6.568	6.656	6.817
MIR31HG	9.072	9.148	9.214	9.071	9.026	9.060
USP25	8.695	8.692	8.699	8.607	8.652	8.751
RGN	7.195	7.119	7.175	7.517	7.364	7.337
AC007614.1	5.872	6.025	5.766	5.459	5.620	5.872
USP22	10.758	10.660	10.952	10.724	10.794	10.630
CARD9	8.283	8.320	8.508	7.905	8.035	8.031
CARD8	8.765	8.691	8.682	8.454	8.644	8.671
NRAD1	5.147	5.183	5.019	5.144	4.930	5.109
CARD6	8.346	8.394	8.422	8.497	8.500	8.387
ERP27	8.621	8.689	8.630	8.612	8.615	8.741
ERP29	12.200	12.329	12.469	12.168	12.160	12.103
RPL4P2	13.400	13.519	13.640	13.401	13.266	13.398
RPL4P4	13.400	13.519	13.640	13.401	13.266	13.398
RPL4P5	13.400	13.519	13.640	13.401	13.266	13.398
AC093512.2	14.208	14.233	14.307	14.280	14.163	14.233
ABCA13	5.791	5.316	5.193	4.814	5.107	5.141
ARHGEF9	7.613	7.511	7.797	7.406	7.310	7.503
ARHGEF6	7.919	7.851	7.997	8.012	7.974	7.888
ARHGEF5	9.415	9.455	9.606	9.251	9.412	9.418
ARHGEF2	9.985	9.944	9.969	9.873	10.011	9.870
ARHGEF3	8.134	8.341	8.429	8.092	8.179	8.096
ADIRF	6.591	6.379	6.850	6.787	6.574	6.603
EOGT	6.534	6.452	6.463	6.074	6.148	6.023
AC009070.1	7.000	7.445	7.331	7.313	7.238	7.347
AC087284.1	7.217	7.208	6.904	7.470	7.309	7.491
RHBG	7.517	7.701	7.746	7.800	7.705	7.929
MTG2	9.399	9.457	9.477	9.524	9.580	9.570
BMP2K	8.433	8.389	8.297	8.295	8.254	8.254
MTG1	8.944	9.060	9.195	9.058	8.924	9.058
TSPEAR-AS2	7.316	7.276	7.324	7.536	7.397	7.458
ZNF318	8.071	8.084	8.287	8.002	8.117	8.027
TSPEAR-AS1	7.316	7.242	7.324	7.536	7.397	7.458
COCH	11.083	11.072	10.948	10.744	10.752	10.717
TEX30	7.622	7.654	7.636	7.603	7.613	7.616
TECRL	3.561	3.518	3.672	3.756	3.577	3.680
TEX37	7.922	7.971	7.641	8.091	8.184	8.221
AL133338.2	9.070	9.202	9.254	9.189	9.373	9.079
SP140L	9.492	9.443	9.358	9.166	9.329	9.250
CDH2	7.366	7.584	7.532	7.540	7.631	7.597
RBSN	8.813	8.775	8.819	8.443	8.666	8.506
AC016739.1	14.279	14.320	14.363	14.330	14.333	14.368
MAEL	5.592	5.617	5.619	6.135	5.852	5.783
RPL7P23	13.823	13.851	13.913	13.938	13.843	13.938
CACFD1	10.012	10.165	10.130	9.869	9.969	10.006
RPL7P27	4.661	4.843	4.734	4.630	4.690	5.014
RPL7P25	4.213	4.256	4.262	4.156	4.220	4.111
PLGLB1	5.577	5.786	5.603	5.688	5.748	5.615
PLGLB2	5.577	5.786	5.603	5.688	5.748	5.615
UFM1	9.436	9.599	9.491	9.289	9.430	9.400
TXNL4A	10.260	10.343	10.522	10.234	10.165	10.209
TXNL4B	8.878	8.961	8.963	8.657	8.829	8.758
FBLL1	7.957	8.126	8.030	7.932	8.001	7.947
RPS6KA2-AS1	6.596	6.628	6.397	6.671	6.493	6.729
NUTM2F	9.217	9.219	9.318	9.335	9.394	9.367
NUTM2G	9.217	9.219	9.318	9.335	9.394	9.367

NUTM2D	7.915	8.171	8.198	8.257	8.311	8.083
NUTM2E	7.915	8.171	8.198	8.257	8.311	8.083
NUTM2B	7.915	8.171	8.198	8.257	8.311	8.083
NUTM2A	7.915	8.171	8.198	8.257	8.311	8.083
POU3F3	5.854	5.864	5.833	6.031	5.941	5.852
POU3F2	7.361	7.146	7.310	7.565	7.378	7.287
POU3F1	7.318	7.166	7.416	7.573	7.550	7.405
POU3F4	5.749	5.675	5.617	6.060	5.523	5.748
FAM53B	8.665	8.664	8.764	8.609	8.812	8.597
HPGDS	6.262	6.374	6.265	6.446	6.371	6.501
FAM53A	6.841	6.880	6.845	6.334	6.527	6.555
FGD2	10.452	10.627	10.546	10.487	10.520	10.615
AHCY	11.157	11.265	11.450	11.108	10.923	11.096
SP100	10.271	10.315	10.380	9.975	10.001	9.702
AOC1	6.249	6.509	6.563	6.630	6.493	6.453
AOC3	6.449	6.120	6.341	6.588	6.354	6.465
AOC2	6.929	6.921	6.909	6.706	7.017	6.967
TIMP3	9.582	9.571	9.581	9.555	9.595	9.508
TIMP2	12.604	12.678	12.699	12.448	12.295	12.477
EPX	7.654	7.633	7.685	7.656	7.652	7.660
PARN	10.559	10.651	10.653	10.403	10.316	10.379
PARL	10.848	10.902	11.037	10.861	10.759	10.791
EPO	7.193	7.220	7.327	7.455	7.566	7.415
ZHX1-C8orf76	7.461	7.541	7.784	7.675	7.673	7.833
AC011092.1	8.278	8.330	8.503	8.137	8.336	8.328
MRPS30-DT	5.019	4.896	5.138	5.209	4.899	4.888
TSACC	9.022	9.075	9.163	9.451	9.280	9.256
PSORS1C1	6.565	6.726	6.684	6.580	6.649	6.411
PSORS1C3	6.035	6.180	6.104	6.593	5.964	6.192
PSORS1C2	7.800	7.761	7.544	7.827	7.794	7.825
PAGE2B	3.577	3.806	3.762	3.805	3.853	3.676
AC116903.2	4.834	4.992	4.670	4.768	4.740	4.591
AC023794.3	6.424	6.614	6.453	6.659	6.566	6.693
AL162425.1	5.780	5.804	5.828	5.978	5.819	5.800
SEMA7A	8.943	8.946	9.018	9.230	9.036	9.060
DDX4	5.356	5.529	5.437	5.422	5.798	5.639
DDX5	12.062	12.189	12.153	12.161	12.007	12.176
DDX6	9.960	9.894	10.012	9.615	9.268	9.664
DDX1	10.839	10.964	11.073	10.828	10.651	10.772
SCN3A	4.934	5.059	4.846	5.417	5.025	5.150
SCN3B	6.078	6.001	6.059	6.123	6.171	6.175
ZNF215	6.759	6.842	6.678	6.749	6.601	6.683
HLA-J	10.504	10.555	10.681	10.528	10.543	10.477
ZNF217	10.012	10.099	10.079	10.009	10.012	10.076
ZNF211	8.135	8.050	8.135	8.043	8.064	8.174
SLC7A8	9.281	9.291	9.365	9.284	9.367	9.220
SLC7A9	7.408	7.682	7.497	7.373	7.326	7.514
HLA-C	13.495	13.635	13.642	13.503	13.533	13.513
SMARCE1P2	6.717	6.514	6.414	6.164	6.407	6.540
HLA-A	13.650	13.726	13.781	13.676	13.731	13.707
SLC7A5	12.461	12.413	12.524	12.380	12.332	12.339
HLA-G	12.480	12.510	12.569	12.426	12.503	12.476
SLC7A3	6.583	6.593	6.639	6.601	6.556	6.575
HLA-E	11.637	11.611	11.700	11.267	11.443	11.393
PHF21B	5.792	5.559	5.643	5.748	5.761	5.595
EZHIP	5.755	6.004	5.641	5.892	5.878	5.777

AMER1	6.730	6.667	6.747	6.862	6.930	6.749
AMER2	7.335	7.444	7.579	7.523	7.451	7.459
AMER3	7.225	7.155	7.215	7.215	7.230	7.235
PRRT3-AS1	8.666	8.565	8.595	8.674	8.631	8.560
AL031656.1	7.199	7.203	7.125	7.025	7.085	7.104
SSR4P1	7.026	7.047	7.126	7.198	7.172	7.150
SNORD104	7.575	7.482	7.794	7.711	7.576	7.622
SNORD108	4.006	4.018	3.855	3.967	4.078	4.011
IPO8	10.036	9.948	10.010	9.888	9.803	9.845
IPO9	11.058	11.080	11.160	10.683	10.980	10.547
IPO7	11.659	11.759	11.742	11.655	11.861	11.500
IPO4	9.400	9.519	9.581	9.345	9.157	9.360
IPO5	8.937	8.940	9.141	8.778	8.800	8.925
NRBP2	9.147	9.110	9.327	9.054	8.728	9.091
NRBP1	9.309	9.338	9.494	9.361	9.300	9.312
AC007068.1	13.203	13.272	13.262	13.249	13.199	13.294
DCAF8L2	7.357	7.320	6.886	7.331	7.361	7.470
AP000526.1	4.376	4.773	4.668	4.763	4.535	4.427
RAB8B	8.982	8.929	8.994	8.703	8.782	8.917
RAB8A	11.544	11.608	11.800	11.454	11.466	11.337
LINC01345	4.999	5.167	5.054	4.948	5.005	5.030
LINC01347	8.340	8.109	7.986	8.227	8.359	8.262
LINC01346	6.046	6.211	6.197	6.121	6.205	6.582
LINC01341	7.531	7.398	7.471	7.424	7.430	7.505
LINC01343	6.477	6.527	6.548	6.521	6.705	6.672
LINC01342	7.724	7.682	7.582	7.655	7.426	7.537
SCPEP1	9.138	9.159	9.105	8.960	8.980	8.972
MGAT5B	7.712	7.962	7.699	7.905	7.783	7.787
AC098869.2	7.403	7.251	7.423	7.751	7.686	7.476
PAQR9	6.203	6.143	6.415	6.264	6.091	5.948
PAQR8	8.780	8.959	8.893	8.558	8.671	8.604
NIFK	10.797	10.894	10.988	10.957	10.988	10.675
GABPB1	9.394	9.224	9.431	9.526	9.569	9.432
AC012309.1	7.477	7.597	7.543	7.231	7.410	7.579
RBM12B-AS1	6.096	6.391	6.328	6.659	6.322	6.213
MRPL20-AS1	9.089	9.021	8.951	8.990	9.060	9.061
PHYHD1	6.356	6.056	6.092	6.365	6.365	6.386
PAQR3	6.991	6.941	7.075	6.748	6.797	6.826
STOX2	7.409	7.228	7.066	7.232	7.342	7.340
PAQR5	8.089	7.940	7.820	7.898	8.084	7.828
PAQR4	10.135	10.224	10.415	10.058	10.047	10.052
PAQR7	7.394	7.333	7.567	7.346	7.465	7.356
PAQR6	8.714	8.730	8.542	8.424	8.514	8.681
AC090821.2	5.935	5.896	5.685	6.054	6.039	5.941
FADS3	9.372	9.344	9.543	9.133	9.136	9.046
FADS2	8.562	8.716	8.936	8.822	8.723	8.883
ALDOAP2	5.882	5.555	5.680	6.224	5.799	6.011
SIL1	9.078	9.112	9.155	8.818	8.847	8.893
PRR5L	6.531	6.475	6.352	6.572	6.524	6.449
CARD8-AS1	6.865	6.803	6.860	6.943	7.008	6.820
LINC01968	6.956	7.228	6.979	7.050	7.267	7.117
LINC01961	5.236	4.952	5.099	5.354	5.191	5.427
LINC01963	7.665	7.646	7.697	7.373	7.329	7.441
ACAD11	8.575	8.579	8.525	8.311	8.263	8.463
AC090707.1	7.274	7.247	7.340	7.418	7.440	7.498
CARNS1	7.195	7.251	7.056	7.311	7.369	7.156

AC100797.3	6.866	6.947	6.572	6.687	6.807	6.806
AC100797.5	6.866	6.947	6.572	6.687	6.807	6.806
PLOD2	11.444	11.431	11.552	11.398	11.574	11.691
CMPK1	12.022	12.110	12.170	12.007	11.964	12.072
CMPK2	10.808	10.846	10.652	10.705	10.913	10.489
PLOD1	10.121	10.181	10.361	10.191	10.252	10.195
AC025810.1	5.609	5.685	5.052	5.559	5.724	5.811
FAAP20	8.266	8.337	8.671	8.131	7.912	8.079
FAAP24	7.292	7.422	7.598	7.360	7.445	7.267
RAD21	10.544	10.526	10.509	10.140	10.205	10.334
ADGRF1	12.102	12.248	12.215	12.060	12.018	12.110
ADGRF2	4.926	5.043	5.026	5.090	5.213	5.319
ADGRF3	7.481	7.498	7.349	7.264	7.538	7.493
ADGRF4	8.290	8.400	8.266	8.238	8.247	8.425
ADGRF5	6.536	6.479	6.278	6.526	6.651	6.475
ALDH6A1	10.386	10.491	10.651	10.440	10.382	10.417
NR2E1	6.778	6.644	6.670	6.755	6.868	6.832
NR2E3	6.579	6.665	6.396	6.445	6.687	6.656
OR2A20P	6.732	6.737	6.688	6.746	6.683	6.540
CEROX1	9.699	9.742	9.562	9.442	9.741	9.691
AL161901.1	4.926	4.880	4.881	5.069	4.811	5.013
AC026725.1	4.617	4.757	4.699	4.767	4.968	4.807
AC114980.1	3.885	3.930	3.849	4.024	3.908	3.828
DUS4L	7.208	6.916	7.048	6.786	6.984	6.955
FABP5P1	10.336	10.387	10.611	10.215	10.289	10.177
FABP5P2	10.336	10.387	10.611	10.215	10.289	10.177
FABP5P7	10.336	10.387	10.611	10.215	10.289	10.177
DAND5	6.753	6.965	6.844	7.174	7.002	7.012
JPH2	7.629	7.805	7.853	7.865	7.775	7.844
JPH3	7.421	7.433	7.425	8.031	7.653	7.699
AC009779.3	8.118	8.221	8.076	8.248	8.361	8.229
JPH1	8.385	8.361	8.316	7.880	7.863	8.026
AL121768.1	5.765	5.835	5.818	5.810	5.768	5.770
JPH4	7.253	7.015	7.149	7.397	7.358	7.326
TYK2	9.152	9.099	9.234	9.008	8.802	8.982
AC078846.1	7.755	7.428	7.701	7.688	7.854	7.642
BOD1	10.230	10.302	10.471	10.294	10.296	10.351
AC009962.1	4.221	4.048	4.099	4.031	4.321	4.076
SLITRK6	6.746	6.826	6.927	7.047	7.037	6.873
SLITRK4	4.456	4.343	4.337	4.571	4.408	4.532
SLITRK5	6.165	6.053	6.156	6.400	6.134	6.107
SLITRK2	7.251	7.304	7.220	7.711	7.547	7.460
SLITRK3	5.782	5.604	5.568	5.580	5.679	5.494
SLITRK1	5.861	5.725	5.671	5.957	5.869	6.000
CR382285.2	6.547	6.458	6.225	6.528	6.437	6.305
CR382285.1	8.174	8.070	8.261	8.185	8.174	8.173
PAPPA	7.110	7.113	6.879	7.123	7.075	7.107
PIK3C2B	9.211	9.325	9.262	9.138	9.202	9.294
PIK3C2A	10.781	10.896	10.904	10.783	10.833	10.803
PIK3C2G	8.447	8.564	8.468	8.515	8.403	8.505
AC055811.1	6.921	7.049	7.055	7.136	7.116	7.134
AC055811.3	7.725	7.936	7.682	7.700	7.989	7.688
AC055811.4	7.347	7.160	7.132	7.269	7.421	7.139
AC109460.3	9.304	9.382	9.542	9.098	9.022	9.087
LST1	7.977	7.994	8.137	8.033	8.227	8.046
LDAH	8.935	9.046	8.971	8.864	8.964	9.134

CEP76	7.231	7.409	7.314	7.114	7.213	7.118
CEP70	9.187	9.090	9.124	9.002	8.966	9.009
AC105339.2	7.259	7.010	6.981	6.784	6.928	7.209
CEP72	6.955	6.984	7.144	6.605	6.854	6.842
CEP78	7.907	7.978	8.132	7.889	7.857	7.909
TSN	11.944	12.061	12.133	12.025	11.938	11.885
BAIAP2-DT	8.325	8.270	8.349	8.221	8.218	8.346
AC136624.1	7.256	7.004	7.129	7.152	6.993	7.140
TPK1	7.828	7.923	7.855	7.530	7.824	7.763
AL591438.1	8.174	8.070	8.261	8.185	8.174	8.173
TANK	11.293	11.428	11.403	11.558	11.579	11.377
MYBPH	7.271	7.498	7.542	7.656	7.607	7.618
MTHFSD	8.382	8.399	8.431	8.523	8.286	8.327
MST1	8.186	8.305	8.122	8.250	8.252	8.202
AC010503.5	8.493	8.660	8.707	8.255	8.272	8.248
AP000280.2	7.959	7.950	7.940	7.924	8.027	7.988
AL391825.1	4.584	4.307	4.632	4.572	4.307	4.663
B3GLCT	7.936	8.038	8.063	8.022	7.909	8.050
CYP3A43	6.861	6.932	6.949	6.997	7.024	7.049
VNN1	7.768	7.606	7.642	7.338	7.368	7.545
SMG7	10.284	10.295	10.286	10.258	10.177	10.273
MSTN	3.772	3.828	4.009	3.864	3.768	3.835
SMG6	8.250	8.196	8.416	8.343	8.349	8.329
SMG1	10.597	10.561	10.378	10.328	10.283	10.463
EEF1A1P19	14.129	14.208	14.172	14.187	14.279	14.288
HMGB1P12	6.771	6.593	6.049	6.952	6.739	6.726
HMGB1P10	13.082	13.095	13.157	12.934	12.997	12.733
HMGB1P17	4.008	4.155	3.849	3.854	4.005	3.713
TRBV25-1	5.215	5.209	5.309	5.375	5.275	5.357
EPDR1	11.064	11.117	11.195	10.998	10.886	10.976
AC006254.1	7.709	7.730	7.809	7.884	8.014	7.993
SYS1	9.821	9.878	10.040	9.954	10.043	9.941
NEURL1B	7.429	7.452	7.136	7.311	7.469	7.484
SLC30A10	5.278	4.909	5.283	5.273	5.526	5.389
FAM204A	8.139	8.077	8.484	8.250	8.198	8.245
LINC02086	7.983	7.985	7.931	7.977	7.919	7.815
LINC02085	4.745	4.637	4.630	5.006	4.751	4.882
AC243629.3	6.645	6.735	6.687	6.662	6.825	6.701
ROM1	7.530	7.411	7.541	7.987	7.566	7.525
PPEF2	3.996	3.897	3.983	3.986	3.925	3.913
PPEF1	5.811	6.027	5.920	5.810	5.797	6.028
AC270289.3	8.551	8.661	8.822	8.340	8.637	8.479
AFDN	10.451	10.512	10.468	10.262	10.222	10.290
LINC02555	3.850	3.803	4.017	3.810	3.852	3.886
FITM2	9.751	9.668	9.879	9.571	9.630	9.594
FITM1	6.170	6.162	6.214	6.136	6.209	6.299
LINC02551	3.469	3.547	3.877	4.159	3.799	3.933
GREB1L	7.614	7.574	7.537	7.921	7.860	7.949
AP1S1	10.032	10.495	10.706	10.607	10.537	10.024
AP1S2	10.375	10.390	10.491	10.138	10.235	10.282
AP1S3	9.431	9.559	9.700	9.278	9.579	9.304
AL049646.1	7.871	7.848	8.037	7.954	7.955	7.949
KAZN	8.148	8.185	8.241	8.086	8.074	8.231
AL096869.3	7.745	7.557	7.485	8.198	7.974	8.055
AL096869.2	4.798	4.701	4.586	4.358	4.499	4.459
PELO	9.294	9.227	9.367	9.096	9.133	9.254

POTED	3.830	4.000	4.066	3.900	3.760	4.082
POTEC	3.830	4.000	4.066	3.900	3.760	4.082
POTEB	3.830	4.000	4.066	3.900	3.760	4.082
AC244258.1	8.034	8.129	8.110	7.831	7.767	7.879
ASIP	7.331	7.341	7.193	7.579	7.336	7.411
C21orf91	7.455	7.403	7.177	7.545	7.616	7.468
DCSTAMP	6.909	7.037	6.891	7.128	7.099	7.115
GPR45	6.599	6.780	6.902	6.926	6.960	7.042
AC022400.3	9.081	9.018	9.114	9.210	9.131	9.065
PITRM1-AS1	6.044	6.192	5.754	5.946	5.937	6.240
AC022400.6	7.822	7.672	7.790	7.876	7.791	7.855
AC022400.7	8.735	8.674	8.797	8.657	8.756	8.827
AC004917.1	7.107	7.075	6.993	7.102	7.041	7.326
ARMCX6	10.408	10.362	10.522	10.048	10.096	10.189
ARMCX5	9.029	8.997	9.071	8.898	8.964	8.996
ARMCX4	7.875	7.940	7.567	7.922	7.993	7.812
ARMCX3	9.189	9.189	9.353	9.106	9.283	9.035
ARMCX2	5.688	6.026	5.709	5.855	5.951	6.000
ARMCX1	4.827	4.910	4.793	4.839	4.832	5.071
FUT7	8.258	8.049	8.428	8.607	8.394	8.223
FUT6	9.940	9.952	9.969	9.933	10.050	9.989
FUT5	8.192	8.332	8.481	8.335	8.519	8.204
FUT4	9.495	9.557	9.517	9.424	9.350	9.472
FUT3	8.760	8.832	8.973	8.849	8.906	8.710
FUT2	7.653	7.556	7.623	7.592	7.601	7.518
FUT1	7.636	7.705	7.820	7.560	7.663	7.574
AL359922.1	9.112	9.178	9.210	8.914	8.743	8.902
MAGEA9B	6.533	6.531	6.239	6.507	6.605	6.747
KRT33A	6.467	6.494	6.591	6.606	6.722	6.590
FUT9	5.357	5.179	5.240	5.237	5.388	5.115
FUT8	10.042	9.984	10.021	9.965	9.929	10.022
GHR	4.772	4.981	5.317	5.443	4.993	5.025
AL450226.1	7.299	7.250	7.167	7.546	7.355	7.281
SF1	11.077	11.049	11.236	10.921	10.812	11.037
VTCN1	10.305	10.366	10.515	10.249	10.296	10.300
H2BW2	6.727	6.467	6.552	6.666	6.609	6.727
AL034370.1	10.882	10.871	10.930	10.811	10.727	10.969
DDX39B	12.229	12.254	12.337	12.254	12.175	12.212
VPS9D1	8.035	8.012	8.169	7.815	7.750	8.001
DDX39A	10.520	10.552	10.632	10.472	10.382	10.458
PXT1	5.770	5.761	5.672	5.776	5.819	5.884
PLXND1	7.559	7.641	7.780	7.642	7.628	7.645
PHETA1	8.986	9.189	9.365	9.104	9.054	8.962
PA2G4P2	7.050	6.859	6.959	6.882	7.014	7.062
AL033527.3	7.692	7.649	7.975	8.287	8.003	8.018
RPL23AP1	9.575	9.758	9.859	9.538	9.695	10.000
RPL23AP2	14.314	14.336	14.355	14.352	14.254	14.327
RPL23AP4	7.667	7.929	8.164	7.639	7.672	7.716
RPL23AP7	7.667	7.929	8.164	7.639	7.672	7.716
PEX11A	8.626	8.791	8.755	8.798	8.767	8.823
PEX11B	9.757	9.744	9.807	9.554	9.637	9.676
OVOL1-AS1	8.692	8.632	8.654	8.964	8.966	8.718
PEX11G	8.415	8.455	8.546	8.616	8.617	8.567
AC006518.7	7.199	7.315	7.449	7.834	7.457	7.479
PAFAH1B1	9.781	9.747	9.831	9.654	9.782	9.619
PAFAH1B3	9.992	10.155	10.309	9.909	9.816	9.982

PAFAH1B2	10.473	10.818	10.958	10.730	10.767	10.312
AC110285.2	7.233	7.202	7.015	7.573	7.358	7.420
AC244260.2	4.546	4.452	4.404	4.410	4.521	4.445
GH1	7.738	7.861	7.945	7.774	7.988	7.983
GH2	7.991	8.026	8.129	8.409	8.242	8.299
SFN	12.259	12.321	12.493	12.124	12.189	12.000
GATAD1	10.227	10.216	10.183	10.229	10.494	9.879
AC018358.1	6.515	6.659	6.605	6.753	6.698	6.859
STARD10	11.072	11.089	11.176	11.197	11.308	11.095
AP003071.5	8.522	8.420	8.664	8.748	8.728	8.559
AP003071.2	5.991	6.047	6.055	6.117	6.351	5.943
CYBC1	8.840	8.872	9.126	8.544	8.703	8.622
MT1X	9.520	9.784	9.844	9.822	10.025	9.887
NADK2-AS1	5.251	5.318	5.281	5.777	5.747	5.374
AC103706.1	7.642	7.635	7.791	7.923	7.765	7.833
MT1M	4.861	5.032	4.790	5.218	5.224	5.037
STARD9	5.246	5.572	5.408	5.399	5.244	5.434
MT1H	10.143	10.158	10.409	10.358	10.512	10.349
MT1E	8.951	9.080	9.062	9.118	9.193	9.185
MT1F	9.295	9.386	9.610	9.484	9.648	9.542
MT1G	9.377	9.639	9.709	9.689	9.790	9.760
BLVRB	11.473	11.561	11.806	11.522	11.452	11.514
BLVRA	10.317	10.405	10.460	10.075	10.144	10.167
GLTPD2	6.854	7.071	7.319	7.274	7.137	7.207
WBP2NL	4.128	3.814	3.992	4.140	4.340	3.922
STARD4	10.563	10.575	10.550	10.446	10.290	10.471
TSPY11P	5.699	6.018	5.845	6.396	6.072	5.960
OR1J4	6.466	6.527	6.430	6.585	6.439	6.461
AC073072.1	12.821	12.916	13.024	12.923	12.903	12.978
OR1J2	8.297	8.523	8.248	8.090	8.330	8.435
AL136040.1	6.066	6.051	6.057	6.034	5.866	6.036
CAPZB	11.626	11.779	11.906	11.689	11.790	11.634
AL355102.3	5.485	5.540	5.457	5.381	5.396	5.581
AL355102.2	7.025	6.908	6.669	6.940	6.915	7.028
AC063949.2	4.704	4.633	4.776	4.817	4.570	4.733
EGFLAM-AS4	5.933	5.752	5.708	6.138	5.813	6.098
FBXL19-AS1	7.695	7.577	7.213	7.464	7.641	7.630
EGFLAM-AS2	5.748	5.635	5.820	5.946	5.782	5.896
CERCAM	8.279	8.342	8.266	8.224	8.415	8.300
NAE1	10.328	10.464	10.581	10.400	10.353	10.325
GXYLT1	7.172	7.126	7.408	7.204	7.221	7.106
GXYLT2	6.441	6.184	6.430	6.330	6.165	6.196
CSPP1	7.913	7.711	7.883	8.029	8.205	7.582
SNTG2-AS1	4.353	4.238	4.337	4.417	4.400	4.358
AL034403.1	6.340	6.292	6.326	6.362	6.226	6.390
TBCEL	6.649	6.592	6.381	6.526	6.702	6.864
PUS1	8.130	8.050	8.130	7.960	7.859	7.925
DNAJC27-AS1	5.693	5.750	5.821	5.622	5.786	5.940
AC087498.1	7.282	7.452	7.449	7.682	7.534	7.449
FGD6	9.500	9.523	9.459	9.485	9.477	9.409
F13A1	7.039	6.992	6.639	6.818	6.968	6.903
NOP58	10.872	10.877	10.869	10.662	10.668	10.645
NOP56	10.151	10.221	10.311	10.049	9.841	10.024
NOP53	12.815	12.922	13.138	12.703	12.544	12.636
ETS2	9.000	9.113	9.228	9.328	9.142	9.338
ETS1	8.778	8.763	8.731	8.575	8.536	8.469

AQP10	6.721	6.619	6.658	6.727	6.696	6.761
AQP11	7.393	7.579	7.388	7.128	7.485	7.458
AC036108.3	6.619	6.602	6.687	6.802	6.760	6.751
AC012417.1	5.911	5.860	6.149	5.894	5.973	6.070
AF106564.1	6.570	6.621	6.441	6.402	6.769	6.634
CHRM4	7.404	7.803	7.678	7.615	7.699	7.860
CHRM3	7.398	7.220	7.070	7.298	7.449	7.495
CHRM2	6.901	6.959	6.730	6.867	6.885	6.646
CHRM1	6.038	6.184	6.123	6.151	6.192	6.218
MGAT1	9.610	9.753	9.850	9.653	9.787	9.685
MGAT2	10.819	10.911	11.086	10.982	10.953	11.005
MGAT3	7.803	7.805	8.080	8.002	8.135	7.922
MGAT5	11.198	11.244	11.382	11.170	11.016	11.175
GLRX	10.053	10.135	10.195	10.164	10.258	10.171
AC073476.3	5.385	5.384	5.337	5.357	5.494	5.563
MMP9	7.469	7.509	7.679	7.479	7.505	7.618
MMP7	10.591	10.673	10.824	10.607	10.575	10.476
MMP2	6.661	6.637	6.666	6.530	6.624	6.737
MMP3	4.758	4.867	4.825	4.788	4.944	4.965
MMP1	10.590	10.698	10.761	10.034	10.313	10.178
TRAPPC2L	10.880	11.018	11.094	10.947	10.826	10.965
XIAPP1	7.349	7.404	7.569	7.391	7.258	6.889
GNPTAB	8.788	8.815	8.734	8.447	8.534	8.642
TRAPPC2B	8.465	8.524	8.878	8.444	8.490	8.579
NGDN	10.248	10.266	10.389	10.183	10.249	10.151
AC256236.2	8.943	8.649	8.679	8.388	8.372	8.812
CDK7P1	9.039	9.118	9.293	8.902	8.991	8.997
CBFA2T3	6.141	6.403	6.250	6.471	6.393	6.528
CBFA2T2	8.886	8.869	8.926	8.790	8.757	8.791
IRF2BP2	12.480	12.525	12.718	12.393	12.468	12.358
IRF2BP1	9.636	9.612	9.760	9.830	10.059	9.697
C9orf85	8.497	8.508	8.593	8.316	8.254	8.405
IRF2BPL	10.738	10.770	10.905	10.702	10.731	10.691
USP38	9.066	9.127	9.188	9.082	9.039	9.070
USP39	8.806	8.893	9.021	8.885	8.911	8.828
USP30	8.084	8.133	8.157	7.945	7.913	8.009
USP31	9.341	9.450	9.552	9.360	9.513	9.365
USP32	8.816	8.726	8.703	8.342	8.439	8.740
USP33	9.954	9.952	9.961	9.813	9.745	9.893
USP34	9.974	9.956	9.900	9.683	9.595	9.834
SHMT2	11.427	11.429	11.571	11.241	11.069	11.210
SHMT1	11.167	11.066	11.210	11.165	11.157	11.186
USP37	8.007	7.888	7.938	7.796	8.005	7.819
AC091729.3	6.797	6.766	6.831	6.831	6.798	6.624
AP000897.1	7.075	7.047	6.943	7.196	7.229	7.121
RPL23AP60	7.667	7.929	8.164	7.639	7.672	7.716
PPFIBP1	8.972	9.574	9.729	9.310	9.326	9.468
PPFIBP2	10.104	10.170	10.233	9.869	9.826	9.903
AL512590.1	6.144	6.237	6.348	6.291	6.465	6.339
LDLRAD4	8.470	8.438	8.319	8.536	8.523	8.372
AC009133.6	9.870	9.936	10.043	9.909	9.829	9.846
AC009133.1	8.492	8.579	8.874	9.016	8.773	8.610
AC009133.2	6.763	6.728	6.921	7.228	6.951	6.823
AL109954.2	6.894	7.069	7.022	6.999	7.113	7.098
HHIPL1	6.561	6.620	6.435	6.651	6.623	6.852
AP001062.1	7.647	7.705	7.944	7.874	7.711	7.906

HHIPL2	7.653	7.698	7.665	7.749	7.845	7.771
C8orf34-AS1	5.155	5.183	5.270	5.460	5.407	5.208
FAM86DP	9.708	9.675	9.729	9.656	9.678	9.650
KIF26A	5.579	5.538	5.806	5.670	5.643	5.454
KIF26B	7.023	7.061	7.036	7.080	7.411	7.118
SAAL1	9.701	9.900	9.854	9.666	9.704	9.622
UHRF1BP1	8.952	8.916	8.828	8.679	8.758	8.835
UBAP2L	10.275	10.280	10.346	10.164	10.298	10.153
SUPT7L	9.225	9.198	9.227	9.203	9.071	9.182
TNRC18	10.788	10.734	10.805	10.601	10.644	10.741
JUN	10.472	10.507	10.521	11.877	11.865	11.984
CLIP1-AS1	5.512	5.246	5.606	5.559	5.500	5.600
JUP	12.570	12.573	12.723	12.607	12.515	12.581
CYP4Z1	4.960	5.076	4.992	5.001	5.102	5.068
REV1	9.029	8.925	8.943	8.931	8.904	8.892
DACT3-AS1	6.773	6.849	6.827	6.817	6.755	6.905
RHBDF1	9.934	10.098	10.046	9.920	9.913	9.995
RHBDF2	8.726	8.754	8.812	8.560	8.599	8.447
AL731537.2	4.267	4.245	4.159	4.498	4.435	4.397
CTNND2	6.510	6.572	6.627	6.722	6.736	6.399
CTNND1	11.109	11.264	11.331	11.272	11.299	11.152
OR8G3P	5.670	5.716	5.271	5.614	5.763	5.628
ATPSCKMT	7.642	7.863	8.074	7.732	7.721	7.773
ZNF536	7.026	6.958	6.869	6.756	6.924	7.054
VASH1-AS1	8.537	8.509	8.652	8.432	8.591	8.460
ZNF532	10.443	10.489	10.448	10.418	10.257	10.465
QSER1	10.304	10.418	10.677	10.464	10.541	9.778
AC079145.1	7.108	7.473	7.593	7.495	7.572	7.540
TOMM70	10.322	10.310	10.327	10.300	10.187	10.185
ZNF649-AS1	7.288	7.171	7.209	7.342	7.281	7.307
AC124067.4	6.528	6.480	6.756	6.674	6.536	6.441
AC015908.6	8.076	8.141	8.045	8.163	8.307	8.180
ATL3	10.871	10.846	10.972	10.734	10.536	10.645
ATL2	9.884	9.923	10.021	9.778	9.960	9.882
ATL1	6.774	6.875	7.218	6.780	7.140	7.035
MEGF9	8.478	8.519	8.611	8.183	8.264	8.355
ZMIZ1-AS1	7.015	6.994	6.859	7.000	6.867	6.891
AC004477.1	7.857	7.903	7.905	7.770	7.912	7.919
SLC5A5	8.841	8.826	8.890	8.818	8.920	8.930
ANOS2P	7.401	7.739	7.637	7.689	7.884	7.839
MOXD1	9.191	9.213	9.278	9.009	9.104	9.073
PIN1	8.469	8.433	8.617	8.338	8.416	8.344
PIN4	9.825	9.885	10.013	9.807	9.654	9.761
SMOX	8.894	8.844	8.878	8.882	8.889	8.935
NPAS4	5.305	5.382	5.436	5.790	5.681	5.662
NPAS3	7.555	7.552	7.623	7.660	7.592	7.758
NPAS2	10.324	10.498	10.574	10.238	10.230	10.235
NPAS1	7.676	7.339	7.682	7.793	7.487	7.483
AC012085.1	12.759	12.888	13.016	12.823	12.758	12.866
LINC02716	7.126	7.115	7.399	7.504	7.332	7.143
AHSA2P	8.062	7.936	7.762	7.741	7.552	8.024
LINC02712	5.217	4.782	4.885	5.208	5.326	5.099
LINC02718	5.277	5.165	5.170	5.499	5.592	5.628
TMEM252-DT	6.797	7.220	6.884	7.317	7.012	7.118
AP4E1	8.320	8.306	8.389	8.132	7.956	8.125
LINC00710	8.317	8.413	8.374	8.409	8.688	8.624

TSEN34	10.823	10.844	11.018	10.697	10.701	10.611
SNRNP40	9.581	9.624	9.805	9.655	9.627	9.708
FAM166A	8.522	8.510	8.688	8.612	8.752	8.637
FAM166B	7.547	7.505	7.462	7.545	7.689	7.582
FAM166C	7.735	7.794	7.897	7.564	7.623	7.628
PURB	10.219	10.441	10.467	10.404	10.448	10.141
PURA	9.534	9.203	9.432	9.556	9.352	9.776
PURG	7.136	7.293	6.799	7.407	7.397	7.425
MAN2B2	8.464	8.576	8.639	8.365	8.209	8.403
MAN2B1	9.721	9.672	9.928	9.586	9.604	9.501
CCDC28B	7.458	7.237	7.640	7.722	7.583	7.583
CCDC28A	8.786	8.752	8.798	8.493	8.441	8.373
AL009179.1	5.168	5.536	5.029	5.303	5.361	5.328
FSTL4	7.865	7.905	7.994	8.018	8.136	7.989
FSTL5	4.538	4.431	4.742	4.736	4.771	4.413
FSTL3	8.180	8.081	8.304	8.294	8.327	8.274
FSTL1	7.625	7.784	7.692	7.601	7.778	7.724
MIR200CHG	7.375	7.617	7.617	7.574	7.660	7.727
NKX6-1	6.804	6.277	6.175	6.560	6.579	6.465
NCKAP5-AS1	7.029	6.916	6.791	6.990	6.970	7.023
KCTD13	8.325	8.323	8.289	8.309	8.253	8.320
NUF2	7.779	7.839	7.747	6.929	7.024	7.227
KCTD11	8.697	8.790	8.933	8.877	8.930	8.741
ANXA8L1	8.459	8.416	8.261	8.458	8.302	8.483
C12orf49	11.576	11.653	11.753	11.444	11.271	11.470
IGHMBP2	10.151	10.251	10.281	10.083	10.235	10.281
TIMM10B	9.741	9.750	9.793	9.477	9.642	9.579
C12orf40	5.748	5.526	5.426	5.949	5.995	5.951
C12orf43	9.962	9.969	10.120	9.861	9.893	9.715
C12orf42	6.356	6.366	6.494	6.215	6.353	6.398
C12orf45	9.570	9.565	9.726	9.734	9.822	9.484
KCTD16	6.004	6.202	6.049	6.088	6.185	6.318
NFAT5	9.458	9.375	9.395	9.152	9.163	9.436
KCTD15	9.072	8.964	9.049	8.965	8.857	8.903
KCTD14	9.621	9.627	9.686	9.411	9.491	9.511
GSK3A	9.884	10.053	9.970	10.187	9.928	9.890
GSK3B	9.427	9.407	9.448	9.045	9.202	9.243
UBD	8.522	8.629	8.590	8.842	8.805	8.562
MSTO1	8.666	8.743	8.755	8.391	8.635	8.536
UBB	13.599	13.634	13.659	13.582	13.502	13.582
UBC	14.623	14.635	14.680	14.650	14.610	14.621
RHOT2	10.067	10.176	10.278	9.962	10.033	10.103
KRTAP10-4	6.914	7.474	7.267	6.793	7.186	7.235
PATE1	5.924	5.805	5.862	5.959	5.932	6.173
PATE2	6.638	6.563	6.526	6.772	6.873	6.816
CARD19	9.064	9.018	9.122	8.835	8.922	8.878
AKR1A1	10.903	11.041	11.124	10.930	10.701	10.880
AL133355.1	6.809	6.889	6.660	6.789	6.649	6.818
ZBTB6	5.866	5.927	5.906	5.710	5.703	5.968
AL928768.1	5.450	5.736	5.437	5.456	5.584	5.472
AL031665.1	7.077	7.030	7.034	6.999	7.078	7.032
RNF220	9.693	9.753	9.845	9.846	9.742	9.692
ZBTB9	9.129	9.234	9.074	8.978	9.169	9.110
RNF227	8.637	8.769	8.532	8.641	8.572	8.583
CARD10	9.668	9.741	9.867	9.458	9.571	9.473
AL513523.13	6.383	6.465	6.469	6.623	6.785	6.698

MED30	7.744	7.583	7.874	7.579	7.715	7.647
MED31	9.038	8.986	9.066	9.221	9.174	9.166
SEZ6L2	10.613	10.606	10.723	10.428	10.507	10.535
AL359649.1	6.146	6.264	6.231	6.172	5.959	6.268
MUC20-OT1	10.442	10.486	10.509	10.273	10.280	10.415
HECW1	8.083	8.353	8.121	8.125	8.373	8.321
HECW2	7.516	7.328	7.269	7.478	7.596	7.472
HRCT1	7.623	7.794	7.883	7.485	7.673	7.751
MIR6752	9.683	9.842	10.086	9.759	9.704	9.625
MIR6755	8.515	8.507	8.571	8.137	8.308	8.268
MIR6759	12.023	12.059	12.223	11.931	11.814	11.903
MIR3150BHG	8.255	8.185	8.065	8.293	8.410	8.230
FGF19	6.229	6.219	6.244	6.177	6.289	6.383
FGF18	7.175	7.287	7.253	7.252	7.199	7.144
AC109349.1	5.956	5.982	5.673	5.988	6.100	5.895
FGF13	5.860	5.886	5.838	5.784	5.788	6.058
FGF12	7.626	7.805	7.877	7.635	7.676	7.658
FGF11	8.222	8.148	8.089	8.445	8.289	8.295
FGF10	5.834	5.823	5.724	5.726	5.838	5.942
FGF17	7.271	7.387	7.549	7.523	7.520	7.523
FGF16	7.282	6.926	7.223	7.251	7.153	7.201
FGF14	6.670	6.707	6.762	6.865	6.898	6.910
AL451085.3	7.114	7.090	7.266	7.621	7.265	7.456
VCAM1	5.468	5.658	5.366	5.515	5.684	5.524
VPS51	10.829	10.832	11.072	10.464	10.405	10.534
TPSB2	7.692	7.866	7.807	8.093	8.088	7.961
OXNAD1	8.228	8.375	8.463	8.291	8.211	8.133
AC092164.1	7.430	7.450	7.147	7.346	7.369	7.512
SLC25A32	8.747	8.805	8.804	8.547	8.667	8.562
ETFBKMT	7.205	6.898	6.835	6.977	7.200	6.956
HS6ST1P1	8.402	8.374	8.583	8.382	8.351	8.484
AC084757.4	5.979	6.083	6.078	6.097	6.011	6.011
AC084757.3	5.087	5.301	5.162	5.506	5.538	5.527
KCNJ13	6.057	6.060	5.641	6.204	5.793	6.298
KCNJ12	6.496	6.635	6.546	6.555	6.588	6.782
KCNJ11	7.128	7.239	7.381	7.252	7.445	7.282
KCNJ10	5.931	6.125	5.958	6.198	6.244	6.086
TFAM	9.034	9.012	9.366	9.022	9.159	8.750
KCNJ16	4.809	5.046	4.982	5.035	5.013	4.970
KCNJ15	9.943	10.077	10.055	9.936	9.893	9.931
KCNJ14	6.887	6.865	6.900	6.635	6.965	6.970
KCNJ18	6.274	6.288	6.010	6.152	6.183	6.018
TMX4	9.234	9.201	9.527	9.159	9.433	8.969
TMX1	11.635	11.721	11.633	11.402	11.355	11.475
TMX3	9.168	9.016	8.947	8.601	8.703	8.893
TMX2	11.616	11.683	11.731	11.623	11.499	11.615
DAZAP1	10.260	10.250	10.279	9.900	9.921	10.038
DAZAP2	12.097	12.116	12.244	12.002	11.948	11.975
MLC1	6.361	6.483	6.510	6.699	6.741	6.520
AC023491.1	9.209	9.151	9.184	9.181	9.090	9.233
TLCD2	8.962	8.979	9.034	8.817	8.867	8.683
TLCD1	10.679	10.712	10.862	10.707	10.696	10.704
TLCD4	9.413	9.443	9.565	9.155	9.094	9.263
TLCD5	8.141	8.201	8.113	8.373	8.227	8.175
IGSF10	8.233	8.267	8.207	8.515	8.324	8.562
IGSF11	4.982	4.991	4.597	5.047	4.912	4.930

H3-3A	13.752	13.863	13.907	13.791	13.836	13.535
H3-3B	13.121	13.160	13.284	13.084	13.091	12.889
C6orf120	9.083	9.244	9.203	9.304	9.272	9.297
CDC42SE2	10.728	10.738	10.882	10.721	10.404	10.652
CDC42SE1	10.820	10.822	10.927	10.775	10.891	10.667
AP002360.2	6.598	6.944	7.057	6.746	6.369	6.749
RERGL	7.301	7.136	7.359	7.296	7.170	7.099
VAC14	9.848	9.653	9.873	9.940	10.072	9.838
ALMS1P1	6.483	6.476	6.328	6.429	6.420	6.591
APRT	11.234	11.381	11.599	11.368	11.401	11.279
TGFB2-OT1	10.096	10.038	10.073	9.862	9.853	10.014
C19orf53	11.217	11.364	11.476	11.354	11.268	11.244
C19orf57	8.535	8.670	8.593	8.771	8.557	8.735
C19orf54	9.270	9.312	9.368	9.153	9.132	9.168
AL136439.1	7.750	7.889	7.864	8.142	8.078	7.970
LACTB	9.090	9.057	9.140	8.997	8.942	8.888
GEMIN7	9.620	9.705	9.789	9.782	9.667	9.798
PUM2	10.532	10.601	10.589	10.381	10.379	10.544
PUM3	9.710	9.754	9.845	9.819	9.630	9.756
PUM1	11.669	11.615	11.667	11.469	11.539	11.586
RAD1	9.564	9.583	9.621	9.509	9.659	9.385
AC078842.3	6.303	6.366	6.403	6.265	6.572	6.534
MRFAP1L1	10.116	10.220	10.269	10.008	9.963	10.001
AL161452.1	8.325	8.520	8.154	8.273	8.305	8.274
EQTN	5.256	5.373	4.913	5.236	5.610	5.269
ANHX	9.245	9.091	9.051	9.332	9.411	9.332
AKTIP	10.030	10.119	10.209	10.013	9.985	10.017
LRRC74A	7.686	7.643	7.822	7.969	7.945	7.828
LRRC74B	5.470	5.317	5.448	5.529	5.492	5.452
ZC3H8	7.577	7.493	7.443	7.516	7.432	7.425
ZC3H3	9.270	9.189	9.361	9.145	9.176	9.233
COL22A1	8.497	8.646	8.430	8.290	8.459	8.675
ZC3H4	10.677	10.592	10.719	10.521	10.581	10.437
ZC3H6	7.210	7.158	7.302	7.424	7.283	7.170
AC078819.1	12.759	12.888	13.016	12.823	12.758	12.866
RADX	7.608	7.644	7.657	7.120	7.364	7.371
CYP2J2	8.811	8.917	9.036	8.788	8.715	8.875
TNFRSF10C	7.703	7.646	7.889	7.879	7.899	7.676
TNFRSF10B	11.040	11.092	11.097	10.959	10.881	10.875
TNFRSF10A	9.889	9.986	10.061	10.102	10.170	9.646
TNFRSF10D	9.131	9.098	9.114	9.276	9.180	9.375
CLCA2	5.941	5.913	5.910	6.077	5.995	5.782
CLCA1	6.765	6.915	6.836	7.337	7.181	7.111
CLCA4	4.701	4.164	4.502	4.582	4.590	4.416
AC108479.1	10.130	10.206	10.336	10.332	10.107	10.248
AL589880.1	9.133	9.460	9.661	9.690	9.788	9.324
WFDC10B	6.170	6.188	6.520	6.507	6.377	6.592
AC026362.2	5.532	5.389	5.425	5.278	5.316	5.363
AC026362.1	10.693	10.699	10.972	10.497	10.419	10.379
WFDC10A	5.951	5.905	6.044	6.207	6.187	6.000
PLEKHA3P1	8.390	8.574	8.573	8.478	8.502	8.409
AC083800.1	5.594	5.746	5.659	5.737	5.678	5.776
AC080188.2	4.508	4.532	4.347	4.370	4.380	4.439
ZNF736	5.345	5.277	5.296	5.433	5.562	5.433
UBIAD1	8.764	8.815	8.864	8.558	8.624	8.613
ZNF738	5.892	5.941	6.043	5.708	5.674	5.820

RADIL	7.736	7.984	7.892	7.789	7.673	7.736
AL109924.1	6.673	6.613	6.558	6.812	6.820	6.657
TRBV16	6.504	6.403	6.564	6.632	6.470	6.508
SLCO5A1	5.231	4.902	5.477	5.561	5.400	5.350
NHS	9.824	9.871	9.841	9.260	9.216	9.290
APOL6	9.595	9.538	9.421	9.624	9.755	9.550
APOL5	7.137	7.429	7.442	7.565	7.517	7.515
APOL4	7.153	7.226	7.126	7.279	7.231	7.357
APOL3	7.089	7.136	7.190	6.911	7.081	7.066
APOL2	9.759	9.926	10.016	10.179	10.041	9.771
APOL1	12.446	12.577	12.728	12.588	12.434	12.623
NOS1	7.835	7.796	7.970	7.763	7.822	7.752
NOS2	7.133	7.114	7.138	7.158	7.323	7.091
NOS3	6.807	7.000	6.879	6.969	7.091	6.946
AC234771.2	6.905	7.001	6.959	7.251	7.235	7.328
LCP2	7.185	7.266	7.031	7.349	7.512	7.308
LCP1	11.582	11.685	11.699	11.529	11.480	11.475
STRCP1	7.456	7.501	7.430	7.273	7.484	7.449
ZNF121	10.004	9.892	9.982	9.698	9.670	9.941
AC007787.1	6.848	7.065	7.012	7.008	7.120	7.049
RPP14	8.993	9.110	9.323	9.208	9.123	9.181
TRDV3	6.070	5.967	6.014	6.361	6.038	6.113
MIR4690	9.136	9.033	9.172	8.817	8.944	8.897
GCM1	6.029	5.728	5.773	5.936	5.779	5.722
GCM2	4.012	3.885	4.150	4.020	4.075	4.067
URGCP	9.590	9.601	9.783	9.616	9.500	9.623
SMAGP	10.569	10.683	10.715	10.610	10.563	10.471
RNU6-548P	6.631	6.606	6.425	6.481	6.853	6.738
VSIG10L	7.983	7.970	8.068	7.524	7.579	7.748
XYLT2	8.744	8.803	8.958	8.564	8.520	8.424
XYLT1	5.333	5.398	5.245	5.552	5.417	5.506
TESK1	8.653	8.645	8.976	8.814	8.746	8.707
TESK2	7.896	8.065	7.974	7.679	7.745	7.943
DPPA3P1	4.039	4.108	4.021	3.947	4.005	4.017
VWC2	3.776	3.736	3.779	3.832	3.923	3.856
AL391987.4	6.728	6.572	6.787	7.070	6.998	6.812
AL391987.2	8.253	8.221	8.282	8.628	8.425	8.403
AL391987.1	8.357	8.464	8.548	8.291	8.197	8.554
RHAG	7.332	7.438	7.562	7.558	7.717	7.898
ITPKA	6.534	6.542	6.776	6.787	6.584	6.639
AC005256.1	7.826	7.800	7.867	8.182	8.021	7.960
ITPKC	9.311	9.323	9.435	9.693	9.631	9.674
ITPKB	8.267	8.497	8.637	8.478	8.481	8.476
AC008750.5	5.841	5.819	5.565	5.932	5.827	5.677
PDE5A	9.421	9.221	9.334	9.472	9.561	9.641
PKM	14.126	14.187	14.204	14.208	14.207	14.151
ITPK1	10.878	10.888	10.979	10.888	10.782	10.701
SNHG3	5.466	5.181	5.395	5.410	5.663	5.497
SMIM2	5.727	6.028	5.937	5.855	5.999	5.862
AC073896.1	11.901	11.943	12.096	11.852	11.862	11.834
CEP170	9.502	9.428	9.452	9.106	9.205	9.357
LZTR1	9.525	9.450	9.496	9.280	9.589	9.456
COMTD1	9.342	9.192	9.532	9.290	9.198	9.328
NAPRT	11.645	11.747	11.872	11.546	11.472	11.468
AC254944.2	6.001	6.008	5.892	6.347	6.123	6.186
AC254944.3	8.279	8.162	8.137	8.002	8.055	8.233

LINC00997	7.296	7.488	7.412	7.122	7.060	7.238
LINC00996	5.203	5.224	5.051	5.189	5.355	5.133
LINC00993	5.995	6.252	5.965	6.540	6.144	6.269
LINC00992	8.973	8.973	9.221	8.689	8.792	8.659
CHAD	7.706	7.818	7.894	7.884	7.965	7.775
AL356056.1	6.030	5.933	5.836	5.839	6.147	5.887
SGO1-AS1	3.804	3.858	3.917	3.897	3.777	4.025
CHAT	7.247	7.275	7.186	7.206	7.270	7.314
MXD4	8.845	8.902	9.034	8.914	8.978	8.820
MXD3	8.973	8.719	8.908	8.768	8.786	8.764
MXD1	9.654	9.607	9.736	9.675	9.723	9.658
AL158212.3	6.771	6.607	6.793	6.902	6.490	6.846
AC007424.1	4.906	4.950	4.960	4.901	5.086	5.014
MED23	8.828	8.748	8.828	8.489	8.613	8.881
ASL	10.587	10.697	10.757	10.415	10.305	10.377
ATP6V0B	10.946	11.096	11.222	11.083	11.008	11.149
ATP6V0C	13.118	13.168	13.219	13.060	12.975	13.021
JCAD	4.777	4.842	4.807	5.126	4.817	5.142
AC093599.2	6.718	6.830	6.793	6.840	7.015	6.841
COL5A2	5.571	5.834	5.701	6.030	5.666	5.595
COL5A3	8.184	8.220	8.148	8.093	8.171	8.239
LAMP5	7.565	7.651	7.603	7.684	7.867	7.775
COL5A1	7.458	7.508	7.431	7.390	7.598	7.471
LAMP3	10.675	10.566	10.523	10.283	10.485	10.155
LAMP2	11.736	11.779	11.935	11.595	11.417	11.610
LAMP1	11.482	11.529	11.695	11.328	11.249	11.272
NAT16	7.542	7.645	7.559	7.670	7.715	7.670
NAT14	8.888	8.906	9.163	8.756	8.843	8.726
DUT	11.738	11.746	11.865	11.560	11.514	11.402
NAT10	10.212	10.241	10.267	10.218	10.052	10.156
ALDH2	12.209	12.230	12.294	12.133	12.015	11.973
UQCR10	12.541	12.655	12.764	12.585	12.566	12.580
UQCR11	11.822	11.891	12.033	11.840	11.901	11.867
RPL7AP71	7.308	7.323	7.416	7.177	7.326	7.293
RPRD1A	9.805	9.872	9.809	9.748	9.703	9.871
RPRD1B	9.950	9.977	10.029	9.802	9.712	9.807
ASAH1	12.605	12.658	12.685	12.560	12.505	12.627
ASAH2	6.963	7.110	7.180	6.986	6.897	7.166
PRSS40A	6.856	7.121	7.014	7.538	7.271	7.209
FAM227B	4.752	4.662	4.328	4.949	4.641	4.838
AC007368.1	6.969	7.074	6.893	7.134	7.229	7.308
AC007368.3	6.969	7.074	6.893	7.134	7.229	7.308
AC010096.1	5.133	5.238	5.155	5.115	5.243	5.316
AP001453.2	6.519	6.555	6.542	6.359	6.684	6.482
AP001453.3	8.221	8.268	8.083	8.409	8.300	8.267
AP001453.4	7.626	7.603	7.746	7.799	7.553	7.548
AP001453.5	9.814	9.878	10.051	9.846	9.822	9.637
CEBPA-DT	8.695	8.569	8.698	8.745	8.722	8.765
AP001372.2	7.946	7.859	8.046	7.575	7.996	7.748
MIR205HG	6.709	6.739	6.474	6.975	6.792	6.896
APOC1P1	8.667	8.545	8.805	8.806	8.698	8.691
CYP2W1	6.468	6.580	6.607	6.958	6.713	6.694
TGM7	6.177	6.117	6.182	6.169	6.231	6.326
ADM5	8.032	8.070	8.064	7.952	8.194	8.080
ADM2	8.266	8.522	8.462	8.508	8.580	8.526
AC103740.1	8.747	8.697	8.532	8.733	8.682	8.674

AC103740.2	6.822	6.866	7.016	7.327	7.183	7.215
CETP	6.388	6.467	6.483	6.839	6.742	6.652
MIPOL1	7.967	7.943	8.005	7.664	7.665	7.935
DHODH	8.024	7.998	8.037	7.918	8.051	7.857
TMEM245	11.147	11.321	11.325	10.841	10.884	11.008
NCAPH	9.206	9.202	9.243	8.835	8.921	8.855
NCAPG	8.602	8.599	8.618	7.768	8.113	7.949
UGT2B24P	8.916	8.824	8.983	8.796	8.862	8.989
CIAO1	9.944	10.067	10.198	10.084	10.176	9.850
CIAO3	8.709	8.696	8.762	8.559	8.546	8.629
MRPS11P1	4.871	4.864	4.818	4.773	4.712	4.750
URB1-AS1	7.522	7.385	7.598	7.657	7.653	7.493
NONO	13.204	13.254	13.298	13.171	13.113	13.111
GARS-DT	8.593	8.727	8.739	8.649	8.689	8.783
TRPM8	6.866	6.905	7.097	7.081	6.935	7.244
TRPM4	7.612	7.612	7.940	7.560	7.270	7.594
TRPM5	6.320	6.338	6.455	6.487	6.668	6.468
AL445471.2	8.103	8.148	8.101	8.267	8.259	8.129
TRPM7	8.219	8.356	8.259	8.139	8.190	8.289
TRPM1	5.038	4.997	4.885	4.814	4.956	4.946
TRPM2	7.666	7.434	7.638	7.817	7.806	7.729
TRPM3	8.170	8.289	7.908	8.225	8.153	8.190
SULT1C2	7.479	7.611	7.637	7.435	7.403	7.353
METRN	10.569	10.747	11.023	10.719	10.725	10.617
NINJ2-AS1	7.308	7.340	7.563	7.281	7.206	7.292
EPN1	9.728	9.752	9.929	10.152	9.976	9.948
DNAJC5G	7.801	7.884	7.493	7.905	7.963	7.827
FAN1	7.523	7.512	7.570	7.553	7.461	7.610
EPN3	8.591	8.626	8.830	8.418	8.360	8.502
PSPC1P1	9.331	9.306	9.290	8.902	8.999	8.784
EPN2	8.508	8.532	8.457	8.282	8.554	8.436
ERBIN	10.478	10.560	10.473	10.301	10.341	10.334
AL162258.1	10.315	10.474	10.681	10.373	10.415	10.418
TFDP3	6.976	6.826	6.903	7.305	7.151	7.084
TFDP2	9.429	9.496	9.440	9.121	9.110	9.130
TFDP1	9.392	9.352	9.476	9.199	9.135	9.268
GNRHR2P1	6.365	6.372	6.516	6.392	6.493	6.497
SLX1B	10.684	10.799	10.996	10.631	10.700	10.565
MIS18A	8.646	8.488	8.637	8.288	8.227	8.370
AC008870.1	8.403	8.427	8.383	8.300	8.351	8.365
BCAS4	8.347	8.144	8.413	8.255	8.362	8.236
AC008870.2	9.194	9.233	9.233	9.073	9.125	8.734
BCAS2	9.568	9.586	9.694	9.331	9.527	9.365
BCAS3	7.427	7.407	7.538	7.256	7.190	7.175
BCAS1	7.752	7.751	7.734	7.657	8.016	7.563
AL357146.1	6.996	6.894	6.631	7.016	6.773	6.727
PABPC1L	8.950	8.792	8.642	8.455	8.462	8.803
AC096921.2	5.150	5.078	4.834	5.185	5.132	5.217
CD81	12.913	13.008	13.160	12.873	12.900	12.923
CD80	8.736	8.573	8.501	8.856	8.908	8.672
CD83	8.871	8.735	8.731	8.789	8.803	8.941
CD82	9.197	9.257	9.392	9.265	9.258	9.176
CD84	8.011	7.996	7.733	8.080	8.155	8.168
CD86	6.606	6.838	6.856	6.923	6.819	6.910
SLC8A1	7.806	7.839	7.787	7.723	7.901	7.778
FGFBP3	5.237	5.111	5.085	5.176	5.156	5.098

SLC8A3	7.823	7.888	7.923	7.958	8.039	8.049
SLC8A2	8.305	8.194	7.986	8.468	8.182	8.225
AC133644.3	8.228	8.464	8.401	7.869	8.135	8.215
AC133644.2	6.950	7.062	6.792	7.113	7.176	7.139
ZBED2	6.163	6.351	6.313	6.313	6.052	6.197
ZBED3	8.707	8.722	8.911	8.638	8.585	8.564
BNIP3L	11.887	12.086	12.246	11.956	12.175	11.687
SHLD2P3	9.066	9.085	9.014	8.765	8.627	8.890
ZBED6	11.002	10.908	10.983	10.739	10.665	10.802
SHLD2P1	10.639	10.715	10.747	10.608	10.656	10.689
ZBED8	7.209	7.330	7.440	7.175	7.513	7.403
ZBED9	5.352	5.642	5.707	5.641	5.652	5.721
ZNNT1	7.622	7.709	7.603	7.691	7.563	7.660
MAIP1	10.274	10.355	10.502	10.228	10.298	10.313
CD8A	7.755	7.464	7.613	7.643	7.676	7.980
CD8B	7.631	7.749	7.814	7.930	7.803	7.722
GALNT16	7.196	7.235	7.246	7.167	7.403	7.338
GALNT17	7.103	7.175	7.049	6.894	6.812	7.041
GALNT14	10.919	10.904	11.036	10.835	10.792	10.744
GALNT15	7.198	7.301	7.289	7.273	7.248	7.344
GALNT12	10.270	10.303	10.436	9.955	9.902	9.968
GALNT13	6.893	6.905	6.857	7.151	6.864	6.998
GALNT10	10.512	10.461	10.591	10.181	10.199	10.271
GALNT11	8.624	8.552	8.683	8.352	8.426	8.295
AF131215.2	4.976	5.133	4.745	5.206	5.081	4.914
AF131215.3	6.515	6.864	6.727	6.754	6.782	6.662
AF131215.6	5.872	6.010	6.138	6.408	6.349	6.010
AF131215.7	4.426	4.392	4.330	4.619	4.585	4.578
AF131215.4	4.676	4.753	4.777	4.942	4.862	4.776
AF131215.5	5.484	5.446	5.382	5.301	5.330	5.288
AL359397.2	6.407	6.462	6.595	6.067	6.208	5.948
HARS1	9.905	10.100	10.217	9.936	9.813	9.923
HARS2	9.480	9.525	9.586	9.301	9.338	9.378
LIMK2	9.200	9.157	9.324	9.233	9.168	9.340
CCDC74B	6.822	6.805	6.932	6.500	6.847	6.879
CCDC74A	5.952	6.000	5.910	6.039	6.262	6.093
LIMK1	7.868	7.952	8.048	8.141	8.240	7.839
OR4F8P	7.745	7.729	7.361	7.729	7.847	7.903
BRI3	10.931	11.058	11.176	10.875	10.949	10.858
AC011603.1	5.335	5.294	5.278	5.338	5.188	5.550
PPL	11.194	11.277	11.229	10.889	10.801	10.933
CT476828.18	8.052	7.820	8.258	8.487	8.319	8.298
CT476828.19	8.052	7.820	8.258	8.487	8.319	8.298
CT476828.10	8.052	7.820	8.258	8.487	8.319	8.298
CT476828.11	8.052	7.820	8.258	8.487	8.319	8.298
CT476828.12	8.052	7.820	8.258	8.487	8.319	8.298
CT476828.13	8.052	7.820	8.258	8.487	8.319	8.298
CT476828.14	8.052	7.820	8.258	8.487	8.319	8.298
CT476828.15	8.052	7.820	8.258	8.487	8.319	8.298
CT476828.16	8.052	7.820	8.258	8.487	8.319	8.298
CT476828.17	8.052	7.820	8.258	8.487	8.319	8.298
AC010132.4	6.079	6.273	6.021	6.123	6.141	6.061
AC124804.1	7.388	7.300	7.400	7.403	7.413	7.468
AC069363.2	7.816	7.672	7.838	7.804	7.930	7.821
AUXG01000058.1	6.961	6.506	6.928	6.867	6.853	6.917
KLRB1	6.877	7.160	7.097	7.108	7.338	7.070

LAT2	7.193	7.398	7.375	7.259	7.425	7.578
NME1-NME2	12.984	13.055	13.204	13.055	12.870	13.071
AFG3L2	10.302	10.395	10.488	10.088	10.145	10.101
CHRM3-AS2	6.492	6.614	6.656	6.818	6.730	6.599
AC007566.1	4.585	4.934	4.944	4.798	4.891	4.700
CEMIP2	9.989	9.989	9.957	9.922	9.822	9.831
SEPTIN7P9	5.375	5.151	5.273	5.066	5.236	5.098
KIAA0825	4.555	4.692	4.607	4.654	4.641	4.567
PTBP3	10.954	10.870	10.966	10.636	10.508	10.496
PTBP2	7.306	7.193	7.291	6.965	7.245	7.054
PTBP1	11.921	12.015	12.072	11.709	11.563	11.785
SEPTIN7P2	6.078	6.138	5.727	5.963	6.187	6.027
AC002056.3	10.761	10.788	10.870	10.685	10.420	10.526
AC002056.1	10.761	10.788	10.870	10.685	10.420	10.526
CACNA2D2	7.712	7.838	7.711	7.842	7.916	7.932
CACNA2D3	5.174	5.097	5.072	5.000	5.182	5.173
PRAP1	6.882	6.877	6.928	6.889	7.013	6.971
CACNA2D4	6.927	6.875	6.670	6.873	6.967	6.860
MTA1	9.903	9.881	9.883	9.317	9.485	9.861
PTGES3L-AARSD	9.374	9.330	9.487	9.271	9.228	9.172
AL050341.2	7.561	7.546	7.683	7.585	7.582	7.697
PIK3R6	6.239	6.294	6.592	6.741	6.543	6.320
PIK3R5	8.231	8.341	8.343	8.073	8.252	8.250
PIK3R4	9.729	9.702	9.867	9.698	9.601	9.600
PIK3R3	10.071	10.131	10.188	10.005	10.006	9.995
PIK3R2	10.931	10.862	11.123	10.697	10.663	10.683
PIK3R1	9.621	9.832	9.976	9.484	9.683	9.413
PRICKLE2-AS3	6.034	5.683	5.845	6.195	5.796	5.782
PTCRA	9.466	9.438	9.607	9.493	9.524	9.570
TUBAL3	6.176	6.061	5.650	5.912	5.952	5.936
MIR483	7.019	6.820	6.730	7.017	6.871	6.950
RAB40B	9.370	9.465	9.658	9.175	9.337	9.408
AC010210.1	6.756	6.972	6.700	6.587	6.799	6.867
SLC51B	7.093	7.214	7.095	7.317	7.179	7.249
GPC3	6.767	6.806	6.828	6.707	6.774	6.602
RTN4RL1	6.219	6.431	6.490	6.565	6.319	6.457
RTN4RL2	6.226	6.319	6.146	6.014	6.261	6.170
AC067904.2	10.683	10.906	10.900	10.813	10.547	10.750
TOMM40L	8.051	7.938	8.092	7.829	8.092	7.901
RBKS	8.658	8.666	8.879	8.516	8.642	8.609
SNTN	4.026	4.118	4.503	4.061	4.223	4.323
NCAM1	8.041	7.937	7.965	8.354	8.295	8.178
NCAM2	5.521	5.346	5.524	5.415	5.475	5.434
TICAM1	7.895	7.889	7.866	8.099	7.999	8.051
TICAM2	9.261	9.374	9.195	9.258	9.302	9.297
CGN	10.528	10.555	10.643	10.411	10.587	10.187
CGA	7.168	7.235	6.804	6.856	7.118	7.042
REEP1	9.027	9.123	9.194	8.948	9.007	8.875
REEP3	9.827	9.841	9.925	9.645	9.739	9.841
REEP2	8.723	8.730	8.716	8.774	8.747	8.681
REEP5	11.764	11.882	11.961	11.863	11.813	11.826
REEP4	9.859	9.946	10.072	9.634	9.703	9.762
REEP6	9.283	9.341	9.562	9.022	9.095	9.141
CECR2	6.943	6.972	6.779	6.944	7.112	7.242
CECR3	5.933	5.913	6.012	6.085	6.136	6.108
SREK1IP1	8.490	8.484	8.678	8.706	8.779	8.803

TSKS	6.814	6.768	6.741	7.153	7.080	6.701
CECR7	9.069	8.997	8.974	8.775	8.756	8.908
ARL6IP6	9.825	9.732	9.822	9.470	9.444	9.670
ARL6IP5	11.715	11.758	11.955	11.619	11.585	11.614
ARL6IP4	10.693	10.699	10.972	10.497	10.419	10.379
ARL6IP1	11.958	12.023	12.068	11.829	11.900	11.908
FEV	8.137	7.883	7.959	8.322	8.026	8.185
FER	8.446	8.539	8.421	8.219	8.436	8.302
FES	7.085	7.222	7.250	6.911	7.217	7.138
MAPK8IP1	8.057	8.183	8.204	8.272	8.039	8.091
MAPK8IP2	9.410	9.524	9.548	9.447	9.503	9.520
AC006141.1	4.159	4.175	4.179	4.191	3.970	4.091
TMEM11	8.902	9.038	9.132	8.785	8.788	8.803
WDR11-AS1	7.893	7.860	7.745	8.156	8.061	7.953
CASC11	5.718	5.668	5.487	5.639	5.752	5.846
CASC17	5.807	5.905	5.761	6.077	6.008	6.218
CASC16	7.226	7.408	7.292	7.113	7.268	7.254
CASC15	7.527	7.445	7.694	7.902	7.691	7.577
CASC18	5.772	5.559	5.710	6.038	5.662	5.543
SLFNL1	7.699	7.560	7.508	7.710	7.784	7.659
SEC31B	8.072	8.298	8.158	8.333	8.438	8.550
SEC31A	10.720	10.774	10.784	10.669	10.737	10.623
UBOX5	8.507	8.759	8.680	8.780	8.559	8.590
FP236315.1	7.782	7.565	7.694	7.908	7.898	8.019
ANKRD6	7.450	7.392	7.507	7.146	7.213	7.191
ANKRD7	4.319	4.388	4.344	4.231	4.629	4.427
ANKRD1	7.818	7.381	7.438	7.556	7.357	7.426
ANKRD2	7.485	7.488	7.634	7.958	7.521	7.569
ANKRD9	9.267	9.410	9.633	8.983	8.953	9.054
NFYC-AS1	7.469	7.275	7.400	7.799	7.883	7.575
DISP1	6.213	6.209	6.309	6.252	6.414	6.497
DISP3	7.823	7.879	7.909	7.893	7.933	7.938
DISP2	7.823	7.559	7.786	7.703	7.939	7.750
LONP2	10.084	9.990	10.196	9.870	9.787	10.045
PDGFA	6.894	6.967	7.042	6.761	7.042	6.905
PDGFB	8.049	8.064	8.181	8.322	8.432	8.286
LONP1	10.465	10.537	10.663	10.251	10.131	10.267
PDGFD	9.820	9.832	9.904	9.488	9.361	9.482
CAPS2-AS1	6.217	6.329	6.132	6.040	6.469	6.248
CSN1S2AP	4.859	4.864	4.649	4.811	4.991	4.807
RCC1	10.161	10.404	10.449	10.332	10.141	10.093
RCC2	10.629	10.605	10.834	10.424	10.400	10.377
HIF3A	8.655	8.183	7.681	8.889	8.600	8.464
DUOXA2	7.866	7.823	8.148	8.487	8.141	8.069
FHIT	7.310	7.423	7.520	7.248	7.321	7.506
FAM83C-AS1	6.269	6.216	5.932	6.140	6.119	6.020
AC145212.1	10.047	10.053	10.201	10.055	10.078	10.039
NIFK-AS1	7.385	7.229	7.264	7.225	7.304	7.402
TRAM1L1	7.055	6.981	7.284	7.206	7.179	7.005
B2M	13.883	13.936	13.932	14.025	13.941	13.956
MRGPRG-AS1	8.619	8.714	8.512	8.541	8.622	8.693
SQLE	12.795	12.856	12.811	12.779	12.685	12.874
SPTLC2	9.691	9.677	9.775	9.596	9.678	9.586
SPTLC3	5.760	5.791	6.001	5.914	5.713	5.776
SPTLC1	10.706	10.776	10.791	10.707	10.706	10.720
ABCD1	9.128	9.210	9.276	9.170	9.149	9.238

ABCD3	10.577	10.670	10.758	10.478	10.675	10.668
AC072061.1	4.561	4.531	4.479	4.442	4.462	4.629
ABCD4	8.239	8.268	8.310	8.226	7.782	8.205
TMLHE	8.698	8.742	8.784	8.694	8.685	8.599
AC004846.2	7.157	7.367	7.096	7.081	7.438	7.415
AC004846.1	7.835	7.560	7.553	7.825	7.926	7.827
SMYD3-IT1	3.861	3.731	3.946	3.681	3.767	3.834
DGAT1	9.194	9.178	9.537	9.134	9.060	9.153
KIR2DS4	9.295	9.181	9.220	8.969	9.458	9.336
DGAT2	8.872	9.056	9.047	8.867	8.935	8.859
KIR2DS3	7.241	7.134	7.110	7.315	7.321	7.394
AC090983.1	6.993	6.910	6.993	6.997	6.963	7.096
GRAP2	7.468	7.530	7.735	7.683	7.654	7.712
SOBP	7.252	7.326	7.201	7.377	7.252	7.291
DSEL	9.074	9.102	9.043	8.794	8.609	8.947
TRAM1	12.408	12.506	12.570	12.481	12.347	12.416
GRAPL	6.483	6.581	6.479	6.705	6.587	6.509
COL21A1	4.138	4.374	4.141	4.033	4.201	4.095
CASP17P	4.843	4.695	4.539	4.615	4.671	4.868
POLH-AS1	4.845	4.639	4.818	4.629	4.869	4.802
KCNH5	4.998	5.088	5.296	5.315	4.792	5.234
KCNH4	6.928	7.008	7.175	7.278	7.131	7.247
KCNH7	6.964	7.159	6.869	7.252	6.974	7.215
KCNH6	6.916	6.950	7.081	7.219	6.990	7.036
KCNH1	6.600	6.632	6.564	6.749	6.902	6.526
KCNH3	7.878	8.048	8.068	8.017	8.087	8.232
KCNH2	7.641	7.719	7.736	7.534	7.668	7.695
KCNH8	5.093	4.864	4.990	5.014	5.131	5.132
AC245140.2	7.497	7.345	7.355	7.747	7.189	7.390
SLC28A2	6.107	6.188	6.295	6.297	6.195	6.114
ZNF296	7.869	8.015	8.117	7.762	7.999	7.807
ZNF292	9.983	10.053	10.162	10.176	10.318	9.780
GPR183	4.454	4.382	4.526	4.829	4.695	4.598
NUP133	9.075	9.202	9.223	8.824	8.863	8.840
AL445430.1	5.952	5.615	5.551	5.766	5.828	5.840
AL445430.2	4.964	4.990	5.244	5.026	5.005	4.954
NDUFAF5	7.337	7.406	7.481	7.520	7.678	7.537
NDUFAF4	9.435	9.551	9.635	9.538	9.482	9.446
NDUFAF7	8.800	8.762	8.843	8.471	8.780	8.803
NDUFAF6	8.371	8.368	8.583	8.271	8.393	8.381
NDUFAF1	8.555	8.778	8.797	8.688	8.592	8.692
NDUFAF3	10.286	10.394	10.536	10.161	10.272	10.155
NDUFAF2	8.968	8.911	9.154	9.085	9.060	9.075
OR2H2	6.564	6.757	6.389	6.617	6.660	6.646
AL133216.2	10.722	10.564	10.478	10.430	10.307	10.610
NDUFAF8	9.676	9.733	9.986	9.731	9.531	9.607
SILC1	6.865	6.966	6.994	6.874	7.010	6.912
ADIRF-AS1	7.787	7.724	7.650	7.940	7.827	7.728
SZT2	8.636	8.666	8.589	8.702	8.648	8.601
PIK3CD-AS2	6.160	6.188	6.218	6.000	6.226	6.094
PIK3CD-AS1	9.665	9.470	9.690	9.722	9.643	9.853
AC092819.1	6.139	6.039	5.840	6.009	6.194	6.313
AC144530.1	12.948	12.969	13.096	13.029	12.922	13.042
PRKAR1A	12.091	12.185	12.230	12.135	12.143	12.063
PRKAR1B	8.449	8.455	8.531	8.541	8.568	8.620
SAR1A	9.668	9.727	9.779	9.842	9.796	9.988

SAR1B	9.407	9.564	9.491	9.618	9.811	9.750
AC007876.1	11.055	11.139	11.245	11.251	11.277	11.318
TTTY2	7.674	7.675	7.508	7.641	7.861	7.792
NIPSNAP2	11.552	11.580	11.708	11.495	11.448	11.648
NIPSNAP1	11.318	11.259	11.468	11.220	11.221	11.142
AC016705.2	4.094	4.070	3.884	4.212	4.272	4.117
TTTY6	7.055	7.071	6.914	6.968	6.991	6.898
TTTY7	7.319	7.372	7.287	7.273	7.310	7.411
TTTY4	5.475	5.250	5.301	5.500	5.602	5.402
TTTY5	7.089	7.248	7.042	6.980	7.277	7.269
TTTY8	6.686	6.560	6.735	6.884	6.688	6.702
DCAKD	9.192	9.167	9.393	8.946	8.822	9.032
CHAF1A	9.349	9.045	9.171	9.051	9.257	8.738
CHAF1B	7.944	8.064	8.265	7.931	7.876	7.887
BIN1	10.019	10.191	10.170	10.014	10.000	10.098
BIN3	9.181	9.098	9.250	8.883	8.956	8.977
BIN2	7.265	7.481	7.242	7.639	7.501	7.467
FAM133CP	9.535	9.496	9.496	9.402	9.565	9.304
TIMM50	10.248	10.317	10.408	10.091	9.912	10.077
ZSCAN12P1	7.845	7.988	7.917	7.974	8.055	7.967
MIR3152	4.962	4.968	4.959	4.876	5.237	5.063
IQCJ-SCHIP1-AS	7.570	7.694	7.497	7.663	7.819	7.768
IGDCC3	8.477	8.539	8.539	8.373	8.665	8.557
HLA-DMB	10.003	10.013	10.176	10.021	10.073	10.057
STAM2	9.309	9.465	9.215	9.138	9.325	9.319
HLA-DMA	11.051	11.032	11.231	10.892	11.073	10.916
SYTL5	8.795	8.671	8.717	8.482	8.530	8.502
SYTL4	7.243	7.113	7.349	6.804	6.772	6.937
SYTL1	8.948	8.947	9.121	8.603	8.819	8.873
SYTL3	8.794	8.795	8.765	8.770	8.632	8.870
SYTL2	8.709	8.738	8.660	8.904	9.131	8.622
FAM182B	7.407	7.450	7.492	7.870	7.679	7.603
FAM182A	7.407	7.450	7.492	7.870	7.679	7.603
PLEKHH3	9.021	9.081	9.238	8.882	8.941	8.959
PLEKHH2	7.647	7.497	7.709	7.583	7.625	7.526
PLEKHH1	7.297	7.339	7.599	7.087	7.420	7.084
TMEM131L	7.616	7.542	7.690	7.547	7.350	7.515
KCNS3	6.496	6.368	6.531	6.587	6.558	6.457
ZCCHC3	9.073	9.129	9.281	9.116	9.160	8.962
DMRTC1	8.957	9.043	8.933	8.974	9.004	9.041
DMRTC2	4.535	4.612	4.542	4.682	4.638	4.705
ZCCHC2	9.744	9.552	9.747	9.604	9.879	9.448
ORM1	6.247	6.419	6.483	6.758	6.514	6.628
AC027307.3	5.534	5.589	5.585	5.665	5.816	5.871
AC027307.2	7.428	7.482	7.587	7.621	7.586	7.429
PXN-AS1	7.579	7.524	7.534	7.436	7.286	7.553
MTRR	8.120	8.068	8.182	7.976	7.862	8.118
MAVS	10.394	10.368	10.430	10.332	10.312	10.396
TEX19	6.138	6.347	6.172	6.261	6.017	6.265
FOXP2	7.685	7.552	7.607	7.842	7.685	7.668
FOXP3	7.457	7.521	7.390	7.672	7.705	7.631
AC100821.2	4.606	4.472	4.535	4.593	4.570	4.737
FOXP1	10.812	10.831	10.875	10.701	10.598	10.522
IL10RA	6.231	6.398	6.348	6.478	6.303	6.310
IL10RB	9.519	9.363	9.317	9.089	9.474	9.604
MSL3	9.321	9.241	9.445	8.980	9.201	9.197

AC022034.1	6.375	6.034	6.369	6.453	6.098	6.386
AC106707.1	3.828	3.888	4.117	3.991	4.097	4.022
MYCN	7.149	7.422	7.438	7.467	7.346	7.417
MYCL	7.240	7.315	7.491	7.335	7.421	7.442
PARPBP	8.019	7.991	8.121	7.960	7.994	7.969
TKT	12.194	12.178	12.445	12.156	12.040	12.236
TMEM45A	6.738	6.844	6.897	6.232	6.594	6.482
TMEM45B	8.863	8.786	8.934	8.702	8.894	8.862
SLC9A3-AS1	7.379	7.548	7.491	7.328	7.204	7.545
NPHP3-ACAD11	8.575	8.579	8.525	8.311	8.263	8.463
ZNF962P	4.338	4.409	4.292	4.455	4.467	4.436
TK2	9.472	9.507	9.542	9.012	9.118	9.189
TK1	10.735	10.781	10.978	10.296	10.475	10.317
OR2M4	5.572	5.747	5.718	5.801	5.946	5.985
BVES-AS1	5.083	4.961	5.225	5.211	5.318	5.196
MSLN	4.930	4.730	4.924	4.830	4.803	4.887
MFNG	8.053	7.893	7.928	8.117	8.050	8.040
MALAT1	13.287	12.735	12.628	12.932	13.268	12.707
LRRC3B	5.998	6.030	5.899	6.082	6.232	6.057
AC008760.2	5.970	5.860	5.497	6.026	6.085	5.852
ECD	8.132	8.081	8.283	8.261	7.972	8.147
LINC02003	6.460	6.687	6.353	6.272	6.616	6.434
LINC02002	3.636	3.714	3.828	3.877	4.052	3.881
AMDHD1	5.311	5.235	5.184	5.369	5.210	5.319
THTPA	9.808	9.891	9.831	9.798	10.010	9.864
LINC02008	6.380	6.507	6.209	6.300	6.349	6.299
CIB2	9.470	9.527	9.638	9.366	9.425	9.440
NDFIP2	9.338	9.509	9.498	9.428	9.541	9.612
NDFIP1	11.353	11.503	11.544	11.410	11.377	11.491
CIB1	11.197	11.291	11.412	11.231	11.149	11.162
PRIMA1	7.239	7.270	7.317	7.358	7.362	7.382
CIB4	5.839	6.219	6.070	6.386	6.307	6.302
ARID3A	6.766	7.065	6.825	7.161	6.886	6.652
FGF13-AS1	5.967	5.810	5.417	5.901	5.823	6.022
RPS6KL1	7.547	7.592	7.763	7.734	7.701	7.633
CELSR3	8.945	9.008	8.974	8.939	8.858	8.990
CELSR2	7.992	8.131	8.023	8.015	8.181	8.258
CELSR1	9.149	9.259	9.282	9.090	8.946	9.180
FAM185BP	5.908	5.813	5.791	5.468	5.770	5.642
LRRC38	4.388	4.458	4.420	4.425	4.326	4.373
LRRC39	4.955	4.731	4.528	4.960	5.050	4.930
LRRC31	7.080	6.994	6.923	6.719	6.719	6.713
LRRC32	7.576	7.680	7.592	7.619	7.810	7.679
LRRC34	4.194	4.368	4.326	4.300	4.424	4.395
LY6G5C	7.304	7.255	7.519	7.059	7.302	7.368
LY6G5B	6.426	6.358	6.313	6.126	6.332	6.345
MFN2	10.324	10.431	10.486	10.154	10.102	10.077
MFN1	9.896	9.948	9.985	9.971	9.897	9.782
FAM117A	9.223	9.270	9.353	9.148	9.091	9.091
FAM117B	7.326	7.354	7.408	7.062	7.161	7.075
SVIL	10.201	10.212	10.262	10.175	10.005	10.281
AL356489.2	6.956	6.937	6.783	7.295	7.193	7.117
AC016910.2	3.996	4.130	4.093	3.861	4.080	4.117
CLCNKA	7.227	7.074	7.227	7.554	7.508	7.568
CLCNKB	8.582	8.839	8.684	8.886	8.707	8.853
NBPF13P	7.467	7.590	7.618	7.576	7.832	7.792

CCDC169	7.019	6.738	6.719	6.786	7.045	6.881
CCDC168	5.376	5.512	5.369	5.395	5.493	5.500
CCDC163	8.131	8.030	8.100	7.992	8.278	8.194
CCDC160	5.588	5.310	5.739	5.501	5.555	5.389
CCDC167	8.624	8.590	8.652	8.809	8.685	8.775
AC239868.1	7.576	7.715	7.524	7.580	7.455	7.563
AC090398.1	6.565	6.761	6.722	6.769	6.767	6.764
SNORA21B	7.493	7.437	7.428	7.214	7.638	7.054
TJAP1	9.053	9.070	9.175	8.905	9.025	9.028
MAGEA12	5.387	5.456	5.500	5.473	5.583	5.483
MAGEA11	5.126	5.189	5.237	5.325	5.258	5.258
MAGEA10	6.807	7.012	6.836	7.055	6.996	6.932
LINC01069	7.413	7.328	7.094	7.266	7.465	7.328
RPS7P10	13.359	13.392	13.529	13.540	13.451	13.548
RPS7P11	13.359	13.392	13.529	13.540	13.451	13.548
BOLA3-AS1	6.371	6.425	6.364	6.656	6.399	6.571
CMKLR1	7.450	7.571	7.391	7.486	7.582	7.507
SH3RF3-AS1	4.998	4.927	5.057	5.137	5.004	4.982
AC013644.1	7.436	7.504	7.315	7.588	7.682	7.599
AL355613.1	5.781	5.695	5.991	5.947	6.099	5.979
UQCRH	12.550	12.633	12.742	12.675	12.548	12.679
UQCRB	11.067	11.218	11.272	11.243	11.350	11.247
AC112204.2	6.232	5.902	5.907	6.273	6.253	6.413
ATP6V0D2	9.113	9.040	8.995	8.852	9.077	9.246
ATP6V0D1	11.332	11.443	11.523	11.218	11.158	11.184
FCAR	6.720	6.830	6.734	6.676	7.104	6.857
FLCN	7.756	7.929	7.967	8.021	7.884	7.811
CCNYL2	5.455	5.391	5.350	5.564	5.438	5.527
AL033381.2	5.876	6.206	5.999	6.166	6.157	6.182
AL033381.1	6.781	6.739	6.656	6.787	6.608	6.805
MIR622	6.808	6.874	6.977	6.884	6.796	6.956
AP000553.1	6.841	6.927	6.872	7.097	6.773	7.126
RP9P	9.809	9.778	9.816	9.830	9.887	9.854
LINC00339	6.909	7.060	7.184	6.796	6.975	7.155
LINC00337	5.886	5.791	5.755	5.942	5.902	5.846
LINC00334	7.105	7.101	7.100	7.079	7.196	7.112
LINC00330	7.482	7.746	7.466	7.707	7.622	7.653
C3orf67	5.067	5.080	4.900	5.245	4.872	5.131
C3orf62	6.056	6.244	6.223	6.267	6.097	5.977
AC097381.1	8.348	8.274	8.413	8.509	8.383	8.335
RNF149	10.617	10.736	10.812	10.580	10.491	10.520
LINC01145	8.038	7.879	8.165	8.613	8.288	8.066
MIR4300HG	7.432	7.425	7.461	7.426	7.462	7.513
C4BPA	7.930	7.971	7.843	7.753	7.794	7.749
C4BPB	8.163	8.244	8.352	8.311	8.407	8.339
AC074194.2	7.200	6.986	7.066	7.232	7.342	7.340
PTPN9	10.181	10.183	10.250	10.060	10.111	10.083
PTPN3	10.076	10.115	10.063	9.968	9.957	9.988
PTPN2	9.755	9.715	9.909	9.704	9.746	9.695
PTPN1	9.592	9.813	10.043	9.866	9.688	9.354
PTPN7	7.810	7.836	7.887	8.281	8.167	8.003
PTPN6	8.639	8.582	8.886	8.574	8.671	8.593
PTPN5	6.409	6.322	6.407	6.954	6.304	6.302
PTPN4	9.249	9.190	9.237	9.099	9.378	9.110
SUMO2P17	12.617	12.628	12.692	12.617	12.655	12.614
BTF3	13.513	13.575	13.615	13.607	13.525	13.575

SMIM10L1	9.930	9.872	10.116	9.760	9.699	9.796
WASH2P	9.341	9.202	9.345	9.278	9.301	9.395
NNMT	10.121	10.149	10.270	10.163	10.061	10.237
AGBL5	8.412	8.535	8.575	8.052	8.159	8.174
AGBL4	6.122	6.300	6.218	6.470	6.334	6.335
AGBL3	3.939	4.003	3.873	3.970	4.060	4.163
AGBL2	5.849	5.731	5.851	5.748	5.386	5.804
AGBL1	6.707	6.656	6.629	6.767	6.905	6.852
EVI5L	8.706	8.634	8.914	8.949	8.777	8.664
RBM12B	8.018	7.845	7.940	7.451	7.718	7.588
ARRB2	8.624	8.665	8.854	8.527	8.696	8.616
ARRB1	9.133	9.329	9.192	9.140	9.311	9.281
MIR24-1	9.457	9.279	9.462	9.378	9.356	9.444
GPNMB	10.418	10.461	10.516	10.320	9.905	10.233
PSMA2	12.535	12.649	12.727	12.589	12.551	12.615
PSMA3	11.619	11.719	11.833	11.783	11.708	11.689
PSMA1	11.955	11.935	11.995	12.035	12.000	12.010
SLC7A6OS	8.969	8.982	9.004	8.870	8.996	8.933
PSMA7	12.741	12.695	12.884	12.761	12.630	12.739
PSMA4	12.483	12.467	12.547	12.529	12.557	12.382
PSMA5	10.326	10.270	10.439	10.217	10.346	10.295
SLC22A18AS	9.635	9.757	9.797	9.709	9.707	9.704
PSMA8	4.328	4.229	4.337	4.281	4.364	4.319
SYNRG	10.457	10.547	10.559	10.252	10.346	10.306
MSH3	8.283	8.346	8.371	8.003	8.218	8.049
AC093627.30	7.823	7.854	7.896	7.871	7.945	7.743
MGLL	11.815	12.207	12.335	12.062	12.094	11.892
OBSCN-AS1	5.837	6.005	5.859	6.075	5.963	5.877
AC069277.1	6.961	7.014	6.905	6.925	6.993	7.079
GUCD1	11.120	11.216	11.385	11.044	10.962	10.906
ENKD1	8.613	8.548	8.761	8.363	8.236	8.399
C9orf131	8.083	7.886	8.109	8.077	8.046	8.101
AGPAT5	10.366	10.577	10.696	10.321	10.220	10.249
AGPAT4	7.927	7.986	7.963	7.905	8.045	8.005
C9orf135	6.959	6.731	7.131	6.902	7.056	6.704
AGPAT2	9.945	10.056	10.223	9.953	10.077	9.976
AGPAT1	11.189	11.317	11.391	11.231	11.239	11.204
FOXO3	11.153	11.057	11.157	10.900	10.949	10.972
TMEM155	5.323	5.322	5.419	5.544	5.633	5.420
FOXO1	8.880	9.154	9.428	9.179	9.418	8.737
FOXO6	7.560	7.573	7.270	7.433	7.460	7.515
FOXO4	7.620	7.660	7.847	7.952	7.932	7.660
TMEM158	7.266	7.503	7.473	7.247	7.309	7.318
ANGEL2	8.794	8.873	8.811	8.450	8.619	8.667
ANGEL1	9.385	9.453	9.495	9.245	9.404	9.347
AC079331.1	6.662	6.680	6.795	6.475	6.642	6.673
OSTF1	8.831	8.797	8.950	8.798	8.790	8.795
C11orf21	6.931	6.980	7.160	6.842	7.037	7.052
CSNK1G2-AS1	7.971	7.834	8.046	7.941	7.860	8.007
C11orf24	9.334	9.375	9.460	9.458	9.490	9.458
ATP6V0E2-AS1	6.285	6.134	5.979	6.521	6.249	6.120
CCN4	7.352	7.351	6.993	7.480	7.425	7.613
CCN5	7.270	7.154	7.207	7.190	7.221	7.140
CCN6	6.721	6.835	6.634	6.984	6.958	6.811
CCN1	12.296	12.425	12.501	12.416	12.347	12.407
CCN2	12.512	12.604	12.792	12.513	12.460	12.514

CCN3	9.022	8.973	9.128	9.048	9.108	9.179
KLHL1	6.865	7.169	6.973	7.220	7.121	7.288
METTL2A	9.415	9.630	9.642	9.576	9.762	9.595
AC244213.1	7.342	7.460	7.438	7.331	7.346	7.164
ALLC	6.954	6.658	6.997	7.127	7.044	7.031
SLC34A1	6.841	6.854	6.963	7.108	7.116	6.956
SLC34A3	10.950	10.786	10.890	10.985	11.011	11.111
SLC34A2	7.546	7.420	7.453	7.355	7.392	7.337
AL031658.2	5.677	5.779	5.971	5.979	6.014	5.897
AL031658.1	9.132	9.202	8.773	9.193	9.344	9.140
EIF3CL	13.532	13.568	13.589	13.385	13.205	13.299
UBR3	8.458	8.493	8.439	8.132	8.079	8.369
UBR2	8.531	8.609	8.549	8.391	8.367	8.641
UBR1	8.931	8.904	8.991	8.724	8.877	8.861
UBR7	9.634	9.540	9.698	9.310	9.366	9.226
UBR5	8.972	9.063	8.971	8.926	8.565	8.939
UBR4	10.399	10.397	10.335	10.152	10.050	10.221
RNU1-39P	7.242	7.087	7.114	7.151	7.114	7.214
SLC44A3-AS1	7.251	7.277	7.242	7.307	7.235	7.246
CCNY	9.872	9.800	9.977	9.822	9.742	9.711
CCNF	8.968	9.052	9.298	8.852	8.824	8.898
CCNC	10.955	11.019	11.126	11.108	10.998	11.060
AL359762.3	12.530	12.641	12.776	12.587	12.551	12.483
CCNO	8.086	8.190	8.247	8.256	8.239	8.148
CCNH	10.031	10.099	10.151	9.894	9.897	9.971
CCNI	12.364	12.353	12.505	12.244	12.431	12.364
CCNJ	6.270	6.055	6.294	6.262	6.223	6.135
CCNK	10.602	10.679	10.785	10.582	10.536	10.542
AC126755.1	7.215	7.288	7.487	7.403	7.143	7.216
AC126755.7	11.306	11.174	11.094	11.070	11.186	11.250
AC126755.4	7.182	7.381	7.328	7.421	7.457	7.500
NCKAP5	4.872	4.845	4.676	4.688	4.817	4.899
NCKAP1	11.437	11.462	11.381	11.202	11.090	11.443
JMY	8.898	8.837	8.884	8.373	8.356	8.669
AC006511.4	7.158	7.438	7.162	7.256	7.467	7.137
AC006511.7	6.360	6.376	6.591	6.594	6.723	6.582
AC006511.6	6.030	6.122	6.269	6.311	6.370	6.339
INVS	7.372	7.555	7.557	7.357	7.589	7.663
RPL9P7	13.154	13.225	13.361	13.496	13.391	13.505
EXOSC10	8.415	8.274	8.447	7.852	7.665	7.903
CDC25C	8.157	7.899	8.025	7.582	7.770	7.558
CDC25B	10.344	10.360	10.482	10.041	10.065	10.090
CDC25A	8.681	8.794	8.771	8.609	8.522	8.390
UAP1	9.337	9.400	9.441	9.066	9.293	9.477
TMPRSS12	5.039	5.027	4.855	5.029	5.109	5.094
TMPRSS13	8.121	8.099	8.117	8.587	8.390	8.165
ZNF821	7.999	7.919	8.068	7.888	7.980	8.014
ZNF827	8.859	8.972	8.993	8.650	8.652	8.619
AC098588.3	5.659	5.378	5.528	6.080	5.485	5.777
ZNF829	5.401	5.437	5.180	5.541	5.429	5.445
LRIG3	9.890	9.863	9.932	9.669	9.663	9.855
LRIG2	8.096	8.241	8.234	8.068	8.189	8.110
LRIG1	7.597	7.756	7.859	7.557	7.604	7.578
LINC00029	6.888	7.117	6.892	6.914	7.153	7.116
AC073389.1	6.633	6.480	6.380	6.638	6.648	6.282
AC062015.1	7.629	7.593	7.580	7.466	7.751	7.641

A1CF	7.331	7.175	7.280	7.570	7.541	7.443
ITPR1-DT	4.235	4.548	4.587	4.367	4.245	4.398
ACAN	7.851	8.207	8.087	8.126	8.213	8.315
AC074212.1	7.339	7.453	7.251	7.631	7.363	7.587
POT1	9.219	9.325	9.274	9.126	9.161	8.984
AC118555.1	6.918	6.876	6.854	6.838	6.793	7.042
CD302	7.727	7.561	7.813	7.285	7.297	7.249
CLHC1	6.265	6.230	6.204	6.104	6.397	6.266
MAMDC4	6.615	6.649	6.497	6.662	6.550	6.717
TMPO-AS1	7.505	7.613	7.723	7.201	7.164	7.238
LYZL4	7.991	7.778	7.997	7.809	7.871	8.006
CLPTM1L	11.021	11.182	11.176	11.071	10.865	10.935
LYZL1	6.015	5.961	5.906	5.930	5.905	5.921
LYZL2	5.573	5.961	5.906	5.842	5.768	5.819
PIAS2	8.287	8.250	8.513	8.203	8.297	8.422
PIAS3	8.300	8.241	8.525	8.216	8.174	8.207
PIAS1	10.062	10.142	10.206	10.047	10.040	9.805
PIAS4	8.305	8.356	8.476	8.167	8.272	8.377
ZNF311	5.833	5.802	5.891	5.805	5.910	5.821
TEX38	6.671	6.529	6.403	6.722	6.772	6.983
ZNF316	8.471	8.617	8.658	8.823	8.788	8.604
ZNF317	9.262	9.303	9.478	9.245	9.207	9.472
TEX33	4.150	4.228	4.333	4.378	4.405	4.100
ZNF319	7.686	7.866	7.963	7.586	7.748	7.736
PET117	8.025	7.866	8.116	7.857	7.933	7.897
PIFO	6.924	7.034	7.133	7.097	7.117	7.287
TEX36	6.054	5.881	6.161	5.919	6.106	6.056
TEX35	7.158	7.111	7.367	7.786	7.474	7.484
AC024075.2	8.685	8.626	8.605	8.281	8.484	8.512
SAE1	11.590	11.581	11.739	11.395	11.235	11.310
CXCL10	8.104	8.156	8.118	8.224	8.592	8.194
AL034430.1	9.588	9.618	9.833	9.436	9.343	9.436
AC023794.2	6.483	6.801	6.796	6.761	6.821	6.788
AL034430.2	9.588	9.618	9.833	9.436	9.343	9.436
AC023794.5	6.413	6.550	6.764	6.116	6.277	6.365
AC023794.4	6.036	6.161	6.015	6.233	6.245	6.182
OTOGL	5.411	5.718	5.319	5.156	5.534	5.433
AP4M1	7.865	7.883	8.144	7.899	7.994	8.049
MYLK3	7.180	7.295	6.765	7.331	7.309	7.090
SLCO1C1	5.547	5.357	5.429	5.552	5.539	5.628
RUNDC3A-AS1	4.447	4.314	4.373	4.405	4.525	4.495
PIF1	7.480	7.712	7.554	7.354	7.525	7.436
SDHA	10.438	10.447	10.431	10.273	10.280	10.415
SDHC	10.880	10.910	11.007	11.033	10.980	10.967
SDHB	9.481	9.563	9.647	9.166	9.177	9.184
SDHD	10.663	10.722	10.743	10.605	10.539	10.637
PSG11	7.297	7.409	7.218	7.314	7.507	7.422
AC009951.4	5.602	5.630	5.664	5.720	5.611	5.767
MSC-AS1	4.081	4.051	4.009	4.078	3.975	3.961
TNXA	8.901	8.893	9.136	9.158	9.166	9.008
DLG1	10.801	10.840	10.741	10.686	10.680	10.778
DLG3	9.965	9.881	9.953	9.650	9.663	9.683
DLG2	5.727	5.742	5.639	5.935	5.884	5.807
DLG5	8.874	8.848	8.950	8.761	8.562	8.680
DLG4	7.800	7.676	7.908	8.263	7.979	7.863
LINC01267	6.746	6.723	6.543	6.728	6.605	6.782

SMG9	9.563	9.670	9.624	9.272	9.517	9.482
SMG8	8.894	9.028	9.078	8.864	8.898	8.911
LINC01260	7.824	7.864	7.781	7.920	7.930	7.931
FAM114A1	9.186	9.165	9.268	9.170	9.152	9.253
GRWD1	9.340	9.351	9.446	9.348	9.283	9.402
FAM114A2	8.065	8.012	8.126	8.040	8.022	8.058
AC027682.1	5.268	5.459	5.170	5.492	5.321	5.401
AC027682.6	7.204	7.212	7.348	7.302	7.343	7.455
AC027682.4	7.404	7.316	7.476	7.424	7.571	7.383
IFNK	4.079	4.123	4.028	4.105	4.165	4.109
LHB	7.835	7.928	7.992	8.175	7.978	7.896
IFNG	5.796	5.905	5.879	5.912	5.794	5.762
IFNE	6.984	6.848	6.495	6.558	6.347	6.847
GMIP	8.690	8.773	8.871	8.402	8.639	8.813
SEC22B4P	8.664	8.834	8.790	8.348	8.536	8.740
R3HCC1L	8.535	8.491	8.567	8.626	8.298	8.442
ADRA1A	6.101	6.254	6.476	6.509	6.708	6.636
ADRA1B	5.408	5.683	5.783	5.720	5.798	5.661
ADRA1D	6.745	6.843	6.854	7.226	6.951	7.022
HSFY1	6.732	6.550	6.046	6.684	6.745	6.551
HSFY2	6.732	6.550	6.046	6.684	6.745	6.551
AL138756.1	8.383	8.593	8.409	7.989	8.120	8.073
SAP25	8.636	8.568	8.560	8.917	8.751	8.750
AC008556.1	7.539	7.541	7.537	7.541	7.577	7.618
GABRG3	5.663	5.808	5.622	5.591	5.667	5.770
GABRG2	5.486	5.554	5.540	5.609	5.797	5.738
GABRG1	5.107	4.989	5.195	5.184	5.241	5.124
AC011448.1	11.039	11.066	11.299	11.049	10.948	11.107
C1orf21	8.817	8.929	9.099	8.799	8.767	8.732
LINC01883	5.831	5.756	5.908	6.072	5.845	6.156
LINC01887	7.752	7.751	7.609	7.793	7.658	7.757
OR7E37P	9.849	9.926	10.029	9.810	9.880	9.911
AC027097.1	6.807	6.817	6.655	6.755	6.731	6.790
AHSA1	11.496	11.590	11.697	11.683	11.605	11.625
METTL27	8.129	8.368	8.459	8.135	8.231	8.092
AC022486.1	4.949	4.987	4.724	5.252	5.196	4.919
GLMP	9.707	9.902	10.033	9.623	9.673	9.661
TOMM7	12.566	12.666	12.876	12.588	12.568	12.693
TOMM5	9.842	9.874	9.936	9.962	9.948	9.874
GLMN	8.406	8.445	8.457	8.541	8.270	8.467
GSTO2	9.566	9.673	9.726	9.377	9.410	9.483
GSTO1	10.358	10.376	10.663	10.390	10.440	10.407
TTC33	6.263	6.346	6.388	6.125	6.385	6.608
TTC32	8.792	8.692	8.903	8.271	8.463	8.316
TTC31	9.397	9.402	9.419	9.037	9.014	9.089
TTC37	9.703	9.736	9.901	9.440	9.463	9.329
HS3ST3B1	7.128	7.092	7.115	7.220	7.243	7.158
U85056.1	8.052	7.820	8.258	8.487	8.319	8.298
TTC34	6.005	6.162	6.018	6.102	6.268	6.276
TTC38	9.304	9.332	9.369	9.417	9.294	9.274
AC133548.2	8.531	8.494	8.447	8.455	8.613	8.488
HMGB2	10.333	10.270	10.415	9.565	9.879	9.670
HMGB1	13.218	13.387	13.396	13.059	12.997	12.987
FRMPD4	6.945	6.836	6.920	7.092	7.062	6.956
FRMPD1	6.189	6.300	6.153	6.234	6.213	6.060
FRMPD3	7.486	7.561	7.569	7.715	7.542	7.501

FRMPD2	6.054	5.909	5.753	6.026	6.098	6.326
FYCO1	9.250	9.330	9.310	9.254	9.417	9.362
PDE10A	7.800	7.859	7.817	7.772	7.881	7.964
STAG3L5P-PVRI(	8.587	8.366	8.369	8.426	8.358	8.640
AC021483.2	4.897	4.878	4.819	4.823	4.959	4.822
GEMIN8	7.732	7.925	8.259	7.875	7.963	7.886
GEMIN2	8.407	8.350	8.490	8.447	8.363	8.305
GTF2H2C	8.310	8.418	8.490	8.307	8.382	8.244
ENHO	7.473	7.440	7.544	7.606	7.697	7.606
GEMIN6	9.708	9.797	9.915	9.856	9.813	9.848
RAB2B	9.780	9.754	9.942	9.637	9.518	9.633
GEMIN4	9.682	9.763	9.843	9.652	9.695	9.716
GEMIN5	8.403	8.457	8.474	8.087	8.253	8.143
BPIFC	6.961	6.989	6.797	6.868	6.970	7.153
KRBA1	9.125	9.250	9.083	9.295	9.250	9.160
KIF9-AS1	8.298	8.368	8.278	8.381	8.630	8.401
KRBA2	7.297	7.223	7.161	7.277	7.261	7.615
LARP1B	7.030	7.002	7.288	6.962	7.071	7.033
MCRS1	9.329	9.305	9.386	9.226	9.200	9.261
MLKL	9.444	9.427	9.452	9.343	9.392	9.192
SNORD3D	7.100	7.324	7.207	7.239	7.288	7.394
SNORD3C	7.100	7.324	7.207	7.239	7.288	7.394
SNORD3A	7.100	7.324	7.207	7.239	7.288	7.394
STX17-AS1	11.145	11.111	11.086	11.091	11.248	11.235
PAN3-AS1	4.699	4.892	5.095	5.188	4.799	4.861
KIF18A	8.130	8.229	8.492	7.803	8.054	8.078
KIF18B	9.461	9.442	9.579	9.038	9.174	9.168
ZSCAN16-AS1	7.273	7.245	7.248	7.245	7.412	7.329
AC004080.16	9.538	9.342	9.084	9.794	9.853	9.785
AC004080.14	5.675	5.419	5.119	5.395	5.476	5.451
AC004080.13	6.399	6.341	6.348	6.633	6.531	6.496
AC004080.10	9.538	9.342	9.084	9.794	9.853	9.785
AC004080.11	4.592	4.351	4.364	4.544	4.436	4.468
SNORD34	14.424	14.395	14.422	14.370	14.350	14.418
SNORD33	14.424	14.395	14.422	14.370	14.350	14.418
TGM1	7.004	6.994	7.163	7.337	7.217	7.225
TGM2	12.394	12.449	12.504	12.461	12.422	12.548
TGM3	6.602	6.422	6.141	6.639	6.799	6.632
TGM4	6.556	6.638	6.704	6.581	6.690	6.399
TGM5	8.001	7.977	7.947	7.879	7.884	8.021
TGM6	7.825	7.842	8.104	8.121	7.818	7.943
SMG5	9.338	9.354	9.459	9.403	9.352	9.254
AL358781.1	7.606	7.682	7.572	7.530	7.734	7.691
AL358781.2	7.848	8.171	8.136	7.928	8.066	8.218
EIF3H	11.796	11.844	12.040	11.840	11.611	11.787
EIF3I	11.720	11.755	11.834	11.661	11.734	11.704
EIF3J	9.957	9.889	10.012	9.851	9.759	9.861
EIF3K	12.335	12.440	12.563	12.469	12.346	12.391
EIF3L	12.863	12.870	12.930	12.590	12.383	12.736
EIF3M	12.053	12.096	12.253	12.008	11.939	11.920
SLX1A	10.684	10.799	10.996	10.631	10.700	10.565
EIF3A	10.959	10.938	10.964	10.770	10.801	10.799
EIF3B	12.163	12.182	12.295	12.054	11.899	12.068
EIF3C	13.532	13.568	13.589	13.385	13.205	13.299
EIF3D	12.396	12.468	12.585	12.459	12.347	12.337
EIF3E	11.954	12.022	12.173	12.045	11.928	11.962

EIF3F	12.521	12.615	12.767	12.462	12.387	12.432
EIF3G	10.931	11.049	11.204	10.884	10.783	10.848
MKKS	9.588	9.618	9.833	9.436	9.343	9.464
NOL11	9.824	9.981	10.022	9.836	9.712	9.817
NOL10	10.119	10.197	10.225	10.132	10.104	10.103
NOL12	8.402	8.430	8.469	8.237	8.355	8.165
BARD1	9.629	9.656	9.699	9.308	9.465	9.452
AC073263.1	7.441	7.205	7.324	7.423	7.463	7.473
UQCRFS1P1	12.728	12.710	12.792	12.777	12.608	12.707
AC073263.3	7.172	7.367	7.075	7.076	7.316	7.301
AC073263.2	6.471	6.700	6.584	6.786	6.739	6.706
AC013271.1	7.564	7.798	7.581	7.620	7.841	7.801
AL365203.2	8.661	8.642	8.562	8.244	8.209	8.363
UBTD2	10.422	10.348	10.483	10.334	10.265	10.336
UBTD1	7.936	8.098	8.194	8.276	8.129	8.090
LINC02210	9.490	9.579	9.608	9.338	9.546	9.512
AL160408.1	6.716	6.915	6.660	6.786	6.743	6.723
LINC02218	6.991	7.287	6.985	7.074	6.884	7.046
TBC1D3P1-DHX4	7.036	7.224	7.048	7.160	7.204	7.353
MYCBPAP	7.568	7.301	7.229	7.767	7.666	7.568
AL390726.4	7.250	7.116	7.153	6.836	6.793	6.949
TUBD1	6.891	6.696	6.901	6.658	6.587	6.781
MRNIP	9.911	10.032	10.042	9.893	9.913	9.851
PPP1R14BP3	10.594	10.616	10.868	10.408	10.337	10.381
SDSL	8.624	8.782	8.837	8.539	8.541	8.523
RALY	10.699	10.913	11.161	10.851	10.698	10.710
LINC01741	8.002	8.119	7.796	8.080	8.309	8.141
LINC01743	7.634	7.733	7.752	7.582	7.736	7.830
RALB	10.749	10.797	10.856	10.793	10.700	10.845
RALA	11.278	11.342	11.452	11.318	11.248	11.335
ISX	5.944	6.212	6.110	6.213	6.322	6.478
CDKN2B-AS1	7.435	7.533	7.411	7.223	7.549	7.475
RPL7AP9	7.177	7.265	6.858	6.953	7.063	6.982
DBF4B	8.675	8.774	8.775	8.437	8.587	8.463
DNAJB9	8.331	8.392	8.379	8.601	8.695	8.576
DNAJB8	6.213	6.286	6.496	6.495	6.400	6.450
AL353726.2	4.699	4.852	4.875	5.110	4.783	4.630
DNAJB3	6.141	5.935	5.883	6.407	6.323	6.303
DNAJB2	10.187	10.379	10.464	10.286	10.332	10.244
DNAJB1	11.717	11.858	11.934	11.905	11.710	11.887
ERCC6L	7.814	8.300	8.123	7.590	7.653	7.846
DNAJB7	4.008	4.041	3.926	4.059	3.963	4.092
DNAJB6	8.842	9.011	9.017	9.008	8.904	9.004
DNAJB5	8.008	7.876	8.176	8.406	8.164	8.133
DNAJB4	7.618	7.367	7.421	7.815	8.005	7.998
GPATCH11	7.392	7.401	7.540	7.245	7.239	7.147
AMDHD2	13.118	13.168	13.219	13.060	12.975	13.021
DIDO1	9.346	9.476	9.507	9.323	9.218	9.417
TTLL13P	6.707	6.766	6.612	7.011	6.884	6.885
KLRC4-KLRK1	7.160	7.040	7.142	7.546	7.298	7.477
GRIK1	5.450	5.331	5.222	5.343	5.258	5.549
GRIK2	6.242	6.164	6.360	6.374	6.258	6.315
GRIK3	5.987	5.967	5.868	5.987	6.256	6.143
GRIK4	5.848	5.857	5.905	5.879	5.992	6.187
GRIK5	9.911	9.813	9.973	10.087	10.028	10.015
ATP5MPL	12.059	12.184	12.288	12.244	12.152	12.235

RNF148	5.512	5.467	5.490	5.432	5.466	5.727
SGSH	8.670	8.768	8.636	8.437	8.433	8.591
RNF141	10.862	10.836	10.898	10.526	10.451	10.525
RNF145	11.768	11.832	11.886	11.872	11.756	11.742
RNF146	9.579	9.720	9.802	9.692	9.676	9.713
GRSF1	10.561	10.737	10.779	10.752	10.585	10.627
NENF	9.885	9.902	10.045	9.777	9.715	9.819
CALU	10.900	10.981	11.038	10.932	10.991	10.915
CSTF3-DT	3.549	3.709	3.607	3.703	3.709	3.741
XCR1	7.130	7.203	7.047	7.156	7.204	6.986
AC079228.1	11.353	11.397	11.535	11.230	11.199	11.340
XPR1	10.551	10.509	10.572	10.466	10.416	10.393
NFXL1	7.616	7.593	7.780	7.582	7.403	7.599
AC073857.1	8.348	8.295	8.231	7.727	7.682	7.942
GREM2	6.451	6.565	6.508	6.544	6.508	6.653
GREM1	4.461	4.482	4.566	4.691	4.545	4.640
PADI1	9.928	9.820	9.898	9.886	9.855	9.938
PADI3	8.338	8.043	8.216	8.111	8.100	7.991
PADI2	8.200	8.153	8.202	8.250	8.143	8.169
PADI4	5.667	5.899	5.707	5.986	5.923	5.799
ZPLD1	4.824	4.944	4.606	4.858	5.116	4.898
GSTP1	11.957	12.047	12.327	12.087	12.059	12.062
TWISTNB	9.176	9.278	9.326	9.095	9.090	9.289
ATP6V1C2	7.061	7.200	7.205	7.188	7.402	7.276
ATP6V1C1	9.695	9.707	9.746	9.302	9.253	9.418
VPS37A	9.156	9.184	9.330	9.058	9.036	9.054
AOC4P	7.903	7.867	7.962	8.065	8.117	8.133
AC103923.1	8.095	8.094	7.666	7.924	8.029	8.069
TOMM6	11.796	11.909	12.049	12.073	12.009	12.047
PPP1R3F	8.334	8.393	8.302	8.416	8.575	8.460
PPP1R3E	7.959	7.812	7.990	7.660	7.719	7.812
PPP1R3D	9.624	9.658	9.708	9.491	9.589	9.427
PPP1R3C	9.001	9.026	9.249	8.786	8.975	8.838
PPP1R3B	10.484	10.452	10.583	10.451	10.308	10.533
PPP1R3A	3.849	3.896	3.949	3.988	3.890	4.033
NSG1	7.432	7.396	7.274	7.310	7.485	7.283
NSG2	8.117	7.993	7.979	8.289	8.142	7.979
AL162727.3	5.954	5.951	5.802	5.922	5.980	5.883
PPP2R3C	8.018	8.174	8.394	7.859	7.736	7.998
TBXAS1	8.125	8.166	8.472	7.980	8.127	8.065
BTN2A3P	7.856	7.682	7.965	7.993	7.773	7.854
PPP1R37	8.762	8.847	9.093	8.731	8.771	8.710
PPP1R36	7.088	6.843	6.760	7.220	7.272	7.005
PPP1R35	8.976	8.929	9.124	8.858	9.029	8.959
PPP1R32	6.332	6.149	6.493	6.706	6.368	6.405
AC275614.1	3.830	4.000	4.066	3.900	3.760	4.082
ZBTB1	9.169	9.298	9.339	9.204	9.082	9.067
INPP5A	9.465	9.534	9.670	9.419	9.299	9.418
INPP5B	7.295	7.488	7.310	7.201	7.269	7.502
INPP5D	7.674	7.663	7.577	7.663	7.775	7.829
INPP5E	9.380	9.139	9.457	9.278	9.258	9.225
INPP5F	7.191	7.187	7.116	6.852	7.033	7.282
VSIG10L2	5.360	5.642	5.392	5.313	5.429	5.508
INPP5J	8.359	8.268	8.480	8.556	8.529	8.439
INPP5K	8.701	8.803	8.871	8.794	8.725	8.731
MSANTD4	8.766	8.860	8.774	8.702	8.630	8.817

MSANTD1	8.608	8.319	8.345	8.572	8.688	8.633
MSANTD2	6.774	6.805	7.036	6.862	7.085	6.671
MSANTD3	8.874	8.919	8.857	8.637	8.821	8.771
AC025165.3	9.725	9.770	9.962	9.715	9.639	9.552
CNST	8.678	8.650	8.626	8.517	8.617	8.599
AL034548.1	5.022	4.924	4.866	4.901	5.080	5.035
AC092597.1	12.772	12.801	12.947	12.791	12.625	12.834
AC112719.2	4.000	4.055	3.886	4.015	3.929	4.146
VPS37D	8.285	8.392	8.456	8.452	8.529	8.327
VPS37C	10.327	10.400	10.533	9.991	10.110	10.179
VPS37B	10.646	10.723	10.909	10.563	10.415	10.531
AL035448.1	4.480	4.583	4.382	4.324	4.429	4.521
GUCA2A	8.744	8.569	8.866	8.930	8.887	8.892
GUCA2B	7.012	7.260	7.129	7.172	7.362	7.285
RPL13P5	8.515	8.558	8.776	8.580	8.650	8.632
AC015910.1	5.921	6.042	5.954	6.125	6.072	6.107
AC025165.6	7.851	7.724	7.940	7.955	7.950	7.940
HOMEZ	6.976	7.039	7.004	6.855	7.200	7.022
AC025171.4	5.154	4.987	4.966	5.055	4.878	5.199
AC093510.1	6.883	6.783	6.665	7.109	6.832	6.817
AC025171.2	8.330	8.223	8.212	8.316	8.350	8.115
COL12A1	6.880	7.238	6.984	7.076	7.119	7.183
PF4V1	3.787	3.979	3.678	3.863	3.831	3.898
CHIA	7.126	7.133	7.135	7.257	7.295	7.257
CARNMT1	8.082	8.271	7.983	7.743	7.824	7.869
SLC38A11	6.084	6.052	5.960	6.022	6.276	6.246
RBM8A	10.963	11.063	11.132	10.803	10.694	10.784
RBM8B	10.963	11.063	11.132	10.803	10.694	10.784
AL445423.1	5.293	5.279	5.348	5.360	5.495	5.400
AL445423.3	5.922	5.986	6.048	6.035	6.015	6.190
CAST	12.281	12.290	12.324	12.231	12.162	12.209
MKNK1-AS1	6.936	6.910	6.726	7.141	7.043	7.162
CASR	8.437	8.460	8.308	8.446	8.491	8.480
CLEC1A	7.469	7.509	7.019	7.514	7.543	7.436
ADAM21	6.549	6.496	6.180	6.144	6.424	6.377
ADAM20	6.852	6.944	6.981	6.886	6.936	7.047
ADAM23	8.377	8.739	8.524	8.436	8.434	8.493
ADAM22	7.998	7.975	8.165	7.912	7.930	8.153
ADAM29	5.776	5.927	5.848	5.771	5.925	5.949
ADAM28	10.433	10.454	10.508	10.277	10.091	10.200
C5orf47	6.771	6.847	6.735	6.817	6.760	6.935
SPAG5-AS1	7.585	7.486	7.188	7.659	7.418	7.583
TMSB15A	6.335	6.577	6.980	6.090	6.340	6.201
PTPRG-AS1	5.631	5.814	5.752	5.865	5.939	5.804
AC093227.1	6.117	6.151	6.307	5.737	5.729	5.921
AC092299.1	10.722	10.564	10.478	10.430	10.307	10.610
MMEL1	8.264	8.355	8.469	8.251	8.260	8.245
TTF2	8.012	7.965	7.888	7.198	7.336	7.367
TTF1	8.283	8.178	8.258	8.082	7.968	8.026
ZNF544	8.868	8.913	8.923	8.792	8.668	8.745
KRTAP8-1	8.066	7.813	8.108	8.211	8.386	8.238
AC093510.2	3.933	3.864	3.957	4.029	3.919	3.904
CBR4	8.880	8.891	8.983	8.713	8.576	8.652
CBR1	9.617	9.699	9.882	9.648	9.471	9.632
CBR3	7.435	7.591	7.755	7.546	7.539	7.468
ANXA1	12.541	12.648	12.773	12.723	12.614	12.697

ANXA3	10.549	10.604	10.734	10.560	10.472	10.584
ANXA2	14.352	14.352	14.362	14.343	14.357	14.360
ANXA5	12.359	12.364	12.516	12.416	12.378	12.351
ANXA4	13.699	13.807	13.835	13.741	13.795	13.835
ANXA7	10.367	10.370	10.467	10.329	10.363	10.358
ANXA6	8.412	8.389	8.399	8.362	8.307	8.291
ANXA9	10.131	10.309	10.466	10.272	10.400	10.235
ANXA8	8.459	8.416	8.261	8.458	8.302	8.483
MEST	10.881	11.008	11.022	10.995	10.836	10.884
FOXD3-AS1	6.722	7.020	6.798	7.010	7.218	7.049
POU5F1	8.619	8.606	8.654	8.646	8.777	8.730
SASS6	6.427	6.369	6.634	6.174	6.071	6.178
POU5F2	5.798	6.167	6.047	6.138	6.067	6.029
MESD	11.182	11.192	11.350	11.029	10.842	10.979
ZNF541	7.382	7.272	7.256	7.268	7.448	7.195
RELCH	9.304	9.362	9.368	9.120	9.096	9.160
HEPACAM	7.492	7.447	7.312	7.504	7.627	7.437
M6PR	11.502	11.686	11.761	11.601	11.751	11.240
SSX4	7.301	7.374	7.153	7.302	7.279	7.432
SSX5	7.180	7.028	7.030	7.279	7.191	7.250
SSX2	7.872	7.778	7.610	7.837	7.973	7.959
SSX3	7.872	7.778	7.610	7.837	7.973	7.959
USH2A	4.485	4.412	4.564	4.613	4.770	4.729
SSX1	7.301	7.374	7.153	7.302	7.279	7.432
MRGBP	9.073	9.125	9.199	9.071	9.084	9.014
PIBF1	6.908	6.952	7.086	6.886	7.004	7.131
MDS2	7.777	7.887	7.911	7.681	8.009	7.984
CPT2	9.939	10.005	10.139	9.625	10.082	9.703
PYGB	12.528	12.554	12.635	12.282	12.153	12.324
PYGL	9.506	9.528	9.667	9.427	9.293	9.456
PYGM	7.081	7.177	7.099	7.224	7.037	7.191
PFAS	8.778	8.861	8.944	8.455	8.683	8.639
CPTP	9.062	9.132	9.354	9.039	9.127	9.109
MIR6804	9.863	9.901	10.100	9.944	9.785	9.728
MIR6807	12.082	12.083	12.360	12.096	12.057	11.958
PMS1	8.830	8.844	8.828	8.848	8.717	8.866
PMS2	9.485	9.466	9.613	9.501	9.346	9.296
ATXN7L3B	11.293	11.253	11.363	11.184	11.199	11.224
MST1R	10.297	10.267	10.399	10.193	10.186	10.204
MST1L	8.186	8.305	8.122	8.250	8.252	8.202
HUNK	7.513	7.609	7.460	7.529	7.468	7.522
GRIP1	7.257	7.237	7.300	7.188	7.208	7.336
GRIP2	8.571	8.386	8.603	8.653	8.836	8.709
SLC19A2	9.454	9.278	9.569	9.426	9.392	9.355
SLC19A3	7.908	7.924	7.847	7.621	7.827	7.792
SLC19A1	8.498	8.694	8.794	8.558	8.778	8.662
TRGC1	7.335	7.503	7.331	7.512	7.583	7.529
MIS12	8.312	8.295	8.421	8.291	8.208	8.392
TRGC2	7.335	7.503	7.331	7.512	7.583	7.529
LINC02458	5.416	5.431	5.369	5.187	5.456	5.372
LINC02450	7.749	7.795	7.555	8.010	7.789	7.794
MED12L	6.972	6.910	6.899	7.005	7.029	7.119
NXF5	7.117	6.978	6.620	6.915	6.982	6.867
NXF1	10.404	10.364	10.616	10.319	10.396	10.373
NXF2	6.784	6.838	6.902	6.852	7.061	6.978
FLG2	4.585	4.810	4.562	4.716	4.705	4.794

FAF1	10.350	10.503	10.513	10.332	10.226	10.354
PGM5	6.695	7.095	7.193	7.083	7.101	7.008
FAF2	10.631	10.499	10.567	10.535	10.454	10.414
AC095057.3	3.922	3.985	4.086	3.932	4.078	3.970
PGM2	8.544	8.669	8.654	8.466	8.420	8.570
PGM3	7.796	7.858	7.792	7.800	7.672	7.709
MTCO2P34	6.122	6.161	5.611	6.619	6.291	6.188
SBK1	7.684	7.643	7.724	7.624	7.824	7.775
AC016876.3	13.137	13.230	13.247	13.064	13.071	13.089
CDC27	9.983	10.020	10.043	9.712	9.705	9.804
CDC26	9.453	9.547	9.557	9.402	9.470	9.515
CDC23	9.413	9.419	9.511	9.158	9.188	9.216
HOXD13	6.692	6.825	6.890	7.180	7.047	6.818
HOXD10	6.675	6.481	6.341	6.517	6.451	6.505
CDC20	9.625	9.634	9.850	9.165	9.339	9.109
SHBG	8.080	8.205	8.192	8.274	8.104	8.238
LIN54	5.360	5.426	5.154	4.968	5.002	5.284
LIN52	9.477	9.405	9.290	9.189	9.412	9.336
TNC	5.435	5.691	5.603	6.060	5.708	5.718
RPL35A	12.696	12.814	12.962	12.934	12.824	12.905
LINC00587	4.458	4.245	4.420	4.450	4.625	4.533
CASK	10.281	10.297	10.431	10.316	10.194	10.119
LINC00582	7.188	7.091	7.108	7.190	7.373	7.329
FABP6-AS1	4.577	4.384	4.554	4.792	4.759	4.667
LINC00588	4.407	4.298	4.263	4.249	4.496	4.347
LINC00589	7.320	7.659	7.365	7.399	7.575	7.566
OSTC	11.211	11.307	11.386	11.538	11.580	11.504
LINC01189	4.141	4.060	4.251	4.180	4.213	4.069
PPP1R12A	9.265	9.150	9.390	9.053	9.073	9.070
PPP1R12B	7.936	7.965	8.039	7.992	7.814	7.934
PPP1R12C	9.400	9.404	9.672	9.719	9.385	9.516
LINC01180	6.071	6.223	6.176	6.100	6.196	6.219
LINC01187	6.895	6.730	6.745	6.890	6.796	6.696
LINC01186	6.413	6.526	6.239	6.437	6.544	6.633
LINC01184	8.305	8.515	8.557	8.621	8.463	8.532
STAG3L4	6.586	6.525	6.313	6.623	6.579	6.535
STAG3L3	7.508	7.414	7.319	7.392	7.633	7.403
STAG3L2	7.508	7.414	7.319	7.392	7.633	7.403
STAG3L1	6.539	6.558	6.624	6.505	6.470	6.551
BCAP31	12.803	12.934	13.042	12.902	12.718	12.824
VPS35	12.469	12.523	12.582	12.558	12.486	12.418
VPS36	8.812	8.744	8.720	8.469	8.346	8.457
VPS39	8.997	9.027	8.976	8.885	8.881	8.939
TAS2R43	7.431	7.488	7.272	7.373	7.513	7.542
TAS2R41	6.164	6.173	6.097	5.977	6.276	6.097
TAS2R40	4.558	4.772	4.788	4.518	4.785	4.841
TAS2R45	7.431	7.488	7.272	7.373	7.513	7.542
KCTD3	8.235	8.597	8.528	7.950	8.041	8.162
KCTD2	9.037	9.120	9.229	9.049	9.040	9.013
KCTD1	10.090	10.047	10.079	9.998	10.146	9.947
KCTD7	7.278	7.367	7.297	7.465	7.104	7.161
KCTD6	7.919	7.925	7.910	7.712	7.973	8.220
KCTD5	10.142	10.257	10.307	10.174	10.110	10.007
KCTD4	7.120	7.295	7.294	7.203	7.302	7.247
KCTD9	8.552	8.656	8.627	8.165	8.190	8.396
LINC-PINT	7.301	7.240	7.168	7.295	7.137	7.226

AL031985.3	8.122	8.092	7.936	8.074	7.960	7.879
PXK	8.111	8.185	8.035	8.070	8.117	8.300
SLC38A10	9.650	9.715	9.939	9.572	9.624	9.669
PXN	11.079	11.154	11.232	10.996	10.902	10.937
ESF1	8.479	8.191	8.493	8.229	8.463	8.142
AC127459.1	7.906	7.974	7.879	7.519	7.556	7.849
NKX6-3	8.257	8.310	8.040	8.390	8.242	8.439
BRAP	8.876	8.919	9.070	8.917	9.011	8.960
USPL1	7.850	7.943	7.851	8.088	7.996	8.149
NEDD4L	10.027	10.042	10.002	10.042	9.954	9.944
UPF3A	8.166	8.131	8.061	7.564	7.605	7.719
BRAF	8.328	8.455	8.316	8.325	8.288	8.443
UAP1L1	7.195	7.143	7.303	6.782	7.041	6.994
IFITM10	8.648	8.766	8.802	8.613	8.596	8.698
UBL5	10.124	10.109	10.447	10.118	10.027	10.167
AC005702.1	7.700	7.562	7.647	7.527	7.430	7.471
STK19	9.245	9.328	9.319	9.203	9.266	9.289
AC025125.1	3.837	3.749	4.095	3.756	3.736	3.864
AC106873.4	4.618	4.379	4.225	4.368	4.621	4.495
NTNG1	7.560	7.585	7.602	7.536	7.514	7.518
NTNG2	5.747	5.705	5.664	5.626	5.748	5.948
ZFYVE16	7.879	7.771	7.820	7.694	7.502	7.658
THY1	7.587	7.577	7.475	7.547	7.784	7.524
AC007342.5	8.470	8.539	8.562	8.643	8.799	8.853
OPN1SW	6.537	6.655	6.599	6.796	6.589	6.721
MIR137HG	4.050	3.900	3.965	4.051	3.894	4.054
TRAF3IP3	6.626	6.765	6.922	6.754	6.863	6.858
TRAF3IP2	9.439	9.584	9.732	9.396	9.412	9.332
FAM242F	7.631	7.537	7.702	8.045	7.791	7.944
FAM242E	7.631	7.537	7.702	8.045	7.791	7.944
TRAF3IP1	8.399	8.384	8.408	8.252	8.270	8.178
ANGPTL7	6.229	6.371	6.282	6.400	6.541	6.443
ANGPTL6	7.269	7.464	7.473	7.467	7.575	7.578
ANGPTL4	8.002	8.099	8.212	8.191	8.118	8.111
ANGPTL3	4.621	4.364	4.632	4.789	4.806	4.747
ANGPTL2	7.300	7.335	7.192	7.386	7.670	7.578
ANGPTL1	6.185	6.216	6.440	6.489	6.549	6.424
ANGPTL8	6.113	5.981	6.085	6.123	6.162	6.246
MMP14	8.985	9.008	9.027	9.030	9.082	9.085
MMP15	9.971	10.181	10.258	10.124	10.026	10.001
MMP16	7.275	7.072	7.189	7.622	7.313	7.149
MMP17	7.919	8.099	8.153	8.083	8.083	8.209
MMP10	4.425	4.411	4.392	4.423	4.504	4.689
MMP11	8.335	8.257	8.437	8.479	8.450	8.426
MMP12	5.600	5.738	5.551	5.651	5.823	5.957
MMP13	6.950	6.736	6.722	7.079	6.877	6.847
AC146944.1	5.859	5.735	5.446	5.456	5.312	5.507
AC146944.2	5.859	5.735	5.446	5.456	5.312	5.507
AC146944.3	6.226	5.977	5.916	5.792	6.012	5.967
MMP19	8.517	8.492	8.465	8.706	8.669	8.655
AL031595.3	5.255	5.188	5.406	5.494	5.403	5.270
CR854858.1	7.406	7.569	7.636	7.983	7.893	7.730
TMSB15B	7.592	7.538	7.717	7.562	7.631	7.574
CDAN1	6.661	6.782	7.005	6.764	6.705	6.970
KCNK16	7.725	7.701	7.726	7.617	7.942	7.746
KCNK17	7.382	7.348	7.295	7.665	7.590	7.593

KCNK12	5.290	5.234	5.141	5.433	5.316	5.333
KCNK13	5.773	6.128	5.892	5.921	5.950	6.049
KCNK10	7.008	7.032	7.148	7.507	7.221	7.251
ZC2HC1A	8.176	8.070	8.294	8.029	8.469	8.034
AC090204.1	6.021	6.384	6.144	5.923	6.369	5.951
MMACHC	7.935	8.030	7.983	7.865	7.716	7.737
FAM199X	9.877	9.874	10.035	9.703	9.768	9.864
C14orf180	7.924	7.806	7.630	7.596	7.820	7.820
AC007998.4	7.517	7.562	7.682	7.749	7.683	7.682
TCN1	12.611	12.747	12.994	12.743	12.858	12.728
TCN2	8.179	8.364	8.334	8.018	8.138	8.130
KRT8P17	7.481	7.629	7.694	7.781	7.761	7.666
KRT8P11	8.195	8.221	8.301	8.343	8.133	8.244
KRT8P12	8.638	8.502	8.724	8.536	8.310	8.600
AC006252.1	8.005	7.922	7.884	7.630	7.963	7.961
SAP30-DT	4.168	4.221	4.133	3.997	3.923	4.079
GPRC5D	7.225	7.400	7.051	7.206	7.430	7.404
GPRC5A	12.985	13.037	13.050	12.994	12.887	13.022
GPRC5C	9.465	9.394	9.713	9.534	9.566	9.535
GPRC5B	11.171	11.216	11.284	10.893	10.910	10.823
SCLT1	7.422	7.175	7.406	6.716	7.017	7.279
AP005264.5	7.752	7.751	7.609	7.793	7.658	7.757
SCAT1	6.019	5.926	6.099	6.033	5.891	6.208
CPOX	7.801	7.982	8.009	7.578	7.802	7.942
ALG10	6.834	7.092	7.042	6.494	7.141	6.771
ALG11	9.579	9.473	9.557	9.413	9.427	9.365
ALG12	9.383	9.354	9.484	9.222	9.279	9.304
ALG13	9.633	9.755	9.917	9.669	9.684	9.653
ALG14	8.468	8.474	8.671	8.118	8.185	8.216
RIOK2	9.200	9.175	9.372	8.932	9.121	9.019
RIOK3	11.012	11.092	11.112	10.767	10.795	10.848
RIOK1	7.417	7.399	7.544	7.243	7.369	7.283
SIX3-AS1	6.440	5.923	6.236	6.700	6.546	6.525
BANF1	11.977	12.013	12.221	12.023	11.864	12.002
BANF2	6.328	6.310	6.306	6.933	6.308	6.492
ZNF259P1	8.623	8.701	8.792	8.576	8.445	8.520
AHSP	7.544	7.582	7.625	7.680	7.477	7.499
AHSG	7.364	7.572	7.609	7.536	7.484	7.665
AL133244.2	6.404	6.625	6.564	6.455	6.756	6.768
SHISA4	8.160	8.152	8.399	8.308	8.214	8.088
SHISA5	11.240	11.347	11.440	11.274	11.357	11.258
AC087482.1	4.957	5.122	5.112	4.890	4.979	5.108
TMEM217	6.327	6.282	6.297	6.611	6.453	6.461
WIPI1	9.061	9.232	9.276	8.934	9.081	9.006
AL022162.1	7.389	7.617	7.741	7.504	7.430	7.390
AL035425.3	6.610	6.772	6.853	6.990	6.827	6.860
AL035425.4	5.268	5.178	5.156	5.427	5.292	5.366
IL37	8.604	8.424	8.611	8.577	8.634	8.447
IL34	8.148	8.128	8.428	8.480	8.388	8.470
IL32	8.171	8.347	8.415	8.673	8.811	8.773
IL33	4.550	4.406	4.724	4.473	4.585	4.319
NKPD1	7.556	7.689	7.798	7.582	7.802	7.803
HMBOX1	9.624	9.742	9.518	9.631	9.483	9.783
AL645937.1	8.529	8.524	8.065	8.442	8.567	8.533
COX14	9.685	9.766	10.017	9.772	9.857	9.778
AL645937.3	7.145	7.246	7.000	7.072	7.096	7.194

OR4C1P	6.525	6.193	6.098	6.559	6.537	6.668
AL133279.3	5.688	5.584	5.345	5.769	5.544	5.804
CAMK2N2	8.693	8.849	8.870	8.669	8.816	8.748
CAMK2N1	11.511	11.475	11.598	11.317	11.259	11.414
RHPN1-AS1	8.047	8.129	8.063	8.141	8.131	8.132
HYOU1	11.279	11.426	11.467	11.374	11.338	11.266
GTSF1L	7.199	7.300	7.309	7.518	7.501	7.497
AL136317.2	4.052	4.223	4.023	3.984	4.149	4.127
AMACR	7.708	7.761	7.739	7.656	7.651	7.690
AC244197.3	11.178	11.306	11.302	11.091	11.030	11.169
AC244197.2	6.859	6.946	6.841	7.015	7.147	6.893
POC1B-AS1	7.609	7.498	7.767	7.655	7.587	7.599
AC231381.1	7.597	7.542	7.551	7.581	7.769	7.718
AZU1	6.606	6.649	6.631	6.431	6.609	6.582
SAYSD1	8.678	8.705	8.789	8.290	8.481	8.311
ERAP2	9.898	9.911	9.859	9.686	9.717	9.714
ERAP1	8.150	8.083	8.085	7.782	7.855	8.035
AC226007.1	6.768	6.598	6.540	6.777	6.848	6.929
NMRK2	7.329	7.364	7.494	7.664	7.333	7.282
NMRK1	8.715	8.873	8.711	8.604	8.770	8.725
PCOTH	7.787	7.839	7.691	7.717	7.729	7.859
PLA2G1B	5.502	5.384	5.289	5.723	5.433	5.650
DDX58	9.554	9.505	9.419	9.552	9.718	9.302
DDX59	9.150	9.185	9.153	9.090	8.969	9.200
AC073283.1	7.491	7.558	7.421	7.343	7.411	7.500
DDX52	11.096	11.136	11.180	11.061	11.224	10.877
DDX53	3.528	3.639	3.701	3.749	3.750	3.863
DDX50	9.444	9.599	9.589	9.384	9.229	9.349
DDX51	7.900	8.034	7.984	8.135	7.859	7.813
DDX56	10.718	10.768	10.939	10.700	10.509	10.612
DDX54	9.220	9.299	9.495	9.240	9.193	9.152
DDX55	8.577	8.553	8.496	8.309	8.416	8.431
GATM	7.373	7.386	7.313	7.305	7.157	7.127
IGHV4-34	6.838	6.893	6.998	6.998	6.973	7.134
GATB	7.785	7.808	7.987	7.605	7.678	7.672
GATC	10.352	10.484	10.682	9.973	10.428	10.124
DNAH2	7.058	7.259	7.448	7.431	7.330	7.403
PLA2G15	7.979	8.028	8.202	7.843	8.094	7.973
EGLN1	10.371	10.443	10.506	10.210	10.099	10.152
DNAH1	7.517	7.498	7.466	7.464	7.490	7.581
DNAH6	8.568	8.664	8.468	8.347	8.648	8.793
DNAH7	7.063	7.250	7.051	6.939	7.316	7.313
DNAH5	7.230	7.428	7.291	7.442	7.149	7.181
DNAH8	6.106	5.937	5.970	5.916	6.046	6.050
DNAH9	7.359	7.546	7.311	7.491	7.501	7.312
AC011676.5	5.181	5.088	5.048	4.944	4.964	5.059
NYAP2	6.732	6.688	6.321	6.571	6.619	6.601
GPR17	5.625	5.609	5.660	5.899	5.718	5.781
AC023154.1	7.336	7.503	7.345	7.381	7.420	7.459
HSD17B10	11.373	11.458	11.510	11.298	11.229	11.293
LAG3	6.321	6.217	6.286	6.439	6.512	6.466
AL136295.2	6.938	7.101	7.036	7.140	7.285	7.004
AL136295.5	11.340	11.371	11.352	11.303	11.317	11.334
AL021877.1	7.427	7.420	7.545	7.360	7.554	7.463
AL136295.4	10.845	10.861	11.040	10.826	10.739	10.727
CYP4F60P	7.165	7.227	7.364	7.707	7.106	7.289

GNB5	9.723	9.764	9.957	9.793	9.994	9.562
GNB4	10.122	10.212	10.163	10.012	9.977	10.075
GNB1	11.698	11.716	11.865	11.657	11.593	11.507
GNB3	7.996	7.924	8.025	8.191	8.031	7.973
GNB2	10.642	10.736	10.890	10.759	10.783	10.667
OPN1LW	6.498	6.624	6.478	6.739	6.566	6.784
AC023509.3	7.597	7.610	7.435	7.711	7.687	7.517
AC023509.2	7.753	7.780	7.798	7.910	7.828	7.784
AC023509.1	8.195	8.190	8.099	8.170	8.299	7.922
PARD3B	7.447	7.451	7.473	7.913	7.750	7.672
CKAP2L	8.316	8.561	8.471	8.028	8.165	8.113
ETFA	11.961	12.038	12.135	12.099	12.017	12.120
AC093616.1	8.228	8.464	8.401	7.869	8.135	8.215
TIGD2	8.621	8.593	8.744	8.331	8.273	8.451
GTF2A1	9.942	9.892	9.893	9.223	9.282	9.282
GTF2A2	11.369	11.468	11.596	11.572	11.570	11.626
IGKV2-28	6.483	6.636	6.681	6.760	6.476	6.601
IL18BP	8.384	8.467	8.546	8.357	8.425	8.578
YBX1P1	13.393	13.414	13.539	13.442	13.322	13.412
RPE	9.601	9.786	9.687	9.627	9.407	9.607
RP1	4.016	3.877	4.131	4.208	4.494	4.146
RP2	9.170	9.232	8.997	8.831	8.810	9.178
AP000944.2	8.356	8.131	7.912	8.317	8.100	8.120
AP000944.3	8.356	8.131	7.912	8.317	8.100	8.120
AP000944.5	9.665	9.675	9.820	9.829	9.909	9.760
DUTP1	6.941	7.154	6.969	6.970	7.032	7.260
CALD1	6.962	6.924	6.763	7.169	7.129	7.134
AL121900.1	9.097	9.201	9.366	8.851	8.819	8.924
AC078881.1	4.208	4.494	4.284	4.296	4.426	4.238
OR1F2P	6.431	6.226	6.415	6.570	6.639	6.350
NUP62CL	8.318	8.447	8.499	7.920	7.945	8.063
CSTF1	8.726	8.716	8.802	8.510	8.631	8.699
CSTF3	10.444	10.493	10.553	10.658	10.517	10.572
CSTF2	9.844	9.825	9.959	9.770	9.675	9.668
AC012360.3	8.328	8.179	8.101	8.393	8.156	8.105
AC025580.3	4.540	4.469	4.493	4.615	4.397	4.630
BPTF	10.076	9.970	10.059	9.739	10.090	9.672
HM13-IT1	6.794	6.655	6.564	6.605	6.711	6.692
SYMPK	8.888	8.902	9.017	8.536	8.563	8.676
TNMD	5.721	5.560	5.560	5.997	5.867	5.674
ADGRV1	7.588	7.389	7.535	7.579	7.411	7.491
SOX30	4.437	4.463	4.868	4.685	4.554	4.579
SERPINC1	6.167	5.955	6.059	6.109	6.112	6.322
AC020915.1	8.845	8.887	8.911	8.976	8.959	9.090
AC020915.3	7.881	7.752	7.970	8.070	8.101	7.882
AC020915.5	8.845	8.887	8.911	8.976	8.959	9.090
AL451062.1	4.505	4.655	4.654	4.734	4.656	4.611
CLN6	10.481	10.612	10.681	10.258	10.358	10.191
CLN5	7.966	7.944	7.997	7.688	8.077	7.798
IGKJ3	4.150	4.226	4.135	4.366	4.272	4.315
CLN3	10.130	10.253	10.370	10.235	10.127	10.137
AL451062.4	9.334	9.428	9.462	9.552	9.513	9.252
AC256234.1	4.957	5.051	4.936	4.868	4.936	5.007
CLN8	8.711	8.989	9.013	8.658	8.982	8.885
CLNK	7.767	7.779	7.652	7.688	7.849	7.823
ACSM2A	6.431	6.439	6.284	6.579	6.419	6.442

ACSM2B	6.431	6.439	6.284	6.579	6.419	6.442
BX890604.2	8.834	8.823	9.001	8.733	8.661	8.696
OR7E104P	11.640	11.466	11.434	11.495	11.677	11.790
GCSAML	3.780	4.013	4.125	4.231	4.127	3.998
AC244097.1	5.842	5.912	6.019	6.408	6.090	6.168
OR51I1	7.435	7.589	7.458	7.547	7.576	7.694
OR51I2	7.069	7.355	7.079	7.385	7.333	7.247
AL022311.1	12.863	12.870	12.930	12.590	12.383	12.736
AL359844.1	5.862	6.224	5.956	6.306	6.200	5.924
RBX1	10.316	10.411	10.531	10.347	10.134	10.376
AC087500.1	6.712	6.949	6.756	6.815	6.656	6.965
HRAS	9.196	9.202	9.373	8.731	8.701	8.841
OR7A5	7.924	7.852	7.842	7.951	7.983	7.952
CDYL2	8.779	8.781	8.530	8.834	8.959	8.925
CLRN1-AS1	5.589	5.587	5.213	5.684	5.528	5.926
KRTAP9-8	7.208	7.171	7.073	7.000	7.246	7.201
KRTAP9-9	4.386	4.274	4.203	4.414	4.487	4.418
KRTAP9-2	7.208	7.171	7.073	7.000	7.246	7.201
KRTAP9-3	4.415	4.531	4.037	4.421	4.389	4.333
KRTAP9-4	7.359	7.435	7.468	7.337	7.524	7.653
TTN-AS1	7.426	7.451	7.324	7.297	7.344	7.386
AC105285.1	7.230	7.202	7.534	7.298	7.492	7.371
JMJD1C	8.999	8.820	8.970	8.847	9.009	8.840
AC138430.1	7.769	7.557	7.726	7.527	7.826	7.653
AC008397.2	10.300	10.317	10.270	10.190	10.027	10.182
DTNBP1	8.342	8.508	8.450	8.290	8.140	8.283
CDC14C	3.843	3.820	3.832	3.769	3.818	3.836
CDC14B	9.178	9.070	9.202	8.845	8.864	8.941
CDC14A	7.375	7.340	7.163	7.412	7.478	7.468
GPR171	5.827	5.742	6.060	5.625	5.730	5.882
SPATA6L	7.369	7.599	7.474	7.232	7.478	7.520
GPR174	5.664	5.683	5.636	5.925	5.637	5.651
AC138915.1	9.106	9.330	9.270	9.257	9.359	9.374
GPR176	8.485	8.506	8.549	8.346	8.399	8.568
MEIOC	7.538	7.231	7.775	6.855	7.083	6.945
MEIOB	4.573	4.515	4.542	4.709	4.540	4.555
SH3YL1	11.421	11.490	11.629	11.263	11.257	11.292
AC245407.2	7.320	7.354	7.450	6.865	7.105	7.264
CEACAM21	7.333	7.530	7.497	7.298	7.388	7.726
SS18L2	9.052	9.090	9.142	9.035	9.063	9.110
SS18L1	9.259	9.342	9.339	8.990	8.913	9.253
AC139495.1	4.268	4.234	4.359	4.378	4.576	4.384
AC083843.3	8.371	8.328	8.294	8.039	8.227	8.265
AC011452.1	5.530	5.527	5.506	5.416	5.579	5.741
AC139495.2	4.268	4.234	4.359	4.378	4.576	4.384
COX8A	12.493	12.589	12.805	12.561	12.479	12.477
UCHL5	9.917	10.016	10.063	9.812	9.897	9.926
LINC002481	7.774	7.914	7.787	7.894	7.895	7.771
UCHL1	9.257	9.273	9.367	9.166	9.011	9.257
UCHL3	9.223	9.173	9.344	8.727	8.831	8.761
RARRES2	6.711	6.782	6.440	6.988	6.831	7.040
RARRES1	13.085	13.167	13.241	13.258	13.169	13.196
ZNF404	7.990	7.916	7.970	7.928	7.774	8.116
RNF126P1	7.450	7.688	7.618	7.693	7.485	7.746
ZNF407	8.100	8.016	7.963	7.728	7.771	7.858
ZNF408	8.909	8.957	9.052	9.001	8.927	8.953

ARPC5L	10.466	10.470	10.666	10.423	10.400	10.459
AC018809.3	7.677	7.742	7.648	7.655	7.741	7.672
PRELID1	11.793	11.889	12.020	11.857	11.790	11.760
LINC01578	9.991	10.005	10.150	9.796	9.775	9.803
PRELID2	9.305	9.388	9.350	9.399	9.429	9.489
FAM171B	8.900	8.780	8.925	8.585	8.695	8.699
AC138907.1	3.826	3.885	3.772	3.930	3.832	3.885
AC006001.4	11.357	11.450	11.685	11.266	11.282	11.197
AC006001.3	11.357	11.450	11.685	11.266	11.282	11.197
AC006001.1	4.735	4.680	4.693	5.104	4.790	5.091
Z99129.4	8.189	8.199	8.100	8.000	7.900	8.054
PPP6R2	8.367	8.310	8.432	8.138	8.257	8.253
PPP6R3	10.387	10.259	10.265	9.964	9.836	10.191
PPP6R1	9.863	9.901	10.100	9.944	9.785	9.728
EFCAB7	6.352	6.393	6.508	6.049	6.341	6.242
EFCAB6	7.375	7.573	7.686	7.814	7.574	7.798
AC108693.1	5.390	5.006	4.842	4.918	5.125	5.173
BAMBI	11.115	11.188	11.326	11.018	10.982	11.054
FASLG	6.729	6.730	6.853	7.119	6.837	6.807
AL158835.1	4.628	4.729	4.790	4.611	4.498	4.639
LINC01398	6.427	6.450	6.431	6.405	6.501	6.571
LINC01399	9.305	9.192	9.238	9.380	9.481	9.707
OR5I1	6.846	6.692	6.611	6.740	6.755	6.811
LINC01393	4.224	4.614	4.403	4.395	4.246	4.366
LINC01395	5.396	5.639	5.486	5.528	5.538	5.454
SETD1A	8.024	7.887	7.982	8.033	7.890	7.764
SETD1B	10.216	10.235	10.225	10.126	10.030	10.134
LINC00620	5.886	6.174	6.035	5.777	6.338	6.147
LINC00623	9.525	9.596	9.783	9.541	9.506	9.445
LINC00622	4.272	4.319	4.496	4.485	4.374	4.493
LINC00624	3.652	3.775	3.730	3.697	3.680	3.736
LINC00626	5.740	6.131	5.872	6.178	5.993	5.829
LINC00629	5.070	5.029	4.890	5.002	5.131	4.972
LINC00628	6.303	6.290	6.254	6.634	6.294	6.498
SIAE	7.812	8.016	8.150	7.888	7.785	7.906
MIR3940	9.118	9.108	9.194	8.966	9.390	9.035
HNF4A-AS1	4.367	4.207	4.224	4.351	4.341	4.280
CU234142.1	4.623	4.658	4.664	4.620	4.660	4.680
TESPA1	6.448	6.588	6.522	6.605	6.569	6.681
TOR1B	9.740	9.805	9.874	9.575	9.751	9.712
DUBR	5.946	6.029	5.745	5.898	5.770	5.627
AC098934.3	12.417	12.487	12.646	12.569	12.532	12.593
AC098934.2	5.994	6.075	5.916	6.109	6.081	6.077
AC114267.1	7.920	7.839	7.862	7.891	7.699	7.761
AC098934.4	7.964	7.750	7.838	7.890	7.752	7.876
PXDN	6.611	7.106	6.892	7.177	6.939	6.969
LCAL1	7.633	7.711	7.721	7.584	7.555	7.630
AL669831.1	10.722	10.564	10.478	10.430	10.307	10.610
AL669831.3	7.159	6.862	6.635	6.551	6.694	6.987
AC022509.2	5.841	6.045	5.626	6.236	6.075	5.872
AC022509.3	7.854	7.627	7.760	7.678	7.834	7.683
LANCL2	8.963	8.990	9.086	8.712	8.795	8.724
LANCL3	5.181	5.132	5.420	5.261	5.046	5.262
LANCL1	9.921	9.942	9.999	9.631	9.469	9.636
NPEPPS	11.454	11.431	11.553	11.465	11.586	11.321
AC231657.1	8.439	8.501	8.169	8.401	8.449	8.520

AC231657.3	9.615	9.732	9.855	9.537	9.495	9.509
RAB7B	6.748	6.904	6.582	6.861	6.738	6.695
RAB7A	12.111	12.193	12.293	12.114	12.112	12.193
GNPNAT1	9.977	10.000	10.070	9.963	9.760	9.884
EFEMP1	9.252	9.391	9.474	9.274	9.257	9.351
EFEMP2	8.115	8.160	8.319	8.386	8.327	8.334
TSPY9P	4.547	4.525	4.638	4.840	4.849	4.586
SCTR	6.953	6.899	7.073	7.122	7.268	6.760
WNT5B	6.905	6.592	6.622	7.007	6.909	6.871
WNT5A	4.545	4.531	4.445	4.502	4.472	4.690
HNRNPH1	11.571	11.399	11.529	11.163	11.251	11.441
HNRNPH3	9.656	9.709	9.665	9.529	9.510	9.572
HNRNPH2	10.786	11.013	10.988	10.811	10.570	10.803
RUSC1-AS1	6.965	6.830	6.878	6.674	6.733	6.894
ICA1L	6.771	6.997	6.897	7.020	6.766	6.944
OXER1	9.295	9.441	9.402	9.164	9.423	9.367
HNRNPUL2	9.394	9.464	9.549	9.107	8.959	9.171
HNRNPUL1	11.005	11.003	10.908	10.744	10.929	10.906
SPAST	7.660	7.786	7.543	7.086	7.530	7.688
STOML3	6.005	6.205	6.066	6.254	6.175	6.142
PAIP2	10.284	10.354	10.411	10.173	10.184	10.288
PAIP1	9.976	10.097	10.085	10.006	10.059	10.061
AC093012.1	7.160	7.207	7.211	7.362	7.207	7.398
FOXG1	6.768	6.784	6.677	6.696	6.780	6.706
AC024940.9	14.076	14.099	14.141	14.125	14.125	14.139
DCAF13	9.799	9.925	9.909	9.809	9.715	9.732
DCAF12	9.516	9.534	9.474	9.271	9.250	9.298
DCAF11	9.648	9.772	9.812	9.437	9.554	9.499
DCAF10	7.802	7.904	7.788	7.688	7.883	7.688
DCAF17	6.210	6.097	6.147	5.880	5.817	6.325
DCAF16	8.439	8.137	8.367	7.888	8.022	7.683
DCAF15	8.570	8.608	8.702	8.382	8.632	8.516
PAPSS1	10.836	10.937	11.133	10.760	10.931	10.563
TXN	11.536	11.661	11.831	11.560	11.489	11.607
PAPSS2	10.931	10.829	11.056	10.680	10.870	10.740
TXK	6.388	6.528	6.488	6.255	6.267	6.233
SLC8A1-AS1	5.134	5.185	5.049	5.273	5.159	5.159
WARS2	8.305	8.385	8.565	8.024	8.061	8.036
AC093028.1	7.354	7.270	6.981	7.164	7.290	7.505
TRMT10A	5.880	5.930	5.787	5.850	5.941	6.171
TRMT10C	9.656	9.637	9.744	9.680	9.669	9.773
TRMT10B	4.394	4.346	4.382	4.488	4.379	4.374
SLC35E1	9.932	9.906	9.846	9.611	9.978	9.758
SLC35E3	9.390	9.414	9.530	8.865	9.109	9.049
SLC35E4	6.528	6.532	6.829	6.370	6.535	6.488
AC007557.4	4.505	4.544	4.302	4.586	4.961	4.588
MTCO1P12	14.216	14.233	14.197	14.261	14.292	14.177
MYOZ2	5.063	4.918	5.014	5.125	5.069	5.147
MYOZ3	9.363	9.429	9.506	9.508	9.543	9.475
MYOZ1	8.522	8.458	8.655	8.737	8.535	8.706
NAALADL2-AS3	3.855	3.885	3.949	4.044	4.128	4.090
HDAC4-AS1	6.693	6.823	6.918	7.164	6.956	6.977
WRN	8.084	8.233	8.103	7.826	7.685	7.966
AP000919.1	9.896	9.869	9.771	9.942	9.931	10.063
SVOPL	5.598	6.075	5.703	5.972	5.713	5.884
AL031073.1	6.851	6.662	6.436	6.802	6.755	6.824

TCF15	7.987	7.780	8.046	8.485	8.055	7.775
SCNM1	10.124	10.080	10.266	9.961	10.065	10.169
TCF12	10.849	10.829	10.854	10.740	10.691	10.682
TCF19	8.940	9.087	9.132	8.757	8.973	8.730
STRA6LP	6.388	6.361	6.409	6.498	6.510	6.580
AC110597.2	3.786	3.865	4.032	3.915	3.920	3.645
AC117378.1	13.769	13.810	13.830	13.734	13.690	13.742
AC110597.1	3.786	3.865	4.032	3.915	3.920	3.645
UROD	9.525	9.581	9.758	9.350	9.256	9.337
AC007365.1	7.241	7.460	7.501	7.735	7.609	7.541
NXF3	6.566	6.463	6.411	6.555	6.532	6.503
EPPK1	10.047	9.912	9.922	9.415	9.471	9.604
AC079594.2	8.848	8.652	8.969	8.612	8.836	8.085
AC015909.3	8.275	8.258	8.218	8.343	8.433	8.439
HMMR	9.527	9.348	9.532	9.199	9.500	9.302
MCPH1-AS1	5.766	5.865	5.428	5.770	6.002	5.674
PGM1	11.412	11.446	11.613	11.378	11.323	11.435
RPP30	9.523	9.585	9.582	9.523	9.457	9.457
RPRD2	10.712	10.902	10.865	10.803	10.979	10.651
GATA2-AS1	7.536	7.447	7.523	7.631	7.721	7.737
SGIP1	5.829	5.642	5.563	5.847	5.838	5.944
TUBA3FP	8.489	8.753	8.722	8.569	8.659	8.787
ANPEP	8.893	8.722	8.879	8.137	8.057	8.174
C8orf33	9.942	10.028	10.109	9.628	9.700	9.753
C8orf31	6.868	7.048	7.051	6.783	6.886	7.057
C8orf37	7.499	7.797	7.784	7.472	7.503	7.438
C8orf34	4.582	4.638	4.590	4.353	4.702	4.508
KLHL25	8.617	8.681	8.699	8.738	8.717	8.792
KLHL24	8.911	8.828	8.881	8.509	8.763	8.503
KLHL26	8.232	8.364	8.394	8.352	8.236	8.283
KLHL21	8.788	8.778	8.816	8.745	8.668	8.639
KLHL20	8.394	8.379	8.491	8.396	8.461	8.179
KLHL23	9.663	9.846	9.928	9.499	9.641	9.519
KLHL22	8.471	8.511	8.588	8.157	8.311	8.451
TMEM80	8.227	8.200	8.252	8.233	8.222	8.181
SMS	11.051	11.150	11.276	11.034	11.075	11.044
KLHL28	8.840	8.891	8.908	8.843	9.016	8.971
SMO	7.978	8.090	7.842	8.102	7.921	8.100
LTB4R	7.646	7.650	7.609	7.686	7.594	7.758
POLE	9.357	9.338	9.249	8.859	8.997	9.039
POLB	9.950	9.810	10.111	10.129	10.174	10.143
POLN	7.651	7.646	7.522	7.790	7.587	7.764
POLM	8.841	8.843	8.862	8.544	8.610	8.784
POLL	8.055	8.144	8.248	8.052	8.055	8.102
POLK	9.161	9.246	9.281	9.029	9.407	9.206
POLI	7.000	6.776	7.001	6.590	6.617	6.812
POLH	7.429	7.731	7.574	7.561	7.616	7.593
TENT4A	9.137	9.004	9.127	8.831	8.775	8.890
GGN	7.819	7.713	7.866	7.865	7.889	7.836
GGH	8.729	8.730	8.870	8.402	8.704	8.740
IL17D	5.825	5.996	6.247	6.216	5.760	6.093
IL17F	5.641	5.522	5.381	5.533	5.392	5.583
PSMD6	10.504	10.619	10.725	10.496	10.447	10.463
NKAPD1	8.518	8.661	8.586	8.427	8.400	8.470
IL17B	5.791	6.301	6.337	6.283	6.218	6.105
IL17C	8.591	8.530	8.456	8.820	8.624	8.725

CHRDL1	5.800	5.785	5.737	5.676	5.836	6.031
CHRDL2	6.421	6.672	6.717	6.713	6.669	6.754
AL137918.1	4.679	4.459	4.694	4.662	4.568	4.718
DCT	7.269	7.501	7.421	7.371	7.449	7.264
AC005076.2	7.523	7.661	7.537	7.404	7.475	7.748
FAM89A	9.534	9.460	9.528	9.576	9.724	9.350
FAM89B	10.694	10.784	10.895	10.720	10.731	10.773
AC068213.1	6.528	6.460	6.646	6.427	6.585	6.630
C2CD2L	7.746	7.766	7.774	7.800	7.657	7.787
HOXD12	5.812	5.819	5.794	5.708	5.830	5.838
EI24	10.704	10.584	10.619	10.381	10.580	10.505
SGMS2	10.513	10.615	10.475	10.163	10.191	10.323
SGMS1	7.790	7.837	7.853	7.488	7.693	7.856
PRC1	11.499	11.434	11.686	10.927	10.960	10.932
HOXD11	6.713	6.728	6.638	6.999	6.786	6.750
FYB1	7.937	8.195	8.290	8.136	8.093	7.718
FYB2	6.781	7.009	7.146	6.935	7.000	7.180
EPS15	9.820	9.979	10.027	9.801	9.658	9.782
LMCD1	8.808	8.892	8.894	8.664	8.519	8.791
ZNF649	7.511	7.657	7.700	7.569	7.741	7.582
ZNF646	8.389	8.311	8.575	8.676	8.525	8.354
ZNF644	9.581	9.565	9.596	9.553	9.458	9.682
CREB5	7.623	7.913	7.747	7.945	7.953	8.005
TOX2	4.545	4.689	4.558	4.597	4.308	4.748
GTPBP10	8.073	8.026	8.166	8.123	8.168	7.930
AL023773.1	6.333	6.215	6.032	6.465	6.439	6.426
MIS18BP1	7.885	7.868	7.851	7.483	7.366	7.365
IGHD	7.851	7.978	7.995	7.735	8.072	8.006
IGHE	7.639	7.751	7.602	7.515	7.631	7.658
OR1G1	7.724	7.871	7.659	7.825	7.742	7.851
AL590226.1	6.735	6.688	6.989	7.118	7.068	6.901
PRCC	9.017	9.147	9.171	8.932	8.860	9.010
PRCD	7.594	7.747	7.726	7.799	7.892	7.734
ZNF398	8.834	8.721	8.813	8.402	8.567	8.789
ZNF391	7.080	7.426	7.220	7.546	7.448	7.360
PRCP	9.821	9.899	10.003	9.712	9.734	9.689
ZNF394	8.239	8.254	8.226	7.931	8.162	8.162
ZNF395	10.119	10.242	10.446	10.127	10.442	9.810
ZNF396	6.658	6.817	6.665	6.731	6.791	6.928
ZNF397	8.493	8.684	8.842	8.622	8.728	8.226
PACRG-AS3	7.302	7.152	6.937	7.468	7.361	7.215
PACRG-AS2	4.548	4.909	4.796	5.235	4.782	4.829
PACRG-AS1	7.066	7.040	7.039	7.058	7.146	7.028
NUDT22	10.323	10.412	10.578	10.175	10.271	10.250
NUDT21	10.435	10.588	10.737	10.497	10.520	10.484
AC100827.3	7.364	7.509	7.213	7.054	7.237	7.483
POPDC2	5.359	5.327	5.195	5.445	5.348	5.372
POPDC3	8.019	8.101	7.951	7.866	8.045	8.033
AL512380.2	6.850	6.895	7.395	7.717	7.456	6.990
SPIN4	9.532	9.545	9.638	9.079	9.053	9.242
SPIN1	10.746	10.823	10.855	10.446	10.490	10.527
SPIN3	8.299	8.184	8.280	7.973	8.316	8.344
AC009412.1	10.297	10.402	10.494	10.251	10.212	10.183
IFT52	9.077	9.209	9.310	9.268	9.229	9.176
IFT57	8.582	8.606	8.716	8.704	8.752	8.758
LPP	11.580	11.529	11.699	11.292	11.344	11.486

LPO	6.569	6.734	6.684	6.792	6.835	6.890
LPL	6.191	6.272	6.299	6.341	6.442	6.393
AL603825.1	14.596	14.604	14.644	14.671	14.651	14.656
GOLGA2P5	7.911	8.219	8.143	7.791	7.907	7.872
GOLGA2P7	8.875	8.968	8.987	8.777	8.722	8.806
AP005329.2	6.055	6.255	5.974	6.277	6.391	6.248
AP005329.3	7.891	7.793	8.073	7.872	7.939	8.059
PCMTD1P7	8.377	8.182	8.370	8.323	8.144	8.422
CLECL1	5.607	5.594	5.454	5.639	5.603	5.673
AC053503.5	8.663	8.779	8.761	8.608	8.709	8.732
AL137220.1	6.789	6.836	6.606	6.749	6.812	6.745
DDAH1	12.541	12.563	12.662	12.528	12.409	12.538
DDAH2	12.367	12.363	12.578	12.326	12.266	12.305
LINC01800	7.172	7.121	6.831	7.264	7.054	7.154
LINC01806	8.638	8.652	8.792	8.660	8.486	8.774
CRELD2	9.857	9.999	10.024	9.910	9.945	10.027
LINC01808	4.659	4.878	4.740	4.892	4.897	4.812
LINC01809	4.595	4.492	4.284	4.662	4.628	4.491
CENPCP1	7.246	7.183	7.118	6.572	6.792	6.612
ARL4D	7.762	7.952	8.003	8.128	8.039	8.060
ARL4C	10.948	10.880	11.019	10.671	10.798	10.493
GLE1	8.905	9.075	9.068	8.998	9.004	9.030
AC005495.1	4.092	4.160	4.359	4.352	4.264	4.030
JAML	7.779	7.510	7.440	7.812	7.720	7.775
TNPO2	10.385	10.285	10.414	10.069	10.170	10.084
JAM2	6.450	6.700	6.464	6.890	6.713	6.698
JAM3	7.168	7.117	7.087	7.259	7.213	7.151
GALNT8	7.662	7.500	7.675	7.655	7.702	7.547
GALNT9	7.667	7.567	7.583	7.711	7.894	7.641
GALNT4	10.314	10.468	10.328	10.236	10.069	10.370
GALNT5	10.065	10.111	10.022	10.112	10.001	10.026
GALNT6	8.769	8.987	9.080	8.654	8.983	8.550
GALNT7	10.294	10.345	10.308	9.984	10.204	10.043
GALNT1	11.314	11.395	11.535	11.315	11.520	11.052
GALNT2	10.548	10.730	10.820	10.416	10.391	10.474
GALNT3	10.905	11.011	11.020	10.865	10.962	11.018
DMWD	10.010	10.034	10.006	10.182	10.212	10.021
AC003973.1	5.348	5.392	5.305	5.309	5.474	5.435
KIF14	8.114	8.429	8.621	7.815	7.815	7.755
KIF15	7.629	7.569	7.712	7.144	7.149	7.203
AC008627.1	6.817	6.897	6.461	6.510	6.889	6.784
KIF17	6.928	7.168	7.064	7.173	7.232	7.343
KIF11	9.213	9.015	9.228	8.255	8.504	8.531
KIF12	9.076	9.147	9.236	9.100	8.937	8.973
AC104564.5	3.975	4.067	3.951	3.846	4.120	4.191
KIF19	5.239	5.241	5.505	5.132	5.223	5.334
NLRP5	6.955	6.962	7.058	7.068	7.342	7.220
NLRP4	5.489	5.543	5.633	5.812	5.831	5.710
NLRP7	6.107	5.972	6.099	5.954	6.079	6.105
NLRP6	6.102	6.474	6.333	6.266	6.418	6.332
NLRP1	8.588	8.572	8.289	8.436	8.690	8.638
NLRP3	6.570	6.807	6.760	6.871	6.805	6.918
NLRP2	10.756	10.672	10.885	10.729	10.631	10.711
LINC00265-2P	10.722	10.564	10.478	10.430	10.307	10.610
PSEN2	8.852	8.836	8.864	8.716	9.046	8.738
PSEN1	10.276	10.333	10.338	10.220	10.191	10.255

NLRP9	7.230	7.402	7.097	7.154	7.098	7.283
NLRP8	7.749	7.962	7.727	7.760	7.899	7.590
WDR26	10.935	11.115	11.085	11.048	11.010	11.028
WDR27	7.733	7.653	7.553	7.747	7.681	7.698
WDR24	8.827	9.055	9.171	8.915	8.962	8.810
WDR25	8.058	8.046	8.202	8.085	8.053	8.093
WDR20	8.012	8.086	8.245	7.925	7.974	7.979
MROH2A	5.938	6.031	6.015	6.681	6.400	6.377
LINC00869	9.525	9.596	9.783	9.541	9.506	9.445
LINC00868	7.822	7.897	7.790	7.729	7.880	7.716
LINC00863	8.201	8.293	8.364	7.882	7.621	7.987
LINC00862	4.739	4.635	4.621	4.511	4.547	4.558
NAGLU	9.625	9.695	9.787	9.429	9.265	9.437
ASCL1	7.662	7.606	7.326	7.779	7.596	7.826
LINC00867	6.121	6.105	6.018	6.150	6.183	6.116
LINC00865	6.636	6.744	6.554	6.652	6.704	6.647
ASCL5	6.248	6.279	6.053	6.381	6.214	6.059
IGF2BP1	8.160	8.082	8.039	8.093	8.354	8.089
IGF2BP3	11.093	11.069	11.035	10.846	11.082	10.932
IGF2BP2	11.019	10.911	10.976	10.987	11.126	10.841
CD101	6.553	6.456	6.251	6.373	6.492	6.590
KIF1A	11.503	11.595	11.663	11.331	11.139	11.341
KIF1B	9.118	9.068	9.093	8.690	8.679	8.770
KIF1C	8.786	8.622	8.651	8.817	8.916	8.610
CD109	6.829	6.959	7.087	6.930	6.938	6.919
AC109449.1	5.663	5.607	5.791	5.763	5.794	5.580
ZFP36	10.990	11.032	10.941	11.429	11.286	11.304
ZFP37	6.321	6.431	6.396	6.428	6.374	6.146
ZFP30	5.338	5.536	5.632	5.566	5.489	5.469
AC139426.2	7.535	7.744	7.432	7.615	7.672	7.655
DKK2	4.226	4.271	4.217	4.053	4.367	4.155
NLRP14	6.642	6.529	6.707	6.700	6.704	6.620
NLRP11	5.582	5.703	5.719	5.695	5.694	5.887
NLRP10	5.897	5.792	5.691	5.801	5.819	5.749
NLRP13	7.118	7.020	7.115	7.129	7.189	7.051
NLRP12	8.267	8.432	8.420	8.345	8.405	8.472
DKK1	12.334	12.434	12.433	12.349	12.307	12.144
LINC00933	4.875	4.862	4.754	4.792	4.714	5.084
LOXL1	7.628	7.791	7.933	7.644	7.811	7.487
LOXL2	8.574	8.645	8.654	8.640	8.784	8.801
LOXL3	7.936	8.028	7.989	7.958	7.843	7.863
LOXL4	8.058	8.203	8.117	8.019	8.240	8.173
SPATC1L	7.660	7.599	7.763	7.311	7.514	7.423
VIPR2	7.729	7.639	7.604	7.842	7.743	7.838
VIPR1	8.956	9.131	9.161	8.909	9.077	9.102
DIRC1	4.146	4.082	4.136	4.246	4.125	4.077
DIRC3	6.428	6.488	6.763	6.607	6.718	6.662
CES4A	8.344	8.311	8.267	8.502	8.567	8.429
FAM207A	8.448	8.390	8.292	8.193	8.363	8.375
CACNG8	8.261	8.228	8.383	8.313	8.271	8.203
PRLR	6.831	6.829	6.738	6.969	6.908	6.830
DEFB129	4.977	4.854	4.926	5.150	5.054	5.103
DEFB127	5.305	5.443	5.608	5.793	5.776	5.555
DEFB126	6.598	6.529	6.446	6.177	6.576	6.517
DEFB125	5.224	5.257	5.053	5.330	5.345	5.047
DEFB124	9.474	9.622	9.608	9.475	9.734	9.718

DEFB123	7.110	7.122	7.099	6.898	7.196	7.273
DEFB122	4.666	4.595	4.512	4.148	4.927	4.772
DEFB121	6.278	6.370	6.332	6.209	6.492	6.360
CACNG7	5.993	6.131	6.122	5.985	6.261	6.124
AC021106.3	5.318	4.953	4.614	5.117	5.183	4.915
RANBP10	8.642	8.574	8.643	8.235	8.387	8.348
RANBP17	9.249	9.064	9.066	8.942	9.027	9.009
SELENBP1	6.872	6.875	7.136	6.873	6.924	7.054
MIR4453HG	7.207	7.133	7.052	6.986	7.171	7.040
PRLH	7.821	7.754	7.827	8.122	7.974	8.007
AC007485.2	6.710	6.675	6.589	6.342	6.525	6.589
NSFL1C	10.394	10.433	10.463	10.151	10.306	10.369
ZNF542P	4.684	4.934	4.554	4.630	4.559	4.690
LAS1L	9.987	10.131	10.133	9.748	9.811	9.822
TRIM74	9.228	9.178	8.973	9.098	9.150	9.334
SLC25A22	8.864	8.966	9.126	8.873	8.893	8.880
SLC25A23	10.031	9.867	10.027	9.716	9.930	9.839
SLC25A20	8.021	8.006	8.167	7.807	8.006	8.077
SLC25A21	5.017	5.279	5.249	4.983	5.205	5.268
SLC25A26	8.735	8.924	8.913	8.626	8.667	8.710
SLC25A27	5.410	5.463	5.198	5.499	5.608	5.577
SLC25A24	11.371	11.421	11.377	11.048	11.099	11.186
SLC25A25	8.882	9.035	9.087	9.016	8.953	8.862
LINC02297	3.504	3.628	3.499	3.603	3.486	3.514
SLC25A28	9.047	9.108	9.242	9.075	9.082	8.978
SLC25A29	8.523	8.623	8.847	8.631	8.628	8.594
TMEM72	6.773	6.899	6.999	6.998	6.976	6.971
FLNB-AS1	7.074	7.022	6.971	7.147	7.088	7.320
AC093867.1	4.910	4.908	4.681	5.059	4.911	5.172
TMEM75	5.188	5.049	4.980	5.053	5.237	5.178
COLCA1	6.153	6.170	5.988	6.218	6.158	6.204
COPB1	11.801	11.848	11.869	11.912	11.842	11.749
COPB2	11.013	11.046	11.088	11.121	10.902	11.102
PRAMEF29P	7.406	7.569	7.636	7.983	7.893	7.730
ZBBX	4.264	4.319	4.207	4.317	4.286	4.254
MKS1	8.332	8.300	8.418	8.210	8.260	8.317
CNN2P10	7.342	7.213	7.471	7.552	7.438	7.430
LINC02838	7.801	7.780	7.779	7.890	7.810	7.955
CADM2-AS1	3.890	3.905	3.891	3.857	3.877	3.780
LINC02832	8.345	8.408	8.150	8.384	8.513	8.454
LINC02833	5.761	5.992	5.932	6.010	5.885	6.421
LINC02837	7.972	8.027	7.789	7.841	7.954	7.964
GLB1L	8.792	8.666	8.707	8.858	8.630	8.770
AC270298.1	6.726	6.645	6.542	6.858	6.869	6.738
AC073332.1	8.066	8.417	8.401	8.239	8.531	8.292
FBXL12	9.041	9.108	9.292	8.934	9.133	9.028
AC018450.1	7.404	7.460	7.213	7.325	7.317	7.390
MAGEC1	6.207	6.083	6.027	6.083	6.201	6.258
MAGEC3	5.255	5.341	5.164	5.184	5.358	5.335
MAGEC2	6.728	6.621	6.707	6.672	6.909	6.788
AC093110.1	5.177	5.175	5.190	5.290	5.392	5.376
PSTPIP2	4.506	4.584	4.367	4.158	4.349	4.526
PSTPIP1	6.447	6.467	6.593	6.424	6.767	6.632
DIPK1C	8.292	8.336	8.226	8.308	8.345	8.433
DIPK1B	7.368	7.389	7.622	7.763	7.472	7.442
DIPK1A	8.475	8.388	8.468	8.192	8.269	8.378

POFUT1	8.898	8.795	8.985	8.752	8.757	8.677
SNX17	10.472	10.517	10.661	10.468	10.380	10.449
SNX16	7.677	7.500	7.526	7.448	7.412	7.575
SNX15	8.680	8.823	8.965	8.818	8.841	8.780
SNX14	9.145	9.183	9.156	9.114	9.001	9.091
SNX13	9.941	10.005	10.069	9.790	9.789	9.973
SNX12	10.553	10.653	10.769	10.540	10.495	10.471
SNX11	9.996	10.087	10.215	9.942	10.104	10.055
SNX10	9.695	9.711	9.845	9.624	9.670	9.759
SNX19	9.413	9.375	9.154	9.293	9.241	9.302
SNX18	8.893	8.954	9.151	8.775	8.811	9.037
ICAM5	6.383	6.471	6.552	6.652	6.795	6.570
RPL13P12	14.391	14.386	14.416	14.405	14.340	14.364
ICAM3	8.053	8.208	8.202	8.095	8.087	8.042
ICAM2	7.123	7.246	7.554	7.563	7.280	7.450
ICAM1	9.937	9.858	9.875	9.767	10.033	10.086
AC096746.1	3.942	3.664	3.932	3.859	3.791	3.798
CTDSP1	10.230	10.344	10.446	10.237	10.180	10.065
CTDSP2	11.329	11.355	11.410	11.249	11.089	11.277
CTDSPL	10.234	10.247	10.389	10.036	9.982	10.100
FBXL14	5.833	5.743	5.833	5.572	5.716	5.854
BRSK2	7.067	7.161	7.049	7.074	7.287	7.322
SMTN	8.360	8.362	8.596	8.253	8.192	8.141
TMEM87B	9.152	9.191	9.078	8.866	9.076	9.061
TMEM87A	10.346	10.295	10.416	10.366	10.382	10.366
NEUROD2	7.304	7.275	7.532	7.454	7.380	7.449
NEUROD1	6.238	6.256	6.286	6.523	6.187	6.251
SGK3	7.873	7.759	7.919	7.935	7.704	7.657
SGK2	9.816	9.746	9.911	9.727	9.848	9.751
NEUROD4	6.703	6.628	6.711	6.701	6.893	6.836
MIR34AHG	7.080	7.280	7.237	7.249	7.505	7.432
BOD1L2	6.716	6.868	6.783	6.993	7.051	7.016
BOD1L1	8.773	8.462	8.508	8.216	8.318	8.277
FO681492.1	9.453	9.439	9.344	9.561	9.621	9.575
SLC3A2	10.518	10.533	10.699	10.494	10.460	10.429
LINC00472	7.149	7.082	7.107	6.933	6.961	7.004
FO681492.2	9.453	9.439	9.344	9.561	9.621	9.575
LINC00474	5.844	5.787	5.797	6.093	5.991	5.867
LINC00475	7.750	7.676	7.638	8.126	7.839	7.680
LINC00476	6.301	6.345	6.301	6.616	6.612	6.519
LINC00477	4.914	4.916	4.903	5.005	4.964	4.876
LINC00479	7.280	7.431	7.094	7.143	7.433	7.410
MAP4K1	7.935	7.947	7.993	8.026	8.038	7.852
MAP4K3	9.305	9.525	9.310	9.086	9.034	9.253
MAP4K2	7.937	7.663	7.925	7.822	7.865	7.791
MAP4K5	9.056	9.130	9.153	9.225	9.099	9.250
MAP4K4	9.444	9.651	9.751	9.469	9.400	9.433
TRIM71	5.234	5.394	5.015	5.499	4.996	5.206
GPD1	7.392	7.110	7.249	7.257	7.597	7.286
AL136164.3	5.882	5.885	5.758	5.691	5.740	5.613
GPD2	10.311	10.413	10.406	10.099	10.016	10.187
TCEA1P4	6.088	6.245	6.389	6.081	6.224	6.415
ZNF204P	5.512	5.436	5.610	5.512	5.329	5.506
MICU3	5.750	5.903	5.870	5.416	5.742	5.759
MICU2	9.628	9.687	9.734	9.481	9.446	9.569
MICU1	10.442	10.544	10.663	10.461	10.374	10.405

AL138826.1	7.920	7.957	8.011	7.798	7.883	7.846
NEFM	5.121	4.987	5.545	5.674	5.480	5.339
NEFL	10.547	10.698	10.662	10.389	10.342	10.316
NEFH	5.068	5.008	5.128	5.535	5.220	5.118
NRG1	8.572	8.679	8.655	8.137	8.381	8.172
NRG3	7.586	7.438	7.518	7.134	7.107	7.235
NRG2	7.499	7.265	7.679	7.764	7.853	7.710
NRG4	4.558	4.613	4.365	4.891	4.732	4.491
AL354822.1	6.703	6.844	6.692	6.574	6.447	6.610
STING1	8.804	8.915	8.865	8.723	8.718	8.699
NRGN	8.060	8.082	7.812	7.686	7.954	7.817
EIF4EBP2	9.357	9.327	9.431	9.038	9.047	9.142
TFCP2L1	9.596	9.691	9.806	9.466	9.495	9.565
NUMB	9.393	9.400	9.375	9.235	9.013	9.140
SBNO1-AS1	6.435	6.332	6.695	6.724	6.733	6.595
CORIN	6.753	7.082	6.751	6.831	7.015	7.122
AC004461.3	6.763	6.722	6.645	6.673	6.623	6.757
PSAT1	10.847	10.802	10.745	10.616	10.624	10.617
TFCP2	8.939	8.858	9.079	8.735	8.793	8.915
PASD1	6.434	6.592	6.603	6.728	6.719	6.795
SLFN5	10.308	10.194	10.212	10.161	10.511	9.644
RBM15B	9.665	9.608	9.756	9.623	9.661	9.546
SPINT2	12.768	12.850	12.969	12.802	12.583	12.772
SPINT3	6.949	6.780	6.826	7.041	7.073	7.084
SPINT1	10.406	10.507	10.580	10.362	10.247	10.413
YBEY	8.817	8.979	9.069	8.971	8.959	8.896
MMADHC	11.801	11.926	11.989	11.965	11.781	12.054
KHK	7.585	7.725	7.915	7.656	7.654	7.701
CT83	9.684	9.699	9.863	9.763	9.691	9.747
OOEP	8.172	8.394	8.409	8.032	8.245	8.258
DNAJA1P4	5.642	5.393	5.291	5.576	5.684	5.493
HELZ2	10.082	10.108	10.207	10.123	10.526	10.032
NXN	7.115	7.262	7.205	7.327	7.002	7.253
FANCD2OS	7.789	7.746	7.678	7.756	8.050	7.894
CASP16P	6.147	6.545	6.504	6.856	6.810	6.605
ZC3H11B	11.002	10.908	10.983	10.739	10.665	10.802
EPSTI1	9.129	9.011	9.075	9.213	9.353	9.052
PPIP5K2	8.735	8.640	8.721	8.571	8.725	8.734
PPIP5K1	7.909	8.049	8.054	7.715	7.770	7.993
OFCC1	7.276	7.356	7.289	7.314	7.219	7.327
AL121594.1	11.735	11.823	11.915	11.911	11.825	11.883
AL451136.1	9.324	9.250	9.263	9.165	9.112	9.078
OTX1	7.773	7.649	7.405	7.684	7.711	7.492
OTX2	4.679	4.664	4.642	4.890	4.560	4.784
ZFC3H1	8.783	8.704	8.753	8.658	8.584	8.745
YEATS4	8.313	8.612	8.520	8.129	8.327	8.454
STAG2	11.122	11.061	11.183	10.899	10.725	10.778
STAG3	8.699	8.668	8.460	8.757	8.746	8.615
STAG1	9.160	9.149	9.164	8.992	9.005	9.054
ASMTL	8.186	8.251	8.391	7.806	7.968	7.768
SHISA8	5.532	5.489	5.419	5.487	5.381	5.623
IGKV1OR-3	8.398	8.520	8.424	8.478	8.571	8.522
IGKV1OR-2	8.398	8.520	8.424	8.478	8.571	8.522
AL049766.2	10.936	11.054	11.301	11.263	11.275	11.341
HMGA2	10.561	10.657	10.640	10.321	10.208	10.386
HMGA1	12.639	12.748	12.770	12.645	12.461	12.549

VSTM2A-OT1	4.251	4.330	4.419	4.511	4.369	4.586
RPL15P14	12.772	12.801	12.947	12.791	12.625	12.834
TAF1A-AS1	6.619	6.840	6.751	6.684	6.650	6.675
AC068631.1	5.817	5.863	5.991	5.892	5.611	5.874
AC068631.3	11.618	11.736	11.872	11.770	11.790	11.640
AL353803.4	6.957	6.803	6.674	7.017	6.944	6.918
HSPB2-C11orf52	8.371	8.462	8.565	8.135	8.220	8.315
AC138811.2	13.687	13.656	13.782	13.960	13.843	13.987
AC135721.1	11.925	11.994	12.146	11.893	11.885	11.984
HAX1	11.076	11.102	11.271	11.115	11.195	11.017
AC108206.2	6.309	6.590	6.348	6.306	6.370	6.608
C8orf76	7.461	7.541	7.784	7.675	7.673	7.833
GGT4P	9.637	9.701	9.818	9.612	9.546	9.572
UPF3B	9.268	9.170	9.268	8.765	8.846	8.835
LCA5L	6.934	7.027	6.886	6.919	6.870	6.750
AC093827.5	4.426	4.567	4.671	4.366	4.613	4.492
C6orf223	10.049	9.976	10.088	10.061	10.102	9.981
C6orf226	9.080	9.197	9.171	9.135	8.955	9.035
PLS1	12.271	12.404	12.389	12.446	12.400	12.423
PLS3	12.762	12.817	12.849	12.743	12.660	12.764
CLASP2	8.413	8.285	8.354	8.183	8.115	8.363
CLASP1	8.831	8.936	8.942	8.573	8.636	8.841
HMGB4	5.730	5.645	5.662	5.539	5.755	5.686
BMERB1	7.607	7.704	7.809	7.376	7.566	7.435
FABP5P11	10.336	10.387	10.611	10.215	10.289	10.177
C5orf58	7.337	7.520	7.367	7.565	7.655	7.601
C5orf51	8.939	9.078	8.943	8.715	8.563	8.839
SSPN	8.527	8.415	8.533	8.402	8.452	8.416
PGM2L1	8.420	8.473	8.546	8.724	8.582	8.354
NELFB	9.591	9.725	9.934	9.366	9.545	9.482
NELFA	8.741	8.872	8.819	8.439	8.366	8.541
TYW1B	8.956	8.968	8.766	8.802	8.816	8.952
NELFE	10.157	10.128	10.422	10.108	10.089	10.119
CD226	5.242	4.946	4.971	5.308	5.183	5.266
AC009126.1	8.012	8.302	7.863	7.923	8.022	8.048
ECI2-DT	6.119	6.581	6.295	6.518	6.159	6.509
AC131902.1	7.189	7.126	6.924	7.502	7.257	7.337
IRX5	6.386	6.475	6.758	6.481	6.536	6.423
IRX4	6.423	6.569	6.448	6.659	6.696	6.745
IRX1	6.228	6.177	6.337	6.237	6.263	6.335
IRX3	6.775	6.965	6.889	6.830	6.636	6.713
IRX2	5.719	5.702	5.634	5.828	5.823	5.705
TCAP	8.869	8.831	9.042	9.007	8.784	8.999
MCM3AP-AS1	7.282	7.282	7.031	6.833	7.074	7.099
MIR6885	9.559	9.540	9.779	9.272	9.588	9.315
MIR6887	12.242	12.299	12.453	12.393	12.433	12.434
MIR6881	7.650	7.663	7.853	7.895	8.151	7.765
MIR6883	7.033	6.990	7.030	7.045	7.047	7.076
AC006230.1	7.966	7.875	7.567	7.628	7.419	8.044
CLEC2L	7.181	7.326	7.280	7.420	7.228	7.466
CLEC2B	4.353	4.666	4.622	4.469	4.730	4.751
CLEC2D	13.203	13.272	13.262	13.249	13.199	13.294
CKMT2	6.019	5.917	5.801	5.919	5.984	6.025
C11orf54	8.346	8.378	8.495	8.156	8.345	8.332
C10orf25	6.399	6.484	5.912	6.447	6.348	6.584
FP325317.1	8.108	8.089	8.007	7.989	8.099	8.301

FP325317.2	8.464	8.364	8.135	8.347	8.305	8.294
HACL1	7.927	7.889	7.809	7.577	7.674	7.659
IQSEC1	8.004	7.954	7.986	7.927	7.966	7.997
APOA5	8.700	8.740	8.524	8.581	8.769	8.743
IQSEC3	6.752	6.877	7.004	7.087	6.968	7.080
IQSEC2	8.181	8.024	8.171	8.121	8.320	8.211
GPRASP2	9.036	8.978	9.038	8.809	8.871	8.832
APOA1	7.474	7.562	7.683	7.632	7.770	7.833
APOA2	5.881	5.837	5.697	5.696	5.812	5.731
GPRASP1	6.713	6.677	6.253	6.174	6.521	6.624
XPO1	11.899	11.848	11.938	11.750	11.530	11.755
AC084879.2	6.905	7.008	7.166	7.166	6.979	7.144
XPO5	9.391	9.337	9.431	9.220	9.241	9.295
AC090517.5	8.544	8.527	8.313	8.403	8.397	8.435
XPO7	10.521	10.568	10.543	10.442	10.369	10.356
XPO6	10.303	10.351	10.467	10.165	10.215	10.314
XPOT	10.848	10.876	10.865	10.631	10.750	10.741
TNRC18P2	7.107	7.148	7.327	7.098	7.192	7.031
TNRC18P3	7.107	7.148	7.327	7.098	7.192	7.031
ZFYVE19	7.649	7.764	7.740	7.614	7.671	7.743
CHCHD3P3	10.602	10.631	10.641	10.581	10.582	10.530
INHBA-AS1	7.180	7.090	7.162	7.010	7.179	7.113
PLCH1-AS1	7.364	7.392	7.102	7.236	7.568	7.266
MINDY4	8.636	8.629	8.771	8.580	8.591	8.622
MINDY1	8.899	8.965	9.011	8.839	8.970	8.817
GIT1	9.934	10.026	10.213	10.027	9.963	9.999
MINDY3	8.066	8.238	8.099	8.018	8.178	8.086
MINDY2	10.127	10.090	10.128	10.018	9.925	10.241
DCST2	5.766	5.563	5.664	6.023	5.867	5.690
DCST1	7.377	7.505	7.093	7.324	7.269	7.400
GHRH	6.461	6.108	6.606	6.380	6.508	6.757
SESN1	7.383	7.488	7.449	7.134	7.284	7.429
SESN2	9.802	9.755	10.015	9.723	9.912	9.514
SESN3	7.070	6.757	7.070	6.654	6.735	7.065
AP004608.1	8.045	8.160	8.140	8.286	8.333	8.173
LINC01103	5.740	5.817	5.439	5.772	5.722	5.633
LINC01102	5.137	5.100	5.048	5.234	5.023	5.070
LINC01101	7.220	7.255	7.281	7.404	7.281	7.412
LINC01107	7.154	7.232	7.206	7.458	7.353	7.322
LINC01106	7.940	7.954	8.122	7.953	7.938	7.880
LINC01104	5.731	5.545	5.984	5.749	5.986	5.841
AC006058.1	6.548	6.256	6.153	6.512	6.381	6.547
AC006058.2	5.797	5.944	5.954	5.946	6.021	6.045
SPARC	5.272	5.028	5.074	5.145	5.226	5.103
HTR7P1	5.743	5.832	5.493	6.021	5.797	5.756
DBNDD2	11.289	11.380	11.504	11.240	11.202	11.234
DBNDD1	8.926	8.996	9.247	9.027	9.117	8.941
NFRKB	9.104	9.131	9.125	9.043	8.973	8.961
DBET	8.052	7.820	8.258	8.487	8.319	8.298
GRTP1-AS1	8.088	8.124	8.081	8.092	8.034	8.257
CD96	5.662	5.928	5.503	5.867	5.827	5.876
LKAAEAR1	7.786	7.937	8.138	8.002	8.012	7.921
NPC1L1	9.834	9.810	9.934	9.598	9.568	9.694
MIR3939	5.318	5.029	5.070	5.100	4.892	4.812
AC010980.2	6.229	6.291	6.309	5.463	6.035	6.080
OR6B1	6.327	6.497	6.290	6.400	6.469	6.166

NATD1	7.478	7.573	7.491	7.467	7.568	7.494
NFX1	9.006	9.082	9.195	8.825	8.848	9.004
LINC00271	5.505	5.527	5.643	5.957	5.688	5.655
SLIT3	7.820	7.797	7.707	7.925	7.728	7.793
SLIT2	7.573	7.455	7.646	7.546	7.622	7.508
SLIT1	7.756	7.665	7.508	7.676	7.673	7.604
SEL1L3	11.141	11.275	11.309	11.078	11.082	11.097
NOLC1	10.889	10.870	10.975	10.741	10.575	10.691
MYRIP	6.244	6.346	6.514	5.946	5.783	5.977
NBN	10.329	10.402	10.488	10.311	10.345	10.235
ARRDC3-AS1	7.939	8.025	7.818	7.956	7.751	7.991
H2BC21	8.739	8.775	8.708	8.828	8.678	8.783
AC012615.6	6.384	6.366	6.249	6.181	6.237	6.264
AC012615.5	5.641	5.808	5.862	6.057	5.892	5.912
CYTOR	11.086	11.192	11.341	11.191	11.247	11.349
AC027309.3	4.326	4.478	4.353	4.371	4.394	4.413
AC027309.2	12.359	12.429	12.496	12.306	12.282	12.215
AC087632.2	7.833	7.815	7.709	7.778	7.857	7.535
AC002456.2	4.860	4.940	5.014	4.821	4.770	4.986
AL592114.3	6.651	6.711	6.689	6.697	6.805	6.949
RGS4	7.233	7.420	7.694	7.730	7.495	7.687
AC100832.2	3.995	3.902	3.861	4.064	4.231	3.874
AP000777.3	7.458	7.611	7.589	7.701	7.850	7.572
RGS7	5.191	5.046	4.810	4.687	5.099	4.917
AL139348.1	6.337	6.538	6.355	6.636	6.515	6.573
CIRBP	10.330	10.296	10.484	9.904	9.711	9.869
DPY30	11.188	11.286	11.388	11.271	11.248	11.259
TBL2	9.667	9.823	9.737	9.588	9.787	9.642
TBL3	9.324	9.262	9.360	8.990	8.952	9.115
PDE8A	10.696	10.652	10.789	10.612	10.602	10.702
PDE8B	6.715	6.664	6.170	6.394	6.511	6.456
DBI	12.972	13.081	13.089	13.112	12.996	13.025
DBH	7.144	7.213	7.350	7.587	7.445	7.356
MANBA	8.788	8.840	9.018	8.679	8.751	8.709
OR7E14P	9.921	9.936	10.016	9.497	9.614	9.615
DBP	7.871	7.895	8.112	7.829	7.879	7.867
CTNNA1P1	5.815	5.538	5.631	5.436	5.666	5.490
TAF5L	9.030	9.044	9.029	9.022	9.166	9.049
DBT	10.610	10.606	10.587	10.303	10.457	10.479
ASTE1	8.509	8.520	8.609	8.412	8.672	8.425
MED1	11.912	11.975	12.040	11.770	11.875	11.801
MED6	9.397	9.570	9.620	9.312	9.364	9.385
MED7	9.501	9.409	9.481	9.244	9.112	9.271
MED4	8.847	8.805	9.030	8.451	8.675	8.680
MED8	9.029	9.026	9.084	8.882	8.899	8.969
MED9	8.054	8.107	8.230	8.340	8.157	8.240
MNX1	8.758	8.744	8.983	8.814	8.917	8.880
AC237298.1	7.926	7.982	8.240	8.057	8.052	7.952
PMS2P10	8.737	8.598	8.642	8.446	8.495	8.529
CCDC90B	8.992	9.002	9.090	8.944	9.149	8.967
AFM	5.647	5.796	5.793	5.492	5.915	5.867
TCF7	8.822	8.866	8.585	8.461	8.411	8.586
TCF3	9.418	9.543	9.664	9.431	9.527	9.380
WNK4	7.313	7.247	7.162	7.085	7.188	7.141
DEPDC1-AS1	4.142	4.365	4.081	4.013	4.275	4.123
AFP	6.580	6.318	6.239	6.503	6.491	6.399

STK32C	8.266	8.317	8.488	8.290	8.332	8.324
STK32B	7.686	7.740	7.533	7.494	7.647	7.788
STK32A	7.801	7.765	8.076	7.640	7.549	7.671
PPP5C	9.404	9.603	9.765	9.423	9.296	9.270
UPF3AP1	7.745	7.820	7.739	7.360	7.302	7.512
RPL17-C18orf32	13.304	13.343	13.490	13.360	13.298	13.417
AC107027.3	6.498	6.650	6.724	6.444	6.433	6.404
ISLR2	7.128	7.050	7.254	7.191	7.519	7.224
MDN1	8.839	8.678	8.657	8.318	8.346	8.570
AMPD3	8.079	8.007	7.929	7.940	8.144	7.912
AMPD2	8.448	8.556	8.612	8.288	8.465	8.361
AMPD1	5.479	5.778	5.482	5.873	6.053	5.697
AF129075.3	7.435	7.614	7.095	7.241	7.508	7.427
AREG	11.933	11.953	12.074	11.703	11.775	11.792
MRGPRX4	6.532	6.556	6.543	6.658	6.526	6.635
UTP11	9.859	9.896	9.987	9.704	9.570	9.660
MRGPRX1	4.858	4.928	5.010	4.856	5.007	4.855
MRGPRX3	5.313	5.294	5.433	5.178	5.341	5.321
MRGPRX2	5.823	6.230	6.120	5.952	6.161	6.107
UTP18	10.982	11.056	11.216	10.907	10.825	10.931
TET1	6.833	6.417	6.324	6.304	6.264	6.300
TET3	8.909	8.745	8.749	8.870	8.925	8.886
TET2	7.593	7.473	7.705	6.995	7.349	7.619
CST3	10.567	10.685	10.875	10.477	10.420	10.516
LINC02318	3.792	3.880	3.929	4.056	4.075	4.029
ANKRD20A9P	8.236	8.156	8.141	8.146	8.143	8.231
TMEM52B	4.072	4.049	4.020	4.029	4.010	3.995
CPNE8-AS1	6.076	5.982	6.034	6.030	6.106	5.925
LINC02314	7.032	6.906	6.860	7.054	7.226	7.151
CST5	6.788	6.778	7.080	6.760	6.926	6.706
PCBD2	7.621	7.778	7.544	7.425	7.486	7.799
PRDX6	12.584	12.635	12.769	12.665	12.619	12.606
PRDX5	12.152	12.259	12.285	11.395	11.368	11.500
PRDX4	12.159	12.259	12.419	12.468	12.275	12.444
PRDX3	11.619	11.609	11.691	11.753	11.797	11.701
PRDX2	10.771	10.823	11.066	10.701	10.683	10.669
PRDX1	13.054	13.107	13.179	13.104	12.922	13.064
AP3S2	10.782	10.833	11.008	10.778	10.738	10.728
AP3S1	11.514	11.538	11.646	11.521	11.500	11.458
AC012354.7	8.631	8.739	8.589	8.681	8.825	8.706
PITX2	3.888	3.814	3.904	3.847	3.809	3.835
PITX3	7.959	7.731	7.992	7.982	8.056	7.970
PITX1	6.043	6.371	6.569	6.339	6.289	6.274
AXIN2	7.586	7.761	7.777	7.847	7.802	7.848
AXIN1	9.990	10.021	10.154	10.059	10.057	9.993
MMRN2	6.140	6.125	5.867	6.311	5.946	6.053
MMRN1	4.194	3.977	4.215	4.208	4.205	4.139
CSTB	13.431	13.536	13.603	13.461	13.481	13.444
RDH10	9.901	9.956	9.893	9.737	9.705	9.671
IGLON5	7.224	7.074	7.061	7.121	7.366	7.209
LINC01669	7.319	7.219	7.358	7.196	7.171	7.432
ABRA	5.355	5.182	5.494	5.330	5.389	5.386
AC004158.1	5.128	5.223	5.075	4.921	5.353	5.421
LINC01667	8.061	8.065	8.074	8.150	8.252	8.090
LINC01664	7.391	7.554	7.518	7.393	7.514	7.618
CD38	7.317	7.443	7.382	7.173	7.464	7.605

TSSK1B	6.220	5.886	6.405	6.090	6.229	6.204
ZFX	8.318	8.201	8.217	8.044	7.992	8.227
ZFR	10.167	10.262	10.249	10.005	9.931	10.150
IFI27	11.727	11.758	11.701	11.656	11.854	11.543
CD33	7.455	7.185	7.173	7.459	7.484	7.442
CD34	8.371	8.604	8.404	8.374	8.379	8.230
CD36	5.204	4.996	4.778	4.974	5.392	5.093
CD37	6.058	5.983	6.150	6.076	5.917	6.268
ZNF321P	5.234	5.167	5.437	5.030	5.429	5.145
OR52K3P	5.653	5.752	5.616	5.770	5.782	5.639
APCDD1	5.806	5.553	5.910	6.090	5.962	6.053
FP671120.6	4.107	4.171	4.060	4.133	4.131	4.177
MGARP	5.351	5.354	5.241	5.427	5.489	5.486
AC005670.2	8.663	8.691	8.820	8.887	8.823	8.740
AC005670.3	9.627	9.805	9.707	9.461	9.563	9.527
R3HDML	7.113	7.331	7.168	7.107	7.363	7.152
PSD2	8.268	8.132	8.156	8.238	8.404	8.218
H1-4	6.366	6.389	6.333	6.370	6.524	6.174
H1-5	4.717	5.046	4.952	5.058	5.155	4.973
H1-2	9.560	9.727	9.948	9.528	9.199	9.544
H1-3	7.238	7.290	7.349	7.108	7.287	7.215
H1-0	12.359	12.405	12.518	12.291	12.072	12.185
PSD4	9.105	9.098	9.144	8.808	8.930	8.828
H1-8	5.926	5.900	5.812	5.817	5.804	5.908
H1-9	6.418	6.449	6.406	6.697	6.440	6.515
R3HDM4	11.291	11.353	11.415	11.204	11.210	11.334
R3HDM1	9.772	9.829	9.717	9.541	9.361	9.441
R3HDM2	10.546	10.482	10.674	10.512	10.382	10.471
BAALC-AS1	5.242	5.211	5.107	5.150	5.249	5.308
BAALC-AS2	7.256	7.525	7.441	7.554	7.266	7.487
ORMDL2	11.571	11.655	11.710	11.748	11.643	11.642
ORMDL3	13.361	13.396	13.522	13.190	13.065	13.151
ORMDL1	10.251	10.282	10.463	10.402	10.434	10.289
AC011755.1	4.146	4.174	4.365	4.126	4.197	4.247
CD3D	7.686	7.643	7.614	7.859	7.821	7.740
CD3E	8.136	8.314	8.201	8.254	8.310	8.260
CD3G	5.893	5.988	5.764	6.114	6.238	6.034
ACADS	8.788	8.920	9.251	8.851	8.817	8.725
AL117378.1	4.291	4.127	4.151	4.156	4.146	4.332
KAT2B	8.076	8.132	8.246	7.747	7.789	8.019
KAT2A	10.000	10.059	10.180	10.145	10.132	10.094
SEC16A	9.771	9.787	9.836	9.493	9.620	9.754
SEC16B	7.472	7.590	7.251	7.495	7.631	7.328
AC022733.2	6.641	6.900	6.897	6.660	6.897	6.878
ACADL	6.508	6.568	6.643	6.775	6.614	6.394
ACADM	9.201	9.149	9.210	8.817	8.832	8.991
SUCO	10.182	10.330	10.306	10.168	10.206	10.125
NFE4	5.688	5.799	5.813	5.838	5.856	5.769
NFE2	5.895	5.788	5.733	6.276	5.825	6.048
MADCAM1	7.808	7.759	7.802	8.087	7.926	8.170
ZC2HC1B	5.967	5.864	6.288	6.427	6.318	6.449
ZC2HC1C	7.275	7.143	7.474	7.339	7.442	7.217
AL049830.3	4.611	4.635	4.770	4.681	4.585	4.596
AC004972.1	4.180	4.025	4.153	4.236	4.085	4.346
AMIGO3	8.819	8.747	8.765	8.921	8.724	8.789
ATOX1	11.046	11.190	11.192	11.106	11.038	11.101

ACAD8	8.923	9.013	9.129	8.673	8.709	8.779
ACAD9	10.382	10.508	10.523	10.255	10.239	10.258
UEVLD	9.221	9.358	9.364	9.143	9.298	9.184
AL109809.4	7.876	8.130	8.081	8.335	8.090	8.039
ADAMTS10	8.063	7.970	8.209	8.508	8.317	8.103
ADAMTS13	8.096	8.170	8.209	8.094	8.113	8.160
ADAMTS12	7.217	7.543	7.375	7.486	7.514	7.577
PLCE1	6.893	6.926	7.107	6.878	6.879	6.882
ADAMTS14	7.184	7.376	7.400	7.496	7.456	7.467
ADAMTS17	6.100	6.126	6.184	6.109	6.266	6.200
ADAMTS16	6.745	6.829	6.839	7.039	6.995	6.943
ADAMTS19	5.295	5.336	5.367	5.265	5.508	5.356
ADAMTS18	7.782	7.769	7.530	7.575	7.645	7.619
AL353579.1	6.327	6.282	6.297	6.611	6.453	6.461
DEF6	7.689	7.849	7.699	7.642	7.597	7.748
DEF8	10.057	10.160	10.216	10.054	9.962	9.955
FAM21FP	6.794	6.863	6.698	6.739	6.816	7.109
LINC01877	6.107	6.192	6.132	6.147	6.326	6.112
ZSCAN29	7.994	7.924	8.126	7.890	8.188	7.910
GALT	8.551	8.706	8.849	8.518	8.500	8.457
AL365232.1	5.291	5.346	5.213	5.178	5.344	5.447
GALP	5.022	5.178	4.913	5.375	5.165	5.454
LINC01875	7.571	7.686	7.634	7.623	7.750	7.841
SORD	10.296	10.383	10.493	10.290	10.272	10.260
GALE	9.860	9.926	10.115	9.894	9.755	9.940
GALC	10.194	10.263	10.168	9.961	9.948	9.950
GALM	9.028	9.380	9.412	9.024	9.122	9.022
CSNK2B	11.996	12.053	12.198	11.947	11.842	11.914
AC134684.6	6.451	6.418	6.257	6.244	6.283	6.307
FCGR3A	4.864	4.666	4.791	4.986	4.652	5.048
FCGR3B	5.902	5.942	6.100	6.051	5.881	5.979
ALDH1A1	5.275	5.241	5.140	5.350	5.176	5.082
ALDH1A2	12.208	12.289	12.427	12.278	12.223	12.165
ALDH1A3	13.089	13.108	13.145	12.994	12.935	13.089
AC270286.1	6.356	6.510	6.566	6.574	6.470	6.666
CD22	8.732	8.739	8.853	8.597	8.718	8.570
AC093010.3	8.153	8.321	8.189	8.331	8.167	8.604
EYA1	6.776	6.961	6.842	6.747	6.925	7.033
TDRD1	5.783	5.917	6.054	6.235	6.246	6.044
EYA3	9.330	9.397	9.330	9.330	9.332	9.354
EYA2	6.899	6.790	6.988	6.929	7.120	6.981
AL162419.1	3.915	3.763	3.637	3.652	3.768	3.471
SMARCB1	10.188	10.252	10.583	10.284	10.187	10.188
PIEZO2	7.589	7.701	7.485	7.532	7.774	7.685
PIEZO1	10.218	10.222	10.352	10.155	9.930	10.080
ZNF891	6.163	6.252	6.125	6.345	6.222	6.463
KRTAP13-1	8.124	8.077	8.137	8.001	8.231	8.187
WFDC11	7.196	7.351	7.387	7.295	7.370	7.392
WFDC13	4.268	4.341	4.431	4.326	4.382	4.317
WFDC12	8.521	8.585	8.456	8.582	8.569	8.592
AC104452.1	8.618	8.583	8.676	8.278	8.456	8.463
AC008162.2	5.635	5.693	5.320	5.666	5.815	5.536
FXYD6	7.312	7.245	7.147	6.930	7.339	7.146
FXYD7	6.814	6.364	6.685	6.852	6.720	6.702
FXYD4	6.955	7.138	7.078	6.897	7.020	6.952
FXYD5	13.371	13.484	13.529	13.459	13.406	13.381

FXYD2	8.943	8.949	9.255	8.995	8.938	8.782
MTBP	5.243	5.436	5.156	5.145	5.229	5.255
FXYD1	7.470	7.756	7.467	7.664	7.743	7.604
CCNQ	8.963	8.967	9.147	9.144	9.041	9.101
MAFK	10.196	10.109	10.127	9.757	10.041	10.060
MAFB	8.066	8.200	8.292	8.230	8.205	8.258
IGFN1	8.040	8.031	7.993	8.339	8.166	8.007
MAFF	11.002	11.086	11.119	10.624	10.759	10.606
MAFG	9.335	9.597	9.600	9.356	9.511	9.422
RBPJ	10.086	9.881	9.913	9.837	9.919	10.014
CCR10	6.510	6.608	6.654	6.627	6.788	6.605
RBP1	7.775	7.633	7.910	7.662	7.739	7.576
RBP2	6.520	6.563	6.730	6.717	6.760	6.577
RBP3	6.507	6.326	6.763	6.702	6.808	6.529
RBP4	6.175	6.217	6.257	6.134	6.436	6.506
RBP5	7.199	6.976	7.092	7.082	7.243	7.233
RBP7	6.432	6.382	6.582	6.750	6.482	6.833
WDR53	6.230	6.366	6.291	6.299	6.210	6.179
TOB1-AS1	6.490	6.691	6.645	6.802	6.840	6.834
MAF1	11.066	11.138	11.333	11.110	11.112	11.004
DBH-AS1	6.949	6.820	6.856	7.057	7.124	7.178
CHTF8	11.422	11.533	11.696	11.388	11.414	11.459
Z98751.2	5.784	5.831	6.062	5.827	5.872	5.780
EIF4G2	12.784	12.864	12.846	12.729	12.654	12.806
ANXA2R	7.756	7.843	7.917	7.811	7.763	7.988
G6PC3	11.202	11.213	11.254	10.899	10.990	10.896
G6PC2	7.657	7.889	7.842	7.699	7.837	7.776
CTIF	7.910	8.061	8.285	8.186	8.024	8.019
CYGB	8.351	8.328	8.121	8.251	8.320	8.348
TSC2	9.939	10.004	10.142	9.782	9.612	9.787
RNF20	9.906	9.945	9.909	9.642	9.576	9.755
TSC1	8.544	8.508	8.591	8.513	8.577	8.509
RCN3	7.679	7.562	7.576	7.554	7.593	7.539
RCN2	10.963	10.994	11.200	10.996	10.904	11.028
RCN1	11.212	11.205	11.261	10.877	10.852	11.173
RHOQ	9.946	9.966	9.953	9.787	10.144	9.767
PSG9	8.150	8.190	8.163	8.147	8.273	8.248
RHOV	7.723	7.856	8.058	8.139	7.769	8.018
RHOU	7.910	7.953	8.217	7.673	7.814	7.849
RHOJ	7.959	7.928	7.813	8.081	8.040	8.132
RHOH	7.961	7.984	7.912	7.986	8.105	8.027
RHOC	11.391	11.479	11.635	11.371	11.340	11.418
RHOB	11.164	11.193	11.381	11.566	11.544	11.485
RHOA	12.743	12.943	12.989	12.878	12.801	12.870
RHOG	10.556	10.640	10.700	10.551	10.515	10.491
RHOF	10.638	10.642	10.820	10.811	10.707	10.600
RHOD	11.160	11.328	11.406	11.138	11.205	11.253
GMPSP1	11.314	11.458	11.665	11.398	11.299	11.297
CIR1	7.982	7.874	7.997	7.914	7.828	7.648
PYROXD2	7.807	7.769	7.952	7.907	7.873	7.960
OGFOD1	9.707	9.868	9.944	9.786	9.867	9.564
OGFOD3	9.424	9.499	9.614	9.017	9.370	9.262
OGFOD2	9.523	9.628	9.763	9.379	9.452	9.481
ZNF488	8.185	8.359	8.282	8.178	8.268	8.197
RASGRF1	6.935	6.836	6.793	6.854	6.939	6.809
RASGRF2	9.005	8.990	8.904	9.002	9.080	8.930

ZNF480	4.521	4.555	4.368	4.511	4.549	4.536
MEIG1	6.347	6.095	6.150	6.547	6.544	6.426
ZNF483	6.576	6.606	6.677	7.176	6.886	6.684
ZNF485	4.230	4.246	4.361	4.205	4.230	4.416
ZNF486	4.590	4.465	4.342	4.447	4.409	4.414
ZNF487	6.025	5.943	5.935	6.036	6.045	6.148
EXOC3L1	7.938	8.085	8.065	7.940	8.100	8.109
NCS1	8.875	8.921	8.817	9.012	8.729	8.947
AC093752.1	4.724	4.658	4.783	4.692	4.771	4.791
PRPF19	10.304	10.312	10.302	10.104	10.276	10.379
PRPF18	8.341	8.385	8.313	7.900	8.010	8.037
HIGD1A	11.575	11.617	11.694	11.671	11.642	11.718
HIGD1B	6.961	7.453	7.399	7.670	7.576	7.491
IDNK	8.546	8.580	8.541	8.431	8.325	8.374
AL445183.3	9.869	10.056	10.044	10.030	10.047	10.037
TRAF1	7.180	7.253	7.212	7.674	7.614	7.779
TRAF2	8.833	8.598	8.911	8.739	8.760	8.725
CPXCR1	4.778	4.772	4.761	4.744	4.657	4.742
LEF1-AS1	5.751	5.479	5.597	5.742	5.821	5.632
AC092376.2	5.686	5.654	5.814	5.877	5.848	5.880
TRAF6	8.966	8.837	8.808	8.674	8.822	8.952
TRAF7	10.112	10.280	10.376	10.286	10.368	10.139
ESD	11.501	11.621	11.789	11.680	11.813	11.564
LINC02667	6.446	6.291	6.290	6.399	6.329	6.239
IGHV4-61	6.990	7.135	7.238	7.185	7.143	7.324
RPL35P8	7.524	7.556	7.699	7.550	7.686	7.900
DSPP	4.391	4.689	4.692	4.860	4.584	4.797
TINAG	9.399	9.519	9.404	9.365	9.363	9.326
AC108449.1	6.080	6.243	5.942	5.914	6.150	6.067
COL27A1	9.652	9.327	9.373	9.521	9.519	9.561
METTL18	8.885	9.043	9.113	8.938	8.831	8.747
MIR374C	6.005	5.634	5.260	5.782	5.731	5.760
SAMMSON	4.282	4.256	4.309	4.258	4.350	4.362
METTL16	9.092	8.996	9.191	8.734	8.619	8.821
METTL17	10.074	10.157	10.190	10.021	10.023	10.187
METTL14	8.077	8.262	8.376	8.121	8.250	7.850
METTL15	8.659	8.786	8.758	8.564	8.674	8.679
NPR1	8.393	8.329	8.451	8.723	8.473	8.467
NPR2	7.909	7.698	7.892	8.049	7.929	8.012
NPR3	7.101	7.107	7.024	7.033	7.100	7.099
SMIM11B	8.357	8.380	8.555	8.248	8.433	8.500
SMIM11A	8.357	8.380	8.555	8.248	8.433	8.500
RPS3AP26	13.488	13.531	13.648	13.817	13.801	13.836
TMEM184A	9.211	9.019	9.251	8.841	9.057	8.890
TMEM184B	10.992	10.959	11.110	10.995	11.015	10.919
TMEM184C	9.102	9.206	9.216	9.060	9.236	8.947
LINC01310	6.807	6.826	6.789	7.104	6.938	7.013
LINC01315	7.544	7.597	7.562	7.544	7.597	7.700
MEF2C-AS1	7.200	7.312	7.382	7.380	7.251	7.245
NUAK1	7.773	7.742	7.824	7.452	7.549	7.502
NUAK2	8.062	8.195	8.096	8.446	8.389	8.515
POLR1A	8.717	8.723	8.558	8.614	8.391	8.516
POLR1B	8.837	8.992	8.868	8.702	8.776	8.648
POLR1C	8.617	8.788	8.813	8.792	8.815	8.789
POLR1D	10.767	10.808	10.988	10.837	10.727	10.813
POLR1E	8.219	8.190	8.260	7.609	8.021	8.005

DUS1L	9.743	9.805	10.035	9.849	9.755	9.811
AC000095.3	7.049	7.195	7.322	7.016	7.141	7.207
CXorf51B	6.785	7.094	6.947	6.942	6.842	7.104
CXorf51A	6.785	7.094	6.947	6.942	6.842	7.104
GRM4	7.963	8.103	8.013	7.856	8.182	8.096
GRM5	9.303	9.163	9.131	8.694	8.733	8.878
GRM6	7.214	7.370	7.212	7.189	7.391	7.318
GRM7	5.802	5.930	5.718	5.992	6.109	6.175
GRM1	6.192	5.911	6.171	6.358	6.264	6.236
NR1H2	9.544	9.595	9.822	9.522	9.484	9.559
GRM3	6.321	6.333	6.099	6.506	6.245	6.479
HOXB8	9.493	9.957	10.082	9.733	9.886	9.349
HOXB9	8.872	8.975	9.069	8.906	8.840	8.846
HOXB2	10.048	10.181	10.282	10.025	10.023	10.004
HOXB3	10.513	10.406	10.468	10.282	10.256	10.299
HOXB1	8.227	8.115	8.117	8.393	8.457	8.180
HOXB6	10.064	10.086	10.149	9.982	10.061	10.083
HOXB7	10.716	10.740	10.767	10.546	10.681	10.723
HOXB4	9.525	9.592	9.502	9.574	9.477	9.512
HOXB5	9.823	9.757	9.717	9.446	9.685	9.580
AL357153.3	9.397	9.570	9.531	9.312	9.364	9.385
AC026304.1	7.800	7.687	7.627	7.615	7.574	7.537
TSPY10	8.003	7.967	8.011	8.092	8.024	8.064
SPDEF	8.917	8.929	9.067	9.065	9.034	8.997
MAGOHB	8.173	8.157	8.084	8.010	8.049	8.056
AC008875.3	8.118	8.153	8.212	8.090	7.992	8.194
CTAG2	6.815	6.772	6.893	7.072	6.972	6.767
TIMM23B-AGAP6	9.686	9.921	9.931	9.645	9.676	9.652
DYRK3	8.044	7.986	8.121	8.085	8.123	8.163
DYRK2	9.638	9.603	9.657	9.398	9.546	9.461
DYRK4	9.101	9.083	9.190	9.183	9.326	9.033
AL157394.1	6.716	6.528	6.534	6.825	6.810	6.767
AL157394.3	5.220	5.419	5.403	5.120	5.276	5.242
AC127502.2	5.407	5.322	5.091	5.184	4.892	5.289
AC127502.1	3.879	3.648	3.709	3.998	3.568	3.769
NR2F6	9.573	9.747	9.960	9.641	9.570	9.636
NR2F1	8.629	8.647	8.723	8.435	8.767	8.643
NR2F2	12.715	12.589	12.773	12.437	12.654	12.480
ELL3	7.744	7.884	7.835	8.132	8.071	8.007
ELL2	9.522	9.605	9.518	9.595	9.667	9.860
CEP44	7.845	7.894	7.759	7.487	7.592	7.778
CEP41	8.291	8.492	8.603	8.198	8.579	8.208
AL590666.2	8.167	8.194	8.140	8.133	8.186	8.168
AL590822.2	5.353	5.162	5.376	5.240	5.343	5.313
AC124798.1	8.124	8.161	8.237	7.990	8.004	7.964
AC233289.1	5.686	5.650	5.654	5.555	5.613	5.958
AC018475.1	12.688	12.701	12.918	12.751	12.591	12.750
HSPE1	11.689	11.785	11.891	11.930	11.936	11.995
CORO2A	9.609	9.665	9.681	9.429	9.565	9.460
EBI3	6.387	6.399	6.343	6.843	6.649	6.771
TTC7B	7.644	7.820	7.806	7.645	7.564	7.728
TTTY14	7.157	6.959	7.254	7.696	7.232	7.169
CTTN	10.794	10.718	10.797	10.617	10.425	10.734
TTTY10	5.462	5.550	5.536	5.359	5.361	5.581
TTTY11	7.380	7.617	7.596	7.546	7.767	7.805
TTTY12	6.495	6.519	6.530	6.097	6.451	6.454

TTTY13	6.977	7.100	6.559	7.153	7.310	7.363
NOXRED1	6.115	6.167	6.432	6.298	6.471	6.573
USP51	7.438	7.613	7.229	7.486	7.378	7.418
EIF2S1	11.597	11.578	11.733	11.727	11.605	11.471
EIF2S3	11.714	11.765	11.813	11.582	11.596	11.514
EIF2S2	12.787	12.754	12.898	12.710	12.626	12.652
MYORG	7.732	7.943	7.824	7.580	7.527	7.716
SELENOS	11.817	11.915	12.007	11.676	11.699	11.727
SELENOP	6.591	6.563	6.568	6.244	6.678	6.330
SELENOW	13.253	13.380	13.401	13.378	13.521	13.475
AC009063.1	4.600	4.560	4.667	4.668	4.670	4.751
SELENOK	10.010	10.074	10.178	10.054	10.072	10.009
SELENOI	9.843	9.952	9.956	9.952	9.799	9.813
SELENOH	11.155	11.150	11.338	11.068	11.044	11.091
SELENOO	8.994	9.146	9.200	9.211	9.241	9.138
SELENON	8.834	8.841	9.060	8.642	8.614	8.868
SELENOM	6.857	7.025	7.094	7.332	7.185	7.246
TMEM222	10.346	10.388	10.478	10.105	10.250	10.234
TMEM223	10.404	10.364	10.616	10.319	10.396	10.373
TMEM220	6.373	6.434	6.415	6.382	6.576	6.403
TMEM221	7.544	7.695	7.656	7.698	7.881	7.728
EFR3A	12.249	12.279	12.306	12.225	12.101	12.254
SELENOF	11.329	11.445	11.380	11.577	11.660	11.605
EFR3B	7.599	7.592	7.418	7.724	7.879	7.792
FAM189B	9.947	10.063	10.115	9.965	9.981	9.949
TPH1	4.978	4.970	5.199	5.170	5.081	4.892
TPH2	4.404	4.261	4.235	4.372	4.427	4.306
AC017048.3	5.312	5.374	5.721	5.348	5.459	5.478
TTTY1B	7.020	6.651	6.774	7.138	6.895	6.895
DLEU2L	5.471	5.564	5.333	5.367	5.569	5.686
AC021355.1	7.553	7.631	7.271	7.544	7.800	7.698
DHTKD1	9.272	9.255	9.268	8.723	8.895	8.826
OSBPL1A	8.901	8.814	8.917	8.287	8.546	8.585
GAMT	9.006	9.015	9.147	9.007	9.047	9.022
DCTPP1	11.886	11.989	12.092	12.158	11.926	12.026
MICD	7.860	7.669	7.728	7.958	8.019	7.945
MICB	9.874	9.858	10.026	9.894	9.879	9.996
MICA	9.412	9.524	9.744	9.582	9.559	9.560
DHRS9	10.606	10.892	10.870	10.775	10.836	10.846
DHRS7	10.766	10.845	10.958	10.906	10.788	10.816
DHRS4	9.786	9.800	9.901	9.508	9.462	9.477
AC233280.17	9.390	9.411	9.316	9.060	9.420	9.381
AC233280.10	9.390	9.411	9.316	9.060	9.420	9.381
DHRS3	10.406	10.317	10.496	10.399	10.085	10.387
AL353644.1	8.286	7.924	8.206	8.188	8.172	8.003
EEF1A1P42	8.037	8.232	8.337	8.225	8.099	8.266
CACUL1	9.940	9.999	10.026	9.714	10.022	9.811
NABP2	10.135	10.132	10.430	10.104	10.381	9.957
MYT1	7.820	7.922	7.873	7.953	7.884	8.007
DHRSX	9.659	9.596	9.709	9.351	9.432	9.399
GHRLOS	7.366	7.378	7.510	7.447	7.561	7.369
TPO	6.423	6.668	6.285	6.337	6.907	6.603
TPR	10.512	10.390	10.568	10.343	10.309	10.143
HRK	7.329	7.283	7.334	7.534	7.471	7.488
SNAI2	4.691	4.529	4.764	4.791	4.598	4.548
SNAI3	5.576	5.550	5.485	5.727	5.607	5.587

HRG	7.198	7.115	6.860	7.101	7.309	7.161
SNAI1	7.319	7.107	7.257	7.330	7.309	7.228
HRC	6.413	6.562	6.471	6.612	6.649	6.634
OSBPL10	9.887	9.940	10.027	9.868	9.765	9.850
OSBPL11	8.734	8.588	8.544	8.594	8.896	8.544
ECHS1	11.052	11.114	11.316	11.008	10.956	11.046
AC005842.1	7.839	7.983	7.813	7.959	7.979	7.876
CT867976.1	6.857	6.969	7.045	6.840	6.973	7.064
AMELX	4.915	5.014	4.885	5.081	5.133	4.820
AMELY	6.058	6.018	6.155	5.949	6.130	6.045
AC003989.1	6.843	6.768	6.742	6.610	6.457	6.926
SHKBP1	9.175	9.035	9.290	8.879	8.961	8.955
EN1	7.577	7.493	7.469	7.598	7.420	7.475
EN2	6.803	6.766	6.506	6.867	6.900	6.756
LINC02502	6.526	6.770	6.624	6.921	6.691	6.763
BLACAT1	8.086	8.043	8.132	7.946	7.925	7.941
LINC02504	8.504	8.505	8.506	8.604	8.835	8.636
AL022069.2	6.142	6.315	6.162	6.054	6.274	6.169
AL022069.3	7.288	7.417	7.380	7.434	7.391	7.498
ENG	7.935	7.890	8.098	7.884	8.048	8.121
KRT40	5.870	6.166	6.115	6.213	6.249	6.128
AL109618.1	11.713	11.782	11.815	11.659	11.938	11.209
LRTM1	7.450	7.458	7.438	7.468	7.607	7.614
AP000349.1	7.266	7.497	7.355	7.077	7.411	7.378
FBRS	8.976	9.062	9.328	9.188	9.170	9.154
TOR2A	7.791	7.814	8.030	7.681	8.010	7.997
CDKAL1	7.668	7.794	7.872	7.610	7.650	7.725
AC092375.2	7.156	7.159	6.911	7.124	6.867	7.203
CYP4B1	7.564	7.833	7.712	7.650	7.645	7.651
GPR75	6.301	5.994	5.918	6.312	6.399	6.323
FAM81B	5.302	5.038	4.941	5.614	5.407	5.291
FLT4	7.450	7.268	7.458	7.622	7.599	7.484
GPR78	7.935	8.175	8.140	8.413	7.842	8.229
FLT1	7.263	7.005	6.967	7.489	7.220	7.302
PODN	7.591	7.535	7.799	7.756	7.827	7.869
SET	12.511	12.534	12.609	12.543	12.693	12.442
VKORC1	11.750	11.798	12.028	11.675	11.577	11.660
AC104809.2	6.866	6.636	6.814	6.935	6.998	6.907
PVALB	6.993	7.017	7.031	7.132	7.047	7.238
SUGP2	8.386	8.252	8.346	8.163	8.274	8.346
SUGP1	9.210	9.215	9.133	8.917	8.932	9.018
AC055822.1	5.584	5.922	5.620	5.673	5.718	5.780
COL25A1	6.743	6.661	6.775	6.654	6.973	6.410
HASPIN	8.010	7.903	7.889	7.969	7.882	7.873
SYNC	5.538	5.563	5.706	5.597	5.508	5.460
ELMOD1	7.867	7.757	7.611	7.822	7.879	7.936
ELMOD3	8.070	8.191	8.174	8.288	8.233	8.141
ELMOD2	9.036	9.032	8.917	9.016	8.694	8.908
AC138776.1	8.236	8.156	8.141	8.146	8.143	8.231
PRKN	5.512	5.280	5.357	5.815	5.471	5.541
CYP17A1	7.056	7.138	7.129	7.175	7.155	7.164
PRKY	7.596	7.750	7.691	7.469	7.430	7.544
PRKX	7.596	7.750	7.691	7.469	7.430	7.544
ELAC1	6.756	7.036	7.190	6.767	6.582	6.612
RPS2P46	14.545	14.545	14.561	14.532	14.532	14.573
AC007406.4	5.542	5.750	5.994	5.817	5.819	5.986

FEM1B	8.950	9.006	9.056	8.787	8.788	8.887
FEM1C	10.360	10.533	10.479	10.388	10.296	10.337
FEM1A	8.609	8.648	8.703	8.497	8.395	8.520
MS4A6A	8.528	8.647	8.382	8.849	8.864	8.765
METTL21C	3.973	4.102	3.998	4.090	4.174	4.117
METTL21A	9.080	9.309	9.191	8.805	9.073	8.780
CYSRT1	8.080	8.046	8.142	8.075	7.988	8.054
OR8B2	4.390	4.395	4.330	4.276	4.410	4.350
PFKFB1	8.299	8.215	8.144	8.153	8.290	8.358
PFKFB3	11.128	11.250	11.180	11.015	10.800	11.068
PFKFB2	8.407	8.438	8.493	8.429	8.390	8.387
PFKFB4	9.008	9.209	9.330	9.108	9.216	9.188
ITPA	9.864	9.985	9.986	9.749	9.848	9.797
SCIN	9.422	9.383	9.440	9.161	9.171	9.351
PPP4R4	6.179	6.349	6.242	6.572	6.272	6.472
AL354733.2	6.882	6.939	6.806	7.018	7.123	7.294
PPP4R1	10.043	10.201	10.149	9.854	9.786	10.001
PPP4R2	9.489	9.495	9.555	9.522	9.334	9.480
TMED10	12.141	12.263	12.396	12.120	12.056	12.183
LTBR	10.473	10.546	10.741	10.441	10.362	10.364
MAP3K8	5.123	5.060	5.287	5.154	5.083	4.981
AC098614.3	9.643	9.716	9.890	9.546	9.756	9.694
MAP3K2	10.976	11.146	11.101	11.015	10.963	10.927
MAP3K3	8.532	8.419	8.518	8.161	8.403	8.462
SLCO6A1	5.629	5.588	5.495	5.475	5.767	5.562
MAP3K1	9.565	9.236	9.301	9.339	9.095	9.673
MAP3K6	7.932	7.855	8.034	8.000	7.837	7.868
MAP3K7	8.599	8.577	8.831	8.691	8.703	8.637
MAP3K4	9.858	9.886	9.972	9.774	9.590	9.752
MAP3K5	9.895	10.019	10.069	9.957	10.212	9.723
RBMY1A1	6.498	6.612	6.640	6.865	6.762	6.723
MARVELD1	9.248	9.199	9.292	9.122	9.237	9.259
MARVELD3	9.499	9.576	9.562	9.322	9.384	9.405
MARVELD2	10.763	10.876	10.919	10.646	10.844	10.553
SMCO3	5.102	5.070	4.800	5.102	5.020	5.109
SMCO1	5.073	4.982	5.060	4.998	5.190	5.280
SMCO4	9.628	9.610	9.791	9.451	9.470	9.338
ZWILCH	10.346	10.320	10.383	10.092	10.267	10.154
SETD9	7.144	7.171	7.410	7.004	6.966	7.195
C14orf178	8.279	8.373	8.383	8.507	8.497	8.354
SETD3	10.040	10.084	10.181	9.991	10.048	10.066
SETD2	9.569	9.456	9.364	9.186	9.280	9.370
SETD5	10.939	10.976	10.947	10.859	10.983	10.893
SETD4	7.706	7.835	7.866	7.677	7.758	7.721
SETD7	10.155	10.182	10.202	10.184	10.071	10.146
SETD6	8.883	8.662	8.835	8.527	8.795	8.397
PSRC1	8.193	8.110	8.415	7.805	8.212	8.016
DZIP3	7.637	7.834	7.825	7.663	8.000	7.765
DZIP1	7.792	7.894	7.971	7.785	7.996	7.874
PRSS27	6.899	6.816	6.558	6.754	7.088	6.952
PRSS21	6.365	5.849	6.240	6.422	6.518	6.240
PRSS22	9.809	9.924	10.008	9.910	9.866	9.690
PRSS23	10.054	10.060	10.114	10.085	9.908	10.069
LXN	10.747	10.772	11.023	10.632	10.503	10.697
AC073130.3	7.134	6.933	7.281	7.281	7.275	7.289
AC073130.1	7.613	7.524	7.453	7.396	7.631	7.651

IGHA2	6.176	6.229	6.543	6.326	6.722	6.304
IGHA1	6.176	6.229	6.543	6.326	6.722	6.304
NUDT4B	9.971	10.062	10.046	9.918	9.987	10.114
VENTX	7.425	7.480	7.490	7.662	7.513	7.548
KRTAP4-9	6.457	6.420	6.207	6.424	6.601	6.439
KRTAP4-8	8.666	8.494	8.147	8.724	8.991	8.793
AL356534.1	6.163	6.204	6.121	5.879	5.988	6.067
KRTAP4-1	6.732	6.803	6.849	6.911	7.066	7.042
KRTAP4-3	5.074	5.209	4.943	5.073	5.170	5.309
KRTAP4-2	7.829	7.849	7.674	8.049	7.887	8.010
KRTAP4-5	7.310	7.316	7.221	7.325	7.711	7.422
KRTAP4-4	5.451	5.579	5.451	5.604	5.814	5.527
KRTAP4-7	4.043	4.198	4.080	4.135	4.131	4.215
KRTAP4-6	7.257	7.364	6.914	7.179	7.428	7.344
RRM2B	7.342	7.312	7.366	7.086	7.035	7.138
AC097173.2	6.526	6.770	6.624	6.921	6.691	6.763
NBPF5P	6.070	6.056	6.077	6.122	6.237	6.143
ZNF451-AS1	4.585	4.511	4.248	4.616	4.539	4.586
AC015912.3	6.141	6.116	6.003	6.184	5.988	6.183
TP53	9.192	9.025	9.330	8.670	8.969	8.765
ST8SIA5	7.313	7.420	7.344	7.200	7.468	7.559
ST8SIA4	5.669	5.550	5.551	5.583	5.832	5.679
ST8SIA3	6.831	6.816	6.726	7.013	6.888	7.108
ST8SIA2	6.310	6.381	6.466	6.487	6.533	6.266
ST8SIA1	4.080	4.222	4.143	4.113	4.209	4.097
FARS2	8.786	8.925	8.788	8.768	8.684	8.733
BTBD19	7.632	7.371	7.142	7.762	7.684	7.566
BTBD17	6.574	6.652	6.784	6.828	6.696	6.913
BTBD16	5.434	5.925	5.777	5.539	5.657	5.723
NLRX1	8.666	8.676	8.794	8.697	8.559	8.768
BTBD10	9.774	9.882	9.926	10.097	9.953	9.992
SRPRB	11.699	11.775	11.900	11.857	11.892	11.764
CTDNEP1	11.018	11.131	11.185	11.157	11.164	11.107
SRPRA	10.637	10.651	10.725	10.642	10.701	10.645
MASTL	8.142	8.140	8.045	7.688	7.725	7.669
AHCYP8	5.474	5.321	5.217	5.170	5.371	5.423
AC132216.1	7.835	7.644	7.352	7.977	7.894	7.863
TPRXL	8.117	8.268	8.074	8.188	8.199	8.260
AL355578.1	4.438	4.722	4.533	4.511	4.601	4.907
WAC	10.835	10.877	10.899	10.864	10.926	10.905
MMUT	9.446	9.387	9.378	9.001	9.461	9.150
MAST2	9.133	9.262	9.246	9.174	9.126	9.063
MAST3	7.697	7.791	7.893	7.670	7.598	7.631
MAST4	8.442	8.234	8.268	8.257	8.330	8.398
CD180	7.636	7.574	7.601	7.703	7.686	7.761
WAS	9.183	9.266	9.188	9.213	9.200	9.202
ECRG4	5.157	5.163	5.167	5.280	5.305	5.234
RBFOX2	10.289	10.144	10.418	10.047	10.073	9.827
GALK2	9.353	9.375	9.510	9.245	9.114	9.187
AC016493.1	4.644	4.689	4.599	4.502	4.505	4.641
FARSB	11.513	11.382	11.563	11.303	11.226	11.367
FARSA	9.416	9.558	9.789	9.417	9.341	9.289
C9orf72	8.002	7.854	7.989	8.022	7.852	8.126
AF196969.1	12.093	12.172	12.338	12.181	12.123	12.200
AC144521.1	5.798	6.038	5.812	5.516	5.881	5.877
C9orf78	8.437	8.264	8.535	8.033	8.217	7.997

IL23A	7.225	7.018	7.246	7.343	7.305	7.261
AC124862.1	7.142	7.228	7.124	7.333	7.408	7.231
IL23R	7.130	7.083	6.994	7.072	7.252	7.256
AC011525.2	5.374	5.395	5.227	5.407	5.652	5.373
AC114737.2	5.936	5.995	5.635	6.292	5.884	6.001
PCDHGA10	11.092	11.102	11.188	11.070	11.043	11.048
PCDHGA11	11.092	11.102	11.188	11.070	11.043	11.048
PCDHGA12	11.092	11.102	11.188	11.070	11.043	11.048
TLN1	8.471	8.510	8.723	8.416	8.732	8.471
RPL23AP32	7.566	7.817	7.775	7.614	7.510	7.872
POLE4	9.452	9.420	9.704	9.202	9.252	9.261
POLE2	9.346	9.448	9.450	9.150	9.128	9.381
STRC	7.456	7.501	7.430	7.814	7.484	7.449
RPL7P1	13.823	13.851	13.913	13.938	13.843	13.938
RPL7P6	13.823	13.851	13.913	13.938	13.843	13.938
RPL7P9	13.823	13.851	13.913	13.938	13.843	13.938
BCL2L12	9.174	9.050	9.311	8.780	8.809	8.878
SYN3	6.588	6.451	6.475	6.700	6.617	6.875
SECISBP2	9.749	9.670	9.691	9.656	9.918	9.518
TMEM167B	9.297	9.323	9.415	9.123	9.278	9.255
TMEM167A	11.463	11.493	11.470	11.328	11.097	11.300
AC016629.3	7.667	7.929	8.164	7.639	7.672	7.716
SNORA16A	8.016	7.882	8.068	7.706	7.838	8.005
SLC27A1	9.968	10.046	10.140	9.757	9.818	9.816
PITRM1	9.392	9.394	9.530	9.205	9.091	9.294
RNASEK	12.049	12.125	12.282	11.981	12.082	12.102
RNASEL	8.409	8.467	8.524	8.465	8.697	8.598
RNASE1	8.648	8.513	8.762	8.629	8.746	8.617
RNASE3	7.340	7.544	7.439	7.593	7.497	7.561
RNASE2	6.050	6.056	6.010	6.242	6.351	6.312
RNASE4	10.035	9.861	10.050	9.641	9.693	9.861
RNASE7	5.624	5.580	5.529	5.603	5.699	5.564
RNASE6	5.839	6.090	5.696	6.100	6.144	5.872
KRTAP17-1	7.634	7.672	7.581	7.527	7.632	7.542
AC241377.4	10.040	10.032	10.324	9.749	9.958	9.735
AC241377.5	4.780	4.822	4.745	4.831	4.874	4.812
CCDC89	3.928	3.845	3.805	3.973	3.797	3.879
SNAP25-AS1	5.744	5.704	5.412	5.709	5.780	5.697
CCDC83	3.971	4.018	3.900	3.962	3.970	3.853
SAXO1	7.399	7.197	7.120	7.201	7.070	7.158
SAXO2	4.183	4.143	4.299	4.300	4.245	4.057
CCDC80	7.425	7.296	7.378	7.601	7.353	7.489
CCDC87	8.298	8.350	8.505	8.256	8.585	8.427
CCDC86	10.287	10.347	10.449	10.269	10.279	10.245
CCDC84	7.949	7.824	7.993	7.922	7.902	8.005
SOD2	9.008	8.940	8.778	8.773	8.621	8.841
ZNF558	8.241	8.102	8.272	8.151	7.995	8.192
ZNF569	6.195	6.126	6.025	6.422	6.270	6.328
ZNF568	5.733	5.526	5.553	5.690	5.557	5.721
ZNF567	7.486	7.497	7.529	7.244	7.527	7.578
ZNF566	6.211	6.460	6.266	5.951	6.108	6.020
ZNF565	7.638	7.625	7.768	7.526	7.410	7.642
ZNF564	7.153	6.994	7.265	7.042	6.991	7.060
ZNF563	5.074	5.303	5.454	5.427	5.221	5.355
ZNF562	8.383	8.288	8.374	8.366	8.606	8.355
ZNF561	7.715	7.514	7.845	7.195	7.356	7.509

ZNF560	4.130	4.149	4.418	3.992	4.351	4.228
HSD3BP1	4.636	4.500	4.425	4.517	4.591	4.570
HSD3BP2	4.908	5.014	4.845	4.704	4.913	5.085
ACTA2-AS1	6.012	5.968	5.944	6.266	5.939	6.224
ACRBP	6.947	6.691	7.036	7.217	7.152	7.001
LINC01432	4.992	5.103	4.882	5.210	5.106	5.305
LINC01431	6.324	6.334	6.323	6.334	6.300	6.574
TOMM20	11.628	11.698	11.777	11.366	11.282	11.456
TOMM22	10.352	10.474	10.657	10.423	10.354	10.428
MIR6792	8.031	8.189	8.306	8.182	8.319	8.121
WDFY3-AS2	6.272	5.978	6.121	5.779	6.082	6.108
FAM161A	8.037	8.037	7.957	7.611	7.989	7.817
FAM161B	9.117	9.130	9.102	8.898	9.129	9.120
STYXL1	10.068	10.170	10.260	10.314	10.271	10.168
AC017002.4	11.086	11.192	11.341	11.191	11.247	11.349
TOP2B	9.898	9.821	9.902	9.503	9.455	9.709
TOP2A	12.805	12.692	12.888	12.301	12.556	12.212
SDAD1	9.612	9.564	9.657	9.507	9.762	9.295
FGF9	6.611	6.606	6.465	6.734	6.705	6.698
FGF8	6.018	5.763	5.736	5.855	5.680	5.777
FGF7	5.375	5.478	5.721	5.375	5.628	5.606
FGF6	8.157	8.320	8.143	8.356	8.271	8.364
FGF5	6.815	6.659	6.484	6.565	6.786	6.698
FGF4	8.082	8.145	8.127	8.311	8.226	8.255
FGF3	5.739	6.061	5.886	6.075	5.940	6.231
FGF2	8.818	8.889	8.868	8.776	8.810	8.747
FGF1	8.211	8.209	8.231	8.211	8.345	8.315
AC034236.2	7.453	7.168	7.348	7.497	7.589	7.386
OLFML2B	6.234	6.235	6.479	6.636	6.423	6.359
OLFML2A	8.011	7.958	7.656	8.248	8.190	8.068
AC034236.1	12.267	12.428	12.443	12.421	12.337	12.473
SDC1	12.713	12.827	12.911	12.640	12.590	12.583
SDC2	7.341	7.424	7.302	7.438	7.335	7.533
SDC3	8.127	8.272	8.252	7.999	8.190	8.067
SDC4	10.569	10.601	10.749	10.242	10.435	10.384
AP001893.1	7.206	6.906	6.970	7.210	7.022	7.098
LINC01297	7.031	6.937	6.926	7.183	7.137	7.205
DEFB1	8.106	8.203	8.438	8.421	8.487	8.465
LINC01293	6.073	6.050	5.943	6.072	6.232	6.049
SGCG	6.198	6.299	6.069	6.326	6.469	6.291
SGCE	8.925	8.959	9.093	8.859	8.744	8.894
SGCD	7.977	7.710	7.731	7.784	7.767	7.831
SGCB	8.325	8.356	8.456	8.062	8.098	8.193
SGCA	5.746	5.757	5.779	6.070	5.822	5.947
SGCZ	4.600	4.612	4.598	4.570	4.795	4.901
SNRNP70	10.714	10.755	10.868	10.486	10.382	10.480
AC011270.2	6.492	6.728	6.328	6.566	6.692	6.714
NKD1	8.478	8.566	8.484	8.558	8.615	8.528
NKD2	7.550	7.676	7.663	7.759	7.799	7.643
AL161911.1	6.535	6.571	6.382	6.818	6.371	6.557
AC100793.4	5.514	5.498	5.516	5.501	5.493	5.522
MIXL1	6.553	6.700	6.595	6.819	6.910	6.873
LRRN4CL	7.001	7.139	6.980	6.943	6.743	6.916
GATA6-AS1	7.315	7.230	7.214	7.300	7.407	7.361
FLACC1	7.030	7.008	7.230	7.228	7.183	7.334
LEP	6.394	6.510	6.435	6.408	6.420	6.499

PCDH1	9.295	9.464	9.624	9.530	9.483	9.462
XYLB	7.322	7.435	7.448	7.286	7.415	7.453
NUMA1	10.317	10.324	10.317	10.174	10.099	10.158
AC138392.1	9.802	9.872	9.827	9.586	9.699	9.748
FO393401.1	6.393	6.644	6.526	6.895	6.682	6.652
PCDH7	7.957	7.924	8.053	8.323	8.431	8.272
IDH3A	9.844	10.016	9.900	9.840	9.639	9.782
IDH3B	10.268	10.321	10.367	10.014	9.977	10.070
C12orf75	12.783	12.898	13.002	12.771	12.861	12.920
IDH3G	10.917	10.952	11.117	10.680	10.579	10.659
C12orf77	4.427	4.428	4.165	4.481	4.503	4.631
DGKQ	8.483	8.550	8.583	8.407	8.257	8.307
TMEM254-AS1	6.626	6.467	6.666	6.695	6.650	6.750
VKORC1L1	8.303	8.183	8.179	7.964	7.955	8.040
DGKK	8.376	8.430	8.100	8.307	8.213	8.307
DGKH	8.049	7.775	7.849	7.514	7.661	7.503
DGKI	5.753	5.702	5.636	6.051	5.721	5.809
DGKB	4.293	4.565	4.107	4.736	4.502	4.407
MIR200C	7.375	7.488	7.617	7.574	7.505	7.727
DGKA	9.001	8.839	8.873	8.898	8.822	8.859
DGKG	7.272	7.371	7.486	7.542	7.638	7.619
DGKD	9.201	9.347	9.273	9.039	9.136	9.089
DGKE	7.443	7.580	7.674	7.472	7.490	7.604
ICK	4.324	4.513	4.399	4.014	4.276	4.481
ZNF169	8.097	8.117	8.042	8.050	8.245	8.118
RTEL1	8.539	8.492	8.553	8.544	8.586	8.557
CR762476.1	7.344	7.403	7.456	7.273	7.478	7.510
TXNDC15	9.192	9.127	9.447	9.127	9.220	9.021
ZNF286A	8.532	8.544	8.447	8.236	8.307	8.424
TXNDC17	11.032	11.113	11.229	11.185	10.996	10.933
TXNDC16	7.988	8.037	8.283	7.230	7.173	7.461
AKR1D1	4.076	4.220	3.783	4.239	4.168	4.035
TXNDC12	11.371	11.395	11.475	11.328	11.457	11.269
C1orf94	7.587	7.539	7.439	7.734	7.667	7.833
DENR	11.601	11.641	11.685	11.479	11.429	11.487
ZNF286B	8.532	8.544	8.447	8.236	8.307	8.424
ZNF160	9.320	9.115	8.858	8.646	9.063	9.209
AC024067.1	6.096	6.001	5.757	5.973	6.075	6.054
IQCB1	8.636	8.759	8.729	8.657	8.664	8.690
ZNF165	6.333	6.504	6.783	6.166	6.224	6.275
AKR1B1	10.839	10.894	10.999	10.690	10.686	10.693
AC106875.1	6.264	6.193	6.200	6.334	6.035	6.061
FOCAD	9.016	8.988	9.019	8.794	8.928	9.000
AC008771.1	6.209	6.348	6.285	6.162	6.064	6.310
EPB41L4A-DT	6.027	5.902	6.253	6.163	6.046	5.863
CNDP1	7.424	7.715	7.479	7.410	7.659	7.621
CNDP2	11.531	11.576	11.636	11.393	11.333	11.445
NPL	7.033	7.154	7.157	7.196	7.253	7.229
STRBP	9.042	9.094	9.077	8.779	8.939	8.922
NPB	10.274	10.345	10.584	10.108	10.245	10.216
GADD45GIP1	9.264	9.215	9.485	9.049	9.099	9.112
NPY	7.669	7.565	7.435	7.578	7.692	7.604
ENC1	11.164	11.118	11.103	11.146	11.017	11.071
COX7A2P2	11.076	10.953	11.002	11.140	11.150	11.194
NPW	4.578	4.392	4.681	4.366	4.419	4.161
KIFBP	9.789	9.767	9.777	9.290	9.260	9.372

AL049844.3	5.967	5.864	6.288	6.427	6.318	6.449
AC239803.1	5.582	5.759	5.666	5.859	5.730	5.725
EMCN	6.121	6.434	6.300	6.382	6.399	6.111
PPWD1	7.062	7.153	7.359	6.569	6.924	6.851
AC091271.1	6.213	6.274	6.569	6.003	5.904	6.035
TECTA	7.876	7.732	7.872	7.925	7.776	7.724
TECTB	8.206	8.381	8.183	8.261	8.391	8.536
LHX1	7.260	7.114	6.951	7.200	7.243	7.194
LHX2	6.161	6.285	6.095	6.312	6.496	6.497
ALDH8A1	5.047	5.023	4.882	4.957	4.867	4.868
VEGFD	6.921	6.900	6.695	6.681	7.037	7.012
LHX3	8.433	8.324	8.399	8.702	8.548	8.611
VEGFA	11.215	11.136	11.309	11.119	11.180	11.253
VEGFC	6.808	6.735	6.720	6.609	6.712	6.916
VEGFB	9.394	9.560	9.756	9.462	9.469	9.299
CRACD	6.917	7.043	6.801	6.510	6.531	6.588
BLOC1S3	8.869	8.890	9.049	8.796	8.954	8.781
UBE2E2	8.812	8.970	8.895	8.712	8.793	8.800
LHX5	6.450	6.716	6.943	7.115	7.227	6.871
LHX6	7.232	7.134	7.462	7.465	7.400	7.396
MIR106B	10.151	10.271	10.422	9.752	9.701	9.810
AL353653.1	7.487	7.648	7.531	7.426	7.607	7.638
EMC9	9.288	9.352	9.440	9.130	9.274	9.317
EMC8	10.914	10.909	10.995	10.660	10.775	10.680
FLOT1	11.222	11.308	11.374	11.341	11.301	11.200
HM190170.1	8.052	7.820	8.258	8.487	8.319	8.298
EMC3	10.343	10.323	10.522	10.427	10.309	10.217
EMC2	9.223	9.300	9.293	9.019	8.998	9.149
EMC1	9.017	9.054	9.211	8.781	8.786	8.773
EMC7	9.583	9.566	9.682	9.394	9.450	9.495
EMC6	9.920	9.980	10.145	10.000	10.003	9.999
EMC4	10.540	10.670	10.696	10.506	10.601	10.516
SERHL2	7.451	7.470	7.431	7.392	7.666	7.599
STKLD1	7.601	7.372	7.588	7.682	7.657	7.567
COL2A1	6.236	6.629	6.117	6.307	6.399	6.332
BSDC1	9.602	9.590	9.649	9.427	9.479	9.527
AC004918.5	6.743	6.686	6.670	6.576	6.938	6.642
AC148477.16	6.553	6.204	5.660	6.346	6.399	6.338
EGLN3	8.053	8.051	8.180	8.047	8.254	8.251
DNAH3	8.242	8.426	8.459	8.586	8.502	8.495
PLA2G10	9.154	9.228	9.331	8.950	9.052	9.023
C6orf118	5.072	5.129	5.135	5.210	5.121	5.120
ARID5B	7.720	7.687	7.745	7.778	7.794	7.760
ARID5A	7.587	7.662	7.835	7.697	7.801	7.511
ANKH	9.598	9.652	9.789	9.388	9.328	9.408
AC008175.1	5.502	5.659	5.466	5.983	5.555	5.487
AARS2	8.395	8.229	8.449	8.426	8.310	8.283
AC010422.3	9.721	9.672	9.928	9.586	9.604	9.501
FGF10-AS1	6.545	6.572	6.328	6.400	6.531	6.605
AC010422.6	7.153	6.994	7.265	7.042	6.991	7.060
AC010422.8	10.625	10.695	10.830	10.628	10.505	10.548
FOLH1	7.129	7.213	7.288	7.206	7.150	7.154
APPBP2	8.757	8.881	8.820	8.589	8.676	8.545
TLDC2	6.795	6.951	6.850	7.532	6.956	7.006
AC110774.1	3.769	3.777	3.664	3.878	3.618	3.828
EXOC2	7.566	7.565	7.585	7.284	7.373	7.514

EXOC3	9.213	9.389	9.479	8.976	9.049	9.142
EXOC1	9.653	9.674	9.718	9.552	9.432	9.604
HAP1	7.993	8.114	7.861	8.125	8.211	8.109
EXOC7	10.105	10.088	10.271	10.062	9.926	10.002
EXOC4	9.485	9.463	9.551	9.393	9.377	9.382
EXOC5	10.751	10.811	10.870	10.775	10.948	10.665
EXOC8	8.005	7.995	8.048	7.636	7.708	7.843
AC024475.3	7.922	7.741	7.744	8.010	7.909	8.109
ANK1	7.389	7.283	7.178	7.702	7.550	7.667
ANK2	6.548	6.791	6.713	6.996	6.773	6.718
ANK3	8.729	8.687	8.691	8.468	8.852	8.479
FAM99A	7.379	7.485	7.504	7.667	7.558	7.440
FAM99B	7.379	7.485	7.504	7.667	7.558	7.440
AC022164.1	5.895	5.933	5.888	5.850	5.930	6.010
AL441883.1	7.320	7.338	7.457	7.512	7.486	7.446
BCRP2	9.282	9.414	9.540	9.237	9.106	9.288
BCRP3	9.282	9.414	9.540	9.237	9.083	9.288
AC091812.3	11.927	12.089	12.206	11.982	12.035	11.920
BCRP6	9.282	9.414	9.540	9.237	9.083	9.288
BCRP7	9.282	9.414	9.540	9.237	9.083	9.288
COTL1	11.477	11.598	11.655	10.968	10.951	11.006
TSPEAR	5.917	5.909	6.245	5.782	5.862	5.926
CENPO	8.227	8.224	8.314	8.454	8.326	8.202
CENPN	9.324	9.400	9.548	8.987	9.135	9.135
DPP10	4.415	4.319	4.310	4.329	4.395	4.504
CENPL	8.263	8.290	8.399	7.794	8.456	7.771
CENPK	7.036	6.827	6.892	6.465	6.831	6.484
CENPJ	6.814	6.690	6.669	6.649	6.886	6.755
CENPI	7.362	7.418	7.379	7.088	7.010	7.067
CENPH	8.023	8.069	8.215	7.358	7.755	7.741
SIMC1	8.863	8.867	8.858	8.757	8.733	8.843
CENPF	10.142	9.893	10.007	9.477	9.473	9.626
CENPE	8.138	8.057	7.967	7.700	7.660	7.828
RAMP1	9.149	9.265	9.419	9.158	9.243	9.163
CENPB	8.887	8.830	9.219	8.881	8.922	8.869
RAMP3	7.906	8.011	7.803	7.825	8.121	7.882
RAMP2	7.277	7.561	7.394	7.413	7.741	7.572
CLCF1	8.877	8.864	8.871	8.965	8.795	8.731
SSH2	7.984	7.972	8.017	7.719	7.700	7.884
SSH3	10.155	9.973	10.225	10.192	10.271	10.045
CENPX	10.156	10.124	10.345	10.084	10.051	10.154
CENPW	9.323	9.181	9.595	8.881	8.950	8.921
CENPV	9.483	9.602	9.704	9.204	9.450	9.351
CENPU	10.415	10.403	10.692	10.144	10.101	10.083
CENPT	8.021	7.877	8.325	8.088	8.412	8.231
CENPS	8.559	8.514	8.847	8.507	8.405	8.388
CENPQ	7.169	7.242	7.373	6.710	6.877	7.052
CENPP	7.802	8.025	8.045	7.697	7.838	7.690
MIF4GD	9.450	9.560	9.660	9.351	9.574	9.521
UCA1	12.228	12.251	12.384	12.278	12.266	12.208
NET1	10.198	10.393	10.487	10.357	10.360	10.329
DHX38	8.896	8.867	8.885	8.802	8.594	8.821
TRIB1	11.218	11.270	11.297	11.281	11.303	11.236
TRIB2	8.295	8.370	8.493	8.192	8.311	8.431
TRIB3	11.756	11.795	11.997	11.688	11.779	11.624
DHX30	9.139	9.328	9.427	9.213	9.267	9.123

DHX32	10.211	10.177	10.313	10.123	10.103	10.139
DHX33	9.271	9.158	9.286	9.108	9.220	9.216
DHX34	8.633	8.557	8.667	8.668	8.702	8.563
DHX35	8.069	8.002	8.296	8.013	7.884	7.997
DHX36	12.325	12.284	12.383	12.532	12.365	12.220
DHX37	8.871	8.948	9.069	8.848	8.871	8.863
CABIN1	8.798	8.885	9.067	9.026	8.856	8.881
RPSAP44	8.391	8.408	8.502	8.636	8.578	8.636
SNRNP27	10.072	10.039	10.090	9.982	9.995	9.983
ZNF768	8.817	8.765	9.113	8.834	8.746	8.927
ZNF765	8.538	8.640	8.636	8.520	8.662	8.697
ZNF764	7.664	7.676	7.670	7.568	7.644	7.509
ZNF766	8.704	8.687	8.811	8.451	8.330	8.497
ZNF763	4.592	4.398	4.390	4.399	4.468	4.453
RPP21	8.653	8.718	8.869	8.590	8.637	8.555
RPP25	10.792	11.000	11.133	10.836	10.945	10.760
PMCH	6.770	6.946	6.785	6.300	6.609	6.552
NKX2-8	6.746	6.857	6.930	6.888	6.854	6.797
HACD1	8.518	8.518	8.720	8.470	8.441	8.418
HACD3	13.064	13.199	13.212	13.049	13.224	12.905
HACD2	10.666	10.734	10.798	10.597	10.471	10.632
HACD4	6.379	6.195	5.961	6.553	6.384	6.324
RIPOR1	10.873	10.978	11.010	10.792	10.960	10.829
RIPOR2	6.324	6.589	6.481	6.264	6.459	6.803
RIPOR3	7.509	7.329	7.276	7.039	6.903	7.247
RBMY2FP	5.626	5.630	5.714	5.651	5.615	5.811
BDNF-AS	4.505	4.663	4.535	4.967	4.739	5.001
AC010485.1	8.753	8.446	8.041	8.633	8.679	8.740
AC093787.2	4.385	4.464	4.479	4.834	4.445	4.587
ANKRD36BP2	8.504	8.360	8.059	8.305	8.402	8.272
AC004922.1	11.652	11.754	11.835	11.721	11.540	11.689
GCNA	6.939	7.270	7.153	6.822	6.927	6.742
GREB1	6.958	6.876	7.196	7.271	7.284	7.130
MACC1	10.605	10.621	10.532	10.518	10.355	10.533
PRR5-ARHGAP8	10.456	10.430	10.490	10.110	10.125	10.208
KRTAP5-8	8.833	9.020	9.043	8.951	9.158	9.100
KRTAP5-9	8.235	8.224	8.192	8.207	8.459	8.285
GCN1	10.190	10.125	10.085	9.894	9.672	10.049
SSX6P	6.529	6.484	6.297	6.417	6.448	6.563
ARSI	6.469	6.602	6.878	6.858	6.952	6.981
BX004987.1	3.848	3.989	3.892	4.180	4.103	4.156
MYO9B	8.635	8.794	8.827	8.941	8.843	9.001
MYO9A	9.060	9.198	9.239	9.313	9.263	9.108
AC068075.1	6.865	6.892	6.679	7.014	6.799	6.895
EZH1	8.790	8.727	8.631	8.567	8.411	8.599
EZH2	8.303	8.046	8.299	7.782	7.834	7.788
TEN1	8.180	8.232	8.437	8.065	7.882	8.043
RAVER1	9.049	9.019	9.074	8.903	8.937	8.928
RAVER2	7.308	7.499	7.734	7.165	7.183	7.188
NAA40	9.534	9.400	9.491	9.123	9.274	8.788
ATAD3C	6.776	6.887	6.876	6.983	6.890	6.886
ATAD3B	9.888	9.950	10.099	10.079	9.947	9.822
ATAD3A	9.517	9.616	9.703	9.782	9.648	9.616
NR4A2	8.468	8.270	8.337	8.752	8.659	8.477
NR4A3	7.655	7.521	7.331	7.990	7.918	7.804
NR4A1	8.508	8.491	8.618	8.474	8.662	8.596

AC020907.6	12.242	12.299	12.453	12.393	12.433	12.434
AC020907.2	7.470	7.756	7.467	7.664	7.743	7.604
ABCC1	10.115	10.104	10.211	10.153	10.043	10.170
BOLA1	7.555	7.734	7.815	7.291	7.407	7.446
BOLA3	10.905	11.029	11.137	11.210	11.237	11.060
BOLA2	10.698	10.766	10.949	10.772	10.875	10.874
NDUFB9	12.510	12.593	12.760	12.707	12.623	12.690
NDUFB8	10.809	10.770	11.012	10.663	10.513	10.652
NDUFB7	7.785	7.789	8.086	7.733	7.759	7.642
NDUFB6	10.731	10.869	10.999	10.760	10.780	10.771
NDUFB5	11.628	11.663	11.830	11.647	11.543	11.597
NDUFB4	11.668	11.676	11.788	11.570	11.531	11.548
NDUFB3	10.396	10.571	10.651	10.620	10.654	10.708
NDUFB2	10.679	10.741	10.923	10.617	10.513	10.618
NDUFB1	12.312	12.371	12.461	12.463	12.398	12.489
MIR1233-2	7.615	7.762	7.518	7.600	7.378	8.064
PDIA3P1	5.869	6.016	5.959	5.988	6.080	6.017
H4C11	7.985	8.191	8.334	8.210	8.208	8.084
H4C13	5.744	5.788	5.968	6.001	5.946	5.938
H4C12	7.985	8.191	8.334	8.210	8.208	8.084
H4C15	7.144	6.928	7.062	7.221	7.167	7.173
H4C14	7.144	6.928	7.062	7.221	7.167	7.173
DNAH11	8.064	8.249	8.077	8.345	8.253	8.163
STAP2	9.014	9.022	9.103	8.935	8.997	8.998
STAP1	5.288	5.215	5.196	5.472	5.241	5.391
UMAD1	8.634	8.815	8.618	8.261	8.415	8.338
Z99572.1	5.484	5.454	5.370	5.362	5.635	5.545
ANKRD45	4.681	4.689	4.675	4.668	4.607	4.546
ANKRD44	6.894	6.802	6.740	6.940	7.157	6.830
ANKRD46	8.384	8.426	8.267	8.248	8.471	8.338
RNF103-CHMP3	10.960	10.863	10.885	10.646	10.596	10.894
LINC00944	8.276	8.386	8.256	8.402	8.537	8.517
ANKRD49	8.891	8.721	8.893	8.942	8.944	8.995
FOXP1-AS1	6.053	6.315	6.095	6.184	6.430	6.406
LINC00942	7.522	7.483	7.527	7.639	7.690	7.703
LINC00943	7.660	7.438	7.184	7.702	7.575	7.520
MRRF	8.853	8.931	8.922	8.721	8.699	8.651
PHB	11.044	11.132	11.291	10.874	10.870	10.957
PGGT1B	8.088	8.153	8.254	7.727	7.774	7.654
AC018755.3	9.229	9.243	9.337	9.196	9.319	9.172
ATRAID	11.691	11.790	12.030	11.705	11.809	11.716
FAM224B	6.665	6.841	6.842	6.949	6.792	6.712
FAM224A	6.665	6.841	6.842	6.949	6.792	6.712
TAGLN2P1	11.722	12.003	12.022	11.882	11.881	11.789
FNBP1L	10.585	10.686	10.776	10.499	10.447	10.626
KANSL3	9.929	9.841	10.016	9.860	9.759	9.764
KANSL2	8.618	8.593	8.713	8.707	8.773	8.880
KANSL1	11.057	11.053	11.053	10.960	11.067	10.981
AC106820.3	5.946	6.153	6.020	6.102	6.007	6.093
AC106820.2	3.879	3.906	3.772	3.837	3.840	3.885
AC106820.4	7.828	7.890	7.726	7.763	7.847	7.859
RBM34	9.699	9.740	9.918	9.500	9.613	9.638
HOXC-AS3	7.245	7.309	7.153	7.306	7.647	7.280
RBM33	8.464	8.517	8.375	8.219	8.235	8.420
SENP6	8.842	8.816	8.921	8.401	8.587	8.722
RBM39	10.882	10.871	10.930	10.811	10.727	10.969

RBM38	8.520	8.585	8.802	8.793	8.810	8.551
EPHB2	8.825	8.843	9.040	8.997	9.042	8.805
EPHB3	8.054	8.122	8.123	8.121	8.093	8.203
EPHB1	7.319	7.442	7.497	7.530	7.811	7.604
EPHB6	7.449	7.217	7.419	7.328	7.275	7.311
EPHB4	9.476	9.379	9.650	9.450	9.216	9.238
TP53INP1	8.162	8.079	8.186	7.836	7.954	8.105
TP53INP2	9.455	9.476	9.517	9.360	9.309	9.364
ANG	9.603	9.648	9.712	9.344	9.371	9.427
MELK	9.000	9.060	9.272	8.424	8.689	8.595
AC097625.2	9.511	9.509	9.543	8.935	8.894	8.988
TIE1	5.585	5.411	5.474	5.673	5.679	5.729
MDFI	7.677	7.820	7.820	7.932	7.950	7.954
RASIP1	6.998	7.316	7.199	7.616	7.685	7.634
XAGE3	6.556	6.834	6.893	6.733	6.882	6.654
AC091230.1	9.442	9.362	9.451	8.945	9.147	9.121
AC091230.2	7.935	7.903	7.876	7.966	7.894	8.052
ZNF34	7.326	7.442	7.682	7.333	7.415	7.493
ZNF35	7.602	7.651	7.735	7.798	7.765	7.615
GAREM2	7.831	7.924	7.992	7.834	7.853	7.884
ZNF30	6.185	6.376	6.420	5.950	6.101	6.168
GAREM1	9.427	9.377	9.352	8.722	8.930	9.450
SH3GL1	9.743	9.884	9.926	9.898	9.809	9.793
AC037459.2	6.697	6.732	6.757	6.826	6.796	6.699
SH3GL2	4.797	5.025	5.112	4.756	4.971	5.037
SLC10A3	9.102	9.201	9.307	9.065	8.895	9.127
SLC10A2	6.654	6.506	6.626	7.025	6.623	6.575
SLC10A1	7.180	6.805	6.948	7.146	7.215	7.323
SLC10A7	7.262	7.320	7.461	6.859	6.999	7.144
SLC10A4	4.645	4.641	4.842	4.220	4.444	4.423
AC013489.1	10.134	10.089	10.234	9.940	9.887	9.951
OVAAL	6.325	6.605	6.351	6.475	6.461	6.479
UGGT2	7.583	7.633	7.662	7.388	7.477	7.478
UGGT1	9.440	9.422	9.478	9.112	9.076	9.254
BSPRY	9.557	9.657	9.809	9.399	9.707	9.417
ITPRIPL2	10.327	10.341	10.476	10.228	10.309	10.145
ITPRIPL1	7.367	7.468	7.136	7.076	7.207	7.442
KIAA1755	7.131	7.339	7.216	7.210	7.434	7.520
RFPL3S	6.178	6.203	6.106	6.107	6.168	6.311
LINC02397	6.443	6.115	5.877	6.515	6.445	6.439
LINC02395	4.323	4.362	4.266	4.213	4.380	4.316
AC022167.2	9.329	9.406	9.412	9.301	9.157	9.217
FRMD8P1	7.815	7.798	7.988	7.937	7.843	7.835
GPA33	8.049	8.093	8.003	7.919	8.337	8.276
DCLRE1B	8.287	8.286	8.220	8.178	8.138	8.005
DCLRE1C	8.621	8.619	8.593	8.112	8.496	8.515
DCLRE1A	8.237	8.306	8.258	8.243	8.141	8.230
CYP4A22	8.527	8.553	8.463	8.571	8.779	8.754
STK19B	9.245	9.328	9.319	9.203	9.266	9.289
ENPP1	6.813	6.825	6.971	6.844	6.842	6.857
ZNF804A	4.476	4.543	4.461	4.844	4.780	4.598
P2RX5-TAX1BP3	11.157	11.201	11.309	10.904	10.861	11.008
ZNF804B	5.185	5.106	4.944	5.493	5.616	5.356
UPP2	6.528	6.554	6.492	6.585	6.810	6.657
UPP1	10.156	10.235	10.433	10.135	10.127	10.188
FAM9B	5.832	5.453	5.726	5.711	5.524	5.718

FAM9C	4.956	4.755	4.895	4.858	4.804	4.716
FAM9A	5.017	5.061	4.962	4.844	5.228	5.014
ECHDC2	9.171	9.183	9.216	8.901	8.884	8.952
LYPLA1	10.479	10.613	10.611	10.434	10.827	10.476
LYPLA2	11.186	11.236	11.318	11.226	11.153	11.128
RNU6-37P	5.132	5.136	4.798	4.959	4.870	5.251
AL158837.1	6.151	6.010	6.062	6.671	6.317	6.150
AC080125.1	8.165	8.063	8.084	8.040	7.951	8.061
AL356124.2	5.897	6.122	5.993	5.987	5.981	5.839
CYBRD1	7.113	7.226	6.719	7.295	7.289	7.382
SPAG8	7.229	7.053	7.246	7.972	7.338	7.562
SPAG9	10.210	10.284	10.373	10.227	10.199	10.032
SPAG6	5.965	6.000	6.086	5.972	6.092	6.114
SPAG7	10.006	10.143	10.326	10.061	9.893	9.984
SPAG4	9.670	9.604	9.988	9.642	9.814	9.823
SPAG5	8.426	8.263	8.480	7.851	7.826	8.002
SPAG1	8.039	7.972	7.933	7.754	7.673	7.806
PSPHP1	8.410	8.321	8.339	7.853	8.029	8.498
CAB39L	7.530	7.577	7.691	7.490	7.683	7.527
FUT11	8.559	8.506	8.368	8.497	8.344	8.606
FUT10	8.766	8.974	8.843	8.727	8.842	8.893
PNLDC1	5.895	6.251	6.167	6.282	6.183	6.325
LINC00535	6.850	6.768	6.962	6.926	6.894	6.986
AL589740.1	3.884	4.047	3.888	3.900	3.765	3.976
LINC00536	4.634	4.471	4.506	4.991	4.863	4.630
DEFB105A	5.161	5.388	5.588	5.296	5.154	5.374
DEFB105B	5.161	5.388	5.588	5.296	5.154	5.374
AC092667.1	4.693	4.689	4.450	4.588	4.442	4.613
HIRIP3	9.290	9.340	9.371	8.803	8.897	8.955
RBPMS2	7.473	7.295	7.435	7.252	7.313	7.463
RP9	9.809	9.778	9.825	9.830	9.887	9.854
DCHS1	7.692	7.638	7.435	7.521	7.544	7.478
AL162458.1	6.121	5.977	6.193	6.128	5.981	6.114
DCHS2	8.167	8.228	8.174	8.268	8.221	8.233
AL163952.1	6.444	6.709	6.543	6.255	6.387	6.486
TMEM132C	7.769	7.723	7.510	7.801	7.724	7.539
ZNF658B	4.490	4.455	4.496	4.536	4.486	4.462
RALY-AS1	8.038	8.324	8.155	8.114	8.038	8.235
CARMIL2	7.574	7.303	7.242	7.656	7.726	7.520
CARMIL1	9.485	9.387	9.472	9.129	9.474	9.263
RASA4CP	7.614	7.679	7.735	7.914	7.765	7.740
TMEM132B	7.691	7.690	7.456	7.950	7.695	7.678
NWD2	7.812	7.595	7.720	7.035	7.346	7.528
AC007938.3	6.804	6.800	6.717	6.828	6.720	6.683
AC093323.3	4.872	5.033	4.829	4.952	5.137	5.084
AC093323.1	8.429	8.334	8.407	8.144	8.110	8.319
TENM3	5.780	5.503	5.819	5.986	5.745	5.757
TENM2	5.381	5.266	5.184	5.139	5.233	5.190
TENM1	5.430	5.354	5.466	5.544	5.541	5.643
TENM4	7.131	7.050	7.136	7.023	7.263	7.091
GAD1	8.507	8.636	8.702	8.279	8.216	8.435
GAD2	7.744	7.685	7.242	7.258	7.344	7.693
MTHFD2L	7.590	7.546	7.493	7.192	7.418	7.427
ESS2	9.759	9.855	9.927	9.445	9.566	9.452
ODF3L2	8.099	7.988	8.140	8.212	7.997	8.192
ODF3L1	6.325	6.432	6.591	6.318	6.629	6.544

DNHD1	8.418	8.311	8.088	8.391	8.127	8.480
CCDC71L	9.077	9.093	9.149	9.011	9.017	9.067
GOPC	9.228	9.144	9.127	9.074	9.046	9.127
C18orf54	6.847	6.646	6.711	6.578	6.567	6.554
JAZF1-AS1	4.475	4.690	4.548	4.747	4.722	4.812
BSN-DT	6.703	6.817	6.598	6.561	6.629	6.862
ARHGEF28	9.853	9.861	9.883	9.702	9.509	9.771
UBE2L5	12.031	12.165	12.312	12.106	12.010	12.060
STEAP1	10.634	10.657	10.813	10.585	10.639	10.626
STEAP3	9.258	9.384	9.379	9.385	9.209	9.307
STEAP2	10.034	10.015	9.934	9.988	10.174	9.904
ARHGEF25	7.851	7.724	7.940	7.955	7.950	7.940
STEAP4	8.343	8.283	8.273	8.242	8.198	8.359
ARHGEF26	9.285	9.267	9.305	9.245	9.150	9.322
DSCAS	5.911	5.860	6.149	5.894	5.973	6.070
Z95115.1	6.703	6.839	6.729	6.684	6.511	6.653
DSCAM	7.678	7.483	7.363	7.799	7.721	7.541
UBASH3A	5.636	5.231	5.272	5.682	5.682	5.528
UBASH3B	9.164	9.175	9.195	8.900	8.970	8.913
OSMR	10.520	10.606	10.618	10.443	10.474	10.439
AL138918.1	3.997	3.908	3.941	3.910	4.144	4.056
WHAMM	8.646	8.795	8.714	8.376	8.320	8.494
BIVM	8.047	8.183	8.232	7.989	7.946	7.969
TDO2	5.392	5.702	5.791	5.748	5.706	5.660
AC005618.2	5.479	5.513	5.552	5.649	5.548	5.740
IL18R1	6.237	5.818	6.306	6.293	6.115	6.013
GPC3-AS1	7.636	7.397	7.248	7.646	7.564	7.528
SEPTIN12	8.346	8.389	8.363	8.574	8.442	8.548
SEPTIN10	10.312	10.317	10.409	10.064	10.143	10.195
SEPTIN11	9.922	9.962	9.977	9.505	9.495	9.721
PDZRN3-AS1	6.867	6.766	6.948	7.082	6.981	7.173
AC073548.1	11.447	11.401	11.520	11.276	11.371	11.214
CFAP54	4.067	3.887	4.106	4.220	4.121	4.160
CFAP57	5.992	6.001	5.748	6.102	6.009	6.029
TMEM39B	8.442	8.429	8.537	8.093	8.179	8.065
CFAP53	6.147	6.506	6.475	6.575	6.628	6.576
CFAP52	6.032	6.091	5.895	5.945	6.119	6.070
GSTCD-AS1	5.664	5.594	5.626	5.800	5.731	5.641
POU2F1	8.960	8.794	8.927	8.595	8.573	8.752
UBE2FP1	6.504	6.386	6.371	5.982	6.127	6.220
RNA5SP257	6.955	7.194	7.044	7.366	7.106	7.378
GPAT4	10.713	10.830	10.875	10.552	10.314	10.621
GPAT3	6.864	7.042	7.211	6.898	7.138	6.504
GPAT2	6.417	6.586	6.446	6.526	6.489	6.524
GABARAP	12.216	12.262	12.346	12.232	12.172	12.171
AC093714.1	4.219	4.261	4.101	4.733	4.670	4.301
MANF	10.037	10.076	10.233	10.199	10.171	10.195
CASC22	4.490	4.558	4.619	4.558	4.657	4.593
CASC23	8.994	8.866	8.568	9.087	9.241	8.909
LAMTOR5-AS1	5.810	5.619	5.825	5.889	5.863	5.784
AP001462.1	5.993	6.053	6.013	5.953	6.060	5.749
CHRNA10	7.749	7.886	7.727	7.849	7.999	8.042
TSPOAP1	7.304	7.153	7.314	7.385	7.396	7.242
SPTSSA	10.078	10.149	10.226	10.155	9.925	10.138
SPTSSB	7.755	7.772	7.960	7.736	7.849	7.740
AC012499.1	5.335	5.275	4.898	5.328	5.090	5.316

ETF1	11.279	11.209	11.362	11.239	11.175	11.219
MLANA	6.438	6.714	6.323	6.871	6.819	6.862
SLC22A4	6.380	6.705	6.295	6.563	6.558	6.611
SLC22A5	8.961	8.816	8.805	8.673	8.795	8.721
SLC22A6	6.752	6.779	7.021	7.024	6.859	6.789
SLC22A7	7.785	7.595	7.765	7.944	7.857	7.711
SLC22A1	6.177	6.475	6.349	6.578	6.319	6.324
PRDM2	9.051	9.246	9.158	9.083	9.353	9.241
SLC22A3	5.399	5.490	5.496	5.061	5.110	5.313
PRDM8	7.128	6.944	7.316	7.403	7.152	7.189
SLC22A9	7.080	7.372	7.226	7.073	7.106	7.394
LCT-AS1	6.427	6.482	6.377	6.268	6.335	6.245
NPDC1	9.947	10.021	10.203	9.851	9.822	9.934
AC005912.1	13.947	14.022	14.018	14.057	14.036	14.057
ADCYAP1	6.561	6.533	6.423	6.477	6.645	6.642
STT3B	11.353	11.507	11.501	11.249	11.165	11.311
HIC1	7.432	7.438	7.469	7.701	7.477	7.570
HIC2	8.417	8.518	8.489	8.375	8.555	8.373
IGKJ2	4.150	4.226	4.135	4.366	4.272	4.315
HFM1	4.174	4.194	4.309	4.471	4.418	4.395
RPL21P2	7.005	7.088	7.111	7.033	7.214	7.277
RPL21P1	12.617	12.677	12.802	12.784	12.769	12.841
IGKJ4	4.150	4.226	4.135	4.366	4.272	4.315
IGKJ5	4.150	4.226	4.135	4.366	4.272	4.315
SERTM1	4.193	4.254	4.268	4.100	4.541	4.190
RXRG	5.606	5.735	5.521	5.563	5.740	5.800
RXRA	9.644	9.615	9.657	9.429	9.592	9.443
RXRB	8.960	9.150	9.313	9.306	9.151	9.183
IGHV3-25	7.299	7.202	7.258	7.559	7.537	7.472
CCNYL1	8.380	8.420	8.416	8.119	8.073	8.155
IGHV3-20	7.732	7.854	7.876	8.122	8.004	8.078
IGHV3-21	6.669	6.626	6.403	6.818	6.565	6.509
IGHV3-23	6.974	6.852	6.943	7.386	7.126	7.195
WRNIP1	9.792	9.818	9.872	9.699	9.476	9.662
ABCA6	5.500	5.191	5.036	5.394	5.292	5.266
ABCA7	7.311	7.335	7.450	7.554	7.282	7.161
ABCA4	7.510	7.545	7.339	7.387	7.477	7.611
ABCA5	6.553	6.425	6.437	6.221	6.306	6.548
ABCA2	8.310	8.146	8.335	8.397	8.326	8.348
ABCA3	7.834	7.836	8.018	7.985	7.911	7.910
ABCA1	6.196	6.299	6.446	6.286	6.539	6.281
CIZ1	10.015	9.936	10.103	9.886	9.779	9.790
NBEAL1	13.555	13.621	13.669	13.572	13.470	13.564
NBEAL2	8.971	8.867	8.945	8.470	8.449	8.688
CBLL1	6.747	6.921	6.744	6.530	6.482	6.704
HARBI1	7.700	7.662	7.782	7.988	7.670	7.794
CBLL2	6.833	6.997	6.941	7.085	7.198	7.285
RTP4	9.455	9.436	9.533	9.446	9.623	9.361
RTP5	7.777	7.680	7.729	7.387	7.404	7.626
RTP3	6.870	7.020	6.864	6.990	7.081	7.004
RTP1	5.726	5.852	5.896	5.829	5.969	5.775
NPM1P22	10.736	10.799	10.817	10.835	10.799	10.912
NPM1P27	13.203	13.272	13.262	13.249	13.199	13.294
SLC25A48-AS1	6.760	6.710	6.678	6.861	6.841	6.787
PPP5D1	5.688	5.357	5.332	5.743	5.502	5.668
SPON2	7.758	7.963	8.068	7.826	8.019	7.941

SPON1	8.960	9.035	8.730	8.881	9.001	9.003
SNORA11	9.145	9.292	9.497	9.026	8.932	9.160
SCN8A	6.871	6.852	6.870	6.875	6.872	6.884
SAA3P	5.911	5.663	6.022	5.929	6.052	5.913
SNORA10	14.545	14.566	14.561	14.591	14.532	14.585
NMUR2	10.740	10.948	10.827	11.057	10.965	10.904
NMUR1	5.699	5.533	5.493	5.759	5.842	5.854
ZC4H2	8.289	8.427	8.564	8.284	8.319	8.408
NCK1	10.156	10.261	10.345	10.381	10.394	10.354
NCK2	11.142	11.129	11.292	11.068	11.076	11.088
LVRN	7.158	7.018	7.027	7.116	7.071	7.066
AC007967.1	8.595	8.714	8.802	9.212	8.992	8.861
C20orf96	7.181	7.007	7.004	7.042	7.173	7.035
ZNF264	9.023	9.014	9.004	8.592	9.063	9.030
ZNF266	8.542	8.534	8.606	8.306	8.225	8.381
KCNU1	6.716	6.914	6.704	6.562	6.816	6.934
ZNF260	9.218	9.268	9.164	9.042	8.955	9.104
ZNF263	9.744	9.834	9.866	9.740	9.620	9.741
ZNF268	8.253	8.251	8.339	8.126	8.251	8.163
TSNAX	9.776	9.785	9.762	9.478	9.540	9.656
C17orf49	12.049	12.125	12.282	11.981	12.082	12.102
RPH3A	7.559	7.581	7.515	7.658	7.725	7.714
AC009299.3	5.610	5.921	5.832	5.878	5.974	5.949
CYP51A1P2	11.508	11.576	11.617	11.262	11.230	11.545
KCTD17	8.348	8.282	8.398	8.375	8.263	8.196
VLDLR	9.354	9.472	9.473	9.494	9.475	9.464
BTF3L4	10.229	10.140	10.340	10.339	10.298	10.285
FTSJ3	9.151	9.117	9.213	9.066	8.828	8.950
FTSJ1	10.516	10.669	10.657	10.555	10.463	10.579
AC023136.1	4.152	4.338	4.192	4.227	4.204	4.140
DERL1	11.224	11.416	11.457	11.250	11.270	11.197
DERL2	8.902	8.850	9.064	8.923	8.837	8.964
DERL3	7.935	7.876	7.933	7.980	7.880	8.097
PRR29	7.926	7.951	7.829	8.301	8.146	7.973
PRR26	8.134	8.166	8.274	8.039	8.247	8.163
PRR22	7.578	7.518	7.663	7.566	7.745	7.727
NCR3	7.487	7.471	7.622	7.529	7.653	7.542
FBN3	6.503	6.431	6.222	6.655	6.401	6.694
LINC01936	6.555	6.681	6.716	6.474	6.718	6.738
IBTK	9.739	9.798	9.733	9.555	9.461	9.638
LINC01933	6.610	6.685	6.624	6.695	6.713	6.853
LINC01931	5.724	5.852	5.819	5.746	5.917	5.972
AL603832.3	11.391	11.479	11.635	11.371	11.340	11.418
HOXA-AS3	7.232	7.038	6.863	7.079	7.130	7.136
HOXA-AS2	6.638	6.750	6.727	7.008	6.924	6.974
AL358472.2	6.422	6.306	6.304	6.102	6.410	6.449
HSPBAP1	6.744	6.741	6.764	6.964	6.758	6.890
AL358472.6	11.865	11.917	12.096	11.901	11.922	11.943
AL358472.7	9.401	9.598	9.678	9.419	9.520	9.429
ZBTB26	6.781	7.102	7.008	7.013	6.976	7.032
S100A7A	6.678	6.762	6.711	6.705	6.925	6.738
ZBTB25	7.307	6.994	7.469	7.246	7.131	6.993
AC090686.1	13.912	13.956	14.032	14.042	14.027	14.131
KHDC3L	6.211	6.169	6.367	6.497	6.419	6.182
IGLC2	6.866	6.903	7.131	6.914	7.023	7.073
IGLC3	6.866	6.903	7.131	6.914	7.023	7.073

ITIH1	7.745	7.846	7.812	7.921	8.017	7.910
ITIH3	6.240	6.305	6.085	6.340	6.234	6.364
ITIH2	8.310	8.385	7.970	8.284	8.242	8.289
NDC80	8.556	8.484	8.636	7.681	7.998	7.775
ITIH4	7.709	7.730	7.809	7.884	8.014	7.993
ITIH6	6.601	6.551	6.811	6.915	6.882	6.754
AC012306.4	6.077	5.886	6.077	6.325	6.128	6.025
AC012306.2	8.201	8.293	8.364	7.882	7.577	7.987
TNFSF9	6.994	6.816	7.018	6.860	6.924	6.857
CHPT1	10.290	10.423	10.638	10.523	10.607	10.307
AZIN1-AS1	6.080	6.042	6.355	6.158	6.333	6.238
TNFSF4	6.551	6.659	6.375	6.317	6.549	6.723
GMPPA	8.925	8.880	9.134	8.960	8.989	8.944
GMPPB	8.819	8.868	9.002	8.921	8.724	8.789
AC022966.1	9.175	9.318	9.321	9.289	9.382	9.258
MAS1	7.772	7.764	7.515	7.876	7.999	8.034
OXGR1	5.457	5.284	5.486	5.830	5.315	5.537
OIT3	5.421	5.703	5.550	5.604	5.273	5.789
MIR6514	9.329	9.260	9.349	9.477	9.334	9.380
HNRNPA1P37	6.149	6.313	6.167	5.998	6.119	6.326
HNRNPA1P35	13.482	13.508	13.526	13.344	13.317	13.319
RAD52	7.038	6.767	6.910	6.916	6.843	6.969
RAD51	9.019	9.189	9.270	9.262	9.013	9.007
RAD50	8.328	8.483	8.449	8.293	8.214	8.335
TTTY9B	5.534	5.482	5.365	5.619	5.244	5.157
RNF38	7.404	7.575	7.278	7.253	7.423	7.348
RNF39	7.905	7.678	7.962	8.107	8.038	7.961
RNF34	10.343	10.416	10.514	10.236	10.335	10.312
RNF32	6.704	6.898	6.847	6.610	6.928	6.981
RNF31	7.887	8.167	8.142	7.850	7.844	7.855
MZF1	8.618	8.567	8.676	8.429	8.263	8.500
TPPP	7.666	7.443	7.616	7.989	7.844	7.656
AC036214.1	4.636	4.855	4.921	4.729	5.027	4.422
AL133414.3	8.835	8.689	8.488	8.744	8.793	8.879
AC036214.3	8.486	8.496	8.795	8.429	8.256	8.420
AC005229.4	7.801	7.840	8.096	7.257	7.432	7.501
INTS3	10.114	10.129	10.174	10.117	10.068	10.079
INTS2	5.949	6.482	6.331	6.004	6.186	6.159
INTS1	9.827	9.911	10.023	9.734	9.628	9.806
INTS7	8.605	8.826	8.776	8.549	8.408	8.665
INTS6	7.863	8.030	8.136	8.072	8.058	8.027
INTS5	9.724	9.821	9.968	9.793	9.839	9.807
INTS4	8.398	8.508	8.592	8.268	8.271	8.268
TPP1	12.304	12.367	12.561	12.612	12.530	12.410
INTS9	9.098	9.016	9.215	8.768	8.802	8.825
INTS8	8.616	8.670	8.747	8.745	8.712	8.707
MIR1182	9.534	9.460	9.528	9.576	9.724	9.350
AL356512.1	4.200	4.135	4.156	4.107	3.994	4.246
FAM181B	7.747	7.448	7.698	7.654	7.707	7.470
FAM181A	7.336	7.507	7.329	7.358	7.466	7.582
AP000289.1	6.458	6.609	6.401	6.370	6.481	6.367
CDRT15	7.226	7.176	7.129	7.251	7.324	7.386
TGIF2-RAB5IF	13.198	13.251	13.397	13.232	13.120	13.212
RGS6	8.071	7.998	7.882	7.820	8.093	7.933
MYL6	13.303	13.419	13.516	13.481	13.419	13.476
MYL7	7.474	7.299	7.457	7.615	7.707	7.516

MYL4	7.479	7.725	7.772	7.579	7.543	7.756
MYL5	7.772	7.767	7.978	7.871	7.715	7.836
MYL2	6.019	5.918	6.003	6.105	6.156	6.187
MYL3	8.314	8.347	8.317	8.234	8.376	8.455
DUXAP8	7.437	7.399	7.258	7.473	7.400	7.613
MYL1	6.681	6.515	6.539	7.189	6.790	6.961
CR936918.2	6.517	6.543	6.395	6.499	6.649	6.716
CDKN2AIPNL	8.202	8.290	8.405	8.101	8.355	8.351
MYL9	7.407	7.500	7.523	7.899	7.533	7.508
NCEH1	10.747	10.931	10.904	10.673	10.691	10.899
TSNARE1	8.214	8.434	8.284	8.164	8.245	8.286
MPP3	8.261	8.296	8.142	8.040	8.174	8.230
MPP2	8.494	8.519	8.416	8.257	8.389	8.415
MPP1	7.722	7.560	7.581	7.884	7.879	7.825
AL135925.1	8.201	8.293	8.364	7.882	7.577	7.987
MPP7	7.107	7.229	7.062	7.039	7.296	7.211
MPP6	7.903	8.044	8.245	7.821	7.764	7.741
MPP5	10.125	10.234	10.166	9.990	9.917	10.046
MPP4	6.112	6.074	5.891	6.041	6.219	6.073
FAM110A	8.263	8.174	8.347	8.259	8.270	8.244
FAM110C	9.642	9.480	9.843	9.385	9.403	9.473
FAM110B	7.555	7.476	7.435	7.595	7.500	7.610
FAM110D	7.034	7.132	7.071	7.217	7.270	7.169
SPTB	7.888	8.040	8.138	8.129	8.153	8.246
AC022613.3	5.481	5.494	5.253	5.563	5.683	5.544
AC022613.1	6.957	7.164	6.808	6.871	7.228	7.005
SNTG1	7.333	7.359	7.268	7.620	7.412	7.271
AC005911.1	6.530	6.462	6.498	6.222	6.377	6.260
SNTG2	5.694	5.336	5.388	5.700	5.543	5.531
LY86-AS1	5.730	5.758	5.920	5.810	5.837	5.928
AC104836.1	3.809	3.763	3.660	3.896	3.662	3.707
HJV	6.170	6.105	5.946	6.143	5.818	5.745
TSPO2	7.497	7.538	7.620	7.598	7.628	7.648
MYLK	7.741	7.650	7.916	7.862	7.838	7.864
AC004241.5	6.836	6.921	6.807	7.048	7.268	7.174
AC004241.4	6.146	6.195	6.301	6.717	6.274	6.358
AC004241.1	6.990	7.077	6.952	6.965	7.261	7.126
YME1L1	11.048	11.034	10.946	10.868	10.785	10.844
GORASP2	11.371	11.408	11.486	11.257	11.262	11.271
GORASP1	8.903	9.004	9.082	8.918	8.873	8.899
PPRC1	8.359	8.382	8.559	8.402	8.147	8.350
CCKAR	6.820	6.740	6.755	7.093	6.919	6.981
AC000120.1	8.402	8.514	8.443	7.512	7.834	7.423
TRIM49D1	5.367	5.231	5.211	5.384	5.336	5.373
TRIM49D2	5.367	5.231	5.211	5.384	5.336	5.373
LINC02582	5.738	5.622	5.622	5.604	5.623	5.597
LINC02583	6.175	6.216	6.069	5.905	6.135	6.100
LINC02580	7.295	7.499	6.978	7.361	7.376	7.508
SUMF1	10.157	10.186	10.357	9.834	9.775	9.788
SURF1	10.885	10.937	11.116	10.856	10.809	10.859
FNDC3A	10.011	9.971	9.947	9.872	9.885	10.064
FNDC3B	11.892	12.103	12.236	11.975	11.992	11.897
SURF2	7.674	7.725	7.940	7.917	7.814	7.747
SURF4	11.419	11.525	11.641	11.527	11.601	11.513
SURF6	8.693	8.771	8.843	8.701	8.750	8.705
RAD21-AS1	5.940	5.981	5.882	5.918	5.970	5.932

EFS	8.281	8.263	8.236	8.251	8.432	8.213
MB21D2	8.886	8.879	8.814	8.894	8.899	8.923
CORO2B	6.175	6.042	6.007	6.304	6.248	6.415
AC005523.1	7.308	7.333	7.635	7.776	7.717	7.607
SPACA4	7.795	7.890	7.847	8.094	8.057	7.774
SPACA6	6.944	6.892	7.076	6.843	6.958	6.982
SPACA7	5.825	5.775	5.677	5.659	5.786	5.724
CCDC130	8.675	8.737	8.908	9.084	8.955	8.878
SPACA1	4.961	5.046	4.765	5.099	5.024	5.237
SPACA3	6.039	5.876	6.004	6.066	5.912	5.930
TIMM23	10.577	10.701	10.712	10.592	10.487	10.576
AC009123.1	7.768	7.820	7.627	7.632	7.731	7.682
CCDC138	6.476	6.420	6.483	6.401	6.491	6.420
SPACA9	6.450	6.764	6.828	6.822	6.549	6.911
AL035071.1	8.903	8.881	8.891	8.563	8.557	8.734
STK31	5.303	5.503	5.561	5.373	5.081	5.501
STK33	7.124	7.339	7.311	7.092	7.178	7.245
STK35	9.777	9.785	9.941	9.370	9.268	9.548
STK36	8.741	8.629	8.694	8.847	8.706	8.864
LINC01588	7.442	7.598	7.536	7.504	7.510	7.383
LINC01587	5.497	5.419	5.777	5.732	5.743	5.283
PHF5A	9.862	10.021	10.230	10.040	10.088	9.807
LINC01585	6.486	6.700	6.838	6.749	6.811	6.880
LINC01583	4.227	4.239	4.308	4.437	4.378	4.400
LINC01581	4.397	4.588	4.454	4.394	4.606	4.448
LINC01580	5.590	5.651	5.675	5.624	5.340	5.667
DNAAF1	7.961	7.909	7.914	7.971	7.966	7.898
DNAAF2	7.947	7.818	7.816	7.857	7.866	7.995
DNAAF3	8.384	8.590	8.413	8.548	8.642	8.746
DNAAF4	6.722	6.637	6.823	6.523	6.755	6.493
DNAAF5	10.372	10.402	10.482	10.229	10.274	10.234
AC026271.3	6.496	6.571	6.284	6.178	6.402	6.378
AC026271.1	9.976	10.097	10.085	10.006	10.059	10.061
RN7SKP115	6.526	6.388	6.396	6.179	6.502	6.468
CHRNB1	7.172	7.104	7.300	7.304	7.277	6.991
CHRNB3	6.208	6.301	6.055	6.348	6.260	6.282
CHRNB2	9.433	9.391	9.556	9.616	9.627	9.548
CHRNB4	4.457	4.373	4.560	4.775	4.687	4.819
HBEGF	8.972	8.999	8.930	8.832	8.747	8.803
AC140479.5	11.782	11.826	11.915	11.839	11.821	11.771
LINC01056	7.824	8.084	7.929	8.096	7.979	8.196
CYP7A1	4.004	4.037	4.004	3.881	4.029	3.947
CRYAA	7.086	7.310	7.555	7.262	7.244	7.349
PODNL1	7.897	7.973	8.060	7.969	8.051	8.088
GSTT2	7.413	7.486	7.589	7.314	7.369	7.502
ZNRD2	10.694	10.784	10.895	10.720	10.731	10.773
ZNRD1	9.231	9.374	9.436	9.462	9.438	9.339
PLXNA3	9.024	8.989	8.837	8.909	8.693	9.062
PLXNA2	8.379	8.380	8.625	8.237	8.258	8.304
PLXNA1	10.440	10.431	10.467	10.125	10.027	10.114
PBDC1	9.972	10.032	10.167	9.877	9.889	9.558
SLC11A1	8.301	8.690	8.812	8.528	8.644	8.586
PLXNA4	7.053	7.275	7.244	7.617	7.301	7.336
AC137630.3	8.166	8.194	8.227	8.022	8.183	8.234
CAPZA1P	4.751	4.813	4.821	4.772	4.685	5.048
PEG3	5.961	5.843	5.357	5.661	5.898	5.636

SCAI	6.002	5.968	5.918	6.518	6.435	6.150
WASH5P	8.680	8.622	8.499	8.424	8.510	8.820
AC068775.1	4.658	4.629	4.662	4.598	4.742	4.586
AC136759.1	5.367	5.231	5.211	5.384	5.336	5.373
TCF4-AS1	6.688	6.886	6.845	6.786	6.826	6.946
MAP2K3	11.460	11.677	11.671	11.488	11.544	11.414
MAP2K2	11.063	11.116	11.325	10.986	10.986	10.980
MAP2K1	11.883	11.876	12.024	11.784	11.683	11.785
MAP2K7	8.869	8.833	8.923	8.764	8.919	8.710
MAP2K6	9.331	9.105	9.295	9.223	9.207	9.177
MAP2K5	8.860	8.853	9.130	8.911	8.800	8.910
MAP2K4	8.404	8.522	8.487	8.551	8.676	8.460
SCAP	9.741	9.849	9.915	9.820	9.737	9.809
MINK1	10.368	10.464	10.503	10.127	10.231	10.220
ACY3	7.075	7.146	7.165	7.372	7.337	7.211
ACY1	10.008	10.064	10.115	9.872	9.784	9.806
ZRSR2	8.373	8.368	8.472	8.382	8.399	8.494
GRPR	8.709	8.834	8.643	8.777	8.809	8.927
AC004690.2	6.062	6.149	6.329	6.302	6.251	6.388
GPR173	7.910	8.120	8.123	8.312	8.197	8.234
MIR367	6.683	6.626	6.864	7.044	7.444	6.745
AC008764.10	5.870	5.824	5.659	5.830	5.978	5.786
PSMF1	10.752	10.887	11.006	10.815	10.771	10.743
BDNF	7.471	7.457	7.533	7.447	7.578	7.264
BORA	6.987	7.127	7.086	6.898	6.999	6.693
AC104051.2	6.293	6.389	6.313	6.311	6.231	6.097
CSNK2A3	10.003	10.016	10.166	9.827	10.080	9.716
CSNK2A2	9.611	9.587	9.830	9.259	9.283	9.412
CSNK2A1	10.120	10.187	10.226	10.110	10.213	10.064
KDM5D	6.788	7.095	6.841	6.823	6.908	6.888
KDM5A	9.410	9.342	9.539	9.076	9.275	8.999
KDM5B	12.417	12.487	12.646	12.569	12.532	12.593
KDM5C	9.655	9.831	9.893	9.859	10.024	9.859
AIPL1	7.230	7.204	7.212	7.398	7.341	7.242
TRAIP	7.337	7.253	7.319	6.915	7.089	7.145
GGTLC5P	10.427	10.413	10.580	10.351	10.206	10.300
GLUL	10.561	10.622	10.704	10.400	10.237	10.408
IFT122	8.849	8.839	8.915	8.552	8.570	8.549
C9orf106	7.250	7.367	7.310	7.267	7.255	7.374
AC121493.1	7.009	7.063	6.854	7.109	7.006	6.990
AC011479.2	11.740	11.806	12.051	11.924	11.678	11.929
AC011479.4	7.444	7.129	7.331	7.863	7.697	7.605
GYS1	10.605	10.772	10.813	10.567	10.636	10.670
GYS2	4.100	4.126	4.340	4.286	4.275	4.443
ETV1	9.913	10.195	10.255	9.841	9.914	9.599
ETV3	9.576	9.451	9.474	9.369	9.378	9.348
ETV2	6.840	6.749	7.131	7.121	6.910	7.137
ETV5	10.925	10.912	11.020	10.638	10.671	10.657
ETV4	9.819	9.931	9.987	9.671	9.776	9.604
ETV7	7.714	7.728	7.829	7.948	7.980	7.915
ETV6	10.210	9.952	10.082	10.147	9.882	10.093
FAM30A	7.492	7.127	7.073	7.019	7.238	7.326
ABHD17AP6	10.190	10.287	10.526	10.113	10.130	10.051
ABHD17AP3	10.190	10.287	10.526	10.113	10.130	10.051
ABHD17AP1	10.190	10.287	10.526	10.113	10.130	10.051
SNF8	10.793	10.892	11.014	10.738	10.766	10.676

AC009229.4	6.540	6.353	5.999	6.581	6.515	6.452
IKZF5	7.530	7.690	7.538	7.766	8.044	7.979
IKZF4	8.033	7.981	8.077	7.877	8.044	7.970
IKZF1	8.668	8.717	8.639	8.771	8.693	8.856
IKZF3	8.137	8.159	8.206	8.318	8.244	8.502
IKZF2	7.441	7.284	7.526	7.071	7.375	7.196
C11orf71	8.719	8.737	8.660	8.594	8.596	8.703
C11orf72	8.831	8.826	8.649	8.585	8.923	8.890
C11orf74	8.530	8.551	8.668	8.351	8.519	8.554
TCEAL3	10.315	10.141	10.372	9.987	10.100	9.913
TCEAL2	4.772	5.060	4.808	4.953	4.960	5.291
TCEAL1	9.575	9.641	9.705	9.646	9.647	9.644
PICSAR	5.543	5.688	5.719	5.811	5.912	5.769
TCEAL4	11.333	11.419	11.475	11.175	11.114	11.110
AP002957.1	7.113	7.154	7.175	7.836	7.354	7.451
TCEAL9	11.303	11.318	11.400	11.383	11.432	11.494
TCEAL8	3.924	4.135	4.196	4.406	4.072	4.195
PTTG3P	7.782	7.948	7.842	7.516	7.573	7.860
AL035252.3	6.949	6.762	6.693	6.972	6.752	6.966
AL353801.3	7.034	6.895	7.038	7.036	7.105	6.972
AL035252.5	6.194	6.448	6.447	5.929	6.522	6.445
PTH1R	6.748	6.719	6.763	6.834	6.773	6.799
HMX2	5.980	5.920	5.926	6.052	6.020	6.086
AC020910.5	5.835	5.629	5.900	6.122	5.811	6.036
AC020688.1	9.479	9.567	9.603	9.525	9.547	9.455
CACYBP	11.459	11.484	11.678	11.547	11.515	11.560
AL391097.1	6.825	6.964	6.962	6.968	7.019	7.139
PRPS2	10.529	10.483	10.602	10.047	10.016	10.199
LHFPL3-AS1	5.888	6.027	5.754	5.978	6.154	6.010
LHFPL3-AS2	7.941	7.606	7.743	7.686	7.963	7.667
PRPS1	10.846	10.923	10.862	10.708	10.746	10.746
CDR2	9.632	9.632	9.577	9.533	9.578	9.684
CDR1	4.186	4.085	3.926	4.296	4.134	4.221
BRPF3	8.378	8.411	8.370	8.122	8.230	8.304
BRPF1	8.730	8.468	8.633	8.462	8.501	8.616
LUZP4	8.204	8.100	7.897	8.243	8.341	8.244
LUZP6	11.772	11.795	11.785	11.704	11.645	11.724
C8orf88	8.871	8.868	8.876	8.407	8.364	8.465
LUZP1	8.756	8.804	8.914	8.847	8.899	8.938
LUZP2	7.838	7.703	7.551	7.549	7.821	7.847
C8orf82	10.440	10.508	10.784	10.349	10.373	10.263
BCL6B	8.310	8.261	8.164	8.324	8.401	8.313
CNTFR-AS1	7.551	7.607	7.668	7.577	7.710	7.645
UBE2Q1	10.493	10.535	10.592	10.351	10.219	10.316
UBE2Q2	10.304	10.444	10.458	10.251	10.332	10.402
REM1	7.657	7.585	7.745	7.834	7.680	7.889
REM2	7.578	7.319	7.263	7.782	7.617	7.808
ATP5MDP1	12.211	12.277	12.348	12.273	12.220	12.288
RPEL1	7.270	7.352	7.378	7.083	6.762	7.072
AC241640.1	8.943	8.649	8.679	8.388	8.372	8.812
ASS1	9.958	10.032	10.219	10.130	9.927	10.129
SPX	7.806	7.888	8.067	8.256	8.166	8.047
ZNF879	5.648	5.660	5.540	5.643	6.001	5.721
ZNF875	7.788	7.691	7.768	7.427	7.466	7.444
COPRS	9.870	9.921	9.937	9.583	9.545	9.616
SPN	8.942	9.088	9.261	9.348	9.427	9.341

AC009120.2	7.840	7.805	7.912	7.780	7.992	7.797
SP8	8.700	8.787	8.919	9.151	9.247	9.048
SP1	9.904	10.036	10.095	9.877	9.987	9.790
SP2	9.507	9.468	9.499	9.017	9.055	9.126
SP3	10.621	10.671	10.728	10.549	10.726	10.581
SP4	9.015	8.822	8.872	8.763	8.592	8.816
SP5	7.141	7.079	7.199	7.270	7.262	7.008
SP6	7.762	7.836	7.741	7.810	7.834	7.890
SP7	6.737	6.766	6.955	7.011	6.826	6.960
GOLIM4	10.494	10.605	10.325	9.905	10.152	10.395
KLLN	7.471	7.609	7.577	7.495	7.538	7.517
SPPL2A	10.868	10.955	11.014	10.817	10.829	10.866
SPPL2C	6.572	6.834	6.979	6.978	6.993	7.017
SPPL2B	8.737	8.754	8.869	8.668	8.632	8.673
UGCG	11.478	11.546	11.653	11.580	11.801	11.305
ATP2A1	6.898	7.072	6.879	7.328	6.994	6.996
ATP2A2	13.182	13.258	13.265	13.267	13.128	13.260
ATP2A3	8.101	8.375	8.142	8.262	8.175	8.216
GAP43	6.885	6.884	6.909	6.952	7.128	7.042
AC090515.3	6.879	7.015	6.751	6.676	6.815	6.932
AL157392.4	4.321	4.275	4.447	4.178	4.204	4.395
AL157392.5	8.341	8.385	8.313	7.900	8.010	8.037
FBXL15	7.369	7.417	7.924	7.443	7.321	7.379
AL157392.3	6.913	6.850	6.795	6.801	6.848	6.877
FBXL17	8.885	8.720	8.886	8.846	8.942	8.840
FBXL16	8.377	8.375	8.643	8.123	8.342	8.244
FAM169A	9.063	9.112	9.182	8.994	9.020	9.088
FBXL18	10.080	10.213	10.195	10.159	10.281	10.291
FAM169B	7.140	7.222	7.201	7.245	7.319	7.375
SDK1	6.627	6.731	6.791	6.541	6.620	6.611
SDK2	8.959	8.934	8.749	8.907	8.930	9.103
KANSL1-AS1	10.087	10.160	10.360	10.000	10.071	9.865
GRP	7.317	7.346	7.093	7.296	7.336	7.292
AC137499.2	7.623	7.706	7.631	7.631	7.621	7.671
AC137499.1	9.106	9.330	9.270	9.257	9.359	9.374
IQCF5-AS1	7.026	7.130	6.661	6.920	7.201	7.004
AC137499.4	7.623	7.706	7.631	7.631	7.621	7.671
HMGB3P1	8.888	9.010	8.932	8.659	8.752	8.691
GRN	12.339	12.395	12.542	12.155	12.075	12.092
LINC02743	5.538	5.399	5.317	5.514	5.490	5.523
LINC02749	5.456	5.772	5.743	6.291	5.736	5.855
AC015802.6	8.709	8.685	8.877	8.666	9.009	8.801
AC092335.1	5.380	5.434	5.378	5.377	5.560	5.245
AFTPH	11.188	10.873	11.011	11.357	11.058	11.385
AC092809.3	8.686	8.747	8.689	8.588	8.871	8.860
AC092809.4	10.611	10.700	10.759	10.627	10.646	10.594
AC092809.5	7.349	7.422	7.233	7.445	7.303	7.370
AL355377.1	5.367	5.093	5.050	5.411	5.143	5.264
AL355377.2	4.473	4.542	4.555	4.577	4.715	4.534
ZC3H13	8.102	8.247	8.051	7.773	7.747	7.636
ZC3H15	11.497	11.552	11.616	11.637	11.809	11.276
MIR3620	11.573	11.676	11.818	11.566	11.526	11.550
AL109804.1	5.489	5.485	5.430	5.924	5.704	5.638
HDHD5-AS1	5.601	5.807	5.745	5.856	5.550	5.684
AC004967.2	9.849	9.926	10.029	9.810	9.880	9.911
HMMR-AS1	5.581	5.698	5.636	5.552	5.871	5.694

PIPOX	6.302	6.374	6.164	6.446	6.175	6.528
LINC01215	3.825	3.929	4.051	3.872	4.034	4.337
ATP1A4	6.367	6.678	6.447	6.305	6.139	6.299
ATP1A3	6.833	6.740	7.112	7.259	7.247	7.233
ATP1A2	6.910	6.784	6.827	7.306	7.090	6.967
ATP1A1	12.317	12.394	12.622	12.247	12.109	12.283
LINC01210	10.870	10.884	10.750	10.971	11.034	11.030
SPHK2	8.281	8.352	8.288	8.151	8.149	8.162
SPHK1	8.457	8.456	8.631	8.305	8.498	8.392
DCTN1-AS1	4.072	4.058	4.066	3.895	4.131	4.018
LINC01579	5.089	4.939	4.828	5.132	5.108	5.100
AC005355.2	6.596	6.887	6.724	6.974	6.695	6.771
BEGAIN	6.783	7.151	7.052	6.977	6.959	7.018
TP53TG5	7.600	7.688	7.724	8.193	7.945	7.794
TP53TG3	7.710	7.765	7.579	7.766	7.843	7.804
IFT80	9.442	9.424	9.308	8.839	8.807	9.080
IFT81	8.677	8.653	8.702	8.086	8.193	8.402
IFT88	8.416	8.516	8.571	8.269	8.361	8.287
XPA	7.126	7.303	7.110	6.909	7.089	6.904
KISS1R	5.687	5.372	5.425	5.464	5.472	5.432
ATG4C	7.964	8.223	8.248	7.986	7.958	8.200
ATG4B	9.959	9.976	10.082	9.951	9.798	10.014
ATG4A	8.799	8.972	8.794	8.869	8.878	8.888
AL445074.1	4.217	3.954	3.900	4.117	4.104	3.981
ATG4D	8.231	8.214	8.264	8.028	8.292	8.270
GPT2	8.782	8.894	9.088	8.806	8.768	8.567
ARL4AP2	9.945	9.938	9.983	10.057	10.090	9.903
CREB3	10.034	10.157	10.320	10.053	10.131	10.179
MIR4500HG	5.712	5.988	5.631	6.018	5.815	5.959
AL365440.2	4.385	4.319	4.365	4.342	4.315	4.449
AL604028.1	7.185	7.045	7.165	6.977	7.089	6.986
NAALAD2	5.199	5.094	4.994	5.381	4.983	5.257
AC100830.3	7.446	7.379	7.261	7.320	7.581	7.489
HMG20A	9.864	9.903	10.003	9.789	9.846	9.855
HMG20B	9.029	9.164	9.304	8.764	8.624	8.824
CREB1	9.282	9.394	9.377	9.332	9.442	9.258
ZFP82	5.445	5.525	5.339	5.488	5.512	5.576
AL031676.1	4.577	4.562	4.634	4.661	4.764	4.783
NRXN3	7.624	7.449	7.344	7.517	7.646	7.732
NRXN2	6.674	6.704	7.063	6.959	7.017	6.811
NRXN1	6.464	6.811	6.478	6.500	6.843	6.492
DDR1-DT	4.173	4.127	3.984	3.838	4.026	4.148
ELOC	11.037	11.209	11.436	11.275	11.038	11.078
AC107021.1	4.216	4.169	4.079	4.006	4.309	4.371
AC107021.2	5.420	5.490	5.275	5.322	5.495	5.615
STAC3	7.624	7.650	7.782	7.994	7.807	7.783
AL929601.3	5.279	5.017	5.088	5.269	5.142	5.236
C11orf1	9.409	9.447	9.522	9.254	9.373	9.199
DPYSL5	7.628	7.554	7.705	7.584	7.722	7.704
DPYSL2	11.178	11.202	11.275	10.918	10.799	11.093
DPYSL3	8.968	8.909	9.009	8.622	8.429	8.653
AC108519.1	4.288	4.328	4.236	4.374	4.410	4.272
ARHGEF7-AS2	4.509	4.304	4.656	4.410	4.421	4.526
TMC4	11.667	11.674	11.756	11.512	11.455	11.474
NSL1	9.929	9.922	10.085	9.994	10.150	9.869
TMC6	10.377	10.435	10.671	10.578	10.435	10.429

TMC7	7.866	7.659	7.914	7.851	7.956	7.846
APIP	10.132	10.169	10.215	10.237	10.132	10.185
TMC1	5.775	5.852	5.946	6.030	5.714	6.018
TMC2	8.886	8.965	9.098	9.013	9.069	9.120
TMC8	8.262	8.042	8.357	8.318	8.406	8.204
MLF1	10.188	10.220	10.463	10.254	10.388	10.338
MLF2	11.239	11.341	11.452	11.244	11.180	11.218
FANCF	9.101	9.211	9.277	8.968	9.004	9.127
AL451074.1	11.077	10.993	10.934	10.744	10.813	10.860
RSPH1	7.500	7.573	7.617	7.534	7.421	7.629
AC093330.1	5.927	5.865	6.012	6.212	5.984	6.065
LARP4B	9.696	9.823	9.814	9.895	9.934	9.682
ASGR1	6.677	6.582	6.716	6.845	6.775	6.691
ASGR2	7.732	7.649	7.353	7.534	7.706	7.599
API5	9.927	9.940	10.006	9.714	9.852	9.843
UBE2NL	10.574	10.712	10.489	10.640	10.636	10.630
HLA-DPA1	8.558	8.571	8.509	8.443	8.220	8.233
HLA-DPA2	6.784	7.005	7.264	7.227	7.167	7.203
KIAA1191	10.466	10.438	10.536	10.172	10.189	10.183
ALOX12P2	7.429	7.345	7.064	7.293	7.242	7.278
ADARB2-AS1	8.423	8.611	8.363	8.323	8.600	8.611
DENND4C	10.323	10.166	10.284	10.106	9.934	10.084
CA5A	7.250	7.199	7.397	7.262	7.320	7.248
RPS4XP16	6.378	6.443	6.651	6.609	6.681	6.699
C19orf25	8.677	8.834	9.040	8.509	8.564	8.581
ITGB1BP1	8.984	8.921	9.187	8.959	8.917	9.040
ITGB1BP2	7.030	7.092	7.223	7.202	7.156	7.096
AC005394.1	7.384	7.563	7.372	7.525	7.726	7.715
ZAP70	6.837	6.759	6.724	7.081	7.026	6.954
LINC02269	4.978	4.868	5.007	5.248	4.992	4.876
LINC02264	5.757	5.842	5.791	5.880	5.844	5.817
SYNDIG1	6.946	6.527	6.425	6.880	6.913	6.862
AC068533.4	10.587	10.697	10.757	10.415	10.305	10.377
LINC02260	8.183	8.107	7.947	8.362	8.474	8.359
CT476828.8	8.052	7.820	8.258	8.487	8.319	8.298
CT476828.9	8.052	7.820	8.258	8.487	8.319	8.298
NEMP1	9.243	9.262	9.348	9.049	8.966	8.986
RAC3	6.716	6.945	6.978	6.965	7.106	6.864
NEMP2	7.373	7.213	7.283	7.359	7.312	7.166
CT476828.2	8.052	7.820	8.258	8.487	8.319	8.298
CT476828.3	8.052	7.820	8.258	8.487	8.319	8.298
CT476828.1	8.052	7.820	8.258	8.487	8.319	8.298
CT476828.6	8.052	7.820	8.258	8.487	8.319	8.298
CT476828.7	8.052	7.820	8.258	8.487	8.319	8.298
CT476828.4	8.052	7.820	8.258	8.487	8.319	8.298
CT476828.5	8.052	7.820	8.258	8.487	8.319	8.298
CPLX2	6.095	6.086	6.207	6.215	6.145	6.205
CPLX3	7.592	7.722	7.383	7.629	7.636	7.761
CPLX1	5.676	5.744	5.814	5.524	5.922	5.540
AC107294.3	7.573	7.505	7.240	7.479	7.477	7.530
AC107294.2	7.910	7.788	7.731	7.887	7.957	7.936
AC107294.1	6.163	6.319	6.170	6.203	6.523	6.451
PBX2	9.489	9.726	9.865	9.745	9.686	9.524
PBX3	7.429	7.208	7.534	7.112	7.455	7.377
PBX1	8.910	9.078	9.112	8.657	8.921	8.827
AIRE	7.501	7.490	7.505	7.448	7.556	7.433

TRAV39	6.215	6.143	6.092	6.356	6.227	6.250
PRAMEF4	7.847	8.084	8.166	8.407	8.226	8.207
PRAMEF5	7.847	8.084	8.166	8.407	8.226	8.207
PRAMEF6	7.847	8.084	8.166	8.407	8.226	8.207
TUBG2	7.426	7.424	7.465	7.180	7.160	7.234
FAM71F1	6.077	6.092	6.142	6.426	6.295	6.322
PRAMEF1	7.406	7.569	7.636	7.983	7.893	7.730
PRAMEF2	7.406	7.569	7.636	7.983	7.893	7.730
FAM71F2	6.805	6.712	6.862	6.736	6.882	6.867
PRAMEF9	7.847	8.084	8.166	8.407	8.226	8.207
CR589904.2	5.474	5.321	5.217	5.170	5.371	5.423
AC093895.2	5.200	5.322	5.389	5.187	5.398	5.001
KMT5B	9.861	9.803	9.874	9.694	9.801	9.649
KMT5C	9.328	9.208	9.410	9.609	9.572	9.448
KMT5A	8.975	8.923	9.166	9.006	8.941	8.856
AL158835.3	6.713	6.953	6.740	6.766	6.840	6.898
AL158835.2	3.706	3.866	3.951	3.719	3.818	3.605
CLEC14A	8.125	8.319	8.415	8.708	8.293	8.290
UXT	11.726	11.761	11.966	11.765	11.707	11.689
FHAD1-AS1	5.915	5.708	5.996	5.864	5.933	5.576
SHLD3	4.754	4.625	4.940	4.605	5.055	4.785
AC253536.7	10.518	10.608	10.873	10.576	10.453	10.549
CLCN3	10.672	10.738	10.718	10.714	10.605	10.632
CLCN2	8.233	8.263	8.341	8.108	8.232	8.216
CLCN1	7.464	7.607	7.490	7.686	7.609	7.479
CLCN7	10.473	10.416	10.521	10.383	10.342	10.341
CLCN6	7.966	8.035	8.058	7.891	8.025	8.012
CLCN5	9.391	9.271	9.304	8.833	8.995	9.051
CLCN4	7.496	7.636	7.520	7.718	7.490	7.678
AC121761.1	7.345	7.206	7.379	7.464	7.213	7.481
OR10D3	7.380	7.377	7.256	7.189	7.430	7.568
DNAJA2	10.428	10.529	10.570	10.506	10.464	10.565
DNAJA3	11.137	11.129	11.178	10.887	10.969	10.978
DNAJA1	12.418	12.468	12.579	12.611	12.505	12.569
DNAJA4	10.173	10.180	10.228	10.299	10.203	10.338
GALR3	8.825	9.025	8.862	8.845	8.983	9.034
GALR2	4.873	5.005	5.246	5.022	5.080	4.941
GALR1	4.633	4.531	4.597	4.921	4.691	4.620
UTRN	9.881	9.819	9.919	9.497	9.488	9.655
MRPL24	10.929	11.033	11.198	11.007	10.899	10.943
MRPL27	10.880	10.946	11.119	10.828	10.684	10.770
MRPL21	10.581	10.679	10.843	10.829	10.794	10.644
MRPL20	10.731	10.707	10.927	10.570	10.677	10.596
MRPL23	10.767	10.843	11.072	10.720	10.660	10.651
MRPL22	9.735	9.829	9.932	9.659	9.701	9.694
MRPL28	10.307	10.164	10.385	10.069	9.885	10.088
AC068620.2	4.627	4.566	4.540	4.543	4.631	4.656
NPTX2	4.921	5.109	5.107	5.162	5.263	5.396
NPTX1	7.261	7.418	7.517	7.404	7.580	7.493
AC007349.3	4.914	4.974	4.714	4.903	4.937	5.014
CHD1L	9.580	9.639	9.692	9.483	9.340	9.409
RNF130	9.473	9.375	9.580	9.163	9.254	9.289
RAPH1	10.323	10.388	10.288	9.991	10.083	10.148
RNF133	6.453	6.364	6.531	6.534	6.602	6.666
RNF135	8.578	8.616	8.734	8.241	8.298	8.444
RNF138	9.466	9.396	9.554	9.338	9.151	9.377

RNF139	10.089	10.239	10.319	10.374	10.366	10.338
GID4	7.770	7.802	7.736	7.417	7.695	7.651
ARNTL2-AS1	3.931	3.994	4.022	3.925	3.891	4.109
TSHZ2	8.027	7.945	8.281	8.316	8.205	8.342
TSHZ3	7.707	7.547	7.630	7.686	7.795	7.505
TSHZ1	8.339	7.950	8.221	8.147	7.952	8.218
GID8	11.254	11.385	11.525	11.351	11.224	11.243
MRC2	8.343	8.295	8.347	8.498	8.545	8.417
AC099342.1	5.037	5.204	5.211	5.309	5.189	5.043
NPTXR	7.498	7.312	7.484	7.271	7.213	7.560
AC005537.2	6.281	6.543	6.266	6.332	6.254	6.471
MYO1H	6.181	6.022	6.035	6.488	6.514	6.394
MYO1G	7.465	7.496	7.644	7.703	7.637	7.562
MYO1F	7.346	7.415	7.310	7.306	7.350	7.323
MYO1E	10.115	10.227	10.203	9.911	9.692	9.923
MYO1D	10.666	10.778	10.851	10.489	10.356	10.400
MYO1C	10.999	11.005	11.113	11.054	11.165	10.944
MYO1B	9.945	9.980	9.930	10.026	9.881	10.059
MYO1A	6.581	6.838	6.935	6.752	6.991	6.801
DENND6B	6.816	6.875	6.867	6.791	6.873	6.973
BCR	9.921	10.012	10.102	9.789	9.776	9.794
DENND6A	9.504	9.510	9.542	9.236	9.155	9.207
PGBD4	6.535	6.725	6.491	6.632	6.580	6.792
PGBD5	10.019	10.066	10.142	9.817	9.831	9.900
PGBD2	7.017	6.621	6.764	6.800	6.601	6.752
CTPS2	8.760	9.133	8.892	8.440	8.645	8.676
CTPS1	9.492	9.555	9.584	9.440	9.432	9.389
LGALSL	9.164	9.285	9.338	9.207	9.089	9.292
PRSS3P1	9.332	9.504	9.629	9.337	9.422	9.489
AL118558.3	6.372	6.013	6.474	6.177	6.442	6.273
AL118558.1	4.638	4.470	4.345	4.544	4.523	4.455
NEMF	8.910	8.917	8.814	8.745	8.653	8.743
PRSS3P3	7.306	7.202	7.209	7.354	7.550	7.313
AC118281.1	7.622	7.782	7.511	7.207	6.952	7.426
FER1L6-AS1	6.126	5.934	6.107	6.033	6.146	6.243
LGALS9	9.523	9.505	9.574	9.420	9.559	9.398
LGALS8	9.576	9.565	9.536	9.545	9.465	9.617
LGALS3	14.115	14.140	14.182	14.023	14.283	14.059
LGALS2	6.887	6.700	7.147	7.252	6.956	6.822
LGALS1	9.371	9.488	9.867	9.389	9.543	9.582
LGALS7	7.046	6.951	7.071	7.095	7.070	7.015
LGALS4	12.879	13.016	13.069	12.924	12.883	12.853
NMU	7.661	7.487	7.719	7.198	7.482	7.471
HLX	5.922	5.986	6.048	6.035	6.015	6.190
AC018865.3	5.900	5.822	5.573	6.014	5.896	5.903
AC211476.5	9.531	9.523	9.413	9.362	9.364	9.406
NMB	9.112	9.113	9.415	9.226	9.309	9.175
MYO19	9.759	9.666	9.716	9.585	9.537	9.477
ELN	6.911	6.999	7.108	7.008	7.101	7.125
MYO16	5.841	5.985	5.699	5.842	6.115	5.891
TRNT1	7.396	7.298	7.664	7.001	7.228	7.245
NMI	10.490	10.616	10.643	10.552	10.620	10.424
AL022238.4	10.779	10.928	11.052	10.878	10.712	10.839
BMPR1AP2	8.096	8.160	8.093	7.947	7.908	8.072
ATG101	9.695	9.652	9.742	9.679	9.632	9.420
LPGAT1	9.413	9.526	9.484	9.028	9.146	9.297

DIO2-AS1	6.553	6.673	5.733	6.470	6.558	6.405
ELL	7.693	8.029	8.180	7.932	8.183	8.048
AC125494.1	6.981	7.374	7.340	7.371	7.394	7.362
MID1IP1	10.657	10.594	10.763	10.683	10.831	10.631
TBC1D3	10.803	10.689	10.641	10.716	10.587	10.876
C20orf187	6.213	6.076	5.631	6.011	6.022	6.172
ILKAP	8.636	8.685	8.915	8.702	8.516	8.629
C20orf181	7.109	6.848	6.900	7.020	7.094	7.018
WRAP53	9.293	9.461	9.426	9.139	9.186	9.258
JCHAIN	4.144	4.051	4.266	4.130	4.290	4.141
TAX1BP1	12.618	12.638	12.776	12.659	12.574	12.613
TAX1BP3	11.157	11.201	11.309	10.904	10.861	11.008
TBC1D4	8.903	8.843	8.842	8.824	8.708	8.774
PTGS2	8.312	8.337	8.367	8.595	8.440	8.544
PTGS1	7.410	7.518	7.440	7.468	7.446	7.428
FP236241.2	7.923	7.782	7.902	7.606	7.461	7.712
ZFAND6	11.160	11.120	11.194	11.199	11.142	11.293
ZFAND5	11.027	11.127	11.192	11.068	10.998	11.013
ZFAND4	4.756	4.623	4.676	4.575	4.854	4.737
ZFAND3	9.564	9.656	9.767	9.546	9.633	9.519
ZFAND1	8.821	8.879	8.840	8.572	8.433	8.667
SLC39A12	4.396	4.508	4.151	4.266	4.286	4.361
CEP128	7.182	7.111	7.172	6.760	6.921	6.865
SLC39A10	11.390	11.413	11.370	11.210	11.030	11.112
SLC39A11	10.182	10.280	10.320	10.036	10.165	10.217
SLC39A14	10.356	10.460	10.390	10.267	10.212	10.338
CEP120	8.270	8.223	8.275	7.848	7.779	8.002
EIF1AX	11.622	11.764	11.789	11.778	11.868	11.642
EIF1AY	4.670	4.670	4.535	4.553	4.696	4.593
CEP126	7.339	7.493	7.365	7.086	7.378	7.301
EIF1AD	9.985	9.914	9.933	9.908	10.004	9.849
SMAD4	8.848	8.934	8.944	8.661	8.715	8.787
AC004674.1	5.404	5.445	5.437	5.459	5.390	5.575
EVI5	7.809	7.951	7.816	7.583	7.651	7.660
AC135178.3	7.297	7.223	7.161	7.277	7.261	7.615
MYSM1	8.361	8.101	8.000	7.946	7.861	8.282
BMP7	8.295	8.427	8.542	8.466	8.349	8.365
BMP6	7.323	7.420	7.151	7.284	7.427	7.474
BMP5	6.957	7.101	6.939	6.979	6.991	7.078
BMP4	9.284	9.565	9.539	9.419	9.672	9.373
BMP3	6.810	6.695	6.941	7.068	6.872	6.674
BMP2	8.727	8.501	8.595	8.508	8.777	8.695
BMP1	8.208	8.315	8.451	8.440	8.409	8.424
SETP14	12.391	12.429	12.609	12.268	12.431	12.157
DR1	9.800	9.837	9.961	9.844	9.919	9.827
MTURN	7.360	7.286	7.402	7.328	7.482	7.384
TERF2	10.013	10.071	10.103	9.922	10.119	9.864
TERF1	8.985	8.971	9.168	8.832	8.933	8.613
RASSF9	7.656	7.767	7.478	7.433	7.275	7.404
RASSF8	6.628	6.385	6.775	6.283	6.356	6.270
RASSF1	8.254	8.294	8.325	8.106	8.107	8.203
RASSF3	11.520	11.422	11.574	11.353	11.521	11.355
RASSF2	6.256	6.229	5.967	6.049	6.263	6.358
RASSF5	7.804	7.792	7.711	7.567	7.672	7.737
RASSF4	7.993	8.093	8.208	8.207	8.134	8.093
RASSF7	10.974	11.057	11.233	10.947	10.980	11.016

RASSF6	9.569	9.546	9.580	9.306	9.274	9.377
CHD1	10.557	10.318	10.370	10.147	10.109	10.229
CHD3	10.422	10.414	10.562	10.508	10.265	10.378
CHD2	9.486	9.432	9.572	9.572	9.530	9.686
CHD5	7.611	7.544	7.711	7.838	7.881	7.867
CHD4	10.135	10.116	10.112	10.074	10.089	9.898
CHD7	9.292	9.207	9.288	9.216	9.367	9.208
CHD6	7.926	8.030	8.007	7.925	7.830	8.035
CHD9	10.555	10.640	10.596	10.545	10.613	10.374
CHD8	9.506	9.502	9.587	9.478	9.594	9.516
CAP1	12.347	12.256	12.418	12.216	12.161	12.332
CAP2	8.701	8.609	8.590	8.520	8.601	8.574
SKIV2L	9.089	9.131	9.178	8.922	8.923	9.066
TTC5	9.094	9.198	9.452	9.300	9.225	9.190
TTC4	9.030	9.142	9.157	8.960	9.016	9.070
TTC6	7.290	7.258	7.135	7.167	7.043	7.217
TTC1	9.735	9.667	9.808	9.794	9.616	9.796
CENPBD1P1	9.862	9.752	9.798	9.588	9.846	9.725
TTC3	11.354	11.287	11.264	11.065	11.002	10.983
PIKFYVE	8.811	8.728	8.750	8.478	8.412	8.580
TTC9	9.758	9.822	9.920	9.725	9.699	9.765
TTC8	8.334	8.571	8.637	8.205	8.227	8.374
CAPS	10.881	10.975	11.325	10.932	10.938	10.957
CHDH	9.474	9.299	9.528	9.509	9.426	9.667
CAPG	9.665	9.752	10.053	9.479	9.622	9.574
AC008982.3	9.570	9.409	9.585	9.177	9.091	9.158
AC008982.1	9.570	9.409	9.585	9.177	9.091	9.158
PRDM16	6.528	6.653	6.749	6.638	6.721	6.769
MPG	9.443	9.618	9.857	9.609	9.654	9.467
PRDM14	7.925	7.763	7.864	7.913	8.089	8.075
PRDM15	8.197	8.333	8.080	7.935	8.234	8.188
PRDM12	7.634	7.607	7.802	7.634	7.711	7.841
PRDM13	7.535	7.693	7.533	7.327	7.385	7.650
PRDM10	7.822	7.982	8.001	8.016	8.003	7.981
PRDM11	8.713	8.734	8.895	9.193	9.061	8.998
SLC22A31	7.988	7.805	7.864	7.962	8.151	8.042
MPL	7.164	7.200	7.100	7.086	7.216	7.139
AVP	7.649	7.546	7.983	7.706	8.017	7.753
MPI	9.925	9.993	10.148	9.700	9.694	9.787
U47924.1	7.369	7.617	7.535	7.503	7.660	7.534
POLRMT	9.158	9.288	9.334	9.079	9.087	9.035
TRPC5OS	5.023	4.754	4.815	4.818	4.882	4.753
AC240274.1	11.009	11.042	10.885	10.932	10.739	11.001
MCPH1	7.448	7.508	7.559	7.328	7.607	7.420
GRPEL1	9.905	9.898	10.086	9.824	9.723	9.790
GRPEL2	9.239	9.334	9.297	9.277	9.163	9.188
SRGAP2B	9.147	8.918	9.207	8.677	8.913	8.476
SRGAP2C	9.147	8.918	9.207	8.677	8.913	8.476
QRSL1	8.966	9.137	9.151	8.836	8.770	8.819
FAM49A	7.232	7.217	7.137	7.204	7.220	7.088
FAM49B	10.347	10.459	10.519	10.425	10.499	10.492
ZFP36L1	11.596	11.565	11.657	11.416	11.401	11.524
ZFP36L2	11.533	11.427	11.607	11.461	11.477	11.582
C10orf99	6.454	6.468	6.476	6.884	6.790	6.837
C10orf90	6.604	6.708	6.516	6.861	6.712	6.723
C10orf91	6.482	6.575	6.466	6.663	6.492	6.578

C10orf95	7.654	7.597	7.511	7.619	7.656	7.737
ADH7	6.146	6.319	6.310	6.545	6.392	6.222
ADH6	7.685	7.780	7.961	7.843	7.898	7.793
ADH5	11.234	11.316	11.409	11.194	11.194	11.109
ADH4	6.722	6.493	6.334	6.541	6.405	6.495
AL157414.2	7.343	7.320	7.255	7.446	7.305	7.359
AC243807.2	9.490	9.579	9.608	9.338	9.546	9.512
AC243807.4	6.669	6.541	6.555	6.856	6.841	6.802
HERC2P3	5.765	5.748	5.288	5.775	5.707	5.775
RMI1	8.497	8.651	8.651	8.255	7.965	8.238
CORO6	6.430	6.233	6.347	6.485	6.558	6.489
CORO7	7.663	7.656	7.908	7.955	7.905	7.688
CIART	7.483	7.487	7.443	7.623	7.685	7.802
FAM155B	9.019	8.919	9.063	8.970	8.935	8.906
TYMP	9.224	9.278	9.441	9.213	9.343	9.236
MIR6850	14.026	14.047	14.096	14.092	13.969	14.080
FAM155A	6.290	6.242	6.034	6.109	6.060	6.135
NPM1P6	13.203	13.272	13.262	13.249	13.199	13.294
ACOT11	8.251	8.373	8.385	8.287	8.253	8.169
ACOT12	4.670	4.408	4.568	4.859	4.398	4.642
ACOT13	9.818	9.884	10.134	9.950	9.916	9.908
BCORP1	5.121	5.016	5.002	5.071	5.216	5.201
FAM153CP	8.275	8.445	8.343	8.661	8.412	8.386
REPIN1	10.344	10.417	10.517	10.180	10.180	10.256
MIR302CHG	6.683	6.626	6.864	7.044	7.444	6.745
PMVK	10.036	10.066	10.249	9.968	9.903	9.908
GRIN2B	7.163	7.122	7.347	7.174	7.298	7.083
GRIN2C	7.448	7.640	7.389	7.574	7.692	7.687
GRIN2A	9.166	9.207	9.062	9.047	9.140	8.938
LINC02465	4.331	4.132	4.004	4.129	4.259	4.024
KLHDC8B	8.381	8.422	8.363	8.323	8.296	8.388
GRIN2D	7.312	7.767	7.523	7.542	7.645	7.661
LINC02466	5.139	5.359	5.318	5.390	5.316	5.396
RN7SL657P	4.821	4.465	4.819	4.353	4.470	4.547
IL6ST	9.921	9.936	9.781	9.700	9.665	9.855
SBF1	9.944	9.955	10.176	9.928	9.762	10.012
SBF2	9.627	9.682	9.808	9.440	9.471	9.521
AC084262.1	5.509	5.511	5.565	5.513	5.485	5.748
DBN1	10.061	10.018	10.194	10.065	10.064	9.970
CDC73	9.520	9.619	9.603	9.532	9.529	9.606
LMBRD2	9.707	9.619	9.748	9.210	9.174	9.389
LMBRD1	10.734	10.783	10.949	10.740	10.783	10.756
AC074143.1	6.504	6.493	6.591	6.144	6.221	6.192
TAS2R16	6.133	6.361	6.021	6.445	6.321	6.379
TAS2R10	5.139	5.135	5.375	5.295	5.295	5.259
BCAT2	10.391	10.400	10.562	10.137	9.929	10.139
BCAT1	8.415	8.422	8.378	8.528	8.393	8.274
TAS2R13	6.327	6.306	6.275	6.379	6.414	6.504
DBNL	10.215	10.301	10.438	10.284	10.293	10.268
AL049838.1	4.824	4.637	4.416	4.779	4.633	4.624
CYP26A1	7.740	7.873	7.831	7.890	7.918	7.861
PSTK	5.797	5.939	6.150	5.635	5.893	5.966
PHPT1	10.156	10.186	10.391	10.078	10.117	10.120
NFU1	9.288	9.473	9.639	9.288	9.378	9.322
RRP1B	9.559	9.558	9.640	9.237	9.242	9.472
NOVA2	9.354	9.280	9.118	9.232	9.189	9.328

NOVA1	6.443	6.594	6.268	6.483	6.545	6.535
HSP90B1	12.780	12.827	12.783	12.735	12.763	12.887
MCF2L	10.135	9.967	9.579	10.274	10.276	10.231
VSX1	6.814	6.852	6.879	7.006	6.831	6.861
DIO1	4.783	4.612	4.392	4.945	4.776	4.828
DIO2	7.466	7.644	7.603	7.740	7.809	7.786
DIO3	8.097	8.272	8.297	8.340	8.214	8.207
Z97634.1	7.571	7.743	7.591	7.574	7.657	7.694
KDM7A-DT	7.070	7.216	7.172	7.108	7.211	7.137
RPL26P37	11.640	11.725	11.844	11.776	11.676	11.746
RPL26P30	9.842	9.903	10.118	9.835	9.985	9.933
CR933844.1	7.344	7.403	7.456	7.273	7.478	7.510
BRF2	10.365	10.525	10.673	10.444	10.464	10.425
CYSTM1	12.390	12.494	12.688	12.475	12.443	12.384
BRF1	8.417	8.434	8.561	8.387	8.282	8.237
CAMSAP2	9.415	9.505	9.523	9.290	9.191	9.497
CAMSAP3	8.031	8.189	8.306	8.182	8.319	8.121
CAMSAP1	8.812	8.630	8.667	8.727	8.603	8.638
AL162595.1	7.485	7.290	7.577	7.584	7.322	7.568
RRP12	8.656	8.617	8.606	8.552	8.614	8.556
RRP15	9.430	9.548	9.613	9.430	9.501	9.420
RNF185-AS1	7.404	7.629	7.175	7.143	7.757	7.679
IPO11	9.469	9.527	9.479	9.432	9.388	9.498
ATOH7	5.735	5.803	5.766	5.733	5.772	5.831
AC113189.2	8.222	8.148	8.089	8.445	8.289	8.295
ACAT2	10.578	10.734	10.888	10.750	10.693	10.843
AC113189.7	8.222	8.148	8.089	8.445	8.289	8.295
AC113189.5	8.222	8.148	8.089	8.445	8.289	8.295
AC113189.4	8.222	8.148	8.089	8.445	8.289	8.295
CD200R1	7.803	7.814	7.720	7.967	7.972	7.724
LIMD1	9.019	9.012	9.011	8.980	8.961	9.038
ATOH8	8.618	8.612	8.426	8.823	8.695	8.616
AC069360.1	7.907	7.907	7.805	7.851	8.021	8.222
SPANXA2-OT1	4.995	4.794	4.887	5.080	5.152	4.909
TOGARAM2	7.009	7.148	7.047	7.132	7.219	7.204
TOGARAM1	8.351	8.528	8.487	8.318	8.363	8.531
TFAP2A-AS1	7.657	7.864	7.603	8.000	7.912	7.932
PLAAT4	10.968	11.170	11.281	11.111	11.216	11.089
PLAAT5	7.604	7.582	7.664	7.648	7.744	7.713
SINHCAF	10.864	11.025	11.133	11.044	10.944	10.892
PLAAT1	8.594	8.797	8.752	8.053	8.366	8.438
PLAAT2	6.692	6.783	6.805	6.528	6.776	6.923
PLAAT3	12.025	12.132	12.249	12.061	11.885	11.985
IL2RA	7.669	7.533	7.426	7.634	7.443	7.678
IL2RB	7.540	7.615	7.449	7.462	7.660	7.698
ZFYVE27	8.982	8.987	9.149	8.933	8.926	8.932
TPCN1	10.636	10.595	10.684	10.456	10.387	10.474
TPCN2	8.522	8.420	8.664	8.748	8.728	8.559
IL2RG	8.270	8.217	8.200	8.495	8.476	8.622
ZFYVE28	7.359	7.636	7.642	7.713	7.744	7.674
MDH1B	6.698	6.666	6.253	6.669	6.626	6.565
CMTR2	9.358	9.501	9.582	9.332	9.254	9.505
CMTR1	9.119	9.093	9.251	9.118	9.106	9.053
ERC1	9.272	9.329	9.277	9.076	9.473	9.110
ERC2	5.346	5.514	5.523	5.384	5.623	5.472
BHMT2	6.221	6.261	6.101	5.997	6.378	6.202

SLC37A2	6.445	6.549	6.370	6.815	6.579	6.589
SLC37A3	9.652	9.699	9.756	9.573	9.570	9.701
CDK18	8.150	8.284	8.512	8.187	8.171	8.295
CDK19	9.163	9.253	9.271	9.494	9.387	9.076
SLC37A4	8.937	8.913	8.952	8.652	8.809	8.872
CDK12	9.560	9.575	9.715	9.180	9.286	9.353
CDK13	9.990	9.959	10.020	10.019	9.998	9.967
CDK10	8.740	8.798	8.981	8.592	8.626	8.788
CDK16	10.387	10.442	10.584	10.506	10.596	10.315
CDK17	9.538	9.520	9.591	9.506	9.519	9.454
CDK14	9.392	9.402	9.306	9.383	9.336	9.302
CDK15	8.777	8.702	8.194	8.874	9.065	8.907
AC011297.1	6.798	6.816	6.674	7.104	7.269	7.009
AC008687.4	6.333	6.095	5.983	6.277	6.301	6.546
AC008687.1	7.556	7.653	7.596	8.049	7.638	7.796
BFSP1	7.069	6.890	7.080	7.323	7.050	7.107
FAM216B	4.951	5.146	5.297	5.102	5.118	5.011
FAM216A	8.861	8.956	9.002	8.850	8.837	8.821
MMP28	9.621	9.669	9.572	9.788	9.907	9.726
MMP25	7.847	7.752	7.877	8.179	8.090	7.900
MMP24	8.317	8.407	8.641	8.607	8.362	8.628
AF188030.1	7.285	7.469	7.416	7.560	7.631	7.587
MMP26	7.333	7.280	6.876	7.173	7.282	6.976
MMP21	4.131	3.993	4.110	4.221	3.892	4.056
MMP20	5.277	5.028	5.067	5.397	5.336	5.291
AL662907.1	5.702	5.694	5.558	5.784	5.713	5.776
KARS1	12.793	12.875	12.960	12.843	12.796	12.799
TCAIM	9.761	9.823	9.881	9.658	9.709	9.816
SESTD1	10.450	10.471	10.595	10.506	10.645	10.116
MMP23B	6.468	6.496	6.586	6.439	6.699	6.685
MMP23A	6.468	6.496	6.586	6.439	6.699	6.685
B3GNT4	8.130	8.389	8.392	8.351	8.305	8.239
B3GNT5	10.168	10.224	10.174	10.376	10.408	10.355
B3GNT6	7.624	7.798	7.732	7.549	7.740	7.939
B3GNT7	6.670	6.608	6.867	6.519	6.710	6.391
B3GNT2	9.666	9.651	9.718	9.628	9.506	9.591
B3GNT3	10.708	10.776	10.990	10.842	10.803	10.712
TSFM	9.725	9.770	9.962	9.715	9.639	9.552
B3GNT8	7.244	7.149	7.462	7.685	7.415	7.444
B3GNT9	7.865	7.933	8.159	7.750	8.059	7.993
AC097376.3	6.420	6.722	6.590	6.398	6.613	6.499
AC245041.2	6.604	6.528	6.502	6.549	6.169	6.541
AC245041.3	6.604	6.528	6.502	6.549	6.169	6.541
GINM1	11.119	11.087	11.247	10.993	11.374	10.737
TBCCD1	8.815	8.910	9.027	8.436	8.773	8.675
AL078645.2	8.085	7.914	8.156	8.198	8.242	8.270
DAAM1	8.497	8.672	8.693	8.300	8.350	8.241
DAAM2	7.683	7.835	7.773	7.733	7.920	7.974
HLA-DRB1	9.931	10.022	10.001	9.890	10.024	9.958
HLA-DRB6	8.411	8.478	8.540	8.382	8.489	8.490
HLA-DRB4	8.068	8.058	8.127	7.897	8.192	8.061
HLA-DRB5	9.281	9.398	9.504	9.163	9.287	9.157
YIF1B	9.611	9.821	9.985	9.464	9.568	9.593
KRT7-AS	9.565	9.738	9.910	9.693	9.828	9.721
YIF1A	11.067	11.175	11.270	10.839	10.928	10.859
TMCO5B	6.446	6.310	6.178	6.688	6.265	6.482

TUFT1	9.732	9.858	9.916	9.639	9.634	9.543
TMCO5A	7.228	6.918	7.189	7.273	7.096	7.267
AL133371.1	6.186	6.050	6.376	6.323	6.707	6.374
SOCS2-AS1	5.768	5.802	5.842	6.060	5.954	5.742
FN1	9.661	9.646	9.645	9.514	9.300	9.582
MYCNOS	6.099	6.488	6.330	6.574	6.517	6.389
RHO	9.144	9.098	8.819	9.275	9.330	9.201
RHD	8.276	8.336	8.390	8.350	8.469	8.533
CAVIN3	8.813	8.864	9.062	8.863	8.800	8.738
AL133352.1	10.422	10.485	10.671	10.481	10.327	10.363
CAVIN1	8.726	8.830	9.015	8.607	8.824	8.878
AC100849.1	5.995	6.131	6.056	6.132	6.211	6.119
CR381653.1	3.848	3.989	3.892	4.180	4.103	4.156
COLQ	6.854	6.696	6.675	6.795	6.720	6.646
AC005034.4	6.029	5.794	5.871	5.024	5.535	5.774
FAM189A2	7.923	8.002	7.925	8.043	7.958	8.002
FAM189A1	6.926	6.709	6.702	7.003	7.013	6.803
AL132656.3	4.886	4.983	4.868	4.902	4.771	4.903
YJEFN3	6.110	6.360	6.354	6.358	6.309	6.400
AL132656.4	8.092	8.058	7.677	7.989	8.158	8.132
AP000696.1	7.323	7.362	7.385	7.209	7.461	7.439
TECRP1	10.572	10.597	10.870	10.317	10.192	10.372
AC067931.2	6.301	6.683	6.432	6.601	6.638	6.532
AC008764.1	5.153	5.104	5.056	5.307	5.114	5.160
AC008764.4	8.766	8.861	9.075	8.725	8.683	8.861
AC008764.7	5.792	5.930	5.955	5.787	5.882	6.001
AC008764.6	6.456	6.524	6.143	6.063	6.494	6.437
AC093668.1	9.289	9.336	9.445	9.220	9.197	9.291
AC011466.4	5.833	6.049	5.944	5.913	6.057	6.080
MRPS35	10.875	10.911	11.000	10.986	10.808	10.939
TBC1D1	8.903	9.011	8.984	8.858	8.899	8.809
KRT75	7.089	7.157	7.164	7.321	7.210	7.320
KRT74	7.424	7.512	7.428	7.572	7.568	7.526
KRT77	6.378	6.279	6.204	6.189	6.399	6.167
KRT76	7.576	7.738	7.564	7.662	7.680	7.548
KRT71	6.892	6.972	6.879	6.699	7.026	7.032
KRT73	7.678	7.879	7.818	7.604	7.797	7.741
KRT72	6.610	6.728	6.702	6.961	6.707	6.797
AL118508.1	6.524	6.530	6.551	6.551	6.645	6.426
KRT79	7.087	7.276	7.437	7.330	7.566	7.440
KRT78	7.101	7.292	7.281	7.252	7.307	7.609
TBC1D5	9.683	9.544	9.709	9.416	9.617	9.485
ZNF571	5.907	5.962	6.052	6.122	6.138	5.847
HECW2-AS1	3.849	3.980	4.025	3.888	4.131	3.944
AAMP	9.868	9.951	10.027	9.652	9.526	9.706
HYPK	12.250	12.325	12.464	12.351	12.356	12.378
FUNDC2P1	10.153	10.226	10.319	10.438	10.375	10.360
AL513327.2	7.107	7.007	6.796	7.092	7.184	7.159
U2SURP	10.322	10.334	10.369	10.333	10.268	10.252
IL6R	7.521	7.411	7.617	7.418	7.459	7.499
CNOT6L	9.610	9.433	9.454	9.378	9.547	9.425
FAXC	5.804	5.711	6.174	5.792	5.876	5.882
VPS35P1	10.910	10.914	10.852	10.901	11.118	10.799
AC020909.2	7.146	7.166	7.052	7.066	7.348	7.331
AC020909.1	9.372	9.354	9.386	9.088	8.998	9.017
SLC16A6P1	6.229	6.298	6.131	6.096	6.316	6.194

PTK2	10.527	10.517	10.572	10.332	10.293	10.442
PTK7	8.499	8.431	8.714	8.553	8.662	8.383
PTK6	9.341	9.487	9.434	9.162	9.391	9.195
PRKAA2	7.691	7.729	7.998	7.455	7.549	7.414
PRKAA1	8.970	9.140	9.027	8.761	8.778	8.843
ITGB3BP	8.968	9.039	9.273	8.880	8.821	8.964
AOPEP	9.457	9.279	9.462	9.378	9.356	9.444
ATG16L1	8.578	8.524	8.691	8.630	8.728	8.576
ATG16L2	8.043	8.077	7.938	7.925	8.059	8.063
C4orf54	4.903	4.996	5.025	5.379	5.180	5.102
AC008663.2	5.940	6.311	5.944	6.400	6.185	6.378
SPECC1L-ADOR/	6.318	6.558	6.534	6.708	6.499	6.555
C15orf62	6.558	6.672	6.787	6.418	6.842	6.719
C15orf61	10.030	10.227	10.275	9.854	9.807	9.961
C15orf65	7.643	7.482	7.572	7.534	7.582	7.478
AL137786.1	5.974	5.922	6.018	5.784	5.880	5.915
DDX23	9.248	9.261	9.332	9.039	9.183	9.035
DDX21	11.346	11.500	11.510	11.327	11.370	11.198
DDX20	8.718	8.731	8.768	8.506	8.555	8.575
ACVR2B-AS1	6.381	6.521	6.426	6.693	6.690	6.621
ZNF157	4.095	4.136	4.062	4.060	3.906	3.902
DDX25	5.718	5.421	5.357	6.057	5.670	5.738
DDX24	11.205	11.283	11.307	11.241	11.129	11.253
DDX28	9.179	9.163	9.358	9.055	9.126	9.059
AC004079.1	4.868	4.812	4.761	4.963	5.150	4.855
AC004079.2	4.868	4.812	4.761	4.963	5.150	4.855
AC004079.4	5.042	5.186	5.169	5.001	5.403	5.070
AC004079.5	4.868	4.812	4.761	4.963	5.150	4.855
RAB3IP	9.944	10.011	10.086	9.919	9.936	9.962
AFF1	9.709	9.667	9.824	9.546	9.485	9.435
TRAV6	5.671	5.706	5.625	5.723	5.881	5.670
PARD6G	7.431	7.535	7.584	7.220	7.452	7.531
TRUB1	9.728	9.769	9.766	9.853	9.793	9.848
TRUB2	9.166	9.293	9.542	9.032	9.334	9.065
PARD6B	10.461	10.450	10.362	10.267	10.325	10.393
PARD6A	8.984	8.887	9.287	9.004	9.126	8.959
AL731567.2	7.511	7.389	7.410	7.801	7.769	7.628
PEX26	8.794	8.969	9.144	8.997	8.938	8.597
AL731567.1	7.511	7.389	7.410	7.801	7.769	7.628
AC091488.1	6.600	6.810	6.683	6.754	6.997	6.988
AC011306.1	7.772	7.932	7.718	7.953	7.953	8.132
SPEN	9.754	9.699	9.569	9.651	9.708	9.772
SPEG	7.902	7.937	8.094	8.177	8.142	8.181
MANEAL	10.314	10.480	10.532	10.236	10.373	10.179
AC091182.1	6.773	6.889	6.547	6.789	6.904	6.924
STOML2	10.219	10.233	10.516	10.331	10.257	10.330
STOML1	9.169	9.235	9.309	9.130	9.189	9.041
FBLIM1	9.098	9.070	9.148	8.851	9.051	8.983
TIMM8A	7.419	7.605	7.726	7.568	7.538	7.512
TIMM8B	11.077	11.068	11.190	11.232	11.160	11.252
DAXX	9.805	9.815	9.917	9.547	9.932	9.655
NT5M	6.565	6.620	6.746	6.938	6.752	6.744
NT5C	8.837	8.799	9.082	8.693	8.791	8.688
TBATA	7.843	7.849	7.787	8.023	7.979	7.747
NT5E	9.048	9.177	9.146	9.106	8.991	9.008
AC112236.1	4.584	4.601	4.628	4.619	4.785	4.743

ZNF32-AS3	4.415	4.199	4.433	4.561	4.561	4.473
TMEM200C	6.519	6.368	6.378	6.277	6.649	6.465
AC025428.1	5.614	5.668	5.423	5.446	5.694	5.747
LBX1	4.905	5.068	5.339	5.344	5.111	5.131
CR788250.1	8.068	8.058	8.127	7.897	8.192	8.061
DSCR9	8.140	8.121	7.978	8.376	8.292	8.353
DSCR8	6.279	6.291	6.339	6.311	6.430	6.332
GNAS	14.257	14.214	14.217	14.230	14.244	14.192
DSCR4	7.379	7.645	7.411	7.502	7.603	7.557
AP002840.2	7.672	7.622	7.684	7.679	7.818	7.796
PTCD2	7.876	7.949	8.088	7.725	7.899	7.814
AC015914.1	5.269	5.151	5.208	5.359	5.544	5.413
GNAZ	7.799	7.910	8.038	7.785	7.900	7.914
PTCD1	8.941	8.924	8.994	8.982	8.989	8.876
VEZF1P1	9.238	9.214	9.214	8.550	9.066	9.184
GNAL	7.242	7.150	7.199	7.277	7.252	7.283
AGFG2	10.167	10.297	10.401	9.965	10.110	10.107
AGFG1	9.618	9.668	9.664	9.546	9.666	9.630
INTS6P1	6.405	6.457	6.604	6.502	6.901	6.494
CRYGS	8.815	8.910	9.027	8.436	8.773	8.675
FST	10.959	10.911	10.921	10.474	10.363	10.560
CRYGN	6.803	6.956	7.111	7.355	7.423	7.254
PWP2	8.752	8.742	8.867	8.463	8.537	8.502
CRYGA	5.881	5.959	5.996	6.226	6.124	6.008
CRYGB	7.607	7.315	7.595	7.777	7.591	7.537
CRYGC	7.067	6.915	6.981	7.170	7.176	7.161
CRYGD	8.269	8.216	8.562	8.715	8.515	8.555
DLG5-AS1	8.576	8.442	8.540	8.719	8.684	8.625
RRNAD1	8.530	8.297	8.483	8.265	8.281	8.317
WASH3P	9.341	9.202	9.345	9.278	9.301	9.395
NMNAT2	7.340	7.296	7.393	7.254	7.453	7.356
NMNAT3	8.306	8.357	8.284	8.548	8.415	8.402
NMNAT1	7.140	7.171	7.136	7.114	7.307	7.082
AC005943.1	11.822	11.891	12.033	11.840	11.901	11.867
UBE2I	12.028	12.151	12.314	11.992	11.882	11.784
PLEKHM1	10.940	11.003	11.143	10.794	10.802	10.788
PLEKHM2	8.221	8.057	8.287	8.117	8.085	8.112
PLEKHM3	6.737	6.533	6.871	6.088	6.509	6.440
FAM72D	9.247	9.156	9.312	8.602	9.021	8.786
FAM72C	9.247	9.156	9.312	8.602	9.021	8.786
FAM72B	9.247	9.156	9.312	8.602	9.021	8.786
FAM72A	9.247	9.156	9.312	8.602	9.021	8.786
LINC00301	5.831	5.465	5.634	5.879	5.943	5.796
AL732372.3	10.722	10.564	10.478	10.430	10.307	10.610
AL732372.2	9.349	9.203	9.195	9.170	9.223	9.503
UBE2N	11.892	11.974	12.128	12.106	12.105	12.052
UPK1A	6.812	7.067	7.185	6.946	7.122	7.013
TREML2	7.802	7.696	7.809	7.827	7.824	7.919
TREML1	7.476	7.759	7.711	7.554	7.653	7.821
GPAA1	11.530	11.562	11.725	11.387	11.364	11.378
TREML4	5.937	6.013	5.655	5.905	5.828	5.920
UBE2C	11.210	11.224	11.413	10.898	11.133	10.821
SNORA44	8.077	7.423	7.713	7.735	7.554	7.718
UBE2B	10.963	11.112	11.187	11.138	11.349	10.930
GPRIN2	7.480	7.512	7.503	7.410	7.378	7.388
GPRIN3	7.279	7.515	7.605	7.423	7.431	7.396

AP005263.1	11.142	11.224	11.372	11.121	10.999	11.129
GPRIN1	8.033	8.036	8.100	8.233	8.065	8.120
ROGDI	8.789	8.862	8.962	8.525	8.765	8.773
SNORA48	8.399	8.627	8.409	8.193	8.186	8.445
FAM236A	6.260	6.062	5.883	6.426	6.185	6.070
FAM236B	6.260	6.062	5.883	6.426	6.185	6.070
RBMY1J	6.498	6.612	6.640	6.865	6.762	6.723
RBMY1B	6.498	6.612	6.640	6.865	6.762	6.723
RBMY1F	6.498	6.612	6.640	6.865	6.762	6.723
RBMY1E	6.498	6.612	6.640	6.865	6.762	6.723
NFIA-AS2	7.153	7.078	7.054	7.410	7.312	7.307
UBE2Z	11.646	11.762	11.832	11.577	11.823	11.374
AC010973.2	10.017	10.193	10.276	10.134	10.246	10.112
SLC44A1	10.776	10.752	10.780	10.735	10.660	10.659
SLC44A2	10.213	10.207	10.313	10.104	10.079	10.100
SLC44A3	9.931	10.001	10.086	9.911	9.870	9.973
SLC44A4	10.119	10.291	10.369	10.018	10.182	10.102
SLC44A5	7.175	7.235	7.074	7.069	6.955	7.228
MSI2	8.941	8.899	8.987	8.706	8.734	8.532
MSI1	6.198	6.166	5.842	6.109	6.096	6.355
ENGASE	9.751	9.741	9.849	9.839	9.737	9.817
CD8B2	7.631	7.749	7.814	7.930	7.803	7.722
ZFHX4	7.628	7.636	7.612	7.567	7.701	7.534
ANKFN1	6.997	6.960	7.042	7.143	7.014	7.116
MRO	7.020	7.023	6.977	7.181	7.196	7.260
COQ9	11.165	11.162	11.353	11.208	11.149	11.186
WT1	6.027	6.101	5.958	5.981	5.901	6.209
ISY1-RAB43	9.271	9.313	9.387	9.158	9.352	9.055
C1S	7.371	7.229	7.100	7.251	7.216	7.224
C1R	6.899	7.105	7.055	7.142	6.974	6.885
COQ7	8.520	8.627	8.776	8.431	8.552	8.494
COQ6	8.422	8.438	8.549	8.393	8.373	8.327
COQ3	8.298	8.482	8.455	8.338	8.613	8.596
COQ2	8.767	8.858	8.821	8.510	8.462	8.594
HBM	6.464	6.937	6.914	6.774	7.008	7.189
HBD	7.243	7.294	7.159	7.313	7.254	7.256
HBB	8.275	8.513	8.286	8.334	8.389	8.578
MUC20	10.442	10.486	10.509	10.224	9.982	10.301
HBZ	6.116	5.837	6.095	6.037	6.134	6.117
WARS1	11.427	11.410	11.524	11.294	11.330	11.209
TPX2	11.260	11.263	11.403	10.846	10.909	10.956
WWC3	10.784	10.887	10.893	10.791	10.633	10.765
WWC2	8.977	8.995	9.131	8.976	9.088	8.741
WWC1	11.104	11.205	11.306	10.978	10.980	11.092
AC025040.1	4.302	4.182	4.162	4.219	4.411	4.216
GPR101	7.030	6.912	7.070	6.994	7.430	7.239
GPR107	9.595	9.556	9.674	9.439	9.277	9.356
GPR108	10.091	10.112	10.187	10.095	10.046	10.073
MFAP3L	7.706	7.551	7.685	7.959	7.996	7.901
SYPL1	10.932	11.084	11.037	10.909	10.852	10.974
BRICD5	8.283	8.424	8.161	8.378	8.463	8.367
LINC02052	4.278	4.298	4.077	4.042	4.243	4.355
LINC02053	5.597	5.769	5.917	6.103	5.739	5.585
LINC02059	4.077	4.039	4.038	4.093	4.042	3.958
BPIFA4P	7.875	7.861	7.680	7.876	7.843	7.845
AC018521.5	8.330	8.433	8.282	8.184	8.235	8.147

AC018521.3	8.033	8.199	7.886	7.899	8.098	8.098
AC018521.1	11.203	11.102	11.318	10.936	10.668	10.967
RNF6	9.766	9.765	9.660	9.511	9.528	9.629
RNF7	11.598	11.672	11.824	11.546	11.375	11.567
RNF4	10.568	10.565	10.664	10.349	10.454	10.379
RNF5	10.061	10.118	10.150	9.869	10.093	10.056
AL121974.1	5.753	5.475	5.475	5.583	5.485	5.530
AVPR2	7.277	7.373	7.228	7.611	7.270	7.375
LRRC63	4.139	3.910	4.138	4.198	3.894	3.955
LRRC61	9.232	9.386	9.346	9.440	9.347	9.254
LRRC66	5.252	5.272	5.331	5.463	5.357	5.357
C7orf33	5.578	5.584	5.609	5.374	5.518	5.534
C7orf31	7.560	7.637	7.613	7.373	7.411	7.386
MSANTD3-TMEFI	4.923	5.117	5.248	4.928	4.937	5.062
RASSF1-AS1	6.526	6.605	6.653	6.872	6.857	6.765
AC067752.1	5.713	5.610	5.212	5.707	5.514	5.686
EPM2A	7.035	7.157	7.133	6.831	7.325	7.173
FAM118A	7.772	7.825	7.846	7.655	7.631	7.779
FAM118B	8.122	8.229	8.320	8.008	8.071	8.191
RECQL	9.244	9.299	9.362	8.681	9.042	8.906
MISP	11.540	11.696	11.736	11.518	11.486	11.577
LINC01505	6.938	6.986	6.905	7.037	7.243	6.966
LINC01504	8.671	8.697	8.715	8.585	8.707	8.752
LINC01503	7.592	7.561	7.498	7.578	7.643	7.689
LINC01500	4.158	4.474	4.517	4.511	4.703	4.506
ACTR8	9.009	9.087	9.151	9.070	9.169	9.191
ACTR6	8.061	8.181	8.197	7.974	7.835	8.136
ACTR5	9.222	9.160	9.137	9.348	9.123	9.178
XPNPEP3	8.728	8.645	8.564	8.307	8.367	8.632
ACTR3	13.031	13.162	13.182	13.145	13.091	13.192
ACTR2	11.504	11.561	11.522	11.338	11.267	11.362
GPANK1	9.317	9.212	9.474	8.782	9.012	9.036
GNRHR	7.038	7.251	6.968	6.950	7.007	7.028
ARRDC1-AS1	7.698	7.740	7.931	7.957	7.915	7.882
SPINK13	5.362	5.293	5.269	5.195	5.417	5.285
HYDIN	7.346	7.509	7.255	7.458	7.374	7.553
C1GALT1C1L	6.071	6.007	6.271	6.144	6.088	6.089
RAPGEFL1	8.402	8.381	8.513	8.347	8.464	8.550
GTDC1	8.670	8.768	8.754	8.716	8.764	8.747
MIATNB	7.059	7.164	7.004	6.866	6.967	7.088
ICOS	6.239	6.254	6.032	6.069	6.077	6.250
ABI3BP	11.466	11.393	11.441	10.961	10.753	10.975
KHDRBS2	7.022	7.099	6.737	7.213	7.421	7.117
IDUA	8.096	8.012	8.055	7.909	8.017	8.005
OR5V1	6.906	6.929	6.728	6.839	6.987	6.802
AP001885.1	7.098	7.161	7.302	7.264	7.620	7.312
GNRH2	7.490	7.631	7.557	7.432	7.661	7.687
GNRH1	5.792	5.932	5.916	5.637	6.055	5.615
MCFD2P1	4.022	4.023	3.877	4.084	4.199	3.804
A1BG-AS1	6.892	6.652	6.849	7.128	6.995	6.931
MIR671	9.039	9.079	9.119	8.874	8.889	9.074
WDSUB1	7.689	7.767	8.009	7.606	7.423	7.539
WNT8A	5.793	6.051	6.055	6.133	6.129	5.942
WNT8B	7.456	7.677	7.613	8.006	7.813	7.704
AL669942.1	7.165	7.227	7.364	7.707	7.106	7.289
GCSHP5	11.692	11.837	11.918	11.632	11.625	11.668

GDE1	10.803	10.654	10.876	10.747	10.658	10.654
ACKR4P1	5.949	5.951	6.022	5.795	6.376	5.837
NPIPA8	11.306	11.174	11.094	11.070	11.186	11.250
NPIPA9	11.306	11.174	11.094	11.070	11.186	11.250
UBA6-AS1	8.075	7.992	8.010	8.077	8.248	8.133
NPIPA5	11.306	11.174	11.094	11.070	11.186	11.250
MINCR	6.432	6.599	6.587	6.592	6.455	6.439
NPIPA7	11.306	11.174	11.094	11.070	11.186	11.250
NPIPA1	11.306	11.174	11.094	11.070	11.186	11.250
NPIPA2	11.306	11.174	11.094	11.070	11.186	11.250
NPIPA3	11.306	11.174	11.094	11.070	11.186	11.250
HIPK3	11.012	11.084	11.037	10.939	10.836	11.034
HIPK2	9.332	9.521	9.454	9.027	9.312	9.122
HIPK1	9.520	9.453	9.756	9.621	9.659	9.301
HIPK4	7.567	7.633	7.468	7.606	7.790	7.570
LEMD3	10.907	10.848	10.922	10.638	10.600	10.560
LEMD2	9.355	9.333	9.426	9.209	9.271	9.107
AC076966.2	3.983	3.843	4.211	4.091	4.086	4.138
ARMH3	9.531	9.495	9.616	9.637	9.480	9.539
ARMH1	6.755	6.858	7.153	6.723	6.933	6.859
ARMH4	4.737	4.612	4.642	4.812	4.685	4.832
NAPG	8.374	8.448	8.459	8.025	8.080	8.132
C3orf18	8.386	8.341	8.292	8.629	8.448	8.368
NAPA	11.447	11.401	11.520	11.276	11.371	11.214
C3orf14	9.830	9.923	10.099	9.745	9.772	9.655
LARP1	11.204	11.663	11.775	11.723	11.698	11.270
LARP4	9.561	9.466	9.397	9.245	9.177	9.426
LARP6	10.459	10.396	10.570	10.121	10.247	10.116
LARP7	9.047	9.034	9.150	8.978	8.849	8.912
RPL19	14.552	14.576	14.608	14.578	14.518	14.533
COL15A1	4.213	4.218	4.282	4.164	4.235	4.140
AL354928.1	5.966	5.985	5.944	6.043	6.019	6.276
LMLN	8.518	8.440	8.308	8.138	8.207	8.509
AP000459.3	7.545	7.546	7.223	7.564	7.656	7.576
ATMIN	10.334	10.395	10.421	10.410	10.444	10.337
LGR5	6.809	7.018	6.390	6.948	7.081	6.973
LGR4	10.061	10.136	10.049	9.793	9.845	10.055
LGR6	7.128	7.362	7.125	7.388	7.328	7.377
CDKN1C	6.722	6.718	6.890	6.640	6.675	6.672
CDKN1B	10.456	10.414	10.553	10.247	10.351	10.260
CDKN1A	10.140	10.078	10.265	10.002	10.019	9.976
AC004890.2	7.136	7.335	7.272	7.022	7.255	7.178
AL139099.4	9.464	9.500	9.588	9.208	9.390	9.346
AC080013.1	6.348	6.228	6.431	6.221	6.294	6.143
AC018692.2	4.236	4.246	4.208	3.994	4.382	4.167
TMEM125	9.976	10.100	10.192	10.078	10.100	10.106
FOXJ1	7.583	7.545	7.715	7.983	7.636	7.777
FOXJ2	9.544	9.381	9.439	9.370	9.484	9.532
FOXJ3	9.572	9.567	9.634	9.471	9.295	9.457
TMEM121	6.547	6.421	6.440	6.394	6.502	6.402
TMEM123	12.220	12.198	12.349	12.095	12.171	12.320
AC060814.2	7.424	7.406	7.675	7.294	7.409	7.495
TMEM129	9.129	9.101	9.261	9.066	8.931	8.921
TMEM128	7.981	8.079	8.214	7.741	8.006	7.734
AL049834.1	7.020	7.330	7.314	7.357	7.212	7.329
SLC35B1	10.717	10.865	10.982	10.862	10.793	10.747

SLC35B3	9.161	9.225	9.286	8.962	8.964	9.164
SLC35B2	11.212	11.317	11.420	11.307	11.284	11.228
SLC35B4	8.315	8.320	8.367	8.047	8.144	8.212
IGFBP6	8.690	8.685	8.837	8.655	8.519	8.721
OR2I1P	7.576	7.417	7.329	7.670	7.991	7.705
PELP1	8.339	8.275	8.481	8.435	8.275	8.293
SH3BP5-AS1	7.622	7.528	7.495	7.546	7.560	7.482
ANXA2P1	7.785	7.862	8.086	8.052	8.005	7.973
NOB1	10.409	10.430	10.647	10.285	10.293	10.153
ANXA2P2	13.101	13.172	13.183	13.190	13.095	13.181
S100A9	6.533	6.612	6.598	6.584	6.670	6.616
S100A8	5.388	5.549	5.529	6.119	5.626	5.496
BIRC6	9.418	9.411	9.337	9.233	9.132	9.288
S100A5	6.562	6.556	6.606	6.572	6.798	6.425
BRWD3	8.592	8.256	8.150	7.921	8.422	8.477
S100A7	6.678	6.762	6.711	6.705	6.925	6.727
BRWD1	8.135	8.064	8.186	7.422	7.766	7.607
S100A1	7.405	7.569	7.578	7.649	7.618	7.598
S100A3	7.595	7.724	7.470	7.619	7.637	7.624
S100A2	7.926	8.074	8.145	7.976	8.104	8.049
LONRF3	8.423	8.397	8.357	8.618	8.408	8.547
LONRF2	7.950	7.857	7.932	7.141	7.284	7.412
LONRF1	9.402	9.497	9.465	9.466	9.330	9.242
TCFL5	9.423	9.476	9.573	9.314	9.271	9.256
AIP	9.683	9.842	10.086	9.759	9.704	9.625
AL672310.1	7.050	7.230	7.077	7.323	7.128	7.096
AC097634.4	10.412	10.523	10.652	10.466	10.462	10.328
PMS2P1	9.531	9.523	9.421	9.474	9.423	9.422
AC091179.4	6.921	7.079	6.981	7.375	7.112	7.090
AC135068.3	4.957	5.051	4.936	4.868	4.936	5.007
PMS2P5	9.313	9.354	9.149	9.020	9.274	9.164
AC135012.3	8.115	8.151	8.201	8.364	8.112	8.244
RNF5P1	10.061	10.118	10.150	9.869	10.093	10.056
EVPLL	7.848	7.650	7.758	8.001	7.965	7.991
LRRIQ1	5.930	5.565	5.891	5.809	5.584	5.640
ZNF366	4.966	4.904	4.770	4.907	4.870	5.029
LRRIQ3	4.503	4.581	4.578	4.979	4.684	4.657
ADAT1	9.764	10.140	10.115	9.665	9.695	9.282
ADAT3	9.552	9.586	9.670	9.777	9.804	9.807
ADAT2	6.627	6.878	6.802	6.709	6.785	6.784
KLHL30-AS1	6.910	6.908	7.100	7.121	7.025	7.199
AC090260.2	5.415	5.460	5.397	5.582	5.532	5.566
AP001180.1	6.354	6.436	6.510	6.357	6.624	6.663
AC114752.2	6.186	6.104	5.845	6.173	6.192	6.104
RBAKDN	7.693	7.648	7.832	8.060	7.926	7.866
NECTIN3-AS1	6.831	7.024	6.959	7.028	7.120	7.045
CLTRN	9.767	9.878	9.769	9.650	9.770	9.751
TUSC7	7.145	7.262	7.011	7.052	7.408	7.261
BRI3BP	11.883	11.929	12.024	11.809	11.665	11.809
TUSC1	10.114	10.034	10.166	9.951	9.998	9.963
TUSC2	9.100	9.106	9.206	9.130	9.112	9.041
AC100768.1	5.474	5.706	5.270	5.565	5.591	5.585
RPGR	6.174	6.041	6.186	5.985	6.048	5.816
TUSC8	4.929	4.920	4.851	5.105	4.937	4.708
AL353997.10	5.967	5.882	6.127	6.072	6.128	6.050
GNG10	10.629	10.741	10.799	10.575	10.709	10.595

GNG11	8.477	8.576	8.696	8.423	8.374	8.476
GNG12	11.800	11.871	11.876	11.633	11.622	11.652
GNG13	5.440	5.456	5.464	5.370	5.469	5.551
URB2	8.159	8.266	8.208	8.007	8.110	7.980
URB1	6.661	6.740	6.734	6.656	6.611	6.749
AC009148.1	6.446	6.500	6.597	6.873	6.569	6.572
NT5C1B	5.911	6.151	5.930	5.937	5.761	6.068
AC079174.2	4.930	4.860	5.173	5.302	5.302	5.207
RIMBP3C	8.535	8.490	8.448	8.513	8.691	8.374
AC242019.1	5.222	5.343	5.304	5.095	5.151	5.261
SEC24D	7.977	7.843	7.967	7.706	7.991	7.847
SEC24A	9.214	9.253	9.209	9.402	9.397	9.271
AC092451.3	4.711	4.698	4.719	4.673	4.885	4.640
SEC24B	9.376	9.460	9.453	9.170	9.074	9.262
C16orf95	8.793	8.867	9.029	8.966	9.193	9.177
C16orf92	6.811	6.740	6.776	6.707	6.868	6.915
C16orf91	9.603	9.580	9.758	9.630	9.582	9.604
TASOR	9.562	9.678	9.543	9.650	9.539	9.563
AL645608.2	6.050	6.028	6.010	5.990	6.032	6.180
ZNF691	8.117	8.171	8.269	8.158	8.361	8.292
ZNF692	8.265	8.183	8.201	7.876	7.842	8.236
ITGB2-AS1	6.398	6.428	6.669	6.889	6.550	6.536
ZNF697	7.946	7.920	7.866	7.506	7.829	7.851
ZNF696	8.018	7.864	8.055	8.245	8.077	8.073
TRIM39-RPP21	8.653	8.718	8.869	8.590	8.637	8.555
COPZ1	11.801	11.831	12.037	11.794	11.842	11.778
COPZ2	8.266	8.331	8.105	8.198	8.280	8.292
RUVBL1	10.757	10.800	10.909	10.744	10.717	10.683
RUVBL2	11.634	11.769	11.965	11.727	11.679	11.631
AC141586.3	7.264	7.243	7.282	7.582	7.588	7.177
AC141586.1	8.417	8.490	8.470	8.472	8.507	8.463
SNORD58A	13.304	13.343	13.490	13.360	13.298	13.417
SNORD58C	7.718	7.647	7.485	7.652	7.707	7.769
GOLGA6L16P	8.131	7.933	7.807	8.082	8.115	8.200
SHB	7.699	7.687	7.686	7.811	7.720	7.719
SHD	6.494	6.753	6.572	6.674	6.569	6.636
SHE	8.038	7.637	7.501	8.084	8.043	8.014
SHF	7.283	7.375	7.404	7.283	7.259	7.462
SHH	7.242	7.296	7.171	7.294	7.323	7.266
TMEM14C	11.497	11.554	11.614	11.559	11.704	11.507
PHEX	7.434	7.382	7.728	7.742	7.781	7.710
DYNC2H1	7.253	7.209	7.526	7.222	7.301	7.247
AC092650.1	4.673	4.837	4.604	4.797	4.901	5.068
RNASEH2CP1	7.707	7.736	7.571	7.931	7.857	7.764
NUDCD2	8.625	8.489	8.749	8.043	7.963	8.409
NUDCD3	10.813	10.847	11.000	10.598	10.644	10.667
NUDCD1	9.363	9.507	9.452	9.373	9.308	9.471
IGKV1OR2-2	8.398	8.520	8.424	8.478	8.571	8.522
SEC22B3P	8.653	8.456	8.743	8.348	8.393	8.317
IGKV1OR2-1	8.398	8.520	8.424	8.478	8.571	8.522
OR1D5	6.260	6.296	6.110	6.295	6.586	6.323
OR1D4	6.260	6.296	6.110	6.295	6.586	6.323
GMNC	7.077	7.060	6.988	7.015	6.910	7.072
OR1D2	7.563	7.764	7.608	7.824	7.712	7.742
GMNN	10.563	10.604	10.796	10.547	10.444	10.432
MPLKIP	10.367	10.410	10.489	10.292	10.310	10.369

TIMMDC1	9.872	9.923	10.082	9.958	10.051	9.990
ZNF343	7.901	7.967	7.785	7.974	8.005	7.841
ZNF341	7.728	7.849	7.876	7.784	7.744	7.728
ZNF347	4.588	4.670	4.725	4.602	4.703	4.846
ZNF346	8.329	8.411	8.426	8.118	8.352	8.272
ZNF345	7.919	7.962	8.148	8.274	8.297	8.449
AC016027.1	5.897	5.721	5.829	5.869	5.876	5.882
RANP1	12.806	12.875	13.026	12.910	12.802	12.842
NUDT12	7.843	7.780	7.938	7.850	7.672	7.829
DLL1	7.060	7.038	7.220	7.241	7.175	7.346
NUDT10	6.502	6.479	6.191	6.349	6.530	6.482
DLL3	7.390	7.564	7.429	7.334	7.448	7.470
DLL4	8.383	8.554	8.728	8.822	8.683	8.575
NUDT17	8.373	8.388	8.178	8.349	8.336	8.326
NUDT14	9.059	9.053	9.371	9.005	9.069	8.895
NUDT15	8.550	8.536	8.642	8.212	8.264	8.295
FRMD6-AS1	6.604	6.666	6.543	6.441	6.417	6.273
NUDT18	7.873	7.906	8.097	7.771	7.603	7.778
NUDT19	10.326	10.360	10.143	10.042	10.112	10.108
STX8	9.058	9.041	9.281	8.965	9.023	9.170
AL162390.2	4.455	4.295	4.361	4.486	4.506	4.198
IFI6	11.121	10.597	10.800	9.884	10.157	10.062
STX3	11.260	11.306	11.354	11.200	11.226	11.287
STX2	9.215	9.243	9.427	9.028	9.158	9.230
STX5	8.879	8.840	9.061	8.753	8.896	8.892
STX4	9.333	9.343	9.457	8.941	9.124	9.137
STX7	9.748	9.799	9.771	9.527	9.585	9.625
STX6	9.904	9.832	9.835	9.744	9.626	9.974
RPS3A	13.488	13.531	13.648	13.817	13.801	13.836
CFHR2	8.141	8.112	8.270	8.032	8.136	8.252
AC026748.3	7.692	7.829	7.922	7.915	7.760	7.824
AC004593.1	5.972	6.057	6.138	6.375	6.243	6.185
AC004593.2	8.252	8.297	8.476	8.390	8.260	8.317
LGI4	8.634	8.747	8.675	8.757	8.818	8.807
LGI1	4.558	4.307	4.261	4.592	4.561	4.503
LGI2	7.660	7.550	7.366	7.822	7.776	7.797
LGI3	7.681	7.969	7.840	7.692	7.751	7.733
CLIC3	5.868	5.426	5.533	5.632	6.049	5.827
DRG2	9.325	9.354	9.455	9.264	9.357	9.251
DRG1	11.465	11.682	11.785	11.619	11.624	11.566
LRSAM1	7.849	7.900	7.962	7.890	7.805	7.705
CLIC1	12.367	12.363	12.578	12.326	12.266	12.305
PHYKPL	9.240	9.321	9.270	9.160	9.176	9.096
GNPTG	9.774	9.889	10.136	9.852	9.804	9.731
TCP11	6.272	6.205	6.077	6.184	5.917	6.202
TCP10	6.740	6.620	6.910	7.118	6.949	6.778
AC041040.1	6.467	6.612	6.480	6.366	6.480	6.581
AL138759.1	7.564	7.512	7.393	7.480	7.485	7.143
TBC1D22B	8.420	8.466	8.461	8.499	8.390	8.643
LINC00167	6.082	5.904	5.901	5.965	5.954	5.777
LINC00165	6.811	7.104	7.181	6.907	7.165	7.116
LINC00163	7.431	7.570	7.498	7.360	7.476	7.414
LINC00161	7.157	6.977	7.165	7.250	7.189	7.338
NKTR	9.914	9.798	9.758	9.596	9.704	9.539
TMEM143	9.181	8.967	9.088	9.084	9.070	9.155
KCNIP2-AS1	8.256	8.234	8.106	8.650	8.511	8.511

TMEM140	8.274	8.259	8.333	8.358	8.119	8.152
AC147067.3	6.875	6.712	6.857	6.703	6.788	6.864
AC022384.1	7.004	7.051	6.911	6.952	6.853	6.965
KIF2A	8.831	8.869	8.911	8.612	8.679	8.600
KIF2C	10.113	10.114	10.231	9.652	9.680	9.726
KIF2B	5.991	6.092	5.920	6.007	5.809	6.249
ENSA	12.095	12.186	12.305	12.044	12.081	12.087
AC068547.1	10.443	10.642	10.657	10.716	10.753	10.320
BRCA1	8.978	8.778	8.911	8.343	8.412	8.377
LY6G6F-LY6G6D	7.290	7.415	7.551	7.424	7.504	7.555
BRCA2	6.823	6.912	6.695	6.400	6.382	6.458
AC131935.2	7.415	7.089	7.238	7.717	7.569	7.757
AC091059.2	6.095	5.972	6.053	6.382	6.125	6.156
AC027601.1	8.060	8.098	7.951	8.077	8.173	8.227
KIF25	7.491	7.625	7.469	7.519	7.598	7.813
KIF27	4.833	4.736	4.964	4.767	4.975	5.036
MAATS1	6.913	7.133	7.004	7.239	7.088	7.160
KIF23	8.999	8.831	8.952	7.997	8.245	8.398
KIF22	8.753	8.653	8.950	8.443	8.458	8.425
JKAMP	10.217	10.119	10.332	10.068	10.082	10.076
AC064852.1	5.046	4.960	4.999	5.020	5.138	5.013
AL022476.1	6.503	6.484	6.495	6.333	6.399	6.524
AP000432.2	4.539	4.442	4.296	4.629	4.606	4.374
AP000432.3	4.539	4.442	4.296	4.629	4.606	4.374
LINC00836	6.757	6.811	6.804	6.701	6.848	6.861
LINC00837	3.980	4.041	3.921	4.092	4.168	4.043
LINC00838	5.493	5.477	5.456	5.601	5.556	5.607
LINC00839	6.899	7.010	6.721	7.056	7.111	7.133
AL031601.2	5.861	5.785	5.791	6.273	6.001	6.099
SMURF1	8.481	8.383	8.386	8.343	8.220	8.350
MIR6788	7.589	7.701	7.485	7.532	7.774	7.685
SMURF2	8.804	8.668	8.618	8.453	8.717	8.369
MIR6782	8.533	8.641	8.854	8.746	9.081	8.474
AC097637.1	6.761	6.880	6.941	6.630	6.942	7.036
TFRC	13.372	13.468	13.482	13.294	13.262	13.350
AKT1S1	9.999	9.966	10.162	9.861	9.879	9.872
TFR2	7.446	7.463	7.463	8.083	7.683	7.459
FLT3	6.948	7.193	6.792	7.395	7.199	7.090
DNAJC25-GNG1(	10.629	10.741	10.799	10.575	10.709	10.595
AC060766.5	6.789	6.901	6.785	6.856	7.000	6.781
AC012146.1	6.708	6.792	7.021	6.855	6.666	6.719
AC060766.7	6.789	6.901	6.785	6.856	7.000	6.781
YTHDF3-AS1	6.877	7.075	7.362	7.192	6.850	7.045
CAAP1	10.204	10.187	10.292	10.106	10.077	10.181
AC073585.2	6.385	6.784	6.656	6.591	6.656	6.762
YY1	12.056	12.038	12.151	11.846	12.027	11.628
YY2	7.271	7.234	7.250	7.187	7.319	7.249
AQP4-AS1	6.588	6.483	6.589	6.628	6.660	6.638
SNORD89	7.180	7.128	7.011	6.891	7.136	7.067
NPHP3-AS1	7.153	7.314	7.206	7.133	7.350	7.478
NOL4L	10.231	10.229	10.220	10.029	9.906	10.098
AC080129.2	6.337	6.390	6.272	6.453	6.357	6.414
SUSD6	10.671	10.629	10.654	10.476	10.265	10.511
SYCE3	6.365	6.447	6.597	6.406	6.526	6.601
SYCE2	6.878	6.887	7.044	6.906	6.930	6.931
FAM106A	8.816	8.726	8.703	8.342	8.439	8.740

CCDC38	6.017	5.989	5.912	6.138	5.789	5.903
CCDC39	3.978	3.941	4.039	4.101	4.001	4.016
CCDC32	7.967	8.057	8.306	8.029	8.248	8.172
CCDC33	7.270	7.615	7.421	7.518	7.616	7.477
CCDC30	6.991	6.946	6.581	7.230	7.078	7.015
CCDC36	7.465	7.558	7.486	7.678	7.660	7.854
CCDC34	9.116	8.942	9.283	8.724	8.795	8.647
AC004944.1	5.556	5.738	5.675	5.801	5.751	5.718
LINC02150	6.310	6.405	6.325	6.293	6.380	6.298
LINC02153	5.496	5.691	5.731	5.741	6.020	5.859
LINC02152	5.944	6.027	6.127	6.018	6.348	6.364
LINC02158	4.341	4.147	4.353	4.294	4.451	4.383
SLC7A14-AS1	4.424	4.532	4.527	4.633	4.546	4.745
ZMAT2	10.678	10.760	10.902	10.554	10.704	10.530
MLNR	5.185	5.059	4.919	5.166	5.059	4.949
SARS2	8.927	9.033	9.010	8.781	8.753	8.701
SARS1	11.407	11.533	11.577	11.338	11.222	11.321
NECAP1	9.611	9.705	9.737	9.543	9.492	9.640
NECAP2	9.440	9.503	9.659	9.384	9.410	9.379
MYBL1	7.661	7.493	7.601	8.079	7.760	7.717
SMOC2	7.874	7.901	7.427	8.091	7.838	7.936
AC006011.2	9.440	9.472	9.672	9.351	9.466	9.363
KYNU	9.783	9.830	9.754	9.902	9.673	9.833
UVRAG	8.735	8.833	8.889	8.958	8.859	8.776
COPG2	6.864	7.116	7.415	6.882	6.710	6.698
COPG1	10.465	10.438	10.614	10.415	10.232	10.284
KERA	4.215	4.090	4.098	4.343	4.354	3.975
GTPBP4	10.370	10.387	10.300	10.076	9.923	10.131
GTPBP6	9.365	9.367	9.606	9.237	9.165	9.214
GTPBP1	9.654	9.640	9.849	9.282	9.446	9.331
GTPBP3	8.591	8.630	8.741	8.351	8.668	8.553
GTPBP2	8.839	8.951	8.942	8.508	8.748	8.697
SPART-AS1	5.626	5.636	5.322	5.564	5.656	5.529
GTPBP8	8.754	8.983	9.035	8.768	9.019	8.694
TIPIN	9.058	9.176	9.208	8.849	8.961	8.818
NAMPTP1	9.937	10.044	10.054	10.061	10.251	10.049
GU182357.4	8.835	8.689	8.488	8.744	8.793	8.879
AC090197.1	7.836	7.937	8.040	7.947	8.111	8.080
TMEM88	7.622	7.686	7.609	7.613	7.728	7.744
PLK1	8.786	8.594	9.028	8.750	8.842	8.467
PLK3	7.815	7.815	7.874	7.963	7.907	7.967
PLK2	8.778	8.849	8.881	8.655	8.747	8.734
PLK5	7.642	7.754	7.546	7.756	7.619	7.748
PLK4	8.293	8.361	8.293	8.055	8.217	8.149
STX16-NPEPL1	8.644	8.723	8.917	8.981	8.801	8.830
AL160236.1	11.499	11.540	11.608	11.335	11.371	11.429
POLQ	8.447	8.502	8.360	8.125	8.376	8.434
CWC15	10.480	10.382	10.637	10.388	10.455	10.350
LINC01711	6.683	7.002	6.875	7.119	7.296	6.977
GPT	7.421	7.299	7.597	7.529	7.483	7.587
LINC01718	7.916	7.948	8.038	8.305	8.174	8.151
LINC01719	7.195	6.888	6.932	7.044	7.164	7.014
KLHL29	8.343	8.332	8.343	8.283	8.175	8.359
AL121990.1	8.035	8.050	7.597	8.103	7.970	8.190
AC016396.2	6.109	6.372	6.248	6.081	6.444	6.473
PCGF1	8.755	8.742	8.821	8.872	9.046	8.903

POLG	8.519	8.534	8.669	8.967	8.521	8.742
PCGF3	8.331	8.211	8.360	8.214	8.112	8.090
PCGF2	10.921	11.009	11.128	10.706	10.701	10.741
PCGF5	8.653	8.651	8.475	8.396	8.558	8.494
CX3CR1	6.244	6.089	6.445	6.510	6.653	6.421
PCGF6	7.158	7.238	7.118	6.970	7.089	7.100
MAGED1	11.520	11.591	11.683	11.298	11.200	11.211
MAGED2	9.145	9.292	9.497	9.048	9.030	9.160
MAGED4	7.555	7.615	7.676	7.555	7.742	7.929
ATP8B3	7.521	7.427	7.594	7.230	7.387	7.398
ATP8B2	8.134	8.120	8.223	8.474	8.168	8.239
ATP8B1	9.987	10.021	9.986	9.841	9.796	9.954
ATP8B4	5.203	5.324	5.384	5.119	5.067	5.188
STC1	11.620	11.778	11.979	11.575	11.712	11.393
STC2	9.501	9.631	9.591	9.605	9.534	9.454
NRDC	10.951	10.805	11.000	10.878	10.820	10.877
AL161910.1	6.415	6.486	6.391	6.747	6.605	6.425
AL353729.1	7.452	7.412	7.614	7.138	7.195	7.223
DPPA2	5.373	5.351	5.339	5.338	5.438	5.322
DPPA3	4.039	4.108	4.021	3.947	4.005	4.017
DPPA4	4.581	4.501	4.431	4.579	4.525	4.455
DPPA5	5.869	5.902	6.165	6.009	6.078	5.992
TLR10	7.052	7.039	6.901	7.160	7.327	7.020
ZBTB39	7.573	7.471	7.504	7.564	7.585	7.651
ZBTB38	10.373	10.355	10.600	10.433	10.603	10.244
H3P36	13.752	13.863	13.907	13.791	13.836	13.535
NEUROG3	7.095	7.086	7.249	7.499	7.432	7.258
NEUROG2	5.014	4.837	4.714	4.931	4.888	4.851
NEUROG1	7.172	7.152	7.397	7.357	7.414	7.540
ZBTB32	6.147	6.098	6.133	6.158	6.158	6.143
LINC00445	6.842	7.167	7.023	6.914	7.117	7.011
ZBTB34	7.547	7.890	7.837	7.463	7.613	7.744
ZBTB37	7.544	7.597	7.487	7.418	7.576	7.557
SMC1A	11.074	11.136	11.137	10.706	10.845	10.857
SMC1B	5.345	5.305	5.252	5.398	5.393	5.371
MDK	8.862	8.895	9.136	8.848	8.839	8.974
AGAP11	9.081	9.018	9.114	9.210	9.131	9.065
SGTB	6.085	6.218	6.175	5.654	5.958	5.811
ANKRD44-IT1	4.749	4.766	4.357	4.625	4.666	4.691
AL137026.1	5.957	5.829	5.779	5.773	6.019	5.919
DIPK2B	7.737	7.687	7.467	7.685	7.881	7.832
TRIM48	5.367	5.231	5.211	5.384	5.336	5.373
DIPK2A	10.094	10.141	10.342	10.054	10.040	10.244
TRIM46	7.363	7.428	7.440	7.390	7.506	7.431
TRIM47	9.773	9.808	9.841	9.949	9.891	9.863
TRIM44	10.592	10.739	10.874	10.538	10.584	10.273
TRIM45	7.633	7.655	7.582	7.585	7.694	7.521
TRIM42	4.663	4.966	4.847	4.682	4.663	4.817
TRIM43	6.793	6.730	6.758	6.858	6.755	6.999
TRIM40	5.368	5.394	5.025	5.238	5.223	5.261
AC010894.5	9.376	9.069	8.681	9.298	9.261	9.101
IVD	9.038	9.131	9.196	9.097	9.262	8.957
IVL	7.886	7.752	7.533	8.012	7.922	7.819
NEB	10.927	10.799	10.791	9.939	9.902	10.137
AL591848.3	7.084	6.952	7.002	7.057	7.190	7.107
AL591848.4	5.237	5.207	4.996	5.106	5.099	5.479

DIO3OS	7.155	7.143	7.003	7.487	7.094	7.167
FAM155A-IT1	4.510	4.280	4.482	4.339	4.548	4.558
AC020916.3	8.515	8.644	8.710	8.753	8.650	8.722
AC020916.1	8.515	8.644	8.710	8.753	8.650	8.722
AC026951.1	8.627	8.424	8.680	8.312	8.478	8.527
AURKAIP1	10.909	10.917	11.192	10.971	10.884	10.876
AFMID	7.149	7.110	6.855	7.151	7.209	7.219
AL353708.1	4.576	4.796	4.432	4.828	5.169	4.627
AL353708.3	6.750	6.838	6.615	7.008	7.000	6.911
AC074183.1	9.003	9.179	9.324	8.937	9.073	8.986
B3GALNT1	8.847	9.065	9.090	8.752	8.822	8.974
AC074183.2	9.003	9.179	9.324	8.937	9.073	8.986
PFDN1	10.409	10.554	10.630	10.583	10.404	10.367
PFDN2	10.426	10.553	10.650	10.349	10.249	10.219
PFDN5	10.837	10.827	10.999	11.055	10.922	11.072
PFDN4	10.187	10.201	10.385	10.072	10.136	10.111
PFDN6	8.967	9.033	9.115	8.713	8.865	8.988
MTHFR	9.423	9.429	9.547	8.996	9.471	9.211
MTHFS	10.325	10.242	10.373	10.338	10.373	10.257
GAGE12H	7.444	7.371	7.139	7.545	7.560	7.476
GAGE12J	7.444	7.371	7.139	7.545	7.560	7.476
SNRPGP10	10.774	10.898	11.100	11.006	10.933	10.986
DTNB	7.802	8.055	7.986	7.902	7.892	8.031
SNRPGP15	10.774	10.898	11.100	11.006	10.933	10.986
DTNA	8.304	8.292	8.244	8.325	8.284	8.382
MEIKIN	5.286	5.356	5.123	5.393	5.460	5.294
GAGE12C	7.444	7.371	7.139	7.545	7.560	7.476
GAGE12D	7.444	7.371	7.139	7.545	7.560	7.476
GAGE12E	7.444	7.371	7.139	7.545	7.560	7.476
GAGE12F	7.444	7.371	7.139	7.545	7.560	7.476
GAGE12G	7.444	7.371	7.139	7.545	7.560	7.476
AC110373.1	7.665	7.890	7.811	7.720	7.785	7.940
AC092718.8	11.692	11.837	11.918	11.632	11.625	11.668
AC092718.3	11.692	11.837	11.918	11.632	11.625	11.668
AC092718.1	4.137	4.112	4.059	4.130	4.153	4.162
AC092718.4	9.743	9.650	9.842	9.575	9.506	9.432
PDE4DIP	7.864	7.954	8.095	8.039	7.953	7.939
LATS2	9.947	10.036	10.192	10.022	10.256	9.722
LATS1	7.645	7.670	7.797	7.268	7.582	7.608
DDOST	12.502	12.555	12.637	12.369	12.367	12.476
CSKMT	7.619	7.724	7.654	7.713	7.699	7.731
IL17A	6.390	6.454	6.373	6.632	6.202	6.490
AC092611.1	7.015	6.873	6.699	6.893	7.117	7.082
SDHCP4	10.439	10.670	10.712	10.761	10.730	10.686
MCOLN1	8.686	8.870	8.872	8.600	8.628	8.574
MCOLN3	8.654	8.714	8.656	8.284	8.204	8.361
MCOLN2	8.232	8.479	8.348	8.190	8.509	8.260
NEXN-AS1	4.480	4.314	4.400	4.188	4.294	4.444
MAJIN	7.223	7.186	7.151	7.492	7.166	7.299
PHYHIPL	7.057	7.138	7.178	7.040	7.229	7.084
AP005131.6	6.874	6.991	6.897	6.829	7.042	7.102
PPP4R3A	9.590	9.629	9.493	9.708	9.542	9.672
PPP4R3B	9.778	9.932	9.811	9.828	9.769	9.982
PPP4R3C	4.006	3.742	3.787	3.890	3.924	3.947
TRAV22	4.644	4.800	4.763	4.574	4.862	4.729
TRAV21	6.673	6.523	6.384	6.695	6.850	6.660

RTN4R	8.183	8.253	8.442	8.196	8.117	8.109
TRAV27	7.898	8.181	7.810	8.175	8.147	8.113
TRAV25	4.802	4.644	4.531	5.139	4.802	4.899
TRAV24	5.359	5.548	5.487	5.580	5.743	5.568
PRSS36	8.671	8.796	8.764	8.743	8.781	8.810
PRSS35	4.053	4.048	4.013	4.395	4.144	4.066
TAF1A	6.346	6.511	6.457	5.829	6.250	6.040
AL356019.2	5.942	5.740	6.019	5.289	5.170	5.593
MON2	9.545	9.392	9.432	9.136	9.104	9.218
RBMS3-AS3	5.048	5.165	4.862	5.041	5.041	5.164
AL662854.2	8.614	8.670	8.743	8.558	8.568	8.605
TRIOBP	10.867	11.066	11.239	10.903	11.011	10.728
TAF1D	9.327	8.974	9.129	8.945	9.033	9.112
AC002310.2	6.990	7.002	7.142	7.423	6.995	6.969
C21orf59-TCP10L	9.815	9.779	9.947	9.643	9.806	9.477
AL445665.1	8.464	8.364	8.135	8.347	8.305	8.294
TBCAP1	12.149	12.164	12.246	11.882	11.970	12.050
HMGN5	8.893	8.881	8.942	8.278	8.479	8.622
HMGN4	11.046	11.072	11.048	10.936	10.866	10.985
HMGN3	11.520	11.501	11.632	11.416	11.297	11.509
HMGN2	12.569	12.690	12.791	12.495	12.553	12.522
HMGN1	11.925	11.994	12.146	11.893	11.885	11.984
MX2	10.064	10.059	10.000	9.813	10.127	9.738
MX1	12.366	12.298	12.335	12.138	12.257	12.050
ACVRL1	6.775	6.964	6.838	6.939	6.900	6.931
CHL1	4.792	4.765	4.740	4.628	4.910	4.746
CRIPT	8.662	8.705	8.722	8.531	8.671	8.575
AC092803.1	5.619	5.480	5.677	5.520	5.648	5.467
EFTUD2	11.353	11.362	11.441	11.103	10.983	11.059
AL161729.1	8.235	8.046	8.136	8.476	8.333	8.337
CBY1	9.150	9.032	9.226	9.001	8.955	9.057
CBY2	6.782	6.850	6.969	6.780	6.714	6.732
TMEM8B	7.973	8.193	8.207	8.222	8.036	7.954
ERLIN1	10.443	10.480	10.619	10.320	10.382	10.466
ERLIN2	10.953	11.002	11.027	10.789	10.753	10.768
CAHM	4.983	5.029	4.971	5.197	5.382	5.125
CRIP1	6.407	6.535	6.627	6.491	6.398	6.485
CRIP3	7.103	6.926	7.003	7.104	7.102	7.047
CRIP2	9.444	9.455	9.720	9.014	9.202	9.208
NUDT16L1	8.659	8.663	8.809	8.660	8.598	8.656
PLP1	5.570	5.616	5.502	5.804	5.631	5.552
PLP2	11.863	11.925	12.101	11.853	11.795	11.829
CLEC5A	5.235	5.176	5.035	5.240	5.134	5.011
AL049555.1	9.174	9.220	9.260	8.814	8.954	9.069
AC096751.1	8.033	7.869	8.010	7.916	7.938	8.005
FAM41C	7.510	7.529	7.579	7.400	7.484	7.379
TNFAIP8	8.763	9.012	8.951	8.764	9.023	8.727
SPART	7.590	7.635	7.370	7.579	7.524	7.696
AL139089.1	5.431	5.383	5.661	5.532	5.777	5.667
C21orf91-OT1	6.211	6.265	6.482	6.228	6.571	6.337
CPSF7	10.246	10.269	10.258	10.244	10.122	10.240
CPSF6	10.144	10.207	10.240	9.803	9.758	9.993
CPSF4	9.535	9.615	9.759	9.497	9.653	9.633
CPSF3	9.519	9.521	9.622	9.394	9.187	9.351
CPSF2	9.020	8.975	8.969	8.458	8.602	8.804
CPSF1	10.331	10.274	10.468	10.286	9.990	10.270

MAD1L1	9.047	8.979	9.186	8.643	8.730	8.661
APOBR	6.887	7.065	6.987	6.964	6.846	7.060
TTLL5	8.728	8.563	8.942	8.525	8.428	8.470
TTLL4	9.287	9.334	9.542	9.336	9.295	9.322
TTLL7	6.386	6.338	6.310	6.544	6.487	6.438
RPL24P8	12.981	13.066	13.218	13.218	13.130	13.174
TTLL1	7.343	7.365	7.544	7.305	7.353	7.289
TTLL3	7.717	7.654	7.793	8.095	7.880	7.808
TTLL2	6.468	6.319	6.216	6.611	6.282	6.287
RPL24P2	12.981	13.066	13.218	13.218	13.130	13.174
AL513548.1	6.022	5.795	5.978	5.854	5.691	6.017
STAT6	11.780	11.839	11.931	11.681	11.652	11.704
AL513548.3	4.475	4.430	4.420	4.346	4.376	4.564
RPL24P4	12.667	12.763	12.929	12.815	12.646	12.860
Z94160.2	6.745	6.316	6.189	6.503	6.539	6.498
Z94160.1	7.350	7.273	7.365	7.284	7.336	7.235
TRARG1	7.465	7.365	7.392	7.456	7.338	7.399
FAM126A	8.036	7.993	7.989	8.187	8.100	8.114
FAM126B	8.546	8.598	8.714	8.298	8.199	8.501
HEPACAM2	3.984	3.859	3.837	4.018	4.026	4.038
CIP2A	6.894	6.960	6.799	6.661	6.737	6.916
AL162431.1	9.004	9.077	9.121	8.847	9.047	9.036
AC145676.1	5.044	4.799	5.011	5.109	5.102	5.180
LINC01159	7.444	7.629	7.589	7.839	7.756	7.748
LINC01153	4.517	4.653	4.996	4.437	4.712	4.793
DBF4	8.222	8.317	8.271	8.007	8.047	8.068
ZNF641	9.217	9.303	9.129	9.291	9.299	9.130
NOA1	10.319	10.345	10.524	10.232	10.261	10.217
MCUB	8.821	8.936	8.987	8.591	8.715	8.600
CCNE2	7.646	7.403	7.568	7.304	7.328	7.234
CCNE1	9.138	8.878	8.961	8.964	8.936	8.930
GCC2	9.612	9.592	9.651	9.338	9.340	9.511
GCC1	8.701	8.727	8.803	8.644	8.559	8.693
TBC1D8B	9.417	9.514	9.488	9.132	9.500	9.298
H2BC12	10.261	10.361	10.461	10.525	10.201	10.328
H2BC13	6.552	6.547	6.808	7.060	6.907	6.917
H2BC10	8.369	8.432	8.579	8.695	8.549	8.419
H2BC17	6.427	6.511	6.399	6.450	6.584	6.556
H2BC14	5.387	5.276	5.463	5.410	5.386	5.462
H2BC15	5.987	6.018	5.747	6.071	5.947	6.101
AL133457.1	4.026	4.086	4.055	4.177	4.010	4.011
CYTL1	5.439	5.462	5.366	5.775	5.799	5.715
C6orf47	8.461	8.481	8.515	8.445	8.516	8.443
LINC00280	5.726	5.653	5.349	5.676	5.859	5.820
SPATA5L1	8.896	8.960	9.005	8.575	8.760	8.570
H4C10P	4.825	5.191	4.793	4.861	4.804	4.987
LRRC2-AS1	6.029	6.067	6.139	6.190	6.123	6.379
BOLA2-SMG1P6	10.698	10.766	10.949	10.772	10.875	10.874
OR7E11P	9.849	9.926	10.029	9.810	9.880	9.911
KRT18P27	6.808	6.874	6.977	6.884	6.796	6.956
MIR205	6.709	6.739	6.474	6.975	6.792	6.896
IGHM	8.337	8.575	8.719	8.611	8.672	8.698
AC007686.3	5.818	5.759	5.878	5.952	5.765	5.779
AC007686.2	3.981	4.112	4.020	3.930	4.043	4.069
DAB2IP	8.889	8.984	9.073	8.949	8.970	8.936
SPATA12	6.303	6.412	6.136	6.266	6.500	6.371

AC100786.1	11.460	11.608	11.958	12.192	12.731	11.871
TRDN	4.648	4.616	4.613	4.792	5.117	4.646
TRDC	6.222	6.348	6.479	6.666	6.519	6.698
FAM229A	7.737	7.724	7.716	7.836	7.813	7.960
FAM229B	8.000	7.868	8.152	8.163	8.026	8.212
TTPA	5.662	5.645	5.749	5.808	6.114	5.766
THORLNC	7.745	7.738	7.595	7.662	7.742	7.903
AL008729.1	5.947	6.177	6.055	6.007	6.118	6.188
KLKB1	7.163	6.892	7.064	7.183	6.970	7.191
RPL3P7	13.670	13.651	13.720	13.644	13.478	13.579
PRAG1	10.522	10.283	10.409	10.199	9.915	10.272
ZGRF1	6.349	6.341	6.346	6.277	6.393	6.302
MCC	8.511	8.600	8.310	8.262	8.396	8.398
FERMT1	10.314	10.451	10.458	10.192	10.303	10.286
ZPLD2P	5.204	5.200	5.369	5.318	5.175	5.357
MCU	9.946	9.997	10.013	9.866	9.995	9.895
AC008746.4	6.440	6.377	6.253	6.213	6.444	6.404
NISCH	9.379	9.411	9.359	9.170	9.375	9.288
UNC5B-AS1	6.627	6.283	6.675	7.002	6.805	6.774
RLN1	5.818	5.926	5.851	5.691	5.887	5.858
RLN2	6.676	6.487	6.619	6.333	6.503	6.637
RLN3	6.669	6.880	6.870	6.867	6.922	6.865
MIR1291	8.618	8.593	8.713	8.707	8.773	8.880
AC008033.3	6.570	6.767	6.503	6.540	6.170	6.573
ZNF354A	7.745	7.661	7.803	7.818	7.672	7.846
C1RL	8.902	8.956	8.798	8.515	8.622	8.670
ZNF354B	7.242	7.087	7.114	7.151	7.114	7.214
VIL1	8.592	8.569	8.628	8.606	8.756	8.633
AC099560.2	13.947	14.022	14.018	14.057	14.036	14.057
MDM1	7.700	7.694	7.768	7.302	7.205	7.544
MDM2	9.060	9.170	9.250	9.114	9.222	9.213
MDM4	8.717	8.363	8.547	8.401	8.063	8.515
AC144652.1	7.694	7.740	7.818	7.527	7.517	7.407
SPATA19	6.847	7.164	7.158	7.041	7.378	7.206
CPB2	5.610	5.817	5.745	5.813	5.813	6.053
CPB1	6.321	6.597	6.487	6.422	6.574	6.620
SYNGR1	8.305	8.324	8.494	8.219	8.217	8.301
WDR92	8.963	8.699	8.848	8.489	8.521	8.616
WDR91	7.788	7.723	7.701	7.748	7.656	7.660
CRP	6.207	6.496	6.631	6.530	6.715	6.552
WDR90	8.682	8.773	8.893	8.531	8.331	8.464
CRX	6.894	7.001	7.047	7.180	7.026	6.947
SYNGR4	6.810	6.949	6.880	6.656	6.823	6.831
CRK	9.894	9.941	10.025	9.968	9.950	9.952
CRH	7.411	7.444	7.368	7.553	7.549	7.520
AC100827.4	8.532	8.295	7.976	8.350	8.524	8.517
SEPTIN14P19	7.159	6.862	6.635	6.551	6.694	6.987
AC009131.1	6.665	6.536	6.510	6.610	6.890	6.696
RTN4IP1	7.912	8.028	8.004	7.630	7.761	7.859
LINC02344	6.893	6.807	6.892	6.849	6.906	7.077
LINC02345	6.316	6.431	6.353	6.228	6.370	6.513
LINC02347	5.053	5.082	4.724	5.180	5.297	4.913
LINC02349	5.575	5.667	5.630	5.946	5.679	5.775
ROMO1	10.047	10.083	10.359	10.110	10.157	10.234
AC092620.2	4.893	4.747	4.477	4.981	5.012	4.665
AC092620.1	8.930	8.975	9.092	8.812	8.849	8.898

AL353150.1	8.900	9.013	8.741	8.892	8.333	8.802
CR2	6.986	7.258	7.198	6.950	7.270	7.121
ASPSCR1	8.624	8.650	8.756	8.426	8.485	8.366
CR1	7.194	7.251	7.219	7.488	7.161	7.291
CR753842.1	11.996	12.053	12.198	11.947	11.842	11.914
AP000295.1	9.519	9.363	9.317	9.089	9.474	9.604
GPD1L	9.108	9.104	9.186	8.931	8.975	8.992
ZNF587	9.747	9.631	9.681	9.600	9.397	9.569
CFAP410	8.263	8.148	8.401	8.563	8.468	8.367
ARNTL2	8.979	9.008	8.961	8.978	8.968	8.902
AC011043.1	7.923	7.782	7.902	7.606	7.461	7.712
RPPH1	6.716	6.951	6.844	6.915	6.843	6.914
CD63	13.456	13.521	13.562	13.587	13.506	13.544
CD69	4.454	4.372	4.498	4.912	4.930	4.764
CELA1	7.541	7.633	7.589	7.629	7.606	7.575
SHCBP1	9.402	9.324	9.326	8.702	8.926	8.836
OR7A10	4.688	4.840	4.731	4.733	4.765	4.661
OR7A17	6.471	6.629	6.394	6.233	6.412	6.379
GABPA	7.553	7.596	7.484	7.358	7.330	7.276
AOX1	8.032	7.983	7.884	8.240	7.986	7.819
AC008124.1	7.485	7.269	7.473	7.834	7.709	7.651
AF228730.6	6.451	6.418	6.257	6.244	6.283	6.307
TIMM9	9.341	9.571	9.565	9.489	9.507	9.455
RPL22P22	8.608	8.758	8.862	8.897	8.860	8.872
MAPKAPK5-AS1	9.339	9.373	9.555	9.230	9.387	9.487
PAEP	6.602	6.557	6.476	6.361	6.608	6.806
NOC4L	9.133	9.197	9.336	8.861	8.902	9.015
AC105133.1	4.571	4.556	4.638	4.648	4.726	4.764
EIF3LP3	7.154	7.282	7.371	7.130	7.138	7.147
PKN1	9.066	9.058	9.321	9.206	9.255	8.893
PKN3	7.698	7.527	7.630	7.356	7.309	7.348
PKN2	10.360	9.891	10.140	9.896	9.894	10.120
CCDC13-AS1	6.162	6.280	6.427	6.157	6.231	6.236
QARS1	11.483	11.492	11.706	11.298	11.158	11.297
MIR568	7.798	7.371	7.160	6.468	7.219	7.657
KCNE2	7.016	6.972	6.891	6.994	7.171	7.051
KCNE3	8.430	8.610	8.438	8.041	8.339	8.305
KCNE1	5.578	5.850	5.893	5.684	5.596	5.738
KCNE4	7.308	7.462	7.364	6.975	7.442	7.424
KCNE5	6.275	6.490	6.473	6.471	6.726	6.532
HEXA-AS1	4.714	4.787	4.710	4.644	4.742	4.679
AC010997.5	6.494	6.507	6.607	6.851	6.759	6.576
AC010997.6	7.486	7.682	7.433	7.467	7.747	7.619
AC010997.3	6.543	6.686	6.539	6.475	6.676	6.752
AC116158.1	4.247	4.261	4.056	4.287	4.331	4.274
TRG-AS1	6.172	6.390	6.367	6.277	6.257	6.386
AL158211.5	3.861	3.933	3.616	4.051	3.879	3.834
RFTN2	6.108	6.113	6.174	6.363	6.168	6.208
AL391069.2	6.585	6.612	6.528	6.761	6.828	6.544
CSNK1D	10.098	10.066	10.251	9.939	9.797	9.798
CSNK1E	8.782	8.831	8.894	8.639	8.595	8.856
LPA	6.786	6.694	6.442	6.599	6.891	6.717
GAPDHP73	10.067	10.269	10.226	10.084	10.269	10.309
ISCU	11.053	11.085	11.126	10.724	10.837	10.889
IL36RN	7.060	6.933	6.692	6.864	6.939	6.796
EHBP1L1	9.007	8.942	9.115	8.616	8.805	8.736

UMODL1	4.317	4.081	4.276	4.220	4.275	4.358
ADAMTS20	4.821	4.662	4.637	4.765	4.847	4.784
AC073073.2	5.032	5.119	4.927	4.964	4.952	4.869
AC006538.2	6.063	6.081	6.144	6.183	6.245	6.219
AC006538.1	6.383	6.256	6.273	6.591	6.321	6.391
AL355103.1	5.742	5.734	5.814	5.804	5.714	5.825
TOMM20P4	11.357	11.450	11.685	11.266	11.282	11.197
GLIS2	9.398	9.220	9.388	9.071	9.157	9.261
GLIS3	9.878	9.685	9.819	9.849	9.996	9.410
GLIS1	6.847	7.027	6.896	6.937	7.112	6.928
BCHE	5.942	5.706	6.157	5.760	5.730	5.699
DCP1B	8.025	8.082	8.150	7.681	7.865	7.685
DCP1A	9.161	9.309	9.294	8.960	9.134	8.992
FAM21EP	6.794	6.863	6.698	6.739	6.816	7.109
AL033538.2	7.057	7.256	7.141	7.365	7.224	7.164
ZNF561-AS1	7.126	7.204	7.002	7.078	7.143	7.247
G6PD	11.510	11.578	11.669	11.597	11.619	11.593
G6PC	7.175	7.004	6.954	6.979	6.986	7.228
AL445223.1	5.247	5.256	4.968	5.212	5.247	5.368
AL353807.1	8.092	8.070	8.255	8.215	8.242	8.091
AL353807.3	8.180	8.132	8.168	8.163	7.979	8.242
SIGLEC17P	6.589	6.869	6.795	6.692	6.754	6.880
SMIM3	7.787	7.989	8.037	7.476	7.849	7.748
NKX2-1-AS1	5.936	6.117	5.910	6.121	6.195	6.237
SMIM1	7.988	7.769	7.814	7.807	7.988	7.785
BX571818.1	9.450	9.234	9.326	9.294	9.596	9.120
SMIM7	9.517	9.675	9.651	9.316	9.385	9.486
SMIM6	9.008	9.100	9.194	9.037	9.111	8.957
SMIM5	7.920	7.921	7.909	7.931	7.959	7.980
SMIM4	8.048	8.044	8.011	8.006	7.796	8.102
SMIM8	7.861	7.874	8.192	7.765	7.937	7.666
TVP23C-CDRT4	7.664	7.761	7.932	7.617	7.538	7.768
AF186192.6	7.664	7.699	7.330	7.729	7.663	7.722
AF186192.1	7.664	7.699	7.330	7.729	7.663	7.722
AP000866.6	6.645	6.484	6.489	6.666	6.712	6.850
AP000866.2	5.819	6.000	6.010	5.887	5.929	6.010
AP000866.1	6.111	6.588	6.471	6.379	6.404	6.473
CRELD1	7.677	7.742	7.648	7.655	7.741	7.672
ERP44	9.730	9.765	9.826	9.894	9.892	9.779
SERPINF1	7.420	7.435	7.223	7.607	7.706	7.581
SERPINF2	7.211	7.481	7.104	7.590	7.304	7.372
OCM2	6.868	6.848	6.885	6.618	6.658	6.865
VPS26A	10.556	10.687	10.763	10.668	10.568	10.560
VPS26C	9.120	9.210	9.168	8.940	8.851	9.065
VPS26B	9.347	9.483	9.465	9.074	9.125	9.247
RNA5SP283	8.342	8.297	8.366	8.512	8.474	8.509
RNA5SP284	8.342	8.297	8.366	8.512	8.474	8.509
SREBF2	10.743	10.658	10.804	10.472	10.309	10.492
SREBF1	10.301	10.384	10.361	9.952	9.843	9.919
OXCT1	9.125	9.279	9.197	9.033	9.080	9.063
OXCT2	7.449	7.489	7.485	7.502	7.464	7.555
CIC	9.512	9.577	9.724	9.659	9.550	9.584
RPL7P47	13.823	13.851	13.913	13.938	13.843	13.938
ZNF324	9.473	9.501	9.679	9.539	9.530	9.446
CIT	9.796	9.760	9.808	9.026	9.277	9.263
TSSK2	5.664	5.745	5.549	5.894	5.881	5.974

UNC45B	5.467	5.211	5.399	5.427	5.438	5.494
ZNF320	5.742	5.923	5.980	5.981	6.110	6.164
EDRF1-DT	6.713	6.953	6.740	6.766	6.840	6.898
BPY2	6.451	6.630	6.565	6.511	6.789	6.752
FOXO3B	11.153	11.057	11.157	10.900	10.949	10.972
DMAC2L	8.938	8.892	9.108	8.988	8.792	8.915
MTA2	7.491	7.041	7.578	7.394	7.213	7.222
MTA3	9.029	9.035	9.185	8.940	8.987	8.917
CUBN	6.738	6.757	6.667	6.871	7.054	7.177
HEPH	4.802	4.849	4.828	5.162	5.073	4.874
MTAP	10.910	10.878	10.961	10.656	10.621	10.744
C2orf49	8.281	8.334	8.308	8.166	8.406	8.111
C2orf42	8.155	8.062	8.108	7.931	8.230	8.082
KCNK15-AS1	6.314	6.237	6.384	6.644	6.465	6.404
GPR180	6.319	6.506	6.446	6.558	6.360	6.463
SLC28A3	7.939	7.851	7.847	7.907	7.997	7.628
GPR182	7.774	7.779	7.669	8.010	8.040	7.870
SLC28A1	7.325	7.299	6.776	7.374	7.426	7.394
SNHG32	9.802	9.805	10.079	9.748	9.638	9.764
SNHG30	7.671	7.629	7.692	7.578	7.734	7.573
MEIS3P2	8.932	9.147	9.183	9.032	8.908	9.023
MEIS3P1	8.932	9.147	9.183	9.032	8.908	9.023
DIMT1	9.489	9.502	9.581	9.429	9.539	9.465
AC008394.1	7.060	7.133	7.103	7.210	7.224	7.454
POLR3GL	8.798	8.823	8.944	8.501	8.605	8.706
AC006460.2	6.914	6.756	7.056	7.037	7.065	7.199
PARP16	9.023	9.008	9.240	8.952	9.128	9.116
PARP14	9.911	9.984	9.863	9.742	9.790	9.646
PARP15	5.594	5.979	5.722	5.811	5.919	6.071
PARP12	9.534	9.605	9.695	9.473	9.698	9.490
PARP10	9.771	9.933	9.960	9.769	9.702	9.826
PARP11	7.121	7.272	7.508	6.816	6.891	6.895
MYRFL	6.183	6.260	5.977	6.964	6.532	6.663
UBAC2	8.679	8.691	8.842	8.594	8.549	8.616
EVL	8.511	8.653	8.780	8.251	8.131	8.350
EVC	7.742	7.706	7.917	7.939	7.982	7.706
ZNF451	9.845	9.868	9.907	9.603	9.895	9.612
POT1-AS1	5.037	4.927	4.756	4.964	5.027	5.061
BACH2	6.701	6.602	6.405	6.689	6.873	6.851
BACH1	8.483	8.518	8.489	8.168	8.170	8.198
AKAIN1	5.025	4.851	5.093	5.365	5.000	5.147
PAM16	8.612	8.619	8.714	8.653	8.608	8.552
WSB1	10.680	10.761	10.840	10.838	11.115	10.518
RASEF	10.307	10.392	10.346	10.088	10.152	10.203
KCNIP4	8.135	8.086	8.139	8.482	8.420	8.268
KCNIP3	8.416	8.476	8.459	8.704	8.590	8.501
KCNIP2	7.864	7.662	7.576	8.050	7.880	7.881
KCNIP1	6.655	6.735	6.708	6.736	6.950	6.807
MOGAT2	6.964	7.045	6.906	7.294	7.145	7.159
MOGAT3	5.808	6.090	5.834	6.089	6.033	6.066
MOGAT1	9.135	9.143	9.002	8.794	8.665	8.912
RPS6KA1	8.560	8.590	8.642	8.475	8.459	8.374
RPS6KA2	8.845	8.830	8.910	8.640	8.828	8.780
RPS6KA3	10.331	10.371	10.312	10.169	10.146	10.212
RPS6KA4	10.341	10.366	10.446	10.401	10.424	10.381
RPS6KA5	9.155	9.143	8.972	8.335	8.937	8.687

RPS6KA6	6.799	6.800	7.097	6.888	6.850	6.829
AL139243.1	7.075	7.341	7.126	7.351	7.378	7.366
FTCD	8.302	8.346	8.348	8.304	8.276	8.550
MINPP1	9.130	9.296	9.319	9.236	9.158	9.248
METTL2B	9.415	9.630	9.642	9.576	9.762	9.595
GANAB	12.144	12.199	12.294	11.881	11.893	12.012
ATXN1-AS1	5.724	5.521	5.889	5.630	5.894	5.794
LINC02632	5.134	5.033	5.261	4.883	5.168	5.016
LINC02637	7.465	7.176	7.336	7.611	7.408	7.238
BUD23	10.192	10.263	10.399	10.400	10.215	10.130
IGHV4-59	6.990	7.135	7.238	7.185	7.143	7.324
MT2A	10.710	10.777	10.843	10.717	10.944	10.802
RGPD2	9.440	9.445	9.356	9.508	9.623	9.229
RGPD3	9.440	9.445	9.356	9.508	9.623	9.229
RGPD1	9.440	9.445	9.356	9.508	9.623	9.229
RGPD6	9.440	9.445	9.356	9.508	9.623	9.306
RGPD4	9.440	9.445	9.356	9.508	9.623	9.229
RGPD5	9.440	9.445	9.356	9.508	9.623	9.306
RGPD8	9.440	9.445	9.356	9.508	9.623	9.306
LINC00672	7.109	7.099	6.932	7.059	7.140	7.143
LINC00671	7.564	7.677	7.429	7.714	7.694	7.724
SLCO1B1	5.533	5.760	5.804	5.580	5.589	5.603
FTLP3	14.413	14.458	14.478	14.437	14.424	14.436
METTL23	10.933	10.999	11.167	10.950	10.937	10.987
METTL22	9.156	9.200	9.368	8.926	8.960	9.078
SPP1	13.721	13.719	13.737	13.672	13.689	13.662
METTL26	11.007	11.028	11.262	10.981	10.967	10.920
METTL25	7.969	8.175	7.995	7.994	7.877	8.037
SPP2	6.610	6.802	6.532	6.596	6.757	6.661
MYL6B	13.303	13.419	13.516	13.481	13.419	13.476
SPNS1	9.304	9.382	9.542	9.098	9.022	9.087
RAB2A	10.466	10.627	10.561	10.437	10.358	10.406
AL009178.2	5.310	5.090	5.204	5.236	5.094	5.198
SPNS2	12.392	12.472	12.519	12.279	12.180	12.206
FRRS1	3.840	3.963	3.832	3.761	3.858	3.814
AC244100.2	7.833	7.880	7.750	7.857	7.852	7.861
AC244100.4	7.816	7.672	7.838	7.804	7.930	7.821
AC090152.1	5.770	5.757	5.382	5.632	5.667	5.778
ACTL7B	6.877	6.980	7.025	7.370	7.183	7.122
ACTL7A	7.375	7.409	7.610	7.390	7.648	7.700
ETNK2	7.713	7.827	7.814	7.940	7.982	7.956
ETNK1	10.724	10.826	10.826	10.292	10.231	10.533
ERMARD	7.661	7.591	7.681	7.509	7.653	7.521
LINC00092	8.266	8.253	8.050	8.194	8.324	8.282
CA14	8.121	8.235	8.165	8.107	8.298	8.128
CA13	7.750	7.862	7.964	7.653	7.405	7.466
CA12	11.386	11.639	11.792	11.779	11.562	11.430
CA11	8.466	8.385	8.689	8.616	8.602	8.637
CA10	6.100	6.354	6.206	6.140	6.305	6.447
RPL7AP6	13.419	13.463	13.555	13.460	13.324	13.444
CHCHD2P6	13.337	13.441	13.505	13.444	13.451	13.475
CHCHD2P2	13.337	13.441	13.505	13.444	13.451	13.475
RAB20	10.168	10.160	10.299	10.059	9.989	10.126
RAB21	11.089	11.135	11.227	10.913	10.854	11.010
RAB23	8.183	8.261	8.426	8.038	8.115	8.268
RAB24	8.699	8.687	8.908	8.461	8.436	8.665

RAB25	11.245	11.370	11.585	11.236	11.326	11.189
RAB26	8.050	8.219	8.167	8.107	8.083	8.255
AP001541.1	4.627	4.790	4.679	4.826	4.730	4.700
GLRA3	5.555	5.599	5.534	5.749	5.660	5.639
GLRA2	8.321	8.328	8.207	8.387	8.353	8.402
GLRA1	5.031	5.085	5.168	5.119	5.306	5.263
DZIP1L	7.357	7.367	7.521	7.704	7.625	7.427
STARD13-AS	6.314	6.334	6.080	6.533	6.401	6.261
PPP2R3B	7.173	7.400	7.315	7.078	7.294	7.145
NTAN1	8.346	8.487	8.831	7.992	8.070	8.330
PPP2R3A	10.274	10.215	10.239	10.101	10.160	10.267
NQO2	8.492	8.603	8.724	8.482	8.549	8.478
NQO1	12.885	12.924	13.032	12.938	12.845	12.894
SFMBT1	8.468	8.390	8.520	8.237	8.229	8.371
SFMBT2	7.047	7.308	7.287	7.379	7.392	7.494
ZPBP2	5.672	5.919	5.373	5.626	5.731	5.770
LEFTY2	7.293	7.498	7.316	7.435	7.453	7.607
LEFTY1	7.438	7.393	7.358	7.385	7.635	7.457
ARRDC4	7.297	7.494	7.485	7.048	7.095	7.205
ARRDC1	9.539	9.627	9.772	9.495	9.498	9.422
ARRDC2	8.351	8.295	8.673	8.570	8.626	8.535
ARRDC3	6.944	6.929	6.984	7.339	7.502	7.663
FAM120AOS	10.363	10.473	10.608	10.336	10.403	10.326
ZIC2	6.382	6.438	6.309	6.206	6.249	6.231
KIAA0930	10.045	10.105	10.200	9.991	10.016	10.094
RABGAP1L	10.514	10.501	10.663	10.515	10.383	10.567
AC092329.4	6.344	6.591	6.340	6.406	6.501	6.517
ELK3	9.843	9.974	9.925	9.595	9.561	9.780
ELK1	10.749	10.728	10.898	10.875	10.837	10.869
GPHB5	7.252	7.361	7.314	7.272	7.294	7.341
ELK4	10.173	10.331	10.344	9.777	9.979	9.956
STRADA	9.261	9.336	9.282	9.117	9.264	9.231
STRADB	8.774	8.788	8.855	8.785	8.784	8.777
AL157882.1	6.882	6.939	6.806	7.018	7.123	7.294
CEP97	6.142	6.201	6.336	6.113	5.949	6.171
CEP95	6.972	6.946	7.173	6.766	6.956	6.887
AL162412.1	6.835	6.674	6.572	6.551	6.651	6.728
AC009283.1	9.939	9.848	9.730	9.716	9.593	9.862
TTTY4C	5.475	5.250	5.301	5.500	5.602	5.402
TTTY4B	5.475	5.250	5.301	5.500	5.602	5.402
LCN15	8.035	8.104	8.156	8.007	8.254	8.072
LCN10	7.711	7.708	7.771	7.725	7.823	7.681
LCN12	5.144	5.376	5.256	5.346	5.554	5.365
TNS3	10.930	10.894	10.953	10.770	10.583	10.767
TNS2	7.707	7.761	7.732	7.748	7.890	7.804
TNS1	7.568	7.750	7.855	7.917	7.857	7.785
AC132872.4	6.014	5.896	5.846	5.623	5.903	5.791
TNS4	8.185	8.197	8.025	8.111	8.268	8.271
NAXE	11.117	11.108	11.245	11.028	11.023	11.079
NAXD	9.103	9.043	9.123	8.747	8.780	8.985
ALDH7A1P1	9.480	9.518	9.821	9.225	9.124	9.137
PIGR	10.855	10.761	10.847	10.700	10.747	10.557
COX18	7.927	7.836	7.940	7.719	7.920	8.039
COX19	12.194	12.308	12.487	12.130	12.165	12.133
SHISA6	4.521	4.519	4.445	4.655	4.487	4.614
SHISA7	7.038	6.926	6.853	7.163	7.156	7.007

AL928654.3	6.407	6.535	6.627	6.491	6.398	6.485
TMEM216	9.452	9.354	9.482	9.471	9.373	9.338
SHISA2	11.169	11.191	11.249	11.054	11.184	11.123
SHISA3	3.804	3.646	3.824	3.800	3.732	3.702
COX10	9.482	9.601	9.612	9.440	9.602	9.419
COX11	9.661	9.834	9.840	9.411	9.516	9.472
TMEM219	10.595	10.726	10.937	10.617	10.614	10.451
TMEM218	9.004	9.064	9.257	8.994	9.053	9.078
ARPP21	6.379	6.202	6.271	6.049	6.199	6.349
COX15	9.680	9.629	9.640	9.389	9.477	9.498
COX16	10.254	10.342	10.450	10.298	10.293	10.245
COX17	9.418	9.379	9.661	9.406	9.471	9.510
TTC36	6.704	6.942	7.162	7.171	7.099	7.097
POTEKP	8.746	8.735	8.681	8.346	8.368	8.583
BET1	9.372	9.306	9.348	9.390	9.372	9.411
ALCAM	11.924	11.917	12.029	11.747	11.685	11.763
TPM4	10.330	10.302	10.497	10.493	10.817	10.411
TPM3	13.164	13.220	13.254	13.064	13.001	13.092
TPM2	10.329	10.394	10.652	10.357	10.256	10.282
TPM1	13.717	13.756	13.782	13.669	13.661	13.725
FBXW8	5.205	5.286	5.201	5.243	5.389	5.390
FBXW9	7.833	7.835	7.867	7.783	7.629	7.929
FOXB1	6.815	6.818	6.899	6.939	7.183	6.804
FBXW2	8.977	8.992	8.964	8.661	8.560	8.880
FBXW4	8.825	8.831	8.981	8.840	8.774	8.855
FBXW5	9.497	9.491	9.727	9.308	9.350	9.292
FBXW7	8.022	8.195	8.099	8.174	8.175	8.152
CDCP1	10.349	10.370	10.426	9.977	10.071	10.208
TGFBR2	11.101	11.189	11.291	11.023	10.831	11.005
TGFBR1	9.695	9.765	9.672	9.330	9.227	9.589
ETFB	11.224	11.340	11.553	11.034	10.801	10.949
SH3RF2	7.759	7.650	8.007	7.564	7.700	7.660
SH3RF3	5.960	5.923	6.084	5.934	6.056	6.081
SH3RF1	10.847	10.886	10.963	10.528	10.752	10.815
TPMT	9.202	9.190	9.418	9.291	9.647	9.326
YKT6	10.566	10.624	10.754	10.439	10.346	10.438
TTLL11-IT1	6.362	6.309	6.228	6.241	6.403	6.364
AC007036.3	7.360	7.286	7.402	7.328	7.482	7.384
TJP2	11.164	11.127	11.175	10.908	10.875	10.870
TJP3	8.868	8.841	9.016	8.705	8.677	8.660
TJP1	9.639	9.642	9.621	9.371	9.325	9.486
AC116609.4	6.357	6.335	6.151	6.515	6.539	6.513
AC023490.6	9.620	9.633	9.591	9.534	9.791	9.460
AC023490.7	7.049	7.195	7.322	7.016	7.141	7.207
DAPK1-IT1	7.206	7.147	7.145	6.964	7.169	7.291
LERFS	7.172	7.284	7.427	7.400	7.319	7.586
TUB	8.826	8.952	8.790	8.896	8.964	9.118
NKILA	8.548	8.631	8.746	8.368	8.297	8.337
ASCL2	6.703	6.797	6.756	6.769	6.735	6.750
TOR1A	9.507	9.565	9.580	9.103	9.155	9.215
ASCL3	7.516	7.575	7.643	7.631	7.699	7.767
CCT6A	12.514	12.543	12.642	12.658	12.561	12.549
CCT6B	7.638	7.643	7.708	7.498	7.729	7.687
RPL7A	13.419	13.463	13.555	13.460	13.324	13.444
CARD11-AS1	6.385	6.394	6.365	6.170	6.474	6.226
CANT1	11.236	11.252	11.325	11.087	11.147	11.155

AL049840.5	6.461	6.344	6.400	6.291	6.489	6.398
TAFA2	6.217	6.083	6.186	6.425	6.472	6.401
TAFA1	4.485	4.600	4.931	4.792	4.859	4.804
TAFA5	7.528	7.478	7.613	7.917	7.499	7.632
TAFA4	4.072	4.162	3.903	4.100	4.189	4.266
PERP	12.736	12.842	12.957	12.666	12.660	12.738
INIP	9.496	9.447	9.530	9.335	9.304	9.341
GAS6-AS1	5.534	5.156	5.493	5.359	5.561	5.204
PNKP	9.667	9.677	9.883	9.316	9.325	9.264
TDRKH	7.674	7.492	7.845	7.462	7.377	7.436
PER3	7.412	7.702	7.486	7.663	7.543	7.595
PER2	9.018	8.788	8.881	8.608	8.784	8.630
PER1	9.368	9.265	9.510	9.796	9.703	9.790
PNKD	10.370	10.532	10.542	10.367	10.330	10.353
ZNF513	7.712	7.549	7.775	7.655	7.759	7.644
KAT8	9.203	9.278	9.451	9.259	9.275	9.327
KAT5	9.688	9.768	9.789	9.803	9.827	9.698
KAT7	10.141	10.139	10.229	9.944	9.998	9.938
AC104116.1	6.451	6.407	6.582	6.316	6.421	6.455
PNRC1	9.554	9.627	9.668	9.587	9.585	9.625
PNRC2	10.757	10.867	10.904	10.732	10.681	10.745
GBA	9.177	9.195	9.333	9.033	9.013	9.060
GNAO1	10.694	10.685	10.243	10.766	10.890	10.881
MROH2B	6.809	6.970	6.905	6.908	6.917	7.054
GAPDH	14.624	14.603	14.678	14.611	14.635	14.611
AC099336.2	13.947	14.022	14.018	14.057	14.036	14.057
TSPYL1	10.997	11.026	11.061	10.859	10.882	10.838
C1QTNF3-AMACI	7.708	7.761	7.739	7.656	7.651	7.690
SNORA68	4.939	4.845	5.015	5.403	5.206	5.111
AP001993.1	4.774	4.966	5.054	4.875	4.920	4.724
TMEM79	8.566	8.588	8.621	8.791	8.577	8.570
AC083801.2	4.906	5.062	4.910	4.988	5.059	4.994
AC004080.8	5.675	5.419	5.119	5.395	5.476	5.451
FAR1P1	7.464	7.486	7.649	7.599	7.669	7.618
TMEM70	10.257	10.324	10.438	10.285	10.317	10.358
AATF	11.514	11.557	11.704	11.494	11.296	11.482
AC004080.6	7.051	7.236	7.286	7.219	7.218	7.138
AC004080.7	5.675	5.419	5.119	5.395	5.476	5.451
TMEM74	6.550	6.602	6.571	6.616	6.660	6.744
AC004080.1	6.422	6.394	6.386	6.728	6.740	6.587
AC004080.2	7.528	7.642	7.604	7.802	7.834	7.865
RPUSD4	8.748	8.869	8.993	8.658	8.664	8.465
RPUSD1	9.323	9.336	9.451	9.500	9.447	9.413
RPUSD2	7.912	8.087	8.217	8.134	8.058	8.079
RPUSD3	8.784	8.910	8.983	8.739	8.823	8.780
AL354718.1	8.174	8.070	8.261	8.185	8.174	8.173
ZFAT	8.245	8.390	8.323	8.375	8.220	8.399
ZNF611	8.437	8.574	8.510	8.284	8.272	8.617
ZNF610	5.609	5.632	5.154	5.734	5.634	5.661
ZNF613	5.452	5.314	4.998	5.421	5.553	5.345
ATP5PO	11.448	11.482	11.710	11.497	11.351	11.428
ZNF615	7.955	7.921	7.866	7.611	7.825	8.132
ZNF614	7.231	7.413	7.297	7.322	7.609	7.372
ZNF616	4.482	4.527	4.568	4.624	4.523	4.484
ATP5PD	12.415	12.474	12.668	12.607	12.545	12.470
ZNF618	8.763	8.710	8.697	8.485	8.701	8.590

ATP5PF	11.041	11.145	11.211	11.074	10.950	11.107
SLC3A1	11.220	11.169	11.331	10.867	10.897	10.935
ATP5PB	11.989	12.011	12.169	12.162	12.147	12.173
EDDM3A	6.331	6.459	5.900	6.678	6.614	6.456
MS4A12	5.244	5.319	4.568	5.238	5.027	5.319
EDDM3B	4.773	4.746	4.662	4.738	4.929	4.776
MS4A14	5.596	5.602	5.619	5.770	5.828	5.678
MS4A15	6.160	6.196	6.244	6.005	6.252	6.279
MSRB1	10.632	10.582	10.777	10.481	10.558	10.473
MSRB3	9.342	9.502	9.482	9.405	9.455	9.578
CTDP1	8.210	8.315	8.323	8.292	8.030	8.119
RPL21P75	12.617	12.677	12.802	12.784	12.769	12.841
GMFG	6.933	6.821	6.924	7.199	6.894	6.950
GMFB	10.649	10.749	10.768	10.629	10.585	10.649
SNORD50B	10.756	10.859	11.059	10.752	10.584	10.774
AP001267.2	3.919	3.976	3.980	3.834	3.891	3.876
AP001267.5	11.170	11.231	11.358	11.098	11.090	11.206
AC068587.4	6.009	5.830	5.919	6.035	5.654	5.949
AC068587.6	6.451	6.418	6.257	6.244	6.283	6.307
FSCN3	10.029	10.185	10.388	10.156	10.144	10.068
FSCN2	5.683	5.638	5.519	5.911	5.969	5.566
FSCN1	7.523	7.671	7.742	7.631	7.699	7.619
G2E3	8.477	8.422	8.499	8.259	8.354	8.400
C5orf64-AS1	7.047	6.816	6.938	7.114	7.239	7.290
AC112487.1	3.555	3.643	3.600	3.579	3.629	3.700
AC068446.3	5.880	5.763	5.614	5.709	5.599	5.562
AC119674.2	10.069	10.075	10.155	10.339	10.357	9.999
AC119674.1	4.072	4.152	4.174	4.159	4.109	4.188
AC005297.3	6.617	6.733	6.557	6.606	6.824	6.548
AC107871.1	10.481	10.612	10.681	10.258	10.358	10.191
CAMK1D	7.591	7.472	7.447	7.564	7.666	7.529
CAMK1G	8.254	8.187	8.194	8.151	8.197	8.264
SMCR8	9.985	9.935	10.030	9.557	9.720	9.468
HHAT	7.660	7.542	7.613	7.175	7.047	7.141
SMCR5	5.465	5.688	5.314	5.359	5.563	5.557
LRWD1	8.698	8.708	8.865	8.676	8.499	8.731
AC096734.1	5.903	6.015	5.779	6.412	6.133	6.097
AC096734.2	7.928	8.034	7.867	8.039	8.116	8.077
AC007906.2	9.165	9.258	9.418	9.206	9.251	9.022
ILRUN	9.951	9.929	10.006	9.935	9.827	9.903
PLAG1	7.243	7.144	7.293	6.905	7.123	7.047
AL161756.1	4.439	4.581	4.464	4.585	4.443	4.412
NKRF	9.357	9.317	9.433	9.251	9.424	9.236
RDH14	9.143	9.219	9.276	8.745	8.701	8.811
RDH16	7.938	8.236	8.052	8.231	8.211	8.334
AC118549.1	9.664	9.647	9.692	9.509	9.389	9.553
NRDE2	7.234	7.149	7.229	6.864	6.821	7.098
RDH11	12.388	12.501	12.563	12.458	12.540	12.531
RDH12	8.190	8.293	7.928	7.895	7.967	8.019
RDH13	9.017	8.897	8.936	8.911	8.628	8.820
AC005261.2	9.231	9.271	9.031	8.689	8.539	9.131
AC005261.1	6.665	6.909	6.907	6.799	6.914	6.849
AC005261.6	7.943	8.199	7.938	8.294	8.126	8.264
VN1R6P	5.688	5.713	5.630	5.580	5.656	5.832
AC005261.4	7.016	7.046	7.022	6.666	6.957	6.869
AC275454.1	8.009	7.993	8.037	7.969	8.005	8.026

OR7E38P	9.849	9.926	10.029	9.810	9.880	9.911
AC275454.2	8.009	7.993	8.037	7.969	8.005	8.026
IGHV3-33-2	6.356	6.471	6.499	6.503	6.500	6.426
WDR5B	7.587	7.671	7.673	7.724	7.795	7.752
OR7E111P	8.629	8.457	8.364	8.673	8.605	8.699
NALCN	7.048	7.080	7.109	6.911	6.795	6.986
GAPLINC	5.004	5.184	5.151	5.176	5.199	5.237
AL365181.3	9.068	9.067	9.024	8.890	9.137	8.959
LINC01876	6.591	6.499	6.356	6.308	6.454	6.376
AL365181.1	4.759	4.855	4.839	4.840	4.785	4.663
LINC01874	6.152	6.340	6.456	6.271	6.649	6.643
ZSCAN25	7.914	8.092	7.963	7.966	7.995	8.063
ZSCAN26	7.093	6.880	7.127	7.112	7.179	7.158
ZSCAN20	6.892	6.816	6.596	6.796	6.694	6.851
ZSCAN21	7.426	7.657	7.728	7.436	7.615	7.554
ZSCAN22	8.099	8.120	8.100	7.994	8.228	8.217
ZSCAN23	4.460	4.428	4.628	4.751	4.800	4.696
TDRD9	5.149	5.080	5.045	5.094	5.107	4.958
MIR570	6.443	6.485	6.180	5.929	6.120	6.219
TDRD3	7.208	7.462	7.283	7.200	7.381	7.394
C9orf24	6.202	6.152	6.115	6.299	6.183	6.153
TDRD7	8.759	8.838	8.954	8.846	8.645	8.819
TDRD6	4.578	4.520	4.653	4.880	4.651	4.609
TDRD5	5.570	5.685	5.724	5.788	5.732	5.843
WDR55	9.596	9.660	9.698	9.553	9.525	9.492
WDR54	10.234	10.383	10.516	10.076	10.025	10.129
MBTPS2	10.316	10.338	10.376	10.219	10.081	10.172
MBTPS1	10.961	10.980	11.141	10.892	10.850	10.841
EIF4G3	8.732	8.752	8.791	8.640	8.722	8.438
AP000766.1	4.304	4.128	4.218	4.125	4.365	4.238
EIF4G1	11.651	11.735	11.808	11.631	11.509	11.616
WDR59	9.240	9.193	9.236	9.064	8.835	9.150
AC113410.3	7.048	6.926	7.003	7.067	7.034	7.126
AC113410.5	7.238	7.298	7.106	7.031	7.208	7.193
ENSAP2	9.495	9.881	9.931	9.634	9.546	9.321
MFSD9	8.020	8.070	8.008	7.743	7.683	7.877
CD151	11.462	11.571	11.788	11.373	11.318	11.249
HCST	7.135	7.376	7.246	7.188	7.153	7.327
FAM200A	5.542	5.868	5.902	5.708	5.824	5.710
FAM200B	8.701	8.702	8.732	8.605	8.474	8.615
CYP2F2P	6.077	6.168	6.317	6.357	6.305	6.259
ABHD14A	7.949	8.158	8.117	7.829	7.955	7.943
AL050343.2	6.967	7.067	7.040	6.987	7.081	7.120
MIR6073	8.775	8.621	8.767	8.621	8.736	8.765
ABHD14B	10.342	10.389	10.645	10.316	10.149	10.280
FO393415.1	4.475	4.607	4.414	4.295	4.533	4.486
TLK1	9.859	9.909	9.929	9.606	9.604	9.745
TLK2	8.869	8.897	9.012	8.849	8.889	8.833
USP54	8.769	8.919	8.908	8.666	8.648	8.661
USP53	7.472	7.513	7.628	7.387	7.429	7.508
AC020891.2	6.666	7.036	7.016	6.886	7.008	7.150
AC020891.3	5.664	5.517	5.422	5.576	5.565	5.540
PRODH	7.830	7.887	7.936	7.808	7.956	7.809
ASCC3	9.158	8.995	9.027	8.832	8.668	8.921
ASCC2	8.819	8.828	8.941	8.700	8.599	8.583
ASCC1	10.369	10.505	10.541	10.416	10.402	10.498

KANTR	7.020	6.824	6.914	6.735	6.987	6.891
HPGD	9.940	9.953	9.952	9.926	9.895	9.873
AC012186.2	12.469	12.523	12.582	12.558	12.486	12.418
AC012186.3	5.261	5.208	5.208	5.303	5.287	5.203
CACNB3	8.758	8.788	8.606	8.623	8.601	8.700
CACNB2	5.632	5.744	5.719	5.661	5.684	5.928
CACNB1	8.256	8.089	7.926	7.939	7.956	8.141
AL591368.1	5.859	5.640	5.684	6.018	5.678	5.874
CACNB4	6.902	6.894	6.687	7.164	7.027	7.058
IL12A-AS1	5.893	5.597	5.585	5.639	5.787	5.913
ETF1P1	10.691	10.740	10.790	10.851	10.642	10.602
ZNF518B	6.945	6.930	7.123	7.083	7.146	7.162
ZNF518A	6.656	6.857	6.774	6.262	6.404	6.570
TGFBRAP1	9.580	9.680	9.614	9.675	9.612	9.665
ANKZF1	8.811	8.834	8.975	8.957	8.776	8.860
B3GALNT2	9.052	9.038	9.073	8.917	8.788	8.822
AP000356.3	9.282	9.414	9.540	9.237	9.106	9.288
AP000356.2	7.351	7.359	7.493	7.514	7.644	7.594
TACC1	11.416	11.360	11.472	11.282	11.191	11.186
TACC2	8.436	8.459	8.521	7.987	8.261	8.301
TACC3	9.417	9.364	9.670	9.113	9.135	9.110
JDP2	8.901	8.925	9.132	8.867	8.991	8.765
KIF20B	8.608	8.567	8.577	8.235	8.268	8.344
KIF20A	9.213	9.162	9.363	8.520	8.915	8.702
MKI67	9.480	9.552	9.649	9.300	9.396	9.280
RPL3L	7.758	7.714	7.608	7.797	7.840	7.710
GNAT1	6.957	6.921	6.842	6.872	6.887	6.939
GNAT3	4.865	5.075	4.907	4.935	4.973	4.989
GNAT2	6.369	6.545	6.276	6.545	6.388	6.482
AC106881.1	4.316	4.456	4.198	4.239	4.306	4.227
ZNF556	7.603	7.499	7.421	7.991	7.725	7.733
ZNF557	7.786	7.643	7.501	7.725	7.731	7.720
ZNF554	8.424	8.405	8.447	8.467	8.625	8.542
ZNF555	7.881	7.537	7.793	7.821	7.983	7.854
ZNF552	8.842	9.025	9.178	8.995	9.498	9.143
ZNF550	8.847	8.703	8.699	8.691	8.651	8.718
ZNF551	7.033	7.082	7.135	6.898	7.187	6.901
ADRM1	11.785	11.893	12.055	11.677	11.549	11.513
ANKRD40	10.146	10.094	10.202	10.141	10.234	10.036
ZNF559	7.093	7.105	7.046	6.636	6.864	6.890
TLCD3A	10.052	10.134	10.308	10.054	9.983	9.906
AL590006.1	4.002	4.067	3.999	3.750	3.976	4.028
LRRC37A7P	4.069	4.154	4.267	4.094	4.228	4.156
WASHC2A	9.457	9.484	9.492	9.379	9.330	9.391
WASHC2C	9.457	9.484	9.492	9.379	9.330	9.391
CACNG1	8.187	8.210	8.197	8.373	8.359	8.226
ZRSR2P1	6.885	6.818	6.961	7.177	6.796	6.893
LEMD1-AS1	6.062	6.342	6.210	6.321	6.310	6.400
LINC01465	4.731	4.969	4.736	4.792	4.815	4.819
LINC01467	5.348	5.292	5.253	5.394	5.700	5.328
PCDH8	3.843	3.709	3.900	3.950	3.865	3.923
PCDH9	7.032	7.135	7.291	6.938	7.277	7.129
CACNG3	7.519	7.164	7.355	7.431	7.440	7.313
RPL26L1	9.842	9.903	10.118	9.835	9.985	9.933
AL008718.2	6.045	6.026	5.819	5.965	6.012	6.177
RPL38	13.575	13.602	13.640	13.645	13.466	13.668

RPL39	14.178	14.223	14.242	14.319	14.368	14.265
RPL36	11.951	12.029	12.336	12.072	12.031	12.135
RPL37	13.187	13.252	13.370	13.289	13.222	13.379
RPL34	13.032	13.010	13.098	13.092	13.086	13.088
RPL35	12.921	12.993	13.123	13.206	13.067	13.155
RPL32	13.951	13.948	14.015	14.017	14.031	14.037
RPL30	13.560	13.599	13.655	13.586	13.482	13.507
RPL31	13.761	13.815	13.887	13.917	13.716	13.887
CACNG6	10.791	10.855	10.804	10.674	10.731	10.707
ATF5	9.044	9.016	9.121	9.158	8.960	8.829
ATF4	12.001	12.006	12.232	11.846	11.858	11.769
ATF7	8.207	8.218	8.187	8.374	8.163	8.283
ATF6	10.144	10.292	10.421	10.145	10.303	10.193
ATF1	7.973	8.028	8.037	7.781	7.818	7.956
IGLV1-51	8.268	8.068	8.195	8.569	8.602	8.402
ATF3	8.541	8.246	8.463	9.773	9.685	9.888
ATF2	9.481	9.446	9.381	8.896	9.137	9.051
SIAH2-AS1	7.973	7.761	7.815	7.945	8.052	7.916
BUB1B	9.688	9.604	9.704	9.062	9.145	9.064
CTTNBP2	5.129	4.998	4.843	4.714	4.869	4.964
LINC01798	6.214	5.991	5.979	6.315	6.154	6.322
LINC01799	4.577	4.496	4.641	4.552	4.497	4.969
LINC01797	6.096	5.729	5.766	6.097	5.943	5.967
MORF4L2-AS1	7.664	7.763	7.514	7.694	7.725	7.747
LINC01792	6.004	6.256	5.858	5.972	6.069	6.055
AL645728.2	6.776	6.887	6.876	6.983	6.890	6.886
KIAA0319	7.144	7.027	7.066	7.039	7.275	7.117
SDF4	11.455	11.495	11.604	11.179	11.204	11.291
SDF2	9.169	9.221	9.353	9.223	9.363	9.297
PTGER4	8.641	8.781	8.902	8.646	8.662	8.748
PTGER1	8.681	8.733	8.738	8.821	9.028	8.917
PTGER2	5.242	5.127	5.339	5.308	5.429	5.441
PTGER3	6.282	6.405	6.183	6.362	6.394	6.489
AF233439.1	4.418	4.422	4.779	4.788	4.380	4.662
B3GALT4	9.028	9.062	9.174	8.867	9.092	8.966
B3GALT5	10.104	10.125	10.011	9.802	9.690	9.989
B3GALT6	10.590	10.667	10.737	10.458	10.508	10.388
CTNNAL1	9.080	9.321	9.482	9.222	9.220	9.242
B3GALT1	6.545	6.596	6.323	6.554	6.521	6.286
B3GALT2	3.956	3.942	3.907	4.210	4.054	3.953
MDFIC	8.252	7.999	8.217	7.902	8.005	8.026
STK3	9.715	9.789	9.835	9.480	9.552	9.628
STK4	10.716	10.665	10.692	10.666	10.633	10.631
SNRNP25	10.633	10.686	10.988	10.317	10.252	10.288
LINC02848	6.240	6.432	6.427	6.488	6.531	6.427
LINC02843	7.107	7.127	7.028	7.407	7.486	7.260
LINC02842	5.000	5.056	4.845	5.058	5.181	5.187
LINC02846	6.820	6.751	6.902	7.011	6.991	7.006
LINC02844	5.572	5.570	5.577	5.406	5.573	5.829
COX7CP1	12.417	12.487	12.646	12.569	12.532	12.593
NKG7	6.763	6.748	6.745	6.844	6.857	6.892
DRP2	7.618	7.657	7.665	7.654	7.729	7.876
ANKS3	8.291	8.206	8.199	8.197	8.190	8.079
TNFRSF8	7.242	7.088	7.213	7.004	7.236	7.302
TNFRSF9	6.561	6.933	6.747	6.908	6.913	7.062
ANKS6	8.854	8.973	9.083	8.915	8.759	8.890

TNFRSF4	7.882	7.734	7.836	7.925	8.021	8.029
GUCY2C	4.774	5.089	4.885	4.951	5.102	5.035
GUCY2D	6.588	6.506	6.457	6.450	6.601	6.655
GUCY2F	6.140	5.899	5.892	6.113	6.158	6.091
SENP8	7.538	7.576	7.823	7.742	7.768	7.718
SENP7	6.795	6.849	7.015	6.787	6.920	6.834
FIBCD1	8.190	7.976	8.278	8.349	8.265	8.217
SENP5	8.888	8.831	8.876	8.959	8.900	8.878
SENP3	9.234	9.234	9.368	9.259	9.194	9.175
SENP2	10.283	10.339	10.310	10.069	9.950	10.109
SENP1	7.798	7.854	7.877	7.730	7.655	7.609
SAMM50	10.917	11.042	11.206	10.929	10.869	10.929
BICRAL	8.383	8.086	8.278	7.872	7.859	7.898
SNPH	7.698	7.639	7.647	7.785	7.601	7.610
AC009690.2	7.082	6.922	6.925	7.059	7.155	7.039
AC009690.1	6.339	6.579	6.391	6.608	6.486	6.659
P3R3URF	6.774	6.666	6.848	6.764	7.021	6.698
CELF2-AS1	7.098	6.969	7.141	7.200	7.452	7.147
NOTUM	8.642	8.643	8.774	8.885	8.808	8.838
INS	6.383	6.508	6.496	6.570	6.546	6.669
GFPT1	11.784	11.873	11.863	11.692	11.732	11.733
GFPT2	7.051	7.231	7.117	7.108	7.664	7.326
ECHDC3	9.895	10.107	10.294	9.824	9.884	9.906
INA	7.915	7.988	8.019	8.039	8.209	8.176
ECHDC1	10.742	10.782	10.909	10.784	10.585	10.733
MIR195	7.403	7.147	7.087	7.225	7.569	7.440
MIR198	7.625	7.784	7.692	7.557	7.778	7.724
SLC2A14	7.293	7.365	7.148	7.214	7.481	7.241
SLC2A13	7.656	7.589	7.736	7.215	7.239	7.604
SLC2A12	8.741	8.720	8.796	8.346	8.407	8.385
SLC2A11	7.732	7.648	7.675	7.399	7.408	7.523
SLC2A10	10.188	10.208	10.408	10.032	9.935	10.135
BZW1P2	11.286	11.436	11.368	11.295	11.293	11.384
BZW1P1	11.286	11.436	11.368	11.295	11.293	11.384
RPL23A	14.341	14.360	14.394	14.370	14.332	14.374
BSN	7.176	6.893	7.091	7.177	7.043	7.025
AL355385.2	8.443	8.568	8.545	8.513	8.825	8.414
LCOR	9.514	9.582	9.473	9.174	9.093	9.672
AL022341.1	6.728	6.985	6.879	7.105	6.857	7.082
BSG	10.979	10.927	11.279	11.154	11.300	11.041
P4HTM	10.619	10.687	10.787	10.399	10.289	10.353
AC005920.1	8.823	8.933	8.970	9.055	9.095	9.074
AC011295.2	6.582	6.672	6.703	6.718	6.700	6.647
GSG1	5.431	5.383	5.434	5.508	5.611	5.413
AC011295.1	13.154	13.225	13.361	13.496	13.391	13.505
PLSCR4	8.390	8.336	8.451	7.758	8.035	8.180
OR4F28P	7.745	7.729	7.361	7.729	7.847	7.903
PLSCR2	6.165	6.446	6.344	6.488	6.596	6.356
PLSCR3	10.512	10.696	10.703	10.606	10.668	10.682
AL138831.3	4.928	4.790	4.855	5.041	4.849	4.966
RNU6-82P	4.405	4.033	4.116	4.204	3.996	4.083
OXTR	5.651	5.502	5.459	5.685	5.700	5.729
HNF4G	8.508	8.539	8.435	8.190	8.344	8.315
NCKAP1L	8.075	8.024	7.926	7.977	8.278	8.186
ODR4	9.718	9.891	9.927	9.709	9.852	9.438
GEN1	5.682	5.537	5.355	5.222	5.426	5.314

LINC00485	3.665	3.746	3.752	3.965	3.764	3.632
GPX1P1	10.870	10.956	11.079	10.914	10.909	11.001
EPS8	11.306	11.479	11.551	11.333	11.253	11.341
ATP5MC3	12.546	12.617	12.726	12.632	12.604	12.613
PARM1	8.665	8.603	8.652	8.680	8.779	8.580
HERPUD2	10.023	9.994	10.137	9.662	9.610	9.714
HERPUD1	10.606	10.706	10.895	10.380	10.397	10.578
ATP5MC2	10.988	11.088	11.325	10.944	10.924	10.964
AC092546.1	5.157	5.202	5.227	5.411	5.316	5.292
SPCS2P4	12.079	12.145	12.232	12.152	12.137	12.103
FCGRT	9.915	9.953	10.236	9.690	9.707	9.794
TRA2A	9.256	9.036	9.105	9.045	9.097	9.025
TRA2B	11.664	11.596	11.683	11.527	11.514	11.527
BRMS1L	8.907	8.873	8.976	8.723	8.636	8.761
MIR133A1	3.830	3.839	3.890	3.846	4.006	3.803
BAAT	8.643	8.794	8.721	8.755	8.784	8.531
TPSD1	7.147	7.392	7.512	7.630	7.499	7.656
SFTPD	6.649	6.557	6.806	6.545	6.524	6.675
AC093151.4	6.148	6.168	5.819	6.387	6.149	6.237
AC093151.2	6.148	6.168	5.819	6.387	6.149	6.237
SFTPB	8.961	9.048	8.881	9.195	9.025	9.118
SFTPC	8.763	8.827	8.765	8.834	8.864	8.880
DNTTIP1	9.893	9.834	10.113	9.922	9.726	9.779
DNTTIP2	10.649	10.667	10.695	10.759	10.719	10.742
PRAMEF22	7.847	8.084	8.166	8.407	8.226	8.207
PRAMEF27	7.847	8.084	8.166	8.407	8.226	8.207
PRAMEF26	7.847	8.084	8.166	8.407	8.226	8.207
PRAMEF25	7.847	8.084	8.166	8.407	8.226	8.207
TSC22D1	11.267	11.364	11.467	11.549	11.546	11.565
TSC22D3	10.362	10.320	10.502	10.210	10.328	10.317
TSC22D2	10.040	10.106	10.165	10.166	10.132	10.268
TSC22D4	8.856	8.982	8.987	8.975	9.086	8.865
AC136475.3	6.956	7.047	6.868	6.991	6.964	6.929
AL133373.3	3.693	3.706	3.764	3.908	3.738	3.898
AL133373.2	3.693	3.706	3.764	3.908	3.738	3.898
CNFN	6.848	6.903	7.011	6.942	6.904	6.973
CD2BP2	10.274	10.362	10.492	10.236	10.124	10.218
KCNJ2-AS1	4.090	3.984	4.093	4.258	4.278	4.088
GRASP	8.411	8.420	8.404	8.649	8.583	8.575
KATNA1	7.705	7.701	7.910	7.442	7.332	7.519
FRG1CP	9.323	9.301	9.371	9.068	9.288	9.194
ADAMTSL2	6.724	6.629	6.716	6.925	6.805	6.713
ADAMTSL3	7.030	7.030	6.813	7.118	7.053	7.064
ADAMTSL1	7.050	7.227	7.309	7.257	7.336	7.173
ADAMTSL4	7.330	7.329	7.188	7.236	7.304	7.219
ADAMTSL5	7.648	7.634	7.605	7.587	7.769	7.587
C6orf141	8.937	8.736	8.595	8.440	8.450	8.550
LUNAR1	5.203	5.138	5.119	4.947	5.389	5.084
KIAA0895L	7.900	7.922	8.008	7.901	7.862	7.999
AC005332.6	7.818	7.776	7.706	7.805	7.814	7.743
AC005332.4	4.774	4.526	4.737	4.816	4.774	4.646
AL590787.1	8.083	7.837	7.525	8.032	7.886	7.907
SARDH	7.365	7.644	7.675	7.887	7.633	7.381
AC011468.1	4.448	4.465	4.433	4.127	4.421	4.483
AC011468.2	5.637	5.602	5.743	5.604	5.544	5.730
EEF1E1	9.723	9.787	9.882	9.915	9.805	9.839

AC093155.3	6.515	6.440	6.493	6.365	6.684	6.552
ANKRD30BP1	4.172	4.376	4.162	4.365	3.959	4.234
ANKRD30BP2	5.821	6.035	5.659	6.080	5.739	5.988
CYP1B1	8.152	7.867	8.097	8.139	8.079	8.396
AC113423.2	5.700	5.577	5.634	5.834	5.652	5.525
AL109918.1	6.513	6.334	6.503	6.567	6.681	6.456
HAS1	7.419	7.672	7.444	7.639	7.508	7.549
HAS2	5.632	5.468	5.422	5.750	5.538	5.581
HAS3	8.260	8.458	8.521	8.001	8.007	8.195
MORC2-AS1	9.051	9.076	9.077	8.902	8.904	8.951
ARSF	7.694	7.728	7.717	7.630	7.645	7.737
ARSG	5.249	5.489	5.612	5.700	5.849	5.535
LY6G6D	7.290	7.415	7.551	7.424	7.504	7.555
KNSTRN	8.622	8.642	8.651	8.418	8.560	8.486
C12orf65	9.057	9.061	9.159	8.851	8.845	8.706
IRS4	6.610	6.772	6.853	6.990	6.827	6.860
AC131097.2	5.305	5.514	5.566	5.676	5.713	5.601
IRS1	9.646	9.671	9.713	9.662	9.633	9.696
IRS2	9.332	9.386	9.412	9.281	9.336	9.237
ARSL	8.788	8.791	8.939	8.658	8.615	8.686
TTC3P1	10.210	10.146	10.170	9.911	9.844	9.996
CA2	9.069	9.076	9.109	8.625	8.619	8.642
RPSAP19	13.432	13.517	13.661	13.465	13.519	13.574
CA7	7.557	7.771	7.674	7.725	7.828	7.772
RPSAP15	13.432	13.517	13.661	13.465	13.519	13.574
RPSAP12	13.432	13.517	13.661	13.465	13.519	13.574
CA6	7.160	7.037	7.303	7.348	7.262	7.113
LY6G6F	7.215	7.371	7.385	7.420	7.504	7.553
HMGA1P4	8.210	8.127	8.338	8.431	8.325	8.249
ARSK	9.243	9.156	9.113	9.073	9.287	9.215
RGL1	8.769	8.790	8.734	8.511	8.690	8.862
RGL3	8.713	8.680	8.667	8.418	8.523	8.732
RGL2	10.162	10.166	10.246	10.075	9.969	10.002
RGL4	5.395	5.325	5.180	5.362	5.530	5.127
NFKBIL1	8.562	8.448	8.677	8.523	8.262	8.493
AMD1	9.962	10.069	10.006	9.979	9.864	10.116
ELOA2	5.811	5.501	5.393	5.854	5.547	5.600
ELOA3	5.658	5.566	5.464	5.809	5.745	5.944
AL356417.2	4.967	5.013	5.014	4.630	4.745	5.186
MATK	7.315	7.316	7.302	7.566	7.467	7.503
PMF1	9.348	9.409	9.540	9.246	9.246	9.128
RAPSN	5.074	4.948	5.007	4.992	5.307	5.128
ZNF791	9.292	9.348	9.337	8.701	8.952	8.964
ZNF792	6.748	6.494	6.482	6.425	6.542	6.358
ZNF793	6.462	6.372	6.531	6.843	6.630	6.448
ZNF799	8.435	8.539	8.568	8.424	8.517	8.527
TSNAXIP1	7.917	8.112	8.066	8.023	8.056	7.983
AL691426.1	5.934	5.576	5.866	5.923	5.608	5.819
AC019080.5	6.975	6.960	6.752	6.816	6.906	6.805
PPP3CB-AS1	7.277	7.232	7.298	7.065	7.253	7.031
AC019080.1	5.013	4.880	5.106	5.349	5.137	5.214
TRGV7	7.091	7.200	6.970	7.018	7.207	7.255
PPP1R7	10.318	10.465	10.605	10.133	9.891	10.091
TRGV5	7.612	7.669	7.726	7.871	7.567	7.916
TRGV3	7.612	7.669	7.726	7.871	7.567	7.916
PPP1R2	10.791	10.879	10.897	10.763	10.748	10.719

CYP4F12	8.599	8.343	8.580	8.694	8.553	8.655
CYP4F11	5.643	5.852	5.918	5.766	5.720	5.610
PPP1R8	9.381	9.508	9.507	9.296	9.283	9.394
BX470111.1	8.885	8.883	9.033	8.878	9.052	8.876
GPAA1P2	7.471	7.500	7.686	7.180	7.427	7.531
GPAA1P1	7.471	7.500	7.686	7.180	7.427	7.531
TRPV5	7.316	7.360	7.620	7.707	7.596	7.436
TRPV4	7.715	7.603	7.764	7.759	8.009	8.012
AC159540.2	7.457	7.393	7.354	7.151	7.137	7.249
TRPV6	7.678	7.899	7.877	7.964	7.890	7.834
TRPV1	7.571	7.535	7.576	7.703	7.805	7.651
TRPV3	8.237	8.181	8.003	8.539	8.271	8.376
TRPV2	7.416	7.326	7.226	7.613	7.467	7.553
POSTN	4.720	4.754	4.610	4.981	4.981	4.879
FGFR4	9.929	9.930	10.094	9.975	9.918	9.834
SAV1	9.779	9.804	9.803	9.617	9.967	9.693
FGFR2	10.283	10.228	10.362	10.041	9.952	9.978
FGFR3	11.595	11.499	11.724	11.156	11.032	11.253
FGFR1	9.908	9.952	9.932	9.817	9.630	9.535
CXCL1	9.961	10.007	10.032	10.264	10.289	10.400
AC084866.1	4.106	4.044	3.828	4.044	3.914	3.904
CXCL3	8.216	8.014	8.295	8.541	8.567	8.763
CXCL2	9.148	8.890	8.919	9.388	9.491	9.565
CXCL5	13.211	13.208	13.201	13.114	13.336	13.322
CXCL6	8.432	8.433	8.241	8.344	8.189	8.427
CXCL9	7.265	7.541	7.131	7.783	7.305	7.707
CXCL8	9.911	10.032	10.042	11.073	11.143	11.061
FGF7P8	5.375	5.478	5.721	5.375	5.628	5.606
FGF7P2	5.375	5.478	5.721	5.375	5.628	5.606
FGF7P3	5.375	5.478	5.721	5.375	5.628	5.606
FGF7P1	5.375	5.478	5.721	5.375	5.628	5.606
FGF7P6	5.375	5.478	5.721	5.375	5.628	5.606
FGF7P7	5.375	5.478	5.721	5.375	5.628	5.606
FGF7P4	5.375	5.478	5.721	5.375	5.628	5.606
FGF7P5	5.375	5.478	5.721	5.375	5.628	5.606
DPH3P1	6.786	6.775	6.778	6.795	6.945	6.980
AP2S1	12.487	12.527	12.703	12.449	12.355	12.335
DCTD	9.997	10.034	10.262	9.539	9.667	9.567
SLCO4C1	7.040	7.193	7.194	6.536	6.898	6.801
AC233968.1	10.910	10.920	10.896	10.979	11.199	10.847
ALDH16A1	9.787	9.796	9.964	9.536	9.440	9.427
ARF1P1	8.708	8.732	8.660	8.793	8.928	8.767
THRSP	6.497	6.546	6.707	6.534	6.719	6.762
GCKR	8.450	8.476	8.510	8.442	8.538	8.397
AC148477.4	6.553	6.204	5.660	6.346	6.399	6.338
NAA16	8.941	8.960	8.788	8.656	8.499	8.769
UBE2H	11.308	11.253	11.482	11.253	11.520	11.000
UBE2K	10.072	10.106	10.288	10.115	10.103	9.925
NAA15	8.500	8.682	8.850	8.773	8.979	8.126
UBE2M	10.982	11.113	11.301	10.903	10.656	10.770
LINC00205	8.316	8.228	8.402	8.406	8.279	8.354
NAA11	6.614	6.606	6.661	6.613	6.710	6.760
UBE2A	10.795	10.878	10.905	10.657	10.707	10.702
AC024614.2	6.339	6.418	6.425	6.352	6.474	6.573
AC024614.1	4.596	4.873	4.809	4.786	4.890	4.828
LINC00208	7.135	6.930	6.947	7.178	7.112	7.106

UBE2F	9.663	9.748	9.757	9.621	9.715	9.706
SPR	10.826	10.970	11.160	10.933	10.764	10.897
NRROS	7.595	7.535	7.533	7.652	7.781	7.796
UBE2S	11.824	11.787	12.057	10.982	11.221	10.899
UBE2U	4.009	3.914	4.009	4.035	4.144	3.980
UBE2T	10.052	9.954	10.253	10.011	9.901	9.816
UBE2W	9.647	9.630	9.683	9.701	9.877	9.764
PTGFR	5.336	5.388	5.755	5.139	5.241	5.195
LIPT1	8.204	8.228	8.297	8.338	8.259	8.379
AC135977.1	8.877	9.046	9.108	9.050	9.163	9.088
RNU1-123P	7.693	7.671	7.661	7.661	7.638	7.631
AC026464.2	7.527	7.668	7.745	7.481	7.571	7.485
AC026464.6	9.800	9.744	9.879	9.579	9.530	9.540
AC026367.2	6.110	5.703	5.665	5.786	5.529	5.694
TALDO1	12.493	12.599	12.697	12.514	12.340	12.513
PDE3A	7.849	7.870	8.081	7.303	7.641	7.686
PDE3B	7.747	7.853	7.727	7.628	7.620	7.725
LFNG	8.914	8.941	9.131	8.950	8.647	8.874
NDUFA6	11.046	11.159	11.292	11.088	10.998	10.991
NDUFA7	9.662	9.593	9.851	9.518	9.446	9.401
NDUFA4	11.698	11.796	11.866	11.942	12.038	11.984
NDUFA5	9.714	9.787	9.871	9.473	9.442	9.576
NDUFA2	10.542	10.593	10.821	10.706	10.497	10.646
NDUFA3	11.521	11.606	11.763	11.497	11.462	11.526
NDUFA1	11.758	11.799	11.892	11.849	11.684	11.852
NDUFA8	9.644	9.768	9.867	9.672	9.593	9.723
NDUFA9	10.764	10.857	10.937	10.738	10.704	10.852
AC004988.1	4.793	4.935	4.503	4.654	4.896	4.995
RPLP0P1	8.180	8.181	8.092	8.212	8.066	8.404
RPLP0P6	14.563	14.551	14.570	14.502	14.525	14.557
AC027288.3	4.560	4.675	4.408	4.743	4.587	4.548
AC106736.1	7.341	7.520	7.356	7.793	7.580	7.467
RPS11P1	8.221	8.204	8.231	8.106	8.389	8.086
ECPAS	9.007	8.955	9.075	8.612	8.639	8.813
BIRC7	7.521	7.639	7.700	7.740	7.511	7.556
EIF1	14.018	14.041	14.061	13.974	13.970	14.021
BIRC5	9.135	8.941	9.273	8.874	9.057	8.997
BIRC3	8.396	8.408	8.272	8.066	8.290	8.506
BIRC2	9.864	9.857	9.898	9.611	9.613	9.591
EIF6	11.297	11.401	11.520	11.361	11.245	11.313
LINC00970	7.878	8.063	8.096	7.971	7.984	8.035
LINC00973	5.996	5.912	6.106	5.995	5.792	5.756
ANKRD16	7.886	7.988	8.038	7.943	8.090	8.088
ANKRD17	10.131	10.131	10.171	10.009	10.219	10.142
ANKRD12	7.982	7.921	7.805	8.136	7.858	7.988
ANKRD10	9.426	9.405	9.436	9.229	9.251	9.383
ANKRD11	10.036	9.985	9.986	9.848	9.900	9.878
AC092535.2	4.504	4.798	4.438	4.570	4.870	4.799
NT5C2	10.082	9.965	10.141	10.067	10.037	10.012
MEA1	10.775	10.812	11.117	10.932	10.890	10.757
MNS1	8.697	8.666	8.939	8.043	8.087	8.047
ELOA-AS1	8.090	8.094	8.116	7.994	7.928	8.064
KRTAP4-11	5.269	5.530	5.489	5.531	5.820	5.436
KRTAP4-12	7.047	7.113	6.631	7.150	7.296	7.174
OR7E19P	5.932	5.940	5.795	5.886	5.797	5.879
KCNK9	7.680	7.702	7.677	8.063	7.868	7.935

CA5BP1	8.635	8.834	8.808	8.757	8.687	8.725
AC044802.2	5.696	5.598	5.550	5.632	5.725	5.736
AC044802.1	4.660	4.565	4.662	4.881	4.639	4.680
CRIM1	10.385	10.427	10.462	9.930	10.467	10.042
TRAT1	4.613	4.436	4.233	4.470	4.541	4.357
ZBED3-AS1	7.116	7.308	7.458	7.083	7.233	7.238
PARD3-AS1	4.040	4.151	4.245	4.405	4.230	4.093
IL21R-AS1	8.198	8.367	8.187	8.233	8.400	8.229
HIBADH	10.822	10.990	11.025	10.665	10.604	10.658
PSMC1P1	11.930	11.960	12.005	11.913	11.789	11.822
ATP6AP1L	7.814	7.930	7.836	7.439	7.893	7.498
ADGB	4.312	4.260	4.228	4.466	4.507	4.422
SUSD4	9.147	9.096	9.178	9.321	9.272	9.278
SUSD5	4.299	4.196	4.316	4.263	4.382	4.248
SUSD2	7.533	7.417	7.522	7.630	7.528	7.684
SUSD3	6.573	6.543	6.830	6.916	6.772	6.930
SUSD1	8.978	8.930	9.047	8.519	8.685	8.721
SLC26A10	6.500	6.671	6.575	6.610	6.690	6.487
SLC26A11	8.824	8.811	8.853	8.829	8.606	8.639
FAM221A	8.666	8.656	8.746	8.602	8.745	8.586
FAM221B	5.431	5.873	5.819	5.785	5.636	5.692
AP001528.2	4.435	4.247	4.452	4.654	4.524	4.481
AP001528.1	6.227	6.189	6.209	6.372	6.479	6.281
CSPG4P3Y	6.957	6.925	6.968	7.021	7.153	7.161
BRINP3	4.005	4.051	4.146	4.039	3.996	4.008
BRINP2	7.305	7.350	7.045	7.397	7.410	7.428
BRINP1	7.451	7.434	7.036	7.685	7.557	7.411
OTUD6B	6.560	6.787	6.782	6.179	6.490	6.231
AL731661.1	5.165	5.294	5.290	5.225	5.374	5.397
F8A1	11.416	11.442	11.553	11.379	11.413	11.348
F8A3	11.416	11.442	11.553	11.379	11.413	11.348
F8A2	11.416	11.442	11.553	11.379	11.413	11.348
FMN1	8.326	8.280	8.177	8.179	8.223	8.276
EPRS1	10.803	10.833	10.830	10.603	10.583	10.582
FMN2	5.619	5.331	5.346	5.505	5.481	5.440
SPRR1A	8.153	8.302	8.276	8.074	8.339	8.250
SPRR1B	6.939	7.150	7.023	7.104	6.940	6.966
AC068831.8	11.499	11.434	11.686	10.927	10.960	10.932
KIAA0556	8.880	8.821	8.969	8.738	8.517	9.022
HAUS8	7.244	7.408	7.933	7.336	7.238	7.308
HAUS6	9.151	9.114	9.081	8.554	8.668	8.636
HAUS7	8.527	8.630	8.739	8.377	8.419	8.510
HAUS4	9.686	9.780	9.996	9.792	9.808	9.696
HAUS5	9.851	9.681	9.584	9.555	9.827	9.681
HAUS2	8.397	8.324	8.289	7.873	8.293	8.305
HAUS3	7.771	7.861	7.773	7.790	7.617	7.764
HAUS1	8.811	8.727	8.992	8.511	8.481	8.651
SLC5A12	4.644	4.628	4.774	4.808	4.576	4.586
SLC5A10	7.642	7.757	7.782	7.612	7.705	7.750
SLC5A11	6.797	6.784	6.865	7.187	7.151	6.968
MYD88	10.637	10.688	10.627	10.546	10.559	10.516
UBQLNL	6.552	6.503	6.978	6.964	6.854	6.913
TPTEP2	7.377	7.464	7.460	7.632	7.646	7.434
TPTEP1	7.508	7.502	7.445	7.516	7.728	7.531
HENMT1	8.894	9.075	9.057	8.769	8.824	8.755
TNFAIP6	8.405	8.450	8.686	7.979	8.244	7.756

TNFAIP1	10.256	10.195	10.345	10.142	10.209	10.316
TNFAIP3	8.787	8.747	8.571	9.607	9.734	9.435
TNFAIP2	9.445	9.439	9.477	9.499	9.506	9.567
SLIT1-AS1	6.987	6.576	6.763	7.285	7.018	6.930
RNPC3	7.967	8.069	8.111	7.846	8.106	7.570
DTX3L	11.190	11.180	11.163	11.012	10.944	11.052
AC011700.1	7.238	7.213	7.148	7.513	7.264	7.223
SDCBP	11.870	11.916	11.978	11.851	11.858	11.829
AL670729.3	7.000	7.221	7.110	7.272	7.175	7.279
LMBR1	8.238	8.319	8.264	8.075	8.193	8.198
COX6A1P2	13.007	13.124	13.178	13.155	13.129	13.142
DEFB106A	9.783	9.817	9.512	9.738	9.862	9.935
DEFB106B	9.783	9.817	9.512	9.738	9.862	9.935
LINC01618	6.582	6.759	6.656	6.816	6.934	6.803
LINC01613	7.166	7.123	7.061	7.233	7.179	7.308
LINC01616	5.729	5.687	5.666	5.608	5.881	5.777
ICAM4	4.688	5.051	4.918	4.934	4.931	4.829
COL26A1	7.798	7.817	7.915	8.432	8.123	8.130
R3HCC1	9.943	10.109	10.289	10.083	10.068	9.922
ARL17B	8.723	8.745	8.676	8.745	8.680	8.749
ARL17A	8.723	8.745	8.676	8.745	8.680	8.749
ZBTB11-AS1	7.257	7.057	7.334	7.425	7.351	7.376
SHPK	7.469	7.261	7.348	7.234	7.194	7.281
ANKRD34A	7.719	7.767	7.655	8.020	7.828	7.833
ANKRD34C	5.252	5.245	5.107	5.295	5.243	5.543
SORCS3-AS1	7.681	7.607	7.648	7.641	7.723	7.821
ABITRAM	8.754	8.585	8.793	8.726	8.712	8.745
NXT2	8.695	8.850	8.868	8.701	8.662	8.880
AC093582.1	6.242	6.510	6.319	6.643	6.419	6.639
NXT1	8.394	8.508	8.654	8.557	8.670	8.640
CSE1L	12.271	12.415	12.493	12.269	12.139	12.112
RFPL3	7.738	7.911	7.746	7.818	7.952	7.968
LINC01619	5.791	5.629	5.822	5.516	5.467	5.554
ETFRF1	9.265	9.411	9.437	9.194	9.340	9.295
AL354798.1	11.284	11.299	11.383	11.158	11.019	11.161
GRIK1-AS1	5.860	5.857	5.836	6.012	6.067	5.881
AL139260.2	10.636	10.675	10.722	10.609	10.569	10.662
SGF29	8.950	8.890	9.122	8.899	8.862	8.770
ARHGEF10L	8.350	8.520	8.437	8.373	8.356	8.514
DNASE2	10.324	10.329	10.462	10.117	10.379	10.189
DNASE1	8.055	8.048	7.902	7.958	8.191	8.163
SYNDIG1L	7.540	7.622	7.667	7.921	7.828	7.648
LINC00563	6.753	6.780	7.074	7.093	7.178	7.094
LINC00560	5.231	5.384	5.347	5.289	5.377	5.377
LINC00561	5.785	5.642	5.720	5.716	5.579	5.579
LINC00566	4.734	4.899	4.502	4.801	4.731	4.700
LINC00567	4.887	4.723	4.776	5.135	4.814	4.966
LINC00564	4.366	4.251	4.230	4.379	4.458	4.440
LINC00565	6.090	6.411	6.168	6.238	6.395	6.284
ZNF324B	7.703	7.766	7.897	7.813	7.828	7.683
GFI1	6.774	6.765	6.715	6.891	6.883	6.919
UNC45A	9.227	9.419	9.507	9.238	9.096	9.175
AL033380.1	7.635	7.443	7.662	7.561	7.570	7.699
ESPN	8.456	8.256	8.123	8.405	8.493	8.441
GMDS-DT	7.252	7.144	7.119	6.991	7.202	7.056
IGKV1D-39	6.932	6.916	6.865	6.927	7.139	7.023

VPS8	9.636	9.533	9.623	9.634	9.644	9.735
IGKV1D-37	7.315	7.353	7.191	7.351	7.391	7.278
OR12D2	7.748	7.721	7.571	7.927	7.836	7.924
OR12D3	7.315	7.429	7.298	7.443	7.671	7.557
AL603962.1	7.140	7.171	7.123	6.908	7.061	7.082
ARPIN	8.740	8.807	8.837	8.426	8.475	8.292
TYW1	8.956	8.968	8.766	8.802	8.816	8.952
AC093884.1	9.431	9.559	9.700	9.278	9.579	9.304
SLC9C2	4.558	4.178	4.520	4.509	4.339	4.259
BRS3	6.657	6.838	6.626	6.496	6.730	6.679
ARHGEF10	8.561	8.656	8.669	8.574	8.406	8.503
ARHGEF11	8.819	8.938	8.986	8.740	8.855	8.806
ARHGEF12	10.046	10.049	10.028	9.939	9.877	10.028
ARHGEF15	8.126	8.147	8.095	8.338	8.266	8.191
ARHGEF16	9.076	9.225	9.292	9.140	9.059	9.195
ARHGEF17	9.084	9.163	9.166	9.242	9.220	9.172
ARHGEF18	10.572	10.646	10.701	10.482	10.385	10.452
ARHGEF19	8.361	8.486	8.503	8.602	8.405	8.286
NEUROD6	7.230	7.194	6.687	7.245	7.284	7.148
AL049545.1	4.007	3.863	3.858	4.084	4.098	3.836
AC011611.4	6.231	6.191	6.155	6.226	6.276	6.359
D2HGDH	8.323	8.299	8.381	8.222	8.110	8.198
MITD1	8.062	8.125	8.246	7.814	7.805	7.940
PAIP2B	7.906	8.069	7.918	7.847	7.821	7.812
SERF1A	8.829	8.864	8.871	8.733	8.624	8.718
AL133500.1	8.320	8.215	8.572	8.452	8.144	8.323
SERF1B	8.829	8.864	8.871	8.733	8.624	8.718
HINFP	8.391	8.397	8.549	8.271	8.406	8.379
Z99774.4	6.221	6.189	6.137	6.357	6.531	6.222
GPLD1	7.455	7.533	7.189	7.337	7.411	7.330
OR2W3	5.511	5.589	5.582	5.824	5.614	5.743
OR2W1	4.772	4.669	4.712	4.744	4.753	4.813
AL359273.2	8.033	8.079	8.139	7.785	7.899	7.949
C1orf162	7.177	7.149	7.286	7.336	7.180	7.473
LINC00471	4.546	4.510	4.350	4.579	4.407	4.486
AC107953.2	7.899	8.038	7.892	8.027	8.127	8.217
AL049840.6	8.877	8.906	8.817	8.598	8.613	8.710
AL049840.7	4.441	4.741	4.587	4.525	4.634	4.457
INMT-MINDY4	8.636	8.629	8.771	8.580	8.591	8.622
AC139783.1	5.577	5.427	5.429	5.491	5.611	5.473
SLC39A8	8.636	8.731	8.814	8.349	8.361	8.430
SLC39A9	11.192	11.319	11.427	11.164	11.224	11.170
SLC39A1	9.401	9.598	9.678	9.419	9.520	9.429
SLC39A2	7.433	7.647	7.407	7.842	7.792	7.588
SLC39A3	9.151	9.357	9.494	9.251	9.308	9.130
SLC39A4	8.145	8.177	8.336	8.121	8.181	8.227
SLC39A5	9.225	9.286	9.223	9.365	9.366	9.311
SLC39A6	10.338	10.444	10.500	10.144	10.038	10.132
SLC39A7	8.666	8.876	9.006	8.853	8.790	8.637
TCAF2	7.650	7.730	7.688	7.716	7.904	7.828
TCAF1	9.720	9.517	9.413	9.339	9.518	9.644
PSIP1	10.012	9.925	10.162	9.459	9.623	9.529
MTHFD1L	9.483	9.586	9.622	9.206	9.209	9.250
AC008895.1	4.904	4.807	4.696	4.804	4.810	4.810
AP001922.1	5.480	5.398	5.648	5.525	5.651	5.645
RET	8.767	8.577	8.359	8.593	8.634	8.825

AC069547.1	5.350	5.334	5.367	5.583	5.713	5.396
RPL21P119	12.617	12.677	12.802	12.784	12.769	12.841
REN	6.998	7.250	7.009	7.091	7.369	7.569
REL	8.209	8.185	8.157	8.417	8.318	8.536
TRIM73	9.228	9.178	8.973	9.098	9.150	9.334
TRIM72	7.564	7.389	7.666	7.433	7.624	7.587
TP53TG3F	7.710	7.765	7.579	7.766	7.843	7.804
TP53TG3D	7.710	7.765	7.579	7.766	7.843	7.804
TP53TG3E	7.710	7.765	7.579	7.766	7.843	7.804
TP53TG3B	7.710	7.765	7.579	7.766	7.843	7.804
TP53TG3C	7.710	7.765	7.579	7.766	7.843	7.804
SH3GL3	6.964	7.075	6.838	6.958	7.139	7.158
MIR9-3HG	7.296	7.397	7.310	7.357	7.458	7.622
MIR155HG	4.376	4.236	4.373	4.654	4.631	4.462
TLCD3B	7.435	7.326	7.553	7.930	7.773	7.421
DNM1P34	10.924	10.924	10.998	10.932	11.119	11.157
DNM1P35	5.750	5.795	5.739	5.968	6.009	5.721
SCAF4	8.473	8.508	8.573	8.369	8.531	8.373
SCAF1	9.007	9.055	9.404	9.237	9.375	9.118
KIR2DL5B	8.835	8.689	8.488	8.744	8.793	8.879
KIR2DL5A	8.835	8.689	8.488	8.744	8.793	8.879
SCAF8	9.575	9.601	9.717	9.376	9.318	9.393
TMEM63A	9.063	9.241	9.319	8.941	8.939	9.127
TMEM63C	6.314	6.522	6.484	6.831	6.491	6.479
TMEM63B	9.599	9.493	9.654	9.777	9.628	9.515
RBM7	9.707	9.800	9.802	9.717	9.810	9.754
RBM6	7.534	7.263	7.534	7.263	7.460	7.344
RBM5	9.642	9.655	9.503	9.484	9.463	9.600
RBM4	10.358	10.473	10.607	10.302	10.352	10.322
RBM3	10.626	10.811	10.782	10.390	10.290	10.510
AL591242.1	5.746	5.759	5.647	5.978	5.828	5.985
CAT	11.444	11.479	11.620	11.490	11.363	11.365
CAD	10.136	10.211	10.234	9.912	9.752	9.953
COA8	8.631	8.787	8.880	8.719	8.668	8.710
COA5	8.952	9.028	9.111	8.855	8.982	9.080
COA4	9.455	9.617	9.722	9.621	9.511	9.667
COA7	10.064	9.828	9.868	9.579	9.760	9.693
COA6	9.899	9.945	10.099	10.008	9.973	9.981
COA1	9.689	9.823	9.932	9.825	9.793	9.736
COA3	11.627	11.673	11.879	11.572	11.570	11.638
SEPSECS	7.574	7.709	7.635	7.323	7.228	7.386
LYZL6	7.445	7.513	7.614	7.435	7.571	7.591
ARSD	10.858	11.011	11.196	11.134	10.831	10.896
CA9	9.425	9.530	9.867	9.453	9.917	9.587
CA8	6.900	6.585	6.710	7.172	7.144	7.128
ARSA	9.160	9.283	9.395	9.302	9.302	9.280
ARSB	7.180	7.478	7.213	7.061	7.115	7.206
CA3	4.357	4.023	4.016	3.858	4.189	4.192
OGT	10.446	10.453	10.488	10.122	10.053	10.366
CA1	8.687	8.743	8.514	8.777	8.798	8.871
HEXB	11.679	11.785	11.958	11.597	11.244	11.577
CEPT1	8.720	8.381	8.623	8.539	8.539	8.521
HEXD	9.157	9.179	9.242	9.137	9.080	9.207
ARSJ	7.545	7.550	7.693	7.463	7.549	7.513
CA4	7.159	7.048	7.299	7.167	7.202	7.320
OGN	4.400	4.214	4.218	4.243	4.407	4.086

OGA	9.356	9.295	9.344	9.167	8.974	9.169
UFC1	11.128	11.231	11.403	11.222	11.098	11.227
MKX	4.074	4.172	4.255	4.307	4.225	4.328
FBLN2	7.063	6.965	7.181	7.273	7.351	7.366
FBLN1	7.888	7.878	7.799	7.770	7.870	7.902
FBLN7	7.554	7.342	7.181	7.354	7.737	7.431
AKIP1	9.897	9.870	9.985	9.425	9.757	9.821
FBLN5	7.390	7.040	7.054	6.966	6.991	7.058
CD24P4	12.880	12.904	12.962	12.564	12.635	12.959
CD24P2	12.754	12.900	12.778	12.564	12.635	12.959
NDUFC2-KCTD14	12.582	12.712	12.796	12.604	12.609	12.639
C2orf74	8.162	8.126	8.265	7.834	7.758	7.998
THUMPD3-AS1	8.870	8.668	8.395	8.795	8.662	8.872
CYLD	8.331	8.373	8.365	8.424	8.374	8.613
HCRTR1	4.995	4.898	4.685	5.224	5.240	5.062
HCRTR2	6.144	6.083	6.167	6.348	6.077	6.184
CBWD4P	9.601	9.604	9.761	9.496	9.589	9.562
ZBED6CL	7.659	7.761	7.930	7.565	7.400	7.463
IGHV3-73	4.849	4.672	4.764	4.938	4.910	4.930
IGHV3-72	5.181	5.139	5.245	5.454	5.070	5.184
IGHV3-74	6.473	6.336	6.420	6.449	6.462	6.415
ZNF185	10.396	10.381	10.478	10.367	10.319	10.357
ZNF184	7.719	7.760	7.884	7.728	7.871	7.676
ZNF181	6.564	6.509	6.580	6.915	6.910	6.939
ZNF180	7.544	7.588	7.633	7.433	7.341	7.429
ZNF182	7.968	7.844	7.983	7.937	7.765	7.737
ZNF189	8.856	8.953	8.828	8.819	8.845	8.849
ALYREF	10.597	10.257	10.581	10.055	9.996	9.837
HYKK	8.093	8.247	8.435	7.947	8.110	7.989
SEMA5B	10.207	10.343	10.323	10.290	10.277	10.507
SEMA5A	8.316	8.427	8.483	8.176	8.213	8.331
LYNX1	8.086	8.270	8.187	8.011	8.379	8.218
SCN5A	7.585	7.795	7.610	7.815	7.710	7.839
CABCOCO1	4.867	4.847	4.756	4.964	4.963	4.893
MOAP1	10.079	9.988	10.202	9.816	9.703	9.760
ZNF239	7.651	7.535	7.602	7.527	7.385	7.588
ZNF236	9.756	9.680	9.370	9.740	9.821	9.906
ZNF235	7.035	7.140	6.942	6.828	6.946	6.957
ZNF234	6.710	6.693	6.726	6.353	6.394	6.537
ZNF233	6.735	6.802	6.865	6.725	6.746	6.928
ZNF232	8.049	8.061	8.535	7.944	7.990	8.029
ZNF230	6.413	6.444	6.458	6.262	6.264	6.195
AC009303.4	7.509	7.329	7.473	7.309	7.255	7.386
AC027702.2	6.220	6.376	6.174	6.465	6.280	6.417
AC092068.3	7.898	7.957	7.968	8.012	7.827	7.943
TLE1P1	8.771	8.866	8.941	8.281	8.392	8.435
AC104663.1	4.180	4.163	4.136	4.244	4.178	4.167
AL157786.1	4.183	4.071	3.921	4.075	4.287	4.238
GON7	9.047	9.174	9.254	9.140	9.193	9.131
KCNRG	7.107	7.265	6.952	7.086	7.193	7.022
RPL5P34	13.001	13.062	13.190	13.066	13.122	13.074
TGFB1	9.196	9.256	9.381	9.060	9.276	9.237
TGFB2	10.096	10.038	10.073	9.862	9.853	10.014
TGFB3	8.384	8.196	8.030	8.302	8.089	8.351
C17orf99	5.954	5.760	5.934	6.162	5.981	5.903
C17orf98	4.349	4.336	4.139	4.070	4.241	4.529

MTERF2	7.659	7.564	7.592	7.661	7.647	7.720
MTERF3	7.671	7.678	7.925	7.619	7.742	7.672
C17orf97	8.438	8.394	8.338	8.234	8.342	8.270
MTERF4	7.893	7.975	7.953	7.783	7.584	7.698
LOH12CR2	7.031	7.260	7.333	6.898	7.162	7.069
GLIDR	8.597	8.585	8.594	8.520	8.633	8.531
AC007336.3	6.977	6.757	6.841	7.116	7.012	6.956
AC135586.2	10.098	10.032	10.305	9.861	9.765	9.915
LINC01362	5.148	5.230	5.410	5.403	5.148	5.466
AC010891.1	5.726	5.653	5.349	5.676	5.859	5.820
LINC01360	5.071	5.117	5.162	5.254	5.166	5.169
LINC01366	6.549	6.511	6.365	6.624	6.779	6.688
LINC01365	7.458	7.600	7.660	7.414	7.619	7.561
LINC01364	5.289	5.190	4.970	5.220	5.210	5.148
PEG13	7.258	7.361	7.336	7.455	7.422	7.404
AC131281.1	7.510	7.529	7.579	7.400	7.484	7.379
PEG10	11.302	11.269	11.352	11.448	11.181	11.437
ARPIN-AP3S2	10.782	10.833	11.008	10.778	10.738	10.728
HSPD1P1	13.826	13.845	13.862	13.917	13.851	13.843
HSPD1P4	13.826	13.845	13.862	13.917	13.851	13.843
VAMP1	6.679	6.666	6.497	6.706	6.732	6.668
MIR4449	10.585	10.697	10.794	10.717	10.645	10.840
VAMP2	8.822	8.718	8.818	8.969	8.892	8.774
VAMP5	6.777	6.464	6.640	6.473	6.475	6.519
VAMP4	7.694	7.817	8.068	7.780	7.587	7.687
VAMP7	10.330	10.350	10.322	10.248	10.054	10.161
VAMP8	10.969	11.078	11.256	11.126	11.053	11.053
AL139246.10	5.987	6.033	6.083	6.526	5.928	6.134
TGFBI	10.643	10.660	10.852	10.508	10.338	10.485
TAPT1	8.913	8.975	9.033	8.656	8.688	8.636
AC009560.1	5.395	5.590	5.769	5.447	5.379	5.628
LINC01949	5.689	5.930	6.003	5.793	5.549	5.626
LINC01943	5.629	5.374	5.700	5.650	5.538	5.584
PECAM1	7.024	7.300	7.004	7.006	7.022	7.196
AL606970.1	6.971	6.968	6.981	7.485	7.424	7.366
VRK1	8.255	8.311	8.293	7.935	7.936	7.830
VRK3	10.468	10.429	10.509	10.284	10.549	10.393
VRK2	8.821	8.861	8.952	9.040	9.006	9.066
CRYL1	8.726	8.767	8.976	8.623	8.682	8.761
AP001021.1	6.378	6.384	6.469	6.675	6.417	6.473
NR2C2	8.588	8.473	8.423	8.147	8.206	8.354
TNIP3	4.715	5.014	4.740	4.879	4.882	4.893
NR2C1	8.263	8.324	8.232	8.065	8.211	8.173
U91319.1	6.892	6.766	6.704	6.849	7.065	6.770
AC127526.1	8.196	8.262	8.287	8.740	8.301	8.304
CYREN	9.945	9.925	10.110	9.931	9.916	9.827
AL162274.2	6.912	6.969	7.361	7.693	7.187	7.033
FRA10AC1	7.928	8.018	8.275	7.539	7.635	7.821
AL136114.1	4.622	4.722	4.583	4.663	4.707	4.663
ATP2C2-AS1	7.794	7.751	8.020	8.241	8.013	7.840
MAU2	9.652	9.717	9.611	9.555	9.789	9.541
FAM184A	7.837	7.807	7.801	7.609	7.611	7.711
FAM184B	5.896	6.086	5.694	5.977	6.180	6.061
SLC23A1	6.858	6.810	6.628	6.866	6.950	7.008
HSPH1	11.570	11.641	11.548	11.637	11.629	11.828
AC008575.1	10.020	10.007	10.002	9.790	9.859	9.910

SEC1P	6.598	6.111	6.516	6.757	6.413	6.491
BASP1	6.690	6.396	6.304	6.625	6.899	6.720
PLEKHB1	10.080	10.155	10.307	9.743	9.798	9.828
SLC23A2	8.515	8.500	8.528	8.205	8.212	8.240
SEC13	10.706	10.688	10.820	10.569	10.603	10.443
GIMAP8	5.581	5.818	5.792	5.657	5.768	5.974
GIMAP4	4.793	4.610	4.706	4.863	4.796	4.785
GIMAP5	8.693	8.874	8.803	8.853	8.997	9.078
GIMAP6	6.402	6.272	6.382	6.397	6.596	6.604
GIMAP7	3.941	3.774	3.858	4.108	4.032	3.941
GIMAP1	7.314	7.344	7.254	7.265	7.286	7.368
GIMAP2	5.636	5.313	5.486	5.726	5.797	5.374
RPL18AP3	13.186	13.284	13.431	13.346	13.253	13.366
JMJD7-PLA2G4B	9.660	9.771	9.686	9.544	9.800	9.749
SPINT1-AS1	8.877	9.042	8.979	8.849	8.917	9.086
CEP19	6.414	6.390	6.709	6.352	6.472	6.285
MYOM3	7.298	7.455	7.653	7.629	7.441	7.541
MYOM2	5.948	6.294	6.251	6.097	6.384	6.431
MYOM1	8.126	7.991	7.962	7.871	7.881	7.897
AP003385.5	9.849	9.926	10.029	9.810	9.880	9.911
SYCE1	6.891	6.926	7.028	6.796	6.958	6.942
FBXO2	7.485	7.602	7.802	7.764	7.396	7.730
FBXO3	8.619	8.789	8.764	8.546	8.540	8.732
FBXO4	8.041	8.066	8.218	7.723	7.939	8.106
FBXO5	8.958	8.766	8.875	8.813	8.721	8.648
FBXO6	7.230	7.426	7.506	6.931	7.360	7.089
FBXO7	10.607	10.707	10.841	10.639	10.715	10.642
FBXO8	8.839	8.847	8.919	8.584	8.578	8.744
FBXO9	10.402	10.339	10.367	10.164	10.111	10.172
IGKV1OR2-108	7.494	7.663	7.798	7.484	7.577	7.686
ASTN1	4.894	5.023	4.802	5.101	5.101	5.050
AL512506.2	9.413	9.526	9.484	9.028	9.146	9.297
AL512506.3	6.367	6.329	6.328	6.504	6.417	6.297
TRAM2-AS1	7.974	8.159	8.291	7.883	8.143	8.059
TAL1	7.963	7.961	8.095	8.142	7.865	8.120
MYEF2	8.977	8.900	8.828	8.848	8.862	9.010
TAL2	8.296	8.215	8.265	8.560	8.350	8.353
RITA1	10.324	10.416	10.529	10.351	10.357	10.323
AC004453.1	13.951	13.948	14.015	14.017	14.031	14.037
AP001160.3	7.172	7.116	6.987	7.204	7.214	7.028
AP001160.2	6.144	6.078	5.929	6.265	6.002	6.119
HROB	8.337	8.189	8.207	8.321	8.270	8.317
AP001160.4	8.879	8.840	9.061	8.753	8.896	8.892
CCDC85C	8.811	8.946	9.097	8.758	8.745	8.673
CCDC85B	10.227	10.286	10.541	10.253	10.127	10.231
CCDC85A	7.690	7.718	7.726	7.716	7.818	7.811
CMSS1	8.862	8.838	9.107	8.898	8.827	8.924
SNTB2	9.412	9.463	9.442	9.060	9.585	9.076
SNTB1	8.500	8.503	8.659	8.440	8.720	8.577
GU182359.4	8.835	8.689	8.488	8.744	8.793	8.879
AC090409.1	6.799	6.866	6.665	6.890	6.891	6.846
KIAA0408	4.512	4.561	4.616	4.594	4.670	4.723
DZANK1	7.107	7.283	7.246	7.146	7.041	7.139
CORO1B	10.930	11.019	11.208	10.968	10.992	10.964
CORO1C	11.872	11.930	11.960	11.943	11.807	11.877
CORO1A	8.179	8.319	8.261	8.190	8.163	8.015

MID2	8.462	8.571	8.426	8.452	8.576	8.427
MID1	10.592	10.415	10.455	10.150	10.132	10.508
WASF1	7.788	7.680	7.567	7.576	7.544	7.717
WASF2	11.406	11.694	11.743	11.437	11.615	11.147
WASF3	8.554	8.474	8.657	8.159	8.174	8.371
NAIPP2	5.835	5.745	5.792	5.711	6.008	5.820
YWHAZ	12.392	12.477	12.468	12.443	12.360	12.522
MYCT1	6.499	6.376	6.209	6.531	6.296	6.285
LINC01361	4.020	4.240	3.795	4.389	4.117	4.235
SORT1	10.688	10.620	10.725	10.662	10.572	10.732
RTL5	7.566	7.710	7.848	8.010	7.728	7.890
MIDN	9.269	9.555	9.661	9.582	9.563	9.355
LINC02579	7.205	7.069	7.176	7.081	7.064	7.227
SHTN1	9.141	9.109	9.139	8.795	8.666	8.930
LINC02573	7.147	7.052	7.038	7.089	7.069	7.251
LINC02577	8.182	8.226	8.094	7.913	7.994	7.917
LINC02575	7.115	7.281	7.258	7.265	7.228	7.347
LINC02574	6.955	7.030	7.172	7.547	7.149	7.180
HECTD2	5.051	5.014	5.100	4.962	4.978	5.225
HECTD3	9.104	9.054	8.905	8.953	8.889	8.809
MALRD1	5.588	5.533	5.664	5.710	5.763	5.676
HECTD4	8.668	8.480	8.687	8.563	8.405	8.450
LZTFL1	8.019	8.030	8.106	7.486	7.730	7.721
ADAD1	7.919	7.849	7.459	7.840	7.945	7.666
ADAD2	7.164	7.311	7.450	7.458	7.317	7.508
DIAPH1-AS1	5.046	4.991	5.002	4.994	5.163	5.022
AC009831.1	5.343	5.255	5.288	5.211	5.267	5.451
FLII	10.160	10.200	10.313	10.178	9.980	10.057
RALGAPA1	7.933	7.716	7.886	7.516	7.416	7.765
RALGAPA2	8.545	8.612	8.636	8.394	8.502	8.584
AC109486.2	7.744	7.705	7.698	7.965	7.686	7.740
AC109486.3	7.744	7.705	7.698	7.965	7.686	7.740
AC073862.1	7.025	7.112	7.218	6.931	7.040	6.873
FLI1	7.476	7.224	7.240	7.432	7.604	7.492
AC073862.5	7.025	7.112	7.218	6.931	7.040	6.873
AC084816.1	5.731	5.937	5.738	5.823	5.788	5.852
GPR22	6.444	6.263	5.922	6.719	6.733	6.511
GPR20	6.644	6.490	6.602	6.662	6.542	6.630
AC100803.3	8.676	8.654	8.707	8.657	8.849	8.725
AC234301.3	7.842	8.095	8.219	8.243	8.177	8.357
AC234301.2	6.356	6.471	6.499	6.503	6.500	6.426
AC100803.7	8.676	8.654	8.707	8.657	8.849	8.725
KCNMB1	8.144	8.158	7.720	8.160	8.261	8.329
KCNMB3	7.578	7.737	7.714	7.448	7.450	7.692
KCNMB2	7.345	7.615	7.284	7.523	7.656	7.553
PNCK	7.597	7.761	7.708	7.676	7.651	7.707
KCNMB4	9.137	9.250	9.386	8.894	8.971	9.014
AC016394.3	7.425	7.305	7.110	7.432	7.140	7.393
AC016394.2	7.042	7.350	7.072	7.001	7.235	7.067
YWHAG	12.237	12.220	12.283	12.171	12.069	12.209
LINC01002	10.722	10.564	10.478	10.430	10.307	10.610
LINC01003	7.502	7.542	7.448	7.421	7.300	7.558
LINC01001	10.722	10.564	10.478	10.430	10.307	10.610
LINC01006	7.715	7.859	7.814	7.716	7.697	7.553
LINC01007	7.222	7.101	6.670	7.054	7.318	7.199
LINC01004	4.499	4.530	4.649	4.407	4.253	4.469

SNORA69	14.178	14.223	14.242	14.319	14.368	14.265
POC5	8.351	8.378	8.460	8.284	8.334	8.404
AC008269.2	5.959	6.073	5.556	5.836	6.071	5.891
SPECC1L	9.113	9.194	9.319	9.098	8.946	9.134
NPRL3	9.536	9.527	9.465	9.579	9.592	9.449
NPRL2	9.494	9.537	9.648	9.451	9.473	9.438
PLXNB2	11.996	12.034	12.188	11.937	11.782	11.917
PLXNB3	8.150	8.135	8.166	8.103	8.284	8.035
SHCBP1L	6.954	6.982	7.015	7.131	7.042	6.990
PLXNB1	9.530	9.551	9.507	9.324	9.328	9.449
EEF1AKNMT	9.920	9.896	9.910	9.874	9.610	9.772
AC100858.4	6.110	6.283	6.136	6.021	6.451	6.280
UQCRHL	12.550	12.633	12.742	12.675	12.548	12.679
FAM230I	7.306	7.390	7.332	7.774	7.596	7.548
SIRT3	10.247	10.350	10.403	10.254	10.456	10.433
SIRT2	9.851	9.876	10.115	9.868	9.764	9.896
SIRT1	8.292	8.373	8.332	8.118	8.219	8.220
SIRT7	9.117	9.082	9.292	8.984	8.987	9.073
SIRT6	9.066	9.181	9.232	9.024	9.059	9.132
SIRT5	7.809	7.760	7.923	7.821	7.765	7.771
SIRT4	5.764	5.542	5.579	5.942	5.715	5.846
MAS1L	7.482	7.276	7.203	7.447	7.091	7.291
C10orf143	5.593	5.728	5.168	5.797	5.722	5.632
FSD1	7.261	7.523	7.673	7.479	7.554	7.718
FSD2	7.029	7.394	7.094	7.389	7.225	7.355
CINP	7.889	7.799	7.778	7.809	7.913	7.924
TRHDE-AS1	7.693	7.561	7.375	7.575	7.631	7.512
AC007405.2	6.256	6.258	5.944	6.338	6.103	6.423
PBOV1	6.176	6.304	6.356	6.429	6.507	6.447
CES5A	6.404	6.401	6.385	6.556	6.493	6.465
KDM6B	9.434	9.423	9.439	9.722	10.048	9.781
DSCAM-AS1	6.351	6.000	6.081	6.177	6.137	6.163
SLC20A2	9.630	9.822	9.725	9.516	9.454	9.610
WASH8P	9.341	9.202	9.345	9.278	9.301	9.395
VAMP3	11.489	11.485	11.627	11.459	11.327	11.371
CC2D2A	8.068	8.018	8.263	8.034	7.932	7.733
CC2D2B	8.504	8.037	7.757	8.443	8.511	8.427
SLC20A1	10.939	10.963	11.030	10.618	10.508	10.534
PRTG	6.815	6.694	6.613	6.860	7.057	6.909
AC013391.3	7.915	7.778	7.625	7.845	7.844	7.902
TRAV26-2	7.012	6.762	7.097	7.369	7.165	7.042
PAPPA2	8.096	8.035	7.915	8.057	8.174	8.136
AC022915.2	6.413	6.591	6.496	6.346	6.592	6.411
ARHGAP11A	4.402	4.506	4.753	4.254	4.255	4.478
ARHGAP11B	5.548	5.505	5.393	5.586	5.636	5.734
CRCT1	5.602	5.887	5.716	5.666	5.990	5.840
ROCK2	8.876	8.739	8.701	8.912	9.121	9.032
TIFAB	7.086	7.207	7.240	7.194	7.247	7.145
AC090825.1	6.105	6.163	6.493	6.391	6.391	6.401
LUARIS	6.145	6.209	6.201	6.388	6.411	6.467
AC006333.1	7.537	7.690	7.729	7.535	7.619	7.794
NAGS	7.528	7.486	7.775	7.595	7.607	7.621
AC009166.1	6.422	6.451	6.643	6.695	6.831	6.186
TENT5B	6.142	6.262	6.303	6.229	6.192	6.259
TENT5C	6.427	6.409	6.466	6.558	6.136	6.539
TENT5A	9.198	9.550	9.635	10.178	10.174	9.857

Log2 normalized expression

TENT5D	4.822	4.874	4.852	4.902	5.049	4.819
AC106795.1	10.796	10.924	11.020	10.731	10.555	10.739
AC106795.4	10.796	10.924	11.020	10.731	10.555	10.739
NAGA	9.915	9.953	10.108	9.699	9.564	9.744
NAGK	10.182	10.222	10.399	10.204	10.043	10.194
TEX36-AS1	4.550	4.699	4.653	4.563	4.703	4.758
FOLH1B	7.129	7.213	7.288	7.206	7.150	7.154
CARTPT	6.531	6.571	6.534	6.713	6.589	6.723
PGAP1	5.862	5.735	5.597	5.839	5.741	5.671
PGAP3	13.594	13.683	13.764	13.533	13.492	13.549
PGAP2	10.008	10.065	10.016	9.900	9.841	9.927
PGAP4	9.160	9.288	9.446	8.996	9.036	9.101
PGAP6	10.454	10.577	10.718	10.512	10.500	10.445
WLS	10.362	10.430	10.421	10.029	10.109	10.193
MFAP5	6.235	5.961	6.099	6.175	6.245	6.264
MFAP4	7.751	7.903	7.780	7.680	7.735	7.853
RTL8C	11.053	11.080	11.218	11.044	11.089	11.089
MFAP2	7.876	7.966	7.909	7.729	7.751	7.909
RTL8A	9.893	9.970	10.054	9.854	9.849	9.793
IGHV3-7	6.371	6.336	6.390	6.466	6.589	6.644
AL139246.3	5.987	6.033	6.083	6.526	5.928	6.134
AC124944.2	9.390	9.411	9.316	9.060	9.420	9.381
TMEM132D-AS2	4.949	4.924	4.990	5.071	4.996	5.016
OFD1	10.020	9.965	10.142	9.780	9.711	9.879
HBS1L	10.228	10.236	10.396	10.249	9.992	10.224
AC025164.2	8.041	8.115	7.992	8.258	8.148	8.019
GYPA	5.659	5.624	5.528	6.080	5.664	5.777
GYPB	8.345	8.470	8.280	8.333	8.615	8.456
GYPC	7.681	7.874	7.673	7.999	8.030	7.998
GYPE	5.245	5.152	5.338	5.250	5.472	5.317
FOXP1-IT1	7.261	7.342	7.398	7.521	7.357	7.528
SNAP25	8.178	8.308	8.402	8.355	8.205	8.573
SNAP23	10.125	10.141	10.197	10.096	10.161	10.118
LITAF	12.578	12.717	12.767	12.498	12.457	12.532
SNAP29	9.414	9.478	9.566	9.345	9.410	9.237
C11orf49	9.289	9.182	9.449	9.176	9.319	9.184
C11orf40	4.916	4.941	4.772	4.798	5.070	5.143
C11orf42	6.984	6.893	6.709	7.017	7.057	7.168
C11orf44	5.884	6.234	5.853	5.902	6.088	6.101
AL662852.1	8.529	8.524	8.065	8.442	8.567	8.533
TNFSF13	9.155	9.155	9.269	8.997	9.062	9.014
TNFSF12	7.525	7.564	7.648	7.722	7.803	7.680
TNFSF11	6.236	6.343	6.484	6.798	6.602	6.650
TNFSF10	11.726	11.740	11.821	11.766	12.057	11.629
DIRAS1	7.237	6.956	6.960	6.894	7.166	7.028
DIRAS3	7.261	7.342	7.483	6.512	7.186	6.922
DIRAS2	6.818	6.983	6.914	6.706	7.035	6.976
TNFSF18	6.488	6.840	6.343	6.762	6.734	6.704
SFXN4	8.896	9.018	8.994	8.894	8.785	8.948
SFXN5	8.079	8.145	8.202	8.128	8.115	8.177
SFXN2	9.337	9.320	9.251	9.093	9.217	9.243
SFXN3	9.298	9.314	9.270	9.188	9.091	9.209
SFXN1	9.432	9.354	9.432	9.198	9.370	9.375
TAS2R1	7.341	7.632	7.421	7.480	7.555	7.550
CCT8	11.776	11.902	11.954	11.851	11.661	11.827
CCT2	12.683	12.748	12.851	12.783	12.570	12.666

CCT3	12.588	12.648	12.793	12.731	12.512	12.683
CCT7	11.835	11.864	12.127	11.853	11.664	11.765
CCT4	13.001	13.028	13.108	13.066	12.902	13.085
CCT5	11.725	11.817	11.947	11.926	11.804	11.816
AC022726.2	4.442	4.383	4.411	4.565	4.610	4.468
CLIC4P1	8.189	8.109	8.191	8.084	8.184	8.120
SWI5	8.373	8.394	8.400	8.379	8.267	8.283
RPL23AP82	7.667	7.929	8.164	7.639	7.672	7.716
RPL23AP84	7.667	7.929	8.164	7.639	7.672	7.716
RPL23AP87	7.667	7.929	8.164	7.639	7.672	7.716
RPL23AP88	7.667	7.929	8.164	7.639	7.672	7.716
AL031719.1	8.563	8.483	8.537	8.619	8.701	8.632
AC005549.1	6.945	7.000	6.977	7.183	7.216	7.247
AC126178.1	4.760	4.855	4.787	5.136	4.850	4.899
MYF5	5.360	5.245	5.389	5.417	5.500	5.253
ASPG	7.846	8.138	8.296	7.889	7.859	7.830
ASPA	6.701	6.432	6.129	6.666	6.637	6.620
ASPM	9.308	9.064	9.189	8.369	8.288	8.495
ASPN	5.549	5.383	5.220	5.540	5.608	5.287
ASPH	12.167	12.265	12.229	11.955	11.899	12.143
CKMT2-AS1	7.215	7.127	7.234	7.031	6.901	7.203
FAM86B2	8.505	8.587	8.677	8.446	8.587	8.509
FAM86B1	9.708	9.675	9.729	9.656	9.678	9.650
UBE2V1	12.225	12.293	12.363	12.065	12.065	12.079
UBE2V2	8.976	9.115	9.154	9.036	8.917	9.127
CMBL	9.469	9.524	9.682	9.319	9.364	9.269
ZNF880	5.438	5.817	5.630	5.576	5.655	5.666
ZNF883	4.573	4.364	4.434	4.515	4.684	4.515
C1GALT1C1	9.438	9.527	9.665	9.501	9.439	9.397
HBZP1	6.116	5.837	6.095	6.037	6.134	6.117
SSTR3	8.009	8.024	7.986	8.084	8.055	8.142
SSTR2	7.830	7.881	7.573	7.884	7.915	7.789
SSTR5	7.111	6.991	7.233	7.426	7.505	7.440
SSTR4	7.330	7.417	7.331	7.264	7.545	7.458
LDLRAP1	8.115	8.346	8.517	7.970	8.186	8.103
CTNNB1	10.660	10.354	10.535	10.196	10.107	10.222
AC011095.1	6.716	6.649	6.369	6.647	6.891	6.693
WNK2	8.466	8.732	8.749	8.553	8.645	8.593
WNK1	10.504	10.477	10.690	10.417	10.447	10.215
ATN1	9.670	9.661	9.726	9.719	9.887	9.732
LRRC2	3.774	3.934	3.914	3.801	3.972	3.925
TOB1	11.269	11.211	11.312	11.294	11.344	11.344
TNIP2	9.081	9.255	9.192	9.025	9.069	9.025
TNIP1	11.511	11.551	11.767	11.505	11.435	11.492
MAN1B1	9.742	9.843	10.112	9.575	9.536	9.647
LINC00470	7.067	7.253	6.976	6.916	7.262	7.150
LBP	7.732	7.650	7.603	8.060	8.035	7.921
LBR	11.323	11.354	11.362	10.951	10.976	11.008
LINC02777	7.151	7.188	7.112	7.075	7.064	7.308
LINC02770	4.995	4.905	4.713	4.995	5.057	4.859
GZF1	6.249	6.434	6.478	6.708	6.444	6.520
IMP3	11.419	11.661	11.810	11.833	11.718	11.621
IMP4	10.143	10.230	10.370	10.158	10.027	10.089
DAOA-AS1	4.393	4.477	4.228	4.379	4.334	4.385
POU5F1P3	6.825	7.002	6.830	6.818	6.850	6.976
RFLNA	7.762	7.714	7.822	7.760	7.725	7.944

Log2 normalized expression

ACIN1	9.052	9.042	9.115	9.205	9.146	8.940
ATP1B2	7.158	7.340	7.211	7.525	7.283	7.177
ATP1B3	11.667	11.626	11.777	11.611	11.580	11.582
POU5F1P4	8.619	8.606	8.654	8.607	8.622	8.640
ATP1B1	13.741	13.847	13.856	13.845	13.982	13.820
BX255923.2	6.037	5.681	5.593	5.953	5.932	5.915
TFB1M	8.862	8.896	8.859	8.812	8.901	8.801
PLD2	8.238	8.176	8.243	8.257	8.272	8.375
NOS1AP	8.432	8.492	8.423	8.432	8.142	8.425
TSEN15	9.992	10.125	9.974	10.010	9.921	9.842
PLD3	9.916	10.055	10.224	9.980	9.890	9.911
PIP5KL1	7.314	7.396	7.481	7.703	7.449	7.453
AP000821.1	6.899	6.897	6.895	7.061	7.029	7.079
AC026950.1	7.600	7.754	7.411	7.762	7.971	7.809
SNX3	12.269	12.336	12.434	12.553	12.493	12.502
SNX2	10.337	10.530	10.617	10.287	10.180	10.298
SNX1	10.611	10.610	10.701	10.404	10.237	10.276
SNX7	9.860	10.044	10.209	9.882	9.836	9.870
SNX6	10.645	10.787	10.836	10.957	10.881	10.880
SNX5	9.965	9.887	10.017	9.798	9.880	9.902
SNX4	9.832	9.934	9.877	9.714	9.863	9.835
RPL27A	13.836	13.822	13.906	13.931	13.893	13.957
SNX8	9.466	9.550	9.671	9.304	9.153	9.042
AC084117.1	13.989	14.054	14.034	14.125	14.099	14.165
AC084117.2	13.989	14.054	14.034	14.125	14.099	14.165
TRAV9-2	5.872	5.642	5.965	5.892	5.882	5.817
MIR3614	9.433	9.480	9.265	9.217	9.513	9.166
KHSRP	10.278	10.340	10.297	9.981	10.462	9.998
TIMELESS	9.330	9.413	9.487	8.919	8.980	9.092
AL603840.1	6.013	5.981	5.854	6.207	6.252	6.114
NUTM2B-AS1	7.117	7.208	7.108	7.153	7.087	7.211
PHKG2	8.778	8.818	8.864	8.651	8.651	8.653
PHKG1	7.263	7.489	7.201	7.631	7.471	7.555
ORAI2	9.772	9.757	9.850	9.618	9.565	9.832
ORAI3	10.165	10.171	10.275	10.024	10.034	9.994
ORAI1	10.463	10.607	10.640	10.614	10.686	10.542
AL118516.1	6.877	6.892	7.142	6.964	7.183	6.972
DLAT	9.190	9.239	9.278	9.119	9.042	9.144
C12orf29	8.961	8.962	9.149	8.961	9.109	9.010
FABP6	6.635	6.470	6.522	6.515	6.421	6.425
FABP7	6.831	7.021	6.681	7.097	7.252	6.903
FABP4	6.378	6.173	6.425	6.519	6.367	6.630
PGAM5	8.475	8.511	8.619	8.310	8.379	8.442
PGAM2	5.946	6.107	5.978	6.278	6.164	6.238
FABP3	6.499	6.614	6.629	6.615	6.700	6.706
FANCD2	8.232	8.256	8.461	7.468	7.513	7.587
PGAM1	12.759	12.888	13.016	12.823	12.758	12.866
AKR1C4	5.380	5.316	5.198	5.428	5.392	5.317
GABRA4	7.617	7.618	7.452	7.659	7.638	7.618
GABRA6	5.039	5.078	4.924	5.354	5.335	5.234
GABRA1	6.717	6.712	6.470	6.796	6.796	6.798
AKR1C1	10.278	10.242	10.387	10.367	10.240	10.318
AKR1C2	10.278	10.242	10.387	10.367	10.240	10.318
AKR1C3	10.840	10.928	11.016	10.958	10.899	10.880
IGLV3-10	7.412	7.631	7.491	7.685	7.526	7.730
IGLV3-19	7.703	7.742	7.933	7.953	7.765	7.890

SEC61B	10.612	10.690	10.872	10.739	10.632	10.668
RRP8	8.641	8.745	8.732	8.855	8.648	8.774
RRP9	9.217	9.312	9.377	9.161	9.250	9.010
DOCK1	8.671	8.632	8.717	8.449	8.571	8.637
AC104461.1	5.223	5.540	5.579	5.683	5.512	5.446
ZNF75D	7.319	7.440	7.551	7.274	7.288	7.247
GRIA4	6.117	6.127	6.024	5.919	6.318	6.247
KANSL1L-AS1	4.099	4.130	4.218	4.205	4.164	4.086
EP300	8.964	9.127	8.908	8.415	8.831	8.819
NPY5R	7.204	7.428	7.202	7.212	7.319	7.503
AC019270.1	4.449	4.724	4.458	5.004	4.886	4.966
NEU4	8.304	8.233	8.389	8.714	8.458	8.562
NEU1	10.699	10.881	10.961	10.999	10.986	10.868
NEU2	6.961	6.888	6.893	6.911	6.870	6.759
NEU3	8.316	8.428	8.178	8.130	8.228	8.243
MIR6738	6.954	6.896	7.125	6.725	6.712	6.870
MIR6734	10.665	10.777	10.942	10.659	10.667	10.711
OPRL1	7.544	7.646	7.494	7.735	7.719	7.671
MED10	9.237	9.203	9.297	9.024	9.211	9.105
MED11	9.282	9.228	9.416	9.036	9.156	9.287
MED12	8.688	8.708	8.589	8.585	8.613	8.604
MED13	10.267	10.156	10.104	9.745	10.034	10.176
MED14	9.328	9.291	9.325	9.176	9.055	9.100
MED15	10.106	10.182	10.308	10.155	10.170	10.107
MED16	8.169	8.260	8.332	7.998	8.135	8.099
MED17	8.730	8.877	8.887	8.573	8.523	8.675
MED18	8.431	8.305	8.596	8.305	8.209	8.331
MED19	10.357	10.243	10.396	10.265	10.483	9.984
AL136531.1	5.531	5.518	5.449	5.449	5.411	5.477
PBX2P1	7.476	7.371	7.688	7.381	7.426	7.312
MORF4L1P1	12.512	12.601	12.691	12.661	12.504	12.598
AC241952.2	7.467	7.590	7.618	7.576	7.832	7.792
AC104667.2	6.885	6.988	7.029	6.838	6.871	6.977
EEF1B2	12.241	12.317	12.505	12.366	12.368	12.394
TLX1	6.804	6.890	6.758	6.869	6.928	6.837
TLX2	6.484	6.602	6.483	6.602	6.705	6.711
TLX3	7.294	7.091	7.250	7.318	7.235	7.207
LIM2	7.298	7.118	7.397	7.283	7.342	7.356
ADH1C	5.489	5.625	5.113	5.452	5.340	5.572
ADH1B	5.623	5.347	5.500	5.685	5.778	5.678
ADH1A	6.975	6.576	6.475	6.941	6.791	6.797
GOLM1	11.959	12.030	12.074	11.674	11.624	11.684
MUSK	6.274	6.450	6.505	6.521	6.441	6.343
KIAA1257	7.399	7.412	7.257	7.503	7.581	7.477
MLEC	12.420	12.240	12.391	12.074	12.390	11.993
SLC30A9	9.272	9.365	9.361	8.903	8.861	9.033
SLC30A8	7.073	7.102	7.148	7.037	7.378	7.599
SLC30A5	9.210	9.252	9.277	8.946	8.846	9.092
SLC30A4	8.318	8.171	8.240	8.084	8.041	8.164
SLC30A7	9.617	9.633	9.574	9.587	9.306	9.454
SLC30A6	9.457	9.499	9.522	9.263	9.163	9.348
SLC30A1	10.363	10.387	10.411	10.509	10.409	10.553
SLC30A3	8.711	8.908	8.687	8.757	8.816	8.820
SLC30A2	7.286	7.479	7.523	8.009	7.680	7.740
EIF5AL1	12.743	12.696	12.917	12.633	12.437	12.525
MT1HL1	10.143	10.158	10.409	10.358	10.512	10.349

EML2	9.200	9.236	9.320	9.492	9.339	9.274
EML3	8.934	8.978	9.202	8.882	8.885	8.885
EML1	7.655	7.949	7.976	7.519	7.555	7.545
EML6	6.660	6.657	6.564	6.746	6.728	6.796
EML4	11.160	11.082	11.286	10.944	10.830	10.947
EML5	5.325	5.230	5.005	4.998	5.107	5.096
PLEKHB2	12.077	12.105	12.149	11.989	11.871	11.973
HLA-DPB1	7.403	7.337	7.500	7.154	7.497	7.470
HLA-DPB2	5.166	4.998	4.977	5.248	5.058	5.189
AC136352.12	7.424	7.406	7.675	7.294	7.409	7.495
KAAG1	6.495	6.403	6.246	6.290	6.361	6.522
AL132777.1	9.349	9.364	9.419	9.154	9.240	9.251
RSPO3	6.159	6.257	6.227	6.377	6.319	6.206
RSPO2	4.148	4.268	4.329	4.279	4.336	4.459
RSPO1	6.796	6.915	6.995	7.013	7.152	7.230
CNN1	5.832	5.726	5.629	6.051	6.042	6.124
CNN3	10.132	10.172	10.357	10.142	10.041	10.278
CNN2	10.398	10.488	10.650	10.378	10.312	10.421
CHAC2	8.212	8.227	8.214	8.195	8.285	8.370
CHAC1	8.794	8.708	8.747	8.525	8.753	8.452
FCMR	8.135	8.147	8.246	8.376	8.209	8.389
AC017104.1	8.725	8.798	8.738	8.900	8.827	8.924
AC105749.1	9.125	9.145	8.927	9.097	9.161	9.159
EIF5B	10.765	10.434	10.542	10.419	10.525	10.354
EIF5A	12.743	12.696	12.917	12.633	12.437	12.525
CDC37P1	7.837	7.915	7.931	7.776	7.875	7.821
CDC37P2	7.837	7.915	7.931	7.776	7.875	7.821
TUBB6	9.699	9.680	9.830	9.348	9.411	9.337
TUBB3	11.493	11.695	11.838	11.596	11.479	11.392
TUBB1	7.181	7.039	7.333	7.388	7.173	7.308
APTR	7.065	7.120	7.248	7.237	6.946	7.179
C19orf73	7.941	7.989	8.034	8.238	7.952	8.227
APTX	9.449	9.514	9.566	9.439	9.516	9.429
AC008878.1	8.375	8.476	8.634	8.423	8.607	8.534
SH3BGRL3	11.042	11.142	11.295	11.009	11.084	10.939
SH3BGRL2	9.783	9.732	9.910	9.651	9.515	9.687
MPRIPP1	7.867	7.950	7.953	7.794	7.781	7.862
ODF3B	8.564	8.542	8.775	8.112	8.380	8.298
FNDC1	5.248	5.246	5.198	5.196	5.331	5.458
OLFM3	4.673	4.612	4.457	4.549	4.704	4.661
OLFM2	7.272	7.481	7.462	7.370	7.639	7.730
OLFM1	8.721	8.660	8.611	8.720	8.886	8.818
OLFM4	7.875	7.884	8.022	7.487	7.719	7.628
STK39	10.990	11.081	11.032	10.669	10.696	10.769
RAF1	9.513	9.558	9.681	9.486	9.584	9.469
DRAM1	9.630	9.622	9.754	9.391	9.367	9.439
DRAM2	9.435	9.402	9.469	9.132	9.319	9.379
KLF17P1	6.289	6.432	6.367	6.658	6.540	6.246
RPL13A	14.424	14.395	14.422	14.370	14.350	14.418
AC136352.4	3.830	4.000	4.066	3.900	3.760	4.082
BCL7C	10.093	10.132	10.300	10.031	10.022	10.034
BCL7B	9.278	9.291	9.459	9.262	9.266	9.225
BCL7A	9.687	9.777	9.899	9.846	9.684	9.687
GDAP1L1	7.502	7.691	7.709	7.857	7.858	7.828
EFNA5	10.227	10.044	10.180	9.944	9.964	10.057
EFNA4	7.605	7.728	7.818	7.704	7.690	7.781

EFNA3	8.240	8.242	8.488	8.334	8.455	8.444
EFNA2	7.113	7.241	7.278	7.219	7.383	7.218
EFNA1	9.500	9.624	9.792	9.637	9.653	9.695
AC114291.1	3.826	3.863	3.882	3.931	3.831	3.841
MBNL1-AS1	8.674	8.715	8.794	8.665	8.696	8.632
AL583722.1	5.137	5.132	5.410	4.762	5.374	5.745
CLCC1	8.191	8.366	8.460	7.923	8.181	8.136
A2M-AS1	5.287	5.276	5.075	5.366	5.155	5.232
UCN2	7.138	6.831	7.195	7.106	7.172	7.206
UCN3	7.475	7.453	7.455	7.463	7.523	7.540
RBMY2CP	5.549	5.417	5.468	5.650	5.551	5.647
APEX1	12.172	12.231	12.261	12.139	11.939	12.018
APEX2	10.094	10.184	10.349	10.086	10.154	10.113
SPOCD1	6.926	6.661	6.922	6.829	6.764	6.889
KMT2C	8.810	8.772	8.854	8.721	8.981	8.631
KMT2B	9.534	9.557	9.516	9.483	9.495	9.649
ESRRG	5.921	6.282	6.056	6.086	6.052	6.019
ESRRA	9.534	9.735	9.816	9.729	9.734	9.654
KMT2E	8.588	8.554	8.507	8.444	8.575	8.441
KMT2D	8.761	8.874	8.958	9.023	8.978	8.843
CLEC11A	7.826	7.822	8.012	7.678	8.015	7.859
ZNF718	7.617	7.478	7.299	7.572	7.616	7.626
FBXL21P	6.742	6.835	6.733	7.143	6.954	6.917
ZNF717	7.926	7.853	8.034	7.865	7.938	7.860
ZNF710	9.057	9.082	8.979	8.868	8.846	8.886
ZNF711	7.284	7.199	7.575	6.815	6.901	7.012
ZNF713	4.524	4.335	4.429	4.263	4.378	4.275
NFAM1	8.123	8.202	8.094	8.355	8.311	8.139
ASB10	9.219	9.391	9.398	9.330	9.391	9.468
ASB11	4.993	5.412	5.285	5.391	5.281	5.340
ASB12	9.524	9.304	8.839	9.831	9.954	9.714
ASB13	8.145	8.234	7.984	8.207	8.235	8.140
ASB14	7.079	7.071	6.931	7.247	7.118	7.129
ASB15	6.957	6.421	6.549	6.636	6.670	6.891
ASB16	9.562	9.441	9.325	9.426	9.482	9.581
ASB17	4.820	4.897	4.736	5.113	5.011	5.235
RPP38	9.313	9.292	9.496	9.281	9.337	9.252
NSMF	9.231	9.211	9.262	9.321	9.141	9.249
XCL2	6.306	6.550	6.322	6.493	6.483	6.471
XCL1	6.306	6.550	6.322	6.493	6.483	6.471
ALDH1L1-AS2	7.124	7.274	7.181	7.009	7.110	7.129
NNT	8.536	8.742	8.593	8.569	8.384	8.409
AF305872.2	8.325	8.419	8.266	8.338	8.528	8.484
LRRC4C	7.430	7.317	7.335	7.590	7.721	7.547
CT66	5.149	5.120	5.126	5.050	5.327	5.125
CT62	4.531	4.531	4.793	5.003	4.626	4.603
CT69	5.914	5.988	5.965	6.036	6.160	6.166
AC048338.2	11.543	11.711	11.798	11.634	11.467	11.613
FDPS	11.811	11.903	12.039	11.568	11.568	11.654
AC048338.1	11.543	11.711	11.798	11.634	11.467	11.613
AC068946.1	9.564	9.400	9.593	9.330	9.449	9.279
AC068946.2	9.184	9.189	9.367	9.097	9.202	9.341
TFPI2	12.059	12.054	12.057	11.623	11.669	11.592
BATF	7.946	8.050	7.748	8.090	8.207	8.039
MACF1	12.380	12.396	12.350	12.113	12.065	12.186
CCDC144CP	4.940	4.732	4.722	4.720	4.691	4.712

AC010733.2	8.209	8.185	8.157	8.417	8.318	8.536
HEXIM1	10.322	10.209	10.283	10.297	10.262	10.308
DENND5B	7.778	7.555	7.551	7.225	7.418	7.374
DENND5A	9.527	9.530	9.714	9.594	9.537	9.648
HEXIM2	8.528	8.466	8.744	8.246	8.394	8.141
AL360093.1	5.885	5.952	6.440	6.319	6.172	6.190
NPTN-IT1	7.444	7.514	7.400	7.345	7.403	7.440
AL121992.3	4.630	4.653	4.637	4.608	4.587	4.641
AQP7P3	8.108	8.089	8.007	7.989	8.099	8.301
AQP7P1	8.108	8.089	8.007	7.989	8.099	8.301
PPIAP31	14.423	14.465	14.415	14.484	14.467	14.475
AQP7P5	8.108	8.089	8.007	7.989	8.099	8.301
AQP7P4	8.108	8.089	8.007	7.989	8.099	8.301
AP000350.1	7.447	7.617	7.613	7.242	7.700	7.578
AP000350.4	13.777	13.817	13.967	13.853	13.826	13.849
CRLS1	8.893	8.928	8.956	8.819	8.652	8.752
GCSH	11.692	11.837	11.918	11.632	11.625	11.668
ARMC8	9.400	9.526	9.563	9.476	9.422	9.557
AL512427.1	3.894	3.882	3.900	3.798	3.857	4.046
ARMC4	7.935	7.867	7.791	8.102	7.991	8.049
GUF1	7.377	7.447	7.589	7.117	7.081	7.223
PML	9.725	9.790	9.866	9.636	9.547	9.608
TIGD1	7.340	7.584	7.666	7.392	7.411	7.394
MRM2	10.583	10.681	10.716	10.263	10.296	10.281
MRM3	8.686	8.801	8.829	8.665	8.747	8.614
MRM1	8.692	8.963	9.068	8.866	8.920	8.860
MLST8	9.905	10.097	10.372	9.896	9.938	9.873
STAU1	12.404	12.466	12.440	12.323	12.283	12.335
CEP152	7.757	7.888	7.954	7.577	7.371	7.686
DICER1	9.759	9.825	9.840	9.460	9.516	9.329
TIGD3	7.177	7.313	7.212	7.377	7.305	7.307
HP1BP3	10.961	10.936	11.093	10.599	10.899	10.489
CHGA	6.476	6.629	6.512	7.023	6.726	6.702
CHGB	5.948	6.107	6.225	6.033	5.968	6.141
MSN	11.044	11.108	11.221	11.044	11.016	11.033
FOPNL	10.186	10.193	10.267	10.043	10.019	10.190
MSC	5.475	5.566	5.585	5.984	5.789	5.745
AQR	8.866	8.939	8.828	8.690	8.646	8.716
MPPE1	9.827	9.758	9.852	9.794	9.733	9.667
ACTL6A	10.925	11.105	10.951	10.989	10.950	11.080
COL8A2	7.641	7.630	7.576	7.583	7.584	7.642
COL8A1	4.991	5.073	5.523	5.306	5.136	5.133
CFLAR-AS1	6.154	6.372	6.198	6.261	6.346	6.501
DUX4L51	7.397	7.410	7.339	7.495	7.594	7.407
SIGLEC8	6.866	6.739	6.869	7.082	6.877	6.913
SIGLEC9	6.900	6.969	6.847	7.030	7.025	7.192
TCP11L1	8.371	8.239	8.303	8.290	8.212	8.234
SIGLEC5	7.649	7.739	7.938	7.604	7.621	7.653
SIGLEC6	8.003	8.073	7.957	8.000	8.221	8.251
SIGLEC7	7.845	7.825	7.921	8.019	7.989	8.048
SIGLEC1	7.467	7.599	7.625	7.361	7.582	7.585
FOLR2	8.453	8.233	8.399	8.614	8.448	8.448
AL671762.1	12.492	12.592	12.692	12.633	12.502	12.672
RPL7AP50	12.854	12.889	13.013	12.967	12.862	12.928
NCBP1	7.830	8.125	8.080	7.893	7.722	7.893
NCBP2	9.932	10.026	9.994	9.809	9.747	9.778

NCBP3	8.185	8.426	8.375	8.320	8.253	8.287
PROCA1	7.604	7.677	7.787	7.940	8.025	7.813
PKP4-AS1	7.314	7.338	7.355	7.720	7.502	7.612
INKA2	8.409	8.480	8.459	8.494	8.545	8.498
CNTROB	8.455	8.633	8.562	8.746	8.597	8.560
INKA1	8.092	8.325	8.310	8.287	8.198	8.226
DBR1	8.833	9.050	9.006	8.672	8.822	8.863
DRAP1	9.355	9.414	9.483	9.235	9.321	9.228
MEI1	8.782	8.875	8.859	8.695	8.731	8.876
CBLC	8.753	8.835	9.062	8.639	8.761	8.682
CBLB	8.083	7.995	8.167	7.682	8.006	7.686
ZNF45	8.365	8.429	8.430	8.213	8.031	8.092
ZNF44	7.623	7.555	7.613	7.263	7.563	7.361
ZNF43	5.769	5.482	5.614	5.602	5.781	5.633
ZNF41	5.554	5.877	5.684	5.492	5.684	5.572
ZNF48	8.857	8.968	9.000	8.806	8.846	8.872
AL445647.1	4.035	4.056	4.261	4.346	4.136	4.097
AC074387.1	4.748	4.941	4.924	4.823	4.858	5.010
PNISR	9.297	8.951	9.103	9.050	9.066	9.039
AC073508.3	8.362	8.240	8.398	8.034	8.062	8.100
AC073508.2	12.485	12.494	12.596	12.412	12.412	12.601
CYP2U1	8.541	8.708	8.694	8.390	8.712	8.714
AC139494.8	4.546	4.452	4.404	4.410	4.521	4.445
LILRB5	5.937	5.862	5.862	6.132	6.158	5.807
LILRB4	6.627	6.861	6.861	7.229	6.734	6.779
LILRB3	8.597	8.738	8.545	8.761	8.665	8.810
LILRB2	7.246	7.378	7.427	7.478	7.395	7.243
LILRB1	7.969	8.094	8.052	8.075	8.116	8.111
AC139494.2	7.820	7.988	7.762	7.789	7.771	7.856
AC139494.3	5.889	5.758	5.891	5.857	5.845	5.843
AC139494.6	4.781	4.830	4.828	4.736	4.646	4.712
AC139494.7	6.930	7.083	6.771	6.933	6.931	6.997
TAS2R14	6.430	6.467	6.141	6.382	6.365	6.479
KSR1	7.937	7.786	7.793	7.717	7.829	7.762
KSR2	7.163	7.230	6.922	6.982	7.172	7.028
AC093908.1	5.773	5.850	5.871	5.976	5.900	6.041
PPIH	9.253	9.326	9.443	9.410	9.357	9.253
PYM1	9.487	9.498	9.575	9.450	9.519	9.482
PPTC7	10.666	10.941	10.993	10.984	10.860	10.473
SSMEM1	5.556	5.476	5.284	5.645	5.540	5.554
AL139241.1	7.777	7.949	7.845	7.790	7.975	8.063
RN7SL587P	6.876	6.738	6.894	7.065	7.151	7.124
LIN37	7.992	8.039	8.260	8.192	8.034	7.920
LINC01692	3.641	3.643	3.581	3.702	3.578	3.561
LINC01697	6.695	6.854	6.811	6.904	6.880	6.952
LINC01694	4.866	4.721	4.726	4.672	4.551	4.698
DNALI1	7.326	7.318	7.576	6.913	7.373	7.338
AC004941.2	9.830	9.934	10.032	9.839	10.124	9.644
AP3D1	11.103	11.266	11.341	11.175	11.071	11.180
SULT1A4	9.509	9.493	9.572	9.371	9.427	9.609
SULT1A1	9.509	9.493	9.572	9.360	9.427	9.609
SULT1A3	9.509	9.493	9.572	9.371	9.427	9.609
SULT1A2	9.509	9.493	9.572	9.360	9.427	9.609
NOL9	9.209	9.093	9.161	9.122	9.088	9.106
NOL8	8.539	8.470	8.501	8.420	8.198	8.403
CHTF18	8.492	8.457	8.670	8.289	8.122	8.230

NOL4	5.822	5.983	6.035	5.924	6.180	6.257
NOL7	10.342	10.477	10.459	10.199	10.302	10.130
NOL6	9.211	9.157	9.091	9.257	9.221	9.102
OLIG2	6.460	6.386	6.456	6.637	6.583	6.543
OLIG3	4.897	5.219	5.047	5.046	5.150	5.328
NOL3	8.898	8.979	9.041	8.658	8.646	8.803
OLIG1	4.246	4.249	4.190	4.253	4.285	4.385
FAM156B	9.680	9.692	9.700	9.587	9.568	9.633
FAM156A	9.680	9.692	9.700	9.587	9.568	9.633
CLPTM1	10.924	10.931	11.120	10.918	10.853	10.858
CDC45	9.106	9.250	9.411	9.108	8.854	9.001
CDC40	9.344	9.327	9.491	9.084	9.008	9.144
CDC42	11.899	11.866	12.365	12.046	12.003	11.565
AC174065.1	7.763	8.056	8.072	7.971	8.074	8.073
LINC00690	4.678	4.488	4.671	5.104	4.755	4.722
SRD5A3-AS1	4.298	4.543	4.443	4.426	4.644	4.520
DPYD	9.117	9.174	9.149	8.881	9.042	9.171
DPYS	5.751	5.760	5.905	6.097	5.663	5.829
YLPM1	9.773	9.877	9.910	9.416	9.500	9.568
GFAP	7.286	7.288	7.435	7.610	7.553	7.380
STEAP1B	8.662	8.764	8.772	8.748	8.722	8.886
UBE2V2P3	7.345	7.403	7.272	7.587	7.491	7.481
LINC00698	7.191	7.094	7.223	7.242	7.437	7.379
SEM1	9.955	10.038	10.140	9.985	9.841	9.945
CT47A7	5.635	5.693	5.320	5.666	5.815	5.536
CT47A6	5.635	5.693	5.320	5.666	5.815	5.536
CT47A5	5.635	5.693	5.320	5.666	5.815	5.536
CT47A4	5.635	5.693	5.320	5.666	5.815	5.536
CT47A3	5.635	5.693	5.320	5.666	5.815	5.536
CT47A2	5.635	5.693	5.320	5.666	5.815	5.536
CT47A1	5.635	5.693	5.320	5.666	5.815	5.536
CT47A9	5.635	5.693	5.320	5.666	5.815	5.536
CT47A8	5.635	5.693	5.320	5.666	5.815	5.536
AC073612.1	7.515	7.662	7.563	7.429	7.421	7.637
GC	5.172	5.092	5.288	5.389	5.242	5.288
DMRTC1B	8.957	9.043	8.933	8.974	9.004	9.041
MED14OS	6.934	6.951	7.191	7.373	7.126	7.034
BRK1	10.968	10.946	11.088	10.885	10.929	10.929
TENT4B	9.984	10.032	10.179	10.286	10.380	9.756
PRL	6.557	6.384	6.638	6.498	6.698	6.758
H2AZ2P1	11.481	11.573	11.753	11.357	11.261	11.283
PRY	7.337	7.057	7.188	7.293	7.443	7.388
PRX	8.361	8.393	8.431	8.489	8.438	8.539
TYRP1	4.506	4.313	4.465	4.509	4.421	4.534
ESX1	6.413	6.637	6.429	6.617	6.582	6.513
HSD3B7	9.611	9.761	9.797	9.458	9.540	9.504
HSD3B1	5.976	6.372	6.052	6.273	6.422	6.319
HSD3B2	6.415	6.629	6.372	6.624	6.485	6.416
CD300C	6.917	6.875	6.830	7.023	6.917	6.780
CD300A	7.169	7.347	7.305	7.304	7.368	7.335
ERVMER34-1	6.627	6.574	6.677	6.593	6.644	6.568
HAAO	8.304	8.347	8.504	8.375	8.252	8.415
RCCD1	10.318	10.339	10.459	10.033	10.074	10.064
LBHD1	9.372	9.336	9.498	9.398	9.379	9.287
TBR1	6.872	6.924	6.848	6.874	6.929	6.934
AC006600.1	7.769	7.920	7.867	7.471	7.518	7.690

DDC-AS1	5.962	5.965	5.970	5.769	6.148	6.198
PLCH2	7.396	7.547	7.379	7.689	7.536	7.444
ERFE	7.695	7.872	7.794	7.720	7.766	7.947
PLCH1	7.930	7.985	7.932	7.833	7.731	7.877
VEZF1	9.238	9.214	9.214	8.893	9.066	9.184
AL359513.1	7.365	7.293	7.307	7.229	7.361	7.327
NUDT9	9.729	9.652	9.685	9.450	9.389	9.488
DLD	9.352	9.411	9.458	9.195	9.219	9.292
NUDT1	8.736	8.806	8.911	8.484	8.576	8.588
NUDT2	8.556	8.407	8.766	8.374	8.493	8.541
NUDT3	10.040	10.103	10.245	9.913	9.895	10.065
NUDT4	9.971	10.062	10.046	9.918	9.987	10.114
NUDT5	9.939	9.954	10.156	9.991	10.071	9.980
NUDT6	7.109	6.920	7.061	6.921	6.895	6.818
NUDT7	7.814	7.919	7.927	6.824	7.344	7.336
AC073342.2	4.787	4.662	4.753	4.831	4.957	4.822
COL16A1	6.208	6.300	6.524	6.657	6.359	6.491
AC012640.4	7.875	7.679	7.720	7.934	7.653	7.839
AL021328.1	3.879	4.042	4.123	4.434	4.174	4.139
AL353719.1	8.041	8.120	7.942	7.859	8.113	8.084
OPN1MW3	6.498	6.624	6.478	6.739	6.566	6.784
OPN1MW2	6.498	6.624	6.478	6.739	6.566	6.784
MEF2C	8.288	8.361	8.486	8.187	8.446	8.062
MEF2B	6.556	6.433	6.864	6.811	7.042	6.828
MEF2A	9.406	9.390	9.511	9.231	9.248	9.238
FETUB	4.793	4.829	4.819	5.174	4.978	5.115
MEF2D	8.409	8.289	8.390	8.369	8.409	8.348
LCORL	8.053	7.820	7.900	7.489	7.699	7.523
EPHX1	9.609	9.613	9.799	9.846	9.833	9.695
EPHX2	6.183	6.175	6.155	5.970	6.208	6.108
EPHX3	6.493	6.645	6.263	6.818	6.712	6.847
EPHX4	7.805	8.142	8.240	7.676	7.803	7.748
RPLP2	13.643	13.679	13.776	13.702	13.587	13.726
RPLP1	14.279	14.320	14.363	14.330	14.333	14.368
RPLP0	14.563	14.551	14.570	14.502	14.525	14.557
CSMD2	7.256	7.325	7.212	7.495	7.367	7.366
CSMD3	4.492	4.860	4.681	4.987	4.964	5.273
CSMD1	5.894	5.635	5.801	5.967	5.853	5.760
CD300LB	9.026	9.065	9.057	8.936	9.142	9.215
CFAP20	11.008	11.093	11.227	10.952	10.917	11.007
EMC3-AS1	7.749	7.718	7.654	7.682	7.673	7.675
TRIL	7.013	7.081	7.056	7.072	7.207	7.175
TRIO	9.370	9.415	9.428	9.444	9.505	9.582
AC115618.3	8.252	8.462	8.627	8.040	8.203	7.956
RABL2A	7.814	7.755	7.824	7.363	7.375	7.426
RABL2B	7.814	7.755	7.824	7.363	7.375	7.426
TRIR	11.372	11.337	11.635	11.350	11.208	11.381
TCP1	11.988	12.017	12.108	12.066	11.916	12.005
KTN1	11.827	11.733	11.768	11.724	11.739	11.750
APOA4	7.523	7.496	7.576	7.743	7.627	7.558
EP400P1	7.275	7.402	7.473	7.458	7.491	7.261
FAM90A1	6.748	6.759	6.790	6.871	6.680	7.047
VPREB1	4.792	4.828	4.854	4.581	5.060	4.449
VPREB3	7.518	7.246	7.375	7.716	7.384	7.470
NINJ1	9.612	9.556	9.651	9.477	9.423	9.482
NINJ2	6.269	6.405	6.280	6.433	6.664	6.404

XPO4	8.558	8.664	8.580	8.569	8.572	8.683
SLC16A9	6.366	6.325	6.280	6.357	6.296	6.287
SLC16A8	7.256	7.292	7.322	7.540	7.467	7.648
SLC16A1	10.235	10.261	10.165	10.128	10.091	10.121
SLC16A3	10.507	10.591	10.749	10.440	10.522	10.625
SLC16A2	7.072	7.292	7.200	7.164	7.303	7.218
SLC16A5	9.808	9.795	9.911	9.233	9.300	9.407
SLC16A4	3.815	3.776	3.670	3.915	4.000	3.701
SLC16A7	10.658	10.506	10.806	10.399	10.349	9.898
SLC16A6	6.229	6.298	6.131	6.096	6.316	6.194
ARHGAP30	7.786	7.671	7.997	8.150	8.046	7.873
ARHGAP31	6.303	6.018	6.092	6.325	6.552	6.406
ARHGAP32	9.379	9.340	9.402	9.027	9.327	8.977
ARHGAP33	8.688	8.774	8.571	8.394	8.386	8.476
ARHGAP35	10.923	10.866	10.935	10.513	10.473	10.590
ARHGAP36	4.782	4.270	4.418	4.865	4.623	4.658
SMIM10L2A	7.315	7.271	7.314	6.882	7.166	6.831
SMIM10L2B	7.315	7.271	7.314	6.882	7.166	6.831
CFAP206	7.737	7.730	7.812	7.918	7.775	7.972
LYVE1	7.238	7.142	7.020	7.254	7.250	7.402
HECTD1	10.716	10.746	10.706	10.588	10.305	10.573
CACNA1H	7.828	7.967	7.903	7.860	7.921	8.013
CACNA1I	7.612	7.606	7.601	7.615	7.693	7.633
CACNA1A	7.909	7.920	8.164	8.268	8.205	8.414
CACNA1B	6.192	6.179	6.436	6.571	6.319	6.358
CACNA1C	6.853	6.895	7.060	7.111	7.074	7.260
CACNA1D	7.880	7.909	7.961	7.789	8.039	7.762
CACNA1E	7.024	7.236	7.229	7.244	7.390	7.253
CACNA1F	7.599	7.677	7.643	7.913	7.725	7.847
CACNA1G	8.934	8.859	8.859	8.917	8.856	8.911
TMEM262	6.962	7.161	7.244	7.403	7.274	7.233
CACNA1S	7.166	7.283	7.282	7.382	7.230	7.226
TTN	6.005	6.032	6.064	6.052	6.244	6.148
BMP8A	8.038	8.238	8.158	8.169	8.220	8.222
BMP8B	8.019	7.891	8.030	7.856	7.701	7.680
HOTAIR	3.821	3.744	3.831	3.940	3.733	3.992
AL049828.1	7.545	7.524	7.658	7.380	7.736	7.745
BGLAP	7.433	7.630	7.597	7.392	7.658	7.514
SLC15A2	7.676	7.449	7.669	7.564	7.573	7.632
SLC15A3	7.502	7.564	7.571	7.471	7.597	7.410
SLC15A1	6.516	6.457	6.418	6.099	6.448	6.456
SLC15A4	9.797	9.741	9.877	9.436	9.534	9.324
AC270301.7	4.976	5.133	4.745	5.206	5.081	4.914
AC270301.6	6.515	6.864	6.727	6.754	6.782	6.662
AC270301.4	4.676	4.753	4.777	4.942	4.862	4.776
AC270301.2	5.872	6.010	6.138	6.408	6.349	6.010
CDY1B	7.045	7.164	6.836	7.174	7.246	7.298
PQBP1	10.679	10.763	10.971	10.598	10.472	10.643
AC270301.8	5.484	5.446	5.382	5.301	5.330	5.288
TUBB4BP7	8.228	8.389	8.254	8.121	8.422	8.262
AC009902.2	7.921	7.945	8.123	8.193	8.112	7.992
AC009902.3	4.988	5.081	4.797	4.927	5.206	4.956
AC007359.1	5.502	5.659	5.466	5.983	5.555	5.487
ZNF734P	6.759	6.711	6.644	6.939	6.943	6.850
SLBP	10.544	10.593	10.653	10.321	10.328	10.369
HIF1A	12.920	12.993	12.967	12.868	12.765	12.942

RNVU1-21	4.675	4.945	4.760	4.770	4.684	4.808
ABCF3	9.944	10.046	10.180	9.878	9.726	9.816
ABCF2	9.032	9.099	9.299	8.882	8.972	9.078
ABCF1	10.070	10.083	10.110	9.895	9.958	9.934
PJA1	11.067	11.239	11.311	11.055	11.005	11.097
PJA2	10.318	10.359	10.320	10.263	10.247	10.084
PAXX	8.203	8.233	8.340	8.381	8.287	8.173
NSMCE1	10.653	10.822	10.967	10.724	10.622	10.672
PRKAB2	8.360	8.373	8.363	8.129	8.354	8.172
PRKAB1	10.324	10.316	10.474	10.176	10.235	10.245
SIKE1	7.934	7.835	7.840	7.424	7.815	7.532
PAX5	8.020	7.991	8.017	8.049	8.031	8.007
PAX4	7.443	7.547	7.559	7.416	7.596	7.462
PAX7	6.950	7.005	7.161	7.178	7.284	7.299
PAX6	7.469	7.424	7.418	7.495	7.441	7.510
PAX1	7.425	7.557	7.405	7.696	7.323	7.502
AC026803.2	13.932	13.980	14.013	13.975	13.978	13.956
PAX3	6.534	6.580	6.679	6.719	6.674	6.605
PAX2	7.903	8.151	7.675	8.001	7.969	8.039
PTH2	8.615	8.559	8.563	8.581	8.528	8.633
PAX9	7.537	7.539	7.538	7.631	7.548	7.458
PAX8	10.974	11.024	11.084	10.920	11.118	11.012
DDR2	8.133	7.945	7.853	8.197	8.033	8.146
DDR1	12.509	12.637	12.713	12.611	12.600	12.610
LRCH4	9.172	9.068	9.080	9.387	9.378	9.178
LRCH1	8.246	8.332	8.348	8.144	8.155	8.267
LRCH2	3.956	4.206	4.229	4.042	4.292	4.286
LRCH3	8.617	8.357	8.389	8.164	7.999	8.470
MBD3L1	6.469	6.498	6.384	6.562	6.685	6.814
MBD3L2	6.806	6.936	7.361	6.869	6.923	7.036
MBD3L3	6.806	6.936	7.361	6.869	6.923	7.036
MBD3L4	6.806	6.936	7.361	6.869	6.923	7.036
MBD3L5	6.806	6.936	7.361	6.869	6.923	7.036
C15orf39	10.017	10.121	10.294	10.160	10.213	10.095
C15orf32	5.021	4.968	4.838	4.874	5.085	4.928
TEX26-AS1	5.970	5.759	5.529	5.810	6.075	5.845
TPPP2	6.425	6.528	6.125	6.688	6.520	6.505
AL162377.1	8.176	8.351	8.272	8.342	8.277	8.325
AL162377.3	7.904	7.925	7.946	7.811	7.887	7.800
DSG2	12.076	12.077	12.188	11.967	11.895	11.993
DSG3	7.568	7.519	7.535	7.647	7.487	7.696
ZNF107	7.697	7.577	7.635	7.727	7.662	7.806
DSG1	4.727	4.739	4.725	4.772	4.718	4.609
ZNF101	7.063	6.930	7.209	6.959	6.980	7.018
ZNF100	5.280	5.305	5.258	5.268	5.214	5.087
DSG4	4.516	4.724	4.727	4.588	4.580	4.659
AC096736.3	3.763	3.951	3.871	3.786	3.908	3.830
AC096736.2	4.363	4.394	4.630	4.689	4.533	4.517
AC005281.1	4.940	5.088	4.897	4.990	4.850	4.879
SH3BP5L	8.266	8.272	8.577	8.250	8.111	8.189
AL138787.1	7.065	7.101	7.058	6.977	7.174	7.154
DET1	8.872	8.843	8.869	8.772	8.868	8.753
PIR	9.042	9.161	9.326	9.100	9.150	9.195
NRBF2	8.165	8.259	8.417	8.292	8.224	8.344
KCNJ3	5.872	5.974	6.181	6.118	6.077	6.093
KCNJ2	6.196	6.116	5.874	6.195	6.247	6.302

KCNJ1	6.969	7.274	7.041	7.144	7.234	7.085
TRAFD1	9.517	9.562	9.704	9.418	9.390	9.441
KCNJ6	6.530	6.467	6.594	6.749	6.691	6.744
KCNJ5	8.344	8.279	8.197	8.541	8.529	8.604
KCNJ4	6.738	6.891	6.726	6.914	7.199	6.859
KCNJ9	7.147	7.083	7.233	7.242	7.512	7.246
KCNJ8	6.776	6.654	6.472	6.758	6.663	6.947
LRRC37A	7.316	7.496	7.592	7.563	7.519	7.583
LRRC37B	8.061	8.058	8.021	7.915	7.840	7.926
AC109635.3	7.822	7.843	7.822	8.011	7.971	7.975
OR2J1	5.816	5.865	5.916	5.849	5.753	5.945
OR2J2	5.816	5.865	5.916	5.849	5.753	5.945
OR2J3	5.097	5.243	4.983	5.187	4.942	5.109
CMYA5	8.154	8.529	8.471	8.482	8.580	8.473
PEX19	9.293	9.408	9.400	9.203	9.250	9.365
NIP7	9.569	9.768	9.803	9.599	9.883	9.454
PEX10	8.718	8.826	8.891	8.836	8.809	8.607
PEX13	8.551	8.617	8.746	8.507	8.381	8.524
PEX12	7.721	7.909	7.951	7.873	7.786	7.967
PEX14	8.583	8.749	8.798	8.514	8.700	8.467
PEX16	10.431	10.529	10.587	10.382	10.363	10.432
AL590867.2	13.836	13.822	13.906	13.931	13.893	13.957
TMEM192	10.416	10.573	10.632	10.298	10.277	10.126
ELK2AP	8.926	8.905	9.098	9.156	9.048	9.143
GIT2	9.722	9.619	9.681	9.405	9.565	9.550
AC007952.5	7.746	7.766	7.813	7.866	7.708	7.733
AC007952.4	7.100	7.324	7.207	7.239	7.288	7.394
AC007952.7	7.746	7.766	7.813	7.866	7.708	7.733
AC007952.1	7.648	7.611	7.631	7.628	7.663	7.739
AC007952.2	7.648	7.611	7.631	7.628	7.663	7.739
AC010435.1	9.962	10.069	10.006	9.979	9.864	10.116
FAM71D	6.321	6.512	6.004	6.347	6.525	6.196
FAM71B	7.279	7.392	7.217	7.399	7.206	7.464
FAM71C	6.571	6.724	6.524	7.002	6.873	6.969
FAM71A	8.098	7.871	7.986	8.338	8.154	8.262
OGG1	7.294	7.347	7.291	7.425	7.329	7.357
GPR21	8.128	8.350	8.157	8.286	8.322	8.200
GPR26	8.053	8.054	7.837	8.056	8.065	8.007
GHRL	7.230	7.011	7.207	7.423	7.369	7.434
GPR27	8.203	7.996	8.270	8.266	8.222	8.150
GPR25	5.045	4.944	5.136	5.568	5.165	4.969
AL021396.1	6.264	6.243	6.027	6.208	6.376	6.248
AC092919.3	6.154	6.324	6.200	5.990	5.905	5.805
Z83844.1	8.402	8.430	8.469	8.237	8.355	8.165
Z83844.3	9.407	9.516	9.600	9.091	9.282	9.250
Z83844.2	7.862	8.115	8.176	8.073	8.018	8.151
CALN1	7.033	6.958	6.711	6.980	7.088	6.985
CSTL1	5.692	5.721	5.631	5.683	5.634	5.903
ATP6V1G2-DDX3	12.229	12.254	12.337	12.254	12.175	12.212
TBRG1	7.792	7.642	7.792	7.663	7.568	7.565
TBRG4	10.047	10.209	10.385	9.974	9.761	10.103
PLEKHJ1	9.164	9.324	9.525	9.246	9.264	9.239
AL513318.2	6.568	6.694	6.779	7.147	6.816	6.730
AL390066.2	5.539	5.426	5.480	5.412	5.689	5.451
SNORA7A	13.951	13.948	14.015	14.017	14.031	14.037
AC102945.2	7.398	7.493	7.389	7.350	7.445	7.605

CRMP1	9.070	8.926	8.906	9.284	9.156	9.042
FN3K	6.464	6.847	6.674	6.613	6.930	6.852
MIR3936HG	7.203	7.391	7.192	7.073	7.193	7.449
RNF44	10.449	10.463	10.541	10.305	10.258	10.291
RNF43	7.691	7.636	7.821	7.790	7.850	7.663
RNF41	8.757	8.874	9.022	8.706	8.880	8.722
RNF40	10.422	10.606	10.607	10.265	10.423	10.479
DCUN1D1	10.286	10.282	10.623	10.529	10.576	10.143
DCUN1D2	6.928	7.005	7.000	6.959	6.974	7.182
DCUN1D3	9.427	9.459	9.352	9.400	9.436	9.486
DCUN1D4	8.173	8.049	8.135	8.029	8.088	8.090
DCUN1D5	9.866	9.945	9.988	9.904	9.811	9.934
SNORA78	8.530	8.516	8.536	8.482	8.518	8.624
SNORA75	12.726	12.752	12.816	12.765	12.622	12.699
C2orf92	6.334	6.348	6.510	6.290	6.470	6.318
SNORA72	4.014	4.012	4.128	4.054	4.183	4.001
SNORA70	13.794	13.873	13.940	13.883	13.836	13.884
DEPTOR	9.294	9.369	9.383	9.276	9.485	9.327
CES3	7.975	8.238	7.984	8.086	8.182	8.185
MTTP	5.113	5.458	5.191	5.170	5.352	5.394
AIFM2	8.400	8.556	8.586	8.369	8.142	8.306
AIFM3	7.805	7.703	7.734	7.790	7.819	7.867
IL17RE	7.367	7.463	8.044	7.698	7.825	7.614
IL17RD	6.474	6.622	6.101	6.120	6.264	6.602
IL17RC	9.118	9.143	9.333	9.181	9.191	9.186
IL17RB	9.383	9.308	9.398	9.094	9.172	9.130
IL17RA	8.966	8.893	8.958	8.896	8.858	8.853
KBTBD11	6.629	6.566	6.589	6.542	6.345	6.619
PTGES3L	5.791	5.967	5.648	5.746	5.754	5.577
FOXR1	5.537	5.680	5.729	5.888	5.785	5.659
FOXR2	4.415	4.440	4.356	4.975	4.409	4.374
HBA1	8.968	8.952	8.822	8.964	9.136	9.114
UCP1	6.417	6.418	6.227	6.418	6.427	6.498
MUC5B	9.449	9.332	9.082	8.642	8.714	8.807
NCOR2	11.291	11.365	11.519	11.284	11.164	11.225
NCOR1	8.836	8.919	8.911	8.577	8.662	8.619
SCAPER	8.511	8.446	8.462	8.110	8.352	8.317
HBA2	8.968	8.952	8.822	8.964	9.136	9.114
AVEN	7.145	7.152	7.567	7.182	7.137	7.245
TEC	7.071	7.253	7.380	7.218	7.078	7.250
REPS1	9.542	9.679	9.708	9.490	9.535	9.644
TEF	8.861	9.018	9.068	8.721	8.787	8.734
REPS2	8.204	8.135	8.197	7.991	8.087	7.973
NECTIN2	10.587	10.609	10.739	10.633	10.891	10.541
NECTIN3	10.073	10.159	10.231	10.009	10.018	10.130
NECTIN1	9.451	9.405	9.535	9.512	9.483	9.423
AC073957.3	7.929	8.008	7.904	7.746	7.941	7.876
FFAR4	5.445	5.534	5.753	5.533	5.563	5.734
NECTIN4	8.285	8.427	8.328	8.191	8.352	8.405
TES	10.918	10.962	11.033	10.758	10.934	10.846
MSS51	4.699	4.928	4.718	4.833	4.712	4.817
RAP2B	11.832	11.849	11.844	11.911	11.982	11.962
RAP2C	8.675	8.790	8.639	8.383	8.337	8.582
RAP2A	9.406	9.363	9.562	9.290	9.467	9.144
CORT	7.912	7.605	7.853	7.960	7.963	8.026
NUP210L	5.717	5.796	5.792	5.734	5.625	5.888

DAPP1	9.389	9.371	9.443	9.438	9.780	9.048
RPL18AP16	9.600	9.721	9.927	9.656	9.649	9.909
BX927138.1	7.344	7.403	7.456	7.273	7.478	7.510
OVOL3	7.174	7.503	7.247	7.490	7.419	7.449
OVOL2	7.893	8.127	8.068	7.995	7.891	7.947
OVOL1	8.997	9.113	9.118	8.991	9.107	9.043
LINC02065	6.817	6.763	6.387	7.005	6.534	6.894
LINC02064	7.809	7.938	8.008	7.967	8.060	8.051
M1AP	4.843	4.795	4.840	5.029	5.092	4.924
HADHB	10.941	11.049	11.105	10.989	10.884	10.894
HADHA	11.311	11.393	11.515	11.343	11.472	11.120
RPS19BP1	10.369	10.468	10.709	10.341	10.282	10.325
ZNF91	9.448	9.301	9.391	8.832	9.145	9.317
KBTBD6	8.010	8.270	8.065	7.730	7.601	7.963
ALS2	8.853	8.784	8.815	8.757	8.792	8.841
TRIM49C	5.312	5.102	5.249	5.279	5.179	5.099
LRRC52	7.877	7.933	7.734	7.909	7.953	8.065
LRRC56	7.431	7.429	7.567	7.615	7.588	7.642
RTF1	9.125	9.005	9.173	8.756	8.853	8.791
RTF2	10.273	10.423	10.447	10.167	10.029	10.121
LRRC55	5.574	5.518	5.530	5.877	5.631	5.936
LRRC58	9.644	9.715	9.541	9.325	9.204	9.507
LRRC59	11.931	12.014	12.024	11.907	11.756	11.949
AC109446.5	6.447	6.786	6.894	6.806	6.901	7.082
AC109446.3	6.447	6.786	6.894	6.806	6.901	7.082
LINC02688	7.576	7.631	7.770	7.710	7.591	7.588
AC026356.1	5.386	5.753	5.660	5.321	5.265	5.257
AP1M2	10.187	10.293	10.483	10.253	10.201	10.166
LINC02683	7.383	7.489	7.261	7.526	7.447	7.605
AP1M1	9.685	9.804	9.918	9.598	9.658	9.538
LINC02685	8.556	8.257	7.988	8.466	8.428	8.348
CCDC146	7.014	7.129	6.961	7.006	7.254	7.323
CCDC141	4.086	4.013	4.145	4.073	4.101	4.135
CCDC140	6.537	6.531	6.610	6.634	6.582	6.629
L1CAM	7.792	8.088	7.951	8.234	8.285	8.189
CCDC149	9.300	9.255	9.085	9.148	9.496	9.563
CCDC148	6.490	6.279	6.179	6.395	6.470	6.205
AC055720.2	4.635	4.477	4.290	4.581	4.712	4.649
AC055720.1	5.629	5.538	5.461	5.594	5.481	5.783
HNRNPA3P10	11.051	11.098	11.160	10.846	10.830	10.950
TKTL1	7.336	7.185	6.991	7.291	7.291	7.006
TKTL2	5.017	4.998	4.806	4.997	5.136	5.065
TAS2R15P	3.843	3.724	3.751	3.678	3.707	3.702
MT-ATP8	14.445	14.476	14.457	14.504	14.599	14.502
AC008753.2	7.282	7.149	7.230	7.410	7.454	7.037
TBX20	5.086	4.990	4.885	5.117	5.068	5.237
MT-ATP6	14.545	14.551	14.548	14.608	14.629	14.551
BDKRB1	7.025	6.908	6.669	6.940	6.915	7.028
TBX22	3.695	3.878	3.609	3.780	3.868	3.814
MTUS2-AS1	6.620	6.559	6.560	6.642	6.888	6.636
OSBPL8	11.593	11.663	11.778	11.496	11.625	11.292
OSBPL9	10.611	10.730	10.678	10.531	10.494	10.544
OSBPL2	10.468	10.469	10.557	10.202	10.145	10.230
OSBPL3	9.800	9.581	9.573	9.457	9.593	9.635
OSBPL6	6.227	6.152	6.215	6.093	6.194	6.260
OSBPL7	8.368	8.309	8.520	8.052	8.242	8.147

OSBPL5	8.154	7.968	8.127	8.176	8.159	8.242
LINC01082	5.150	5.231	5.136	5.556	5.291	5.504
LINC01087	4.174	4.019	4.427	4.329	4.094	4.181
LINC01085	4.130	3.957	3.926	4.142	4.040	4.094
TUBB4B	12.925	12.972	13.155	12.873	12.754	12.750
LINC01088	6.284	6.372	6.599	6.322	6.208	6.201
LINC01089	8.256	8.281	8.334	8.197	8.256	8.157
RPS7P1	13.359	13.392	13.529	13.540	13.451	13.548
GAS8-AS1	7.449	7.529	7.345	7.252	7.552	7.406
GPR137C	5.732	5.820	5.924	5.674	5.601	5.342
GPR137B	7.515	7.616	7.780	7.284	7.362	7.273
MIR1909	7.696	7.873	8.053	7.886	8.125	7.893
LINC00689	7.767	7.747	7.736	7.726	7.703	7.723
LINC00687	6.761	6.544	6.697	6.746	6.959	7.182
LINC00685	7.641	7.659	7.608	7.643	7.527	7.586
LINC00683	8.086	8.160	8.040	8.299	8.250	8.174
LINC00682	5.822	5.617	5.623	5.951	5.852	5.839
LINC00680	6.121	6.108	6.209	6.082	6.370	6.288
DCDC1	3.766	3.688	3.767	3.839	3.750	3.673
DCDC2	9.513	9.446	9.464	9.146	9.174	9.238
TDRP	7.044	6.950	6.881	7.107	7.028	7.069
MIR600	6.759	6.698	6.907	7.009	6.270	6.814
MIR601	6.843	7.122	6.986	6.996	6.808	6.967
AC090987.1	5.285	5.150	5.088	5.330	5.211	5.330
ERMP1	10.772	10.820	10.787	10.571	10.521	10.517
LINC00355	4.843	4.589	4.910	4.821	4.870	4.868
LINC00354	5.292	5.340	5.015	5.288	5.625	5.368
FAM153A	8.275	8.445	8.343	8.661	8.412	8.386
LINC00358	4.173	4.081	4.222	4.298	4.172	4.399
GUSBP11	8.896	8.620	8.785	8.528	8.209	8.176
AL117329.1	6.551	6.674	6.656	6.575	6.686	6.831
WRB-SH3BGR	7.311	7.468	7.381	7.540	7.568	7.613
AC270277.1	4.739	4.553	4.759	4.748	4.673	4.634
AC122718.1	9.535	9.496	9.496	9.402	9.565	9.304
AL445985.1	7.389	7.249	7.452	7.160	7.455	7.411
CCBE1	7.223	6.924	6.943	7.107	7.303	7.139
SS18	8.839	8.885	8.780	8.696	8.855	8.843
DANT2	5.395	5.276	5.228	5.350	5.308	5.446
AADACL2	4.533	4.411	4.454	4.662	4.616	4.735
DUX4L11	8.052	7.820	8.258	8.487	8.319	8.298
LMO1	6.440	6.741	6.486	6.674	6.693	6.651
LMO2	5.818	5.771	5.881	6.139	6.135	5.768
LMO3	6.478	6.481	6.251	6.655	6.377	6.510
LMO4	11.210	11.186	11.247	11.048	11.303	11.060
LMO7	10.187	9.960	9.981	9.764	10.038	9.712
C3orf49	5.982	6.043	6.116	5.897	6.143	5.851
ERBB2	14.381	14.406	14.441	14.365	14.235	14.363
ALG1L15P	8.642	8.787	8.948	8.382	8.713	8.503
ST8SIA6-AS1	7.174	7.226	6.970	7.399	7.132	7.264
LARS1	9.638	9.760	9.632	9.367	9.291	9.379
AFAP1	4.884	4.810	5.043	4.722	4.917	4.943
AC004687.2	9.944	9.998	10.089	10.078	10.054	10.060
SHC2	8.495	8.384	8.415	8.253	8.189	8.410
GJB1	11.187	11.283	11.325	11.086	10.936	11.082
GJB3	9.879	10.022	10.123	9.891	9.962	9.859
GJB2	6.596	6.697	6.624	6.219	6.537	6.271

GJB5	6.891	6.949	6.879	6.958	7.070	6.910
GJB6	4.438	4.716	4.643	4.618	4.814	4.790
ZNF75A	9.456	9.273	9.275	9.150	9.218	9.315
PSMC1	11.930	11.960	12.005	11.913	11.789	11.822
PSMC2	10.285	10.288	10.426	10.290	10.282	10.339
PSMC3	11.133	11.193	11.249	10.914	10.870	10.944
PSMC4	11.813	11.821	11.946	11.805	11.696	11.817
PSMC5	10.598	10.652	10.814	10.385	10.106	10.421
PSMC6	9.851	9.934	10.076	9.855	9.847	10.025
AL121581.1	7.820	7.922	7.873	7.953	7.884	8.007
CEP85	9.002	9.023	9.047	8.904	9.048	8.939
AL391056.1	9.140	9.125	9.133	9.111	9.225	9.146
TLR8-AS1	6.380	6.511	6.394	6.321	6.503	6.538
LTO1	7.336	7.410	7.540	7.368	7.449	7.461
PIP4P2	5.869	6.069	5.899	5.517	5.628	5.812
PIP4P1	10.220	10.242	10.380	10.097	10.174	10.271
FN3KRP	10.486	10.598	10.793	10.293	10.386	10.404
C9orf152	6.853	6.799	6.861	6.634	6.625	6.781
AL136419.1	9.786	9.800	9.901	9.508	9.462	9.477
AC133552.5	7.759	7.920	7.945	7.738	7.688	7.600
AC084121.4	6.451	6.418	6.257	6.244	6.283	6.307
AC133552.1	5.380	5.692	5.480	5.429	5.683	5.827
AL049775.1	5.066	4.942	5.158	5.122	5.441	4.971
AC133552.2	5.746	5.989	5.784	5.811	5.802	5.683
MT-CO1	14.677	14.690	14.674	14.688	14.718	14.710
MT-CO2	14.677	14.690	14.674	14.688	14.718	14.710
MT-CO3	14.545	14.551	14.548	14.608	14.629	14.551
CFTR-AS1	3.449	3.539	3.627	3.711	3.717	3.657
AC093157.1	7.237	7.102	7.208	7.320	7.497	7.467
FOXI1	5.624	5.529	5.551	5.556	5.490	5.550
TMEM177	8.937	9.108	9.257	8.771	8.701	8.778
TMEM174	8.194	8.147	8.000	8.078	8.243	8.149
TMEM175	8.369	8.235	8.338	8.382	8.358	8.210
CLSTN1	10.127	10.191	10.242	10.055	10.042	10.008
CLSTN2	5.980	6.222	6.114	6.217	6.560	6.291
CLSTN3	8.384	8.373	8.340	8.453	8.375	8.411
TMEM100	4.356	4.386	4.345	4.350	4.408	4.529
AC012508.1	5.784	5.790	5.595	5.784	5.961	6.032
LINC00276	4.142	4.218	4.162	4.416	4.060	4.279
ELSPBP1	6.080	6.371	6.094	6.138	6.153	6.192
CDKN2B	9.960	10.002	9.983	9.975	9.881	10.082
SMPDL3B	9.574	9.572	9.501	9.621	9.491	9.520
CDKN2A	12.506	12.502	12.569	12.424	12.420	12.382
CDKN2D	6.940	7.045	7.391	6.939	6.946	7.056
EDEM2	9.451	9.556	9.758	9.471	9.446	9.376
EDEM3	10.348	10.371	10.433	10.042	10.429	10.395
EDEM1	9.686	9.752	9.800	9.587	9.505	9.717
SAP30L	8.054	8.103	8.223	7.778	7.888	7.920
CCL2	6.014	6.061	5.895	6.072	6.285	6.236
CCL3	5.971	5.771	6.090	5.970	6.216	5.995
CCL1	5.751	6.007	5.864	5.753	6.005	5.842
CCL7	6.007	5.929	5.967	5.822	6.017	6.177
CCL4	7.582	7.586	7.362	7.557	7.737	7.595
CCL5	8.558	8.524	8.575	8.625	8.777	8.688
CCL8	5.200	5.193	5.382	5.258	5.234	5.259
AL139287.1	6.455	6.324	6.550	6.174	6.364	6.484

AP000347.1	5.801	5.603	5.522	5.620	5.549	5.741
VPS13A-AS1	7.455	7.756	7.678	7.610	7.681	7.557
HCCS	10.351	10.420	10.532	10.368	10.314	10.432
AP002495.1	7.305	7.230	7.225	7.305	7.283	7.296
UBP1	10.044	10.033	10.162	9.954	9.890	10.097
AL935156.1	7.145	7.246	7.000	7.072	7.096	7.194
PERM1	7.908	7.915	8.041	8.412	8.263	8.008
RTL9	6.926	7.007	6.815	6.803	6.980	6.945
ZNF582-AS1	7.902	8.008	7.676	8.082	7.711	8.061
AP000873.4	7.594	7.369	7.480	7.081	7.336	7.348
AP000873.2	4.741	4.779	4.738	4.660	4.698	4.471
C8orf58	8.132	8.148	8.174	7.958	8.139	8.082
TENM3-AS1	7.934	7.877	7.643	7.981	7.941	7.903
KDM6A	8.613	8.482	8.658	8.610	8.579	8.613
AC099778.1	4.620	4.794	4.651	4.742	4.838	4.648
ZNF808	4.741	4.652	4.485	4.548	4.528	4.736
ZNF805	7.016	7.046	7.022	6.666	6.957	6.869
ZNF800	8.432	8.061	8.029	8.169	8.066	8.107
QRFPR	4.910	4.908	4.681	5.059	4.911	5.172
AC009093.2	6.670	6.631	6.935	6.977	6.810	6.684
AC009093.1	5.457	5.472	5.475	5.251	5.364	5.425
AC009093.9	9.409	9.632	9.458	9.533	9.370	9.493
AC009093.8	6.670	6.631	6.935	6.977	6.810	6.684
PHB2	12.483	12.609	12.720	12.500	12.404	12.487
ACCS	7.872	7.752	7.809	7.840	7.683	7.826
CD320	9.508	9.439	9.639	9.291	9.315	9.313
AL355592.1	9.016	8.901	9.030	9.124	9.119	9.039
AC012615.1	6.023	5.833	6.189	6.044	5.798	6.009
C16orf46	7.684	7.683	7.769	7.872	7.827	7.926
TRIM8	10.012	10.018	10.129	9.975	9.864	9.956
INTU	5.223	5.145	5.395	5.231	5.186	5.153
AL161851.2	4.600	4.504	4.568	4.706	4.941	4.871
GJA10	7.932	7.953	7.887	7.951	7.997	8.076
COL9A1	6.765	7.035	6.898	6.952	6.977	7.143
AGTPBP1	7.906	7.982	7.778	7.495	7.663	7.734
GPIHBP1	7.015	6.795	6.749	6.943	7.223	7.031
CLIC1P1	6.134	6.166	5.997	6.084	6.162	6.206
AC112200.2	7.974	7.930	7.844	8.071	7.960	7.988
AC146949.1	5.859	5.735	5.446	5.456	5.312	5.507
AL359736.1	7.195	7.286	7.209	6.862	7.461	6.955
AL606534.7	8.340	8.109	7.986	8.227	8.359	8.262
AL606534.6	5.165	5.294	5.290	5.225	5.374	5.397
AL606534.4	10.722	10.564	10.478	10.430	10.307	10.610
AL606534.3	5.165	5.294	5.290	5.225	5.374	5.397
AC241409.1	6.880	6.877	6.878	6.815	6.879	6.936
TMEM191B	8.320	8.443	8.592	8.648	8.459	8.560
TMEM191C	8.320	8.443	8.592	8.648	8.459	8.560
CR936875.2	8.614	8.670	8.743	8.558	8.568	8.605
LUC7L3	10.554	10.511	10.564	10.337	10.318	10.488
LUC7L2	10.243	10.299	10.338	10.279	10.343	10.154
FAM131A	9.045	9.029	9.259	9.050	9.087	9.033
FAM131B	6.740	6.530	6.651	6.726	6.934	6.637
FAM131C	7.755	7.704	7.729	7.806	7.881	7.816
AC090574.1	4.260	4.263	4.179	4.357	4.246	4.169
EHHADH	8.471	8.530	8.473	7.997	8.269	8.316
AC040168.2	4.981	4.959	4.883	5.107	4.930	4.937

AC040168.1	5.794	5.976	5.806	5.809	5.888	5.963
NME4	10.425	10.618	10.817	10.852	10.797	10.538
NME5	7.034	7.118	7.080	7.056	7.344	6.834
NME6	8.250	8.367	8.351	8.119	8.417	8.253
NME7	13.665	13.786	13.843	13.682	13.870	13.645
NME1	12.235	12.336	12.445	12.403	12.283	12.318
NME2	12.984	13.055	13.204	13.055	12.870	13.071
NME3	9.625	9.636	9.886	9.429	9.470	9.650
NME8	5.765	5.842	5.878	5.896	6.181	6.046
NME9	6.662	6.707	6.324	6.587	6.659	6.747
FREM3	4.425	4.451	4.298	4.450	4.443	4.461
FREM2	9.095	9.072	9.084	8.520	8.716	8.875
FREM1	6.067	5.967	6.162	6.107	6.200	6.343
TEX55	5.594	5.746	5.659	5.737	5.678	5.776
TEX54	7.256	7.379	7.292	7.257	7.638	7.411
PID1	5.322	5.445	5.437	5.401	5.471	5.500
ZNF37A	7.530	7.643	7.564	7.522	7.512	7.340
TBC1D27P	7.656	7.652	7.726	7.741	7.761	7.911
AP004609.3	6.682	6.809	6.500	6.777	6.713	6.749
EPB42	6.689	6.878	6.968	6.859	6.927	6.955
EPB41	9.088	9.281	9.297	9.028	8.916	9.070
GSKIP	10.805	10.886	10.913	11.059	11.049	11.060
MATR3	12.546	12.600	12.591	12.532	12.365	12.521
AC005324.4	7.638	7.937	7.866	7.949	7.941	8.171
PDCL3	9.319	9.461	9.550	9.171	9.293	9.245
AC006059.1	7.719	7.785	7.726	8.062	7.874	7.944
AC006059.2	7.769	7.726	7.878	8.019	7.636	7.716
LINC01241	6.083	6.096	6.111	6.109	6.263	6.148
LINC01242	4.127	3.826	3.973	3.962	4.099	3.950
LINC01243	5.446	5.133	5.075	5.108	5.194	5.397
AL627230.5	8.357	8.464	8.548	8.291	8.197	8.554
AL117209.1	7.469	7.639	7.381	7.456	7.529	7.614
AC105052.4	4.558	4.912	4.936	4.458	4.592	4.634
TIAM2	7.215	7.465	7.511	7.163	7.393	7.105
AC105052.1	7.614	7.679	7.735	7.914	7.765	7.740
AC105052.3	10.960	11.004	11.108	10.912	10.811	10.780
GFOD2	8.657	8.668	8.664	8.773	8.778	8.633
GFOD1	8.398	8.553	8.437	8.458	8.466	8.408
TINCR	7.627	7.686	7.726	8.235	7.959	7.751
AC106017.2	7.648	7.611	7.631	7.628	7.663	7.739
KATNAL1	7.223	6.946	6.964	6.413	6.997	6.762
KATNAL2	6.236	6.120	6.427	6.098	6.416	6.333
ATG10	7.444	7.564	7.220	7.463	7.523	7.539
ATG12	10.019	10.078	10.115	9.783	10.176	9.669
ATG13	10.185	10.298	10.258	10.146	10.217	10.037
ATG14	8.713	8.884	8.717	8.694	8.494	8.688
AC010266.2	5.478	5.662	5.574	5.772	5.697	5.544
AP001178.3	7.087	7.177	7.085	7.018	7.111	7.158
AC018738.1	13.528	13.573	13.649	13.437	13.393	13.460
AC008132.1	7.431	7.554	7.493	7.569	7.741	7.624
VSTM2L	7.595	7.796	7.662	7.840	7.794	7.694
BX005266.2	8.597	8.585	8.594	8.520	8.633	8.531
AL353660.1	3.892	3.877	4.033	4.073	4.139	4.217
C1orf43	12.804	12.856	12.886	12.877	12.745	12.865
PTAFR	8.591	8.635	8.539	8.698	8.655	8.653
SPICE1	6.937	6.889	7.112	6.590	6.625	6.822

LINC00599	9.009	8.875	8.472	8.829	8.897	9.080
LINC00598	6.781	6.865	6.971	6.964	7.047	7.048
PKNOX1	8.434	8.254	8.395	8.083	8.138	8.194
NRSN2-AS1	6.243	6.299	6.209	6.336	6.267	6.184
LINC00115	4.708	4.836	4.639	4.899	4.758	4.860
LINC00113	5.644	6.022	5.397	5.838	5.855	5.801
LINC00112	6.522	6.639	6.749	6.705	6.763	6.655
AC004148.1	9.796	10.013	10.119	9.941	9.887	10.055
THRAP3	9.895	9.642	9.606	9.470	9.618	9.544
AC007389.3	8.438	8.493	8.255	8.519	8.588	8.486
GLCE	8.397	8.297	8.305	8.068	8.151	8.160
AC007389.1	5.764	5.760	5.621	5.872	5.946	5.896
AC073264.3	7.619	7.517	7.823	7.654	7.893	7.774
AIF1L	9.432	9.445	9.619	8.967	9.110	9.124
GSTM1	9.007	9.084	9.197	9.078	9.128	9.103
AL773541.2	8.614	8.670	8.743	8.558	8.568	8.605
GSTM3	10.597	10.632	10.907	10.699	10.617	10.653
GSTM4	9.007	9.084	9.197	9.078	9.128	9.103
GSTM5	8.461	8.592	8.640	8.536	8.587	8.655
LINC02387	6.403	6.522	6.376	6.491	6.676	6.411
LINC02381	9.861	9.877	10.161	9.802	10.105	9.700
COLGALT2	8.837	8.765	8.928	8.288	8.424	8.585
COLGALT1	10.143	10.203	10.322	10.187	10.243	10.201
LINC02382	5.984	5.935	5.777	6.029	5.842	5.958
MIR1245A	6.507	6.629	6.617	6.633	6.551	6.675
KRCC1	11.012	11.028	11.111	11.370	11.324	11.448
GPR42	6.974	7.173	7.185	7.378	7.221	7.152
RCHY1	7.429	7.650	7.664	7.572	7.650	7.726
ZC3H12D	4.180	4.243	4.151	4.036	4.097	4.145
DSTNP2	8.515	8.558	8.776	8.580	8.650	8.632
CPT1A	10.276	10.305	10.331	10.133	10.044	10.033
CPT1C	6.779	6.622	6.687	6.990	7.022	6.689
CPT1B	8.498	8.436	8.331	8.381	8.199	8.495
TPST2	8.575	8.598	8.682	8.491	8.536	8.586
TPST1	7.323	7.310	7.419	7.264	7.058	7.238
AL133320.2	6.740	6.629	6.829	7.144	6.887	6.908
MCM7	10.151	10.271	10.422	9.752	9.701	9.810
MCM6	11.287	11.346	11.404	11.408	11.249	11.280
MCM5	10.196	10.194	10.414	9.634	9.651	9.611
MCM4	9.865	9.939	9.974	9.635	9.500	9.576
DUSP19	6.229	6.263	6.158	6.074	6.112	6.264
DUSP18	8.545	8.662	8.616	8.553	8.576	8.527
N4BP2L2-IT2	6.211	6.431	6.453	6.673	6.399	6.512
DUSP15	7.725	7.429	7.797	7.796	7.804	7.828
DUSP14	10.685	10.784	10.796	10.758	10.898	10.747
DUSP16	8.180	8.209	8.462	8.451	8.524	8.474
DUSP11	9.268	9.372	9.427	9.278	9.366	9.374
DUSP10	9.267	9.264	9.404	9.459	9.335	9.409
DUSP13	7.829	7.808	7.858	7.899	8.025	7.812
DUSP12	9.295	9.340	9.261	9.120	9.055	9.109
SQSTM1	12.699	12.882	12.834	12.923	12.877	12.890
CSH2	7.785	7.620	7.627	7.744	7.836	7.768
CSH1	7.785	7.620	7.627	7.744	7.836	7.768
EPC2	8.276	8.493	8.550	8.352	8.281	8.292
EPC1	9.169	9.314	9.367	9.424	9.593	9.203
AF213884.3	7.752	7.903	7.713	7.913	7.848	7.996

HSPB11	10.408	10.482	10.566	10.163	10.288	10.187
OPRD1	7.279	7.371	7.162	7.320	7.221	7.232
AL356585.6	6.903	7.143	7.110	6.878	6.864	6.899
AL356585.1	6.903	7.143	7.110	6.878	6.864	6.899
PKMYT1	9.815	9.945	10.084	9.723	9.785	9.844
SACS-AS1	5.689	5.541	5.387	5.680	5.705	5.522
SBF2-AS1	7.134	7.148	7.174	6.965	7.161	7.148
AL121970.1	6.713	6.672	6.606	6.822	6.692	6.723
AL109627.1	7.601	7.491	7.783	7.704	7.476	7.630
BCS1L	9.354	9.399	9.558	9.350	9.384	9.391
AL445685.3	11.371	11.395	11.475	11.328	11.457	11.269
AC096564.2	3.951	3.885	4.082	3.949	4.091	4.198
AL356599.1	7.279	7.206	6.908	7.239	7.023	7.124
AL021937.4	8.737	8.526	8.393	8.779	8.936	8.750
AL021937.1	8.737	8.526	8.393	8.779	8.936	8.750
AL513477.2	6.191	6.186	6.146	6.010	6.258	6.142
TMF1	7.879	7.946	8.079	7.573	7.986	7.724
FCGBP	8.937	8.648	8.709	8.646	8.545	8.664
ANKHD1-EIF4EBI	10.030	10.021	10.062	9.697	9.801	9.935
SNORD54	13.772	13.812	13.879	13.778	13.633	13.771
ZADH2	8.615	8.647	8.653	8.640	8.517	8.514
FO538757.1	8.024	8.146	8.047	7.916	7.969	7.788
CCDC65	4.926	5.105	5.193	4.693	4.737	4.682
CCDC66	6.641	6.755	6.802	6.526	6.574	6.711
CCDC61	6.714	6.565	6.649	6.433	6.593	6.606
CCDC60	7.850	8.168	7.928	7.978	7.941	8.017
CCDC63	6.866	6.909	6.927	7.080	7.080	6.827
CCDC62	8.436	8.480	8.364	8.393	8.358	8.564
CCDC69	5.753	5.750	5.863	6.014	5.923	5.890
CCDC68	9.248	9.246	9.140	8.816	8.842	8.895
EP400	9.204	9.082	9.161	8.811	8.959	9.075
CABS1	4.072	4.205	4.159	4.348	4.231	4.360
AC212493.1	5.127	5.288	5.288	5.447	5.225	5.303
LINC02180	6.236	6.318	6.356	6.329	6.371	6.343
ANKRD18B	6.264	5.975	6.200	6.209	6.187	6.312
ANKRD18A	4.909	4.796	4.662	5.020	5.069	4.916
LINC02189	6.153	6.254	6.087	6.191	6.294	6.371
SH2D5	7.661	7.814	7.770	7.743	7.793	7.795
SH2D6	6.703	6.586	6.933	6.911	6.964	6.992
APLN	8.463	8.259	8.304	8.478	8.326	8.466
SART3	10.329	10.242	10.387	10.005	10.352	10.051
SART1	8.603	8.554	8.725	8.272	8.329	8.272
APLF	7.164	7.143	7.073	7.445	7.374	7.160
SLC25A40	8.568	8.753	8.734	8.056	8.258	8.341
SLC25A41	6.243	6.293	6.574	6.037	5.825	6.386
SLC25A42	8.140	8.161	8.233	8.161	8.151	8.121
AC007066.2	7.102	7.147	7.235	7.175	7.104	7.226
SLC25A44	9.373	9.372	9.432	9.067	9.127	9.081
LINC02239	6.792	6.542	6.415	6.866	6.614	6.732
SLC25A46	10.335	10.405	10.363	10.415	10.339	10.405
SLC25A47	7.334	7.478	7.434	7.703	7.633	7.496
SLC25A48	7.317	7.379	7.343	7.303	7.416	7.379
LINC02237	3.763	3.508	3.715	3.737	3.857	3.906
LINC02232	4.196	4.116	4.168	4.308	4.315	4.341
PDZD7	7.651	7.762	7.828	7.949	7.944	7.992
PDZD4	7.147	7.143	7.450	7.719	7.215	7.476

PDZD3	8.217	8.387	8.216	8.301	8.232	8.365
PDZD2	4.871	4.915	4.855	5.006	5.257	4.812
ANKEF1	7.921	7.905	7.783	7.798	7.805	7.839
OCLNP1	9.283	9.329	9.413	9.127	9.342	9.409
PDZD9	5.955	5.844	5.766	5.692	6.040	5.742
PDZD8	9.054	9.126	9.192	8.710	8.838	8.851
CD2BP2-DT	6.560	6.747	7.139	6.602	6.717	6.705
AC243547.3	8.798	8.823	8.944	8.501	8.605	8.706
C21orf62-AS1	8.301	8.177	8.049	8.177	8.227	8.347
AL513314.2	7.460	7.324	7.353	7.526	7.548	7.571
SGPP2	10.845	10.859	10.961	10.861	10.812	10.844
SGPP1	10.198	10.195	10.197	10.091	9.997	10.117
WTAP	10.993	10.813	11.034	10.648	10.837	10.778
ATP8A2	6.886	6.966	6.883	6.950	6.987	7.196
AL929561.2	8.529	8.524	8.065	8.442	8.567	8.533
ATP8A1	5.767	5.990	5.668	5.684	6.086	5.822
ADTRP	7.695	7.880	7.588	7.693	7.673	7.722
BLOC1S5-TXNDC	11.661	11.750	11.864	11.797	11.740	11.568
C1orf100	6.553	6.451	6.590	6.605	6.857	6.655
LRRC14B	6.770	6.853	6.884	7.243	6.961	7.150
OR10A3	5.847	6.139	6.023	6.496	5.977	6.047
OR10A2	6.376	6.675	6.559	6.449	6.517	6.852
OR10A5	7.329	7.616	7.309	7.520	7.408	7.392
C1orf105	7.373	7.373	7.127	7.540	7.467	7.589
C1orf109	8.300	8.337	8.450	7.876	7.979	8.281
UST	7.120	7.327	7.376	7.009	7.445	7.287
SLC4A1APP1	9.231	9.138	9.239	8.926	9.010	9.104
TOLLIP-AS1	5.839	5.941	6.176	5.462	5.909	5.732
NRAV	8.430	8.419	8.571	8.342	8.379	8.447
AC108451.1	6.363	6.374	6.390	6.091	6.552	6.288
PDZD11	10.834	10.928	11.075	10.834	10.875	10.847
NRAP	6.827	6.712	6.824	6.950	6.848	6.828
CABLES1	8.680	8.738	8.893	8.652	8.576	8.702
CABLES2	9.598	9.443	9.471	9.128	9.131	9.159
UMOD	7.276	7.478	7.558	7.436	7.529	7.384
LIX1L	8.404	8.517	8.563	8.125	8.030	8.442
CR759834.1	8.529	8.524	8.065	8.442	8.567	8.533
PACS1	9.237	9.317	9.373	9.260	9.403	9.134
PACS2	10.956	11.013	10.952	10.733	10.825	10.869
CR759834.2	7.145	7.246	7.000	7.072	7.096	7.194
SBNO2	9.012	8.995	9.227	9.290	9.239	9.103
SBNO1	9.566	9.535	9.635	9.377	9.564	9.510
RNF167	10.710	10.670	10.812	10.738	10.701	10.728
RNF166	7.100	7.336	7.236	6.697	6.777	6.976
RNF165	6.867	6.949	6.558	6.872	6.984	6.962
RNF169	8.388	8.365	8.365	8.203	8.171	8.214
RNF168	9.630	9.679	9.545	9.302	9.336	9.473
ZBTB40	8.560	8.521	8.414	8.424	8.310	8.347
ZBTB41	9.152	9.316	9.161	8.797	8.980	9.111
ZBTB42	8.565	8.742	8.797	8.431	8.393	8.337
ZBTB43	8.571	8.522	8.554	8.514	8.623	8.441
ZBTB44	9.101	9.237	9.317	9.131	8.946	8.769
ZBTB45	8.945	9.111	9.238	9.067	9.035	9.111
ZBTB46	7.121	7.059	7.009	7.493	7.242	7.321
ZBTB47	8.348	8.357	8.456	8.508	8.476	8.393
ZBTB48	7.938	7.933	8.277	7.644	7.890	7.789

ALOX15B	7.384	7.441	7.426	7.624	7.581	7.564
PCA3	6.092	5.964	5.950	5.939	5.977	5.876
AP000842.3	5.358	5.468	5.334	5.255	5.261	5.462
ZNF609	9.400	9.218	9.332	9.135	9.364	9.133
DRICH1	7.188	7.326	7.206	6.999	7.050	7.168
P4HA2	9.877	10.048	10.206	9.952	9.869	9.936
EPCAM-DT	5.885	5.689	5.797	5.889	6.018	6.067
AC025437.5	5.832	5.892	5.783	5.799	5.724	5.791
P4HA1	10.331	10.457	10.553	10.403	10.534	10.601
FDXR	7.698	7.555	7.787	7.581	7.331	7.557
AC068042.1	4.149	4.130	4.192	4.280	4.190	4.224
MRPL54	9.478	9.472	9.711	9.432	9.393	9.388
MRPL55	9.027	9.034	9.125	8.859	8.820	8.842
MRPL57	10.877	10.869	11.054	10.903	10.886	10.807
MRPL50	9.469	9.547	9.576	9.447	9.481	9.546
MRPL51	11.654	11.759	11.845	11.704	11.690	11.691
MRPL52	9.682	9.761	9.967	9.708	9.810	9.755
MRPL53	10.914	11.007	11.160	10.889	10.842	10.942
MRPL58	9.167	9.225	9.358	9.064	8.959	8.998
TRIQK	9.154	9.222	8.995	9.013	9.068	9.173
IQCC	7.779	7.494	7.823	8.007	7.822	7.566
IQCG	7.864	7.703	7.875	8.037	7.855	7.821
IQCE	10.116	10.230	10.125	10.041	9.904	9.988
IQCD	7.115	7.114	7.101	6.796	7.032	7.107
IQCK	9.965	10.022	10.149	9.911	9.781	9.881
IQCH	5.818	5.783	5.925	6.120	6.123	5.921
IQCN	5.467	5.290	5.382	5.664	5.538	5.701
IQCM	4.648	4.588	4.376	4.711	4.799	4.787
PMS2P11	9.313	9.354	9.149	9.020	9.274	9.164
WDTC1	9.434	9.524	9.425	9.502	9.493	9.511
FDX2	8.598	8.548	8.677	8.515	8.525	8.580
MFAP3	8.230	8.389	8.316	8.264	8.146	8.400
FDX1	10.315	10.457	10.499	10.104	10.072	10.097
MFAP1	10.121	10.276	10.326	9.917	9.976	10.059
TCF4	6.738	6.801	6.656	6.735	6.738	6.794
DSTYK	7.645	7.369	7.468	7.350	7.621	7.369
H2BW3P	5.801	5.991	5.823	5.624	5.876	5.507
PPP1R1A	6.875	6.698	6.899	7.110	6.992	6.894
PPP1R1C	8.049	8.098	8.054	8.017	7.945	8.073
PPP1R1B	7.969	8.059	7.827	8.035	8.371	7.995
AC009237.14	6.335	6.323	6.774	6.318	6.407	6.460
GSX2	7.725	7.855	7.924	8.109	7.962	8.091
AC123768.4	5.891	5.762	5.710	5.673	5.823	5.810
GSX1	8.213	8.231	7.879	8.598	8.234	8.295
AC123768.3	6.001	6.008	5.892	6.347	6.123	6.186
AL138752.2	9.706	9.772	9.885	9.962	9.948	9.802
NF2	10.144	10.142	10.225	10.139	10.026	10.193
NF1	8.731	8.865	8.807	8.756	8.831	8.866
IL5RA	7.414	7.304	7.406	7.474	7.462	7.623
AC079336.2	5.576	6.070	6.008	5.759	5.856	5.784
BLM	9.328	9.180	9.474	9.113	9.147	9.192
PPP1R10	9.995	10.059	10.125	10.133	9.984	10.136
GOLGA2P2Y	6.210	6.139	6.179	6.290	6.315	6.341
PPP1R17	6.998	6.954	7.044	7.125	7.056	7.011
RPL10P9	13.794	13.873	13.940	13.883	13.836	13.884
PPP1R18	10.614	10.607	10.686	10.372	10.366	10.371

RPL10P2	11.769	11.914	12.101	11.666	11.734	11.927
ERVK3-1	8.845	8.887	8.911	8.976	8.959	9.090
SIRPB2	5.234	5.266	5.124	5.327	5.388	5.308
SIRPB1	8.098	7.973	7.930	8.037	8.232	8.058
OBI1	7.597	7.533	7.498	7.045	7.312	7.226
ATXN2	9.745	9.874	10.086	9.950	9.869	9.899
ATXN3	7.667	7.668	7.700	7.136	7.259	7.235
ATXN1	7.609	7.693	7.599	7.299	7.270	7.343
AC270297.1	4.418	4.422	4.779	4.788	4.380	4.662
RECK	4.983	4.994	4.898	4.903	5.025	5.027
TRAV10	6.930	7.078	6.957	7.075	7.135	7.253
TRAV16	6.073	6.051	6.130	6.215	6.206	6.117
TRAV17	5.602	5.618	5.691	5.681	5.688	5.692
AC018558.1	7.673	7.707	7.849	8.100	8.089	7.830
STRIP1	8.470	8.442	8.435	8.526	8.482	8.533
AP006621.2	6.225	6.252	6.225	6.018	6.081	6.322
AP006621.5	8.154	8.211	8.052	7.985	7.951	8.107
IFI44L	11.005	11.121	11.013	11.142	11.266	11.025
AREL1	8.954	9.079	8.963	8.830	8.664	8.700
HMGN2P7	12.569	12.690	12.791	12.495	12.553	12.522
HMGN2P6	12.569	12.690	12.791	12.495	12.553	12.522
HMGN2P5	12.569	12.690	12.791	12.495	12.553	12.522
OR7E156P	8.562	8.561	8.519	8.407	8.723	8.734
HMGN2P3	12.569	12.690	12.791	12.495	12.553	12.522
TAOK1	10.220	10.235	10.178	9.885	10.078	10.054
HMGN2P9	9.164	9.150	9.247	9.182	9.153	9.376
LEPR	6.930	7.018	7.030	7.008	7.024	7.042
LASP1	13.449	13.507	13.567	13.539	13.527	13.527
TMEM132D-AS1	7.126	7.239	7.141	7.416	7.188	7.201
MICALL1	10.301	10.305	10.338	10.191	10.151	10.154
MICALL2	9.024	9.026	9.243	8.946	8.733	8.952
SNORD116-20	6.639	6.757	6.724	6.961	6.931	6.798
TAOK3	9.710	9.809	9.922	9.806	9.810	9.466
ZNF813	4.235	4.271	4.237	4.410	4.594	4.484
PEAR1	7.437	7.323	7.304	7.671	7.403	7.565
ZNF816	7.530	7.711	7.712	7.910	8.297	7.856
CRISP1	6.566	6.621	6.033	6.511	6.454	6.525
CRISP3	7.630	7.599	7.620	7.069	7.359	7.183
CRISP2	4.150	4.152	4.260	4.251	4.370	4.260
ZNF814	7.908	7.849	7.970	6.851	7.502	7.434
HHIP-AS1	7.607	7.778	7.665	7.795	7.876	7.852
VEPH1	9.362	9.265	9.396	9.149	9.121	9.336
MREG	9.563	9.607	9.777	9.660	9.765	9.615
KLF10	8.849	8.949	8.812	8.783	8.933	8.959
AC011481.1	5.917	6.160	5.948	6.062	6.221	6.198
AL391845.2	6.543	6.325	6.294	6.352	6.455	6.347
AC018943.1	6.886	6.972	7.153	7.073	6.994	7.191
AP001972.5	9.133	9.329	9.192	9.140	9.311	9.281
FGF14-AS2	6.405	6.296	6.237	6.332	6.220	6.204
AL132989.2	6.575	6.647	6.132	6.635	6.635	6.806
SYNJ2BP	9.717	9.605	9.644	9.421	9.373	9.569
EEFSEC	9.613	9.650	9.842	9.732	9.731	9.711
DPY19L1P2	9.021	9.027	9.063	8.745	8.843	8.895
DPY19L1P1	5.054	5.088	5.022	5.109	4.958	5.035
AC006978.1	8.276	7.989	8.037	8.042	7.756	8.281
NHEJ1	9.564	9.400	9.593	9.330	9.449	9.279

KPTN	8.334	8.311	8.233	8.149	8.270	8.192
SCARB2	12.369	12.393	12.412	12.117	12.037	12.133
SCARB1	10.433	10.452	10.491	10.350	10.338	10.363
AL117379.1	7.522	7.395	7.261	7.258	7.414	7.366
HULC	5.338	5.408	5.197	5.347	5.374	5.472
RRAD	6.186	6.306	6.220	6.978	6.636	6.927
RRAS	10.247	10.425	10.747	10.142	10.335	10.161
AC013275.1	4.256	4.218	3.951	4.231	4.192	4.116
AL354743.2	6.961	6.667	6.739	7.060	6.869	6.950
RNPS1	11.871	11.908	11.941	11.543	11.535	11.525
AC019069.1	7.511	7.430	7.576	7.386	7.476	7.256
NCF1C	6.773	6.804	7.004	7.053	6.965	6.805
NCF1B	6.773	6.804	7.004	7.053	6.965	6.805
AL591379.1	8.464	8.364	8.135	8.347	8.305	8.294
C11orf45	6.743	6.737	6.792	6.330	6.599	6.545
MYBPC3	8.658	8.622	8.665	8.757	8.718	8.708
MYBPC2	6.321	6.469	6.165	6.165	6.512	6.420
MYBPC1	6.157	6.121	6.153	6.340	6.254	6.192
PRPF40B	7.998	8.143	8.221	8.154	8.134	8.157
SPRY3	6.188	6.249	6.268	6.332	6.252	6.182
SPRY2	7.847	7.998	8.084	7.799	7.956	7.899
SPRY1	9.100	9.148	9.182	9.144	9.166	9.080
TRPC1	7.935	8.034	8.161	7.725	7.768	7.837
TRPC6	5.600	5.526	5.295	5.717	5.684	5.710
TRPC7	5.733	5.847	5.883	6.120	5.851	5.886
TRPC4	7.121	7.133	7.392	7.565	7.233	7.301
SPRY4	9.373	9.465	9.353	9.391	9.452	9.475
RASSF10	6.109	6.227	6.352	6.571	6.782	6.326
TNFSF15	8.030	8.029	8.068	7.857	7.869	7.905
LINC02436	4.212	4.311	4.336	4.323	4.217	4.448
TNFSF14	7.364	7.485	7.212	7.648	6.982	7.468
LINC02433	3.940	3.820	3.815	4.131	3.958	3.939
LINC02431	6.817	6.657	6.575	7.264	7.153	6.971
TBX10	8.016	7.810	7.845	8.332	8.207	8.084
USF3	8.059	7.489	7.534	7.861	7.900	7.837
USF1	7.380	7.272	7.203	7.329	7.325	7.403
TBX15	7.052	6.862	6.761	7.114	7.120	6.860
TBX18	7.768	7.781	7.743	7.724	7.786	7.649
TBX19	7.630	7.667	7.723	7.814	7.930	7.934
UTP15	7.396	7.633	7.474	7.099	7.285	7.366
AMBRA1	8.971	9.184	9.231	9.077	9.126	9.007
AC131392.2	4.268	4.234	4.359	4.378	4.576	4.384
MIR3976HG	5.847	5.829	5.660	5.855	5.779	5.895
AC027031.2	8.132	8.146	8.214	7.931	7.972	8.136
DICER1-AS1	5.942	5.883	5.874	6.054	5.845	5.710
AC079922.2	7.906	7.764	8.151	7.693	7.859	7.514
RBMX	11.739	11.805	11.872	11.619	11.607	11.569
TRAV8-1	7.660	7.529	7.870	8.025	7.788	7.703
TRAV8-3	6.717	6.441	6.408	6.558	6.615	6.546
TRAV8-6	7.759	7.892	7.852	7.836	7.920	7.968
SLIRP	11.631	11.743	11.812	11.422	11.306	11.340
NDUFS1	9.682	9.679	9.826	9.489	9.452	9.378
AC109479.5	7.883	7.866	7.860	7.971	7.699	7.896
NDUFS2	11.154	11.208	11.306	10.870	11.122	10.829
LINC01165	5.623	5.697	5.508	5.746	5.680	5.788
NOD2	5.420	5.688	5.837	5.715	5.883	5.781

LINC01169	4.899	5.025	5.070	5.438	5.109	5.297
DYNC1H1	10.746	10.674	10.731	10.414	10.204	10.497
CCNB3	7.419	7.387	7.231	7.296	7.482	7.386
CCNB2	10.559	10.473	10.724	10.086	10.270	10.083
CCNB1	9.252	9.350	9.563	9.089	9.249	9.020
VPS18	8.944	9.149	9.093	8.826	9.006	8.941
VPS16	8.751	8.710	8.754	8.549	8.516	8.492
VPS11	9.011	8.966	9.095	8.978	8.993	8.929
LRRK2-DT	4.041	3.958	3.902	4.012	3.908	3.914
PPY2P	4.994	5.234	5.153	5.110	4.914	4.968
TRBV5-2	7.863	7.561	7.531	7.693	7.734	7.792
AC017083.3	8.963	8.699	8.848	8.489	8.521	8.616
C6orf99	6.349	6.598	6.596	6.650	6.446	6.408
PZP	7.266	7.041	6.763	7.235	7.113	7.405
QPCTL	7.075	7.409	7.211	7.153	7.527	7.497
AF067845.6	5.512	5.568	5.763	5.548	5.607	5.654
CHMP2B	10.187	10.198	10.297	10.230	10.525	10.279
AC024558.2	9.072	9.126	9.178	8.714	8.714	8.789
GATD3B	10.399	10.354	10.596	10.369	10.330	10.402
AC226101.1	4.748	4.941	4.924	4.823	4.858	5.010
AP000577.2	4.610	4.640	4.779	4.570	4.679	4.751
TCF7L1	6.586	6.608	6.640	6.582	6.855	6.750
TCF7L2	10.727	10.740	10.706	10.489	10.460	10.645
AL163636.3	10.035	9.861	10.050	9.641	9.693	9.861
SPESP1	3.877	3.829	3.918	3.936	3.877	3.979
PCDHGB7	11.092	11.102	11.188	11.070	11.043	11.048
GBF1	9.286	9.299	9.320	9.362	9.403	9.300
FMO6P	6.607	6.544	6.398	6.635	6.868	6.470
MFF	10.686	10.673	10.700	10.350	10.454	10.657
BICC1	9.395	9.243	9.294	9.147	9.100	9.157
TM4SF20	5.438	5.245	5.547	5.541	5.361	5.347
AC011840.1	6.688	6.564	6.764	6.565	6.691	6.823
DDT	10.518	10.608	10.873	10.576	10.453	10.549
DDO	7.308	7.252	7.258	7.160	7.288	7.415
DDN	8.224	8.227	8.199	8.469	8.455	8.295
LAMC2	11.047	11.142	11.120	11.134	11.060	11.051
LAMC3	7.134	7.301	7.407	7.362	7.441	7.504
FAM240C	5.215	5.277	5.562	5.380	5.408	5.475
LAMC1	11.307	11.269	11.428	11.150	11.052	11.229
RWDD4	8.068	8.204	8.159	8.050	8.032	8.168
RWDD3	8.001	7.929	8.104	7.814	7.847	7.915
RWDD1	9.014	9.030	9.111	8.911	9.070	9.086
AL121821.2	6.402	6.328	6.098	6.159	6.193	6.408
AC008581.2	8.767	8.788	8.763	8.712	9.140	8.464
TMEM161B-AS1	6.841	6.623	7.054	7.255	7.149	7.076
TCHP	9.606	9.534	9.395	9.318	9.438	9.434
TCHH	5.705	6.002	5.686	5.993	5.738	5.794
AL132857.1	4.229	4.140	4.229	4.208	4.335	4.296
AL132857.2	5.439	5.756	5.726	5.399	5.655	5.648
ERN1	8.488	8.418	8.530	8.149	8.209	8.294
PCBD1	10.135	10.224	10.331	10.146	10.168	10.110
RIMBP2	7.201	7.331	7.285	7.146	7.166	7.011
RIMBP3	8.535	8.490	8.448	8.513	8.691	8.374
API5P1	8.902	8.847	8.964	9.045	8.940	8.823
AC213203.3	6.671	6.434	6.543	6.909	6.630	6.641
CATIP-AS1	8.342	8.381	8.126	8.350	8.284	8.270

AC009407.1	8.631	8.732	8.898	8.778	8.979	8.924
TRAC	8.321	8.046	8.218	8.465	8.444	8.309
TPTEP2-CSNK1E	8.174	7.978	8.256	8.037	8.053	8.062
AC007608.3	5.547	5.502	5.583	5.755	5.488	5.450
MDH2	11.656	11.806	11.899	11.567	11.604	11.622
MDH1	12.210	12.227	12.388	12.232	12.104	12.209
TGIF2LY	6.987	6.837	7.069	7.086	7.241	7.154
TGIF2LX	6.987	6.837	7.069	7.086	7.241	7.154
CNTNAP3B	8.431	8.367	8.369	8.136	8.111	8.292
RMST	6.465	6.592	6.553	6.788	6.780	6.679
ECEL1	7.895	8.087	7.929	8.011	8.076	8.162
TERT	7.574	7.484	7.552	7.468	7.650	7.379
SORD2P	10.296	10.383	10.493	10.290	10.272	10.260
AP003307.2	7.149	7.231	7.498	7.149	7.342	7.176
AL159141.1	8.260	8.449	8.539	8.321	8.324	8.320
ZNF367	8.186	8.231	8.178	7.924	8.035	8.036
SYNPO2L	8.032	7.874	7.559	7.837	8.121	8.055
EMC10	11.534	11.578	11.752	11.544	11.391	11.458
CASP6	8.449	8.846	9.036	8.484	8.451	8.154
CASP7	9.298	9.537	9.370	9.361	9.343	9.456
CASP4	8.498	8.524	8.674	8.369	8.537	8.547
CASP5	7.166	7.145	7.333	7.212	7.217	7.174
CASP2	10.193	10.183	10.240	9.739	9.816	9.769
CASP3	8.518	8.670	8.623	8.412	8.413	8.278
CASP1	8.553	8.656	8.599	8.558	8.643	8.611
CASP8	9.632	9.567	9.531	9.380	9.422	9.444
CASP9	8.573	8.757	8.744	8.689	8.674	8.745
ANKRD20A7P	8.236	8.156	8.141	8.146	8.143	8.231
LINC02372	4.483	4.522	4.410	4.623	4.483	4.756
LINC02375	4.599	4.487	4.360	4.502	4.707	4.410
DOLPP1	9.896	9.947	10.016	9.860	9.839	9.889
AKT1	10.539	10.705	10.790	10.628	10.640	10.456
AKT2	9.961	9.870	10.053	9.739	9.745	9.695
AKT3	10.072	10.087	10.177	10.093	10.102	10.005
UPB1	8.187	8.092	8.403	8.293	8.502	8.238
PKD1L1	5.993	6.042	5.933	6.128	6.116	6.043
PKD1L2	7.957	8.168	7.959	7.872	8.308	8.056
CROT	7.889	7.868	7.591	7.497	7.624	7.760
KRT23	12.771	12.832	12.915	12.752	12.746	12.618
KRT20	6.543	6.721	6.770	6.601	6.792	6.541
KRT27	6.664	6.843	6.620	6.606	6.684	6.974
KRT24	6.979	6.726	6.993	7.040	7.070	6.955
KRT25	5.844	5.738	5.660	5.928	5.848	5.815
AC005009.1	4.940	4.779	4.778	5.064	4.799	4.845
HYAL3	7.180	7.082	7.424	7.189	7.408	7.267
HYAL2	8.941	9.081	9.196	9.078	9.042	8.965
HYAL1	7.010	7.272	7.184	7.174	7.241	7.174
HYAL4	6.048	6.025	6.074	6.463	6.321	6.201
AL121994.1	9.645	9.664	9.679	9.555	9.635	9.543
AC231533.2	10.837	10.833	11.041	10.739	10.687	10.776
AL159169.2	4.952	4.772	4.916	4.921	5.151	5.126
Z92544.1	7.875	8.165	8.133	8.248	8.160	8.195
PNMA1	10.521	10.488	10.552	10.168	10.232	10.309
PNMA3	8.186	8.223	8.190	8.154	8.240	8.364
PNMA2	9.458	9.490	9.676	9.038	9.047	9.129
SLC4A9	7.331	7.430	7.552	7.810	7.430	7.372

AC008763.2	9.342	9.460	9.558	9.416	9.426	9.493
AC008763.3	9.973	10.007	10.291	9.954	9.872	9.987
AC004232.2	6.100	6.032	5.974	6.144	6.167	5.991
SRP19	10.020	10.007	10.002	9.790	9.859	9.910
AC099508.1	5.856	5.837	6.009	5.932	6.004	5.994
AC020636.2	8.891	8.613	8.703	8.869	8.941	8.895
LRIG2-DT	4.595	4.762	4.526	4.640	4.787	4.639
CIITA	8.422	8.391	8.202	8.474	8.474	8.355
NCF2	6.319	6.233	6.278	6.078	6.112	6.236
NCF1	6.773	6.804	7.004	7.053	6.965	6.805
Z93929.1	4.671	4.650	4.483	4.643	4.569	4.563
NCF4	6.197	6.189	5.934	6.007	6.132	6.251
CSMD2-AS1	8.012	8.023	7.967	8.155	7.885	8.212
AL390719.2	8.288	8.312	8.319	8.400	8.250	8.307
AL390719.1	7.902	7.747	7.806	7.705	7.825	7.867
AC003986.1	7.111	7.375	7.073	7.165	7.244	7.317
AC125437.1	5.791	5.650	5.740	5.929	5.892	5.724
NBPF19	11.550	11.483	11.377	11.343	11.231	11.508
LIMD1-AS1	8.333	8.451	8.504	8.570	8.511	8.432
BOLA2B	10.698	10.730	10.949	10.678	10.375	10.636
ABHD16B	5.951	6.231	6.221	6.177	6.204	6.301
ABHD16A	8.862	8.931	9.098	8.915	8.886	8.916
UNC13D	8.312	8.314	8.311	8.417	8.531	8.383
UNC13B	10.028	10.071	10.171	9.967	9.915	9.981
UNC13C	6.800	6.852	6.634	6.861	6.878	7.087
UNC13A	7.398	7.372	7.369	7.495	7.638	7.430
KCNB2	7.792	7.668	7.685	7.908	8.152	8.025
KCNB1	7.657	7.551	7.444	7.731	7.782	7.785
DEGS1	10.268	10.339	10.377	10.263	10.271	10.262
DEGS2	7.538	7.519	7.623	7.720	7.654	7.703
OSGIN2	9.837	9.915	10.018	9.864	9.725	9.760
RFTN1P1	6.018	6.074	6.046	6.143	6.098	6.246
OSGIN1	8.145	8.327	8.375	8.425	8.371	8.323
PHACTR2-AS1	5.126	5.131	4.816	5.250	5.150	4.973
GABRA2	4.655	4.481	4.662	4.736	4.685	4.646
RUNDC3B	4.513	4.430	4.426	4.783	4.589	4.433
RUNDC3A	7.866	7.983	8.091	8.068	8.085	8.051
CNTNAP1	7.376	7.279	7.334	7.197	7.444	7.389
CNTNAP3	8.431	8.367	8.369	8.136	8.111	8.292
CNTNAP2	6.931	7.074	6.916	7.115	7.460	7.023
CNTNAP5	7.261	7.361	6.881	7.109	7.080	7.034
CNTNAP4	8.099	8.095	8.102	7.811	7.980	7.992
ZNF433-AS1	5.760	5.533	5.825	5.828	5.769	5.804
BLZF1	8.059	8.181	8.113	7.764	8.010	8.125
BHLHE22	7.578	7.435	7.248	7.422	7.411	7.603
BHLHE23	6.192	6.529	6.281	6.463	6.202	6.473
USP27X	7.590	7.650	7.786	7.418	7.488	7.594
MAP7D3	7.629	7.693	7.561	7.521	7.530	7.614
MAP7D2	5.677	5.793	5.467	5.529	5.924	5.770
MAP7D1	9.687	9.910	9.941	9.632	9.655	9.694
HORMAD2	7.181	7.104	7.134	7.017	7.016	7.351
KIAA1211L	9.059	8.945	9.050	8.824	8.931	8.823
HORMAD1	4.811	4.625	4.382	4.894	4.729	4.867
ZSCAN32	8.928	8.877	8.889	8.865	8.814	8.943
G3BP1	11.479	11.520	11.537	11.402	11.355	11.419
G3BP2	11.135	11.156	11.184	11.003	10.915	11.131

OR2B2	5.416	5.358	4.900	5.839	5.445	5.362
OR2B3	6.854	7.019	6.695	6.772	6.980	7.069
OR2B6	6.411	6.226	6.351	6.360	6.440	6.608
ZFY	5.073	5.072	5.059	4.927	5.026	4.955
IFNAR2	9.322	9.487	9.476	9.392	9.441	9.474
IFNAR1	8.787	8.808	8.930	8.609	8.628	8.696
OSTCP1	4.317	4.418	4.053	4.195	4.284	4.252
AC027644.3	7.070	6.965	7.108	7.038	6.894	6.757
AC027644.4	7.985	8.194	8.101	7.820	7.947	7.949
Z84492.1	7.297	7.230	7.359	6.956	7.170	7.272
Z84492.2	7.297	7.230	7.359	6.956	7.170	7.272
IRAK2	8.371	8.438	8.509	8.584	8.717	8.680
IRAK3	6.797	6.635	6.827	6.812	6.813	6.719
IRAK1	12.486	12.591	12.721	12.443	12.353	12.384
AC091167.7	6.707	6.766	6.612	7.011	6.884	6.885
AC091167.2	12.100	12.172	12.264	12.179	12.116	12.160
SMARCD3	7.218	7.260	7.396	6.977	7.204	7.159
SMARCD2	8.763	8.756	8.971	8.655	8.607	8.520
SMARCD1	9.771	9.676	9.740	9.890	9.707	9.618
CEP295	7.843	7.512	7.809	7.313	7.915	7.330
CEP290	8.222	8.111	8.186	7.914	8.119	8.088
IPCEF1	5.637	5.614	5.450	5.778	5.531	5.685
TNKS	8.042	8.089	8.163	7.430	7.700	7.738
AC008038.1	14.111	14.146	14.160	14.135	14.090	14.165
PRR34-AS1	9.564	9.597	9.826	9.667	9.504	9.722
ACVR1B	10.049	10.094	10.173	10.000	10.051	9.932
GABBR1	8.522	8.629	8.590	8.842	8.805	8.562
GABBR2	7.636	7.551	7.589	7.969	7.691	7.733
SERPINE1	7.586	7.701	7.471	7.680	7.612	7.532
SERPINE2	11.351	11.452	11.496	11.026	11.105	11.034
SOX18	4.234	4.463	4.436	4.238	4.324	4.520
SSTR1	6.928	7.032	6.868	6.867	7.118	6.950
TNNT3	7.067	7.195	7.027	7.494	7.182	7.341
TNNT2	8.270	8.381	8.368	8.475	8.525	8.425
SOX10	8.724	8.728	8.692	8.900	8.916	8.685
SOX11	6.100	5.869	5.895	6.079	6.213	5.827
SOX12	7.731	7.697	7.963	7.550	7.725	7.599
SOX13	9.090	9.072	9.017	9.069	9.092	8.980
SOX14	5.304	5.530	5.433	5.530	5.538	5.483
SOX15	7.100	7.173	7.131	7.663	7.478	7.335
SOX17	5.891	6.096	5.845	6.122	6.042	5.985
AC132217.2	7.019	6.935	6.993	7.196	7.062	7.018
C4A	8.508	8.681	8.617	8.611	8.632	8.722
DMAP1	8.017	8.119	8.279	7.671	7.759	7.803
ARIH2OS	5.361	5.412	5.643	5.320	5.318	5.235
TOPORS	9.309	9.178	9.319	9.025	9.287	9.126
PDLIM4	7.209	7.232	7.363	7.107	7.136	7.255
PDLIM5	11.376	11.441	11.413	11.171	11.082	11.212
PDLIM7	9.648	9.676	9.956	9.766	9.807	9.631
PDLIM1	10.535	10.735	10.788	10.433	10.572	10.429
PDLIM2	8.075	8.269	8.287	8.221	8.146	8.119
PDLIM3	6.865	7.269	7.250	7.574	7.375	7.287
FTX	8.519	8.559	8.020	8.188	8.161	8.122
TEKT4P2	10.047	10.053	10.201	10.055	10.078	10.039
MCCC2	10.695	10.808	10.903	10.649	10.631	10.614
MCCC1	11.315	11.328	11.329	11.218	11.029	11.083

AL021368.3	6.022	5.795	5.978	5.854	5.691	6.017
AL021368.2	6.208	6.243	5.920	6.406	6.416	6.188
TNK1	9.178	9.253	9.301	9.301	9.395	9.246
ZDHHC2	10.072	10.030	9.912	9.917	9.853	10.034
ZDHHC5	11.354	11.367	11.374	11.138	11.110	11.135
ZDHHC4	10.338	10.493	10.588	10.271	10.288	10.219
ZDHHC7	11.328	11.452	11.546	11.360	11.197	11.128
ZDHHC6	9.675	9.782	9.816	9.634	9.561	9.795
ZDHHC9	11.026	11.127	11.180	11.138	11.342	10.901
ZDHHC8	9.888	9.906	9.917	9.851	9.860	9.857
MTLN	9.147	9.152	9.368	9.190	9.360	9.246
TTC39C	7.808	7.841	7.845	7.565	7.462	7.546
HES1	10.618	10.563	10.733	11.141	11.184	10.786
HES2	8.189	8.439	8.391	8.272	8.262	8.396
HES4	9.672	9.706	10.021	9.471	9.650	9.599
HES5	6.558	6.485	6.494	6.763	6.733	6.669
HES6	8.514	8.311	8.439	8.275	8.476	8.314
HES7	7.816	7.982	8.116	7.949	7.948	7.899
AC027801.1	7.952	7.998	8.050	7.745	7.874	7.794
CAPNS1	13.247	13.360	13.400	13.195	13.216	13.191
CAPNS2	7.211	7.308	7.133	7.475	7.278	7.366
CDC123	10.252	10.289	10.439	10.122	10.098	10.115
IGSF9	7.052	6.979	7.180	7.005	7.058	7.080
PSD3	8.700	8.933	8.958	8.335	8.715	8.205
RPS28P7	13.528	13.573	13.649	13.437	13.393	13.460
GNAS-AS1	5.004	4.935	4.931	4.826	5.147	4.970
H1-7	7.185	7.353	7.374	7.503	7.620	7.539
CROCCP3	7.408	7.510	7.443	7.417	7.620	7.443
CROCCP2	8.374	8.412	8.425	8.205	8.416	8.417
C2orf16	8.672	8.689	8.711	8.706	8.607	8.922
OCRL	9.418	9.391	9.349	9.273	9.332	9.435
C2orf15	6.442	6.553	6.626	6.058	6.254	6.164
H1-1	6.506	6.302	6.379	6.739	6.506	6.359
IGSF1	7.056	7.026	7.037	6.877	7.194	7.076
WDR62	8.292	8.528	8.614	8.497	8.404	8.332
WDR63	5.117	4.994	5.014	5.122	5.036	5.006
ENO1-AS1	5.596	5.367	5.619	5.366	5.329	5.369
WDR60	7.640	7.273	7.479	7.302	7.355	7.374
GPR153	9.281	9.379	9.424	9.387	9.532	9.430
GPR157	8.292	8.402	8.321	8.247	8.450	8.260
GPR156	8.414	8.569	8.566	8.571	8.763	8.607
GPR155	7.849	7.748	7.915	7.756	8.003	7.791
GPR158	7.061	6.945	6.736	6.861	6.939	6.960
ADGRG1	12.082	12.124	12.214	11.963	11.860	12.004
TG	7.791	7.632	7.578	8.004	7.851	7.600
WDR64	6.030	6.167	6.162	6.205	6.146	6.274
TMEM151B	7.166	7.426	7.284	7.439	7.549	7.380
TMEM151A	7.248	7.414	7.463	7.373	7.411	7.285
TEPSIN	8.488	8.462	8.427	8.567	8.485	8.420
AC022007.1	5.916	5.556	5.668	5.647	5.757	5.635
MITF	10.500	10.475	10.581	10.398	10.325	10.151
AC104024.2	7.349	7.328	7.363	7.179	7.347	7.319
RPTOR	8.021	8.067	8.048	8.223	8.360	8.196
QKI	9.995	10.002	10.023	9.748	9.822	9.930
PPFIA2	6.196	6.441	6.386	6.541	6.474	6.340
PPFIA3	7.496	7.522	7.771	7.508	7.477	7.511

PPFIA1	9.952	9.940	9.971	9.656	9.615	9.896
CR392039.1	6.476	6.270	6.787	6.705	6.102	6.706
TM4SF1	13.400	13.486	13.447	13.494	13.496	13.443
TM4SF4	13.446	13.501	13.472	13.534	13.518	13.529
TM4SF5	6.612	6.833	6.856	7.134	6.938	6.834
TVP23CP2	9.099	9.065	9.036	8.806	9.078	9.206
EIF1AXP1	11.622	11.764	11.789	11.778	11.868	11.642
RYBP	10.334	10.409	10.509	10.368	10.542	10.096
MIR5188	5.373	5.837	5.602	5.726	5.482	5.592
RTN4	13.438	13.473	13.480	13.352	13.417	13.384
KPNB1	12.784	12.773	12.879	12.703	12.902	12.466
RTN1	5.641	5.834	5.568	5.890	5.401	5.684
RTN2	7.891	8.054	8.073	7.699	7.730	7.868
RTN3	11.781	11.915	11.924	11.754	11.741	11.855
HGF	7.440	7.583	7.575	7.554	7.678	7.690
HGD	8.903	9.021	9.133	8.993	9.075	8.978
C7orf69	6.506	6.571	6.528	6.982	6.872	6.831
BACE2	10.005	10.017	10.219	9.907	10.183	9.719
IER5	9.073	9.207	9.283	8.942	8.906	9.051
SLU7	9.060	9.010	9.178	8.843	9.061	9.028
IER2	11.654	11.705	11.767	11.801	11.739	11.754
IER3	11.978	11.979	12.321	12.040	12.022	12.049
CILP	8.112	8.158	7.865	8.128	8.110	8.296
ZNF468	8.236	8.130	8.279	7.867	7.990	8.005
ZNF469	8.347	8.446	8.397	8.534	8.493	8.677
AC048341.2	6.866	6.700	6.635	6.469	6.359	6.710
CYB561D1	9.072	9.114	9.209	9.053	8.992	9.137
CYB561D2	9.545	9.481	9.632	9.414	9.486	9.489
AC048341.1	6.217	5.942	6.123	6.449	6.064	6.433
ZNF467	7.154	7.107	7.462	7.323	7.374	7.366
MUC20P1	10.442	10.486	10.509	10.224	9.982	10.301
HUWE1	11.717	11.773	11.738	11.593	11.518	11.658
LINC01554	5.645	5.857	5.668	5.774	5.668	5.786
LINC01555	5.945	5.886	5.877	6.229	5.869	5.967
PKP4	10.228	10.310	10.380	10.226	10.140	10.215
LINC01550	6.883	6.917	7.034	7.115	7.067	7.328
LINC01551	7.174	6.825	6.955	7.052	7.010	7.127
PKP1	7.256	7.429	7.200	7.550	7.463	7.473
LINC01553	3.917	4.023	3.928	4.000	4.010	4.013
LINC01558	7.112	7.300	7.002	7.132	7.198	7.133
ROS1	6.304	6.275	6.099	6.532	6.374	6.410
METTL7A	8.450	8.414	8.450	8.032	8.161	8.156
METTL7B	10.458	10.596	10.669	10.396	10.316	10.329
SNORD73B	13.488	13.531	13.626	13.781	13.787	13.813
EYS	4.239	4.139	4.104	4.266	4.121	4.073
NQO2-AS1	6.069	6.003	5.986	6.200	6.193	5.906
LINC02600	7.778	7.749	7.802	8.007	7.853	7.866
LINC02603	6.055	6.245	5.906	6.108	6.175	6.169
LINC02604	8.210	8.076	7.849	8.255	8.259	8.228
LINC02606	6.480	6.279	6.249	6.414	6.395	6.263
LINC02609	3.630	4.036	3.983	3.970	4.147	3.954
WFS1	8.784	8.856	8.868	8.690	8.570	8.770
ELMO1	7.199	7.241	6.831	7.249	7.239	7.271
ELMO2	9.460	9.496	9.570	9.247	9.347	9.426
ELMO3	9.670	9.710	9.863	9.491	9.389	9.494
MBLAC2	8.333	8.412	8.418	8.144	8.207	8.044

MBLAC1	7.639	7.712	7.516	7.598	7.670	7.763
AL118522.1	7.359	7.328	7.203	7.156	7.309	7.362
AC002463.1	4.739	4.680	4.662	4.593	4.756	4.697
LINC00607	5.618	5.781	5.510	5.644	5.634	5.675
LINC00606	5.170	5.127	5.137	5.173	5.279	5.153
MARCHF10	6.473	6.565	6.397	6.552	6.683	6.423
MARCHF11	6.104	6.261	5.939	6.185	6.213	6.393
TIRAP	8.552	8.730	8.448	8.589	8.378	8.728
LINC00608	5.672	5.705	5.932	5.955	5.925	5.838
PLXDC2	6.324	6.161	6.398	6.221	6.382	6.475
PLXDC1	6.831	6.698	6.745	6.993	6.941	6.900
MIR600HG	7.827	7.727	7.768	7.657	7.705	7.783
AC018653.7	5.687	6.129	6.100	6.331	6.038	6.128
AC018653.3	5.687	6.129	6.100	6.331	6.038	6.128
PRKCQ-AS1	6.729	6.913	6.844	6.850	6.822	6.876
PAAF1	9.291	9.373	9.438	9.144	9.310	9.255
AL451165.2	8.099	8.241	8.311	8.249	8.372	8.274
AC108482.2	5.441	5.231	5.382	5.417	5.308	5.277
FO393400.1	7.838	7.850	7.855	8.009	8.072	8.031
FILIP1L	7.715	7.731	7.768	7.730	7.661	7.705
NOXO1	6.830	6.615	6.959	6.653	6.733	6.786
ATXN7L3	10.010	9.912	9.996	9.916	9.963	9.957
AC022447.2	7.651	7.744	7.803	7.865	7.772	7.870
ATXN7L1	7.597	7.776	7.823	7.319	7.597	7.509
ZNF350	6.271	6.331	6.640	6.302	5.890	6.333
BTLA	5.991	6.162	6.182	6.268	6.296	6.280
RAB18	9.771	9.857	9.809	9.788	9.678	9.913
CCPG1	9.950	10.024	9.954	9.989	10.197	10.083
RAB10	12.044	12.147	12.079	12.020	12.027	12.010
RAB13	11.043	11.060	11.192	10.961	10.928	10.915
RAB12	10.204	9.957	10.069	9.417	9.722	9.655
RAB15	9.975	9.976	10.097	9.757	9.700	9.772
RAB14	11.033	11.025	11.074	10.612	11.096	10.891
RAB17	10.309	10.388	10.550	10.165	10.225	10.236
AC080080.1	6.542	6.662	6.270	6.507	6.590	6.648
RAB1A	12.996	13.090	13.036	13.107	13.098	13.112
MIR7111	13.719	13.776	13.864	13.830	13.759	13.867
MIR7112	10.009	10.037	10.162	9.876	9.724	9.874
GPHA2	7.489	7.621	7.524	7.760	7.813	7.670
MIR4307HG	4.572	4.726	4.600	4.623	4.669	4.753
RAB5IF	13.198	13.251	13.397	13.232	13.120	13.212
KHDC4	9.221	9.222	9.312	9.460	9.447	9.489
AF117829.1	7.592	7.297	7.267	7.590	7.618	7.525
CRNDE	9.592	9.496	9.797	9.138	9.221	9.318
TNNI2	6.981	6.975	6.941	7.069	7.307	7.178
TNNI3	5.965	6.050	6.170	6.008	5.857	6.030
SUZ12	11.120	11.011	11.100	10.773	11.086	10.913
TNNI1	6.752	6.500	6.687	6.946	6.736	6.698
LMAN2L	9.887	10.070	10.171	10.259	10.086	10.215
LSM8	9.693	9.660	9.739	9.599	9.553	9.434
LSM4	10.452	10.480	10.760	10.440	10.471	10.371
LSM5	11.126	11.252	11.311	11.112	11.282	11.141
LSM6	8.867	8.815	8.894	8.647	8.842	8.706
LSM7	9.757	9.821	10.036	9.577	9.509	9.680
LSM1	11.815	11.848	11.976	12.023	11.961	11.951
LSM2	9.187	9.296	9.467	9.414	9.497	9.274

LSM3	10.036	10.140	10.292	10.115	10.054	10.144
AC008770.2	6.592	6.643	6.584	6.419	6.579	6.830
NCK1-DT	7.989	8.018	7.963	7.936	7.899	7.835
MZT1	8.967	9.021	9.257	8.928	8.972	9.101
USP48	8.919	8.960	8.977	8.994	8.762	8.865
DCBLD2	11.317	11.513	11.523	11.351	11.138	11.287
DCBLD1	8.266	8.325	8.430	8.330	8.230	8.339
AC254813.1	7.619	7.517	7.823	7.654	7.893	7.774
TPBG	11.456	11.545	11.570	11.511	11.732	11.474
SLC35G2	10.256	10.388	10.362	10.454	10.591	10.424
SLC35G3	8.133	8.291	7.986	7.939	8.331	8.161
SLC35G1	7.858	7.655	7.558	7.490	7.620	7.665
SLC35G6	8.133	8.291	7.986	7.939	8.331	8.161
SLC35G5	8.133	8.291	7.986	7.939	8.331	8.161
UBE2DNL	6.265	6.510	6.323	6.366	6.521	6.673
PRODH2	8.507	8.522	8.450	8.412	8.530	8.502
SCGB2B2	7.918	7.651	7.647	7.750	7.985	7.836
COX20	9.381	9.445	9.521	9.155	9.168	9.223
MYRF	10.858	10.866	11.037	10.771	10.785	10.726
CDKN2A-DT	6.246	6.351	6.054	6.474	6.297	6.226
SMUG1	9.790	9.866	9.912	9.718	9.698	9.755
TTTY7B	7.319	7.372	7.287	7.273	7.310	7.411
FOXA1	7.952	7.772	7.699	7.294	7.655	7.417
FOXA3	9.063	9.227	9.382	8.873	9.194	9.157
FOXA2	7.611	7.485	7.447	7.546	7.688	7.508
AC011824.2	6.589	6.747	6.594	6.647	6.806	6.921
AC011824.3	6.876	6.850	6.814	6.897	6.991	6.895
AL445931.1	5.198	5.427	5.147	5.375	5.412	5.163
ISG20L2	10.487	10.567	10.722	10.533	10.811	10.162
KIAA1671	10.791	10.731	10.797	10.621	10.507	10.634
GLB1	10.647	10.648	10.768	10.541	10.441	10.555
NUP50-DT	8.145	8.206	8.102	8.052	8.191	8.176
MIIP	9.528	9.580	9.676	9.616	9.513	9.531
RN7SL725P	6.210	6.139	6.179	6.290	6.315	6.341
PCDHB9	6.705	6.496	6.340	6.767	6.733	6.573
PCDHB8	4.458	4.756	4.695	4.828	4.558	4.659
SEPTIN1	6.174	6.272	6.465	6.527	6.330	6.165
PCDHB3	6.835	6.708	6.936	7.217	7.109	6.956
PCDHB2	6.835	6.708	6.936	7.217	7.109	6.956
PCDHB1	6.882	6.866	6.529	6.920	6.933	6.944
PCDHB7	5.444	4.985	5.348	5.698	5.059	5.357
PCDHB6	6.653	6.587	6.504	6.858	6.759	6.880
PCDHB5	4.585	4.441	4.415	4.756	4.714	4.707
PCDHB4	5.713	5.541	5.501	5.645	5.570	5.738
PITPNM1	8.856	8.879	9.009	8.818	8.761	8.717
NKX3-2	5.801	5.530	5.797	5.901	5.862	5.984
PITPNM3	7.150	7.279	7.269	6.796	6.763	6.978
PITPNM2	8.318	8.363	8.409	8.285	8.412	8.315
AP003733.3	6.450	6.327	6.280	6.126	6.457	6.282
RPAP2	9.009	8.977	9.116	9.142	9.019	9.029
RPAP3	8.575	8.702	8.703	8.547	8.538	8.682
RPAP1	9.604	9.500	9.664	9.533	9.534	9.552
TINAGL1	10.564	10.807	10.846	10.498	10.739	10.660
AC087499.2	7.700	7.562	7.647	7.527	7.430	7.471
KIAA0100	10.121	10.042	10.143	9.878	9.905	10.000
SEC23A	9.315	9.368	9.265	9.271	9.172	9.471

SEC23B	9.254	9.470	9.301	9.289	9.377	9.391
GTF2IRD2P1	6.816	6.890	7.008	6.958	6.709	6.915
EPPIN	6.736	6.638	6.465	6.624	6.835	6.713
AC233728.1	11.373	11.458	11.510	11.298	11.229	11.293
PDYN	6.407	6.381	6.447	6.702	6.744	6.560
LRTOMT	8.399	8.357	8.399	8.073	8.205	8.299
LBH	7.965	7.867	7.830	7.767	7.874	8.012
AC246793.2	5.461	5.418	5.406	5.359	5.455	5.416
AL355390.2	4.419	4.375	4.495	4.527	4.458	4.535
NEAT1	11.892	11.797	11.317	11.897	11.719	12.095
URM1	9.551	9.611	9.735	9.444	9.636	9.489
RALYL	6.361	6.425	6.188	6.322	6.524	6.329
AC139491.1	7.941	8.046	8.013	7.997	8.065	8.231
AC139491.2	6.247	6.207	6.427	6.189	6.582	6.234
AC139491.5	5.128	5.127	5.139	5.184	5.260	5.222
FGB	6.588	6.668	6.650	6.930	6.791	6.727
AC138393.1	6.311	6.208	6.614	5.780	5.917	6.267
CHRNA5	9.554	9.491	9.536	9.540	9.214	9.614
GOLGA6L11P	8.131	7.933	7.807	8.082	8.115	8.200
AC084824.6	10.634	10.730	10.709	10.742	10.670	10.762
AC084824.5	6.121	6.359	6.362	5.960	5.854	6.095
AC084824.2	10.634	10.730	10.709	10.742	10.670	10.762
MIR17HG	4.637	4.495	4.501	4.480	4.730	4.625
BX284668.5	7.978	7.875	7.780	7.680	7.972	7.899
SCAF11	9.100	9.061	9.015	9.084	9.275	8.959
OCEL1	8.610	8.660	8.812	8.713	8.661	8.628
AC026412.1	9.390	9.411	9.316	9.060	9.420	9.381
PDCD2L	9.206	9.340	9.334	9.351	9.324	9.268
SON	10.601	10.664	10.719	10.553	10.468	10.710
GAN	5.256	5.560	5.506	5.405	5.500	5.504
GAL	6.727	7.077	6.899	6.580	6.797	6.740
GAK	10.106	10.274	10.301	9.670	9.735	9.866
RFLNB	6.675	6.629	6.417	6.398	6.566	6.666
ATP1B4	5.690	5.706	5.713	5.583	5.805	5.489
GAA	9.970	10.203	10.277	9.878	9.889	9.889
SUV39H1	8.344	8.433	8.631	8.203	8.373	8.355
SUV39H2	8.613	8.617	8.701	8.609	8.803	8.425
AC087190.3	9.823	9.634	9.733	9.451	9.259	9.431
ZNF32	8.115	8.159	8.437	8.122	8.262	8.296
SLC66A1L	7.293	7.421	7.720	7.285	7.398	7.389
RGS7BP	4.211	4.153	4.418	4.511	4.358	4.280
DOP1B	7.036	7.304	7.112	7.272	7.069	7.063
DOP1A	7.465	7.465	7.692	7.359	7.379	7.448
PON1	5.795	5.938	5.859	5.998	5.794	5.969
PON3	8.228	8.283	8.258	8.052	8.549	8.185
PON2	10.968	11.005	11.027	10.902	10.900	10.833
WASHC3	10.213	10.217	10.388	10.274	10.296	10.336
WASHC1	9.341	9.202	9.345	9.278	9.301	9.395
WASHC4	10.954	11.004	11.066	10.958	11.107	10.592
WASHC5	10.097	10.148	10.150	10.005	9.994	10.004
PNMA6A	7.428	7.261	7.287	7.074	7.327	7.214
ANKRD40CL	6.098	5.724	5.622	5.741	5.767	5.655
SUMO2P1	12.617	12.628	12.692	12.617	12.655	12.614
SCO2	9.282	9.323	9.552	9.213	9.343	9.236
SCO1	8.755	8.804	8.981	8.768	8.657	8.672
UGP2	11.617	11.685	11.807	11.699	11.562	11.708

AL158066.1	6.981	6.869	7.047	6.961	6.911	6.714
AC019197.1	5.315	5.304	5.127	5.224	5.069	5.013
NDST1	8.751	8.881	8.895	8.752	8.702	8.751
NDST2	8.735	8.674	8.797	8.657	8.756	8.827
NDST3	5.155	5.186	5.284	5.447	5.282	5.278
NDST4	6.515	6.658	6.723	6.752	6.777	7.019
ZNF668	7.486	7.585	7.586	7.536	7.367	7.435
ZNF669	7.208	7.386	7.189	7.384	7.371	7.409
ZNF660	5.784	5.563	5.633	5.615	5.741	5.787
ZNF662	5.527	5.822	5.642	5.402	5.712	5.381
ZNF664	11.420	11.438	11.486	11.327	11.141	11.311
ZNF665	8.100	8.176	8.137	8.094	8.043	8.078
ZNF667	5.915	5.655	5.517	5.725	5.616	5.445
WWC2-AS2	6.882	6.767	6.745	6.959	6.915	6.680
CELA3B	7.097	7.309	7.376	7.264	7.341	7.309
CELA3A	7.095	7.173	7.241	7.098	7.183	7.167
OR1A2	5.984	5.782	5.829	6.162	6.203	6.314
OR1A1	7.244	7.054	6.728	7.232	7.360	7.252
SCOC	11.671	11.644	11.815	11.772	11.715	11.923
RPL21P28	12.617	12.677	12.802	12.784	12.769	12.841
LINC02126	6.247	6.584	6.299	6.179	6.604	6.446
AC009088.1	4.798	4.627	4.662	4.901	4.797	4.762
GFRA4	7.358	7.293	7.170	7.418	7.323	7.489
GFRA1	7.000	7.018	6.980	7.387	7.037	7.163
GFRA3	7.836	7.770	7.975	7.869	7.958	7.959
GFRA2	7.344	7.462	7.362	7.315	7.577	7.502
ADAMTS15	7.386	7.155	7.262	7.561	7.612	7.762
PRSS47	7.383	7.509	7.524	7.552	7.537	7.711
AC023157.3	7.061	7.017	6.741	6.718	6.883	6.931
RNF217-AS1	3.989	3.967	3.824	4.068	3.894	3.944
AC120498.2	7.147	7.392	7.512	7.630	7.499	7.656
FAM177A1	8.711	8.732	8.671	8.169	8.422	8.392
AP4S1	8.692	8.611	8.744	8.561	8.593	8.659
SLMAP	9.379	9.350	9.376	9.188	9.236	9.217
NBEA	8.302	8.202	8.425	8.135	8.428	8.138
LINC00645	4.070	4.213	4.113	4.308	4.531	4.440
ATG9A	9.184	9.189	9.367	9.097	9.202	9.341
ATG9B	7.055	6.916	7.054	6.975	6.850	7.146
GPR1-AS	4.928	4.818	4.790	5.532	5.057	4.990
LNX2	8.464	8.438	8.578	8.241	8.361	8.156
LNX1	7.562	7.641	7.587	7.594	7.719	7.763
IFT74	7.258	7.283	7.527	6.441	7.138	6.726
MELTF-AS1	7.798	7.751	7.745	7.908	7.829	7.910
SPTY2D1	9.427	9.476	9.375	9.224	9.287	9.314
AC004540.1	7.093	7.081	6.973	7.201	7.069	6.927
DCAF4L1	4.818	4.703	4.736	4.851	4.863	4.709
DCAF4L2	4.570	4.761	4.531	4.743	4.747	4.535
EHD4	9.776	9.906	9.873	9.871	9.783	9.717
EHD3	7.479	7.525	7.565	7.535	7.386	7.471
EHD2	11.228	11.235	11.361	11.124	11.079	11.179
EHD1	10.261	10.333	10.429	10.215	10.315	10.274
Z82186.1	6.819	6.786	6.787	6.705	6.887	6.822
ANKRD10-IT1	7.407	7.296	6.910	6.768	7.087	7.109
AC092192.1	4.328	4.422	4.117	4.375	4.321	4.435
AL135999.1	9.334	9.093	8.775	9.175	9.428	9.262
DIS3L	9.667	9.894	9.992	9.502	9.543	9.567

SNX9	10.150	10.267	10.227	10.101	10.254	9.847
SYDE2	4.142	4.265	4.358	4.209	4.267	4.282
SYDE1	9.554	9.543	9.524	9.724	9.648	9.705
RNU6-343P	9.110	8.961	9.060	9.000	9.064	9.101
GLUD2	9.727	9.810	9.967	9.578	9.631	9.543
GLUD1	10.695	10.808	10.986	10.654	10.654	10.431
LINC01828	4.136	4.131	4.146	4.558	4.281	4.488
MIR3618	7.937	7.857	7.905	7.915	7.913	7.841
PSLNR	7.031	6.937	6.926	7.183	7.137	7.205
AL445524.2	10.371	10.443	10.506	10.210	10.099	10.152
AL445524.1	8.595	8.718	8.109	8.730	8.745	8.635
AC096536.2	5.860	5.803	5.820	5.847	6.043	5.926
ISG20	10.547	10.587	10.671	10.576	10.814	10.444
LINC01821	5.780	5.874	5.770	5.851	5.948	5.912
C9orf16	11.251	11.373	11.566	11.218	11.480	11.199
UGT1A8	11.432	11.431	11.462	11.446	11.328	11.440
UGT1A9	11.432	11.431	11.462	11.446	11.328	11.440
UGT1A4	11.432	11.431	11.462	11.446	11.328	11.440
UGT1A5	11.432	11.431	11.462	11.446	11.328	11.440
AC011447.7	7.086	7.031	7.225	7.213	7.245	7.099
UGT1A7	11.432	11.431	11.462	11.446	11.328	11.440
UGT1A1	11.432	11.431	11.462	11.446	11.328	11.440
UGT1A3	11.432	11.431	11.462	11.446	11.328	11.440
SH3PXD2A-AS1	7.179	6.949	7.042	7.161	7.307	7.305
CD163L1	8.965	9.016	8.952	9.253	9.288	9.326
MCEE	8.494	8.430	8.612	8.198	8.321	8.297
MGAM2	6.683	6.757	6.675	6.854	6.863	6.759
LINC00881	6.947	7.028	7.098	7.146	7.059	7.155
LINC00880	6.373	6.366	6.096	6.111	6.164	6.278
LINC00882	5.635	5.729	5.746	6.015	5.821	5.988
LINC00884	7.403	7.147	6.871	7.303	7.335	7.273
LINC00887	4.807	5.126	4.916	4.940	4.980	5.063
LINC00886	8.549	8.452	8.504	8.516	8.436	8.395
PDIA6	12.110	12.241	12.352	12.255	12.277	12.296
PDIA5	9.225	9.267	9.471	8.993	9.084	9.038
PDIA4	10.068	10.171	10.313	10.097	10.126	10.102
PDIA3	11.425	11.585	11.685	11.539	11.387	11.497
PDIA2	7.542	7.630	7.734	8.088	7.893	7.836
AC010336.2	5.991	6.471	6.525	6.369	6.490	6.465
DACH1	5.417	5.358	5.240	5.395	5.607	5.403
DACH2	4.204	4.269	4.196	4.063	3.958	4.150
OTULINL	6.603	6.651	6.921	6.760	6.652	6.553
CD160	5.782	6.039	5.983	6.161	6.024	6.023
CD163	6.956	6.690	6.758	7.151	6.915	6.944
TTC21B	6.383	6.423	6.465	6.629	6.533	6.561
CD164	12.148	12.260	12.301	12.214	12.168	12.181
TTC21A	7.792	7.957	7.585	8.020	7.725	7.707
TSGA13	7.021	6.928	6.220	6.842	6.988	6.891
TSGA10	7.333	7.497	7.271	7.403	7.647	7.571
AC104129.1	8.016	8.137	8.049	8.008	7.815	8.006
ZFP57	7.470	7.381	7.435	7.479	7.586	7.633
SNAI3-AS1	8.083	7.667	7.672	8.155	8.121	8.150
AC018682.1	7.128	7.048	7.137	7.269	7.317	7.363
DNAJC3-DT	4.700	5.057	4.771	5.052	4.823	4.623
DCANP1	6.469	6.262	6.235	6.495	6.401	6.401
ADIPOQ	4.495	4.569	4.336	4.359	4.633	4.513

CRBN	8.852	9.066	9.225	9.062	9.279	8.879
JRKL	8.761	8.745	8.682	8.412	8.280	8.537
AC011455.2	8.927	9.033	9.010	8.781	8.753	8.701
AC011455.3	8.927	9.033	9.010	8.781	8.753	8.701
MUC2	6.879	6.951	6.615	6.830	6.825	7.041
MUC1	11.364	11.363	11.374	11.219	11.260	11.258
MUC7	4.391	4.661	4.773	5.120	4.825	4.754
MUC6	6.875	6.965	7.176	7.225	7.128	6.942
CRB3	9.290	9.342	9.484	9.256	9.175	9.241
MUC4	6.405	6.614	6.848	6.585	6.725	6.636
AL935212.1	6.728	6.572	6.787	7.070	6.998	6.812
FAM205C	7.287	7.291	7.242	7.420	7.536	7.678
FAM205A	8.654	8.825	8.721	8.924	8.885	8.798
TLNRD1	11.312	11.385	11.553	11.291	11.286	11.253
AL445645.1	6.427	6.647	6.844	6.495	6.494	6.738
NRTN	5.167	5.139	5.915	4.477	4.654	4.792
AC067838.1	7.525	7.445	7.431	6.972	7.276	7.312
TSG101	11.024	11.056	11.209	11.029	11.028	11.015
AL139247.1	5.751	5.763	5.684	5.797	5.960	5.943
LINC02109	4.892	4.990	5.174	4.946	4.963	5.090
ADRB1	5.472	5.257	5.316	5.489	5.316	5.338
SRFBP1	8.350	8.141	8.222	7.988	7.875	8.267
AC004943.2	7.858	7.918	7.975	7.726	7.815	7.839
H2AZ1	12.507	12.593	12.712	12.406	12.395	12.472
H2AZ2	11.481	11.573	11.753	11.357	11.261	11.283
ASB16-AS1	7.795	7.740	7.841	7.220	7.443	7.394
AC069444.1	4.113	4.156	4.262	4.149	4.093	4.321
DNASE1L1	9.555	9.518	9.749	9.521	9.498	9.404
DNASE1L2	8.046	7.990	8.144	7.974	7.972	7.920
DNASE1L3	7.302	7.320	7.284	7.410	7.572	7.267
AC004852.4	5.949	5.924	5.982	6.181	5.980	6.010
AL355379.2	6.235	6.431	6.355	6.640	6.411	6.457
AC004852.2	5.949	5.924	5.982	6.181	5.980	6.010
HPDL	8.420	8.473	8.552	8.163	8.267	8.085
ZEB2	6.085	6.173	6.170	6.175	6.337	6.205
CD40LG	8.297	8.248	8.287	8.347	8.263	8.326
AMD1P3	9.962	10.069	10.006	9.979	9.864	10.116
AC092813.1	6.171	6.049	5.827	5.969	6.010	6.080
ATE1	8.741	8.853	8.671	8.114	8.280	8.416
AIG1	9.806	9.853	9.964	9.730	9.708	9.772
AP000943.3	6.440	6.438	6.356	6.393	6.373	6.270
ZNF890P	7.130	7.281	7.396	7.334	7.337	7.303
ENOPH1	10.589	10.597	10.715	10.529	10.436	10.549
ERVK13-1	6.831	6.904	6.920	6.847	6.753	6.905
FGL1	7.964	8.049	8.004	7.934	8.022	7.914
FGL2	5.282	5.385	5.072	5.313	5.461	5.319
CXXC1	10.749	10.778	10.858	10.664	10.529	10.688
CXXC5	11.626	11.661	11.766	11.559	11.667	11.378
CXXC4	7.331	7.512	7.327	7.733	7.556	7.696
WTIP	7.762	7.726	7.895	7.890	7.667	7.681
CTHRC1	4.453	4.744	4.612	4.650	4.947	4.649
MAGEA9	6.533	6.531	6.239	6.507	6.605	6.747
MAGEA8	5.639	5.359	5.543	5.711	5.410	5.569
MAGEA3	6.405	6.518	6.417	6.637	6.748	6.480
MAGEA2	4.642	4.389	4.736	4.435	4.816	4.636
MAGEA1	5.345	5.524	5.391	5.336	5.494	5.421

MAGEA6	6.405	6.518	6.417	6.637	6.748	6.480
MAGEA5	6.357	6.488	6.284	6.527	6.829	6.543
MAGEA4	4.951	4.899	4.951	4.906	4.897	4.983
TEX13B	7.715	7.704	7.739	7.783	7.763	7.722
TEX13A	6.521	6.466	6.310	6.731	6.774	6.676
STN1	9.127	9.240	9.402	8.965	8.949	9.035
Z99496.1	6.436	6.506	6.467	6.925	6.761	6.652
AC005072.1	4.420	4.583	4.431	4.581	4.609	4.656
HOMER1	9.512	9.323	9.254	8.818	8.783	9.531
FIP1L1	8.029	7.867	7.971	7.581	7.388	7.585
HOMER3	8.582	8.700	8.752	8.573	8.511	8.425
HOMER2	9.428	9.451	9.651	9.581	9.689	9.382
PCGF7P	7.158	7.238	7.118	6.970	7.089	7.100
FBXW4P1	6.584	6.824	6.910	6.953	6.752	6.737
MIR765	8.819	8.938	8.986	8.740	8.855	8.806
THAP1	8.050	8.091	8.215	7.863	7.999	7.973
THAP2	5.424	5.522	5.381	5.296	5.375	5.374
SNW1	10.621	10.719	10.836	10.670	10.580	10.522
THAP4	9.435	9.460	9.537	9.449	9.299	9.343
THAP5	7.179	7.075	7.136	6.902	6.799	7.024
THAP6	7.859	7.827	7.791	7.622	8.173	7.338
THAP7	8.703	8.716	8.890	8.604	8.513	8.753
THAP8	7.777	7.948	7.966	7.740	7.848	7.974
THAP9	6.642	6.814	7.010	6.406	6.474	6.497
MNX1-AS1	6.814	6.952	7.176	6.882	7.006	6.909
LINC00415	6.169	6.163	6.165	6.166	5.964	6.468
LINC00410	6.811	6.640	6.391	6.668	6.802	6.522
SYBU	7.754	7.986	7.917	7.495	7.442	7.542
TRIM11	9.445	9.574	9.653	9.481	9.605	9.433
TRIM10	7.203	7.363	7.285	7.361	7.404	7.354
TRIM13	8.624	8.728	8.843	8.769	8.729	8.649
TRIM15	8.599	8.597	8.715	8.687	8.601	8.602
TRIM14	9.822	9.612	9.512	9.324	9.882	9.264
TRIM17	7.089	6.973	7.073	7.198	7.173	7.036
TRIM16	8.796	8.798	8.807	8.359	8.555	8.964
CCRL2	7.698	7.705	7.533	7.629	7.779	7.758
MIR492	7.038	7.238	7.469	7.419	6.940	7.172
RCBTB2	5.772	5.858	6.243	5.672	5.808	5.812
MIR497	7.403	7.147	7.087	7.225	7.569	7.440
AC024592.2	6.968	7.104	6.979	6.965	7.075	6.999
AC024592.3	10.505	10.518	10.723	10.566	10.544	10.629
AC091304.1	8.692	8.361	8.050	8.620	8.552	8.728
ZNF587B	8.690	8.624	8.631	8.439	8.622	8.630
AL137224.1	6.523	6.443	6.348	6.606	6.582	6.541
AL353747.1	5.728	5.713	5.558	5.752	5.917	5.735
EYA4	8.488	8.498	8.498	8.367	8.388	8.339
KHNYN	10.013	10.155	10.107	9.868	9.983	9.852
FTOP1	5.090	5.164	5.336	5.092	5.272	5.187
PGAM4	12.759	12.888	13.016	12.823	12.758	12.866
ZNF174	7.980	7.943	8.096	8.238	8.167	8.161
FABP5	10.336	10.387	10.611	10.215	10.289	10.177
ZNF175	8.411	8.418	8.618	8.346	8.062	8.445
FABP2	6.873	6.836	6.997	7.202	7.057	6.892
UQCRFS1	12.728	12.710	12.792	12.777	12.608	12.707
ZNF177	6.191	6.246	6.127	5.971	6.292	6.021
AC093591.1	12.617	12.677	12.802	12.784	12.769	12.841

FABP1	4.211	4.234	4.069	4.445	4.506	4.316
GABRA5	6.738	6.700	6.546	6.706	6.571	6.797
Z94721.2	5.431	5.263	5.264	5.594	5.534	5.502
SNRPEP4	11.739	11.912	12.050	11.783	11.753	11.727
SNRPEP2	11.739	11.912	12.050	11.783	11.753	11.727
GABRA3	5.872	5.790	5.752	6.132	6.067	6.327
PPP1R9A	8.234	8.162	8.284	7.796	7.997	8.038
PPP1R9B	10.058	10.123	10.225	10.073	10.213	10.097
BVES	6.700	6.473	6.511	6.828	6.621	6.626
AC079148.1	3.870	4.099	3.707	3.890	3.782	3.862
SCMH1	8.529	8.619	8.451	8.305	8.424	8.459
EPS8L1	8.903	8.970	9.165	8.767	8.836	8.831
EPS8L2	11.108	11.163	11.366	11.110	10.999	11.099
EPS8L3	9.944	10.081	10.105	9.783	9.777	9.751
DEPP1	7.982	8.161	8.274	8.020	8.143	8.196
BTD	7.932	8.212	8.146	8.110	7.982	8.121
BTC	8.051	8.026	8.026	7.396	7.685	7.753
NXPH3	8.507	8.402	8.518	8.519	8.652	8.641
NXPH2	6.355	6.556	6.359	6.716	6.394	6.661
NXPH1	4.099	4.183	4.125	4.257	4.148	4.214
BTK	7.447	7.480	7.772	8.040	7.688	7.636
NXPH4	7.943	7.859	7.991	8.141	8.027	7.987
APCDD1L-DT	9.203	9.258	9.084	9.214	9.291	9.268
INTS6L	7.007	6.779	6.917	6.860	6.795	7.086
AL096711.2	6.707	6.826	6.941	7.042	6.836	7.004
PRELP	8.580	8.671	8.785	8.569	8.854	8.718
TNFRSF6B	7.806	7.971	7.815	7.626	7.816	7.993
DPAGT1	9.375	9.456	9.540	9.289	9.268	9.375
ATXN2L	8.642	8.631	8.938	8.993	9.052	8.706
YOD1	8.077	8.115	8.033	8.019	7.965	8.244
LEXM	5.851	6.053	5.953	6.044	6.025	6.145
IGKV1OR9-1	8.398	8.520	8.424	8.478	8.571	8.522
IGKV1OR9-2	8.398	8.520	8.424	8.478	8.571	8.522
AC092125.1	5.535	5.671	5.466	5.773	5.786	5.570
RESF1	9.452	9.413	9.345	9.155	9.353	9.337
CROCC	7.453	7.302	7.421	7.863	7.677	7.785
NDUFB2-AS1	6.664	6.707	6.770	6.735	6.904	6.757
AC110491.1	4.865	4.659	4.479	4.969	4.780	4.854
APOE	8.129	8.257	8.502	8.286	8.249	8.255
LACC1	6.929	6.833	6.842	6.504	6.472	6.514
FGD5-AS1	10.428	10.436	10.471	10.170	10.171	10.219
KATNB1	9.231	9.256	9.417	9.069	9.143	8.930
SCGB3A1	8.041	8.016	7.928	8.046	8.042	7.940
SCGB3A2	6.703	6.834	6.548	6.924	6.938	6.956
AC040162.1	9.803	9.864	10.151	9.775	9.690	9.589
AC040162.3	9.079	9.178	9.367	8.895	8.914	8.951
AL136102.1	5.362	5.321	5.474	5.519	5.722	5.487
ERV3-1	4.438	4.451	4.371	4.568	4.427	4.580
CBS	10.172	10.258	10.319	9.943	9.869	9.923
TSC22D1-AS1	6.517	6.639	6.630	6.846	6.458	6.740
TXNDC9	10.272	10.462	10.461	10.236	10.183	10.266
TXNDC8	6.432	6.618	6.698	6.810	6.673	6.716
TXNDC2	7.703	7.804	7.722	7.749	7.929	7.791
TXNDC5	11.661	11.750	11.864	11.797	11.740	11.568
FXYD3	12.242	12.299	12.453	12.393	12.433	12.434
AC243824.1	7.256	7.004	7.129	7.152	6.993	7.140

C1orf220	4.358	4.295	4.139	4.444	4.337	4.324
PRMT8	7.731	7.538	7.619	7.676	7.703	7.587
PRMT9	8.443	8.350	8.446	8.289	8.215	8.313
C1orf226	7.800	7.977	7.793	7.592	7.490	7.822
PRMT2	9.811	9.872	10.077	9.752	9.802	9.873
PRMT3	8.151	8.133	8.296	8.039	8.097	8.104
PRMT1	11.938	12.046	12.222	11.984	11.820	11.901
PRMT6	9.011	9.080	9.078	8.649	8.770	8.779
PRMT7	7.622	7.651	7.811	7.484	7.413	7.589
PRMT5	10.885	11.010	11.157	10.837	10.753	10.848
TMEM176A	7.915	7.926	7.954	7.931	8.264	7.877
HMGCR	11.084	11.182	11.326	11.161	11.027	11.208
TMEM176B	6.366	6.476	6.377	6.332	6.193	6.439
AC083805.1	7.544	7.637	7.577	7.663	7.789	7.823
AC083805.3	8.093	8.114	8.313	8.454	8.226	8.355
LYPD6B	7.228	7.210	7.167	7.129	7.280	7.137
REG3A	6.098	6.228	6.500	6.530	6.576	6.524
PMS2CL	9.485	9.466	9.613	9.501	9.346	9.296
AC111170.4	6.032	6.142	6.048	6.037	6.155	6.009
CD209	7.616	7.581	7.551	7.592	7.777	7.570
CD200	6.495	6.417	6.371	6.240	6.346	6.446
AL031708.1	9.288	9.497	9.493	9.344	9.321	9.251
AL121757.1	7.205	7.024	7.184	7.322	7.586	7.400
CD207	6.924	7.303	6.996	6.970	7.051	7.060
AC024940.7	6.659	6.528	6.564	6.204	6.254	6.374
AC024940.6	10.864	11.025	11.133	11.044	10.944	10.892
AC024940.3	14.076	14.099	14.141	14.125	14.125	14.139
AC024940.2	6.659	6.528	6.564	6.204	6.254	6.374
AC024940.1	6.528	6.650	6.671	6.810	6.644	6.427
PPARGC1A	6.619	6.983	6.828	6.587	6.344	6.685
PPARGC1B	6.981	7.176	7.077	7.114	7.325	7.099
AC024940.8	6.528	6.650	6.671	6.810	6.644	6.427
CX3CL1	7.890	8.051	8.193	8.123	8.274	7.988
RPSAP26	13.432	13.517	13.661	13.465	13.519	13.574
UMPS	9.977	10.026	10.122	9.770	9.851	9.726
RPSAP20	9.071	9.010	9.096	9.186	9.212	9.311
AL035456.1	10.297	10.231	10.351	9.893	10.088	10.088
NANOS1	7.630	7.671	7.796	7.381	7.492	7.528
NANOS3	6.921	7.351	7.209	7.055	7.051	7.165
NANOS2	6.421	6.521	6.396	6.656	6.543	6.417
CYP2E1	6.778	6.675	6.680	6.746	6.383	6.712
MIR3681HG	7.537	7.630	7.633	7.804	7.711	7.841
DHDH	7.095	7.042	7.396	7.265	7.043	7.070
DHX58	7.563	7.667	7.838	7.731	7.613	7.763
DHX57	7.758	7.794	7.917	7.940	7.841	7.898
PPBP	5.155	5.343	5.201	5.494	5.548	5.429
AC233699.8	10.910	10.920	10.896	10.979	11.199	10.847
AC233699.1	10.910	10.920	10.896	10.979	11.199	10.847
DDIT4L	6.226	6.345	6.251	6.037	6.175	6.265
FO624990.1	4.897	4.802	4.795	4.775	4.928	4.743
DEFT1P2	5.904	6.100	6.027	5.989	5.957	6.028
KIAA2012-AS1	5.807	5.865	5.691	5.844	6.151	5.847
CYP4F22	5.618	5.796	5.786	6.082	5.656	6.076
NUP50	10.480	10.405	10.474	10.341	10.128	10.386
NUP54	9.253	9.196	9.426	9.139	9.243	9.211
NUP58	7.941	7.877	8.020	7.763	8.205	7.945

SCARNA13	5.644	5.654	5.171	5.724	5.538	5.606
SCARNA17	6.271	6.333	6.470	6.193	6.295	6.507
SCARNA15	7.259	7.010	6.981	6.784	6.928	7.209
TMEM74B	6.917	6.910	6.887	6.954	6.972	7.004
GCDH	8.566	8.659	8.662	8.513	8.543	8.662
PRICKLE1	8.498	8.613	8.802	8.541	8.839	8.621
PRICKLE2	7.134	7.129	7.049	7.056	7.216	7.215
PRICKLE3	7.790	7.762	7.921	7.460	7.942	7.902
CCNJL	8.236	8.347	8.541	8.240	8.367	8.282
PCYOX1	10.424	10.353	10.416	10.153	10.178	10.308
MKRN7P	6.403	6.534	6.209	6.534	6.579	6.664
SLC16A14	8.577	8.587	8.694	8.250	8.430	8.446
SLC16A11	7.525	7.553	7.400	7.523	7.547	7.679
CKAP4	11.105	11.168	11.419	10.881	11.077	10.924
SPINK7	5.664	5.711	5.598	5.921	5.704	5.500
SPINK6	6.492	6.613	6.419	6.443	6.680	6.648
SPINK5	8.445	8.419	8.406	8.477	8.591	8.421
SPINK4	7.573	7.451	7.563	7.678	7.897	7.640
SPINK2	6.934	7.202	7.071	7.225	7.301	7.089
SPINK1	12.713	12.860	12.966	12.952	12.714	12.904
AL450998.2	7.349	7.295	7.253	7.019	6.973	7.226
AC135983.3	3.879	3.648	3.709	3.998	3.568	3.769
RSPH6A	7.731	7.754	7.819	8.038	7.946	8.006
HAVCR1	8.373	8.331	8.357	7.785	7.910	7.859
HAVCR2	7.793	7.884	7.868	7.922	8.049	8.026
DGKZP1	9.639	9.685	9.727	9.532	9.470	9.445
AC004692.1	7.824	7.955	8.142	7.986	8.038	8.031
AC087477.2	7.602	7.564	7.754	7.401	7.417	7.170
DMGDH	7.005	6.931	6.816	6.908	6.762	6.957
CREBL2	10.012	10.116	10.200	9.776	9.873	9.761
PLIN4	7.500	7.495	7.583	7.571	7.465	7.631
PLIN5	8.040	8.120	8.052	8.058	7.988	8.026
PLIN2	6.003	5.935	5.944	5.946	6.305	5.920
PLIN3	10.374	10.177	10.402	10.220	10.197	10.143
PLIN1	8.279	8.053	8.476	8.283	8.316	8.321
SMAP1	10.399	10.399	10.403	10.238	10.351	10.395
SMAP2	8.683	8.837	8.765	8.677	8.622	8.762
CYP2A13	7.555	7.498	7.691	8.072	8.012	7.830
ARPC1B	11.370	11.567	11.714	11.344	11.442	11.100
ARPC1A	11.652	11.754	11.835	11.721	11.540	11.689
EGFL7	8.450	8.383	8.574	8.667	8.781	8.457
EGFL6	6.346	6.181	6.213	6.307	6.295	6.263
ACSL3	11.341	11.415	11.397	11.184	11.229	11.251
CYTIP	4.919	4.770	4.951	5.230	4.813	4.957
EGFL8	7.787	7.777	7.897	8.212	8.057	7.746
HTR5A	7.895	7.714	7.562	7.784	7.772	7.831
ACSL1	9.918	10.009	10.116	9.913	9.826	9.968
TMEM120B	8.777	8.910	8.900	8.798	8.870	8.880
TMEM120A	9.027	9.029	9.215	8.866	8.867	8.893
TRMT11	8.580	8.534	8.648	8.350	8.310	8.266
THAP12	10.805	10.879	10.978	10.843	10.868	10.791
KREMEN2	8.403	8.355	8.346	8.580	8.431	8.518
KREMEN1	7.709	7.361	7.471	7.540	7.748	7.654
FILNC1	4.444	4.505	4.338	4.667	4.694	4.522
NEK10	7.320	7.266	7.399	7.562	7.322	6.981
NEK11	8.628	8.707	8.741	8.509	8.524	8.687

HMGB1P6	13.218	13.387	13.396	13.059	12.997	12.987
MNT	9.314	9.389	9.408	9.019	9.050	9.016
ETS1-AS1	5.288	5.025	5.248	5.181	5.183	5.236
IGF2-AS	7.668	7.371	7.558	8.154	7.902	7.814
TRMT12	8.584	8.566	8.660	8.375	8.649	8.594
AC234582.2	9.177	9.195	9.333	9.033	9.013	9.060
AC234582.1	5.834	5.774	5.583	5.758	5.595	5.888
IL22RA2	5.855	5.921	6.043	5.855	5.935	5.871
IL22RA1	7.455	7.513	7.585	7.236	7.339	7.443
SPSB4	7.167	7.375	7.453	7.477	7.566	7.328
FLYWCH2	8.130	8.074	8.222	8.166	8.149	7.986
FLYWCH1	8.904	8.958	8.928	8.730	8.808	8.991
MN1	6.837	6.667	6.702	6.625	6.623	6.584
CR381670.1	8.236	8.156	8.141	8.146	8.143	8.231
ANKRD27	9.324	9.334	9.507	9.267	9.259	9.221
ANKRD26	6.942	6.872	6.716	6.926	6.834	6.913
LINC00928	6.811	6.700	6.811	6.737	6.739	6.854
LINC00929	7.085	7.143	6.987	7.248	7.531	7.421
ANKRD23	8.385	8.486	8.484	8.239	8.358	8.221
ANKRD22	10.325	10.390	10.437	10.500	10.803	10.472
LINC00922	7.015	7.048	7.032	6.955	6.932	7.141
LINC00923	5.688	5.372	5.553	5.601	5.656	5.689
PTGES	8.243	8.319	8.150	8.104	8.198	8.295
LINC00921	6.480	6.325	6.243	6.640	6.388	6.463
LINC00926	8.139	7.962	7.996	8.267	8.307	8.136
LINC00927	6.411	6.600	6.536	6.813	6.586	6.557
PBK	9.523	9.544	9.671	9.135	9.247	9.161
ANKRD28	9.570	9.658	9.606	9.352	9.483	9.368
FCGR1CP	6.966	6.873	7.018	6.966	6.957	6.852
TRIM33	9.852	10.139	10.239	10.223	10.175	9.913
AC007681.1	3.974	3.874	3.824	3.907	3.993	4.077
TRIM31	9.187	9.225	9.180	9.127	9.095	9.049
OR2A7	7.717	7.900	7.748	7.796	7.697	7.761
AL139393.3	7.530	7.571	7.457	7.008	7.072	7.045
MLXIP	10.092	10.220	10.302	10.059	10.184	10.044
TRIM35	8.288	8.327	8.423	8.334	8.259	8.330
ADA	8.244	8.331	8.394	8.218	8.374	8.356
ADO	9.922	9.994	10.013	9.833	9.777	9.834
ADM	9.703	9.738	9.963	9.834	9.741	9.903
ADK	10.260	10.327	10.459	10.383	10.227	10.382
VIPAS39	8.785	8.905	9.055	8.834	8.893	8.909
AC105942.1	7.071	7.218	7.281	7.398	7.289	7.432
H3P6	13.752	13.863	13.907	13.791	13.836	13.535
KALRN	8.147	8.208	7.999	8.002	8.106	7.994
JUNB	10.000	10.021	10.220	10.473	10.483	10.244
JUND	11.552	11.562	11.688	11.259	11.081	11.309
H1FX-AS1	7.813	7.881	7.872	7.782	7.947	7.814
AC099668.1	8.186	8.305	8.122	8.250	8.252	8.202
CENPS-CORT	7.912	7.605	7.853	7.960	7.963	8.026
NDUFA6-DT	6.854	7.018	7.068	6.702	6.831	6.818
AL391095.5	9.596	9.562	9.719	9.469	9.487	9.569
AL137798.2	6.719	6.805	6.764	6.834	6.732	6.855
CCDC81	6.074	6.095	6.002	6.067	6.103	5.912
AC055839.2	6.427	6.359	6.105	6.366	6.559	6.426
AC025449.1	5.482	5.293	5.014	4.582	4.726	5.066
RSU1	9.401	9.471	9.479	9.260	9.383	9.385

MOV10	8.611	8.637	8.726	8.357	8.599	8.329
MOCS3	8.758	8.864	8.958	8.498	8.695	8.834
MOCS2	8.718	8.675	8.596	8.724	8.799	8.892
RNU1-70P	4.069	4.222	4.371	4.369	4.292	4.177
UBE2D3-AS1	7.540	7.588	7.528	7.520	7.631	7.600
AC005522.1	5.936	5.995	5.635	6.292	5.884	6.001
UCKL1	9.434	9.319	9.542	9.040	9.275	9.204
LINC01644	6.627	6.682	6.410	6.484	6.444	6.552
ATP5MC1	10.530	10.643	10.919	10.668	10.698	10.623
LINC01648	7.809	7.909	7.730	7.878	7.861	7.903
LINC01649	4.758	4.696	4.417	4.691	4.811	4.718
CD1D	5.499	5.729	5.592	5.607	5.391	5.804
CD1E	7.590	7.386	7.557	7.912	7.799	7.550
CD1B	7.194	7.448	7.105	7.618	7.633	7.650
CD1C	7.333	7.575	7.371	7.417	7.543	7.770
CD1A	7.755	7.508	7.582	7.948	7.931	8.010
CELF6	6.339	6.579	6.391	6.608	6.486	6.659
CELF5	6.487	6.421	6.520	6.257	6.499	6.416
CELF4	7.909	7.804	7.885	7.986	7.968	7.987
CELF3	8.669	8.588	8.726	8.841	8.745	8.647
CELF2	7.249	7.266	7.276	7.490	7.300	7.304
CELF1	11.380	11.272	11.364	11.181	11.046	11.030
TANGO6	8.059	8.195	8.332	8.031	8.038	8.205
TANGO2	9.468	9.605	9.700	9.263	9.327	9.371
SPATA31A1	6.077	6.174	6.080	6.325	6.400	6.411
AL356130.1	5.976	6.268	6.126	6.034	6.352	6.164
SPATA31A3	6.077	6.174	6.080	6.325	6.400	6.411
AC027612.3	8.975	8.923	9.166	9.006	8.941	8.856
SPATA31A5	6.077	6.174	6.080	6.325	6.400	6.411
SPATA31A7	6.077	6.174	6.080	6.325	6.400	6.411
AL512306.3	8.025	7.884	7.958	8.019	8.266	8.320
AL163195.2	7.098	7.106	6.964	7.266	7.247	7.114
AL163195.3	5.025	4.965	5.201	5.131	5.159	4.559
HAGLR	7.925	7.845	7.774	7.804	8.004	7.946
OR52A1	7.099	7.186	6.960	7.202	7.122	7.421
ASPDH	7.787	7.888	8.158	8.141	8.151	8.054
PPARG	9.568	9.638	9.836	9.630	9.602	9.672
PPARD	8.342	8.452	8.542	8.374	8.450	8.446
PPARA	8.615	8.754	8.772	8.697	8.716	8.729
SPAM1	5.713	5.800	5.743	6.003	6.184	5.988
CD14	6.762	6.728	7.101	6.875	6.938	6.683
BTN1A1	6.274	6.287	6.132	5.964	6.168	6.069
PARTICL	6.989	6.850	6.892	7.121	6.936	6.911
CD19	7.195	7.361	7.242	7.326	7.336	7.297
MORC1	4.699	4.446	4.630	4.635	4.612	4.863
AC010442.1	7.989	8.093	8.179	7.801	7.759	7.856
ENPEP	4.936	4.870	5.092	5.066	4.930	5.233
OR4F1P	7.745	7.729	7.361	7.729	7.847	7.903
PTX3	4.894	4.771	5.068	7.032	7.149	6.988
SEZ6	8.174	8.280	8.186	8.393	8.313	8.235
DDB1	10.809	10.890	11.013	10.713	10.631	10.718
OR4F16	7.745	7.729	7.361	7.729	7.847	7.903
AL121933.2	4.733	4.644	4.646	4.927	4.780	4.700
SMAD1-AS2	5.000	5.164	5.219	5.499	5.340	5.235
U72788.1	4.526	4.772	4.535	4.904	4.748	4.703
AL031276.1	6.896	6.975	6.911	6.984	6.909	6.781

PLCG2	7.938	7.857	7.824	8.058	8.032	8.079
PLCG1	8.851	9.052	9.089	9.112	8.908	9.014
BSND	7.900	8.066	8.094	8.067	8.105	7.958
ZNF436-AS1	7.117	7.171	7.221	7.374	7.405	7.279
N4BP1	11.270	11.425	11.401	11.300	11.443	11.406
N4BP3	6.845	6.743	6.568	6.957	6.692	6.751
N4BP2	5.565	5.595	5.478	4.708	4.951	4.966
HEATR6	8.027	7.945	7.881	7.846	7.745	8.068
HEATR4	6.172	5.812	6.194	6.115	6.176	6.348
HEATR3	8.104	8.071	7.960	7.660	7.711	8.017
HEATR1	9.506	9.571	9.592	9.521	9.497	9.536
HEATR9	5.449	5.719	5.261	5.737	5.559	5.624
GANC	7.901	7.881	7.742	7.866	7.965	8.093
AP001604.1	7.887	7.981	7.896	7.986	8.031	8.220
DEDD	9.810	9.850	10.018	9.735	9.804	9.737
KLRK1	7.160	7.040	7.142	7.546	7.298	7.477
AP001148.1	5.785	5.775	5.668	5.831	5.751	5.918
FUCA1	10.282	10.301	10.381	10.232	10.089	10.164
FUCA2	11.041	11.099	11.216	10.774	10.733	10.833
SYCP2L	5.528	5.739	5.676	5.746	5.945	5.884
IKBKB	9.038	9.056	9.140	8.936	8.752	9.008
IKBKG	10.308	10.254	10.278	10.317	10.236	10.286
FCGR1A	6.966	6.873	7.018	6.966	6.957	6.852
IKBKE	8.167	8.258	8.147	8.199	8.139	8.108
ENTHD1	4.366	4.282	4.396	4.356	4.405	4.257
GNL3	9.631	9.466	9.584	9.474	9.416	9.594
GNL2	8.990	8.954	9.080	8.678	8.620	8.684
GNL1	8.588	8.487	8.632	8.739	8.555	8.563
AL844175.1	4.602	4.595	4.534	4.592	4.656	4.744
AP001636.3	8.158	8.168	7.990	8.000	8.175	8.188
AL671681.1	7.344	7.403	7.456	7.273	7.478	7.510
DYRK1A	9.479	9.383	9.521	9.360	9.203	9.435
DYRK1B	8.585	8.546	8.568	8.503	8.729	8.387
LINC01775	7.137	7.249	7.219	7.153	7.072	7.306
GNLY	6.163	6.297	6.260	6.507	6.278	6.292
SMARCAL1	8.402	8.496	8.560	8.381	8.486	8.328
IMPA1	9.309	9.358	9.457	9.176	9.282	9.321
IMPA2	9.441	9.529	9.719	9.196	9.186	9.218
AL355001.2	6.811	6.820	6.996	6.822	6.626	6.855
KITLG	9.119	9.046	9.159	9.092	9.050	9.236
AC135048.1	10.165	10.171	10.275	10.024	10.034	9.994
RSPO4	7.838	7.790	8.042	8.195	8.010	8.151
RPS26P28	12.821	12.916	13.024	12.923	12.903	12.978
DIXDC1	7.886	7.949	7.952	7.827	7.791	7.830
PLAGL2	8.817	8.878	8.937	8.767	8.763	8.860
MAPK1	11.775	11.735	11.763	11.595	11.621	11.487
PLAGL1	9.093	9.089	9.198	8.834	9.141	8.608
FAM24A	5.239	5.314	5.352	5.172	5.352	5.341
FAM24B	6.561	6.686	6.558	6.613	6.480	6.549
VCP	11.504	11.634	11.756	11.494	11.289	11.331
CBWD6	11.277	11.323	11.404	11.049	11.073	11.051
METRNL	9.167	9.255	9.433	8.927	9.057	8.736
IGFL2	7.003	6.961	6.611	7.025	7.149	7.128
IGFL1	7.240	7.326	7.281	7.430	7.386	7.415
MTDH	10.872	10.642	10.823	10.845	10.825	10.736
CBWD2	11.277	11.323	11.404	11.049	11.073	11.051

MADD	8.894	8.953	9.154	8.741	8.886	8.876
CBWD1	11.277	11.323	11.404	11.049	11.073	11.051
EXO1	8.332	8.302	8.466	8.254	8.291	8.370
EXO5	7.802	7.755	7.809	7.656	7.685	7.702
LRRTM4	3.813	3.736	3.531	3.591	3.763	3.840
LRRTM1	7.811	7.762	7.711	7.763	7.785	7.764
LRRTM2	5.718	5.447	5.415	5.537	5.730	5.752
ZNF277	8.185	8.425	8.438	8.292	8.458	8.191
SLC46A2	5.612	5.685	5.665	5.912	5.558	5.734
SLC46A3	7.836	7.861	7.626	7.979	7.775	7.739
SLC46A1	9.049	9.174	9.116	9.164	9.236	9.218
AP000350.12	13.777	13.817	13.967	13.853	13.826	13.849
AC077690.1	5.064	5.337	5.293	5.281	5.167	5.077
AGMAT	8.055	8.070	7.938	7.448	7.789	7.634
RFK	9.000	8.994	8.983	8.855	8.799	8.851
EXOG	8.145	8.066	7.886	8.092	7.999	8.064
EPAS1	10.210	10.224	10.266	10.175	9.851	10.057
AP006545.1	7.180	7.213	7.089	6.940	7.274	7.313
SERPINA12	6.679	6.690	6.618	6.577	6.920	6.683
SERPINA10	7.000	6.911	6.722	7.199	6.951	7.044
PRKCA-AS1	4.625	4.726	4.583	4.704	4.715	4.890
ADHFE1	8.718	8.636	8.735	8.681	8.791	8.766
PWAR5	4.006	4.018	3.855	3.967	4.078	4.011
AC025283.2	7.980	7.943	8.096	8.238	8.167	8.161
AFG1L	5.407	5.270	5.529	5.455	5.360	5.169
RPL7P32	13.823	13.851	13.913	13.938	13.843	13.938
SOX21	6.883	6.848	7.191	7.101	7.293	7.261
ATP2A1-AS1	7.278	7.233	7.306	7.182	7.200	7.291
RCL1	8.910	9.034	9.189	8.940	8.896	8.872
FIGNL1	9.758	9.668	9.749	9.326	9.216	9.371
CLU	10.677	10.735	10.925	10.757	10.814	10.737
CHAMP1	8.358	8.444	8.499	8.316	8.234	8.336
KNG1	6.671	6.728	6.732	6.792	6.684	6.901
COBL	10.516	10.394	10.530	10.393	10.291	10.393
PNPT1	10.552	10.535	10.567	10.705	10.761	10.444
ALOX15	6.101	5.992	6.124	6.118	6.286	6.111
ALOX12	7.294	7.133	7.288	7.236	7.405	7.420
SEMA6D	7.540	7.648	7.430	7.694	7.634	7.642
SEMA6B	8.503	8.433	8.563	8.641	8.526	8.674
SEMA6C	8.500	8.457	8.417	8.678	8.659	8.550
SEMA6A	9.257	9.245	9.454	9.117	9.456	9.031
BRWD1-AS2	6.739	6.736	6.599	6.613	6.701	6.669
ALKBH4	7.826	7.934	8.055	8.105	7.915	7.874
ADCK5	8.256	8.201	8.406	8.165	8.037	8.235
ADCK2	8.547	8.650	8.752	8.476	8.479	8.491
AC138866.1	5.859	5.735	5.446	5.456	5.312	5.507
SCN2B	7.319	7.204	7.392	7.547	7.471	7.430
SCN2A	6.432	6.223	6.205	6.515	6.654	6.579
PTGER4P3	6.925	7.126	7.107	7.121	7.103	7.270
ASF1A	8.681	8.821	8.867	8.822	8.768	8.925
ASF1B	10.529	10.395	10.734	10.139	10.247	10.255
LMAN1	12.452	12.519	12.544	12.514	12.497	12.478
LMAN2	10.315	10.576	10.646	10.305	10.273	10.374
SH3BGR	7.311	7.468	7.381	7.540	7.568	7.613
RNU2-52P	7.157	7.530	7.385	7.443	7.426	7.591
ACTG1	14.253	14.283	14.243	14.190	14.241	14.265

ACTG2	7.082	7.183	7.106	7.168	7.288	7.208
ZDBF2	4.907	4.786	4.726	4.804	4.654	4.715
AC253572.1	9.707	9.662	9.784	9.264	9.334	9.270
AC156455.2	6.980	7.205	7.021	7.011	7.061	7.078
PRPF39	7.364	7.114	7.097	6.818	6.891	7.228
AC156455.1	6.980	7.205	7.021	7.011	7.061	7.078
PRPF31	10.357	10.410	10.540	10.053	10.003	10.044
SPOPL	9.244	9.183	9.260	8.971	8.849	9.025
AC018695.3	6.878	7.125	6.796	6.965	7.029	7.125
EDIL3-DT	6.665	6.733	6.680	6.889	6.814	7.074
SNORA28	6.969	7.119	7.018	7.070	7.057	6.698
ZNF207	11.569	11.609	11.690	11.525	11.445	11.573
RPS27AP16	13.293	13.335	13.375	13.366	13.329	13.396
ZNF202	7.512	7.653	7.536	7.627	7.625	7.880
DLG1-AS1	5.449	5.479	5.508	5.499	5.531	5.524
IDH2	11.967	12.009	12.230	11.715	11.602	11.564
IDH1	13.108	13.186	13.191	13.192	13.124	13.204
ZNF208	5.121	5.271	5.106	5.165	4.915	5.092
PASK	7.656	7.860	7.637	7.540	7.667	7.836
XAF1	8.850	8.623	8.476	8.578	9.167	8.392
AC007731.2	8.391	8.397	8.395	8.236	8.358	8.212
DUX4	8.052	7.820	8.258	8.487	8.319	8.298
PYCR3	9.042	9.164	9.262	8.803	9.010	8.995
GATD3A	10.399	10.354	10.596	10.369	10.330	10.402
TRAV12-3	4.154	4.311	4.314	4.156	3.989	4.263
SNORD118	6.181	6.396	6.348	5.653	6.072	6.056
GLYR1	10.444	10.614	10.645	10.562	10.537	10.637
TRAV12-2	4.974	4.899	4.920	4.844	4.959	4.848
PTOV1-AS2	8.171	8.350	8.453	8.728	8.513	8.419
PTOV1-AS1	6.837	6.719	6.857	7.021	6.791	6.918
NPPB	6.614	6.625	6.510	6.614	6.515	6.625
NPPC	6.017	6.139	6.132	6.184	6.409	6.171
NPPA	8.335	8.169	8.183	8.477	8.252	8.246
AC018904.2	7.089	7.081	7.157	7.348	7.387	7.221
RPS3AP47	13.488	13.531	13.648	13.817	13.801	13.836
AC005632.3	6.265	6.259	6.073	5.990	5.906	6.037
AL139407.1	4.500	4.514	4.574	4.516	4.504	4.432
DNER	5.773	5.934	6.030	5.995	5.539	5.812
LINC01339	7.837	8.319	7.989	8.320	8.137	8.110
PALB2	8.773	8.866	9.014	8.596	8.525	8.629
LINC01333	6.029	6.112	5.890	5.804	6.140	6.069
AC090241.3	4.281	4.311	4.386	4.329	4.302	4.412
TSPY7P	4.547	4.525	4.638	4.840	4.849	4.586
RPS15A	13.687	13.656	13.782	13.960	13.843	13.987
AL109811.3	8.308	8.102	8.208	8.000	8.026	8.095
AL109811.2	6.303	6.453	6.403	6.647	6.533	6.524
NSMAF	9.182	9.006	9.035	9.032	9.016	8.974
SIK1	8.518	8.535	8.471	8.560	8.466	8.528
SIK3	8.389	8.410	8.450	7.994	8.182	8.221
SIK2	7.955	7.903	7.823	7.836	7.900	7.830
CST9L	5.456	5.425	5.348	5.465	5.461	5.401
DUS3L	7.867	7.812	8.052	7.662	7.686	7.605
AL360178.1	5.040	4.832	4.970	5.499	5.197	5.004
AC027808.2	6.763	6.981	6.557	6.763	6.682	6.873
AC027808.1	6.763	6.981	6.557	6.763	6.682	6.873
RAB9A	10.937	10.978	11.128	10.986	10.892	11.031

RAB9B	7.433	7.461	7.418	7.405	7.527	7.319
AC245128.30	7.716	7.884	7.662	7.998	7.979	7.906
LIPA	12.664	12.778	12.823	12.676	12.574	12.702
CUTA	10.896	11.066	11.240	10.907	10.882	10.929
BPGM	6.963	7.120	7.143	6.591	6.871	7.093
CUTC	7.422	7.615	7.399	7.221	7.295	7.539
ADGRE5	9.107	9.109	9.250	8.772	8.747	8.783
ADGRE1	6.939	7.141	7.053	7.381	7.467	7.485
IFITM1	11.157	11.155	11.113	11.114	11.385	10.859
ADGRE3	6.227	6.097	6.003	6.228	6.257	6.072
ADGRE2	4.948	4.887	4.719	4.727	4.942	4.911
MCF2L-AS1	6.153	6.132	6.004	6.175	5.814	6.224
ATP11C	8.166	8.127	8.074	7.795	7.641	7.766
ATP11B	9.800	9.838	9.781	9.707	9.779	9.748
ATP11A	9.658	9.621	9.593	9.197	9.319	9.491
RHOQP1	8.936	8.925	8.970	8.863	9.224	8.725
RHOQP2	7.671	7.774	7.728	7.525	7.733	7.625
RHOQP3	9.946	9.966	9.953	9.787	10.144	9.767
SHANK2-AS2	7.347	7.025	7.038	7.082	7.154	7.151
SHANK2-AS3	7.207	7.132	7.378	7.318	7.377	7.152
HPCAL4	7.513	7.662	7.620	7.770	7.832	7.649
LIPF	6.056	6.088	6.141	6.163	6.051	6.146
LIPI	4.294	4.402	4.297	4.798	4.643	4.606
LIPH	10.762	10.722	10.810	10.463	10.699	10.341
OGDHL	7.180	7.225	7.292	7.067	7.216	7.144
PI16	8.262	8.244	8.331	8.626	8.341	8.322
PI15	7.066	7.140	6.991	7.385	7.162	7.322
CEP68	8.558	8.674	8.863	8.452	8.654	8.140
CEP63	8.489	8.523	8.619	8.182	8.072	8.265
COQ10B	10.087	10.133	10.234	10.193	10.177	9.991
COQ10A	8.570	8.525	8.563	8.442	8.203	8.414
A4GALT	7.857	7.693	7.898	8.010	8.042	7.916
CTRL	7.192	7.219	7.360	7.210	7.293	7.221
CTRC	6.920	7.205	7.260	7.103	7.199	7.252
TRMT61A	9.521	9.578	9.762	9.551	9.475	9.415
TRMT61B	8.557	8.675	8.777	8.642	8.598	8.647
ZFYVE9	8.657	8.733	8.675	8.624	8.694	8.713
PCDHB17P	4.699	4.768	4.720	4.700	4.730	4.755
ZFYVE1	8.658	8.794	8.906	8.638	8.685	8.662
LDOC1	7.515	7.529	7.372	7.420	7.654	7.745
RXFP1	5.928	5.989	6.142	5.997	6.114	6.115
CCDC88A	8.038	7.988	8.037	7.897	8.007	8.049
RXFP3	5.387	5.277	5.125	5.406	5.271	5.317
CCDC88C	8.971	8.980	9.035	8.874	8.855	8.778
AC022031.1	6.848	7.068	6.726	7.209	7.226	7.058
MPEG1	5.447	5.672	5.501	5.790	5.699	5.546
QTRT2	8.300	8.274	8.195	7.901	8.022	8.009
QTRT1	8.620	8.678	8.744	8.374	8.494	8.601
FBXL8	6.504	6.493	6.591	6.144	6.221	6.192
ROBO1	8.054	8.045	8.085	7.482	7.410	7.592
ROBO3	7.318	7.210	7.394	7.318	7.269	7.174
ROBO2	5.621	5.657	5.349	5.589	5.671	5.785
SIGLECL1	4.967	5.245	4.733	5.277	5.328	5.154
FBXL4	7.427	7.439	7.474	7.106	7.168	7.424
FBXL7	7.497	7.616	7.594	7.610	7.448	7.529
FBXL6	8.693	8.680	8.782	8.621	8.589	8.565

NIPSNAP3A	7.479	7.564	7.519	6.889	7.150	7.211
CTR9	11.673	11.706	11.864	11.695	11.674	11.766
NIPSNAP3B	6.460	6.379	6.244	6.147	6.456	6.376
AL445209.1	5.165	5.419	5.633	5.471	5.416	5.282
NMRAL2P	6.970	7.156	6.802	6.890	7.280	7.200
TRAF3	7.892	8.015	8.163	7.903	7.871	7.995
RNH1	10.871	11.006	11.100	10.947	10.868	10.868
TRAF4	9.919	10.028	10.099	10.090	10.376	9.973
TRAF5	7.163	7.319	7.422	7.043	7.049	7.097
SOX21-AS1	4.812	4.957	4.946	4.865	4.840	4.923
MRPL41	11.132	11.181	11.346	11.124	10.954	11.141
MIAT	8.106	7.921	7.956	8.051	8.232	8.187
TRH	7.990	7.926	7.864	7.938	7.943	7.998
FRMPD2B	6.054	5.909	5.753	6.026	6.098	6.326
TRO	7.002	6.913	6.814	6.737	7.072	7.108
SLC26A1	6.341	6.498	6.440	6.664	6.861	6.753
SLC26A2	9.134	9.315	9.283	8.916	8.960	8.853
SLC26A3	4.109	4.348	3.977	4.151	4.032	4.051
SLC26A4	4.329	4.288	4.211	4.370	4.354	4.120
MIA3	9.066	9.067	9.112	8.851	8.866	8.935
MIA2	7.489	7.688	7.641	7.581	7.647	7.635
SLC26A8	7.027	6.916	6.853	7.157	6.857	7.038
SLC26A9	6.495	6.507	6.707	6.446	6.819	6.758
TRIP4	9.768	9.797	10.060	9.676	9.600	9.638
HTT	8.608	8.730	8.765	8.572	8.688	8.657
MFSD13A	8.272	8.189	8.138	7.912	8.107	7.927
AL596325.1	5.021	5.383	5.111	5.478	5.568	5.310
POM121C	10.486	10.539	10.542	10.343	10.291	10.297
DIABLO	11.543	11.711	11.798	11.634	11.467	11.613
LINC00051	4.677	4.880	4.722	4.563	4.745	4.559
AC027237.2	8.089	7.940	7.820	7.898	8.084	7.828
KIAA1586	4.637	4.701	4.923	4.470	4.475	4.528
XKRY2	4.480	4.377	4.275	4.476	4.624	4.561
AC087164.1	7.412	7.462	7.596	7.414	7.421	7.524
AL357673.2	6.584	6.446	6.081	6.666	6.568	6.656
LINC02525	7.333	7.408	7.027	7.373	7.536	7.761
AL357673.1	7.650	7.326	7.660	7.596	7.552	7.421
LRRC8E	8.355	8.350	8.440	7.978	8.471	8.247
LRRC8D	9.700	9.694	9.709	9.737	9.830	9.635
LRRC8A	10.604	10.662	10.796	10.554	10.512	10.534
LRRC8C	8.344	8.222	8.234	8.270	8.526	8.496
LRRC8B	9.753	9.818	9.874	9.537	9.498	9.533
MSTO2P	8.666	8.743	8.755	8.391	8.635	8.536
ZUP1	7.678	7.636	7.699	7.738	7.648	7.632
HDC	7.470	7.478	7.523	7.548	7.565	7.680
TOR4A	9.830	9.990	10.300	9.670	9.950	9.951
MAGEB18	3.914	3.617	3.800	3.610	3.707	3.681
CDC42P6	11.899	11.866	12.365	12.046	12.003	11.565
C21orf62	7.959	7.950	7.940	7.924	8.027	7.988
GPR52	7.882	7.851	7.488	7.953	8.017	7.960
GPR50	6.540	6.630	6.516	6.602	6.601	6.653
GPR55	6.994	6.996	7.121	7.025	7.135	7.143
FAM83C	6.813	6.907	6.929	7.062	6.995	7.030
FAM83B	9.174	9.220	9.260	8.814	8.954	9.069
FAM83A	8.378	8.482	8.423	8.443	8.479	8.498
FAM83G	8.411	8.484	8.634	8.370	8.372	8.437

FAM86B3P	7.981	7.880	7.994	8.183	7.956	7.961
FAM83E	8.131	8.250	8.378	8.285	8.412	8.073
FAM83D	10.991	10.911	11.149	10.698	10.792	10.597
UNC93B4	9.103	9.078	9.349	9.121	9.095	8.982
AL162231.2	6.576	6.438	6.206	6.452	6.440	6.468
ATP5MF	10.953	11.025	11.100	11.042	10.912	11.083
ATP5ME	10.312	10.394	10.548	10.538	10.311	10.480
AL162231.1	7.268	7.206	7.502	7.458	7.448	7.361
AL162231.4	7.268	7.206	7.502	7.458	7.448	7.361
BMF	7.741	7.714	7.890	7.787	7.746	7.838
NPM1	13.203	13.272	13.262	13.249	13.199	13.294
NPM3	9.554	9.605	9.756	9.543	9.694	9.540
NPM2	9.023	9.139	9.164	9.045	9.110	9.251
PEMT	8.974	9.015	9.177	8.997	8.887	8.882
NEK1	8.211	8.185	8.250	8.077	8.537	8.124
TMEM26	4.245	4.417	4.485	4.476	4.344	4.492
TMEM25	7.875	7.804	8.047	8.034	7.819	7.957
DGCR11	8.381	8.210	7.991	8.106	8.205	8.275
DGCR10	6.977	7.015	7.003	7.019	7.013	7.128
AL603756.1	6.590	6.583	6.347	6.526	6.512	6.700
PPP3R2	6.472	6.138	6.049	6.379	6.351	6.398
PPP3R1	8.138	8.169	8.442	8.893	8.073	7.985
SCG5	5.002	4.906	4.749	5.078	5.109	5.012
SCG3	4.137	4.030	4.123	4.188	4.250	4.202
SCG2	5.760	5.965	5.748	5.892	6.072	6.074
ACOD1	7.359	7.207	7.248	7.684	7.456	7.410
AP5S1	8.582	8.558	8.436	8.142	8.434	8.339
BYSL	9.579	9.633	9.668	9.491	9.621	9.600
Z85995.1	7.862	8.124	8.014	7.897	8.029	8.236
VCY1B	8.470	8.155	8.014	8.567	8.399	8.342
AC104109.2	7.266	7.297	7.242	7.153	7.312	7.358
AL390237.1	3.894	3.882	3.900	3.798	3.857	4.046
GIP	6.140	6.195	6.187	6.094	6.208	6.244
SCCPDH	10.101	10.194	10.300	10.109	9.942	10.114
SERP2	6.446	6.315	6.116	6.348	6.488	6.299
SERP1	12.844	12.930	13.016	12.982	13.016	13.012
AC099343.4	7.736	7.914	7.782	8.080	8.112	8.043
MS4A4A	8.334	8.133	8.293	8.541	8.340	8.226
LRP5	9.390	9.689	9.829	9.639	9.715	9.296
CXorf65	5.625	5.743	5.541	5.696	5.688	5.543
LAMA4-AS1	7.198	6.992	7.193	7.309	7.245	7.341
SCGN	7.783	7.611	7.761	7.827	7.941	7.924
COLEC12	6.618	6.672	6.736	6.708	6.771	6.777
COLEC10	5.921	6.325	6.124	6.102	6.318	6.299
COLEC11	7.601	7.740	7.343	7.718	7.636	7.512
DHPS	9.782	9.792	9.991	9.713	9.601	9.687
LARGE-AS1	7.583	7.657	7.584	7.410	7.552	7.703
BNIPL	9.154	9.347	9.373	8.978	9.078	9.144
MAGI2-AS2	5.475	5.573	5.669	5.821	5.531	5.641
MAGI2-AS3	7.565	7.309	7.254	7.639	7.626	7.679
PAICS	11.599	11.581	11.713	11.501	11.383	11.374
OR1I1	7.966	8.186	8.154	8.392	8.261	8.282
GABRG3-AS1	7.820	7.846	7.553	7.616	7.691	7.931
AC022364.1	6.864	6.639	6.657	6.851	6.817	6.787
VWA5B2	8.474	8.244	8.359	8.450	8.600	8.502
VWA5B1	6.728	6.757	6.760	6.823	7.064	6.890

VTA1	9.683	9.882	9.841	9.680	9.648	9.692
PUS7L	7.936	7.812	7.656	7.851	7.940	7.997
ZNF492	4.078	4.073	3.984	4.086	4.066	4.182
COMMD9	10.082	9.967	10.235	10.006	10.012	9.856
BNIP1	8.130	8.145	8.428	8.127	8.170	8.131
BNIP2	8.244	8.252	8.226	8.023	7.918	8.173
BNIP3	11.058	11.086	11.391	11.286	11.397	11.321
ZNF491	7.035	7.258	6.973	6.975	7.211	7.508
AC119403.1	7.588	7.584	7.602	7.935	7.880	7.737
NAB1	9.283	9.290	9.251	9.278	9.005	9.416
NAB2	9.117	9.208	9.276	9.075	8.928	8.927
IYD	4.367	4.196	4.783	3.974	3.979	3.974
AKAP14	4.275	4.437	4.436	4.587	4.611	4.514
AKAP12	8.165	8.313	8.415	7.852	7.678	8.037
AKAP13	10.624	10.633	10.637	10.343	10.459	10.611
AKAP10	8.380	8.531	8.454	8.290	8.461	8.380
AKAP11	9.038	8.978	9.100	8.643	8.736	9.017
ZSCAN5A	7.304	7.246	7.415	7.209	7.233	7.287
SKIDA1	4.895	5.088	4.897	4.868	4.819	4.887
AL513534.3	4.670	4.626	4.868	4.681	4.620	4.733
AC068473.8	6.869	6.866	6.988	6.951	7.091	6.788
AC068473.3	6.812	6.801	6.682	6.776	7.055	6.931
AC068473.5	6.869	6.866	6.988	6.951	7.091	6.788
AC068473.6	6.812	6.801	6.682	6.776	7.055	6.931
LNP1	8.252	8.361	8.192	8.671	8.549	8.644
NOTCH2NLC	9.707	9.662	9.784	9.264	9.334	9.270
NOTCH2NLB	9.707	9.662	9.784	9.264	9.334	9.270
NOTCH2NLA	9.707	9.662	9.784	9.264	9.334	9.270
LNPK	9.632	9.683	9.873	9.630	9.644	9.771
CKS1BP3	11.113	11.134	11.460	11.121	11.202	11.148
CKS1BP2	11.113	11.134	11.460	11.121	11.202	11.148
PTMAP4	12.043	12.081	12.297	11.941	12.292	11.943
PTMAP5	13.414	13.436	13.389	13.470	13.514	13.402
AL049557.1	7.569	7.645	7.502	7.705	7.592	7.658
PTMAP1	7.449	7.501	7.470	7.599	7.467	7.427
PTMAP2	13.414	13.436	13.389	13.470	13.514	13.402
PTMAP3	12.699	12.748	12.812	12.754	12.867	12.745
PTMAP9	12.043	12.081	12.297	11.941	12.292	11.943
USP32P3	8.816	8.726	8.703	8.342	8.439	8.740
TP73	7.436	7.518	7.596	7.634	7.566	7.620
AL645929.2	7.256	7.358	7.378	7.195	7.328	7.531
TBP	7.761	7.636	7.842	7.621	7.847	7.766
PRR20B	6.354	6.375	5.959	6.267	6.135	6.357
GLS2	6.742	6.715	7.097	6.775	6.567	6.557
AC011944.2	4.419	4.441	4.498	4.695	4.495	4.420
PRR20C	6.354	6.375	5.959	6.267	6.135	6.357
EGFR	10.960	11.021	11.209	10.889	10.893	10.830
WDR86	6.363	6.377	6.484	6.367	6.493	6.522
ALOX5AP	6.875	7.068	6.950	7.187	7.088	7.010
WDR81	8.903	8.886	8.981	8.791	8.783	8.863
WDR82	10.415	10.329	10.407	10.243	10.291	10.321
WDR83	7.968	8.161	8.270	8.155	8.162	8.208
WDR88	6.178	6.197	6.182	6.178	6.111	6.212
WDR89	9.437	9.298	9.400	9.530	9.432	9.380
CAMLG	10.274	10.261	10.525	10.216	10.199	10.033
ZMYM4	9.063	9.088	9.109	8.864	8.930	8.937

IMPDH1	9.450	9.570	9.781	9.432	9.450	9.495
IMPDH2	11.497	11.465	11.738	11.315	11.135	11.311
PSMD6-AS1	5.989	6.211	5.987	6.299	6.250	6.370
PSMD6-AS2	5.989	6.211	5.987	6.299	6.250	6.370
AC004675.1	7.833	7.880	7.750	7.857	7.852	7.861
JAG2	8.656	8.822	8.792	8.595	8.606	8.812
JAG1	12.132	12.218	12.220	12.124	12.101	12.264
AJUBA	9.945	9.878	9.958	9.737	9.605	9.842
QRICH2	6.705	6.681	6.785	6.893	6.921	6.811
QRICH1	9.901	9.927	9.982	9.846	9.727	9.785
E4F1	8.046	7.990	8.144	7.974	7.972	7.920
PTPN21	7.529	7.600	7.768	7.668	7.700	7.729
PLEKHF1	8.293	8.338	8.336	8.218	8.238	8.325
PITPNA-AS1	7.038	7.360	7.320	7.134	7.348	7.461
AC011487.2	7.044	7.026	6.674	7.372	7.061	6.898
CHRNG	7.670	7.693	7.702	7.637	7.604	7.719
CHRND	6.492	6.541	6.767	6.706	6.679	6.607
CHRNE	6.623	6.784	7.027	7.064	7.042	6.923
AC097359.2	7.685	7.617	7.582	7.565	7.670	7.562
TNRC6C	7.619	7.772	7.548	7.343	7.678	7.369
TNRC6B	9.341	9.185	9.224	9.063	9.260	9.112
TNRC6A	9.332	9.278	9.343	9.303	9.542	9.389
USP28	8.423	8.474	8.424	8.290	8.394	8.286
USP26	5.878	5.511	5.629	5.924	5.824	5.792
AC020763.2	7.421	7.524	7.511	7.273	7.437	7.573
USP24	8.027	8.089	7.939	7.670	7.622	7.756
AC018645.3	7.296	7.043	7.132	7.121	7.061	7.171
USP21	8.856	8.808	8.903	8.673	8.841	8.802
USP20	8.220	8.312	8.315	8.307	8.274	8.096
ADAMTS5	6.156	6.145	5.984	6.382	6.153	6.311
ADAMTS4	8.200	8.176	8.035	8.070	8.106	8.137
ADAMTS7	7.252	7.226	7.167	7.371	7.396	7.325
ADAMTS6	6.015	6.137	5.840	5.691	5.899	5.877
ADAMTS1	7.088	7.087	7.212	7.408	6.956	7.004
ADAMTS3	4.457	4.512	4.642	4.918	4.673	4.300
ADAMTS2	7.478	7.339	7.259	7.483	7.355	7.346
CCSER2	9.053	9.013	8.654	9.283	9.184	9.139
ARHGEF7	8.536	8.644	8.474	8.356	8.420	8.618
ARHGEF4	7.345	7.282	7.150	7.360	7.536	7.455
CCSER1	5.759	5.803	5.552	6.004	5.910	5.865
ADAMTS9	9.233	9.153	9.241	9.153	8.949	9.065
ADAMTS8	7.181	7.273	7.344	7.373	7.336	7.516
ARHGEF1	8.995	8.884	9.169	9.077	8.937	9.073
CDH9	5.940	5.893	6.250	6.581	6.278	6.224
CDH8	6.667	6.890	6.884	6.890	7.069	6.787
PROKR2	6.490	6.729	6.656	6.381	6.471	6.650
RPL23AP53	6.418	6.533	6.515	6.451	6.556	6.405
CDH1	12.626	12.725	12.768	12.727	12.657	12.712
CDH3	10.904	10.871	11.012	10.769	10.714	10.699
RP1L1	6.827	6.938	6.738	6.972	7.220	6.889
CDH5	4.969	4.742	4.746	4.680	5.069	4.909
CDH4	7.045	7.061	6.928	7.084	7.122	6.871
CDH7	6.614	6.533	6.466	6.628	6.700	6.822
CDH6	8.594	8.568	8.464	8.602	8.833	8.770
TERF2IP	9.904	10.022	10.012	9.801	9.822	9.889
AC018659.4	5.353	5.180	5.281	5.445	5.466	5.259

AC018659.6	6.465	6.382	6.553	6.753	6.624	6.679
AC018659.3	6.453	6.592	6.418	6.788	6.780	6.615
NETO1	6.146	6.189	6.129	6.340	6.269	6.280
NETO2	10.871	10.961	10.999	10.659	10.642	10.643
RPS23P6	7.268	7.539	7.574	7.394	7.522	7.525
RFESD	6.600	6.940	6.809	7.148	6.886	6.980
AL390728.6	6.124	5.925	5.875	5.888	5.786	6.041
KMT2A	8.809	8.957	9.073	8.768	8.871	8.567
AL390728.4	5.629	5.493	5.450	5.738	5.684	5.926
LCE1E	9.167	9.266	9.003	9.075	9.517	9.325
LCE1B	6.721	6.695	6.673	6.743	6.974	6.864
OR8G2P	5.670	5.716	5.271	5.614	5.763	5.628
ESRRB	7.303	7.472	7.361	7.330	7.518	7.410
PGBD1	5.208	4.952	5.116	4.882	5.114	4.774
DSCR10	8.070	8.101	8.196	8.244	8.275	8.400
AC092171.4	7.511	7.270	7.223	7.509	7.328	7.490
ECSIT	8.384	8.455	8.475	8.291	8.273	8.275
JTB	12.002	12.031	12.096	11.939	11.957	12.003
SUPT6H	10.039	10.046	10.016	9.894	9.917	9.809
RNF144A-AS1	8.520	8.477	8.262	8.479	8.534	8.439
CILP2	6.671	6.716	6.578	6.645	6.516	6.632
ZNF501	4.247	4.304	4.317	4.275	4.418	4.378
ZNF500	8.615	8.386	8.305	8.077	8.258	8.271
ZNF503	7.954	7.955	7.856	7.872	8.103	7.898
ZNF502	7.896	7.886	7.970	8.231	8.153	7.992
ZNF507	8.979	9.003	8.856	8.678	8.518	8.842
ZNF506	7.567	7.572	7.805	7.289	7.331	7.460
TREM1	6.819	6.739	6.414	6.873	6.914	6.746
TREM2	8.143	8.220	8.358	8.195	8.343	8.262
RPL41	14.596	14.604	14.644	14.671	14.651	14.656
Z84468.1	7.200	7.414	7.266	7.519	7.236	7.449
RACGAP1	10.000	9.926	9.944	9.204	9.419	9.436
FAM167B	7.456	7.246	7.381	7.585	7.419	7.496
FAM167A	6.792	6.954	7.217	7.071	7.016	7.240
NRSN1	6.462	6.591	6.414	6.388	6.458	6.500
AC147067.1	6.875	6.712	6.857	6.703	6.788	6.864
CFAP97D1	4.625	4.536	4.325	4.800	4.652	4.349
ZNF430	8.229	8.265	8.197	8.268	8.270	8.248
FGD5	7.516	7.532	7.431	7.693	7.641	7.663
FGD4	9.759	9.917	9.946	9.461	9.687	9.510
AL845472.1	6.925	7.126	7.107	7.121	7.103	7.270
PUS3	9.017	8.926	9.231	9.011	9.314	8.962
FGD1	7.488	7.402	7.332	7.416	7.622	7.706
FGD3	8.368	8.633	8.721	8.778	8.652	8.736
PUS7	9.140	9.260	9.251	9.068	9.017	9.076
VTN	6.526	6.736	6.661	6.685	6.894	6.844
MERTK	6.575	6.696	6.902	6.939	6.938	6.862
NPAP1	7.320	7.460	7.310	7.529	7.618	7.482
AC133134.1	13.772	13.812	13.879	13.778	13.633	13.771
KEAP1	9.287	9.346	9.520	9.048	9.165	9.071
LINC02727	5.325	5.616	5.174	5.261	5.421	5.463
LINC02724	5.094	4.903	5.355	5.467	5.287	5.242
LINC02728	4.977	4.859	4.940	4.963	5.057	4.866
ZBTB8OS	9.223	9.209	9.393	8.969	9.136	9.244
SMN2	7.158	7.220	7.146	7.159	7.206	7.174
POLA2	8.924	8.887	9.071	8.710	8.741	8.852

POLA1	8.417	8.190	8.176	7.979	7.873	8.175
SMN1	7.158	7.220	7.146	7.159	7.206	7.174
AC092159.3	6.998	6.976	6.956	6.913	7.155	7.075
FBRSL1	9.289	9.282	9.336	9.002	8.973	9.066
SLC5A1	8.420	8.411	8.724	8.473	8.452	8.483
MEGF8	8.400	8.370	8.614	8.384	8.338	8.354
SLC5A3	9.956	10.065	10.099	10.042	9.926	10.059
SLC5A4	6.782	6.668	6.642	6.541	6.831	6.797
CCNB1IP1	9.912	9.911	10.054	9.847	9.910	9.853
SLC5A6	9.381	9.354	9.351	9.266	9.522	9.437
SLC5A7	7.109	7.050	6.901	7.337	7.293	7.313
SLC5A8	7.455	7.455	7.456	7.444	7.456	7.452
SLC5A9	7.055	7.074	7.064	7.135	7.258	7.077
MEGF6	8.111	8.224	8.190	7.912	7.956	7.877
UCN	6.229	6.322	6.322	6.418	6.158	6.403
HIP1R	11.495	11.561	11.642	11.328	11.189	11.309
ZBTB3	8.122	7.994	8.144	7.869	7.920	8.071
ZBTB2	8.130	8.102	8.175	8.059	8.101	7.997
MAP3K7CL	4.155	4.081	4.066	4.234	4.239	4.093
CARD18	6.536	6.576	6.486	6.539	6.542	6.730
TOM1L1	9.763	9.849	9.969	9.830	9.821	9.844
ZBTB5	7.925	7.955	7.840	7.753	7.886	7.895
ZBTB4	9.259	9.203	9.309	9.275	9.262	9.066
NBR2	11.925	11.994	12.146	11.893	11.885	11.984
CARD11	9.663	9.747	9.881	9.780	9.581	9.664
NBR1	11.115	11.239	11.322	11.046	10.959	11.017
CARD16	7.975	7.484	7.242	8.162	7.915	7.749
CARD14	7.584	7.779	7.775	7.760	7.740	7.681
LINC00494	8.091	8.050	8.069	8.186	8.122	8.126
LINC00491	10.180	9.939	9.676	10.035	10.132	10.152
AC240565.4	8.340	8.109	7.986	8.227	8.359	8.262
AC240565.5	8.340	8.109	7.986	8.227	8.359	8.262
AC240565.6	7.688	7.417	7.266	7.457	7.674	7.621
AC240565.3	7.688	7.417	7.353	7.526	7.674	7.621
EIF5AP4	12.743	12.696	12.917	12.633	12.437	12.525
AL049839.2	13.952	14.012	14.082	13.968	13.892	13.975
AC017091.2	4.181	3.997	3.949	4.134	3.829	4.022
AC010319.2	8.403	8.526	8.459	8.432	8.510	8.636
AC017091.1	4.181	3.997	3.949	4.134	3.829	4.022
CUL4A	8.200	8.245	8.337	8.144	7.974	7.983
CUL4B	10.479	10.504	10.520	10.469	10.348	10.390
IMMT	10.167	10.177	10.343	10.074	9.926	10.003
GLCCI1	8.269	8.034	8.059	8.422	8.073	8.531
SLC6A3	7.451	7.642	7.517	7.513	7.660	7.560
SLC6A2	8.516	8.587	8.395	8.825	8.562	8.651
SLC6A1	4.524	4.621	4.497	4.818	4.696	4.695
SLC6A7	7.435	7.502	7.491	7.318	7.785	7.562
SLC6A6	10.213	10.185	10.220	9.605	9.697	9.840
SLC6A5	7.373	7.306	7.341	7.351	7.340	7.359
SLC6A4	7.973	7.790	7.741	7.848	7.821	7.886
SLC6A9	6.953	7.049	7.001	6.596	7.010	6.831
SLC6A8	9.568	9.560	9.737	9.575	9.537	9.519
LRPPRC	11.430	11.478	11.421	11.363	11.206	11.376
C12orf56	7.352	7.531	7.380	7.341	7.209	7.251
C12orf57	11.391	11.444	11.677	11.240	11.213	11.453
C12orf54	6.681	6.690	6.719	7.118	7.182	6.898

C12orf50	4.518	4.582	4.248	4.511	4.506	4.831
FMNL2	10.431	10.291	10.363	10.152	10.230	10.327
FMNL3	7.161	7.174	7.118	7.071	7.261	7.252
FMNL1	7.442	7.571	7.757	7.768	7.784	7.647
PACC1	8.828	8.804	9.008	8.749	8.818	8.882
NEXN	6.344	6.209	6.159	6.362	6.458	6.510
LNCOC1	5.130	5.226	5.086	5.078	5.462	5.174
GRM8	7.215	7.273	7.321	7.419	7.308	7.272
AL671277.2	4.808	4.793	4.857	4.591	4.736	4.528
MAPRE1	12.466	12.569	12.655	12.481	12.375	12.484
MAPRE3	8.479	8.386	8.546	8.749	8.700	8.489
MAPRE2	9.884	9.874	10.064	9.750	9.730	9.717
NR1H4	8.595	8.563	8.694	8.362	8.465	8.468
PATZ1	9.485	9.573	9.637	9.311	9.360	9.348
SCARNA2	7.544	7.153	7.252	7.258	7.315	7.360
GTF2I	10.885	10.859	10.911	10.785	10.745	10.628
EIF1B-AS1	8.846	8.668	8.670	8.908	8.998	8.896
MCRIP1	8.663	8.756	8.760	8.553	8.660	8.686
IGLV3-25	5.747	5.670	5.679	5.914	5.728	5.749
MCRIP2	9.969	10.040	10.354	10.133	10.174	10.080
GRM2	7.922	7.921	7.978	7.836	8.041	7.981
RNF215	8.024	8.144	8.095	7.977	8.021	8.011
RNF214	8.691	8.745	8.744	8.665	8.706	8.808
RNF217	8.029	8.094	8.139	7.768	7.905	8.020
RNF216	8.983	8.965	8.956	8.887	9.022	9.080
RNF213	10.554	10.577	10.559	10.464	10.467	10.276
RNF212	8.585	8.441	8.505	8.680	8.593	8.459
MED29	9.882	9.942	9.956	9.476	9.396	9.500
MED28	8.899	8.830	9.061	8.929	9.006	8.879
MED21	9.281	9.319	9.368	9.264	9.357	9.310
MED20	8.921	8.836	8.941	8.970	8.849	8.891
ENAH	9.558	9.547	9.623	9.499	9.478	9.535
MED22	8.469	8.531	8.692	8.005	8.184	8.134
MED25	10.108	10.187	10.277	9.864	9.958	9.827
MED24	11.711	11.728	11.871	11.591	11.556	11.599
MED27	9.307	9.186	9.350	9.152	9.180	9.166
MED26	8.838	8.808	8.890	8.853	8.861	8.899
AP002008.4	6.004	5.936	6.084	6.233	6.379	6.234
AP002008.1	6.780	6.906	6.771	6.921	6.922	6.810
AP002008.3	5.010	5.381	5.289	5.177	5.468	5.474
AC107419.1	5.990	6.066	6.062	5.965	5.913	6.305
PRORY	7.315	7.234	7.346	7.391	7.258	7.264
PRORP	9.362	9.367	9.477	9.343	9.414	9.457
TRIAP1	10.547	10.572	10.735	10.664	10.558	10.597
AP002884.3	6.702	6.525	6.834	6.861	6.872	6.801
ACTG1P17	6.745	6.620	6.811	6.284	6.626	6.678
MIR6764	12.056	12.038	12.151	11.846	12.027	11.628
PARP8	8.296	8.243	8.351	8.284	8.277	8.098
PARP9	10.546	10.557	10.524	10.396	10.321	10.334
PARP4	11.284	11.299	11.383	11.158	11.019	11.161
PARP6	9.857	9.900	9.921	9.737	9.651	9.947
MIR6768	8.524	8.673	8.657	8.394	8.510	8.493
PARP1	10.854	10.832	10.986	10.735	10.431	10.580
PARP2	9.548	9.690	9.744	9.450	9.521	9.548
PARP3	7.908	8.036	7.768	7.951	7.708	7.757
ALDH4A1	8.451	8.479	8.633	8.460	8.620	8.297

NVL	8.290	8.200	8.318	8.106	7.983	8.022
FGF22	8.052	8.303	8.281	8.234	8.425	8.469
FGF23	7.098	7.195	7.020	7.088	7.108	7.104
FGF20	4.755	4.726	4.838	4.933	4.811	4.898
FGF21	6.564	6.803	6.476	6.624	6.758	6.629
AC009078.3	6.756	6.738	6.885	6.848	6.812	6.757
AC009078.4	6.756	6.738	6.885	6.848	6.812	6.757
FRG1BP	8.645	8.715	8.824	8.474	8.590	8.485
AC025362.1	7.575	7.482	7.794	7.711	7.576	7.622
MPST	10.466	10.473	10.699	10.320	10.243	10.328
GCNT1P1	6.998	6.831	6.745	7.034	6.928	6.836
CSGALNACT1	8.713	8.811	8.805	8.627	8.582	8.714
YWHAEP7	8.215	8.174	7.962	8.265	8.311	8.276
ALDH7A1	9.480	9.518	9.821	9.225	9.124	9.137
AC005005.3	7.793	7.725	7.954	7.846	7.836	7.914
AP000812.5	8.399	8.357	8.399	8.073	8.205	8.299
HMGXB3	9.755	9.770	9.811	9.775	9.808	9.854
HMGXB4	9.150	9.175	9.444	9.157	9.115	9.043
BAHD1	8.540	8.647	8.627	8.638	8.639	8.589
MYLPF	8.085	7.886	8.015	8.184	8.065	8.074
YES1	9.798	9.809	9.783	9.566	9.550	9.605
ECT2	11.162	11.172	11.170	10.783	10.901	10.944
AC091825.1	6.736	6.888	6.842	6.866	6.702	6.904
AC091825.3	5.847	5.825	5.646	5.842	5.780	5.604
AC091825.2	6.736	6.888	6.842	6.866	6.702	6.904
ZCCHC9	8.506	8.645	8.772	8.517	8.558	8.550
ZCCHC8	9.999	10.044	10.150	9.944	10.023	10.138
TTTY8B	6.686	6.560	6.735	6.884	6.688	6.702
AC005011.1	10.192	10.361	10.528	10.360	10.318	10.298
BX927139.2	8.439	8.433	8.212	8.323	8.555	8.407
ZCCHC7	6.652	6.752	6.564	5.895	6.230	6.224
ZCCHC4	7.692	7.921	7.775	7.668	7.783	7.862
RENBP	7.823	7.771	7.696	7.839	7.801	7.993
AC023389.3	5.094	5.230	5.085	5.213	5.281	5.235
AC010969.2	8.657	8.765	8.881	9.117	8.841	8.710
CNMD	6.793	6.564	6.581	6.903	6.961	6.924
AC027804.1	5.080	5.088	5.075	5.111	5.035	5.141
CHADL	6.716	6.634	6.808	6.637	6.789	6.734
C6orf132	8.636	8.654	8.685	8.540	8.717	8.408
ARID3B	8.486	8.377	8.435	8.596	8.546	8.476
C6orf136	8.960	9.011	9.064	8.859	8.954	8.951
AL590560.4	6.760	6.871	6.709	6.809	6.826	6.609
AL590560.2	7.326	7.572	7.541	7.471	7.667	7.502
AL590560.3	7.762	7.994	7.914	8.040	8.001	7.954
LRRC77P	5.869	5.812	5.571	5.839	5.894	5.632
AL031775.1	7.039	7.263	7.152	6.917	6.856	7.031
L3HYPDH	7.003	7.025	7.176	7.049	7.044	7.078
C19orf44	7.054	6.863	6.855	7.033	7.347	7.008
C19orf47	8.082	8.063	8.136	8.094	8.121	8.041
C19orf48	10.753	10.776	10.988	10.545	10.601	10.548
CEMP1	7.112	7.031	7.090	7.131	7.018	6.985
AC138969.4	7.182	7.381	7.328	7.421	7.457	7.500
NECAB3	9.631	9.727	9.881	9.507	9.584	9.609
NECAB2	8.387	8.262	8.181	8.169	8.293	8.120
NECAB1	4.136	4.295	4.260	4.251	4.132	4.137
AC138969.1	11.306	11.174	11.094	11.070	11.186	11.250

SARAF	11.666	11.732	11.765	11.595	11.483	11.809
HTR6	7.783	7.565	7.624	7.910	7.889	7.799
HTR7	7.245	7.370	7.377	7.583	7.673	7.562
HTR4	8.594	8.812	8.792	8.754	8.755	8.838
RAE1	10.525	10.587	10.644	10.485	10.561	10.482
TNRC6C-AS1	8.604	8.622	8.625	8.489	8.171	8.665
KDELR2	12.641	12.780	12.815	12.794	12.864	12.748
KDELR3	10.037	10.104	10.199	10.013	10.212	10.126
KDELR1	12.041	12.120	12.162	12.112	12.090	12.175
GU182343.2	8.835	8.689	8.488	8.744	8.793	8.879
CLEC12B	5.256	5.244	4.934	5.234	5.164	5.177
CLEC12A	5.674	5.628	5.495	5.630	5.713	5.827
LRRD1	4.193	3.873	3.902	4.036	4.144	4.138
ST14	11.425	11.352	11.561	11.238	11.257	11.273
ST13	12.367	12.411	12.560	12.394	12.296	12.369
AC013401.2	4.140	4.221	4.337	4.195	4.342	4.429
ST18	7.071	7.203	7.114	7.220	7.122	6.962
NKAPL	6.781	6.716	6.773	7.118	6.658	6.829
PCTP	9.850	9.900	10.167	9.928	9.776	9.948
UBR5-AS1	5.642	5.694	5.656	5.674	5.274	5.743
SLC5A2	7.990	8.118	8.146	8.280	8.241	8.139
PVT1	9.201	9.195	9.048	9.235	9.002	9.206
PLEC	11.347	11.415	11.546	11.196	11.150	11.251
ZNF546	4.289	4.410	4.324	4.355	4.425	4.355
NELL1	6.501	6.682	6.540	6.687	6.693	6.721
NELL2	7.270	7.329	7.084	7.334	7.286	7.269
PLEK	8.407	8.249	8.226	8.678	8.511	8.331
GOT1L1	5.665	5.647	5.531	5.601	5.643	5.647
SPOCK1	6.509	6.669	6.697	6.686	6.567	6.652
SPOCK2	10.891	10.965	11.121	10.813	10.715	10.679
SPOCK3	4.693	4.929	4.964	5.012	4.960	4.986
FNBP1	7.898	7.809	7.854	7.711	7.818	7.775
BTN3A2	9.552	9.529	9.589	9.603	9.514	9.507
BTN3A3	9.552	9.529	9.589	9.603	9.514	9.507
FNBP4	9.230	9.323	9.214	8.983	9.015	9.192
APOOL	10.223	10.233	10.237	10.215	10.184	10.062
ZNF687-AS1	7.956	7.928	8.031	8.074	8.056	8.067
LINC00924	6.964	6.942	6.647	6.955	7.082	6.999
AL024495.1	6.909	6.774	6.718	7.010	6.957	6.966
ZNF747	9.393	9.526	9.655	9.647	9.725	9.656
ZNF746	7.880	8.016	8.142	7.958	7.937	8.044
ZNF740	8.185	8.239	8.415	8.286	8.043	8.045
AC007996.1	6.845	6.668	6.874	7.056	6.758	6.947
ZNF749	4.860	4.962	4.715	5.254	4.854	5.179
AGTRAP	9.520	9.715	9.669	9.491	9.506	9.367
TNFRSF13B	8.064	8.003	8.251	8.278	8.118	8.018
TNFRSF13C	8.212	8.401	8.318	8.218	8.439	8.400
YTHDC1	10.438	10.485	10.523	10.304	10.310	10.342
YTHDC2	9.012	8.849	8.767	8.348	8.951	8.651
TRPS1	9.052	8.916	8.982	8.992	9.006	8.973
AL023653.1	7.364	7.467	7.300	7.564	7.652	7.553
DELE1	8.962	8.906	8.821	8.632	8.752	8.794
LINC02653	6.735	6.773	6.684	6.661	6.921	6.830
SLX1B-SULT1A4	10.684	10.799	10.996	10.631	10.700	10.565
GCLM	10.267	10.298	10.269	10.248	10.211	10.205
GRHL1	8.184	8.319	8.332	8.299	7.953	8.147

GRHL2	10.829	10.675	10.641	10.282	10.402	10.356
GRHL3	7.360	7.583	7.664	7.398	7.407	7.630
GCLC	9.682	9.787	9.685	9.544	9.580	9.666
NR3C2	8.120	8.178	8.154	7.780	7.916	8.071
NR3C1	10.084	10.091	10.107	10.159	10.224	10.381
VCPKMT	7.254	7.077	7.222	6.935	7.112	7.244
SNORD36A	13.419	13.463	13.555	13.460	13.324	13.444
SNORD36B	13.419	13.463	13.555	13.460	13.324	13.444
MIR4680	9.175	9.057	9.177	8.676	8.892	8.760
SSX4B	7.301	7.374	7.153	7.302	7.279	7.432
RAP2C-AS1	6.684	6.564	6.605	6.582	6.633	6.596
INTS6-AS1	6.378	6.443	6.651	6.609	6.681	6.699
MYO7A	8.623	8.359	8.383	8.470	8.363	8.455
MYO7B	7.818	7.834	7.968	8.018	8.075	7.924
AL672142.1	7.283	7.387	7.246	7.271	7.328	7.215
RTCA-AS1	4.691	4.556	4.353	4.558	4.768	4.582
SEC23IP	9.930	10.036	10.007	10.084	10.081	9.777
NAA60	10.665	10.732	10.858	10.641	10.621	10.658
AL359711.2	5.519	5.695	5.711	5.447	5.309	5.404
NAPSA	7.701	7.797	7.983	7.944	8.152	8.095
NAPSB	6.025	6.513	6.255	6.397	6.268	6.448
WIPI2	11.035	11.105	11.230	10.844	10.938	10.691
SAMD3	5.119	5.231	5.148	5.275	5.386	5.415
PDHB	10.833	10.763	10.766	10.437	10.307	10.519
VCX2	7.570	7.633	7.681	7.804	7.550	7.687
AC019117.3	5.405	5.563	5.397	5.786	5.331	5.583
PKDCC	8.734	8.655	8.810	8.670	8.765	8.770
AC019117.2	6.806	6.410	6.165	6.702	6.447	7.009
CSRP1	9.817	10.012	10.231	10.058	10.010	10.074
CSRP3	6.244	6.041	6.117	6.108	6.059	6.139
CSRP2	8.008	7.919	8.402	7.959	8.121	8.027
NYAP1	6.771	6.960	6.788	6.784	7.092	6.745
AC109583.1	7.029	6.991	6.979	7.194	7.444	7.205
AL136295.1	10.845	10.861	11.040	10.826	10.739	10.727
ATP6V1D	10.874	10.999	11.083	10.979	11.019	10.704
AL136295.3	6.170	6.162	6.214	6.136	6.209	6.299
ATP6V1F	11.203	11.349	11.447	11.457	11.497	11.345
ATP6V1A	11.197	11.184	11.189	11.062	11.147	11.007
RASL10A	7.503	7.592	7.567	7.614	7.645	7.615
RASL10B	8.818	8.797	8.919	9.343	9.184	8.876
DTL	9.998	9.979	10.028	9.697	9.615	9.482
AL136295.9	6.170	6.162	6.214	6.136	6.209	6.299
ATP6V1H	9.552	9.744	9.773	9.587	9.255	9.495
IGFLR1	8.912	8.829	9.096	8.677	8.707	8.584
RHEBP1	12.016	11.997	12.130	12.031	12.050	12.061
RHEBP2	12.016	11.997	12.130	12.031	12.050	12.061
AC027271.1	4.801	5.052	4.965	4.921	5.172	4.937
LINC00382	5.231	5.207	5.316	5.412	5.451	5.323
AL354714.2	11.051	11.150	11.276	11.034	11.075	11.044
RAP1GDS1	9.306	9.455	9.428	9.358	9.371	9.343
LINC00320	5.397	5.218	5.115	5.302	5.279	5.437
AC027559.1	6.105	6.163	6.493	6.391	6.391	6.401
PDE6B	7.369	7.589	7.571	7.305	7.570	7.576
PDE6C	3.857	3.945	3.824	3.931	3.785	3.828
PDE6A	7.973	7.813	7.813	7.918	7.969	7.979
PDE6G	8.472	8.145	8.395	8.490	8.434	8.595

PDE6D	9.342	9.390	9.417	9.184	9.271	9.120
IL11RA	8.551	8.706	8.849	8.518	8.500	8.466
PDE6H	4.922	4.707	4.545	4.948	4.719	4.781
FAM226B	5.247	5.220	5.037	5.134	5.141	5.261
RBM12	9.974	10.104	10.139	9.730	9.776	9.859
RBM11	4.558	4.371	4.398	4.516	4.425	4.466
RBM10	9.953	9.957	10.119	9.949	9.709	9.842
RBM17	10.056	10.131	10.225	9.987	9.989	9.914
RBM15	9.749	9.814	9.926	9.604	9.444	9.645
RBM14	10.024	10.056	10.189	9.893	9.991	10.047
RBM19	8.713	8.702	8.793	8.651	8.438	8.559
RBM18	9.030	9.089	9.134	8.819	8.999	9.033
AL445363.3	10.130	10.289	10.251	10.091	10.154	10.239
MVP	11.271	11.394	11.529	11.195	11.222	11.241
AC093535.2	6.034	5.947	5.765	5.903	6.096	6.084
RPL7AP66	13.369	13.436	13.521	13.353	13.281	13.358
MVD	9.080	9.165	9.384	9.191	9.249	9.236
MVK	10.703	10.856	10.924	10.827	10.839	10.849
ALK	7.482	7.589	7.355	7.591	7.505	7.229
SUCLA2	9.321	9.455	9.440	9.254	9.287	9.461
ALB	4.484	4.606	4.484	4.632	4.725	4.772
RELL1	9.175	9.230	9.278	8.762	8.813	8.819
RELL2	8.443	8.483	8.594	8.457	8.466	8.457
RFT1	8.882	8.949	9.032	8.699	8.838	8.754
ZNF18	7.778	7.941	7.950	8.023	8.125	8.020
ZNF19	8.904	8.820	8.878	8.727	8.956	8.989
ZNF14	6.914	7.111	7.253	7.255	7.338	7.166
ZNF16	8.221	8.091	8.197	8.147	8.085	8.092
ZNF10	7.110	7.298	7.118	7.188	7.259	7.206
ZNF12	10.072	10.063	10.088	10.058	10.027	10.092
AC100814.2	4.332	4.133	4.525	4.113	4.030	4.130
PLEKHG4B	6.770	6.853	6.884	7.243	6.961	7.150
SELPLG	8.718	8.460	8.691	8.907	8.776	8.795
HECW1-IT1	4.579	4.685	4.523	4.801	4.506	4.556
AC092865.3	6.451	6.418	6.257	6.244	6.283	6.307
RRN3	9.784	9.869	9.787	9.594	9.550	9.788
LILRA6	8.597	8.738	8.545	8.761	8.665	8.810
LILRA4	7.193	7.300	7.274	7.480	7.263	7.483
LILRA5	6.701	6.931	6.931	6.940	6.987	7.063
LILRA2	8.155	8.435	8.366	8.414	8.374	8.502
LILRA3	6.623	6.464	6.514	6.632	6.752	6.406
LILRA1	6.318	6.289	6.318	6.483	6.300	6.055
AK4P1	11.529	11.637	11.831	11.799	11.904	11.498
AK4P3	11.529	11.637	11.831	11.799	11.904	11.498
UPRT	8.381	8.422	8.459	8.246	8.183	8.290
SELENOT	11.590	11.746	11.861	12.043	11.983	12.003
RMC1	8.663	8.750	8.793	8.574	8.745	8.647
NFE2L3	8.731	8.885	8.976	8.593	8.724	8.607
NFE2L2	11.183	11.311	11.468	11.192	11.393	11.204
NFE2L1	11.635	11.671	11.766	11.643	11.651	11.591
EXOC3-AS1	6.402	6.365	6.398	6.687	6.354	6.418
AL353746.1	5.246	4.671	4.910	5.122	5.246	5.056
P3H4	8.796	8.705	9.104	8.441	8.601	8.536
P3H3	6.467	6.614	6.612	6.551	6.773	6.518
P3H2	8.853	8.900	9.028	8.961	8.775	8.702
P3H1	8.294	8.286	8.662	8.431	8.270	8.365

ASNSD1	9.371	9.449	9.555	9.464	9.375	9.481
SCAND1	10.996	11.076	11.208	10.789	10.625	10.883
KIRREL2	6.887	6.916	7.112	7.321	7.137	7.251
KIRREL3	7.215	7.170	7.191	7.225	7.207	7.253
KIRREL1	8.128	8.051	8.121	7.889	8.029	7.807
STK17B	9.427	9.329	9.567	9.384	9.519	9.487
AC108713.1	7.145	7.262	7.011	7.052	7.408	7.261
AC096644.3	7.371	7.477	7.341	7.465	7.340	7.452
STK17A	9.637	9.845	9.773	9.954	9.835	9.904
ZC3H11A	11.002	10.908	10.983	10.739	10.665	10.802
IAH1	11.227	11.314	11.389	11.320	11.424	11.331
AL355999.1	7.762	7.904	7.790	7.575	7.497	7.479
TMEM225	6.888	6.849	6.773	6.614	6.903	7.082
CYB5R4	8.242	8.257	8.371	8.329	8.418	8.450
CYB5R3	12.040	12.085	12.138	11.850	11.960	11.662
CYB5R2	7.958	8.186	8.447	7.938	7.945	7.993
CYB5R1	10.330	10.452	10.516	10.145	10.230	10.297
SECTM1	7.693	7.790	7.962	7.752	8.057	7.832
AP003170.2	4.269	4.489	4.298	4.169	4.286	4.535
TGFBR3	8.376	8.291	8.349	8.198	8.106	8.240
AC025575.2	4.985	4.916	4.951	5.080	5.109	5.097
SRMS	5.438	5.575	5.582	5.757	5.672	5.758
ASNS	10.043	9.984	10.015	9.568	9.652	9.804
FAHD2A	9.431	9.641	9.714	9.478	9.585	9.534
MLLT10	8.444	8.377	8.519	8.595	8.550	8.610
MLLT11	9.013	9.061	9.003	8.836	8.952	8.909
BCAR4	5.725	5.927	5.919	5.861	6.020	6.244
BCAR1	9.308	9.352	9.519	9.399	9.400	9.340
BCAR3	7.941	7.836	7.860	7.948	7.918	7.871
LINC00518	5.333	5.369	5.371	5.577	5.413	5.421
CD99	10.242	10.365	10.536	10.158	10.213	10.336
LINC00513	6.781	7.001	6.819	7.058	6.979	6.949
LINC00511	9.795	9.840	9.985	9.943	10.001	9.831
CD93	7.407	7.662	7.469	7.397	7.651	7.641
LINC00514	8.585	8.308	8.753	8.795	8.723	8.641
AC025569.1	7.817	7.957	7.879	7.908	8.050	7.633
CYB5RL	8.066	8.096	8.073	7.948	8.067	7.797
DEFB103B	7.175	7.211	7.170	7.304	7.435	7.308
DEFB103A	7.175	7.211	7.170	7.304	7.435	7.308
EGFLAM	6.984	7.074	7.044	6.968	7.140	7.124
LSM12P1	8.897	8.978	9.124	9.030	9.068	8.503
CT47B1	5.635	5.693	5.320	5.666	5.815	5.536
AL162574.3	6.527	6.594	6.309	6.482	6.611	6.474
AC106876.1	8.806	8.937	8.827	8.719	8.861	8.959
JOSD1	10.455	10.490	10.585	10.385	10.270	10.313
JOSD2	8.325	8.349	8.600	8.471	8.799	8.375
FAM234B	8.671	8.559	8.338	8.599	8.571	8.652
PTPA	9.377	9.415	9.530	9.378	9.503	9.252
ADAMTS7P4	7.252	7.226	7.167	7.371	7.396	7.325
ADAMTS7P1	7.252	7.226	7.167	7.371	7.396	7.325
ADAMTS7P3	5.058	5.415	5.425	5.217	5.229	5.218
ROCK1P1	10.599	10.495	10.565	10.515	10.284	10.438
SOX8	5.922	5.919	6.093	6.041	6.165	6.032
SOX9	12.403	12.437	12.708	12.550	12.662	12.419
ANO7L1	7.282	7.171	7.381	7.381	7.533	7.495
AC024060.2	6.677	6.563	6.209	6.542	6.615	6.783

SOX2	7.311	7.143	6.829	7.353	7.262	6.998
SOX3	6.977	6.911	6.953	6.999	6.931	6.913
BBC3	8.517	8.366	8.707	8.762	8.795	8.589
SOX1	5.832	5.700	5.621	5.805	5.788	5.774
SOX6	8.775	8.621	8.767	8.621	8.736	8.765
SOX7	4.554	4.395	4.529	4.294	4.628	4.423
SOX4	11.709	11.702	11.749	11.640	11.685	11.678
SOX5	6.664	6.781	6.591	6.484	6.872	6.922
EMBP1	10.329	10.293	10.375	10.062	10.059	10.086
AC011632.1	4.518	4.513	4.500	4.609	4.737	4.585
KLRC1	4.260	4.306	4.229	4.183	4.317	4.344
KLRC3	4.658	4.629	4.662	4.598	4.742	4.586
KLRC2	4.260	4.306	4.229	4.183	4.317	4.344
KLRC4	3.934	3.938	3.800	4.206	3.864	4.032
TGOLN2	12.050	12.091	12.134	11.868	11.901	11.917
SLC1A5	11.054	11.197	11.318	11.084	11.060	10.986
SLC1A6	7.839	7.901	7.980	8.167	7.906	8.037
LYST	8.188	8.435	8.143	8.255	8.357	8.179
ARHGEF40	8.238	8.224	8.365	8.132	8.211	8.170
AC024958.1	5.032	5.299	5.157	5.062	5.337	5.257
AC239800.1	4.780	4.822	4.745	4.831	4.874	4.812
LURAP1L	11.050	11.106	11.253	11.118	11.120	11.126
NALCN-AS1	6.902	6.924	6.901	7.283	7.077	7.054
AC009264.1	5.402	5.358	5.131	5.373	5.134	5.253
CHRNA9	5.521	5.725	5.461	5.846	5.500	5.823
INHBA	8.239	7.844	7.703	8.497	8.302	9.090
INHBB	6.686	6.653	6.704	6.899	6.949	6.749
INHBC	8.261	8.294	8.042	8.033	8.271	8.396
INHBE	5.593	5.270	5.470	5.242	5.389	4.996
FAM210A	8.198	8.191	8.467	8.243	8.182	8.147
FAM210B	9.630	9.937	10.107	9.597	9.909	9.540
STAU2	7.965	7.893	7.854	8.029	7.724	7.911
CFAP74	8.194	8.519	8.102	8.341	8.352	8.370
CFAP73	7.698	7.681	7.861	7.839	7.978	8.080
CFAP70	4.651	4.615	4.679	4.718	4.569	4.608
AL359260.1	6.254	6.506	6.118	6.444	6.576	6.530
AL353644.3	8.286	7.924	8.206	8.188	8.172	8.003
AP004782.1	6.277	6.511	6.334	6.199	6.346	6.401
FAM111A-DT	8.955	8.955	8.953	8.524	8.870	8.670
AC234917.1	9.147	9.110	9.327	9.054	8.728	9.091
GSPT2	11.076	11.070	11.163	11.060	10.990	10.995
TSHB	5.995	6.107	5.868	6.001	6.045	6.030
CRTC1	9.129	9.145	9.390	9.500	9.345	9.447
CRTC3	9.157	9.144	9.182	8.910	8.992	8.983
CRTC2	9.056	9.161	9.084	8.797	9.114	9.085
TSHR	6.010	5.937	6.031	5.902	5.980	6.022
LY86	7.841	7.830	7.847	7.620	7.826	7.891
SDHAF1	9.673	9.697	9.773	9.523	9.552	9.621
SDHAF2	9.688	9.764	9.858	9.718	9.678	9.754
SDHAF3	7.618	7.994	7.899	7.587	7.956	8.124
SDHAF4	7.190	7.213	7.221	7.158	7.183	7.156
NCALD	7.387	7.566	7.117	7.196	7.294	7.369
AC017100.1	6.775	6.807	6.629	6.773	6.862	6.718
MALL	11.782	11.826	11.915	11.839	11.821	11.771
AC103798.1	3.630	3.526	3.653	3.812	3.648	3.627
SMARCAD1	9.037	8.889	9.013	9.112	9.015	8.931

HECA	8.133	8.100	8.206	7.861	7.705	7.882
AC099689.1	4.355	4.253	4.407	4.259	4.619	4.483
AC099689.2	4.355	4.253	4.407	4.259	4.619	4.483
RALGPS1	7.913	7.910	8.020	7.843	7.921	8.009
AP001931.1	11.155	11.150	11.338	11.068	11.044	11.091
AP001931.2	11.109	11.264	11.331	11.272	11.299	11.152
MAL2	12.042	12.095	12.084	11.976	11.981	12.084
ARV1	9.574	9.663	9.672	9.554	9.482	9.537
AC015849.5	5.420	5.489	5.541	5.247	5.220	5.464
AC015849.4	8.051	8.095	7.557	7.582	7.389	7.862
PRTFDC1	8.592	8.576	8.787	7.834	8.011	7.995
AGTR2	6.677	6.921	6.677	6.926	6.895	6.902
AGTR1	5.583	5.662	5.873	5.603	5.710	5.644
ARHGAP27P1-BF	11.882	11.907	11.917	11.581	11.775	11.682
GGNBP2	11.515	11.531	11.615	11.496	11.196	11.468
AC139493.2	7.522	7.529	7.454	7.353	7.645	7.618
DHRS2	8.041	8.166	8.221	7.944	8.140	8.180
CD4	7.582	7.427	7.548	7.553	7.574	7.592
CD5	8.400	8.531	8.274	8.530	8.440	8.516
CD6	8.743	8.811	8.949	9.015	9.011	9.070
CD7	7.127	7.033	7.239	7.128	7.047	7.042
CD2	8.258	8.223	7.964	8.223	8.349	8.307
KIR3DL1	6.970	7.032	6.960	6.968	7.282	7.016
DHRS1	8.323	8.276	8.221	8.082	8.025	8.160
CDY2A	7.045	7.164	6.836	7.174	7.246	7.298
CD9	10.738	10.879	10.972	10.924	10.881	10.849
CDY2B	7.045	7.164	6.836	7.174	7.246	7.298
AC012676.5	6.949	6.394	6.353	6.934	7.037	6.761
NUSAP1	10.540	10.438	10.613	9.838	10.070	9.986
AC117500.2	7.603	7.777	7.672	7.671	7.694	7.622
AC117500.1	4.982	5.255	5.063	5.150	5.253	5.211
AL359504.2	6.505	6.720	6.366	6.606	6.517	6.742
BTAF1	7.382	7.337	7.378	7.321	7.066	7.420
RHEX	9.446	9.221	9.433	9.146	9.259	9.358
CDA	8.156	8.100	8.252	8.465	8.250	8.264
MRPS27	9.747	9.855	9.893	9.649	9.743	9.893
RHEB	12.016	11.997	12.130	12.031	12.050	12.061
MRPS25	9.266	9.152	9.323	9.015	9.109	9.061
AACS	10.043	10.130	10.231	9.874	9.771	9.912
GTF2IRD2B	8.323	8.301	8.564	7.924	8.144	8.144
ADCYAP1R1	7.483	7.622	7.293	7.505	7.642	7.576
NR1H3	6.979	6.864	6.827	6.960	7.163	7.107
SPANXB1	7.833	7.992	8.035	8.055	8.070	8.173
SLC2A4RG	11.161	11.315	11.512	11.152	11.176	11.176
BAZ1B	9.042	9.057	9.148	9.048	8.901	8.930
ABCC8	7.349	7.493	7.500	7.428	7.336	7.498
ABCC9	6.602	6.613	6.774	6.934	6.578	6.605
ABCC4	8.252	8.266	8.431	8.170	8.280	8.080
ABCC5	8.679	8.820	8.859	8.638	8.688	8.818
ABCC6	7.536	7.684	7.757	7.553	7.471	7.421
COL20A1	7.691	7.718	7.552	7.783	7.757	7.715
ABCC2	6.627	6.695	6.601	6.170	6.633	6.497
ABCC3	10.994	10.866	10.917	10.723	10.550	10.665
MIRLET7D	7.706	7.417	7.408	7.756	7.767	7.756
MIRLET7I	6.866	6.700	6.635	6.469	6.359	6.710
PMP2	4.616	4.426	4.444	4.911	4.661	4.569

AC092306.1	7.943	7.938	7.814	7.919	8.008	8.055
RPL22L1	11.235	11.376	11.525	11.444	11.459	11.464
RYR1	5.875	6.011	5.862	5.799	5.728	6.059
RYR3	4.601	4.749	4.469	4.669	4.811	4.647
RYR2	6.128	6.106	6.118	6.386	6.299	6.277
TBC1D22A	10.010	10.122	10.246	9.826	10.054	10.040
GOLGA1	9.042	9.167	8.974	8.888	8.891	8.956
GOLGA2	10.263	10.294	10.311	10.287	10.295	10.166
GOLGA3	9.888	9.936	10.072	9.929	9.891	9.868
GOLGA4	9.853	9.878	9.942	9.703	9.325	9.690
GOLGA5	9.758	9.839	9.965	9.706	9.653	9.620
GOLGA7	10.838	10.996	11.086	10.801	10.886	10.783
AC011124.1	7.195	7.137	6.820	7.491	7.438	7.244
AC011124.2	6.769	6.653	6.664	6.681	6.692	6.858
SLCO3A1	9.111	8.992	9.049	9.009	9.306	9.029
ENPP6	3.861	3.967	4.081	3.930	3.804	3.837
ENPP7	7.595	7.414	7.350	7.796	7.416	7.372
ENPP4	8.604	8.681	8.811	8.659	8.670	8.846
ENPP5	8.871	8.852	8.980	8.461	8.655	8.685
ENPP2	6.447	6.392	6.219	6.537	6.756	6.440
ENPP3	8.803	8.915	8.835	8.722	8.847	8.903
NAMPT	9.937	10.044	10.088	10.061	10.251	10.049
PTMA	13.414	13.436	13.389	13.470	13.514	13.402
ZZEF1	8.671	8.742	8.801	8.493	8.286	8.595
PTMS	6.900	6.794	7.018	7.337	7.193	6.928
PRORSD1P	5.706	5.714	5.546	5.460	5.408	5.551
DDX3Y	6.899	6.982	6.549	6.612	6.936	6.651
FA2H	11.477	11.548	11.481	11.089	11.155	11.261
BTN3A1	8.394	8.532	8.452	8.508	8.479	8.530
DNM1	8.345	8.228	8.301	8.032	8.064	8.048
DNM3	5.541	5.580	5.767	5.564	5.937	5.454
DNM2	9.998	9.975	10.091	9.797	9.805	9.794
NORAD	12.469	12.518	12.513	12.436	12.280	12.437
TRAP1	10.821	10.895	11.025	10.704	10.718	10.703
AC022532.1	7.356	7.381	7.355	7.285	7.365	7.467
ZNF287	4.403	4.306	4.352	4.253	4.417	4.436
ZNF284	8.500	8.290	8.345	8.344	8.257	8.344
ZNF285	7.865	8.066	7.856	7.759	7.889	7.756
ZNF282	9.001	9.182	9.282	8.976	9.020	9.028
ZNF283	4.853	4.950	4.979	4.715	5.172	4.928
ZNF281	10.175	9.936	10.054	9.722	9.969	9.752
PLPPR4	4.417	4.532	4.434	4.671	4.423	4.564
PLPPR5	6.058	5.778	5.817	6.358	6.104	6.088
PLPPR1	5.872	6.020	5.686	5.856	6.041	5.965
PLPPR2	8.826	9.012	8.934	9.045	8.993	8.980
PLPPR3	7.833	7.912	8.163	8.251	7.960	8.124
CPLANE2	7.729	7.689	7.832	7.790	7.870	7.748
CPLANE1	6.012	5.596	5.493	5.747	5.760	5.591
RFXAP	6.673	6.707	6.688	6.080	6.192	6.166
SNAI1P1	6.146	6.013	5.851	6.405	6.259	6.394
AC024597.1	8.069	8.169	8.075	8.005	8.102	8.141
RAD23B	11.427	11.486	11.544	11.444	11.374	11.341
RAD23A	11.589	11.645	11.704	11.462	11.362	11.356
TBC1D3D	10.803	10.689	10.641	10.716	10.587	10.876
RHOXF2B	7.546	7.991	7.732	7.725	8.072	7.867
SACM1L	9.107	9.175	9.077	8.912	9.125	9.179

IL4I1	7.517	7.578	7.872	7.671	7.746	7.768
AC008393.1	4.153	4.296	4.264	3.931	4.149	3.906
CST11	6.572	6.690	6.348	6.369	6.382	6.363
LINC01917	4.161	4.264	4.084	4.193	3.984	4.166
LINC01913	6.961	6.869	6.577	6.933	6.755	7.022
AC007325.3	9.209	9.151	9.184	9.181	9.090	9.233
AC007325.2	7.830	7.887	7.936	7.808	7.956	7.809
AC007325.4	10.503	10.564	10.866	10.550	10.504	10.580
IFNA8	4.391	4.524	4.267	4.463	4.445	4.568
IFNA2	4.948	4.547	4.644	4.776	4.792	4.520
IFNA1	9.392	8.929	9.080	9.326	9.257	9.306
IFNA7	6.266	6.381	6.168	6.329	6.403	6.205
IFNA6	5.450	5.280	5.279	5.609	5.413	5.588
IFNA5	6.176	6.111	5.706	6.247	6.421	6.376
IFNA4	6.088	5.946	5.929	5.962	6.065	5.988
TIMM29	7.757	7.946	8.186	7.612	7.686	7.688
AC171558.5	4.957	5.051	4.936	4.868	4.936	5.007
AC171558.1	4.957	5.142	4.936	4.954	4.989	5.132
TIMM22	8.578	8.672	8.848	8.691	8.991	8.674
TIMM21	9.920	9.886	9.927	9.825	9.877	9.828
CRYAB	6.998	6.994	6.901	6.925	7.068	7.081
SLC33A1	10.802	10.896	10.886	10.971	10.740	10.961
ELF1	10.850	10.695	10.838	10.536	10.820	10.447
ELF3	11.429	11.527	11.722	11.742	11.567	11.676
ELF2	8.629	8.759	8.672	8.433	8.540	8.597
ELF5	6.698	6.960	6.599	6.697	6.825	6.762
ELF4	9.394	9.269	9.304	9.177	9.166	9.126
LINC00319	6.768	6.561	6.654	6.798	6.995	6.666
MIR3149	5.569	5.673	5.558	5.664	5.633	5.533
UPK3B	7.926	7.982	8.240	8.057	8.052	7.952
AL157831.2	6.024	6.105	5.812	6.431	6.094	6.184
UPK3A	6.972	6.726	6.736	6.818	6.778	6.838
AC008114.1	5.033	5.165	5.178	5.124	5.094	5.066
LDHD	5.963	5.822	6.189	6.012	6.064	5.899
GMPR2	9.932	9.996	10.169	9.798	9.706	9.828
LDHA	13.989	14.054	14.034	14.125	14.099	14.165
LDHB	13.710	13.721	13.754	13.664	13.598	13.679
LDHC	7.682	7.868	7.770	8.078	7.805	7.883
AL121899.2	6.602	6.422	6.141	6.639	6.799	6.632
AL121899.1	7.594	7.133	7.379	7.497	7.371	7.295
SLFN11	8.055	8.040	8.021	7.843	7.676	7.809
AL596244.1	8.034	8.159	8.197	7.939	7.956	7.901
CFL2	9.131	9.108	9.142	8.937	9.041	9.118
CFL1	14.210	14.220	14.223	14.241	14.204	14.224
DNM1L	9.883	9.944	9.856	9.663	9.763	9.515
FOXD1	8.606	8.592	8.755	8.439	8.517	8.458
PLEKHG2	9.291	9.543	9.297	9.286	9.456	9.368
PLEKHG3	9.069	9.129	9.110	9.123	9.107	9.048
PLEKHG1	7.177	7.345	7.302	6.959	7.191	7.244
PLEKHG6	8.373	8.383	8.437	8.343	8.405	8.324
PLEKHG7	4.823	4.970	4.933	4.962	4.947	5.070
PLEKHG5	8.028	7.859	8.169	8.585	8.400	8.202
FOLR1	8.049	7.956	8.086	7.771	7.913	8.008
AC004076.2	6.817	6.803	6.728	6.963	6.908	6.831
AC004076.1	5.964	5.729	5.686	5.930	5.976	5.784
AC010642.2	8.670	8.816	8.851	8.771	9.090	8.923

SERPINH1	10.087	10.192	10.360	10.364	10.485	10.235
FOLR3	7.405	7.213	7.460	7.435	7.462	7.283
RNF14	8.635	8.791	8.814	8.707	8.722	8.766
RNF17	6.546	6.534	6.118	6.543	6.443	6.428
RNF10	10.767	10.840	10.985	10.764	10.824	10.800
RNF11	10.790	10.781	10.759	10.692	10.586	10.868
RNF13	11.672	11.765	11.916	11.959	11.997	11.689
LRRC37A16P	6.160	6.530	6.310	6.367	6.412	6.472
CAMK1	6.021	6.143	6.222	6.147	6.072	6.164
CAMK4	6.973	6.991	6.820	7.003	6.852	6.826
HMGCLL1	6.490	6.347	6.083	6.414	6.557	6.384
FOXQ1	11.035	11.040	11.172	11.016	11.051	10.785
CAMKV	7.211	7.233	7.400	7.236	7.350	7.336
COL7A1	8.470	8.294	8.263	8.576	8.421	8.487
AC068491.3	6.921	6.997	6.919	6.878	6.744	6.877
RIT2	4.682	4.531	4.704	4.633	4.438	4.671
RIT1	8.537	8.522	8.660	8.490	8.526	8.718
AL513190.1	6.275	6.435	6.457	6.522	6.439	6.504
ZNF37BP	6.098	6.153	6.526	6.594	6.232	6.414
HRH4	6.344	6.193	6.081	6.213	6.436	6.155
HRH2	7.664	7.790	7.748	8.086	7.842	7.929
HRH3	8.528	8.338	8.516	8.620	8.862	8.683
HRH1	7.988	8.179	8.009	8.054	8.222	8.000
TPRN	9.972	9.996	10.062	9.735	9.742	9.932
GTF2IRD1	9.487	9.484	9.548	9.389	9.392	9.395
COL4A6	6.774	6.829	7.042	6.829	6.856	7.004
COL4A5	8.368	8.123	8.310	7.751	7.757	8.093
COL4A4	8.375	8.300	8.302	8.128	8.212	7.928
COL4A3	7.357	7.296	7.336	7.230	7.507	7.509
COL4A2	6.503	6.712	6.727	6.855	6.668	6.577
COL4A1	6.513	6.774	6.583	6.494	6.820	6.731
LINC02032	5.773	5.744	5.553	5.709	5.896	5.790
LINC02036	5.919	6.126	5.824	5.787	5.966	6.032
LINC02035	7.710	7.274	7.552	7.155	7.156	7.513
HLCS	7.988	7.926	8.241	8.234	8.023	8.069
COX7C	12.688	12.743	12.897	12.851	12.704	12.887
HLF	6.768	6.679	6.606	6.431	6.238	6.508
RNA5SP530	8.342	8.297	8.366	8.512	8.474	8.509
TMPRSS11B	4.800	4.913	4.929	4.955	5.129	5.104
TMPRSS11D	7.664	7.576	7.774	7.744	7.617	7.736
TMPRSS11E	5.130	5.155	4.932	5.405	5.047	5.167
AL139237.1	8.365	8.397	8.108	8.381	8.391	8.387
TUBB7P	6.658	6.482	6.346	6.841	6.717	6.647
TMEM213	6.586	6.661	6.748	6.274	6.388	6.315
COX4I2	8.176	8.125	8.166	8.247	7.937	8.345
COX4I1	12.896	12.964	13.039	12.974	12.872	12.958
TMEM212	6.846	6.785	6.801	6.844	6.868	6.979
EDA	8.448	8.205	8.416	8.541	8.415	8.337
CCDC116	7.230	7.290	7.196	7.126	7.228	7.213
CCDC117	9.807	9.937	9.974	9.825	9.842	10.041
CCDC114	5.962	5.806	5.931	5.809	5.680	5.877
CCDC115	10.290	10.346	10.462	10.009	10.015	10.059
CCDC112	6.428	6.466	6.235	6.211	6.336	6.277
CCDC113	4.916	5.298	5.142	4.990	4.930	5.014
CCDC110	4.672	4.828	4.567	4.992	4.996	4.923
UBXN10-AS1	7.763	7.971	7.986	7.865	7.876	7.908

AC116362.2	5.861	6.094	5.839	5.837	6.119	6.228
BPESC1	4.005	4.040	4.013	4.103	3.931	4.131
AC012184.2	9.664	9.832	9.852	9.649	9.662	9.596
IPO13	8.684	8.811	8.938	8.750	8.866	8.843
ZNF780A	7.825	7.787	7.853	7.678	7.538	7.681
ZNF780B	7.394	7.630	7.583	7.197	7.538	7.415
FRG1	8.645	8.715	8.824	8.474	8.590	8.485
C20orf141	8.276	8.239	8.393	8.594	8.485	8.403
STARD7-AS1	7.318	7.238	7.426	7.067	7.478	7.247
MCM10	8.870	8.858	8.935	8.396	8.642	8.593
IGFBPL1	6.572	6.883	7.119	6.687	7.037	7.058
LRGUK	6.199	6.118	6.147	6.136	5.975	6.103
OR5P3	6.336	6.348	6.316	6.313	6.367	6.451
OR5P2	6.994	6.825	6.813	6.989	6.880	6.838
ATP6V0E1	12.436	12.494	12.631	12.548	12.419	12.497
ATP6V0E2	9.643	9.753	9.847	9.450	9.435	9.416
TMEM214	9.955	9.871	10.049	9.926	9.913	9.875
AC100810.1	8.329	8.449	8.590	8.091	8.440	8.180
AC100810.3	4.784	5.053	5.051	4.901	5.251	4.930
AC100810.4	8.329	8.449	8.590	8.091	8.440	8.180
AC100810.5	4.784	5.053	5.051	4.901	5.251	4.930
AL121985.1	6.826	6.856	6.928	6.846	6.617	6.981
SIX4	8.109	8.171	8.268	8.053	8.267	8.103
SIX5	8.585	8.670	8.872	8.630	8.756	8.698
SIX6	6.961	6.862	6.733	7.224	7.044	6.880
SIX1	7.852	8.045	8.029	7.924	7.996	7.784
SIX2	6.602	6.572	6.618	6.640	6.762	6.633
SIX3	6.528	6.343	6.426	6.715	6.643	6.683
LINC00308	6.343	6.226	6.055	6.290	6.491	6.400
LINC00309	4.804	4.689	4.518	4.802	4.448	4.819
PLCZ1	5.289	5.375	5.680	5.719	5.612	5.459
BX679664.3	13.304	13.343	13.490	13.360	13.298	13.417
KNDC1	7.193	7.127	7.054	7.314	7.220	7.331
LINC00307	4.932	4.939	4.918	4.953	5.152	4.895
LINC00304	7.157	7.325	7.168	7.265	7.294	7.364
LINC00305	4.477	4.379	4.290	4.566	4.496	4.552
C3orf79	3.988	4.059	4.036	4.033	4.105	4.148
C3orf70	5.138	5.046	5.047	4.992	5.030	5.010
MARCHF8	8.306	8.237	8.410	7.948	7.899	8.064
MARCHF9	9.325	9.404	9.579	9.292	9.503	9.406
MARCHF1	5.427	5.310	5.365	5.497	5.527	5.417
MARCHF2	8.465	8.530	8.745	8.326	8.329	8.463
MARCHF3	7.707	7.782	7.581	7.615	7.808	7.696
MARCHF4	6.879	7.123	7.242	7.355	7.153	7.172
MARCHF5	9.309	9.321	9.359	9.214	9.226	9.357
MARCHF6	10.313	10.417	10.460	10.386	10.340	10.332
MARCHF7	9.725	9.747	9.834	9.418	9.813	9.637
FAM81A	8.508	8.489	8.502	8.213	8.215	8.173
UQCRC2	11.532	11.569	11.690	11.534	11.428	11.531
XPNPEP1	10.698	10.818	10.907	10.602	10.680	10.572
XPNPEP2	7.019	7.083	7.260	7.411	7.309	7.010
UQCRC1	11.469	11.604	11.759	11.557	11.443	11.444
GGCX	9.400	9.402	9.416	9.388	9.440	9.411
PAPLN	7.239	7.232	7.267	7.437	7.100	7.285
RETSAT	11.537	11.598	11.767	11.480	11.403	11.386
KHDRBS1	12.423	12.493	12.590	12.260	12.178	12.251

KHDRBS3	9.069	9.238	9.484	9.055	9.355	9.218
GGCT	11.805	11.948	12.020	11.708	11.555	11.665
AP000705.5	6.343	6.226	6.055	6.290	6.491	6.400
LHCGR	4.100	4.148	4.220	4.480	4.261	4.278
AC013460.1	7.300	7.434	7.488	7.251	7.574	7.562
OR1Q1	7.468	7.713	7.630	7.372	7.585	7.634
INF2	9.917	9.963	10.161	9.930	10.148	9.874
AL353709.1	5.891	5.877	5.866	5.952	6.103	6.065
ZSWIM5	6.881	6.772	6.677	6.937	6.824	7.064
KRTAP10-12	6.914	7.474	7.267	6.793	7.186	7.235
KRTAP10-11	8.082	8.053	8.154	8.081	8.317	8.518
BOP1	10.009	10.037	10.162	9.876	9.724	9.874
GJA1	11.535	11.503	11.446	11.419	11.312	11.383
GJA3	7.718	7.553	7.727	7.616	7.608	7.807
GJA4	8.233	8.147	7.987	8.266	8.267	8.155
GJA5	6.333	6.265	6.149	6.299	6.453	6.179
AC022916.2	6.192	6.286	6.108	6.290	6.403	6.137
GJA8	8.168	7.976	8.154	8.048	8.256	8.187
GJA9	6.576	6.610	6.400	6.551	6.660	6.551
PRR7-AS1	7.440	7.381	7.372	7.600	7.452	7.481
AL592188.2	8.286	7.924	8.206	8.188	8.172	8.003
AL592188.3	8.286	7.924	8.206	8.188	8.172	8.003
KDM3B	10.232	10.239	10.281	9.976	9.924	10.116
KDM3A	9.609	9.692	9.774	9.570	9.432	9.595
AC011468.5	6.668	7.080	6.869	7.077	6.989	6.742
EIF2B4	9.648	9.720	9.867	9.551	9.548	9.614
EIF2B5	9.770	9.654	9.897	9.672	9.604	9.718
EIF2B2	9.448	9.373	9.454	9.266	9.158	9.264
EIF2B3	9.005	9.156	9.231	9.164	9.082	9.136
EIF2B1	10.494	10.565	10.605	10.420	10.333	10.320
ZKSCAN2-DT	7.033	6.792	6.829	7.054	6.986	7.155
QPRT	8.356	8.277	8.154	8.466	8.381	8.259
IFT140	7.349	7.469	7.338	7.511	7.580	7.658
CDRT15P1	7.226	7.176	7.129	7.251	7.324	7.386
TMEM14B	11.497	11.554	11.614	11.559	11.704	11.507
TMEM14A	10.677	10.613	10.855	10.608	10.624	10.654
AC117377.1	7.192	7.040	6.914	7.239	7.305	7.155
LURAP1	7.325	7.435	7.517	7.407	7.529	7.520
TPBGL	8.203	8.320	8.384	8.428	8.373	8.410
MYT1L	5.293	5.302	5.367	5.468	5.358	5.273
AC254562.2	8.206	8.266	8.340	8.286	8.325	8.436
CFHR3	5.253	5.364	5.169	5.217	5.360	5.255
CFHR1	8.274	8.178	8.217	8.142	8.003	8.163
CFHR4	5.147	5.364	5.014	5.168	5.177	5.078
CFHR5	4.158	4.521	4.445	4.329	4.447	4.589
LRFN5	6.660	6.758	6.592	6.775	6.821	6.699
TMEM147	11.507	11.611	11.784	11.648	11.577	11.448
TMEM145	7.293	7.224	7.455	7.325	7.324	7.489
TMEM144	8.129	8.171	8.253	7.939	7.730	7.988
FOXL2	6.339	6.225	6.077	5.992	6.194	6.039
TMEM141	9.741	9.704	9.955	9.585	9.539	9.713
FOXL1	9.243	9.368	9.413	9.062	9.138	9.101
RTKN2	4.076	4.253	4.472	4.072	3.835	3.884
PSMB8-AS1	7.344	7.403	7.456	7.273	7.478	7.510
FAM32A	9.924	10.021	10.165	9.934	9.752	9.932
FER1L4	9.653	9.599	9.578	9.624	9.510	9.813

FER1L5	5.094	5.099	4.966	5.136	5.081	5.019
BARX1-DT	7.781	7.938	7.872	8.042	7.961	7.797
ZMAT1	5.463	6.015	5.626	5.923	5.680	5.701
ZMAT3	9.249	9.208	9.218	9.217	9.214	9.144
AC012314.18	11.667	11.674	11.756	11.512	11.455	11.474
ZMAT5	8.445	8.658	8.670	8.691	8.705	8.491
ZMAT4	7.848	7.951	7.859	7.678	8.002	7.873
AC012314.13	10.357	10.410	10.540	10.053	10.003	10.044
AC012314.12	10.357	10.410	10.540	10.053	10.003	10.044
SMOC1	9.291	9.336	9.428	9.234	9.314	9.425
AC012314.10	11.667	11.674	11.756	11.512	11.455	11.474
AC012314.17	11.667	11.674	11.756	11.512	11.455	11.474
AC012314.16	10.357	10.410	10.540	10.053	10.003	10.044
AC012314.15	10.357	10.410	10.540	10.053	10.003	10.044
AC012314.14	11.667	11.674	11.756	11.512	11.455	11.474
CR759772.1	8.439	8.433	8.212	8.323	8.555	8.407
C11orf16	9.198	9.098	8.933	9.388	9.414	9.466
MYLK-AS1	7.543	7.690	7.497	7.649	7.615	7.705
AC092127.1	7.335	7.557	7.223	7.594	7.372	7.540
AC245297.3	4.232	4.165	4.506	4.480	4.299	4.279
AC245297.2	5.590	5.663	5.442	5.619	5.476	5.643
AC245297.1	4.486	4.626	4.587	4.711	4.614	4.633
AC245297.4	7.865	7.879	8.165	7.721	8.167	7.814
CCDC185	5.563	5.558	5.603	5.745	5.713	5.451
CADPS	6.861	6.862	6.965	7.236	6.937	7.018
BX664615.2	5.375	5.478	5.721	5.375	5.628	5.606
AC245452.1	6.477	6.418	6.471	6.373	6.374	6.398
AC139272.1	4.268	4.234	4.359	4.378	4.576	4.384
TUFM	12.227	12.368	12.507	12.265	12.113	12.173
C1QTNF1-AS1	6.440	6.536	6.680	6.885	6.448	6.781
MAMSTR	8.658	8.533	8.588	8.567	8.440	8.654
PDF	9.800	9.744	9.879	9.579	9.530	9.540
FAM66D	7.432	7.219	7.300	7.196	7.238	7.473
FAM66C	5.177	5.175	5.138	5.245	5.279	5.155
AL390879.1	6.655	6.878	6.720	6.631	6.553	6.610
CPPED1	6.551	6.736	6.730	6.925	6.785	6.835
OSER1-DT	7.851	7.960	7.982	7.878	8.104	8.061
SRPX2	8.635	8.848	8.883	8.461	8.540	8.676
MBNL1	12.031	11.934	12.046	11.900	11.911	11.683
MBNL2	8.515	8.445	8.311	8.208	8.158	8.377
MBNL3	9.854	9.766	9.659	9.428	9.312	9.356
A1BG	6.625	6.663	6.769	6.609	6.870	6.676
AL031777.2	7.447	7.364	7.916	7.833	7.685	7.781
AC092139.2	7.942	8.017	7.974	8.104	8.133	8.163
AC145676.2	5.044	4.799	5.011	5.109	5.102	5.180
HNRNPA3P5	11.051	11.098	11.160	10.846	10.830	10.950
MYCBP	9.431	9.659	9.711	9.592	9.582	9.639
HNRNPA3P3	11.051	11.098	11.160	10.846	10.830	10.950
C16orf74	7.547	7.693	7.862	7.628	7.505	7.718
C16orf70	8.382	8.542	8.619	8.416	8.568	8.426
C16orf71	6.861	7.084	7.212	7.286	7.399	7.405
C16orf72	10.697	10.692	10.815	10.618	10.620	10.723
LYRM4	9.195	9.190	9.420	9.131	9.151	9.240
C16orf78	6.359	6.281	6.534	6.860	6.734	6.612
HABP4	8.791	8.783	8.919	8.979	8.988	8.798
ZNF853	7.912	7.765	7.981	7.814	7.819	7.922

ZNF852	3.852	4.003	3.946	3.892	4.026	3.874
HABP2	8.971	9.046	9.051	8.699	8.735	8.797
ZNF850	7.266	6.986	6.993	6.484	6.656	6.757
NDRG1	10.422	10.563	10.753	10.588	10.578	10.591
NDRG2	8.809	8.649	8.840	8.583	8.690	8.707
NDRG3	9.641	9.619	9.637	9.479	9.393	9.505
NDRG4	7.134	7.141	6.917	6.793	6.922	6.962
SRSF5	11.264	11.252	11.375	10.948	10.685	11.028
SRSF4	10.959	10.941	11.094	10.849	10.943	10.705
SRSF7	11.636	11.651	11.847	11.819	11.663	11.745
SRSF6	10.886	10.926	10.952	10.842	10.631	10.982
SRSF1	11.305	11.351	11.289	11.273	11.310	11.265
SRSF3	11.914	11.918	12.084	12.065	12.008	12.021
SRSF2	11.676	11.647	11.731	11.405	11.415	11.396
SRSF9	12.514	12.566	12.758	12.505	12.350	12.323
SRSF8	10.542	10.728	10.722	10.505	10.610	10.573
CISD2	9.336	9.556	9.561	9.367	9.359	9.374
CISD3	9.896	9.984	10.263	9.988	10.025	10.033
CISD1	11.047	11.210	11.434	11.058	11.293	10.799
CLNS1A	10.677	10.740	10.909	10.594	10.600	10.597
ZNF581	10.384	10.495	10.588	10.279	10.295	10.207
ZNF580	10.067	9.994	10.175	9.984	9.986	9.851
ZNF583	7.221	6.996	7.059	7.006	7.097	7.084
ZNF582	7.879	7.871	7.795	7.873	7.949	8.043
TIPARP	10.117	10.106	10.255	10.459	10.420	10.450
ZNF584	8.200	8.339	8.346	8.277	8.372	8.393
AC055874.1	8.475	8.444	8.209	8.374	8.379	8.433
ZNF586	8.397	8.253	8.344	8.253	8.189	8.255
ZNF589	8.326	8.377	8.168	8.140	8.238	8.234
AC011476.4	8.340	8.224	8.370	8.418	8.070	8.323
AP001122.1	5.316	5.233	5.148	5.383	5.316	5.551
MTRF1L	9.729	9.501	9.553	9.718	9.426	9.848
SRMP1	6.470	6.653	6.838	6.604	6.744	6.672
ZER1	8.939	8.969	9.054	8.908	8.921	8.886
FAM160B1	7.028	7.140	7.064	6.805	6.877	6.956
FAM160B2	8.791	8.917	9.047	8.703	8.633	8.768
TTYH1	6.647	6.848	6.806	6.745	6.695	6.936
TSSK6	7.420	7.503	7.510	7.694	7.774	7.638
SRC	11.191	11.212	11.346	11.123	11.205	11.166
TSSK4	7.190	7.221	7.064	7.132	7.147	7.004
ZNF326	8.438	8.533	8.436	8.179	8.316	8.428
SRF	8.276	8.288	8.368	8.245	8.251	8.230
TSSK3	9.057	9.106	9.071	8.905	9.122	9.314
ZNF322	7.997	7.899	8.031	7.885	7.953	7.916
SRI	10.681	10.814	10.971	10.811	10.802	10.684
ZNF329	7.520	7.544	7.604	7.280	7.629	7.667
SRL	7.031	6.968	7.140	7.268	7.019	7.004
SRM	9.988	10.006	10.130	9.935	9.861	9.798
SRR	8.509	8.384	8.430	8.390	8.506	8.271
FAM220A	10.297	10.402	10.494	10.251	10.212	10.183
SRY	6.567	6.269	6.208	6.361	6.552	6.420
EIF2AK1	12.307	12.323	12.323	12.211	12.148	12.093
EIF2AK3	9.257	9.341	9.280	9.237	9.232	9.292
EIF2AK2	11.067	11.062	10.914	10.759	10.783	10.796
EIF2AK4	7.154	7.265	7.151	6.975	6.913	7.016
FAM136A	10.308	10.498	10.520	10.341	10.355	10.377

PLAUR	9.765	9.978	9.987	10.092	10.188	10.005
LOR	7.374	7.043	7.253	7.587	7.498	7.394
SLCO1B3	8.684	8.730	8.715	8.645	8.706	8.907
LOX	6.290	6.566	6.688	6.570	6.773	6.778
TIMM17B	10.574	10.707	10.880	10.682	10.706	10.601
TIMM17A	10.593	10.637	10.748	10.685	10.430	10.561
AL162417.1	8.353	8.249	8.527	8.129	8.381	8.154
AL359921.1	6.387	6.342	6.297	6.475	6.616	6.601
GSN-AS1	7.436	7.506	7.217	7.610	7.534	7.591
PIGA	9.022	9.044	9.083	9.114	8.994	9.198
PIGB	8.846	8.700	8.806	8.387	8.378	8.439
PIGC	10.101	10.072	10.194	10.146	10.080	10.081
PIGF	9.464	9.500	9.588	9.208	9.390	9.346
PIGG	9.812	9.817	9.789	9.667	9.707	9.809
PIGH	8.822	8.940	9.056	8.748	8.848	8.734
PIGK	9.607	9.671	9.725	9.269	9.327	9.404
PIGL	8.101	8.192	8.187	8.133	8.371	8.146
PIGM	9.750	9.786	9.702	9.568	9.748	9.814
PIGN	8.889	8.867	9.004	8.755	8.551	8.604
PIGO	10.077	10.163	10.327	9.915	10.021	10.021
PIGP	10.778	10.968	11.053	10.916	10.845	10.781
PIGQ	9.091	9.072	8.959	9.153	9.160	9.170
AC235565.2	6.784	6.838	6.902	6.852	7.061	6.978
PIGS	11.047	11.177	11.232	10.965	10.863	10.781
PIGT	10.396	10.440	10.546	10.273	10.133	10.152
PIGU	10.354	10.491	10.647	10.282	10.139	10.283
PIGV	8.815	8.962	9.066	8.742	8.743	8.832
PIGW	9.216	9.353	9.143	8.412	8.676	9.003
PIGX	9.796	9.935	10.074	9.592	10.009	9.542
PIGZ	8.903	9.058	8.974	8.347	8.565	8.508
RPS2P45	4.868	4.880	4.812	4.743	4.928	4.784
FAM47B	4.342	4.642	4.429	4.736	4.630	4.404
LINC01278	8.236	8.047	8.171	7.925	7.695	7.847
BBIP1	8.801	8.905	8.890	8.543	8.660	8.602
LINC01271	6.917	7.147	7.171	7.145	7.213	7.221
LINC01270	6.469	6.723	6.593	6.450	6.399	6.454
RAB9BP1	4.495	4.611	4.741	4.776	4.665	4.963
PUF60	11.470	11.543	11.843	11.367	11.317	11.369
IGKV1-37	7.315	7.353	7.191	7.351	7.391	7.278
IGKV1-39	6.932	6.916	6.865	6.927	7.139	7.023
PLVAP	7.711	7.729	7.589	7.712	7.864	7.722
MIR1-1HG	8.194	8.480	8.472	8.229	8.435	8.643
NTRK1	5.262	5.047	5.055	5.270	5.448	5.248
NTRK2	8.193	8.281	8.165	8.199	8.447	8.198
NTRK3	8.284	8.352	8.234	8.491	8.590	8.388
SAP18	11.364	11.409	11.597	11.514	11.302	11.395
STPG3-AS1	7.245	7.193	7.263	7.110	7.397	7.275
AC102941.1	6.523	6.786	6.822	6.596	6.788	6.588
AP000593.2	5.475	5.382	5.296	5.197	5.178	5.347
COL19A1	5.888	6.190	6.225	5.969	6.029	6.231
LINC01898	6.859	6.818	6.831	7.016	7.028	7.077
UMODL1-AS1	6.151	6.154	6.084	6.094	6.257	6.429
ATG2A	9.157	9.279	9.298	8.990	8.917	9.025
AC234778.1	11.439	11.480	11.673	11.542	11.429	11.546
ATG2B	8.606	8.697	8.652	8.187	8.320	8.350
LINC01896	6.743	6.964	6.935	6.971	6.956	6.971

GPR3	7.232	7.109	7.361	7.255	7.236	7.120
GPR1	3.935	4.011	4.003	4.022	3.810	3.935
GPR6	7.937	8.015	8.060	7.846	8.057	7.971
GPR4	6.988	6.953	7.172	7.308	7.168	7.342
C1orf35	8.937	8.951	9.156	8.847	8.781	8.930
AL021392.1	7.831	7.884	8.095	8.248	8.014	7.978
SWT1	6.635	6.481	6.652	6.558	6.692	6.800
DOCK8-AS1	6.326	6.314	6.509	6.554	6.290	6.286
ZNF519	7.100	7.303	6.837	7.126	7.264	7.243
AL118505.1	4.020	3.918	3.814	3.970	4.120	3.801
PTOV1	11.083	11.106	11.441	11.193	11.282	11.056
AC116533.1	13.408	13.485	13.653	13.701	13.471	13.603
FKTN	7.389	7.394	7.472	7.427	7.310	7.629
NHP2	11.891	11.964	12.136	11.851	11.859	11.840
MIR142	7.329	7.469	7.529	7.826	7.364	7.621
MIR141	7.375	7.617	7.617	7.574	7.660	7.727
AL118511.1	5.980	5.729	5.720	6.125	6.041	5.880
RNU6-244P	6.783	6.945	6.922	6.922	6.792	6.892
TPRKB	10.055	10.151	10.168	9.716	9.766	9.720
OR7E36P	9.849	9.926	10.029	9.810	9.880	9.911
AC120045.2	7.535	7.744	7.432	7.615	7.672	7.655
HNF4A	9.301	9.227	9.275	9.001	9.264	9.052
AC008676.3	8.175	8.149	8.135	8.252	8.292	8.345
MS4A6E	4.513	4.724	4.700	4.854	4.553	4.796
H2AC17	6.553	6.418	6.750	6.891	6.792	7.003
H2AC16	5.446	5.380	5.568	5.543	5.337	5.308
H2AC15	5.608	5.690	5.724	5.871	5.887	5.621
H2AC14	8.640	8.483	8.650	8.681	8.705	8.764
H2AC13	8.640	8.483	8.650	8.681	8.705	8.764
H2AC11	5.380	5.340	5.694	5.195	5.169	5.426
SOWAHCP2	6.361	6.626	6.537	6.407	6.447	6.518
MIR1226	9.139	9.328	9.427	9.213	9.267	9.123
H2AC19	11.168	11.355	11.404	11.580	11.423	11.436
H2AC18	11.168	11.355	11.404	11.580	11.423	11.436
ZNF571-AS1	7.639	7.665	7.602	7.603	7.418	7.726
HELLS	9.226	9.208	9.342	8.606	8.884	8.398
AKAP17A	8.897	8.918	8.933	8.852	8.950	8.947
PDILT	5.955	6.252	6.026	6.102	5.997	6.032
AL049597.2	6.060	5.701	5.771	5.894	5.741	5.973
TEDC2	8.524	8.673	8.657	8.394	8.510	8.493
ISM1-AS1	5.577	5.503	5.638	5.841	5.738	5.809
AL136526.1	5.697	5.482	5.593	5.647	5.776	5.686
HDHD5	10.472	10.548	10.761	10.399	10.458	10.547
TMA7	12.359	12.429	12.496	12.306	12.282	12.215
HDHD3	8.891	9.002	9.271	8.956	9.069	8.951
HDHD2	8.840	8.972	8.960	8.579	8.528	8.710
OR9A1P	4.471	4.309	4.112	4.471	4.399	4.441
AL133399.1	6.718	6.765	6.702	6.891	6.854	6.796
TBC1D31	7.303	7.284	7.433	6.786	6.667	6.841
MBL2	4.396	4.571	4.617	4.867	4.538	4.785
SNORD23	12.815	12.922	13.138	12.703	12.544	12.636
SNORD22	9.959	9.803	9.788	9.844	9.766	9.997
MLH3	8.036	7.987	8.132	7.965	7.955	7.954
CRY1	8.813	8.912	8.688	8.531	8.362	8.499
CRY2	8.734	8.804	8.891	8.942	8.934	8.991
ZNF517	6.103	6.065	6.186	6.100	6.447	6.228

CRYZ	11.699	11.732	11.841	11.798	11.660	11.753
CRYM	8.986	9.066	9.193	9.011	9.021	9.194
AL137796.1	6.769	6.802	6.641	6.429	6.655	6.709
AC073534.1	8.373	8.331	8.357	7.785	7.910	7.859
PRLHR	5.788	5.906	5.944	5.971	6.186	5.990
AC116366.3	8.328	8.483	8.449	8.293	8.214	8.335
AC005772.1	4.445	4.558	4.381	4.548	4.363	4.633
LINC02204	5.703	6.032	5.759	5.862	5.569	5.907
LINC02201	6.082	5.900	6.209	6.381	6.490	6.470
AL355581.1	4.774	4.650	4.738	4.911	4.620	4.724
LINC02203	5.651	5.789	5.544	5.480	5.658	5.651
WEE1	9.818	9.638	9.760	9.728	9.796	9.759
LINC02209	6.878	6.886	6.853	7.002	6.986	7.112
AKAP4	6.386	6.461	6.713	6.582	6.577	6.854
AKAP5	6.694	6.719	6.662	7.054	6.761	6.784
AKAP6	6.600	6.830	6.355	6.728	6.678	6.820
AKAP7	8.366	8.473	8.586	8.670	8.649	8.591
AKAP1	11.042	11.079	11.192	10.768	11.080	10.819
AKAP3	7.000	7.242	7.015	7.136	7.229	7.340
EIF2D	9.663	9.593	9.741	9.343	9.453	9.364
AKAP8	9.192	9.125	9.048	9.115	9.018	9.136
AKAP9	8.656	8.643	8.547	8.215	7.902	8.426
EIF2A	11.946	12.043	12.143	12.029	11.953	11.944
AC004039.1	5.688	5.746	5.375	5.635	5.582	5.887
AC133485.2	3.826	3.885	3.772	3.930	3.832	3.885
TUBE1	6.594	6.484	6.581	6.452	6.614	6.621
MIR3142HG	6.641	6.892	6.771	7.170	6.864	6.668
TMEM98	7.439	7.511	7.554	7.494	7.444	7.436
TMEM99	12.134	12.140	12.333	12.130	12.163	12.138
PMFBP1	6.945	7.140	7.167	6.935	7.238	7.121
OVGP1	8.429	8.741	8.708	8.517	8.634	8.627
CETN3	9.266	9.284	9.368	9.058	9.232	9.236
CETN2	10.867	10.894	11.093	10.863	10.783	10.991
CETN1	8.238	8.394	7.910	8.013	8.022	8.184
LTC4S	6.952	6.954	7.095	7.051	6.858	6.998
PCLO	7.688	7.579	7.523	7.417	7.427	7.530
AC018445.2	5.045	5.183	5.002	5.027	5.136	4.892
UCK1	9.239	9.382	9.506	9.200	9.064	9.247
UCK2	10.014	10.070	10.165	10.074	10.026	10.092
PHRF1	8.779	8.753	9.003	8.732	8.660	8.721
MAP3K9	8.785	8.900	8.822	8.893	9.082	8.727
FSIP2	3.737	3.708	3.958	3.836	3.989	3.866
FSIP1	4.486	4.198	4.353	4.495	4.388	4.307
DNAJC8	10.156	10.262	10.359	10.136	10.040	10.156
DNAJC9	10.297	10.231	10.351	9.893	10.088	10.088
DNAJC4	8.687	8.742	8.861	8.748	8.781	8.671
DNAJC5	10.710	10.848	11.020	10.706	10.715	10.660
DNAJC6	7.326	7.155	7.408	6.957	6.756	7.088
DNAJC7	10.309	10.464	10.493	10.066	10.173	10.294
DNAJC1	10.215	10.416	10.601	10.420	10.538	9.880
DNAJC2	8.076	8.104	8.252	8.052	7.871	7.855
DNAJC3	9.779	9.823	9.981	9.451	9.628	9.288
GOSR2	8.932	8.875	8.924	8.887	8.823	8.740
GOSR1	10.256	10.395	10.487	10.298	10.328	10.246
NRN1	5.486	5.338	5.453	5.362	5.448	5.358
DEAF1	8.614	8.740	8.880	8.402	8.566	8.516

PCARE	7.011	6.885	7.003	7.332	7.509	7.030
AC098657.2	4.879	4.735	4.816	4.936	4.812	4.836
LMX1A-AS2	8.325	8.244	8.400	8.266	8.318	8.361
LMX1A-AS1	6.821	6.659	6.772	7.029	6.805	6.879
AC093278.2	4.966	4.904	4.770	4.907	4.870	5.029
RNF152	4.656	4.610	4.600	4.592	4.724	4.620
RNF150	6.921	7.222	7.174	7.088	7.130	7.108
RNF151	7.631	7.686	7.802	7.728	7.845	7.767
RNF157	8.941	8.785	8.495	8.736	8.957	8.706
MECP2	9.253	9.181	9.353	8.887	9.270	8.912
BX072566.1	3.830	4.000	4.066	3.900	3.760	4.082
TAS2R8	5.658	5.796	5.674	5.718	5.808	5.719
TAS2R9	5.717	5.461	5.345	5.391	5.524	5.339
TAS2R7	6.325	6.563	6.592	6.733	6.837	6.848
TAS2R4	6.592	6.297	6.142	6.474	6.466	6.100
TAS2R5	6.744	6.563	6.677	6.692	6.649	6.628
TAS2R3	6.887	6.990	6.745	7.170	7.005	7.108
SPAG16	9.797	9.798	9.944	9.415	9.670	9.587
SPAG17	5.667	5.409	5.274	5.559	5.644	5.504
CDK5RAP2	7.967	7.940	8.193	8.029	7.921	7.894
CDK5RAP3	11.203	11.102	11.318	10.936	10.668	10.967
CDK5RAP1	9.963	9.902	10.061	9.882	9.772	9.743
IGHV5-51	6.895	6.905	7.098	7.158	6.817	6.946
CCDC144B	3.910	3.950	3.754	3.810	3.836	3.547
CCDC144A	4.940	4.732	4.722	4.720	4.691	4.712
BAX	8.961	8.918	9.204	8.723	8.805	8.675
BAD	8.794	8.604	8.662	8.554	8.560	8.559
AL160269.1	7.634	7.775	7.766	7.781	7.651	7.535
PMPCB	9.015	8.969	9.029	8.668	8.870	8.861
PMPCA	9.472	9.553	9.674	9.276	9.038	9.317
NEO1	9.191	9.326	9.259	9.037	9.036	9.105
AC090543.3	12.821	12.916	13.024	12.923	12.903	12.978
ATP6V1B1	6.627	6.933	6.843	7.318	6.912	7.008
ATP6V1B2	10.885	10.962	10.951	10.670	10.697	10.820
AL844853.2	9.245	9.328	9.319	9.203	9.266	9.289
GTF2H2C_2	8.310	8.418	8.490	8.307	8.382	8.244
P2RY8	6.386	6.298	6.312	6.286	6.575	6.400
P2RY2	7.821	7.864	7.933	7.754	7.785	7.797
SEC61G	11.899	12.035	12.222	12.111	11.955	12.198
P2RY1	8.854	8.791	8.699	8.329	8.443	8.429
P2RY6	7.009	7.129	7.046	6.985	7.025	7.017
SRP9P1	12.303	12.337	12.348	12.151	12.176	12.312
P2RY4	7.289	7.461	7.432	7.423	7.625	7.543
NCL	12.726	12.752	12.816	12.765	12.622	12.699
AL450384.2	5.772	6.038	6.107	6.098	5.969	5.888
CYP4F25P	7.165	7.227	7.364	7.707	7.106	7.289
MIR1-1HG-AS1	8.397	8.474	8.527	8.492	8.585	8.566
TRAV40	5.433	5.425	5.415	5.588	5.476	5.407
AC069281.2	9.172	9.068	9.080	9.387	9.378	9.178
GUK1	11.049	11.149	11.366	11.065	10.899	11.154
WRAP73	7.555	7.503	7.642	7.406	7.471	7.386
CDCA8	9.953	10.056	10.066	9.709	9.720	9.606
L3MBTL4-AS1	6.266	6.313	6.235	6.382	6.040	6.076
Z97832.2	7.813	7.626	7.875	7.883	7.957	7.824
AL445222.2	6.592	6.453	6.717	6.787	6.628	6.708
MEP1A	5.281	5.545	5.425	5.404	5.312	5.677

MEP1B	6.704	6.761	6.751	6.802	6.988	6.962
RBMXP2	11.739	11.805	11.872	11.619	11.607	11.569
NCSTN	10.672	10.695	10.722	10.623	10.636	10.610
CEP104	9.192	9.146	9.259	9.066	9.348	9.123
AC118459.1	4.709	4.757	4.746	4.818	4.866	4.547
AC118459.2	5.859	5.735	5.446	5.456	5.312	5.507
AC023043.1	6.194	6.363	6.466	6.292	6.305	6.465
TCP11L2	8.935	8.473	8.150	8.873	9.325	9.051
AC023043.4	8.337	8.270	8.280	8.261	8.354	8.364
ANGPT2	5.351	5.411	5.309	5.437	5.414	5.506
ANGPT1	6.692	7.018	6.590	7.163	6.701	6.857
ANGPT4	7.271	7.145	7.336	7.074	7.102	7.328
AC116096.1	4.996	5.385	5.417	5.603	5.437	5.339
AC006115.2	6.023	5.985	6.124	6.419	6.146	6.203
TBK1	9.914	10.019	10.014	10.065	9.952	9.930
DNAAF4-CCPG1	9.814	9.826	9.925	9.589	9.595	9.743
AC113174.1	7.876	7.966	7.909	7.729	7.751	7.909
AC092435.3	4.922	4.941	4.989	5.349	4.961	4.849
CNTNAP3P2	8.431	8.367	8.369	8.136	8.111	8.292
AC092435.4	4.430	4.327	4.260	4.654	4.405	4.426
RPS29P28	3.956	4.146	4.259	4.355	4.388	4.250
GUCA1A	7.936	8.034	8.096	8.183	8.276	8.247
GUCA1C	6.299	6.319	6.440	6.412	6.387	6.369
GUCA1B	7.314	7.397	7.584	7.439	7.380	7.658
ATM	8.782	8.705	8.617	8.517	8.546	8.625
C17orf100	7.237	7.358	7.233	7.082	7.159	7.167
ADAM32	6.029	6.081	6.033	6.144	6.169	6.127
ADAM33	9.003	9.052	9.077	8.966	8.937	9.048
ADAM30	7.176	7.161	7.250	7.566	7.281	7.013
DNAJC5B	8.068	7.873	7.656	7.982	8.093	7.956
CBSL	10.172	10.258	10.319	9.943	9.869	9.923
DOT1L	9.070	9.081	9.118	8.787	9.014	8.989
AC010542.2	10.340	10.483	10.701	10.340	10.296	10.168
AC010542.4	7.530	7.532	7.516	7.456	7.456	7.536
AC010542.5	7.530	7.532	7.516	7.456	7.456	7.536
TCL1A	7.316	7.359	7.316	7.428	7.361	7.378
TRAM2	8.624	8.676	8.637	8.323	8.344	8.648
CARF	5.340	5.147	5.225	5.354	5.180	5.316
SYCP3	4.391	4.432	4.415	4.475	4.592	4.405
TAOK2	9.095	9.057	9.228	9.163	9.083	9.008
SYCP1	5.565	5.417	5.407	5.588	5.581	5.454
MCEMP1	7.773	7.800	7.603	7.588	7.687	7.595
SLC22A18	10.799	10.865	11.006	10.678	10.778	10.642
ADAM3A	4.930	4.748	4.692	4.935	4.969	4.874
SLC22A16	7.012	7.044	6.962	7.355	7.102	6.806
SLC22A17	6.862	7.082	7.306	7.373	7.344	7.246
SLC22A14	9.473	9.397	9.534	9.536	9.635	9.638
SLC22A15	7.660	7.647	7.702	7.573	7.754	7.549
SLC22A12	7.801	7.702	7.715	7.987	8.123	7.890
SLC22A13	7.210	7.274	7.182	7.211	7.181	7.297
SLC22A11	6.320	6.231	6.248	6.635	6.606	6.486
AP003110.1	7.588	7.648	7.757	7.740	7.838	7.758
NSMCE2	10.003	10.057	10.272	10.026	9.970	10.046
NSMCE3	8.116	8.308	8.400	8.361	8.349	8.259
SERGEF	7.865	7.971	8.153	8.009	7.980	8.024
IGLV2-14	8.720	8.794	8.767	8.933	8.906	8.919

SCARA3	7.762	7.849	7.835	8.111	7.866	7.933
SCARA5	8.171	8.253	8.120	8.215	8.399	8.290
ARHGDIG	7.725	7.952	8.212	8.466	8.139	8.050
ARHGDIB	10.548	10.661	10.725	10.434	10.507	10.411
ANOS1	6.053	6.150	6.005	5.947	6.142	6.086
ARHGDIA	11.609	11.700	11.844	11.531	11.469	11.499
USH1C	9.433	9.374	9.605	9.219	9.290	9.164
USH1G	9.572	9.635	9.603	9.586	9.585	9.689
TOP1MT	8.259	8.463	8.467	8.107	8.137	8.144
DENND10P1	9.433	9.625	9.751	9.441	9.458	9.192
TANC1	9.469	9.494	9.539	9.397	9.243	9.455
TANC2	8.267	8.298	8.281	8.180	8.295	8.370
CES1	6.929	7.137	6.962	6.779	7.063	6.934
AC234781.1	4.529	4.576	4.480	4.526	4.567	4.684
CES2	10.126	10.174	10.289	10.074	10.208	10.120
KBTBD12	5.019	5.075	4.791	5.050	5.019	5.035
AC234781.5	8.698	8.742	8.784	8.694	8.685	8.599
FFAR2	6.192	6.335	6.287	6.085	6.289	6.250
FFAR3	6.974	7.173	7.185	7.378	7.221	7.152
KDM8	7.990	7.978	7.946	8.233	8.015	8.079
FFAR1	6.530	6.612	6.681	6.583	6.513	6.627
MDP1	8.338	8.289	8.624	8.297	8.232	8.353
ZNF90	4.665	4.697	4.484	5.065	4.626	4.706
RPL32P3	7.128	7.089	7.029	6.953	7.029	7.256
ZNF92	7.176	7.265	7.452	6.923	6.934	7.159
ZNF93	8.822	8.714	8.882	9.135	9.017	8.985
ZNF99	3.940	3.882	3.956	4.141	3.898	3.836
SAC3D1	9.456	9.477	9.640	9.089	8.966	9.120
RAET1E	6.834	6.746	6.773	6.956	6.840	6.891
TBX21	8.012	7.953	7.965	8.094	7.838	7.993
FAM3A	9.578	9.610	9.801	9.532	9.498	9.560
FAM3B	5.442	5.357	5.298	5.521	5.663	5.268
FAM3C	12.001	12.088	12.042	11.952	11.933	12.022
FAM3D	8.400	8.305	8.316	8.487	8.578	8.393
AC245100.8	10.040	10.032	10.324	9.749	9.958	9.735
AC245100.9	7.865	7.879	8.165	7.721	8.167	7.693
PRB1	10.010	9.870	9.856	10.196	10.133	10.042
AC245100.1	4.890	4.952	4.745	4.635	4.937	4.822
AC245100.4	6.895	7.126	6.730	6.946	6.810	6.971
MIR6838	8.841	8.843	8.862	8.544	8.610	8.784
PRB4	8.583	8.474	8.256	8.753	8.803	8.623
FAM153B	8.275	8.445	8.343	8.661	8.412	8.386
MIR6833	10.061	10.118	10.150	9.869	10.093	10.056
MIR6834	8.967	9.033	9.115	8.713	8.865	8.988
RNPEPL1	9.558	9.631	9.879	9.734	9.758	9.672
MIR6836	8.311	8.360	8.632	8.046	7.928	7.809
AC012020.1	7.999	8.089	7.952	7.879	8.164	8.063
ATPAF2	7.732	7.632	7.839	7.300	7.586	7.297
ERBB3	11.745	11.797	11.845	11.503	11.729	11.442
ATPAF1	9.465	9.446	9.581	9.238	9.298	9.413
SHC1	10.923	10.962	11.114	10.706	10.814	10.684
ERBB4	6.811	6.848	6.663	6.738	6.755	6.678
SHC3	7.643	7.580	7.813	7.829	7.608	7.682
FPR1	7.131	7.049	6.977	7.012	7.164	7.070
FPR2	7.527	7.476	7.355	7.636	7.749	7.670
FPR3	7.347	7.444	7.386	7.614	7.546	7.552

AL591926.7	8.033	8.265	8.068	8.048	7.969	8.265
SMPDL3A	7.693	7.798	7.975	7.461	7.528	7.315
AL591926.3	6.728	6.572	6.787	7.070	6.998	6.812
TRANK1	9.125	9.145	8.927	9.097	9.161	9.159
ZCWPW2	4.298	4.371	4.411	4.352	4.326	4.345
ZCWPW1	8.299	8.562	8.471	8.559	8.500	8.544
LINC02443	7.708	7.758	7.538	7.860	7.805	7.931
AL096678.1	4.506	4.291	4.462	4.485	4.719	4.510
AC009244.1	9.453	9.547	9.557	9.402	9.470	9.515
SSC4D	8.171	8.019	8.308	8.525	8.519	8.276
MT-TR	14.545	14.551	14.548	14.608	14.629	14.551
MT-TM	13.966	14.006	13.987	14.087	14.178	14.074
MT-TI	13.966	14.006	13.987	14.087	14.178	14.074
MT-TK	14.445	14.476	14.457	14.504	14.599	14.502
MT-TE	9.881	9.831	10.136	10.496	11.142	10.154
MT-TD	14.677	14.690	14.674	14.688	14.718	14.710
MT-TG	14.545	14.551	14.548	14.608	14.629	14.551
CDC16	9.029	8.915	8.886	8.855	8.843	8.929
AHNAK	10.893	10.925	10.920	10.777	10.478	10.651
AL139353.2	4.517	4.640	4.556	4.511	4.556	4.591
LINC00592	6.271	6.178	6.196	6.203	6.260	6.234
FRMD8	10.428	10.511	10.562	10.209	10.328	10.310
LINC00595	7.107	6.917	6.665	7.099	7.081	7.116
IMMP1L	8.179	8.267	8.473	8.296	8.335	8.451
FRMD5	8.476	8.554	8.650	8.484	8.371	8.398
FRMD6	9.548	9.542	9.646	9.344	9.575	9.342
FRMD7	5.294	5.555	5.713	5.875	5.431	5.522
FRMD1	7.197	7.300	7.426	7.345	7.406	7.418
PKNOX2	8.521	8.764	8.555	8.587	8.582	8.787
FRMD3	8.260	7.865	8.135	8.069	7.966	8.069
MED13L	9.045	8.962	9.176	8.896	8.988	8.822
DHFRP3	3.910	3.878	3.816	3.778	4.037	3.727
DHFRP1	8.496	8.523	8.589	8.171	8.402	8.412
LBX2-AS1	8.794	8.716	8.752	8.498	8.566	8.704
DPF2	9.927	10.021	10.178	9.849	9.962	9.815
DPF3	8.293	8.251	8.236	8.132	8.101	8.112
DPF1	8.172	8.197	8.342	8.412	8.318	8.186
RPS12	13.691	13.753	13.856	13.823	13.660	13.829
GPALPP1	8.021	7.903	8.062	7.709	7.947	7.760
AF096876.1	6.802	6.916	6.613	6.710	6.865	6.870
VPS45	8.448	8.531	8.645	8.378	8.424	8.437
VPS41	11.052	11.091	11.154	10.913	10.875	10.908
BCAP29	9.177	9.176	8.898	9.341	9.327	9.365
TAS2R38	7.584	7.903	7.688	7.695	7.944	7.933
TAS2R39	7.214	7.215	6.944	7.137	7.342	7.223
RPL36A	13.408	13.485	13.653	13.701	13.471	13.603
VPS4A	9.930	10.086	10.135	9.862	10.009	9.847
VPS4B	10.843	10.807	10.892	10.437	10.412	10.514
GMEB2	9.697	9.762	9.924	9.742	9.784	9.780
AP001627.1	5.381	5.073	5.096	5.081	5.363	5.160
GMEB1	8.238	8.266	8.275	8.077	8.161	8.005
RRP36	9.958	9.974	9.995	9.808	9.905	9.951
SZRD1	10.770	10.810	10.872	10.698	10.771	10.520
LNC-LBCS	6.201	6.192	5.844	6.122	6.188	6.112
PPP1R15A	10.806	10.872	11.021	11.388	11.429	11.260
PPP1R15B	10.442	10.463	10.399	10.249	10.441	10.393

BLMH	8.794	8.828	8.948	8.724	8.729	8.698
SPDYE5	6.170	5.946	5.937	6.285	5.830	5.845
SPDYE1	6.170	5.946	5.937	6.285	5.830	5.845
CR753812.1	8.439	8.433	8.212	8.323	8.555	8.407
TEX46	5.360	5.091	5.264	5.418	5.389	5.504
KLF13	9.131	9.119	9.218	8.774	8.781	8.940
KLF12	8.041	8.041	7.853	8.052	8.180	8.116
KLF11	8.077	8.206	8.209	7.813	7.924	7.912
EBAG9	9.303	9.473	9.484	9.156	9.171	9.221
KLF17	6.753	6.701	6.559	6.536	6.535	6.679
KLF16	8.865	8.999	9.200	9.213	9.319	8.956
KLF15	9.543	9.271	9.360	9.370	9.584	9.389
KLF14	7.526	7.388	7.370	7.343	7.324	7.373
KLF18	5.970	6.021	5.967	5.903	5.927	5.967
TNFSF12-TNFSF	9.155	9.155	9.269	8.997	9.062	9.014
KRTDAP	5.074	5.091	5.030	5.149	5.411	5.317
AC138894.1	10.130	10.253	10.370	10.235	10.127	10.137
ABBA01000935.2	8.034	8.129	8.110	7.831	7.767	7.879
GBE1	10.102	10.220	10.337	10.014	10.040	10.051
AC079171.1	5.939	6.130	5.597	5.872	6.202	5.967
CD99P1	7.434	7.352	7.388	7.386	7.549	7.521
AC004552.1	12.821	12.916	13.024	12.923	12.903	12.978
CXorf40A	10.001	9.995	10.160	9.937	9.872	9.879
CXorf40B	10.001	9.995	10.160	9.937	9.872	9.879
NDUFS3	10.664	10.686	10.935	10.529	10.417	10.505
NDUFS4	9.704	9.804	9.822	9.728	9.774	9.773
NDUFS5	11.422	11.565	11.740	11.469	11.438	11.418
NDUFS6	9.991	10.041	10.124	9.719	9.799	9.814
NDUFS7	9.930	10.001	10.219	9.787	9.649	9.714
NDUFS8	10.875	10.897	11.137	10.798	10.838	10.747
ZRANB1	8.310	8.417	8.404	8.315	8.446	8.406
ZRANB3	7.796	7.686	7.748	7.654	7.584	7.637
ZRANB2	9.990	9.834	9.906	10.024	9.789	10.117
EPHA5-AS1	7.677	7.311	7.673	7.780	7.761	7.751
SVEP1	8.436	8.358	8.474	8.399	8.330	8.400
ATP9B	8.522	8.600	8.503	8.505	8.360	8.529
ATP9A	10.663	10.742	10.811	10.421	10.428	10.445
MICAL1	7.888	7.899	7.961	7.933	7.715	7.894
MICAL2	10.489	10.527	10.439	10.073	10.118	10.000
MICAL3	7.863	7.910	7.894	7.624	7.751	7.741
AL096814.1	7.936	8.034	8.096	8.183	8.276	8.247
FAM218A	4.668	4.787	4.757	4.737	4.857	4.903
WSB2	12.333	12.441	12.426	12.449	12.410	12.416
BX248093.1	8.529	8.524	8.065	8.442	8.567	8.533
GASK1B	7.088	7.068	7.174	6.292	6.697	6.659
GASK1A	5.013	4.939	4.744	5.465	4.912	5.014
AL139339.1	6.991	6.927	6.853	6.736	7.057	6.954
ZNF844	8.406	8.325	8.391	8.514	8.379	8.431
CLTA	11.691	11.731	11.932	11.774	11.727	11.703
ZNF846	6.326	6.400	6.435	6.621	6.410	6.578
CLTC	12.474	12.532	12.600	12.429	12.217	12.473
CLTB	10.761	10.879	10.960	10.795	10.763	10.701
TMEM59L	7.798	7.897	8.068	8.213	7.936	8.004
FLG	4.458	4.425	4.495	4.503	4.496	4.423
BTBD11	7.329	7.547	7.394	7.424	7.751	7.539
AP000229.1	7.835	7.866	7.886	8.025	7.840	7.913

ARMCX7P	10.408	10.362	10.522	10.048	10.096	10.189
PCOLCE	7.268	7.096	7.479	7.202	7.334	7.335
ISCA1	9.409	9.489	9.428	9.132	9.270	9.394
ISCA2	9.774	9.806	9.935	9.703	9.717	9.683
AL596442.1	6.388	6.438	6.416	6.275	6.408	6.570
MALSU1	11.602	11.651	11.799	11.643	11.505	11.610
AGAP10P	9.081	9.018	9.114	9.210	9.131	9.065
AC010761.1	9.514	9.660	10.145	10.210	10.489	9.844
FAM215A	7.123	6.987	6.908	7.217	7.171	7.179
HHEX	10.626	10.691	10.783	10.622	10.640	10.688
PTENP1-AS	7.471	7.609	7.577	7.495	7.538	7.517
AC120049.1	6.742	6.535	6.376	6.882	6.820	6.713
ACTRT2	7.467	7.503	7.493	7.627	7.746	7.420
ACTRT3	7.305	7.298	7.317	7.358	7.509	7.376
AP001000.1	8.660	8.572	8.661	8.656	8.502	8.755
SEPTIN2	12.408	12.436	12.545	12.328	12.289	12.341
SEPTIN3	7.335	7.482	7.430	7.297	7.374	7.421
SEPTIN4	7.655	7.717	7.502	7.760	7.896	7.934
SEPTIN5	6.960	6.880	6.988	7.423	7.152	7.098
NKX3-1	8.307	8.434	8.153	8.339	8.556	8.511
SEPTIN7	12.041	12.032	12.121	12.078	11.985	11.979
SEPTIN8	9.807	9.839	9.859	9.628	9.547	9.633
SEPTIN9	13.441	13.437	13.451	13.294	13.418	13.367
CPED1	6.745	6.884	6.638	6.860	6.963	7.024
ZNF597	6.644	6.663	6.745	6.885	6.617	6.657
ZNF594	5.576	5.352	5.488	5.323	5.352	5.476
CPNE9	7.658	7.506	7.729	8.178	7.524	7.742
CPNE8	8.710	8.721	8.763	8.627	8.689	8.499
CPNE3	9.736	9.774	9.766	9.713	9.587	9.731
CPNE2	8.280	8.355	8.463	7.858	8.154	8.052
CPNE1	10.887	11.011	11.280	10.968	10.832	11.017
CPNE7	8.180	8.045	8.165	8.262	7.947	8.102
CPNE6	8.372	8.274	8.388	8.367	8.494	8.457
CPNE5	7.472	7.361	7.596	7.464	7.529	7.494
CPNE4	9.595	9.462	9.558	9.378	9.507	9.568
DNMT3B	7.362	7.590	7.760	7.245	7.325	7.462
DNMT3A	8.840	8.920	8.855	8.744	8.668	8.837
DNMT3L	6.492	6.705	6.462	6.459	6.519	6.734
AAK1	10.125	10.060	10.031	9.770	9.925	9.843
SNRPN	7.598	7.489	7.410	7.452	7.482	7.398
PTPRN	6.672	6.873	6.864	7.234	7.021	6.859
SNRPB	12.167	12.261	12.414	12.399	12.390	12.347
SNRPC	10.478	10.469	10.536	10.530	10.483	10.537
SNRPA	10.400	10.524	10.555	10.206	10.268	10.305
SNRPF	11.259	11.368	11.470	11.202	11.261	11.208
SNRPG	10.774	10.898	11.100	11.006	10.933	10.986
SNRPE	11.739	11.912	12.050	11.783	11.753	11.727
FAM170B-AS1	7.694	7.557	7.624	7.953	7.920	7.902
MAST1	6.953	7.151	7.387	6.977	7.055	7.108
AC078852.2	8.529	8.710	8.610	8.533	8.607	8.699
RGS9BP	6.421	6.329	6.421	6.410	6.501	6.309
ZNF790-AS1	5.648	5.704	5.751	5.702	5.701	5.661
ZDHHC15	4.624	4.647	4.646	4.764	4.525	4.837
ZDHHC14	8.695	8.637	8.886	8.501	8.664	8.677
ZDHHC17	8.401	8.364	8.376	7.984	8.106	8.200
ZDHHC16	8.858	9.080	9.181	8.866	9.036	9.073

ZDHHC11	8.252	8.226	8.374	8.526	8.559	8.422
ZDHHC13	9.262	9.307	9.348	8.931	8.985	9.076
ZDHHC12	9.809	9.819	9.869	9.688	9.700	9.590
PRKAG1	10.092	10.247	10.167	10.067	10.176	10.141
PRKAG3	7.281	7.278	7.147	7.087	7.481	7.484
PRKAG2	8.770	8.954	8.859	8.812	8.805	8.659
ZDHHC19	7.804	8.109	8.115	7.785	7.822	7.814
ZDHHC18	8.773	8.947	9.077	8.621	8.808	8.779
AL034397.3	6.963	7.042	6.757	7.390	7.136	6.999
AL034397.1	11.739	11.805	11.872	11.619	11.607	11.569
DPEP2NB	8.177	8.236	8.066	8.246	8.234	8.364
NCAN	7.191	6.997	6.900	6.956	7.234	6.967
AP001992.1	6.582	6.485	6.331	6.640	6.517	6.969
ESPL1	10.837	10.827	10.999	11.055	10.922	11.072
AC011815.1	6.395	6.212	6.420	6.158	6.312	6.280
AC011815.3	4.911	4.849	4.951	5.130	5.095	4.871
AL358933.1	5.334	5.340	5.528	5.298	5.328	5.363
ISM2	6.765	6.715	6.753	6.901	6.970	6.814
ISM1	6.073	6.241	6.181	6.166	6.169	6.162
CD81-AS1	6.871	7.145	7.044	6.930	6.921	7.057
MACROD2-AS1	5.754	5.886	5.623	5.719	5.683	5.895
RPSAP9	13.432	13.517	13.661	13.465	13.519	13.574
DDX49	8.987	8.957	9.181	8.773	8.744	8.785
DDX41	10.237	10.291	10.509	10.100	10.108	10.158
FDFT1	12.790	12.896	12.987	13.072	12.929	12.956
DDX43	7.983	7.898	7.903	7.431	7.187	7.232
DDX42	9.125	9.304	9.294	8.806	8.753	8.847
PTER	9.784	9.912	9.962	9.703	10.081	9.643
DDX47	10.079	10.080	10.129	10.167	10.167	10.147
DDX46	10.123	10.206	10.250	9.934	9.822	9.986
PTEN	11.021	10.943	10.903	10.898	10.800	10.785
ELAVL4	5.946	6.164	6.019	6.189	6.367	6.240
ELAVL1	11.825	11.843	11.941	11.785	11.734	11.739
ELAVL2	5.692	5.824	5.556	5.810	5.847	6.035
ELAVL3	7.869	7.807	8.212	8.540	8.245	8.071
LSM14B	8.709	8.845	8.983	8.836	8.781	8.691
LSM14A	10.045	10.159	10.204	10.092	10.122	10.043
EMSLR	4.558	4.414	4.640	4.529	4.304	4.329
AC012065.5	8.968	8.911	9.154	9.085	9.060	9.075
DNAI1	6.717	6.836	6.700	6.908	6.651	6.708
DNAI2	6.437	6.558	6.510	6.770	6.482	6.810
NFIL3	8.464	8.455	8.772	8.514	8.631	8.728
REG3G	6.887	6.895	6.733	6.754	6.971	6.852
AL512791.2	6.303	6.353	6.251	6.863	6.562	6.336
AL512791.1	7.552	7.602	7.038	7.575	7.611	7.693
FRAS1	8.837	8.816	8.813	8.099	8.341	8.598
AC012645.1	7.037	7.010	6.911	7.069	7.190	6.983
SULT4A1	8.370	8.470	8.482	8.405	8.306	8.554
AC005520.4	7.424	7.497	7.553	7.573	7.699	7.494
AC009065.10	8.531	8.481	8.588	8.491	8.308	8.552
AC005520.3	10.324	10.307	10.396	10.393	10.354	10.526
SPSB3	10.541	10.537	10.712	10.360	10.382	10.516
SPSB2	8.469	8.439	8.639	8.181	8.274	8.292
CLUAP1	8.684	8.640	8.677	8.434	8.488	8.307
HINT1	12.418	12.532	12.613	12.688	12.629	12.647
HINT2	10.920	11.009	11.206	10.897	10.871	10.720

HINT3	7.595	7.587	7.669	7.302	7.677	7.141
OR2A4	7.717	7.900	7.748	7.796	7.697	7.761
AC021016.2	8.027	8.073	7.975	8.015	8.072	8.064
PTTG1	10.975	11.007	11.220	10.788	10.948	10.808
PTTG2	4.746	4.679	4.913	4.602	4.708	4.932
THEM4	8.152	8.050	8.023	7.815	8.043	7.659
AC010632.2	6.491	6.401	6.420	6.440	6.498	6.341
AL161757.4	6.797	6.473	6.771	7.166	6.962	6.742
C20orf204	6.378	6.123	6.330	6.756	6.775	6.510
C20orf203	7.838	7.850	7.855	8.009	8.072	8.031
C20orf202	7.077	7.030	7.034	6.999	7.078	7.032
AL935043.1	7.344	7.403	7.456	7.273	7.478	7.510
SST	6.017	6.048	6.016	6.034	5.965	5.837
GU182352.5	5.465	5.240	5.208	5.596	5.463	5.357
GU182352.4	8.835	8.689	8.488	8.744	8.793	8.879
EFHB	3.942	3.953	3.887	3.928	3.853	3.754
COL10A1	7.300	7.294	6.983	7.317	7.564	7.285
CHKB-CPT1B	8.976	8.966	9.050	8.658	8.853	8.811
AC068724.5	6.689	6.878	6.968	6.859	6.927	6.955
AC099329.2	6.734	7.040	7.040	7.027	7.164	7.226
RS1	7.535	7.569	7.417	7.740	7.664	7.611
AC107398.3	6.636	6.710	6.312	6.785	6.622	6.692
ATP13A1	9.687	9.747	9.849	9.426	9.406	9.464
ATP13A3	11.004	11.061	10.988	11.060	11.046	11.058
ATP13A2	8.547	8.795	8.781	8.572	8.386	8.581
ATP13A5	7.572	7.727	7.368	7.721	7.814	7.833
ATP13A4	8.764	8.568	8.744	8.176	8.112	8.233
LINC01996	7.356	7.288	7.242	7.238	7.454	7.325
LINC01990	4.244	4.353	4.496	4.405	4.349	4.305
LINC01992	6.920	7.101	6.787	6.896	7.009	7.150
LINC01998	6.021	5.962	5.764	5.876	6.026	6.070
AL031848.2	6.504	6.415	6.349	6.389	6.628	6.466
PLEKHO2	7.976	8.216	8.199	8.109	8.193	8.187
AC006386.2	14.596	14.604	14.644	14.671	14.651	14.656
PLEKHO1	6.338	6.583	6.767	6.900	6.953	6.993
E2F7	9.578	9.397	9.555	8.670	9.033	8.939
E2F6	9.209	9.151	9.184	9.181	9.090	9.233
E2F5	8.055	7.932	8.033	7.953	8.135	8.112
E2F4	11.385	11.367	11.552	11.539	11.600	11.579
E2F3	8.999	9.008	9.083	9.058	9.339	9.022
E2F2	8.875	9.003	8.883	8.745	8.820	8.996
E2F1	9.745	9.679	9.861	9.669	9.543	9.576
E2F8	8.113	8.025	8.006	7.379	7.578	7.402
EEF2	13.554	13.639	13.732	13.573	13.479	13.620
SNORA20	11.988	12.017	12.108	12.066	11.916	12.005
SNORA21	13.979	14.042	14.002	13.909	13.815	14.061
AC110769.1	5.399	5.329	5.408	5.633	5.569	5.391
SNORA24	8.553	8.568	8.746	8.367	8.345	8.366
SNORA25	7.030	6.890	6.650	6.616	6.727	6.999
SNORA26	10.585	10.697	10.794	10.717	10.645	10.840
SNORA27	12.617	12.677	12.802	12.784	12.769	12.841
RLBP1	7.147	7.231	7.312	7.423	7.550	7.418
TLN2	7.785	7.810	7.797	7.865	7.784	7.695
IZUMO1	4.542	4.381	4.325	4.082	4.430	4.372
IZUMO2	7.381	7.751	7.769	7.701	7.474	7.681
IZUMO4	7.134	7.117	7.237	7.373	7.171	7.336

AC024361.2	7.841	7.816	7.411	8.193	7.964	7.887
PYCR2	10.189	10.199	10.297	10.318	10.365	10.088
PYCR1	9.642	9.680	9.863	9.579	9.548	9.622
FAM234A	10.325	10.513	10.664	10.353	10.140	10.285
OR51J1	7.718	7.624	7.698	7.717	7.910	7.712
CTBS	9.282	9.514	9.372	9.233	9.465	9.142
AC005000.1	12.617	12.628	12.692	12.617	12.655	12.614
ZNF331	7.439	7.591	7.589	7.606	7.649	7.598
IL36A	7.074	6.922	6.765	7.229	6.928	6.935
IL36B	6.690	6.888	6.497	6.740	6.868	6.705
IL36G	6.381	6.170	6.420	6.348	6.358	6.256
IFITM3	12.727	12.776	12.759	12.751	12.832	12.558
IFITM2	11.157	11.155	11.113	11.114	11.385	10.859
LIPC	6.132	6.177	6.165	6.070	6.195	6.116
HPCAL1	11.353	11.364	11.562	11.317	11.357	11.345
AL078621.1	11.706	11.756	11.828	11.257	11.375	11.346
LIPG	8.265	8.418	8.466	8.461	8.572	8.378
AL078621.2	6.489	6.368	6.478	6.771	6.803	6.615
AC026746.1	6.164	6.034	5.720	5.830	6.255	5.966
GAGE2E	7.444	7.371	7.139	7.545	7.560	7.476
LIPJ	6.550	6.552	6.534	6.496	6.176	6.653
GAGE2A	7.444	7.371	7.139	7.545	7.560	7.476
TMEM229B	8.585	8.565	8.687	8.329	8.618	8.599
CD300LG	9.656	9.753	9.794	9.730	9.746	9.863
CD300LF	7.438	7.503	7.375	7.559	7.654	7.685
SNORA2C	8.618	8.593	8.713	8.707	8.773	8.880
AL139011.2	8.940	8.845	9.062	9.073	9.134	8.991
AL139011.1	4.829	4.812	4.764	4.781	4.789	4.805
RILP	9.696	9.859	9.970	10.102	9.892	9.932
HDX	6.398	6.334	6.173	6.253	6.405	6.147
MIR99AHG	7.119	7.214	6.579	7.317	7.295	7.236
ARFGAP2	10.250	10.359	10.455	10.196	10.100	10.130
ARFGAP3	9.038	9.066	8.957	8.929	9.050	9.050
ARFGAP1	9.319	9.326	9.467	9.209	9.148	9.134
U2AF1	11.314	11.348	11.538	11.066	11.070	11.140
U2AF2	10.877	11.032	10.865	10.441	10.648	10.698
GPR162	7.498	7.493	7.698	7.775	7.823	7.727
GPR160	12.281	12.341	12.445	12.307	12.329	12.244
GPR161	7.863	7.884	7.955	8.229	8.073	7.931
COX7A1	6.591	6.418	6.823	6.891	6.714	6.875
COX7A2	11.855	11.878	12.065	12.006	11.914	12.038
MTHFD2	11.374	11.380	11.478	10.951	11.104	11.172
MTHFD1	11.505	11.577	11.644	11.362	11.158	11.352
PRR20A	6.354	6.375	5.959	6.267	6.135	6.357
AL441992.2	8.852	8.842	8.992	8.540	8.790	8.803
AL441992.3	8.249	8.325	8.400	8.266	8.256	8.222
PRR20D	6.354	6.375	5.959	6.267	6.135	6.357
PRR20E	6.354	6.375	5.959	6.267	6.135	6.357
AC079322.1	4.490	4.503	4.363	4.643	4.523	4.482
MMP24OS	11.178	11.203	11.339	11.270	11.288	11.216
LIPC-AS1	4.533	4.500	4.425	4.338	4.532	4.478
IFI27L1	8.242	8.236	8.223	8.014	8.296	8.077
IFI27L2	9.062	9.144	9.243	9.221	9.181	9.127
SLX4	8.727	8.764	8.825	8.637	8.844	8.792
AZI2	7.931	7.789	7.873	7.684	7.552	7.778
ZNF436	8.524	8.728	8.797	8.725	8.607	8.891

ZNF431	6.196	6.204	6.459	6.298	6.353	6.315
ABRACL	9.647	9.714	9.816	9.822	9.806	9.927
ZNF432	7.163	7.371	7.365	7.209	7.107	7.318
ZNF439	4.935	4.648	4.921	5.173	4.862	4.943
ZNF438	8.163	8.224	8.246	8.472	8.313	8.205
CCDC196	3.669	3.661	3.798	3.866	3.835	3.722
CCDC197	6.817	6.924	6.899	6.822	6.838	6.945
RALGDS	8.353	8.249	8.527	8.129	8.381	8.154
CCDC190	4.476	4.721	4.358	4.543	4.523	4.583
CCDC191	6.882	6.803	6.830	6.878	6.902	6.768
CCDC198	9.257	9.209	9.170	9.006	8.831	9.177
LINC01569	8.777	8.607	8.834	8.910	8.842	8.843
LINC01568	5.932	6.235	5.752	6.102	5.905	6.388
LINC01565	7.497	7.491	7.504	7.671	7.514	7.494
LINC01566	8.029	7.975	7.941	8.072	7.931	7.836
LINC01561	7.326	7.364	7.275	7.418	7.407	7.156
LINC01560	6.786	6.867	6.959	6.516	6.718	6.601
LINC01563	6.966	6.786	6.869	7.202	6.756	6.967
TM6SF1	6.183	6.063	6.014	6.083	6.402	6.075
TM6SF2	7.754	7.659	7.608	7.765	7.790	7.719
AC006111.2	6.186	6.387	6.187	5.822	6.155	6.092
RTCB	10.935	11.023	11.219	10.956	10.792	10.717
RTCA	9.645	9.750	9.796	9.478	9.383	9.573
ACTL9	5.291	5.429	5.702	5.683	5.570	5.443
ACTL8	7.961	8.197	8.117	8.244	7.994	8.015
ZFHX2-AS1	9.808	9.891	9.831	9.798	10.010	9.864
SLC4A11	7.747	7.967	7.876	8.033	8.109	8.069
KIAA0232	9.157	9.125	9.228	8.926	8.951	8.943
AL596211.1	3.820	3.929	4.079	3.846	4.026	3.914
IFIT3	11.892	11.811	11.728	11.724	12.040	11.699
AL079343.1	4.587	4.349	4.404	4.470	4.507	4.292
ICA1	9.750	9.844	9.900	9.789	9.701	9.830
AC010271.2	6.418	6.399	6.202	6.661	6.559	6.482
USP2-AS1	6.851	6.802	6.698	6.897	6.858	7.004
AP1B1	9.438	9.427	9.680	9.456	9.505	9.531
CIDECP1	6.060	6.189	6.005	6.080	6.140	6.084
OR5H1	4.250	4.300	4.076	4.222	4.293	4.306
TXLNG	9.004	9.076	8.885	8.858	9.017	9.006
TXLNB	3.986	3.996	3.866	3.985	3.982	3.896
TXLNA	9.939	9.923	9.908	9.753	9.824	9.662
AL591441.1	4.052	4.223	4.023	3.984	4.149	4.127
FAM170B	6.560	6.434	6.489	6.470	6.594	6.659
FAM170A	6.774	6.867	6.978	6.706	7.172	6.967
FEZ1	6.699	6.678	6.787	6.989	7.095	6.804
GTF3C3	9.696	9.762	9.768	9.511	9.546	9.676
FEZ2	11.064	11.152	11.189	11.030	11.102	11.072
RAB4B	8.846	8.922	9.077	8.907	8.815	8.883
RAB4A	9.710	9.773	9.987	9.591	9.383	9.624
AL020989.1	5.733	6.167	6.100	6.147	5.994	6.007
MIR650	8.720	8.794	8.767	8.933	8.906	8.919
AL355877.2	8.351	8.294	8.440	8.069	8.252	8.266
AP002754.1	6.419	6.753	6.523	6.655	6.575	6.862
IL12RB1	7.475	7.615	7.700	7.893	7.659	7.572
IL12RB2	5.285	5.477	5.181	5.312	5.344	5.217
ATP6AP1	12.039	12.115	12.170	11.855	11.846	11.973
ATP6AP2	12.217	12.362	12.399	12.165	12.110	12.273

SEPTIN14P8	7.159	6.862	6.635	6.551	6.694	6.987
BICDL1	8.773	8.716	8.878	8.516	8.613	8.543
BICDL2	9.124	9.304	9.478	9.297	9.272	9.076
SEPTIN14P4	7.159	6.862	6.635	6.551	6.694	6.987
PRY2	7.337	7.057	7.188	7.293	7.443	7.388
OR4N4	4.957	5.142	4.936	4.954	4.989	5.132
SEPTIN14P3	7.159	6.862	6.635	6.551	6.694	6.987
ACTL10	8.110	8.214	8.379	7.986	8.118	7.916
ACOT8	9.027	9.129	9.190	8.982	9.096	8.961
ACOT9	10.254	10.291	10.267	10.182	10.138	10.166
ACOT6	5.213	5.178	5.153	5.473	5.284	5.023
ACOT7	9.347	9.527	9.601	9.394	9.184	9.323
ACOT4	9.588	9.548	9.760	9.504	9.537	9.419
ACOT2	8.700	8.800	8.893	8.462	8.531	8.522
ACOT1	8.700	8.800	8.893	8.462	8.531	8.522
RBBP9	7.466	7.619	7.483	7.324	7.284	7.416
RBBP8	10.008	9.987	9.929	9.881	9.824	9.835
RBBP7	11.823	11.824	11.940	11.665	11.601	11.676
RBBP6	9.091	9.247	9.163	9.306	9.267	9.023
RBBP5	8.464	8.402	8.302	7.931	8.168	7.964
RBBP4	10.858	10.991	11.137	11.025	10.960	10.911
RAB42	8.135	8.157	8.246	8.131	8.303	8.226
RAB43	8.719	8.813	8.783	8.875	9.033	8.729
ABCD1P2	6.039	6.087	6.012	6.364	6.214	6.257
C12orf4	7.941	7.992	8.036	7.827	7.906	7.917
ZFP28	6.631	6.754	6.339	6.521	6.464	6.666
NT5C3AP1	11.665	11.677	11.683	11.774	11.862	11.359
NARF	9.317	9.348	9.438	9.193	9.263	9.250
AL117336.1	7.938	8.083	8.122	7.805	7.936	8.116
GSPT1	11.364	11.469	11.454	11.222	11.176	11.254
RALGPS2	8.932	8.978	9.054	8.895	8.773	8.836
MIER1	9.060	8.946	9.008	8.875	8.980	9.089
MIER2	8.739	8.674	8.770	8.871	8.741	8.724
MIER3	7.030	7.195	7.145	6.944	7.066	7.112
AL133215.2	6.691	6.611	6.642	6.892	6.571	6.497
LRBA	10.071	10.170	10.183	9.944	9.799	10.046
TOLLIP	9.926	10.120	10.190	9.902	9.865	9.968
AL022329.1	4.307	4.226	4.058	4.325	4.299	4.144
AC243899.3	6.624	6.679	7.071	6.635	6.748	6.671
PPP2R5D	9.382	9.576	9.655	9.406	9.421	9.466
PPP2R5E	10.544	10.615	10.782	10.585	10.623	10.324
PPP2R5A	10.227	10.280	10.442	10.252	10.192	10.278
PPP2R5B	8.764	8.726	8.917	8.946	8.935	9.137
PPP2R5C	10.113	10.098	10.122	9.895	10.014	10.029
LCMT1	9.481	9.557	9.750	9.444	9.449	9.375
LCMT2	8.694	8.752	8.687	8.452	8.465	8.648
AC009403.1	7.633	7.630	7.509	7.535	7.501	7.378
AC009403.2	6.162	6.418	6.195	6.558	6.214	6.456
ITPRID1	5.959	5.857	5.995	6.024	5.947	6.109
AL353743.4	4.479	4.255	4.634	4.479	4.463	4.600
ITPRID2	12.144	12.307	12.363	12.138	12.148	12.089
SCRG1	6.352	6.311	5.904	6.060	6.479	6.219
Z69667.1	6.744	6.624	6.790	6.737	6.769	6.698
FOXD4	7.180	7.185	7.366	7.415	7.286	7.122
FOXD2	6.279	6.403	6.405	6.381	6.458	6.406
FOXD3	5.389	5.315	5.721	5.472	5.654	5.402

SLFN12	4.824	4.724	4.452	4.805	4.682	4.577
SLFN13	7.402	7.572	7.836	7.165	7.155	7.019
AC026462.3	8.344	8.366	8.563	8.396	8.475	8.586
AL049776.1	4.772	4.714	4.710	4.391	4.648	4.561
AC006019.1	7.066	7.185	7.400	7.255	7.358	7.430
CAPN15	8.891	8.952	9.055	8.851	8.890	8.896
CAPN14	4.223	4.084	4.276	4.409	4.363	4.269
CAPN13	8.911	9.050	9.070	8.895	8.939	9.056
CAPN12	7.726	7.946	7.956	8.257	7.879	7.888
CAPN11	6.810	6.852	6.858	7.073	6.824	7.038
CAPN10	9.152	9.281	9.331	9.254	9.316	9.322
SLC35D3	4.883	4.999	4.819	4.916	5.252	5.027
SLC35D2	8.247	8.358	8.413	8.135	8.279	8.129
SLC35D1	9.462	9.467	9.471	9.334	9.227	9.271
COX7B	11.598	11.612	11.765	11.912	11.855	11.912
HIBCH	9.164	9.232	9.238	8.975	8.991	9.307
IL1RAPL1	6.778	6.554	6.856	6.856	6.745	6.679
IL1RAPL2	7.176	7.191	6.920	7.323	7.253	7.205
HYI	8.688	8.675	8.927	8.575	8.663	8.565
MPRIP	11.609	11.627	11.605	11.499	11.316	11.455
C11orf98	9.372	9.336	9.498	9.398	9.379	9.287
C11orf97	5.041	5.030	4.831	5.002	4.922	4.923
C11orf96	6.034	6.276	6.220	6.552	6.614	6.542
C11orf95	8.701	8.608	8.759	8.416	8.325	8.628
C11orf94	7.645	7.498	7.568	7.663	7.894	7.838
THUMPD1	9.836	9.903	10.006	9.752	9.860	9.730
THUMPD3	9.251	9.357	9.340	9.175	9.329	9.249
THUMPD2	8.448	8.501	8.586	8.570	8.674	8.681
CHORDC1	9.896	9.869	9.771	9.942	9.931	10.063
PXDNL	4.319	4.511	4.346	4.369	4.450	4.374
MLLT6	11.056	11.050	11.021	10.846	10.689	10.913
MLLT1	9.565	9.598	9.640	9.467	9.365	9.553
MLLT3	9.172	9.107	9.184	8.815	8.792	8.689
SYNJ1	6.860	6.812	6.932	6.792	6.661	6.923
SYNJ2	8.604	8.495	8.729	9.030	8.802	8.728
AC026191.1	5.927	5.935	6.271	5.853	5.890	6.076
RNF213-AS1	4.119	4.047	4.091	4.220	4.088	4.208
PCDHA2	10.289	10.323	10.367	10.104	10.324	10.100
PCDHA3	10.289	10.323	10.367	10.104	10.324	10.100
PCDHA1	10.289	10.323	10.367	10.104	10.324	10.100
PCDHA6	10.289	10.323	10.367	10.104	10.324	10.100
PCDHA7	10.289	10.323	10.367	10.104	10.324	10.100
PCDHA4	10.289	10.323	10.367	10.104	10.324	10.100
PCDHA5	10.289	10.323	10.367	10.104	10.324	10.100
MIR670HG	7.733	7.774	7.677	7.763	7.770	7.857
IL15RA	9.246	9.224	9.254	9.267	9.302	9.154
PCDHA8	10.289	10.323	10.367	10.104	10.324	10.100
PCDHA9	10.289	10.323	10.367	10.104	10.324	10.100
AL133372.2	5.353	5.335	5.573	5.568	5.580	5.769
EPM2AIP1	8.965	8.873	8.909	8.718	8.738	8.762
CDX1	8.620	8.693	8.661	8.760	8.899	8.755
CDX2	8.002	7.947	7.897	8.323	8.149	7.959
CDX4	6.054	6.345	6.475	6.498	6.355	6.588
LRFN3	8.226	8.212	8.468	8.069	8.219	8.090
LRFN2	5.768	6.143	5.983	6.133	6.039	6.067
LRFN1	7.801	7.938	8.043	8.015	7.926	7.877

LYRM7	9.098	9.113	9.101	8.879	8.788	9.010
HNRNPA3P1	11.051	11.098	11.160	10.846	10.830	10.950
LRFN4	8.933	8.959	9.062	8.911	9.031	8.847
NKX2-2	4.863	4.799	5.023	5.009	5.056	5.016
NKX2-3	5.214	5.134	5.085	5.386	5.249	5.586
LYRM9	7.437	7.227	7.233	7.209	7.443	7.346
NKX2-1	7.286	7.263	7.480	7.381	7.392	7.413
NKX2-5	7.533	7.485	7.496	7.714	7.667	7.684
OAS1	11.334	11.424	11.484	11.389	11.568	11.364
OAS3	11.598	11.632	11.598	11.524	11.553	11.202
OAS2	10.784	10.798	10.587	10.809	11.024	10.593
PEX5L-AS2	4.617	4.649	4.506	4.570	4.767	4.954
OASL	10.680	10.759	10.794	10.832	11.131	10.587
CCIN	7.335	7.302	7.531	7.364	7.191	7.436
AL031767.1	6.863	6.766	6.853	6.848	7.019	6.891
C22orf42	7.316	7.618	7.576	7.570	7.993	7.767
CCDC82	8.377	8.468	8.336	8.073	8.021	8.346
DTHD1	4.218	4.116	4.214	4.153	4.195	4.127
AL139288.2	8.303	8.476	8.551	8.317	8.390	8.388
AL139288.3	8.303	8.476	8.551	8.317	8.390	8.388
ASPHD2	7.169	7.183	7.094	7.111	7.231	7.026
ASPHD1	10.490	10.536	10.784	10.433	10.475	10.369
PTGFRN	9.634	9.690	9.606	9.460	9.505	9.545
AC018630.2	7.199	7.315	7.449	7.834	7.457	7.479
UBAP2	10.435	10.397	10.494	10.422	10.416	10.178
UBAP1	9.927	10.063	10.065	10.072	10.115	10.178
DHRS4-AS1	8.882	9.015	9.005	8.897	8.960	8.929
CDC6	12.075	12.086	12.094	11.615	11.696	11.989
TMEM9B	10.777	10.833	10.926	10.820	10.814	10.713
TNFSF13B	6.798	7.095	7.181	7.047	7.244	6.738
DDX60L	8.257	8.349	8.263	7.961	7.955	8.395
KLHL36	10.260	10.262	10.336	9.950	9.944	10.033
KLHL34	4.273	4.058	4.047	4.241	4.085	4.063
KLHL35	7.347	7.540	7.774	7.379	7.430	7.418
KLHL32	3.666	3.924	4.064	3.744	3.836	3.812
KLHL31	6.027	6.026	5.954	6.213	6.287	6.201
TMEM92	8.717	8.700	8.850	8.628	8.579	8.706
TMEM91	6.730	6.538	6.735	7.006	6.886	6.812
TMEM97	12.764	12.916	13.135	13.069	13.133	12.676
TMEM94	9.433	9.443	9.523	9.341	9.369	9.349
TMEM95	8.612	8.805	8.703	8.766	8.967	8.755
MTNR1B	6.569	6.754	6.743	6.934	6.964	6.964
MTNR1A	6.352	6.382	6.466	6.451	6.484	6.560
PEX1	7.338	7.298	7.243	7.340	7.461	7.410
PEX3	9.162	9.367	9.538	9.110	9.154	9.212
PEX2	8.960	9.171	9.222	9.127	9.054	9.347
PEX5	9.394	9.385	9.534	9.493	9.464	9.420
PEX7	8.923	8.881	8.867	8.872	8.858	8.849
PEX6	8.623	8.739	8.904	8.839	8.797	8.876
REG4	7.252	7.413	7.073	7.375	7.708	7.498
H2AB1	6.817	7.013	6.841	6.785	6.895	6.735
H2AB2	6.817	7.013	6.841	6.785	6.895	6.735
H2AB3	6.817	7.013	6.841	6.785	6.895	6.735
ZNF106	8.881	8.934	9.039	8.762	8.856	8.855
FP325335.1	7.479	7.578	7.430	7.550	7.670	7.655
GNAI3	11.056	11.211	11.227	11.012	10.966	11.081

GNAI1	8.842	8.849	8.870	8.590	8.733	8.624
POMP	12.232	12.264	12.371	12.417	12.368	12.334
TFPI	11.396	11.388	11.350	11.132	11.032	11.313
POMC	6.986	7.208	7.045	7.476	7.251	7.362
ACHE	8.421	8.579	8.552	8.585	8.735	8.496
PRB2	10.010	9.870	9.856	10.196	10.133	10.042
PRB3	7.885	7.662	7.703	7.949	8.062	7.841
AL049692.3	7.917	7.702	7.673	7.865	7.964	7.912
AL049692.2	7.917	7.702	7.673	7.865	7.964	7.912
POMK	7.597	7.509	7.438	7.405	7.631	7.492
WSCD1	7.939	7.818	7.831	7.674	7.889	7.740
WSCD2	8.019	8.253	8.316	8.354	8.359	8.425
GDA	9.189	9.180	9.251	9.152	9.089	9.177
RPS13	13.793	13.874	13.905	13.979	13.935	13.958
AL606537.1	5.070	5.273	5.290	5.355	5.379	5.409
RPS11	13.941	13.910	14.013	13.958	13.800	13.945
RPS10	13.767	13.833	13.946	13.964	13.837	13.963
RPS17	14.164	14.198	14.199	14.193	14.160	14.234
RPS16	14.289	14.301	14.376	14.333	14.373	14.372
RPS15	13.953	13.944	14.039	13.928	13.758	13.841
RPS14	13.823	13.863	13.897	14.042	13.992	14.063
SMG7-AS1	5.986	5.992	5.899	5.975	6.078	6.068
RPS19	14.125	14.120	14.181	13.996	13.838	13.977
RPS18	14.097	14.100	14.202	14.276	14.229	14.242
RHOXF2	7.546	7.991	7.732	7.725	8.072	7.867
RHOXF1	7.443	7.546	7.515	7.557	7.617	7.569
AC012314.6	10.357	10.410	10.540	10.053	10.003	10.044
OR1F1	8.373	8.113	8.194	8.146	8.229	8.190
PUSL1	8.165	8.379	8.476	7.951	8.080	8.000
RPL21P11	12.617	12.677	12.802	12.784	12.769	12.841
AC022217.4	7.322	7.298	7.327	7.392	7.336	7.202
PLCXD3	6.813	6.904	6.545	6.697	6.875	6.947
PLCXD2	7.449	7.554	7.464	7.555	7.800	7.634
PLCXD1	9.321	9.226	9.283	9.156	9.141	9.129
TSSC4	8.770	8.882	9.084	8.740	8.686	8.867
RRAGD	9.866	10.034	10.249	9.767	9.971	9.634
CR759747.2	8.614	8.670	8.743	8.558	8.568	8.605
RRAGA	11.415	11.520	11.631	11.481	11.467	11.301
RRAGC	10.636	10.675	10.722	10.609	10.569	10.662
RRAGB	7.428	7.440	7.560	7.616	7.480	7.626
GGT2	10.427	10.413	10.580	10.351	10.206	10.300
GGT1	12.008	12.085	12.242	11.941	11.781	11.833
GGT6	6.681	6.672	6.811	6.801	6.860	6.585
GGT7	8.197	8.116	8.425	8.119	8.234	8.219
GGT5	7.815	7.916	7.971	8.032	7.843	7.924
TIAF1	10.175	10.160	10.103	9.929	9.839	9.967
PTPRU	9.494	9.580	9.626	9.651	9.566	9.491
PTPRT	7.615	7.824	7.648	7.552	7.784	7.742
PTPRS	8.554	8.435	8.752	8.204	8.375	8.437
PTPRR	10.386	10.408	10.509	10.319	10.287	10.460
AL078644.1	7.471	7.507	7.052	7.502	7.612	7.481
TINF2	10.354	10.422	10.515	10.327	10.305	10.234
PTPRG	7.072	7.163	6.971	6.952	7.032	7.170
PTPRF	11.868	11.884	11.997	11.713	11.514	11.754
PTPRE	7.700	7.981	7.659	7.551	7.752	7.767
PTPRD	5.750	5.817	5.845	5.866	5.909	5.903

PTPRC	6.389	6.370	6.365	6.289	6.395	6.368
PTPRB	9.719	9.693	9.804	9.331	9.321	9.411
PTPRA	8.315	8.455	8.435	8.068	8.395	8.321
PTPRO	5.821	5.835	5.660	5.653	5.712	6.196
PVRIG	6.932	7.130	6.773	6.941	6.732	6.868
PTPRM	8.601	8.641	8.594	8.388	8.652	8.383
PTPRK	11.763	11.769	11.890	11.629	11.512	11.516
PTPRJ	9.973	10.031	10.104	9.907	9.841	9.921
PTPRH	9.068	9.200	9.131	9.089	8.988	9.078
IFT27	9.201	9.262	9.318	9.059	9.226	9.180
IFT20	9.783	9.635	9.989	9.909	9.791	9.730
ARHGAP19-SLIT'	7.968	8.005	7.906	8.170	8.077	8.163
IFT22	9.324	9.255	9.493	9.194	9.179	9.209
AC091181.1	7.482	7.701	7.764	7.748	7.756	7.718
SAA2-SAA4	7.600	7.678	7.772	7.716	7.942	7.785
GAPVD1	9.266	9.167	9.324	9.042	8.857	9.143
DMPK	8.495	8.517	8.531	8.639	8.661	8.634
LINC01812	6.563	6.421	6.506	6.269	6.517	6.522
LINC01815	4.414	4.484	4.292	4.388	4.457	4.520
AC099329.1	11.575	11.617	11.694	11.671	11.642	11.718
RNASEH1	9.392	9.435	9.403	9.235	9.271	9.154
DMP1	4.789	4.922	4.861	4.942	4.890	4.874
RC3H1	9.286	9.330	9.352	9.245	9.220	9.307
RC3H2	10.626	10.765	10.710	10.771	10.790	10.605
IFIT5	9.978	9.971	9.946	9.705	9.826	9.598
MIR3663HG	4.163	4.523	4.160	4.259	4.245	4.188
IFIT2	11.135	11.194	11.189	11.196	11.378	11.146
IFIT1	11.681	11.734	11.700	11.720	11.819	11.581
MIR3064	12.062	12.189	12.153	12.161	12.007	12.176
AC002470.2	9.525	9.450	9.496	9.280	9.589	9.456
ISG15	12.480	12.444	12.542	12.382	12.338	12.265
MAP1LC3A	8.941	9.115	9.003	9.038	8.976	9.003
BTF3L4P2	10.229	10.140	10.340	10.339	10.298	10.285
MAP1LC3C	6.623	6.423	6.645	6.764	6.406	6.717
MAP1LC3B	11.675	11.604	11.748	11.459	11.477	11.472
GGTLC1	9.637	9.701	9.818	9.612	9.546	9.572
GGTLC3	10.427	10.413	10.580	10.351	10.206	10.300
GGTLC2	10.427	10.413	10.580	10.351	10.206	10.300
BRCC3	9.672	9.776	9.885	9.724	9.503	9.544
AP001527.1	4.639	4.774	4.625	4.681	4.931	4.479
ARL5A	10.443	10.642	10.657	10.716	10.753	10.320
ARL5C	8.301	8.155	8.253	8.128	8.224	8.374
ARL5B	8.751	8.695	8.759	8.690	8.604	8.783
AC098679.1	6.816	6.827	6.850	7.055	6.571	6.812
WDR35	8.585	8.354	8.343	8.362	8.266	8.195
WDR34	10.740	10.607	10.942	10.364	10.464	10.307
WDR37	8.321	8.466	8.443	8.093	8.191	8.121
WDR36	9.259	9.397	9.391	8.919	8.807	9.060
WDR31	7.044	6.996	6.978	6.789	7.025	6.863
WDR33	9.726	9.735	9.902	9.526	9.550	9.539
EIF4A1	13.137	13.230	13.247	13.064	13.071	13.089
WDR38	7.688	7.634	7.456	8.015	7.964	7.796
EIF4A3	11.465	11.427	11.643	11.443	11.242	11.332
EIF4A2	13.267	13.395	13.433	13.418	13.350	13.416
GIGYF1	9.545	9.379	9.457	9.666	9.615	9.546
GIGYF2	9.873	9.892	9.775	9.803	9.862	9.887

TLE3	8.785	8.880	9.006	8.726	8.875	8.796
TLE2	9.165	9.165	9.213	9.361	9.240	9.331
TLE1	9.826	9.835	10.025	9.917	9.826	9.857
TLE6	7.516	7.521	7.575	7.954	7.642	7.458
TLE5	9.658	9.717	10.017	9.731	9.885	9.646
TLE4	7.446	7.256	7.298	7.437	7.541	7.508
LINC00857	7.713	7.574	7.605	7.585	7.648	7.696
AUNIP	7.610	7.599	7.674	7.448	7.622	7.637
LINC00852	6.152	6.057	6.189	6.017	6.136	5.979
LINC00853	6.440	6.123	6.458	6.838	6.639	6.680
LINC00851	7.281	7.003	7.164	7.252	7.230	7.402
LINC00858	5.785	5.721	5.797	5.652	5.780	5.606
CDC7	8.221	8.325	8.252	7.757	7.879	7.899
AC108058.1	7.451	7.396	7.534	7.482	7.545	7.614
TFPT	8.957	8.888	9.218	8.690	8.751	8.700
AL160006.1	6.993	7.121	6.856	6.634	6.670	6.753
PCDHB13	7.707	7.858	7.713	8.259	7.694	7.979
PCDHB12	7.391	6.995	7.013	7.590	7.488	7.267
PCDHB11	7.391	6.995	7.013	7.590	7.488	7.267
PCDHB10	4.171	4.196	4.131	4.059	3.993	4.062
PCDHB16	4.458	4.756	4.695	4.828	4.558	4.659
PCDHB15	6.832	6.794	6.826	7.157	6.936	7.008
PCDHB14	5.122	5.270	5.407	5.407	5.324	5.207
SNRPCP11	8.196	8.262	8.287	8.740	8.301	8.304
AC004836.1	4.843	4.631	4.736	4.822	4.897	4.821
AC090617.10	6.818	6.695	6.717	6.752	6.675	6.699
EEF1DP5	7.396	7.624	7.609	7.504	7.307	7.498
SLC16A1-AS1	7.382	7.767	7.503	7.550	7.679	7.615
SNRPCP14	8.196	8.262	8.287	8.740	8.301	8.304
AC004528.2	6.813	6.825	6.629	7.087	7.070	6.931
AC004528.1	5.293	5.019	5.117	5.250	5.344	5.071
TMIE	7.402	7.477	7.511	7.423	7.590	7.535
KIAA2026	8.299	8.347	8.283	8.204	8.271	8.342
AC243919.1	13.154	13.225	13.361	13.496	13.391	13.505
AC048382.6	7.919	7.847	7.631	7.845	7.965	7.950
APCS	6.738	6.789	6.859	7.060	6.876	7.001
AC007823.1	6.795	6.731	6.560	6.510	6.861	6.863
LTBP4	7.896	7.936	8.015	7.823	7.790	7.970
LTBP3	9.709	9.770	9.880	9.234	9.048	9.367
LTBP2	8.192	8.272	7.896	8.156	8.156	8.192
LTBP1	8.567	8.552	8.640	8.260	8.349	8.421
APC2	7.667	7.773	7.936	8.127	7.908	7.993
CRAT	8.838	8.980	8.863	9.120	9.083	8.968
LINC02175	7.904	7.891	7.900	7.829	7.818	7.764
LINC02172	6.752	6.619	6.308	6.708	6.754	6.599
LINC02171	8.002	8.003	7.890	8.326	8.179	8.084
LINC02179	5.533	5.375	5.700	5.710	5.534	5.858
MBD5	6.990	6.972	6.909	7.029	6.865	7.054
MBD4	10.337	10.334	10.473	10.053	10.423	9.980
MBD6	8.907	8.994	9.066	8.603	8.704	8.869
MBD1	9.739	9.578	9.705	9.686	9.655	9.702
AMY1C	5.089	5.094	5.291	5.046	4.927	5.253
MBD3	10.264	10.376	10.500	10.243	10.358	10.300
AL353732.1	3.875	3.890	3.840	3.923	3.880	3.884
MLPH	11.002	11.047	11.098	11.038	11.052	10.980
REXO1	7.696	7.873	8.053	7.886	8.125	7.893

REXO2	10.544	10.590	10.765	10.616	10.508	10.571
REXO5	7.541	7.620	7.605	7.185	7.464	7.467
REXO4	8.805	8.875	9.030	8.643	8.772	8.738
SLC25A19	8.581	8.629	8.626	8.403	8.469	8.393
SLC25A18	6.898	6.823	6.853	7.026	7.171	6.815
SLC25A17	9.112	9.069	9.080	8.779	8.827	8.913
AC011495.1	12.988	13.007	13.216	12.976	12.758	13.030
LINC02287	6.944	7.070	7.175	7.003	6.983	7.096
SLC25A14	8.710	8.762	8.851	8.828	8.804	8.880
SLC25A13	8.656	8.691	8.828	8.392	8.626	8.571
SLC25A12	9.031	9.100	9.194	8.965	8.914	8.966
LINC02283	6.064	6.004	6.114	5.903	6.150	6.183
LINC02282	6.285	6.325	6.317	6.281	6.275	6.354
TUBA3C	9.083	9.228	9.091	8.919	8.685	8.784
TUBA3E	8.422	8.491	8.622	8.087	8.050	8.072
TUBA3D	8.422	8.491	8.622	8.087	8.050	8.072
CCDC14	8.770	8.583	8.616	8.597	8.381	8.810
CCDC15	7.718	7.692	7.763	7.701	7.959	7.608
CCDC17	7.737	7.767	7.641	7.549	7.646	7.651
CCDC12	9.126	9.222	9.334	9.092	9.054	9.045
AC005258.1	7.479	7.209	7.199	7.393	7.485	7.316
AC093503.2	8.587	8.687	8.867	8.824	9.431	8.689
AC093503.3	6.704	6.715	6.494	7.111	7.142	6.981
OR3A1	6.178	6.295	6.190	6.383	6.301	6.212
HIGD1AP14	10.638	10.682	10.788	10.885	10.931	10.945
AC270301.1	4.426	4.392	4.330	4.619	4.585	4.578
ZNF280D	8.279	8.077	7.965	8.543	8.320	8.285
ZNF280C	5.438	5.516	5.354	5.288	5.235	5.219
ZNF280B	8.853	8.691	8.835	8.467	8.702	8.580
ZNF280A	7.454	7.606	7.510	7.698	7.672	7.804
GK3P	7.665	7.890	7.811	7.720	7.785	7.940
LINC01487	4.312	4.283	4.230	4.310	4.551	4.280
VIP	3.984	4.063	3.861	3.908	3.987	3.991
LINC01482	7.847	7.941	7.698	7.686	7.884	7.942
VIT	7.071	7.131	6.903	6.968	7.150	6.994
STAR	5.660	5.741	6.094	5.969	6.010	5.904
LINC02825	6.610	6.812	6.619	6.837	6.947	6.811
STAC	9.667	9.821	9.816	9.890	9.771	9.851
COX6B1	11.643	11.756	11.886	11.842	11.691	11.751
COX6B2	8.082	8.111	8.232	8.525	8.287	8.285
IGLV1-47	6.163	5.986	6.079	6.289	6.267	6.191
TMEM51-AS1	7.172	7.171	7.346	7.339	7.306	7.343
LAGE3P1	9.218	9.356	9.394	9.210	9.284	9.274
ARIH1	10.274	10.291	10.359	10.148	10.244	9.951
LINC01734	5.474	5.670	5.341	5.745	6.000	5.551
LINC01735	4.192	4.148	4.169	4.343	4.323	4.325
LINC01737	6.192	6.321	6.266	6.126	6.284	6.333
LINC01730	5.489	5.485	5.430	5.924	5.704	5.638
AC018647.1	6.781	6.921	6.411	6.790	6.777	6.836
AC018647.2	7.213	7.353	7.119	6.978	7.068	7.244
CLDN8	5.191	5.551	5.205	5.332	5.288	5.331
CLDN9	7.919	7.973	8.009	7.928	8.006	7.973
FIG4	8.724	8.637	8.710	8.558	8.661	8.631
AL157373.2	6.047	5.921	6.216	6.227	5.919	6.117
CLDN1	12.840	12.913	13.047	12.860	12.719	12.830
CLDN2	10.475	9.752	9.948	10.058	9.772	9.780

CLDN3	11.548	11.716	11.907	11.734	11.842	11.632
CLDN4	10.339	10.462	10.553	10.554	10.580	10.483
CLDN5	6.915	6.670	7.061	7.206	7.047	7.020
CLDN6	8.092	8.070	8.255	8.215	8.242	8.091
CLDN7	12.400	12.534	12.679	12.417	12.165	12.452
FIGN	7.497	7.499	7.561	7.550	7.510	7.555
LGALS7B	7.046	6.951	7.071	7.095	7.070	7.015
MAGEB2	5.208	5.336	5.047	5.335	5.413	5.325
MAGEB3	4.402	4.394	4.486	4.511	4.809	4.448
MAGEB1	5.970	5.835	5.861	6.049	6.172	6.130
MAGEB6	3.490	3.457	3.618	3.572	3.598	3.416
MAGEB4	5.970	5.835	5.861	6.049	6.172	6.130
MRFAP1	12.533	12.568	12.685	12.415	12.376	12.462
IRF3	10.527	10.553	10.789	10.602	10.640	10.465
IRF2	8.407	8.486	8.521	8.413	8.461	8.354
IRF1	9.278	9.217	9.438	9.577	9.645	9.608
PCNX1	8.140	8.132	8.150	8.116	8.001	8.094
IRF7	11.233	11.127	11.224	11.037	11.173	10.931
IRF6	10.082	10.136	10.164	10.111	10.221	10.115
PCNX4	7.341	7.456	7.300	7.102	7.146	7.438
IRF4	7.549	7.443	7.410	7.553	7.539	7.601
IRF9	11.340	11.371	11.352	11.303	11.317	11.334
IRF8	8.702	8.826	8.811	8.857	8.818	8.731
SNX29	8.224	8.440	8.457	8.284	8.298	8.395
AC093583.1	7.066	7.131	6.903	7.262	7.297	7.150
SNX22	7.488	7.820	7.651	7.738	7.773	7.713
SNX20	6.938	6.972	6.862	7.074	6.950	7.002
SNX21	8.423	8.525	8.664	8.583	8.550	8.542
SNX27	9.686	9.595	9.816	9.399	9.729	9.315
SNX24	8.594	8.655	8.834	8.527	8.748	8.544
SNX25	8.158	8.242	8.507	8.112	8.307	8.217
RUNDC1	9.191	9.144	9.231	8.925	9.099	9.108
PANX1	7.952	8.100	8.236	7.912	7.843	8.001
PANX2	10.141	10.233	10.238	10.283	10.294	10.315
PANX3	4.832	4.907	4.802	4.936	4.828	4.891
ZNF271P	8.035	7.743	7.749	7.874	7.522	7.752
ZBTB12	7.313	7.378	7.537	7.420	7.389	7.318
ZBTB11	8.207	8.288	8.230	7.996	7.908	8.280
PTGDR2	7.458	7.611	7.589	7.701	7.850	7.572
ZBTB17	8.113	8.360	8.316	8.214	8.351	8.335
ZBTB16	7.634	7.696	7.627	7.691	7.805	7.766
ZBTB14	7.641	7.606	7.869	7.356	7.447	7.577
ZBTB18	10.386	10.412	10.607	10.229	10.087	10.386
TEX101	7.781	7.501	7.590	7.733	7.641	7.578
AL161421.1	6.030	5.801	6.208	5.993	5.855	5.938
H3P16	13.752	13.863	13.907	13.791	13.836	13.535
C1orf159	8.598	8.585	8.856	8.818	8.764	8.724
C1orf158	6.258	6.252	6.165	6.306	6.572	6.381
LINC00467	6.988	7.165	7.094	6.828	7.056	6.823
DDX19A	9.664	9.832	9.852	9.649	9.662	9.596
DDX19B	9.606	9.748	9.784	9.523	9.234	9.454
LINC00463	6.034	5.867	5.697	6.043	6.031	5.973
LINC00462	5.216	5.132	5.102	5.220	5.240	5.044
LINC00461	5.616	5.535	5.560	5.542	5.371	5.332
LINC00460	7.798	7.606	7.487	7.581	7.857	7.800
MIR100HG	6.668	6.686	6.544	6.628	6.744	6.831

TRIM68	9.464	9.420	9.571	9.143	9.265	9.319
TRIM69	8.526	8.450	8.479	8.493	8.644	8.588
DUSP4	10.823	11.090	11.378	11.260	11.377	10.638
TRIM61	7.667	7.968	7.849	8.273	8.007	8.181
TRIM62	8.330	8.313	8.297	8.325	8.276	8.268
TRIM63	5.523	5.331	5.393	5.393	5.652	5.470
TRIM65	8.886	8.723	8.897	8.766	8.945	8.835
TRIM66	8.121	8.107	8.195	7.843	7.998	8.047
AL034550.1	6.249	6.547	6.561	6.323	6.703	6.405
TRIT1	8.815	8.852	8.753	8.613	8.856	8.843
ITK	5.420	5.633	5.439	5.496	5.457	5.428
NRF1	7.581	7.558	7.642	7.420	7.776	7.595
NAGPA	9.027	9.113	9.166	8.779	8.851	8.891
LCAT	6.775	6.844	6.780	6.444	6.560	6.768
AL357507.1	6.405	6.557	6.304	6.632	6.692	6.762
SERTAD1	9.497	9.532	9.736	9.882	9.924	9.907
SERTAD2	9.415	9.128	9.179	9.287	9.114	9.627
SERTAD3	8.965	9.002	9.070	8.986	8.972	9.057
ARAP3	7.849	7.774	7.847	7.646	7.773	7.862
BIK	9.922	10.010	10.198	9.979	9.958	10.036
ARAP1	9.192	9.091	9.160	9.163	9.117	9.094
BID	10.276	10.263	10.473	10.307	10.130	10.246
GIN1	7.978	7.906	7.985	7.718	7.481	7.805
MBD2	10.217	10.275	10.333	9.967	9.906	9.874
PYDC1	6.169	6.172	6.375	6.313	6.403	6.280
OR51E1	7.105	6.807	6.860	7.237	7.218	7.132
AC025442.1	7.508	7.298	7.494	7.521	7.470	7.563
ACMSD	5.517	5.508	5.425	5.364	5.390	5.454
DHCR24	13.076	13.177	13.280	13.132	12.990	13.141
LCA5	8.582	8.286	8.312	8.246	8.435	8.185
DEC1	4.949	4.935	4.939	5.236	5.042	5.207
AP000926.2	6.840	6.810	6.880	6.961	7.016	6.971
EGR3	5.146	5.140	5.103	5.304	5.275	5.721
AC069257.4	9.028	9.183	9.287	9.182	9.213	9.194
FGFRL1	9.197	9.108	9.229	8.757	8.657	8.978
EGR1	9.492	9.151	9.429	10.935	10.845	11.155
CRLF2	8.041	7.821	8.081	8.024	7.988	7.992
CRLF3	8.878	8.941	8.852	8.786	8.873	8.636
CRLF1	7.486	7.744	7.630	7.837	7.389	7.624
PARK7	12.152	12.299	12.438	12.307	12.223	12.305
AC107952.2	6.223	6.402	6.209	6.437	6.505	6.413
NXPE1	6.526	6.722	6.627	6.695	6.810	6.730
NXPE3	7.736	7.960	7.989	8.012	7.895	7.857
NXPE4	7.444	7.142	7.051	7.361	7.442	7.372
DMTF1	8.262	8.200	8.191	8.123	7.980	8.037
GUSB	9.367	9.394	9.492	8.694	8.699	9.012
TELO2	8.604	8.670	8.803	8.404	8.437	8.401
KIZ	7.307	7.394	7.186	6.979	7.196	7.347
AC123777.1	6.726	6.645	6.542	6.858	6.869	6.738
KIT	5.814	5.932	5.579	5.784	5.771	5.856
KIN	7.697	7.692	7.791	7.566	7.539	7.631
EDAR	7.813	7.804	7.886	7.768	7.631	7.912
ERVFRD-1	7.004	7.011	6.462	7.106	7.159	7.073
AL590764.2	5.625	5.743	5.541	5.696	5.688	5.543
TNFRSF17	4.677	4.819	4.691	4.655	4.771	4.841
TNFRSF14	6.336	6.337	6.341	6.395	6.293	6.372

AC098484.3	8.265	8.335	8.466	8.039	8.329	8.224
AC098484.2	6.248	6.129	6.306	6.473	6.294	6.524
SLC25A15	9.226	9.344	9.379	9.297	9.251	9.309
TEDC1	7.996	8.160	8.151	8.044	7.804	7.966
TNFRSF19	7.935	7.877	8.299	7.567	7.895	7.806
TSGA10IP	6.882	7.180	7.390	7.266	7.158	7.318
CHODL	7.111	7.137	7.142	7.509	7.350	7.271
PPP4R1L	7.846	7.898	7.782	8.443	8.136	8.120
AC005162.2	4.148	4.430	4.339	4.489	4.400	4.430
TPTE2P1	6.525	6.702	6.659	6.515	6.718	6.763
SLC25A11	10.015	10.080	10.216	9.910	9.976	9.925
CHRFAM7A	6.743	6.757	6.617	6.803	6.702	6.551
TNFRSF1B	6.068	6.291	6.355	6.156	6.273	6.405
TNFRSF1A	10.261	10.268	10.405	10.338	10.234	10.272
ATP1A1-AS1	7.020	7.105	7.264	7.392	7.213	7.214
POLR2J4	10.960	11.004	11.108	10.912	10.811	10.780
EGLN2	9.863	9.905	10.153	9.962	9.978	9.954
POLR2J2	10.960	11.004	11.108	10.912	10.811	10.780
POLR2J3	10.960	11.004	11.108	10.912	10.811	10.780
AC026803.1	5.714	5.544	5.432	5.608	5.561	5.570
HPRT1	11.422	11.526	11.618	11.387	11.394	11.324
SLC49A4	9.220	9.364	9.365	9.130	9.047	9.071
FOSL1	9.862	9.922	9.977	9.934	9.920	9.867
FOSL2	9.753	9.830	9.826	9.702	9.696	9.754
NDUFA10	11.140	11.295	11.368	11.115	10.949	11.066
AP001816.1	8.097	8.174	8.137	8.221	8.224	8.067
CCDC13	8.880	8.927	8.930	8.944	8.841	8.969
ISOC2	8.971	9.036	9.270	8.863	8.867	8.953
ISOC1	9.902	9.993	9.973	9.657	9.575	9.743
CCDC18	6.300	6.158	6.118	5.781	5.904	5.999
SLIT2-IT1	4.561	4.547	4.443	4.509	4.699	4.558
CHRD	7.283	7.261	7.385	7.387	7.536	7.366
AC114928.1	3.641	3.780	3.612	3.657	3.649	3.570
C1orf210	9.086	8.983	9.064	8.656	8.778	8.912
C1orf216	8.055	8.064	8.347	7.717	7.921	8.040
WASL	10.748	10.652	10.755	10.397	10.366	10.474
BCL9L	10.053	10.081	10.249	10.206	10.487	9.835
AC004948.1	4.105	4.116	4.167	4.299	4.226	4.229
CLEC3B	6.302	6.259	6.093	6.059	6.199	6.377
MIR7113	9.113	9.115	9.316	8.751	8.619	8.790
CLEC3A	6.942	7.046	6.947	7.009	7.086	7.025
AMBP	6.798	6.826	6.828	6.790	6.762	6.851
AL353763.1	5.624	5.282	5.602	5.472	5.420	5.213
AMBN	7.376	7.455	6.911	7.490	7.466	7.434
AC068733.1	6.565	6.803	6.670	6.818	6.925	6.961
MAD2L1BP	9.303	9.296	9.435	9.123	9.182	9.210
DGKZ	9.639	9.685	9.727	9.532	9.470	9.445
OR5AK4P	7.759	7.925	7.689	7.856	7.742	7.897
C12orf73	9.040	9.132	9.286	8.913	8.936	8.974
RHOBTB2	8.555	8.589	8.625	8.380	8.353	8.468
RHOBTB3	10.117	10.032	10.194	9.807	10.022	9.586
RHOBTB1	6.910	6.957	7.026	6.821	6.809	6.804
INO80D	8.743	8.587	8.488	8.650	8.663	8.715
INO80E	9.775	9.782	10.034	9.537	9.678	9.724
INO80B	9.450	9.417	9.344	9.385	9.354	9.329
INO80C	10.186	10.147	10.302	10.155	10.106	10.070

DIS3L2	7.890	7.894	7.870	7.726	7.828	7.682
FAM43B	7.607	7.356	7.279	7.504	7.439	7.446
FAM43A	7.559	7.816	7.629	7.478	7.672	7.790
FAM124B	7.382	7.195	6.904	7.447	7.437	7.256
FAM124A	7.524	7.678	7.613	7.752	7.701	7.696
AC244034.2	7.179	7.312	7.054	7.001	7.345	7.133
AC079447.1	9.968	9.978	10.093	10.085	10.117	9.975
CYP2F1	6.077	6.168	6.317	6.357	6.305	6.259
AC244034.1	12.854	12.889	13.013	12.967	12.862	12.928
RNU6-172P	7.043	7.102	6.917	7.199	7.077	7.057
AL390026.1	4.957	5.015	5.165	5.324	5.569	5.263
FNTA	11.304	11.352	11.460	11.283	11.141	11.251
FNTB	10.012	10.125	10.048	9.966	9.944	10.070
ADORA2A-AS1	7.554	7.605	7.173	7.416	7.586	7.562
AC010359.2	6.250	6.156	5.873	6.119	5.770	6.096
YJU2	8.444	8.298	8.554	8.060	8.254	8.326
VIM	11.363	11.384	11.652	10.997	11.092	10.961
AC069288.1	10.583	10.681	10.716	10.263	10.296	10.281
AC092069.1	10.182	10.150	10.441	10.353	10.606	10.400
TTLL7-IT1	6.120	5.916	5.556	5.987	5.958	6.038
OR7C2	7.657	7.760	7.595	7.698	7.610	7.541
OR7C1	6.260	6.293	6.318	6.457	6.289	6.260
TRPM2-AS	7.149	7.059	7.188	7.281	7.082	7.120
AC091962.1	4.249	4.160	4.116	4.182	4.095	4.019
LINC01139	5.444	5.426	5.540	5.401	5.441	5.538
LINC01136	7.626	7.751	7.708	7.936	7.827	7.949
LINC01137	8.148	8.287	8.310	7.973	8.277	8.107
LINC01134	8.633	8.730	8.716	8.412	8.829	8.834
LINC01135	7.485	7.561	7.693	7.406	7.362	7.191
LINC01133	5.547	5.516	5.566	5.680	5.665	5.639
AL135926.1	6.913	6.995	7.059	7.097	7.107	7.003
AC005899.5	7.596	7.581	7.258	7.582	7.626	7.556
TCERG1L	5.784	5.798	5.712	6.045	5.942	5.988
GATD1	8.870	8.807	9.050	8.734	8.443	8.599
CCNG1	11.228	11.308	11.434	11.183	11.154	11.111
CCNG2	9.919	9.867	10.031	9.509	9.487	9.567
MIR4657	9.364	9.591	9.571	9.555	9.556	9.523
MIR4653	8.135	8.825	9.052	8.706	8.555	8.107
SPDYE16	6.170	5.946	5.937	6.285	5.830	5.845
AP2M1	12.417	12.470	12.605	12.220	12.165	12.343
MICOS10-NBL1	10.926	10.919	11.145	10.898	10.876	10.951
GCAT	9.113	9.199	9.364	9.022	9.032	9.010
GHSR	7.196	7.176	7.196	7.272	7.342	7.155
AC005041.1	8.796	8.692	8.549	8.781	8.566	8.877
LINC00261	6.339	6.278	6.366	6.465	6.417	6.379
AC104770.1	5.911	5.678	5.951	6.012	6.008	6.122
LINC00265	6.932	6.896	6.793	7.697	7.527	7.538
AC005041.4	6.135	6.176	6.316	6.212	6.451	6.089
LINC00269	8.135	8.397	8.306	8.234	8.559	8.377
FBXO44	7.870	7.830	8.042	8.261	8.221	8.249
TXNDC11	9.588	9.673	9.632	9.527	9.455	9.674
PDE9A	8.268	8.333	8.593	8.159	8.243	8.172
STAM	9.322	9.400	9.329	9.172	9.109	9.249
TAF6L	7.986	8.083	8.092	7.771	8.087	8.051
DAO	7.196	7.142	6.806	7.019	7.144	7.147
TATDN1	9.769	9.860	9.890	9.909	9.764	9.850

TATDN3	7.712	7.717	7.766	7.550	7.606	7.725
TATDN2	9.219	9.242	9.307	9.166	9.136	9.242
WWTR1	11.871	11.960	11.999	11.654	11.412	11.716
LRP1B	4.096	3.954	3.729	4.184	3.912	3.963
DAP	11.659	11.797	11.917	11.563	11.594	11.574
LRP10	11.869	11.960	12.003	11.773	11.745	11.639
LRP11	9.802	9.947	9.902	9.645	9.688	9.718
LRP12	7.548	7.549	7.534	7.058	7.124	7.292
PRPS1L1	5.791	5.786	5.556	5.630	5.615	5.471
AL135744.1	7.523	7.574	7.623	7.376	7.457	7.420
AP001020.2	5.952	5.862	5.827	5.771	5.751	5.810
GAPDHS	6.137	6.291	5.799	6.349	6.215	6.254
BICD2	9.582	9.487	9.525	9.371	9.689	9.420
BICD1	9.013	8.635	8.428	8.638	8.721	8.988
BBS9	8.592	8.459	8.602	8.594	8.495	8.546
OR7E28P	9.849	9.926	10.029	9.810	9.880	9.911
BBS1	8.654	8.756	8.873	8.547	8.395	8.636
AC010332.3	6.327	6.074	5.997	6.454	6.217	6.235
BBS2	8.193	8.363	8.326	7.855	7.736	8.000
BBS5	5.530	5.469	5.731	5.122	5.611	5.030
BBS4	9.017	8.930	8.944	8.517	8.898	8.688
BBS7	6.423	6.031	6.330	6.083	6.010	6.089
TBCC	9.601	9.651	9.669	9.620	9.649	9.604
ESM1	4.047	3.942	4.065	4.247	4.127	4.095
EPS15P1	4.148	4.356	4.272	4.249	4.241	4.239
AGL	9.022	9.050	9.055	8.727	8.671	8.880
AGK	9.334	9.259	9.316	9.120	8.994	9.064
NIPA2	10.219	10.223	10.239	10.030	10.063	10.119
NIPA1	7.797	7.700	7.785	7.652	7.651	7.657
AGA	8.994	9.156	9.182	8.899	8.953	8.982
MT-ND4L	14.647	14.647	14.633	14.660	14.699	14.664
LINC01732	8.764	8.400	8.430	9.041	9.089	8.804
AGT	10.298	10.346	10.424	10.121	9.977	10.124
SLC50A1	10.030	10.224	10.174	10.164	10.168	10.118
MRGPRF-AS1	8.911	9.035	8.899	8.951	8.962	8.950
RAB11A	12.939	13.065	13.119	13.077	13.031	13.041
AC026316.3	4.391	4.314	4.445	4.354	4.164	4.400
PIK3CA	8.981	9.114	9.204	8.875	8.772	8.851
PPP4C	11.220	11.313	11.525	11.092	11.066	11.107
PIK3CD	7.779	7.682	7.793	7.761	7.623	7.877
PIK3CG	5.539	5.632	5.541	5.486	5.564	5.511
AC127537.2	6.409	6.569	6.388	6.316	6.356	6.422
AC127537.1	6.409	6.569	6.388	6.316	6.356	6.422
RAB11B	8.633	8.333	8.682	8.674	9.142	8.680
ZSWIM1	9.517	9.617	9.530	9.614	9.623	9.530
ZSWIM2	4.418	4.379	4.412	4.766	4.500	4.716
ZSWIM3	8.354	8.441	8.615	8.509	8.480	8.371
ZSWIM8	8.843	8.832	8.863	8.627	8.524	8.820
ZSWIM9	7.976	7.851	7.972	8.210	8.272	7.981
AC104806.2	4.772	4.719	4.603	4.696	4.674	4.694
AC131097.5	5.305	5.514	5.566	5.676	5.713	5.601
MEG3	7.644	7.800	7.505	7.561	7.694	7.626
PIK3C3	8.698	8.766	8.816	8.754	8.851	8.873
MEG9	6.143	6.202	6.012	6.377	6.263	6.275
MEG8	6.594	6.850	6.601	6.575	6.754	6.842
PRRG4	10.289	10.275	10.397	10.107	10.315	10.204

PRRG1	9.599	9.627	9.589	9.396	9.559	9.573
PRRG2	8.781	8.941	9.091	8.804	8.759	8.755
PRRG3	7.955	8.079	8.004	7.914	8.003	7.975
MAX	10.840	10.963	11.045	10.814	10.914	10.765
MAZ	10.388	10.369	10.663	10.659	10.741	10.344
ERI2	8.569	8.591	8.521	8.298	8.213	8.353
ERI3	9.209	9.264	9.493	9.187	9.167	9.219
ERI1	8.534	8.690	8.642	8.169	8.097	8.308
MAG	6.316	6.169	6.290	6.452	6.241	6.519
MAF	6.428	6.299	6.347	6.207	6.196	6.437
MAK	5.836	5.901	5.887	6.040	5.881	5.729
MAL	6.280	6.337	6.380	6.537	6.381	6.430
CSDE1	11.962	11.903	11.917	11.573	11.870	11.573
ZNF426-DT	7.421	7.390	7.295	7.356	7.386	7.476
SCART1	7.290	7.305	7.154	7.432	7.327	7.333
AL121832.2	8.285	8.178	8.400	8.257	8.303	8.189
JAGN1	10.205	10.239	10.380	10.131	10.179	10.230
AC068205.2	4.706	4.477	4.495	4.627	4.615	4.640
LINGO1-AS1	7.250	7.406	7.461	7.334	7.513	7.464
HMCN2	8.196	8.319	8.565	8.531	8.466	8.476
HMCN1	3.902	3.693	3.642	3.997	3.772	3.807
LINGO1-AS2	7.954	8.065	7.842	7.966	7.881	8.075
KIAA1210	6.036	5.880	5.775	6.130	5.764	6.044
AC092807.4	4.675	4.567	4.604	4.549	4.541	4.435
UTP25	8.897	8.663	8.624	8.717	8.553	8.713
UTP23	9.380	9.367	9.631	9.378	9.604	9.319
UTP20	9.212	9.149	9.188	9.028	9.036	8.876
IGF2BP2-AS1	6.689	6.626	6.744	6.604	6.818	6.981
NPHP3	7.945	7.722	7.576	7.401	7.559	7.578
AC005014.2	6.755	6.487	6.420	6.498	6.609	6.663
AC073314.1	4.024	4.060	4.118	4.337	4.274	4.141
NPHP4	8.155	8.069	8.170	8.106	8.158	8.206
FZD10-AS1	7.292	7.324	7.048	7.353	7.364	7.288
CTH	8.958	8.813	8.921	8.524	8.779	8.524
PCOLCE-AS1	8.460	8.640	8.616	8.595	8.786	8.779
ARF3	11.783	11.898	11.977	11.733	11.704	11.775
ARF1	13.078	13.171	13.289	13.154	13.082	13.118
ARF6	11.770	11.880	11.959	11.839	11.994	11.910
ARF5	10.029	10.185	10.388	10.156	10.144	10.068
ARF4	12.270	12.376	12.456	12.388	12.355	12.425
LINC02325	6.225	6.190	6.100	6.225	6.420	6.273
LNCAROD	5.866	5.900	5.891	5.834	5.897	6.010
PCNX2	7.930	7.982	8.198	7.963	8.048	7.834
THAP10	9.767	9.799	9.868	9.522	9.629	9.688
PCNX3	9.136	9.033	9.172	8.817	8.944	8.897
GLP1R	9.429	9.340	9.159	9.548	9.498	9.483
SCN4A	6.752	6.800	6.397	6.843	6.660	6.804
BGLT3	8.115	7.726	8.075	8.084	7.906	7.956
RAB11FIP3	9.259	9.315	9.134	9.077	9.096	9.068
RAB11FIP2	7.801	7.756	7.628	7.520	7.631	7.520
RAB11FIP1	12.657	12.739	12.743	12.696	12.763	12.519
IRF5	8.463	8.408	8.524	8.473	8.324	8.430
RAB11FIP5	8.734	8.768	8.871	8.518	8.516	8.679
RAB11FIP4	8.166	8.293	8.373	7.923	7.926	8.034
AC005062.1	6.101	6.066	5.922	6.102	6.197	6.162
AC090360.1	8.052	8.104	8.034	8.110	7.945	8.256

TTC41P	7.870	8.102	7.894	8.123	7.967	8.100
AL159152.1	3.646	3.694	3.707	3.625	3.573	3.747
AASS	7.484	7.341	7.224	6.930	7.542	7.105
CLIP3	8.102	8.235	8.075	7.967	8.458	8.359
CLIP2	7.954	8.059	8.306	7.983	7.834	8.015
CLIP1	10.378	10.388	10.376	9.942	10.138	10.110
SOX5P1	5.447	5.457	5.080	5.274	5.454	5.406
CLIP4	10.119	10.161	10.196	9.900	9.904	10.172
LINC01677	5.676	5.530	5.229	5.763	5.622	5.324
LINC01676	6.844	6.686	6.006	6.782	6.940	6.722
CDC37L1	8.153	8.181	8.271	8.255	8.345	8.246
CD48	6.894	6.996	6.926	6.951	7.054	7.084
AC119427.2	10.943	11.119	11.226	11.416	11.332	11.123
CD44	13.143	13.178	13.156	13.041	12.985	13.137
CD47	11.411	11.467	11.607	11.534	11.482	11.491
CD46	12.008	12.139	12.198	12.091	11.908	12.123
CD40	10.129	10.231	10.318	10.142	10.238	10.214
FPGT	7.918	8.074	7.905	7.778	7.886	8.029
FPGS	9.346	9.363	9.322	8.938	9.147	9.149
VMA21	11.343	11.425	11.487	11.286	11.039	11.169
TBL1X	9.321	9.074	9.173	8.847	8.899	9.071
TBL1Y	6.041	6.022	5.913	6.018	6.404	5.992
FAR1	9.893	10.022	9.934	9.448	9.482	9.525
FAR2	9.288	9.290	9.195	9.144	9.149	9.336
AC008268.1	4.814	4.800	4.660	4.760	4.595	4.737
AC245884.8	6.440	6.377	6.253	6.213	6.444	6.404
SLC25A21-AS1	4.700	4.680	4.763	4.923	4.784	4.643
CEACAMP3	5.524	5.816	5.775	5.697	5.852	5.643
RB1-DT	7.490	7.632	7.191	7.499	7.257	7.604
IL31RA	5.257	5.215	5.228	5.249	5.372	5.202
CYP3A7-CYP3A5	7.056	7.009	6.853	7.020	6.897	7.005
PAK4	10.974	11.031	11.190	10.943	11.009	10.833
PAK5	7.171	7.358	7.300	7.368	7.422	7.458
PAK6	8.181	8.398	8.249	8.039	8.102	8.250
PAK1	10.147	10.149	10.289	9.977	9.983	10.084
PAK2	10.071	10.213	10.202	10.112	10.276	10.163
PAK3	8.182	8.045	7.618	8.265	8.293	8.263
NSDHL	9.627	9.739	9.919	9.804	9.684	9.741
KCNG1	7.589	7.771	7.685	7.838	7.786	7.837
KCNG2	5.961	6.403	6.153	6.231	6.267	6.365
KCNG3	5.365	5.545	5.395	5.465	5.382	5.502
KCNG4	7.176	7.282	7.080	7.381	7.358	7.319
NUBPL	8.331	8.184	8.043	7.615	7.707	8.091
CARS-AS1	6.039	6.256	6.179	6.166	6.238	6.475
IGLV9-49	4.999	5.189	4.953	5.168	5.330	5.158
GAST	7.257	6.983	7.355	7.273	7.377	7.245
SUB1	11.828	11.982	12.029	12.006	12.138	12.110
MFSD2A	8.156	8.206	8.355	8.460	8.174	8.295
MFSD2B	8.364	8.176	8.377	8.191	8.165	8.244
AXDND1	4.382	4.448	4.353	4.510	4.575	4.484
KAT14	8.025	7.866	8.116	7.857	7.933	7.897
HEXA	11.158	11.345	11.466	11.078	10.945	11.034
ARID2	8.393	8.492	8.714	8.611	8.792	8.196
ZBTB10	7.617	7.503	7.540	7.093	7.099	7.335
OR8D1	6.679	6.902	6.855	6.896	6.957	7.014
OR8D2	7.189	7.348	7.128	7.250	7.231	7.243

ARGLU1	8.796	8.869	8.773	8.679	8.652	8.721
GLYCTK	8.171	8.255	8.310	8.058	8.136	8.154
AC008040.1	6.456	6.592	6.580	6.701	6.741	6.548
GAS8	8.889	8.917	8.976	8.753	8.782	8.861
NUBP1	9.092	9.174	9.277	8.993	8.856	8.979
GAS1	5.537	5.734	5.526	5.632	5.523	5.531
GAS2	5.958	5.796	6.210	5.679	5.924	5.907
GAS5	11.218	11.269	11.295	11.166	11.186	11.276
GAS6	9.344	9.396	9.417	9.360	9.387	9.423
RN7SKP88	4.232	4.165	4.506	4.480	4.299	4.279
AP002748.4	7.622	7.732	7.786	7.796	7.899	7.969
AP002748.5	8.654	8.756	8.873	8.547	8.395	8.636
UGT2A3	4.278	4.535	4.207	4.225	4.087	4.163
UGT2A2	4.611	4.675	4.703	4.711	4.782	4.727
SCN10A	7.936	8.014	8.010	8.216	8.208	8.310
ZNF195	9.068	8.988	9.005	9.071	9.131	8.928
AL442639.1	5.151	5.509	5.089	5.149	5.352	5.306
C22orf39	7.973	8.005	8.105	7.443	7.719	7.489
C22orf31	6.995	7.227	6.891	7.454	7.232	7.373
CYP4F35P	8.473	8.608	8.508	8.605	8.706	8.603
C22orf34	6.427	6.252	6.350	6.251	6.456	6.430
PLPP1	9.801	9.864	9.977	9.817	9.739	9.821
AC092042.3	7.014	6.941	7.054	7.101	7.126	7.098
PLPP2	10.983	11.080	11.211	10.780	10.725	10.790
PLPP5	10.809	10.898	10.939	10.703	10.880	10.732
PLPP4	6.334	6.107	6.447	6.486	6.453	6.436
PLPP7	7.390	7.648	7.665	7.434	7.489	7.612
PLPP6	8.611	8.735	8.656	8.428	8.450	8.525
AGAP2-AS1	8.372	8.410	8.583	8.111	8.019	8.183
AC138627.1	5.558	5.597	5.683	5.870	5.706	5.539
LINC00466	5.464	5.702	5.266	5.490	5.610	5.605
GKAP1	7.092	7.175	7.432	6.998	6.959	7.076
SH3PXD2B	8.745	8.855	8.926	8.639	8.722	8.727
SH3PXD2A	7.567	7.555	7.502	7.537	7.769	7.461
LYSMD2	10.238	10.319	10.309	10.178	10.160	10.196
CHPF2	9.039	9.079	9.119	8.874	8.889	9.074
LYSMD1	7.794	7.536	7.527	7.490	7.496	7.368
AC126177.1	7.091	7.156	7.144	6.993	7.133	7.151
CLGN	8.072	8.067	8.239	7.834	7.907	7.866
SMARCA1	8.077	8.157	8.237	7.828	7.805	7.900
SMARCA2	9.395	9.487	9.573	9.218	9.283	9.218
SMARCA4	9.901	9.909	10.148	9.887	9.608	9.780
SMARCA5	9.693	9.757	9.700	9.390	9.391	9.511
POMZP3	8.569	8.674	8.851	8.413	8.585	8.498
AL355336.2	6.884	6.762	6.441	6.623	6.920	6.771
GOLGA6L9	7.449	6.960	6.903	6.742	6.653	7.251
GOLGA6L4	7.449	6.960	6.903	6.742	6.653	7.251
GOLGA6L6	8.692	8.361	8.050	8.620	8.552	8.728
FYN	10.196	10.323	10.577	10.164	10.490	10.105
GOLGA6L1	8.692	8.361	8.050	8.620	8.552	8.728
GOLGA6L2	5.087	5.195	5.205	5.304	5.341	5.302
CPB2-AS1	4.507	4.333	4.446	4.523	4.572	4.472
ZNF213-AS1	6.506	6.592	6.858	6.548	6.466	6.582
OST4	11.198	11.340	11.514	11.509	11.448	11.356
FCGR2B	8.197	8.128	8.137	8.163	8.277	8.128
FCGR2C	8.197	8.128	8.137	8.163	8.277	8.128

FCGR2A	7.517	7.559	7.499	7.640	7.741	7.887
OLA1	11.713	11.782	11.885	11.822	11.908	11.858
WBP1	10.130	10.206	10.336	10.332	10.107	10.248
CALCR	6.731	6.672	6.882	6.606	6.784	6.970
PSAPL1	7.902	7.972	7.994	8.086	8.126	8.241
CPS1-IT1	6.324	6.354	6.350	6.440	6.392	6.373
CALCA	7.864	7.799	7.966	7.782	7.845	8.027
CALCB	6.053	6.189	6.200	5.738	5.888	5.913
WBP4	7.322	7.288	7.363	7.171	7.313	7.303
IGLV2-8	8.720	8.794	8.767	8.933	8.906	8.919
CKB	9.535	9.309	9.580	9.218	9.245	9.261
CKM	6.732	6.827	6.889	6.958	6.904	6.824
UNG	10.712	10.668	10.794	10.341	10.182	10.385
BX322639.1	3.663	3.628	3.692	3.686	3.810	3.874
OR51B4	7.172	7.160	6.730	7.121	6.974	7.171
OR51B6	7.031	7.500	6.980	7.239	7.451	7.265
OR51B2	7.275	7.264	6.920	7.177	7.374	7.455
UNK	8.367	8.238	8.477	8.342	8.379	8.373
LIX1	6.469	6.727	6.269	6.960	6.858	6.723
CUL9	8.091	7.971	8.062	7.966	7.939	7.827
CUL5	8.677	8.777	8.760	8.383	8.473	8.367
CUL7	9.257	9.255	9.354	9.352	9.234	9.229
CUL1	8.747	8.762	8.936	8.390	8.471	8.743
CUL2	8.317	8.236	8.273	8.065	7.986	8.276
CUL3	10.567	10.577	10.715	10.486	10.296	10.561
FAM135A	9.517	9.609	9.570	9.429	9.336	9.501
AL499627.1	7.820	7.960	7.751	8.021	8.032	7.907
AL121827.2	5.726	5.867	5.754	5.767	5.913	6.135
AC004789.1	6.949	6.394	6.353	6.934	7.037	6.761
ZNF295-AS1	7.684	7.297	7.557	7.833	7.773	7.691
FCER2	7.659	7.480	7.613	7.561	7.754	7.596
AC010931.1	5.399	5.302	5.258	5.572	5.510	5.433
AL391704.1	5.738	5.405	5.553	5.757	5.568	5.627
RNF157-AS1	4.857	5.258	4.982	5.236	4.847	4.815
AL513210.1	7.290	7.515	6.975	7.525	7.527	7.326
MMGT1	11.046	11.070	11.080	10.900	10.727	10.698
SP140	7.397	7.430	7.478	7.412	7.421	7.321
AP001120.3	8.133	8.291	7.986	7.939	8.331	8.161
AP001120.2	7.559	7.552	7.405	7.429	7.405	7.683
AP001120.1	7.200	7.081	7.125	7.342	7.199	7.164
ARHGAP8	10.456	10.430	10.490	10.110	10.125	10.208
ARHGAP9	7.851	7.314	7.058	7.657	7.613	7.512
ARHGAP4	6.937	7.002	7.175	7.163	7.035	7.088
ARHGAP5	9.702	9.791	9.693	9.411	9.474	9.578
ARHGAP6	5.804	5.865	5.714	5.942	6.011	5.900
ICOSLG	8.562	8.689	8.776	8.901	9.049	8.853
ARHGAP1	11.532	11.568	11.718	11.306	11.358	11.364
AC025419.1	6.079	6.332	6.241	6.218	6.514	6.081
KHDC1L	5.291	5.346	5.213	5.178	5.344	5.447
RIDA	8.860	8.921	9.097	8.774	8.661	8.747
ANXA11	12.365	12.451	12.586	12.202	11.943	12.088
ANXA10	12.644	12.686	12.686	12.635	12.518	12.629
ANXA13	9.942	9.789	10.054	9.662	9.634	9.592
H1-6	6.944	7.222	7.127	6.983	7.244	7.243
CCT6P3	7.590	7.666	7.707	7.692	7.405	7.708
CCT6P1	7.590	7.666	7.707	7.692	7.405	7.708

MRPL4	9.842	9.974	10.105	9.980	9.909	9.950
AC245033.4	14.164	14.198	14.199	14.193	14.160	14.234
MRPL2	8.981	9.009	9.296	9.265	9.342	9.111
MRPL3	12.632	12.733	12.843	12.719	12.690	12.680
BORCS8-MEF2B	6.556	6.433	6.864	6.811	7.042	6.828
MRPL1	8.970	9.027	9.307	9.084	9.134	8.868
MRPL9	10.437	10.616	10.687	10.447	10.467	10.426
AC022596.2	7.349	7.328	7.363	7.179	7.347	7.319
WBP1LP2	6.546	6.213	6.060	6.713	6.607	6.480
AL355974.1	5.794	5.419	5.495	5.707	5.780	5.647
RTKN	8.908	8.896	9.057	8.717	8.776	8.782
AC004839.4	6.630	6.972	6.797	6.975	6.779	6.927
CD79B	7.053	6.956	7.159	7.561	7.072	7.322
MEOX1	5.826	6.027	6.204	6.065	6.084	6.106
AC004839.2	6.630	6.972	6.797	6.975	6.779	6.927
CD79A	8.132	8.180	8.408	8.538	8.398	8.141
CBLN4	6.939	6.900	6.865	7.236	7.089	7.021
CBLN2	7.411	7.329	7.012	7.348	7.533	7.456
CBLN3	7.752	7.859	7.759	8.099	8.125	7.950
CBLN1	5.782	6.074	6.037	5.956	5.952	5.892
CYB561A3	9.954	9.959	10.122	9.971	9.886	9.929
SLPI	11.943	11.937	12.058	11.851	11.756	11.989
TSTA3	11.017	11.112	11.283	10.970	10.887	10.967
FXYD6-FXYD2	8.943	8.949	9.255	8.995	8.938	8.782
RPS6KC1	8.602	8.663	8.718	8.105	8.325	8.319
IDO2	5.777	5.727	5.648	5.729	5.871	5.815
IDO1	8.869	8.819	8.884	9.000	9.075	8.884
AC129778.1	8.464	8.364	8.135	8.347	8.305	8.294
LDHAL6B	5.287	5.525	5.535	5.814	5.905	5.802
LDHAL6A	7.699	7.765	7.633	7.967	8.011	7.704
ZNF876P	3.969	3.710	3.596	3.991	3.925	3.853
LINC02656	4.591	4.683	4.446	4.510	4.533	4.755
EIF4BP7	11.753	11.772	11.939	11.764	11.728	11.676
AMHR2	7.978	8.012	8.040	8.211	8.153	8.112
CATSPER1	7.780	7.975	7.913	7.829	7.676	7.951
CATSPER2	7.725	7.587	7.677	7.711	7.740	7.694
CATSPER3	5.756	5.949	5.736	5.879	6.032	6.015
MAPKAP1	8.801	8.967	8.990	8.931	8.857	8.929
TMTC1	6.827	6.846	6.753	7.074	6.903	6.823
SLC22A25	8.652	8.820	8.627	8.456	8.783	8.742
APMAP	9.752	9.803	9.884	9.615	9.607	9.538
FLOT2	10.629	10.792	10.910	10.724	10.675	10.661
CATSPERZ	8.221	8.268	8.083	8.409	8.300	8.267
LINC00654	7.205	7.024	7.184	7.322	7.586	7.400
AL354718.3	7.972	7.912	8.183	8.328	8.259	8.126
LINC00656	6.740	7.079	6.853	6.698	6.742	6.933
LINC00652	8.514	8.396	8.479	8.792	8.771	8.599
LINC00653	6.898	7.157	7.196	7.362	7.309	7.163
CATSPERD	4.438	4.438	4.462	4.753	4.504	4.420
CATSPERE	5.793	5.852	5.753	5.820	5.842	5.690
MIR548N	5.968	5.997	5.943	6.185	6.201	6.060
CATSPERG	7.056	7.213	6.925	7.112	7.255	7.163
LINC00658	5.545	5.536	5.706	5.787	5.627	5.459
LINC00659	6.485	6.621	6.506	6.655	6.621	6.504
CATSPERB	7.430	7.458	7.406	7.303	7.596	7.356
ARAP2	8.196	8.056	7.866	8.057	7.778	8.053

DEDD2	10.864	11.022	11.117	10.950	11.036	11.081
FAM178B	8.454	8.380	8.265	8.508	8.511	8.454
DOLK	8.835	9.017	9.092	8.734	8.870	8.776
AC007663.2	7.209	7.675	7.811	7.465	7.552	7.688
AC133561.1	8.827	8.879	8.945	8.881	8.964	8.865
ENOSF1	9.341	9.315	9.243	9.044	8.971	9.250
CIAPIN1	10.571	10.667	10.756	10.588	10.525	10.554
HSP90AB1	13.296	13.428	13.464	13.438	13.273	13.385
LINC01300	6.108	6.053	5.807	6.358	6.289	6.022
PTP4A1	10.567	10.759	10.690	10.802	10.876	10.728
LINC01304	6.560	6.718	6.804	6.592	6.847	6.878
PTP4A3	8.093	8.172	8.311	8.504	8.266	8.207
PTP4A2	12.700	12.760	12.845	12.693	12.638	12.688
ERG28	10.690	10.703	10.906	10.559	10.628	10.494
TRHDE	9.744	9.799	9.601	9.411	9.359	9.383
AC110760.2	7.086	7.294	6.902	7.062	7.055	7.217
AC110760.1	5.223	5.252	5.221	5.332	5.177	5.308
PA2G4	11.510	11.478	11.623	11.570	11.631	11.385
HEMK1	8.102	8.224	8.296	8.038	8.014	7.971
AC136628.3	7.236	6.877	7.115	7.650	7.276	7.517
NR1I3	6.558	6.677	6.413	6.495	6.493	6.442
NR1I2	8.061	8.048	8.014	8.051	7.923	8.014
GRB2	11.254	11.390	11.516	11.000	11.058	11.160
ULBP2	9.044	9.211	9.219	8.985	9.097	9.173
ULBP3	7.567	7.532	7.826	7.583	7.327	7.650
GRB7	13.988	13.999	14.033	13.881	13.853	13.911
ULBP1	6.639	6.852	6.650	6.789	6.953	6.736
UQCC1	10.085	9.995	10.034	9.998	10.116	10.065
UQCC3	8.176	8.190	8.460	7.955	7.964	8.200
HMGB3P22	5.550	5.673	5.637	5.511	5.732	5.587
HOXC9	7.034	6.602	6.565	6.723	6.886	7.013
HOXC8	7.358	7.189	7.293	7.543	7.570	7.389
HOXC5	7.501	7.782	7.693	7.655	7.596	7.837
HOXC4	8.126	8.141	8.047	8.042	8.005	8.213
HOXC6	8.125	8.389	8.617	8.027	8.268	7.948
OR4F3	7.745	7.729	7.361	7.729	7.847	7.903
UROC1	5.274	5.310	5.561	5.410	5.320	5.450
TUBB2BP1	9.453	9.551	9.668	9.470	9.494	9.423
SUCLG2-AS1	8.111	8.057	8.124	8.127	8.186	8.255
RBMX2	9.434	9.445	9.577	9.213	9.336	9.333
POLR2E	11.327	11.380	11.504	11.195	11.158	11.190
POLR2D	9.440	9.472	9.672	9.351	9.466	9.363
POLR2G	11.090	11.213	11.371	11.160	11.139	11.152
POLR2F	9.529	9.578	9.875	9.661	9.698	9.641
POLR2A	9.385	9.380	9.519	9.178	9.273	9.274
POLR2C	10.716	11.030	11.077	11.016	10.785	10.674
POLR2B	10.274	10.364	10.382	10.363	10.000	10.194
POLR2M	10.331	10.280	10.325	10.168	10.116	10.108
POLR2L	12.021	12.181	12.389	12.282	12.225	12.301
POLR2I	10.627	10.764	10.962	10.534	10.488	10.503
POLR2H	11.166	11.270	11.509	11.371	11.357	11.390
F11R	12.136	12.242	12.354	12.061	11.964	12.018
POLR2J	10.960	11.004	11.108	10.912	10.811	10.780
SERHL	7.373	7.375	7.431	7.242	7.499	7.564
OSER1	9.738	9.782	9.886	9.871	9.784	9.954
DSCAML1	6.596	6.724	6.753	6.684	6.576	6.710

VBP1	11.280	11.359	11.453	11.342	11.381	11.278
TYRO3P	5.821	5.760	5.763	6.428	6.050	6.032
UBE2Q2P6	6.582	6.672	6.703	6.718	6.700	6.647
CTNS	7.553	7.655	7.777	7.789	7.682	7.578
TTTY2B	7.674	7.675	7.508	7.641	7.861	7.792
HAUS6P3	7.239	7.144	7.102	6.091	6.607	6.681
AFDN-DT	6.040	5.964	5.383	5.962	6.176	5.919
ATP12A	7.520	7.747	7.552	7.768	7.893	7.649
ANKRD20A11P	8.236	8.156	8.141	8.146	8.146	8.231
ITFG2	8.070	8.019	7.989	7.852	7.924	7.959
ITFG1	11.419	11.442	11.493	11.321	11.228	11.328
TMEM239	6.862	7.134	6.907	6.762	6.882	6.852
TMEM235	6.087	6.270	6.116	6.055	6.185	6.202
TMEM234	8.068	8.127	8.096	7.910	8.044	7.933
TMEM237	8.944	9.018	9.020	8.427	8.619	8.463
TMEM231	8.080	8.245	8.252	7.888	7.870	7.882
TMEM230	12.150	12.230	12.258	12.092	12.057	12.085
TMEM232	5.688	5.590	5.431	5.607	5.586	5.818
ANKHD1	10.030	10.021	10.062	9.697	9.801	9.935
CTU2	7.913	7.906	8.189	7.810	7.706	7.862
CYP4F62P	5.675	5.822	5.573	6.014	5.852	5.828
MSX1	7.218	7.088	7.242	6.930	7.106	7.023
MSX2	7.580	7.705	7.598	7.393	7.570	7.323
SIN3B	8.100	8.075	8.209	8.320	8.202	8.187
SIN3A	9.349	9.347	9.401	9.183	9.304	9.207
AC011375.1	9.668	9.618	9.641	9.370	9.232	9.624
TVP23C	9.099	9.080	9.036	8.999	9.116	9.206
TVP23B	9.099	9.065	9.036	8.806	9.078	9.206
TVP23A	6.048	5.939	5.934	6.081	6.091	5.909
MIR4527HG	6.884	6.868	6.928	6.942	7.044	7.179
PRRX2-AS1	7.819	7.938	7.953	7.951	8.024	7.981
TAB3	9.953	9.996	9.943	9.688	9.534	9.771
RPS26P3	12.821	12.916	13.024	12.923	12.903	12.978
TAB1	7.730	7.932	7.960	7.799	7.897	8.010
RPS26P6	12.821	12.916	13.024	12.923	12.903	12.978
HYDIN2	4.232	4.169	4.235	4.298	4.540	4.412
Z97206.1	5.666	5.644	5.532	5.690	5.688	5.858
SUMO1	11.662	11.660	11.761	11.772	11.659	11.651
TMCO1	11.658	11.698	11.821	11.624	11.595	11.623
AC010547.1	6.782	6.809	6.943	7.079	6.859	6.799
SUMO2	12.617	12.628	12.692	12.617	12.655	12.614
TMCO4	8.276	8.295	8.304	8.108	8.377	8.388
SUMO4	10.610	10.773	10.808	10.665	10.788	10.722
TMCO6	8.060	8.089	8.074	7.662	8.017	7.862
AC010547.4	7.430	7.687	7.645	7.471	7.195	7.620
HSF2	7.117	7.165	7.309	6.741	6.807	7.159
ANKIB1	10.143	10.106	10.141	9.857	9.946	9.898
EEF2K	10.154	10.210	10.330	10.126	10.151	10.188
MIB1	9.167	9.072	9.227	8.864	8.929	8.813
MIB2	7.092	7.178	7.097	6.917	7.054	7.088
TOR3A	9.039	9.054	9.377	8.969	9.064	8.858
AC019097.1	9.705	9.757	9.710	9.291	9.331	9.317
TGFB1I1	8.702	8.861	9.042	8.470	8.859	8.350
CXCL11	6.756	7.043	6.615	7.084	7.415	6.728
AC090114.2	6.972	6.837	6.787	7.108	6.943	6.877
AC090114.1	13.722	13.830	13.901	13.964	13.777	13.914

CXCL12	8.383	8.272	8.278	8.310	8.489	8.343
CXCL14	6.760	6.621	6.439	6.686	6.879	6.982
CXCL17	8.600	8.550	8.616	8.521	8.449	8.620
CXCL16	9.530	9.697	9.705	9.495	9.682	9.554
HCN1	8.072	8.174	8.207	8.263	8.467	8.532
HCN3	7.995	8.115	8.130	8.273	8.007	8.244
HCN2	7.902	7.916	7.980	7.843	7.838	7.820
HCN4	7.543	7.599	7.556	7.741	7.851	7.581
AC136612.1	7.710	7.765	7.579	7.766	7.843	7.804
LINC02511	5.014	5.207	5.046	4.964	5.028	5.267
LINC02510	4.321	4.386	4.189	4.330	4.293	4.455
LINC02517	7.143	7.186	7.101	7.470	7.443	7.346
AC079089.2	9.111	8.784	9.129	9.047	9.021	8.412
LRIT1	6.290	6.425	5.881	6.849	6.489	6.420
GOLGA7B	7.288	7.409	7.450	7.746	7.506	7.534
SPTY2D1OS	6.776	6.870	6.879	7.156	7.042	6.889
PEPD	10.130	10.177	10.278	10.167	10.032	10.160
UACA	8.824	8.762	8.960	8.537	8.606	8.518
ZIC4	6.354	6.179	6.339	6.308	6.273	6.135
ZIC5	6.604	6.866	6.814	6.751	6.815	6.853
INGX	6.674	6.676	6.895	7.044	7.131	6.876
ZIC3	6.169	6.645	6.434	6.563	6.499	6.653
ZIC1	6.251	6.171	6.270	6.282	6.383	6.390
ING4	8.733	8.752	8.760	8.869	8.698	8.744
ING5	8.000	7.980	8.123	8.034	7.937	7.986
C21orf58	8.165	8.280	8.191	8.319	8.120	8.188
ING1	8.263	8.385	8.184	8.242	8.173	8.268
ING2	8.201	8.219	8.485	8.129	8.221	8.156
ING3	8.103	8.240	8.370	8.398	8.481	8.116
SGK1	7.820	7.721	7.696	7.373	7.671	7.601
AC097652.1	5.164	5.536	5.218	5.249	5.266	5.540
AC068135.2	5.858	5.962	5.485	5.893	6.159	5.883
RN7SKP187	7.418	7.539	7.537	7.628	7.629	7.579
ACP4	7.888	8.182	7.992	7.794	7.847	8.008
ACP5	7.104	7.255	7.292	7.541	7.493	7.469
ACP6	11.196	11.104	11.325	10.801	10.774	10.853
ACP1	11.226	11.279	11.400	11.067	10.964	11.078
ACP2	9.820	9.822	9.887	9.629	9.749	9.829
SNIP1	8.377	8.478	8.479	8.472	8.537	8.612
GLA	9.877	9.986	10.121	9.907	9.871	9.919
CCT8L2	6.884	7.144	6.966	7.155	7.292	6.918
FSBP	7.477	7.532	7.528	7.400	7.446	7.287
GLS	9.989	10.082	10.042	9.957	9.854	10.046
RNU6-195P	8.000	8.019	7.910	8.048	7.958	8.048
AL162742.2	8.328	8.260	8.068	8.170	8.333	8.227
TMEM17	8.384	8.506	8.633	7.984	8.622	8.061
TMEM18	9.762	9.794	9.929	9.657	9.664	9.608
TMEM19	9.853	9.949	10.081	9.826	9.887	9.854
NT5C3A	11.665	11.677	11.683	11.774	11.862	11.359
NT5C3B	10.300	10.380	10.615	10.512	10.380	10.382
AMPH	6.677	6.488	6.490	6.649	6.600	6.675
AC026401.3	8.099	7.982	8.231	7.620	7.926	7.816
ZNF639	10.160	10.231	10.339	10.168	9.963	10.171
ZNF638	10.566	10.560	10.527	10.498	10.360	10.288
ACPP	7.311	7.090	7.452	7.234	7.557	7.423
ZNF630	7.481	7.475	7.222	7.312	7.552	7.708

EDDM13	8.014	7.710	7.775	8.319	8.004	7.986
UGT2B28	8.916	8.824	8.983	8.796	8.862	8.989
ANKRD20A1	8.236	8.156	8.141	8.146	8.143	8.231
RPL21P93	12.617	12.677	12.802	12.784	12.769	12.841
MINAR1	6.040	6.176	6.226	6.204	6.200	6.300
ZBTB49	7.073	7.271	7.280	7.229	7.273	7.269
ACER3	9.239	9.205	9.224	8.990	8.960	9.048
PRSS12	8.502	8.481	8.637	8.173	8.220	8.284
HLA-DRA	8.663	8.804	8.671	8.843	8.826	8.813
PRSS16	8.458	8.361	8.621	8.422	8.360	8.374
MEX3D	9.720	9.944	9.949	9.764	9.719	9.614
MEX3B	6.539	6.434	6.456	6.660	6.334	6.615
MEX3C	9.444	9.538	9.667	9.317	9.357	9.370
MEX3A	8.415	8.492	8.552	9.045	8.727	8.666
LACTB2	10.173	10.201	10.343	10.192	10.415	10.050
AIFM1	10.016	10.038	10.263	9.859	9.852	9.864
BTRC	8.353	8.412	8.407	8.422	8.607	8.388
LRRC10B	7.370	7.288	7.168	7.381	7.359	7.546
UROS	9.597	9.592	9.776	9.570	9.476	9.532
PET100	9.342	9.460	9.558	9.416	9.426	9.493
CLCA3P	3.781	3.691	4.022	3.717	3.766	3.685
STYK1	7.882	8.075	7.992	7.728	7.693	7.919
AC124068.2	6.560	6.502	6.481	6.592	6.639	6.695
SCRT1	7.051	7.068	6.999	7.095	6.950	6.978
AC007622.2	4.827	5.221	4.924	4.609	4.921	4.698
AL445072.1	3.887	4.046	3.926	4.030	3.960	4.108
UBLCP1	9.576	9.664	9.539	9.168	9.563	9.504
AC073610.2	10.564	10.848	10.899	10.685	10.699	10.647
ROR2	4.971	4.986	5.171	5.330	5.087	4.979
MTMR9	9.315	9.516	9.482	9.482	9.636	9.494
MTMR8	7.825	8.049	8.129	8.328	8.093	8.287
MTMR7	6.618	6.702	6.722	6.640	6.827	6.650
MTMR6	8.604	8.640	8.680	8.700	8.586	8.700
MTMR4	9.085	9.232	9.293	9.028	8.944	9.138
MTMR3	9.316	9.408	9.416	9.252	9.407	9.373
MTMR2	8.476	8.405	8.330	8.386	8.539	8.484
MTMR1	10.494	10.556	10.601	10.310	10.269	10.291
NAAA	8.113	8.152	8.222	7.987	8.200	8.213
TMEM256-PLSCF	10.512	10.696	10.703	10.606	10.668	10.682
AC069287.1	7.159	6.862	6.635	6.551	6.694	6.987
KIAA0895LP1	5.656	5.977	5.730	5.832	5.999	6.004
AP000351.7	7.447	7.617	7.613	7.242	7.700	7.578
Z98883.1	9.091	9.072	8.959	9.153	9.160	9.170
DNASE2B	5.320	5.307	5.162	5.493	5.329	5.323
C5orf66-AS1	5.659	5.607	5.701	5.640	5.801	5.660
GGACT	7.301	7.354	7.272	7.152	7.104	7.288
PPATP1	9.591	9.542	9.658	9.623	9.617	9.494
CYB561	10.675	10.729	10.913	10.809	10.906	10.792
MIR5195	4.456	4.724	4.497	4.383	4.525	4.513
AC104964.4	3.848	3.917	3.935	4.055	4.059	3.935
AC243791.4	11.076	11.006	10.913	10.944	11.072	11.072
TACSTD2	12.988	13.081	13.142	12.928	12.804	12.907
NABP1	8.943	9.011	8.798	8.594	8.556	8.807
MIR5193	8.415	8.606	8.651	8.312	8.504	8.406
FARP2	9.005	8.992	9.208	8.902	8.809	8.915
FARP1	8.916	8.946	9.112	8.831	8.731	8.911

C9orf40	7.575	7.528	7.626	7.439	7.496	7.631
C9orf43	5.829	6.009	6.076	6.102	6.139	6.035
TEK	8.949	8.837	8.901	8.838	8.858	9.004
AC242426.2	9.580	9.639	9.692	9.483	9.340	9.409
SNAP47	9.311	9.377	9.476	9.156	9.174	9.293
DEPDC1	8.830	8.914	8.930	8.594	8.670	8.617
DEPDC7	4.983	5.306	5.296	4.708	4.641	4.898
DEPDC4	4.372	4.382	4.248	4.628	4.499	4.609
DEPDC5	7.994	8.003	8.219	8.055	7.961	7.928
SCYL2	10.335	10.401	10.383	10.378	10.357	10.465
SCYL3	8.546	8.674	8.717	8.563	8.562	8.753
SCYL1	8.504	8.549	8.797	8.451	8.276	8.340
STXBP1	8.769	8.844	8.936	8.623	8.701	8.728
STXBP3	9.608	9.581	9.674	9.438	9.480	9.557
STXBP2	9.701	9.711	9.826	9.737	9.674	9.698
STXBP5	7.146	7.408	7.421	7.392	7.590	7.426
STXBP4	7.065	7.049	7.133	6.873	6.818	6.719
STXBP6	7.640	7.730	7.917	7.708	7.816	7.680
AL161636.1	6.430	6.603	6.556	6.620	6.795	6.551
AC012467.2	6.814	6.936	7.025	6.842	6.647	6.924
S1PR3	7.198	7.243	7.094	7.424	7.215	7.265
S1PR2	8.327	8.229	8.343	8.453	8.484	8.302
S1PR1	6.114	6.204	6.161	6.220	6.111	6.242
TMEM200A	4.313	4.354	4.286	4.601	4.196	4.575
S1PR5	6.856	6.960	6.854	6.701	6.956	6.786
S1PR4	7.973	7.794	7.989	8.209	8.164	7.992
ENY2	9.882	9.913	10.052	9.991	9.709	9.945
CCZ1	11.738	11.824	11.875	11.801	11.716	11.768
AC087289.2	5.898	5.783	5.764	5.659	5.862	5.889
AC087289.3	9.809	9.809	10.055	9.834	9.852	9.818
AC012184.3	4.565	4.621	4.606	4.367	4.486	4.610
EXOC6	8.874	9.057	9.142	9.155	8.870	9.084
AL109946.1	5.117	5.064	4.959	5.019	5.007	5.228
AC011472.2	6.956	7.183	7.038	7.182	7.161	7.153
ICE2	8.485	8.640	8.583	8.540	8.438	8.671
AC107982.1	3.910	3.950	3.754	3.810	3.836	3.547
ICE1	8.309	8.387	8.419	8.177	8.043	8.311
CDK1	10.478	10.537	10.629	10.024	10.055	10.110
CDK2	10.241	10.251	10.280	9.781	9.842	10.020
CDK3	8.004	7.971	8.076	8.002	8.159	8.133
CDK4	12.023	12.059	12.223	11.931	11.814	11.903
CDK5	8.885	8.983	9.129	8.970	8.960	9.006
CDK6	9.773	9.708	9.992	9.601	9.947	9.434
CDK7	9.039	9.118	9.293	8.902	8.991	8.997
CDK8	7.388	7.410	7.499	7.531	7.364	7.512
HAPLN4	7.769	7.557	7.726	7.527	7.826	7.653
RPL23AP25	7.667	7.929	8.164	7.639	7.672	7.716
HAPLN1	6.849	7.088	6.855	7.150	7.150	7.165
RPL23AP22	5.046	4.991	4.882	5.297	5.190	5.148
HAPLN3	8.465	8.637	8.685	8.797	8.767	8.689
HAPLN2	6.774	6.732	6.878	7.061	7.060	6.867
AL137003.1	5.433	5.435	5.424	5.630	5.691	5.504
RERE	9.460	9.503	9.496	9.109	9.021	9.184
RERG	9.652	9.565	9.671	9.334	9.234	9.446
DMBX1	4.684	4.678	4.459	4.654	4.779	4.902
SUPT3H	7.904	8.072	8.002	8.005	7.952	7.866

TNFRSF18	7.147	7.317	7.348	7.709	7.724	7.370
PRKCI	12.237	12.192	12.391	12.214	12.262	11.937
PRKCH	9.568	9.478	9.593	9.326	9.272	9.349
PRKCA	9.973	9.928	9.941	9.594	9.649	9.808
PRKCB	7.847	7.886	7.779	7.793	7.813	7.936
PRKCE	8.342	8.442	8.425	8.465	8.383	8.250
PRKCD	8.589	8.530	8.518	8.460	8.143	8.629
PRKCG	8.732	8.614	8.643	8.599	8.640	8.706
PRKCZ	8.964	8.964	9.163	8.899	8.785	8.773
RER1	11.324	11.416	11.487	11.292	11.318	11.135
SGSM2	10.155	10.062	10.258	9.910	9.960	10.065
SGSM1	8.454	8.106	8.339	8.445	8.440	8.463
HM13	10.760	10.851	10.894	10.935	10.887	10.800
CKLF-CMTM1	11.156	11.428	11.428	11.266	11.398	11.369
AC005747.1	8.396	8.329	8.298	8.456	8.425	8.497
CCL15-CCL14	7.686	7.980	7.709	8.014	8.015	7.939
BX248516.1	12.229	12.254	12.337	12.254	12.175	12.212
AC074386.1	6.732	6.737	6.688	6.746	6.683	6.540
MUL1	9.774	9.949	9.963	9.911	9.876	9.961
AP000253.1	6.040	6.011	5.994	6.220	6.162	6.273
AC093915.1	5.378	5.149	4.871	5.123	5.162	5.077
PHLPP2	8.785	8.798	8.625	8.565	8.446	8.777
PPIL2	9.100	9.244	9.261	9.124	9.134	9.193
PPIL3	9.776	9.867	10.077	9.882	9.932	9.872
PPIL4	7.240	7.457	7.481	7.171	7.056	7.149
PPIL6	6.539	6.458	6.597	6.700	6.565	6.719
CCDC96	6.595	6.817	6.754	6.494	6.695	6.704
CCDC97	8.965	9.099	9.155	8.935	8.919	8.875
CCDC91	7.558	7.540	7.586	7.284	7.362	7.454
CCDC92	10.131	10.058	10.165	9.934	9.862	9.886
CCDC93	8.401	8.319	8.426	8.295	8.010	8.247
ZNF570	6.269	6.189	6.322	6.185	6.512	6.306
AC021078.1	11.688	11.634	11.844	11.621	11.544	11.686
ZNF572	6.636	6.586	6.554	6.588	6.626	6.581
ZNF573	6.283	6.657	6.654	5.941	6.224	6.037
AC093909.4	3.823	4.097	4.035	4.215	4.032	4.164
ZNF575	8.193	8.090	8.031	8.135	8.339	8.140
ZNF576	8.863	8.937	8.887	8.804	8.889	8.857
ZNF577	7.511	7.657	7.700	7.569	7.741	7.582
ZNF579	10.013	10.105	10.223	10.102	10.000	10.005
MAP3K21	9.054	8.900	9.122	8.781	8.694	8.740
MAP3K20	8.970	8.996	9.004	8.908	8.868	9.123
SETDB2	7.041	6.928	7.023	6.892	7.063	7.032
LRRC37A5P	5.614	5.710	5.410	5.663	5.616	5.455
KCNAB1	8.486	8.328	8.161	8.289	8.547	8.474
KCNAB3	6.750	6.994	6.954	6.943	7.072	6.953
KCNAB2	7.696	7.728	7.793	7.760	7.913	7.799
RPL14	12.602	12.676	12.871	12.830	12.747	12.797
RPL15	12.772	12.801	12.947	12.791	12.710	12.834
TOMM34	10.683	10.717	10.859	10.547	10.599	10.644
RPL17	13.304	13.343	13.490	13.360	13.298	13.417
RPL10	13.794	13.873	13.940	13.883	13.836	13.884
RPL11	13.285	13.365	13.443	13.379	13.179	13.347
RPL12	13.555	13.621	13.669	13.572	13.470	13.564
LINC01409	6.311	6.208	6.614	5.780	5.917	6.267
GPATCH4	9.603	9.494	9.629	9.443	9.537	9.472

LINC01405	5.229	4.745	5.276	5.429	5.470	5.472
GPATCH2	8.328	8.258	8.404	8.460	8.338	8.551
GPATCH3	8.960	8.836	9.022	8.956	8.942	8.920
CCDC9B	8.384	8.349	8.513	8.424	8.239	8.291
GPATCH1	8.735	8.673	8.777	8.395	8.223	8.389
NAA10	9.609	9.758	9.890	9.735	9.584	9.643
LINC02619	3.778	3.686	3.871	3.717	4.097	3.970
SYK	8.727	8.656	8.835	8.458	8.450	8.476
NINL	7.402	7.436	7.475	7.250	7.440	7.464
SYP	7.861	7.778	7.519	7.678	7.784	7.620
MIPEPP3	4.041	3.998	4.126	4.011	3.712	3.919
PDZK1IP1	11.354	11.483	11.662	11.420	11.107	11.336
MBL1P	6.115	6.053	6.116	6.152	6.291	6.196
AC021683.1	6.314	6.275	6.070	6.400	6.269	6.305
STIL	9.294	9.298	9.340	8.792	9.022	8.817
AC021683.3	6.195	6.157	6.195	6.423	6.452	6.255
AP001781.2	7.896	7.902	8.074	7.660	8.062	7.824
PHKA1	8.305	8.331	8.358	8.221	8.258	8.350
PHKA2	9.469	9.575	9.572	9.419	9.367	9.517
PCAT19	6.660	6.880	7.112	6.967	6.934	6.884
PCAT18	5.670	5.794	6.139	5.866	5.829	6.001
EEF1AKMT4	8.997	9.014	9.143	9.026	9.093	8.996
EEF1AKMT3	9.350	9.313	9.330	9.217	9.255	9.364
EEF1AKMT2	6.337	6.394	6.569	6.101	6.318	6.500
EEF1AKMT1	7.829	7.885	8.084	7.814	7.951	7.941
FAM162A	11.021	11.080	11.310	11.264	11.248	11.209
FAM162B	6.475	6.506	6.621	6.570	6.670	6.600
FTH1P2	13.980	13.991	14.082	13.979	13.810	13.985
FTH1P5	13.685	13.744	13.746	13.727	13.739	13.846
FGGY	8.081	8.223	8.315	8.183	8.117	8.075
TOP3A	8.396	8.606	8.431	8.432	8.493	8.463
TOP3B	7.548	7.599	7.762	7.392	7.440	7.365
MIR7-1	12.826	12.956	12.948	12.901	12.847	12.836
AL050335.1	7.630	7.650	7.717	7.732	7.790	7.858
ILK	11.241	11.307	11.425	11.196	11.245	11.273
LINC01731	7.696	7.533	7.547	7.848	7.724	7.748
RABEP2	9.307	9.239	9.376	9.392	9.403	9.368
RABEP1	8.497	8.622	8.683	8.302	8.059	8.365
MORF4L2	13.242	13.319	13.307	13.310	13.234	13.312
MORF4L1	12.787	12.825	12.897	12.773	12.722	12.781
AC090772.3	8.604	8.600	8.569	8.226	8.347	8.556
AC090772.5	6.916	6.774	6.857	6.093	6.302	6.386
RABEPK	8.953	8.995	9.093	8.875	9.078	9.054
FLT3LG	5.886	5.679	5.710	5.823	6.232	6.093
FAM171A2	7.914	7.939	7.948	8.052	7.921	7.825
FAM171A1	7.720	7.606	7.541	8.128	8.058	7.980
TBL1XR1	12.354	12.415	12.430	12.341	12.266	12.292
PAX8-AS1	10.622	10.788	10.765	10.699	10.598	10.612
SNRK	9.502	9.603	9.588	9.530	9.461	9.583
C12orf80	8.313	8.279	8.241	8.473	8.622	8.505
IL6	7.502	7.567	7.538	8.305	8.298	8.352
IL7	4.883	5.172	5.029	4.891	5.198	4.960
IL4	5.637	5.571	5.849	5.665	5.691	5.620
IL5	4.739	4.327	4.643	4.686	4.625	4.595
IL2	3.958	4.070	4.254	4.231	4.275	4.241
IL3	5.512	5.927	5.468	5.762	5.691	5.582

CFC1B	7.590	7.407	7.658	7.744	7.615	7.552
PAXBP1-AS1	5.951	5.841	5.799	5.824	5.950	5.782
IL9	4.269	4.529	4.206	4.333	4.550	4.652
DTX4	11.325	11.378	11.487	11.393	11.291	11.261
SAT1	12.143	12.227	12.306	12.073	12.215	12.040
SAT2	9.166	9.298	9.424	9.234	9.198	9.289
SPINDOC	9.108	9.157	9.240	9.368	9.597	9.174
DTX2	7.959	8.172	8.373	8.235	8.212	8.099
DTX3	8.624	8.433	8.700	8.396	8.514	8.420
AC127496.1	7.893	7.925	7.565	7.744	7.778	7.590
AC127496.5	6.668	6.627	6.697	6.730	6.790	6.682
CENPM	9.476	9.275	9.719	9.050	9.130	9.060
AC127496.7	8.547	8.283	8.350	8.370	8.555	8.415
OXR1	8.465	8.297	8.576	8.094	8.568	8.258
CMAHP	6.574	6.451	6.176	6.575	6.387	6.627
RB1CC1	9.003	9.096	9.073	8.889	9.048	8.907
SPRN	7.815	7.885	7.838	7.935	7.983	7.967
AKR1E2	8.734	8.840	8.663	8.903	8.951	8.829
NSF	10.931	10.911	11.051	10.632	10.660	10.790
LINC02060	4.228	4.180	4.376	4.286	4.330	4.294
SENP3-EIF4A1	13.137	13.230	13.247	13.064	13.071	13.089
DMC1	6.262	6.171	6.125	5.974	6.232	6.091
CENPC	7.246	7.183	7.118	6.572	6.792	6.612
CENPA	9.109	8.940	9.030	8.554	8.890	8.677
RPS15AP1	13.687	13.656	13.782	13.960	13.843	13.987
AC008758.1	6.587	6.862	6.969	6.702	7.123	6.785
RNF212B	6.321	6.227	6.290	6.207	6.249	6.249
CYCSP55	13.090	13.181	13.174	13.245	13.190	13.207
ODC1-DT	7.014	7.138	7.123	7.214	7.108	7.130
GSTCD	8.229	8.256	8.273	8.159	8.167	8.292
WDCP	6.903	7.113	7.042	6.968	6.858	7.004
PARS2	8.552	8.564	8.549	8.690	8.658	8.553
SSH1	9.543	9.450	9.708	9.464	9.680	9.111
MCF2L2	3.803	3.760	3.840	3.744	3.814	3.692
MAPK10-AS1	7.502	7.617	7.486	7.513	7.651	7.615
PI4K2B	9.321	9.419	9.460	9.221	9.325	9.193
PI4K2A	9.167	9.432	9.343	9.119	9.106	9.065
INAFM1	9.046	9.111	9.109	8.765	9.005	8.901
ARHGEF7-IT1	5.783	5.849	5.819	5.468	5.713	5.711
INAFM2	8.624	8.775	8.906	8.696	8.784	8.682
NHLRC3	7.640	7.789	7.637	7.437	7.468	7.688
NHLRC2	7.007	6.829	7.247	6.563	6.463	6.639
NHLRC4	8.585	8.542	8.638	8.468	8.499	8.645
GSE1	9.842	9.790	9.826	9.463	9.642	9.422
CGREF1	7.619	7.832	7.902	7.800	7.641	7.502
MAP3K14-AS1	6.668	6.804	6.802	6.910	6.904	6.878
AC090510.1	6.814	6.948	6.923	6.802	6.956	6.889
AC051649.5	8.109	8.099	8.208	8.273	8.408	8.309
AC051649.4	8.109	8.099	8.208	8.273	8.408	8.309
GSEC	7.288	7.347	7.233	7.243	7.265	7.421
RARA-AS1	9.820	9.736	9.906	9.677	9.613	9.632
AC051649.2	8.109	8.099	8.208	8.273	8.408	8.309
AC037486.1	7.152	7.437	6.891	7.062	7.148	7.352
AC108726.1	5.589	5.587	5.213	5.684	5.528	5.926
BRSK1	7.511	7.353	7.697	7.685	7.798	7.354
TLR2	6.287	6.213	6.302	6.296	6.158	6.287

TLR3	7.975	7.844	8.048	7.728	8.072	7.856
TLR1	5.084	5.086	4.897	5.120	5.066	4.911
TLR6	6.729	6.893	6.598	7.184	6.880	6.934
TLR7	7.270	7.050	6.957	7.014	7.160	7.030
TLR4	6.387	6.602	6.616	6.700	6.479	6.630
TLR5	8.264	8.199	8.391	8.141	8.276	8.262
TLR8	6.236	6.407	6.035	6.309	6.427	6.589
TLR9	6.761	6.880	6.941	6.630	6.942	7.036
NUP205	9.457	9.378	9.482	9.167	9.115	9.256
AC092338.2	4.018	3.926	3.944	4.155	4.187	4.020
ARID4A	7.883	7.877	7.830	7.385	7.748	7.485
ARID4B	9.699	9.740	9.918	9.500	9.613	9.638
COX6B1P3	8.031	8.048	8.242	8.164	8.074	8.316
MMS19	9.221	9.221	9.322	9.107	9.080	9.115
ADGRG7	6.108	6.086	5.945	6.070	5.996	5.974
ADGRG6	10.515	10.601	10.596	10.426	10.331	10.378
ADGRG5	6.402	6.315	6.174	6.288	6.320	6.377
PLEKHA7	9.323	9.297	9.358	9.113	9.080	9.210
UBXN8	8.009	8.029	8.048	7.739	7.907	7.848
TFE3	9.526	9.536	9.592	9.634	9.725	9.560
UBXN4	10.377	10.485	10.370	10.288	10.491	10.416
UBXN6	9.391	9.319	9.564	9.331	9.258	9.181
UBXN7	8.757	8.820	8.859	8.305	8.435	8.413
UBXN1	9.887	9.960	10.121	9.898	9.966	9.832
ADGRG2	6.230	6.261	6.117	6.430	6.480	6.272
FMC1	8.064	8.034	8.248	8.170	8.217	8.109
TFEC	4.566	4.530	4.687	4.770	4.579	4.740
TFEB	8.619	8.734	8.684	8.682	8.746	8.804
AL122035.2	5.188	5.208	4.973	5.120	5.359	4.980
KIAA1109	8.211	8.061	8.145	7.885	7.776	8.091
INSM1	4.614	4.557	4.568	4.398	4.647	4.563
ECI2	9.712	9.733	9.886	9.601	9.622	9.732
ECI1	10.078	10.128	10.398	9.940	9.944	9.919
AC025674.4	4.509	4.255	4.248	4.300	4.233	4.442
SLC49A3	5.960	5.894	6.227	6.356	6.112	5.891
LRRC57	7.126	7.458	7.366	6.711	7.137	7.080
SMCHD1	8.681	8.750	8.795	8.735	8.733	8.711
AL645730.1	6.798	6.808	6.688	7.025	6.661	6.801
JAZF1	8.038	8.228	8.153	7.832	8.004	7.841
AP001273.1	4.482	4.222	4.047	4.293	4.272	4.460
C6orf163	6.018	6.144	6.215	6.429	6.270	6.193
SYVN1	12.943	13.018	13.182	13.107	12.912	13.120
DRAXIN	6.551	6.622	6.560	6.561	6.794	6.711
AC125257.1	10.394	10.351	10.343	10.328	10.204	10.336
AC024051.9	7.882	7.717	7.633	8.115	8.061	7.935
AC024051.4	7.882	7.717	7.633	8.115	8.061	7.935
AC024051.5	7.882	7.717	7.633	8.115	8.061	7.935
AC024051.6	7.882	7.717	7.633	8.115	8.061	7.935
AC024051.7	7.882	7.717	7.633	8.115	8.061	7.935
AC024051.1	7.882	7.717	7.633	8.115	8.061	7.935
AC024051.2	7.882	7.717	7.633	8.115	8.061	7.935
AC024051.3	7.882	7.717	7.633	8.115	8.061	7.935
KIF1C-AS1	7.721	7.763	7.733	7.800	7.979	7.799
TMEM243	9.024	8.989	9.232	8.784	8.926	8.888
DGUOK	10.628	10.595	10.863	10.610	10.611	10.554
MAK16	11.363	11.321	11.438	11.375	11.253	11.324

AC105383.1	3.958	3.797	3.628	4.153	3.947	4.014
AMZ2P1	8.483	8.433	8.557	8.547	8.438	8.341
EEF1B2P3	12.241	12.317	12.505	12.366	12.368	12.394
EEF1B2P2	12.241	12.317	12.505	12.366	12.368	12.394
AWAT1	6.425	6.480	6.389	6.392	6.484	6.433
EEF1B2P6	12.241	12.317	12.505	12.366	12.368	12.394
C5orf22	8.840	8.963	8.887	8.536	8.712	8.787
C5orf24	10.653	10.533	10.641	10.533	10.504	10.553
AC015631.1	4.377	4.230	4.437	4.331	4.355	4.294
VLDLR-AS1	7.820	7.956	7.698	7.709	7.865	7.961
ANLN	11.037	11.093	10.980	10.655	10.796	10.788
SSBP1	11.168	11.229	11.301	10.970	11.046	10.962
SSBP3	7.858	7.741	7.704	8.078	8.064	7.811
SSBP2	8.220	8.197	8.331	8.132	8.331	8.206
BCL10	11.898	11.853	11.915	11.868	11.920	11.812
IL27RA	7.254	7.239	7.374	7.108	7.332	7.109
AKR1C6P	7.203	7.362	7.091	7.220	7.042	7.334
ZCCHC12	6.370	6.092	6.391	6.566	6.207	6.405
AC138207.8	3.925	3.859	3.958	3.926	3.930	3.804
ZNF84-DT	7.151	7.387	7.308	7.362	7.539	7.618
POGLUT1	8.406	8.386	8.330	8.301	8.443	8.356
POGLUT2	6.090	5.946	6.189	5.795	5.822	5.862
PGGHG	8.045	8.153	8.023	8.192	8.186	8.180
MIF-AS1	6.779	7.101	7.143	6.732	7.034	7.076
KIR3DX1	7.054	6.997	6.606	6.949	7.057	6.836
AMZ2	10.684	10.819	10.972	10.646	10.695	10.670
VN1R3	5.825	5.809	5.507	5.478	5.716	5.674
HBQ1	6.702	6.866	6.897	6.946	7.008	6.929
FOXN3-AS1	7.849	8.043	8.050	7.934	8.064	7.909
FOXN3-AS2	6.580	6.755	6.630	6.896	6.780	6.858
ZNF710-AS1	8.376	8.327	8.285	8.005	8.020	8.140
NPEPPSP1	10.910	10.920	10.896	10.979	11.199	10.847
AC010198.1	4.050	3.952	3.938	4.103	4.031	4.142
AC008467.1	7.408	7.398	7.200	7.466	7.390	7.615
FTH1P16	13.980	13.991	14.082	13.979	13.810	13.985
FTH1P11	13.980	13.991	14.082	13.979	13.810	13.985
DHX29	8.346	8.516	8.540	8.047	8.109	8.114
OR51A10P	5.997	6.080	6.258	6.196	6.125	6.169
SH3GL1P2	6.951	7.351	7.436	7.174	7.321	7.273
SH3GL1P1	4.269	4.489	4.298	4.169	4.286	4.535
EIF2AK3-DT	6.561	6.769	6.753	6.496	6.566	6.658
ZNF778	9.258	9.168	9.209	9.241	9.143	9.265
ZNF776	8.781	8.699	8.718	8.064	8.336	8.488
ZNF777	7.398	7.522	7.767	7.235	7.383	7.323
ZNF775	7.894	7.943	7.998	8.085	7.832	7.810
ZNF770	8.260	8.431	8.304	8.042	8.208	7.983
ZNF771	8.292	8.301	8.520	8.364	8.356	8.274
FBXW12	6.516	6.374	6.360	6.447	6.626	6.569
FBXW10	7.433	7.263	7.209	7.520	7.537	7.656
FBXW11	9.896	9.916	9.682	9.539	9.682	9.666
CCDC142	10.914	11.007	11.160	10.889	10.842	10.942
SNORD35A	13.368	13.354	13.450	13.348	13.261	13.358
AC107884.1	7.097	7.251	6.905	7.132	7.201	7.172
SMIM31	5.840	6.050	6.012	6.166	6.347	5.893
BAZ2A	9.357	9.564	9.751	9.576	9.456	9.362
BAZ2B	8.988	8.908	8.932	8.746	8.737	8.806

SMIM32	6.918	7.048	6.965	7.090	7.224	7.038
CYP11A1	6.054	6.128	5.894	5.912	6.099	5.994
RBMY3AP	7.051	7.089	7.274	7.856	7.356	7.452
LCTL	4.005	4.025	4.056	3.828	3.841	3.945
GDF15	11.407	11.423	11.523	11.243	11.330	11.053
GDF11	8.272	8.261	8.332	8.503	8.472	8.465
GDF10	4.780	4.771	4.781	4.918	4.855	4.959
SPRTN	5.669	5.563	5.702	5.310	5.490	5.632
CCDC144NL-AS1	6.930	6.729	6.728	7.029	6.836	7.040
RPH3AL	8.138	8.121	8.166	7.751	7.895	8.048
AC020928.1	7.584	7.650	7.565	7.654	7.641	7.657
IAPP	6.072	6.083	5.832	6.395	6.184	6.229
AC002316.1	8.532	8.470	8.570	8.247	8.242	8.244
GZMB	6.440	6.402	6.418	6.514	6.489	6.638
GZMM	6.632	6.723	6.746	6.847	6.761	6.793
AL162632.3	6.463	6.497	6.291	6.400	6.307	6.303
GZMH	7.118	6.845	6.823	7.223	7.296	7.255
GZMK	7.401	7.400	7.331	7.652	7.734	7.457
MSRB2	9.165	9.301	9.457	8.942	9.014	8.998
MYH10	10.399	10.300	10.430	10.117	10.093	10.178
MYH11	8.261	8.067	8.035	8.346	8.423	8.321
SMC5-AS1	6.991	7.089	7.197	7.155	7.013	7.076
MYH13	6.727	6.738	6.698	6.801	6.763	6.987
MYH14	10.928	11.025	11.171	10.948	11.135	11.105
MYH15	6.213	5.961	5.963	6.228	6.028	6.088
MYH16	5.088	4.857	4.861	4.986	5.012	4.884
LMF2	11.023	11.086	11.177	10.979	10.951	10.975
LMF1	8.281	8.395	8.477	8.375	8.270	8.231
RTRAF	11.872	12.014	12.112	11.973	11.895	11.961
PIK3AP1	11.820	11.722	11.846	11.669	11.601	11.616
SYCP2	7.791	7.666	7.697	7.154	7.061	7.507
RSPH14	7.856	7.768	7.609	7.803	7.825	7.881
SLCO4A1	10.747	10.864	11.044	10.987	10.984	10.916
AC233300.1	6.325	6.338	6.283	6.352	6.382	6.293
AC136616.1	8.827	8.879	8.945	8.881	8.964	8.865
LEF1	7.396	7.348	7.329	7.533	7.547	7.435
RNU7-45P	7.460	7.190	6.535	6.511	6.772	6.888
MTFMT	8.105	8.108	8.309	8.045	7.925	8.054
NAA38	9.795	9.844	10.094	9.825	9.780	9.771
NAA35	9.474	9.427	9.580	8.958	9.521	9.129
NAA30	9.178	9.161	9.245	8.920	9.101	9.136
RPS10P3	13.722	13.830	13.901	13.964	13.777	13.914
RPS10P2	7.899	8.109	7.912	7.935	8.291	8.163
RPS10P5	10.137	10.291	10.402	10.294	10.401	10.236
RPS10P7	6.805	6.728	6.510	6.905	6.839	6.781
ATAD2B	7.182	7.331	7.416	7.344	7.180	7.358
PLGLA	4.558	4.375	4.427	4.329	4.533	4.517
PRKG1-AS1	5.556	5.599	5.567	5.823	5.680	5.809
AC020658.7	5.411	5.509	5.679	5.838	5.523	5.550
EBLN3P	9.316	9.194	9.366	9.234	9.364	8.897
AP001642.1	6.214	6.124	6.062	6.134	6.329	6.156
REC114	6.928	6.830	6.728	6.670	6.755	6.884
PGF	9.543	9.533	9.442	9.337	9.181	9.470
PGD	10.988	11.103	11.108	11.111	10.963	11.078
PGC	7.493	7.490	7.623	7.564	7.514	7.606
PDE1C	8.382	8.385	8.162	8.004	8.124	8.244

PDE1B	7.917	8.060	7.978	8.193	8.127	7.958
PDE1A	5.188	5.119	5.327	5.347	5.528	5.217
DYM	8.487	8.635	8.767	8.305	8.333	8.384
PGR	6.856	6.915	6.823	6.934	6.917	6.992
PGP	10.942	10.897	11.076	10.744	10.811	10.802
IVNS1ABP	10.632	10.654	10.617	10.570	10.227	10.478
NDUFC1	11.486	11.335	11.493	11.116	11.092	11.171
NDUFC2	12.582	12.712	12.796	12.604	12.609	12.639
SLC45A1	7.103	7.349	7.375	7.188	7.165	7.434
BMI1	8.339	8.454	8.460	8.247	8.286	8.367
MRS2	8.639	8.680	8.529	8.185	8.520	8.571
PTGR2	4.682	4.730	5.015	4.415	4.380	4.483
PDE12	10.084	9.876	9.943	9.854	9.838	9.879
DMXL2	8.141	8.057	7.933	7.395	7.485	7.869
LYPLAL1-AS1	7.499	7.378	7.240	7.413	7.623	7.469
DMXL1	8.282	8.292	8.001	7.726	7.629	7.814
MIR122HG	7.823	7.866	7.882	8.041	8.016	8.143
LINC00958	7.615	7.597	7.482	7.514	7.454	7.566
CEP112	5.606	5.506	5.518	5.544	5.715	5.731
MIA	7.583	7.805	7.792	8.067	8.014	8.019
LINC00957	10.678	10.732	10.880	10.534	10.650	10.751
LINC00955	4.390	4.472	4.379	4.338	4.239	4.531
MIF	13.777	13.817	13.967	13.853	13.826	13.849
SNRK-AS1	6.016	5.970	6.001	6.139	5.876	5.890
MIP	6.893	6.749	6.852	7.061	6.945	7.041
AL353763.2	6.250	6.206	6.048	6.179	6.038	6.201
CBFB	10.854	10.844	10.989	10.681	10.697	10.713
AC087392.3	6.051	6.402	6.077	6.190	6.319	6.388
AC087392.2	6.886	6.676	6.996	6.932	6.955	7.002
CGNL1	8.843	9.075	8.891	8.511	8.336	8.511
AC005776.2	5.241	5.257	5.221	5.449	5.090	5.132
EID2	9.740	9.675	9.852	9.489	9.513	9.589
EID3	4.354	4.354	4.477	4.577	4.426	4.246
EID1	11.733	11.720	11.775	11.549	11.530	11.642
TUBB4A	11.493	11.695	11.824	11.596	11.479	11.392
WNT2B	7.254	7.340	7.191	7.233	7.205	7.169
GHRHR	7.236	7.398	7.266	7.363	7.247	7.389
AC107993.1	5.861	5.819	5.865	5.719	5.875	6.044
SRRM2-AS1	7.677	7.674	7.546	7.583	7.651	7.603
ADAR	11.755	11.666	11.711	11.594	11.545	11.576
AC107983.1	13.528	13.573	13.649	13.437	13.393	13.460
ADAL	7.738	7.730	7.681	7.704	7.953	7.782
SF3A1	10.742	10.890	10.956	10.649	10.746	10.627
SF3A2	9.352	9.281	9.551	9.394	9.461	9.282
SF3A3	9.126	9.198	9.197	8.816	8.940	8.928
AC145124.1	7.832	7.752	7.733	7.678	7.326	7.465
AC022148.1	4.586	4.454	4.404	4.480	4.587	4.514
TCAF2P1	7.650	7.730	7.688	7.716	7.904	7.828
ADA2	7.468	7.539	7.281	7.519	7.623	7.668
TTPAL	9.660	9.649	9.741	9.610	9.542	9.688
TIFA	8.441	8.476	8.727	8.496	8.534	8.574
ZNF25	4.457	4.480	4.426	4.477	4.441	4.509
ZNF24	10.083	10.177	10.339	10.190	10.349	10.073
ZNF26	8.265	8.227	8.191	8.216	8.144	8.028
FAM223B	6.232	6.067	6.109	5.896	5.995	6.318
ZNF20	7.644	7.582	7.620	7.712	7.631	7.816

ZNF23	7.430	7.687	7.645	7.471	7.195	7.620
FAM223A	6.232	6.067	6.109	5.896	5.995	6.318
CSPG4P1Y	6.957	6.925	6.968	7.021	7.153	7.161
AL589987.1	5.516	5.524	5.602	5.664	5.538	5.614
SH3TC2	7.371	7.355	7.364	7.389	7.543	7.518
SH3TC1	6.783	6.926	7.115	6.990	6.942	7.030
BBS10	7.533	7.513	7.433	7.349	7.192	7.416
BBS12	6.796	6.765	7.086	6.998	6.880	7.049
AC044849.1	4.599	4.473	4.445	4.594	4.756	4.708
UBL4A	9.742	9.794	9.902	9.781	9.792	9.737
RNPEP	10.711	10.772	10.814	10.569	10.493	10.566
RNF113A	10.111	10.208	10.366	10.225	10.134	10.207
SNCA	11.870	11.947	12.068	11.838	11.735	11.650
RANBP9	10.372	10.392	10.369	10.171	10.148	10.264
RANBP6	8.446	8.395	8.449	8.445	8.259	8.355
RANBP1	12.005	12.052	12.177	11.879	11.686	11.806
RANBP3	8.880	8.890	8.955	8.769	8.941	8.755
RANBP2	9.691	9.718	9.693	9.508	9.623	9.568
TMEM9	10.465	10.587	10.825	10.378	10.265	10.303
TSPO	11.219	11.261	11.474	11.150	11.142	11.150
ARNT	8.854	8.908	8.785	8.932	9.047	8.903
AC006960.4	6.143	6.192	6.086	6.136	5.997	6.105
AP000662.1	5.970	5.965	5.926	5.907	6.103	6.015
SLAMF9	7.760	7.844	7.843	7.876	7.936	7.910
SLAMF8	6.817	6.793	6.692	6.753	7.085	6.726
SLAMF7	8.064	8.235	8.185	8.124	8.043	8.139
SLAMF6	8.659	8.482	7.991	8.620	8.731	8.638
SLAMF1	7.974	7.749	7.665	7.802	7.804	7.819
AL021578.1	5.550	5.492	5.498	5.634	5.777	5.466
MTATP6P1	14.445	14.476	14.457	14.504	14.599	14.502
AC008669.1	3.952	3.850	3.642	3.744	3.783	3.877
UIMC1	9.236	9.235	9.273	9.319	9.172	9.281
FKRP	9.354	9.493	9.528	9.339	9.299	9.312
DEFB104B	6.804	6.898	6.699	6.994	6.975	6.924
DEFB104A	6.804	6.898	6.699	6.994	6.975	6.924
ATF7-NPFF	8.097	8.128	8.088	8.374	8.163	8.197
AC006994.2	7.353	7.539	7.629	7.447	7.576	7.411
SSNA1	8.994	8.965	9.240	8.894	9.020	8.835
SPATA31D1	8.739	8.700	8.573	8.832	9.065	8.877
AL022097.1	7.708	7.643	7.440	7.799	7.839	7.590
PODXL	8.263	8.247	8.097	8.070	8.051	8.150
CAPRIN2	8.686	8.470	8.448	8.443	8.457	8.704
CAPRIN1	12.416	12.562	12.556	12.406	12.291	12.369
CCNT1	9.529	9.500	9.486	9.507	9.537	9.499
BBOF1	7.845	7.635	7.893	8.097	7.918	7.861
CCNT2	8.308	8.009	8.205	7.906	7.874	8.153
AL109659.3	4.747	4.682	4.445	4.688	4.728	4.685
AL109659.2	6.172	6.392	6.154	6.164	6.417	6.376
CREBZF	7.792	7.736	7.835	7.779	7.808	7.814
DARS-AS1	7.147	7.343	7.119	7.186	7.210	7.300
LINC00543	5.835	6.160	6.108	6.433	5.986	5.934
LINC00544	6.787	6.839	6.835	6.833	6.712	6.872
LINC00545	7.121	7.431	7.356	7.264	7.439	7.380
AL136140.2	7.117	7.340	7.152	7.244	7.297	7.274
MC1R	7.840	7.862	7.939	8.065	8.013	8.013
TMEM272	5.906	5.916	5.371	5.741	5.948	5.645

SEPTIN10P1	8.630	8.705	8.817	8.590	8.649	8.705
AL512408.1	6.634	6.840	6.757	6.700	6.836	6.843
AC241851.2	4.385	4.464	4.479	4.834	4.445	4.587
SVIL-AS1	7.213	7.468	7.566	7.351	7.454	7.489
AC021066.1	6.992	7.031	7.082	6.873	7.153	7.119
RPL39L	8.839	8.807	9.046	8.555	8.740	8.672
FHP1	7.017	7.038	7.175	6.732	7.023	6.995
RUNX2	7.899	7.872	7.842	8.034	8.053	8.019
RUNX3	7.331	7.298	7.623	7.805	7.515	7.319
APOBEC1	7.333	7.394	7.603	7.237	7.330	7.472
RUNX1	9.680	9.638	9.619	9.467	9.484	9.529
APOBEC4	7.643	7.762	7.394	7.552	7.714	7.646
AC092143.1	11.493	11.695	11.838	11.596	11.479	11.392
AC005785.1	7.925	7.954	7.864	7.880	7.900	7.931
AC005785.2	4.186	4.195	4.151	4.180	4.122	4.145
ESRG	4.603	4.684	4.635	4.990	4.710	4.747
OR5G1P	5.847	5.856	5.591	5.900	6.076	6.117
IGKV1D-17	6.685	6.554	6.707	6.553	6.594	6.673
IGKV1D-13	8.513	8.519	8.493	8.474	8.731	8.605
SLC9A5	7.627	7.644	7.676	7.670	7.774	7.644
SLC9A6	9.787	9.944	9.919	9.575	9.580	9.623
SLC9A7	10.090	10.105	10.249	9.849	9.951	9.865
SLC9A1	8.941	8.938	8.868	8.834	8.736	8.861
SLC9A2	7.693	7.791	7.523	7.550	7.777	7.256
SLC9A3	7.989	8.093	8.179	7.801	7.759	7.856
PBXIP1	9.365	9.420	9.567	9.181	9.301	9.343
SLC9A8	9.055	9.136	9.124	8.878	8.892	8.972
AC270276.1	3.848	3.917	3.935	4.055	4.059	3.935
AC026458.1	7.981	7.880	7.994	8.183	7.956	7.961
ARHGEF37	8.019	7.922	8.201	8.002	7.871	7.936
PLCB2	8.367	8.348	8.436	8.494	8.475	8.633
PLCB3	8.221	8.364	8.365	8.362	8.256	8.369
PLCB4	7.811	7.844	7.911	7.380	7.345	7.593
ARHGEF38	7.683	7.961	7.592	7.843	7.722	7.616
ARHGEF39	7.935	7.881	7.965	7.718	7.858	7.813
C17orf113	8.263	8.323	8.267	8.462	8.389	8.274
ESR2	7.493	7.501	7.620	7.659	7.625	7.637
AC004938.2	6.750	6.682	6.616	6.749	6.926	6.855
ESR1	8.017	8.058	8.125	8.104	8.226	8.259
AC012531.2	7.501	7.782	7.693	7.655	7.596	7.837
AC012531.3	7.501	7.782	7.693	7.655	7.596	7.837
AC012531.1	7.530	7.487	7.314	7.532	7.525	7.595
MSMO1	12.030	12.128	12.199	12.182	12.136	12.334
EARS2	9.302	9.293	9.275	9.208	9.292	9.160
MOB3B	10.100	9.826	9.831	9.887	9.848	10.022
MOB3C	8.321	8.239	8.329	8.353	8.364	8.246
MOB3A	9.256	9.348	9.472	9.150	9.393	9.263
TBX6	8.165	8.286	8.229	8.498	8.343	8.278
TBX4	6.729	6.739	6.794	6.931	6.831	6.764
TBX5	7.311	7.523	7.431	7.616	7.455	7.407
A2M	6.706	6.598	6.404	6.910	6.799	6.531
KLRF1	4.559	4.422	4.545	4.641	4.460	4.701
C18orf21	8.325	8.280	8.269	8.264	8.294	8.291
NAV1	8.187	8.104	8.061	8.203	8.277	8.322
TMEM38B	9.444	9.547	9.561	9.464	9.424	9.574
C18orf25	9.175	9.128	9.245	8.889	9.002	9.106

CLBA1	8.125	8.059	8.202	8.407	8.117	8.086
AC120024.1	7.176	7.291	7.127	7.330	7.328	7.322
LIG3	8.656	8.484	8.677	8.479	8.437	8.556
PRRT1	7.823	7.886	7.823	8.160	7.880	7.696
PRRT3	7.774	7.934	8.108	8.070	7.889	8.004
PRRT2	6.462	6.623	6.740	6.787	6.799	6.908
BASP1-AS1	5.437	5.439	5.140	5.224	5.172	5.430
TBXT	8.692	8.971	8.758	8.777	8.920	8.872
AL592435.1	7.061	6.893	7.069	6.862	6.931	7.186
REG1CP	7.054	7.183	7.044	7.279	7.336	7.318
CFAP46	7.123	7.228	7.041	7.339	7.252	7.443
CFAP47	6.836	6.979	6.666	6.907	6.808	6.770
CFAP44	5.724	5.797	5.575	5.698	5.845	5.697
CFAP45	7.569	7.687	7.468	7.954	7.808	7.604
CFAP43	5.883	5.865	5.936	5.922	5.881	5.959
FAU	12.943	13.018	13.182	13.107	12.912	13.120
FAH	8.507	8.525	8.744	8.426	8.279	8.370
HNRNPA2B1	12.639	12.647	12.714	12.624	12.503	12.662
RSRP1	7.497	7.418	7.643	7.518	7.716	7.651
AMY2A	5.089	5.094	5.291	5.046	4.927	5.253
AMY2B	7.008	6.922	6.762	6.787	6.895	7.018
DNM1P51	7.802	7.940	7.850	7.942	7.944	8.168
TUBAP2	13.801	13.821	13.852	13.686	13.719	13.708
OAF	9.602	9.747	9.825	9.665	9.618	9.626
CCS	9.052	9.128	9.223	9.054	8.890	8.953
OAT	10.601	10.729	10.785	10.582	10.470	10.535
CCK	6.943	6.731	6.788	6.888	7.042	7.106
AC003102.1	7.503	7.348	7.379	7.188	7.382	7.430
BX927194.3	8.614	8.670	8.743	8.558	8.568	8.605
COG8	9.800	9.744	9.879	9.579	9.530	9.540
COG3	8.324	8.207	8.363	8.399	8.231	8.330
COG2	9.977	9.998	10.035	10.004	10.065	10.066
COG1	9.548	9.511	9.573	9.197	9.323	9.259
CHTOP	10.373	10.486	10.630	10.419	10.499	10.362
COG7	9.011	9.087	9.300	8.914	9.071	8.873
COG6	8.225	8.385	8.366	8.089	8.013	8.203
COG5	7.979	8.125	7.996	7.903	8.044	8.054
COG4	9.618	9.789	9.941	9.564	9.491	9.534
ADAMTSL4-AS1	11.992	12.041	12.124	12.015	12.007	12.071
ADAMTSL4-AS2	6.970	7.204	7.124	6.759	7.119	6.903
CSPG4	6.245	6.219	6.327	6.502	6.345	6.494
CFTR	8.642	8.750	8.862	8.475	8.474	8.561
AC097468.3	7.443	7.243	7.310	7.321	7.360	7.285
AC097468.1	6.903	6.836	6.765	7.030	7.000	7.156
GOT2P1	7.569	7.545	7.133	7.553	7.421	7.581
GUCY1A1	9.824	9.901	9.886	9.820	9.978	9.838
PCBP4	8.637	8.963	9.080	8.816	8.943	8.808
GUCY1A2	6.716	6.798	6.631	7.106	6.835	6.744
PCBP2	12.719	12.801	12.901	12.554	12.572	12.673
PCBP3	7.593	7.581	7.374	7.681	7.837	7.548
PCBP1	12.297	12.387	12.545	12.222	12.156	12.244
LZTS2	8.893	8.886	9.001	8.904	9.083	8.938
LZTS3	8.102	8.147	8.088	7.909	8.061	8.154
LZTS1	8.166	8.012	7.967	8.238	8.322	8.218
C1QL1	8.205	8.148	8.211	8.043	8.201	8.164
AC129502.1	6.372	6.572	6.644	6.460	6.740	6.844

NHSL1	7.975	8.070	8.022	8.089	8.185	7.986
NHSL2	7.124	7.003	7.096	7.160	7.162	7.219
PAK1IP1	8.275	8.243	8.289	8.251	8.453	8.133
HOPX	6.895	7.084	7.228	7.433	7.264	7.216
POU6F2-AS2	3.956	3.901	4.204	3.842	4.026	3.964
SCN7A	4.492	4.705	4.774	5.101	4.730	4.598
RFWD3	8.878	8.894	8.846	8.430	8.769	8.569
AD000671.1	10.499	10.454	10.684	10.436	10.575	10.366
RPS20P22	6.535	6.679	6.521	6.420	6.490	6.723
RPS20P21	5.045	5.143	5.181	5.108	5.129	5.255
AD000671.2	8.912	8.829	9.096	8.677	8.707	8.584
IGHV3-54	6.356	6.471	6.499	6.503	6.500	6.426
CIRBP-AS1	8.140	8.205	8.029	8.098	8.032	8.253
TRMT6	8.940	8.981	8.782	8.533	8.632	8.675
ZEB1-AS1	7.435	7.324	7.339	6.957	6.954	6.953
TRMT5	11.499	11.540	11.608	11.335	11.371	11.429
TRMT1	8.444	8.411	8.578	8.413	8.308	8.610
AC226740.1	7.972	7.912	8.183	8.328	8.259	8.126
MNAT1	8.590	8.705	8.705	8.685	8.755	8.783
SEMA3A	7.967	7.836	7.869	7.721	7.788	8.116
SEMA3C	10.072	10.106	10.055	9.865	9.731	10.142
SEMA3B	7.182	7.413	7.500	7.322	7.355	7.545
SEMA3E	6.885	7.140	7.002	6.515	6.582	6.579
SEMA3D	4.777	4.681	4.883	4.709	4.870	4.784
SEMA3G	6.784	6.777	6.554	7.142	6.922	6.851
SEMA3F	8.817	8.769	8.983	8.894	8.824	8.961
AC125793.1	4.917	4.931	5.148	5.224	5.190	5.010
GLP2R	6.088	6.257	6.336	6.549	6.375	6.308
DESI2	9.978	9.956	10.189	9.880	9.950	9.789
DESI1	9.972	10.060	10.182	10.151	10.096	9.955
ZNF251	7.074	7.162	7.254	7.014	6.931	6.996
ZNF250	7.367	7.270	7.445	7.172	7.531	7.520
ZNF253	8.007	8.083	8.018	8.145	8.204	8.170
ZNF257	4.052	4.169	4.364	4.461	4.352	4.520
DERA	10.335	10.356	10.536	10.253	10.312	10.244
DDX11-AS1	4.298	4.367	4.569	4.208	4.382	4.305
TMEM183A	10.258	10.380	10.484	10.548	10.520	10.482
TMEM183B	10.258	10.380	10.484	10.548	10.520	10.482
SNORD14E	13.524	13.671	13.656	13.728	13.636	13.710
SNORD14D	13.524	13.671	13.656	13.728	13.636	13.710
SNORD14C	13.795	13.904	13.879	13.960	13.927	14.010
SYNPR-AS1	6.636	6.618	6.540	6.685	6.679	6.537
AC020978.7	9.370	9.228	9.292	9.283	9.234	9.347
AC020978.5	9.894	9.463	9.575	9.472	9.415	9.634
AR	7.655	7.830	7.699	7.657	7.946	8.057
RPL34P20	6.526	6.452	6.412	6.189	6.600	6.551
C20orf85	5.414	5.748	5.781	5.863	5.835	5.727
C1QTNF9	5.869	5.866	5.753	5.809	5.889	5.854
C1QTNF2	6.194	6.127	6.015	6.350	6.214	6.222
C1QTNF3	6.900	6.982	6.852	7.199	7.051	7.042
C1QTNF1	8.069	8.324	8.265	8.195	8.286	8.323
C1QTNF6	7.672	7.611	7.725	7.643	7.592	7.687
C1QTNF7	6.132	6.019	5.845	6.060	5.674	5.955
C1QTNF4	4.674	4.841	5.109	4.743	4.894	4.784
C1QTNF5	8.719	8.775	8.662	8.644	8.661	8.727
CREB3L2	9.410	9.526	9.552	9.492	9.807	9.371

CREB3L3	4.957	4.950	5.047	5.215	5.203	5.212
CREB3L1	8.456	8.483	8.033	8.528	8.689	8.561
CREB3L4	9.080	9.072	9.156	8.821	8.876	8.810
AC090236.1	7.043	7.239	7.263	7.358	7.364	7.323
C17orf77	3.979	4.047	3.680	3.989	4.104	3.898
C17orf75	5.977	5.968	6.041	5.526	5.862	5.877
C17orf78	5.767	5.890	5.573	5.657	5.818	5.720
CHKB-DT	8.448	8.537	8.543	8.198	8.529	8.384
RARS2	8.840	8.880	8.887	8.719	8.622	8.751
RARS1	10.520	10.469	10.594	10.323	10.373	10.333
HMGN1P37	11.925	11.994	12.146	11.893	11.885	11.984
HMGN1P38	11.925	11.994	12.146	11.893	11.885	11.984
AL360219.2	5.193	4.922	5.033	4.677	4.695	4.569
NDUFAB1	12.255	12.247	12.444	12.410	12.251	12.301
AC105053.1	3.699	3.689	3.808	3.834	3.910	3.851
AL359715.1	4.328	4.531	4.417	4.294	4.615	4.671
HEBP1	7.940	8.089	8.241	7.583	7.732	7.849
AL359715.3	5.633	5.927	5.664	5.509	5.503	5.601
TLCD4-RWDD3	8.001	7.929	8.104	7.814	7.847	7.915
DUS2	9.140	9.244	9.149	9.118	9.135	9.154
ASH1L-AS1	7.942	7.633	7.706	7.482	7.718	7.520
MIR1233-1	7.615	7.762	7.518	7.600	7.378	8.064
AC022730.4	5.675	5.681	5.660	5.683	5.561	5.578
IWS1	9.799	9.772	9.856	9.528	9.543	9.555
L2HGDH	8.613	8.722	8.704	8.484	8.533	8.650
LECT2	5.169	5.102	5.027	5.053	5.243	5.498
ATF7IP2	8.294	8.324	8.345	8.241	8.347	8.373
PRR12	8.618	8.268	8.552	8.557	8.603	8.422
PRR13	11.530	11.550	11.675	11.374	11.322	11.443
PRR11	10.032	10.017	10.040	9.184	9.504	9.468
PRR16	6.048	6.247	6.309	6.473	6.231	6.250
PRR14	10.051	10.113	10.237	10.247	10.138	10.204
PRR15	10.554	10.638	10.809	10.612	10.577	10.567
PRR18	6.112	6.076	6.071	6.037	5.993	6.022
PRR19	7.681	7.609	7.914	8.099	8.010	7.935
LINC01924	6.954	6.975	6.694	6.983	6.896	7.007
LINC01927	7.569	7.639	7.627	7.774	7.763	7.646
LINC01926	7.339	7.339	7.167	7.337	7.445	7.495
LINC01921	5.541	5.688	5.401	5.609	5.720	5.517
LINC01920	6.793	6.828	6.829	7.001	6.771	6.940
LINC01922	6.293	6.595	6.330	6.340	6.545	6.724
AC104134.1	7.450	7.235	7.277	7.091	7.379	7.377
IFNL1	6.846	6.583	6.490	7.227	6.775	6.855
IFNL2	7.397	7.492	7.155	7.971	7.808	7.706
IFNL3	5.007	5.082	5.104	5.294	5.390	5.461
LINC00032	7.054	7.114	7.140	7.027	7.137	7.106
TIMM13	9.826	9.829	9.970	9.557	9.370	9.578
TIMM10	10.741	10.740	10.842	10.679	10.713	10.671
OPCML	6.745	6.864	6.791	6.926	6.892	6.873
BMS1P1	8.528	8.250	7.921	8.147	8.189	8.394
LRR1	9.486	9.472	9.549	9.229	9.387	9.373
WDPCP	8.536	8.435	8.540	8.319	8.388	8.490
AL136038.5	4.942	4.874	4.646	4.858	4.619	4.604
AC093525.2	13.118	13.168	13.219	13.060	12.975	13.021
AC093525.6	8.466	8.495	8.514	8.432	8.360	8.428
AC093525.7	8.804	8.965	8.790	8.808	8.814	8.709

GHET1	6.974	6.959	6.786	7.153	7.107	6.763
FAM186B	7.679	7.480	7.365	7.629	7.830	7.620
AC134878.2	8.666	8.777	8.881	8.661	8.673	8.861
ADGRB1	6.996	7.186	7.277	7.337	7.245	7.339
ADGRB2	5.553	5.660	5.771	5.696	5.735	5.944
ADGRB3	4.306	4.572	4.179	4.809	4.756	4.634
TAF7	10.791	10.853	10.964	10.975	10.949	10.973
OSCP1	7.506	7.364	7.485	7.373	7.356	7.367
TAF4	9.960	9.960	10.052	9.749	9.795	9.700
BPHL	8.502	8.618	8.602	8.422	8.579	8.417
LINC01010	7.134	7.317	7.227	7.259	7.297	7.163
RNF25	8.601	8.531	8.661	8.521	8.444	8.491
RNF24	7.833	8.071	8.028	7.941	8.051	8.033
RNF26	9.243	9.271	9.446	9.096	9.155	9.118
NT5C1B-RDH14	9.143	9.219	9.276	8.745	8.701	8.811
SRGAP3-AS2	5.863	6.086	5.855	6.137	6.134	6.022
LINC01138	7.865	7.879	8.165	7.721	8.167	7.778
LSP1	7.235	7.363	7.520	7.435	7.315	7.463
ARHGEF26-AS1	5.315	5.245	5.280	5.525	5.461	5.242
A2ML1	7.479	7.371	7.468	7.585	7.573	7.665
TOX	4.622	4.562	4.143	4.244	4.369	4.421
TOM1	8.459	8.582	8.702	8.331	8.339	8.390
SPARCL1	5.740	5.807	5.753	5.838	5.974	5.788
MYOT	4.544	4.563	4.506	4.746	4.552	4.813
AC004687.1	7.329	7.469	7.529	7.826	7.364	7.621
MYOC	8.157	8.387	8.405	8.461	8.508	8.500
TAF8	7.549	7.692	7.706	7.379	7.620	7.574
CYS1	8.485	8.403	8.633	7.699	7.686	7.734
MYOF	12.458	12.466	12.512	12.456	12.327	12.479
RNU6-1099P	9.897	9.985	10.145	9.770	9.942	9.849
LARS2	7.734	7.821	7.913	7.837	7.756	7.719
TMEM41A	10.093	10.111	10.224	10.098	10.055	10.114
CHI3L1	4.878	5.066	5.210	5.308	5.189	5.005
TMEM41B	10.965	10.937	11.036	10.901	10.854	10.890
AC048380.2	4.409	4.347	4.446	4.390	4.491	4.406
CNOT9	9.357	9.364	9.484	8.811	9.130	9.035
CNOT8	9.658	9.742	9.832	9.676	9.738	9.755
CNOT3	8.837	8.831	9.165	9.068	9.208	8.893
AARS1	11.681	11.693	11.819	11.551	11.479	11.488
CNOT1	11.588	11.529	11.619	11.371	11.300	11.402
CNOT7	11.963	11.847	11.977	11.981	11.933	11.894
CNOT6	10.003	10.013	9.998	9.687	9.525	9.803
CNOT4	8.220	8.159	8.259	8.125	7.914	8.203
DDX11L9	8.024	8.146	8.047	7.916	7.969	7.788
DDX11L8	8.024	8.146	8.047	7.916	7.969	7.788
DDX11L1	8.024	8.146	8.047	7.916	7.969	7.788
DDX11L2	8.024	8.146	8.047	7.916	7.969	7.788
ERVH48-1	6.886	6.950	6.896	6.756	6.936	6.970
YARS2	9.137	9.088	9.037	9.060	9.067	8.973
GLRX3P2	9.469	9.554	9.477	9.554	9.549	9.547
FAM92A1P1	6.682	6.827	6.722	6.709	6.714	6.912
MYO6	10.019	9.938	9.901	9.706	9.946	9.602
EAPP	9.325	9.353	9.409	9.401	9.344	9.305
AC005833.1	6.215	6.374	6.312	6.266	6.211	6.302
GNPDA2	8.191	8.201	8.326	7.915	7.934	8.078
GNPDA1	10.382	10.528	10.579	10.336	10.317	10.421

INSRR	7.197	7.167	7.324	7.500	7.420	7.601
RASAL1	9.078	9.272	9.299	9.044	9.160	9.190
RASAL3	8.318	8.146	8.401	8.847	8.464	8.377
RASAL2	9.791	9.835	9.817	9.626	9.761	9.655
MTATP8P1	14.445	14.476	14.457	14.504	14.599	14.502
SLC12A5	6.850	6.758	6.707	7.274	6.906	6.887
SLC12A4	8.293	8.352	8.479	8.251	8.320	8.301
SLC12A7	11.116	11.119	11.181	11.144	11.137	11.076
SLC12A6	9.214	9.057	9.083	8.999	9.200	8.979
SLC12A1	6.703	6.446	6.499	6.747	6.640	6.681
SLC12A3	7.454	7.693	7.858	7.843	7.753	7.880
SLC12A2	11.582	11.593	11.691	11.149	11.148	11.299
SLC12A9	8.716	8.731	8.829	8.788	8.743	8.766
SLC12A8	7.380	7.197	7.629	7.476	7.572	7.420
AC107223.1	3.974	4.021	3.961	4.098	4.365	4.103
MRVI1	9.024	8.900	8.923	9.006	9.076	9.167
AC092447.4	7.085	7.168	6.842	7.110	7.046	6.996
AC092447.5	4.681	4.674	4.537	4.881	4.861	4.840
AC092447.8	4.276	4.380	4.002	4.175	4.293	4.192
Z93930.2	5.038	5.004	4.916	5.019	4.820	5.052
LINC02591	8.038	7.858	8.111	8.613	8.288	8.066
LINC02593	8.115	8.129	8.208	7.987	8.235	8.232
LINC02595	5.054	4.847	4.964	4.831	4.854	5.100
MIR124-2HG	6.397	6.282	6.010	6.491	6.552	6.517
AC105275.2	5.942	5.936	5.807	5.996	5.854	6.194
LEPROTL1	10.729	10.809	10.855	10.701	10.639	10.656
WHRN	8.007	8.006	8.110	8.187	8.039	8.012
CCDC127	9.108	9.155	9.386	9.135	9.115	9.113
CCDC126	7.876	7.845	8.019	7.912	7.928	7.739
CCDC125	6.227	6.235	6.107	6.287	6.293	6.460
CCDC124	7.995	7.978	8.226	7.960	7.997	7.752
SLC11A2	9.210	9.237	9.273	9.157	9.234	9.143
CCDC122	6.239	6.343	6.341	6.214	6.352	6.320
CCDC121	4.816	4.576	4.816	4.866	4.729	4.587
ASMT	6.309	6.532	6.236	6.651	6.576	6.388
ZIK1	6.568	6.486	6.414	6.454	6.430	6.605
STK26	10.068	10.164	10.126	9.992	10.094	10.130
STK25	9.213	9.399	9.545	9.230	9.078	9.117
STK24	11.619	11.754	11.820	11.518	11.623	11.459
L1TD1	5.422	5.150	5.518	5.224	5.227	5.351
AC007599.2	6.078	6.020	6.162	5.928	5.935	5.897
TUBB2A	11.493	11.695	11.838	11.596	11.479	11.392
SLC25A25-AS1	8.152	8.228	7.853	7.762	7.894	7.932
SDCBPP2	6.242	6.223	6.341	6.015	5.804	6.088
ARNILA	7.151	7.251	7.349	7.291	7.264	7.208
LINC01020	5.706	5.573	5.625	5.659	5.588	5.696
AC008080.1	7.542	7.705	7.692	7.749	7.900	7.740
LINC01023	6.856	6.866	6.963	7.009	6.841	6.833
DTYMK	10.252	10.245	10.533	9.806	10.044	9.991
LINC01029	6.970	7.147	7.021	7.427	7.284	7.400
H2BS1	10.261	10.361	10.461	10.525	10.201	10.328
PIWIL4	8.153	8.260	8.274	8.242	8.051	8.238
PIWIL2	7.103	7.221	7.213	7.178	7.334	7.243
PIWIL1	4.118	4.149	4.308	4.598	4.330	4.311
H2AC10P	8.450	8.621	8.834	8.314	8.339	8.428
ITGA9-AS1	5.611	5.668	5.539	5.869	5.989	5.787

SPDYA	5.607	5.478	5.610	5.567	5.689	5.600
CPVL	11.346	11.418	11.655	11.437	11.317	11.372
DNMBP	9.844	9.821	9.771	9.752	9.787	9.732
MRVI1-AS1	8.206	8.177	8.082	8.104	8.278	8.488
AP005901.6	4.385	4.464	4.479	4.834	4.445	4.587
AP005901.1	5.821	6.035	5.659	6.080	5.739	5.988
GKN1	6.443	6.591	6.358	6.476	6.672	6.419
GKN2	7.557	7.558	7.350	7.602	7.710	7.710
TCF24	3.863	3.788	3.708	4.015	3.794	4.084
PDCD7	8.615	8.607	8.609	8.237	8.287	8.332
NELFCD	11.620	11.625	11.715	11.624	11.551	11.551
TCF20	9.712	9.745	9.846	9.710	9.832	9.518
LENG1	8.039	8.149	8.280	8.207	7.936	7.954
IFT172	9.255	9.233	9.228	9.056	9.024	9.003
NPFF	8.097	8.128	8.088	8.374	8.163	8.197
LENG9	7.322	7.630	7.761	7.667	7.649	7.333
LENG8	7.908	7.758	7.900	7.486	7.534	7.700
MICOS13	9.408	9.389	9.650	9.505	9.483	9.387
MICOS10	10.926	10.919	11.145	10.898	10.876	10.951
AP000843.1	6.859	6.628	6.600	6.950	6.770	6.768
AC139887.2	6.923	6.691	6.890	6.584	7.046	7.061
AC139887.3	6.918	6.817	6.749	6.766	6.899	6.747
CXorf38	8.919	8.863	8.871	8.551	8.783	8.390
CU024913.1	4.623	4.658	4.664	4.620	4.660	4.680
PRR9	5.601	5.593	5.576	5.466	5.316	5.598
GABARAPL1	9.682	9.666	9.793	9.403	9.540	9.264
MIR372	7.282	7.149	7.230	7.410	7.454	7.037
GABARAPL2	11.825	11.928	12.050	12.096	11.982	12.137
PRR3	8.382	8.412	8.520	8.302	8.215	8.424
PRR4	7.199	7.315	7.449	7.834	7.457	7.479
PRR5	8.539	8.777	8.763	8.351	8.572	8.537
PRR7	8.874	9.049	9.388	9.126	9.078	8.986
DVL2	8.882	8.940	9.155	8.771	8.834	8.806
DVL3	11.063	11.057	11.012	10.810	10.787	10.866
KDM4A	9.334	9.428	9.462	9.552	9.513	9.252
DVL1	9.329	9.419	9.457	9.210	9.056	9.230
AC067930.6	8.644	8.760	9.280	9.043	9.825	8.772
AC097639.1	7.501	7.519	7.615	7.574	7.657	7.406
CRACR2B	8.189	8.148	8.371	8.244	8.138	8.324
CRACR2A	8.178	7.907	7.902	7.798	7.561	7.932
HDLBP	11.173	11.193	11.304	11.128	11.049	11.184
PSME4	9.599	9.638	9.582	9.505	9.353	9.485
PSME2	11.833	11.858	11.931	11.903	11.876	11.874
PSME3	11.876	11.883	11.957	11.732	11.744	11.729
PSME1	12.157	12.178	12.364	12.260	12.278	12.224
MTSS2	7.719	7.839	7.736	8.119	8.390	7.947
MTSS1	8.549	8.721	8.534	8.300	8.167	8.259
MTFR1L	8.092	8.322	8.354	7.971	8.168	8.166
TOPBP1	10.388	10.338	10.327	10.174	10.114	10.230
FRY-AS1	3.864	3.630	3.777	3.744	3.703	3.858
AL451042.2	7.034	6.961	6.960	7.031	7.057	6.929
ZNF511-PRAP1	6.882	6.877	6.928	6.889	7.013	6.971
AL512635.1	4.935	5.005	4.972	5.101	5.342	5.191
CLEC4GP1	7.168	7.398	7.087	7.216	7.301	7.237
SMBD1P	6.909	6.854	6.683	6.671	6.829	6.818
GRHPR	9.500	9.540	9.624	9.380	9.398	9.398

FURIN	9.406	9.440	9.594	9.664	9.624	9.577
AC009948.1	10.917	11.003	11.124	10.864	10.682	10.882
CDK5R1	6.984	7.215	6.943	7.286	6.997	6.941
TAGLN2	12.308	12.456	12.492	12.415	12.328	12.193
GJD4	6.804	6.620	6.842	7.023	6.818	6.742
GJD3	8.300	8.373	8.536	8.353	8.460	8.389
GJD2	7.653	7.809	7.889	7.905	7.774	8.195
AL133492.1	6.438	6.193	6.266	6.491	6.441	6.476
ZMYND8	10.619	10.789	10.803	10.283	10.471	9.954
NARS2	9.366	9.206	9.320	9.370	9.077	9.196
NARS1	12.341	12.382	12.402	12.179	12.084	12.195
CDK5R2	6.545	6.557	6.808	6.643	6.622	6.482
NAIP	7.508	7.289	7.527	7.633	7.611	7.536
GMPR	5.831	5.963	5.968	6.246	6.150	5.943
GMPS	11.314	11.458	11.665	11.398	11.299	11.297
AL161772.1	8.324	8.429	8.413	8.114	8.164	8.312
NOP10	10.890	11.001	11.128	11.125	11.063	11.074
NEGR1-IT1	6.909	6.813	6.758	6.849	7.191	6.896
EXOC3L2	9.755	9.831	9.661	9.766	9.892	9.854
CZIB	8.843	9.038	8.969	8.855	8.876	8.850
NOP16	10.475	10.610	10.713	10.431	10.469	10.317
OR2A9P	6.732	6.737	6.688	6.746	6.683	6.540
AC091057.3	6.001	6.008	5.892	6.347	6.123	6.186
AC091057.1	5.831	5.929	5.609	5.610	5.666	5.707
AC091057.4	8.279	8.162	8.137	8.002	8.055	8.233
SLC25A51P3	7.246	7.409	7.518	7.686	7.515	7.591
AP001630.1	6.489	6.726	6.826	6.974	6.663	7.018
TMEM171	7.110	7.270	7.232	7.051	7.285	7.169
LARGE1	8.704	8.648	8.569	8.753	8.654	8.777
LARGE2	8.352	8.455	8.502	8.405	8.554	8.546
APOLD1	10.079	10.080	10.129	10.167	10.167	10.147
NYNRIN	8.744	8.774	8.780	8.815	8.713	8.742
C11orf68	9.707	9.840	9.937	9.528	9.625	9.576
C11orf65	3.847	3.762	3.952	4.142	3.746	4.036
CCR1	8.071	8.120	8.071	8.060	8.373	7.856
CCR2	5.915	6.086	5.730	6.079	5.917	6.081
CCR3	6.342	6.226	6.350	6.299	6.517	6.401
CCR4	7.136	7.296	7.191	7.313	7.156	7.377
CCR5	8.876	9.155	8.752	9.014	9.066	9.223
CCR6	5.431	5.263	5.264	5.594	5.534	5.502
CCR7	5.976	6.139	5.956	6.161	6.150	6.271
CCR8	7.412	7.593	7.357	7.293	7.429	7.327
CCR9	5.447	5.729	5.779	5.457	5.647	5.743
UST-AS1	3.676	3.723	3.730	3.733	3.788	3.695
AC123767.1	4.056	4.184	4.266	4.105	3.954	4.148
AL117350.1	8.416	8.555	8.672	8.343	8.565	8.475
AC008937.3	3.828	3.753	3.730	3.682	3.804	3.878
HSPD1P21	6.517	6.558	6.465	6.424	6.506	6.713
FADD	10.295	10.407	10.604	10.355	10.418	10.380
AL136981.4	7.512	7.495	7.668	7.549	7.815	7.555
LYPLA2P1	10.457	10.582	10.686	10.281	10.365	10.305
AL136981.3	5.930	5.886	6.013	6.169	5.864	6.116
CDS1	9.887	9.946	9.898	9.879	9.828	9.882
CDS2	9.056	9.073	9.143	8.760	8.895	8.946
CCDC169-SOHLI	3.880	3.809	3.867	4.059	3.948	3.966
CDKN2C	10.355	10.356	10.243	9.998	10.036	10.090

AL627309.1	7.159	6.862	6.635	6.551	6.694	6.987
AL627309.2	8.340	8.109	7.986	8.227	8.359	8.262
AL627309.5	7.688	7.417	7.266	7.457	7.674	7.621
AL627309.6	10.722	10.564	10.478	10.430	10.307	10.610
H3C10	7.647	7.475	7.585	7.724	7.855	8.008
H3C11	5.653	5.450	5.655	5.939	5.720	5.254
H3C12	7.360	7.394	7.326	7.088	7.501	7.690
S100G	5.749	5.981	5.855	6.079	6.038	6.012
NEXMIF	5.771	5.418	5.752	5.918	5.890	5.648
S100B	6.610	6.600	6.484	6.994	6.825	6.834
PSMG3-AS1	7.670	7.797	7.670	7.608	7.854	7.715
AC079741.1	5.115	5.148	4.510	5.168	5.153	5.030
AL157895.2	4.810	4.882	4.844	5.021	4.878	4.776
CYP4V2	9.772	9.914	9.837	9.660	9.630	9.779
CLDN20	4.840	4.796	4.657	4.787	4.937	5.041
PSMC3IP	9.214	9.318	9.200	9.132	9.247	9.186
THAP7-AS1	7.843	7.914	7.845	7.810	7.985	8.218
THG1L	7.693	7.851	7.829	7.650	7.628	7.656
ZNF865	8.731	8.796	8.898	8.731	8.738	8.926
ZNF862	8.849	9.031	8.927	8.901	8.956	8.862
AL162171.1	5.600	5.563	5.579	5.770	5.729	5.657
AL162171.3	6.057	6.220	5.996	6.039	6.218	6.210
ZNF512B	9.679	9.673	9.766	9.237	9.439	9.552
FAM160A1	8.419	8.512	8.236	8.356	8.337	8.605
GSS	11.537	11.527	11.692	11.308	11.247	11.343
GSR	10.809	10.932	10.991	10.868	10.774	10.644
DHCR7	11.739	11.867	11.948	11.757	11.731	11.803
CASP10	8.311	8.451	8.486	8.500	8.457	8.500
CASP12	4.863	4.884	4.891	4.880	4.930	5.021
CASP14	7.188	7.132	7.126	7.142	7.249	7.151
AL031847.2	13.111	13.257	13.313	13.229	13.072	13.208
XAGE2	7.907	7.930	7.986	8.252	8.055	8.001
THPO	6.622	6.759	6.548	6.673	6.839	6.734
GSN	10.928	11.073	11.263	10.728	10.673	10.769
ZFP1	7.368	7.300	7.497	7.310	7.504	7.120
ZFP2	4.625	4.859	4.819	5.129	4.938	5.287
ZFP3	5.653	5.488	5.188	5.803	5.597	5.863
CACNG2	7.419	7.433	7.377	7.543	7.552	7.667
SERPINB8	9.202	8.978	8.749	9.155	9.086	9.171
ATP2B1	10.862	10.839	10.963	10.654	10.809	10.445
ATP2B3	7.012	7.064	7.208	7.341	7.314	7.335
ATP2B2	7.951	8.054	8.122	8.111	8.130	8.037
ATP2B4	8.555	8.381	8.449	8.432	8.443	8.524
CACNG4	11.200	11.213	11.236	11.256	11.242	11.277
XRN1	9.112	9.220	9.175	8.840	8.808	9.124
CYP27C1	4.045	4.271	4.215	4.385	4.194	4.517
AC013268.1	7.564	7.798	7.581	7.620	7.841	7.801
AC013268.5	7.471	7.500	7.686	7.180	7.427	7.531
CORO7-PAM16	8.612	8.619	8.714	8.653	8.608	8.552
AC092162.2	5.361	5.525	5.335	5.286	5.278	5.319
AC092162.3	5.778	5.756	5.709	5.946	6.084	5.889
CACNG5	7.671	7.744	7.826	7.923	7.765	7.939
PABPC4	11.097	11.230	11.402	11.123	11.007	11.082
PABPC5	3.871	3.797	3.773	3.582	3.772	3.814
DQX1	7.203	7.412	7.428	7.448	7.637	7.461
ZBTB20-AS1	6.444	6.674	6.598	6.421	6.633	6.835

LINC02753	7.328	7.492	7.483	7.460	7.530	7.545
LINC02757	5.814	6.021	5.876	5.829	5.786	6.129
LINC02754	6.320	6.242	5.864	5.752	5.944	5.967
AC115223.1	13.671	13.713	13.720	13.661	13.586	13.672
PPME1	10.417	10.462	10.643	10.457	10.422	10.499
AL355488.1	4.749	4.939	4.699	4.847	4.730	4.829
CTNNBL1	9.901	10.034	10.129	9.940	9.872	9.865
PTPRN2	7.319	7.420	7.300	7.439	7.498	7.562
AL031118.1	5.138	5.172	5.001	5.175	5.211	5.227
HSF2BP	7.187	7.272	7.198	7.263	7.039	7.210
CATSPER2P1	7.725	7.587	7.677	7.711	7.740	7.694
AL109741.1	4.903	4.983	4.865	4.990	4.761	4.838
AC027020.1	7.119	6.978	6.951	7.353	7.388	7.501
AASDHPPT	9.894	10.047	10.064	9.859	9.773	9.789
POF1B	8.790	8.813	8.786	8.910	8.794	8.774
AC130689.1	10.880	10.910	11.007	11.033	10.980	10.967
IDS	11.178	11.306	11.302	11.091	11.030	11.169
NFATC3	9.370	9.228	9.292	9.283	9.234	9.347
GLIPR1L1	5.920	5.750	5.822	5.772	6.049	5.981
IDE	8.919	8.962	8.821	8.831	9.004	9.020
SORL1	8.920	9.057	9.083	8.955	8.824	9.071
OBP2B	7.114	7.295	7.052	7.351	7.249	7.088
TMEM203	9.972	9.996	10.062	9.735	9.742	9.932
OBP2A	9.131	9.199	9.222	9.218	9.228	9.376
UPK3BP1	10.800	10.883	10.960	10.854	11.053	11.082
LINC01222	6.578	6.493	6.101	6.611	6.697	6.511
POLD1	9.372	9.354	9.386	9.088	8.998	9.017
LINC01220	4.482	4.498	4.662	4.435	4.445	4.566
POLD3	8.697	8.686	8.936	8.813	8.681	8.632
POLD4	10.177	10.217	10.445	10.206	10.164	10.139
LINC01227	5.739	6.165	5.932	5.976	5.862	5.755
LINC01224	6.720	6.896	7.110	6.902	7.037	6.882
DLC1	8.551	8.661	8.822	8.340	8.637	8.479
PI4KAP1	9.891	10.001	9.999	9.719	9.650	9.851
PI4KAP2	9.891	10.001	9.999	9.719	9.650	9.851
NUB1	9.410	9.436	9.621	9.197	9.251	9.203
HNRNPKP4	12.826	12.956	12.948	12.901	12.847	12.836
AC007114.2	5.758	5.773	5.879	6.053	5.911	6.002
ID4	6.584	6.714	6.734	6.688	6.880	6.945
ID2	11.808	11.838	11.942	11.473	11.898	11.626
ID3	7.404	7.268	7.327	7.448	7.438	7.226
ID1	11.257	11.303	11.416	11.297	11.245	11.334
MGME1	7.851	7.964	8.133	7.560	7.622	7.547
CPAMD8	7.037	6.972	7.198	7.230	7.389	7.091
NHP2P2	8.708	8.778	8.935	8.806	8.784	8.800
OPLAH	8.147	8.279	8.451	8.096	7.979	8.057
GRAMD2B	9.244	9.198	9.323	9.101	8.945	8.990
GRAMD2A	7.391	7.496	7.247	7.429	7.308	7.369
AHDC1	7.873	7.767	8.194	7.870	8.210	7.722
GLI3	8.390	8.194	8.421	8.385	8.389	8.263
GLI1	7.508	7.375	7.405	7.681	7.570	7.389
GLI4	7.384	7.322	7.304	7.282	7.283	7.239
AC026691.1	7.087	7.043	7.086	7.200	7.255	7.197
ORC6	9.604	9.515	9.701	9.223	9.183	9.319
ORC4	9.923	10.090	10.126	10.189	10.095	10.100
ORC5	8.394	8.423	8.647	8.341	8.277	8.246

ORC2	8.774	8.773	8.814	8.833	8.733	8.755
ORC3	8.585	8.588	8.575	8.301	8.342	8.274
ORC1	7.578	7.469	7.559	7.096	7.158	7.151
DMKN	10.156	10.193	10.397	10.219	10.208	10.078
MAGEA10-MAGE	6.357	6.488	6.284	6.527	6.829	6.543
MIR194-2	8.516	8.399	8.578	8.583	8.641	8.734
GSTK1	10.265	10.408	10.597	10.337	10.285	10.192
AL121758.1	9.825	9.850	10.015	9.912	9.924	9.776
NR0B2	8.164	8.205	8.282	8.410	8.357	8.188
NR0B1	5.892	6.013	6.049	6.093	6.010	6.239
AC022167.3	6.644	6.570	6.683	6.952	7.079	7.129
C2CD3	8.161	8.238	8.222	8.102	8.049	8.182
C2CD2	8.916	8.806	8.891	8.778	8.654	8.807
OSGEP	8.459	8.461	8.653	8.158	8.430	8.397
C2CD6	6.372	6.076	5.723	6.290	6.261	6.182
C2CD5	9.031	9.003	9.037	8.806	8.727	8.708
PRKAG2-AS1	5.298	5.397	5.251	5.692	5.493	5.419
CR753889.1	7.344	7.403	7.456	7.273	7.478	7.510
PPP1R14D	6.937	6.998	7.144	7.124	7.286	7.185
AC243301.1	6.888	6.846	6.941	7.070	7.063	7.099
AC243301.2	6.888	6.846	6.941	7.070	7.063	7.099
AC106785.2	4.146	4.013	4.234	4.252	4.082	4.324
KIZ-AS1	4.871	5.121	5.269	5.045	5.057	4.975
SBSN	6.492	6.607	6.718	6.575	6.868	6.653
CSN2	6.827	6.904	6.890	7.091	7.015	7.134
CSN3	14.082	14.075	14.075	14.068	14.038	14.065
AL021939.1	10.241	10.250	10.344	10.169	10.153	10.173
AC011997.1	4.428	4.371	4.262	4.406	4.398	4.462
EXOC5P1	8.893	9.080	8.953	8.962	9.153	8.753
CNTFR	7.881	7.805	7.733	7.822	7.824	7.961
TACR3	6.051	6.144	6.331	6.086	6.200	6.229
TACR2	7.718	7.836	7.883	7.996	8.044	7.893
TACR1	6.973	6.933	6.948	7.360	7.209	7.071
SERBP1	12.050	12.085	12.112	11.907	11.806	11.884
PIK3CB	9.700	9.708	9.766	9.581	9.513	9.686
KIAA0586	8.551	8.622	8.565	8.621	8.642	8.554
AL355674.1	4.751	5.054	4.827	4.992	5.035	4.926
UBXN7-AS1	5.121	5.064	4.885	5.015	5.148	4.999
ENTR1	9.561	9.617	9.554	9.591	9.542	9.610
AP003680.1	8.023	8.076	7.846	8.083	8.032	8.030
ZSWIM6	9.283	9.205	9.281	9.347	9.259	9.295
ZSWIM7	8.329	8.449	8.462	8.310	8.377	8.458
AC009486.1	7.689	7.714	7.714	7.801	7.834	7.804
FAM71E1	8.378	8.513	8.642	8.398	8.454	8.460
FAM71E2	7.662	7.571	7.855	7.878	7.689	7.786
BARX2	8.247	8.212	8.309	8.167	8.263	8.431
CABYR	6.732	6.607	6.628	6.629	6.648	6.696
BARX1	6.750	6.798	6.752	7.214	7.022	6.831
SH2B2	8.347	8.355	8.512	7.969	8.241	8.256
SH2B3	9.211	9.163	9.112	9.122	9.096	9.129
C19orf18	6.966	6.719	6.745	6.689	6.792	7.241
C19orf12	8.675	8.560	8.592	8.374	8.568	8.466
HCCAT5	8.323	8.622	8.398	8.604	8.602	8.579
ADAMTS9-AS2	7.351	7.441	7.250	7.379	7.344	7.445
ADAMTS9-AS1	4.773	5.071	4.955	5.052	4.972	4.978
AC005064.1	4.919	4.842	4.649	5.094	4.848	4.959

AC015818.3	7.848	7.650	7.758	8.001	7.965	7.991
AC015818.2	6.862	6.915	6.755	6.856	6.890	6.962
LINC02254	5.314	5.046	4.940	5.483	5.415	5.527
AC022893.2	7.223	7.378	7.279	7.338	7.467	7.465
PGLYRP3	6.083	6.263	6.153	6.381	6.489	6.181
PGLYRP2	5.983	5.978	6.167	6.177	6.009	6.047
PGLYRP1	7.416	7.646	7.700	7.762	7.731	7.676
AL158050.1	8.571	8.609	8.615	8.596	8.649	8.578
FIZ1	11.014	10.911	11.064	11.089	11.091	11.090
AL022318.4	8.367	8.476	8.415	8.424	8.563	8.500
RGS22	6.703	7.001	6.924	6.925	6.996	6.946
RGS20	7.043	7.048	7.015	7.041	7.413	7.210
PIPSL	11.433	11.508	11.690	11.319	11.050	11.287
UTS2	5.198	5.134	5.100	5.559	5.420	5.486
FADS1	11.507	11.492	11.554	11.204	11.275	11.429
VPS53	8.608	8.493	8.509	8.512	8.491	8.522
PLB1	8.300	8.376	8.299	8.140	8.252	8.209
SASH3	7.364	7.467	7.300	7.564	7.652	7.553
SASH1	9.777	9.810	9.651	9.481	9.652	9.770
ALKBH5	11.221	11.211	11.398	11.210	11.076	11.049
CYB5A	11.303	11.362	11.461	11.409	11.359	11.349
CYB5B	10.031	10.259	10.349	10.009	9.996	10.110
AL133319.1	5.566	5.265	5.664	5.621	5.838	5.909
ALKBH1	10.006	9.948	10.090	9.929	10.062	9.978
PCK2	9.023	9.082	9.311	8.747	8.805	8.759
PCK1	4.468	4.514	4.528	4.394	4.515	4.512
AC024651.2	6.971	6.819	6.975	7.069	7.111	6.998
AC024651.1	5.315	5.353	5.244	5.329	5.332	5.204
UBE2Q2L	6.582	6.672	6.703	6.718	6.700	6.647
FP236383.5	10.511	10.453	10.731	11.036	10.643	10.881
FP236383.4	10.511	10.453	10.731	11.036	10.643	10.881
FP236383.1	8.286	7.924	8.206	8.188	8.172	8.003
FP236383.3	6.513	6.545	6.726	6.904	6.666	6.760
FP236383.2	8.286	7.924	8.206	8.188	8.172	8.003
MTMR9LP	7.552	7.505	7.788	7.765	7.597	7.589
AC004233.2	8.925	8.905	8.861	8.777	8.776	8.927
ESRP2	10.074	9.999	10.091	10.077	9.842	9.955
ESRP1	9.982	9.962	10.037	9.724	9.730	9.822
DCST1-AS1	7.548	7.427	7.574	7.709	7.771	7.501
ZMPSTE24	11.382	11.467	11.501	11.561	11.295	11.474
MRPL36	9.960	10.026	10.109	9.864	9.918	9.997
MRPL37	10.499	10.590	10.771	10.347	10.312	10.287
MRPL34	8.783	8.863	9.017	8.870	8.774	8.820
MRPL35	9.595	9.541	9.695	9.672	9.582	9.575
MRPL32	11.005	11.117	11.245	11.035	11.055	11.004
NPSR1-AS1	7.107	7.266	6.968	7.056	7.397	7.048
MRPL30	10.248	10.339	10.369	10.194	10.232	10.286
MRPL38	9.809	9.809	10.055	9.834	9.852	9.818
MRPL39	9.108	9.265	9.320	9.209	9.222	9.208
FAM92A	9.046	9.024	9.183	9.146	9.218	9.194
THOC3	10.796	10.924	11.020	10.731	10.555	10.739
THOC2	9.930	9.722	9.888	9.820	9.945	9.605
THOC1	8.703	8.870	8.749	8.541	8.641	8.669
THOC7	9.567	9.581	9.692	9.185	9.507	9.304
THOC6	8.555	8.588	8.829	8.328	8.315	8.338
THOC5	9.084	9.038	9.099	9.028	8.970	8.943

SYS1-DBNDD2	11.289	11.380	11.504	11.240	11.202	11.234
RNF103	10.695	10.793	10.861	10.556	10.496	10.356
RECQL5	8.881	8.949	8.919	8.822	8.839	8.866
RECQL4	10.181	10.114	10.276	9.858	9.898	10.013
C1DP1	9.358	9.532	9.441	9.302	9.362	9.478
SAA4	7.600	7.678	7.772	7.716	7.942	7.785
SAA2	8.137	8.157	8.215	8.120	8.139	8.143
SAA1	8.137	8.157	8.215	8.120	8.139	8.143
NPIPB15	9.065	8.917	8.951	8.872	8.622	9.028
PPOX	8.093	8.067	8.187	7.753	7.871	7.839
YTHDF1	11.531	11.614	11.747	11.522	11.410	11.585
HOXA10-AS	5.487	5.481	5.431	5.226	5.446	5.566
NPIPB11	11.306	11.174	11.094	11.070	11.186	11.250
GTSF1	10.944	10.928	11.088	11.059	11.014	10.998
NPIPB12	11.306	11.174	11.094	11.070	11.186	11.250
AC099552.4	6.574	7.080	6.771	6.976	6.989	7.105
AC022506.1	10.350	10.161	10.485	10.437	10.348	10.271
AL161630.1	5.710	5.525	5.682	5.327	5.701	5.668
NLK	8.481	8.379	8.468	8.082	8.317	8.140
AL365217.1	9.096	9.250	9.086	8.965	9.186	9.065
NLN	9.223	9.444	9.005	9.487	9.332	9.539
TSBP1	4.774	4.876	4.551	5.092	5.015	4.967
SENCR	8.432	8.391	8.233	8.398	8.532	8.546
PHLDB1	8.384	8.512	8.651	8.605	8.585	8.476
PHLDB2	8.527	8.386	8.456	8.073	8.023	8.290
PHLDB3	8.854	8.954	8.970	8.646	8.804	8.732
PDCD6IPP2	6.905	6.937	6.849	7.091	7.161	7.066
AC124319.3	7.427	7.378	7.374	7.308	7.441	7.520
RSL24D1	11.178	11.237	11.277	11.221	11.188	11.299
AC010323.1	9.662	9.593	9.851	9.518	9.446	9.401
KIAA0087	4.174	4.086	4.275	4.258	4.207	4.366
LETMD1	9.192	9.204	9.356	8.816	8.900	8.983
MTMR12	9.628	9.718	9.789	9.432	9.440	9.603
MTMR11	8.588	8.638	8.643	8.445	8.552	8.509
MTMR10	7.573	7.712	7.844	7.819	7.502	7.735
PTCHD4	6.520	6.672	6.388	6.564	6.670	6.222
PTCHD1	5.379	5.705	5.194	5.407	5.391	5.574
BBX	10.166	10.167	10.353	10.119	10.182	9.884
METAP1D	7.677	7.729	7.767	7.486	7.450	7.648
CAVIN2	5.157	4.887	5.172	5.061	5.159	5.091
AL590396.1	6.196	6.243	6.055	6.086	6.331	6.259
CEP57L1	5.176	5.206	5.125	5.262	5.211	5.096
C20orf197	4.039	4.053	4.038	4.006	3.993	4.009
C20orf194	8.534	8.580	8.768	8.581	8.624	8.717
MTND1P33	5.537	5.340	5.322	5.147	5.374	5.206
RNU1-129P	8.002	8.079	7.999	8.131	8.278	8.202
POR	8.945	8.993	9.120	8.724	8.749	8.810
ATP6V0E1P2	6.425	6.515	6.679	6.465	6.581	6.570
AC106786.1	7.196	7.183	7.433	7.371	7.392	7.426
ST3GAL3	10.886	11.148	11.014	10.835	11.074	11.093
ST3GAL2	7.838	7.898	7.893	8.015	7.841	8.010
ST3GAL1	10.918	11.014	11.057	11.003	11.002	10.955
ST3GAL6	10.120	10.240	10.390	9.926	10.068	9.848
ST3GAL5	8.192	8.079	8.062	8.236	8.283	8.373
ST3GAL4	7.994	7.946	8.209	8.261	8.107	7.944
CEP131	7.451	7.632	7.607	7.202	7.269	7.405

CEP135	7.623	7.479	7.364	7.401	7.008	7.023
BCKDHB	8.450	8.342	8.325	8.192	8.353	8.358
BCKDHA	10.322	10.438	10.621	10.377	10.286	10.318
WIPF3	8.779	8.738	8.529	8.829	8.776	9.031
WIPF2	12.860	13.150	13.169	13.155	13.069	12.919
WIPF1	8.160	8.141	8.127	8.224	8.397	8.180
MON1A	8.377	8.423	8.482	8.417	8.458	8.535
MON1B	10.118	10.183	10.170	10.155	10.178	9.959
MSH5-SAPCD1	7.937	7.693	7.892	7.743	7.590	7.680
RBM4B	8.788	8.712	8.729	8.559	8.737	8.678
ELOCP3	7.040	7.038	7.084	6.946	7.034	7.141
TMEM178B	9.026	8.920	9.018	8.384	8.546	8.571
TMEM178A	5.697	5.712	5.544	5.345	5.409	5.603
AC187652.1	8.568	8.283	8.438	8.753	8.615	8.475
MC2R	8.823	8.759	8.536	8.862	8.833	8.943
RPS17P5	10.328	10.454	10.530	10.349	10.390	10.604
RBM41	8.335	8.281	8.319	8.287	8.412	7.903
RBM42	10.907	10.998	11.111	10.719	10.749	10.700
RBM43	8.260	8.052	8.145	7.747	8.016	7.640
RBM45	8.832	8.764	8.813	8.670	8.620	8.554
RBM46	4.005	4.011	4.053	4.216	4.142	4.207
RBM47	12.079	11.844	11.967	11.686	11.760	11.850
RBM48	6.960	6.980	7.004	6.698	6.859	6.949
AL354707.1	11.739	11.805	11.872	11.619	11.607	11.569
HTD2	8.993	9.110	9.323	9.208	9.123	9.181
PPM1G	10.237	10.283	10.385	10.220	9.959	10.061
PPM1F	9.128	9.153	9.168	9.047	9.005	9.079
PPM1E	6.337	6.483	6.112	6.283	6.489	6.468
PPM1D	7.469	7.160	7.387	7.817	7.419	7.361
PPM1B	10.963	10.970	11.070	10.907	10.916	10.866
PPM1A	10.089	10.051	10.211	10.023	9.796	9.801
PPM1N	8.402	8.300	8.467	8.435	8.617	8.571
PPM1M	7.866	7.875	7.852	8.014	7.807	7.790
PPM1L	7.502	7.620	7.668	7.342	7.310	7.423
PPM1K	8.960	8.997	8.895	8.797	8.883	8.834
PPM1J	6.716	6.743	6.730	6.705	7.075	6.824
PPM1H	10.080	10.140	10.104	9.942	9.909	10.011
MYCNUT	7.181	7.203	7.398	7.209	7.177	7.231
CHEK1	8.472	8.355	8.381	8.292	8.320	8.471
LIN28B	4.517	4.583	4.422	4.813	4.774	4.858
GCFC2	8.037	7.978	7.962	7.776	7.557	7.688
AC067956.1	5.420	5.193	5.000	5.195	5.116	5.177
GINS2	10.318	10.363	10.527	10.205	10.120	9.994
GINS3	8.928	8.702	8.875	8.714	8.681	8.602
GINS1	8.244	8.136	8.184	7.686	7.997	7.848
GINS4	8.888	8.858	8.830	8.412	8.366	8.443
PLD4	6.442	6.398	6.445	6.597	6.520	6.377
PLD5	5.070	5.014	5.252	5.065	5.156	5.167
ALG10B	6.303	6.223	6.059	5.526	5.753	5.796
CYP2S1	7.964	7.992	7.755	7.966	8.198	7.934
ADPRH	8.202	8.174	8.100	8.124	8.331	8.117
ADPRM	7.670	7.619	7.748	7.522	7.602	7.772
ADI1	11.093	11.230	11.379	11.277	11.133	11.180
TNK2	8.866	9.029	8.959	8.925	8.778	8.877
RPL10AP6	13.719	13.776	13.864	13.830	13.759	13.867
C9	4.344	4.556	4.400	4.611	4.555	4.294

C3	10.631	10.596	10.561	10.580	10.409	10.563
C2	7.862	7.644	8.029	7.929	7.825	7.760
C7	7.356	7.428	7.166	7.421	7.553	7.562
C6	6.300	6.482	6.276	6.667	6.462	6.761
C5	10.026	9.951	9.951	9.769	9.745	9.807
OGFRP1	8.848	9.031	8.899	8.714	8.931	8.967
EXPH5	9.172	9.319	9.276	9.094	9.102	8.905
ADIG	7.922	7.958	7.953	8.307	8.356	8.345
CS	11.591	11.642	11.719	11.599	11.488	11.465
CP	9.971	9.878	10.059	9.842	10.096	9.838
RMND5A	10.131	10.084	9.996	10.023	9.938	10.008
RMND5B	9.246	9.369	9.373	9.266	9.260	9.256
MIR6847	10.472	10.581	10.785	10.512	10.425	10.389
MIR6848	9.194	9.178	9.537	9.134	9.060	9.153
PISD	8.836	8.849	8.808	8.777	8.674	8.881
AP3B1	10.589	10.588	10.638	10.528	10.513	10.624
PP2D1	4.159	4.392	4.192	4.386	4.389	4.324
AP3B2	6.643	6.762	6.739	6.730	6.794	6.825
ZNF861P	7.938	8.012	7.973	8.075	8.070	8.161
PROX1-AS1	3.988	3.938	3.832	3.815	4.079	3.992
LINC02499	6.551	6.475	6.510	6.418	6.709	6.536
P4HB	13.369	13.469	13.531	13.495	13.426	13.407
LINC02492	5.290	5.458	5.452	5.650	5.561	5.299
LINC02493	6.504	6.373	6.121	6.664	6.576	6.390
PGA3	7.769	7.814	7.771	7.818	7.834	7.782
PGA4	7.769	7.814	7.771	7.818	7.834	7.782
PGA5	7.769	7.814	7.771	7.818	7.834	7.782
VCX3B	7.570	7.633	7.681	7.804	7.550	7.687
VCX3A	7.570	7.633	7.681	7.804	7.550	7.687
PPP1CB	10.785	10.960	10.944	10.805	10.728	10.740
PPP1CC	12.193	12.250	12.362	12.331	12.119	12.302
PPP1CA	11.339	11.475	11.588	11.280	11.095	11.291
MPPED1	7.628	7.671	7.656	7.641	7.585	7.555
MPPED2	5.305	5.369	5.069	5.434	5.290	5.305
PCLAF	12.368	12.370	12.525	12.105	12.125	12.064
SRP9	12.303	12.337	12.348	12.151	12.176	12.312
AC011511.4	9.049	9.019	9.074	8.903	8.937	8.928
AC011511.1	8.598	8.548	8.677	8.515	8.525	8.580
AL138690.1	4.245	4.391	4.235	4.341	4.305	4.273
ZYX	10.623	10.642	10.688	10.667	10.660	10.531
ZNF460	6.168	5.951	6.059	5.800	5.869	5.878
VSIG10	10.055	9.936	9.972	9.751	10.028	9.656
HIF1AN	8.851	8.841	9.007	8.877	8.923	8.761
AL033530.1	6.667	6.912	6.610	6.828	6.808	6.904
DENND3	8.420	8.434	8.432	8.393	8.356	8.281
CREBRF	8.216	8.292	8.332	8.045	8.081	7.943
PPP1R16A	9.051	9.128	9.255	9.257	9.190	9.075
PPP1R16B	7.322	7.110	7.012	7.586	7.511	7.522
BLNK	7.177	7.188	7.107	6.775	7.026	6.953
KIAA1841	6.969	6.943	7.292	7.072	6.892	6.914
NUCB1	10.609	10.618	10.850	10.596	10.589	10.601
HSDL1	7.540	7.538	7.642	7.242	7.185	7.549
HSDL2	9.214	9.166	9.320	8.918	8.974	8.995
NUCB2	8.861	8.927	8.997	8.973	9.065	8.973
SOCS5	9.238	9.289	9.325	9.206	9.121	9.203
SOCS4	9.228	9.090	9.111	8.911	8.894	9.031

SOCS7	10.416	10.394	10.382	10.035	10.081	10.166
SOCS6	7.965	7.971	7.925	7.940	7.884	7.914
SOCS1	7.435	7.393	7.418	7.605	7.690	7.556
SOCS3	8.024	8.244	8.310	8.304	8.484	8.037
SOCS2	5.282	5.176	5.189	5.330	5.232	5.375
PPP2CA	11.555	11.622	11.695	11.628	11.549	11.577
PPP2CB	12.090	12.176	12.206	11.990	11.945	11.922
NPHP1	6.249	6.532	6.438	6.011	6.080	6.324
SRPX	5.609	5.731	5.566	5.463	5.350	5.369
NR2F1-AS1	8.522	8.627	8.570	8.531	8.597	8.665
ENAM	8.338	8.158	7.925	8.230	8.284	8.201
AC012456.1	7.183	7.255	6.898	6.868	7.135	7.365
IL1R2	6.405	6.484	6.328	6.729	6.522	6.415
IL1R1	7.546	7.588	7.514	7.178	7.211	7.237
PTN	7.916	7.994	7.876	7.861	7.908	7.944
AC008749.1	6.359	6.437	6.304	6.335	6.605	6.327
ABHD18	7.917	7.955	7.879	7.964	7.895	7.914
PTH	4.005	4.016	3.997	4.095	4.272	4.172
ABHD15	8.500	8.730	8.878	8.594	8.779	8.479
ABHD10	9.345	9.268	9.168	9.089	9.171	9.182
ABHD11	9.862	9.869	10.038	9.924	10.006	9.888
ABHD12	8.932	8.984	9.101	8.481	8.785	8.738
ABHD13	4.572	4.745	4.679	4.643	4.734	4.563
NGRN	12.100	12.172	12.264	12.179	12.116	12.160
PTS	9.511	9.582	9.733	9.507	9.495	9.421
AC096649.1	8.220	8.364	8.463	8.407	8.371	8.327
HSPBP1	10.443	10.473	10.608	10.323	10.306	10.336
ARPC3	12.178	12.366	12.490	12.429	12.479	12.468
ARPC2	13.290	13.162	13.354	13.276	13.290	13.274
AC016596.3	14.596	14.604	14.644	14.671	14.651	14.656
ARPC5	11.910	11.978	12.086	12.038	11.905	12.035
ARPC4	10.705	10.675	10.869	11.014	10.966	10.847
ITPR2	7.798	7.684	7.859	7.900	8.080	8.096
ITPR3	11.210	11.181	11.343	10.973	10.923	11.023
ITPR1	6.274	6.351	6.387	6.312	6.388	6.388
IL1RN	10.371	10.452	10.469	10.295	10.412	10.252
AC011379.2	6.064	6.103	6.090	5.912	6.147	6.008
ERCC6L2	7.737	7.728	7.638	7.305	7.228	7.269
CASKIN2	9.043	9.207	9.258	9.068	9.057	8.987
CASKIN1	8.526	8.762	8.457	8.349	8.690	8.547
LAP3	10.618	10.701	10.712	10.455	10.566	10.409
AL121871.1	11.640	11.725	11.844	11.776	11.676	11.746
FAM217A	6.450	6.239	6.349	6.474	6.484	6.517
FAM217B	9.233	9.185	9.296	9.015	8.924	9.174
OPN5	7.241	7.428	7.246	7.262	7.407	7.356
OPN4	8.021	8.010	7.982	8.159	8.148	8.082
OPN3	9.922	10.007	10.103	10.020	9.789	9.984
HADHAP1	7.449	7.231	7.447	7.556	7.629	7.465
RPL14P1	12.602	12.676	12.871	12.830	12.747	12.797
CHRM5	6.959	6.791	6.656	6.979	7.106	6.968
AC093831.2	4.333	4.252	4.181	4.216	4.116	4.391
TMEM108-AS1	6.680	6.650	6.751	6.705	6.628	6.880
HIVEP1	6.917	7.105	6.948	7.045	7.039	7.013
HIVEP2	8.787	8.848	8.874	8.971	8.866	9.026
HIVEP3	9.049	9.204	9.139	9.053	9.158	9.131
RASL12	7.314	7.204	7.314	7.285	7.355	7.481

FP325313.3	6.809	6.865	6.701	7.148	7.106	6.941
AP000679.1	8.036	8.182	8.007	8.061	8.311	8.118
OOSP2	5.484	5.455	5.222	5.537	5.583	5.586
CPVL-AS1	7.395	7.486	7.567	7.465	7.681	7.672
PRADC1	8.747	8.858	8.951	8.714	8.857	8.895
STPG3	7.922	8.021	7.789	8.111	7.985	8.045
STPG2	6.969	7.225	7.075	7.054	7.189	7.124
STPG1	7.827	7.884	7.972	7.885	8.035	7.899
STPG4	6.174	6.361	6.163	6.429	6.231	6.269
PGRMC2	9.409	9.454	9.415	9.145	9.305	9.317
PGRMC1	12.304	12.387	12.475	12.226	12.195	12.253
FOXCUT	8.595	8.776	8.712	8.680	8.933	8.864
AC245088.1	7.597	7.542	7.551	7.581	7.769	7.718
MIR29B2CHG	7.181	7.193	7.221	7.143	6.856	7.309
NSUN5P2	9.809	9.780	9.862	9.586	9.679	9.665
NSUN5P1	9.809	9.780	9.862	9.586	9.679	9.665
AP006222.1	7.667	7.929	8.164	7.639	7.672	7.716
AP000280.1	7.959	7.950	7.940	7.924	8.027	7.988
MTND2P39	4.384	4.319	4.298	4.361	4.368	4.409
SAMD14	7.702	7.495	7.723	7.837	7.864	7.573
SAMD15	9.387	8.955	8.902	9.310	9.091	9.267
SAMD12	4.547	4.430	4.518	4.504	4.775	4.431
SAMD13	8.033	8.079	8.139	7.785	7.899	7.949
SAMD10	7.954	8.091	8.110	7.991	8.078	8.130
SAMD11	7.152	7.309	7.191	7.178	7.176	7.240
SPNS3	7.334	7.696	7.636	7.711	7.369	7.520
AC006262.1	6.498	6.437	6.358	6.514	6.585	6.413
PCDH11X	5.767	5.865	5.921	5.519	5.724	5.729
PCDH11Y	5.767	5.865	5.921	5.519	5.724	5.729
IGF1R	9.392	9.447	9.562	9.080	9.383	9.245
COQ8B	9.355	9.362	9.657	9.683	9.450	9.443
COQ8A	8.852	8.836	8.864	8.716	9.046	8.738
PHETA2	7.825	7.621	7.836	8.089	7.962	7.995
SEC14L1P1	6.970	6.785	6.672	6.799	6.974	6.769
AL158071.4	6.614	6.677	6.686	6.655	6.831	6.840
TEX9	6.065	6.094	6.045	5.780	5.808	5.775
CD3EAP	9.444	9.491	9.563	9.354	9.232	9.305
TEX2	8.966	8.965	9.014	8.922	9.066	8.892
ARHGAP17	9.806	9.769	9.801	9.725	9.616	9.602
STARD8	8.070	8.151	8.133	8.357	8.250	8.424
ARHGAP15	6.734	6.453	6.374	6.905	6.886	6.784
ARHGAP12	9.118	9.214	9.126	9.035	8.888	9.098
ARHGAP10	8.247	8.302	8.351	8.169	8.145	8.230
STARD3	13.340	13.374	13.495	13.293	13.181	13.187
STARD6	4.924	5.202	5.024	5.046	5.019	5.189
STARD7	11.800	11.871	11.997	11.861	11.694	11.716
ARHGAP18	10.947	10.823	10.916	10.871	11.164	10.717
ARHGAP19	7.961	8.004	8.142	7.649	7.834	7.807
IL7R	6.972	6.896	6.876	7.005	6.908	7.013
PTRHD1	9.719	9.711	9.955	9.724	9.771	9.758
OR51A1P	7.136	7.229	6.980	7.091	7.401	7.159
AC015726.1	4.829	4.658	5.000	4.946	4.742	4.799
AC091151.4	4.499	4.483	4.444	4.537	4.656	4.582
TDGF1P3	7.201	7.200	7.349	7.310	7.139	7.101
AC091151.3	5.647	5.339	5.495	5.452	5.784	5.531
AC091151.2	4.499	4.483	4.444	4.537	4.656	4.582

UBA7	8.415	8.606	8.651	8.312	8.504	8.406
AC073916.1	7.654	7.768	7.885	7.964	7.998	7.790
BCDIN3D	7.966	8.010	7.959	7.803	7.908	7.939
SPIDR	7.796	7.846	8.017	7.686	7.777	7.744
HID1	9.704	9.705	9.810	9.886	9.882	9.672
SND1-IT1	7.834	7.791	7.614	7.671	7.828	7.713
RING1	10.688	10.737	10.852	10.672	10.600	10.648
AC008012.1	8.671	8.717	8.787	8.666	8.611	8.617
BACH1-IT1	6.517	6.877	6.519	6.706	6.749	6.746
MIR4458HG	6.899	6.783	6.987	6.875	6.850	6.861
CBARP	8.140	8.054	8.302	8.388	8.311	8.181
FMR1NB	3.961	3.999	4.002	4.246	4.138	4.147
ITPRIP	8.328	8.260	8.068	8.170	8.333	8.227
DDTL	10.518	10.608	10.873	10.576	10.453	10.549
SETX	8.598	8.785	8.689	8.341	8.481	8.459
CARMIL3	8.030	7.896	7.922	8.139	8.068	8.092
SATB2-AS1	7.435	7.351	7.460	6.927	7.270	7.209
TIMD4	6.739	6.651	6.510	6.622	6.758	6.835
C4orf47	6.360	6.210	6.344	6.278	6.368	6.315
C4orf46	8.103	8.031	7.940	7.419	7.472	7.573
C4orf45	6.598	6.584	6.363	6.509	6.705	6.669
PBRM1	9.394	9.525	9.487	9.123	9.334	8.921
C4orf48	9.515	9.641	9.938	9.601	9.579	9.613
AL031587.1	7.431	7.685	7.588	7.526	7.607	7.723
AL031587.3	6.814	6.586	6.640	6.573	6.672	6.796
CBWD3	11.277	11.323	11.404	11.049	11.073	11.051
CR354443.1	11.996	12.053	12.198	11.947	11.842	11.914
CR933537.1	7.145	7.246	7.000	7.072	7.096	7.194
C15orf56	6.352	6.160	6.204	6.621	6.569	6.374
C15orf54	5.564	5.483	5.412	5.604	5.615	5.580
ERAL1	9.814	9.744	9.916	9.719	9.719	9.583
DDX17	12.739	12.780	12.880	12.778	12.652	12.774
ZNF124	6.943	6.812	6.908	7.036	7.185	7.011
AC002059.1	7.969	7.762	7.607	7.872	8.131	7.868
DDX10	9.260	9.291	9.426	9.059	9.048	9.203
DDX11	10.054	10.138	10.088	9.811	9.917	9.945
VN1R108P	3.803	3.692	3.779	3.763	3.946	3.992
DDX18	10.933	10.995	10.982	11.101	11.008	10.900
VWCE	7.780	7.748	7.672	7.756	7.875	7.814
AC024884.2	7.553	7.703	7.804	7.710	7.872	7.743
ARFIP1	9.790	9.887	9.820	9.644	9.693	9.658
ARFIP2	11.000	11.030	11.102	11.011	10.784	10.876
AC007656.3	4.432	4.451	4.480	4.515	4.607	4.546
SCP2D1	6.929	6.805	7.356	7.135	6.912	7.256
TRIP10	9.160	9.138	9.390	9.072	9.224	9.265
TRIP11	8.250	8.289	8.328	8.360	8.373	8.402
TRIP12	10.564	10.645	10.692	10.511	10.365	10.493
TRIP13	9.055	9.090	9.308	8.702	8.786	8.829
NCLN	8.744	8.992	9.036	8.846	9.097	8.778
DNAL4	8.483	8.631	8.778	8.528	8.608	8.485
DNAL1	8.310	8.375	8.324	8.468	8.265	8.199
MIR4751	9.044	9.016	9.121	9.158	8.960	8.829
MIR4750	9.002	9.259	9.254	8.827	8.968	8.939
AC244635.2	5.835	5.745	5.792	5.711	6.008	5.820
AC244635.3	4.546	4.452	4.404	4.410	4.521	4.445
LINC01679	7.398	7.139	7.333	7.473	7.383	7.275

AP005061.2	3.590	3.864	4.036	3.811	3.712	3.757
IDI2-AS1	7.107	7.183	7.320	7.203	7.559	7.557
FGFBP2	5.430	5.252	5.348	5.594	5.567	5.335
FGFBP1	8.628	8.671	8.808	8.624	8.807	8.759
ARMC10	9.444	9.534	9.588	9.472	9.496	9.373
LINC01859	4.886	4.731	4.719	4.952	4.914	4.872
ARMC12	6.990	7.191	7.174	7.372	7.187	7.267
BX908738.3	7.145	7.246	7.000	7.072	7.096	7.194
BX908738.2	6.517	6.543	6.395	6.499	6.649	6.716
USF2	11.449	11.461	11.672	11.427	11.548	11.401
SUCLG2P2	9.971	10.087	10.072	9.808	9.823	9.824
NTPCR	8.789	8.853	9.095	8.808	8.963	8.976
DLEU2	6.793	6.807	6.557	6.805	6.617	6.759
CSTF2T	8.809	9.050	9.042	8.767	8.719	8.623
RPL13AP17	9.543	9.432	9.582	9.699	9.741	9.658
TAS1R1	6.677	6.858	6.559	6.744	6.841	6.931
TAS1R2	6.967	6.826	6.867	6.949	7.148	6.986
AL645568.1	6.628	7.039	6.979	7.005	6.877	6.732
AP001830.1	6.946	6.746	7.145	7.215	7.149	6.991
MIR7-3HG	5.347	5.634	5.600	5.789	5.686	5.590
HSBP1	11.417	11.505	11.621	11.638	11.554	11.689
GTF2E1	8.094	8.158	8.193	7.928	8.057	8.116
GTF2E2	9.868	9.843	9.943	9.920	9.881	9.850
ST20-MTHFS	10.325	10.242	10.373	10.338	10.373	10.257
AC000120.3	11.508	11.576	11.617	11.262	11.230	11.545
TEX53	6.095	6.078	6.066	6.280	6.265	6.282
PABPC1L2B	8.138	8.274	8.299	8.070	8.252	8.375
PLEK2	10.337	10.420	10.466	10.230	10.283	10.302
AL445531.1	4.737	4.880	4.526	4.864	4.842	4.899
AC000120.2	7.754	7.632	7.654	7.083	7.234	7.328
TCTE3	9.398	8.977	8.681	9.538	9.339	9.330
TCTE1	6.437	6.464	6.430	6.415	6.533	6.484
AC000123.2	5.024	5.060	4.785	5.055	5.118	5.214
AC000123.3	6.301	6.101	5.879	5.759	5.974	5.949
MIR3196	7.521	7.639	7.700	7.740	7.511	7.556
RPS26P15	12.821	12.916	13.024	12.923	12.903	12.978
CLSPN	7.508	7.471	7.613	7.150	7.153	7.098
ARLNC1	7.000	7.445	7.331	7.313	7.238	7.347
SLC2A1-AS1	7.091	6.768	6.784	6.995	6.882	7.042
FRK	9.169	9.068	9.154	9.090	9.066	9.183
SNORA51	8.486	8.505	8.685	8.393	8.476	8.334
FRY	6.407	6.332	6.428	6.378	6.436	6.315
AC025271.2	4.838	4.943	4.975	4.972	4.959	4.947
AC099524.1	5.789	5.709	5.711	5.873	5.973	6.136
OXLD1	9.462	9.474	9.770	9.453	9.486	9.525
MSH2	9.839	9.880	9.995	9.595	9.318	9.640
HELB	7.810	7.934	7.908	7.978	7.863	7.860
MSH4	4.651	4.812	4.950	4.826	4.704	5.044
MSH5	7.937	7.693	7.892	7.743	7.590	7.680
MSH6	10.052	10.029	10.202	10.025	9.816	9.945
TMEM169	8.145	8.205	8.135	8.008	8.166	8.160
MYOCD	6.349	6.364	6.441	6.251	6.379	6.473
AC010327.6	9.863	9.901	10.100	9.944	9.785	9.728
AC010327.5	7.540	7.790	7.967	8.062	7.730	7.861
AC010327.4	7.695	7.865	7.715	7.957	7.849	7.864
MUC3A	9.114	9.032	9.134	9.185	9.146	9.088

ASMTL-AS1	8.115	7.831	7.854	8.013	7.886	8.218
BNIP3P1	10.207	10.262	10.578	10.406	10.415	10.458
COP1	9.412	9.641	9.605	9.455	9.450	9.487
PACSIN1	8.614	8.481	8.618	8.947	8.688	8.757
PACSIN3	7.798	7.866	8.033	7.765	7.856	7.820
PACSIN2	12.734	12.759	12.829	12.470	12.424	12.649
COPA	12.107	12.179	12.233	11.962	12.107	11.901
COPE	10.403	10.416	10.712	10.608	10.533	10.428
H2BC19P	7.171	7.016	7.373	7.097	7.323	7.445
PM20D1	7.717	7.823	7.445	7.669	7.661	7.795
PM20D2	9.978	10.328	10.218	10.311	10.351	10.065
TOE1	9.204	9.243	9.460	9.190	9.217	9.068
CDIPTOSP	7.681	7.612	7.565	7.682	7.844	7.937
GPR135	6.866	6.749	6.829	7.223	7.074	7.045
GPR137	9.334	9.476	9.432	9.589	9.586	9.497
GPR132	6.966	7.037	6.959	7.047	7.128	7.163
LY6E-DT	7.103	7.298	7.309	7.143	7.262	7.364
WDR76	6.903	7.352	7.467	6.986	6.925	7.110
MFRP	8.719	8.775	8.662	8.644	8.661	8.727
ATF7IP	9.538	9.467	9.468	9.408	9.465	9.382
LINC02043	7.435	7.496	7.631	7.690	7.587	7.441
LINC02042	5.595	5.906	5.469	5.503	5.664	5.538
FAM95B1	7.165	7.227	7.364	7.707	7.106	7.289
LINC02047	5.682	5.599	5.609	5.855	5.722	5.973
LINC02044	6.297	6.377	6.050	6.125	6.572	6.362
AP000251.1	8.341	8.337	8.230	8.141	8.187	8.151
AC040160.2	6.766	6.939	6.988	7.065	6.993	7.041
AC040160.1	8.401	8.443	8.513	8.251	8.152	8.356
TIAM1	8.418	8.403	8.449	8.043	8.011	8.187
ANKRD30B	4.172	4.376	4.162	4.365	3.965	4.234
HAL	7.667	7.656	7.543	7.636	7.774	7.664
ERLEC1	11.273	11.369	11.442	11.625	11.573	11.543
GAS5-AS1	6.852	6.825	6.885	6.937	6.964	6.704
MCMDC2	4.600	4.635	4.488	4.536	4.571	4.639
AC124312.5	7.614	7.618	7.490	7.729	7.716	7.734
LRRC70	4.701	4.734	4.620	4.814	4.654	4.610
LRRC71	7.593	7.935	7.945	7.905	7.949	7.887
LRRC72	5.856	5.964	5.904	5.986	6.158	6.068
LRRC73	6.950	6.778	7.194	7.259	7.279	6.805
RAI14	8.736	8.966	8.914	8.703	8.825	8.710
CHURC1-FNTB	10.012	10.125	10.048	9.966	9.944	10.070
AL136967.1	9.120	9.162	8.889	9.214	9.279	9.241
LINC01532	7.157	6.981	7.131	7.137	6.950	6.923
LINC01537	3.602	3.793	3.651	3.839	3.672	3.770
LINC01534	7.468	7.349	7.314	7.339	7.152	7.282
LINC01535	6.483	6.280	6.139	6.372	6.524	6.371
LINC01538	4.074	4.089	3.953	4.073	4.112	4.096
LINC01539	7.456	7.628	7.173	7.524	7.511	7.553
PNPLA3	8.736	8.668	8.830	8.500	8.749	8.623
PNPLA2	12.518	12.378	12.501	12.619	12.562	12.504
PNPLA1	4.004	4.368	4.069	4.227	3.893	4.240
PNPLA7	6.952	6.955	6.976	6.879	7.007	6.978
PNPLA6	9.161	9.192	9.356	9.100	9.121	9.165
PNPLA5	6.427	6.637	6.489	6.489	6.536	6.717
PNPLA4	9.191	9.119	9.240	9.182	9.183	9.166
PNPLA8	8.568	8.597	8.669	8.499	8.626	8.383

AL359532.1	5.441	5.464	5.536	5.453	5.400	5.555
AL121672.1	7.172	6.797	6.759	7.164	7.297	7.007
PINLYP	6.431	6.658	6.315	6.467	6.335	6.511
SFPQ	11.124	11.209	11.228	11.081	11.037	11.007
HMGN2P46	5.493	5.499	5.436	5.433	5.517	5.501
PWWP3A	8.611	8.628	8.648	8.509	8.490	8.514
HMGN2P41	12.569	12.690	12.791	12.495	12.553	12.522
PWWP3B	4.332	4.374	4.297	4.136	4.289	4.321
AC243725.1	11.460	11.677	11.671	11.488	11.544	11.414
THSD7B	5.184	5.090	5.173	5.405	5.202	5.274
PHTF2	8.516	8.536	8.692	8.341	8.314	8.453
PHTF1	7.684	7.729	7.630	7.761	7.934	7.767
THSD7A	8.850	8.926	8.709	8.726	8.778	8.631
SAPCD1-AS1	7.481	7.537	7.684	7.497	7.376	7.559
GPR88	4.322	4.473	4.297	4.508	4.360	4.392
WBP1L	9.731	9.767	9.885	9.538	9.378	9.537
VCPIP1	7.128	7.237	7.195	7.000	7.072	7.230
PDCD4-AS1	6.143	6.087	6.019	6.078	5.948	5.916
GPR82	6.904	7.000	7.086	6.909	7.378	7.209
GPR83	5.554	5.524	5.662	5.946	5.819	5.622
GPR84	6.303	6.334	6.035	6.373	6.360	6.300
GPR85	4.934	4.752	5.162	5.135	4.854	4.854
GPR87	6.040	6.103	6.178	6.235	6.324	6.160
FOXP4-AS1	5.382	5.543	5.699	5.657	5.676	5.648
WBP11	10.756	10.892	10.918	10.623	10.639	10.613
ZC3HAV1L	4.054	3.875	4.052	3.899	3.993	4.126
TARDBP	10.833	10.745	10.830	10.562	10.493	10.559
PRKRIP1	8.805	8.810	8.892	8.541	8.433	8.629
ZFP64	10.059	9.994	10.203	9.877	9.890	9.927
PALLD	8.067	8.102	8.254	8.011	7.969	8.050
RBMS1	10.452	10.323	10.361	10.325	10.323	10.527
RBMS3	7.001	6.937	7.330	7.209	7.372	7.291
RBMS2	10.370	10.439	10.420	10.234	10.109	10.193
NOXA1	8.376	8.428	8.616	8.576	8.451	8.614
DAD1	11.721	11.846	11.881	11.781	11.684	11.801
GDF9	7.069	7.186	7.055	7.272	6.924	7.298
GDF3	7.577	7.395	7.652	7.546	7.597	7.633
GDF2	8.153	8.278	8.400	8.564	8.378	8.208
GDF1	7.718	7.798	7.840	7.459	7.611	7.721
GDF7	7.415	7.231	7.321	7.414	7.429	7.567
GDF5	8.016	8.052	7.865	8.148	8.055	8.209
NPIPB3	11.306	11.174	11.094	11.070	11.186	11.250
NPIPB2	11.306	11.174	11.094	11.070	11.186	11.250
NPIPB5	11.306	11.174	11.094	11.070	11.186	11.250
NPIPB4	11.306	11.174	11.094	11.070	11.186	11.250
NPIPB6	9.065	8.917	8.951	8.872	8.622	9.028
AC007431.1	6.900	6.890	6.805	6.646	6.865	6.701
C3orf20	7.813	7.740	7.920	8.066	8.058	7.915
C3orf22	7.169	7.181	7.205	7.209	7.400	7.207
WNT9B	6.722	6.757	6.635	6.729	6.937	6.826
WNT9A	6.641	6.698	6.967	6.835	6.812	6.699
AC046185.3	8.031	8.184	8.059	8.292	8.080	8.112
AC046185.1	9.261	9.336	9.282	9.117	9.264	9.231
AC008073.3	5.333	5.382	5.291	5.671	5.621	5.675
ABCA11P	6.101	5.876	5.792	6.010	5.695	5.834
LGSN	4.101	4.165	4.009	4.180	4.382	3.908

CCDC102A	7.068	7.100	7.172	7.029	7.165	7.005
CCDC102B	3.763	3.565	3.754	3.625	3.729	3.687
CNTRL	7.803	7.656	7.593	7.578	7.715	7.578
COX8C	3.953	4.129	4.047	4.060	4.077	4.071
AC139530.2	9.992	10.057	10.225	9.966	9.955	9.987
AC139530.1	7.389	7.341	7.343	7.271	7.391	7.368
VSTM2A	7.060	7.024	7.064	6.968	6.898	7.183
AC006455.5	7.341	7.285	7.472	7.436	7.497	7.359
LSG1	10.308	10.296	10.289	10.139	10.428	10.164
AL359510.2	5.610	5.595	5.777	5.832	5.749	5.707
RALGAPA1P1	7.486	7.516	7.402	6.946	7.074	7.014
HNRNPLL	9.248	9.406	9.478	9.190	9.278	9.240
BRMS1	9.657	9.741	9.853	9.614	9.673	9.708
FOXK2	9.822	9.813	9.922	9.761	9.787	9.832
FOXK1	8.892	8.800	8.720	8.404	8.416	8.370
DPY19L1	9.795	9.695	9.723	9.607	9.661	9.689
RFXANK	8.506	8.548	8.764	8.458	8.333	8.443
TMEM119	6.255	6.232	6.089	6.472	6.479	6.195
GTF3C2-AS1	7.680	7.452	7.492	7.648	7.729	7.676
DPY19L4	8.876	8.855	8.842	8.684	8.692	8.760
IL6R-AS1	5.422	5.617	5.218	5.408	5.517	5.526
TMEM115	8.712	8.784	8.918	8.728	8.681	8.715
TMEM116	8.023	8.169	8.201	7.989	8.017	7.991
TMEM117	5.987	5.766	5.823	5.882	6.093	5.914
AC104794.4	4.841	5.043	5.002	4.990	5.010	4.919
SLC35A4	10.746	10.769	10.787	10.592	10.497	10.623
SLC35A5	8.714	8.882	8.867	8.593	8.636	8.721
SLC35A1	9.175	9.233	9.283	9.171	9.178	9.270
SLC35A2	10.593	10.685	10.703	10.542	10.533	10.521
SLC35A3	9.884	9.857	9.856	9.675	9.652	9.950
MPDZ	6.846	6.847	6.909	7.021	6.871	6.965
POLRMTP1	6.736	6.850	6.992	6.515	6.929	6.691
ASIC5	6.295	6.333	6.401	6.372	6.348	6.361
ASIC4	7.040	6.928	6.897	7.170	7.005	7.009
ASIC1	8.505	8.481	8.599	8.547	8.502	8.547
ASIC3	6.501	6.463	6.558	6.389	6.491	6.468
ASIC2	6.856	6.720	6.695	7.035	6.981	6.872
ABCB11	6.683	6.550	6.652	6.367	6.485	6.473
ABCB10	7.855	7.990	7.962	7.526	7.437	7.681
MMD2	8.001	8.055	8.210	8.001	8.110	8.096
PMP22	7.168	7.172	7.267	7.189	7.272	7.172
RBPMS	11.555	11.609	11.775	11.477	11.488	11.515
FMC1-LUC7L2	10.243	10.299	10.338	10.279	10.343	10.154
TOX4	10.593	10.659	10.876	10.693	10.507	10.681
TOX3	9.591	9.581	9.560	9.220	9.221	9.400
STON2	8.432	8.458	8.587	8.194	8.200	8.286
STON1	7.264	7.147	7.093	7.244	7.166	7.366
BEST3	6.498	6.281	6.500	6.335	6.492	6.396
BEST2	7.634	7.623	7.246	7.779	7.673	7.632
BEST1	9.612	9.593	9.640	9.447	9.496	9.562
BEST4	4.610	4.892	4.657	4.672	4.533	4.664
AP000708.1	3.804	3.929	3.844	4.036	3.864	3.891
BX005019.1	6.043	6.078	5.542	6.050	6.056	6.054
CU633967.1	7.923	7.782	7.902	7.606	7.461	7.712
PXDC1	9.027	9.035	9.248	8.979	8.873	8.958
FYTTD1	11.035	11.056	11.117	11.027	10.963	11.039

KIAA1614	7.538	7.565	7.572	7.523	7.683	7.567
AP000534.1	3.504	3.628	3.499	3.603	3.486	3.514
AC132938.2	7.348	7.635	7.484	7.404	7.520	7.637
TNPO3	9.688	9.527	9.660	9.664	9.356	9.494
TNPO1	11.861	12.014	12.147	11.676	11.877	11.409
SIK1B	8.518	8.535	8.471	8.560	8.466	8.528
SPA17	7.668	7.757	7.534	7.527	7.783	7.653
CLUHP3	8.323	8.131	8.289	8.262	8.200	8.541
SP3P	6.054	6.183	6.199	6.082	6.276	6.278
AC275673.1	3.830	4.000	4.066	3.900	3.760	4.082
CFAP58	6.723	6.778	6.810	6.755	6.885	7.009
METTL8	8.215	8.320	8.417	8.090	8.268	7.723
METTL9	11.044	11.184	11.336	11.326	11.157	11.106
AC005632.2	7.250	7.199	7.397	7.262	7.320	7.248
METTL1	9.369	9.416	9.551	9.463	9.486	9.428
COLCA2	7.394	7.485	7.282	7.489	7.324	7.298
METTL3	8.895	8.914	9.077	8.852	8.936	8.879
METTL4	8.196	8.376	8.351	8.056	8.100	8.243
METTL5	10.639	10.757	10.825	10.606	10.533	10.683
METTL6	8.073	7.964	8.093	7.793	7.746	7.793
GLULP4	8.278	8.330	8.503	8.137	8.336	8.328
KU645198.1	7.337	7.221	7.177	7.296	7.440	7.287
ANO7	9.125	9.198	9.056	9.161	9.130	9.150
TMEM39A	8.915	8.954	8.860	8.961	9.034	9.068
SLC18B1	9.914	9.898	9.960	9.713	9.729	9.673
HCFC2	7.174	7.158	7.194	7.033	7.219	7.042
HCFC1	9.569	9.670	9.605	9.536	9.386	9.507
ROPN1	7.349	7.622	7.194	7.343	7.300	7.324
ASZ1	3.723	3.840	3.856	4.130	4.131	3.824
FOXD4L1	7.180	7.185	7.366	7.415	7.286	7.122
MOV10L1	8.344	8.298	8.256	8.548	8.493	8.481
STON1-GTF2A1L	3.879	3.831	3.816	3.941	3.865	3.832
PCSK1N	10.622	10.726	10.940	10.736	10.698	10.667
AC270306.4	7.236	6.877	7.115	7.650	7.276	7.517
C8orf74	7.980	8.091	8.139	8.175	8.251	8.145
PDPK1	10.621	10.595	10.730	10.476	10.308	10.274
AL928970.1	5.019	5.179	5.401	5.395	5.216	5.137
AL596202.1	6.465	6.506	6.528	6.549	6.537	6.548
RPF1	10.334	10.441	10.534	10.349	10.345	10.328
RPF2	9.828	9.797	9.960	9.759	9.968	9.688
CR933842.1	6.517	6.543	6.395	6.499	6.649	6.716
ZNF688	8.424	8.585	8.771	8.272	8.266	8.390
ZNF689	9.571	9.575	9.481	9.383	9.871	9.547
AL356299.3	11.157	11.265	11.450	11.108	10.923	11.096
ZNF682	4.551	4.313	4.410	4.571	4.640	4.625
ZNF683	8.116	8.135	8.250	8.333	8.272	8.212
ZNF680	6.874	6.891	6.904	6.920	6.770	6.932
ZNF681	6.189	6.170	6.250	6.041	5.946	6.045
ZNF687	9.666	9.407	9.669	9.826	9.859	9.680
ZNF684	4.781	4.802	5.010	4.757	5.016	5.085
AP5M1	10.099	10.132	10.059	10.056	9.938	10.106
PCDHGC3	11.092	11.102	11.188	11.070	11.043	11.048
PCDHGC4	11.092	11.102	11.188	11.070	11.043	11.048
PCDHGC5	11.092	11.102	11.188	11.070	11.043	11.048
RFTN1	6.899	7.006	7.091	6.749	6.906	6.916
POP1	7.402	7.409	7.430	7.545	7.653	7.553

POP7	9.297	9.499	9.681	9.185	9.338	9.328
POP5	10.905	11.069	11.158	10.803	10.816	10.967
POP4	10.460	10.478	10.588	10.385	10.356	10.406
ACE2	9.511	9.509	9.543	8.935	8.894	8.988
GSTM2	9.007	9.084	9.197	9.078	9.128	9.103
TUBB8P2	6.929	6.971	6.890	6.808	6.932	6.882
OR2L13	4.005	3.865	3.950	3.754	3.941	3.697
FKBP2	9.814	9.878	10.051	9.846	9.822	9.637
OR7E162P	9.849	9.926	10.029	9.810	9.880	9.911
MYO18A	10.175	10.160	10.103	9.929	9.839	9.967
MYO18B	7.040	7.069	7.003	7.153	7.234	6.979
PRG4	4.650	4.481	4.268	4.568	4.349	4.565
PRG3	7.677	7.735	7.809	7.647	7.860	7.867
PRG2	6.651	6.687	6.598	6.657	6.805	6.719
FKBP8	10.021	9.994	10.257	10.151	10.382	10.027
TIMM23B	9.686	9.921	9.931	9.645	9.676	9.652
LINC00703	6.153	6.147	6.043	6.320	6.482	6.345
AP002364.1	4.751	4.475	4.443	4.697	4.551	4.586
AP002364.2	4.751	4.475	4.443	4.697	4.551	4.586
FAM133B	9.535	9.496	9.496	9.402	9.565	9.304
FAM133A	5.917	6.010	6.111	6.141	6.287	6.157
NCAPD2	9.651	9.434	9.470	9.036	9.172	9.018
NCAPD3	10.022	9.875	9.830	9.690	9.525	9.664
MIR4290HG	5.011	5.366	5.137	5.243	5.244	5.226
OR2L1P	6.113	6.211	6.177	5.927	6.144	6.105
ILF3-DT	7.690	7.690	7.613	7.097	7.280	7.248
AC093010.2	4.545	4.642	4.402	4.568	4.701	4.563
PNMA8B	7.630	7.651	7.703	7.718	7.597	7.784
PNMA8A	5.494	5.474	5.358	5.304	5.516	5.419
ZNF358	8.786	8.729	8.939	8.802	8.782	8.793
SOAT2	7.299	7.493	7.364	7.304	7.215	7.404
WDR93	8.385	8.302	8.301	8.437	8.596	8.601
SOAT1	10.409	10.505	10.523	10.131	10.251	10.421
SRGAP1	10.382	10.438	10.491	9.980	10.149	10.049
SRGAP3	7.500	7.465	7.345	7.303	7.304	7.259
SRGAP2	8.637	8.377	8.451	8.437	8.508	8.323
DLK1	6.187	6.251	6.252	6.711	6.722	6.536
DLK2	7.764	8.044	8.034	7.850	7.906	7.961
AC004812.2	7.946	7.773	7.805	7.990	7.815	7.867
SMC3	9.928	9.816	9.834	9.338	9.834	9.317
SMC2	8.999	9.005	8.973	8.536	8.406	8.530
SMC5	9.067	9.147	9.016	8.539	8.695	8.759
SMC4	11.275	11.216	11.271	10.856	10.856	11.082
PYROXD1	9.656	9.759	9.685	9.414	9.551	9.534
SMC6	8.743	8.758	8.880	8.630	8.613	8.700
STYX	8.306	8.294	8.251	7.742	7.805	7.885
SMCP	7.656	7.554	7.589	7.771	7.750	7.855
LTB	7.394	7.281	7.461	7.605	7.634	7.350
LTA	7.057	7.071	7.108	7.047	7.106	7.148
GRIPAP1	9.017	9.121	9.087	8.874	8.755	8.897
AL365184.2	5.848	5.860	5.939	5.940	6.002	5.960
LTF	7.732	7.797	7.681	8.014	7.853	7.828
LTK	7.667	7.489	7.711	7.748	7.733	7.699
IFNA10	8.082	8.266	7.972	7.982	8.096	8.166
LTV1	8.591	8.532	8.430	8.536	8.459	8.390
AL445989.1	5.352	5.471	5.265	5.284	5.424	5.506

AL445989.2	5.352	5.471	5.265	5.284	5.424	5.506
MARF1	8.889	8.780	8.844	8.015	8.264	8.471
ARL8B	11.034	11.101	11.173	10.967	10.852	10.999
ARL8A	9.125	9.268	9.516	9.178	9.334	9.190
LRATD2	11.754	11.841	11.853	11.733	11.747	11.685
C1orf68	7.609	7.621	7.639	7.816	7.990	7.693
LRATD1	7.925	7.934	8.028	7.737	8.019	7.901
TTC7A	8.191	8.407	8.338	8.310	8.234	8.243
C1orf61	8.607	8.738	8.708	8.827	8.752	8.860
GYG1	11.337	11.455	11.585	11.560	11.562	11.505
GYG2	8.336	8.352	8.426	8.224	8.464	8.342
TWIST1	5.517	5.326	5.367	5.327	5.332	5.213
LINC00174	12.764	12.952	13.000	12.813	12.829	12.952
TWIST2	5.625	5.528	5.564	5.825	5.741	5.890
LINC00173	7.291	7.264	7.211	7.235	7.255	7.412
HNF1B	11.843	11.807	12.020	11.875	11.821	11.727
AL137247.2	7.508	7.471	7.538	7.499	7.517	7.525
HNF1A	7.995	7.998	8.125	7.995	7.973	7.976
MPRIP-AS1	4.988	5.018	5.014	5.086	4.840	5.004
PDIK1L	6.932	7.008	7.065	6.813	6.799	6.919
AC119868.1	7.326	7.112	7.313	7.343	7.360	7.369
AL121929.3	7.392	7.499	7.252	7.485	7.436	7.728
Z83843.1	6.403	6.322	6.077	6.314	6.090	6.355
GNAI2	10.116	10.051	10.144	10.152	9.861	10.044
PDGFRA	6.282	6.134	6.222	6.498	6.364	6.433
PTK2B	7.743	7.643	7.872	7.601	7.754	7.604
AL031864.1	5.545	5.660	5.635	5.567	5.523	5.522
UBBP1	10.412	10.493	10.308	10.323	10.305	10.643
UBBP2	7.213	7.122	7.228	6.949	7.116	7.129
MKLN1	7.833	7.915	7.793	7.882	7.935	7.913
AC091925.1	5.318	5.502	5.455	5.855	5.563	5.500
PROM2	10.251	10.316	10.125	10.175	10.362	10.364
PROM1	11.254	11.222	11.404	10.987	10.870	11.116
LINC00824	7.632	7.707	7.582	7.417	7.885	7.846
SLC41A1	8.818	8.929	8.789	8.706	8.774	8.864
SLC41A3	10.726	10.794	10.905	10.542	10.610	10.529
SLC41A2	9.142	9.052	9.060	8.890	8.845	9.000
MIR6795	8.354	8.512	8.459	8.209	8.641	8.424
AC074351.1	7.873	7.842	7.898	7.994	7.928	7.843
CSF1	8.373	8.391	8.571	8.590	8.549	8.511
CSF2	7.189	7.380	7.022	7.175	7.391	7.401
CSF3	7.969	8.018	8.022	8.146	8.195	8.102
AC024896.1	6.113	5.975	6.112	6.226	6.035	6.335
CNTN2	7.624	7.621	7.580	7.772	7.787	7.748
CNTN3	4.201	4.102	4.005	4.101	3.902	3.937
CNTN1	6.844	6.939	6.699	6.891	7.122	7.069
CNTN6	4.256	4.316	4.235	4.369	4.284	4.368
CNTN4	6.258	6.270	6.226	6.234	6.102	6.200
CNTN5	5.058	5.234	5.141	5.101	5.094	4.878
CDC42BPG	7.829	7.796	7.715	7.862	7.910	7.704
CDC42BPA	9.554	9.468	9.495	9.369	9.445	9.426
CDC42BPB	10.393	10.399	10.525	10.150	10.101	10.281
TXNRD2	8.580	8.609	8.790	8.572	8.583	8.465
TXNRD3	9.072	9.126	9.178	8.714	8.714	8.789
TXNRD1	12.122	12.201	12.232	12.105	11.918	12.034
UBE2E1	11.237	11.287	11.389	11.243	11.185	11.296

UBE2E3	11.697	11.741	11.783	11.421	11.391	11.509
C3P1	8.590	8.415	8.418	8.237	8.240	8.391
CNGA1	8.170	8.112	8.199	8.099	8.038	7.990
CNGA3	4.865	4.956	4.747	5.236	5.428	5.192
CNGA4	5.300	5.396	5.711	5.524	5.596	5.388
MIR1306	7.937	7.857	7.905	7.915	7.913	7.841
AL031058.1	5.461	5.763	5.836	5.580	5.576	5.634
RPL7L1	10.946	10.950	10.993	10.763	10.561	10.747
MCM3	10.439	10.388	10.701	10.426	10.233	10.290
MCM2	11.609	11.694	11.832	11.508	11.459	11.438
CCDC47	11.004	11.070	10.992	10.941	10.762	10.932
CCDC43	10.032	10.030	10.085	9.642	9.805	9.639
CCDC42	6.004	6.044	5.944	6.071	6.054	5.977
CCDC40	7.374	7.313	7.349	7.486	7.501	7.425
AC007541.1	7.326	7.371	7.550	7.258	7.430	7.344
COASY	11.041	10.980	11.131	10.893	10.715	10.930
AP006547.1	8.156	8.154	8.174	8.317	8.333	8.430
CDKL5	8.567	8.466	8.422	8.026	8.036	8.290
CDKL1	6.668	6.391	6.582	6.661	6.771	6.829
CDKL3	7.186	7.113	6.816	7.302	7.173	7.207
CDKL2	6.957	6.969	7.006	7.273	7.120	7.087
FBXO22	8.981	9.170	9.304	9.223	9.091	9.114
FBXO21	11.408	11.494	11.493	11.250	11.495	11.003
FBXO27	8.353	8.499	8.618	8.293	8.657	8.351
MCM9	8.844	8.909	8.910	8.751	8.585	8.806
FBXO25	9.204	9.325	9.410	8.986	9.045	9.077
FBXO24	6.652	6.437	6.897	6.763	6.812	6.621
FBXO28	10.792	10.897	10.986	10.730	10.623	10.743
YBX1P10	13.364	13.413	13.539	13.346	13.247	13.374
AC099548.2	7.619	7.517	7.823	7.654	7.893	7.774
AIM2	5.454	5.576	5.447	5.848	5.733	5.699
AC087741.1	6.703	6.417	6.509	6.548	6.281	6.167
AL353997.8	7.848	7.650	7.758	8.001	7.965	7.991
AL353997.3	7.848	7.650	7.758	8.001	7.965	7.991
AL353997.2	5.967	5.882	6.127	6.072	6.128	6.050
UNC80	5.332	4.994	4.953	5.604	5.423	5.337
FAM107A	7.282	7.285	7.495	7.551	7.355	7.418
FAM107B	10.790	10.808	10.945	10.530	10.435	10.282
MCMBP	9.411	9.456	9.493	9.217	9.189	9.251
SRXN1	9.825	9.850	10.015	9.912	9.924	9.776
VWA8-AS1	4.104	4.301	4.121	4.639	4.427	4.222
PLPP3	10.069	10.075	10.155	10.339	10.357	9.999
AC092042.2	10.597	10.747	10.698	10.515	10.854	10.509
AL450472.1	6.903	6.977	6.730	7.325	6.956	6.821
IQGAP1	11.730	11.761	11.865	11.569	11.512	11.556
IQGAP2	9.332	9.091	9.126	8.715	8.809	8.778
IQGAP3	9.727	9.330	9.638	8.677	8.841	8.814
AC090921.1	4.698	4.898	4.908	4.773	4.786	4.815
XXYLT1	9.198	9.099	9.173	8.801	9.345	9.074
SMNDC1	9.502	9.512	9.445	9.214	9.334	9.318
ZG16B	7.715	7.778	7.856	7.905	7.765	7.780
PCMTD2	9.366	9.452	9.438	9.055	8.917	9.255
PCMTD1	8.751	8.812	8.797	8.507	8.519	8.792
TUBA4A	11.268	11.359	11.507	11.203	11.130	11.279
TUBA4B	8.022	8.065	7.933	7.868	8.047	7.948
AL360181.2	7.977	7.836	7.861	8.155	7.839	8.020

AL360181.3	8.944	9.060	9.195	9.058	8.924	9.058
FIBP	11.179	11.285	11.486	11.034	10.991	10.937
AC105219.5	6.804	6.950	6.863	6.808	6.797	6.983
LINC01704	6.999	7.101	6.886	6.808	7.088	6.952
LINC01706	5.154	5.225	5.172	5.228	5.259	5.223
LINC01700	8.417	8.496	8.313	8.406	8.507	8.471
LINC01703	6.845	6.901	7.203	6.791	6.944	6.992
POU2F2	8.260	8.142	8.337	8.382	8.395	8.385
POU2F3	8.231	8.390	8.408	8.307	8.523	8.318
AC027373.1	6.969	6.861	6.934	7.360	6.961	6.888
C1orf127	8.118	8.275	8.276	8.167	8.283	8.320
C1orf122	10.430	10.475	10.670	10.342	10.257	10.305
PCCB	10.047	10.073	10.150	9.967	9.921	9.894
OR10C1	6.529	6.445	6.472	6.560	6.815	6.834
PCCA	7.001	7.095	6.925	6.882	7.096	6.943
SIVA1	10.165	10.313	10.592	10.043	10.161	10.067
AC009159.3	7.957	7.925	7.787	8.175	7.954	7.991
ISL2	7.789	7.770	7.947	8.143	8.342	8.180
AL358075.4	8.008	8.101	8.058	7.598	7.797	7.958
THAP9-AS1	8.401	8.491	8.489	7.977	8.257	8.246
TOR1AIP2	9.997	9.956	9.974	9.760	9.814	9.885
TOR1AIP1	11.199	11.251	11.373	11.131	11.106	11.175
RNF185	9.369	9.411	9.600	9.321	9.356	9.161
RNF187	10.530	10.597	10.803	10.403	10.316	10.358
RNF186	7.131	7.274	7.308	7.284	7.205	7.393
RNF181	11.695	11.716	11.983	11.733	11.732	11.708
RNF180	4.473	4.301	4.382	4.599	4.145	4.287
RNF183	7.390	7.439	7.530	7.394	7.514	7.595
RNF182	8.359	8.404	8.509	8.215	8.200	8.208
BMPR2	9.836	9.839	9.895	9.522	9.389	9.728
ZBTB22	8.776	8.732	8.892	8.856	8.828	8.877
ZBTB20	8.896	8.940	8.798	8.774	8.831	8.857
ZBTB21	9.052	8.858	8.878	8.823	8.801	8.746
CLVS1	6.159	6.403	6.459	6.714	6.231	6.619
LINC01448	5.579	5.691	5.686	5.822	5.851	5.936
ZBTB24	8.325	8.316	8.238	8.103	7.963	8.185
CLVS2	5.164	5.305	5.370	5.057	5.215	4.959
KIFAP3	9.211	9.362	9.447	9.213	9.087	9.352
RPS2P5	14.545	14.545	14.561	14.532	14.532	14.573
EEF1E1-BLOC1S	7.326	7.326	7.423	7.353	7.216	7.239
UBE2F-SCLY	9.663	9.748	9.757	9.621	9.715	9.706
MS4A5	7.206	7.254	7.367	7.508	7.232	7.417
AL008628.2	6.122	6.224	6.193	6.316	6.265	6.265
AL008628.1	6.122	6.224	6.193	6.316	6.265	6.265
MS4A2	5.744	5.755	5.776	6.195	5.957	5.904
SYT12	7.167	7.267	7.619	7.354	7.234	7.221
SYT13	10.821	10.871	10.953	10.448	10.294	10.432
SYT11	6.746	6.372	6.411	6.596	6.699	6.722
SYT16	8.317	8.257	8.132	8.142	8.009	8.365
SYT17	8.270	8.234	8.410	8.218	8.113	8.203
SYT14	4.705	4.745	4.758	4.692	4.515	4.840
SYT15	9.453	9.439	9.344	9.561	9.621	9.575
HDAC2-AS2	7.463	7.553	7.004	7.515	7.411	7.528
AC007406.5	6.206	6.182	6.341	5.587	5.510	5.734
GPHN	8.994	9.161	9.093	8.871	8.982	8.990
GSTT4	7.447	7.617	7.613	7.242	7.700	7.578

MBIP	7.957	8.348	8.142	7.954	7.920	8.450
GSTT1	9.716	9.798	9.958	9.424	9.534	9.562
ATP6V1G3	5.489	5.254	5.492	5.506	5.888	5.575
ATP6V1G2	6.781	6.730	6.835	6.756	6.539	6.784
ATP6V1G1	11.861	11.826	11.907	11.785	11.777	11.721
LAMTOR4	8.560	8.594	8.889	8.668	8.356	8.684
LAMTOR5	10.768	10.840	11.039	10.854	10.674	10.981
LAMTOR1	11.261	11.356	11.466	11.358	11.286	11.282
LAMTOR2	9.650	9.730	10.064	9.602	9.678	9.651
LAMTOR3	9.414	9.426	9.540	9.431	9.475	9.405
AC133551.1	7.710	7.765	7.579	7.766	7.843	7.804
DDX31	8.605	8.708	8.696	8.648	8.548	8.506
LINC01816	6.969	7.015	7.156	7.256	7.326	7.136
STRN4	10.329	10.383	10.593	10.362	10.384	10.279
STRN3	9.041	9.077	9.155	8.923	9.057	9.115
SDR16C5	6.755	6.937	6.842	6.554	6.650	6.666
KDR	5.053	5.112	4.887	5.089	5.112	5.236
NDN	7.219	7.195	7.064	7.167	7.301	7.259
MVB12B	8.483	8.479	8.485	8.417	8.400	8.413
NDP	5.587	5.563	5.668	5.379	5.447	5.449
MVB12A	9.094	9.278	9.468	8.727	9.001	8.930
HSD11B1L	8.708	8.500	8.692	8.751	8.802	8.689
COL13A1	7.517	7.509	7.608	7.776	7.638	7.526
GPSM2	8.253	8.218	8.333	8.006	8.121	8.052
GPSM3	8.270	8.449	8.510	8.516	8.575	8.463
GPSM1	8.512	8.476	8.590	8.508	8.580	8.665
CSAG3	6.182	5.991	6.383	6.493	6.444	6.263
AIMP2	11.166	11.242	11.292	11.130	11.110	11.137
AIMP1	11.067	11.161	11.198	11.248	11.171	11.124
CBL	8.374	8.438	8.523	8.262	8.386	8.558
CYCS	13.090	13.181	13.174	13.245	13.190	13.207
AC087393.3	6.930	6.729	6.728	7.029	6.836	7.040
HSH2D	8.084	7.977	8.172	7.852	8.114	7.891
SOD3	9.043	9.072	9.200	9.211	9.241	8.991
GPX1	10.870	10.956	11.079	10.914	10.909	11.001
IGLV4-60	6.227	5.939	6.362	6.387	6.337	6.274
PRDM6	8.306	8.402	8.291	8.412	8.525	8.528
VPS33B	9.539	9.622	9.731	9.437	9.492	9.528
VPS33A	9.025	9.049	9.148	8.798	8.886	8.885
PDRG1	10.536	10.430	10.828	10.482	10.385	10.533
PRDM1	8.029	8.258	8.183	8.215	8.100	8.215
STAC2	9.274	9.151	9.330	9.512	9.429	9.326
SLC22A2	7.200	7.267	7.186	7.173	7.186	7.314
FAM47E-STBD1	7.942	8.123	8.364	8.165	8.011	8.113
SIRPB3P	7.773	7.592	7.661	7.538	7.724	7.799
AC009803.1	7.019	7.180	7.024	7.204	7.303	7.274
SLC22A8	7.219	7.087	7.022	7.240	6.979	7.055
PRDM9	6.001	5.827	5.952	6.074	6.284	6.189
TRAV34	4.942	5.051	4.851	5.020	4.897	4.989
TRAV30	7.222	7.184	6.961	7.136	7.230	7.222
OLAH	7.874	7.824	7.968	8.188	8.095	8.008
PICALM	10.668	10.781	10.823	10.636	10.420	10.629
UBE2QL1	5.608	5.655	5.686	5.625	5.658	5.718
HIPK1-AS1	6.062	6.072	6.009	6.139	6.028	5.906
CYP2B7P	11.859	11.821	11.840	11.319	11.236	11.239
AL583810.1	9.734	9.751	9.654	9.560	9.685	9.783

MRC1	6.150	5.997	5.967	5.958	6.213	6.105
CHML	8.819	8.995	8.837	7.957	8.280	8.436
TRMT13	7.397	7.353	7.261	7.116	7.104	7.356
ANAPC15	9.080	9.260	9.320	8.866	8.942	9.015
ANAPC16	11.195	11.233	11.317	11.200	11.118	11.211
ANAPC11	10.274	10.345	10.584	10.108	10.245	10.216
ANAPC10	8.143	8.253	8.481	7.757	8.034	7.947
ANAPC13	11.945	11.953	12.035	11.901	11.874	11.760
PRXL2B	9.875	9.914	10.079	9.588	9.628	9.732
PRXL2C	8.692	8.826	8.951	8.488	8.683	8.656
PRXL2A	11.154	11.169	11.259	10.933	10.947	10.934
MYC	10.838	10.928	11.057	11.098	10.849	10.936
MYB	6.523	6.938	6.998	6.619	6.910	6.570
SLC9B1P4	5.861	5.785	5.791	6.273	6.001	6.099
SLC9B1P3	5.861	5.785	5.791	6.273	6.001	6.099
SLC9B1P1	5.861	5.785	5.791	6.273	6.001	6.099
ALOX5	8.820	8.919	8.973	8.535	8.795	8.596
AC007546.2	7.224	7.207	7.181	7.365	7.187	7.364
TRMT1L	8.270	8.299	8.188	7.898	8.198	8.040
SHC4	6.971	7.025	7.122	6.855	7.020	6.960
SPC25	9.238	9.223	9.467	8.799	8.998	8.900
SPC24	7.793	7.754	7.948	7.386	7.460	7.417
METAP1	9.926	10.030	10.064	9.959	9.865	10.038
METAP2	10.908	10.986	11.039	10.895	10.850	10.964
MIR9-2	5.616	5.535	5.560	5.542	5.147	5.332
AC225613.1	6.624	6.679	7.071	6.635	6.748	6.671
AC091946.1	4.467	4.673	4.529	4.466	4.478	4.365
PILRA	8.587	8.366	8.369	8.426	8.358	8.640
PILRB	8.587	8.366	8.369	8.426	8.358	8.640
AC079385.2	6.054	6.484	6.318	6.469	6.210	6.282
TMEM170B	5.161	4.990	5.004	4.785	4.811	5.117
TMEM170A	10.045	9.988	10.058	9.754	9.749	9.829
RHNO1	10.062	10.059	10.231	9.432	9.415	9.481
SNORD113-3	4.631	4.602	4.514	4.673	4.641	4.441
HADH	9.140	9.212	9.371	9.072	8.924	9.206
MRAP2	6.831	6.985	6.874	6.857	6.798	6.891
AC001228.3	8.192	8.393	8.294	8.448	8.466	8.384
CCDC37-DT	7.606	7.776	7.595	7.859	7.736	7.673
MTRES1	8.003	8.099	8.331	8.118	8.324	8.182
VDR	8.168	8.300	8.238	8.036	8.162	8.304
AC005725.1	5.601	5.761	5.829	5.851	5.922	5.857
MAP3K20-AS1	4.800	5.127	4.916	5.259	5.137	5.094
TBC1D8	9.574	9.551	9.503	9.173	9.129	9.276
TBC1D9	9.767	9.841	9.866	9.768	9.687	9.745
AC090286.1	6.312	6.443	6.527	6.677	6.683	6.647
MRPS34	9.829	9.938	10.083	9.909	9.808	9.886
TBC1D2	8.180	8.144	8.375	8.383	8.109	8.090
MRPS36	9.721	9.820	9.815	9.644	9.944	9.678
MRPS31	9.237	9.387	9.674	9.074	9.347	8.722
MRPS30	9.883	10.009	9.973	9.825	9.833	9.833
MRPS33	10.115	10.234	10.417	10.363	10.256	10.262
TBC1D7	7.667	7.753	7.904	7.663	7.733	7.754
HSPA12B	7.226	7.421	7.452	7.643	7.726	7.502
MRTFA	8.537	8.299	8.327	8.599	8.183	8.427
MRTFB	9.706	9.593	9.689	9.249	9.156	9.388
HSPA12A	6.498	6.453	6.556	6.590	6.602	6.553

AC010300.1	6.972	7.141	6.995	6.957	6.867	7.004
SNUPN	9.324	9.428	9.596	9.390	9.337	9.426
WAPL	9.274	9.334	9.328	9.268	9.212	9.302
TPRA1	9.044	9.095	9.323	8.838	8.924	8.988
NRSN2	7.748	7.738	7.782	7.667	7.909	7.683
AMOT	6.286	6.164	6.152	6.112	6.238	6.113
LEKR1	5.746	5.708	5.695	5.742	5.883	5.705
AC005730.2	7.393	7.213	7.151	7.334	7.420	7.308
FRMD4A	7.821	7.873	7.798	7.620	7.914	7.813
FRMD4B	7.793	7.690	7.867	7.666	7.917	7.845
TRPA1	6.965	6.995	6.796	6.990	6.849	6.972
LINC02415	4.982	5.255	5.063	5.150	5.253	5.211
SNORD38A	13.214	13.233	13.392	13.147	13.175	13.114
ARVCF	8.891	8.865	9.089	9.231	9.206	9.176
LSM12	10.437	10.454	10.579	10.445	10.412	10.303
LINC02419	5.187	5.077	4.975	5.425	5.279	5.119
LSM10	9.410	9.417	9.613	9.631	9.452	9.523
LSM11	7.837	7.867	7.850	7.777	7.722	7.977
AC109347.1	5.797	5.735	5.501	6.373	6.068	5.966
AC004997.1	8.407	8.327	8.459	8.292	8.415	8.246
TBX5-AS1	4.764	5.069	5.053	5.189	4.962	4.827
AC012005.2	6.582	6.672	6.703	6.718	6.700	6.647
AC012005.1	14.596	14.604	14.644	14.671	14.651	14.656
PRPF38A	9.778	9.925	9.920	9.706	9.656	9.699
PRPF38B	8.455	8.419	8.261	8.074	8.207	8.324
AC004987.2	7.751	7.444	7.692	8.078	8.064	7.811
AC138932.3	7.182	7.381	7.328	7.421	7.457	7.500
AC138932.4	10.412	10.613	10.670	10.443	10.511	10.502
SHFL	10.766	10.780	10.874	10.728	10.724	10.656
AC027279.1	5.253	5.097	5.349	5.156	5.200	5.126
AC027279.3	5.773	5.688	5.822	5.863	6.158	5.992
AC027279.4	5.580	5.580	5.609	5.679	5.694	5.761
ALDH9A1	11.077	10.993	10.934	10.744	10.813	10.860
GPM6A	7.715	7.862	7.499	7.957	8.117	8.045
FABP3P2	6.762	6.850	6.934	7.086	6.986	6.781
GPM6B	6.701	6.725	6.724	6.490	6.697	6.776
LINC02186	5.078	5.508	5.128	5.362	5.533	5.740
AC104687.2	7.087	7.263	6.996	7.077	7.084	7.173
AL390860.1	8.240	8.105	8.124	8.388	8.256	8.342
LINC01146	4.428	4.531	4.299	4.816	4.502	4.517
KIF25-AS1	8.032	7.921	7.795	8.110	8.141	7.982
LINC01144	7.526	7.543	7.633	7.785	7.787	7.771
LINC01142	6.570	6.384	6.428	6.507	6.746	6.429
LINC01141	7.312	7.335	7.174	7.315	7.351	7.264
LINC01140	7.463	7.593	7.276	7.522	7.630	7.478
OR7D2	5.566	5.515	5.470	5.570	5.591	5.632
LINC01149	4.521	4.745	4.541	4.466	4.514	4.494
LINC01148	5.278	5.245	4.916	5.044	5.455	5.516
DYSF	7.450	7.585	7.655	7.389	7.552	7.499
AL049795.2	9.460	9.638	9.598	9.025	9.497	9.301
AC130456.7	5.882	5.659	5.629	5.548	5.558	5.874
GATA6	9.581	9.609	9.639	9.365	9.422	9.467
GATA4	8.160	8.453	8.299	8.344	8.536	8.408
GATA5	5.597	5.573	5.529	5.853	5.819	5.670
GATA2	8.115	8.211	8.099	8.163	8.327	8.158
GATA3	9.363	9.376	9.440	9.328	9.427	9.273

GATA1	8.416	8.520	8.280	8.455	8.432	8.348
AL512624.2	5.279	5.017	5.088	5.269	5.142	5.236
CCND1	11.462	11.583	11.587	11.295	11.082	11.456
CCND3	10.728	10.851	10.955	10.531	10.496	10.597
CCND2	6.159	6.283	6.203	6.171	6.259	6.535
MIR4668	11.478	11.546	11.653	11.580	11.801	11.305
RPS27L	11.432	11.452	11.537	11.458	11.271	11.579
RPS27A	13.293	13.335	13.375	13.366	13.329	13.396
AC016027.4	6.938	6.891	7.073	6.862	6.958	6.899
OR51B5	7.666	7.616	7.527	7.668	7.737	7.784
AL109810.2	7.122	7.236	6.993	7.157	7.214	7.151
CXXC1P1	6.573	6.405	6.500	6.597	6.644	6.713
AC106872.12	9.763	9.664	9.559	9.555	9.749	9.782
RPL10P10	6.427	6.478	6.217	6.136	6.241	6.367
RPL10P16	13.794	13.873	13.940	13.883	13.836	13.884
RPL10P17	6.896	6.975	6.911	6.984	6.909	6.781
AC090136.3	4.362	4.251	4.366	4.471	4.615	4.465
DPCD	9.603	9.626	9.758	9.859	9.785	9.655
HNRNPCL1	7.849	7.817	7.775	7.929	7.877	7.875
AC139100.1	5.587	5.787	5.573	6.207	6.019	6.082
INSIG2	10.600	10.645	10.673	10.530	10.371	10.622
INSIG1	11.159	11.279	11.413	11.260	11.228	11.236
PAFAH2	7.655	7.859	7.908	7.899	8.071	7.938
ABCA8	5.942	6.141	5.960	6.381	6.170	6.132
AC018512.1	7.744	7.884	7.835	8.132	8.071	8.007
ABCA9	6.659	6.640	6.406	6.713	6.658	6.698
LINC00421	6.938	6.920	6.689	6.892	6.937	6.932
LINC00427	7.292	7.426	7.389	7.631	7.458	7.428
TAF13	9.275	9.314	9.209	9.039	9.018	9.212
TAF12	8.754	8.722	8.793	8.506	8.463	8.502
TAF11	8.274	8.284	8.384	8.112	8.176	8.085
TAF10	12.304	12.367	12.561	12.612	12.530	12.410
TAF15	9.154	9.282	9.440	9.462	8.993	8.790
FH	10.206	10.251	10.435	10.059	10.050	10.197
NDUFV3	9.362	9.477	9.515	9.279	9.255	9.314
NDUFV2	11.142	11.224	11.372	11.121	10.999	11.129
NDUFV1	10.998	11.167	11.327	10.888	10.793	10.773
LAMA1	5.141	5.046	4.980	4.963	4.964	5.059
LAMA2	6.792	6.929	6.966	7.125	6.914	6.943
LAMA3	10.357	10.506	10.461	10.232	10.069	10.162
LAMA4	8.891	8.897	8.882	8.367	8.472	8.647
LAMA5	11.197	11.175	11.207	11.007	10.856	11.053
SLC25A43	8.768	8.880	8.789	8.618	8.711	8.657
SLC25A45	7.486	7.417	7.392	7.626	7.583	7.298
TAF1C	8.413	8.513	8.588	8.214	8.344	8.447
TAF1B	7.748	7.548	7.689	7.601	7.784	7.631
F2	7.584	7.733	7.951	8.340	7.976	7.975
F3	12.136	12.219	12.218	12.358	12.345	12.319
F5	9.464	9.240	9.298	9.128	9.328	9.085
POC1B-GALNT4	10.314	10.468	10.328	10.236	10.069	10.370
F7	7.173	7.220	6.981	7.338	7.168	7.151
F8	7.299	7.570	7.589	7.334	7.570	7.312
F9	5.519	5.413	5.157	5.408	5.422	5.438
AC002310.1	5.735	5.842	5.833	5.716	5.704	5.877
TAF1L	8.017	7.841	7.390	7.816	7.777	7.771
AC004832.3	9.784	9.767	9.982	9.734	9.683	9.699

AC004832.1	6.743	6.677	6.838	6.964	6.875	6.832
HTR3B	6.851	6.398	6.558	6.902	6.904	6.800
AC008429.1	5.880	6.078	5.878	6.129	6.262	6.182
HTR3A	7.558	7.565	7.418	7.514	7.642	7.384
AC007969.1	13.953	13.944	14.039	13.928	13.758	13.841
FCAMR	5.892	5.921	5.787	6.005	5.960	5.967
HS2ST1	10.025	10.124	10.046	9.808	9.807	9.933
BAALC	6.913	6.815	6.562	6.957	6.907	6.880
AC106028.3	6.062	6.298	6.453	6.492	6.682	6.557
DYNLRB2	5.065	5.205	5.110	5.345	5.641	5.241
DYNLRB1	12.454	12.526	12.685	12.494	12.468	12.459
ZNRD1ASP	5.977	6.002	5.903	5.711	5.921	6.015
OR2F2	5.925	6.014	5.987	6.161	6.164	6.147
ABO	7.555	7.652	7.729	7.611	7.588	7.847
ABR	9.703	9.694	9.832	9.560	9.491	9.615
ATP4A	7.244	7.570	7.372	7.594	7.503	7.607
ATP4B	5.830	6.078	5.859	5.990	5.782	5.874
FAM228B	7.568	7.680	7.648	7.778	7.765	7.710
FAM228A	5.333	5.382	5.291	5.671	5.621	5.675
ARAF	10.803	10.802	10.947	10.662	10.728	10.794
ATP8A2P3	4.169	4.065	4.482	4.256	4.260	4.287
SERTAD4	9.042	9.113	9.103	8.808	9.059	8.667
TEPP	5.838	6.032	5.887	6.085	5.958	6.104
AC021269.3	4.094	4.296	4.196	4.227	4.330	4.429
ZNF705G	5.512	5.528	5.370	5.395	5.531	5.771
ZNF705B	5.512	5.528	5.370	5.395	5.531	5.771
ZNF705A	5.512	5.528	5.370	5.395	5.531	5.771
TEP1	7.650	7.872	7.777	7.299	7.613	7.673
NR2F2-AS1	7.346	7.335	7.406	7.245	7.268	7.247
CYP2A7P1	5.521	5.896	5.699	6.210	5.734	5.645
TEX261	11.148	11.090	11.187	10.939	10.947	10.946
TEX264	9.014	9.186	9.376	9.085	8.976	9.031
Z83847.1	7.270	7.063	7.178	7.062	7.203	7.189
SAMD9L	9.175	9.165	9.045	9.095	9.405	9.100
ANKRD20A5P	8.236	8.156	8.141	8.146	8.146	8.231
C1QC	6.849	6.670	6.878	7.181	7.085	6.891
C1QB	6.303	6.384	6.564	6.471	6.174	6.525
C1QA	6.953	7.359	6.997	7.354	7.089	7.209
PKHD1	8.249	8.227	8.145	8.122	7.996	8.220
LINC02355	7.440	7.233	7.427	7.216	7.328	7.566
LINC02353	4.443	4.375	4.398	4.587	4.569	4.652
LINC02352	8.263	8.325	8.558	8.655	8.525	8.414
VSIG4	7.373	7.001	7.354	7.289	7.491	7.076
VSIG2	7.058	7.153	7.216	7.153	7.145	7.036
VSIG1	9.183	9.038	8.978	9.053	9.243	8.836
SHPRH	7.072	7.116	7.011	6.303	6.213	7.018
ADORA2A	6.318	6.558	6.534	6.708	6.499	6.555
ADORA2B	9.428	9.529	9.563	9.452	9.453	9.529
NKAP	8.952	8.896	9.118	8.721	8.817	8.844
RBFADN	5.299	5.281	5.227	5.873	5.329	5.334
CSK	10.806	10.901	11.040	10.763	10.843	10.897
CNOT11	10.953	11.027	11.250	10.996	11.026	11.029
CNOT10	7.869	7.964	8.074	7.625	7.488	7.776
SSPO	6.886	6.701	6.876	6.887	7.032	7.018
TRIM49B	5.312	5.102	5.249	5.279	5.179	5.099
SINHCAFP2	9.891	9.903	10.113	9.812	10.131	9.607

CD74	11.323	11.351	11.491	11.493	11.525	11.290
CD70	8.161	8.233	8.374	8.523	8.469	8.305
CD72	6.714	6.581	6.684	6.869	6.714	6.870
CLOCK	8.857	9.040	8.952	8.947	8.969	9.029
SH3BP4	10.176	10.226	10.339	10.267	10.246	10.125
SH3BP5	7.916	7.979	8.181	7.764	7.849	7.828
SH3BP1	8.193	8.267	8.434	8.510	8.529	8.483
SH3BP2	8.853	8.896	8.863	8.664	8.936	9.011
AL354861.3	6.683	6.870	6.782	6.834	6.820	6.896
FASTKD5	8.700	8.698	8.782	8.538	8.618	8.610
FASTKD2	9.290	9.388	9.485	9.455	9.476	9.500
FASTKD3	7.788	7.836	7.786	7.701	7.828	7.932
FASTKD1	8.555	8.568	8.624	8.822	8.751	8.834
CYB5D1	8.531	8.730	8.607	8.369	8.581	8.509
CYB5D2	8.036	7.994	7.931	7.820	7.814	7.952
SRP72	10.390	10.448	10.508	10.301	10.351	10.338
MAPK7	8.435	8.521	8.510	8.813	8.563	8.640
MAPK4	5.636	5.594	5.618	5.651	5.919	5.684
PAF1	8.999	9.019	9.188	8.841	8.732	8.786
KIR2DP1	8.115	8.042	7.807	8.213	8.188	8.126
SPATC1	5.412	5.436	5.380	5.227	5.346	5.458
MILR1	6.350	6.351	6.290	6.194	6.071	6.322
AATK	8.837	8.721	9.001	8.863	8.862	8.910
RBBP4P1	10.858	10.991	11.137	11.025	10.960	10.911
NTIA01000060.1	9.381	9.305	9.474	9.304	9.506	9.391
ESPNP	4.826	4.967	4.870	4.905	5.060	5.056
MYOCD-AS1	5.486	5.699	6.067	6.114	5.770	5.914
GNB1L	8.974	9.014	9.280	9.062	9.012	9.161
UNC119	11.047	11.177	11.232	10.965	10.863	10.781
OR4F7P	7.745	7.729	7.361	7.729	7.847	7.903
TMEM202-AS1	7.207	7.335	7.589	7.452	7.496	7.494
KCND1	8.025	8.205	8.128	8.109	8.128	8.067
MFSD8	8.082	8.083	8.126	8.135	8.105	8.173
KCND3	6.550	6.687	6.776	7.008	6.771	6.862
KCND2	6.039	5.993	6.124	5.846	5.665	5.764
CCHCR1	9.052	9.041	9.018	8.824	8.871	8.897
C2orf27AP3	7.972	7.912	8.183	8.328	8.259	8.126
MFSD1	12.440	12.589	12.642	12.527	12.353	12.460
MFSD3	8.972	8.967	9.225	8.742	8.768	8.778
MFSD5	9.513	9.591	9.593	9.380	9.411	9.427
MFSD6	10.394	10.408	10.409	10.230	10.182	10.212
TAMM41	7.400	7.308	7.423	7.446	7.435	7.465
MAP6D1	8.113	7.997	8.187	8.259	8.179	8.146
GAPDHP62	8.384	8.426	8.267	8.106	8.471	8.335
NCDN	9.574	9.704	9.717	9.412	9.508	9.379
KAT6B	9.111	8.960	9.039	8.895	9.103	8.883
KAT6A	10.402	10.251	10.327	10.456	10.331	10.419
AL450442.1	5.135	4.923	4.942	5.009	5.122	5.155
UBN2	7.780	7.600	7.590	7.715	7.869	7.554
CASTOR1	8.407	8.327	8.459	8.292	8.415	8.246
CASTOR3	6.283	6.486	6.422	6.663	6.363	6.367
CASTOR2	8.276	8.328	8.446	8.536	8.362	8.437
TCAM1P	4.569	4.638	4.289	4.916	4.726	4.395
BHLHE40	10.808	10.867	10.855	11.071	10.981	11.034
BHLHE41	9.896	9.974	9.869	9.633	9.657	9.685
OR8G1	6.803	6.817	6.569	6.730	6.941	6.845

MAGI3	7.982	7.985	7.972	7.867	7.726	7.905
MAGI2	6.571	6.496	6.657	6.526	6.610	6.612
MAGI1	9.112	9.209	9.300	9.125	9.064	8.886
TMEM185AP1	8.836	9.009	9.044	8.708	8.717	8.784
UGT2B4	4.978	5.249	4.953	4.960	5.143	5.003
NGEF	8.057	7.989	8.101	7.616	7.982	7.845
ST7-OT4	4.618	4.379	4.225	4.368	4.621	4.495
GAPT	4.666	4.574	4.561	4.614	4.542	4.531
MAGIX	8.758	8.880	8.934	8.794	8.854	8.708
CCL16	6.913	6.947	6.851	6.990	7.104	7.033
BST1	5.624	5.481	5.688	5.529	5.742	5.641
BST2	8.376	8.320	8.204	8.241	8.279	8.163
TRIM64DP	4.161	4.224	4.180	4.164	4.199	4.288
CARM1	8.255	8.282	8.357	8.194	8.256	8.352
KRTAP7-1	6.536	6.621	6.553	6.491	6.840	6.658
STAT5B	9.372	9.508	9.095	9.256	9.398	9.499
STAT5A	7.874	7.828	7.908	7.707	7.932	7.863
AC010619.3	5.581	5.628	5.710	5.492	5.709	5.729
AC010619.2	5.886	5.679	5.603	5.823	6.232	6.093
SDHAP2	10.438	10.447	10.431	10.273	10.280	10.415
SDHAP3	9.773	9.876	9.899	9.644	9.566	9.763
SDHAP1	10.438	10.447	10.431	10.273	10.280	10.415
SMTNL1	7.284	7.343	7.292	7.589	7.335	7.360
SMTNL2	6.053	5.930	5.910	6.479	6.168	6.184
GU182351.3	8.835	8.689	8.488	8.744	8.793	8.879
IMPG2	5.826	6.048	5.967	5.802	5.744	5.763
IMPG1	3.905	4.213	3.926	4.057	3.944	4.143
VWA5A	8.063	8.130	8.017	8.018	8.216	8.135
CARMN	7.841	7.726	8.062	7.956	7.825	7.930
AL031598.1	7.134	7.253	7.320	7.660	7.511	7.321
AC245033.1	14.164	14.198	14.199	14.193	14.160	14.234
TNIK	8.758	8.681	8.783	8.634	8.806	8.687
SERPING1	9.456	9.305	9.482	9.060	9.064	8.946
AL663093.2	8.439	8.433	8.212	8.323	8.555	8.407
AC079248.3	7.099	7.224	6.970	7.103	7.228	7.099
GCSAM	6.637	6.605	6.461	6.598	6.426	6.653
OR10A4	6.376	6.675	6.559	6.449	6.517	6.852
ID2-AS1	7.669	7.785	7.741	7.452	7.606	7.585
ZNF267	8.355	8.280	8.309	8.138	8.237	8.473
AC004805.1	7.586	7.761	7.777	7.847	7.802	7.848
MARCKSL1	12.823	12.865	12.962	12.785	12.832	12.722
LY6E	11.572	11.680	11.942	11.684	11.818	11.633
LY6D	7.189	7.091	7.126	7.432	7.051	6.911
RPL7P52	11.980	12.036	12.035	12.063	12.028	12.106
AC090181.3	6.764	6.802	6.377	6.400	6.608	6.232
AL139260.3	9.431	9.659	9.711	9.592	9.582	9.639
LY6K	7.019	6.960	6.935	6.986	7.221	7.205
LY6H	5.803	5.574	5.711	5.999	5.917	5.779
OR51M1	7.092	7.369	7.154	7.342	7.416	7.471
OCLN	11.143	11.148	11.163	10.988	11.059	10.962
TYW5	8.096	8.194	8.164	8.157	8.296	8.073
OCLM	7.057	7.034	6.958	7.164	7.176	7.097
EBNA1BP2	9.787	9.739	9.977	9.513	9.577	9.538
TYW3	9.533	9.452	9.578	9.674	9.625	9.521
GPR37L1	6.885	7.041	7.123	7.205	7.120	7.067
AL353194.1	7.065	7.124	7.095	6.937	7.081	7.140

CFC1	7.590	7.407	7.658	7.744	7.615	7.552
AC108488.2	9.392	9.435	9.403	9.235	9.271	9.154
SPATA22	8.237	8.181	8.003	8.539	8.271	8.376
SPATA20	10.539	10.637	10.814	10.310	10.237	10.374
SPATA21	6.998	7.003	7.143	6.995	7.002	7.272
SPATA24	11.637	11.679	11.203	11.508	11.640	11.566
SPATA25	6.024	6.345	6.643	7.170	6.576	6.595
MCUR1	9.257	9.222	9.370	8.736	8.831	8.850
AC016687.2	3.882	3.758	3.960	3.991	3.839	3.892
AC016687.3	3.762	3.907	3.814	4.123	3.748	3.832
WAKMAR2	5.039	4.977	4.940	4.778	5.033	5.094
C2orf72	8.298	8.384	8.345	8.154	8.161	8.097
C2orf73	6.031	5.847	5.678	6.070	6.120	5.836
PCP4L1	5.815	5.799	5.874	5.955	6.097	5.941
C2orf76	6.659	6.764	6.960	6.780	7.128	7.115
SERPINA1	11.949	12.046	12.154	12.054	12.236	12.133
AC009113.1	6.561	6.468	6.542	6.298	6.164	6.292
SNHG20	5.418	5.342	5.388	5.041	5.096	5.736
SNHG21	7.259	7.010	6.981	6.784	6.928	7.209
SNHG22	6.271	6.333	6.470	6.193	6.295	6.507
SNHG26	5.234	5.201	5.033	5.329	5.163	5.175
SNHG27	4.236	4.246	4.208	3.994	4.382	4.167
SNHG28	7.326	7.572	7.541	7.471	7.667	7.502
SNHG29	11.440	11.575	11.802	11.951	12.180	11.982
EXOC6B	8.570	8.585	8.685	8.526	8.627	8.401
CYC1	11.716	11.773	12.008	11.703	11.648	11.643
SPATA2L	8.634	8.861	8.920	8.863	8.877	8.660
NRAS	9.838	10.018	10.192	10.046	9.728	9.883
AC006557.1	6.292	6.387	6.310	6.382	6.466	6.554
AC006557.6	9.608	9.556	9.575	9.852	9.888	9.741
AL671859.3	9.231	9.374	9.436	9.462	9.438	9.339
SNORA3A	13.836	13.822	13.906	13.931	13.893	13.957
AC007092.1	4.295	4.086	4.243	4.255	4.131	4.192
CYP39A1	7.485	7.620	7.651	7.646	7.586	7.764
CRYZL2P-SEC16	7.288	7.327	7.251	7.332	7.090	7.210
SCAMP4	9.559	9.609	9.798	9.864	9.924	9.807
SCAMP5	9.143	9.204	9.393	9.078	9.062	9.147
SCAMP2	10.901	10.971	11.112	11.136	11.255	10.848
SCAMP3	10.482	10.519	10.725	10.436	10.364	10.440
WAC-AS1	10.033	10.054	10.146	9.838	9.952	9.972
SCAMP1	10.320	10.497	10.466	10.339	10.516	10.415
RTL3	6.992	7.121	7.064	7.027	7.072	7.138
RTL6	10.639	10.680	10.748	10.659	10.528	10.525
CRYBB2P1	7.727	7.657	7.822	7.543	7.478	7.646
TSPAN2	6.196	6.021	6.125	6.205	6.026	6.232
TSPAN3	13.405	13.479	13.483	13.352	13.344	13.350
TSPAN1	12.371	12.488	12.576	12.499	12.417	12.428
TSPAN6	11.415	11.458	11.572	11.400	11.359	11.472
TSPAN7	6.667	6.830	6.579	6.760	6.801	6.980
TSPAN4	9.606	9.779	9.837	9.376	9.531	9.452
TSPAN5	8.571	8.719	8.665	8.336	8.322	8.385
TSPAN8	12.921	13.031	13.128	13.000	12.982	12.920
TSPAN9	8.457	8.524	8.678	8.407	8.256	8.610
ZNF444	9.719	9.761	9.933	9.513	9.597	9.494
ZNF445	8.708	8.920	9.066	8.923	9.063	8.919
ZNF446	9.473	9.501	9.679	9.539	9.530	9.446

ZNF440	5.169	5.109	5.236	4.876	5.169	5.060
ZNF441	4.620	4.599	4.590	4.589	4.797	4.515
ZNF442	5.235	5.078	5.111	5.434	5.183	5.343
ZNF443	8.435	8.539	8.568	8.424	8.517	8.527
ZNF449	7.511	7.584	7.538	7.541	7.535	7.562
L3MBTL1	8.509	8.487	8.668	8.804	8.684	8.591
L3MBTL3	6.960	6.946	6.643	6.773	6.799	6.649
L3MBTL2	10.202	10.116	10.219	10.367	10.288	10.280
L3MBTL4	7.087	6.932	6.595	7.121	7.070	7.167
SERAC1	7.109	7.245	7.448	6.972	7.057	6.955
CCDC148-AS1	5.105	5.225	5.233	5.373	5.362	5.162
AC092354.2	4.621	4.609	4.752	4.618	4.550	4.642
AC092354.1	4.621	4.609	4.752	4.618	4.550	4.642
BBOX1-AS1	6.017	6.012	6.093	6.142	6.177	6.122
RASD2	7.283	7.294	7.421	7.529	7.636	7.626
RASD1	7.871	7.754	7.777	8.151	8.045	8.147
PSENEN	10.499	10.454	10.684	10.436	10.575	10.366
AC016747.4	8.162	8.126	8.265	7.834	7.758	7.998
AC016747.1	8.560	8.775	8.989	8.604	8.690	8.295
TRABD	9.186	9.251	9.486	9.149	9.166	9.230
THBS4	8.118	8.174	8.123	8.186	8.158	8.018
THBS2	6.753	6.690	6.581	6.801	6.825	6.785
THBS3	8.634	8.567	8.774	8.607	8.562	8.675
THBS1	8.367	8.200	8.195	8.392	8.450	8.456
MEOX2	3.803	3.863	3.754	3.681	3.700	3.672
MYL10	8.468	8.360	8.291	8.823	8.638	8.588
DSTN	11.704	11.783	11.884	11.781	11.693	11.759
TTC28-AS1	7.057	7.256	7.141	7.365	7.224	7.164
AP1G1	11.038	10.969	11.085	10.881	10.806	10.689
AP1G2	9.923	9.829	9.963	9.559	9.531	9.776
LINC02624	6.118	6.118	6.102	6.139	6.010	6.188
LINC02622	6.735	6.562	6.527	6.613	6.412	6.574
LINC02620	5.329	5.444	5.197	5.339	5.352	5.409
BUD31	9.787	9.797	10.005	9.815	9.809	9.771
DLGAP2-AS1	7.285	7.469	7.416	7.560	7.631	7.587
DDIAS	7.829	7.872	8.117	7.618	7.843	7.765
RPE65	3.916	3.929	3.754	4.236	3.933	3.846
XKRY	4.480	4.377	4.275	4.476	4.624	4.561
XKRX	7.074	6.995	7.161	7.255	7.292	7.265
AC007128.1	8.123	8.172	8.247	8.058	8.163	8.043
LINC00668	8.604	8.523	8.408	8.671	8.753	8.765
LINC00665	6.483	6.280	6.139	6.372	6.524	6.371
LINC00664	5.870	5.706	5.698	5.943	5.853	6.038
LINC00667	9.152	9.231	9.207	8.939	9.085	8.929
EWSAT1	7.785	8.101	7.940	7.683	8.071	8.053
LINC00661	6.982	6.910	7.018	6.747	7.054	6.933
LINC00663	5.724	5.952	5.656	6.195	5.951	5.832
LINC00662	7.514	7.487	7.708	7.471	7.566	7.466
DERPC	11.422	11.533	11.696	11.388	11.414	11.459
GRAP	7.602	7.619	7.742	7.633	7.670	7.595
NPVF	4.763	4.772	4.604	4.759	4.759	4.809
HMGB3P19	5.837	5.712	5.875	5.837	5.980	5.930
H2BC1	5.038	5.088	4.888	5.081	5.124	4.932
H2BC3	5.963	6.051	5.961	6.080	6.198	6.256
H2BC4	7.525	7.668	7.704	7.298	7.788	7.410
H2BC5	10.433	10.445	10.552	10.685	10.625	10.628

H2BC6	10.433	10.445	10.552	10.685	10.625	10.628
H2BC7	9.086	9.206	9.224	9.362	9.226	9.281
H2BC8	7.160	7.415	7.430	8.236	7.961	8.365
H2BC9	8.901	9.028	8.966	9.093	9.056	9.098
XKR4	4.663	4.834	4.706	5.025	5.041	5.024
XKR7	7.141	6.925	6.868	7.053	6.904	6.873
XKR6	6.995	6.946	6.983	6.850	7.175	7.061
H1-10	11.087	11.188	11.367	11.017	11.020	10.845
XKR8	9.786	9.896	9.971	9.763	9.830	9.895
AC117490.2	4.840	4.929	5.014	4.893	4.997	5.097
HSP90AA1	13.979	13.895	13.881	13.929	13.961	13.917
PALD1	7.052	7.187	6.872	6.990	6.832	7.100
SCP2	11.032	11.076	11.167	11.004	10.887	11.021
CALML3-AS1	4.171	4.153	4.212	4.102	4.144	4.098
NNAT	8.321	8.305	8.553	8.457	8.306	8.493
IQCJ-SCHIP1	8.267	8.417	8.572	8.161	8.496	8.409
SSU72P8	6.755	6.768	6.688	6.690	6.842	6.851
RAB37	6.845	6.852	7.058	6.688	6.835	6.918
RAB36	8.080	8.104	8.147	7.757	8.050	7.981
RAB35	10.224	10.549	10.721	10.555	10.434	10.387
RAB34	9.581	9.619	9.855	9.545	9.467	9.524
HJURP	9.669	9.806	9.807	9.209	9.355	9.238
RAB32	10.030	10.162	10.204	10.250	10.262	10.414
RAB31	6.917	7.052	7.104	6.759	6.997	6.957
RAB30	7.110	6.960	6.822	7.202	7.205	7.108
RAB38	9.028	9.095	9.157	8.903	9.053	8.922
DOK2	8.041	8.046	7.850	8.026	8.248	8.187
DOK3	7.171	7.262	7.097	7.068	7.176	7.317
DOK1	7.933	8.038	8.133	8.222	7.993	7.991
DOK6	6.973	6.970	6.907	7.285	7.020	7.003
DOK7	7.417	7.624	7.652	7.502	7.585	7.741
DOK4	8.752	8.855	9.110	8.986	8.976	8.912
DOK5	7.788	7.619	7.312	7.476	7.527	7.581
AC010186.4	6.790	6.727	6.829	6.296	6.680	6.656
AC010186.2	6.790	6.727	6.829	6.296	6.680	6.656
AC010186.3	6.763	6.799	6.661	6.567	6.952	6.726
CR759763.2	9.231	9.374	9.436	9.462	9.438	9.339
RAB3D	8.466	8.468	8.743	8.613	8.495	8.599
RAB3C	5.710	5.970	5.766	5.945	5.931	5.953
RAB3B	8.067	7.900	7.788	8.073	7.979	7.878
RAB3A	7.294	7.241	7.399	7.387	7.404	7.091
ARMC9	7.441	7.429	7.343	7.205	7.519	7.301
ARMC6	8.974	9.115	9.169	9.269	9.024	9.102
ARMC7	8.597	8.691	8.815	8.744	8.658	8.727
LRG1	9.773	9.757	9.883	9.817	9.760	9.906
ARMC5	8.471	8.303	8.457	8.663	8.439	8.330
ARMC2	4.598	4.580	4.242	4.533	4.580	4.728
ARMC3	4.988	5.012	5.098	5.028	4.968	5.190
ARMC1	9.525	9.409	9.603	9.563	9.503	9.563
PDCD6IP	10.791	10.840	10.831	10.767	10.724	10.757
ZDHHC8P1	6.906	7.164	7.013	7.005	6.856	7.022
GDNF	6.843	6.732	6.837	6.726	6.964	7.013
ACTL6B	6.962	6.955	6.744	6.973	6.828	6.915
EIF5	12.367	12.394	12.497	12.704	12.556	12.449
WNT16	7.612	7.903	7.849	7.583	7.971	7.662
WNT11	7.564	7.509	7.702	7.871	8.025	7.781

RANGRF	8.864	8.902	9.081	8.633	8.842	8.671
HSPA2	7.787	7.700	7.926	7.180	7.272	7.412
HSPA7	7.321	7.321	7.391	8.592	8.844	9.142
HSPA6	7.321	7.321	7.391	8.592	8.844	9.142
HSPA5	11.792	11.865	11.925	11.790	11.867	11.827
HSPA4	10.805	10.970	10.899	10.965	10.964	10.997
HSPA9	12.003	12.014	12.143	11.716	11.648	11.670
HSPA8	13.795	13.904	13.879	13.960	13.927	14.010
GLTPP1	9.574	9.697	9.976	9.702	9.988	9.266
LIFR	7.595	7.527	7.665	7.397	7.727	7.487
AC106793.1	5.516	5.814	5.792	5.938	5.703	5.746
DANCR	10.585	10.697	10.794	10.717	10.645	10.840
AC087235.1	4.596	4.597	4.691	4.528	4.642	4.736
PPP2R2D	8.175	8.204	8.454	8.299	8.244	8.277
PPP2R2A	10.544	10.598	10.749	10.599	10.679	10.672
PPP2R2C	8.458	8.573	8.667	8.597	8.825	8.234
PPP2R2B	7.539	7.513	7.372	7.528	7.819	7.662
CEP89	8.158	8.153	8.188	8.287	8.252	8.171
AC092821.2	6.659	6.528	6.564	6.204	6.254	6.374
AC092821.4	6.790	6.727	6.829	6.296	6.680	6.656
CEP83	7.036	7.087	7.175	6.808	6.892	7.049
GK	8.207	8.302	8.322	8.274	8.306	8.209
HNRNPDL	10.943	10.885	10.903	10.828	10.733	10.818
P4HA3	8.138	8.010	8.025	8.157	8.191	8.018
AL008627.1	7.951	8.129	8.045	7.980	8.116	8.232
EEF2KMT	9.728	9.851	9.931	9.748	9.729	9.671
NTHL1	8.264	8.307	8.532	8.323	8.366	8.284
TEX51	6.077	6.160	6.243	6.070	6.126	6.260
AC010624.1	5.016	4.741	4.722	4.991	5.256	4.916
AC010624.2	7.109	7.206	7.106	7.421	7.183	7.258
SYNPR	5.405	5.415	5.081	5.375	5.678	5.647
TTR	8.766	8.847	8.810	8.627	8.908	8.811
TMEM266	7.217	7.106	6.947	7.167	7.147	7.253
TMEM267	7.649	7.787	7.673	7.898	7.733	7.752
TMEM265	9.488	9.608	9.790	9.768	9.895	9.826
MYPN	7.969	7.869	7.748	7.843	7.861	7.855
TMEM263	9.801	9.765	9.944	9.680	9.806	9.884
TMEM260	9.022	9.120	9.161	9.499	9.546	9.009
TTL	8.467	8.640	8.542	8.304	8.503	8.532
TMEM268	6.753	6.632	6.701	6.418	6.160	6.367
SFT2D3	9.688	9.720	9.786	9.530	9.417	9.569
SFT2D2	9.964	10.022	9.983	9.707	9.569	9.900
SFT2D1	11.745	11.801	11.870	11.707	11.613	11.665
DAP3	10.554	10.670	10.784	10.441	10.467	10.388
TOP1	11.844	11.841	12.022	11.817	11.900	11.685
TMEM190	7.474	7.625	7.628	7.693	7.556	7.681
AL157902.1	6.857	6.624	6.578	6.879	6.740	6.732
AC022107.1	6.527	6.637	6.591	6.466	6.602	6.479
TMEM196	6.836	6.881	6.810	6.984	6.995	6.939
TMEM198	8.855	8.787	8.770	8.638	8.665	8.715
TMEM199	9.845	9.822	9.881	9.829	9.964	9.932
FOXC2	9.372	9.298	9.372	9.211	9.258	9.255
FOXC1	8.745	8.584	8.829	8.892	8.908	8.808
SHROOM3	10.710	10.711	10.884	10.495	10.579	10.535
SHROOM2	8.752	8.678	8.771	8.599	8.632	8.668
SHROOM1	8.790	8.718	8.895	8.808	8.731	8.800

MIOX	7.018	7.269	7.268	7.275	7.333	7.217
SHROOM4	7.958	7.843	7.967	8.203	8.181	8.027
MIOS	9.729	9.736	9.756	9.756	9.720	9.786
PELI1	8.968	9.001	9.073	8.889	8.983	8.994
PELI3	7.299	7.529	7.553	7.401	7.399	7.304
PELI2	8.217	8.278	8.245	8.095	7.842	8.243
F11-AS1	4.370	4.439	4.267	4.088	4.198	4.257
MIR30C2	7.149	7.082	7.107	6.933	6.961	7.004
MYEOV	10.393	10.405	10.407	10.106	10.130	10.283
AL022324.4	6.390	6.066	6.649	6.405	6.522	6.479
AL022324.3	7.631	7.604	7.585	7.500	7.776	7.607
GVINP1	7.137	6.950	7.005	7.014	7.275	7.222
SRPK1	10.014	9.946	10.098	9.934	9.832	9.920
SRPK3	8.150	8.135	8.166	8.103	8.284	8.035
SRPK2	8.644	8.510	8.525	8.607	8.741	8.500
CFAP300	7.478	7.639	7.755	7.392	7.630	7.642
BDKRB2	6.807	6.765	6.922	7.004	7.095	6.987
AC007666.1	8.127	8.152	8.158	8.172	8.058	8.141
ZNF322P1	7.556	7.702	7.770	7.415	7.591	7.573
RDH8	6.835	6.742	6.851	6.761	7.003	6.786
RDH5	8.288	8.295	8.454	8.347	8.322	8.266
UXS1	10.561	10.611	10.566	10.705	10.664	10.452
UBL7-AS1	7.811	7.681	7.759	7.200	7.586	7.429
EIF4A1P10	13.137	13.230	13.247	13.064	13.071	13.089
MROH7-TTC4	9.030	9.142	9.157	8.960	9.016	9.070
RPN1	11.550	11.670	11.751	11.685	11.602	11.653
RPN2	13.673	13.658	13.630	13.614	13.670	13.490
EIF4BP6	11.753	11.772	11.939	11.764	11.728	11.676
SLC48A1	9.054	9.116	9.239	9.161	9.347	9.197
CYP4F8	5.482	5.951	5.420	5.932	5.683	5.681
PES1	9.981	10.021	10.229	9.916	9.852	9.889
NOSTRIN	9.912	9.895	10.047	9.765	9.796	9.577
EIF4BP3	11.753	11.772	11.939	11.764	11.728	11.676
AC005363.1	13.985	14.011	14.089	14.022	13.964	14.014
THEMIS	4.255	4.090	4.179	4.258	4.131	4.264
CYP4F2	7.618	7.784	7.672	7.594	7.526	7.718
CYP4F3	7.626	7.836	7.820	7.639	7.602	7.728
ASB2	7.057	6.940	7.023	6.980	7.089	6.870
ASB3	8.916	9.186	9.371	9.089	9.377	8.822
ASB1	9.014	9.062	9.116	8.817	8.736	8.857
ASB6	8.730	8.694	8.841	8.656	8.668	8.595
ASB7	9.132	9.106	9.319	8.749	8.804	8.748
ASB4	6.629	6.588	6.593	6.817	6.661	6.788
ASB5	3.777	4.015	3.856	3.706	3.933	3.880
ASB8	8.578	8.511	8.760	8.732	8.906	8.344
ASB9	8.906	8.908	9.198	8.598	8.651	8.712
SAG	7.369	7.959	7.720	7.820	7.956	8.085
AC091951.4	6.905	6.937	6.849	7.091	7.161	7.066
AL365209.1	7.537	7.316	7.581	7.535	7.650	7.608
TMEM44	8.176	8.149	8.343	8.104	8.082	8.162
TMEM47	12.865	12.958	12.984	12.915	12.815	12.919
TMEM40	7.252	7.253	7.319	7.308	7.159	7.281
TMEM43	9.661	9.693	9.727	9.577	9.635	9.574
TMEM42	8.931	8.981	9.162	9.042	9.014	8.957
NHLH1	8.037	8.058	8.041	8.436	8.252	8.153
BX248084.1	7.145	7.246	7.000	7.072	7.096	7.194

AC025263.2	9.880	10.010	10.076	9.919	9.936	9.962
AC025263.3	5.453	5.575	5.407	5.519	5.583	5.473
AC025263.1	5.919	5.681	5.944	6.007	6.013	5.988
ZNF600	6.171	6.200	6.379	6.138	6.399	6.473
ZNF606	8.077	8.216	8.147	8.176	8.128	8.180
ZNF607	5.876	5.747	5.889	5.663	5.775	5.697
ZNF605	7.805	7.831	7.815	7.541	7.291	7.655
TREX2	8.527	8.630	8.739	8.377	8.419	8.510
ITIH5	7.249	7.279	7.201	7.236	7.341	7.271
ZNF608	7.839	7.866	7.848	7.762	7.840	7.662
TREX1	9.860	9.929	10.002	9.845	9.924	9.818
PIRT	7.081	6.983	7.321	7.497	7.368	7.390
GCA	9.486	9.590	9.596	9.196	9.424	9.253
OR1C1	6.312	6.381	6.225	6.262	6.695	6.467
GCG	4.794	4.760	4.851	5.067	4.965	4.879
GCK	6.965	7.085	7.065	7.324	7.273	7.249
AC073869.1	9.394	9.406	9.580	9.048	8.942	9.120
AL589674.1	10.929	11.039	11.181	10.944	10.887	11.004
AC073869.3	9.464	9.500	9.588	9.208	9.390	9.346
TMSB4XP6	13.840	13.910	13.927	13.935	13.998	13.997
PROZ	7.377	7.392	7.480	7.322	7.623	7.488
AZGP1	7.964	7.926	8.049	8.136	7.778	8.007
PROC	6.776	6.715	7.276	7.140	6.956	6.734
AC013394.1	8.310	8.296	8.384	8.039	8.119	8.177
NUTF2	10.828	10.703	10.997	10.559	10.661	10.303
PRSS30P	6.597	6.529	6.646	6.846	6.703	6.714
CYLC1	7.341	7.209	7.311	7.253	7.311	7.277
CYLC2	5.597	5.602	5.523	5.572	5.501	5.649
TSPY6P	4.547	4.525	4.638	4.840	4.849	4.586
AC004057.1	12.821	12.916	13.024	12.923	12.903	12.978
SNRPA1	11.706	11.756	11.828	11.257	11.375	11.346
PRPF40A	11.866	11.785	11.825	11.822	11.643	11.609
TBC1D29P	6.921	6.802	6.928	7.039	7.267	7.194
ENKUR	5.573	5.542	5.407	5.611	5.840	5.741
TNFSF8	8.028	7.903	7.800	7.906	7.902	7.917
TMSB4XP8	13.840	13.910	13.927	13.935	13.998	13.997
C4B_2	8.508	8.681	8.617	8.611	8.632	8.722
FUT8-AS1	7.515	7.522	7.309	7.389	7.391	7.437
GPCPD1	7.754	7.657	7.640	7.585	7.493	7.621
AC108099.1	4.315	4.120	4.330	4.321	4.483	4.381
SEPHS2	11.148	11.204	11.302	11.027	10.837	10.945
SEPHS1	9.464	9.491	9.539	9.140	9.138	9.095
RPS4X	13.861	13.930	13.929	13.939	13.927	13.951
AC138331.1	5.754	5.503	5.729	5.780	5.721	5.780
AC121247.2	6.234	6.083	5.931	6.246	6.158	6.208
AF067845.3	5.447	5.369	5.353	5.338	5.388	5.407
AF067845.2	5.512	5.568	5.763	5.548	5.607	5.654
AF067845.1	5.447	5.369	5.353	5.338	5.388	5.407
DLST	9.200	9.239	9.365	9.178	8.971	9.315
IGKV1OR1-1	8.398	8.520	8.424	8.478	8.571	8.522
EIF3J-DT	7.885	7.629	7.822	7.778	7.565	7.629
IL1F10	7.370	7.386	7.373	7.324	7.304	7.176
SDCBP2	10.254	10.277	10.337	10.293	10.191	10.289
RAB30-DT	8.181	8.129	8.187	8.092	8.127	8.070
ZNF559-ZNF177	6.191	6.246	6.127	5.971	6.292	6.021
SNAPIN	10.441	10.372	10.590	10.480	10.347	10.448

AL360294.1	7.187	7.235	6.896	7.016	7.190	6.985
HSCB	7.913	8.094	8.308	7.864	7.829	8.090
VASN	8.600	8.560	8.629	8.549	8.563	8.708
NBPF15	11.550	11.483	11.377	11.343	11.231	11.508
NBPF14	11.550	11.483	11.377	11.343	11.231	11.508
NBPF12	11.550	11.483	11.377	11.343	11.231	11.508
NBPF11	11.550	11.483	11.377	11.343	11.231	11.508
NBPF10	11.550	11.483	11.377	11.343	11.231	11.508
AC109479.1	7.883	7.866	7.860	7.971	7.699	7.896
VASP	11.544	11.612	11.796	11.496	11.512	11.398
PAPOLG	8.321	8.331	8.439	8.549	8.664	8.601
PAPOLA	11.426	11.420	11.454	11.424	11.221	11.454
PAPOLB	5.078	5.144	4.760	5.324	5.283	5.554
LYPLAL1	9.993	10.018	10.253	9.798	10.000	10.016
GOLGA8CP	6.446	6.294	6.356	6.148	6.123	6.590
LINC01847	6.255	6.298	6.253	6.242	6.420	6.346
LINC01844	6.163	6.350	6.062	6.228	5.966	6.063
LINC01845	6.219	6.326	6.075	6.048	6.389	6.322
LINC01842	6.294	6.222	6.117	6.313	6.077	6.581
LINC01843	7.445	7.567	7.746	7.599	7.620	7.673
ZSCAN31	6.792	6.664	7.050	6.977	7.139	6.975
ZSCAN30	8.689	8.540	8.577	8.641	8.870	8.782
TDRG1	9.193	8.774	8.660	8.977	8.816	8.985
EPB41L4A-AS1	9.789	9.785	10.068	9.448	9.407	9.508
UBE2SP1	11.824	11.787	12.057	10.982	11.221	10.899
UBE2SP2	11.824	11.787	12.057	10.982	11.221	10.899
CYP51A1	11.508	11.576	11.617	11.262	11.230	11.545
TUT1	7.346	7.507	7.604	7.401	7.358	7.323
CDNF	4.992	4.851	5.114	4.766	4.948	4.878
ASRGL1	11.299	11.208	11.378	10.973	10.678	10.716
WDR61	10.778	10.874	11.026	10.862	10.863	10.914
WDR66	7.140	7.445	7.420	7.347	7.228	7.461
TUT4	9.148	9.063	9.034	8.849	9.010	8.850
TUT7	8.687	8.635	8.712	8.538	8.534	8.659
WEE2-AS1	6.847	6.762	7.006	6.884	7.015	7.095
SLC25A3	13.356	13.409	13.477	13.362	13.290	13.365
SLC25A2	6.483	6.570	6.539	6.312	6.679	6.556
SLC25A1	11.631	11.707	11.956	11.592	11.684	11.486
TONSL	8.689	8.831	8.843	8.763	8.508	8.831
SLC25A6	12.268	12.388	12.597	12.171	12.037	12.142
SLC25A5	13.094	13.173	13.259	13.094	13.081	13.141
SLC25A4	9.282	9.306	9.473	9.241	9.204	9.267
SGMS1-AS1	6.710	6.628	6.690	6.445	6.639	6.796
TTC23L	7.207	7.279	6.950	7.118	7.140	7.097
CEBPZOS	9.534	9.483	9.566	9.396	9.681	9.526
KIF5A	7.907	7.983	7.842	7.890	8.082	8.140
KIF5B	11.474	11.437	11.456	11.350	11.340	11.322
KIF5C	6.763	7.039	7.147	7.225	6.921	7.281
MASP1	8.090	8.157	8.111	8.041	8.108	8.151
MASP2	7.269	7.309	7.573	7.573	7.560	7.448
AP001486.2	7.121	7.457	7.292	7.298	7.338	7.508
PEBP1P2	11.537	11.598	11.767	11.480	11.403	11.386
GIHCG	7.433	7.572	7.547	7.270	7.476	7.284
IFNGR1	9.619	9.758	9.689	9.502	9.528	9.564
IFNGR2	11.217	11.295	11.342	11.382	11.231	11.343
CMIP	10.732	10.849	10.852	10.867	10.997	10.612

IARS1	11.708	11.673	11.784	11.645	11.566	11.535
IARS2	11.209	11.247	11.311	11.034	10.905	11.129
ANXA2P3	9.364	9.436	9.543	9.391	9.401	9.285
USP49	7.920	7.888	7.700	7.539	7.721	7.664
ECSCR	6.937	7.000	6.938	7.087	7.113	6.929
USP45	6.128	6.189	6.020	5.612	5.715	6.029
USP44	7.691	7.587	7.612	7.667	7.845	8.023
USP47	9.891	9.859	9.952	9.689	9.663	9.684
USP46	7.749	7.877	7.822	7.938	7.911	7.894
USP40	9.018	9.113	9.274	8.824	8.830	8.962
USP43	7.646	7.676	7.454	7.708	7.685	7.695
USP42	8.784	8.803	8.789	8.706	8.904	8.865
HCP5	8.339	8.271	8.468	8.305	8.352	8.295
AL157935.3	8.316	8.125	8.364	8.417	8.464	8.306
HNRNPA1P31	5.712	5.528	5.851	6.099	6.005	5.856
GGTA1P	7.655	7.758	7.443	7.624	7.649	7.512
PPP1R11	11.365	11.417	11.529	11.228	11.352	11.201
ZPR1	8.623	8.701	8.792	8.576	8.445	8.520
TPRG1L	9.854	9.944	10.050	9.765	9.527	9.723
TEDDM1	6.061	6.144	6.226	6.235	6.292	6.213
CFAP299	4.782	4.949	4.970	5.034	5.048	4.960
VAT1L	7.782	7.662	7.547	8.011	7.773	7.671
IGKV4-1	6.928	6.935	7.263	7.194	7.106	7.047
BLK	6.505	6.510	6.566	6.638	6.627	6.775
RPS7	13.359	13.392	13.529	13.540	13.451	13.548
RPS6	13.934	13.979	14.032	14.131	14.031	14.132
RPS5	13.948	14.013	14.071	14.142	14.111	14.155
RPS3	13.793	13.884	13.989	13.907	13.914	13.957
RPS2	14.545	14.566	14.561	14.591	14.532	14.585
LINC02123	5.812	5.607	5.660	5.726	5.909	5.787
VDAC3	11.931	12.079	12.142	12.065	12.160	12.084
VDAC2	11.560	11.653	11.787	11.596	11.450	11.551
VDAC1	12.800	12.921	12.930	12.846	12.843	12.896
RPS9	12.988	13.007	13.216	12.976	12.758	13.030
RPS8	13.214	13.233	13.392	13.147	13.175	13.114
LINC01301	4.337	4.099	4.053	3.976	4.241	4.020
B3GAT3P1	8.181	8.258	8.580	8.241	8.413	8.353
GRM8-AS1	6.517	6.430	6.520	6.434	6.550	6.389
HPF1	9.042	8.999	9.246	8.943	9.051	9.054
NAPA-AS1	7.288	7.270	7.466	7.417	7.536	7.355
AJM1	10.156	10.186	10.391	10.078	10.117	10.120
LINC01305	4.697	4.662	4.734	4.715	4.636	4.617
RNU6-914P	9.059	9.153	8.975	9.045	9.115	9.175
IGSF22	7.105	7.259	7.320	7.264	7.364	7.322
IGSF21	6.110	6.120	6.076	6.059	6.077	6.187
RPSA	13.432	13.517	13.661	13.465	13.519	13.574
TAPBP	11.754	11.634	11.807	11.470	11.413	11.561
AL133330.1	6.740	6.648	6.563	6.524	6.735	6.622
ADRB2	8.408	8.450	8.470	8.294	8.512	8.196
ADRB3	7.419	7.363	7.489	7.644	7.627	7.600
MCM3AP	9.209	9.260	9.392	9.166	9.045	9.225
IGHV1-69D	7.655	7.838	7.869	7.825	7.775	7.867
LINC01477	5.844	5.733	5.612	5.814	6.063	5.933
RPL29	13.159	13.195	13.371	13.208	13.111	13.211
RPL28	14.030	14.079	14.129	14.035	13.946	14.058
RPL24	12.981	13.066	13.218	13.218	13.130	13.174

RPL27	13.614	13.623	13.714	13.796	13.787	13.898
RPL21	12.617	12.677	12.802	12.784	12.769	12.841
RPL23	13.979	14.042	14.002	13.909	13.815	14.061
RPL22	13.111	13.257	13.313	13.229	13.072	13.208
ATG3	10.037	10.065	10.077	9.952	9.944	9.927
ATG7	8.693	8.799	8.767	8.740	8.690	8.623
ATG5	9.470	9.612	9.749	9.679	9.714	9.746
AC011503.1	5.277	5.171	5.224	5.375	5.184	5.148
AC011503.2	7.266	7.476	7.206	7.218	7.155	7.140
TTK	8.382	8.474	8.543	8.255	8.311	8.243
LINC01788	5.177	5.251	5.487	5.351	5.447	5.311
ATXN7	8.500	8.678	8.530	8.452	8.610	8.445
LINC01785	6.981	6.965	6.836	6.957	6.886	6.903
AC006453.3	7.347	7.553	7.386	7.650	7.400	7.673
LINC01782	6.082	6.127	5.874	6.376	6.172	6.088
AC104472.3	9.989	10.161	10.162	10.282	10.120	10.215
UGT1A10	11.432	11.431	11.462	11.446	11.328	11.440
SNORA11E	6.419	6.313	6.674	6.599	6.492	6.414
SNORA11D	6.419	6.313	6.674	6.599	6.492	6.414
SNORA11F	7.425	7.305	7.110	7.432	7.140	7.393
IGLV4-3	7.754	8.019	8.042	8.188	7.829	8.037
AC021517.1	5.647	5.339	5.495	5.452	5.784	5.531
SNRNP35	8.722	8.733	8.912	8.853	8.588	8.577
AC010947.1	6.437	6.540	6.397	6.569	6.745	6.542
AC004908.1	6.509	6.232	6.516	6.437	6.622	6.494
AC083799.1	8.445	8.511	8.465	8.015	8.141	8.174
AC004908.3	6.176	6.128	6.098	6.319	5.843	6.189
AC004908.2	4.708	4.836	4.639	4.899	4.758	4.860
AL512625.1	8.597	8.585	8.594	8.520	8.633	8.531
ACYP1	8.026	8.153	8.180	7.829	7.883	7.747
ACYP2	8.848	8.866	8.976	8.592	8.947	8.956
H3P47	13.752	13.863	13.907	13.791	13.836	13.535
SGO2	8.479	8.368	8.485	7.890	8.852	8.063
SGO1	7.416	7.508	7.406	7.424	7.555	7.436
LINC00437	4.101	4.061	4.063	4.228	4.014	4.027
ACSL6	5.651	5.315	5.200	5.710	5.418	5.561
ACSL4	9.399	9.528	9.484	9.245	9.366	9.492
ACSL5	10.514	10.619	10.701	10.491	10.445	10.453
SMPX	6.211	6.243	5.994	6.180	6.296	6.350
AC082651.3	5.038	5.218	5.014	4.927	5.110	5.078
AC082651.1	7.741	7.720	7.495	7.738	7.843	7.741
ATF4P3	12.001	12.006	12.232	11.846	11.858	11.769
TRIM39	9.354	9.341	9.277	9.095	9.038	9.183
TRIM38	9.750	9.712	9.792	9.824	10.030	9.540
AC010735.2	7.460	7.389	7.329	7.474	7.596	7.509
AC010735.1	6.440	6.538	6.366	7.008	6.613	6.625
HR	9.073	9.060	9.079	9.137	9.152	9.202
TRIM32	7.910	8.163	8.127	7.757	7.935	7.854
HP	6.839	6.551	6.650	6.973	6.835	6.597
TRIM37	9.077	9.053	8.991	8.630	8.716	8.740
TRIM36	5.456	5.396	5.335	5.704	5.713	5.471
AC019129.2	8.880	8.704	8.950	8.972	9.067	8.986
TRIM34	9.012	8.999	9.043	8.865	8.983	8.951
CCDC180	6.388	6.361	6.409	6.498	6.510	6.580
NEBL	7.842	7.759	7.987	7.976	7.959	7.997
AP1AR	9.471	9.450	9.598	9.458	9.477	9.204

ATF4P4	4.269	4.489	4.298	4.169	4.286	4.535
AL691459.1	6.828	6.617	6.429	6.470	6.577	6.473
ZNF585A	7.477	7.597	7.543	7.231	7.410	7.579
ZNF585B	8.624	8.523	8.684	8.514	8.426	8.648
CCDC187	7.577	7.376	7.232	7.380	7.399	7.528
CCDC186	7.655	7.700	7.495	7.231	8.061	7.259
TPP2	8.384	8.322	8.278	8.027	8.145	8.229
C14orf39	3.690	3.682	3.750	3.650	3.676	3.680
FPGT-TNNI3K	5.802	5.688	5.584	6.198	5.891	5.874
DNAJC30	9.420	9.473	9.559	9.368	9.206	9.372
STRIP2	6.342	6.204	6.304	6.434	6.285	6.219
AC016590.1	5.564	5.850	5.755	5.988	5.720	5.794
UQCC2	9.492	9.490	9.791	9.611	9.683	9.692
IQCF6	5.904	5.673	5.953	6.123	5.925	5.937
IQCF5	5.731	5.643	5.485	5.815	5.833	5.888
IQCF2	6.460	6.583	6.465	6.760	6.427	6.629
IQCF3	6.337	6.162	6.285	6.305	6.353	6.341
IQCF1	6.882	7.299	7.445	7.064	7.386	7.419
P2RY10	6.516	6.681	6.216	6.404	6.448	6.676
Z99756.1	5.805	5.858	5.711	5.969	5.840	5.756
AC099494.5	5.053	5.083	5.157	5.143	5.168	5.314
NEURL1	7.215	7.141	7.271	7.401	7.530	7.222
NEURL2	7.103	7.116	7.071	7.287	7.060	7.104
NEURL3	9.607	9.516	9.623	9.742	9.627	9.697
NEURL4	7.543	7.308	7.452	7.514	7.482	7.471
AC099494.2	5.053	5.083	5.157	5.143	5.168	5.314
LDHAL6CP	6.242	6.298	6.114	6.204	6.226	6.205
AL691447.2	6.386	6.150	6.103	6.285	6.268	6.064
PROX1	6.328	6.405	6.469	6.343	6.449	6.291
PROX2	4.823	4.794	4.893	4.881	4.731	5.018
ATCAY	7.340	7.468	7.446	7.420	7.508	7.400
USHBP1	7.477	7.329	7.598	7.630	7.652	7.574
TRAV12-1	5.773	5.624	5.384	5.793	5.557	5.747
RETREG1	8.581	8.505	8.490	8.143	8.272	8.487
RETREG2	10.094	10.240	10.364	10.003	9.985	10.039
RETREG3	10.782	10.957	11.027	10.844	10.781	10.733
DECR2	9.606	9.630	9.697	9.538	9.374	9.500
DECR1	10.212	10.271	10.377	10.148	10.167	10.234
MIR133A1HG	4.286	4.270	4.319	4.321	4.379	4.271
TFF1	10.027	10.015	10.091	9.726	10.066	9.999
TFF2	10.174	10.209	10.390	10.027	10.251	10.171
TFF3	7.580	7.621	7.642	7.623	7.516	7.598
SNORD6	7.030	6.890	6.650	6.616	6.727	6.999
SNORD7	7.671	7.629	7.692	7.578	7.734	7.573
SNORD8	7.001	6.998	6.488	6.895	6.887	7.082
KLB	5.213	5.117	5.074	5.046	5.162	5.172
TMPRSS2	9.370	9.372	9.370	9.369	9.230	9.311
TMPRSS3	8.601	8.646	8.774	8.516	8.739	8.714
TMPRSS4	11.023	11.185	11.290	11.084	11.038	10.986
TMPRSS5	8.070	8.091	8.216	8.015	8.171	8.136
TMPRSS6	8.552	8.770	8.710	8.697	8.810	8.876
ABCC12	6.427	6.608	6.539	6.523	6.568	6.888
ABCC13	6.914	6.694	6.801	6.822	6.979	7.035
ABCC10	8.182	8.208	8.248	8.064	8.211	8.208
ABCC11	8.052	8.106	8.039	8.040	8.222	8.199
PRAMEF33	7.104	7.179	7.162	7.393	7.499	7.346

EMG1	10.084	10.226	10.423	10.087	10.078	10.238
RNA5SP392	8.384	8.349	8.513	8.424	8.239	8.291
TRIM16L	8.796	8.798	8.807	8.359	8.555	8.964
COMMD8	9.901	9.940	10.188	9.970	10.051	10.106
C9orf62	7.265	7.497	7.511	7.606	7.474	7.550
COMMD2	9.648	9.670	9.900	9.114	9.502	9.203
COMMD3	9.623	9.527	9.690	9.884	9.686	9.903
COMMD1	13.432	13.517	13.661	13.465	13.519	13.574
COMMD6	9.738	9.775	10.034	9.866	9.720	9.828
COMMD7	10.576	10.646	10.856	10.820	10.669	10.819
COMMD4	10.707	10.793	10.974	10.563	10.487	10.629
COMMD5	9.019	9.169	9.328	8.937	9.071	9.024
AL662796.1	8.663	8.804	8.671	8.843	8.826	8.813
ZMYM2	8.476	8.639	8.660	8.347	8.393	8.508
ZMYM3	9.940	9.915	9.951	9.725	9.878	9.782
ZMYM1	7.406	7.374	7.334	7.111	7.242	7.343
ZMYM6	7.544	7.649	7.563	7.444	7.486	7.773
AC018901.3	6.358	6.363	6.500	6.540	6.649	6.693
ZMYM5	8.098	8.110	7.992	8.000	8.119	8.212
FDPSP5	6.995	7.089	7.101	6.937	6.984	6.974
LMNTD1	8.068	8.132	8.037	8.147	8.122	8.328
DEPDC1B	10.258	10.349	10.298	10.221	10.184	10.202
LMNTD2	7.545	7.787	7.812	7.639	7.471	7.640
AL353052.2	5.197	5.228	5.288	5.282	5.389	5.365
H2AX	11.090	11.139	11.265	10.735	10.687	10.582
H2AP	6.597	6.410	6.327	6.485	6.421	6.396
TRMT9B	6.991	7.102	7.266	7.934	7.681	7.784
H2AW	9.364	9.390	9.452	9.365	9.336	9.383
H2AJ	12.038	12.061	12.298	12.085	11.878	12.065
ANO8	7.880	7.635	7.908	7.979	7.866	7.956
ANO9	8.642	8.514	8.702	8.640	8.679	8.664
ANO4	7.284	7.420	7.387	7.540	7.457	7.335
ANO5	6.722	6.972	6.776	6.727	6.755	6.607
ANO6	10.145	10.190	10.058	9.912	9.991	10.022
DUXAP9	4.439	4.773	4.668	4.763	4.712	4.732
ANO1	7.378	7.260	7.326	7.607	7.413	7.393
ANO2	6.029	6.015	5.982	6.031	6.174	6.078
ANO3	5.197	5.096	5.078	5.108	5.286	5.414
COL3A1	6.249	6.355	6.410	6.531	6.428	6.110
AL513550.1	6.151	6.132	5.953	6.121	6.125	5.746
AC008026.1	13.767	13.833	13.946	13.964	13.837	13.963
AC008026.3	5.549	5.701	5.334	5.701	5.730	5.634
GTF2B	10.112	10.208	10.306	10.363	10.340	10.287
PALM2AKAP2	9.158	9.188	9.040	8.842	8.847	8.911
AC100791.2	8.199	8.185	8.419	8.328	8.403	8.420
CHUK	7.797	7.828	7.917	7.486	7.516	7.890
CSGALNACT2	8.516	8.656	8.469	8.093	8.336	8.477
LRMDA	7.945	7.958	7.957	7.817	7.932	7.704
AC008735.3	8.262	8.116	7.994	8.393	8.406	8.190
C5orf17	6.039	6.205	6.069	5.991	6.121	6.139
C5orf15	11.358	11.426	11.547	11.385	11.271	11.405
MYZAP	8.084	8.234	8.239	8.064	8.118	8.141
ASH2L	11.237	11.230	11.326	11.188	10.956	11.041
AC004947.1	5.768	5.737	5.686	5.768	5.752	5.720
FTHL17	4.147	4.299	4.297	4.320	4.522	4.282
RGMB	9.061	9.001	9.027	9.122	9.101	9.043

RGMA	7.645	8.016	7.953	7.810	7.828	7.935
UTF1	6.061	6.110	6.081	6.143	6.036	5.995
AC011995.1	7.526	7.554	7.701	7.412	7.530	7.532
AC104072.1	4.435	4.342	4.238	4.391	4.368	4.717
GIMAP1-GIMAP5	8.693	8.874	8.803	8.853	8.997	9.078
POLR2K	9.550	9.706	9.739	9.568	9.681	9.578
BHLHB9	7.023	7.243	7.004	6.688	6.870	6.558
GRID1	6.394	6.439	6.569	6.509	6.503	6.505
GRID2	6.174	6.436	6.101	6.326	6.702	6.254
ST7L	7.593	7.796	7.566	7.590	7.813	7.778
CYP2C9	9.500	9.442	9.225	9.454	9.477	9.537
CYP2C8	5.856	5.670	5.669	5.620	5.836	5.647
APOBEC3D	8.367	8.476	8.415	8.424	8.563	8.500
APOBEC3G	9.072	9.183	9.204	9.469	9.339	9.280
APOBEC3F	8.017	8.175	8.157	7.959	8.142	8.071
APOBEC3A	7.439	7.473	7.552	6.526	6.844	6.824
APOBEC3C	8.923	8.987	9.225	8.841	8.738	8.630
APOBEC3B	7.439	7.473	7.552	6.526	6.844	6.824
C10orf67	8.038	8.160	8.141	8.110	8.155	8.228
AC008695.1	8.740	8.846	8.768	8.881	8.880	8.563
C10orf62	8.219	8.247	8.372	8.449	8.363	8.359
GGT3P	10.427	10.413	10.580	10.351	10.206	10.300
DHFR	10.004	9.924	10.156	9.679	9.787	9.678
GIPR	7.935	7.973	7.971	7.981	8.012	7.853
LPIN2	8.657	8.720	8.707	8.635	8.579	8.739
LPIN3	7.010	7.129	7.173	7.420	7.114	7.174
LPIN1	10.662	10.760	11.043	10.681	10.540	10.344
MIR4800	8.560	8.501	8.720	8.341	8.468	8.399
CTBP1-AS	5.437	5.356	5.382	5.444	5.109	5.280
SMIM41	8.595	8.578	8.492	8.458	8.660	8.477
BATF3	7.986	7.928	8.072	8.016	8.145	8.147
KLHDC7B	8.153	7.956	8.160	7.938	8.121	7.902
KLHDC7A	9.575	9.681	9.708	9.270	9.388	9.371
MUSTN1	6.125	6.381	6.414	6.489	6.390	6.238
NUP35	9.662	9.752	9.776	9.430	9.288	9.531
NUP37	9.954	10.045	10.195	9.942	9.993	9.964
WHAMMP3	10.060	9.866	9.609	10.116	10.267	10.103
WHAMMP2	10.060	9.866	9.609	10.116	10.267	10.103
SLC1A4	10.132	10.135	10.213	9.953	9.906	9.911
CYP11B2	8.058	8.225	8.365	8.457	8.416	8.279
CYP11B1	5.533	5.865	5.700	5.968	5.792	5.945
SLC1A7	7.649	7.711	7.802	7.856	7.632	7.825
AL133238.1	3.838	3.960	3.971	3.948	3.984	4.181
SLC1A1	12.508	12.551	12.539	12.335	12.160	12.304
SLC1A2	7.862	7.897	8.040	8.267	8.120	8.193
SLC1A3	7.850	7.870	7.863	8.014	7.899	7.848
AL390039.1	10.952	11.038	11.036	10.953	10.934	10.876
RSPH4A	4.054	4.058	3.957	4.039	4.062	4.173
AC106741.2	6.837	6.927	7.007	6.846	6.742	6.922
AP001432.1	8.140	8.121	7.978	8.376	8.292	8.353
MIR503HG	8.618	8.474	8.711	8.715	8.546	8.939
MIPEP	8.907	8.952	9.084	9.053	9.025	9.010
VSIR	8.619	8.629	8.704	8.444	8.529	8.541
CCNL1	10.287	10.124	10.072	10.477	10.271	10.584
CCNL2	9.188	8.840	9.136	8.984	8.789	9.159
SSX2B	7.872	7.778	7.610	7.837	7.973	7.959

NOMO2	12.700	12.750	12.798	12.773	12.583	12.712
NOMO3	12.700	12.750	12.798	12.773	12.583	12.712
NOMO1	12.700	12.750	12.798	12.773	12.583	12.712
BAZ1A	9.193	9.176	9.013	9.124	9.141	9.197
MTFP1	9.784	9.767	9.982	9.734	9.683	9.699
LEAP2	6.405	6.406	6.291	6.441	6.524	6.437
TRAF3IP2-AS1	6.683	6.511	6.611	6.934	6.743	6.546
LELP1	6.728	6.722	6.715	6.878	6.776	6.693
SLC2A9	7.498	7.841	7.554	7.899	7.891	7.859
SLC2A8	8.037	8.104	8.084	7.990	7.904	8.056
SLC2A6	8.416	8.297	8.423	8.116	8.292	8.228
SLC2A5	7.697	7.914	7.629	7.661	7.678	7.795
SLC2A4	6.305	6.313	6.420	6.431	6.548	6.334
SLC2A3	7.293	7.365	7.397	7.705	7.481	7.358
SLC2A2	4.731	4.668	4.396	4.433	4.788	4.728
SLC2A1	10.224	10.324	10.423	10.248	10.181	10.241
AC103724.3	7.725	7.545	7.621	7.599	7.618	7.756
TBC1D3B	10.803	10.689	10.641	10.716	10.587	10.876
TBC1D3C	10.803	10.689	10.641	10.716	10.587	10.876
DAB1-AS1	4.751	4.969	4.712	4.877	4.906	4.780
TBC1D3E	10.803	10.689	10.641	10.716	10.587	10.876
TBC1D3F	10.803	10.689	10.641	10.716	10.587	10.876
TBC1D3G	10.803	10.689	10.641	10.716	10.587	10.876
TBC1D3H	10.803	10.689	10.641	10.716	10.587	10.876
TBC1D3I	10.803	10.689	10.641	10.716	10.587	10.876
TBC1D3J	10.803	10.689	10.641	10.716	10.587	10.876
TBC1D3K	10.803	10.689	10.641	10.716	10.587	10.876
TBC1D3L	10.803	10.689	10.641	10.716	10.587	10.876
SMLR1	6.142	6.227	6.236	6.098	6.290	6.308
TAB2	9.615	9.700	9.676	9.578	9.683	9.639
AL365271.1	6.083	6.306	6.361	6.316	6.303	6.226
AL136309.3	4.156	4.451	4.349	4.255	4.180	4.219
CEACAM1	10.804	10.960	11.052	10.956	10.903	10.879
ATRIP	9.860	9.929	10.002	9.845	9.924	9.818
CEACAM3	8.467	8.516	8.561	8.681	8.607	8.513
CEACAM5	10.995	11.216	11.152	10.839	10.898	10.712
CEACAM4	8.300	8.247	8.430	8.492	8.433	8.406
CEACAM7	9.722	9.853	9.816	9.208	9.423	9.358
CEACAM6	13.232	13.297	13.305	13.223	13.269	13.170
CEACAM8	4.490	4.604	4.408	4.586	4.579	4.524
SNORD114-9	4.976	4.928	4.987	5.012	5.075	5.122
GTF2IRD2	8.323	8.301	8.564	7.924	8.144	8.144
KRT18P50	6.557	6.747	6.772	6.796	6.744	6.717
PRR23D2	5.370	5.394	5.407	5.751	5.892	5.738
FZD7	8.242	8.223	8.303	8.135	8.293	8.166
TBC1D30	7.880	7.943	7.849	7.942	7.953	7.832
FZD8	8.951	9.019	9.181	9.143	9.117	8.917
TBC1D32	5.048	4.849	5.046	4.654	4.889	4.926
C20orf144	5.858	5.748	5.777	6.125	6.007	6.115
FZD9	7.283	7.222	7.292	7.334	7.288	7.314
CREBBP	9.631	9.638	9.619	9.528	9.544	9.676
SHISAL2A	7.289	7.368	7.462	7.104	7.444	7.660
TRNAU1AP	7.977	8.244	8.359	8.082	8.096	7.901
TMEM126A	9.813	9.846	10.035	9.641	9.910	9.819
TMEM126B	9.550	9.567	9.615	9.879	9.918	10.035
MOCOS	9.356	9.366	9.572	9.260	9.274	9.318

JAKMIP2-AS1	6.682	6.793	6.775	7.027	6.868	6.905
AL355075.1	6.716	6.951	6.844	6.915	6.843	6.914
AC073195.2	6.592	6.674	6.711	6.677	6.667	6.709
AL355075.4	6.716	6.951	6.844	6.915	6.843	6.914
GSG1L	7.766	7.864	8.031	8.046	7.978	8.035
RNU2-15P	5.133	5.152	5.335	5.288	5.248	5.101
LINC00900	5.707	5.874	5.811	5.624	5.578	5.967
LINC00901	4.298	4.335	4.240	4.339	4.481	4.551
LINC00905	7.520	7.515	7.380	7.273	7.714	7.696
LINC00906	6.382	6.272	6.184	6.258	6.386	6.342
LINC00907	6.478	6.526	6.625	6.590	6.506	6.662
LINC00909	8.230	8.171	8.340	8.248	8.204	8.182
PDC	4.889	4.772	4.749	4.812	4.865	4.966
H2BE1	5.938	5.683	5.673	5.636	5.778	5.875
AL138733.2	6.590	6.698	6.453	6.858	7.099	6.847
TAF9B	8.615	8.734	8.566	8.348	8.329	8.059
AC011476.3	6.690	6.934	6.887	6.748	6.817	6.904
AC011476.2	8.340	8.224	8.370	8.418	8.070	8.323
AL662828.1	7.787	7.777	7.897	8.212	8.057	7.746
AL080315.2	4.298	4.335	4.371	4.342	4.512	4.423
SF3B5	11.581	11.652	11.876	11.604	11.552	11.439
SF3B4	11.316	11.400	11.463	11.159	11.269	11.187
SF3B6	11.517	11.573	11.687	11.504	11.484	11.379
SF3B1	11.947	12.005	12.082	11.975	11.800	11.880
SF3B3	10.507	10.634	10.755	10.645	10.484	10.580
SF3B2	11.223	11.289	11.391	11.092	10.907	11.059
CNIH1	11.654	11.806	11.866	11.961	11.976	12.038
CNIH3	4.970	5.160	4.965	5.180	4.970	4.906
CNIH2	7.461	7.688	7.882	7.829	7.767	7.647
NSMCE4A	9.216	9.196	9.091	8.780	8.996	8.994
CNIH4	11.620	11.594	11.789	11.738	11.685	11.664
RLIM	13.179	13.076	13.119	13.096	13.248	13.081
SUMF2	11.911	11.951	12.082	11.887	11.894	11.978
NUDT16P1	9.059	9.079	9.169	9.008	9.008	9.116
MLN	7.522	7.581	7.561	7.819	7.545	7.634
NAT8B	6.898	6.791	6.847	6.773	6.742	6.452
MIR1229	11.984	12.002	12.105	11.694	11.496	11.720
MIR1224	8.474	8.244	8.359	8.450	8.600	8.502
MIR1225	8.103	8.227	8.278	8.711	8.454	8.244
MLX	11.040	11.117	11.185	11.153	11.127	11.078
MIR1227	9.164	9.324	9.525	9.246	9.264	9.239
NAT8L	7.993	8.047	8.090	8.024	7.985	8.042
EDRF1	8.032	7.742	7.902	7.576	7.544	7.667
AL353572.4	6.368	6.524	6.642	6.469	6.741	6.437
AC112694.2	5.404	5.410	5.766	5.233	5.645	5.382
ADD3	11.105	11.128	11.163	10.992	11.185	10.809
ADD2	8.168	8.199	8.259	8.407	8.345	8.230
ADD1	9.930	9.802	9.869	9.867	9.866	9.912
PKD2L2	4.544	4.563	4.506	4.746	4.552	4.813
PKD2L1	7.997	7.993	8.083	8.081	8.209	8.127
CWC27	8.741	8.628	8.859	8.510	8.697	8.317
CWC25	10.350	10.450	10.463	10.536	10.519	10.387
CWC22	7.836	7.761	7.851	7.768	7.649	7.821
AP006287.3	4.375	4.361	4.141	4.288	4.312	4.277
FMOD	7.610	7.742	7.809	7.783	7.892	7.758
FMO2	8.272	8.175	8.104	8.288	8.409	8.412

FMO3	5.658	5.953	5.950	6.006	6.143	6.168
FMO1	6.413	6.783	6.656	7.002	6.794	6.741
CASZ1	8.495	8.654	8.702	8.394	8.493	8.528
FMO4	8.826	8.801	8.787	8.816	8.686	8.818
FMO5	7.948	7.934	7.836	8.307	7.766	7.946
FOXN2	9.775	9.703	9.808	9.567	9.645	9.632
SH2D2A	7.700	7.704	7.648	7.501	7.677	7.704
PRR14L	9.554	9.473	9.561	9.242	9.063	9.389
AC010255.3	6.168	6.207	6.091	6.333	6.214	6.270
AC142472.1	7.171	6.932	6.898	7.372	7.089	7.201
RGP1	8.638	8.592	8.627	8.436	8.473	8.523
CCNT2-AS1	6.030	6.061	5.784	6.295	6.135	6.129
AKNA	8.141	8.241	8.219	7.822	8.182	7.996
VENTXP1	3.884	4.074	4.068	4.321	4.134	4.088
AP006333.1	8.447	8.474	8.335	8.633	8.481	8.423
JADE3	8.115	8.192	8.307	8.002	8.046	8.007
JADE2	10.219	10.170	10.263	9.983	10.026	10.012
JADE1	8.993	8.951	9.051	8.886	8.710	8.950
AC019294.3	7.127	6.851	6.598	6.865	7.009	7.029
AC091138.1	5.009	4.718	4.732	4.990	4.917	4.974
AC021443.5	5.621	5.860	5.659	5.569	5.786	5.885
AC021443.1	5.621	5.860	5.659	5.569	5.786	5.885
SPRR2D	7.739	7.577	7.823	7.629	7.793	7.886
SPRR2G	7.920	7.916	7.896	8.038	8.138	8.125
SPRR2A	8.854	8.861	8.745	8.936	8.974	8.879
SPRR2C	5.242	5.488	5.481	5.534	5.544	5.559
SPRR2B	7.739	7.577	7.823	7.629	7.793	7.886
AL121753.1	11.297	11.401	11.520	11.361	11.245	11.313
SHQ1	8.736	8.764	8.717	8.643	8.702	8.753
LINC01622	6.259	6.238	6.346	6.393	6.095	6.315
LINC01620	7.687	7.906	7.731	7.646	7.936	7.856
STX1A	9.022	9.145	9.174	9.047	9.043	9.138
STX1B	7.902	7.952	7.934	7.555	7.869	8.028
AC005523.2	7.422	7.395	7.441	7.070	7.672	7.273
SRGN	6.253	6.138	6.163	6.311	6.228	6.285
SPATA31C2	4.796	4.786	4.698	4.893	4.826	4.896
SPATA31C1	4.796	4.786	4.698	4.893	4.826	4.896
MIR497HG	7.403	7.147	7.087	7.225	7.569	7.440
KRT80	9.112	9.174	9.173	8.970	8.961	9.027
KRT81	8.102	8.057	7.989	8.253	8.300	8.058
KRT82	7.541	7.590	7.327	7.568	7.620	7.388
KRT83	7.572	7.511	7.715	7.761	7.629	7.704
KRT85	8.630	8.730	8.709	8.721	8.913	8.799
TSEN2	7.030	7.088	7.144	6.911	7.153	7.056
CCDC134	7.524	7.471	7.570	7.598	7.666	7.479
CCDC136	6.753	7.038	7.072	6.914	6.790	6.909
CCDC137	9.232	9.160	9.419	9.006	9.167	9.005
FAIM	9.043	8.991	9.181	8.854	8.896	9.008
LMNTD2-AS1	8.383	8.316	8.566	8.702	8.604	8.533
STX11	6.158	5.743	6.072	6.179	6.427	6.225
STX10	10.056	10.058	10.225	10.082	10.035	10.006
STX12	9.975	10.061	10.020	10.018	10.021	9.947
AC245078.1	7.199	7.315	7.449	7.834	7.457	7.479
STX17	8.923	8.974	8.988	8.800	9.061	8.822
STX16	10.328	10.259	10.334	10.075	10.033	10.187
STX19	6.591	6.731	6.651	6.942	7.005	7.044

STX18	9.579	9.654	9.739	9.489	9.411	9.453
LINC00572	6.031	6.255	6.212	6.375	6.440	6.381
LINC00574	7.484	7.706	7.385	7.597	7.740	7.697
MOSPD3	9.415	9.377	9.442	9.175	9.224	9.380
MOSPD2	10.264	10.171	10.106	9.959	10.221	10.105
MOSPD1	9.687	9.740	9.666	9.632	9.605	9.847
PAN3	8.773	8.780	8.772	8.357	8.409	8.723
PAN2	10.934	10.957	11.152	10.857	10.788	10.840
AC004817.1	5.966	6.102	6.179	6.080	5.946	6.038
DPP8	10.998	11.383	11.447	11.148	11.157	10.654
DPP9	8.359	8.281	8.601	8.416	8.488	8.365
DPP3	10.478	10.628	10.699	10.410	10.280	10.454
DPP4	9.962	10.018	10.111	9.995	9.896	10.072
SUOX	10.041	9.998	10.051	10.008	10.047	10.033
DPP6	8.263	8.259	8.267	8.208	8.380	8.230
DPP7	8.104	8.338	8.336	8.159	8.063	8.382
ELAC2	8.355	8.436	8.662	8.313	8.187	8.311
STK38	9.533	9.563	9.692	9.477	9.346	9.499
IK	10.203	10.241	10.416	10.243	10.020	10.083
AC015813.2	11.305	11.351	11.289	11.273	11.310	11.265
AC015813.1	8.690	8.369	8.296	8.556	8.264	8.456
AC141257.1	3.826	3.885	3.772	3.930	3.832	3.885
RO60	10.501	10.275	10.409	10.458	10.160	10.450
HS1BP3	10.135	10.313	10.302	10.127	10.158	10.212
NOC3L	8.953	9.045	9.150	8.864	8.979	9.051
TMIGD2	8.482	8.235	8.293	8.474	8.580	8.403
ZNF300P1	6.245	5.936	5.978	6.167	6.117	6.115
POM121L2	7.913	7.776	7.809	7.682	7.780	7.950
AL353743.1	4.833	4.736	4.964	4.767	4.975	5.036
NDUFB11	10.561	10.672	10.924	10.608	10.497	10.623
NDUFB10	11.321	11.432	11.653	11.216	11.143	11.254
GOT2	11.560	11.528	11.583	11.511	11.444	11.571
GOT1	10.143	10.170	10.254	9.985	10.026	9.989
METTL21EP	5.573	5.517	5.283	5.525	5.366	5.669
AC113418.1	5.056	5.224	5.169	5.186	5.181	5.400
OR2T2	6.846	6.574	6.640	6.713	6.771	6.677
BRAT1	10.195	10.370	10.537	10.070	9.994	10.145
MIR222HG	6.003	5.820	5.745	5.855	5.663	6.085
SH3D21	8.713	8.472	8.730	9.226	8.908	8.927
SCAND2P	7.835	8.026	7.985	7.998	7.870	7.994
C18orf12	7.144	7.273	6.972	7.122	7.311	7.072
C22orf46	7.345	7.552	7.490	7.439	7.378	7.509
C18orf15	7.076	7.214	7.325	7.464	7.348	7.401
AC099518.2	3.946	3.895	4.099	4.043	3.982	4.022
TRAV13-1	4.183	4.138	4.034	4.310	4.152	4.232
TRAV13-2	4.680	4.828	4.805	5.475	4.964	5.155
AC037482.2	5.043	4.814	4.886	5.034	5.252	5.107
AC037482.3	4.043	4.198	4.080	4.135	4.131	4.215
STMN4	7.790	8.064	7.786	8.090	7.905	7.949
STMN1	8.964	9.110	9.255	8.861	8.694	8.868
STMN3	7.593	7.567	7.540	7.429	7.308	7.499
STMN2	5.522	5.690	5.300	5.769	5.615	5.669
IKBIP	8.230	8.205	8.300	7.776	7.739	7.710
AP002907.1	5.118	5.068	4.966	5.217	5.322	5.302
AC022929.2	3.989	3.963	4.073	4.071	4.235	4.088
AP003419.1	10.177	10.217	10.445	10.206	10.164	10.139

APH1B	9.205	9.178	9.191	9.110	9.174	9.215
ABRAXAS2	9.100	9.001	9.031	9.059	9.159	9.015
ABRAXAS1	6.578	6.620	6.966	6.830	6.706	6.506
CRYZL2P	6.209	6.126	6.273	5.837	6.265	6.096
BCAN	8.814	9.009	8.969	9.096	9.083	9.017
BCAM	9.127	9.062	9.162	9.302	9.414	9.114
CYP4F30P	3.859	3.902	4.083	3.848	4.080	3.960
ELOVL6	10.361	10.401	10.504	10.261	10.387	10.395
ELOVL7	10.818	10.853	10.801	10.786	10.555	10.627
ELOVL4	4.829	5.026	4.989	4.932	4.829	4.705
ELOVL5	11.876	11.933	12.004	11.987	11.810	11.943
ELOVL2	6.555	6.601	6.334	6.514	6.492	6.586
ELOVL3	6.694	6.764	6.574	6.885	6.879	6.589
ELOVL1	10.665	10.777	10.942	10.659	10.667	10.711
WNT7B	10.239	10.280	10.570	10.408	10.254	10.267
AMY1A	5.089	5.094	5.291	5.046	4.927	5.253
AC090994.1	4.575	4.459	4.395	4.660	4.489	4.656
CFAP97	10.840	10.870	10.913	10.683	10.771	10.643
AMY1B	5.089	5.094	5.291	5.046	4.927	5.253
AP002761.1	5.939	5.654	5.936	5.760	5.739	5.837
CWF19L1	8.715	8.779	8.793	8.625	8.728	8.669
CWF19L2	6.890	6.805	6.829	6.956	7.050	6.979
HEYL	7.546	7.659	7.816	7.680	7.822	7.765
RD3	6.640	6.711	6.520	6.688	6.426	6.493
AL135960.1	6.305	6.398	6.318	6.514	6.276	6.255
TECR	10.572	10.597	10.870	10.317	10.192	10.372
AC140479.4	7.564	7.798	7.581	7.620	7.841	7.801
DUSP9	6.274	6.537	6.515	6.700	6.690	6.638
DUSP8	9.054	9.063	8.948	9.232	9.244	9.330
AL157871.6	8.523	8.542	8.599	8.631	8.579	8.481
DUSP5	10.259	10.428	10.344	10.462	10.309	10.365
GCSIR	7.333	7.363	7.297	7.462	7.488	7.332
DUSP7	7.171	7.270	7.476	7.311	7.365	7.183
DUSP6	11.599	11.546	11.626	11.532	11.787	11.553
DUSP1	9.586	9.616	9.745	10.874	10.790	10.947
DUSP3	11.470	11.504	11.647	11.490	11.375	11.197
DUSP2	7.906	7.984	8.154	8.179	7.969	8.091
GBGT1	7.576	7.407	7.276	7.597	7.442	7.530
RBL1	7.440	7.138	7.236	6.703	7.091	6.694
RBL2	9.927	9.972	10.184	9.655	9.749	9.814
JPT2	11.957	12.114	12.223	11.944	11.799	11.885
RDX	10.431	10.400	10.446	10.349	10.239	10.266
OR51E2	7.332	7.103	7.167	7.208	7.378	7.304
JPT1	11.793	11.888	12.142	11.693	11.666	11.577
HEY2	7.391	7.189	7.492	7.589	7.393	7.494
RIOX2	9.329	9.206	9.109	8.929	9.189	8.770
RIOX1	9.686	9.826	9.943	9.668	9.766	9.723
HEY1	7.562	7.693	7.213	7.383	7.399	7.609
AC005906.2	7.345	7.522	7.396	7.839	7.320	7.277
RPS10-NUDT3	13.767	13.833	13.946	13.964	13.837	13.963
MTF1	9.057	8.984	9.085	9.055	9.035	8.988
MTF2	10.171	10.153	10.295	10.144	10.111	9.916
TPTE2P2	4.154	4.162	3.913	4.229	4.205	4.166
AC120057.2	12.216	12.262	12.346	12.232	12.172	12.171
TPTE2P6	4.487	4.387	4.312	4.496	4.484	4.219
CSN1S1	5.590	5.405	5.532	5.444	5.502	5.250

FBL	12.801	12.865	12.989	12.712	12.605	12.654
AL359643.3	7.522	7.493	7.359	7.798	7.568	7.730
PARP4P2	11.284	11.299	11.383	11.158	11.019	11.161
BMP10	6.776	6.944	6.904	7.146	7.356	7.111
BMP15	6.627	6.437	6.350	6.797	6.642	6.837
AC243837.1	7.717	7.900	7.748	7.796	7.697	7.761
AC243837.2	10.096	10.073	10.117	10.027	10.081	10.109
TRIM52-AS1	7.588	7.673	7.789	7.415	7.562	7.473
UFL1	9.361	9.010	9.206	8.974	9.147	9.121
TMEM106B	11.713	11.838	11.786	11.774	11.727	11.827
TMEM106C	10.269	10.220	10.457	10.086	10.124	10.066
CNP	11.071	11.086	11.317	11.114	10.987	11.025
TMEM106A	6.822	6.842	6.570	6.469	6.817	6.771
DLG3-AS1	7.354	7.270	7.433	7.505	7.502	7.247
AL590399.4	8.174	8.070	8.261	8.185	8.174	8.173
AL590399.5	4.141	4.060	4.251	4.180	4.213	4.069
AL590399.1	8.597	8.585	8.594	8.520	8.633	8.531
AC010638.1	5.242	5.313	5.085	5.318	5.177	5.148
AL512310.3	3.504	3.628	3.499	3.603	3.486	3.514
RHCG	7.042	7.300	6.943	7.182	7.214	7.194
RHCE	8.276	8.336	8.390	8.350	8.469	8.533
AHNAK2	9.894	9.909	10.005	9.629	9.274	9.541
RIC3	6.840	7.042	6.794	6.809	6.992	6.848
RIC1	7.689	7.663	7.780	7.785	7.752	7.853
AP003080.1	7.176	7.370	7.313	7.486	7.335	7.483
SEMA4A	8.443	8.473	8.611	8.687	8.797	8.675
SEMA4B	11.718	11.690	11.884	11.648	11.636	11.546
SEMA4C	8.845	8.970	9.127	8.887	8.994	9.094
SEMA4D	8.784	8.879	8.960	8.727	8.715	8.604
SEMA4F	9.148	9.120	9.005	9.071	9.077	8.980
SEMA4G	8.811	8.943	8.963	8.819	8.853	9.006
CBLIF	6.255	6.507	6.564	6.586	6.653	6.678
MEDAG	8.791	8.873	8.779	8.577	8.680	8.472
SP110	11.400	11.358	11.364	11.131	11.322	11.069
SCN4B	5.968	6.015	5.579	6.368	6.011	6.153
SUMO3	11.018	11.106	11.199	10.880	10.918	10.872
TMCO3	9.757	9.794	9.819	9.703	9.653	9.723
FGF14-IT1	5.268	5.401	5.327	5.348	5.340	5.442
AC005083.1	8.689	8.828	8.748	8.638	8.892	8.881
AL392086.3	4.367	4.289	4.356	4.292	4.383	4.475
RGMB-AS1	6.252	6.463	6.341	6.522	6.295	6.424
ACTA2	7.660	7.756	7.899	7.817	7.818	7.803
GTF2H2B	8.310	8.418	8.490	8.307	8.382	8.244
ACTA1	6.742	6.549	6.656	7.051	6.869	6.628
PDS5A	9.095	8.985	9.020	8.881	8.682	8.951
PDS5B	9.085	8.953	8.964	8.648	8.683	8.724
TSTD1	10.583	10.616	10.860	10.473	10.552	10.477
TSTD2	8.543	8.433	8.542	8.157	8.079	8.443
TSTD3	6.814	6.669	6.568	6.851	6.618	6.497
RSBN1L	8.432	8.314	8.435	8.453	8.166	8.386
UBXN2B	7.989	7.966	7.793	7.698	7.627	7.666
UBXN2A	8.729	8.884	8.942	8.714	8.961	8.596
AP000550.1	7.431	7.554	7.493	7.569	7.741	7.624
AP000550.4	9.620	9.633	9.591	9.534	9.791	9.460
EDARADD	6.988	7.146	6.787	7.191	7.237	7.237
ZNF221	7.034	6.942	6.920	7.194	7.149	6.872

ZNF222	7.540	7.700	7.589	7.476	7.517	7.707
ZNF223	7.171	7.041	7.147	7.050	7.228	6.910
ZNF224	7.750	7.451	7.478	7.771	7.704	7.779
ZNF226	8.532	8.540	8.643	8.522	8.313	8.365
ZNF227	8.341	8.282	8.443	8.061	8.084	8.126
ZNF229	5.295	5.356	5.527	5.473	5.396	5.347
KCNQ4	6.705	6.587	6.882	6.886	6.847	6.757
KCNQ5	5.569	5.435	5.381	5.598	5.575	5.650
KCNQ2	7.455	7.510	7.540	7.592	7.608	7.479
KCNQ3	8.150	8.190	7.969	8.192	8.337	8.242
KCNQ1	8.748	8.636	8.722	8.585	8.738	8.771
HOXC11	8.562	8.596	8.591	8.687	8.688	8.735
HOXC10	7.005	6.912	6.543	6.822	6.954	6.974
HOXC13	6.314	6.434	6.504	6.508	6.424	6.490
HOXC12	5.468	5.684	5.760	6.228	5.767	5.818
PDE4A	7.231	7.207	7.387	7.548	7.364	7.199
AC097382.3	5.260	5.383	5.583	5.776	5.535	5.633
RTTN	8.433	8.444	8.153	7.971	8.407	8.478
METTL15P1	6.761	6.930	7.001	6.824	6.793	6.866
KIR2DL3	7.778	7.899	7.866	7.730	7.785	7.897
KIR2DL1	8.115	8.042	7.866	8.213	8.188	8.126
KIR2DL4	6.952	7.170	7.148	7.083	7.272	7.464
OTUD5	10.845	10.895	10.938	10.888	10.911	10.891
OTUD4	9.468	9.481	9.459	9.317	9.105	9.303
OTUD3	7.533	7.577	7.400	7.736	7.454	7.587
OTUD1	8.554	8.476	8.607	8.106	8.155	8.198
SPEM1	7.635	7.627	7.819	7.858	7.811	7.839
SPEM2	4.153	4.265	4.206	4.353	4.383	4.339
HTRA4	6.456	6.408	6.301	6.301	6.427	6.589
HTRA1	7.873	7.790	7.789	7.803	7.872	7.857
HTRA2	9.721	9.629	9.815	9.580	9.647	9.649
HTRA3	8.242	8.275	8.324	8.423	8.458	8.330
SPI1	5.602	5.909	5.918	6.121	6.013	6.147
UNC5CL	7.377	7.488	7.569	7.301	7.122	7.434
MIR378I	8.212	8.401	8.318	8.218	8.439	8.400
AC007229.1	7.810	7.980	8.091	7.755	7.792	7.976
AC136759.2	5.367	5.231	5.211	5.384	5.336	5.373
AC108471.3	4.779	4.881	4.748	4.650	4.931	4.890
C17orf82	8.857	8.923	8.966	9.062	9.008	8.976
C17orf80	8.447	8.537	8.583	8.455	8.500	8.500
SPIB	7.223	7.198	7.283	7.343	7.356	7.394
SPIC	3.903	3.891	3.914	3.829	4.035	3.874
LINC01359	7.842	7.745	7.297	7.616	7.755	7.555
AC079089.1	6.982	6.983	6.743	7.107	7.068	6.954
LINC01357	6.598	6.675	6.633	6.770	6.679	6.742
LINC01354	4.454	4.090	4.209	4.244	4.479	4.210
LINC01355	6.790	6.796	6.900	6.630	6.916	6.829
LINC01352	5.892	6.118	5.940	5.923	5.787	6.295
LINC01350	5.702	5.961	5.883	6.471	5.932	5.978
LINC01351	5.188	5.396	5.388	5.607	5.715	5.275
AC120114.3	7.043	6.995	6.542	6.473	6.917	6.456
AC120114.1	5.712	5.660	5.607	5.550	5.685	5.559
AC120114.4	9.870	9.936	10.043	9.909	9.829	9.846
SPCS1	12.220	12.319	12.427	12.228	12.208	12.326
SPCS2	12.079	12.145	12.232	12.152	12.137	12.103
SPCS3	10.118	10.187	10.319	10.065	9.998	10.099

EMID1	8.065	8.272	8.300	8.253	8.391	8.181
RCVRN	6.594	6.769	6.892	6.846	7.023	7.012
SIM2	7.835	7.920	7.892	7.785	8.022	7.855
SIM1	5.894	6.072	5.803	5.813	6.097	6.027
ACER1	6.065	6.085	6.326	6.304	5.888	6.159
PEA15	10.995	11.072	11.183	11.061	11.143	10.930
MAN1B1-DT	8.507	8.480	8.504	8.034	8.189	8.296
AC108002.2	6.515	6.574	6.354	6.778	6.755	6.856
LINC01970	6.164	6.070	6.087	6.053	6.134	6.097
LINC01977	5.949	5.748	6.010	5.813	5.946	5.929
LINC01974	6.432	6.366	6.314	6.660	6.445	6.472
AC234779.1	7.292	7.258	7.348	7.267	7.330	7.457
BNC1	5.392	5.433	5.432	5.273	5.650	5.517
BNC2	7.361	7.440	7.491	7.398	7.735	7.533
GNA14-AS1	5.668	5.890	5.585	6.112	5.699	5.508
MGAT4D	4.866	4.778	4.695	4.978	5.202	5.021
MGAT4A	8.659	8.627	8.663	8.598	8.449	8.627
MGAT4C	4.122	3.917	4.048	4.024	4.144	4.010
MGAT4B	11.984	12.002	12.105	11.694	11.496	11.720
BZW1	11.434	11.543	11.562	11.441	11.293	11.471
BZW2	12.012	12.104	12.181	12.147	12.062	11.986
RAD17	8.946	9.104	9.062	8.981	8.972	8.860
RAD18	7.793	7.802	7.686	7.692	7.624	7.672
AC064807.1	6.510	6.431	6.475	6.418	6.426	6.576
STXBP5-AS1	5.505	5.461	5.756	5.452	5.569	5.630
TNNC1	6.972	7.188	7.626	7.172	7.058	7.353
TNNC2	7.484	7.403	7.766	7.552	7.897	7.753
CYP3A7	8.449	8.341	8.428	8.482	8.452	8.608
AFAP1-AS1	9.221	9.353	9.354	9.189	9.100	9.204
CXCL13	5.070	4.794	5.006	5.447	5.164	5.083
CYP3A5	8.210	8.333	8.080	8.459	8.450	8.553
TOX4P1	8.275	8.099	8.218	7.717	7.928	8.163
GMCL2	8.815	8.462	8.436	7.953	8.457	8.210
GMCL1	10.603	10.460	10.681	10.061	10.221	10.191
TBX2	7.231	7.310	7.450	7.443	7.628	7.507
MAPK8IP1P2	8.057	8.183	8.204	8.272	8.039	8.091
MAPK8IP1P1	8.057	8.183	8.204	8.272	8.039	8.091
DMAC2	10.477	10.586	10.712	10.328	10.326	10.332
DMAC1	10.888	11.044	11.210	10.983	10.992	10.980
UBE4B	9.023	9.119	9.100	9.089	8.982	9.102
ZG16	7.696	7.780	7.904	7.706	7.635	7.731
LDB2	5.443	5.644	5.021	5.356	5.436	5.158
LDB3	7.029	7.144	7.308	7.476	7.296	7.288
RUBCNL	5.737	5.819	5.742	5.780	5.893	5.734
LDB1	8.788	8.734	8.759	8.877	8.833	8.851
SNU13	11.626	11.731	11.833	11.590	11.547	11.500
TRPC2	7.015	7.364	7.110	7.037	7.387	7.494
PDXDC1	11.646	11.751	11.824	11.601	11.406	11.533
TRPC3	4.326	4.243	4.262	4.555	4.561	4.461
RPLP2P1	7.295	7.288	7.313	7.230	7.553	7.409
TSPY26P	7.862	7.775	7.730	7.909	7.923	7.952
PLEKHA4	8.243	8.162	8.350	8.454	8.428	8.245
PLEKHA5	10.407	10.403	10.455	10.317	10.527	10.245
PLEKHA6	8.947	8.956	8.943	8.848	8.706	8.862
ADGRG4	6.045	6.044	5.937	6.241	5.957	6.008
ADGRG3	7.138	7.222	7.413	7.250	7.222	7.449

PLEKHA1	10.533	10.288	10.328	10.095	9.990	10.294
PLEKHA2	10.147	10.144	10.178	10.110	9.971	10.070
PLEKHA3	9.389	9.395	9.403	9.125	9.137	9.226
AC025674.2	4.509	4.255	4.248	4.300	4.233	4.442
PLEKHA8	8.383	8.352	8.322	7.977	8.066	8.229
DEFT1P	5.904	6.100	6.027	5.989	5.957	6.028
AC079035.1	4.955	4.876	4.698	4.868	4.783	5.027
TRPC5	4.406	4.516	4.477	4.404	4.348	4.463
SEC62	11.919	11.937	11.955	11.888	11.779	11.906
SEC63	10.418	10.212	10.202	9.722	10.417	10.266
MGAM	4.879	4.778	4.895	5.039	5.005	4.906
B3GNTL1	7.051	6.807	6.873	7.183	7.223	7.163
VN1R2	5.979	5.875	5.760	5.903	5.499	6.067
TPTE	4.289	4.521	4.101	4.378	4.376	4.379
VN1R1	5.964	5.729	5.686	5.930	5.976	5.784
VN1R4	5.688	5.713	5.630	5.580	5.656	5.832
VN1R5	6.614	6.660	6.748	6.691	6.746	6.723
TPT1	14.364	14.418	14.399	14.447	14.483	14.528
AL139300.1	8.859	8.964	9.006	8.645	8.433	8.454
AL359547.1	7.946	7.935	7.783	7.952	7.894	7.853
FAM185A	5.908	5.813	5.791	5.468	5.770	5.642
IGKV1OR2-118	8.398	8.520	8.424	8.478	8.571	8.522
AL683842.1	7.152	7.314	7.181	7.311	7.531	7.550
AC087897.2	5.335	5.848	5.958	5.980	5.814	5.914
POTEB2	3.830	4.000	4.066	3.900	3.760	4.082
POTEB3	3.830	4.000	4.066	3.900	3.760	4.082
MYH2	6.318	5.884	5.969	6.065	6.323	6.225
MYH3	5.524	5.341	5.343	5.525	5.543	5.569
MYH1	4.138	4.120	4.090	4.015	4.088	4.043
MYH6	5.915	6.158	6.281	5.977	6.265	6.106
MYH7	6.348	6.524	6.366	6.433	6.470	6.362
MYH4	5.227	4.915	4.916	4.994	5.046	5.174
MYH8	6.716	6.752	6.672	6.908	6.710	6.876
MYH9	11.712	11.740	11.887	11.705	11.505	11.641
BAGE2	8.288	8.441	8.451	8.473	8.807	8.519
AC023055.1	9.594	9.536	9.553	9.254	9.088	9.455
BACE1	9.806	9.848	9.876	9.792	9.750	9.916
VGLL2	7.642	7.731	7.537	7.735	7.651	7.599
VGLL3	9.239	9.297	9.199	8.987	8.837	9.170
CADM4	7.510	7.574	7.599	7.889	7.669	7.697
VGLL1	11.129	11.287	11.410	11.003	10.863	10.995
CADM2	6.237	6.417	6.265	6.254	6.084	6.430
CADM3	8.362	8.486	8.544	8.547	8.477	8.464
VGLL4	9.992	10.025	10.338	10.005	10.250	9.771
CADM1	7.414	7.390	7.574	7.817	7.545	7.594
SMIM2-AS1	5.068	5.330	5.128	5.309	5.441	5.243
TMEM147-AS1	8.180	8.099	7.960	8.216	8.056	8.163
SATB2	7.520	7.605	7.478	7.575	7.422	7.559
SATB1	8.980	8.717	8.713	8.828	8.714	8.893
FAM149B1	7.778	7.909	7.862	7.762	7.685	7.763
CNNM1	8.066	7.990	8.119	7.939	8.029	8.044
CNNM3	9.985	10.044	10.225	9.890	10.101	9.660
CNNM2	8.653	8.909	8.741	8.757	8.917	8.734
CNNM4	8.725	8.907	8.881	8.668	8.506	8.637
SPATA3-AS1	6.895	6.964	7.085	7.184	7.179	7.219
PIP5K1C	9.269	9.348	9.486	9.261	9.244	9.286

PIP5K1B	8.624	8.826	8.967	8.545	8.838	8.801
PIP5K1A	10.218	10.514	10.443	10.613	10.466	10.283
LINC02091	7.419	7.545	7.403	7.515	7.519	7.615
LINC02092	8.001	7.982	8.215	8.277	8.268	8.306
LINC02097	6.396	6.429	6.376	6.270	6.533	6.500
LINC02099	5.054	5.195	5.219	5.224	5.214	5.377
PHF14	8.878	8.927	8.920	8.782	8.889	8.753
PHF10	9.241	9.354	9.367	9.289	9.362	9.332
PHF11	9.186	9.136	9.196	9.072	9.307	9.161
PHF12	8.545	8.464	8.543	8.189	8.358	8.449
PHF13	9.017	9.118	9.231	8.782	8.824	8.976
PHF19	8.819	8.708	8.755	8.350	8.553	8.470
TIA1	9.308	9.170	9.203	9.284	9.273	9.293
LDC1P	8.489	8.383	8.110	8.522	8.573	8.530
UBN1	9.771	9.477	9.795	9.544	9.889	9.427
PNLIPRP1	7.685	7.719	7.656	7.897	7.959	8.069
PNLIPRP3	4.638	4.724	4.688	4.764	4.690	4.980
PNLIPRP2	4.627	4.416	4.354	4.569	4.578	4.531
PITHD1	9.588	9.694	9.777	9.626	9.601	9.532
ALX3	6.963	7.276	7.035	7.476	7.287	7.214
ALX1	7.161	6.672	7.074	6.939	7.057	7.024
ALX4	6.604	6.740	6.673	6.829	6.830	6.929
LINC02546	4.996	4.953	5.050	5.119	4.876	5.044
LINC02544	7.175	7.204	7.052	7.128	7.198	7.015
CTBP2P4	11.522	11.601	11.721	11.679	11.730	11.416
CTBP2P8	11.522	11.601	11.721	11.679	11.730	11.416
LINC02549	5.353	5.419	5.552	5.389	5.431	5.476
CYP21A2	7.212	7.474	7.435	7.470	7.513	7.440
S100PBP	8.425	8.405	8.499	8.204	8.380	8.406
SYNGAP1	8.255	7.920	8.356	8.613	8.311	8.302
COL24A1	4.253	4.073	4.176	4.356	4.193	4.138
GPR39	9.129	9.021	9.072	8.793	8.667	9.009
BFSP2-AS1	7.151	7.306	7.345	7.054	7.109	7.241
GPR31	7.013	7.078	7.123	7.012	7.278	6.944
GPR32	6.983	7.037	7.065	7.462	7.020	7.068
GPR35	9.985	10.040	10.252	9.896	9.935	9.993
GPR34	5.489	5.588	5.257	5.634	5.490	5.694
GPR37	8.225	8.180	8.400	8.164	8.316	8.107
SUGT1	11.154	11.144	11.390	11.158	11.316	11.198
LINC01019	5.520	5.594	5.498	5.474	5.358	5.628
LINC01018	6.701	6.803	6.545	6.469	6.968	6.849
AL078605.3	7.185	7.101	7.094	6.947	7.052	6.903
ZC3HAV1	9.942	10.273	10.175	10.298	10.576	10.050
AL078605.1	7.185	7.101	7.094	6.947	7.052	6.903
DGCR6L	10.503	10.564	10.866	10.550	10.504	10.580
LINC01013	6.418	6.318	6.550	6.496	6.422	6.720
LINC01015	6.997	6.987	7.047	6.974	7.098	7.071
LINC01016	10.417	10.624	10.271	10.358	10.655	10.516
TBC1D25	9.357	9.407	9.466	9.426	9.425	9.540
TBC1D24	8.701	8.581	8.723	8.307	8.685	8.167
AL023284.4	6.890	6.939	6.952	6.675	6.930	6.880
AL356968.2	8.774	8.788	8.855	8.785	8.784	8.777
PHC1P1	8.829	8.816	8.734	8.559	8.611	8.730
PRAMEF32P	7.406	7.569	7.636	7.983	7.893	7.730
H3-2	4.798	4.855	5.126	5.060	4.953	4.904
WASH9P	9.341	9.202	9.345	9.278	9.301	9.395

MEAK7	8.097	7.994	8.088	7.835	7.809	7.802
SCEL	7.408	7.706	7.461	7.135	7.080	7.348
CC2D1A	8.258	8.367	8.424	8.197	8.370	8.163
CC2D1B	8.485	8.645	8.783	8.482	8.570	8.408
ZNF70	8.214	8.351	8.109	8.008	8.276	8.056
NKAPP1	7.144	6.846	7.122	6.958	7.090	7.069
GK2	5.848	6.002	5.738	5.687	6.139	5.936
GK5	7.041	6.988	6.922	6.147	6.286	6.735
CCER1	6.084	6.095	6.088	6.224	6.181	6.192
H3-4	6.216	6.162	6.235	5.976	6.425	6.247
AL390195.2	8.965	9.142	9.059	8.978	8.932	8.987
AL390195.3	5.665	6.022	5.825	6.117	5.665	5.883
PTPDC1	7.217	7.379	7.410	7.192	7.381	7.257
BCCIP	9.152	9.129	9.206	8.997	8.938	8.843
CPEB1-AS1	6.342	6.358	6.308	6.466	6.278	6.340
NADK	10.393	10.346	10.547	10.154	10.064	10.057
KRT8P3	13.948	14.005	14.050	14.066	14.014	14.045
CRYAA2	7.086	7.310	7.555	7.262	7.244	7.349
PUS10	7.350	7.395	7.487	7.195	7.137	7.334
VASH2	7.378	7.525	7.482	7.349	7.511	7.562
VASH1	7.886	8.031	7.918	8.085	8.097	8.199
CTDSPL2	8.916	8.952	9.079	8.759	8.808	8.742
AC114936.1	10.796	10.924	11.020	10.731	10.555	10.739
DIP2A	9.192	9.049	9.055	9.083	9.041	9.285
DIP2B	9.425	9.487	9.442	9.030	9.144	9.277
DIP2C	8.991	8.961	8.698	8.799	8.777	9.159
AC005696.4	7.856	7.884	7.950	8.168	7.946	7.887
AC007292.2	7.319	7.436	7.330	7.481	7.489	7.604
AC007383.2	8.513	8.552	8.655	8.273	8.241	8.372
MIR7515HG	6.885	6.740	6.527	6.981	6.887	6.850
WDR83OS	10.625	10.695	10.830	10.628	10.505	10.548
ADPRHL1	7.773	7.828	7.688	7.946	8.009	7.872
ADPRHL2	9.866	9.892	10.059	9.826	9.784	9.703
CEP85L	6.391	6.343	6.688	6.461	6.542	6.526
AHCYL1	10.957	11.144	11.311	11.233	11.304	10.938
AHCYL2	9.114	9.311	9.255	8.956	9.177	9.131
STUB1	12.040	12.168	12.299	12.103	11.930	12.141
A4GNT	7.285	7.324	7.226	7.273	7.534	7.410
AC002985.1	7.617	7.563	7.842	7.450	7.404	7.559
CAPN7	7.957	8.019	8.124	7.844	7.920	7.792
CAPN6	6.038	6.033	5.821	6.231	6.165	6.014
CAPN5	10.012	9.979	10.182	9.949	10.054	9.986
CAPN3	7.120	7.136	7.288	7.300	7.122	7.328
CAPN2	12.557	12.654	12.711	12.587	12.422	12.585
CAPN1	10.569	10.606	10.706	10.612	10.533	10.487
NOD1	8.414	8.236	8.284	8.312	8.550	8.563
DLEU1	9.272	9.257	9.265	9.598	9.361	9.340
CAPN9	6.546	6.564	6.617	6.821	6.670	6.622
CAPN8	9.753	9.824	9.770	9.645	9.402	9.551
PRRC2C	10.860	10.918	10.823	10.644	10.624	10.804
PRRC2B	10.193	10.066	10.202	9.872	9.986	10.016
PRRC2A	9.881	9.845	9.926	9.869	9.743	9.761
MPIG6B	5.865	5.813	6.073	6.093	6.152	6.296
HBG2	7.191	7.300	7.218	7.328	7.503	7.422
AL135902.1	6.993	7.059	7.128	7.273	7.067	7.169
FAXDC2	11.486	11.577	11.511	11.421	11.537	11.629

AANAT	6.321	6.431	6.354	6.272	6.344	6.297
AC099506.1	6.430	6.540	6.436	6.591	6.619	6.456
CDV3	11.819	11.893	11.937	11.939	11.837	11.918
RPL23AP74	14.341	14.360	14.355	14.364	14.332	14.374
SOHLH1	7.834	7.789	7.660	7.703	7.882	7.824
SOHLH2	7.204	7.055	6.862	7.189	7.429	7.353
CMAS	8.367	8.566	8.546	7.733	8.018	8.092
AL683813.2	6.301	6.293	6.323	6.306	6.325	6.285
GRAMD4	9.119	9.230	9.278	8.831	8.849	8.936
AC099521.2	4.072	4.047	3.875	3.879	3.889	4.040
TRAPPC3L	4.744	4.650	4.759	4.740	4.871	4.656
CMA1	5.959	6.186	5.861	5.948	6.031	6.126
AC124312.4	7.614	7.618	7.490	7.729	7.716	7.734
AC124312.3	6.639	6.757	6.724	6.961	6.931	6.798
AC124312.1	6.185	6.335	6.344	5.986	6.220	6.019
POMGNT1	8.893	8.924	9.103	8.643	8.838	8.783
POMGNT2	8.678	8.903	9.075	8.673	8.527	8.622
SLC6A10P	8.827	8.879	8.945	8.881	8.964	8.865
TKFC	8.814	9.010	8.938	8.678	8.613	8.599
TUBBP5	6.990	6.958	7.190	6.745	6.890	6.895
TUBBP2	13.281	13.318	13.351	13.119	13.047	13.095
RSKR	5.919	5.782	5.470	5.990	5.881	5.822
CLDN12	8.830	8.835	9.030	8.718	8.605	8.713
CLDN10	10.980	11.015	11.231	10.820	10.810	10.873
CLDN11	7.724	7.620	7.547	7.543	7.717	7.545
CLDN16	7.638	8.007	7.582	7.575	7.611	7.486
CLDN17	8.286	8.548	8.335	8.133	8.445	8.440
CLDN14	5.849	5.649	6.055	5.996	6.260	6.076
CLDN15	9.523	9.567	9.612	9.507	9.547	9.612
CLDN18	7.927	7.698	7.579	8.040	7.928	7.823
CLDN19	8.730	8.694	8.834	8.724	8.802	8.745
HYI-AS1	3.911	3.774	3.711	3.815	4.075	3.921
LCE3D	7.337	7.290	7.341	7.338	7.378	7.390
PHIP	9.762	9.662	9.693	9.489	9.487	9.575
ZNF521	4.975	5.023	5.115	4.968	5.162	4.989
ZCRB1	9.920	9.836	10.217	10.054	9.857	9.764
ZNF527	8.585	8.485	8.416	8.279	8.300	8.448
ZNF526	8.837	8.995	9.081	8.858	9.068	8.943
ZNF524	8.497	8.549	8.774	8.961	8.788	8.614
ZNF529	7.889	7.864	7.822	7.740	7.787	7.879
ZNF528	7.807	7.820	7.994	7.965	8.110	7.978
JRK	9.979	9.986	10.021	9.904	9.757	9.945
CYP2T1P	6.945	7.094	6.883	6.867	6.969	6.993
DBIL5P2	6.587	6.446	6.055	6.518	6.573	6.318
RNF144B	8.520	8.618	8.614	8.383	8.458	8.460
RNF144A	8.719	8.786	8.691	8.490	8.518	8.486
AC138356.2	6.774	6.894	6.945	7.153	7.060	7.101
AC138356.3	4.459	4.567	4.551	4.765	4.914	4.607
AC138356.1	7.384	7.497	7.425	7.333	7.471	7.555
BCL11A	8.045	8.054	8.058	8.114	8.168	8.022
BCL11B	5.349	5.337	5.314	5.258	5.554	5.520
AL358074.1	6.211	6.133	5.684	6.139	6.023	6.192
AC091060.1	4.162	4.137	3.899	4.240	4.116	4.132
C1GALT1	9.869	9.897	9.925	9.726	10.034	9.831
MIR5087	4.675	4.945	4.760	4.770	4.684	4.808
RAX2	7.827	7.944	7.984	8.020	7.828	8.031

PABPN1	11.770	11.818	12.043	11.535	11.668	11.517
DPY19L3	8.076	8.073	8.058	7.678	7.666	8.005
PIM2	8.514	8.514	8.638	8.521	8.431	8.523
PIM3	10.410	10.494	10.697	10.742	10.641	10.625
PIM1	7.783	8.116	8.079	8.139	7.976	8.055
MAN1C1	7.377	7.395	7.408	7.430	7.477	7.596
MIR21	10.769	10.425	10.480	10.782	10.632	10.895
MIR25	10.120	10.184	10.253	9.752	9.700	9.810
LINC02708	7.447	7.425	7.491	7.568	7.606	7.459
LINC02709	7.539	7.819	7.744	7.625	7.780	7.646
FAM138C	6.874	7.146	6.938	6.912	7.237	6.777
FAM138B	6.874	7.146	6.938	6.912	7.237	6.777
FAM138A	6.874	7.146	6.938	6.912	7.237	6.777
FAM138F	6.874	7.146	6.938	6.912	7.237	6.777
FAM138E	6.874	7.146	6.938	6.912	7.237	6.777
FAM138D	6.874	7.146	6.938	6.912	7.237	6.777
GORAB	7.793	7.800	7.890	7.793	7.828	8.031
LTB4R2	8.371	8.233	8.344	8.251	8.175	8.217
POLG2	8.180	8.064	8.103	7.983	7.821	7.909
FKBP4	11.160	11.240	11.357	11.296	11.254	11.180
FKBP5	9.021	8.966	8.947	8.683	8.830	8.666
FKBP6	7.981	8.002	7.973	8.380	8.173	7.961
FKBP7	6.246	6.287	6.527	6.135	6.010	6.557
LINC00708	5.033	4.698	4.818	5.038	5.147	4.915
FKBP3	10.583	10.709	10.818	10.585	10.826	10.439
LINC00705	7.364	7.559	7.349	7.468	7.679	7.563
LINC00702	5.124	5.355	5.048	5.223	5.303	5.206
FKBP9	11.203	11.183	11.321	11.147	11.146	11.053
LINC00700	5.575	5.630	5.818	5.856	5.932	5.906
AL139352.1	7.824	7.864	7.781	7.920	7.930	7.931
TYROBP	5.520	5.568	5.462	5.726	5.417	5.697
AL023755.1	8.809	8.749	8.552	8.615	8.786	8.730
AC012321.1	9.458	9.375	9.395	9.152	9.163	9.436
AC087203.3	7.432	7.219	7.300	7.196	7.238	7.473
AC108941.2	6.374	6.433	6.560	6.187	6.493	6.494
IFNA13	9.392	8.929	9.080	9.326	9.257	9.306
AC105235.1	6.809	6.555	6.426	6.774	6.722	6.620
IFNA17	8.082	8.266	7.972	7.982	8.096	8.166
IFNA16	6.266	6.381	6.052	6.329	6.403	6.205
IFNA14	6.266	6.381	6.052	6.329	6.403	6.205
AC008403.1	6.887	6.865	6.900	6.635	6.965	6.970
MAN2C1	9.471	9.619	9.745	9.438	9.354	9.432
FKBPL	7.942	8.053	8.237	7.943	7.966	7.974
GM2A	9.806	9.821	9.922	9.578	9.545	9.576
FDXACB1	7.018	7.316	7.062	7.235	7.242	7.333
AL136962.1	6.440	6.463	6.484	6.912	6.715	6.673
PRRC1	10.543	10.573	10.554	10.371	10.510	10.433
LAT	7.885	7.783	7.679	7.827	7.734	7.751
AC009245.1	13.671	13.713	13.720	13.661	13.586	13.672
TBCE	9.149	9.283	9.321	9.200	9.201	9.129
RASSF8-AS1	6.393	6.189	6.234	6.605	6.650	6.480
AL713998.4	5.122	5.105	5.038	5.306	5.252	4.992
TBCB	11.741	11.882	11.939	11.703	11.702	11.590
TBCA	12.149	12.164	12.246	11.882	11.970	12.050
PANK1	8.112	8.233	8.104	7.980	7.877	8.119
NPNT	10.137	10.152	10.222	9.744	9.704	9.889

PANK3	10.309	10.412	10.323	10.148	10.226	10.347
PANK2	9.162	9.118	9.278	8.915	8.944	9.010
PANK4	7.653	7.786	7.902	7.556	7.527	7.615
ETAA1	7.267	7.315	7.356	6.948	6.938	7.214
FAM181A-AS1	6.646	6.873	6.674	6.899	7.029	6.739
CYP51A1-AS1	5.192	5.209	5.129	5.333	5.283	5.231
SLC26A7	7.095	7.064	6.710	7.410	7.090	7.111
AC005410.2	7.778	7.941	7.950	8.023	8.125	8.020
NFASC	7.771	7.915	8.024	8.372	8.151	8.026
AP001626.1	7.213	7.308	7.379	7.294	7.338	7.580
MTX1P1	10.838	10.813	11.024	10.681	10.718	10.575
FAM167A-AS1	6.017	6.055	5.991	5.908	6.109	6.053
TPD52	11.189	11.241	11.287	11.139	11.218	11.100
GPX3	7.230	6.802	6.754	7.171	7.196	7.257
GPX2	11.807	11.815	12.015	11.851	11.804	11.796
GPX5	5.422	5.498	5.591	5.456	5.469	5.531
GPX4	11.618	11.646	11.865	11.533	11.480	11.460
GPX7	6.001	5.848	6.010	5.733	6.014	5.967
GPX8	8.829	8.844	9.005	8.848	8.892	8.991
AL358613.3	7.740	7.873	7.831	7.890	7.918	7.861
RCAN1	9.834	9.800	9.919	9.758	9.828	9.743
RCAN3	8.452	8.461	8.593	8.165	8.042	8.158
RCAN2	6.399	6.283	6.410	6.699	6.464	6.372
BRIX1	7.941	7.960	7.928	7.900	8.182	8.009
IBA57-DT	5.840	5.963	5.691	5.711	6.070	5.967
RPS26P8	12.821	12.916	13.024	12.923	12.903	12.978
PARVB	8.653	8.721	8.725	8.718	8.712	8.723
PARVA	8.719	8.769	8.881	8.616	8.691	8.505
PARVG	8.037	8.005	7.672	7.822	8.080	8.020
MIR122	4.651	4.762	4.611	4.802	4.592	4.718
BEND6	4.722	4.855	4.627	4.904	4.640	4.682
BEND7	7.006	7.020	6.491	7.165	6.755	6.794
BEND4	4.876	5.078	5.018	5.064	4.919	4.958
BEND5	5.813	5.895	6.079	6.125	6.064	6.011
BEND2	4.858	4.624	4.743	4.949	4.815	4.851
BEND3	8.480	8.514	8.463	8.429	8.446	8.476
MIR6748	10.985	10.923	11.145	11.014	10.861	10.954
AL133173.2	9.106	9.330	9.270	9.257	9.359	9.374
P4HA3-AS1	7.361	7.344	7.390	7.626	7.545	7.722
OPRM1	7.309	7.197	6.835	7.363	7.175	7.465
CNEP1R1	9.185	9.235	9.286	9.059	9.115	9.107
GRAMD1C	6.518	6.699	6.603	6.416	6.557	6.567
GRAMD1B	7.283	7.092	7.261	7.087	7.258	7.190
GRAMD1A	8.888	8.873	9.123	8.813	8.777	8.921
YAP1	10.612	10.618	10.589	10.546	10.483	10.421
NTS	7.932	7.850	7.791	7.644	7.651	7.611
NTM	6.691	6.348	6.309	6.733	6.648	6.709
TUBGCP2	9.404	9.500	9.699	9.342	9.257	9.277
TUBGCP3	6.720	6.735	6.761	6.149	6.372	6.195
TUBGCP4	9.202	9.062	8.827	9.041	9.076	9.211
TUBGCP5	5.779	5.937	6.124	5.919	5.710	6.022
TUBGCP6	7.665	7.858	7.711	7.765	7.847	7.894
RBMS2P1	9.110	8.961	9.060	9.000	9.064	9.101
FLAD1	9.087	9.229	9.268	9.158	9.134	9.075
AC005837.1	7.130	7.141	6.947	7.078	7.150	7.107
MTND4P12	14.452	14.479	14.426	14.490	14.644	14.603

PROP1	7.795	7.753	7.759	8.057	7.876	7.901
LINC01356	6.949	6.861	6.716	7.027	7.166	6.953
BANP	9.351	9.282	9.459	9.392	9.125	9.421
PSMA3-AS1	8.646	8.606	8.467	8.623	8.285	8.659
MCFD2	10.767	10.989	11.008	10.791	10.758	10.897
ST6GALNAC5	5.794	6.015	5.977	5.719	5.666	5.650
ST6GALNAC4	8.089	8.174	8.266	8.021	8.274	8.177
ST6GALNAC3	5.488	5.347	5.131	5.343	5.319	5.379
ST6GALNAC2	8.709	8.685	8.877	8.666	9.009	8.801
ST6GALNAC1	10.340	10.413	10.530	10.125	9.998	10.124
KIF13A	9.195	9.212	9.176	8.924	8.961	8.965
KIF13B	10.984	11.036	11.063	10.582	10.459	10.751
AVPR1B	8.453	8.562	8.611	8.640	8.641	8.785
AVPR1A	8.112	8.166	8.093	8.016	8.334	8.223
TGDS	7.656	7.688	7.925	8.030	7.921	7.908
CELP	7.251	7.059	7.248	7.364	7.510	7.538
HPS5	9.357	9.411	9.410	9.256	9.504	9.013
HPS4	8.318	8.170	8.410	8.161	8.230	8.243
HPS6	9.063	9.125	9.150	8.990	9.109	9.049
HPS1	8.827	8.843	8.988	8.841	8.822	8.864
HPS3	10.686	10.809	10.811	10.844	10.904	10.751
AL513175.1	10.094	10.213	10.182	10.294	10.154	10.278
ROPN1B	7.349	7.622	7.194	7.343	7.300	7.324
ROPN1L	5.931	6.134	5.951	5.890	5.945	6.244
GABPB1-IT1	7.350	7.283	7.396	7.127	7.084	7.043
UCP3	8.168	8.150	8.241	8.446	8.165	8.147
AC105219.1	6.804	6.950	6.863	6.808	6.797	6.983
AC108488.1	7.516	7.410	7.331	7.357	7.414	7.408
NCKIPSD	9.610	9.592	9.686	9.511	9.478	9.491
MCHR2	7.644	7.622	7.701	7.755	7.896	7.801
MCHR1	6.424	6.516	6.508	6.389	6.678	6.548
CACHD1	5.943	5.988	5.653	6.169	6.030	5.962
AL049650.2	5.635	5.658	5.835	5.875	5.575	5.748
AL049697.1	9.175	9.233	9.283	9.171	9.178	9.270
HPSE	8.351	8.381	8.518	7.905	8.073	7.972
ARID1B	8.630	8.612	8.712	8.517	8.657	8.562
ARID1A	10.237	10.499	10.579	10.577	10.668	10.498
DDX11L16	8.024	8.146	8.047	7.916	7.969	7.788
AP000757.1	7.267	7.386	7.056	7.249	7.123	7.260
LIMA1	10.814	10.905	10.919	10.812	10.784	10.804
YIPF1	8.923	8.922	9.069	8.963	8.859	8.972
YIPF3	9.180	9.201	9.333	8.802	8.829	8.907
YIPF2	9.238	9.347	9.523	9.299	9.570	9.342
YIPF5	10.328	10.563	10.509	10.115	10.459	10.233
YIPF4	8.344	8.465	8.487	7.967	8.470	8.363
YIPF6	11.260	11.195	11.163	11.198	11.217	11.253
SDR39U1	10.198	10.325	10.422	10.066	10.099	9.952
SPTAN1	10.913	10.853	11.131	10.924	10.775	10.844
SUDS3	9.747	9.665	9.862	9.667	9.692	9.612
LRRC75B	8.593	8.276	8.597	8.292	8.524	8.407
LRRC75A	7.296	7.224	7.497	7.120	7.233	7.532
YPEL5	9.538	9.671	9.799	9.555	9.342	9.639
YPEL4	7.441	7.736	7.698	7.693	7.783	8.004
YPEL1	7.133	7.044	7.019	7.179	7.186	7.167
YPEL3	8.690	8.866	8.824	8.512	8.695	8.558
YPEL2	7.775	7.699	7.932	7.680	7.825	7.784

SAPCD1	7.937	7.693	7.892	7.743	7.590	7.680
SAPCD2	9.408	9.369	9.610	9.178	9.178	9.175
UNC93A	5.728	5.713	5.558	5.752	5.917	5.735
RAG2	4.263	4.067	4.155	4.481	4.377	4.238
RAG1	5.981	5.877	5.793	5.969	6.031	5.851
EIF4H	11.619	11.677	11.793	11.518	11.459	11.471
EIF4E	10.849	10.892	10.958	10.870	10.800	10.938
EIF4B	11.753	11.772	11.939	11.764	11.728	11.676
HOTTIP	9.538	9.342	9.084	9.794	9.853	9.785
HAT1	10.012	10.119	10.262	9.849	9.789	9.766
C1QTNF12	7.249	7.143	7.224	7.164	7.139	7.139
EFNB2	8.889	8.934	8.846	8.827	8.805	8.846
EFNB3	8.138	8.233	8.259	8.397	8.463	8.423
EFNB1	8.506	8.567	8.692	8.395	8.751	8.533
PI4KB	9.758	9.732	9.917	9.643	9.622	9.644
PI4KA	9.891	10.001	9.999	9.719	9.650	9.851
TPT1P8	5.721	6.042	5.923	6.159	6.146	6.099
ZAR1	6.210	6.260	6.516	6.366	6.302	6.556
FAM95C	7.250	7.116	7.153	6.836	6.793	6.949
NHLH2	5.929	6.151	6.010	6.113	5.878	6.011
OXSR1	9.859	9.863	9.964	9.676	9.560	9.706
AL354863.1	6.831	6.922	6.937	6.823	7.047	6.962
KCNMB2-AS1	4.436	4.543	4.629	4.616	4.511	4.411
UCMA	6.557	6.750	6.465	6.822	6.963	6.836
TATDN1P1	9.769	9.860	9.890	9.909	9.764	9.850
RRS1	9.489	9.466	9.527	9.461	9.237	9.372
ANKRD26P3	6.859	6.790	6.729	6.831	7.029	6.799
SND1	10.965	11.116	11.179	11.044	10.991	11.045
AC011330.2	7.458	7.489	7.306	7.431	7.194	7.422
AC011330.3	4.733	4.855	4.734	4.580	4.654	4.788
OR10H2	5.264	5.412	5.232	5.257	5.164	5.193
OR10H3	7.297	7.242	7.139	7.126	7.223	7.054
Z99289.3	5.731	5.684	5.498	5.724	5.681	5.734
Z99289.2	5.815	5.690	5.841	5.791	5.665	5.569
KL	4.504	4.648	4.614	4.521	4.524	4.413
RPL10A	13.719	13.776	13.864	13.830	13.759	13.867
ENDOD1	9.658	9.724	9.800	9.618	9.671	9.526
RPL10L	7.590	7.765	7.810	7.454	7.739	7.819
KY	7.749	7.759	7.877	7.684	7.955	7.946
USP8	9.941	9.899	10.035	9.933	10.013	9.784
UGT2B25P	8.916	8.824	8.983	8.796	8.862	8.989
USP1	11.177	11.119	11.220	10.616	10.541	10.702
USP2	7.563	7.450	7.649	7.804	7.515	7.571
USP3	9.851	9.936	9.961	9.718	9.712	9.810
USP4	9.089	9.107	9.182	8.885	8.889	8.904
USP5	8.855	8.847	8.974	9.088	8.966	8.959
USP6	8.859	8.780	8.717	8.769	8.712	8.905
USP7	10.949	10.934	11.042	10.758	10.702	10.768
ZNF721	7.291	7.215	7.422	6.906	7.002	7.022
ZNF720	8.631	8.501	8.716	8.312	8.359	8.463
ZNF727	3.863	4.023	3.800	3.893	3.870	3.908
RALGAPB	9.859	9.823	9.734	9.551	9.450	9.710
AL512625.2	8.069	8.342	8.384	8.466	8.460	8.437
AL512625.3	8.342	8.297	8.366	8.512	8.474	8.509
RNU6-646P	6.126	6.281	6.303	6.352	6.469	6.285
ZC3H7B	9.574	9.888	10.153	10.371	10.256	9.643

ZC3H7A	9.433	9.379	9.463	8.884	9.423	8.979
TNFRSF11A	10.297	10.491	10.464	10.241	10.262	10.220
TNFRSF11B	7.047	7.077	7.176	7.148	7.064	6.805
RBMY2BP	5.549	5.417	5.468	5.650	5.551	5.647
TCEAL7	3.867	3.931	4.009	4.060	3.984	3.777
TGIF2	9.365	9.262	9.490	9.350	9.338	9.326
TGIF1	9.963	10.076	10.299	10.111	10.171	9.968
CU639417.5	6.768	6.561	6.654	6.798	6.995	6.666
CU639417.3	7.187	7.272	7.198	7.263	7.039	7.210
CU639417.1	10.261	10.361	10.461	10.525	10.201	10.328
BGN	7.163	7.163	7.107	7.207	7.258	7.317
CT75	5.967	5.836	6.079	6.185	6.181	6.049
BLCAP	11.381	11.386	11.472	11.301	11.270	11.305
C16orf86	8.262	8.182	8.485	8.428	8.504	8.349
LINGO4	7.241	7.060	7.248	7.477	7.331	7.241
LINGO3	8.543	8.719	8.723	8.721	8.866	8.841
LINGO2	7.486	7.358	7.235	7.444	7.476	7.531
LINGO1	6.339	6.159	6.318	6.302	6.348	6.218
PRPSAP1	9.532	9.631	9.778	9.277	9.435	9.365
PRPSAP2	9.018	8.930	9.152	9.120	8.986	9.194
ETFDH	6.354	6.242	6.545	6.282	6.229	6.183
AL137060.1	9.272	9.257	9.265	9.598	9.361	9.340
NIN	7.889	8.204	8.118	7.901	7.961	8.103
AL137060.3	7.123	7.142	6.901	7.141	7.391	7.057
MYO5C	11.077	10.989	11.082	10.814	10.749	10.830
MYO5B	10.814	10.879	10.926	10.776	10.627	10.786
MYO5A	8.009	7.988	8.167	7.776	7.879	7.843
FAM13B	8.029	8.044	8.009	7.522	7.745	7.488
FAM13C	4.300	4.236	4.425	4.285	4.353	4.047
FAM13A	9.237	9.200	9.383	9.267	9.522	9.170
NAA80	8.053	8.059	8.167	7.970	8.015	7.935
CACYBPP2	11.459	11.484	11.678	11.547	11.515	11.560
PPIAP29	14.423	14.465	14.415	14.484	14.467	14.475
PPIAP22	14.423	14.465	14.415	14.484	14.467	14.475
PPIAP21	7.093	7.236	7.312	6.795	6.837	7.039
ERC2-IT1	5.571	5.307	5.223	5.499	5.740	5.514
PLCE1-AS2	7.615	7.575	7.344	7.745	7.616	7.615
EHBP1	10.339	10.339	10.228	10.217	10.116	10.253
GBP3	11.371	11.432	11.435	11.280	11.359	11.240
GBP2	10.090	10.102	10.268	9.921	10.062	9.810
GBP1	10.306	10.279	10.378	10.496	10.842	10.100
IFNLR1	9.059	9.094	8.978	8.997	9.194	9.175
GBP5	5.723	5.537	5.676	5.724	5.779	5.776
GBP4	6.538	6.441	6.436	6.460	6.622	6.563
PPBPP2	6.077	6.270	5.902	6.002	6.244	6.214
OTOA	6.880	6.877	6.878	6.815	6.879	6.936
PCDHGA8	11.092	11.102	11.188	11.070	11.043	11.048
WIZ	9.563	9.485	9.641	9.389	9.437	9.315
GU182353.3	5.465	5.240	5.208	5.596	5.463	5.357
AC004817.2	6.613	6.588	6.730	6.746	6.552	6.704
KNOP1	9.286	9.315	9.259	9.131	9.165	9.162
CERNA1	5.264	4.977	5.143	4.876	4.931	4.853
OTOR	6.831	6.609	6.754	6.991	6.601	7.186
OTOS	6.226	6.512	6.457	6.516	6.632	6.416
HMX1	9.024	8.929	8.773	9.044	8.853	8.725
THEG	7.637	7.783	7.887	7.780	7.755	7.907

TERB2	3.910	3.814	3.799	3.866	3.925	3.947
TERB1	6.247	6.151	6.187	6.561	6.390	6.429
DARS1	12.590	12.706	12.758	12.601	12.764	12.476
DDC	8.673	8.670	8.717	8.252	8.268	8.312
DARS2	9.346	9.276	9.442	9.125	9.162	9.168
CEP164	8.484	8.531	8.523	8.493	8.400	8.449
APH1A	11.433	11.575	11.853	11.658	11.787	11.364
STAT4	7.697	7.911	7.485	7.822	7.915	7.871
STAT3	11.966	12.125	12.186	11.978	12.318	12.008
STAT2	10.864	10.906	10.976	10.757	10.813	10.692
STAT1	12.331	12.300	12.276	12.196	12.238	12.103
CEP162	7.286	7.155	7.331	7.586	7.508	7.491
HTATSF1	10.070	10.125	10.142	9.809	9.763	9.807
SNORD114-10	4.976	4.928	4.987	5.012	5.075	5.122
STATH	4.445	4.702	4.637	4.825	4.780	4.712
PLG	6.786	6.694	6.442	6.599	6.891	6.717
PLN	5.933	5.789	6.170	6.207	6.163	5.955
PDE4B	6.877	7.049	6.648	7.087	6.717	6.784
PDE4C	10.300	10.317	10.270	10.190	10.027	10.182
PDE4D	7.251	7.287	7.398	7.233	7.174	7.306
AC013717.1	11.220	11.169	11.331	10.867	10.897	10.935
CSPG4P4Y	6.957	6.925	6.968	7.021	7.153	7.161
AC007638.1	6.998	7.126	6.816	7.187	7.019	7.017
BMPR1A	8.096	8.160	8.093	7.947	7.908	8.072
BMPR1B	8.808	8.881	8.782	8.421	8.151	8.453
CT47A12	5.635	5.693	5.320	5.666	5.815	5.536
CT47A11	5.635	5.693	5.320	5.666	5.815	5.536
CT47A10	5.635	5.693	5.320	5.666	5.815	5.536
DUX4L28	8.052	7.820	8.258	8.487	8.319	8.298
DUX4L29	8.052	7.820	8.258	8.487	8.319	8.298
DUX4L24	8.052	7.820	8.258	8.487	8.319	8.298
DUX4L25	8.052	7.820	8.258	8.487	8.319	8.298
DUX4L22	8.052	7.820	8.258	8.487	8.319	8.298
DUX4L23	8.052	7.820	8.258	8.487	8.319	8.298
DUX4L20	8.052	7.820	8.258	8.487	8.319	8.298
DUX4L21	8.052	7.820	8.258	8.487	8.319	8.298
CRAMP1	7.864	7.837	7.550	7.766	7.803	7.872
MIR1915HG	9.620	9.628	9.781	9.776	9.810	9.782
RPL7AP49	8.174	8.070	8.261	8.185	8.174	8.173
MTR	8.342	8.350	8.452	8.017	8.007	8.202
SUCLG2	10.076	10.147	10.086	9.997	9.913	9.963
SUCLG1	11.191	11.212	11.349	11.305	11.258	11.195
ALDOA	14.208	14.233	14.307	14.280	14.163	14.233
ALDOC	7.627	7.473	7.585	7.663	7.637	7.668
ALDOB	8.363	8.252	8.340	8.246	8.406	8.377
CAND2	6.246	6.256	6.198	6.296	6.376	6.392
CAND1	10.427	10.487	10.590	10.324	10.233	10.386
ARX	6.177	6.299	6.166	6.402	6.531	6.354
MAP3K14	8.046	8.068	7.889	8.419	8.499	8.335
PHF3	8.908	8.923	8.874	8.649	8.581	8.697
ARC	6.417	6.423	6.460	6.376	6.416	6.416
HAO1	6.124	5.882	5.854	6.189	5.990	5.990
HAO2	7.077	6.917	6.837	6.919	7.138	6.972
ZNF625-ZNF20	7.644	7.582	7.620	7.712	7.631	7.816
TMOD4	6.024	6.130	5.655	6.040	5.937	5.817
TMOD1	7.830	7.729	7.733	7.604	7.862	7.690

AK3P3	4.613	4.509	4.591	4.490	4.515	4.427
TMOD3	10.497	10.601	10.376	10.160	10.112	10.372
TMOD2	4.677	4.810	4.630	4.751	4.726	4.656
ZNF79	7.618	7.739	7.880	7.912	7.835	7.863
APLP1	7.653	7.674	7.666	7.694	7.706	7.625
APLP2	12.981	13.023	13.106	12.932	12.817	12.917
ZNF71	7.966	8.028	8.207	8.266	8.126	8.011
ZNF76	8.852	8.976	9.084	8.932	8.873	8.962
ZNF77	7.889	7.881	7.945	8.014	7.963	8.111
ZNF74	7.902	7.939	7.975	7.967	7.853	7.964
AC092368.3	8.403	8.462	8.577	8.420	8.481	8.387
SFTPA2	6.837	6.941	7.005	6.801	6.861	6.641
SFTPA1	5.153	5.071	5.331	5.036	5.485	5.158
VAT1	11.613	11.652	11.760	11.578	11.456	11.569
GIPC1	11.059	10.988	11.227	11.177	11.051	11.046
GIPC2	7.288	7.095	7.369	6.414	6.643	6.673
GIPC3	8.279	8.239	8.196	8.236	8.339	8.212
ELFN1	7.645	7.363	7.734	7.898	7.779	7.673
ELFN2	7.814	7.824	7.699	7.878	7.966	7.853
RN7SL301P	5.892	5.747	6.067	6.084	6.012	5.853
FZR1	8.869	8.831	9.083	9.140	9.081	8.988
MEGF10	7.208	7.370	7.278	7.253	7.446	7.404
MEGF11	7.930	8.073	7.992	7.927	8.036	8.094
HBG1	7.191	7.300	7.218	7.328	7.503	7.422
CD2AP	10.750	10.743	10.730	10.498	10.667	10.629
CPS1	6.757	6.658	6.362	6.738	6.795	6.757
MTDHP3	4.475	5.093	4.662	5.020	4.836	4.709
MTDHP4	4.475	5.093	4.662	5.020	4.836	4.709
AP000346.2	8.896	8.620	8.785	8.528	8.209	8.176
AL160191.3	5.933	6.058	6.004	6.222	6.100	6.073
AL160191.2	8.488	8.568	8.483	8.691	8.653	8.636
MUC12-AS1	7.770	7.935	8.101	8.428	8.026	8.029
BET1L	10.447	10.509	10.718	10.476	10.555	10.489
IGKV3-20	6.535	6.877	6.890	6.793	6.883	6.915
KIAA0040	8.127	8.205	8.254	8.051	8.247	8.058
AP000894.4	5.832	5.522	5.810	5.424	5.589	5.753
BTBD9	8.828	8.793	8.990	8.566	8.601	8.642
BTBD8	5.897	6.058	5.944	5.827	5.971	5.850
BTBD7	9.554	9.661	9.644	9.546	9.723	9.607
BTBD6	10.360	10.433	10.559	10.285	10.327	10.294
BTBD1	11.098	11.150	11.166	11.014	10.876	10.915
BTBD3	8.266	8.389	8.479	8.048	8.102	8.315
BTBD2	9.672	9.781	10.001	9.802	9.833	9.736
SULT1B1	7.777	8.016	7.515	7.915	7.720	7.981
AL079338.1	6.817	6.912	6.858	6.812	6.762	6.976
ZNF354C	7.855	7.786	7.863	7.769	8.202	8.100
AC073188.6	7.341	7.285	7.472	7.436	7.497	7.359
AC073188.5	5.868	6.077	5.687	5.831	5.845	5.863
NOM1	8.319	8.501	8.615	8.069	8.263	8.145
AC073188.3	4.483	4.417	4.707	4.780	4.721	4.810
DDX11L10	8.024	8.146	8.047	7.916	7.969	7.788
IKBKGP1	10.308	10.254	10.278	10.317	10.236	10.286
PRKCZ-AS1	7.843	8.141	7.974	7.906	8.038	8.214
SPATS1	6.454	6.752	6.483	6.530	6.595	6.743
NFIC	9.589	9.496	9.660	9.227	9.661	9.039
SPATS2	8.956	8.694	8.962	8.622	8.784	8.668

AL662899.4	11.996	12.053	12.198	11.947	11.842	11.914
AL662899.3	8.862	8.931	9.098	8.915	8.886	8.916
RNASEK-C17orf4	12.049	12.125	12.282	11.981	12.082	12.102
PSPN	10.101	9.960	10.154	9.528	10.026	9.733
ZNF767P	7.549	7.244	7.106	7.549	7.509	7.559
FAAH	8.357	8.296	8.414	8.326	8.340	8.344
SLC8B1	8.883	8.859	8.842	8.627	8.824	8.704
PSPH	9.073	9.081	9.084	8.706	8.960	9.204
PHF20L1	9.872	9.758	9.759	9.711	9.710	9.850
AC087667.1	4.588	4.671	4.658	4.554	4.673	4.718
VCAN	5.849	5.774	5.895	5.880	5.743	5.781
AL138724.2	6.702	6.903	6.887	7.013	6.784	6.756
AC023813.4	8.032	7.741	7.959	8.078	8.143	8.017
AC114812.1	6.141	5.935	5.883	6.407	6.323	6.303
KRBOX1	7.014	6.941	7.054	7.101	7.126	7.098
KRBOX4	7.383	7.443	7.346	7.199	7.235	7.045
ERVW-1	7.165	7.354	7.228	7.319	7.270	7.380
ACAP1	8.335	8.256	8.523	8.695	8.470	8.265
ACAP2	9.886	9.996	9.800	9.626	9.605	9.745
ACAP3	8.743	8.838	8.855	8.695	8.763	8.705
ZNRF2P1	4.674	5.026	4.850	4.862	4.723	4.786
ZNRF2P2	4.674	5.026	4.850	4.862	4.723	4.786
ATRN	8.787	8.720	8.725	8.627	8.465	8.658
SELL	8.455	8.624	8.535	8.249	8.288	8.285
NFIB	10.859	10.663	10.698	10.462	10.353	10.801
NFIA	9.377	9.327	9.383	9.118	9.191	9.087
SLC7A14	6.310	6.253	6.334	6.190	6.260	6.501
SELE	4.772	4.994	4.784	4.727	4.731	4.747
SLC7A10	7.194	7.079	7.114	7.168	7.026	6.968
SLC7A11	10.335	10.818	10.803	10.157	10.173	10.024
AC084375.1	5.809	5.711	5.535	5.774	5.728	5.631
SLC7A13	4.800	4.867	4.918	4.713	4.834	4.744
TULP2	6.700	6.391	6.450	6.216	6.469	6.655
GNGT2	7.397	7.466	7.315	7.394	7.616	7.393
GNGT1	4.144	4.033	4.299	4.304	4.168	4.082
TULP3	8.866	8.877	9.081	9.064	9.051	8.759
DIAPH2-AS1	6.382	6.420	6.346	6.232	6.374	6.245
NFIX	8.222	8.097	8.447	8.364	8.330	8.336
SELP	7.947	8.042	7.648	8.011	8.092	8.067
CT476828.20	8.052	7.820	8.258	8.487	8.319	8.298

Log2 normalized expression

Supplementary file 2: Raw read counts of RNA-seq experiment (GEO accession: GSE147507) SARS-CoV-2 infection.

GSM_ID	GSM4432378	GSM4432379	GSM4432380	GSM4432381	GSM4432382	GSM4432383	GSM4462413	GSM4462414	GSM4462415	GSM4462416
Cell_Information	NHBE_WT	NHBE_WT	NHBE_WT	NHBE_SARS2	NHBE_SARS2	NHBE_SARS2	Lung_WT	Lung_WT	Lung_Patient	Lung_Patient
ENSG00000280454	0	0	0	0	0	0	0	0	0	0
ENSG00000280453	0	0	0	0	0	0	0	0	0	0
ENSG00000280452	0	0	0	0	0	0	0	0	0	0
ENSG00000280451	0	0	0	0	0	0	0	2	0	0
ENSG00000280450	0	0	0	0	0	0	0	0	0	0
ENSG00000280449	0	0	0	0	0	0	0	0	0	0
ENSG00000280448	0	0	0	0	0	0	0	0	0	0
ENSG00000280447	32	33	40	18	20	52	111	85	0	1
ENSG00000280446	0	0	0	0	0	0	0	0	0	0
ENSG00000280445	0	0	1	0	0	0	0	0	0	0
ENSG00000280444	0	0	0	0	0	0	0	0	0	0
ENSG00000280443	0	0	0	0	0	0	0	0	0	0
ENSG00000280442	0	0	0	0	0	0	0	0	0	0
ENSG00000280441	38	38	37	33	27	62	159	2013	2	9
ENSG00000280440	0	0	0	0	0	1	0	0	0	0
ENSG00000280439	0	0	0	0	0	0	21	2	0	2
ENSG00000280438	0	0	0	0	0	0	0	0	0	0
ENSG00000280437	56	59	79	51	63	145	654	220	0	0
ENSG00000280436	0	0	0	0	0	0	0	0	0	0
ENSG00000280435	206	222	337	256	262	808	9	0	0	0
ENSG00000280434	2	3	10	3	4	9	130	57	2	10
ENSG00000280433	338	296	416	299	282	901	1220	556	1	2
ENSG00000280432	0	0	0	0	0	0	0	0	0	0
ENSG00000280431	0	0	0	0	0	0	0	0	0	0
ENSG00000280430	0	0	0	0	0	0	0	0	0	0
ENSG00000280429	0	0	0	0	0	0	0	9	0	0
ENSG00000280427	0	0	0	0	0	0	0	0	0	0
ENSG00000280426	1	1	2	2	1	1	3	0	0	0
ENSG00000280425	0	0	0	1	0	0	0	0	0	0
ENSG00000280424	0	1	1	0	0	0	2	0	0	0
ENSG00000280423	0	0	0	0	0	0	2	0	0	0
ENSG00000280422	3	1	0	0	2	2	5	15	0	0
ENSG00000280421	0	0	0	0	0	0	0	0	0	0
ENSG00000280420	0	0	0	0	0	0	0	0	0	0
ENSG00000280419	0	0	0	0	0	0	0	0	0	0
ENSG00000280418	0	0	0	0	0	0	0	5	0	0
ENSG00000280417	0	3	0	0	0	0	0	0	0	0
ENSG00000280416	0	0	0	0	0	0	0	0	0	0
ENSG00000280415	0	0	0	0	0	0	0	0	0	0
ENSG00000280414	0	0	0	0	0	0	0	0	0	0
ENSG00000280413	0	0	0	0	0	0	11	7	0	0
ENSG00000280412	0	0	0	0	0	0	0	0	0	0
ENSG00000280411	0	0	0	0	0	0	0	122	0	0
ENSG00000280410	0	0	0	0	0	0	0	0	0	0
ENSG00000280409	0	0	0	0	0	0	0	4	0	0
ENSG00000280408	0	0	0	0	0	0	1	4	0	0
ENSG00000280407	8	16	13	13	3	29	21	5	0	0
ENSG00000280406	0	0	0	0	0	0	0	9	0	0
ENSG00000280405	0	0	0	0	0	0	0	0	0	0
ENSG00000280404	0	0	0	0	0	0	2	0	0	0
ENSG00000280403	31	38	52	19	29	62	11	13	0	2
ENSG00000280402	7	10	9	7	11	37	10	27	0	0
ENSG00000280401	1	0	4	0	1	4	6	1	0	0

ENSG00000280400	0	0	0	0	0	0	0	0	0	0
ENSG00000280399	0	0	5	1	2	6	30	18	0	0
ENSG00000280398	0	0	0	0	0	0	0	0	0	0
ENSG00000280397	0	3	0	0	0	0	1	1	0	0
ENSG00000280396	0	0	0	0	0	0	0	4	0	1
ENSG00000280395	2	1	1	0	0	0	3	28	0	2
ENSG00000280394	0	0	0	0	0	0	0	0	0	0
ENSG00000280393	0	0	0	0	0	0	0	0	0	0
ENSG00000280392	0	0	0	0	0	0	19	0	0	0
ENSG00000280391	0	0	1	0	0	0	1	5	1	3
ENSG00000280390	0	0	0	0	0	0	0	0	0	0
ENSG00000280389	0	0	0	0	0	0	0	1	0	0
ENSG00000280388	9	11	6	4	5	13	6	0	0	0
ENSG00000280387	0	0	2	2	1	10	1	3	0	4
ENSG00000280386	0	0	0	0	0	0	0	0	0	0
ENSG00000280385	67	61	112	43	55	145	210	59	0	0
ENSG00000280384	0	2	0	0	0	0	3	15	0	3
ENSG00000280383	5	6	13	3	1	14	38	33	0	0
ENSG00000280382	0	0	0	0	0	0	2	0	0	0
ENSG00000280381	0	0	3	0	0	2	34	27	0	0
ENSG00000280380	0	0	0	0	0	0	0	0	0	0
ENSG00000280379	0	0	0	0	0	0	0	5	0	1
ENSG00000280378	4	9	4	4	1	12	7	4	0	0
ENSG00000280377	0	0	2	0	0	0	1	0	0	0
ENSG00000280376	1	2	0	1	1	4	0	0	0	0
ENSG00000280375	0	0	0	0	0	0	7	0	0	0
ENSG00000280374	4	2	1	2	1	6	13	8	0	0
ENSG00000280373	0	0	0	0	0	0	0	0	0	0
ENSG00000280372	0	0	0	0	0	0	2	1	0	0
ENSG00000280371	0	0	0	0	0	0	0	3	0	0
ENSG00000280370	3	11	6	1	5	9	15	101	5	13
ENSG00000280369	0	0	0	1	0	0	0	0	0	0
ENSG00000280368	0	1	0	0	0	0	0	0	0	0
ENSG00000280367	1	2	1	1	0	1	13	8	0	0
ENSG00000280366	0	0	0	0	0	0	0	0	0	0
ENSG00000280365	0	0	0	0	0	0	1	2	0	0
ENSG00000280364	0	0	0	0	0	0	0	0	0	0
ENSG00000280363	0	0	0	0	0	0	0	0	0	0
ENSG00000280362	2	0	1	1	0	0	0	0	0	0
ENSG00000280361	0	0	0	0	0	0	0	0	0	0
ENSG00000280360	0	0	0	0	0	0	0	0	0	0
ENSG00000280359	0	0	0	0	0	0	0	0	0	0
ENSG00000280358	0	0	0	0	0	0	0	0	0	0
ENSG00000280357	0	0	0	0	0	0	0	0	0	0
ENSG00000280356	0	0	0	0	0	0	0	2	0	0
ENSG00000280355	5	5	14	3	5	13	29	8	0	0
ENSG00000280354	0	0	0	0	0	0	1	0	0	0
ENSG00000280353	0	1	0	1	2	3	1	3	0	0
ENSG00000280352	0	0	0	0	0	0	0	0	0	0
ENSG00000280351	31	33	37	21	22	58	1	9	0	0
ENSG00000280350	0	0	0	0	0	0	0	0	0	0
ENSG00000280349	0	0	0	0	0	0	0	0	0	0
ENSG00000280348	0	0	0	0	0	0	0	3	0	0
ENSG00000280347	22	20	51	14	11	56	103	37	0	0
ENSG00000280346	0	0	0	0	2	0	0	3	0	0
ENSG00000280345	0	1	4	2	1	0	1	4	0	0
ENSG00000280344	0	0	0	0	0	0	0	0	0	0

ENSG00000280343	0	0	0	0	0	0	0	0	0	0
ENSG00000280342	0	0	0	0	0	0	0	0	0	0
ENSG00000280341	25	67	222	23	27	80	122	33	0	6
ENSG00000280340	0	0	0	0	0	0	0	0	0	0
ENSG00000280339	0	0	0	0	0	2	17	198	0	0
ENSG00000280338	0	0	0	0	0	0	0	0	0	0
ENSG00000280337	0	0	0	0	0	1	0	0	0	0
ENSG00000280336	0	0	0	0	1	0	0	0	0	0
ENSG00000280335	0	3	3	0	0	0	6	1	0	0
ENSG00000280334	4	5	3	2	4	9	4	1	0	0
ENSG00000280333	0	0	0	0	1	0	1	2	0	0
ENSG00000280332	21	14	24	13	13	26	49	45	0	0
ENSG00000280331	0	0	3	1	0	3	8	7	0	0
ENSG00000280330	2	0	7	1	0	0	1	0	0	0
ENSG00000280329	0	0	0	0	0	0	0	0	0	0
ENSG00000280328	4	10	8	9	18	20	2	2	0	0
ENSG00000280327	2	1	7	2	3	4	4	11	0	0
ENSG00000280326	0	0	0	0	0	0	7	1	0	0
ENSG00000280325	0	0	1	0	1	0	2	0	0	0
ENSG00000280324	0	0	0	0	0	0	0	0	0	0
ENSG00000280323	0	0	0	0	0	0	9	43	0	8
ENSG00000280322	0	0	0	0	0	0	0	0	0	0
ENSG00000280321	0	0	0	0	0	0	2	0	0	0
ENSG00000280320	0	0	0	0	0	0	0	0	0	0
ENSG00000280319	0	0	0	0	0	0	1	0	0	0
ENSG00000280318	0	0	0	0	0	0	0	0	0	0
ENSG00000280317	0	0	0	0	0	0	0	0	0	0
ENSG00000280316	4	6	1	0	2	3	4	6	0	0
ENSG00000280315	0	0	0	0	0	0	0	0	0	0
ENSG00000280314	0	0	0	0	0	0	0	0	0	0
ENSG00000280313	0	0	0	0	0	0	0	0	0	0
ENSG00000280312	0	0	0	0	0	0	0	1	0	0
ENSG00000280311	0	0	1	0	0	0	0	0	0	0
ENSG00000280310	0	0	0	0	0	0	0	3	0	0
ENSG00000280309	0	0	0	0	0	0	0	0	0	0
ENSG00000280308	11	6	16	7	9	21	16	6	1	0
ENSG00000280307	0	0	0	0	0	0	0	0	0	0
ENSG00000280306	0	0	0	0	0	0	0	0	0	0
ENSG00000280305	0	0	0	0	0	0	0	9	0	0
ENSG00000280304	0	0	0	0	0	0	0	9	0	0
ENSG00000280303	1	0	4	4	0	0	47	0	0	0
ENSG00000280302	0	0	0	1	0	0	5	4	0	0
ENSG00000280301	0	0	0	0	0	0	0	1	0	0
ENSG00000280300	5	8	2	2	2	10	10	1	0	0
ENSG00000280299	0	0	0	0	0	0	0	0	0	0
ENSG00000280298	32	38	53	18	27	58	8	12	0	1
ENSG00000280297	0	0	2	0	0	2	4	0	0	0
ENSG00000280296	0	0	0	0	0	0	1	2	0	0
ENSG00000280295	0	0	0	0	0	0	1	0	0	0
ENSG00000280294	0	7	9	1	4	4	3	8	0	0
ENSG00000280293	0	0	0	0	0	0	0	0	0	0
ENSG00000280292	0	1	3	0	0	2	16	4	0	0
ENSG00000280291	0	0	1	0	0	2	0	1	0	0
ENSG00000280290	0	0	0	0	0	0	0	0	0	0
ENSG00000280289	0	0	0	0	0	0	0	0	0	0
ENSG00000280288	0	0	0	0	0	0	0	0	0	0
ENSG00000280287	9	8	19	11	11	20	40	14	0	0

ENSG00000280286	0	1	5	0	0	0	1	3	0	0
ENSG00000280285	0	0	1	0	1	2	24	9	0	2
ENSG00000280284	0	0	0	0	0	0	0	0	0	0
ENSG00000280283	0	1	0	0	0	0	0	0	0	0
ENSG00000280282	0	0	0	0	0	0	0	0	0	0
ENSG00000280281	0	0	0	0	0	0	0	11	0	0
ENSG00000280280	0	0	0	0	0	0	0	0	0	0
ENSG00000280279	190	217	343	142	172	535	39	1	0	4
ENSG00000280278	0	1	1	1	0	0	0	0	4	0
ENSG00000280277	0	1	0	0	0	0	0	1	0	0
ENSG00000280276	0	0	0	0	0	0	1	0	0	0
ENSG00000280275	0	0	0	0	0	0	0	9	0	0
ENSG00000280274	0	0	0	0	0	0	0	2	0	0
ENSG00000280273	1	1	3	0	1	8	2	4	0	0
ENSG00000280272	0	0	0	0	0	0	0	0	0	0
ENSG00000280270	0	0	0	0	0	0	0	0	0	0
ENSG00000280269	1	1	2	0	0	3	4	0	0	0
ENSG00000280268	0	0	0	0	0	0	0	0	0	0
ENSG00000280267	0	0	0	0	0	0	0	0	0	0
ENSG00000280266	0	0	0	0	0	0	1	0	0	0
ENSG00000280265	0	0	0	0	0	0	27	0	0	0
ENSG00000280264	21	36	44	17	26	69	6	6	1	2
ENSG00000280263	0	0	0	0	0	0	0	0	0	0
ENSG00000280262	0	0	0	0	0	0	0	0	0	0
ENSG00000280261	0	0	0	0	0	0	0	0	0	0
ENSG00000280260	0	0	0	0	0	0	2	0	0	0
ENSG00000280259	0	0	0	0	0	0	0	0	0	0
ENSG00000280258	0	0	0	0	0	0	21	20	0	1
ENSG00000280257	0	0	0	0	0	0	0	0	0	0
ENSG00000280256	0	0	0	0	0	0	0	0	0	0
ENSG00000280255	0	0	0	0	0	0	0	69	0	0
ENSG00000280254	13	10	19	15	9	47	8	27	1	0
ENSG00000280253	0	0	0	0	0	0	0	0	0	0
ENSG00000280252	26	34	54	31	27	57	30	20	0	0
ENSG00000280251	0	0	0	0	0	0	0	0	0	0
ENSG00000280250	0	0	0	0	0	0	1	0	0	0
ENSG00000280249	0	0	1	0	0	0	0	2	0	0
ENSG00000280248	0	1	0	3	1	1	1	16	0	3
ENSG00000280247	0	0	1	0	1	3	19	1	0	0
ENSG00000280246	0	0	0	0	0	1	3	7	0	0
ENSG00000280245	0	3	2	0	0	7	1	0	0	0
ENSG00000280244	1	0	2	0	0	4	1	27	0	0
ENSG00000280243	2	2	0	3	1	4	2	0	0	0
ENSG00000280242	1	0	0	0	0	0	0	7	0	0
ENSG00000280241	1	0	0	0	0	0	1	0	0	0
ENSG00000280240	0	0	0	0	0	1	0	6	0	0
ENSG00000280239	4	10	7	3	3	13	4	16	0	5
ENSG00000280238	0	0	0	0	0	0	0	0	0	0
ENSG00000280237	8	11	14	9	7	16	302	224	0	0
ENSG00000280236	0	0	0	0	0	0	0	0	0	0
ENSG00000280234	2	1	3	0	0	5	4	1	0	0
ENSG00000280233	1	1	0	0	0	0	1	0	0	0
ENSG00000280232	0	0	2	1	1	0	1	0	0	0
ENSG00000280231	6	9	9	6	4	17	3	57	0	0
ENSG00000280230	4	6	7	0	1	6	18	4	0	0
ENSG00000280229	0	0	0	0	0	0	0	0	0	0
ENSG00000280228	1	0	0	0	0	0	6	6	0	8

ENSG00000280227	0	0	0	0	0	0	0	0	0	0
ENSG00000280226	0	0	0	0	0	0	0	0	0	0
ENSG00000280225	0	0	0	0	0	0	0	5	0	0
ENSG00000280224	0	0	0	0	0	0	0	0	3	0
ENSG00000280223	0	0	0	0	0	0	0	0	0	0
ENSG00000280222	0	0	0	0	0	0	0	0	0	0
ENSG00000280221	0	0	0	0	0	0	2	8	0	0
ENSG00000280220	0	0	0	0	0	0	0	0	0	0
ENSG00000280219	4	6	6	1	3	4	226	46	0	12
ENSG00000280217	0	0	0	0	0	0	0	6	0	0
ENSG00000280216	0	0	0	0	0	0	1	6	0	0
ENSG00000280215	0	0	0	0	0	0	0	0	0	0
ENSG00000280214	1	0	2	4	3	7	1	0	0	4
ENSG00000280213	10	16	19	8	8	18	10	4	0	0
ENSG00000280212	0	1	1	1	1	2	18	11	0	2
ENSG00000280211	1	4	3	3	3	9	20	30	0	0
ENSG00000280209	0	0	0	0	0	0	0	0	0	0
ENSG00000280208	0	1	0	0	0	0	6	3	0	0
ENSG00000280207	0	0	0	0	0	0	0	0	0	0
ENSG00000280206	62	45	103	39	70	170	100	41	0	0
ENSG00000280205	0	0	0	0	0	0	9	1	0	1
ENSG00000280204	0	0	0	0	0	0	0	0	0	0
ENSG00000280203	0	0	0	0	0	0	0	0	0	0
ENSG00000280202	4	1	3	2	0	0	15	9	0	0
ENSG00000280201	0	0	0	0	0	0	0	0	0	0
ENSG00000280200	0	0	0	0	0	0	103	18	0	0
ENSG00000280199	0	0	0	0	0	0	0	0	0	0
ENSG00000280198	3	1	3	1	1	11	3	10	0	0
ENSG00000280197	0	0	0	0	0	0	0	0	0	0
ENSG00000280196	0	0	0	0	0	0	0	0	0	0
ENSG00000280195	5	13	20	1	2	18	49	44	0	0
ENSG00000280194	1	3	3	1	6	4	27	3	0	1
ENSG00000280193	6	5	15	4	7	14	5	16	0	0
ENSG00000280192	0	0	0	0	0	0	0	0	0	0
ENSG00000280191	15	17	20	30	24	36	11	31	0	0
ENSG00000280190	0	0	0	0	0	0	0	0	0	0
ENSG00000280189	1	0	0	0	0	1	0	5	0	0
ENSG00000280188	0	0	0	0	0	0	0	0	0	0
ENSG00000280187	17	17	32	21	9	40	4	14	0	0
ENSG00000280186	3	0	4	0	2	0	0	10	0	0
ENSG00000280185	0	0	0	0	0	0	0	0	0	0
ENSG00000280184	1	0	1	5	1	0	26	6	0	0
ENSG00000280183	1	1	0	0	1	6	0	2	0	0
ENSG00000280182	0	0	0	0	0	0	0	4	0	0
ENSG00000280181	0	0	0	0	0	0	2	1	0	0
ENSG00000280180	1	3	0	0	2	9	4	16	0	0
ENSG00000280179	57	58	52	58	44	140	8	12	0	0
ENSG00000280178	0	0	0	0	0	0	0	0	0	0
ENSG00000280177	0	0	0	0	0	0	0	13	0	0
ENSG00000280176	2	3	3	1	1	5	3	0	0	0
ENSG00000280175	0	0	0	0	0	0	0	0	0	0
ENSG00000280174	0	0	0	0	0	0	0	0	0	0
ENSG00000280173	8	13	19	13	15	46	13	10	0	0
ENSG00000280172	0	0	0	0	0	1	1	0	0	0
ENSG00000280171	0	0	0	0	0	0	0	0	0	0
ENSG00000280170	26	29	39	13	18	42	60	71	0	0
ENSG00000280169	0	0	0	0	0	0	0	0	0	0

ENSG00000280168	0	0	0	0	0	0	0	2	0	0
ENSG00000280167	0	0	0	0	0	0	2	0	0	0
ENSG00000280166	0	0	0	0	0	0	0	0	0	0
ENSG00000280165	0	0	0	0	0	0	0	0	0	0
ENSG00000280164	6	8	5	3	9	13	6	11	0	0
ENSG00000280163	3	1	1	0	0	1	1	12	0	0
ENSG00000280162	6	1	7	4	5	10	47	14	0	0
ENSG00000280161	0	0	1	1	2	13	7	0	0	0
ENSG00000280160	2	2	0	1	3	6	3	2	0	0
ENSG00000280159	0	0	0	0	0	0	3	3	0	0
ENSG00000280158	0	0	0	0	0	0	0	0	0	0
ENSG00000280157	1	2	1	1	3	11	36	2	0	0
ENSG00000280156	141	183	358	112	120	413	233	339	11	11
ENSG00000280155	0	0	0	0	0	0	0	0	0	0
ENSG00000280154	0	0	0	0	0	0	0	0	0	0
ENSG00000280153	4	3	5	9	3	11	183	151	0	0
ENSG00000280152	13	17	23	17	9	42	42	30	0	0
ENSG00000280151	22	28	47	8	20	41	23	19	0	0
ENSG00000280150	0	0	0	0	0	0	0	0	0	0
ENSG00000280149	5	8	13	5	6	27	10	10	0	6
ENSG00000280148	0	0	0	0	0	0	0	0	0	0
ENSG00000280147	4	2	12	3	4	9	1	0	0	0
ENSG00000280146	0	0	0	0	0	0	0	0	0	0
ENSG00000280145	7	6	20	4	3	10	38	36	0	0
ENSG00000280144	0	0	0	0	0	0	0	0	0	0
ENSG00000280143	0	4	0	0	2	3	86	818	0	0
ENSG00000280142	0	0	0	0	0	0	0	0	0	0
ENSG00000280141	38	47	59	21	37	68	11	4	0	4
ENSG00000280140	0	0	0	0	0	0	0	8	0	0
ENSG00000280139	0	0	0	1	0	0	0	0	0	0
ENSG00000280138	96	102	142	78	85	273	296	872	3	25
ENSG00000280137	0	0	0	0	0	0	0	0	0	0
ENSG00000280136	16	10	41	10	11	46	7	10	0	7
ENSG00000280135	0	0	0	0	0	0	28	2	0	0
ENSG00000280134	0	0	0	0	0	0	0	0	0	0
ENSG00000280133	0	0	0	0	0	0	0	0	0	0
ENSG00000280132	4	2	9	4	7	3	2	8	0	0
ENSG00000280131	0	0	0	0	0	0	0	0	0	0
ENSG00000280130	44	45	77	32	39	80	63	44	0	0
ENSG00000280129	0	1	6	1	0	4	2	0	0	0
ENSG00000280128	50	88	126	60	66	146	112	84	0	0
ENSG00000280127	17	19	37	12	14	27	6	8	0	2
ENSG00000280126	0	1	0	0	0	1	0	0	0	0
ENSG00000280125	0	0	0	0	0	0	0	0	0	0
ENSG00000280124	0	0	0	0	0	0	0	0	0	0
ENSG00000280123	16	21	24	10	15	38	3	21	0	0
ENSG00000280122	0	1	1	0	0	1	3	1	0	0
ENSG00000280121	0	0	0	0	0	0	0	0	0	0
ENSG00000280120	4	3	1	2	4	5	59	7	0	0
ENSG00000280119	0	0	0	0	0	0	8	4	0	0
ENSG00000280118	0	0	0	0	0	0	0	10	0	0
ENSG00000280117	0	1	0	0	0	1	0	15	0	0
ENSG00000280116	0	0	0	0	0	0	0	0	0	0
ENSG00000280115	0	0	0	0	0	0	0	2	0	0
ENSG00000280114	0	0	2	1	0	0	0	0	0	0
ENSG00000280113	1	1	0	1	1	2	0	0	0	0
ENSG00000280112	0	0	0	0	0	0	0	0	0	0

ENSG00000280111	38	26	60	31	38	100	180	210	0	6
ENSG00000280110	0	0	0	0	0	0	0	0	0	0
ENSG00000280109	6	1	7	3	8	8	106	1	0	0
ENSG00000280108	0	0	0	0	0	0	0	0	0	0
ENSG00000280107	0	0	6	1	0	8	12	15	0	0
ENSG00000280106	0	0	0	0	0	2	25	35	0	0
ENSG00000280105	0	0	0	0	0	0	0	5	0	0
ENSG00000280104	0	0	0	0	0	0	1	0	0	0
ENSG00000280103	0	0	0	0	0	0	1	11	0	0
ENSG00000280102	0	0	0	0	0	0	0	0	0	0
ENSG00000280101	0	0	0	0	0	0	0	0	0	0
ENSG00000280100	0	0	0	0	0	0	0	0	0	0
ENSG00000280099	21	18	32	16	16	38	65	30	0	0
ENSG00000280098	0	0	0	0	0	0	0	0	0	0
ENSG00000280097	0	0	0	0	0	0	2	0	0	0
ENSG00000280096	56	60	85	84	43	181	57	140	0	2
ENSG00000280095	4	3	8	7	1	16	0	9	0	0
ENSG00000280094	0	0	0	0	0	0	0	0	0	0
ENSG00000280093	0	0	0	0	0	0	0	6	0	0
ENSG00000280092	0	0	0	0	0	0	0	0	0	0
ENSG00000280091	0	0	0	0	0	0	4	0	0	1
ENSG00000280090	0	0	0	0	0	0	0	0	0	0
ENSG00000280089	0	0	0	0	0	0	3	0	0	0
ENSG00000280088	9	16	29	3	7	57	283	100	0	12
ENSG00000280087	4	2	3	2	3	3	7	3	0	2
ENSG00000280086	0	0	0	0	0	0	0	0	0	0
ENSG00000280085	0	0	2	1	0	1	0	0	0	0
ENSG00000280084	0	0	0	0	0	0	0	0	0	0
ENSG00000280083	3	0	1	1	2	5	15	8	0	0
ENSG00000280082	0	0	0	0	0	0	0	0	0	0
ENSG00000280081	0	0	0	0	0	1	0	0	0	0
ENSG00000280080	0	0	0	0	0	1	7	0	0	0
ENSG00000280079	6	2	5	0	1	11	23	9	0	0
ENSG00000280078	0	0	0	0	0	0	2	0	0	0
ENSG00000280077	15	12	24	11	10	14	132	101	0	1
ENSG00000280076	0	0	0	0	0	0	0	1	0	0
ENSG00000280075	0	0	0	0	0	0	0	0	0	0
ENSG00000280074	0	0	0	1	0	0	1	20	0	0
ENSG00000280073	0	1	0	0	0	1	4	31	0	0
ENSG00000280071	48	37	54	53	47	113	92	83	0	0
ENSG00000280070	0	0	0	0	0	0	0	0	0	0
ENSG00000280069	4	1	10	4	4	8	13	6	0	0
ENSG00000280068	1	0	0	0	0	0	0	0	0	0
ENSG00000280067	5	6	10	8	11	18	19	13	0	0
ENSG00000280065	0	0	0	0	0	0	3	13	0	2
ENSG00000280064	0	0	0	0	0	0	0	0	0	0
ENSG00000280063	21	7	29	16	20	45	15	4	0	0
ENSG00000280062	0	0	0	0	0	2	2	0	0	0
ENSG00000280061	0	0	0	0	0	0	6	9	0	0
ENSG00000280060	0	0	5	0	0	0	37	30	0	0
ENSG00000280059	0	0	0	0	0	0	0	7	0	0
ENSG00000280058	0	0	0	0	0	0	0	12	0	0
ENSG00000280057	0	0	2	0	1	0	1	0	0	0
ENSG00000280056	0	0	0	0	0	0	0	0	0	0
ENSG00000280055	1	1	3	2	1	3	31	6	0	0
ENSG00000280054	5	5	8	4	3	7	17	46	0	0
ENSG00000280053	0	2	0	0	0	0	4	2	0	0

ENSG00000280052	0	0	0	0	0	0	0	0	0	0
ENSG00000280051	0	0	0	0	0	0	0	0	0	0
ENSG00000280050	0	0	0	0	0	0	0	0	0	0
ENSG00000280049	0	0	0	0	0	0	0	0	0	0
ENSG00000280048	0	0	1	0	0	1	1	21	0	0
ENSG00000280047	15	22	33	18	14	59	80	27	0	0
ENSG00000280046	15	22	31	16	12	81	55	29	0	4
ENSG00000280045	0	0	0	0	0	0	0	0	0	0
ENSG00000280044	0	0	0	0	0	0	0	0	0	0
ENSG00000280043	0	0	1	0	0	0	0	0	0	0
ENSG00000280042	0	0	0	1	0	2	12	1	0	0
ENSG00000280041	37	47	59	21	37	70	7	15	0	2
ENSG00000280040	0	0	1	0	0	3	0	0	0	0
ENSG00000280039	1	4	4	5	0	4	2	15	0	0
ENSG00000280038	12	16	33	18	5	34	8	65	0	0
ENSG00000280037	0	5	0	0	0	0	1	5	0	0
ENSG00000280036	0	1	0	0	0	0	3	5	0	0
ENSG00000280035	2	2	0	0	0	1	3	0	0	0
ENSG00000280034	30	47	61	24	30	92	20	9	0	3
ENSG00000280033	0	0	0	3	0	1	16	16	0	0
ENSG00000280032	67	77	97	56	63	174	4	18	0	0
ENSG00000280031	0	0	0	0	0	0	0	0	0	0
ENSG00000280029	0	0	0	0	0	0	0	0	0	0
ENSG00000280028	0	0	0	0	0	0	0	0	0	0
ENSG00000280027	2	0	1	0	1	2	3	3	0	0
ENSG00000280026	0	0	0	0	0	0	0	0	0	0
ENSG00000280025	0	0	0	0	0	0	0	4	0	0
ENSG00000280024	0	0	0	0	0	0	0	12	0	0
ENSG00000280023	0	0	1	0	0	0	1	0	0	0
ENSG00000280022	0	10	9	2	1	6	2	5	0	0
ENSG00000280021	0	0	0	0	0	0	0	0	0	0
ENSG00000280020	0	0	0	0	0	0	0	0	0	0
ENSG00000280019	0	0	0	0	0	0	0	0	0	0
ENSG00000280018	14	3	12	6	5	38	20	15	0	0
ENSG00000280017	0	0	0	0	0	0	3	18	0	0
ENSG00000280016	0	0	0	0	0	0	0	0	0	0
ENSG00000280015	0	0	0	0	0	0	0	0	0	0
ENSG00000280014	0	0	0	0	0	0	0	0	0	0
ENSG00000280013	22	29	51	26	16	79	0	0	0	0
ENSG00000280012	0	0	17	0	0	4	1	0	1	0
ENSG00000280011	0	0	4	0	0	1	9	17	0	0
ENSG00000280010	0	5	10	6	4	6	47	2	0	0
ENSG00000280009	0	0	0	0	0	0	0	0	0	0
ENSG00000280008	0	0	0	0	0	0	13	7	0	0
ENSG00000280007	6	9	9	5	11	17	25	65	1	1
ENSG00000280006	51	79	96	42	51	164	160	165	0	3
ENSG00000280005	0	0	0	0	0	0	0	0	0	0
ENSG00000280004	0	0	0	0	0	0	1	0	0	0
ENSG00000280003	1	0	0	0	0	0	10	0	0	3
ENSG00000280002	0	0	0	0	0	0	9	6	0	0
ENSG00000280001	0	0	0	0	0	0	0	0	0	0
ENSG00000280000	0	0	0	0	0	0	0	0	0	0
ENSG00000279999	0	0	0	0	0	0	0	0	0	0
ENSG00000279998	1	0	2	0	0	0	1	0	0	0
ENSG00000279997	0	0	1	0	0	0	0	7	0	0
ENSG00000279996	0	0	0	1	0	0	16	0	0	0
ENSG00000279995	0	0	0	0	0	0	0	0	0	0

ENSG00000279994	0	0	0	0	0	0	0	0	0	0
ENSG00000279993	0	0	0	0	0	0	16	0	0	0
ENSG00000279992	0	0	0	0	0	0	0	0	0	0
ENSG00000279991	0	0	0	0	0	0	2	1	0	0
ENSG00000279990	0	0	0	0	0	0	0	0	0	0
ENSG00000279989	12	20	19	25	13	55	3	0	0	0
ENSG00000279988	0	0	0	0	0	0	1	0	0	0
ENSG00000279987	1	0	0	0	0	0	0	0	0	0
ENSG00000279986	0	0	0	0	0	0	0	0	0	0
ENSG00000279985	0	0	0	0	0	0	5	0	0	0
ENSG00000279983	0	0	0	0	0	0	0	0	0	0
ENSG00000279982	2	6	3	0	2	3	2	0	0	0
ENSG00000279981	0	0	0	0	0	0	8	3	0	0
ENSG00000279980	0	1	1	0	0	0	1	1	0	0
ENSG00000279979	0	0	0	0	0	0	0	0	0	0
ENSG00000279978	67	76	105	38	67	140	390	636	6	35
ENSG00000279977	1	0	0	1	0	2	8	2	0	0
ENSG00000279976	0	0	0	0	0	0	9	6	0	0
ENSG00000279975	0	0	0	0	0	0	0	1	0	0
ENSG00000279973	0	0	0	0	0	0	0	0	0	0
ENSG00000279972	0	0	0	0	0	0	0	0	0	0
ENSG00000279971	0	0	0	0	0	0	1	3	0	0
ENSG00000279970	2	1	2	3	1	0	21	2	0	0
ENSG00000279968	3	6	4	3	1	4	0	9	0	0
ENSG00000279967	5	5	2	3	2	7	1	4	0	0
ENSG00000279966	0	0	0	0	0	0	0	0	0	0
ENSG00000279965	9	5	5	4	1	8	4	3	0	0
ENSG00000279964	1	0	0	0	0	0	3	0	0	0
ENSG00000279963	0	0	0	0	0	0	0	0	0	0
ENSG00000279962	0	1	0	0	0	0	2	4	0	0
ENSG00000279961	0	0	0	0	0	0	0	0	0	0
ENSG00000279960	0	0	0	1	0	0	0	11	1	4
ENSG00000279959	0	0	0	0	0	0	0	0	0	0
ENSG00000279958	0	0	0	0	0	0	0	0	0	0
ENSG00000279957	0	0	0	0	0	0	2	3	0	0
ENSG00000279956	0	1	0	0	0	0	0	0	0	0
ENSG00000279955	9	7	3	9	5	22	3	10	0	0
ENSG00000279954	0	0	1	0	0	0	0	0	0	0
ENSG00000279953	2	1	3	0	0	0	4	4	0	0
ENSG00000279952	0	0	0	0	0	0	0	0	0	0
ENSG00000279951	1	2	0	1	1	0	6	4	0	0
ENSG00000279950	0	0	0	0	0	0	0	0	0	0
ENSG00000279949	0	0	1	0	0	1	0	0	0	0
ENSG00000279948	3	8	8	1	2	7	6	9	0	0
ENSG00000279947	0	0	0	0	0	0	0	0	0	0
ENSG00000279946	0	0	0	0	0	0	21	8	0	0
ENSG00000279945	0	0	0	0	0	0	0	0	0	0
ENSG00000279944	0	0	0	0	0	0	0	0	0	0
ENSG00000279943	0	0	2	0	0	0	7	4	0	0
ENSG00000279942	0	0	0	0	0	0	5	0	0	0
ENSG00000279941	0	0	0	0	0	0	0	0	0	0
ENSG00000279940	0	0	0	0	0	0	20	0	0	0
ENSG00000279939	0	0	0	0	0	0	0	0	0	0
ENSG00000279938	0	0	0	0	0	0	0	0	0	0
ENSG00000279937	2	0	1	1	0	0	1	7	0	0
ENSG00000279936	2	0	0	0	0	0	2	0	0	0
ENSG00000279935	0	0	0	0	0	0	0	0	0	0

ENSG00000279934	0	0	0	0	0	0	0	0	0	0
ENSG00000279933	0	0	0	0	0	0	0	0	0	1
ENSG00000279932	0	0	0	0	0	0	0	0	0	0
ENSG00000279931	0	0	0	0	0	0	0	0	0	0
ENSG00000279930	4	9	5	2	1	11	2	4	0	0
ENSG00000279929	1	0	0	0	0	0	0	6	0	0
ENSG00000279928	0	0	0	0	0	1	3	0	0	0
ENSG00000279927	0	0	0	0	0	0	1	6	0	0
ENSG00000279926	0	2	2	2	0	2	18	6	0	0
ENSG00000279925	0	0	0	0	0	0	0	0	0	0
ENSG00000279924	0	0	0	0	0	0	0	0	0	0
ENSG00000279923	6	3	7	1	3	2	12	10	0	0
ENSG00000279922	0	0	0	0	0	0	0	0	0	0
ENSG00000279921	40	41	64	31	40	82	67	31	0	0
ENSG00000279920	0	0	0	0	0	0	0	0	0	0
ENSG00000279919	0	0	0	0	0	0	0	0	0	0
ENSG00000279918	0	0	0	0	0	0	0	0	0	0
ENSG00000279917	1	0	1	0	0	3	21	59	0	0
ENSG00000279916	0	0	0	0	0	0	0	0	0	0
ENSG00000279915	0	0	0	0	0	0	0	0	0	0
ENSG00000279914	0	0	0	0	0	1	0	0	0	0
ENSG00000279913	0	3	0	2	3	4	0	0	0	4
ENSG00000279912	321	337	707	200	248	718	260	60	10	5
ENSG00000279911	0	0	0	0	0	0	0	0	0	0
ENSG00000279910	0	0	0	0	0	0	0	3	0	0
ENSG00000279909	0	0	0	0	0	0	0	0	0	0
ENSG00000279908	0	1	0	1	0	2	5	0	0	0
ENSG00000279907	0	0	0	0	0	0	0	0	0	0
ENSG00000279906	0	0	0	0	0	0	0	0	0	0
ENSG00000279905	0	0	1	0	0	0	0	3	0	0
ENSG00000279904	0	0	0	0	0	0	0	8	0	0
ENSG00000279903	0	0	0	0	0	0	1	2	0	0
ENSG00000279901	4	3	0	2	1	5	9	2	0	0
ENSG00000279900	3	1	11	5	4	17	1	0	0	0
ENSG00000279899	0	0	0	0	0	0	0	0	0	0
ENSG00000279898	0	0	0	0	0	0	0	0	0	0
ENSG00000279897	0	0	0	0	0	0	1	1	0	0
ENSG00000279896	0	0	0	0	0	0	0	0	0	0
ENSG00000279895	0	0	0	0	0	0	0	0	0	0
ENSG00000279894	1	7	0	0	0	0	3	7	0	0
ENSG00000279893	0	0	0	0	0	0	0	0	0	0
ENSG00000279892	0	0	0	0	0	0	0	0	0	0
ENSG00000279891	0	1	0	0	0	0	24	0	0	0
ENSG00000279890	0	0	0	0	0	0	0	0	0	0
ENSG00000279889	0	0	0	0	0	0	0	0	0	0
ENSG00000279888	0	0	0	0	0	0	0	0	0	0
ENSG00000279887	0	0	0	0	0	0	0	3	0	0
ENSG00000279886	0	0	0	0	0	0	0	0	0	0
ENSG00000279885	0	0	0	0	0	0	11	6	0	0
ENSG00000279884	0	0	2	0	0	0	23	2	0	0
ENSG00000279883	0	0	0	0	0	0	3	2	0	0
ENSG00000279882	0	8	15	1	2	12	6	2	0	0
ENSG00000279881	0	0	0	0	0	0	0	18	0	0
ENSG00000279880	2	1	0	0	0	7	3	0	0	0
ENSG00000279879	1	3	1	1	0	1	2	6	0	1
ENSG00000279878	1	0	1	3	2	4	2	0	0	0
ENSG00000279877	0	0	0	0	0	0	0	0	0	0

ENSG00000279876	0	0	0	0	0	0	0	0	0	0
ENSG00000279875	0	0	0	0	0	0	1	0	0	0
ENSG00000279874	0	0	0	0	0	0	0	0	0	0
ENSG00000279873	2	0	0	1	0	4	7	0	0	3
ENSG00000279872	56	59	85	84	43	181	64	131	0	2
ENSG00000279871	0	0	0	0	0	0	0	0	0	0
ENSG00000279870	0	0	0	0	0	0	0	0	0	0
ENSG00000279869	0	0	0	0	0	0	0	0	0	0
ENSG00000279868	0	0	0	0	0	0	0	0	0	0
ENSG00000279867	0	0	0	0	0	0	0	0	0	0
ENSG00000279866	3	1	1	0	1	7	6	24	0	2
ENSG00000279865	0	1	5	1	3	10	12	5	0	3
ENSG00000279864	0	0	0	0	0	0	0	0	0	0
ENSG00000279863	7	5	2	8	7	18	34	32	0	8
ENSG00000279862	0	0	0	0	1	0	13	0	0	44
ENSG00000279861	8	17	28	4	5	27	49	50	0	17
ENSG00000279860	0	0	0	0	0	0	0	6	0	0
ENSG00000279859	0	0	0	0	0	0	0	0	0	0
ENSG00000279858	0	0	0	0	0	0	6	1	0	0
ENSG00000279857	0	0	0	0	0	0	0	0	0	0
ENSG00000279856	0	0	0	0	0	0	0	1	0	0
ENSG00000279855	0	0	0	0	0	0	0	0	0	0
ENSG00000279854	0	0	0	0	0	0	0	0	0	0
ENSG00000279853	1	0	0	0	0	0	3	0	0	0
ENSG00000279852	0	3	7	2	5	18	17	1	0	0
ENSG00000279851	0	1	2	0	0	1	0	1	0	0
ENSG00000279850	0	0	0	0	0	0	0	0	0	0
ENSG00000279849	5	4	23	7	6	8	35	17	0	2
ENSG00000279848	0	0	0	0	0	0	1	0	0	0
ENSG00000279847	0	0	0	0	0	0	0	0	0	0
ENSG00000279846	0	0	0	0	0	0	0	0	0	0
ENSG00000279845	0	1	0	0	0	0	4	1	0	0
ENSG00000279844	0	0	0	0	0	0	1	0	0	0
ENSG00000279843	0	0	0	0	0	0	0	0	0	0
ENSG00000279842	0	0	0	0	0	0	0	0	0	0
ENSG00000279841	0	1	0	0	0	8	31	16	0	0
ENSG00000279840	0	0	0	0	0	0	0	2	0	0
ENSG00000279839	0	0	0	0	0	0	6	5	0	0
ENSG00000279838	8	6	18	8	5	7	24	18	2	7
ENSG00000279837	3	5	4	0	2	8	2	2	0	0
ENSG00000279836	0	0	0	0	0	0	1	0	0	0
ENSG00000279835	0	0	0	0	0	0	0	0	0	0
ENSG00000279834	0	0	0	0	0	0	3	4	0	0
ENSG00000279833	0	1	2	1	6	7	16	12	0	0
ENSG00000279832	0	0	0	0	0	0	0	0	0	0
ENSG00000279831	0	0	0	0	0	0	0	0	0	0
ENSG00000279829	0	1	3	0	0	2	2	0	0	0
ENSG00000279828	0	0	0	0	0	0	0	0	0	0
ENSG00000279827	0	0	0	0	0	0	3	0	0	0
ENSG00000279826	0	0	0	0	0	0	0	0	0	0
ENSG00000279825	0	2	2	0	1	3	39	2	0	0
ENSG00000279824	0	0	0	0	0	0	0	0	0	0
ENSG00000279823	0	0	0	0	0	0	0	0	0	0
ENSG00000279822	3	0	0	0	1	3	8	2	0	0
ENSG00000279821	26	32	44	30	32	77	33	20	0	0
ENSG00000279820	0	0	0	0	0	0	0	0	0	0
ENSG00000279819	0	0	0	0	0	0	8	0	0	0

ENSG00000279818	0	0	0	0	0	0	0	0	0	0
ENSG00000279817	0	0	1	0	0	6	2	0	0	0
ENSG00000279816	1	0	0	0	0	0	3	0	0	0
ENSG00000279815	0	0	0	0	0	0	0	0	0	0
ENSG00000279814	0	0	0	0	0	0	1	2	0	0
ENSG00000279813	0	0	0	0	0	0	0	0	0	0
ENSG00000279812	3	2	3	3	6	8	3	3	0	0
ENSG00000279811	1	0	0	0	0	0	3	3	2	0
ENSG00000279810	0	0	0	0	0	0	3	4	0	0
ENSG00000279809	0	0	0	0	0	0	0	0	0	0
ENSG00000279808	0	0	0	0	0	0	0	3	0	0
ENSG00000279807	0	0	0	0	0	0	0	4	1	1
ENSG00000279806	1298	1206	1728	1036	1142	3360	1	7	0	0
ENSG00000279805	0	0	0	1	0	0	110	98	0	0
ENSG00000279804	0	0	0	0	0	0	2	0	0	0
ENSG00000279803	2	1	1	1	2	10	1	6	0	0
ENSG00000279802	0	0	0	1	0	0	19	13	0	0
ENSG00000279801	4	6	4	6	3	4	16	0	0	0
ENSG00000279800	561	501	932	412	440	1300	332	56	0	2
ENSG00000279799	0	2	3	0	0	4	3	5	0	0
ENSG00000279798	0	0	1	0	0	0	2	0	0	0
ENSG00000279797	0	0	0	0	0	0	0	0	0	0
ENSG00000279796	0	0	0	0	0	0	0	4	0	0
ENSG00000279795	0	2	0	2	0	1	1	2	0	0
ENSG00000279794	2	1	14	1	3	11	10	7	0	0
ENSG00000279793	0	0	0	0	0	0	0	0	0	0
ENSG00000279792	4	5	2	4	0	6	6	4	0	0
ENSG00000279791	2	1	0	0	0	2	10	2	0	0
ENSG00000279790	0	0	1	0	0	0	0	5	0	0
ENSG00000279789	20	35	58	17	26	79	9	14	0	0
ENSG00000279788	0	0	0	0	0	0	0	0	0	0
ENSG00000279787	0	0	0	0	0	0	0	0	0	0
ENSG00000279786	2	0	0	2	0	3	15	12	0	0
ENSG00000279785	0	0	0	0	0	0	1	2	0	0
ENSG00000279784	0	0	0	0	1	0	0	0	0	0
ENSG00000279783	0	0	0	0	0	0	0	0	0	0
ENSG00000279782	0	0	0	0	0	0	6	0	0	0
ENSG00000279781	1	0	0	0	0	0	3	0	0	0
ENSG00000279780	0	0	7	0	1	1	2	0	0	0
ENSG00000279779	0	0	0	0	0	0	0	0	0	0
ENSG00000279778	0	0	0	0	0	0	1	7	0	0
ENSG00000279777	23	51	91	40	38	125	87	115	0	1
ENSG00000279776	0	2	0	0	0	4	6	4	0	0
ENSG00000279775	0	0	0	0	0	0	1	0	0	0
ENSG00000279774	1	0	0	0	0	0	0	2	0	0
ENSG00000279773	5	9	7	6	3	24	1	0	0	0
ENSG00000279772	0	0	0	0	0	0	0	0	0	0
ENSG00000279771	0	0	0	0	0	0	0	0	0	0
ENSG00000279770	0	0	0	0	0	0	1	0	0	0
ENSG00000279769	2	6	8	2	6	23	1	0	0	0
ENSG00000279768	0	0	0	0	0	0	0	0	0	0
ENSG00000279767	0	0	0	0	0	0	0	0	0	0
ENSG00000279766	0	3	3	0	0	2	53	10	0	0
ENSG00000279765	0	0	0	0	0	0	0	0	0	0
ENSG00000279764	0	0	0	0	0	0	0	0	0	0
ENSG00000279763	0	1	0	2	0	8	14	0	0	0
ENSG00000279762	1	0	0	0	1	1	17	11	0	0

ENSG00000279761	0	0	0	0	0	0	0	0	0	0
ENSG00000279760	0	0	0	0	0	0	0	0	0	0
ENSG00000279759	45	113	112	68	83	178	20	8	0	0
ENSG00000279758	0	0	0	0	0	0	4	0	0	0
ENSG00000279757	0	1	0	0	0	0	0	0	0	0
ENSG00000279756	0	0	0	0	0	0	0	1	0	1
ENSG00000279755	0	0	0	0	0	0	0	0	0	0
ENSG00000279754	0	0	0	0	0	0	0	0	0	0
ENSG00000279753	0	0	0	0	0	0	0	0	0	0
ENSG00000279752	0	0	0	0	0	0	0	4	0	0
ENSG00000279751	0	0	0	0	0	0	0	0	0	0
ENSG00000279750	0	0	0	0	0	0	4	7	0	0
ENSG00000279749	0	0	0	0	0	0	0	0	0	0
ENSG00000279748	0	0	0	0	1	0	8	3	0	0
ENSG00000279747	0	0	0	0	0	0	0	0	0	0
ENSG00000279746	0	0	0	0	0	0	0	0	0	0
ENSG00000279745	0	0	0	0	0	0	4	0	0	0
ENSG00000279744	14	11	10	6	8	19	28	13	0	0
ENSG00000279743	0	0	0	0	0	0	0	0	0	0
ENSG00000279742	45	85	100	48	50	122	67	833	10	67
ENSG00000279741	0	2	0	1	0	0	0	0	0	0
ENSG00000279740	0	0	0	0	0	0	0	0	0	0
ENSG00000279739	1	0	10	4	2	4	0	0	0	0
ENSG00000279738	23	17	30	11	17	50	32	65	2	4
ENSG00000279737	0	0	0	0	0	0	0	0	0	0
ENSG00000279736	0	0	0	0	0	0	0	0	0	0
ENSG00000279735	0	0	0	1	1	2	0	0	0	0
ENSG00000279734	1	0	13	2	1	3	1	0	0	0
ENSG00000279733	0	0	0	0	0	0	2	0	0	0
ENSG00000279732	0	0	0	0	0	0	0	0	0	0
ENSG00000279730	3	0	18	2	3	12	6	1	0	0
ENSG00000279729	0	0	0	0	0	0	16	1	0	0
ENSG00000279728	0	0	0	0	0	0	0	0	0	0
ENSG00000279727	0	1	0	2	0	0	6	5	0	0
ENSG00000279726	0	0	0	0	0	0	0	0	0	0
ENSG00000279725	0	0	0	0	0	0	0	0	0	0
ENSG00000279724	0	0	0	0	0	0	0	1	0	0
ENSG00000279723	0	0	0	0	0	0	0	0	0	0
ENSG00000279722	1	6	6	5	4	4	30	21	0	0
ENSG00000279721	0	1	0	0	0	0	1	6	0	1
ENSG00000279720	2	2	14	1	1	3	4	1	0	0
ENSG00000279719	0	0	0	0	0	0	0	0	0	0
ENSG00000279718	2	3	6	2	1	5	3	0	0	0
ENSG00000279717	0	0	0	0	0	0	0	4	0	0
ENSG00000279716	2	6	1	4	1	4	23	7	0	0
ENSG00000279715	0	0	0	0	0	0	0	0	0	0
ENSG00000279714	4	0	5	1	1	3	8	26	0	0
ENSG00000279713	9	11	6	0	2	15	0	0	0	0
ENSG00000279712	0	0	0	0	0	0	0	2	0	0
ENSG00000279711	1	1	1	1	3	8	0	0	0	0
ENSG00000279710	0	0	0	0	0	0	0	0	0	0
ENSG00000279709	0	0	0	0	0	0	0	0	0	0
ENSG00000279708	0	0	0	0	0	0	0	0	0	0
ENSG00000279707	0	0	0	0	0	0	0	0	0	0
ENSG00000279706	1	1	0	0	0	3	7	7	0	0
ENSG00000279705	0	0	0	0	0	0	0	3	0	0
ENSG00000279704	0	0	0	0	0	0	0	0	0	0

ENSG00000279703	0	0	0	0	0	0	0	0	0	0
ENSG00000279702	0	0	0	0	0	0	0	0	0	0
ENSG00000279701	0	0	0	0	0	0	0	0	0	0
ENSG00000279700	2	4	3	0	0	1	18	2	0	0
ENSG00000279699	1	2	0	3	2	7	4	0	0	0
ENSG00000279698	0	0	0	0	0	0	1	0	0	0
ENSG00000279697	0	0	0	0	0	0	0	3	0	0
ENSG00000279696	10	10	15	3	8	16	52	8	0	0
ENSG00000279695	0	0	0	0	0	0	0	0	0	0
ENSG00000279694	0	0	0	0	0	0	1	0	0	0
ENSG00000279693	0	0	0	0	0	0	2	0	0	0
ENSG00000279692	1	0	0	0	0	0	6	9	0	5
ENSG00000279691	0	0	0	0	0	0	23	10	0	0
ENSG00000279690	0	0	0	0	0	0	0	0	0	0
ENSG00000279689	3	10	7	3	8	13	13	4	0	3
ENSG00000279688	2	0	0	2	4	7	4	3	0	0
ENSG00000279687	0	0	0	0	0	0	2	3	0	0
ENSG00000279686	0	0	0	0	0	0	0	0	0	0
ENSG00000279685	0	0	0	0	0	0	0	0	0	0
ENSG00000279684	2	2	3	0	0	0	0	4	0	0
ENSG00000279683	0	0	0	0	0	0	0	0	0	0
ENSG00000279682	0	0	0	0	0	0	0	2	0	0
ENSG00000279681	0	0	0	0	0	1	1	0	0	0
ENSG00000279680	0	0	0	0	0	0	0	0	0	0
ENSG00000279679	0	0	0	0	0	0	0	0	0	0
ENSG00000279678	0	0	0	0	0	0	0	0	0	0
ENSG00000279677	0	0	0	0	0	0	0	0	0	0
ENSG00000279676	9	10	1	5	3	25	1	0	0	0
ENSG00000279675	0	0	0	0	0	0	1	4	0	0
ENSG00000279674	0	0	0	0	0	0	0	0	0	0
ENSG00000279673	4	2	1	1	2	3	9	4	0	6
ENSG00000279672	7	6	13	9	7	23	12	0	0	0
ENSG00000279671	0	0	0	0	0	0	0	0	0	0
ENSG00000279670	4	3	15	6	5	11	4	4	0	0
ENSG00000279669	0	0	0	0	0	0	0	0	0	0
ENSG00000279668	0	0	0	0	0	0	0	0	0	0
ENSG00000279667	1	1	1	0	1	5	1	0	0	0
ENSG00000279666	0	0	0	0	0	0	0	0	0	0
ENSG00000279665	0	0	1	0	0	0	1	0	0	0
ENSG00000279664	0	0	0	0	0	0	0	0	0	0
ENSG00000279663	0	0	0	0	0	0	0	2	1	0
ENSG00000279662	1	9	11	7	5	12	173	15	0	0
ENSG00000279660	0	0	0	0	0	0	14	9	0	0
ENSG00000279659	1	2	6	1	0	0	1	1	0	0
ENSG00000279658	0	0	0	0	0	0	4	0	0	0
ENSG00000279657	0	0	0	0	0	0	0	16	0	0
ENSG00000279656	11	15	15	4	10	25	24	28	0	0
ENSG00000279655	0	0	0	0	0	0	0	0	0	0
ENSG00000279654	45	56	68	25	37	109	18	11	0	4
ENSG00000279653	1	1	0	1	0	0	1	2	0	0
ENSG00000279652	0	0	0	0	0	0	15	26	0	0
ENSG00000279651	0	0	0	0	0	0	0	0	0	0
ENSG00000279650	0	0	4	0	0	0	0	0	0	0
ENSG00000279649	13	11	23	10	9	22	14	17	0	0
ENSG00000279648	0	3	0	1	0	0	0	18	0	0
ENSG00000279647	0	0	0	0	0	0	0	0	0	0
ENSG00000279646	37	17	6	26	38	73	2	0	0	0

ENSG00000279645	1	0	0	0	1	2	0	0	3	0
ENSG00000279644	0	0	0	0	0	0	2	0	0	0
ENSG00000279642	0	0	0	0	0	0	0	0	0	0
ENSG00000279641	5	2	8	5	6	13	73	1	0	0
ENSG00000279640	0	1	0	0	0	1	9	0	0	0
ENSG00000279639	0	0	0	0	0	0	0	0	0	0
ENSG00000279638	0	0	0	0	0	0	0	0	0	0
ENSG00000279637	0	0	0	0	0	0	16	0	0	0
ENSG00000279636	1	1	1	1	3	7	10	3	0	0
ENSG00000279635	0	0	0	0	0	0	0	0	0	0
ENSG00000279634	0	0	0	0	0	0	0	0	0	0
ENSG00000279633	1	0	2	0	3	0	11	2	0	0
ENSG00000279632	4	7	4	0	0	1	4	1	0	0
ENSG00000279631	2	2	6	2	6	4	10	4	0	0
ENSG00000279630	0	0	0	0	0	0	0	0	0	0
ENSG00000279629	0	0	0	0	0	0	3	0	0	0
ENSG00000279628	0	0	0	1	0	3	0	2	0	0
ENSG00000279627	0	0	0	0	0	0	0	0	0	0
ENSG00000279626	0	0	0	0	0	0	0	0	0	0
ENSG00000279625	0	0	0	0	0	0	0	0	0	0
ENSG00000279624	0	0	0	0	0	0	0	0	0	0
ENSG00000279623	0	0	0	0	0	0	4	0	0	0
ENSG00000279622	0	0	0	0	0	0	4	4	0	0
ENSG00000279621	0	0	0	0	0	0	0	0	0	0
ENSG00000279620	0	0	0	0	0	0	2	0	0	0
ENSG00000279619	11	17	16	5	8	9	0	0	0	0
ENSG00000279618	0	0	0	0	0	0	0	0	0	0
ENSG00000279617	0	0	0	0	0	0	0	0	0	0
ENSG00000279616	0	0	1	0	0	1	3	6	0	0
ENSG00000279615	0	0	0	0	0	0	0	0	0	0
ENSG00000279613	1	0	0	0	0	1	1	0	0	0
ENSG00000279611	2	1	5	1	3	9	3	0	0	0
ENSG00000279610	0	0	0	0	0	0	0	0	0	0
ENSG00000279609	1	0	0	0	0	0	0	0	0	0
ENSG00000279608	2	1	4	1	3	8	28	10	0	0
ENSG00000279607	0	0	0	0	0	0	0	3	0	0
ENSG00000279606	0	0	0	0	0	0	0	2	0	0
ENSG00000279605	0	2	1	3	1	1	21	21	0	0
ENSG00000279604	0	0	0	0	0	0	0	0	0	0
ENSG00000279603	0	0	0	0	0	0	0	0	0	0
ENSG00000279602	3	8	8	3	2	14	121	119	0	0
ENSG00000279601	0	1	3	0	0	1	3	4	0	0
ENSG00000279600	0	0	0	0	0	0	0	0	0	0
ENSG00000279599	0	0	0	0	0	0	0	0	0	0
ENSG00000279598	0	1	0	0	0	0	4	5	0	0
ENSG00000279597	0	0	0	0	0	0	0	0	0	0
ENSG00000279596	0	0	0	0	0	0	0	0	0	0
ENSG00000279595	1	1	3	2	0	7	3	2	0	0
ENSG00000279594	0	0	0	1	0	0	13	10	0	0
ENSG00000279593	0	0	0	0	0	0	0	0	0	0
ENSG00000279592	0	0	0	0	0	0	0	0	0	0
ENSG00000279591	0	0	0	0	0	0	1	1	0	0
ENSG00000279590	0	0	0	0	0	0	0	5	0	0
ENSG00000279589	0	0	0	0	0	4	0	8	0	0
ENSG00000279588	0	0	0	0	0	0	0	4	0	0
ENSG00000279587	0	0	0	0	0	0	1	0	0	0
ENSG00000279586	0	0	0	0	0	0	1	0	0	0

ENSG00000279585	0	0	0	0	0	0	0	0	0	0
ENSG00000279584	0	0	0	0	0	0	0	5	0	0
ENSG00000279583	4	0	1	0	1	1	16	0	0	0
ENSG00000279582	1	0	0	0	0	0	0	9	0	0
ENSG00000279581	0	0	0	0	0	0	64	20	0	0
ENSG00000279580	0	0	0	0	0	0	0	0	0	0
ENSG00000279579	0	3	11	1	4	7	3	0	0	0
ENSG00000279578	0	0	0	0	0	0	0	0	0	0
ENSG00000279576	0	0	0	0	0	0	0	0	0	0
ENSG00000279575	0	0	0	0	0	0	0	2	0	0
ENSG00000279574	0	0	0	0	0	0	0	0	0	0
ENSG00000279573	13	27	37	18	13	46	39	47	0	6
ENSG00000279572	0	0	0	0	0	0	0	0	0	0
ENSG00000279571	7	3	5	4	2	17	22	22	0	0
ENSG00000279570	0	4	3	1	5	1	7	0	0	0
ENSG00000279569	1	0	1	1	0	0	3	0	0	0
ENSG00000279568	7	9	2	7	5	40	16	0	0	0
ENSG00000279567	0	0	0	0	0	0	1	7	0	2
ENSG00000279565	0	0	0	0	0	0	0	2	0	0
ENSG00000279564	0	0	0	0	0	0	0	0	0	0
ENSG00000279563	24	15	33	25	26	81	17	8	0	0
ENSG00000279562	0	0	0	0	0	0	0	0	0	0
ENSG00000279561	2	0	3	3	0	1	6	5	0	0
ENSG00000279560	94	52	58	93	99	289	33	28	0	0
ENSG00000279559	0	0	0	0	0	0	0	0	0	0
ENSG00000279558	0	0	0	0	0	0	0	0	0	0
ENSG00000279557	0	0	0	0	0	0	0	0	0	0
ENSG00000279556	0	0	0	0	0	0	0	0	0	0
ENSG00000279555	0	2	0	0	0	0	7	3	0	0
ENSG00000279554	0	0	0	0	0	0	0	0	0	0
ENSG00000279553	0	0	0	0	0	0	0	0	0	0
ENSG00000279551	0	0	0	0	0	0	0	0	0	0
ENSG00000279550	0	0	0	0	0	0	0	0	0	0
ENSG00000279549	0	1	2	3	3	9	7	2	0	1
ENSG00000279548	0	0	0	0	0	0	4	4	0	0
ENSG00000279547	0	0	0	0	0	0	0	0	0	0
ENSG00000279546	0	0	0	0	0	0	0	0	0	0
ENSG00000279545	0	0	0	0	0	0	0	0	0	0
ENSG00000279544	0	0	0	0	0	0	12	5	0	0
ENSG00000279543	0	0	0	0	0	0	0	0	0	0
ENSG00000279542	0	0	0	0	0	0	0	0	0	0
ENSG00000279541	2	2	0	0	0	6	32	16	0	0
ENSG00000279540	0	0	0	0	0	0	0	0	0	0
ENSG00000279539	7	5	18	4	5	10	15	0	0	0
ENSG00000279538	0	0	0	0	0	0	2	0	0	0
ENSG00000279537	0	0	3	1	0	2	3	0	0	0
ENSG00000279536	0	0	0	1	0	1	6	0	0	0
ENSG00000279535	0	0	0	0	0	0	0	0	0	0
ENSG00000279534	0	0	0	0	0	0	0	0	0	0
ENSG00000279533	42	50	65	23	35	102	22	9	0	0
ENSG00000279532	2	1	2	0	0	2	0	2	0	0
ENSG00000279531	0	0	0	0	0	0	0	0	0	0
ENSG00000279530	0	1	10	0	3	5	18	0	0	0
ENSG00000279529	4	9	7	11	11	23	86	18	0	0
ENSG00000279528	14	5	17	5	6	19	50	27	0	0
ENSG00000279527	0	0	0	0	0	0	0	0	0	0
ENSG00000279526	0	0	0	0	1	0	24	4	0	0

ENSG00000279525	0	0	0	0	0	0	0	0	0	0
ENSG00000279524	24	30	33	11	17	61	15	7	1	2
ENSG00000279523	0	0	0	0	0	0	0	0	0	0
ENSG00000279522	1	1	9	4	4	12	37	10	1	2
ENSG00000279521	0	0	0	0	0	0	0	6	0	0
ENSG00000279520	7	17	12	17	4	35	28	28	0	0
ENSG00000279519	28	36	56	34	35	78	30	20	0	0
ENSG00000279518	6	3	19	9	1	23	105	8	0	0
ENSG00000279517	0	0	0	0	0	0	0	0	0	0
ENSG00000279516	0	0	0	0	0	0	0	0	0	0
ENSG00000279515	0	0	0	0	0	0	0	0	0	0
ENSG00000279514	0	0	0	0	0	0	0	0	0	0
ENSG00000279513	0	1	1	0	0	0	1	0	0	0
ENSG00000279512	0	0	0	0	0	0	4	0	0	0
ENSG00000279511	0	0	0	0	0	0	0	0	0	0
ENSG00000279510	13	21	32	12	18	20	8	6	0	0
ENSG00000279509	0	0	0	0	0	0	0	0	0	0
ENSG00000279508	3	8	4	8	6	12	17	10	0	10
ENSG00000279507	0	0	0	0	0	1	1	4	0	0
ENSG00000279506	0	0	0	0	0	0	0	0	0	0
ENSG00000279505	0	0	0	0	0	0	0	0	0	0
ENSG00000279504	0	0	0	0	0	0	0	0	0	0
ENSG00000279503	0	0	0	0	0	0	0	0	0	0
ENSG00000279502	0	0	0	0	0	1	2	6	0	0
ENSG00000279501	0	0	0	0	0	0	0	0	0	0
ENSG00000279500	6	5	4	4	7	29	5	3	0	0
ENSG00000279499	0	0	0	0	0	0	0	0	0	0
ENSG00000279498	0	0	0	0	0	0	0	0	0	0
ENSG00000279497	0	0	0	0	0	0	0	0	0	0
ENSG00000279496	0	0	0	0	0	0	67	252	0	6
ENSG00000279495	26	17	43	19	27	61	23	5	0	0
ENSG00000279494	0	0	0	0	0	0	3	9	0	0
ENSG00000279493	0	0	0	0	0	0	0	0	0	0
ENSG00000279492	0	0	0	0	0	0	0	0	0	0
ENSG00000279491	1	0	4	2	0	5	23	16	1	0
ENSG00000279490	0	0	0	0	0	0	4	0	0	0
ENSG00000279489	1	0	2	2	1	7	20	7	0	0
ENSG00000279488	0	0	0	0	0	0	0	0	0	0
ENSG00000279487	0	0	1	0	1	1	0	0	0	0
ENSG00000279486	0	0	0	0	0	0	0	0	0	0
ENSG00000279485	0	0	0	0	0	0	0	0	0	0
ENSG00000279484	0	0	0	0	0	4	1	26	0	0
ENSG00000279483	2950	2541	4871	2406	2374	8270	15873	8712	43	410
ENSG00000279482	0	0	0	0	0	0	0	0	0	0
ENSG00000279481	4	2	8	3	3	5	14	4	5	8
ENSG00000279480	0	0	0	0	0	0	0	0	0	0
ENSG00000279479	0	0	0	0	0	0	0	0	0	0
ENSG00000279478	0	0	0	0	0	0	0	0	0	0
ENSG00000279477	0	0	0	0	0	0	0	0	0	0
ENSG00000279476	0	0	3	0	0	1	38	4	0	4
ENSG00000279475	0	0	0	0	0	0	0	0	0	0
ENSG00000279474	0	0	0	1	1	0	9	6	0	3
ENSG00000279473	0	0	0	0	0	0	0	0	0	0
ENSG00000279472	0	0	0	0	0	0	0	0	0	0
ENSG00000279471	0	0	0	1	1	4	0	0	0	0
ENSG00000279470	0	0	0	0	0	0	0	0	0	0
ENSG00000279469	0	0	0	0	0	3	1	7	0	0

ENSG00000279468	10	21	25	16	16	59	6	2	0	0
ENSG00000279467	2	0	1	1	1	3	1	22	0	2
ENSG00000279466	0	0	0	0	0	0	0	0	0	0
ENSG00000279465	0	0	0	0	0	0	2	3	0	0
ENSG00000279464	3	8	8	5	7	18	56	35	0	2
ENSG00000279463	0	0	0	0	0	0	0	0	0	0
ENSG00000279462	0	0	0	0	0	0	0	3	0	0
ENSG00000279461	0	1	2	0	0	2	5	2	0	1
ENSG00000279460	0	0	0	0	0	0	0	0	0	0
ENSG00000279459	1	0	0	0	0	6	8	0	0	0
ENSG00000279458	4	5	11	0	0	5	0	0	0	0
ENSG00000279457	241	280	399	277	212	645	854	1384	0	10
ENSG00000279456	6	7	15	5	6	20	7	5	0	0
ENSG00000279455	0	0	0	0	0	0	0	0	0	0
ENSG00000279454	0	0	0	0	0	0	0	0	0	0
ENSG00000279453	13	25	34	15	23	44	194	196	0	0
ENSG00000279452	6	12	5	3	4	20	26	0	0	0
ENSG00000279451	0	0	0	0	0	0	0	0	0	0
ENSG00000279450	0	0	0	0	0	0	0	0	0	0
ENSG00000279449	0	0	0	0	0	0	0	0	0	0
ENSG00000279448	2	5	7	1	2	12	3	1	0	0
ENSG00000279447	0	0	0	0	0	0	64	68	0	0
ENSG00000279446	0	0	0	0	0	0	0	0	0	0
ENSG00000279445	0	0	0	0	0	0	0	4	0	0
ENSG00000279444	0	0	0	0	0	2	0	0	0	0
ENSG00000279443	56	45	93	38	59	117	7	3	0	0
ENSG00000279442	11	22	38	7	11	27	10	0	0	0
ENSG00000279441	0	0	0	0	0	0	0	0	0	0
ENSG00000279440	0	0	0	0	0	0	0	3	0	0
ENSG00000279439	0	0	0	0	0	0	0	8	0	0
ENSG00000279438	0	0	0	0	0	0	0	0	0	0
ENSG00000279437	0	0	0	0	0	0	0	3	0	0
ENSG00000279436	0	0	0	0	0	0	0	0	0	0
ENSG00000279435	0	0	0	0	0	0	0	0	0	0
ENSG00000279434	3	3	2	1	1	10	30	24	0	1
ENSG00000279433	0	0	0	2	1	0	12	6	0	0
ENSG00000279432	2	1	6	2	4	13	13	11	0	1
ENSG00000279431	0	1	0	0	0	0	0	0	0	0
ENSG00000279430	2	1	0	1	1	3	27	34	0	0
ENSG00000279429	0	0	0	0	0	0	0	2	0	0
ENSG00000279428	1	1	0	0	1	0	1	3	0	0
ENSG00000279427	0	0	0	0	0	0	1	0	0	0
ENSG00000279426	0	0	0	0	0	0	0	0	0	0
ENSG00000279425	3	9	9	2	4	4	18	9	0	0
ENSG00000279424	0	1	0	0	0	0	6	9	0	0
ENSG00000279423	97	106	127	105	81	324	835	453	0	57
ENSG00000279422	0	2	2	3	1	5	0	0	0	0
ENSG00000279421	0	0	0	0	0	0	0	0	0	0
ENSG00000279420	0	0	0	0	0	0	0	0	0	0
ENSG00000279419	5	5	8	2	6	4	6	13	0	0
ENSG00000279418	0	0	0	0	0	0	0	0	0	0
ENSG00000279417	214	246	541	154	181	548	278	50	7	4
ENSG00000279416	0	0	0	0	0	0	0	4	0	0
ENSG00000279415	0	3	0	0	0	0	10	0	0	5
ENSG00000279414	1	2	0	2	1	0	3	3	0	0
ENSG00000279413	1	1	1	2	1	1	9	6	0	0
ENSG00000279412	16	38	42	21	23	75	9	0	0	0

ENSG00000279411	0	0	0	0	0	0	0	0	0	0
ENSG00000279410	0	0	0	0	0	0	4	0	0	0
ENSG00000279409	1	3	0	0	0	0	0	0	0	0
ENSG00000279408	0	0	0	0	0	0	0	0	0	0
ENSG00000279407	28	23	20	23	26	57	13	14	0	0
ENSG00000279406	3	3	8	5	3	17	8	9	0	0
ENSG00000279405	1	0	0	1	1	0	0	0	0	0
ENSG00000279404	2	0	5	0	0	0	2	0	0	0
ENSG00000279403	0	0	0	0	0	0	0	0	0	0
ENSG00000279402	0	0	0	0	0	0	0	0	0	0
ENSG00000279401	0	0	0	0	0	0	0	0	0	0
ENSG00000279400	2	0	1	1	1	0	8	8	0	0
ENSG00000279399	0	0	0	0	0	0	0	0	0	0
ENSG00000279398	0	0	0	0	0	0	0	0	0	0
ENSG00000279397	0	0	0	0	0	0	0	0	0	0
ENSG00000279396	0	0	0	0	0	0	14	1	0	0
ENSG00000279395	0	0	0	0	0	0	0	0	0	0
ENSG00000279394	0	1	1	0	0	0	6	17	0	0
ENSG00000279393	0	0	0	0	0	0	14	16	0	0
ENSG00000279392	31	42	146	20	31	64	72	11	0	8
ENSG00000279391	0	0	0	0	0	0	1	0	0	0
ENSG00000279390	1	0	0	1	1	0	69	0	0	0
ENSG00000279388	0	0	0	0	0	0	0	0	0	0
ENSG00000279387	0	0	0	0	0	0	0	0	0	0
ENSG00000279386	20	17	20	16	18	42	3	0	0	0
ENSG00000279385	23	20	47	10	14	60	13	0	0	0
ENSG00000279384	1	2	4	2	0	11	5	1	0	0
ENSG00000279383	0	0	0	0	0	0	0	0	0	0
ENSG00000279382	1	18	17	12	9	32	17	2	0	0
ENSG00000279381	0	1	0	0	0	3	0	0	0	0
ENSG00000279380	0	0	0	0	0	2	2	0	0	0
ENSG00000279379	0	0	0	0	0	0	0	0	0	0
ENSG00000279378	0	0	0	0	0	0	1	7	0	0
ENSG00000279377	2	1	12	0	1	9	42	32	0	0
ENSG00000279376	0	0	0	0	0	0	0	0	0	0
ENSG00000279375	0	0	0	0	0	0	0	0	0	0
ENSG00000279374	0	0	0	0	0	0	1	0	0	0
ENSG00000279373	0	0	7	1	0	4	2	1	0	0
ENSG00000279372	0	0	0	0	0	1	0	0	0	0
ENSG00000279371	0	0	0	0	0	0	0	0	0	0
ENSG00000279370	0	0	0	0	0	0	0	0	0	0
ENSG00000279369	3	2	1	2	3	4	22	37	0	0
ENSG00000279368	4	3	0	2	1	0	0	0	0	0
ENSG00000279367	0	0	0	0	0	0	0	0	0	0
ENSG00000279366	0	0	0	0	0	0	1	0	0	0
ENSG00000279365	2	3	3	0	1	3	0	3	0	0
ENSG00000279364	0	0	0	0	0	0	0	0	0	0
ENSG00000279363	0	0	0	0	0	0	0	0	0	0
ENSG00000279362	0	0	0	0	0	0	0	0	0	0
ENSG00000279361	0	0	0	0	0	0	19	10	0	0
ENSG00000279360	0	0	0	0	0	0	0	0	0	0
ENSG00000279359	0	0	0	0	0	0	3	13	0	0
ENSG00000279358	0	0	0	0	0	0	0	7	0	0
ENSG00000279357	1	0	4	0	0	0	3	4	0	0
ENSG00000279356	2	4	9	1	0	16	0	0	0	0
ENSG00000279355	9	9	27	8	9	39	8	9	0	0
ENSG00000279354	0	0	0	0	0	0	1	3	0	0

ENSG00000279353	0	1	0	0	0	1	4	0	0	0
ENSG00000279352	60	64	106	50	53	142	113	51	0	0
ENSG00000279351	0	0	0	0	0	0	0	0	0	0
ENSG00000279349	0	0	0	0	0	0	5	0	0	7
ENSG00000279348	31	50	64	26	31	82	36	24	0	0
ENSG00000279347	8	2	2	0	4	3	0	0	0	0
ENSG00000279346	0	0	0	0	0	0	0	0	0	0
ENSG00000279345	2	1	0	1	0	3	0	5	0	0
ENSG00000279344	0	1	2	0	0	0	8	2	0	0
ENSG00000279343	0	0	0	0	0	0	0	0	0	0
ENSG00000279342	0	0	1	0	0	0	12	9	0	0
ENSG00000279341	0	0	0	0	0	0	0	0	0	0
ENSG00000279340	0	0	0	0	0	0	0	1	0	0
ENSG00000279339	0	0	0	0	0	0	2	13	0	0
ENSG00000279338	29	41	54	30	34	65	159	113	0	0
ENSG00000279337	27	26	55	30	23	123	16	9	0	0
ENSG00000279336	0	0	0	1	2	0	0	1	0	0
ENSG00000279335	0	0	0	0	0	0	0	0	0	0
ENSG00000279334	0	0	0	0	0	0	4	0	0	0
ENSG00000279333	0	0	0	0	0	0	0	0	0	0
ENSG00000279332	0	0	0	0	0	0	2	6	0	0
ENSG00000279331	2	2	6	3	6	5	13	0	0	0
ENSG00000279330	0	1	1	0	0	1	16	1	2	0
ENSG00000279329	48	46	35	32	29	50	19	15	0	0
ENSG00000279328	0	0	0	1	1	0	12	3	0	0
ENSG00000279327	0	0	0	0	0	0	0	0	0	0
ENSG00000279326	0	0	0	0	0	0	0	0	0	0
ENSG00000279325	0	0	0	0	0	0	0	0	0	0
ENSG00000279324	0	0	0	0	0	0	0	8	0	0
ENSG00000279323	0	0	0	0	0	0	0	0	0	0
ENSG00000279322	0	0	0	0	0	0	0	0	0	0
ENSG00000279321	0	0	0	0	0	0	0	0	0	0
ENSG00000279320	0	2	1	0	0	3	60	14	0	4
ENSG00000279319	0	0	0	0	0	0	1	2	0	0
ENSG00000279317	0	0	0	0	0	0	1	4	0	0
ENSG00000279316	0	0	0	0	0	0	0	0	0	0
ENSG00000279315	0	0	0	0	2	2	0	3	0	0
ENSG00000279314	0	0	0	0	0	0	27	13	0	0
ENSG00000279313	0	0	0	0	0	0	0	0	0	0
ENSG00000279312	12	17	52	12	10	21	264	82	0	0
ENSG00000279311	2	3	0	1	2	15	4	12	0	0
ENSG00000279310	0	0	0	0	0	0	1	0	0	0
ENSG00000279309	0	0	0	0	0	0	0	0	0	0
ENSG00000279308	0	0	0	0	0	0	0	0	0	0
ENSG00000279307	0	3	7	2	0	4	27	5	0	0
ENSG00000279306	0	0	0	1	1	1	0	2	0	0
ENSG00000279305	0	0	0	0	0	0	2	0	0	0
ENSG00000279304	0	0	0	0	0	0	0	1	0	0
ENSG00000279303	5	5	2	3	2	7	1	4	0	0
ENSG00000279302	0	0	0	0	0	0	2	0	0	0
ENSG00000279301	0	0	0	0	0	0	0	0	0	0
ENSG00000279300	0	0	0	0	0	0	0	0	0	0
ENSG00000279299	0	0	0	0	0	0	0	0	0	0
ENSG00000279298	0	0	0	0	0	0	1	6	0	0
ENSG00000279297	0	0	0	0	0	0	0	0	0	0
ENSG00000279296	0	0	0	0	0	0	8	0	0	2
ENSG00000279295	0	0	0	0	0	0	0	0	0	0

ENSG00000279294	0	0	2	1	0	0	20	6	0	0
ENSG00000279293	0	0	0	0	0	0	0	0	0	0
ENSG00000279292	0	0	0	0	0	0	0	0	0	0
ENSG00000279291	0	0	0	0	0	0	0	0	0	0
ENSG00000279290	0	0	0	0	0	0	0	0	0	0
ENSG00000279289	4	0	8	1	1	10	55	37	0	0
ENSG00000279288	0	0	0	0	0	0	0	15	0	0
ENSG00000279287	0	0	0	0	0	0	0	1	0	0
ENSG00000279286	6	4	13	4	3	20	4	5	0	0
ENSG00000279285	0	0	0	0	0	0	0	17	0	0
ENSG00000279284	0	0	0	0	0	0	0	0	0	0
ENSG00000279283	4	11	5	1	1	6	11	11	0	0
ENSG00000279282	0	0	0	0	0	0	0	0	0	0
ENSG00000279281	0	0	0	0	0	0	3	23	0	0
ENSG00000279279	0	0	0	0	0	0	0	0	0	0
ENSG00000279278	64	80	162	57	52	156	83	69	1	6
ENSG00000279277	0	1	0	0	0	0	14	0	0	0
ENSG00000279276	0	0	0	0	0	0	0	2	0	0
ENSG00000279275	0	0	0	0	0	0	0	0	0	0
ENSG00000279274	5	5	50	0	3	14	156	70	0	37
ENSG00000279273	0	0	0	0	0	0	0	1	0	0
ENSG00000279272	0	0	0	0	0	0	0	0	0	0
ENSG00000279271	0	0	0	0	0	0	0	0	0	0
ENSG00000279270	0	0	0	0	0	0	0	0	0	0
ENSG00000279269	0	0	0	0	0	0	0	0	0	0
ENSG00000279268	2	0	0	0	1	5	0	0	0	0
ENSG00000279267	19	23	31	8	14	55	150	91	0	0
ENSG00000279266	0	5	1	6	0	1	9	1	0	0
ENSG00000279265	2	7	8	5	2	3	45	19	0	0
ENSG00000279264	0	0	0	0	0	0	0	0	0	0
ENSG00000279263	0	0	0	0	0	0	0	0	0	0
ENSG00000279262	0	0	0	0	0	0	0	0	0	0
ENSG00000279261	0	0	0	0	0	0	2	0	0	0
ENSG00000279260	0	0	0	0	0	0	0	0	0	0
ENSG00000279259	1	1	3	0	0	0	4	1	0	0
ENSG00000279258	0	0	0	0	0	0	0	0	0	0
ENSG00000279257	0	0	0	0	0	0	0	0	0	0
ENSG00000279256	0	0	0	0	0	0	0	0	0	0
ENSG00000279255	2	9	14	2	7	17	0	5	0	0
ENSG00000279254	0	0	2	1	0	0	17	4	0	0
ENSG00000279253	54	72	114	50	53	134	131	100	2	1
ENSG00000279252	10	12	44	4	12	44	40	13	1	10
ENSG00000279251	0	0	0	0	0	0	0	0	0	0
ENSG00000279250	2	1	0	1	0	3	0	4	0	0
ENSG00000279249	0	0	0	0	0	0	1	0	0	0
ENSG00000279248	0	0	0	0	0	0	0	0	0	0
ENSG00000279246	0	0	0	0	0	0	0	0	0	0
ENSG00000279245	2	2	4	0	1	0	33	25	0	0
ENSG00000279244	0	0	0	0	0	0	0	1	0	0
ENSG00000279243	0	0	2	0	0	0	2	0	0	0
ENSG00000279242	0	0	0	0	0	0	0	0	0	0
ENSG00000279241	0	0	0	0	0	1	6	0	0	0
ENSG00000279240	0	0	0	0	0	0	0	0	0	0
ENSG00000279239	0	0	0	0	0	0	0	0	0	0
ENSG00000279238	0	0	0	0	0	2	0	0	0	0
ENSG00000279237	0	0	0	0	0	0	32	10	0	0
ENSG00000279236	0	0	2	1	0	2	11	0	0	0

ENSG00000279235	0	0	0	0	0	0	5	0	0	0
ENSG00000279233	26	23	36	29	27	86	32	20	0	0
ENSG00000279232	2	0	0	0	0	4	4	0	0	0
ENSG00000279231	0	2	0	0	0	0	0	2	0	0
ENSG00000279230	0	0	0	0	1	0	0	0	0	0
ENSG00000279229	0	0	0	0	0	0	0	0	0	0
ENSG00000279228	0	0	0	0	0	0	0	0	0	0
ENSG00000279227	10	4	17	11	9	17	45	15	0	6
ENSG00000279226	0	0	0	0	0	0	0	0	0	0
ENSG00000279225	5	3	5	4	6	11	5	5	0	14
ENSG00000279224	0	0	0	0	0	0	1	0	0	0
ENSG00000279223	0	0	0	0	0	0	0	0	0	0
ENSG00000279222	0	0	0	0	0	0	0	1	0	0
ENSG00000279221	0	0	0	0	0	0	0	10	0	0
ENSG00000279220	1	0	0	0	0	0	1	0	0	0
ENSG00000279219	0	0	0	0	0	0	0	0	0	0
ENSG00000279218	0	0	0	0	0	0	0	0	0	0
ENSG00000279217	0	1	9	1	1	5	5	21	0	1
ENSG00000279216	0	0	0	0	0	0	0	0	0	0
ENSG00000279214	0	0	0	0	0	0	0	0	0	0
ENSG00000279213	0	0	0	0	0	0	0	0	0	0
ENSG00000279212	0	0	0	0	0	1	2	7	0	0
ENSG00000279211	0	0	0	0	0	0	0	0	0	0
ENSG00000279210	0	0	0	0	0	0	0	0	0	0
ENSG00000279209	0	0	0	0	0	3	0	2	0	0
ENSG00000279208	1	7	23	0	2	2	16	137	0	0
ENSG00000279207	106	114	187	87	111	293	295	110	0	4
ENSG00000279206	0	0	2	0	0	0	1	0	0	0
ENSG00000279205	0	0	0	0	0	0	0	10	0	0
ENSG00000279204	0	0	5	0	0	0	4	45	0	2
ENSG00000279203	1	0	0	1	1	1	10	19	0	0
ENSG00000279202	1	0	0	0	0	0	2	0	0	0
ENSG00000279201	0	0	0	0	0	0	0	0	0	0
ENSG00000279200	0	1	2	0	0	0	2	7	0	0
ENSG00000279199	0	1	1	0	0	1	0	3	0	0
ENSG00000279198	3	5	4	3	5	2	27	23	0	0
ENSG00000279197	0	0	0	0	0	0	0	0	0	0
ENSG00000279196	1	2	2	0	2	0	1	0	0	0
ENSG00000279195	0	0	0	0	0	0	0	0	0	0
ENSG00000279194	0	0	0	0	0	0	0	0	0	0
ENSG00000279193	0	0	0	0	0	0	0	0	0	0
ENSG00000279192	21	18	47	12	14	39	15	8	0	0
ENSG00000279191	25	21	22	16	19	48	155	23	0	4
ENSG00000279190	0	0	2	0	0	0	0	3	0	0
ENSG00000279189	0	0	0	0	0	0	0	0	0	0
ENSG00000279188	0	1	0	0	0	0	0	0	0	0
ENSG00000279187	0	0	0	0	0	0	2	2	0	0
ENSG00000279186	1	2	1	3	1	3	1	4	0	0
ENSG00000279185	2	6	14	1	3	2	25	33	0	4
ENSG00000279184	38	37	155	27	34	83	76	74	2	11
ENSG00000279183	0	0	0	0	0	0	3	0	0	0
ENSG00000279182	0	0	0	0	0	0	0	0	0	0
ENSG00000279181	0	0	0	0	0	0	5	0	0	0
ENSG00000279180	0	0	0	0	0	0	0	3	0	0
ENSG00000279179	7	10	13	2	6	21	6	5	0	0
ENSG00000279178	0	0	0	0	0	0	0	0	0	0
ENSG00000279177	2	0	7	1	0	0	1	0	0	0

ENSG00000279176	0	0	0	0	1	1	6	6	0	0
ENSG00000279175	67	73	153	82	42	190	124	729	8	20
ENSG00000279174	0	0	0	0	1	0	0	3	0	0
ENSG00000279173	0	0	0	0	0	0	0	0	0	0
ENSG00000279172	0	0	0	0	0	0	0	0	0	0
ENSG00000279171	0	0	0	0	0	0	0	0	0	0
ENSG00000279170	0	0	0	1	1	1	0	8	0	0
ENSG00000279169	0	0	0	0	0	0	0	0	0	0
ENSG00000279168	2	3	3	1	0	0	26	257	0	0
ENSG00000279167	0	0	0	0	0	0	0	0	0	0
ENSG00000279166	0	0	0	0	0	0	20	0	1	0
ENSG00000279165	0	0	0	0	0	0	3	2	0	0
ENSG00000279164	0	1	0	1	0	7	11	6	0	0
ENSG00000279163	0	0	0	0	0	0	0	0	0	0
ENSG00000279162	4	1	0	1	2	0	1	1	0	0
ENSG00000279161	0	0	0	0	0	0	4	6	0	0
ENSG00000279160	0	0	0	0	0	0	1	8	0	0
ENSG00000279159	25	50	84	45	37	89	157	65	1	0
ENSG00000279158	0	0	0	0	0	0	0	0	0	0
ENSG00000279157	0	0	0	0	0	0	0	0	0	0
ENSG00000279156	0	0	0	0	0	0	0	0	0	0
ENSG00000279155	1	1	0	1	2	10	6	0	0	0
ENSG00000279154	0	0	2	0	0	0	2	9	0	2
ENSG00000279153	0	0	0	0	0	0	0	0	0	0
ENSG00000279152	0	0	0	0	0	0	0	0	0	0
ENSG00000279151	0	0	0	0	0	0	0	2	0	0
ENSG00000279150	0	0	0	0	0	0	0	0	0	0
ENSG00000279149	0	0	0	0	0	0	7	0	0	0
ENSG00000279148	7	1	5	1	6	13	14	3	0	0
ENSG00000279147	0	0	2	1	0	3	1	13	0	0
ENSG00000279146	0	0	0	0	0	0	0	0	0	0
ENSG00000279145	4	17	17	6	7	20	15	6	0	0
ENSG00000279144	0	0	0	1	0	0	0	0	0	0
ENSG00000279143	0	0	0	0	0	0	0	0	0	0
ENSG00000279142	0	0	0	0	0	0	0	0	0	0
ENSG00000279141	2	3	4	0	0	0	0	1	0	0
ENSG00000279140	0	1	0	0	0	0	33	9	0	0
ENSG00000279139	0	0	0	0	0	0	0	0	0	0
ENSG00000279138	2	2	6	5	2	4	5	5	0	0
ENSG00000279137	0	0	0	0	0	0	11	6	0	0
ENSG00000279136	0	0	0	0	0	1	0	0	0	0
ENSG00000279135	0	0	0	1	0	0	4	8	0	0
ENSG00000279134	0	4	4	0	1	0	4	10	0	0
ENSG00000279133	5	1	3	3	1	8	47	27	1	2
ENSG00000279132	0	0	0	0	0	0	0	0	0	0
ENSG00000279131	0	0	0	0	0	0	0	0	0	0
ENSG00000279130	0	0	0	0	1	2	5	4	0	0
ENSG00000279129	0	0	0	0	0	0	0	0	0	0
ENSG00000279128	0	0	0	0	0	0	14	0	0	0
ENSG00000279127	0	1	0	0	0	0	0	2	0	0
ENSG00000279126	46	55	69	25	38	109	23	10	0	1
ENSG00000279125	0	0	0	0	0	0	0	0	0	0
ENSG00000279124	27	38	96	26	23	98	62	0	0	0
ENSG00000279123	2	0	0	1	2	1	7	5	2	1
ENSG00000279122	0	0	0	0	0	0	0	0	0	0
ENSG00000279121	0	0	0	0	0	1	7	0	0	0
ENSG00000279120	0	0	0	0	0	0	0	1	0	0

ENSG00000279119	0	0	0	0	0	0	0	0	0	0
ENSG00000279118	6	23	32	10	10	38	103	29	0	0
ENSG00000279117	4	11	32	5	5	24	331	375	0	1
ENSG00000279116	0	0	0	0	0	0	0	0	0	0
ENSG00000279115	5	5	50	0	3	14	149	77	1	46
ENSG00000279114	1	1	2	1	0	0	0	8	5	0
ENSG00000279113	0	0	0	0	0	0	3	0	0	0
ENSG00000279111	0	0	0	0	0	0	0	0	0	0
ENSG00000279110	2	5	2	3	2	25	40	16	0	4
ENSG00000279109	1	0	0	2	0	4	8	0	0	0
ENSG00000279108	3	3	0	0	2	0	34	20	0	8
ENSG00000279107	0	2	0	0	0	0	3	21	0	0
ENSG00000279106	0	0	0	0	0	0	5	4	0	0
ENSG00000279105	0	0	0	0	0	0	0	0	0	0
ENSG00000279104	0	0	0	0	0	0	1	1	0	0
ENSG00000279103	0	4	0	0	0	0	9	4	1	0
ENSG00000279102	0	0	0	0	0	0	0	0	0	0
ENSG00000279101	0	0	0	0	0	0	0	0	0	0
ENSG00000279100	0	0	0	0	0	0	0	0	0	0
ENSG00000279099	0	0	0	0	0	0	0	0	0	0
ENSG00000279098	0	0	0	0	0	0	0	3	0	0
ENSG00000279097	0	0	0	0	0	0	0	0	0	0
ENSG00000279096	0	0	0	0	0	3	0	0	0	0
ENSG00000279095	168	165	276	161	139	504	1153	280	0	2
ENSG00000279094	16	10	25	7	13	22	7	24	0	0
ENSG00000279093	0	0	0	0	0	0	2	2	0	0
ENSG00000279092	13	18	16	13	20	60	147	12	0	0
ENSG00000279091	17	27	37	20	19	71	25	88	0	2
ENSG00000279090	0	0	0	0	0	0	0	0	0	0
ENSG00000279089	2	8	17	4	4	18	27	37	0	0
ENSG00000279088	192	171	220	141	146	425	215	311	0	12
ENSG00000279087	0	0	0	0	0	0	0	0	0	0
ENSG00000279086	0	0	1	0	0	3	64	8	0	0
ENSG00000279085	0	0	0	0	1	0	5	6	0	0
ENSG00000279084	0	0	0	0	0	0	2	0	0	0
ENSG00000279083	0	0	0	0	0	0	9	0	0	0
ENSG00000279082	0	0	0	0	0	0	0	0	0	0
ENSG00000279081	6	7	7	4	3	2	3	3	0	0
ENSG00000279080	8	10	29	3	10	9	32	371	10	27
ENSG00000279079	0	0	0	0	0	0	0	0	0	0
ENSG00000279078	2	2	0	0	0	1	15	4	0	1
ENSG00000279077	0	0	0	1	0	6	5	0	0	0
ENSG00000279076	0	0	0	0	0	0	0	0	0	0
ENSG00000279075	0	1	0	0	1	0	3	0	0	0
ENSG00000279074	0	0	0	0	0	0	0	0	0	0
ENSG00000279073	0	0	0	0	0	0	0	0	0	0
ENSG00000279072	21	33	48	11	16	69	7	11	0	0
ENSG00000279071	0	0	0	0	0	0	6	0	0	0
ENSG00000279070	0	0	0	0	0	0	1	0	0	0
ENSG00000279069	0	1	5	17	11	16	37	5	0	0
ENSG00000279068	0	0	0	0	0	0	0	0	0	0
ENSG00000279067	0	0	0	0	0	0	0	0	0	0
ENSG00000279066	4	3	9	4	9	12	1	6	0	0
ENSG00000279065	0	0	0	0	1	0	2	0	0	0
ENSG00000279064	1	1	0	1	5	6	0	0	0	0
ENSG00000279063	0	0	0	0	0	0	1	0	0	0
ENSG00000279062	0	0	0	1	1	0	0	5	0	0

ENSG00000279061	0	0	0	0	0	0	0	3	0	0
ENSG00000279060	0	0	0	0	0	0	0	0	0	0
ENSG00000279059	50	43	95	36	39	106	23	73	0	0
ENSG00000279058	36	55	114	33	30	80	46	62	0	0
ENSG00000279057	10	10	8	7	11	30	19	25	0	0
ENSG00000279056	0	0	0	0	0	0	0	0	0	0
ENSG00000279055	0	0	0	0	0	0	0	0	0	0
ENSG00000279054	0	0	0	1	0	0	0	0	0	0
ENSG00000279053	0	0	0	0	0	0	0	0	0	0
ENSG00000279052	0	0	0	0	0	0	0	0	0	0
ENSG00000279051	0	0	0	0	0	0	0	0	0	0
ENSG00000279050	2	0	2	1	2	0	3	3	0	0
ENSG00000279049	0	1	9	3	10	11	0	15	0	0
ENSG00000279048	0	0	2	0	0	3	3	15	0	0
ENSG00000279047	0	0	0	0	0	0	0	5	0	0
ENSG00000279046	0	0	0	0	0	0	0	0	0	0
ENSG00000279045	0	0	0	0	0	0	0	0	0	0
ENSG00000279044	0	0	4	0	0	1	0	0	0	0
ENSG00000279043	0	0	5	0	0	3	0	2	0	0
ENSG00000279042	0	0	0	0	0	0	0	0	0	0
ENSG00000279041	21	25	46	18	27	79	55	3	0	0
ENSG00000279040	0	0	0	0	0	0	4	0	0	0
ENSG00000279039	0	1	0	0	0	0	0	0	0	0
ENSG00000279038	1	2	4	7	7	14	55	0	0	0
ENSG00000279037	0	0	0	0	0	0	0	0	0	0
ENSG00000279036	0	0	3	0	0	1	3	0	0	0
ENSG00000279035	0	0	4	0	0	1	0	0	0	0
ENSG00000279034	0	1	0	0	0	0	0	0	0	0
ENSG00000279033	0	0	0	0	0	0	0	0	0	0
ENSG00000279032	0	0	0	0	0	0	0	6	0	0
ENSG00000279031	0	0	0	1	1	1	15	14	0	0
ENSG00000279030	0	0	0	1	0	0	11	0	0	0
ENSG00000279029	0	0	0	0	0	1	28	0	0	0
ENSG00000279028	0	0	0	0	0	0	0	0	0	0
ENSG00000279027	0	0	0	0	0	0	0	0	0	0
ENSG00000279026	1	1	1	1	0	0	32	31	0	0
ENSG00000279025	0	0	0	0	0	1	4	0	0	0
ENSG00000279024	0	0	0	0	0	0	8	0	0	0
ENSG00000279023	0	0	0	0	0	0	0	0	0	0
ENSG00000279022	1	3	3	0	0	2	1	2	0	0
ENSG00000279021	0	0	0	0	0	0	47	0	0	0
ENSG00000279020	0	0	0	0	0	0	2	4	0	0
ENSG00000279019	2	4	3	2	1	10	7	2	0	0
ENSG00000279018	0	0	0	0	0	0	0	0	0	0
ENSG00000279017	0	0	1	0	0	0	4	2	0	0
ENSG00000279016	0	0	0	0	0	0	7	0	0	0
ENSG00000279015	0	0	0	0	0	0	0	0	0	0
ENSG00000279014	0	0	0	0	0	0	0	0	0	0
ENSG00000279013	0	0	0	0	0	0	0	5	0	0
ENSG00000279012	0	0	0	0	0	0	0	0	0	0
ENSG00000279011	0	0	0	0	0	0	0	0	0	0
ENSG00000279010	269	242	424	220	290	739	89	74	5	9
ENSG00000279009	0	0	0	0	0	0	0	0	0	0
ENSG00000279008	0	0	0	0	0	0	0	0	0	0
ENSG00000279007	25	34	54	17	18	68	124	53	0	1
ENSG00000279006	0	0	0	0	0	0	9	0	0	0
ENSG00000279005	0	0	0	0	0	0	0	0	0	0

ENSG00000279004	0	0	0	0	0	0	0	0	0	0
ENSG00000279003	0	0	1	0	0	1	0	4	0	4
ENSG00000279002	0	0	0	0	0	0	0	0	0	0
ENSG00000279001	0	0	0	0	0	0	0	0	0	0
ENSG00000279000	0	0	0	0	0	0	0	0	0	0
ENSG00000278999	1	0	2	0	0	0	0	3	0	0
ENSG00000278998	0	1	2	0	1	3	0	0	0	0
ENSG00000278997	6	6	5	6	2	15	26	12	0	0
ENSG00000278996	71	56	76	63	45	117	187	2076	2	9
ENSG00000278995	0	0	4	0	1	0	0	0	0	0
ENSG00000278994	0	0	0	0	0	0	0	9	0	0
ENSG00000278993	3	2	8	9	4	10	45	5	0	0
ENSG00000278992	0	0	0	0	0	0	0	0	0	0
ENSG00000278991	2	1	4	0	3	4	18	4	0	0
ENSG00000278990	0	0	0	0	0	0	0	0	0	0
ENSG00000278989	2	3	9	2	4	3	31	38	0	0
ENSG00000278988	0	0	0	0	0	0	0	0	0	0
ENSG00000278987	1	5	6	10	11	16	8	4	0	0
ENSG00000278986	2	3	3	0	0	6	2	0	0	0
ENSG00000278985	1	1	2	0	0	2	7	26	0	0
ENSG00000278984	0	0	0	0	0	0	0	0	0	0
ENSG00000278983	1	0	0	1	1	4	57	18	0	0
ENSG00000278982	0	0	0	0	0	0	2	0	0	0
ENSG00000278981	0	0	0	0	0	0	0	0	0	0
ENSG00000278980	0	0	0	0	0	0	0	0	0	0
ENSG00000278979	0	0	0	0	0	0	0	4	0	0
ENSG00000278978	96	90	112	67	94	234	160	11	0	0
ENSG00000278977	0	0	0	0	0	0	0	0	0	0
ENSG00000278976	0	0	0	0	0	0	39	0	0	0
ENSG00000278975	0	0	0	0	0	0	0	0	0	0
ENSG00000278974	101	118	181	97	82	368	610	322	1	14
ENSG00000278973	0	0	0	0	0	4	0	0	0	0
ENSG00000278972	0	0	0	0	0	0	0	0	0	0
ENSG00000278971	0	0	0	0	0	0	0	0	0	0
ENSG00000278970	43	53	68	35	28	101	494	466	1	8
ENSG00000278969	0	2	0	0	0	0	6	6	0	0
ENSG00000278968	0	0	0	0	0	0	0	0	0	0
ENSG00000278967	0	0	0	0	0	0	3	0	0	0
ENSG00000278966	21	25	32	14	18	47	9	5	0	1
ENSG00000278965	0	1	0	0	0	0	6	8	0	0
ENSG00000278964	0	0	0	0	0	0	0	0	0	0
ENSG00000278963	0	0	0	0	2	0	0	4	0	0
ENSG00000278962	0	0	1	0	0	1	28	7	0	0
ENSG00000278961	0	0	0	1	0	0	7	7	0	0
ENSG00000278960	0	0	0	0	0	0	0	0	0	0
ENSG00000278959	3	5	3	3	0	2	65	2	0	0
ENSG00000278958	0	0	0	0	0	0	0	0	0	0
ENSG00000278957	0	0	0	0	0	0	0	0	0	0
ENSG00000278956	1	0	4	1	0	1	0	0	0	0
ENSG00000278955	0	0	0	0	0	0	0	0	0	0
ENSG00000278954	4	2	8	6	4	4	7	0	0	0
ENSG00000278953	0	0	0	0	0	0	0	1	0	0
ENSG00000278952	4	0	13	3	2	10	4	0	0	0
ENSG00000278951	0	0	0	0	0	0	0	0	0	0
ENSG00000278950	5	2	3	1	1	4	1	6	0	0
ENSG00000278949	8	9	15	3	5	14	6	14	0	0
ENSG00000278948	117	92	186	103	86	302	8	0	0	0

ENSG00000278947	0	0	3	0	0	0	0	0	0	0
ENSG00000278946	0	0	0	0	0	0	0	0	0	0
ENSG00000278945	0	0	0	0	0	0	0	0	0	0
ENSG00000278944	0	0	0	0	0	0	0	0	0	0
ENSG00000278943	9	3	10	1	2	16	40	31	2	14
ENSG00000278942	0	0	0	0	0	0	0	0	0	0
ENSG00000278941	0	0	0	0	0	0	0	0	0	0
ENSG00000278940	0	0	0	0	0	0	0	0	0	0
ENSG00000278939	0	1	0	2	0	0	1	0	0	0
ENSG00000278938	37	47	58	21	37	70	10	8	1	2
ENSG00000278937	0	0	0	0	0	0	0	3	0	0
ENSG00000278936	0	0	0	0	0	0	0	0	0	0
ENSG00000278935	1	1	3	3	1	7	3	2	0	0
ENSG00000278934	0	0	0	0	0	0	4	7	0	0
ENSG00000278933	0	0	0	0	0	0	0	0	0	0
ENSG00000278932	12	22	29	15	10	28	17	79	0	0
ENSG00000278931	6	3	3	1	3	10	15	140	0	0
ENSG00000278930	0	0	1	0	0	0	26	4	1	9
ENSG00000278929	0	0	0	0	0	0	0	1	0	0
ENSG00000278928	1	0	1	0	1	1	0	2	0	0
ENSG00000278927	1	0	0	0	1	2	1	0	0	0
ENSG00000278926	0	0	0	0	0	0	0	5	0	0
ENSG00000278925	4	2	3	1	5	7	18	15	0	0
ENSG00000278924	0	0	0	0	0	0	0	0	0	0
ENSG00000278923	0	0	0	0	0	0	0	0	0	0
ENSG00000278922	3	6	1	6	4	9	4	9	0	0
ENSG00000278921	10	14	16	8	8	6	14	31	0	0
ENSG00000278920	0	0	4	3	2	4	2	23	6	3
ENSG00000278919	0	0	1	0	0	3	29	2	0	0
ENSG00000278918	6	2	7	2	6	2	40	25	0	0
ENSG00000278917	5	6	4	5	3	14	5	60	0	3
ENSG00000278916	3	7	3	1	0	5	9	4	0	0
ENSG00000278915	0	0	0	0	0	0	0	0	0	0
ENSG00000278914	1	0	0	0	0	0	0	0	0	0
ENSG00000278913	0	0	0	0	0	0	0	0	0	0
ENSG00000278912	0	0	0	0	0	4	0	0	0	0
ENSG00000278911	0	0	0	0	0	0	0	0	0	0
ENSG00000278910	0	0	0	0	0	0	15	20	0	0
ENSG00000278909	0	0	0	0	0	0	8	5	0	0
ENSG00000278908	0	0	0	0	0	0	0	0	0	0
ENSG00000278907	0	0	0	0	0	0	0	0	0	0
ENSG00000278906	0	0	0	0	0	0	0	0	0	0
ENSG00000278905	0	0	0	0	0	0	1	0	0	0
ENSG00000278904	0	0	0	0	0	0	0	0	0	0
ENSG00000278903	16	6	25	8	12	28	27	31	0	0
ENSG00000278902	0	0	0	0	0	0	0	0	0	0
ENSG00000278901	0	0	0	0	0	0	0	0	0	0
ENSG00000278900	0	0	0	0	0	0	0	0	0	0
ENSG00000278899	1	3	4	2	1	2	0	12	0	0
ENSG00000278898	0	0	1	0	0	0	0	0	0	0
ENSG00000278897	1	1	1	3	5	0	3	1	3	0
ENSG00000278896	0	2	1	0	2	0	3	0	0	0
ENSG00000278895	3	9	16	1	3	10	31	9	0	1
ENSG00000278894	0	0	0	0	1	0	0	0	0	0
ENSG00000278893	0	0	0	0	1	0	3	4	0	0
ENSG00000278892	0	0	0	0	0	0	1	0	0	0
ENSG00000278891	0	0	0	2	1	0	2	2	0	2

ENSG00000278890	0	0	0	0	0	0	0	6	0	5
ENSG00000278889	0	0	0	0	0	0	0	0	0	0
ENSG00000278888	1	1	4	1	1	0	0	0	0	0
ENSG00000278887	0	0	0	0	0	0	0	0	0	0
ENSG00000278886	0	0	0	0	1	0	2	1	0	0
ENSG00000278885	0	0	0	0	0	0	0	0	0	0
ENSG00000278884	0	0	2	0	1	7	0	0	0	0
ENSG00000278883	0	0	0	0	0	0	0	0	0	0
ENSG00000278882	14	26	37	16	9	47	117	67	0	1
ENSG00000278881	1	3	1	0	3	4	12	2	0	0
ENSG00000278880	0	0	0	0	0	0	0	0	0	0
ENSG00000278879	2	0	0	1	0	1	12	5	0	0
ENSG00000278878	0	0	0	0	0	0	0	0	0	0
ENSG00000278877	0	0	0	0	0	0	0	1	0	0
ENSG00000278876	1	0	1	3	4	0	4	0	0	0
ENSG00000278875	2	3	0	0	4	2	4	0	0	0
ENSG00000278874	0	0	0	0	0	0	0	0	0	0
ENSG00000278873	0	0	0	1	0	0	4	3	0	0
ENSG00000278872	0	0	0	0	0	0	0	0	0	0
ENSG00000278871	0	0	0	0	0	0	0	0	0	0
ENSG00000278870	0	0	0	0	0	0	0	0	0	0
ENSG00000278869	2	0	6	1	1	7	33	5	0	0
ENSG00000278868	0	0	0	0	0	0	0	0	0	0
ENSG00000278867	43	33	58	32	29	86	76	78	0	4
ENSG00000278866	0	0	0	0	0	0	0	0	0	0
ENSG00000278865	0	0	0	0	0	0	0	0	0	0
ENSG00000278864	67	94	153	97	87	291	100	22	0	0
ENSG00000278863	0	0	0	0	0	0	7	0	0	0
ENSG00000278862	0	0	0	0	0	0	0	5	0	0
ENSG00000278861	0	3	5	0	1	1	0	0	0	0
ENSG00000278860	0	0	0	0	0	0	0	0	0	0
ENSG00000278859	1	0	0	0	0	0	0	0	0	0
ENSG00000278857	0	0	0	0	0	0	29	81	0	0
ENSG00000278854	0	0	0	0	0	0	0	0	0	0
ENSG00000278852	0	0	0	0	0	0	0	0	0	0
ENSG00000278851	0	0	0	0	0	0	0	0	0	0
ENSG00000278849	0	0	0	0	0	0	0	7	0	0
ENSG00000278848	3	6	3	4	3	14	1	4	0	0
ENSG00000278847	0	0	0	0	0	0	1	1	0	0
ENSG00000278845	748	548	917	523	585	1568	539	544	2	16
ENSG00000278844	0	0	0	0	0	0	0	0	0	0
ENSG00000278842	0	0	2	0	0	4	0	0	0	0
ENSG00000278841	0	0	0	0	0	0	2	0	0	0
ENSG00000278840	0	0	0	0	0	0	0	0	0	0
ENSG00000278838	0	0	0	0	0	0	0	0	0	0
ENSG00000278837	0	0	0	0	0	0	0	0	0	0
ENSG00000278834	9	7	12	10	13	24	6	43	0	0
ENSG00000278832	0	0	0	0	0	0	2	0	0	0
ENSG00000278831	0	1	4	0	2	0	5	2	0	0
ENSG00000278830	0	0	0	0	0	0	0	0	0	0
ENSG00000278829	0	0	0	0	0	0	14	0	0	0
ENSG00000278828	10	18	37	20	13	33	32	12	0	5
ENSG00000278825	0	0	0	0	0	0	0	0	0	0
ENSG00000278824	0	0	0	0	0	0	0	0	0	0
ENSG00000278822	0	0	0	0	0	0	0	0	0	0
ENSG00000278820	0	0	0	0	0	0	0	0	0	0
ENSG00000278819	0	0	0	0	0	0	1	0	0	0

ENSG00000278818	1	0	0	0	0	0	0	0	0	0
ENSG00000278816	0	0	4	0	1	0	3	0	0	0
ENSG00000278815	0	0	0	0	0	0	0	0	0	0
ENSG00000278814	0	0	0	0	0	0	0	0	0	0
ENSG00000278813	0	0	0	0	0	0	0	0	0	0
ENSG00000278811	1	2	1	0	0	1	6	0	0	0
ENSG00000278810	0	0	0	0	0	0	0	8	0	0
ENSG00000278807	31	35	30	43	27	118	11	15	0	0
ENSG00000278804	0	0	0	0	0	0	0	0	0	0
ENSG00000278803	0	0	0	0	0	0	0	0	0	0
ENSG00000278802	0	0	0	0	0	0	0	0	0	0
ENSG00000278800	0	0	0	0	0	0	0	2	0	0
ENSG00000278799	0	0	0	0	0	0	0	0	0	0
ENSG00000278798	0	0	0	0	0	0	0	0	0	0
ENSG00000278797	0	0	0	0	0	0	0	0	0	0
ENSG00000278794	0	1	0	0	0	0	2	1	0	0
ENSG00000278792	0	0	0	0	0	0	0	0	0	0
ENSG00000278791	0	0	0	0	0	0	0	0	0	0
ENSG00000278790	0	0	0	0	0	1	0	0	0	0
ENSG00000278785	1	1	1	0	1	0	0	0	0	0
ENSG00000278784	34	35	56	27	31	94	66	72	0	0
ENSG00000278783	0	0	0	0	0	0	1	0	0	0
ENSG00000278778	0	0	0	0	0	0	0	0	0	0
ENSG00000278775	0	0	0	0	0	0	18	2	0	0
ENSG00000278774	0	1	1	4	1	11	243	31	21	290
ENSG00000278773	0	0	0	0	0	0	0	0	0	0
ENSG00000278772	0	0	0	0	0	3	2	0	0	0
ENSG00000278771	0	0	0	0	0	0	0	0	0	0
ENSG00000278770	0	0	0	2	1	1	4	0	0	0
ENSG00000278769	0	0	0	0	0	0	0	0	0	0
ENSG00000278768	17	13	21	11	12	71	20	34	2	0
ENSG00000278766	15	14	28	24	19	45	6	2	0	0
ENSG00000278765	7	11	22	6	5	20	16	12	0	0
ENSG00000278764	0	0	0	0	0	0	0	0	0	0
ENSG00000278763	3	6	14	9	4	13	2	4	0	0
ENSG00000278761	0	0	0	0	0	1	0	0	0	0
ENSG00000278757	0	0	3	0	0	0	0	9	0	0
ENSG00000278756	0	0	0	0	0	0	0	0	0	0
ENSG00000278754	0	0	0	0	0	0	0	0	0	0
ENSG00000278753	0	0	0	0	0	0	0	0	0	0
ENSG00000278752	0	0	0	0	0	0	0	0	0	0
ENSG00000278751	0	0	0	0	0	0	0	0	0	0
ENSG00000278750	0	0	0	0	0	0	0	0	0	0
ENSG00000278748	0	0	0	0	0	0	0	0	0	0
ENSG00000278746	0	0	0	0	0	0	0	3	0	0
ENSG00000278745	0	0	0	0	0	0	0	0	0	0
ENSG00000278744	0	0	0	0	0	3	1	0	0	0
ENSG00000278743	1	0	0	1	0	2	23	26	0	0
ENSG00000278740	0	0	0	0	0	2	23	6	0	0
ENSG00000278739	0	0	0	0	0	0	0	0	0	0
ENSG00000278737	3	0	1	1	1	1	3	12	0	0
ENSG00000278736	0	0	0	0	0	0	0	0	0	0
ENSG00000278735	0	2	0	0	0	1	1	0	0	0
ENSG00000278734	0	0	0	0	0	0	0	0	0	0
ENSG00000278733	0	0	0	0	2	0	1	1	0	0
ENSG00000278732	0	0	0	0	0	0	0	8	0	0
ENSG00000278730	83	76	186	74	76	303	424	397	0	0

ENSG00000278727	14	7	16	9	3	31	5	25	0	0
ENSG00000278725	0	4	2	1	1	1	0	1	0	0
ENSG00000278724	0	0	0	0	0	0	0	0	0	0
ENSG00000278722	0	0	0	0	0	0	0	0	0	0
ENSG00000278720	0	0	0	0	0	0	1	0	0	0
ENSG00000278719	0	0	0	1	0	0	2	0	0	0
ENSG00000278717	0	0	0	0	0	0	0	0	0	0
ENSG00000278716	2	3	2	3	0	2	0	5	0	0
ENSG00000278715	0	0	0	0	0	0	0	0	0	0
ENSG00000278713	0	0	0	0	0	0	0	0	0	0
ENSG00000278709	45	35	56	44	48	167	54	7	0	0
ENSG00000278708	0	0	0	0	0	0	0	0	0	0
ENSG00000278705	1	0	0	1	0	0	11	0	0	0
ENSG00000278703	0	0	3	1	0	0	9	17	0	0
ENSG00000278702	0	0	0	0	0	0	0	1	1	0
ENSG00000278701	0	0	0	0	0	0	0	3	0	0
ENSG00000278700	1	0	5	0	0	1	2	8	0	0
ENSG00000278698	0	0	0	0	0	0	0	0	0	0
ENSG00000278696	0	0	0	0	0	0	0	0	0	0
ENSG00000278694	0	0	0	0	0	0	0	0	0	0
ENSG00000278690	0	0	0	0	0	0	0	0	0	0
ENSG00000278687	0	0	0	0	0	0	0	0	0	0
ENSG00000278686	0	0	0	0	0	0	0	0	0	0
ENSG00000278685	0	0	0	0	0	0	1	3	0	0
ENSG00000278684	0	0	0	0	0	0	0	0	0	0
ENSG00000278683	0	1	0	0	0	0	0	0	0	0
ENSG00000278681	0	0	0	0	0	0	0	0	0	0
ENSG00000278679	2	2	5	1	1	2	1	0	0	2
ENSG00000278678	0	0	0	0	0	0	0	0	0	0
ENSG00000278677	0	0	0	0	0	0	0	0	0	0
ENSG00000278675	0	0	0	0	0	0	0	0	0	0
ENSG00000278674	0	3	0	0	0	0	3	0	0	0
ENSG00000278672	0	0	0	0	0	0	0	0	0	0
ENSG00000278671	0	0	0	0	0	0	0	0	0	0
ENSG00000278669	0	0	0	0	0	0	0	0	0	0
ENSG00000278668	5	9	15	6	5	22	29	0	0	0
ENSG00000278667	0	0	0	0	0	0	0	0	0	0
ENSG00000278665	1	2	2	2	1	1	0	6	0	0
ENSG00000278664	0	0	0	0	0	1	0	0	0	0
ENSG00000278663	0	0	0	0	0	0	0	0	0	0
ENSG00000278662	120	179	288	102	98	311	127	162	0	0
ENSG00000278661	0	0	0	0	0	0	0	0	0	0
ENSG00000278660	0	0	0	0	0	0	0	0	0	0
ENSG00000278658	0	0	0	0	0	0	0	0	0	0
ENSG00000278657	0	0	0	0	0	0	0	0	0	0
ENSG00000278655	0	0	0	0	0	0	1	0	0	0
ENSG00000278654	0	0	0	0	0	0	0	0	0	0
ENSG00000278652	0	0	0	0	0	0	0	0	0	0
ENSG00000278651	0	0	0	0	0	0	0	0	0	0
ENSG00000278650	0	0	0	0	0	0	0	0	0	0
ENSG00000278648	0	0	0	0	0	0	0	0	0	0
ENSG00000278647	0	0	0	0	0	0	0	0	0	0
ENSG00000278646	0	0	0	0	0	0	0	0	0	0
ENSG00000278645	0	0	0	0	0	0	0	0	0	0
ENSG00000278643	0	0	0	0	0	0	0	7	0	0
ENSG00000278642	2	3	0	0	0	0	3	0	0	0
ENSG00000278641	0	0	0	0	0	1	0	0	0	0

ENSG00000278640	0	0	0	0	0	0	0	0	0	0
ENSG00000278639	0	0	0	0	0	2	2	0	0	0
ENSG00000278638	1	0	0	0	0	1	1	0	0	0
ENSG00000278637	0	0	0	0	0	0	4	0	0	0
ENSG00000278636	0	0	0	0	0	0	0	0	0	0
ENSG00000278635	1	1	2	0	1	5	4	0	0	0
ENSG00000278631	0	0	0	0	0	0	0	0	0	0
ENSG00000278630	0	0	0	0	0	0	0	0	0	0
ENSG00000278629	0	0	0	0	0	0	0	0	0	0
ENSG00000278628	0	0	0	0	0	0	0	0	0	0
ENSG00000278627	2	5	2	2	7	10	0	1	0	0
ENSG00000278626	0	2	2	0	0	0	0	1	0	0
ENSG00000278621	0	0	0	0	0	0	0	0	0	0
ENSG00000278619	84	48	76	62	54	137	151	84	0	0
ENSG00000278618	0	0	0	0	0	0	0	0	0	0
ENSG00000278617	0	0	0	0	0	0	0	0	0	0
ENSG00000278616	6	6	7	8	9	30	16	0	0	0
ENSG00000278615	0	0	0	0	0	0	2	2	0	0
ENSG00000278612	0	0	0	0	0	0	0	0	0	0
ENSG00000278611	42	31	69	26	33	102	5	46	0	0
ENSG00000278610	0	0	0	0	0	0	0	0	0	0
ENSG00000278609	0	0	0	0	0	0	0	0	0	0
ENSG00000278607	1	0	0	0	0	0	12	22	0	0
ENSG00000278604	0	0	0	0	0	0	0	0	0	0
ENSG00000278603	0	0	0	0	0	0	0	0	0	0
ENSG00000278602	0	0	0	0	0	0	0	0	0	0
ENSG00000278601	0	0	1	0	0	0	4	2	0	0
ENSG00000278600	37	45	45	46	48	103	179	54	1	0
ENSG00000278599	207	218	328	238	204	579	44	26	0	0
ENSG00000278598	0	1	0	0	0	0	0	0	0	0
ENSG00000278597	5	10	21	9	4	8	112781	2678	129	1302
ENSG00000278596	0	0	2	0	0	0	0	3	0	0
ENSG00000278595	3	0	0	0	0	0	1	0	0	0
ENSG00000278594	1	0	0	0	0	0	2	12	0	0
ENSG00000278593	0	0	0	0	0	0	0	4	0	0
ENSG00000278592	0	0	0	0	0	0	0	0	0	0
ENSG00000278591	0	1	5	6	1	11	208	18	28	272
ENSG00000278590	0	1	3	1	1	6	0	0	0	0
ENSG00000278589	0	0	0	0	0	0	0	0	0	0
ENSG00000278588	0	0	0	1	0	3	4	0	0	0
ENSG00000278587	0	0	0	0	0	0	0	0	0	0
ENSG00000278586	0	0	0	0	0	0	0	0	0	0
ENSG00000278585	0	0	0	0	0	0	0	0	0	0
ENSG00000278584	0	0	0	0	0	0	0	0	0	0
ENSG00000278582	0	0	0	0	0	0	0	0	0	0
ENSG00000278581	0	0	0	0	0	0	0	0	0	0
ENSG00000278580	0	0	0	0	0	0	0	0	0	0
ENSG00000278579	0	0	0	0	0	0	0	0	0	0
ENSG00000278578	0	0	0	0	0	0	0	0	0	0
ENSG00000278577	0	0	0	0	0	0	0	2	0	0
ENSG00000278576	1	5	7	3	1	1	21	9	0	0
ENSG00000278575	0	0	0	0	0	0	0	0	0	0
ENSG00000278572	1	3	0	1	0	1	6	2	0	0
ENSG00000278571	0	0	0	0	0	0	0	0	0	0
ENSG00000278570	3	5	4	4	1	13	24	10	0	0
ENSG00000278569	0	0	0	0	0	0	0	0	0	0
ENSG00000278568	0	0	0	0	0	0	0	0	0	0

ENSG00000278566	0	0	0	0	0	0	1	0	0	0
ENSG00000278564	0	0	0	0	0	0	0	0	0	0
ENSG00000278561	1	1	0	1	0	5	0	0	0	0
ENSG00000278560	0	0	0	0	0	0	0	0	0	0
ENSG00000278558	11	15	16	17	10	35	19	64	0	0
ENSG00000278557	1	0	1	2	0	2	2	41	0	0
ENSG00000278556	0	0	0	0	0	0	0	0	0	0
ENSG00000278554	0	0	0	0	0	0	0	0	0	0
ENSG00000278552	0	0	0	0	0	0	0	0	0	0
ENSG00000278551	0	0	0	0	0	0	0	0	0	0
ENSG00000278549	0	0	0	0	1	0	0	0	0	0
ENSG00000278547	0	0	0	0	0	0	0	0	0	0
ENSG00000278546	0	0	0	0	0	0	34	0	0	0
ENSG00000278544	0	0	0	0	0	0	0	0	0	0
ENSG00000278543	0	0	0	0	2	0	0	1	0	0
ENSG00000278542	0	0	0	0	0	0	1	0	0	0
ENSG00000278541	0	0	0	0	0	0	0	0	0	0
ENSG00000278540	3125	2703	4348	2658	2902	8715	1015	471	0	14
ENSG00000278537	0	0	0	0	0	0	0	0	0	0
ENSG00000278536	0	0	0	0	0	0	0	0	0	0
ENSG00000278535	62	73	111	52	49	206	165	217	0	0
ENSG00000278534	0	0	0	0	0	0	0	0	0	0
ENSG00000278532	0	0	0	0	0	0	1	2	0	0
ENSG00000278531	0	0	0	0	0	0	0	0	0	0
ENSG00000278530	11	7	10	3	11	7	7	0	0	0
ENSG00000278529	0	0	0	0	0	0	0	0	0	0
ENSG00000278528	0	0	0	0	0	0	0	1	0	0
ENSG00000278527	0	0	0	0	0	0	0	0	0	0
ENSG00000278526	0	0	0	0	0	0	0	0	0	0
ENSG00000278525	0	0	0	0	0	0	0	2	0	0
ENSG00000278524	0	0	0	0	0	0	0	0	0	0
ENSG00000278523	0	0	0	0	0	0	1	0	0	0
ENSG00000278522	0	0	0	0	0	0	0	0	0	0
ENSG00000278521	0	0	0	0	0	0	0	0	0	0
ENSG00000278520	1	1	0	0	0	0	0	1	0	0
ENSG00000278518	0	0	0	1	2	0	0	0	0	0
ENSG00000278517	0	0	2	0	1	0	0	0	0	0
ENSG00000278514	2	1	1	2	1	5	3	0	0	0
ENSG00000278513	0	0	0	0	0	0	1	2	0	0
ENSG00000278512	0	0	0	0	0	0	0	0	0	0
ENSG00000278509	0	0	0	0	0	0	0	3	0	0
ENSG00000278507	0	0	0	0	0	0	0	0	0	0
ENSG00000278506	0	0	0	0	0	0	0	0	0	0
ENSG00000278505	0	0	0	0	0	0	0	4	0	0
ENSG00000278504	0	1	0	1	0	4	4	0	0	0
ENSG00000278502	0	0	0	0	0	0	0	0	0	0
ENSG00000278500	0	0	0	0	0	0	0	0	0	0
ENSG00000278499	0	0	0	0	0	0	0	0	0	0
ENSG00000278498	0	0	0	0	0	0	0	0	0	0
ENSG00000278497	0	0	0	0	0	0	0	0	0	0
ENSG00000278496	0	0	0	0	0	0	1	0	0	0
ENSG00000278493	5	5	1	5	3	9	23	6	0	0
ENSG00000278492	0	0	0	0	0	0	2	1	0	0
ENSG00000278491	0	0	0	0	0	0	0	0	0	0
ENSG00000278487	0	0	0	0	0	0	0	0	0	0
ENSG00000278486	0	0	2	1	0	0	0	0	0	0
ENSG00000278485	0	0	0	0	0	0	0	0	0	0

ENSG00000278484	0	0	0	0	0	0	0	0	0	0
ENSG00000278483	0	0	0	0	0	0	0	1	0	0
ENSG00000278482	0	0	0	0	0	0	0	0	0	0
ENSG00000278480	0	0	0	1	1	3	1	0	0	0
ENSG00000278479	0	0	0	0	0	0	0	0	0	0
ENSG00000278478	0	0	0	0	0	0	0	0	0	0
ENSG00000278477	0	0	0	0	0	0	0	0	0	0
ENSG00000278475	0	0	0	0	0	0	3	0	0	0
ENSG00000278473	0	0	0	0	0	0	0	3	0	0
ENSG00000278472	0	0	0	1	0	0	1	15	0	0
ENSG00000278471	0	0	0	0	0	0	0	0	0	0
ENSG00000278469	0	0	0	0	0	0	0	0	0	0
ENSG00000278467	7	6	1	2	2	6	3	5	0	0
ENSG00000278465	0	0	0	0	0	0	0	0	0	0
ENSG00000278464	0	0	0	0	0	0	22	4	0	0
ENSG00000278463	0	0	2	0	0	1	1	0	0	0
ENSG00000278462	0	0	0	0	0	0	3	6	0	9
ENSG00000278460	0	0	0	0	0	0	0	0	0	0
ENSG00000278459	0	0	0	0	0	0	0	0	0	0
ENSG00000278456	14	24	33	19	15	53	0	0	0	0
ENSG00000278455	4	13	30	4	8	23	5	0	0	0
ENSG00000278454	0	0	0	0	0	0	0	0	0	0
ENSG00000278453	0	0	0	0	0	0	0	1	0	0
ENSG00000278452	0	0	0	0	0	0	0	0	0	0
ENSG00000278451	0	0	0	0	0	0	0	0	0	0
ENSG00000278449	0	0	0	0	0	0	0	0	0	0
ENSG00000278448	0	0	0	0	0	0	0	0	0	0
ENSG00000278447	0	0	0	0	0	0	1	0	0	0
ENSG00000278446	0	0	0	0	0	0	0	0	0	0
ENSG00000278445	1	0	0	1	4	6	0	0	0	0
ENSG00000278441	0	0	0	0	0	0	0	0	0	0
ENSG00000278439	0	0	0	1	1	0	0	0	0	0
ENSG00000278438	0	0	0	0	0	0	0	0	0	0
ENSG00000278435	0	0	0	0	0	0	0	0	0	0
ENSG00000278434	2	0	7	0	0	0	7	3	0	0
ENSG00000278433	0	0	0	0	0	0	0	0	0	0
ENSG00000278431	0	0	0	0	0	0	0	0	0	0
ENSG00000278428	0	0	0	0	0	0	0	0	0	0
ENSG00000278424	0	0	0	0	0	0	0	0	0	0
ENSG00000278423	0	0	0	0	0	0	0	0	0	0
ENSG00000278422	1	1	1	0	1	0	0	0	0	0
ENSG00000278421	0	0	0	0	0	0	0	0	0	0
ENSG00000278420	0	1	3	0	0	1	1	3	0	0
ENSG00000278419	0	0	0	0	0	0	0	0	0	0
ENSG00000278418	0	1	0	1	1	0	0	0	0	0
ENSG00000278416	31	32	62	40	44	101	18	15	2	0
ENSG00000278413	0	0	0	0	0	0	0	0	0	0
ENSG00000278412	0	0	0	0	0	0	1	0	0	0
ENSG00000278410	0	0	0	0	0	0	0	0	0	0
ENSG00000278408	0	0	0	0	0	0	2	0	0	0
ENSG00000278406	0	0	0	0	0	0	0	0	0	0
ENSG00000278405	0	0	0	0	0	0	0	0	0	0
ENSG00000278404	0	0	0	0	0	0	0	0	0	0
ENSG00000278400	0	0	0	0	0	0	0	0	0	0
ENSG00000278399	0	0	2	0	0	4	2	0	0	0
ENSG00000278398	0	0	0	0	0	0	0	0	0	0
ENSG00000278396	2	2	3	6	3	7	3	2	0	0

ENSG00000278395	0	0	0	0	0	0	0	21	0	0
ENSG00000278393	0	0	0	0	0	0	0	0	0	0
ENSG00000278392	0	0	0	0	0	0	0	0	0	0
ENSG00000278391	0	0	0	0	0	0	0	0	0	0
ENSG00000278390	11	10	9	6	6	30	0	13	0	0
ENSG00000278389	0	0	0	1	0	0	4	0	0	0
ENSG00000278388	0	0	0	0	0	0	0	2	0	0
ENSG00000278385	0	1	3	0	3	3	1	2	0	0
ENSG00000278383	0	0	0	0	1	0	3	0	0	0
ENSG00000278382	0	0	0	0	0	0	0	0	0	0
ENSG00000278381	0	0	0	0	0	0	0	0	0	0
ENSG00000278380	0	0	0	0	0	0	0	0	0	0
ENSG00000278376	13	12	23	8	15	20	39	84	0	0
ENSG00000278375	0	0	0	1	0	0	0	3	0	0
ENSG00000278374	3	1	4	1	5	10	2	0	0	0
ENSG00000278371	0	0	0	0	0	0	0	0	0	0
ENSG00000278370	1	0	0	0	1	0	4	0	0	0
ENSG00000278367	0	0	0	0	0	0	0	4	0	0
ENSG00000278366	0	0	0	0	0	0	0	0	0	0
ENSG00000278359	0	0	0	0	0	0	0	0	0	0
ENSG00000278358	0	0	0	0	0	0	0	0	0	0
ENSG00000278356	0	0	0	0	0	0	0	0	0	0
ENSG00000278353	0	0	0	0	0	0	0	0	0	0
ENSG00000278352	0	0	0	0	0	0	1	0	0	0
ENSG00000278351	0	0	0	0	0	0	4	0	0	0
ENSG00000278350	0	0	0	0	0	0	2	0	0	0
ENSG00000278349	0	0	0	0	0	0	0	0	0	0
ENSG00000278348	0	0	0	0	0	0	0	0	0	0
ENSG00000278346	0	0	0	0	0	0	0	0	0	0
ENSG00000278344	1	1	1	5	0	8	0	0	0	0
ENSG00000278343	0	0	0	0	0	0	0	0	0	0
ENSG00000278342	0	0	0	0	0	0	0	0	0	0
ENSG00000278341	20	4	12	11	6	31	19	2	0	0
ENSG00000278340	0	0	0	0	0	0	1	0	0	0
ENSG00000278338	3	7	4	5	1	13	0	7	0	0
ENSG00000278337	0	0	0	0	0	0	0	0	0	0
ENSG00000278335	0	0	0	0	0	0	0	0	0	0
ENSG00000278334	0	0	0	0	0	0	0	0	0	0
ENSG00000278333	0	0	0	0	0	0	0	0	0	0
ENSG00000278332	0	0	0	0	0	0	0	0	0	0
ENSG00000278331	0	0	0	0	0	0	0	0	0	0
ENSG00000278330	2	7	7	0	2	7	41	0	0	0
ENSG00000278328	0	0	0	0	0	0	0	0	0	0
ENSG00000278324	0	0	0	0	0	0	0	0	0	0
ENSG00000278323	0	0	0	0	0	0	6	111	0	7
ENSG00000278321	0	0	0	0	0	0	0	0	0	0
ENSG00000278319	0	0	0	0	0	0	0	0	0	0
ENSG00000278318	171	121	285	133	168	444	57	32	0	0
ENSG00000278317	0	0	0	0	0	0	0	0	0	0
ENSG00000278313	0	0	0	0	0	3	5	0	0	0
ENSG00000278311	927	939	1661	878	906	2345	3783	1251	3	26
ENSG00000278309	3	1	0	0	0	2	37	59	0	2
ENSG00000278305	0	0	0	0	0	0	0	0	0	0
ENSG00000278301	4	4	11	3	0	6	9	48	0	0
ENSG00000278299	231	267	341	228	161	583	142	50	1	0
ENSG00000278297	0	0	0	0	0	0	1	0	0	0
ENSG00000278295	0	0	0	0	0	0	0	0	0	0

ENSG00000278292	2	0	0	0	0	0	0	0	0	0
ENSG00000278291	0	1	1	1	0	6	3	0	0	1
ENSG00000278290	0	0	0	0	0	0	2	0	0	0
ENSG00000278289	0	0	0	0	0	0	0	8	0	0
ENSG00000278287	0	0	0	0	0	0	0	0	0	0
ENSG00000278285	0	0	0	0	0	0	0	0	0	0
ENSG00000278283	0	0	0	0	0	0	0	0	0	0
ENSG00000278282	0	0	0	0	0	0	2	0	0	0
ENSG00000278281	0	0	0	0	0	0	1	5	0	0
ENSG00000278278	0	0	0	0	0	0	0	0	0	0
ENSG00000278276	0	0	0	0	0	0	0	0	0	0
ENSG00000278275	0	0	0	0	0	0	0	0	0	0
ENSG00000278274	0	0	0	0	0	0	0	0	0	0
ENSG00000278273	6	5	13	0	1	4	4	5	0	0
ENSG00000278272	0	6	0	6	1	0	6	0	0	0
ENSG00000278267	1	1	1	2	0	5	5	3	0	0
ENSG00000278266	1	1	3	0	0	4	7	9	0	0
ENSG00000278265	0	0	0	0	0	0	0	0	0	0
ENSG00000278264	0	0	0	0	0	0	0	0	0	0
ENSG00000278263	0	0	0	0	0	0	1	6	0	0
ENSG00000278261	0	0	0	0	0	0	0	0	0	0
ENSG00000278260	0	0	0	0	0	0	0	0	0	0
ENSG00000278259	664	567	737	486	457	1372	1371	372	0	23
ENSG00000278258	0	0	0	0	0	0	0	0	0	0
ENSG00000278256	0	0	0	0	0	0	0	0	0	0
ENSG00000278255	0	0	0	0	0	0	1	0	0	0
ENSG00000278254	0	0	0	0	0	0	0	8	0	0
ENSG00000278251	0	0	0	0	0	0	0	0	0	0
ENSG00000278250	9	17	15	9	7	25	26	321	5	5
ENSG00000278249	0	0	0	0	0	0	0	0	0	0
ENSG00000278244	0	0	0	0	0	0	0	0	0	0
ENSG00000278242	0	0	0	0	0	0	0	0	0	0
ENSG00000278239	0	0	2	0	0	5	1	8	0	2
ENSG00000278238	10	14	14	7	9	16	8	5	0	0
ENSG00000278237	0	0	0	0	0	0	0	0	0	0
ENSG00000278236	1	4	0	1	1	1	0	0	0	0
ENSG00000278234	0	0	0	0	0	0	0	0	0	0
ENSG00000278233	30	31	69	41	19	77	45873	6206	184	1150
ENSG00000278231	0	0	0	0	0	0	25	39	0	4
ENSG00000278228	0	0	0	0	0	0	0	0	0	0
ENSG00000278227	0	0	0	0	0	0	0	0	0	0
ENSG00000278226	0	0	0	0	0	0	0	0	0	0
ENSG00000278224	3	1	3	1	0	0	0	0	0	0
ENSG00000278223	0	0	0	0	0	0	0	0	0	0
ENSG00000278222	0	0	0	0	0	0	0	0	0	0
ENSG00000278221	0	0	0	0	0	0	0	0	0	0
ENSG00000278219	0	1	1	0	0	1	6	0	0	0
ENSG00000278218	0	0	0	0	0	0	0	0	0	0
ENSG00000278217	0	0	0	0	0	0	0	0	0	0
ENSG00000278215	1	0	0	0	0	0	0	0	0	0
ENSG00000278214	0	0	0	0	0	0	0	0	0	0
ENSG00000278213	0	0	0	0	1	1	0	3	0	0
ENSG00000278212	3	1	7	6	1	1	6	6	0	0
ENSG00000278206	0	1	9	0	4	4	2	5	0	0
ENSG00000278205	0	0	0	0	0	0	0	0	0	0
ENSG00000278204	0	0	0	0	0	0	0	0	0	0
ENSG00000278203	0	0	0	0	0	0	0	0	0	0

ENSG00000278202	0	0	0	0	0	0	0	0	0	0
ENSG00000278200	0	0	0	0	0	0	1	0	0	0
ENSG00000278197	0	0	0	0	0	0	0	0	0	0
ENSG00000278196	0	0	0	0	0	0	0	0	0	0
ENSG00000278195	0	0	0	0	0	0	17	1	0	0
ENSG00000278193	0	0	0	0	0	0	0	0	0	0
ENSG00000278192	9	6	17	6	6	32	5	0	0	0
ENSG00000278189	30	31	69	41	19	77	45623	6254	167	1111
ENSG00000278188	0	0	0	0	0	0	0	0	0	0
ENSG00000278187	0	0	0	0	0	0	0	0	0	0
ENSG00000278185	6	6	6	5	4	5	5	6	0	0
ENSG00000278184	0	0	0	0	0	0	0	0	0	0
ENSG00000278182	0	1	3	0	0	0	0	0	0	0
ENSG00000278181	0	0	0	0	0	0	0	0	0	0
ENSG00000278180	0	0	0	0	0	0	0	0	0	0
ENSG00000278179	0	0	0	0	0	0	0	0	0	0
ENSG00000278177	1	0	0	1	0	0	1	0	0	0
ENSG00000278176	0	0	0	0	0	0	0	0	0	0
ENSG00000278175	0	0	0	0	0	0	0	0	0	0
ENSG00000278172	0	0	0	0	0	0	0	0	0	0
ENSG00000278171	0	0	0	0	0	0	0	0	0	0
ENSG00000278170	0	0	0	0	0	0	0	0	0	0
ENSG00000278166	0	0	0	0	0	0	0	0	0	0
ENSG00000278164	0	0	0	0	0	0	0	0	0	0
ENSG00000278160	0	0	0	0	0	0	0	0	0	0
ENSG00000278159	0	0	0	0	0	0	0	0	0	0
ENSG00000278158	0	0	0	0	0	0	6	0	0	0
ENSG00000278156	9	8	6	5	1	8	4	11	0	0
ENSG00000278153	0	0	0	1	0	0	3	10	0	0
ENSG00000278151	0	0	0	0	0	0	0	0	0	0
ENSG00000278150	0	0	0	0	0	0	0	0	0	0
ENSG00000278148	0	0	0	0	0	0	0	0	0	0
ENSG00000278147	0	0	0	0	0	0	2	1	0	0
ENSG00000278146	0	0	0	0	0	0	0	6	0	0
ENSG00000278144	0	0	0	0	0	0	0	0	0	0
ENSG00000278143	0	0	0	0	0	0	0	0	0	0
ENSG00000278142	0	0	0	0	0	0	0	0	0	0
ENSG00000278139	0	0	0	0	0	0	0	0	0	0
ENSG00000278138	0	0	0	0	0	0	0	0	0	0
ENSG00000278136	0	0	2	0	0	5	0	0	0	0
ENSG00000278135	0	0	0	0	0	0	0	0	0	0
ENSG00000278134	0	1	1	0	1	0	1	0	0	0
ENSG00000278133	11	25	55	30	21	53	60	53	0	0
ENSG00000278131	0	0	8	1	1	6	2	3	0	0
ENSG00000278130	0	0	0	0	0	0	0	0	0	0
ENSG00000278129	175	160	265	133	161	529	175	127	0	0
ENSG00000278128	0	0	3	0	0	0	0	0	0	0
ENSG00000278126	0	1	5	1	0	5	21	29	0	0
ENSG00000278125	0	0	0	0	0	0	0	0	0	0
ENSG00000278124	0	0	0	0	0	0	0	1	0	0
ENSG00000278123	0	0	0	0	0	0	0	0	0	0
ENSG00000278122	0	0	0	0	0	0	0	0	0	0
ENSG00000278113	0	0	0	0	0	0	0	0	0	0
ENSG00000278112	3	0	9	5	5	11	4	3	0	0
ENSG00000278110	0	0	0	0	0	0	0	0	0	0
ENSG00000278109	0	0	0	0	0	0	0	0	0	0
ENSG00000278108	0	0	0	0	0	0	0	0	0	0

ENSG00000278107	0	0	2	0	0	2	2	10	0	0
ENSG00000278106	0	0	0	0	0	0	0	0	0	0
ENSG00000278105	0	0	0	0	0	0	0	0	0	0
ENSG00000278104	0	0	0	0	0	0	0	0	0	0
ENSG00000278103	0	0	0	0	0	0	0	0	0	0
ENSG00000278102	0	0	0	0	0	0	0	0	0	0
ENSG00000278100	0	0	0	0	0	0	0	0	0	0
ENSG00000278099	0	0	0	0	0	0	18	1	9	52
ENSG00000278098	0	0	0	0	0	0	0	0	0	0
ENSG00000278097	0	0	0	0	0	0	1	1	0	0
ENSG00000278095	0	0	0	0	0	0	5	6	0	0
ENSG00000278094	0	0	0	0	0	0	0	0	0	0
ENSG00000278093	0	0	0	0	0	0	0	0	0	0
ENSG00000278090	0	0	0	0	0	0	4	0	0	0
ENSG00000278089	0	0	0	0	0	0	0	0	0	0
ENSG00000278085	0	0	0	0	0	0	0	0	0	0
ENSG00000278084	0	0	0	0	0	0	0	0	0	0
ENSG00000278083	0	0	0	0	0	0	2	0	0	0
ENSG00000278080	33	44	32	48	29	125	10	18	0	0
ENSG00000278078	0	0	0	0	0	0	3	0	0	0
ENSG00000278077	1	0	0	0	0	0	0	0	0	0
ENSG00000278076	0	0	0	0	0	0	0	0	0	0
ENSG00000278075	1	0	0	1	0	4	3	0	0	0
ENSG00000278073	0	0	0	0	0	0	0	0	0	0
ENSG00000278072	0	0	0	0	0	0	0	0	0	0
ENSG00000278071	0	0	2	0	0	0	5	1	0	0
ENSG00000278069	0	0	0	0	0	0	0	0	0	0
ENSG00000278068	0	0	0	0	0	0	2	0	0	0
ENSG00000278067	0	0	0	0	0	0	0	0	0	0
ENSG00000278065	0	0	0	0	0	0	1	0	0	0
ENSG00000278060	0	0	0	0	0	0	0	0	0	0
ENSG00000278059	0	0	0	0	0	0	0	0	0	0
ENSG00000278058	6	5	5	3	6	11	15	14	0	0
ENSG00000278057	0	0	0	0	0	0	0	0	0	0
ENSG00000278056	0	0	0	0	0	0	2	0	0	0
ENSG00000278055	0	0	0	0	0	0	1	0	0	0
ENSG00000278054	0	0	0	0	0	0	0	0	0	0
ENSG00000278053	484	525	977	398	394	1363	786	295	1	5
ENSG00000278052	0	0	0	0	0	0	0	0	0	0
ENSG00000278050	0	0	0	0	0	0	0	0	0	0
ENSG00000278048	0	1	5	6	2	12	247	27	20	301
ENSG00000278047	11	20	25	4	6	13	945	596	120	484
ENSG00000278045	0	0	0	0	0	0	0	0	0	0
ENSG00000278044	0	0	0	0	0	0	0	5	0	0
ENSG00000278041	2	1	7	2	2	10	0	0	0	0
ENSG00000278040	0	0	0	0	0	0	0	0	0	0
ENSG00000278039	0	0	0	0	0	0	0	0	0	0
ENSG00000278038	0	0	0	0	0	0	0	0	0	0
ENSG00000278035	1	2	1	4	2	5	0	7	0	0
ENSG00000278034	0	0	0	0	0	0	0	0	0	0
ENSG00000278030	0	0	0	0	0	0	51	0	0	0
ENSG00000278029	0	0	0	0	0	0	0	0	0	0
ENSG00000278028	0	2	0	0	0	0	0	0	0	0
ENSG00000278027	0	0	0	0	0	0	0	0	0	0
ENSG00000278026	0	0	0	0	0	0	0	0	0	0
ENSG00000278023	6	3	5	5	3	1	1	0	0	0
ENSG00000278022	0	3	0	0	1	1	52	20	0	0

ENSG00000278020	0	0	0	0	0	0	0	0	0	0
ENSG00000278017	6	1	0	0	0	5	7	2	0	0
ENSG00000278014	0	0	0	0	0	0	0	1	0	0
ENSG00000278013	0	2	0	0	0	0	2	0	0	0
ENSG00000278012	0	0	0	0	0	0	0	0	0	0
ENSG00000278011	0	0	3	0	0	0	0	0	0	0
ENSG00000278010	0	0	0	0	0	0	0	0	0	0
ENSG00000278009	0	0	3	0	0	2	3	0	0	0
ENSG00000278008	0	0	0	0	0	0	0	0	0	0
ENSG00000278007	0	0	0	0	0	0	0	0	0	0
ENSG00000278005	0	0	0	0	0	0	0	0	0	0
ENSG00000278004	0	0	0	0	0	0	1	0	0	0
ENSG00000278002	0	5	3	0	0	4	3	4	0	0
ENSG00000278001	0	0	0	0	0	0	0	0	0	0
ENSG00000278000	12	8	23	18	9	30	38	7	0	0
ENSG00000277999	0	0	0	0	0	0	45	12	0	0
ENSG00000277998	2	1	7	1	2	1	56	26	1	4
ENSG00000277997	0	0	0	0	0	0	0	0	0	0
ENSG00000277994	0	0	0	0	0	0	0	0	0	0
ENSG00000277992	0	0	0	0	0	0	0	0	0	0
ENSG00000277991	418	333	634	257	301	838	162	40	0	0
ENSG00000277990	0	0	0	0	0	0	0	0	0	0
ENSG00000277988	0	0	0	0	0	0	0	0	0	0
ENSG00000277987	0	0	0	0	0	0	1	6	0	0
ENSG00000277986	0	0	0	0	0	0	0	0	0	0
ENSG00000277985	0	0	0	0	0	0	0	0	0	0
ENSG00000277984	0	0	0	0	0	0	0	0	0	0
ENSG00000277981	0	0	0	0	0	0	0	11	0	0
ENSG00000277979	0	0	0	0	0	0	0	0	0	0
ENSG00000277978	0	1	0	1	3	0	30	8	0	0
ENSG00000277977	2	2	0	1	2	4	1	9	0	0
ENSG00000277975	0	0	0	0	0	0	0	0	0	0
ENSG00000277973	0	0	0	0	0	0	0	0	0	0
ENSG00000277972	85	81	165	83	73	228	717	622	0	3
ENSG00000277971	0	0	0	0	0	0	0	0	0	0
ENSG00000277970	0	2	0	0	0	0	0	0	0	0
ENSG00000277969	7	14	12	8	9	11	709	201	3	0
ENSG00000277968	1	0	1	1	0	0	5	0	0	0
ENSG00000277967	0	0	0	0	0	0	0	0	0	0
ENSG00000277966	0	0	0	0	0	0	0	0	0	0
ENSG00000277965	0	0	0	0	0	0	1	0	0	0
ENSG00000277962	0	0	0	0	0	0	0	7	0	0
ENSG00000277960	0	0	0	0	0	0	0	0	0	0
ENSG00000277959	8	9	4	8	3	26	31	18	0	0
ENSG00000277958	0	0	1	0	0	0	0	3	0	0
ENSG00000277957	0	0	1	2	2	14	10	3	0	0
ENSG00000277955	0	0	0	0	0	0	0	0	0	0
ENSG00000277954	0	0	0	0	0	3	2	14	0	0
ENSG00000277952	0	0	1	0	0	0	0	0	0	0
ENSG00000277951	0	0	0	0	0	0	0	0	0	0
ENSG00000277950	0	0	0	0	0	0	1	0	0	0
ENSG00000277948	0	0	0	0	0	0	0	0	0	0
ENSG00000277947	0	0	0	0	0	0	0	0	0	0
ENSG00000277946	0	0	0	0	0	0	0	0	0	0
ENSG00000277945	0	0	0	0	0	2	1	0	0	0
ENSG00000277942	0	0	0	0	0	0	0	0	0	0
ENSG00000277941	0	0	0	0	0	0	0	0	0	0

ENSG00000277939	0	0	0	0	0	0	0	0	0	0
ENSG00000277938	7	21	43	11	10	25	21	13	0	0
ENSG00000277937	0	0	0	0	0	0	0	0	0	0
ENSG00000277935	0	0	0	0	0	0	0	0	0	0
ENSG00000277934	0	0	0	0	1	2	27	0	0	0
ENSG00000277933	0	0	0	0	0	0	0	0	0	0
ENSG00000277932	0	0	0	0	0	0	0	0	0	0
ENSG00000277930	0	0	0	0	0	0	0	1	0	0
ENSG00000277929	0	0	0	0	0	0	0	0	0	0
ENSG00000277925	0	0	0	0	0	0	0	0	0	0
ENSG00000277922	0	0	0	0	0	0	0	0	0	0
ENSG00000277920	0	0	0	0	0	0	0	0	0	0
ENSG00000277919	0	0	0	0	0	0	0	0	0	0
ENSG00000277918	3	1	8	2	2	5	493	4	46	375
ENSG00000277916	0	3	0	0	0	10	14	0	0	0
ENSG00000277914	0	0	0	0	0	0	0	0	0	0
ENSG00000277913	0	0	0	0	0	0	0	0	0	0
ENSG00000277912	0	0	0	0	0	0	0	0	0	0
ENSG00000277911	0	0	0	0	0	0	1	0	0	0
ENSG00000277910	0	0	0	0	0	0	0	0	0	0
ENSG00000277907	0	0	0	0	0	0	0	0	0	0
ENSG00000277906	0	0	0	0	0	0	0	0	0	0
ENSG00000277904	0	0	0	0	0	0	0	0	0	0
ENSG00000277903	0	1	5	6	1	11	240	25	27	292
ENSG00000277901	1	2	0	0	0	0	11	2	0	0
ENSG00000277900	0	0	0	0	0	0	0	0	0	0
ENSG00000277899	0	0	0	0	0	0	0	0	0	0
ENSG00000277896	0	0	0	0	0	0	0	0	0	0
ENSG00000277895	0	0	0	0	1	4	11	0	0	0
ENSG00000277893	0	0	0	0	0	5	16	4	0	0
ENSG00000277892	0	2	0	0	0	0	2	0	0	0
ENSG00000277889	0	0	0	0	0	0	0	0	0	0
ENSG00000277888	0	0	0	0	0	0	0	0	0	0
ENSG00000277887	0	0	0	0	0	0	0	0	0	0
ENSG00000277886	0	0	0	0	0	0	0	0	0	0
ENSG00000277884	0	0	0	0	0	0	0	0	0	0
ENSG00000277883	8	21	23	16	7	39	3	0	0	0
ENSG00000277882	0	0	0	0	0	0	0	0	0	0
ENSG00000277881	0	0	0	0	0	0	0	0	0	0
ENSG00000277880	0	0	0	0	0	0	0	0	0	0
ENSG00000277879	17	7	11	4	12	20	44	47	0	0
ENSG00000277878	0	0	0	0	0	0	0	0	0	0
ENSG00000277877	3	3	10	4	4	0	15	8	0	0
ENSG00000277876	0	0	0	0	0	0	2	0	0	0
ENSG00000277875	0	0	0	0	0	0	0	0	0	0
ENSG00000277874	2	5	2	2	2	4	3	6	0	0
ENSG00000277873	2	0	3	1	2	7	5	0	0	0
ENSG00000277872	0	0	0	0	0	0	0	0	0	0
ENSG00000277870	0	0	0	0	0	2	0	2	0	0
ENSG00000277869	0	0	0	0	0	0	0	0	0	0
ENSG00000277867	4	2	15	2	3	4	6	11	0	0
ENSG00000277866	0	0	0	0	0	0	0	0	0	0
ENSG00000277865	0	6	8	0	3	16	2	6	0	0
ENSG00000277864	0	0	0	0	0	0	0	0	0	0
ENSG00000277863	0	0	0	0	0	0	0	0	0	0
ENSG00000277862	0	2	0	0	0	0	0	0	0	0
ENSG00000277861	0	0	0	0	1	0	0	2	0	0

ENSG00000277859	0	0	0	0	0	0	0	0	0	0
ENSG00000277858	1	0	0	0	0	0	3	6	0	0
ENSG00000277855	1	0	0	0	0	0	0	0	0	0
ENSG00000277854	0	0	0	0	0	0	0	0	0	0
ENSG00000277852	0	0	0	0	0	0	0	0	0	0
ENSG00000277851	0	0	0	0	0	0	1	6	0	0
ENSG00000277847	0	0	0	0	0	0	0	0	0	0
ENSG00000277846	0	0	0	0	0	0	6	0	0	0
ENSG00000277843	0	0	0	0	0	0	1	0	0	0
ENSG00000277842	0	0	0	0	0	0	0	0	0	0
ENSG00000277841	0	0	0	0	0	0	0	0	0	0
ENSG00000277840	0	0	0	0	0	7	2	2	0	0
ENSG00000277839	0	0	0	0	0	0	0	0	0	0
ENSG00000277838	0	0	0	0	0	0	0	0	0	0
ENSG00000277837	0	0	0	0	0	0	0	5	0	0
ENSG00000277832	0	0	0	0	0	0	0	0	0	0
ENSG00000277831	0	0	1	0	0	0	1	0	0	0
ENSG00000277830	0	0	0	0	0	0	0	0	0	0
ENSG00000277829	0	0	0	0	0	1	1	0	0	0
ENSG00000277828	0	0	0	0	0	0	0	0	0	0
ENSG00000277827	0	0	0	0	0	0	0	0	0	0
ENSG00000277826	0	0	0	0	0	0	0	0	0	0
ENSG00000277825	0	0	0	0	1	0	0	4	0	1
ENSG00000277822	0	0	0	0	0	0	0	0	0	0
ENSG00000277821	0	0	0	0	0	0	0	0	0	0
ENSG00000277819	0	0	0	0	0	0	0	0	0	0
ENSG00000277818	0	0	0	0	0	0	0	0	0	0
ENSG00000277817	0	1	2	0	0	0	0	0	0	0
ENSG00000277815	0	0	0	0	0	0	0	0	0	0
ENSG00000277814	0	0	0	0	0	0	0	0	0	0
ENSG00000277813	0	0	0	0	0	0	0	0	0	0
ENSG00000277812	0	0	2	0	0	0	0	0	0	0
ENSG00000277810	0	0	0	0	1	0	5	0	0	0
ENSG00000277809	0	0	0	0	0	0	0	0	0	0
ENSG00000277806	0	0	0	0	0	0	2	3	0	0
ENSG00000277805	0	0	0	0	0	0	0	0	0	0
ENSG00000277803	0	0	0	0	0	0	0	0	0	0
ENSG00000277801	0	0	0	0	0	0	0	0	0	0
ENSG00000277798	0	0	0	0	0	0	0	0	0	0
ENSG00000277797	0	0	0	0	0	0	2	0	0	0
ENSG00000277795	0	0	0	0	0	0	0	0	0	0
ENSG00000277794	0	0	0	0	0	1	0	4	0	0
ENSG00000277793	0	0	0	0	0	0	0	0	0	0
ENSG00000277792	0	0	0	0	0	0	0	0	0	0
ENSG00000277791	787	719	1376	789	755	2443	5521	2609	16	157
ENSG00000277790	0	0	0	0	0	0	0	0	0	0
ENSG00000277788	0	0	0	0	0	0	0	4	0	0
ENSG00000277787	0	0	0	0	0	0	0	0	0	0
ENSG00000277785	0	0	0	0	0	0	0	0	0	0
ENSG00000277784	0	0	0	0	0	0	0	0	0	0
ENSG00000277782	10	8	20	9	9	29	39	113	0	0
ENSG00000277780	0	0	0	0	0	0	0	0	0	0
ENSG00000277779	0	0	0	0	0	0	0	0	0	0
ENSG00000277778	20	23	47	22	22	65	23	55	0	0
ENSG00000277777	0	0	0	0	0	0	0	0	0	0
ENSG00000277775	0	0	0	4	0	0	3	0	0	0
ENSG00000277774	0	0	0	0	0	0	0	0	0	0

ENSG00000277772	0	0	0	0	0	0	0	0	0	0
ENSG00000277771	0	0	0	0	0	0	0	0	0	0
ENSG00000277770	0	0	0	0	0	2	1	0	0	0
ENSG00000277769	0	0	0	0	0	0	0	2	0	0
ENSG00000277767	0	1	0	0	0	3	15	0	0	0
ENSG00000277766	0	0	0	0	0	0	0	0	0	0
ENSG00000277764	0	0	0	0	0	0	0	0	0	0
ENSG00000277763	0	0	0	0	0	0	5	0	0	0
ENSG00000277762	0	0	0	0	0	0	1	6	0	0
ENSG00000277760	0	0	0	0	0	0	0	0	0	0
ENSG00000277759	0	0	0	0	0	0	2	0	0	0
ENSG00000277758	29	27	37	25	18	50	79	192	0	7
ENSG00000277757	0	0	0	0	0	0	0	0	0	0
ENSG00000277756	0	0	0	0	0	0	2	0	0	0
ENSG00000277755	0	0	0	0	0	0	0	0	0	0
ENSG00000277754	0	0	0	0	0	0	0	2	0	0
ENSG00000277752	0	0	0	0	0	0	0	0	0	0
ENSG00000277749	0	0	0	0	0	0	3	0	0	7
ENSG00000277748	1	0	0	1	0	0	4	0	0	0
ENSG00000277747	0	0	0	0	0	0	0	0	0	0
ENSG00000277746	0	0	0	0	0	0	0	3	0	0
ENSG00000277745	6	0	3	3	0	0	7	6	0	0
ENSG00000277744	2	3	3	1	1	7	11	1	0	0
ENSG00000277743	0	0	0	0	0	0	0	0	0	0
ENSG00000277741	47	72	104	27	40	114	3	17	0	0
ENSG00000277740	0	0	0	0	0	0	0	0	0	0
ENSG00000277739	30	31	69	41	19	77	45598	6176	190	1174
ENSG00000277738	0	0	0	0	0	0	2	0	0	0
ENSG00000277737	2	0	0	2	0	0	6	14	0	0
ENSG00000277736	0	0	0	0	0	0	0	0	0	0
ENSG00000277735	0	0	0	0	0	0	0	0	0	0
ENSG00000277734	8	3	3	3	1	10	900	136	2	7
ENSG00000277732	0	0	0	0	0	0	0	0	0	0
ENSG00000277731	0	0	0	0	0	0	0	0	0	0
ENSG00000277728	4	3	6	3	6	1	5	0	0	0
ENSG00000277727	0	0	0	0	0	0	0	0	0	0
ENSG00000277726	0	0	0	0	0	0	0	0	0	0
ENSG00000277724	0	0	0	0	0	0	0	0	0	0
ENSG00000277723	0	0	0	0	0	0	0	0	0	0
ENSG00000277721	0	0	0	0	0	0	0	0	0	0
ENSG00000277720	0	0	0	0	0	0	0	0	0	0
ENSG00000277719	0	0	0	0	0	0	0	0	0	0
ENSG00000277718	1	1	0	0	0	5	1	0	0	0
ENSG00000277717	0	0	0	0	0	0	0	0	0	0
ENSG00000277716	0	0	0	0	0	0	0	0	0	0
ENSG00000277715	1	9	14	6	7	18	6	15	0	0
ENSG00000277714	0	0	0	0	0	0	0	0	0	0
ENSG00000277713	0	0	0	0	0	0	0	0	0	0
ENSG00000277712	0	0	0	0	0	4	0	0	0	0
ENSG00000277711	0	0	0	0	0	2	0	0	0	0
ENSG00000277710	0	0	0	0	0	0	0	0	0	0
ENSG00000277708	0	0	0	0	0	0	0	0	0	0
ENSG00000277706	0	0	0	0	0	1	1	0	0	0
ENSG00000277705	0	0	0	0	0	0	0	0	0	0
ENSG00000277704	0	0	0	0	0	0	0	0	0	0
ENSG00000277703	0	0	0	0	0	0	0	0	0	0
ENSG00000277702	1	1	0	2	0	4	6	33	0	0

ENSG00000277701	704	692	1083	542	611	1644	424	273	0	0
ENSG00000277700	0	0	0	0	0	0	0	0	0	0
ENSG00000277699	0	0	0	0	0	0	0	0	0	0
ENSG00000277698	0	0	0	0	0	0	0	0	0	0
ENSG00000277697	0	0	0	0	0	0	0	0	0	0
ENSG00000277695	0	0	0	0	2	3	0	5	0	0
ENSG00000277694	0	0	0	0	0	0	0	0	0	0
ENSG00000277693	1	0	0	0	1	0	0	0	0	0
ENSG00000277692	0	0	0	0	0	0	16	15	0	0
ENSG00000277690	0	0	0	0	0	0	0	0	0	0
ENSG00000277689	0	0	0	0	0	0	0	0	0	0
ENSG00000277688	0	0	0	0	0	0	0	0	0	0
ENSG00000277687	4	5	2	1	2	3	2	7	0	0
ENSG00000277686	0	0	0	0	0	0	0	0	0	0
ENSG00000277685	0	0	0	0	0	0	0	0	0	0
ENSG00000277684	0	0	0	0	0	0	8	0	0	0
ENSG00000277683	0	0	0	0	0	0	0	0	0	0
ENSG00000277682	0	0	0	0	0	0	0	0	0	0
ENSG00000277681	0	1	4	1	0	0	0	0	0	0
ENSG00000277678	0	0	0	0	2	1	2	0	0	0
ENSG00000277677	0	0	0	0	0	0	5	1	0	7
ENSG00000277675	2	0	1	0	0	8	1	0	0	0
ENSG00000277673	0	0	0	0	0	0	0	0	0	0
ENSG00000277672	0	0	0	0	0	0	0	0	0	0
ENSG00000277671	0	0	0	0	0	0	19	1	0	0
ENSG00000277670	0	0	0	0	0	0	0	0	0	0
ENSG00000277669	0	0	0	0	0	0	0	0	0	0
ENSG00000277668	0	0	0	0	0	0	0	0	0	0
ENSG00000277665	0	0	0	0	0	0	0	0	0	0
ENSG00000277664	0	0	0	0	0	0	0	2	0	0
ENSG00000277663	0	0	0	0	0	0	0	0	0	0
ENSG00000277662	0	1	2	1	0	8	0	1	0	0
ENSG00000277661	0	0	0	0	0	0	0	0	0	0
ENSG00000277655	0	0	0	0	0	0	1	0	0	0
ENSG00000277654	1	0	0	0	0	0	0	1	0	0
ENSG00000277653	0	0	0	0	0	0	0	0	0	0
ENSG00000277652	0	0	0	0	0	0	0	0	0	0
ENSG00000277651	0	0	0	0	0	0	0	0	0	0
ENSG00000277648	0	0	0	0	0	0	0	0	0	0
ENSG00000277647	0	0	0	0	0	0	0	0	0	0
ENSG00000277646	0	0	0	0	0	0	0	0	0	0
ENSG00000277645	0	0	0	0	0	0	0	1	0	0
ENSG00000277644	5	10	21	9	4	8	112777	2682	129	1302
ENSG00000277640	0	0	0	0	0	0	0	0	0	0
ENSG00000277639	2	6	11	5	8	26	79	98	1	48
ENSG00000277637	0	0	0	0	0	0	0	0	0	0
ENSG00000277636	0	0	0	0	0	0	0	0	0	0
ENSG00000277635	0	0	0	0	0	0	0	1	0	0
ENSG00000277634	0	0	0	0	0	0	1	1	0	0
ENSG00000277632	0	0	0	0	0	0	2173	30	43	264
ENSG00000277631	0	0	0	0	0	6	8	105	0	0
ENSG00000277628	0	0	0	0	0	0	0	0	0	0
ENSG00000277627	0	0	0	0	0	2	0	11	0	0
ENSG00000277624	3	1	0	2	1	12	0	0	0	0
ENSG00000277622	0	0	0	0	0	0	0	0	0	0
ENSG00000277621	0	0	0	0	0	0	1	0	0	0
ENSG00000277619	2	2	4	1	1	10	0	0	0	0

ENSG00000277618	0	0	0	0	0	1	0	2	0	0
ENSG00000277617	0	0	0	0	0	0	0	0	0	0
ENSG00000277613	0	0	3	0	0	0	0	6	0	0
ENSG00000277612	0	0	0	0	0	0	0	0	0	0
ENSG00000277611	0	0	0	0	0	0	0	0	0	0
ENSG00000277610	0	0	0	2	1	0	4	1	0	0
ENSG00000277609	0	0	0	0	0	0	0	0	0	0
ENSG00000277608	0	0	0	0	0	0	0	0	0	0
ENSG00000277605	0	0	0	0	0	0	0	0	0	0
ENSG00000277604	0	0	0	0	0	0	0	0	0	0
ENSG00000277602	0	1	1	0	1	0	7	0	0	0
ENSG00000277601	0	0	0	0	0	0	0	0	0	0
ENSG00000277599	0	0	0	0	0	0	0	0	0	0
ENSG00000277598	0	0	0	0	0	0	0	0	0	0
ENSG00000277597	0	0	0	1	0	0	0	0	0	0
ENSG00000277595	3	3	1	4	0	3	20	0	0	0
ENSG00000277593	0	0	0	0	0	0	0	0	0	0
ENSG00000277592	0	0	0	0	0	0	0	5	0	0
ENSG00000277591	0	0	0	0	0	0	0	0	0	0
ENSG00000277590	0	0	0	0	0	0	0	0	0	0
ENSG00000277589	0	0	0	0	0	0	15	18	0	0
ENSG00000277588	0	0	0	0	0	0	0	0	0	0
ENSG00000277587	0	1	0	0	0	0	0	5	0	0
ENSG00000277586	12	12	17	8	11	14	6	0	0	0
ENSG00000277583	0	0	0	0	0	0	0	0	0	0
ENSG00000277582	0	0	0	0	0	0	0	0	0	0
ENSG00000277581	0	0	0	1	0	0	0	0	0	0
ENSG00000277579	0	0	0	1	0	1	2	8	0	0
ENSG00000277578	0	0	0	0	0	0	0	0	0	0
ENSG00000277577	0	0	0	0	0	0	0	0	0	0
ENSG00000277576	0	0	0	0	0	0	0	0	0	0
ENSG00000277575	0	0	0	0	0	0	0	2	0	0
ENSG00000277573	0	0	0	0	0	0	7	0	0	0
ENSG00000277572	0	0	0	1	0	0	0	0	0	0
ENSG00000277569	0	0	0	0	0	0	0	0	0	0
ENSG00000277568	0	0	1	0	0	0	0	2	0	0
ENSG00000277566	0	2	7	8	5	7	5	0	0	0
ENSG00000277565	0	0	0	0	0	0	0	0	0	0
ENSG00000277563	0	2	0	0	0	0	0	0	0	0
ENSG00000277562	0	0	0	0	0	0	0	0	0	0
ENSG00000277561	9	13	25	12	7	26	6	4	0	0
ENSG00000277559	6	7	2	1	1	12	3	0	0	0
ENSG00000277558	0	0	0	0	0	0	0	0	0	0
ENSG00000277556	0	1	0	0	0	0	0	0	0	0
ENSG00000277555	2	0	0	0	0	0	6	2	0	0
ENSG00000277553	0	0	0	0	0	0	0	0	0	0
ENSG00000277550	0	0	1	0	0	0	0	0	0	0
ENSG00000277549	0	0	0	0	0	0	0	0	0	0
ENSG00000277548	0	0	2	2	0	4	5	3	0	0
ENSG00000277547	0	0	0	0	0	0	0	0	0	0
ENSG00000277545	0	0	0	0	0	0	0	0	0	0
ENSG00000277544	0	0	0	0	0	0	0	0	0	0
ENSG00000277543	0	0	0	0	0	0	0	0	0	0
ENSG00000277542	0	0	0	0	0	0	0	0	0	0
ENSG00000277541	0	0	0	0	0	0	2	0	0	0
ENSG00000277539	0	0	0	0	0	0	0	0	0	0
ENSG00000277537	0	0	0	0	0	0	0	0	0	0

ENSG00000277535	0	0	0	0	0	0	0	0	0	0
ENSG00000277534	16	36	34	21	34	61	15	13	0	0
ENSG00000277533	0	0	0	0	0	0	0	0	0	0
ENSG00000277532	0	0	0	0	0	0	2	0	0	0
ENSG00000277531	0	0	0	0	0	3	0	0	0	0
ENSG00000277529	0	0	1	0	0	0	0	0	0	0
ENSG00000277528	0	0	0	0	0	0	0	0	0	0
ENSG00000277527	0	0	0	0	0	0	1	4	0	0
ENSG00000277526	0	0	0	0	0	0	0	5	0	0
ENSG00000277525	0	0	0	0	0	0	0	0	0	0
ENSG00000277524	0	0	0	0	0	0	0	0	0	0
ENSG00000277521	0	0	0	0	0	0	0	0	0	0
ENSG00000277520	0	0	0	0	0	0	0	0	0	0
ENSG00000277519	0	0	0	0	0	0	0	0	0	0
ENSG00000277516	0	0	0	0	0	0	0	0	0	0
ENSG00000277515	0	0	0	0	0	0	0	0	0	0
ENSG00000277514	0	0	0	0	0	0	0	0	0	0
ENSG00000277513	0	0	0	0	0	0	0	0	0	0
ENSG00000277512	0	0	0	0	0	0	0	0	0	0
ENSG00000277511	2	0	2	0	2	6	51	9	0	29
ENSG00000277510	0	0	0	0	0	0	0	0	0	1
ENSG00000277509	0	0	0	0	0	0	0	0	0	0
ENSG00000277508	0	0	0	0	0	0	0	0	0	0
ENSG00000277506	0	0	0	0	0	0	0	0	0	0
ENSG00000277505	0	0	0	0	0	0	0	0	0	0
ENSG00000277504	0	0	0	0	0	0	0	0	0	0
ENSG00000277503	0	0	0	0	0	0	0	0	0	0
ENSG00000277502	0	0	0	0	0	0	0	0	0	0
ENSG00000277501	0	0	0	0	0	0	9	3	0	0
ENSG00000277500	0	0	0	0	0	0	0	0	0	0
ENSG00000277499	0	0	0	0	0	0	0	0	0	0
ENSG00000277498	1	0	1	1	1	3	1	19	0	0
ENSG00000277497	0	0	0	0	0	0	0	0	0	0
ENSG00000277496	0	0	0	0	1	3	83	52	0	0
ENSG00000277494	0	0	0	0	0	0	24	3984	0	0
ENSG00000277493	0	2	0	0	0	0	0	0	0	0
ENSG00000277492	0	0	0	0	0	0	0	0	0	0
ENSG00000277491	0	2	6	3	8	1	3	6	0	0
ENSG00000277490	0	0	0	0	0	0	0	0	0	0
ENSG00000277489	0	0	0	0	0	0	0	0	0	0
ENSG00000277488	0	0	0	0	0	0	0	0	0	0
ENSG00000277487	0	0	0	0	0	0	0	0	0	0
ENSG00000277486	0	0	0	0	0	0	0	1	0	0
ENSG00000277483	0	0	0	0	0	0	0	2	0	0
ENSG00000277482	0	0	0	0	0	0	0	0	0	0
ENSG00000277481	2	3	0	1	1	4	8	4	0	0
ENSG00000277479	0	0	5	0	0	2	4	0	0	0
ENSG00000277478	0	0	0	0	0	0	0	0	0	0
ENSG00000277476	5	20	23	17	6	20	84	141	0	0
ENSG00000277474	0	0	0	0	0	0	0	0	0	0
ENSG00000277473	0	0	0	0	0	0	0	0	0	0
ENSG00000277472	0	0	0	0	0	0	0	0	0	0
ENSG00000277469	0	0	0	0	0	0	0	0	0	0
ENSG00000277468	0	0	0	0	0	0	0	0	0	0
ENSG00000277467	0	0	0	0	0	0	0	0	0	0
ENSG00000277466	0	0	0	0	0	0	0	0	0	0
ENSG00000277465	0	0	0	0	0	0	0	0	0	0

ENSG00000277464	0	0	0	0	0	0	0	0	0	0
ENSG00000277463	0	1	0	1	0	3	10	0	0	0
ENSG00000277462	7	5	21	15	7	8	538	59	0	4
ENSG00000277460	0	0	0	0	0	0	0	0	0	0
ENSG00000277459	0	3	2	2	0	1	11	0	0	0
ENSG00000277458	0	0	0	0	0	0	0	0	0	0
ENSG00000277455	0	0	0	0	0	0	0	0	0	0
ENSG00000277453	0	1	0	0	0	1	2	5	0	0
ENSG00000277452	0	0	0	2	0	0	8	3	0	0
ENSG00000277451	0	0	0	0	0	0	0	0	0	0
ENSG00000277450	0	0	0	0	0	0	2	0	0	0
ENSG00000277449	5	4	13	3	4	11	11	54	0	0
ENSG00000277448	0	0	0	0	0	0	0	0	0	0
ENSG00000277447	0	1	2	0	1	3	4	2	0	0
ENSG00000277446	0	0	0	0	0	0	0	0	0	0
ENSG00000277444	0	0	0	0	0	0	0	0	0	0
ENSG00000277443	1937	2429	4996	1711	1895	5423	6396	1359	91	3455
ENSG00000277441	0	0	0	0	0	0	0	0	0	0
ENSG00000277440	0	1	4	1	0	9	1	3	0	1
ENSG00000277438	0	0	0	0	0	0	0	0	0	0
ENSG00000277437	0	0	0	0	0	0	68	14	3	9
ENSG00000277436	0	0	0	0	0	0	0	1	0	0
ENSG00000277435	0	2	0	0	0	4	7	4	0	0
ENSG00000277433	0	0	0	0	0	0	0	0	0	0
ENSG00000277431	0	0	0	0	0	0	0	0	0	0
ENSG00000277430	0	0	0	0	0	0	0	5	0	1
ENSG00000277427	0	0	0	0	0	0	0	0	0	0
ENSG00000277426	0	0	0	0	0	0	0	0	0	0
ENSG00000277425	0	4	0	1	0	4	0	0	0	0
ENSG00000277423	0	0	2	1	0	0	2	0	0	0
ENSG00000277422	0	0	0	0	0	0	0	0	0	0
ENSG00000277420	0	0	0	2	2	4	14	10	0	0
ENSG00000277418	0	0	0	0	0	0	0	0	0	0
ENSG00000277417	0	0	0	0	0	0	0	0	0	0
ENSG00000277416	0	0	0	0	0	0	0	0	0	0
ENSG00000277413	0	0	0	0	0	0	0	0	0	0
ENSG00000277412	1	0	0	0	0	0	0	12	0	0
ENSG00000277411	0	0	0	0	2	0	3	0	0	0
ENSG00000277410	0	0	0	0	0	0	0	0	0	0
ENSG00000277408	0	0	0	0	0	0	0	0	0	0
ENSG00000277406	1	7	6	3	2	4	41	7	0	0
ENSG00000277402	0	0	0	0	0	0	3	0	0	0
ENSG00000277399	5	2	1	3	0	2	7	1	0	0
ENSG00000277397	0	0	0	0	0	0	0	0	0	0
ENSG00000277396	0	0	0	0	1	1	2	9	0	0
ENSG00000277394	0	0	0	0	0	0	0	4	0	0
ENSG00000277393	0	0	0	0	0	0	0	0	0	0
ENSG00000277391	0	0	0	2	0	0	2	0	0	0
ENSG00000277388	0	0	0	0	0	0	0	0	0	0
ENSG00000277387	0	0	0	0	0	0	2	0	0	0
ENSG00000277386	0	0	0	0	0	0	4	2	0	0
ENSG00000277385	0	0	0	0	0	0	0	1	0	0
ENSG00000277383	1	1	0	3	0	1	17	4	0	0
ENSG00000277382	0	2	2	1	0	1	1	2	0	0
ENSG00000277379	0	0	0	0	0	0	24	4	1	0
ENSG00000277373	0	0	0	0	0	0	3	0	0	0
ENSG00000277372	0	0	0	0	0	0	0	1	0	0

ENSG00000277371	0	1	2	0	0	0	0	0	0	0
ENSG00000277370	0	0	0	0	0	0	0	0	0	0
ENSG00000277369	14	10	6	8	3	24	16	14	0	0
ENSG00000277368	1	0	0	0	0	0	0	0	0	0
ENSG00000277367	0	0	0	0	0	0	0	0	0	0
ENSG00000277363	27	20	45	20	29	84	9	14	0	0
ENSG00000277360	0	0	0	0	0	0	0	0	0	0
ENSG00000277358	0	0	0	0	0	0	0	0	0	0
ENSG00000277357	0	0	0	0	0	0	0	0	0	0
ENSG00000277353	0	0	0	0	0	0	0	0	0	0
ENSG00000277352	0	0	0	0	0	0	0	0	0	0
ENSG00000277351	0	0	0	0	0	0	3	64	0	0
ENSG00000277350	0	0	0	0	0	0	0	3	0	0
ENSG00000277349	1	10	3	3	4	12	4	1	0	0
ENSG00000277348	0	0	0	0	0	0	0	0	0	0
ENSG00000277347	0	0	0	0	0	0	0	0	0	0
ENSG00000277346	0	0	0	0	0	0	0	0	0	0
ENSG00000277344	0	0	0	0	0	0	0	0	0	0
ENSG00000277342	2	0	0	0	0	1	9	2	0	0
ENSG00000277341	0	0	0	0	0	0	0	1	0	0
ENSG00000277340	1	1	0	0	0	0	0	1	0	0
ENSG00000277338	0	0	0	0	0	0	0	0	0	0
ENSG00000277333	0	0	0	0	0	0	0	0	0	0
ENSG00000277332	0	0	0	0	0	0	0	0	0	0
ENSG00000277331	0	0	0	0	0	0	3	0	0	0
ENSG00000277330	0	0	0	0	0	0	0	0	0	0
ENSG00000277329	0	0	0	0	0	0	0	0	0	0
ENSG00000277328	0	0	0	0	0	0	0	0	0	0
ENSG00000277327	1	0	1	1	1	1	1	0	0	0
ENSG00000277326	0	0	0	0	0	0	0	0	0	0
ENSG00000277325	0	0	0	0	0	0	0	0	0	0
ENSG00000277324	0	1	0	0	0	2	1	14	0	0
ENSG00000277323	0	0	0	0	0	0	0	0	0	0
ENSG00000277322	0	4	4	0	1	8	0	5	0	0
ENSG00000277321	0	0	0	0	0	0	0	0	0	0
ENSG00000277320	0	0	0	0	0	0	0	0	0	0
ENSG00000277319	0	0	0	0	0	0	0	0	0	0
ENSG00000277315	0	0	0	0	0	0	0	0	0	0
ENSG00000277313	0	0	0	0	0	0	0	1	0	0
ENSG00000277312	0	0	0	0	0	0	0	0	0	0
ENSG00000277311	0	0	0	0	0	0	0	0	0	0
ENSG00000277310	6	0	2	1	3	6	2	1	0	6
ENSG00000277308	0	0	0	0	0	0	0	0	0	0
ENSG00000277306	0	0	0	0	0	0	0	0	0	0
ENSG00000277305	0	0	0	0	0	0	0	0	0	0
ENSG00000277304	33	35	51	21	21	83	7	7	0	0
ENSG00000277302	0	0	0	0	0	0	0	0	0	0
ENSG00000277301	0	0	0	0	0	0	0	7	0	0
ENSG00000277300	0	0	0	0	0	0	0	0	0	0
ENSG00000277299	0	0	1	0	0	0	1	1	0	0
ENSG00000277297	4	5	0	1	2	7	0	0	0	0
ENSG00000277296	0	0	0	0	0	0	0	0	0	0
ENSG00000277295	0	0	0	0	0	0	0	0	0	0
ENSG00000277294	0	0	0	0	0	0	0	0	0	0
ENSG00000277290	4	4	5	5	2	16	11	2	0	0
ENSG00000277289	0	0	0	0	0	0	0	0	0	0
ENSG00000277288	0	0	0	0	0	0	1	4	0	0

ENSG00000277287	34	38	48	27	25	64	3	19	0	0
ENSG00000277285	0	0	0	0	0	0	0	0	0	0
ENSG00000277284	0	0	0	0	0	0	0	0	0	0
ENSG00000277283	16	25	14	21	14	60	23	68	0	0
ENSG00000277282	0	0	0	0	0	0	0	0	0	0
ENSG00000277277	2	2	7	1	3	6	6	5	0	0
ENSG00000277275	0	0	0	0	0	0	0	0	0	0
ENSG00000277274	0	0	0	0	0	1	0	0	0	0
ENSG00000277270	1	1	0	0	0	1	2	0	0	0
ENSG00000277269	0	0	0	0	0	0	0	0	0	0
ENSG00000277268	1	0	0	1	0	0	0	0	0	0
ENSG00000277266	0	0	0	0	0	0	0	0	0	0
ENSG00000277265	0	0	0	0	0	0	0	10	0	0
ENSG00000277264	0	0	0	0	0	0	0	0	0	0
ENSG00000277260	0	0	0	0	0	0	0	0	0	0
ENSG00000277259	0	0	0	0	0	0	0	0	0	0
ENSG00000277258	480	592	648	472	393	1295	630	420	4	27
ENSG00000277257	0	0	0	0	0	0	0	0	0	0
ENSG00000277255	0	0	0	0	0	0	6	0	0	0
ENSG00000277253	2	1	3	2	1	14	6	0	0	0
ENSG00000277252	0	0	0	0	0	0	0	0	0	0
ENSG00000277250	0	0	0	0	0	0	0	5	0	0
ENSG00000277249	3	2	1	2	2	4	0	0	0	0
ENSG00000277248	0	0	0	0	0	0	0	0	0	0
ENSG00000277247	0	0	0	0	0	0	0	0	0	0
ENSG00000277246	1	1	0	4	4	3	8	8	0	0
ENSG00000277245	0	0	0	0	0	0	2	0	0	0
ENSG00000277244	0	0	0	0	0	0	3	0	0	0
ENSG00000277243	0	0	0	0	0	0	0	0	0	0
ENSG00000277241	0	0	0	0	0	0	0	0	0	0
ENSG00000277239	0	0	0	0	0	0	0	0	0	0
ENSG00000277237	1	1	1	0	0	1	1	7	0	0
ENSG00000277235	1	0	0	0	0	0	3	2	0	0
ENSG00000277234	0	0	0	0	0	0	0	0	0	0
ENSG00000277233	0	0	0	0	0	0	2	7	0	0
ENSG00000277232	2	3	3	2	0	14	1	4	0	0
ENSG00000277231	0	0	0	0	0	0	0	0	0	0
ENSG00000277230	0	0	0	0	0	0	0	0	0	0
ENSG00000277229	1	0	0	0	0	1	0	0	0	0
ENSG00000277228	0	0	0	0	0	0	1	0	0	0
ENSG00000277227	0	0	0	0	0	0	0	0	0	0
ENSG00000277225	0	0	0	0	0	0	0	0	0	0
ENSG00000277224	5	1	7	5	4	14	3	0	0	0
ENSG00000277223	0	0	0	0	0	0	0	0	0	0
ENSG00000277220	0	2	4	0	0	0	5	11	0	3
ENSG00000277218	1	2	5	1	1	0	1	0	0	0
ENSG00000277217	0	0	0	0	0	0	0	0	0	0
ENSG00000277216	0	0	0	0	0	0	0	0	0	0
ENSG00000277215	0	0	0	0	0	0	0	2	0	0
ENSG00000277214	0	0	0	0	0	0	0	0	0	0
ENSG00000277213	0	0	0	0	0	0	0	0	0	0
ENSG00000277212	0	0	0	0	0	0	0	0	0	0
ENSG00000277211	0	0	0	0	0	0	0	0	0	0
ENSG00000277210	0	0	0	0	0	0	0	9	0	0
ENSG00000277209	0	0	0	0	0	0	0	0	0	0
ENSG00000277208	0	0	0	0	0	0	1	1	0	0
ENSG00000277207	0	0	0	0	0	0	0	0	0	0

ENSG00000277206	0	0	0	0	0	0	0	0	0	0
ENSG00000277204	0	0	0	0	0	0	0	0	0	0
ENSG00000277203	230	191	270	201	226	541	158	118	7	145
ENSG00000277202	0	0	0	0	0	0	0	0	0	0
ENSG00000277201	0	0	0	1	0	0	3	0	0	0
ENSG00000277200	1	2	1	4	1	3	0	7	0	0
ENSG00000277199	0	0	0	0	0	0	1	21	0	0
ENSG00000277198	0	0	0	0	0	0	0	0	0	0
ENSG00000277197	0	0	0	0	0	0	0	0	0	0
ENSG00000277194	0	0	0	0	0	0	0	0	0	0
ENSG00000277192	0	0	0	0	0	0	0	0	0	0
ENSG00000277191	0	0	0	0	0	0	0	0	0	0
ENSG00000277189	0	0	0	0	0	0	0	0	0	0
ENSG00000277188	0	0	0	0	0	0	0	0	0	0
ENSG00000277186	0	0	0	0	0	0	0	26	0	0
ENSG00000277184	0	0	0	0	0	0	0	0	0	0
ENSG00000277182	1	0	7	2	5	5	5	12	0	0
ENSG00000277180	0	0	0	0	0	0	0	0	0	0
ENSG00000277174	0	0	0	0	0	0	0	0	0	0
ENSG00000277173	1	0	0	1	2	0	1	0	0	0
ENSG00000277172	0	0	0	0	0	0	0	0	0	0
ENSG00000277171	0	0	0	0	0	0	0	0	0	0
ENSG00000277170	0	1	2	1	0	1	2	11	0	0
ENSG00000277169	0	0	0	0	0	0	0	0	0	0
ENSG00000277168	0	0	0	0	0	0	0	1	0	0
ENSG00000277166	0	0	0	0	0	0	0	1	0	0
ENSG00000277162	0	0	0	0	0	0	0	0	0	0
ENSG00000277161	43	45	88	39	36	126	330	150	0	3
ENSG00000277160	0	0	0	0	0	0	4	0	0	0
ENSG00000277159	0	0	6	0	1	3	3	5	0	0
ENSG00000277158	0	0	0	0	0	0	0	0	0	0
ENSG00000277157	0	3	11	4	1	11	10	0	0	0
ENSG00000277156	1	0	0	0	0	0	2	19	0	0
ENSG00000277155	0	0	0	0	0	0	0	0	0	0
ENSG00000277152	0	0	0	1	0	0	32	0	4	0
ENSG00000277151	0	0	0	0	0	0	15	18	0	0
ENSG00000277150	107	91	136	104	104	285	107	104	6	125
ENSG00000277149	57	45	77	36	43	138	309	141	0	0
ENSG00000277147	116	125	201	130	104	382	533	264	3	10
ENSG00000277146	0	0	0	0	0	0	0	0	0	0
ENSG00000277144	0	2	2	0	0	0	12	20	0	0
ENSG00000277143	0	0	0	0	0	0	0	0	0	0
ENSG00000277142	1	6	7	4	6	18	8	1	0	0
ENSG00000277141	0	0	0	0	0	0	0	0	0	0
ENSG00000277138	0	0	0	0	0	0	0	0	0	0
ENSG00000277137	0	0	0	0	0	0	0	0	0	0
ENSG00000277136	0	0	0	0	0	0	0	0	0	0
ENSG00000277135	0	0	0	0	0	0	2	39	0	0
ENSG00000277133	0	0	0	0	0	0	0	0	0	0
ENSG00000277130	0	0	0	0	0	0	2	16	3	0
ENSG00000277129	0	0	0	0	0	0	0	0	0	0
ENSG00000277128	0	0	0	0	0	0	0	0	0	0
ENSG00000277127	0	0	0	0	0	0	1	0	0	0
ENSG00000277125	33	46	65	26	39	80	127	55	3	9
ENSG00000277124	0	0	0	0	0	0	0	0	0	0
ENSG00000277123	0	0	0	0	0	0	0	0	0	0
ENSG00000277122	0	0	0	0	0	0	0	0	0	0

ENSG00000277121	0	0	0	0	0	0	0	0	0	0
ENSG00000277120	0	0	0	0	0	0	0	0	0	0
ENSG00000277119	0	0	0	0	0	0	0	0	0	0
ENSG00000277118	0	0	0	0	0	0	0	0	0	0
ENSG00000277117	36	33	29	45	34	81	598	149	0	0
ENSG00000277116	0	0	0	0	0	0	0	0	0	0
ENSG00000277115	0	0	0	0	0	0	0	0	0	0
ENSG00000277112	0	2	2	0	0	0	1	13	0	0
ENSG00000277109	0	0	0	0	0	0	0	0	0	0
ENSG00000277108	0	0	0	0	0	0	0	0	0	0
ENSG00000277105	9	43	13	26	4	13	64079	27330	182	1354
ENSG00000277103	1	0	0	0	0	0	0	0	0	0
ENSG00000277102	0	0	0	0	0	0	0	0	0	0
ENSG00000277100	0	0	0	0	0	0	0	0	0	0
ENSG00000277099	0	0	0	0	0	0	0	0	0	0
ENSG00000277098	0	0	0	0	0	0	0	0	0	0
ENSG00000277097	0	0	0	0	0	0	0	0	0	0
ENSG00000277096	0	0	0	0	0	0	0	0	0	0
ENSG00000277095	0	0	2	0	0	0	2	0	0	0
ENSG00000277089	2	0	0	0	0	0	32	1	0	0
ENSG00000277087	0	0	0	0	0	0	0	12	0	0
ENSG00000277084	0	0	0	0	0	0	0	0	0	0
ENSG00000277083	0	0	0	0	0	0	0	0	0	0
ENSG00000277082	0	0	0	0	0	0	0	0	0	0
ENSG00000277081	0	0	0	0	0	0	0	0	0	0
ENSG00000277077	0	0	0	0	0	0	0	0	0	0
ENSG00000277075	6	8	16	14	8	8	15	1	0	0
ENSG00000277074	0	0	0	0	0	0	0	0	0	0
ENSG00000277073	0	0	0	0	0	0	0	0	0	0
ENSG00000277072	193	192	277	154	191	479	99	90	0	5
ENSG00000277067	405	324	600	253	291	807	154	27	0	0
ENSG00000277066	0	0	0	0	0	0	0	0	0	0
ENSG00000277064	0	0	0	0	0	0	0	0	0	0
ENSG00000277063	0	0	0	0	0	0	0	0	0	0
ENSG00000277062	0	0	0	0	0	0	0	4	0	0
ENSG00000277061	0	0	0	0	0	0	0	0	0	0
ENSG00000277058	0	0	0	0	0	0	0	20	0	0
ENSG00000277057	0	0	0	0	1	0	0	0	0	0
ENSG00000277056	0	0	0	0	0	0	0	0	0	0
ENSG00000277054	0	0	0	0	0	0	0	1	0	0
ENSG00000277053	4285	4233	6281	3393	3968	11153	4424	3536	2	41
ENSG00000277052	0	0	0	0	0	0	0	0	0	0
ENSG00000277051	0	0	0	0	0	1	0	0	0	0
ENSG00000277050	0	0	0	0	0	0	2	2	0	0
ENSG00000277049	0	0	0	0	0	0	1	0	0	0
ENSG00000277047	0	0	0	0	0	0	0	4	0	0
ENSG00000277045	0	0	0	0	0	0	0	0	0	0
ENSG00000277043	0	0	0	0	0	0	0	0	0	0
ENSG00000277041	0	0	0	0	0	0	2	0	0	0
ENSG00000277040	0	0	0	0	0	0	0	0	0	0
ENSG00000277039	0	0	0	1	0	0	1	0	0	0
ENSG00000277035	0	0	0	0	0	0	0	0	0	0
ENSG00000277034	0	0	0	0	0	0	0	0	0	0
ENSG00000277031	0	0	0	0	0	0	0	0	0	0
ENSG00000277030	0	0	0	0	0	0	11	0	0	0
ENSG00000277029	0	0	0	0	0	0	0	1	0	0
ENSG00000277027	0	0	0	0	0	0	0	0	0	0

ENSG00000277026	0	0	0	0	0	0	0	0	0	0
ENSG00000277024	1	0	0	0	0	0	0	10	0	0
ENSG00000277023	0	0	0	0	0	0	0	0	0	0
ENSG00000277022	0	0	2	0	0	2	6	0	0	0
ENSG00000277021	0	0	0	0	0	0	0	2	0	0
ENSG00000277020	1	0	0	0	1	0	2	10	0	0
ENSG00000277017	0	0	0	0	0	0	0	0	0	0
ENSG00000277014	2	1	0	0	5	4	1	0	0	0
ENSG00000277013	5	7	8	5	1	19	26	3	0	0
ENSG00000277011	0	0	0	0	0	3	0	0	0	0
ENSG00000277010	0	0	0	0	0	0	5	32	0	0
ENSG00000277009	0	0	0	0	0	0	0	0	0	0
ENSG00000277008	0	0	0	0	0	0	0	0	0	0
ENSG00000277007	8	2	10	0	4	4	8	20	0	0
ENSG00000277004	0	0	0	0	0	0	0	0	0	0
ENSG00000277003	0	0	0	0	0	0	1	1	0	0
ENSG00000277002	0	0	0	0	0	0	0	0	0	0
ENSG00000277001	0	0	0	0	0	0	0	2	0	0
ENSG00000277000	0	0	0	0	0	0	0	0	0	0
ENSG00000276998	0	0	0	0	0	0	0	1	0	0
ENSG00000276997	37	51	82	31	40	136	28	12	0	4
ENSG00000276995	4	1	0	0	1	0	3	39	0	0
ENSG00000276993	0	0	0	0	0	0	0	0	0	0
ENSG00000276992	0	0	0	0	0	0	0	0	0	0
ENSG00000276991	0	0	0	0	0	0	0	0	0	0
ENSG00000276990	0	0	0	0	0	0	0	0	0	0
ENSG00000276989	0	0	0	0	0	0	0	2	0	0
ENSG00000276988	1	0	0	2	1	1	0	0	0	0
ENSG00000276987	0	0	0	0	0	0	0	0	0	0
ENSG00000276984	0	0	0	2	0	0	0	4	0	0
ENSG00000276981	0	0	0	0	0	0	0	0	0	0
ENSG00000276980	42	52	69	101	110	402	102	11	17	112
ENSG00000276978	0	0	0	0	0	0	0	0	0	0
ENSG00000276975	5	7	3	5	2	17	45	13	0	0
ENSG00000276972	0	0	0	0	0	0	3	0	0	0
ENSG00000276968	1	1	5	1	1	8	9	7	0	0
ENSG00000276967	0	0	0	0	0	0	0	2	0	0
ENSG00000276966	2	1	2	4	2	11	88	0	0	0
ENSG00000276965	0	0	0	0	0	0	0	0	0	0
ENSG00000276964	0	0	0	0	0	0	0	0	0	0
ENSG00000276963	0	0	0	0	0	0	0	0	0	0
ENSG00000276962	0	0	0	0	0	0	0	0	0	0
ENSG00000276961	0	0	0	0	0	0	3	0	0	0
ENSG00000276960	0	0	0	0	0	4	0	0	0	0
ENSG00000276958	0	0	0	0	0	0	0	0	0	0
ENSG00000276957	0	0	0	0	0	0	0	2	0	0
ENSG00000276956	0	0	0	0	0	0	0	1	0	0
ENSG00000276955	0	0	0	0	0	0	0	0	0	0
ENSG00000276953	3	2	0	0	0	2	8	0	0	0
ENSG00000276952	22	31	29	21	28	51	3	8	0	0
ENSG00000276951	0	0	0	0	0	0	2	0	0	0
ENSG00000276950	0	1	0	0	0	0	2	4	0	0
ENSG00000276949	0	0	0	0	0	0	0	0	0	0
ENSG00000276945	2	0	0	0	1	2	2	1	0	0
ENSG00000276943	0	0	0	0	0	0	0	0	0	0
ENSG00000276942	0	0	0	0	0	0	0	2	0	0
ENSG00000276941	0	0	0	0	0	0	0	0	0	0

ENSG00000276940	0	0	0	0	0	0	0	0	0	0
ENSG00000276939	0	0	0	0	0	0	0	0	0	0
ENSG00000276934	0	0	0	2	0	6	9	6	0	7
ENSG00000276933	0	0	0	0	0	0	7	26	2	8
ENSG00000276931	0	3	2	0	0	0	3	0	0	0
ENSG00000276929	0	0	0	0	0	0	0	0	0	0
ENSG00000276928	0	0	0	0	0	0	0	2	0	0
ENSG00000276927	0	0	0	0	0	0	0	0	0	0
ENSG00000276926	1	0	0	2	0	3	2	0	0	0
ENSG00000276925	0	0	0	0	0	2	0	0	0	0
ENSG00000276924	11	20	25	4	6	13	958	590	122	497
ENSG00000276923	0	0	0	0	0	0	0	0	0	0
ENSG00000276922	0	0	0	0	0	0	0	1	0	0
ENSG00000276921	0	0	0	0	0	0	0	0	0	0
ENSG00000276919	0	0	0	0	0	0	0	0	0	0
ENSG00000276918	0	0	0	0	0	0	0	0	0	0
ENSG00000276917	0	0	0	0	0	0	0	0	0	0
ENSG00000276916	4	14	15	2	3	13	10	3	0	0
ENSG00000276915	0	0	0	0	0	0	0	0	0	0
ENSG00000276911	0	0	0	0	0	0	0	0	0	0
ENSG00000276908	0	0	0	0	0	0	0	0	0	0
ENSG00000276905	0	0	0	0	0	0	0	0	0	0
ENSG00000276904	0	0	0	0	0	1	0	0	0	0
ENSG00000276903	0	0	0	0	0	0	3	0	0	0
ENSG00000276902	0	0	0	0	0	0	0	0	0	0
ENSG00000276900	16	24	21	19	7	53	43	26	1	22
ENSG00000276899	0	0	0	0	0	0	1	0	0	0
ENSG00000276898	0	0	0	0	0	0	0	3	0	0
ENSG00000276897	0	0	0	0	0	0	0	0	0	0
ENSG00000276894	0	0	0	0	0	0	0	4	0	0
ENSG00000276893	0	0	0	0	0	0	0	0	0	0
ENSG00000276892	0	0	0	0	0	0	0	0	0	0
ENSG00000276891	0	0	0	0	0	0	0	0	0	0
ENSG00000276890	0	0	0	0	0	0	0	0	0	0
ENSG00000276889	1	0	0	1	0	0	0	0	0	0
ENSG00000276888	0	0	0	0	0	0	0	3	0	0
ENSG00000276884	0	0	0	0	0	0	0	0	0	0
ENSG00000276883	0	0	0	0	0	0	0	0	0	0
ENSG00000276878	0	0	0	0	0	0	0	4	0	0
ENSG00000276877	0	0	0	0	0	0	0	0	0	0
ENSG00000276874	0	0	0	0	0	0	0	0	0	0
ENSG00000276873	0	0	0	0	0	0	0	0	0	0
ENSG00000276871	0	0	0	0	0	0	0	0	0	0
ENSG00000276869	0	0	0	0	0	0	0	0	0	0
ENSG00000276868	0	1	0	0	0	1	1	0	0	0
ENSG00000276867	1	0	2	0	1	3	77	10	0	0
ENSG00000276866	0	0	0	0	0	0	0	0	0	0
ENSG00000276864	0	0	0	0	0	0	0	0	0	0
ENSG00000276863	1	0	0	0	0	0	2	4	0	0
ENSG00000276862	0	0	0	0	0	0	0	0	0	0
ENSG00000276861	0	0	0	0	0	0	0	0	0	0
ENSG00000276859	0	0	0	0	0	0	0	0	0	0
ENSG00000276857	0	0	0	0	0	0	0	0	0	0
ENSG00000276855	3	8	8	4	1	15	194	174	0	24
ENSG00000276854	0	0	0	0	0	0	0	0	0	0
ENSG00000276853	0	0	6	0	0	0	11	17	0	0
ENSG00000276852	0	0	0	0	0	0	0	0	0	0

ENSG00000276851	18	16	35	17	13	47	19	3	0	0
ENSG00000276850	731	786	1324	648	614	1780	153	142	0	0
ENSG00000276846	2	2	1	0	3	1	3	11	0	0
ENSG00000276845	0	0	2	0	0	0	0	0	0	0
ENSG00000276844	0	0	0	0	0	0	0	0	0	0
ENSG00000276843	0	0	0	0	0	0	0	0	0	0
ENSG00000276842	0	0	0	0	0	0	1	0	0	0
ENSG00000276841	0	0	0	0	0	0	0	0	0	0
ENSG00000276840	25	32	34	22	22	54	19	25	0	0
ENSG00000276839	0	0	0	0	0	0	1	0	0	0
ENSG00000276837	0	0	0	0	0	0	0	0	0	0
ENSG00000276835	0	0	0	0	0	0	0	0	0	0
ENSG00000276832	0	2	6	1	1	4	0	2	0	0
ENSG00000276831	1	0	4	1	0	1	2	0	0	0
ENSG00000276830	0	0	0	0	0	0	1	0	0	0
ENSG00000276829	0	0	0	0	0	0	0	5	0	0
ENSG00000276828	0	0	0	0	0	0	0	0	0	0
ENSG00000276827	0	0	0	0	0	0	0	0	0	0
ENSG00000276824	0	0	0	0	0	0	0	0	0	0
ENSG00000276823	1	0	1	0	2	0	1	1	0	7
ENSG00000276822	0	0	0	0	0	0	0	1	0	0
ENSG00000276819	0	0	0	0	0	0	2	0	0	0
ENSG00000276818	0	0	0	0	0	0	0	0	0	0
ENSG00000276817	0	0	0	0	0	0	0	0	0	0
ENSG00000276815	0	0	0	0	0	0	0	0	0	0
ENSG00000276814	0	0	1	2	0	6	20	14	0	0
ENSG00000276813	0	0	0	0	0	0	0	0	0	0
ENSG00000276812	0	0	0	0	0	0	0	0	0	0
ENSG00000276811	0	0	0	0	0	0	0	0	0	0
ENSG00000276810	0	0	0	0	0	2	0	4	0	0
ENSG00000276809	1	0	0	0	2	0	16	8	0	0
ENSG00000276807	0	0	0	0	0	0	0	0	0	0
ENSG00000276805	2	1	3	3	1	1	2	3	0	0
ENSG00000276803	0	0	0	0	0	0	0	4	0	0
ENSG00000276801	0	0	0	0	0	0	0	0	0	0
ENSG00000276800	0	0	0	0	0	0	0	0	0	0
ENSG00000276797	0	0	2	0	0	2	4	0	0	0
ENSG00000276796	0	0	0	0	0	0	66	12	0	2
ENSG00000276795	1	0	0	0	0	0	0	0	0	0
ENSG00000276793	0	0	0	0	0	0	0	0	0	0
ENSG00000276792	0	0	0	0	0	0	0	0	0	0
ENSG00000276791	3	2	0	4	3	2	16	7	0	0
ENSG00000276790	0	0	0	0	1	0	10	0	0	0
ENSG00000276789	0	0	0	0	0	0	0	0	0	0
ENSG00000276788	0	0	0	0	0	0	0	0	0	0
ENSG00000276786	0	0	0	0	0	0	0	0	0	0
ENSG00000276784	0	0	0	0	0	0	0	0	0	0
ENSG00000276783	0	0	0	0	0	0	0	0	0	0
ENSG00000276782	0	0	0	0	0	0	0	0	0	0
ENSG00000276778	0	0	0	0	0	0	1	1	0	0
ENSG00000276775	0	0	0	0	0	0	0	0	0	0
ENSG00000276773	0	0	0	0	0	0	0	0	0	0
ENSG00000276772	0	0	0	0	0	7	107	4	0	0
ENSG00000276771	0	0	0	0	0	0	0	0	0	0
ENSG00000276770	0	0	0	0	0	2	0	0	0	0
ENSG00000276769	0	0	0	0	0	0	0	0	0	0
ENSG00000276768	1	0	1	0	1	0	2	1	0	0

ENSG00000276765	0	0	0	0	0	0	2	0	0	0
ENSG00000276764	0	0	0	0	0	0	0	0	0	0
ENSG00000276763	2	0	2	0	0	0	0	0	0	0
ENSG00000276762	0	0	0	0	0	0	0	0	0	0
ENSG00000276759	0	0	0	0	0	0	0	0	0	0
ENSG00000276758	0	0	0	0	0	0	42	0	0	0
ENSG00000276757	0	0	0	1	1	3	2	0	0	0
ENSG00000276756	6	5	13	0	1	4	5	7	0	0
ENSG00000276755	0	0	0	0	0	0	3	0	0	0
ENSG00000276754	0	0	0	0	0	0	0	0	0	0
ENSG00000276753	0	0	0	0	0	0	0	0	0	0
ENSG00000276752	0	0	0	0	0	0	0	0	0	0
ENSG00000276751	0	1	2	0	0	0	0	0	0	0
ENSG00000276750	0	0	0	0	0	0	0	0	0	0
ENSG00000276748	0	0	0	0	0	0	0	0	0	0
ENSG00000276747	1	0	0	0	0	0	0	0	0	0
ENSG00000276746	0	0	0	0	0	0	0	0	0	0
ENSG00000276745	0	0	0	0	0	0	0	0	0	0
ENSG00000276744	1	3	2	1	0	0	9	4	0	0
ENSG00000276743	0	0	0	0	0	0	0	0	0	0
ENSG00000276742	0	1	3	0	1	3	0	0	0	0
ENSG00000276741	0	0	0	0	0	0	0	0	0	0
ENSG00000276740	0	0	0	0	0	0	7	13	0	0
ENSG00000276738	0	0	0	0	0	0	0	0	0	0
ENSG00000276737	0	0	0	0	0	0	218	14	1	0
ENSG00000276735	1	0	0	0	0	0	1	1	0	0
ENSG00000276733	0	0	0	0	0	0	0	0	0	0
ENSG00000276730	0	0	0	0	0	0	0	0	0	0
ENSG00000276729	0	0	0	0	0	0	0	0	0	0
ENSG00000276728	8	9	9	11	6	11	18	52	0	0
ENSG00000276727	0	0	0	0	0	0	0	0	0	0
ENSG00000276724	1	3	9	3	2	0	21	42	0	0
ENSG00000276723	0	0	0	0	0	0	0	0	0	0
ENSG00000276722	0	0	0	0	0	0	0	0	0	0
ENSG00000276721	0	0	0	0	0	0	3	0	0	0
ENSG00000276718	1	0	0	1	2	0	14	15	0	0
ENSG00000276717	0	0	0	0	0	0	0	0	0	0
ENSG00000276715	0	0	0	0	0	0	3	5	0	0
ENSG00000276713	0	0	0	0	0	0	0	0	0	0
ENSG00000276712	2	8	5	6	1	30	20	39	0	0
ENSG00000276710	110	156	162	113	97	321	42	7	0	0
ENSG00000276707	0	0	0	0	0	0	0	0	0	0
ENSG00000276706	0	0	0	0	0	0	0	0	0	0
ENSG00000276704	0	0	0	0	0	0	3	6	0	3
ENSG00000276703	0	0	0	0	0	0	0	0	0	0
ENSG00000276702	0	0	0	0	0	0	3	2	0	0
ENSG00000276699	0	0	0	0	0	0	0	0	0	0
ENSG00000276698	6	0	3	3	2	6	13	4	0	0
ENSG00000276696	0	0	0	0	0	0	0	0	0	0
ENSG00000276695	0	0	0	0	0	0	0	0	0	0
ENSG00000276694	0	0	0	0	0	0	0	0	0	0
ENSG00000276693	0	0	0	0	0	0	0	0	0	0
ENSG00000276692	30	21	19	13	10	33	23	29	0	0
ENSG00000276691	1	0	0	0	0	0	5	0	0	0
ENSG00000276690	0	0	0	0	0	0	0	0	0	0
ENSG00000276689	0	0	0	0	0	0	1	0	0	0
ENSG00000276688	0	0	0	0	0	0	0	0	0	0

ENSG00000276687	0	0	0	0	0	0	0	0	0	0
ENSG00000276685	0	0	0	0	0	0	0	0	0	0
ENSG00000276683	0	0	0	0	0	0	0	0	0	0
ENSG00000276682	0	0	0	0	0	0	0	0	0	0
ENSG00000276680	1	0	0	0	0	2	0	0	0	0
ENSG00000276678	0	0	0	0	0	0	0	0	0	0
ENSG00000276677	0	0	0	0	0	0	0	0	0	0
ENSG00000276675	0	0	0	0	0	0	0	0	0	0
ENSG00000276674	1	0	0	0	0	0	12	7	0	0
ENSG00000276673	0	0	0	0	0	0	0	0	0	0
ENSG00000276672	1	6	3	3	3	0	1	31	0	1
ENSG00000276670	0	0	0	0	0	0	0	0	0	0
ENSG00000276669	0	0	0	0	0	0	0	0	0	0
ENSG00000276668	2	4	3	2	1	2	2	0	0	0
ENSG00000276667	0	0	0	0	0	0	0	0	0	0
ENSG00000276664	0	0	0	0	0	0	0	0	0	0
ENSG00000276663	0	2	2	0	0	2	6	4	0	0
ENSG00000276662	0	0	0	1	0	0	4	0	0	0
ENSG00000276661	0	0	0	0	0	0	0	0	0	0
ENSG00000276660	0	0	0	0	0	0	0	0	0	0
ENSG00000276659	0	0	0	0	0	0	0	0	0	0
ENSG00000276656	0	0	0	0	0	0	0	0	0	0
ENSG00000276653	0	0	0	0	0	0	0	0	0	0
ENSG00000276652	0	0	0	0	0	0	0	0	0	0
ENSG00000276651	0	0	0	0	2	0	7	12	0	0
ENSG00000276650	0	0	0	0	0	0	0	0	0	0
ENSG00000276649	10	7	7	6	7	20	27	18	0	3
ENSG00000276648	0	0	0	0	0	0	0	0	0	0
ENSG00000276647	0	0	0	0	0	0	0	0	0	0
ENSG00000276645	0	0	1	0	0	0	4	2	0	0
ENSG00000276644	0	0	0	0	0	0	41	308	0	0
ENSG00000276643	0	0	0	0	0	0	0	0	0	0
ENSG00000276642	0	0	0	0	0	0	0	0	0	0
ENSG00000276641	0	0	0	0	0	0	0	0	0	0
ENSG00000276639	0	0	0	0	0	0	0	0	0	0
ENSG00000276638	0	0	0	0	0	0	0	0	0	0
ENSG00000276637	0	0	0	0	0	0	0	0	0	0
ENSG00000276635	0	0	0	0	0	0	0	0	0	0
ENSG00000276633	0	0	0	0	0	0	0	5	0	0
ENSG00000276632	0	0	0	0	0	0	0	0	0	0
ENSG00000276631	0	0	0	0	0	0	0	0	0	0
ENSG00000276630	0	0	0	0	0	0	0	0	0	0
ENSG00000276629	0	1	0	0	0	2	1	0	0	0
ENSG00000276627	0	0	0	0	0	0	0	0	0	0
ENSG00000276626	0	0	0	0	0	0	0	0	0	0
ENSG00000276624	0	0	0	0	0	0	0	0	0	0
ENSG00000276623	0	0	0	0	0	0	0	0	0	0
ENSG00000276622	0	0	0	0	0	0	0	0	0	0
ENSG00000276621	0	0	0	0	0	0	0	3	2	0
ENSG00000276620	0	0	0	0	0	0	0	0	0	0
ENSG00000276619	0	0	0	0	0	0	0	0	0	0
ENSG00000276617	0	0	0	0	0	0	0	0	0	0
ENSG00000276614	0	0	0	0	0	0	0	0	0	0
ENSG00000276612	0	0	0	0	0	0	0	0	0	0
ENSG00000276610	0	0	0	0	0	0	0	0	0	0
ENSG00000276609	0	0	0	0	0	0	0	0	0	0
ENSG00000276607	0	0	0	0	0	0	1	0	0	0

ENSG00000276605	0	0	0	0	0	0	1	0	0	0
ENSG00000276603	1	5	1	5	0	3	8	4	0	5
ENSG00000276602	0	0	0	0	0	0	0	0	0	0
ENSG00000276601	0	0	0	0	0	0	0	0	0	0
ENSG00000276600	788	777	1184	482	480	1495	277	184	0	0
ENSG00000276599	0	0	0	0	0	0	0	0	0	0
ENSG00000276598	0	0	0	0	0	0	0	0	0	0
ENSG00000276597	0	0	0	0	0	0	1	0	0	0
ENSG00000276596	0	1	5	6	1	11	245	20	17	293
ENSG00000276593	0	0	0	0	0	0	0	5	0	0
ENSG00000276592	1	2	1	0	1	1	5	0	0	0
ENSG00000276591	0	0	0	0	0	0	3	1	0	0
ENSG00000276588	0	0	0	0	0	0	0	0	0	0
ENSG00000276587	0	0	0	0	0	0	0	0	0	0
ENSG00000276586	0	0	0	0	0	0	0	0	0	0
ENSG00000276584	0	0	0	0	0	0	0	3	0	0
ENSG00000276581	0	0	0	0	0	0	0	0	0	0
ENSG00000276580	0	4	1	1	1	3	2	0	0	0
ENSG00000276578	2	1	1	2	1	3	3	0	0	0
ENSG00000276577	0	0	0	0	0	0	0	0	0	0
ENSG00000276576	0	0	0	0	0	0	0	3	0	0
ENSG00000276575	1	0	0	0	0	0	2	0	0	0
ENSG00000276574	0	0	0	0	0	0	0	0	0	0
ENSG00000276573	4	1	0	1	0	0	0	6	0	0
ENSG00000276571	0	1	4	1	1	2	1	2	0	0
ENSG00000276570	1	0	1	0	1	2	8	6	0	0
ENSG00000276569	0	0	0	0	0	0	0	0	0	0
ENSG00000276568	0	0	0	0	0	0	0	0	0	0
ENSG00000276566	0	0	0	0	0	0	2	351	0	0
ENSG00000276564	3	2	3	0	4	3	27	66	0	0
ENSG00000276563	0	0	0	0	0	0	1	0	0	0
ENSG00000276562	0	0	0	0	0	0	0	0	0	0
ENSG00000276558	0	0	0	0	0	0	0	0	0	0
ENSG00000276557	0	0	0	0	0	0	28	0	0	0
ENSG00000276556	0	0	0	0	0	0	0	0	0	0
ENSG00000276555	0	0	1	0	0	2	0	40	0	0
ENSG00000276554	0	0	0	0	0	0	0	0	0	0
ENSG00000276550	1082	1155	1620	960	1016	3054	2091	816	2	14
ENSG00000276549	0	0	0	0	0	0	0	0	0	0
ENSG00000276548	0	0	0	0	0	0	0	0	0	0
ENSG00000276547	113	90	159	58	78	212	43	46	0	8
ENSG00000276546	0	0	0	0	0	0	0	0	0	0
ENSG00000276545	0	1	0	0	0	0	5	2	0	0
ENSG00000276544	0	0	1	0	0	0	0	8	0	0
ENSG00000276543	0	0	0	0	0	0	0	0	0	0
ENSG00000276542	0	2	0	0	2	0	0	5	0	0
ENSG00000276540	0	0	0	0	0	0	0	0	0	0
ENSG00000276538	0	0	0	0	0	0	0	0	0	0
ENSG00000276535	0	0	0	0	0	0	0	2	0	0
ENSG00000276533	0	0	0	0	0	0	127	65	0	6
ENSG00000276532	0	0	0	0	0	0	1	4	0	5
ENSG00000276531	0	0	0	0	0	0	0	0	0	0
ENSG00000276530	0	0	0	0	0	0	0	0	0	0
ENSG00000276529	23	25	33	22	26	60	12	21	0	0
ENSG00000276528	0	0	0	0	0	0	0	0	0	0
ENSG00000276527	1	0	1	0	1	2	0	0	0	0
ENSG00000276525	0	0	0	0	0	0	0	0	0	0

ENSG00000276524	0	0	0	0	0	0	4	3	0	0
ENSG00000276523	4	1	5	4	2	7	5	0	0	4
ENSG00000276522	0	0	0	0	0	0	0	0	0	0
ENSG00000276521	0	0	0	0	0	0	1	0	0	0
ENSG00000276520	0	0	0	0	0	0	0	0	0	0
ENSG00000276519	0	0	0	0	0	0	0	0	0	0
ENSG00000276517	0	0	3	2	1	6	35	60	0	0
ENSG00000276515	0	0	0	0	0	0	0	0	0	0
ENSG00000276514	0	0	0	0	0	0	0	0	0	0
ENSG00000276513	0	0	0	0	0	0	0	0	0	0
ENSG00000276511	0	0	0	0	0	0	0	0	0	0
ENSG00000276510	0	0	0	0	0	0	0	0	0	0
ENSG00000276509	5	5	5	5	2	11	15	46	0	0
ENSG00000276508	0	0	0	0	0	0	2	0	0	0
ENSG00000276507	0	0	0	0	0	0	0	9	0	0
ENSG00000276506	0	0	0	0	0	0	0	0	0	0
ENSG00000276505	0	0	1	0	0	0	0	2	0	0
ENSG00000276502	0	0	0	0	0	0	0	0	0	0
ENSG00000276500	1	3	3	1	2	2	2	2	1	0
ENSG00000276499	0	0	0	0	0	0	0	0	0	0
ENSG00000276496	0	0	0	0	0	0	0	0	0	0
ENSG00000276494	0	0	0	0	0	0	0	0	0	0
ENSG00000276493	0	0	0	0	0	0	0	0	0	0
ENSG00000276490	0	0	0	0	0	0	0	0	0	0
ENSG00000276489	0	0	0	0	0	0	0	0	0	0
ENSG00000276488	3	4	4	6	12	22	0	6	0	0
ENSG00000276487	0	0	0	0	0	0	0	0	0	0
ENSG00000276486	0	0	0	0	0	0	0	0	0	0
ENSG00000276484	0	0	0	0	0	0	1	0	0	0
ENSG00000276483	70	62	156	68	79	165	152	142	0	0
ENSG00000276479	0	0	0	0	0	0	0	0	0	0
ENSG00000276478	0	0	0	0	0	0	0	0	0	0
ENSG00000276477	0	0	0	0	0	0	0	0	0	0
ENSG00000276476	4	5	4	1	4	1	11	6	0	0
ENSG00000276475	0	0	0	0	0	0	0	0	0	0
ENSG00000276474	0	0	0	0	0	0	0	0	0	0
ENSG00000276473	0	0	0	0	0	0	0	0	0	0
ENSG00000276472	0	0	0	0	0	0	0	0	0	0
ENSG00000276471	0	0	0	0	0	0	0	0	0	0
ENSG00000276470	0	0	0	0	0	0	0	0	0	0
ENSG00000276467	0	0	0	0	0	0	0	0	0	0
ENSG00000276462	2	0	0	1	7	0	1039	37	0	0
ENSG00000276460	0	0	0	0	0	0	0	0	3	0
ENSG00000276457	0	0	0	0	0	0	0	0	0	0
ENSG00000276456	0	0	0	0	0	0	0	0	0	0
ENSG00000276454	0	0	2	0	0	5	2	0	0	0
ENSG00000276449	2	6	2	3	1	6	5	30	0	0
ENSG00000276445	1	1	2	0	5	14	32	1	0	0
ENSG00000276444	0	0	0	0	0	0	0	0	0	0
ENSG00000276443	0	0	0	0	0	0	0	0	0	0
ENSG00000276442	0	0	0	0	0	0	0	0	0	0
ENSG00000276436	0	1	0	0	0	0	0	0	0	0
ENSG00000276434	1	1	2	0	0	1	0	0	0	0
ENSG00000276431	0	0	0	0	0	0	0	0	0	0
ENSG00000276430	1	1	8	2	9	11	1	0	0	0
ENSG00000276428	0	0	0	0	0	0	0	0	0	0
ENSG00000276427	0	0	0	0	0	0	1	0	0	0

ENSG00000276426	0	0	0	0	0	0	0	0	0	0
ENSG00000276422	0	0	0	0	0	0	1	5	0	0
ENSG00000276419	0	0	0	0	0	0	0	0	0	0
ENSG00000276418	0	0	0	0	0	0	0	0	0	0
ENSG00000276417	0	0	4	0	1	0	2	0	0	0
ENSG00000276413	0	0	0	0	0	0	17	0	0	0
ENSG00000276412	0	0	0	0	0	0	0	0	0	0
ENSG00000276411	0	0	0	0	0	0	0	0	0	0
ENSG00000276410	0	0	0	0	0	0	5	0	0	0
ENSG00000276409	0	0	0	0	0	0	4	86	0	0
ENSG00000276408	0	0	5	0	0	4	4	4	0	0
ENSG00000276407	0	0	0	0	0	0	0	0	0	0
ENSG00000276406	0	0	0	0	0	0	0	0	0	0
ENSG00000276405	0	0	0	2	0	0	6	1	0	0
ENSG00000276404	0	0	0	0	0	2	5	0	0	0
ENSG00000276403	0	0	0	0	0	0	0	2	0	0
ENSG00000276401	0	0	0	0	0	0	0	0	0	0
ENSG00000276400	0	0	0	0	0	0	0	0	0	0
ENSG00000276399	0	0	0	0	0	0	0	0	0	0
ENSG00000276398	0	0	0	0	0	0	0	0	0	0
ENSG00000276397	0	0	0	0	0	0	0	0	0	0
ENSG00000276396	0	0	0	0	0	0	6	0	0	0
ENSG00000276393	0	0	0	0	0	0	0	0	0	0
ENSG00000276391	0	0	0	0	0	0	0	0	0	0
ENSG00000276390	0	0	0	1	1	0	5	24	0	0
ENSG00000276389	0	0	0	0	0	0	0	0	0	0
ENSG00000276388	0	0	0	0	0	0	0	0	0	0
ENSG00000276386	50	49	73	35	37	113	16	169	0	0
ENSG00000276385	0	0	0	0	0	0	0	0	0	0
ENSG00000276384	1	1	1	0	1	1	3	9	0	0
ENSG00000276382	0	3	0	0	0	4	8	1	0	0
ENSG00000276381	0	0	0	0	0	0	6	3	0	0
ENSG00000276380	0	0	0	0	0	0	0	0	0	0
ENSG00000276376	0	0	0	0	0	0	0	0	0	0
ENSG00000276375	0	1	0	0	1	0	0	26	1	2
ENSG00000276373	0	0	0	0	0	0	0	0	0	0
ENSG00000276372	0	0	0	0	0	0	0	0	0	0
ENSG00000276369	0	0	0	0	0	0	0	0	0	0
ENSG00000276368	5	5	1	4	1	5	3	0	0	0
ENSG00000276366	0	0	0	0	0	0	0	0	0	0
ENSG00000276365	0	0	0	0	0	0	0	0	0	0
ENSG00000276364	0	0	0	0	0	0	0	0	0	0
ENSG00000276362	0	0	0	0	0	0	0	0	0	0
ENSG00000276359	0	0	0	0	0	0	0	0	0	0
ENSG00000276354	2	0	0	3	0	1	8	0	0	0
ENSG00000276353	0	0	0	0	0	0	0	0	0	0
ENSG00000276352	0	0	0	0	0	0	0	0	0	0
ENSG00000276350	0	0	0	0	0	0	0	0	0	0
ENSG00000276348	1	0	0	0	0	0	0	6	0	0
ENSG00000276347	0	0	0	0	0	0	0	0	0	0
ENSG00000276343	0	0	0	0	0	0	13	6	1	16
ENSG00000276338	1	0	1	2	0	2	3	36	0	0
ENSG00000276337	0	0	3	3	0	2	29	15	0	0
ENSG00000276335	18	8	25	20	19	34	0	0	0	0
ENSG00000276334	7	9	10	1	3	3	49	25	0	0
ENSG00000276332	0	0	0	0	0	0	0	0	0	0
ENSG00000276331	0	0	0	0	0	0	0	0	0	0

ENSG00000276326	0	0	0	0	1	0	0	0	0	0
ENSG00000276325	1	0	0	1	0	0	4	0	0	0
ENSG00000276322	0	0	0	0	0	0	7	21	0	0
ENSG00000276319	0	0	0	0	0	0	0	0	0	0
ENSG00000276318	0	0	0	0	0	0	0	0	0	0
ENSG00000276317	0	1	1	0	1	0	16	19	0	0
ENSG00000276316	0	0	0	0	0	0	0	0	0	0
ENSG00000276315	0	0	0	0	0	0	0	0	0	0
ENSG00000276314	0	0	0	0	0	0	0	0	0	0
ENSG00000276311	0	0	0	0	0	0	0	0	0	0
ENSG00000276309	0	0	0	0	0	0	0	0	0	0
ENSG00000276308	0	0	0	0	0	0	9	0	0	0
ENSG00000276305	0	0	1	0	0	2	0	37	0	0
ENSG00000276304	0	0	0	0	0	0	0	1	0	0
ENSG00000276302	0	1	0	0	1	1	1	0	0	0
ENSG00000276301	0	0	0	0	0	0	0	0	0	0
ENSG00000276298	0	0	0	0	0	0	1	0	0	0
ENSG00000276294	0	0	0	0	0	0	0	0	0	0
ENSG00000276293	720	564	1050	581	621	1687	1006	1247	4	17
ENSG00000276292	0	0	3	0	0	0	2	0	0	0
ENSG00000276291	45	46	108	44	34	127	108	99	0	0
ENSG00000276290	0	0	0	0	0	0	0	0	0	0
ENSG00000276289	0	0	0	0	0	0	60	18	1	12
ENSG00000276288	5	1	1	1	2	7	2	5	0	0
ENSG00000276284	0	0	0	0	0	0	0	0	0	0
ENSG00000276283	0	0	0	0	0	0	0	3	0	0
ENSG00000276282	0	0	0	0	0	0	4	4	0	0
ENSG00000276281	0	0	0	0	0	0	0	0	0	0
ENSG00000276280	0	0	0	0	0	0	0	0	0	0
ENSG00000276278	0	0	0	0	0	0	0	0	0	0
ENSG00000276277	0	0	0	0	0	0	0	0	0	0
ENSG00000276273	0	0	0	0	0	0	0	0	0	0
ENSG00000276272	1	0	0	0	0	0	2	0	0	0
ENSG00000276271	0	0	0	0	0	0	0	0	0	0
ENSG00000276270	0	0	0	0	0	0	0	0	0	0
ENSG00000276269	0	0	0	0	0	0	0	17	0	0
ENSG00000276268	0	0	0	0	0	0	1	0	0	0
ENSG00000276266	0	4	7	1	0	13	11	0	0	0
ENSG00000276261	3	2	9	2	3	8	18	25	0	0
ENSG00000276259	1	3	0	0	2	2	32	38	0	0
ENSG00000276257	0	0	0	0	0	0	1	0	0	0
ENSG00000276255	0	2	2	0	1	0	3	0	0	0
ENSG00000276252	2	4	4	2	0	2	0	0	0	0
ENSG00000276251	0	0	2	0	0	0	0	5	0	0
ENSG00000276250	0	1	0	0	0	0	8	2	0	0
ENSG00000276248	18	22	52	22	16	34	33	10	0	0
ENSG00000276246	0	0	0	0	0	0	0	0	0	0
ENSG00000276244	0	0	0	0	0	0	0	0	0	0
ENSG00000276242	0	0	0	0	0	0	0	0	0	0
ENSG00000276241	5	1	3	2	1	21	1	6	0	0
ENSG00000276240	0	0	0	0	0	0	0	0	0	0
ENSG00000276237	0	0	0	0	0	0	0	2	0	0
ENSG00000276236	0	0	0	0	0	0	0	0	0	0
ENSG00000276235	0	0	0	0	0	0	0	0	0	0
ENSG00000276234	170	174	257	137	170	434	118	108	0	8
ENSG00000276233	0	0	0	0	0	0	0	0	0	0
ENSG00000276232	0	0	0	0	0	0	0	0	0	0

ENSG00000276231	0	0	0	0	0	0	128	24	0	0
ENSG00000276229	0	0	0	0	0	0	0	0	0	0
ENSG00000276225	0	0	0	0	0	0	0	0	0	0
ENSG00000276223	0	0	0	0	0	0	2	2	0	0
ENSG00000276221	0	0	0	0	0	0	0	0	0	0
ENSG00000276216	0	0	3	0	0	2	10	0	0	0
ENSG00000276215	0	0	0	0	0	0	0	0	0	0
ENSG00000276214	0	0	0	0	2	0	2	0	0	0
ENSG00000276213	0	0	0	0	0	0	0	0	0	0
ENSG00000276210	0	0	0	0	0	0	0	0	0	0
ENSG00000276208	0	0	0	0	0	0	0	0	0	0
ENSG00000276206	0	0	0	0	0	0	0	0	0	0
ENSG00000276205	0	0	0	0	0	0	0	0	0	0
ENSG00000276204	0	0	0	0	0	0	0	0	0	0
ENSG00000276203	4	1	3	0	1	5	29	33	0	0
ENSG00000276202	0	0	0	0	0	0	0	6	0	0
ENSG00000276201	0	0	0	0	0	0	0	0	0	0
ENSG00000276200	0	0	0	0	0	0	0	0	0	0
ENSG00000276199	0	0	0	0	0	0	0	0	0	0
ENSG00000276198	0	0	0	0	0	0	0	0	0	0
ENSG00000276195	0	0	0	0	0	0	0	0	0	0
ENSG00000276193	0	0	0	0	0	0	0	0	0	0
ENSG00000276190	0	0	0	0	0	0	0	0	0	0
ENSG00000276189	0	0	0	0	0	0	0	0	0	0
ENSG00000276188	2	0	0	0	0	0	7	3	0	0
ENSG00000276185	0	0	0	0	0	0	0	0	0	0
ENSG00000276183	0	0	0	0	0	0	0	0	0	0
ENSG00000276182	1	0	0	0	0	0	13	7	0	5
ENSG00000276181	0	0	0	0	0	0	0	0	0	0
ENSG00000276180	56	59	85	35	56	135	520	99	0	31
ENSG00000276179	0	0	0	0	0	0	1	0	0	0
ENSG00000276178	2	2	7	1	0	2	3	2	0	0
ENSG00000276176	0	0	0	0	0	0	2	0	0	0
ENSG00000276174	0	0	0	0	0	3	5	1	0	0
ENSG00000276172	0	2	0	1	0	0	1	5	0	0
ENSG00000276171	680	680	928	488	631	2185	4149	56342	4	14
ENSG00000276170	143	125	170	101	89	318	88	33	0	0
ENSG00000276169	0	0	0	0	0	0	0	0	0	0
ENSG00000276168	0	0	0	0	0	0	0	0	0	0
ENSG00000276166	1	1	1	0	5	12	15	0	0	0
ENSG00000276165	0	0	0	0	0	0	0	0	0	0
ENSG00000276164	0	0	0	0	0	1	0	0	0	0
ENSG00000276162	0	0	0	0	0	0	0	0	0	0
ENSG00000276161	0	0	0	0	0	0	0	0	0	0
ENSG00000276160	1	2	0	0	2	3	39	19	0	0
ENSG00000276158	0	0	0	0	0	0	0	0	0	0
ENSG00000276157	0	0	0	0	0	0	0	0	0	0
ENSG00000276156	0	0	0	0	0	0	0	0	0	0
ENSG00000276151	0	0	0	0	0	0	0	0	0	0
ENSG00000276150	0	0	0	0	0	0	0	0	0	0
ENSG00000276149	0	0	0	0	0	0	0	0	0	0
ENSG00000276148	3	2	1	0	1	1	45	22	0	0
ENSG00000276147	0	0	0	0	0	0	0	0	0	0
ENSG00000276144	0	0	0	0	0	0	0	0	0	0
ENSG00000276141	55	40	81	26	59	108	177	179	0	0
ENSG00000276140	0	0	0	0	0	0	0	0	0	0
ENSG00000276138	0	0	0	0	0	0	0	0	0	0

ENSG00000276137	0	0	0	0	0	0	0	0	0	0
ENSG00000276136	2	2	2	2	0	1	14	7	0	7
ENSG00000276135	48	58	77	31	53	117	6	0	0	0
ENSG00000276131	0	0	0	0	0	0	0	0	0	0
ENSG00000276128	0	0	0	1	0	0	5	9	0	0
ENSG00000276127	0	0	0	1	0	0	2	12	0	0
ENSG00000276124	0	0	1	0	0	0	0	0	0	0
ENSG00000276123	0	0	0	0	0	0	0	0	0	0
ENSG00000276122	0	0	0	0	0	0	0	1	0	0
ENSG00000276121	0	0	0	0	0	0	40	0	0	0
ENSG00000276119	0	1	0	0	0	0	0	0	0	0
ENSG00000276118	0	0	0	0	0	0	0	0	0	0
ENSG00000276117	0	0	0	0	0	0	0	2	0	0
ENSG00000276116	0	0	0	2	0	0	2	2	0	0
ENSG00000276115	2	2	1	0	1	2	12	5	0	0
ENSG00000276110	0	0	2	0	0	0	0	3	0	0
ENSG00000276109	0	0	0	0	0	2	0	0	0	0
ENSG00000276108	0	0	0	0	0	0	0	0	0	0
ENSG00000276107	24	24	36	23	37	66	52	4	0	0
ENSG00000276106	0	0	0	0	0	0	0	0	0	0
ENSG00000276105	0	0	0	0	0	0	0	0	0	0
ENSG00000276104	0	0	0	0	0	0	140	2	0	0
ENSG00000276103	0	1	0	0	0	2	0	0	0	0
ENSG00000276102	0	0	1	0	0	0	1	0	0	0
ENSG00000276101	0	0	0	0	0	0	0	0	0	0
ENSG00000276100	0	0	0	0	0	0	0	0	0	0
ENSG00000276098	0	0	0	0	0	0	0	0	0	0
ENSG00000276097	0	0	0	0	0	0	0	0	0	0
ENSG00000276096	2	2	0	2	1	0	0	0	0	0
ENSG00000276095	0	0	0	0	0	0	0	0	0	0
ENSG00000276094	0	0	0	0	0	0	0	0	0	0
ENSG00000276093	0	0	0	0	0	0	0	7	0	0
ENSG00000276092	0	0	0	0	0	0	0	4	0	0
ENSG00000276090	0	0	0	0	0	0	0	0	0	0
ENSG00000276089	0	0	0	0	0	0	0	0	0	0
ENSG00000276088	0	0	0	0	0	0	0	3	0	0
ENSG00000276087	0	0	0	0	0	0	0	0	0	0
ENSG00000276085	0	0	0	0	0	0	3029	23	1124	14562
ENSG00000276083	0	0	0	0	0	0	0	0	0	0
ENSG00000276081	1	0	0	0	0	4	0	2	0	0
ENSG00000276079	0	0	0	0	0	0	0	0	0	0
ENSG00000276078	0	0	0	0	0	0	0	0	0	0
ENSG00000276077	405	324	604	255	291	812	162	29	0	0
ENSG00000276076	0	0	0	0	0	0	0	0	0	0
ENSG00000276075	0	0	0	2	0	3	7	9	0	0
ENSG00000276074	0	0	0	0	0	0	0	0	0	0
ENSG00000276073	0	3	6	0	4	2	19	6	0	0
ENSG00000276071	2	5	3	3	5	3	5	0	0	0
ENSG00000276070	0	0	0	0	0	0	2081	88	970	19472
ENSG00000276067	0	0	4	0	0	0	0	0	0	0
ENSG00000276064	0	0	0	0	0	0	0	0	0	0
ENSG00000276063	0	0	0	0	0	0	0	0	0	0
ENSG00000276059	2	0	1	0	1	0	0	0	0	0
ENSG00000276058	2	0	0	0	0	0	7	0	0	0
ENSG00000276057	0	0	0	0	0	0	0	0	0	0
ENSG00000276056	0	0	0	0	0	0	0	0	0	0
ENSG00000276055	0	0	0	0	0	0	0	0	0	0

ENSG00000276054	0	0	0	0	0	0	0	0	0	0
ENSG00000276050	0	0	0	0	0	0	0	0	0	0
ENSG00000276048	0	0	0	0	0	0	0	0	0	0
ENSG00000276047	0	0	0	0	1	0	0	3	0	0
ENSG00000276046	0	0	0	0	0	0	0	0	0	0
ENSG00000276045	60	78	85	101	56	219	823	426	0	2
ENSG00000276043	258	271	377	266	241	624	65	51	1	0
ENSG00000276040	0	0	0	0	0	0	0	2	0	0
ENSG00000276039	0	0	0	0	0	0	0	0	0	0
ENSG00000276038	0	0	0	0	0	0	0	0	0	0
ENSG00000276037	0	0	0	0	0	0	0	0	0	0
ENSG00000276036	0	0	0	0	0	1	0	0	0	0
ENSG00000276032	3	12	7	10	8	7	0	16	0	0
ENSG00000276031	0	0	0	0	0	0	0	2	0	0
ENSG00000276030	0	0	0	0	0	1	4	2	0	0
ENSG00000276029	3	2	2	0	4	0	2	1	0	0
ENSG00000276027	0	0	0	0	0	0	0	0	0	0
ENSG00000276026	4	9	4	1	6	9	0	6	0	0
ENSG00000276024	0	0	0	0	0	0	0	0	0	0
ENSG00000276023	813	650	1043	653	679	2184	1439	356	0	15
ENSG00000276019	0	0	0	0	0	0	0	0	0	0
ENSG00000276018	0	0	0	0	0	0	0	0	0	0
ENSG00000276015	0	0	0	0	0	0	0	0	0	0
ENSG00000276014	0	0	0	0	0	0	0	23	0	0
ENSG00000276012	0	0	0	0	1	0	4	0	0	0
ENSG00000276008	16	12	21	7	16	26	19	30	0	0
ENSG00000276007	0	5	14	2	4	3	4	20	0	0
ENSG00000276006	0	0	0	0	0	0	0	0	0	0
ENSG00000276003	0	0	0	0	0	0	0	0	0	0
ENSG00000276002	0	0	0	0	0	0	0	0	0	0
ENSG00000275998	0	0	0	0	0	0	0	0	0	0
ENSG00000275997	0	0	0	0	0	0	0	0	0	0
ENSG00000275996	0	0	0	0	0	0	0	0	0	0
ENSG00000275995	0	0	0	0	0	0	0	1	0	0
ENSG00000275994	0	0	0	0	0	0	0	0	0	0
ENSG00000275993	1302	1522	2046	1417	1188	3662	5047	4015	0	50
ENSG00000275992	0	0	0	0	0	0	0	0	0	0
ENSG00000275989	0	0	0	0	0	0	3	0	0	0
ENSG00000275988	0	0	0	0	0	0	0	0	0	0
ENSG00000275986	0	0	0	0	0	0	0	0	0	0
ENSG00000275982	0	0	0	0	0	0	0	0	0	0
ENSG00000275981	0	0	0	0	3	1	15	2	0	0
ENSG00000275980	0	0	0	0	0	0	0	0	0	0
ENSG00000275978	0	0	0	0	0	0	0	0	0	0
ENSG00000275977	0	0	0	0	0	0	0	0	0	0
ENSG00000275976	44	44	51	51	36	169	31	21	0	0
ENSG00000275975	0	0	0	0	0	0	0	0	0	0
ENSG00000275971	0	0	0	0	0	0	0	0	0	0
ENSG00000275969	0	0	0	0	0	0	0	0	0	0
ENSG00000275968	0	0	0	0	0	0	0	0	0	0
ENSG00000275967	0	0	0	0	0	0	0	0	0	0
ENSG00000275966	0	1	0	0	0	0	11	3	0	0
ENSG00000275965	0	0	0	0	0	0	0	0	0	0
ENSG00000275964	10	6	5	5	4	10	1	32	0	0
ENSG00000275963	2	0	0	1	0	0	3	9	0	0
ENSG00000275961	0	0	0	0	0	0	0	0	0	0
ENSG00000275959	0	0	0	0	0	0	0	0	0	0

ENSG00000275958	0	0	0	0	0	0	0	0	0	0
ENSG00000275957	0	0	0	0	0	0	0	0	0	0
ENSG00000275956	1	0	0	0	0	0	5	0	0	0
ENSG00000275955	0	0	0	0	0	2	0	0	0	0
ENSG00000275954	205	235	286	193	143	491	88	26	0	0
ENSG00000275953	0	0	0	0	0	0	0	0	0	0
ENSG00000275952	0	0	0	0	0	0	0	0	0	0
ENSG00000275950	0	0	0	0	0	0	27	4	0	0
ENSG00000275948	0	0	0	0	0	0	2	0	0	0
ENSG00000275945	6	7	7	4	3	2	4	1	0	0
ENSG00000275944	0	0	0	0	0	0	0	0	0	0
ENSG00000275943	2	0	0	0	0	0	0	0	0	0
ENSG00000275942	0	0	0	0	0	0	0	0	0	0
ENSG00000275940	0	0	0	0	0	0	0	0	0	0
ENSG00000275939	0	0	0	0	0	0	0	0	0	0
ENSG00000275936	2	1	0	1	1	5	3	1	0	0
ENSG00000275934	0	0	0	0	0	0	0	0	0	0
ENSG00000275933	0	0	0	0	0	0	0	1	0	0
ENSG00000275930	2	3	7	1	0	2	3	2	0	0
ENSG00000275929	0	0	0	0	0	0	0	0	0	0
ENSG00000275928	0	0	0	0	0	0	0	1	0	0
ENSG00000275927	0	0	0	0	0	0	4	0	0	0
ENSG00000275926	0	0	0	0	0	0	0	0	0	0
ENSG00000275925	0	0	0	0	0	0	0	0	0	0
ENSG00000275924	0	0	0	0	0	0	2	0	0	0
ENSG00000275923	0	0	0	0	0	0	0	0	0	0
ENSG00000275922	0	0	0	0	0	0	0	0	0	0
ENSG00000275919	0	0	0	0	1	0	3	0	0	0
ENSG00000275916	0	0	0	0	0	0	0	0	0	0
ENSG00000275915	0	0	0	0	0	0	0	0	0	0
ENSG00000275912	0	0	0	0	0	0	2	0	0	0
ENSG00000275910	4	5	8	2	1	6	3	0	0	0
ENSG00000275909	0	0	0	0	0	0	0	2	0	0
ENSG00000275908	0	0	0	0	0	0	1	0	0	0
ENSG00000275906	0	0	0	0	0	0	0	0	0	0
ENSG00000275905	0	0	0	0	0	0	0	0	0	0
ENSG00000275904	0	0	0	0	0	0	0	2	0	0
ENSG00000275902	0	0	0	0	0	0	0	0	0	0
ENSG00000275901	0	1	0	0	0	0	0	0	0	0
ENSG00000275900	0	0	0	0	0	0	0	0	0	0
ENSG00000275899	0	0	0	0	0	0	1	0	0	0
ENSG00000275898	0	0	0	0	0	0	0	1	2	0
ENSG00000275897	6	4	10	5	5	13	3	0	0	0
ENSG00000275896	0	0	0	0	0	0	6	8	0	0
ENSG00000275895	830	768	1376	604	775	2148	6348	3227	0	66
ENSG00000275894	8	5	5	5	5	6	81	21	0	0
ENSG00000275892	0	0	0	0	0	0	0	0	0	0
ENSG00000275891	0	0	0	0	0	0	0	0	0	0
ENSG00000275888	3	13	20	9	4	36	80	81	0	0
ENSG00000275884	0	0	0	0	0	0	0	0	0	0
ENSG00000275883	0	0	0	0	0	0	0	0	0	0
ENSG00000275882	87	107	159	114	80	314	305	213	0	0
ENSG00000275881	0	0	0	0	1	1	0	3	0	0
ENSG00000275880	24	18	48	18	26	41	3	4	0	0
ENSG00000275878	0	0	0	0	0	0	0	0	0	0
ENSG00000275877	0	0	0	0	0	0	0	0	0	0
ENSG00000275876	0	0	0	0	0	0	0	0	0	0

ENSG00000275875	0	0	0	0	0	0	0	0	0	0
ENSG00000275874	0	0	0	0	0	0	18	3	0	0
ENSG00000275871	0	0	0	0	0	0	0	0	0	0
ENSG00000275870	0	0	0	0	0	0	0	0	0	0
ENSG00000275868	0	0	0	0	0	0	0	0	0	0
ENSG00000275866	0	0	0	0	0	0	0	0	0	0
ENSG00000275864	0	0	0	0	0	0	0	0	0	0
ENSG00000275863	0	0	0	0	0	0	0	0	0	0
ENSG00000275862	0	0	0	0	0	0	0	0	0	0
ENSG00000275860	0	0	0	0	0	0	2	0	0	0
ENSG00000275859	0	0	0	0	0	0	0	0	0	0
ENSG00000275858	0	0	0	0	1	0	0	0	0	0
ENSG00000275857	0	0	0	0	0	0	0	0	0	0
ENSG00000275856	0	0	0	0	0	0	0	0	0	0
ENSG00000275854	0	0	0	0	0	0	3	3	0	0
ENSG00000275853	0	0	0	0	0	1	0	0	0	0
ENSG00000275852	0	0	0	0	0	0	0	0	0	0
ENSG00000275850	0	0	0	0	0	0	9	4	0	0
ENSG00000275849	0	0	0	0	0	0	0	0	0	0
ENSG00000275847	0	0	0	0	0	0	0	0	0	0
ENSG00000275846	1	0	0	0	1	1	6	3	0	0
ENSG00000275845	0	0	0	0	0	0	0	0	0	2
ENSG00000275842	0	0	0	0	0	0	0	0	0	0
ENSG00000275840	0	1	0	0	0	0	0	0	0	0
ENSG00000275839	0	1	0	0	0	0	0	3	0	0
ENSG00000275836	0	0	0	0	0	0	0	0	0	0
ENSG00000275835	288	265	454	253	268	797	158	219	0	3
ENSG00000275834	0	0	0	0	0	0	0	0	0	0
ENSG00000275833	1	0	0	0	0	0	0	0	0	0
ENSG00000275832	1835	1918	2706	1666	1557	4479	1233	863	13	79
ENSG00000275831	0	0	0	0	0	0	0	0	0	0
ENSG00000275830	1	2	0	3	1	1	8	19	0	0
ENSG00000275829	0	0	0	0	0	0	0	0	0	0
ENSG00000275828	0	0	0	0	0	0	0	0	0	0
ENSG00000275827	0	0	0	0	0	0	0	0	0	0
ENSG00000275826	0	0	0	0	0	0	0	0	0	0
ENSG00000275823	0	0	0	0	0	0	0	0	0	0
ENSG00000275820	0	0	0	0	0	0	0	0	0	0
ENSG00000275818	0	0	0	0	0	0	0	0	0	0
ENSG00000275816	0	0	0	0	0	0	0	0	0	0
ENSG00000275813	0	0	0	0	0	0	0	0	0	0
ENSG00000275812	0	0	0	0	0	0	0	0	0	0
ENSG00000275811	0	0	0	0	0	0	4	0	0	0
ENSG00000275810	0	0	0	0	0	0	0	0	0	0
ENSG00000275808	0	0	0	0	0	0	0	0	0	0
ENSG00000275807	7	4	5	0	1	9	5	0	0	0
ENSG00000275805	0	0	0	0	1	3	0	10	0	0
ENSG00000275803	0	0	0	1	0	0	0	0	0	0
ENSG00000275802	0	0	0	0	0	0	0	0	0	0
ENSG00000275801	0	0	0	0	0	0	1	0	0	0
ENSG00000275800	0	0	0	0	0	0	0	0	0	0
ENSG00000275799	0	0	2	0	0	0	6	0	0	0
ENSG00000275793	2	3	2	1	0	4	13	2	0	0
ENSG00000275791	0	0	0	0	0	0	31	1	0	0
ENSG00000275790	0	0	0	0	0	0	0	0	0	0
ENSG00000275789	0	0	0	0	0	0	0	0	0	0
ENSG00000275788	0	0	0	0	0	2	0	0	0	0

ENSG00000275787	0	0	0	0	0	0	0	0	0	0
ENSG00000275785	0	0	0	0	0	0	0	0	0	0
ENSG00000275784	1	0	5	0	0	3	0	3	0	0
ENSG00000275783	0	0	0	0	0	0	38	19	0	0
ENSG00000275778	0	0	0	0	0	0	0	0	0	0
ENSG00000275776	0	0	0	0	0	0	1	0	0	0
ENSG00000275774	0	0	1	0	1	0	0	0	0	0
ENSG00000275773	0	0	0	0	0	0	0	3	0	0
ENSG00000275772	0	0	0	0	0	0	0	7	0	0
ENSG00000275771	0	0	0	0	0	0	0	0	0	0
ENSG00000275770	0	0	0	0	0	0	0	0	0	0
ENSG00000275769	0	0	0	0	0	0	4	1	0	0
ENSG00000275768	0	0	0	0	0	0	0	0	0	0
ENSG00000275767	0	0	7	0	0	0	0	0	0	0
ENSG00000275765	7	6	7	9	4	8	9	38	0	0
ENSG00000275764	35	70	202	55	56	145	324	110	2	2
ENSG00000275763	5	11	11	2	5	7	1	0	0	0
ENSG00000275762	0	0	0	0	0	0	0	0	0	0
ENSG00000275761	0	0	0	0	0	0	1	0	0	0
ENSG00000275759	2	3	1	1	0	9	16	14	0	0
ENSG00000275756	0	0	0	0	0	0	7	0	0	0
ENSG00000275747	0	0	0	0	0	0	0	0	0	0
ENSG00000275745	0	0	0	0	0	0	0	0	0	0
ENSG00000275743	0	0	0	0	0	0	8	0	0	0
ENSG00000275742	0	0	0	0	0	0	0	0	0	0
ENSG00000275741	0	0	0	0	0	0	0	0	0	0
ENSG00000275740	0	0	1	0	0	0	0	0	0	0
ENSG00000275739	0	0	0	0	0	0	0	0	0	0
ENSG00000275734	0	0	0	1	2	1	1	8	0	0
ENSG00000275733	0	0	0	0	0	0	0	0	0	0
ENSG00000275729	0	0	0	0	0	0	0	0	0	0
ENSG00000275728	0	0	0	0	0	0	0	0	0	0
ENSG00000275727	1	0	1	2	0	2	2	43	0	0
ENSG00000275726	0	0	0	0	0	0	0	0	0	0
ENSG00000275725	0	0	0	0	0	0	1	6	0	0
ENSG00000275722	0	0	0	0	0	0	0	5	0	0
ENSG00000275720	1	0	1	0	1	0	6	4	0	0
ENSG00000275719	2	2	0	2	1	3	7	2	0	0
ENSG00000275718	0	0	0	0	0	0	40	3	0	15
ENSG00000275716	0	0	0	0	0	0	0	0	0	0
ENSG00000275714	1	0	1	6	1	0	7	4	0	0
ENSG00000275713	6	4	1	5	4	12	11	0	0	0
ENSG00000275711	0	0	0	0	0	0	0	0	0	0
ENSG00000275710	0	0	0	0	0	0	0	0	0	0
ENSG00000275709	0	0	0	0	0	0	7	0	0	0
ENSG00000275708	0	0	0	0	0	0	52	44	0	0
ENSG00000275707	0	0	0	0	0	0	0	0	0	0
ENSG00000275704	0	0	0	0	0	0	0	0	0	0
ENSG00000275703	0	1	1	1	0	2	8	1	0	0
ENSG00000275702	0	0	0	0	0	0	0	0	0	0
ENSG00000275701	0	0	0	0	0	0	0	0	0	0
ENSG00000275700	956	766	1173	874	874	2617	2468	1269	4	31
ENSG00000275696	0	0	0	0	0	0	0	1	0	0
ENSG00000275695	0	0	2	1	1	0	1	2	0	0
ENSG00000275694	0	0	0	0	0	0	0	0	0	0
ENSG00000275693	0	0	0	0	0	0	8	0	0	4
ENSG00000275692	0	0	0	0	0	0	24	3	1	0

ENSG00000275691	0	0	0	0	0	0	1	0	0	0
ENSG00000275690	0	0	0	0	0	0	0	0	0	0
ENSG00000275689	0	0	0	0	0	0	0	0	0	0
ENSG00000275688	0	0	0	0	0	0	0	0	0	0
ENSG00000275687	0	0	0	0	0	0	0	0	0	0
ENSG00000275686	0	0	0	0	0	0	4	0	0	0
ENSG00000275685	0	0	0	0	0	0	0	0	0	0
ENSG00000275682	1	1	0	0	0	0	0	0	0	0
ENSG00000275681	0	0	0	0	0	0	0	0	0	0
ENSG00000275680	0	0	0	0	0	0	0	1	0	0
ENSG00000275678	0	0	0	0	0	0	0	4	0	0
ENSG00000275677	0	0	0	0	0	0	0	0	0	0
ENSG00000275676	0	0	0	0	0	0	0	4	0	9
ENSG00000275675	1	2	1	1	2	1	0	0	0	0
ENSG00000275674	0	0	0	0	0	0	0	0	0	0
ENSG00000275673	0	0	0	0	0	0	0	0	0	0
ENSG00000275672	2	3	9	2	0	2	5	21	0	4
ENSG00000275670	0	0	0	0	0	0	0	0	0	0
ENSG00000275669	0	0	0	0	0	0	0	0	0	0
ENSG00000275668	0	0	0	0	0	0	0	0	0	0
ENSG00000275667	0	0	0	0	0	0	0	0	0	0
ENSG00000275666	0	0	0	0	0	0	0	0	0	0
ENSG00000275665	0	0	0	0	0	3	0	0	0	0
ENSG00000275664	0	0	0	0	0	0	18	1	0	0
ENSG00000275663	0	0	0	0	0	0	0	0	0	0
ENSG00000275662	0	0	0	0	0	0	0	0	0	0
ENSG00000275657	0	0	0	0	0	0	0	0	0	0
ENSG00000275656	0	0	0	0	0	0	0	0	0	0
ENSG00000275655	0	0	0	0	0	0	0	0	0	0
ENSG00000275654	0	0	0	0	0	0	0	0	0	0
ENSG00000275653	0	0	0	0	0	0	0	0	0	0
ENSG00000275652	0	0	0	0	0	0	0	0	0	0
ENSG00000275651	0	0	0	0	0	0	0	0	0	0
ENSG00000275649	0	0	0	2	0	0	3	10	0	0
ENSG00000275647	0	0	0	0	0	0	1	0	0	0
ENSG00000275646	0	0	0	0	0	0	0	0	0	0
ENSG00000275645	0	0	1	1	1	2	12	0	0	0
ENSG00000275643	0	0	0	0	0	0	0	2	0	0
ENSG00000275642	0	0	0	0	0	0	0	0	0	0
ENSG00000275640	0	0	0	0	0	0	0	0	0	0
ENSG00000275639	0	0	0	0	0	0	0	0	0	0
ENSG00000275638	0	0	0	0	0	0	0	0	0	0
ENSG00000275636	0	0	0	0	0	0	1	0	0	0
ENSG00000275635	0	0	0	0	0	0	0	0	0	0
ENSG00000275634	0	0	0	0	0	0	0	0	0	0
ENSG00000275632	4	1	6	1	0	1	10	3	0	0
ENSG00000275631	0	0	0	0	0	0	0	0	0	0
ENSG00000275630	0	2	0	0	0	0	6	3	0	0
ENSG00000275628	1	0	0	0	0	0	0	12	0	0
ENSG00000275625	0	0	0	0	0	0	0	0	0	0
ENSG00000275622	0	0	0	0	0	0	0	0	0	0
ENSG00000275620	6	14	17	5	8	26	0	0	0	0
ENSG00000275616	0	1	5	5	1	11	232	21	21	292
ENSG00000275614	0	0	0	0	0	5	0	0	0	0
ENSG00000275613	0	0	0	0	0	0	0	0	0	0
ENSG00000275612	1	1	2	0	0	0	7	0	0	0
ENSG00000275611	0	0	0	0	0	0	22	3	0	0

ENSG00000275610	0	0	0	0	0	1	0	0	0	0
ENSG00000275609	0	0	0	2	0	1	0	0	0	0
ENSG00000275607	0	0	0	0	0	0	0	0	0	0
ENSG00000275604	0	0	0	0	0	0	0	0	0	0
ENSG00000275603	0	0	0	0	0	0	0	0	0	0
ENSG00000275601	3	4	5	5	3	6	16	10	0	0
ENSG00000275598	0	0	0	0	0	0	0	0	0	0
ENSG00000275597	0	0	0	0	0	0	0	2	0	0
ENSG00000275595	0	0	0	0	0	0	0	0	0	0
ENSG00000275592	0	0	0	0	0	0	0	0	0	0
ENSG00000275591	0	0	0	0	1	0	0	3	0	0
ENSG00000275589	0	0	0	0	0	0	0	0	0	0
ENSG00000275588	0	0	0	0	0	0	0	0	0	0
ENSG00000275586	0	0	0	0	0	0	3	0	0	0
ENSG00000275585	0	2	4	2	1	0	3	0	0	0
ENSG00000275582	13	10	16	3	11	22	12	8	0	0
ENSG00000275580	2	3	1	0	1	0	4	3	0	0
ENSG00000275578	0	0	0	0	0	0	0	0	0	0
ENSG00000275576	0	0	0	0	0	0	0	10	0	0
ENSG00000275572	0	0	0	0	0	0	0	0	0	0
ENSG00000275569	0	0	0	0	0	0	6	5	0	0
ENSG00000275567	0	0	0	0	0	0	6	0	0	0
ENSG00000275563	0	0	0	0	0	0	0	17	0	0
ENSG00000275562	0	0	0	0	0	0	0	0	0	0
ENSG00000275560	3	7	12	1	4	15	27	5	0	3
ENSG00000275559	0	0	0	0	0	0	0	0	0	0
ENSG00000275558	0	0	0	0	0	0	0	0	0	0
ENSG00000275557	4	2	8	3	2	17	60	44	0	0
ENSG00000275554	0	0	0	0	0	0	0	0	0	0
ENSG00000275553	0	3	0	0	0	0	0	0	0	0
ENSG00000275552	4	1	9	2	2	3	4	4	0	0
ENSG00000275550	0	0	0	0	0	0	4	0	0	0
ENSG00000275549	1	1	2	0	0	0	3	0	0	0
ENSG00000275547	0	0	0	0	0	0	0	0	0	0
ENSG00000275542	3	0	6	3	0	0	2	7	0	0
ENSG00000275541	0	0	0	0	0	0	0	0	0	0
ENSG00000275540	0	3	3	2	1	5	0	1	0	0
ENSG00000275538	0	0	0	0	0	0	7	0	0	0
ENSG00000275537	0	0	0	0	0	0	0	0	0	0
ENSG00000275535	0	0	0	0	0	0	0	0	0	0
ENSG00000275534	0	0	0	0	0	0	0	0	0	0
ENSG00000275533	0	0	0	0	0	0	0	0	0	0
ENSG00000275532	2	1	4	4	1	2	7	0	0	0
ENSG00000275530	13	77	28	57	8	24	95108	32803	272	2180
ENSG00000275529	0	0	0	0	0	0	0	0	0	0
ENSG00000275527	0	0	0	0	0	0	0	0	0	0
ENSG00000275524	0	0	0	0	0	0	0	0	0	0
ENSG00000275523	0	0	0	0	0	0	0	0	0	0
ENSG00000275520	0	0	1	0	0	0	6	0	0	0
ENSG00000275519	0	0	0	0	0	0	0	0	0	0
ENSG00000275518	0	0	0	0	0	0	0	0	0	0
ENSG00000275516	0	0	0	0	0	0	0	0	0	0
ENSG00000275515	0	0	0	0	0	0	0	0	0	0
ENSG00000275512	28	34	61	14	35	98	85	43	2	2
ENSG00000275510	0	0	0	0	0	0	0	0	0	0
ENSG00000275509	0	0	0	0	0	0	0	0	0	0
ENSG00000275508	0	0	0	0	0	0	0	0	0	0

ENSG00000275507	0	0	0	0	0	0	0	0	0	0
ENSG00000275506	0	1	0	0	0	0	0	4	0	0
ENSG00000275505	0	0	0	0	0	0	0	0	0	0
ENSG00000275504	0	0	0	0	0	0	0	0	0	0
ENSG00000275503	0	0	0	0	0	0	0	0	0	0
ENSG00000275502	0	0	0	0	0	0	0	0	0	0
ENSG00000275497	0	0	0	0	0	0	4	0	0	0
ENSG00000275496	404	324	601	254	291	807	152	28	0	0
ENSG00000275494	0	0	3	0	1	0	8	32	0	0
ENSG00000275493	0	0	0	0	0	0	0	0	0	0
ENSG00000275491	1	1	0	2	4	7	17	16	0	0
ENSG00000275490	0	0	0	0	0	0	0	0	0	0
ENSG00000275489	0	0	0	0	0	4	1	0	0	0
ENSG00000275488	0	0	0	0	0	0	1	2	0	0
ENSG00000275485	0	0	0	0	0	0	9	0	0	0
ENSG00000275484	2	2	1	0	1	1	30	4	0	0
ENSG00000275481	2	0	1	1	0	3	11	0	0	0
ENSG00000275480	0	0	0	0	0	0	0	0	0	0
ENSG00000275479	0	1	0	0	0	0	0	6	0	0
ENSG00000275476	1	2	2	0	2	2	8	20	0	0
ENSG00000275475	0	0	0	0	0	0	0	0	0	0
ENSG00000275469	0	0	0	0	0	0	0	0	0	0
ENSG00000275468	5	2	3	3	1	9	0	0	0	0
ENSG00000275467	0	0	0	0	0	0	0	0	0	0
ENSG00000275466	0	0	0	0	0	0	0	0	0	0
ENSG00000275465	0	0	0	0	0	0	0	2	0	0
ENSG00000275464	338	290	470	380	327	976	1642	491	0	3
ENSG00000275461	0	0	0	0	0	0	0	0	0	0
ENSG00000275460	0	0	0	0	0	0	0	0	0	0
ENSG00000275459	0	0	0	0	0	0	0	0	0	0
ENSG00000275458	0	0	0	0	0	0	8	0	0	0
ENSG00000275457	1	1	4	1	3	0	10	19	0	0
ENSG00000275455	0	0	0	0	0	0	0	0	0	0
ENSG00000275454	1	0	7	3	0	0	9	6	0	0
ENSG00000275453	0	0	0	0	0	0	0	0	0	0
ENSG00000275451	0	0	0	0	0	0	0	0	0	0
ENSG00000275450	0	1	1	1	0	0	1	12	0	8
ENSG00000275449	0	0	0	0	0	0	0	3	0	0
ENSG00000275448	0	0	0	0	0	0	0	0	0	0
ENSG00000275447	0	0	0	0	0	0	0	0	0	1
ENSG00000275446	0	0	0	0	0	0	0	0	0	0
ENSG00000275445	0	0	2	0	0	0	0	4	0	0
ENSG00000275443	1	1	0	1	1	3	0	0	0	0
ENSG00000275441	0	0	0	0	0	0	13	4	0	8
ENSG00000275439	0	0	0	0	0	0	0	4	0	0
ENSG00000275438	0	0	0	0	0	0	0	2	0	0
ENSG00000275437	4	1	3	2	3	3	11	0	1	0
ENSG00000275432	0	0	0	0	0	0	0	0	0	0
ENSG00000275431	0	0	0	0	0	1	3	2	0	0
ENSG00000275429	0	0	0	0	0	0	1	0	0	0
ENSG00000275427	0	0	0	0	0	0	0	5	0	0
ENSG00000275426	0	0	0	0	0	0	3	0	0	0
ENSG00000275425	0	0	0	0	0	0	0	0	0	0
ENSG00000275423	0	0	0	0	0	0	0	0	0	0
ENSG00000275419	0	0	0	0	0	0	0	0	0	0
ENSG00000275418	0	0	0	0	0	0	0	0	0	0
ENSG00000275417	0	0	0	0	0	0	0	0	0	0

ENSG00000275414	0	0	0	0	0	0	0	0	0	0
ENSG00000275413	0	0	0	0	0	0	14	3	0	0
ENSG00000275411	0	0	0	0	0	0	0	0	0	0
ENSG00000275410	0	2	0	2	1	2	160	27	0	7
ENSG00000275409	0	0	2	0	0	0	11	3	0	0
ENSG00000275408	0	0	0	0	0	0	0	0	0	0
ENSG00000275406	0	0	0	0	0	0	0	0	0	0
ENSG00000275403	0	0	0	0	0	0	0	0	0	0
ENSG00000275402	0	0	0	0	0	0	0	0	0	0
ENSG00000275401	1	0	5	3	0	1	12	0	0	0
ENSG00000275400	14	20	36	14	28	56	476	158	0	0
ENSG00000275398	0	0	0	0	0	0	0	0	0	0
ENSG00000275395	9	5	13	2	3	0	43	49	0	4
ENSG00000275393	7	8	8	5	7	21	0	2	0	0
ENSG00000275392	0	0	0	0	0	0	1	3	0	0
ENSG00000275391	0	0	0	0	0	0	0	0	0	0
ENSG00000275390	0	0	0	0	0	0	0	0	0	0
ENSG00000275389	2	1	0	0	0	8	0	0	0	0
ENSG00000275388	0	1	0	0	0	0	0	0	0	0
ENSG00000275387	0	0	0	0	0	0	0	3	0	0
ENSG00000275386	0	0	0	0	0	0	0	0	0	0
ENSG00000275385	0	0	0	0	0	0	2533	2914	42	1731
ENSG00000275383	0	0	9	0	3	1	3	10	0	0
ENSG00000275382	0	0	0	0	0	0	0	0	0	0
ENSG00000275381	0	0	0	0	0	0	0	1	0	0
ENSG00000275380	0	0	0	0	0	0	0	0	0	0
ENSG00000275379	0	0	0	1	0	0	3	0	0	0
ENSG00000275378	0	0	0	0	0	0	0	0	0	0
ENSG00000275377	0	0	0	0	0	0	0	0	0	0
ENSG00000275374	0	6	3	2	0	1	1	1	0	0
ENSG00000275373	1	0	0	0	0	0	3	0	0	0
ENSG00000275372	0	0	0	0	0	0	0	0	0	0
ENSG00000275371	1	3	4	3	4	3	12	2	0	0
ENSG00000275370	0	0	0	0	0	0	0	1	0	0
ENSG00000275369	0	0	0	0	0	0	0	0	0	0
ENSG00000275367	10	5	1	1	0	0	12	71	0	0
ENSG00000275364	0	0	0	0	0	0	0	0	0	0
ENSG00000275363	1	0	0	0	0	1	2	0	0	0
ENSG00000275362	0	0	0	0	0	0	0	0	0	0
ENSG00000275361	0	0	0	0	0	0	0	3	0	0
ENSG00000275360	0	0	0	0	0	0	0	0	0	0
ENSG00000275359	0	0	0	0	0	0	0	0	0	0
ENSG00000275358	0	0	0	0	0	0	0	0	0	0
ENSG00000275356	0	0	0	0	0	0	0	0	0	0
ENSG00000275355	0	0	1	0	0	1	0	1	0	0
ENSG00000275352	0	0	0	0	0	0	0	0	0	0
ENSG00000275350	0	0	0	0	0	0	0	4	0	0
ENSG00000275348	0	0	0	0	0	0	0	0	0	0
ENSG00000275347	0	0	0	0	0	0	0	0	0	0
ENSG00000275345	0	0	0	0	0	0	0	0	0	0
ENSG00000275344	0	0	0	0	0	0	0	0	0	0
ENSG00000275343	0	0	0	0	0	0	7	0	0	0
ENSG00000275342	345	359	464	265	193	714	207	39	0	2
ENSG00000275340	0	0	0	0	1	0	2	1	0	0
ENSG00000275339	2	1	0	0	0	2	2	0	0	0
ENSG00000275337	0	0	0	0	0	0	0	0	0	0
ENSG00000275335	0	0	0	0	0	0	0	0	0	0

ENSG00000275334	0	0	0	0	0	0	0	0	0	0
ENSG00000275332	0	0	0	0	0	0	0	0	0	0
ENSG00000275329	0	1	3	1	0	4	0	6	0	0
ENSG00000275328	0	0	0	0	1	3	1	0	0	0
ENSG00000275327	0	0	0	0	0	0	0	0	0	0
ENSG00000275325	142	134	238	98	139	365	256	74	0	0
ENSG00000275322	1	7	9	2	2	9	0	1	0	0
ENSG00000275320	0	0	6	1	0	0	0	0	0	0
ENSG00000275319	0	0	0	0	0	0	0	0	0	0
ENSG00000275318	2	1	3	1	4	4	1	9	0	0
ENSG00000275315	0	0	0	0	0	0	0	0	0	0
ENSG00000275314	0	0	0	0	0	0	0	0	0	0
ENSG00000275312	0	0	0	0	1	7	0	0	0	0
ENSG00000275310	0	0	0	0	0	0	0	0	0	0
ENSG00000275309	0	0	0	0	0	0	0	0	0	0
ENSG00000275308	0	0	0	0	0	0	0	0	0	0
ENSG00000275307	0	0	0	0	0	0	0	0	0	0
ENSG00000275305	0	0	0	0	0	0	0	0	0	0
ENSG00000275303	0	0	0	0	0	0	0	0	0	0
ENSG00000275302	0	0	0	0	0	0	4796	411	991	21982
ENSG00000275301	0	0	0	0	0	0	4	0	0	0
ENSG00000275297	1	1	16	6	1	11	1	35	0	0
ENSG00000275296	0	0	0	0	0	0	0	0	0	0
ENSG00000275295	0	0	0	0	0	0	0	4	0	0
ENSG00000275294	2	2	1	1	1	6	2	5	0	0
ENSG00000275293	0	0	0	0	0	0	0	0	0	0
ENSG00000275292	0	0	0	0	0	0	0	0	0	0
ENSG00000275291	2	1	3	0	0	2	0	0	0	0
ENSG00000275287	0	0	0	0	0	0	1	0	0	0
ENSG00000275286	0	0	0	0	0	0	0	0	0	0
ENSG00000275285	0	0	0	0	0	0	0	2	0	0
ENSG00000275284	0	0	0	0	0	0	0	0	0	0
ENSG00000275281	0	0	0	0	0	0	0	0	0	0
ENSG00000275280	0	0	0	0	0	0	0	0	0	0
ENSG00000275279	5	1	4	1	0	8	4	4	0	0
ENSG00000275278	0	0	0	1	0	0	0	1	0	0
ENSG00000275277	0	0	0	0	0	0	0	0	0	0
ENSG00000275273	0	0	0	0	0	0	0	0	0	0
ENSG00000275271	0	0	0	0	0	0	0	1	0	1
ENSG00000275270	0	0	0	0	0	0	0	0	0	0
ENSG00000275268	0	0	0	0	0	0	0	0	0	0
ENSG00000275267	0	0	0	0	0	0	0	0	0	0
ENSG00000275266	0	0	0	0	0	0	0	0	0	0
ENSG00000275265	0	0	0	0	0	0	0	4	0	0
ENSG00000275264	0	0	0	0	0	0	0	0	0	0
ENSG00000275263	0	1	4	1	0	0	0	0	0	0
ENSG00000275261	0	0	0	0	0	4	0	0	0	0
ENSG00000275260	0	1	0	1	0	0	20	0	0	45
ENSG00000275259	0	0	0	0	0	0	0	0	0	0
ENSG00000275258	0	0	7	0	0	0	0	0	0	0
ENSG00000275256	0	0	0	0	0	0	0	0	0	0
ENSG00000275254	0	0	0	0	0	0	0	0	0	0
ENSG00000275252	0	0	0	0	0	0	65	12	0	2
ENSG00000275251	0	0	0	0	0	0	0	0	0	0
ENSG00000275250	0	0	0	0	0	0	0	0	0	0
ENSG00000275248	0	0	0	0	0	0	0	0	0	0
ENSG00000275243	0	0	0	0	0	0	0	0	0	0

ENSG00000275241	0	0	0	0	0	0	0	0	0	0
ENSG00000275239	0	0	0	0	0	0	0	0	0	0
ENSG00000275238	0	0	0	0	0	0	6	0	0	0
ENSG00000275236	1	4	1	0	0	6	1	7	0	0
ENSG00000275235	0	0	0	0	0	0	0	5	0	0
ENSG00000275234	91	99	164	89	101	278	222	59	0	0
ENSG00000275233	0	0	0	0	0	0	0	0	0	0
ENSG00000275232	0	0	0	0	0	0	0	0	0	0
ENSG00000275231	0	0	0	0	0	0	0	0	0	0
ENSG00000275230	0	0	0	0	0	0	0	0	0	0
ENSG00000275229	1	0	2	0	0	0	1	0	0	0
ENSG00000275228	0	0	0	0	0	0	1	0	0	0
ENSG00000275227	0	0	0	0	2	1	0	0	0	0
ENSG00000275226	0	0	0	0	0	0	0	4	0	0
ENSG00000275224	0	0	0	0	0	0	0	0	0	0
ENSG00000275223	0	4	3	5	1	2	2	0	0	0
ENSG00000275222	0	0	0	0	0	0	0	0	0	0
ENSG00000275221	0	0	0	0	0	0	0	0	0	0
ENSG00000275220	0	0	0	0	0	0	0	0	0	0
ENSG00000275219	0	1	5	6	1	11	231	22	20	293
ENSG00000275218	0	0	0	0	0	0	0	0	0	0
ENSG00000275217	0	0	0	0	0	0	0	0	0	0
ENSG00000275216	1517	1686	2605	1326	1556	4414	343	23	8	75
ENSG00000275215	30	31	69	41	19	77	45701	6146	188	1164
ENSG00000275213	0	0	0	0	0	0	0	0	0	0
ENSG00000275212	0	0	0	0	0	1	0	0	0	0
ENSG00000275210	0	1	0	0	0	0	3	2	1	0
ENSG00000275209	5	10	21	9	4	8	112780	2682	129	1303
ENSG00000275208	0	0	0	0	0	0	0	0	0	0
ENSG00000275207	0	0	0	0	2	0	0	0	0	0
ENSG00000275206	0	0	0	0	0	0	0	0	0	0
ENSG00000275202	10	9	5	8	7	16	31	38	0	0
ENSG00000275201	0	0	0	0	0	0	0	0	0	0
ENSG00000275200	0	0	0	0	0	0	0	0	0	0
ENSG00000275198	0	3	2	2	2	5	10	3	0	0
ENSG00000275197	0	0	0	0	0	0	1	0	0	0
ENSG00000275195	0	0	0	0	1	0	0	0	0	0
ENSG00000275194	0	0	0	0	0	0	0	0	0	0
ENSG00000275191	0	0	3	1	0	0	0	1	0	0
ENSG00000275186	0	0	0	0	0	0	4	2	0	0
ENSG00000275185	0	0	0	0	0	0	0	0	0	0
ENSG00000275184	0	0	0	0	0	0	0	0	0	0
ENSG00000275183	20	15	24	34	27	81	26	37	0	0
ENSG00000275182	0	0	0	0	0	0	0	0	0	0
ENSG00000275180	0	0	0	2	4	6	221	36	0	4
ENSG00000275179	0	0	0	0	0	0	0	0	0	0
ENSG00000275178	1	0	0	1	1	0	3	0	0	0
ENSG00000275175	0	0	0	0	0	0	1	0	0	0
ENSG00000275174	0	0	0	0	0	0	0	0	0	0
ENSG00000275173	0	0	0	0	0	0	0	0	0	0
ENSG00000275170	0	0	0	0	0	0	0	0	0	0
ENSG00000275167	0	0	0	0	0	0	2	0	0	0
ENSG00000275166	0	0	0	0	0	0	0	0	0	0
ENSG00000275163	0	0	0	0	0	0	0	0	0	0
ENSG00000275162	0	0	0	0	0	0	2	0	0	0
ENSG00000275161	0	0	0	0	0	0	0	0	0	0
ENSG00000275160	23	29	28	12	17	49	29	9	0	0

ENSG00000275159	0	0	0	0	0	0	0	0	0	0
ENSG00000275158	0	0	0	0	0	0	9	0	0	0
ENSG00000275157	0	0	0	0	0	0	0	0	0	0
ENSG00000275155	0	0	0	0	0	0	7	9	0	0
ENSG00000275154	0	0	0	0	0	0	0	0	0	0
ENSG00000275152	0	0	0	0	0	0	2	14	0	0
ENSG00000275150	0	0	0	0	0	0	0	0	0	0
ENSG00000275149	3	4	6	1	0	1	13	4	0	0
ENSG00000275147	1	0	0	0	0	0	0	0	0	0
ENSG00000275146	0	0	0	0	0	0	0	0	0	0
ENSG00000275144	0	0	0	0	0	0	0	0	0	0
ENSG00000275143	0	0	0	0	0	0	0	0	0	0
ENSG00000275142	0	0	4	0	0	0	3	10	0	0
ENSG00000275141	0	0	0	0	0	0	0	0	0	0
ENSG00000275140	11	15	53	11	16	41	123	3	0	9
ENSG00000275139	0	1	0	0	0	0	1	10	0	2
ENSG00000275138	0	0	0	0	0	0	0	0	0	0
ENSG00000275135	0	0	0	0	0	0	0	0	0	0
ENSG00000275134	0	0	0	0	0	0	0	0	0	0
ENSG00000275132	1	0	0	2	0	1	1	0	0	0
ENSG00000275131	247	275	396	157	225	562	938	120	0	0
ENSG00000275130	0	0	0	0	0	0	0	0	0	0
ENSG00000275129	2	1	6	0	0	2	0	0	0	0
ENSG00000275128	0	0	0	0	0	0	0	0	0	0
ENSG00000275127	0	0	0	0	0	0	0	0	0	0
ENSG00000275126	0	0	0	0	0	0	3	0	0	0
ENSG00000275124	0	0	0	0	0	0	0	0	0	0
ENSG00000275121	0	0	0	0	0	0	0	0	0	0
ENSG00000275120	0	0	0	0	0	0	2	6	0	0
ENSG00000275119	0	0	0	0	0	0	1	0	0	0
ENSG00000275115	2	0	3	0	0	0	1	0	0	0
ENSG00000275114	0	0	0	0	0	0	1	0	0	0
ENSG00000275113	0	0	0	0	0	0	0	0	0	0
ENSG00000275111	87	74	146	75	84	205	146	56	0	0
ENSG00000275110	0	0	0	0	0	0	0	0	0	0
ENSG00000275109	0	0	0	0	0	0	2	0	0	0
ENSG00000275108	0	1	0	0	1	1	0	0	0	0
ENSG00000275107	0	0	0	0	0	0	0	0	0	0
ENSG00000275106	0	0	5	1	0	0	7	9	0	0
ENSG00000275105	0	0	0	0	0	0	0	0	0	0
ENSG00000275103	0	0	0	0	0	0	38	0	0	0
ENSG00000275101	0	1	0	0	0	0	0	0	0	0
ENSG00000275100	0	0	0	0	0	0	0	1	0	0
ENSG00000275098	0	0	0	0	0	0	0	0	0	0
ENSG00000275097	0	0	0	0	0	0	4	0	0	0
ENSG00000275096	0	0	0	0	0	0	0	0	0	0
ENSG00000275094	0	0	0	0	0	0	0	0	0	0
ENSG00000275092	0	0	0	0	0	0	15	9	0	0
ENSG00000275091	0	0	0	0	0	0	4	0	0	0
ENSG00000275090	0	0	0	0	0	0	0	0	0	0
ENSG00000275089	0	0	0	0	0	0	0	0	0	0
ENSG00000275088	0	0	0	0	0	0	0	0	0	0
ENSG00000275085	0	0	0	0	0	0	0	0	0	0
ENSG00000275084	0	0	0	0	0	0	0	0	0	0
ENSG00000275078	0	0	0	0	0	0	0	0	0	0
ENSG00000275077	0	0	0	0	0	0	0	0	0	0
ENSG00000275076	0	0	0	0	0	0	1	0	0	0

ENSG00000275075	0	0	0	0	0	0	0	0	0	0
ENSG00000275074	57	76	73	57	55	154	113	148	0	0
ENSG00000275072	0	0	0	0	0	0	0	0	0	0
ENSG00000275071	0	0	0	0	0	0	0	0	0	0
ENSG00000275070	0	0	1	0	0	0	19	7	0	0
ENSG00000275069	0	0	0	0	0	0	0	0	0	0
ENSG00000275068	0	0	0	0	0	0	0	0	0	0
ENSG00000275067	0	0	0	0	0	0	0	0	0	0
ENSG00000275066	633	626	920	483	506	1518	635	260	1	14
ENSG00000275065	0	0	0	0	0	0	0	0	0	0
ENSG00000275064	0	2	0	0	0	0	17	0	0	0
ENSG00000275061	0	0	0	0	0	0	0	0	0	0
ENSG00000275060	0	0	0	0	0	0	0	0	0	0
ENSG00000275058	0	0	0	0	0	0	0	0	0	0
ENSG00000275056	2	2	2	3	4	14	21	33	0	0
ENSG00000275055	4	10	14	4	7	25	7	8	0	0
ENSG00000275054	0	0	0	0	0	0	0	0	0	0
ENSG00000275053	0	0	0	0	0	0	0	0	0	0
ENSG00000275052	849	803	1636	552	643	1845	2928	1087	5	97
ENSG00000275048	0	0	0	0	0	0	0	0	0	0
ENSG00000275046	0	0	0	0	0	0	0	0	0	0
ENSG00000275044	0	0	0	0	0	0	0	1	0	0
ENSG00000275043	0	0	0	0	0	0	0	0	0	0
ENSG00000275042	0	0	0	0	0	0	0	0	0	0
ENSG00000275041	0	0	0	0	0	0	3	0	0	0
ENSG00000275040	0	0	0	0	0	0	0	0	0	0
ENSG00000275039	0	0	0	0	0	0	0	0	0	0
ENSG00000275038	0	0	0	0	0	0	0	0	0	0
ENSG00000275036	0	0	0	0	0	0	0	0	0	0
ENSG00000275034	3	6	3	4	3	14	0	1	0	0
ENSG00000275033	0	0	0	0	0	0	0	0	0	0
ENSG00000275030	0	0	0	0	0	0	0	0	0	0
ENSG00000275029	5	14	23	6	6	18	33	14	0	0
ENSG00000275027	0	0	0	0	0	0	0	1	0	0
ENSG00000275026	0	0	0	0	0	0	4	1	0	0
ENSG00000275025	4	1	1	0	1	0	7	2	0	0
ENSG00000275024	0	0	4	0	0	2	3	5	0	0
ENSG00000275023	707	854	1027	776	672	1901	3396	3072	23	112
ENSG00000275022	0	1	1	2	1	12	1	0	0	0
ENSG00000275020	0	0	0	0	0	0	0	0	0	0
ENSG00000275017	0	2	3	1	4	11	7	0	0	0
ENSG00000275016	0	0	1	0	0	1	2	6	0	0
ENSG00000275015	0	0	0	0	0	0	0	0	0	0
ENSG00000275014	1	0	0	0	0	0	0	1	0	0
ENSG00000275012	0	0	0	0	0	0	0	0	0	0
ENSG00000275011	0	3	0	2	2	2	0	0	0	0
ENSG00000275010	0	0	0	0	0	0	0	0	0	0
ENSG00000275006	0	0	0	0	0	0	0	14	0	0
ENSG00000275005	0	0	0	0	0	1	0	7	0	0
ENSG00000275004	16	13	31	13	12	18	13	8	0	0
ENSG00000275002	0	0	0	0	0	0	0	0	0	0
ENSG00000275001	0	0	0	0	0	0	0	0	0	0
ENSG00000275000	0	0	0	0	0	0	0	0	0	0
ENSG00000274999	0	0	0	0	0	0	0	0	0	0
ENSG00000274998	0	0	0	0	0	0	0	0	0	0
ENSG00000274997	0	1	2	0	0	0	2	0	0	0
ENSG00000274996	0	0	0	0	0	0	0	0	0	0

ENSG00000274995	0	0	0	0	0	0	0	3	0	0
ENSG00000274994	0	0	0	0	0	0	0	0	0	0
ENSG00000274993	0	0	0	0	0	2	2	0	0	0
ENSG00000274992	0	0	0	0	0	0	0	2	0	0
ENSG00000274991	2	0	1	0	0	0	2	0	0	0
ENSG00000274988	0	0	0	0	0	0	0	0	0	0
ENSG00000274987	0	0	0	0	0	0	10	19	0	0
ENSG00000274986	0	0	0	0	0	0	0	0	0	0
ENSG00000274985	0	0	0	0	0	0	0	0	0	0
ENSG00000274984	0	0	0	0	0	0	0	1	0	0
ENSG00000274983	0	0	0	0	0	0	2	0	0	0
ENSG00000274982	0	0	0	0	0	0	0	0	0	0
ENSG00000274981	0	0	0	0	0	0	0	0	0	0
ENSG00000274979	0	0	0	0	0	2	1	4	0	0
ENSG00000274978	0	0	0	0	0	0	0	0	0	0
ENSG00000274977	0	0	0	0	0	0	0	0	0	0
ENSG00000274976	2	0	1	0	2	0	0	1	0	0
ENSG00000274975	0	0	0	0	0	0	0	0	0	0
ENSG00000274973	0	0	0	0	0	0	0	0	0	0
ENSG00000274970	0	0	0	0	0	0	0	0	0	0
ENSG00000274969	0	0	0	0	0	0	0	0	0	0
ENSG00000274967	0	0	6	0	0	2	0	0	0	0
ENSG00000274966	0	0	2	2	0	0	1	1	0	0
ENSG00000274964	6	3	10	0	2	8	19	20	0	0
ENSG00000274963	0	1	1	0	0	0	8	4	0	0
ENSG00000274962	0	0	0	0	0	0	0	0	0	0
ENSG00000274961	0	0	0	0	0	0	0	0	0	0
ENSG00000274959	9	22	21	19	9	73	11	17	0	0
ENSG00000274956	0	0	0	0	0	0	0	10	0	0
ENSG00000274954	0	0	0	0	0	0	1	0	0	0
ENSG00000274951	0	0	0	0	0	0	0	4	0	0
ENSG00000274949	0	0	0	0	0	0	1	0	0	0
ENSG00000274944	1	0	15	3	2	0	0	0	0	0
ENSG00000274943	1	0	0	1	0	0	5	5	0	0
ENSG00000274940	0	0	0	0	0	0	1	1	0	0
ENSG00000274937	0	0	0	0	0	0	0	11	0	0
ENSG00000274934	0	0	0	0	0	0	0	0	0	0
ENSG00000274933	292	352	474	297	249	793	78	29	0	0
ENSG00000274932	0	0	0	0	0	0	0	0	0	0
ENSG00000274930	0	0	0	0	0	0	0	0	0	0
ENSG00000274929	0	2	0	1	1	2	3	3	0	0
ENSG00000274928	1	0	2	0	2	0	0	12	0	0
ENSG00000274927	6	5	13	0	1	4	3	3	0	0
ENSG00000274925	2	11	10	10	9	25	71	21	0	0
ENSG00000274924	0	0	0	0	0	0	0	0	0	0
ENSG00000274923	0	0	0	0	0	0	0	0	0	0
ENSG00000274922	4	3	7	1	2	20	19	66	0	0
ENSG00000274919	0	0	0	0	0	0	0	0	0	0
ENSG00000274918	0	1	0	1	0	0	9	5	0	0
ENSG00000274916	0	0	0	0	0	0	0	0	0	0
ENSG00000274915	0	0	0	0	0	0	0	0	0	0
ENSG00000274913	0	0	0	0	0	0	0	0	0	0
ENSG00000274911	46	33	29	41	37	71	3	1	0	0
ENSG00000274910	0	0	0	0	0	0	0	0	0	0
ENSG00000274904	0	0	0	0	0	0	0	0	0	0
ENSG00000274903	0	0	0	0	0	0	0	0	0	0
ENSG00000274902	1	0	2	2	2	2	2	6	0	4

ENSG00000274901	0	0	0	0	0	0	0	0	0	0
ENSG00000274900	0	0	0	0	0	0	0	0	0	0
ENSG00000274899	0	0	0	0	0	0	0	3	0	0
ENSG00000274898	0	0	0	0	0	0	1	4	0	0
ENSG00000274897	4	1	1	3	4	14	2	5	0	0
ENSG00000274895	0	0	0	0	0	0	0	0	0	0
ENSG00000274893	0	0	5	0	0	0	6	12	0	0
ENSG00000274892	0	0	0	0	0	0	0	2	0	0
ENSG00000274890	0	0	0	0	0	0	0	0	0	0
ENSG00000274888	0	0	0	0	0	0	0	0	0	0
ENSG00000274886	0	0	0	0	0	0	0	0	0	0
ENSG00000274885	0	0	0	0	0	0	0	3	0	0
ENSG00000274884	0	0	0	0	0	0	0	0	0	0
ENSG00000274883	0	0	0	0	0	0	0	6	0	0
ENSG00000274880	0	0	0	0	0	0	0	0	0	0
ENSG00000274879	0	0	0	0	0	0	5	0	0	0
ENSG00000274878	0	0	0	0	0	0	0	0	0	0
ENSG00000274877	4	3	5	3	2	11	34	8	0	4
ENSG00000274874	0	0	0	0	0	0	0	0	0	0
ENSG00000274872	0	0	0	0	0	0	0	0	0	0
ENSG00000274871	0	0	0	0	0	0	0	0	0	0
ENSG00000274869	0	1	0	0	0	0	4	0	0	0
ENSG00000274868	0	0	0	0	0	0	17	2	0	0
ENSG00000274867	0	0	0	0	0	0	0	0	0	0
ENSG00000274866	0	0	0	0	0	0	0	0	0	0
ENSG00000274864	0	0	0	0	0	0	0	0	0	0
ENSG00000274863	0	0	0	0	0	0	0	0	0	0
ENSG00000274862	0	1	5	6	1	11	246	17	24	292
ENSG00000274860	0	0	0	0	0	2	0	11	0	0
ENSG00000274859	0	0	0	0	0	0	0	0	0	0
ENSG00000274856	0	0	0	0	0	0	0	0	0	0
ENSG00000274855	0	0	0	0	0	0	0	0	0	0
ENSG00000274852	0	0	0	0	0	0	0	0	0	0
ENSG00000274849	0	1	1	1	0	5	3	5	0	6
ENSG00000274848	0	0	0	0	0	0	0	2	0	0
ENSG00000274845	0	0	0	0	0	0	0	0	0	0
ENSG00000274844	0	0	0	0	0	0	0	0	0	0
ENSG00000274841	0	0	0	0	0	0	2	0	0	0
ENSG00000274840	0	0	0	0	0	0	0	3	0	0
ENSG00000274838	0	0	0	0	0	0	0	0	0	0
ENSG00000274837	0	0	0	0	0	0	0	0	0	0
ENSG00000274836	0	0	0	0	0	0	0	0	0	0
ENSG00000274835	0	0	0	0	0	0	0	1	0	0
ENSG00000274834	0	0	0	0	0	0	1	0	0	0
ENSG00000274833	0	0	0	0	0	0	2	5	0	0
ENSG00000274832	0	0	0	0	0	0	0	0	0	0
ENSG00000274831	0	0	0	0	0	0	0	0	0	0
ENSG00000274828	43	33	67	20	26	55	133	62	0	0
ENSG00000274827	0	0	0	0	0	0	0	27	0	0
ENSG00000274825	3	0	2	0	3	1	1	0	0	0
ENSG00000274824	0	0	0	0	0	0	2	0	0	0
ENSG00000274823	0	0	0	0	0	0	0	0	0	0
ENSG00000274822	0	0	0	0	0	0	1	0	0	0
ENSG00000274820	0	0	0	0	0	0	0	0	0	0
ENSG00000274819	0	0	0	0	0	0	0	0	0	0
ENSG00000274818	2	3	10	2	0	10	18	10	0	0
ENSG00000274817	0	0	0	0	0	0	0	0	0	0

ENSG00000274816	0	2	0	0	0	0	1	0	0	0
ENSG00000274815	0	0	0	0	0	0	0	0	0	0
ENSG00000274814	0	0	0	0	0	0	0	0	0	0
ENSG00000274813	0	0	0	0	0	0	0	0	0	0
ENSG00000274810	0	0	0	0	0	0	0	0	0	0
ENSG00000274809	0	0	0	0	0	0	0	0	0	0
ENSG00000274808	282	300	414	294	238	775	72	36	0	0
ENSG00000274805	0	0	0	0	0	0	0	0	0	0
ENSG00000274802	0	0	0	0	0	0	0	0	0	0
ENSG00000274800	0	0	0	0	0	0	0	6	0	0
ENSG00000274799	0	1	2	0	0	0	0	1	0	0
ENSG00000274798	3	2	0	0	0	0	1	0	0	0
ENSG00000274797	0	0	0	0	0	0	2	0	0	0
ENSG00000274794	0	0	0	0	0	0	0	0	0	0
ENSG00000274793	0	0	0	0	0	0	0	0	0	0
ENSG00000274791	4	1	5	0	2	3	12	12	0	0
ENSG00000274790	0	0	0	0	0	0	0	0	0	0
ENSG00000274787	0	0	0	0	0	0	0	0	0	0
ENSG00000274784	0	0	0	0	0	0	0	0	0	0
ENSG00000274778	0	0	0	0	0	0	0	0	0	0
ENSG00000274777	0	0	0	0	0	0	0	0	0	0
ENSG00000274776	0	1	9	0	1	1	5	5	0	0
ENSG00000274775	0	0	0	0	0	0	4	5	0	0
ENSG00000274774	0	0	0	0	0	0	0	0	0	0
ENSG00000274773	0	0	0	0	0	0	0	0	0	0
ENSG00000274772	0	1	0	0	0	0	1	7	0	0
ENSG00000274770	0	0	0	0	0	0	0	0	0	0
ENSG00000274769	0	0	0	0	0	0	0	0	0	0
ENSG00000274767	0	0	0	0	0	0	4	4	0	0
ENSG00000274766	0	0	0	0	0	0	0	0	0	0
ENSG00000274765	0	0	0	0	0	0	0	0	0	0
ENSG00000274764	0	0	0	0	0	0	0	0	0	0
ENSG00000274762	0	0	0	0	0	0	0	0	0	0
ENSG00000274761	0	0	0	0	0	0	0	0	0	0
ENSG00000274760	0	0	0	0	0	0	0	0	0	0
ENSG00000274759	0	0	0	0	0	0	3	0	0	0
ENSG00000274758	0	0	0	0	0	0	0	0	0	0
ENSG00000274756	3	6	7	4	3	8	14	1	0	0
ENSG00000274755	0	0	0	0	0	0	0	0	0	0
ENSG00000274753	0	0	0	0	0	0	0	0	0	0
ENSG00000274752	2	0	0	0	0	2	11	1	0	0
ENSG00000274751	1	0	0	2	2	5	1	0	0	0
ENSG00000274750	8	13	23	12	13	38	15	0	0	0
ENSG00000274749	0	1	0	0	0	0	1	0	0	0
ENSG00000274748	0	0	0	0	0	0	0	0	0	0
ENSG00000274747	0	0	0	0	0	0	1	4	0	5
ENSG00000274744	0	3	0	0	0	0	3	0	0	0
ENSG00000274743	0	0	0	0	0	0	0	0	0	0
ENSG00000274742	0	0	0	0	0	0	0	0	0	0
ENSG00000274740	0	0	0	0	0	0	0	0	0	0
ENSG00000274737	1	1	3	1	0	1	7	2	0	0
ENSG00000274736	0	0	0	0	0	0	173	281	1	0
ENSG00000274735	13	77	28	57	8	24	95086	32784	272	2185
ENSG00000274732	0	0	0	0	0	0	0	0	0	0
ENSG00000274730	0	0	0	0	0	0	0	0	0	0
ENSG00000274728	0	0	0	0	0	0	0	0	0	0
ENSG00000274727	0	0	0	0	0	0	0	0	0	0

ENSG00000274725	5	1	4	1	0	8	4	4	0	0
ENSG00000274723	0	0	0	0	0	0	2	0	0	0
ENSG00000274721	3	0	7	2	3	1	3	9	0	0
ENSG00000274719	0	0	0	0	0	0	4	3	0	0
ENSG00000274718	0	0	0	0	0	0	2	0	0	0
ENSG00000274717	0	0	0	0	0	0	18	1	0	0
ENSG00000274716	0	0	0	0	0	0	0	0	0	0
ENSG00000274713	0	0	0	0	0	0	0	0	0	0
ENSG00000274712	16	21	39	8	9	38	47	38	0	0
ENSG00000274711	0	0	0	0	0	0	0	0	0	0
ENSG00000274709	0	0	0	0	0	0	0	0	0	0
ENSG00000274705	0	0	2	0	0	0	0	0	0	0
ENSG00000274704	0	0	0	0	0	0	0	0	0	0
ENSG00000274702	0	0	0	0	0	0	0	0	0	0
ENSG00000274701	0	0	0	0	0	0	0	0	0	0
ENSG00000274698	1	1	0	0	0	4	0	8	0	0
ENSG00000274697	0	0	0	0	0	0	0	0	0	0
ENSG00000274695	0	0	4	0	0	1	0	0	0	0
ENSG00000274694	0	0	0	0	0	0	0	0	0	0
ENSG00000274693	0	0	0	0	0	0	0	0	0	0
ENSG00000274691	0	0	0	0	0	0	0	0	0	0
ENSG00000274689	0	0	0	0	0	0	0	0	0	0
ENSG00000274688	0	0	0	0	0	0	0	0	0	0
ENSG00000274687	0	0	0	0	0	0	0	0	0	0
ENSG00000274686	0	0	0	0	0	2	0	0	0	0
ENSG00000274685	0	0	0	0	0	0	0	0	0	0
ENSG00000274683	0	0	0	0	0	0	0	0	0	0
ENSG00000274682	0	0	0	0	0	0	0	0	0	0
ENSG00000274680	6	5	8	3	1	13	8	1	0	0
ENSG00000274678	0	2	0	0	1	0	9	17	0	0
ENSG00000274677	1	2	1	1	3	7	6	0	0	0
ENSG00000274674	0	0	0	0	0	0	0	0	0	0
ENSG00000274673	0	0	0	0	0	0	0	0	0	0
ENSG00000274670	0	0	0	0	0	0	0	0	0	0
ENSG00000274668	0	0	0	0	0	0	0	0	0	0
ENSG00000274667	0	0	2	0	0	1	2	8	0	0
ENSG00000274666	0	0	0	0	0	0	1	0	0	0
ENSG00000274664	0	0	0	0	0	0	0	0	0	0
ENSG00000274663	0	0	0	0	0	0	0	0	0	0
ENSG00000274662	0	0	0	0	0	0	0	0	0	0
ENSG00000274661	0	0	0	0	0	0	0	0	0	0
ENSG00000274660	0	0	0	0	0	0	0	0	0	0
ENSG00000274659	0	0	0	0	0	0	1	0	0	0
ENSG00000274658	19	21	20	25	11	23	13	9	0	0
ENSG00000274656	0	0	0	0	0	0	0	0	0	0
ENSG00000274655	0	0	0	0	0	0	0	0	0	0
ENSG00000274654	0	0	0	0	0	0	0	0	0	0
ENSG00000274653	1	5	3	4	2	7	2	0	0	0
ENSG00000274652	0	0	0	0	0	0	0	0	0	0
ENSG00000274649	0	0	0	0	0	0	0	0	0	0
ENSG00000274648	0	0	0	0	0	0	1	0	0	0
ENSG00000274647	0	0	0	0	0	0	0	0	0	0
ENSG00000274645	0	0	0	0	0	0	0	0	0	0
ENSG00000274644	0	0	0	0	0	0	0	0	0	0
ENSG00000274642	4	4	11	3	4	15	148	18	0	0
ENSG00000274641	1	0	2	2	1	1	6	0	0	0
ENSG00000274640	0	0	0	0	0	0	0	0	0	0

ENSG00000274637	0	0	0	0	0	0	0	0	0	0
ENSG00000274635	0	0	0	0	0	0	0	0	0	0
ENSG00000274634	0	0	0	0	0	0	0	0	0	0
ENSG00000274633	0	0	0	0	0	0	0	0	0	0
ENSG00000274632	0	0	0	0	0	0	0	0	0	0
ENSG00000274631	0	0	0	0	0	0	0	0	0	0
ENSG00000274630	0	0	0	0	0	0	0	0	0	0
ENSG00000274629	0	0	0	0	0	0	1	0	0	0
ENSG00000274628	15	12	23	10	13	25	136	86	1	0
ENSG00000274627	50	44	67	31	34	120	50	40	0	0
ENSG00000274625	0	0	0	0	0	0	0	0	0	0
ENSG00000274624	0	0	0	0	0	0	31	4	0	0
ENSG00000274622	0	0	0	0	0	0	2	5	0	2
ENSG00000274621	0	0	0	0	1	0	0	0	0	0
ENSG00000274620	1	0	0	0	0	0	4	0	0	0
ENSG00000274618	0	0	0	0	0	0	10	0	0	0
ENSG00000274617	0	0	0	0	0	0	0	0	0	0
ENSG00000274615	227	301	518	216	214	618	488	182	1	8
ENSG00000274614	0	0	0	0	0	0	3	0	0	0
ENSG00000274612	0	0	0	0	0	0	0	0	0	0
ENSG00000274611	554	681	826	515	476	1429	132	70	0	0
ENSG00000274608	0	0	0	0	0	0	0	1	0	0
ENSG00000274607	0	1	0	0	0	0	0	0	0	0
ENSG00000274606	0	0	0	0	0	0	0	0	0	0
ENSG00000274605	56	47	70	36	29	132	40	21	0	0
ENSG00000274604	0	0	0	0	0	0	0	0	0	0
ENSG00000274603	0	0	0	0	0	0	0	0	0	0
ENSG00000274602	80	139	171	96	109	311	854	708	0	0
ENSG00000274601	0	0	0	0	0	0	0	0	0	0
ENSG00000274600	2	4	1	0	0	2	11	2	0	0
ENSG00000274599	0	0	0	0	0	1	0	0	0	0
ENSG00000274598	0	3	2	0	6	8	64	14	0	0
ENSG00000274596	0	0	0	0	0	0	0	0	0	0
ENSG00000274594	0	0	0	0	0	1	2	3	0	0
ENSG00000274592	0	0	0	0	0	0	0	0	0	0
ENSG00000274591	0	0	0	0	0	0	1	3	0	0
ENSG00000274590	0	0	0	0	0	0	0	0	0	0
ENSG00000274589	0	1	0	0	0	3	0	3	0	0
ENSG00000274588	0	0	0	0	0	0	4	4	0	0
ENSG00000274585	0	1	5	6	1	11	214	20	26	308
ENSG00000274584	20	19	39	14	16	18	42	10	0	0
ENSG00000274583	0	0	0	0	0	0	0	0	0	0
ENSG00000274582	0	0	0	0	0	0	0	0	0	0
ENSG00000274579	0	0	0	0	0	0	0	0	0	0
ENSG00000274578	0	0	0	0	0	0	0	0	0	0
ENSG00000274576	0	0	0	0	0	0	28	45	0	0
ENSG00000274572	0	0	0	0	0	0	0	0	0	0
ENSG00000274570	36	42	46	43	32	126	29	20	0	0
ENSG00000274569	0	0	0	0	0	0	0	0	0	0
ENSG00000274568	0	0	0	0	0	0	0	0	0	0
ENSG00000274565	3	3	3	4	4	6	13	31	0	0
ENSG00000274562	0	0	0	0	0	0	0	0	0	0
ENSG00000274561	5	6	14	9	10	20	48	72	0	0
ENSG00000274560	0	0	0	0	0	0	0	0	0	0
ENSG00000274559	21	28	43	21	23	50	83	20	0	0
ENSG00000274557	0	0	0	0	0	0	0	0	0	0
ENSG00000274555	0	0	0	1	0	0	0	0	0	0

ENSG00000274554	0	0	0	0	0	0	2	0	0	0
ENSG00000274553	10	16	33	8	8	35	5	0	0	0
ENSG00000274552	0	0	0	0	0	2	0	0	0	0
ENSG00000274551	0	0	0	0	0	0	0	0	0	0
ENSG00000274550	0	0	0	0	0	0	0	0	0	0
ENSG00000274549	0	0	0	0	0	0	0	0	0	0
ENSG00000274548	0	0	0	0	0	0	0	0	0	0
ENSG00000274547	0	0	0	0	0	0	0	0	0	0
ENSG00000274546	6	9	13	6	7	10	28	33	0	0
ENSG00000274544	0	0	0	0	0	0	0	0	0	0
ENSG00000274541	0	0	0	0	0	0	0	0	0	0
ENSG00000274537	0	0	0	0	0	0	0	0	0	0
ENSG00000274536	2	6	5	3	1	0	150	157	65	931
ENSG00000274535	0	0	0	0	0	0	1	0	0	0
ENSG00000274532	0	0	0	0	0	0	0	0	0	0
ENSG00000274529	0	0	0	0	0	0	0	0	0	0
ENSG00000274528	12	6	18	9	8	28	19	1	0	0
ENSG00000274527	0	0	0	0	0	0	0	0	0	0
ENSG00000274526	0	0	0	0	0	0	0	0	0	0
ENSG00000274525	0	0	0	0	1	0	0	0	0	0
ENSG00000274524	0	0	0	0	0	0	0	0	0	0
ENSG00000274523	529	523	734	474	435	1331	869	860	0	3
ENSG00000274521	0	0	0	0	0	0	0	0	0	0
ENSG00000274520	0	0	0	0	0	0	0	0	0	0
ENSG00000274519	0	0	0	0	0	0	14	1	0	9
ENSG00000274516	0	0	0	0	0	0	0	0	0	0
ENSG00000274515	3	1	1	0	4	10	1	2	0	0
ENSG00000274514	0	0	0	0	0	11	0	0	0	0
ENSG00000274512	458	580	732	410	409	1181	89	28	2	0
ENSG00000274510	0	0	0	0	0	0	0	1	0	0
ENSG00000274509	0	0	0	1	0	0	0	0	0	0
ENSG00000274508	0	0	0	0	0	0	20	0	0	0
ENSG00000274507	0	0	1	0	0	0	3	0	0	0
ENSG00000274505	0	0	0	0	0	0	1	2	0	0
ENSG00000274503	0	0	0	0	0	0	0	0	0	0
ENSG00000274502	0	0	0	0	0	0	0	0	0	0
ENSG00000274501	0	0	0	1	0	2	1	0	0	0
ENSG00000274500	0	0	0	0	0	0	0	0	0	0
ENSG00000274499	2	2	4	2	1	3	0	0	0	0
ENSG00000274496	0	0	0	0	0	0	0	0	0	0
ENSG00000274494	0	0	0	0	0	0	0	0	0	0
ENSG00000274493	0	0	0	0	0	0	0	0	0	0
ENSG00000274492	2	1	0	0	0	0	0	13	0	0
ENSG00000274491	0	0	0	0	0	0	0	0	0	0
ENSG00000274490	0	0	0	0	0	0	0	0	0	0
ENSG00000274489	0	0	0	0	0	0	0	0	0	0
ENSG00000274487	274	370	608	285	250	742	571	200	1	8
ENSG00000274486	0	1	0	0	0	0	0	0	0	0
ENSG00000274484	0	0	0	0	0	0	0	0	0	0
ENSG00000274483	0	2	4	1	0	1	17	0	0	0
ENSG00000274481	0	0	0	0	0	0	0	0	0	0
ENSG00000274479	0	0	0	0	0	0	0	0	0	0
ENSG00000274478	0	0	0	0	0	0	21	10	0	0
ENSG00000274475	0	1	0	1	0	7	1	0	0	0
ENSG00000274472	0	0	0	0	0	0	0	3	0	0
ENSG00000274471	43	43	68	29	32	74	40	27	0	0
ENSG00000274470	0	0	0	0	0	0	0	0	0	0

ENSG00000274469	0	0	0	0	0	0	0	0	0	0
ENSG00000274468	0	2	0	0	0	0	0	0	0	0
ENSG00000274467	0	0	0	0	0	0	0	0	0	0
ENSG00000274466	0	0	0	0	0	0	0	3	0	0
ENSG00000274464	0	0	0	0	0	0	0	0	0	0
ENSG00000274461	1	0	1	2	2	1	2	0	0	0
ENSG00000274460	1	2	5	10	2	8	35	3	0	0
ENSG00000274459	0	0	0	0	0	0	0	0	0	0
ENSG00000274458	0	0	0	0	0	0	0	0	0	0
ENSG00000274457	0	0	0	0	0	0	0	0	0	0
ENSG00000274455	0	1	2	1	0	0	0	0	0	0
ENSG00000274453	0	0	0	0	0	0	0	0	0	0
ENSG00000274452	0	0	4	2	0	5	182	18	21	230
ENSG00000274451	0	0	0	0	0	0	0	0	0	0
ENSG00000274450	0	0	0	0	0	0	0	0	0	0
ENSG00000274448	0	0	0	0	0	0	0	0	0	0
ENSG00000274447	0	0	0	0	0	0	1	0	0	0
ENSG00000274446	0	0	0	0	0	0	0	0	0	0
ENSG00000274445	0	0	0	0	0	0	0	0	0	0
ENSG00000274444	0	0	0	0	0	0	1	2	0	0
ENSG00000274443	1	0	0	0	0	1	0	4	0	0
ENSG00000274441	0	1	0	0	0	0	7	0	0	0
ENSG00000274433	0	0	0	0	0	0	0	0	0	0
ENSG00000274432	0	1	5	6	1	11	251	25	19	263
ENSG00000274431	0	0	0	0	0	0	0	0	0	0
ENSG00000274430	0	0	0	0	0	0	0	0	0	0
ENSG00000274428	10	5	27	3	3	25	1	2	0	0
ENSG00000274427	0	4	4	3	2	9	1	0	0	0
ENSG00000274426	0	0	0	0	0	0	6	1	0	0
ENSG00000274425	0	4	2	1	3	3	3	0	0	0
ENSG00000274424	0	0	0	0	0	0	0	0	0	0
ENSG00000274423	11	15	53	11	16	41	123	3	0	9
ENSG00000274422	0	0	3	2	0	2	11	0	0	0
ENSG00000274421	0	0	0	0	0	1	5	3	0	0
ENSG00000274420	0	0	0	0	0	0	0	0	0	0
ENSG00000274419	213	234	315	230	153	536	120	51	1	0
ENSG00000274417	0	0	0	0	0	0	0	0	0	0
ENSG00000274416	0	0	0	0	0	0	0	0	0	0
ENSG00000274415	0	0	0	0	0	0	0	0	0	0
ENSG00000274414	45	67	91	36	50	160	9	23	0	3
ENSG00000274409	0	0	0	0	0	0	0	0	0	0
ENSG00000274408	0	0	0	0	0	0	0	0	0	0
ENSG00000274403	3	4	10	1	3	8	12	17	0	0
ENSG00000274400	0	0	0	0	0	0	0	0	0	0
ENSG00000274399	0	0	0	0	0	0	0	0	0	0
ENSG00000274398	0	0	0	0	0	0	0	0	0	0
ENSG00000274397	0	0	0	0	0	0	7	0	0	0
ENSG00000274396	0	0	0	0	0	0	0	0	0	0
ENSG00000274395	0	0	0	0	0	0	0	0	0	0
ENSG00000274391	0	0	0	0	0	0	0	0	0	0
ENSG00000274390	0	0	0	0	0	0	0	0	0	0
ENSG00000274389	0	0	0	0	0	0	0	0	0	0
ENSG00000274388	0	0	0	0	0	0	0	0	0	0
ENSG00000274387	0	0	0	0	0	0	0	0	0	0
ENSG00000274386	0	1	1	1	1	0	0	3	0	0
ENSG00000274385	0	0	0	0	0	0	0	0	0	0
ENSG00000274383	0	0	0	1	0	0	5	1	0	0

ENSG00000274381	0	0	0	0	0	0	0	0	0	0
ENSG00000274380	0	0	0	0	0	0	3	0	0	0
ENSG00000274379	0	0	0	0	0	0	0	0	0	0
ENSG00000274378	2	0	0	0	2	0	0	3	0	0
ENSG00000274377	0	0	0	0	0	0	0	0	0	0
ENSG00000274376	0	0	0	0	0	2	1	4	0	0
ENSG00000274374	0	0	0	0	0	0	0	0	0	0
ENSG00000274373	0	0	0	0	0	0	0	0	0	0
ENSG00000274372	1	4	4	2	0	11	9	19	0	0
ENSG00000274370	0	0	0	0	0	1	15	19	0	0
ENSG00000274369	0	0	0	0	0	0	0	0	0	0
ENSG00000274368	2	0	0	1	0	0	0	0	0	0
ENSG00000274367	0	0	0	1	0	0	6	5	0	0
ENSG00000274366	0	0	0	0	0	0	0	0	0	0
ENSG00000274365	0	0	0	0	0	0	0	0	0	0
ENSG00000274364	0	0	0	0	0	0	0	0	0	0
ENSG00000274363	0	0	0	3	0	0	2	0	0	0
ENSG00000274361	0	0	0	0	0	0	0	0	0	0
ENSG00000274358	3	1	3	0	1	0	0	5	0	0
ENSG00000274357	0	0	0	0	0	0	0	0	0	0
ENSG00000274356	0	0	0	0	0	0	2	0	0	0
ENSG00000274355	0	2	0	0	0	0	0	0	0	0
ENSG00000274354	0	0	0	0	0	0	0	0	0	0
ENSG00000274353	0	0	0	0	0	0	0	0	0	0
ENSG00000274351	0	0	0	0	0	0	1	0	0	0
ENSG00000274350	0	0	0	0	0	0	0	0	0	0
ENSG00000274349	155	152	221	79	125	288	31	66	0	0
ENSG00000274347	0	0	0	0	0	0	0	0	0	0
ENSG00000274346	0	0	0	0	0	0	0	0	0	0
ENSG00000274344	0	0	0	0	0	0	0	0	0	0
ENSG00000274343	0	0	0	0	0	0	0	0	0	0
ENSG00000274342	0	0	0	0	0	0	0	0	0	0
ENSG00000274341	1	0	0	0	0	3	20	0	0	0
ENSG00000274340	0	1	0	0	0	0	2	0	0	0
ENSG00000274335	0	0	0	0	0	0	2	0	0	0
ENSG00000274333	421	338	635	261	307	843	187	51	0	0
ENSG00000274331	0	0	0	0	0	0	0	7	0	0
ENSG00000274330	2	1	0	0	0	0	19	0	0	0
ENSG00000274329	0	0	0	0	0	0	0	0	0	0
ENSG00000274328	0	0	0	0	0	0	3	0	0	0
ENSG00000274326	0	0	0	0	0	0	0	0	0	0
ENSG00000274325	0	0	0	0	0	0	0	0	0	0
ENSG00000274322	7	4	8	8	2	18	11	2	0	0
ENSG00000274321	0	0	0	0	0	0	0	0	0	0
ENSG00000274319	0	0	0	0	0	0	0	0	0	0
ENSG00000274317	1	3	7	1	1	3	11	3	0	0
ENSG00000274316	0	0	0	0	0	0	0	0	0	0
ENSG00000274315	13	13	19	13	17	30	8	7	0	0
ENSG00000274314	0	0	0	0	0	0	0	0	0	0
ENSG00000274312	0	0	0	0	0	0	3	0	0	0
ENSG00000274310	0	0	0	0	0	0	4	0	0	0
ENSG00000274309	0	0	0	0	0	0	0	0	0	0
ENSG00000274308	0	2	0	0	0	1	2	0	0	0
ENSG00000274307	1	0	2	1	3	0	52	32	0	0
ENSG00000274306	0	0	0	0	0	0	0	0	0	0
ENSG00000274304	0	0	0	0	0	0	0	0	0	0
ENSG00000274303	0	0	0	0	0	0	2	0	0	0

ENSG00000274302	0	0	0	0	0	0	0	0	0	0
ENSG00000274301	0	0	0	0	0	0	0	0	0	0
ENSG00000274300	2	1	2	3	2	7	0	4	0	0
ENSG00000274299	0	0	0	0	0	0	1	0	0	0
ENSG00000274298	0	0	0	0	0	0	0	0	0	0
ENSG00000274297	0	0	0	0	0	0	3	3	0	0
ENSG00000274295	0	0	0	0	0	0	0	0	0	0
ENSG00000274294	0	0	4	1	0	0	1	0	0	0
ENSG00000274293	0	0	0	0	0	0	0	0	0	0
ENSG00000274292	2	0	0	2	0	0	1	0	0	0
ENSG00000274291	0	0	0	0	0	0	0	0	0	0
ENSG00000274290	1	0	4	0	0	4	10	3	0	0
ENSG00000274289	0	0	0	0	0	0	2	0	0	0
ENSG00000274286	0	0	0	0	1	0	106	54	0	0
ENSG00000274284	0	0	0	0	0	0	0	14	0	0
ENSG00000274282	0	0	0	0	0	0	0	0	0	0
ENSG00000274281	31	32	114	24	20	52	145	33	0	12
ENSG00000274280	0	0	0	0	0	0	0	0	0	0
ENSG00000274279	0	0	0	0	0	0	0	0	0	0
ENSG00000274278	0	0	0	0	0	0	0	0	0	0
ENSG00000274277	0	2	1	1	0	0	0	0	0	0
ENSG00000274276	55	72	93	74	48	153	185	156	0	2
ENSG00000274275	0	0	0	0	0	0	0	3	0	0
ENSG00000274274	0	0	0	0	0	0	3	0	0	0
ENSG00000274272	3	15	21	10	0	11	8	7	0	0
ENSG00000274270	0	2	2	2	0	2	10	1	0	0
ENSG00000274269	0	0	0	0	0	0	0	0	0	0
ENSG00000274268	0	0	0	0	0	0	0	0	0	0
ENSG00000274267	0	0	0	1	0	0	2	0	0	0
ENSG00000274266	0	0	0	0	0	0	0	0	0	0
ENSG00000274265	88	75	105	80	77	219	170	142	6	23
ENSG00000274263	0	0	0	0	0	0	0	0	0	0
ENSG00000274261	0	0	0	0	0	0	0	0	0	0
ENSG00000274259	0	0	1	1	0	0	2	0	0	0
ENSG00000274258	0	0	0	0	0	0	3	0	0	0
ENSG00000274256	0	0	0	0	0	0	0	0	0	0
ENSG00000274255	0	0	0	0	1	0	0	0	0	0
ENSG00000274253	1	0	0	1	0	0	13	15	0	0
ENSG00000274252	0	0	1	1	1	2	45	14	0	0
ENSG00000274251	1	3	8	2	0	9	5	0	0	0
ENSG00000274250	0	0	0	0	0	0	0	0	0	0
ENSG00000274249	0	0	0	0	0	0	0	6	4	0
ENSG00000274248	0	0	0	1	0	0	3	14	0	0
ENSG00000274246	0	0	0	0	0	0	0	0	0	0
ENSG00000274245	0	0	0	0	0	0	104	13	0	13
ENSG00000274244	0	0	0	0	0	0	3	0	0	0
ENSG00000274242	0	0	0	0	0	0	1	4	0	0
ENSG00000274241	0	0	0	0	0	0	0	0	0	0
ENSG00000274238	1	3	6	0	0	2	0	0	0	0
ENSG00000274237	0	0	0	0	0	0	0	0	0	0
ENSG00000274235	0	0	0	0	0	0	10	0	0	0
ENSG00000274234	0	0	0	0	0	0	0	0	0	0
ENSG00000274231	0	0	0	0	0	0	0	0	0	0
ENSG00000274230	0	0	0	0	0	0	0	0	0	0
ENSG00000274228	0	0	0	0	0	0	0	0	0	0
ENSG00000274227	0	0	3	0	0	1	3	4	0	0
ENSG00000274226	225	233	354	234	200	595	46	11	0	0

ENSG00000274225	2	2	1	0	2	5	4	8	0	0
ENSG00000274223	0	0	0	0	0	0	0	0	0	0
ENSG00000274222	0	0	0	0	0	0	0	0	0	0
ENSG00000274220	3	5	8	2	6	3	2	8	0	0
ENSG00000274219	0	0	0	0	0	0	0	0	0	0
ENSG00000274216	0	0	0	0	0	0	0	0	0	0
ENSG00000274215	0	0	0	0	0	0	5	0	0	0
ENSG00000274214	0	0	0	0	1	0	0	0	0	0
ENSG00000274213	1	0	0	3	1	6	231	143	1	45
ENSG00000274212	0	0	0	0	0	0	0	0	0	0
ENSG00000274211	329	340	528	275	289	745	417	218	0	0
ENSG00000274210	0	0	0	0	1	0	14	0	0	0
ENSG00000274209	0	0	0	0	0	1	0	2	0	0
ENSG00000274207	0	0	0	0	0	0	0	0	0	0
ENSG00000274206	0	0	0	0	0	0	1	1	0	0
ENSG00000274204	1	0	0	2	1	1	7	15	0	2
ENSG00000274203	0	0	0	0	0	0	0	0	0	0
ENSG00000274200	0	0	0	0	0	0	0	0	0	0
ENSG00000274199	0	0	0	0	0	0	0	0	0	0
ENSG00000274198	0	0	0	0	0	0	0	0	0	0
ENSG00000274197	0	0	0	0	0	0	2	5	0	0
ENSG00000274196	0	0	0	0	0	0	0	0	0	0
ENSG00000274191	1	0	0	0	0	1	1	0	0	0
ENSG00000274190	0	0	0	0	0	0	0	0	0	0
ENSG00000274188	0	0	0	0	0	0	0	0	0	0
ENSG00000274186	0	0	1	0	0	0	0	8	0	0
ENSG00000274184	2	5	4	1	0	15	19	1	0	0
ENSG00000274183	6	0	3	3	0	0	7	2	0	0
ENSG00000274182	0	0	0	0	0	0	0	0	0	0
ENSG00000274181	0	0	0	0	0	0	0	0	0	0
ENSG00000274180	186	146	229	134	151	404	214	190	0	0
ENSG00000274178	0	0	0	0	0	0	0	0	0	0
ENSG00000274177	0	0	0	0	0	0	0	0	0	0
ENSG00000274173	75	78	151	50	60	174	565	106	0	0
ENSG00000274172	0	0	0	0	0	0	0	0	0	0
ENSG00000274170	0	0	0	0	0	0	0	0	0	0
ENSG00000274168	0	0	0	0	0	0	0	0	0	0
ENSG00000274167	0	0	0	0	0	0	1	12	0	0
ENSG00000274164	0	0	0	0	0	0	0	0	0	0
ENSG00000274162	0	0	0	0	1	0	0	0	0	0
ENSG00000274160	0	0	0	0	0	0	0	0	0	0
ENSG00000274159	0	0	0	0	0	0	0	7	0	0
ENSG00000274157	0	0	0	0	0	0	2	0	0	0
ENSG00000274156	0	2	0	1	1	1	17	0	0	0
ENSG00000274155	0	0	0	0	0	0	0	0	0	0
ENSG00000274154	0	0	0	0	0	0	0	0	0	0
ENSG00000274153	0	0	0	0	0	0	0	0	0	0
ENSG00000274151	0	0	0	0	0	0	0	0	0	1
ENSG00000274150	0	0	0	0	0	3	0	0	0	0
ENSG00000274149	0	0	0	0	0	0	0	0	0	0
ENSG00000274141	0	0	0	0	0	0	0	0	0	0
ENSG00000274140	0	0	0	0	0	0	0	0	0	0
ENSG00000274139	0	0	0	0	0	0	1	0	0	0
ENSG00000274136	0	0	0	0	0	0	0	0	0	0
ENSG00000274135	0	0	3	1	0	0	0	1	0	0
ENSG00000274134	0	0	0	0	0	0	0	0	0	0
ENSG00000274133	0	0	0	0	0	0	0	0	0	0

ENSG00000274128	0	0	0	0	0	0	0	7	0	0
ENSG00000274127	0	0	0	0	0	0	0	0	0	0
ENSG00000274124	0	2	2	0	1	0	0	5	0	0
ENSG00000274121	0	0	0	0	0	0	0	0	0	0
ENSG00000274118	0	0	0	0	0	0	0	0	0	0
ENSG00000274116	0	0	0	0	0	0	0	0	0	0
ENSG00000274115	0	0	0	1	0	0	1	0	0	0
ENSG00000274114	5	4	2	3	3	11	1	0	0	0
ENSG00000274111	0	0	0	0	0	0	0	0	0	0
ENSG00000274105	1	1	1	0	0	2	8	1	0	0
ENSG00000274104	1	3	3	1	3	9	54	44	0	0
ENSG00000274103	0	0	0	0	0	0	0	0	0	0
ENSG00000274102	0	0	0	0	0	0	0	0	0	0
ENSG00000274100	1	2	0	2	1	7	0	0	0	0
ENSG00000274099	0	0	0	0	0	0	0	6	0	0
ENSG00000274098	0	0	0	0	0	0	0	0	0	0
ENSG00000274097	0	0	0	0	0	0	0	0	0	0
ENSG00000274096	0	0	0	0	0	0	0	0	0	0
ENSG00000274095	0	0	0	0	0	0	0	0	0	0
ENSG00000274094	0	0	0	0	0	0	0	0	0	0
ENSG00000274093	0	0	0	0	0	0	0	0	0	0
ENSG00000274092	1	0	4	4	0	0	4	6	0	2
ENSG00000274091	0	0	0	0	0	0	0	0	0	0
ENSG00000274090	0	0	0	0	0	0	0	0	0	0
ENSG00000274088	0	0	0	0	0	0	0	0	0	0
ENSG00000274087	0	0	0	0	0	0	0	0	0	0
ENSG00000274086	0	0	0	0	0	0	0	0	0	0
ENSG00000274083	0	0	0	0	0	0	0	0	0	0
ENSG00000274080	0	1	0	3	0	0	0	0	0	0
ENSG00000274079	0	0	0	0	0	0	0	6	0	0
ENSG00000274077	0	0	0	0	0	0	0	0	0	0
ENSG00000274076	0	0	0	0	0	0	0	0	0	0
ENSG00000274075	0	0	0	0	0	0	0	0	0	0
ENSG00000274074	0	0	0	0	0	0	0	0	0	0
ENSG00000274072	0	0	0	0	0	0	0	0	0	0
ENSG00000274070	17	17	29	11	5	31	14	18	0	0
ENSG00000274069	0	0	0	0	0	0	0	0	0	0
ENSG00000274068	0	0	0	0	0	0	0	0	0	0
ENSG00000274067	0	0	0	0	0	0	0	0	0	0
ENSG00000274066	0	0	0	0	0	0	3	0	0	0
ENSG00000274065	0	0	0	0	0	0	0	0	0	0
ENSG00000274064	0	0	0	0	0	0	3	0	0	0
ENSG00000274062	0	1	5	6	1	11	224	26	25	281
ENSG00000274061	0	0	0	0	0	0	0	0	0	0
ENSG00000274060	0	0	0	0	0	0	24	1	1	0
ENSG00000274059	0	0	0	0	0	0	1	0	0	0
ENSG00000274057	2	3	0	0	1	0	0	0	0	0
ENSG00000274056	0	0	0	0	0	0	0	0	0	0
ENSG00000274055	0	0	0	0	0	0	0	0	0	0
ENSG00000274054	0	0	0	0	0	0	0	0	0	0
ENSG00000274052	0	0	0	0	0	0	0	4	0	0
ENSG00000274051	0	0	0	0	0	0	4	0	0	0
ENSG00000274049	0	0	0	0	0	0	0	0	0	0
ENSG00000274046	0	0	0	0	0	0	0	0	0	0
ENSG00000274044	17	35	123	12	12	26	19	31	0	4
ENSG00000274043	0	0	0	0	0	0	3	0	0	0
ENSG00000274038	0	1	1	0	0	0	19	0	0	0

ENSG00000274037	0	0	0	0	0	0	0	0	0	0
ENSG00000274034	0	0	0	0	0	0	0	0	0	0
ENSG00000274033	0	0	0	0	0	0	0	0	0	0
ENSG00000274031	0	0	0	0	2	0	3	1	0	0
ENSG00000274029	0	0	0	0	0	0	7	1	0	0
ENSG00000274028	0	0	0	0	0	0	0	0	0	0
ENSG00000274027	0	0	0	0	0	0	0	0	0	0
ENSG00000274026	86	87	122	65	87	205	10	13	0	0
ENSG00000274025	0	0	0	0	0	0	0	0	0	0
ENSG00000274024	0	2	0	0	0	0	0	0	0	0
ENSG00000274023	1	0	1	0	0	0	0	0	0	0
ENSG00000274022	0	0	0	0	0	0	0	0	0	0
ENSG00000274021	4	1	19	2	8	15	8	13	0	0
ENSG00000274020	56	45	83	57	58	155	177	90	4	26
ENSG00000274019	0	0	0	0	0	0	3	0	0	0
ENSG00000274017	0	0	0	0	0	0	0	0	0	0
ENSG00000274015	2	2	0	0	2	4	33	6	0	1
ENSG00000274014	0	0	0	0	0	0	0	0	0	0
ENSG00000274013	0	0	0	0	0	0	0	0	0	0
ENSG00000274012	0	0	0	0	0	0	0	0	0	0
ENSG00000274011	1	0	0	0	0	0	0	0	0	0
ENSG00000274010	1	2	0	1	0	3	0	0	0	0
ENSG00000274008	0	0	0	0	0	0	2	0	0	0
ENSG00000274006	0	0	0	0	0	0	0	0	0	0
ENSG00000274004	0	0	0	0	0	0	0	0	0	0
ENSG00000274002	0	0	0	0	0	0	4	6	0	0
ENSG00000274001	0	1	0	0	0	0	4	11	0	0
ENSG00000273999	0	0	0	0	0	0	0	0	0	0
ENSG00000273998	0	2	2	1	0	2	0	0	0	0
ENSG00000273997	0	0	0	0	0	0	0	0	0	0
ENSG00000273996	0	0	0	0	0	0	0	0	0	0
ENSG00000273994	0	0	0	0	0	0	0	0	0	0
ENSG00000273989	0	0	0	0	0	0	3	7	0	0
ENSG00000273988	0	0	0	0	0	0	0	0	0	0
ENSG00000273987	8	3	8	2	2	17	4	24	0	0
ENSG00000273986	0	0	0	0	0	0	0	3	0	0
ENSG00000273985	0	0	0	0	0	0	0	0	0	0
ENSG00000273983	4	0	18	2	0	2	13	12	0	0
ENSG00000273982	1	0	0	0	0	0	1	0	1	0
ENSG00000273981	0	0	0	0	0	0	0	0	0	0
ENSG00000273980	0	0	0	0	1	0	0	0	0	2
ENSG00000273979	2	0	0	1	0	0	2	0	0	0
ENSG00000273978	0	0	0	0	0	0	0	0	0	0
ENSG00000273976	0	3	9	0	2	5	2	2	0	0
ENSG00000273975	0	0	0	0	0	0	0	2	0	0
ENSG00000273974	0	0	0	0	0	0	0	0	0	0
ENSG00000273973	1	1	0	0	0	0	2	0	0	0
ENSG00000273972	2	1	7	2	3	6	18	21	0	0
ENSG00000273971	0	0	0	0	0	0	1	0	0	0
ENSG00000273970	0	0	9	1	0	0	6	0	0	4
ENSG00000273967	0	0	3	2	6	11	0	1	0	0
ENSG00000273966	0	0	0	0	0	0	0	0	0	0
ENSG00000273965	0	0	0	0	0	0	0	0	0	0
ENSG00000273964	0	0	0	0	0	0	0	0	0	0
ENSG00000273963	0	0	0	0	0	0	0	0	0	0
ENSG00000273962	0	0	0	0	0	0	10	17	0	0
ENSG00000273961	0	0	0	0	0	0	0	0	0	0

ENSG00000273960	0	0	0	0	0	0	0	0	0	0
ENSG00000273958	0	0	0	1	0	0	2	0	1	0
ENSG00000273957	0	0	0	0	0	0	0	0	0	0
ENSG00000273956	0	0	0	0	1	1	1	0	0	0
ENSG00000273952	0	0	0	0	0	0	0	0	0	0
ENSG00000273951	1	0	0	0	2	0	3	14	0	0
ENSG00000273950	0	0	0	0	0	0	6	0	0	0
ENSG00000273949	0	0	0	0	0	0	0	0	0	0
ENSG00000273948	0	0	0	0	0	0	0	0	0	0
ENSG00000273946	0	0	1	0	0	0	0	1	0	0
ENSG00000273945	0	0	0	0	0	0	0	0	0	0
ENSG00000273942	0	0	0	0	0	0	0	0	0	0
ENSG00000273941	0	0	0	0	0	0	0	0	0	0
ENSG00000273940	0	0	0	0	0	0	0	0	0	0
ENSG00000273939	0	0	0	0	0	0	0	0	0	0
ENSG00000273933	2	3	2	0	1	7	0	0	0	0
ENSG00000273932	0	0	0	0	0	0	0	0	0	0
ENSG00000273930	0	0	0	0	0	0	0	0	0	0
ENSG00000273927	0	0	0	0	0	0	0	2	0	0
ENSG00000273926	0	0	0	0	0	0	0	0	0	0
ENSG00000273925	0	0	0	0	0	0	3	0	0	0
ENSG00000273923	0	0	0	0	0	0	0	1	0	0
ENSG00000273920	0	0	0	0	0	0	0	1	0	0
ENSG00000273919	0	0	0	0	0	0	0	0	0	0
ENSG00000273917	0	0	0	0	0	0	0	0	0	0
ENSG00000273915	0	0	0	0	0	0	0	0	0	0
ENSG00000273913	1	0	0	1	0	4	0	3	0	0
ENSG00000273912	0	0	0	0	0	0	0	0	0	0
ENSG00000273910	0	0	0	0	0	0	0	0	0	0
ENSG00000273908	0	0	0	0	0	0	0	0	0	0
ENSG00000273907	0	0	2	0	0	2	0	0	0	0
ENSG00000273906	0	0	0	0	0	0	8	10	0	0
ENSG00000273904	0	0	0	0	0	0	2	0	0	0
ENSG00000273903	0	0	0	0	0	0	3	0	0	0
ENSG00000273902	0	0	0	0	0	0	0	0	0	0
ENSG00000273901	2	2	1	1	1	0	1	0	0	0
ENSG00000273900	0	0	0	0	0	0	3	0	0	0
ENSG00000273899	26	45	29	28	28	75	102	40	0	0
ENSG00000273898	0	0	0	0	0	0	0	0	0	0
ENSG00000273897	33	46	65	26	39	80	114	61	7	9
ENSG00000273895	0	0	0	0	0	0	0	0	0	0
ENSG00000273894	0	0	0	0	0	0	0	0	0	0
ENSG00000273893	3	3	3	4	0	3	46	44	0	0
ENSG00000273892	0	0	0	0	0	0	0	0	0	0
ENSG00000273891	7	9	8	2	3	15	14	7	0	0
ENSG00000273890	0	0	0	1	4	1	1	0	0	0
ENSG00000273888	61	69	102	85	46	199	80	652	2	10
ENSG00000273886	0	0	0	0	0	0	0	0	0	0
ENSG00000273885	0	0	0	0	0	0	0	0	0	0
ENSG00000273882	0	0	0	0	0	0	0	11	0	0
ENSG00000273879	0	0	0	0	0	0	0	0	0	0
ENSG00000273878	0	0	0	0	0	0	0	0	0	0
ENSG00000273877	0	0	0	0	0	0	4	14	0	0
ENSG00000273876	0	0	0	0	0	0	0	5	0	0
ENSG00000273874	0	0	0	0	0	0	0	3	0	0
ENSG00000273872	0	0	0	0	0	0	0	6	0	0
ENSG00000273870	0	2	1	0	2	0	0	0	0	0

ENSG00000273869	0	0	0	0	0	0	0	0	0	0
ENSG00000273868	0	0	0	0	0	0	0	0	0	0
ENSG00000273867	0	0	0	0	0	0	0	0	0	0
ENSG00000273866	0	0	0	0	1	0	1	2	0	0
ENSG00000273863	0	0	0	0	0	0	0	0	0	0
ENSG00000273862	0	0	0	0	0	0	0	0	0	0
ENSG00000273860	0	0	0	0	0	0	0	2	0	0
ENSG00000273858	0	0	0	0	0	0	0	0	0	0
ENSG00000273857	0	0	0	0	0	0	0	0	0	0
ENSG00000273855	0	0	0	0	0	0	0	0	0	0
ENSG00000273854	0	0	0	0	0	0	0	0	0	0
ENSG00000273853	0	0	0	0	0	0	0	0	0	0
ENSG00000273851	1	0	0	0	0	0	0	0	0	0
ENSG00000273849	0	0	0	0	0	0	0	0	0	0
ENSG00000273848	0	0	0	0	0	0	0	0	0	0
ENSG00000273846	0	0	0	0	0	0	0	0	0	0
ENSG00000273844	0	0	0	0	0	0	0	0	0	0
ENSG00000273842	0	0	0	0	0	0	0	0	0	0
ENSG00000273841	625	579	881	473	541	1459	1312	534	6	32
ENSG00000273840	0	0	0	0	0	0	0	0	0	0
ENSG00000273838	0	0	0	0	0	0	0	0	0	0
ENSG00000273837	0	0	0	0	0	0	55	0	2	7
ENSG00000273836	0	0	0	0	0	0	0	0	0	0
ENSG00000273835	0	0	0	0	0	0	0	0	0	0
ENSG00000273834	0	0	0	0	0	0	0	0	0	0
ENSG00000273832	0	0	0	0	0	0	0	0	0	0
ENSG00000273830	0	0	0	0	0	0	0	0	0	0
ENSG00000273829	0	0	0	0	0	0	0	0	0	0
ENSG00000273828	1	1	4	2	3	8	6	0	0	0
ENSG00000273826	0	0	0	0	0	0	0	0	0	0
ENSG00000273825	0	0	8	0	0	0	0	0	0	0
ENSG00000273824	0	0	0	0	0	0	3	0	0	0
ENSG00000273823	0	0	0	0	0	0	0	0	0	0
ENSG00000273821	0	2	0	1	1	1	0	4	0	0
ENSG00000273820	90	74	117	51	72	176	105	38	1	0
ENSG00000273819	0	0	0	0	0	0	0	0	0	0
ENSG00000273818	0	0	0	0	0	0	0	0	0	0
ENSG00000273817	0	0	0	0	0	0	0	0	0	0
ENSG00000273816	1	0	0	0	0	0	0	0	0	0
ENSG00000273813	0	0	0	0	0	0	0	0	0	0
ENSG00000273812	0	0	0	0	0	2	49	12	0	0
ENSG00000273811	0	0	0	1	0	0	1	0	0	0
ENSG00000273810	0	0	0	0	0	0	0	0	0	0
ENSG00000273809	0	0	0	0	0	0	0	0	0	0
ENSG00000273806	0	0	0	0	0	0	0	4	0	1
ENSG00000273805	0	0	0	0	0	0	0	4	0	0
ENSG00000273804	0	0	0	0	0	0	1	0	0	0
ENSG00000273802	34	48	93	45	46	140	19	0	0	0
ENSG00000273801	0	0	0	0	0	0	0	0	0	0
ENSG00000273800	0	0	0	0	0	0	0	0	0	0
ENSG00000273798	0	0	0	0	0	0	0	0	0	0
ENSG00000273797	0	0	0	0	0	0	19	26	0	2
ENSG00000273796	4	2	6	1	1	4	14	67	0	0
ENSG00000273792	0	0	0	0	2	0	0	0	0	0
ENSG00000273791	0	0	0	0	0	0	0	0	0	0
ENSG00000273790	0	0	0	0	0	0	0	0	0	0
ENSG00000273789	0	0	0	0	0	0	1	0	0	0

ENSG00000273788	0	0	0	0	0	0	0	0	0	0
ENSG00000273787	0	0	0	0	0	0	0	0	0	0
ENSG00000273786	1	0	0	0	0	0	0	0	0	0
ENSG00000273784	1	0	0	0	0	0	0	0	0	0
ENSG00000273783	1	2	3	4	1	4	3	4	0	0
ENSG00000273782	0	0	0	0	0	0	0	0	0	0
ENSG00000273781	0	0	0	0	0	0	0	0	0	0
ENSG00000273779	0	0	0	0	0	0	0	0	0	0
ENSG00000273778	0	0	0	0	0	0	0	0	0	0
ENSG00000273777	0	0	0	0	0	0	6	1	0	0
ENSG00000273776	0	0	0	0	0	0	0	0	0	0
ENSG00000273774	0	0	0	0	0	0	0	0	0	0
ENSG00000273773	0	0	0	0	0	0	0	0	0	0
ENSG00000273771	0	0	0	0	0	0	0	123	0	0
ENSG00000273769	1	1	0	0	0	0	0	5	0	0
ENSG00000273768	51	49	65	45	70	107	3969	85	105	1272
ENSG00000273767	0	0	0	0	0	0	0	0	0	0
ENSG00000273765	0	0	0	0	0	0	0	0	0	0
ENSG00000273763	1	0	0	1	2	1	1	6	0	0
ENSG00000273762	0	0	0	0	0	0	0	0	0	0
ENSG00000273760	370	391	643	366	361	1167	36	10	0	0
ENSG00000273759	4	4	3	5	1	6	43	33	0	0
ENSG00000273758	0	0	0	0	0	0	0	0	0	0
ENSG00000273756	0	2	6	0	0	0	2	0	0	0
ENSG00000273754	0	0	0	0	0	0	0	0	0	0
ENSG00000273751	0	0	0	0	0	0	0	0	0	0
ENSG00000273750	0	0	0	0	0	0	0	0	0	0
ENSG00000273749	2467	2144	3195	2309	2240	6608	4300	1551	7	153
ENSG00000273747	0	0	5	0	1	0	2	0	0	0
ENSG00000273746	0	0	0	0	0	0	0	0	0	0
ENSG00000273745	0	0	1	1	0	0	0	0	0	0
ENSG00000273744	0	0	0	0	0	0	0	0	0	0
ENSG00000273743	2	0	1	0	1	0	0	0	0	0
ENSG00000273742	0	0	0	0	0	0	2	0	0	0
ENSG00000273736	0	0	0	0	0	0	0	0	0	0
ENSG00000273734	0	0	0	0	0	0	0	0	0	0
ENSG00000273733	2	2	0	0	2	1	1	15	0	0
ENSG00000273732	0	0	0	0	0	0	0	0	0	0
ENSG00000273731	0	0	0	0	0	0	0	0	0	0
ENSG00000273729	2	4	9	1	0	3	25	24	0	6
ENSG00000273727	3	4	5	0	1	3	0	0	0	0
ENSG00000273725	0	0	0	0	0	0	0	1	0	0
ENSG00000273724	0	0	0	0	0	0	3	1	0	0
ENSG00000273723	5	0	6	2	2	3	51	8	0	0
ENSG00000273721	0	0	0	0	0	0	0	0	0	0
ENSG00000273720	0	0	0	0	0	0	0	0	0	0
ENSG00000273719	0	0	0	0	0	0	0	4	0	0
ENSG00000273718	0	0	0	0	0	0	0	0	0	0
ENSG00000273717	0	0	0	0	0	0	0	0	0	0
ENSG00000273712	4	3	3	6	2	6	1	0	0	0
ENSG00000273711	0	0	0	0	0	0	0	0	0	0
ENSG00000273710	0	0	0	0	0	0	0	0	0	0
ENSG00000273709	0	1	5	6	1	11	255	24	25	293
ENSG00000273706	0	0	3	0	0	0	0	10	0	0
ENSG00000273705	0	0	0	0	0	0	0	0	0	0
ENSG00000273704	0	0	0	0	0	0	0	0	0	0
ENSG00000273703	0	0	0	0	0	2	0	0	0	0

ENSG00000273702	2	4	18	5	5	12	15	41	0	1
ENSG00000273701	0	0	0	0	0	0	0	0	0	0
ENSG00000273700	0	0	0	0	0	0	0	0	0	0
ENSG00000273699	0	0	0	0	0	0	0	0	0	0
ENSG00000273698	0	0	0	0	0	0	0	0	0	0
ENSG00000273697	0	0	0	0	0	0	0	0	0	0
ENSG00000273696	0	0	0	0	0	0	0	9	0	0
ENSG00000273695	0	0	0	0	0	0	0	0	0	0
ENSG00000273694	0	0	0	0	0	0	2	0	0	0
ENSG00000273693	0	0	0	0	1	0	0	0	0	0
ENSG00000273692	0	0	0	0	0	0	0	0	0	0
ENSG00000273691	1	2	0	0	0	0	51	13	0	0
ENSG00000273690	0	0	0	0	0	0	3	0	0	0
ENSG00000273689	0	0	0	0	0	0	3	0	0	0
ENSG00000273687	0	0	5	1	0	0	5	11	0	0
ENSG00000273685	0	0	0	0	0	0	0	0	0	0
ENSG00000273684	0	0	0	0	0	0	0	0	0	0
ENSG00000273682	0	0	0	0	1	4	0	0	0	0
ENSG00000273680	2	1	5	0	0	0	9	8	0	0
ENSG00000273679	0	1	7	0	0	0	5	0	0	0
ENSG00000273678	0	0	0	0	0	0	0	0	0	0
ENSG00000273677	0	0	0	0	0	0	0	0	0	0
ENSG00000273676	0	0	0	0	0	0	0	0	0	0
ENSG00000273675	0	0	0	0	0	0	1	10	0	0
ENSG00000273674	2	0	4	0	0	3	1	7	0	0
ENSG00000273672	0	0	0	0	0	0	0	1	0	0
ENSG00000273669	0	1	0	0	0	3	13	12	0	0
ENSG00000273668	0	0	0	0	0	0	2	0	0	0
ENSG00000273667	0	0	0	0	0	0	0	0	0	0
ENSG00000273665	0	0	0	0	0	0	0	0	0	0
ENSG00000273664	2	2	0	0	0	1	0	2	0	0
ENSG00000273663	0	0	0	0	0	0	0	0	0	0
ENSG00000273662	0	2	0	0	0	0	0	4	0	0
ENSG00000273659	0	0	0	0	0	2	0	0	0	0
ENSG00000273658	0	0	2	1	0	4	14	1	0	0
ENSG00000273657	0	0	0	0	0	0	0	0	0	0
ENSG00000273654	28	36	84	27	26	113	106	32	0	0
ENSG00000273653	0	0	0	0	0	0	0	0	0	0
ENSG00000273650	0	0	0	0	0	0	1	10	0	0
ENSG00000273648	0	0	0	0	0	0	0	0	0	0
ENSG00000273644	0	0	0	0	0	0	0	0	0	0
ENSG00000273643	0	0	0	0	0	0	0	0	0	0
ENSG00000273640	0	0	0	0	0	0	0	0	0	0
ENSG00000273639	0	0	0	0	0	0	0	0	0	0
ENSG00000273637	0	0	0	0	0	0	0	0	0	0
ENSG00000273634	0	0	0	0	0	0	0	0	0	0
ENSG00000273632	0	0	0	0	0	0	0	0	0	0
ENSG00000273631	0	0	0	0	0	0	0	0	0	0
ENSG00000273630	0	0	0	0	0	0	0	0	0	0
ENSG00000273629	0	0	0	0	0	0	0	0	0	0
ENSG00000273628	26	31	42	12	14	46	70	69	4	0
ENSG00000273627	0	0	0	0	0	0	0	0	0	0
ENSG00000273626	0	0	0	0	0	0	0	0	0	0
ENSG00000273625	0	0	1	1	0	2	0	0	0	0
ENSG00000273623	0	0	0	0	0	0	0	0	0	0
ENSG00000273621	0	0	0	0	0	0	0	0	0	0
ENSG00000273619	6	7	6	6	1	12	5	14	0	0

ENSG00000273618	0	0	0	1	0	6	0	1	0	0
ENSG00000273617	0	0	0	0	0	0	0	0	0	0
ENSG00000273615	2	8	9	1	0	4	1	0	0	0
ENSG00000273614	0	0	0	0	0	0	0	0	0	0
ENSG00000273613	0	0	0	0	0	0	0	0	0	0
ENSG00000273612	0	0	0	0	0	0	0	0	0	0
ENSG00000273611	197	183	366	230	169	547	709	430	3	3
ENSG00000273610	0	0	0	0	0	0	0	0	0	0
ENSG00000273609	0	0	6	0	0	0	0	0	0	0
ENSG00000273606	0	0	0	0	0	0	0	0	0	0
ENSG00000273604	176	127	237	160	125	413	313	596	0	0
ENSG00000273600	0	0	0	0	0	0	0	0	0	0
ENSG00000273599	32	48	51	23	34	105	96	32	0	0
ENSG00000273598	42	50	74	46	47	130	31	12	0	0
ENSG00000273597	0	0	0	1	1	0	11	0	0	0
ENSG00000273596	0	0	0	0	0	0	0	0	0	0
ENSG00000273595	0	0	0	0	0	0	0	0	0	0
ENSG00000273593	0	0	0	0	0	0	0	0	0	0
ENSG00000273591	0	0	0	0	0	0	0	0	0	0
ENSG00000273590	137	97	240	117	165	352	279	330	0	0
ENSG00000273589	0	0	0	0	0	0	0	0	0	0
ENSG00000273588	0	0	0	0	0	0	0	0	0	0
ENSG00000273587	0	0	0	0	0	0	0	0	0	0
ENSG00000273586	0	0	0	0	0	0	0	0	0	0
ENSG00000273585	0	0	0	0	0	0	0	0	0	0
ENSG00000273584	0	0	0	0	0	0	2	17	0	0
ENSG00000273583	0	0	0	0	0	0	0	0	0	0
ENSG00000273582	0	0	0	0	0	0	7	0	0	0
ENSG00000273581	0	0	0	0	0	0	0	6	0	0
ENSG00000273580	0	0	5	0	0	1	1	0	0	0
ENSG00000273577	0	0	0	0	0	0	0	0	0	0
ENSG00000273576	20	14	26	24	18	35	145	45	0	17
ENSG00000273573	0	0	0	0	0	0	0	0	0	0
ENSG00000273571	0	0	0	0	0	0	0	0	0	0
ENSG00000273570	0	0	0	0	0	0	0	0	0	0
ENSG00000273569	0	0	0	0	0	0	0	0	0	0
ENSG00000273568	11	7	16	15	8	36	10	7	0	0
ENSG00000273567	0	0	0	0	0	0	0	0	0	0
ENSG00000273566	0	0	0	0	0	0	0	0	0	0
ENSG00000273565	2	0	0	1	1	0	4	0	0	0
ENSG00000273561	0	0	0	0	0	0	0	0	0	0
ENSG00000273560	0	0	0	0	0	0	0	0	0	0
ENSG00000273559	344	324	563	303	341	982	2283	838	4	42
ENSG00000273558	0	0	0	0	0	0	0	19	0	0
ENSG00000273557	0	3	0	0	0	0	1	1	0	0
ENSG00000273556	0	0	0	0	0	0	0	1	0	0
ENSG00000273555	0	0	0	0	0	0	2	0	0	0
ENSG00000273553	0	0	0	0	0	0	0	0	0	0
ENSG00000273552	0	0	0	0	0	0	0	0	0	0
ENSG00000273551	0	0	0	0	0	0	1	2	0	0
ENSG00000273550	0	0	0	0	0	0	0	3	0	0
ENSG00000273549	0	0	0	0	0	0	0	5	0	0
ENSG00000273547	0	0	0	0	0	0	1	0	0	0
ENSG00000273545	0	0	0	0	0	0	0	0	0	0
ENSG00000273544	0	0	0	0	0	0	0	0	0	0
ENSG00000273542	8	41	53	21	9	61	67	30	0	0
ENSG00000273541	0	0	0	0	0	0	0	0	0	0

ENSG00000273540	1	0	0	0	0	0	0	0	0	0
ENSG00000273537	0	0	0	0	0	0	0	0	0	0
ENSG00000273536	0	0	0	0	0	0	0	0	0	0
ENSG00000273534	0	0	0	0	0	0	0	0	0	0
ENSG00000273531	0	0	0	0	0	0	0	0	0	0
ENSG00000273530	0	0	0	0	0	0	0	0	0	0
ENSG00000273527	0	0	0	0	0	0	0	0	0	0
ENSG00000273524	0	0	0	0	0	3	0	1	0	0
ENSG00000273523	0	0	0	0	0	0	8	10	0	0
ENSG00000273521	8	7	9	8	7	30	15	11	0	0
ENSG00000273520	42	39	41	47	32	163	18	15	0	0
ENSG00000273516	0	0	0	0	0	0	0	0	0	0
ENSG00000273515	0	0	0	0	0	0	0	0	0	0
ENSG00000273514	2	6	10	7	0	8	13	0	0	0
ENSG00000273513	374	415	570	373	314	1032	139	59	1	0
ENSG00000273512	0	0	0	0	0	0	0	0	0	0
ENSG00000273509	0	0	1	0	0	0	0	0	0	0
ENSG00000273507	0	0	0	0	1	0	2	9	0	0
ENSG00000273504	0	0	0	0	0	0	0	0	0	0
ENSG00000273500	0	0	0	0	0	0	0	0	0	0
ENSG00000273499	0	0	0	0	0	0	0	0	0	0
ENSG00000273497	0	0	0	0	0	0	0	0	0	0
ENSG00000273493	0	0	0	0	0	0	0	0	0	0
ENSG00000273492	1	0	0	1	0	0	2	10	0	0
ENSG00000273489	4	1	4	0	2	2	48	10	0	0
ENSG00000273488	4	7	22	5	2	7	0	3	0	0
ENSG00000273487	2	2	3	0	3	4	8	1	0	0
ENSG00000273486	9	10	5	5	5	39	11	18	0	0
ENSG00000273485	0	0	0	0	0	0	2	0	0	0
ENSG00000273484	0	0	0	0	0	0	0	0	0	0
ENSG00000273483	3	0	4	2	3	0	21	9	0	0
ENSG00000273481	0	0	2	0	0	0	7	0	0	0
ENSG00000273478	10	14	3	5	7	15	30	4	0	0
ENSG00000273477	3	4	0	1	0	1	2	20	0	0
ENSG00000273476	0	0	0	0	0	2	2	0	0	0
ENSG00000273474	0	0	0	0	0	0	3	0	0	0
ENSG00000273473	1	0	0	0	0	0	0	0	0	0
ENSG00000273472	2	3	8	1	0	8	30	21	0	0
ENSG00000273471	0	0	0	0	0	0	0	0	0	0
ENSG00000273466	0	0	0	0	0	0	2	2	0	0
ENSG00000273464	0	0	0	0	0	0	29	10	0	0
ENSG00000273461	0	0	0	0	0	0	0	0	0	0
ENSG00000273456	6	6	4	7	5	13	47	48	0	0
ENSG00000273455	0	0	0	0	0	0	3	0	0	0
ENSG00000273454	2	8	2	0	4	14	1	0	0	0
ENSG00000273451	1	0	0	2	0	0	3	0	0	0
ENSG00000273450	0	2	5	4	2	5	49	9	0	0
ENSG00000273449	0	1	14	2	5	4	38	6	0	0
ENSG00000273448	59	95	171	57	55	162	89	817	4	9
ENSG00000273447	4	8	12	8	5	27	9	5	0	0
ENSG00000273445	0	4	0	0	0	0	97	3	0	0
ENSG00000273444	0	0	0	0	0	0	0	0	0	0
ENSG00000273443	2	2	4	2	1	3	8	0	0	0
ENSG00000273442	1	0	0	1	1	2	1	0	0	0
ENSG00000273437	2	1	3	1	0	8	48	48	0	0
ENSG00000273434	0	0	0	0	0	0	0	0	0	0
ENSG00000273433	0	0	0	0	0	0	0	0	0	0

ENSG00000273432	2	4	8	5	4	5	18	8	0	0
ENSG00000273428	0	0	0	1	0	0	20	12	0	0
ENSG00000273424	0	0	0	0	0	0	7	1	0	0
ENSG00000273423	0	0	0	0	0	0	0	0	0	0
ENSG00000273420	0	0	0	0	0	0	0	0	0	0
ENSG00000273419	2	1	4	3	1	2	8	0	0	0
ENSG00000273416	1	0	0	0	0	1	4	3	0	0
ENSG00000273415	56	22	63	46	46	135	0	0	0	0
ENSG00000273413	0	0	0	0	0	0	0	0	0	0
ENSG00000273409	0	0	0	0	0	0	0	4	0	0
ENSG00000273408	0	0	0	0	0	0	0	0	0	0
ENSG00000273407	0	0	0	0	0	0	3	0	0	0
ENSG00000273406	0	0	0	0	0	0	4	0	0	0
ENSG00000273403	0	0	0	0	0	0	4	1	0	0
ENSG00000273402	0	0	1	0	0	0	10	1	0	0
ENSG00000273399	0	0	0	1	0	2	11	0	0	0
ENSG00000273398	0	0	0	0	0	0	0	0	0	0
ENSG00000273396	0	0	0	0	0	0	0	1	0	0
ENSG00000273394	0	0	0	0	0	0	1	0	0	0
ENSG00000273391	5	1	2	1	3	1	15	12	0	0
ENSG00000273389	0	0	0	2	0	0	3	2	0	0
ENSG00000273388	0	0	0	0	0	0	11	5	0	0
ENSG00000273387	3	1	4	1	6	25	21	4	0	0
ENSG00000273384	0	0	0	0	0	0	0	0	0	0
ENSG00000273382	3	4	14	9	3	29	6	19	0	0
ENSG00000273381	7	14	16	11	5	16	8	3	0	0
ENSG00000273377	0	0	0	0	0	0	0	0	0	0
ENSG00000273375	0	1	0	0	2	0	15	30	0	0
ENSG00000273374	0	0	0	2	0	6	1	0	0	0
ENSG00000273373	6	5	12	3	4	13	229	50	0	0
ENSG00000273372	0	0	0	0	0	0	1	2	0	0
ENSG00000273370	0	0	0	0	0	0	0	0	0	0
ENSG00000273369	2	2	1	0	1	5	3	6	0	0
ENSG00000273368	0	0	0	0	0	2	3	2	0	0
ENSG00000273367	2	3	0	1	3	1	0	0	0	0
ENSG00000273366	4	4	8	4	4	5	3	0	0	0
ENSG00000273365	0	0	0	0	0	0	0	0	0	0
ENSG00000273363	0	0	0	0	0	0	6	2	0	0
ENSG00000273362	0	0	0	0	0	0	0	0	0	0
ENSG00000273361	1	0	1	0	0	0	0	0	0	0
ENSG00000273360	0	0	0	0	0	0	0	0	0	0
ENSG00000273356	6	3	6	2	3	6	151	29	0	0
ENSG00000273355	2	0	1	0	0	2	7	0	0	0
ENSG00000273353	0	1	0	0	0	0	0	0	0	0
ENSG00000273352	1	1	0	0	0	0	10	6	0	0
ENSG00000273350	21	32	39	11	37	64	4	0	0	0
ENSG00000273348	2	0	0	0	0	0	6	2	0	0
ENSG00000273345	2	5	0	1	1	1	8	2	1	0
ENSG00000273344	23	17	23	14	20	46	71	83	0	0
ENSG00000273343	1	3	3	4	0	0	1	1	0	0
ENSG00000273342	0	0	0	1	0	0	2	3	0	0
ENSG00000273341	1	3	0	0	0	0	10	20	0	0
ENSG00000273340	7	10	11	3	5	29	16	1	0	0
ENSG00000273338	0	0	4	0	0	0	15	18	0	8
ENSG00000273336	0	1	1	0	0	0	0	0	0	0
ENSG00000273335	0	0	2	1	0	0	2	0	0	0
ENSG00000273333	0	0	0	0	0	0	1	0	0	0

ENSG00000273331	0	0	0	0	0	0	0	0	0	0
ENSG00000273330	0	0	0	0	0	0	0	0	0	0
ENSG00000273329	16	12	27	20	14	44	41	49	0	0
ENSG00000273328	0	6	2	1	1	13	8	2	1	0
ENSG00000273327	2	0	3	0	0	0	0	0	0	0
ENSG00000273325	0	0	0	0	0	0	2	4	0	0
ENSG00000273321	0	0	0	0	0	0	2	2	0	0
ENSG00000273320	0	0	2	0	0	3	0	0	0	0
ENSG00000273319	0	0	3	1	1	0	184	14	0	0
ENSG00000273314	12	14	17	6	7	28	43	55	0	9
ENSG00000273313	0	0	0	1	0	0	0	0	0	0
ENSG00000273312	3	0	4	1	0	0	0	1	0	0
ENSG00000273311	33	40	71	37	41	122	19	27	0	0
ENSG00000273308	10	7	0	2	2	15	7	4	0	0
ENSG00000273307	0	0	0	0	0	0	0	0	0	0
ENSG00000273306	22	18	31	14	12	34	37	35	0	0
ENSG00000273305	0	0	0	0	0	0	6	3	0	0
ENSG00000273303	0	0	0	0	0	0	0	0	0	0
ENSG00000273302	2	4	2	9	1	5	0	0	0	0
ENSG00000273301	0	0	0	0	0	0	2	5	0	0
ENSG00000273300	0	0	0	0	0	0	0	0	0	0
ENSG00000273299	0	0	0	0	0	0	0	0	0	0
ENSG00000273297	46	33	58	26	21	80	3	9	0	1
ENSG00000273295	0	0	0	0	0	4	26	0	0	0
ENSG00000273294	0	0	0	0	0	0	0	0	0	0
ENSG00000273293	0	0	0	0	1	0	0	0	0	0
ENSG00000273291	1	0	0	0	0	0	11	2	0	0
ENSG00000273289	2	0	5	3	0	0	0	2	0	0
ENSG00000273288	0	0	0	0	0	0	0	0	0	0
ENSG00000273287	0	0	0	1	0	0	0	3	0	0
ENSG00000273284	1	2	0	0	2	8	73	17	0	3
ENSG00000273283	0	0	0	0	0	0	0	0	0	0
ENSG00000273281	38	34	66	30	41	72	7	9	0	0
ENSG00000273275	0	0	0	0	0	0	0	0	0	0
ENSG00000273274	0	0	0	2	0	0	1	29	0	0
ENSG00000273272	0	0	0	0	0	2	0	0	0	0
ENSG00000273271	3	3	6	2	5	7	80	65	0	1
ENSG00000273270	62	54	76	55	42	151	29	56	1	5
ENSG00000273269	0	0	0	0	0	0	0	0	0	0
ENSG00000273267	0	0	0	0	0	0	1	9	0	0
ENSG00000273265	0	0	0	0	0	0	5	5	0	0
ENSG00000273264	0	0	0	0	0	0	2	8	0	0
ENSG00000273262	0	0	0	0	0	0	0	0	0	0
ENSG00000273261	0	0	1	0	0	2	12	3	0	0
ENSG00000273259	0	0	0	0	1	0	10	4	0	0
ENSG00000273258	0	0	0	0	0	0	1	0	0	0
ENSG00000273257	3	1	0	1	0	1	2	5	0	0
ENSG00000273255	0	0	0	0	0	0	0	0	0	0
ENSG00000273254	0	0	0	0	0	0	4	0	0	0
ENSG00000273253	6	5	2	5	3	8	3	8	0	0
ENSG00000273252	0	0	0	0	0	0	0	0	0	0
ENSG00000273249	2	1	0	0	0	3	2	0	0	0
ENSG00000273248	0	0	0	0	0	0	0	0	0	0
ENSG00000273247	15	15	12	6	10	28	54	27	1	0
ENSG00000273245	2	1	1	0	4	1	1	1	0	0
ENSG00000273244	0	0	0	0	0	0	0	0	0	0
ENSG00000273243	1	0	10	2	1	8	4	7	0	0

ENSG00000273240	1	0	0	0	1	0	2	0	0	0
ENSG00000273238	0	0	0	0	0	0	0	10	0	0
ENSG00000273237	2	7	21	5	9	23	166	8	0	11
ENSG00000273234	1	1	2	2	4	2	4	0	0	0
ENSG00000273233	1	0	0	1	0	0	0	0	0	0
ENSG00000273232	0	0	0	0	0	0	0	1	0	0
ENSG00000273230	46	26	58	23	33	85	11	5	0	0
ENSG00000273228	0	0	0	0	0	0	0	0	0	0
ENSG00000273226	2	2	5	7	0	7	39	49	0	0
ENSG00000273225	1	0	0	2	0	0	0	0	0	0
ENSG00000273221	0	0	0	0	0	0	0	0	0	0
ENSG00000273219	0	0	0	0	0	0	0	0	0	0
ENSG00000273218	1	4	6	0	3	11	7	21	0	0
ENSG00000273217	0	0	0	0	0	0	0	0	0	0
ENSG00000273216	2	0	0	0	0	1	2	6	0	0
ENSG00000273214	0	0	0	0	0	0	0	0	0	0
ENSG00000273213	0	0	0	0	0	0	0	0	0	0
ENSG00000273212	0	2	0	0	0	6	2	0	0	0
ENSG00000273211	0	0	0	0	0	0	0	0	0	0
ENSG00000273210	1	0	2	0	0	0	4	0	0	0
ENSG00000273209	0	2	0	0	0	0	6	2	0	0
ENSG00000273204	0	0	0	0	0	0	0	0	0	0
ENSG00000273203	6	6	11	2	6	9	3	5	0	0
ENSG00000273199	2	3	0	2	0	0	3	2	0	0
ENSG00000273198	0	0	0	0	0	0	1	0	0	0
ENSG00000273196	1	0	0	0	0	1	0	0	0	0
ENSG00000273193	1	0	0	0	0	0	0	2	0	0
ENSG00000273192	0	0	0	0	0	0	0	0	0	0
ENSG00000273189	0	0	0	0	0	0	7	3	0	0
ENSG00000273188	4	4	11	4	3	7	2	7	0	0
ENSG00000273186	7	14	25	9	8	20	1	12	0	0
ENSG00000273185	1	0	0	0	0	0	1	0	0	0
ENSG00000273184	0	0	0	0	0	0	0	0	0	0
ENSG00000273183	0	0	1	1	3	3	18	5	0	0
ENSG00000273181	0	2	0	0	0	0	0	0	0	0
ENSG00000273180	0	0	0	0	1	0	0	0	0	0
ENSG00000273179	5	2	2	0	0	2	18	11	0	0
ENSG00000273177	0	0	0	0	0	0	2	0	0	0
ENSG00000273176	0	0	0	0	0	0	6	0	0	0
ENSG00000273175	0	0	0	0	0	0	1	0	0	0
ENSG00000273174	3	10	2	5	7	7	22	26	0	0
ENSG00000273173	0	1	0	0	1	0	2	6	0	0
ENSG00000273172	0	0	0	0	0	0	1	5	0	0
ENSG00000273171	0	0	0	0	0	0	0	0	0	0
ENSG00000273170	5	4	12	3	4	14	5	0	0	0
ENSG00000273167	0	0	0	0	0	0	0	0	0	0
ENSG00000273165	0	0	1	0	0	0	2	0	0	0
ENSG00000273164	0	1	0	0	3	0	0	0	0	0
ENSG00000273162	0	2	2	4	1	5	0	0	0	0
ENSG00000273160	0	0	0	1	0	0	1	1	0	0
ENSG00000273156	0	0	0	0	0	0	23	57	0	8
ENSG00000273155	0	0	0	0	0	0	0	0	0	0
ENSG00000273154	0	0	6	0	0	1	0	0	0	0
ENSG00000273153	0	0	0	0	0	0	0	9	0	0
ENSG00000273151	33	46	53	37	54	140	33	4	0	0
ENSG00000273149	0	0	0	0	0	0	0	0	0	0
ENSG00000273148	11	29	30	9	13	25	19	8	0	0

ENSG00000273145	2	4	7	4	2	19	27	5	0	0
ENSG00000273143	0	0	0	0	0	2	23	11	0	0
ENSG00000273142	36	55	84	43	30	131	48	77	0	0
ENSG00000273141	0	0	0	0	0	0	0	0	0	0
ENSG00000273139	0	2	0	0	0	0	0	2	0	0
ENSG00000273138	0	0	0	0	0	0	0	0	0	0
ENSG00000273137	8	14	9	8	1	7	2	16	0	0
ENSG00000273136	2035	2129	3261	1744	1890	5504	1618	420	4	3
ENSG00000273133	0	0	1	0	0	4	3	1	0	0
ENSG00000273132	5	16	16	11	6	19	13	0	0	0
ENSG00000273129	0	1	2	0	2	9	43	4	0	0
ENSG00000273125	0	1	4	0	0	3	1	25	0	0
ENSG00000273124	0	0	0	0	0	0	6	0	0	0
ENSG00000273123	0	0	0	0	0	3	19	10	0	0
ENSG00000273119	0	0	0	0	0	0	0	0	0	0
ENSG00000273118	0	0	0	0	0	0	0	0	0	0
ENSG00000273117	8	3	4	2	3	12	14	15	0	0
ENSG00000273115	0	0	0	0	0	0	1	4	0	0
ENSG00000273113	14	28	41	12	6	50	4	0	0	0
ENSG00000273112	0	0	0	0	0	0	1	0	0	0
ENSG00000273111	0	0	0	0	0	0	0	0	0	0
ENSG00000273110	0	0	0	0	0	0	0	0	0	0
ENSG00000273108	2	11	7	3	1	14	6	8	0	0
ENSG00000273107	0	0	0	1	1	0	4	4	0	0
ENSG00000273106	0	0	0	0	0	0	1	3	0	0
ENSG00000273104	0	0	0	0	0	0	1	0	0	0
ENSG00000273102	0	0	0	0	0	1	0	0	0	0
ENSG00000273100	0	0	0	0	0	0	228	212	0	0
ENSG00000273098	0	0	0	0	0	0	0	0	0	0
ENSG00000273096	1	0	0	2	1	0	7	5	0	6
ENSG00000273093	0	0	0	0	0	0	0	0	0	0
ENSG00000273091	0	0	0	0	0	0	0	7	0	0
ENSG00000273090	0	0	0	0	0	0	1	0	0	0
ENSG00000273088	0	0	0	0	0	0	0	0	0	0
ENSG00000273085	0	0	0	0	0	0	0	0	0	0
ENSG00000273084	5	4	5	4	3	12	2	9	0	0
ENSG00000273082	0	0	0	0	0	0	0	0	0	0
ENSG00000273081	2	4	16	5	7	24	1	5	0	1
ENSG00000273080	0	0	0	1	0	8	0	2	0	0
ENSG00000273079	0	4	1	0	1	0	5	17	0	0
ENSG00000273077	0	0	0	0	0	0	2	0	0	0
ENSG00000273076	0	2	1	1	0	3	10	0	0	0
ENSG00000273073	1	6	3	2	1	3	258	98	0	18
ENSG00000273071	0	0	0	0	0	0	0	0	0	0
ENSG00000273069	0	0	0	2	0	0	6	2	0	0
ENSG00000273068	0	0	0	0	0	0	0	0	0	0
ENSG00000273066	0	4	4	1	2	14	16	19	0	0
ENSG00000273065	0	0	0	0	0	0	0	0	0	0
ENSG00000273064	0	2	0	0	0	3	8	5	0	0
ENSG00000273063	0	0	0	0	0	0	1	6	0	0
ENSG00000273062	2	0	6	5	1	3	0	0	0	0
ENSG00000273061	13	3	25	13	0	27	6	18	0	0
ENSG00000273059	0	0	0	0	0	0	0	0	0	0
ENSG00000273058	3	6	8	3	3	11	53	64	0	0
ENSG00000273056	2	1	5	2	6	17	0	0	0	0
ENSG00000273055	8	8	24	11	12	52	6	31	0	1
ENSG00000273051	0	0	0	0	0	0	0	0	0	0

ENSG00000273049	0	0	0	0	0	0	0	0	0	0
ENSG00000273048	0	0	0	0	0	0	0	0	0	0
ENSG00000273047	0	0	0	0	0	0	0	0	0	0
ENSG00000273046	0	0	0	0	0	0	0	0	0	0
ENSG00000273045	1	6	17	6	6	11	6	3	0	0
ENSG00000273044	0	0	0	0	0	0	396	14	0	3
ENSG00000273041	0	2	1	0	0	0	4	1	0	0
ENSG00000273038	171	150	190	168	162	407	604	117	0	12
ENSG00000273036	0	0	0	0	0	0	0	0	0	0
ENSG00000273035	0	0	0	0	0	0	3	0	0	8
ENSG00000273033	167	171	276	142	141	336	245	107	1	0
ENSG00000273032	9	9	4	2	2	13	1	1	0	0
ENSG00000273030	0	3	0	0	1	0	2	1	0	0
ENSG00000273027	2	1	1	1	1	4	3	0	0	0
ENSG00000273026	3	5	9	1	2	7	35	6	0	0
ENSG00000273025	0	0	0	0	0	0	0	0	0	0
ENSG00000273024	10	4	2	2	7	14	28	20	0	0
ENSG00000273019	0	0	0	0	0	0	0	0	0	0
ENSG00000273018	2	6	11	1	1	0	35	23	0	0
ENSG00000273017	0	0	1	0	0	0	13	3	0	0
ENSG00000273015	68	76	134	56	57	158	726	378	0	19
ENSG00000273014	7	4	5	6	1	25	8	24	0	0
ENSG00000273013	0	0	0	0	0	2	0	0	0	0
ENSG00000273012	0	0	0	0	0	0	0	0	0	0
ENSG00000273011	0	0	0	1	0	0	3	0	0	0
ENSG00000273010	0	3	2	1	0	5	6	0	0	0
ENSG00000273009	0	0	1	0	0	0	0	0	0	0
ENSG00000273008	4	0	0	0	0	2	8	12	0	0
ENSG00000273007	1	2	2	0	2	4	9	1	0	0
ENSG00000273006	0	0	0	0	0	0	0	0	0	0
ENSG00000273004	7	12	17	7	6	29	17	0	0	0
ENSG00000273003	0	0	0	0	0	0	3	0	0	0
ENSG00000273002	3	9	10	4	3	15	81	6	0	0
ENSG00000273001	5	2	2	1	2	1	14	2	0	0
ENSG00000273000	0	0	0	0	0	0	1	0	0	0
ENSG00000272995	0	0	0	0	0	0	5	2	0	0
ENSG00000272994	26	15	61	24	19	46	53	17	2	0
ENSG00000272993	0	0	0	0	0	0	0	0	0	0
ENSG00000272992	0	0	0	0	0	0	28	7	0	0
ENSG00000272991	0	0	0	0	0	0	0	0	0	0
ENSG00000272990	1	5	0	0	1	0	33	14	0	0
ENSG00000272989	2	3	5	0	0	10	4	5	0	0
ENSG00000272988	0	0	0	0	0	0	1	0	0	0
ENSG00000272987	0	0	0	0	0	0	0	0	0	0
ENSG00000272986	0	0	0	0	0	0	6	0	0	2
ENSG00000272984	0	0	0	0	0	0	0	0	0	0
ENSG00000272983	7	10	6	3	0	13	12	21	0	0
ENSG00000272982	0	0	0	0	0	0	0	0	0	0
ENSG00000272981	0	0	0	0	0	0	0	0	0	0
ENSG00000272980	0	0	0	0	0	0	16	1	0	0
ENSG00000272979	0	0	0	1	0	4	3	19	0	0
ENSG00000272977	44	37	43	43	39	121	127	26	0	0
ENSG00000272975	0	0	0	0	2	0	0	0	0	0
ENSG00000272973	0	0	0	0	0	0	0	0	0	0
ENSG00000272971	0	0	0	0	0	0	1	9	0	0
ENSG00000272970	0	0	1	0	0	0	9	5	0	0
ENSG00000272969	0	1	2	0	0	2	1	3	0	0

ENSG00000272968	0	0	0	0	0	0	0	0	0	0
ENSG00000272967	0	0	0	0	0	0	0	0	0	0
ENSG00000272966	0	0	0	0	0	1	3	8	0	0
ENSG00000272963	0	0	0	0	0	0	0	0	0	0
ENSG00000272960	11	6	11	12	16	41	5	8	0	0
ENSG00000272958	0	0	0	0	0	0	2	0	0	0
ENSG00000272954	0	0	0	1	0	0	1	0	0	0
ENSG00000272953	2	1	1	0	2	5	7	0	0	0
ENSG00000272950	0	0	0	1	0	1	16	5	0	0
ENSG00000272949	4	5	6	3	0	11	1	8	0	0
ENSG00000272948	0	0	3	0	0	0	0	0	0	0
ENSG00000272947	2	3	13	15	5	17	13	15	0	0
ENSG00000272945	4	2	5	2	5	2	3	2	0	0
ENSG00000272944	5	2	8	10	1	4	2	0	0	0
ENSG00000272942	0	0	0	0	0	0	0	0	0	0
ENSG00000272941	1	5	2	4	6	13	26	8	0	0
ENSG00000272940	0	2	0	0	0	0	0	3	0	0
ENSG00000272937	0	0	0	0	0	0	0	0	0	0
ENSG00000272936	0	0	0	0	0	0	0	0	0	0
ENSG00000272934	0	0	0	0	0	0	0	0	0	0
ENSG00000272933	13	27	58	12	13	62	35	28	0	0
ENSG00000272931	0	1	1	0	0	0	0	0	0	0
ENSG00000272927	0	0	0	0	0	0	9	4	0	0
ENSG00000272923	0	0	0	0	0	0	0	0	0	0
ENSG00000272922	0	0	0	0	0	0	1	22	0	0
ENSG00000272921	0	0	0	0	0	0	0	0	0	0
ENSG00000272920	0	0	0	0	0	0	0	0	0	0
ENSG00000272918	8	9	12	6	5	12	13	8	0	0
ENSG00000272917	0	0	0	0	0	0	0	0	0	0
ENSG00000272916	0	0	0	0	0	0	0	0	0	0
ENSG00000272915	0	0	0	0	0	1	0	0	0	0
ENSG00000272914	1	0	3	0	0	2	20	20	0	0
ENSG00000272913	18	11	12	8	7	23	10	6	0	0
ENSG00000272912	0	0	0	0	0	0	0	5	0	0
ENSG00000272911	0	1	0	1	0	0	0	5	0	0
ENSG00000272910	1	1	0	0	1	4	3	3	0	0
ENSG00000272909	3	6	11	1	3	6	22	9	0	0
ENSG00000272908	0	0	0	0	0	0	25	6	0	0
ENSG00000272906	3	0	1	0	1	3	13	0	0	0
ENSG00000272905	0	0	0	0	0	0	1	6	0	0
ENSG00000272904	0	0	2	0	0	2	0	0	0	0
ENSG00000272902	6	11	15	5	3	15	3	6	0	0
ENSG00000272900	0	0	0	0	0	0	0	0	0	0
ENSG00000272899	16	12	20	12	15	30	40	19	0	0
ENSG00000272897	0	0	0	0	0	0	0	0	0	0
ENSG00000272896	0	0	0	0	0	0	0	0	0	0
ENSG00000272895	0	0	0	0	0	0	3	0	0	0
ENSG00000272894	16	23	29	8	11	47	68	45	0	2
ENSG00000272892	0	1	7	0	1	5	0	0	0	0
ENSG00000272888	65	71	220	56	74	162	3647	2089	4	88
ENSG00000272887	0	0	0	0	0	0	0	0	0	0
ENSG00000272886	287	278	385	249	286	771	2495	536	0	9
ENSG00000272885	0	0	4	0	0	0	4	0	0	0
ENSG00000272884	0	0	0	0	0	2	0	0	0	0
ENSG00000272882	0	0	0	0	0	0	0	0	0	0
ENSG00000272880	0	0	0	0	0	0	0	0	0	0
ENSG00000272874	0	0	0	0	0	0	0	0	0	0

ENSG00000272872	2	1	11	4	3	6	22	4	0	0
ENSG00000272871	2	2	5	0	4	2	63	31	0	0
ENSG00000272870	12	7	39	15	13	33	63	79	0	0
ENSG00000272869	0	0	0	0	1	0	0	5	0	0
ENSG00000272866	0	0	0	0	0	0	0	3	0	0
ENSG00000272865	1	1	1	0	2	0	0	0	0	0
ENSG00000272864	0	0	0	0	0	0	3	1	0	0
ENSG00000272862	0	0	0	0	0	0	0	0	0	0
ENSG00000272861	0	0	0	0	0	0	2	3	0	0
ENSG00000272859	0	0	0	0	0	0	1	6	0	0
ENSG00000272858	0	0	0	0	1	1	8	4	0	0
ENSG00000272856	0	0	0	0	0	0	0	3	0	0
ENSG00000272855	0	0	0	0	0	0	0	3	0	0
ENSG00000272854	1	0	0	0	0	0	2	0	0	0
ENSG00000272853	1	4	6	2	3	10	8	8	0	0
ENSG00000272851	0	0	0	0	0	0	0	0	0	0
ENSG00000272849	9	25	13	9	8	35	15	7	0	0
ENSG00000272848	0	1	0	3	1	5	5	6	0	0
ENSG00000272844	0	0	0	1	1	1	4	0	0	0
ENSG00000272843	3	1	0	0	2	2	7	0	0	0
ENSG00000272842	0	0	1	0	0	0	12	3	0	0
ENSG00000272841	13	9	21	4	6	13	20	23	0	0
ENSG00000272840	0	0	0	0	0	0	1	0	0	0
ENSG00000272839	0	0	0	0	0	0	3	7	0	0
ENSG00000272837	0	0	0	0	0	0	0	0	0	0
ENSG00000272836	6	8	13	7	1	9	3	5	0	0
ENSG00000272834	0	3	2	2	2	4	1	3	0	0
ENSG00000272832	0	0	0	0	0	0	3	12	0	0
ENSG00000272831	0	0	2	0	0	6	0	0	0	0
ENSG00000272829	1	1	9	1	4	3	1	1	0	0
ENSG00000272827	0	0	0	0	0	0	0	0	0	0
ENSG00000272825	2	1	1	2	1	6	7	1	0	0
ENSG00000272824	0	4	2	1	1	1	1	15	0	0
ENSG00000272823	0	0	0	0	0	0	3	13	0	0
ENSG00000272822	0	0	0	1	1	0	0	3	0	0
ENSG00000272821	9	22	18	27	16	56	65	28	0	0
ENSG00000272817	0	0	0	0	0	0	3	0	0	0
ENSG00000272815	0	0	0	0	0	0	1	0	0	0
ENSG00000272814	0	0	0	0	0	0	0	0	0	0
ENSG00000272812	4	3	10	3	3	22	32	4	0	0
ENSG00000272810	0	0	1	0	0	0	7	2	0	0
ENSG00000272808	2	3	10	5	3	9	8	16	0	0
ENSG00000272807	1	0	0	0	0	0	6	5	0	0
ENSG00000272804	0	0	0	0	0	0	0	0	0	0
ENSG00000272801	0	0	0	0	0	0	0	0	0	0
ENSG00000272800	0	0	2	0	0	0	18	0	0	0
ENSG00000272799	0	0	0	0	0	0	3	2	0	0
ENSG00000272798	0	0	0	0	0	0	0	0	0	0
ENSG00000272797	0	0	0	0	0	0	1	1	0	0
ENSG00000272796	32	22	44	16	22	86	14	0	0	0
ENSG00000272795	0	0	1	0	0	0	0	0	0	0
ENSG00000272792	1	2	0	3	0	0	6	12	0	0
ENSG00000272791	2	2	4	0	1	6	6	23	0	0
ENSG00000272789	0	0	0	0	0	0	28	350	0	0
ENSG00000272788	0	0	2	1	2	3	4	0	0	0
ENSG00000272787	0	0	1	2	1	5	9	0	0	0
ENSG00000272784	0	2	0	1	0	1	7	7	0	0

ENSG00000272783	0	0	0	0	0	0	28	26	0	0
ENSG00000272780	0	0	0	0	0	0	0	0	0	0
ENSG00000272779	1	3	9	0	3	3	56	18	0	2
ENSG00000272777	1	0	5	0	1	0	16	25	0	0
ENSG00000272774	0	0	0	0	0	0	0	0	0	0
ENSG00000272772	0	0	0	0	0	0	0	0	0	0
ENSG00000272770	0	0	0	0	0	0	0	0	0	0
ENSG00000272769	0	2	4	1	2	1	10	8	0	0
ENSG00000272768	0	1	5	1	1	2	10	0	0	0
ENSG00000272767	1	3	5	2	2	0	6	15	0	11
ENSG00000272764	0	0	0	0	0	0	7	2	0	0
ENSG00000272763	0	2	3	1	2	0	8	7	0	0
ENSG00000272762	0	0	0	0	0	0	0	0	0	0
ENSG00000272761	97	139	229	107	84	245	90	491	0	22
ENSG00000272760	8	4	25	10	8	22	42	52	0	6
ENSG00000272758	8	8	12	2	8	10	16	30	0	0
ENSG00000272755	1	3	3	5	7	4	15	31	0	0
ENSG00000272754	0	1	2	0	0	0	2	14	0	0
ENSG00000272752	3	0	0	1	0	10	2	3	0	0
ENSG00000272750	4	2	6	1	2	13	68	59	0	4
ENSG00000272748	1	0	0	0	0	0	1	0	0	0
ENSG00000272746	0	0	0	0	0	0	1	3	0	0
ENSG00000272745	0	0	0	0	0	0	4	0	0	0
ENSG00000272744	1	2	0	1	2	6	20	2	0	0
ENSG00000272742	0	0	0	0	0	0	0	0	0	0
ENSG00000272741	0	0	0	0	0	0	0	0	0	0
ENSG00000272736	0	0	0	0	0	0	0	0	0	0
ENSG00000272735	0	0	0	0	0	4	4	0	0	0
ENSG00000272734	50	43	74	38	36	95	486	99	0	0
ENSG00000272733	0	0	0	0	0	0	10	9	0	0
ENSG00000272732	0	1	2	1	1	4	62	23	0	0
ENSG00000272729	0	0	0	0	0	0	0	0	0	0
ENSG00000272727	0	0	0	1	0	1	0	0	0	0
ENSG00000272724	0	0	0	0	0	0	10	7	0	0
ENSG00000272721	1	0	2	0	0	0	2	0	0	0
ENSG00000272720	26	21	30	13	17	49	3	7	0	0
ENSG00000272719	0	0	0	1	0	0	3	0	0	0
ENSG00000272717	0	0	0	1	1	0	4	2	0	0
ENSG00000272716	1	7	3	1	2	6	0	6	0	0
ENSG00000272715	4	4	0	3	3	1	0	0	0	0
ENSG00000272714	0	0	0	0	0	0	0	0	0	0
ENSG00000272711	20	16	9	9	12	28	13	11	0	0
ENSG00000272710	0	0	0	0	0	0	1	0	0	0
ENSG00000272707	3	1	1	2	3	3	9	2	0	0
ENSG00000272703	1	2	2	0	0	1	0	0	0	0
ENSG00000272702	0	0	5	0	0	0	13	0	0	0
ENSG00000272701	0	0	0	0	0	0	0	0	0	0
ENSG00000272699	1	0	0	0	0	0	6	0	0	0
ENSG00000272696	2	5	8	1	6	17	6	13	0	0
ENSG00000272695	32	21	33	14	18	102	27	63	0	5
ENSG00000272694	0	0	0	0	0	0	0	0	0	0
ENSG00000272693	0	0	0	0	0	0	0	0	0	0
ENSG00000272692	6	5	6	2	2	13	12	14	0	0
ENSG00000272691	0	0	0	0	0	1	4	0	0	0
ENSG00000272690	0	3	1	1	2	1	22	9	0	0
ENSG00000272689	8	9	4	7	7	16	5	0	0	0
ENSG00000272688	0	0	0	0	0	0	0	0	0	0

ENSG00000272686	15	9	24	10	13	46	80	92	0	0
ENSG00000272685	0	0	0	0	0	0	0	0	0	0
ENSG00000272682	1	0	1	0	1	1	6	0	0	0
ENSG00000272681	2	2	4	0	1	0	33	25	0	0
ENSG00000272679	0	0	0	0	0	0	0	0	0	0
ENSG00000272678	0	0	0	0	0	0	0	0	0	0
ENSG00000272677	0	1	0	1	0	0	8	15	0	0
ENSG00000272676	0	0	0	0	0	0	0	0	0	0
ENSG00000272674	1	0	0	0	2	7	37	75	1	0
ENSG00000272672	0	0	0	0	0	0	0	0	0	0
ENSG00000272669	5	5	2	6	2	20	201	111	0	16
ENSG00000272668	5	0	0	1	1	5	11	19	0	0
ENSG00000272667	16	36	30	9	12	41	53	62	0	5
ENSG00000272666	2	1	2	3	4	3	35	2	3	29
ENSG00000272664	0	0	0	0	0	0	0	0	0	0
ENSG00000272663	2	0	3	0	1	4	4	1	0	0
ENSG00000272662	0	0	0	0	0	0	3	3	0	0
ENSG00000272661	0	0	0	0	0	0	0	0	0	0
ENSG00000272660	0	0	0	0	0	2	0	0	0	0
ENSG00000272659	0	0	0	0	0	0	2	4	0	0
ENSG00000272657	0	0	0	0	0	0	0	0	0	0
ENSG00000272656	0	0	0	0	0	0	4	0	0	0
ENSG00000272655	30	38	65	30	22	98	42	54	0	0
ENSG00000272654	5	22	26	12	16	55	181	46	0	0
ENSG00000272650	1	0	0	0	0	0	9	17	0	0
ENSG00000272647	0	0	0	0	0	0	0	0	0	0
ENSG00000272646	0	0	0	0	1	0	5	5	0	0
ENSG00000272645	69	102	119	88	63	192	149	156	0	5
ENSG00000272644	4	4	3	2	1	0	31	26	0	0
ENSG00000272642	0	0	0	0	0	0	0	0	0	0
ENSG00000272639	0	0	0	0	0	0	0	0	0	0
ENSG00000272638	0	0	0	0	0	0	0	0	0	0
ENSG00000272636	1	1	2	1	2	1	199	830	1	8
ENSG00000272635	0	0	0	0	0	0	0	0	0	0
ENSG00000272634	0	0	0	0	0	0	0	0	0	0
ENSG00000272632	0	0	0	0	0	0	3	2	0	0
ENSG00000272631	8	7	15	1	3	16	18	32	0	1
ENSG00000272630	5	1	5	2	4	7	84	8	0	9
ENSG00000272627	0	0	0	0	0	0	15	0	0	0
ENSG00000272626	0	0	0	0	0	0	0	4	0	0
ENSG00000272625	0	0	0	0	0	0	3	7	0	0
ENSG00000272622	4	4	1	3	1	8	1	7	0	0
ENSG00000272620	11	6	4	5	5	15	7	5	0	0
ENSG00000272619	0	0	0	0	0	0	1	1	0	0
ENSG00000272617	0	0	0	0	0	0	0	0	0	0
ENSG00000272610	1	0	0	0	0	0	29	36	0	0
ENSG00000272609	0	0	0	0	0	0	0	0	0	0
ENSG00000272606	0	0	0	0	0	0	0	0	0	0
ENSG00000272604	3	4	1	6	5	14	16	4	0	0
ENSG00000272602	94	137	212	80	98	317	396	192	7	12
ENSG00000272601	0	0	0	0	0	0	0	0	0	0
ENSG00000272600	0	0	0	0	0	0	0	0	0	0
ENSG00000272599	0	1	7	1	2	8	12	3	0	0
ENSG00000272597	0	0	0	0	0	0	1	5	0	0
ENSG00000272595	0	0	0	0	0	0	0	0	0	0
ENSG00000272593	0	0	0	0	0	0	0	0	0	0
ENSG00000272592	0	0	0	0	0	0	0	0	0	0

ENSG00000272589	0	0	0	0	0	0	1	0	0	0
ENSG00000272588	0	3	0	0	1	0	4	4	0	0
ENSG00000272583	1	2	0	0	5	3	0	0	0	0
ENSG00000272582	0	0	0	0	0	0	0	0	0	0
ENSG00000272579	6	3	12	6	6	5	288	142	0	2
ENSG00000272578	14	17	40	17	13	29	15	32	0	0
ENSG00000272576	0	0	0	0	0	0	4	8	0	0
ENSG00000272575	0	0	0	0	0	0	0	0	0	0
ENSG00000272574	10	3	4	7	2	17	1	3	0	0
ENSG00000272573	0	0	1	2	1	1	0	0	0	0
ENSG00000272572	0	1	0	0	0	2	4	10	0	0
ENSG00000272569	0	0	0	0	0	0	0	0	0	0
ENSG00000272568	0	0	0	0	0	0	7	7	0	0
ENSG00000272567	0	0	0	0	0	0	20	2	0	0
ENSG00000272566	0	0	0	0	0	0	0	0	0	0
ENSG00000272565	80	89	131	72	65	237	142	64	0	9
ENSG00000272564	0	0	0	0	1	0	3	7	0	0
ENSG00000272563	3	11	19	4	3	13	1	13	0	5
ENSG00000272562	0	0	0	0	0	0	0	1	0	0
ENSG00000272559	0	0	0	0	0	0	0	0	0	0
ENSG00000272558	0	0	0	0	0	0	0	0	0	0
ENSG00000272556	5	5	3	1	0	5	12	13	0	3
ENSG00000272555	0	0	0	0	0	0	25	12	0	0
ENSG00000272554	0	0	0	0	0	0	0	0	0	0
ENSG00000272551	0	0	0	0	1	0	2	1	0	0
ENSG00000272549	0	0	0	0	0	5	0	1	0	0
ENSG00000272546	0	0	0	0	0	0	0	0	0	0
ENSG00000272545	0	0	0	0	0	0	0	0	0	0
ENSG00000272543	0	0	0	0	0	0	0	0	0	0
ENSG00000272542	0	1	6	0	0	0	0	1	0	0
ENSG00000272541	0	0	0	0	1	4	15	17	0	0
ENSG00000272540	25	30	42	23	32	94	19	5	0	0
ENSG00000272537	0	0	0	0	0	0	1	0	0	0
ENSG00000272536	0	0	0	0	0	0	0	14	0	0
ENSG00000272533	2	0	0	0	2	5	6	0	0	6
ENSG00000272529	0	1	0	0	0	1	12	6	0	0
ENSG00000272525	2	2	13	1	3	0	2	4	0	0
ENSG00000272524	1	0	0	0	0	0	1	0	0	0
ENSG00000272523	0	1	0	2	0	0	33	104	0	0
ENSG00000272520	110	148	236	88	91	305	46	29	0	0
ENSG00000272519	0	0	0	0	2	1	0	0	0	0
ENSG00000272518	18	26	29	14	19	35	12	9	0	0
ENSG00000272516	0	0	0	0	0	0	0	0	0	0
ENSG00000272515	0	0	0	0	0	0	0	0	0	0
ENSG00000272514	0	0	0	0	0	0	0	5	0	0
ENSG00000272512	0	5	7	1	2	2	18	10	0	0
ENSG00000272511	0	0	1	0	0	0	19	218	0	0
ENSG00000272510	1	0	0	0	0	0	0	0	0	0
ENSG00000272509	0	0	0	0	0	0	0	13	0	0
ENSG00000272508	0	0	0	0	0	0	3	0	0	0
ENSG00000272507	0	0	0	0	0	0	0	2	0	0
ENSG00000272506	0	0	0	0	0	0	4	15	0	0
ENSG00000272505	7	4	6	1	2	12	49	39	0	0
ENSG00000272502	0	0	0	0	0	0	1	1	0	0
ENSG00000272501	3	2	4	3	1	9	78	72	0	0
ENSG00000272498	1	6	0	0	1	0	12	3	0	0
ENSG00000272494	0	0	0	0	0	0	0	0	0	0

ENSG00000272491	0	2	0	0	0	3	8	12	0	0
ENSG00000272489	3	0	0	1	0	1	9	3	0	0
ENSG00000272486	0	2	2	0	4	2	21	7	0	0
ENSG00000272485	0	0	1	0	0	0	1	0	0	0
ENSG00000272483	0	0	0	0	0	0	9	11	0	0
ENSG00000272482	12	16	7	11	7	38	9	7	0	0
ENSG00000272480	0	0	0	0	0	0	0	0	0	0
ENSG00000272479	0	0	0	0	0	0	0	4	0	0
ENSG00000272478	0	0	0	0	0	0	0	0	0	0
ENSG00000272477	0	0	0	0	0	0	0	2	0	0
ENSG00000272476	11	7	17	6	5	26	11	13	0	0
ENSG00000272474	0	0	0	0	0	0	0	0	0	0
ENSG00000272473	0	1	1	1	0	5	6	3	0	0
ENSG00000272472	0	0	0	0	0	0	0	1	0	0
ENSG00000272469	0	0	0	0	0	0	0	0	0	0
ENSG00000272468	1	3	4	4	4	9	4	0	0	0
ENSG00000272465	1	0	1	1	0	0	5	5	0	0
ENSG00000272463	0	0	0	0	0	0	18	69	2	1
ENSG00000272462	7	12	30	10	8	32	41	50	0	0
ENSG00000272461	0	0	0	0	0	0	0	30	0	0
ENSG00000272460	0	0	0	0	0	0	0	0	0	0
ENSG00000272459	4	5	3	0	3	4	5	5	0	0
ENSG00000272458	0	0	0	0	0	0	0	0	0	0
ENSG00000272457	0	2	3	1	2	2	0	0	0	0
ENSG00000272456	0	0	0	0	0	0	3	2	0	0
ENSG00000272455	79	59	123	45	47	180	127	54	0	6
ENSG00000272452	31	34	61	31	20	114	87	58	0	6
ENSG00000272449	5	2	6	1	2	9	54	17	0	0
ENSG00000272447	31	31	44	23	20	49	106	64	0	5
ENSG00000272446	0	0	0	0	0	0	0	0	0	0
ENSG00000272445	0	0	0	0	0	0	1	0	0	0
ENSG00000272444	0	1	1	1	3	7	2	10	0	0
ENSG00000272443	0	0	0	0	0	0	0	0	0	0
ENSG00000272442	1	0	0	0	0	4	2	2	0	0
ENSG00000272440	3	1	0	3	1	0	4	11	0	0
ENSG00000272439	0	0	0	0	0	0	0	0	0	0
ENSG00000272438	1	0	0	0	1	0	2	0	0	0
ENSG00000272436	0	0	0	0	0	0	0	0	0	0
ENSG00000272435	0	0	0	0	0	0	0	0	0	0
ENSG00000272434	1	0	0	0	0	0	6	24	0	0
ENSG00000272432	1	0	2	1	1	0	3	3	0	0
ENSG00000272431	0	0	0	0	0	0	0	0	0	0
ENSG00000272430	0	0	0	0	0	0	0	3	0	4
ENSG00000272428	0	0	0	0	0	0	1	0	0	0
ENSG00000272426	1	0	0	0	0	0	11	0	0	0
ENSG00000272425	0	0	0	0	0	1	1	7	0	0
ENSG00000272420	2	6	2	3	2	5	6	1	0	0
ENSG00000272419	209	203	342	224	180	634	400	204	1	24
ENSG00000272418	0	0	0	0	0	0	0	0	0	0
ENSG00000272417	0	0	0	0	0	0	0	0	0	0
ENSG00000272416	0	0	0	0	0	0	7	4	0	0
ENSG00000272414	0	0	0	0	0	0	0	0	0	0
ENSG00000272412	0	2	0	0	0	0	0	4	0	0
ENSG00000272411	0	1	0	0	1	0	7	2	0	0
ENSG00000272410	0	0	0	0	0	0	0	0	0	0
ENSG00000272407	0	0	0	0	0	0	0	0	0	0
ENSG00000272406	0	0	0	0	0	0	6	0	0	0

ENSG00000272405	24	26	46	25	18	60	2	19	0	0
ENSG00000272403	16	13	19	14	24	68	46	20	0	0
ENSG00000272402	2	0	0	4	3	13	4	3	0	0
ENSG00000272400	0	0	0	0	0	0	0	0	0	0
ENSG00000272398	18251	20326	36812	15537	16054	47050	1697	268	4	60
ENSG00000272397	0	0	0	0	0	0	0	0	0	0
ENSG00000272396	0	0	0	0	0	0	0	0	0	0
ENSG00000272395	0	1	5	1	0	0	0	0	0	0
ENSG00000272393	0	0	0	0	0	0	0	1	0	0
ENSG00000272391	1477	1563	1843	1281	1165	3509	2585	1111	0	4
ENSG00000272389	0	2	1	4	1	3	20	3	0	0
ENSG00000272387	0	0	0	0	0	0	0	0	0	0
ENSG00000272386	0	0	0	0	0	0	1	1	0	0
ENSG00000272385	0	0	0	0	0	0	0	0	0	0
ENSG00000272384	0	0	0	1	1	0	36	3	0	0
ENSG00000272383	1	0	0	0	0	0	2	0	0	0
ENSG00000272382	0	0	1	0	0	0	10	9	0	0
ENSG00000272381	0	0	0	0	0	0	0	7	1	1
ENSG00000272380	0	0	0	0	0	0	0	0	0	0
ENSG00000272379	0	0	0	0	0	0	0	0	0	0
ENSG00000272375	4	2	0	0	1	3	22	5	0	0
ENSG00000272374	3	4	7	0	0	6	56	9	0	0
ENSG00000272372	0	0	0	0	0	0	0	0	0	0
ENSG00000272371	0	0	0	0	0	0	0	0	0	0
ENSG00000272370	0	0	0	0	0	4	5	9	0	0
ENSG00000272369	3	0	5	0	2	9	2	11	0	0
ENSG00000272368	0	6	9	3	4	2	21	28	0	1
ENSG00000272367	0	1	3	2	0	5	14	9	0	0
ENSG00000272366	0	0	0	0	0	0	1	0	0	0
ENSG00000272365	9	7	9	3	3	20	11	14	0	0
ENSG00000272362	5	5	8	6	3	15	0	0	0	0
ENSG00000272361	0	0	0	0	1	1	5	5	0	0
ENSG00000272360	0	0	0	0	0	0	0	6	0	0
ENSG00000272359	0	1	0	0	0	0	2	11	0	0
ENSG00000272356	4	6	4	8	3	8	12	12	0	2
ENSG00000272354	1	2	1	1	1	0	3	0	0	0
ENSG00000272351	0	0	0	0	0	0	3	0	0	5
ENSG00000272347	0	1	2	0	0	0	4	27	0	0
ENSG00000272346	0	0	0	0	0	0	0	0	0	0
ENSG00000272345	0	3	2	0	0	0	12	17	0	0
ENSG00000272344	0	0	0	0	0	0	0	0	0	0
ENSG00000272343	0	0	1	0	0	0	19	4	0	0
ENSG00000272342	0	0	0	0	0	0	0	3	0	0
ENSG00000272341	2	1	11	0	7	7	185	48	0	0
ENSG00000272338	9	19	24	5	11	26	23	19	0	0
ENSG00000272337	0	0	0	0	0	0	1	0	0	0
ENSG00000272335	30	28	67	21	25	64	188	75	0	5
ENSG00000272334	0	0	0	1	0	0	13	0	0	0
ENSG00000272333	513	515	733	562	469	1441	748	431	0	6
ENSG00000272330	0	0	0	0	0	0	0	3	0	0
ENSG00000272329	0	0	0	0	0	0	0	0	0	0
ENSG00000272328	0	0	0	0	0	0	0	0	0	0
ENSG00000272327	14	18	38	18	16	72	213	4	0	2
ENSG00000272325	208	199	270	136	165	490	476	370	0	23
ENSG00000272324	0	0	0	0	0	0	0	0	0	0
ENSG00000272323	3	2	1	1	0	5	14	4	0	0
ENSG00000272321	0	0	0	0	0	0	2	4	0	0

ENSG00000272320	7	12	20	11	5	21	9	8	0	0
ENSG00000272319	1	0	0	0	0	0	1	0	0	0
ENSG00000272316	32	64	79	29	33	93	89	86	0	0
ENSG00000272312	0	0	0	0	0	0	0	16	0	0
ENSG00000272311	0	0	0	0	0	0	0	0	0	0
ENSG00000272308	7	3	11	4	9	13	2	0	0	0
ENSG00000272305	1	1	5	1	2	8	0	3	0	0
ENSG00000272304	0	0	0	0	0	0	0	0	0	0
ENSG00000272301	0	0	3	0	0	3	1	0	0	0
ENSG00000272299	0	0	0	0	0	0	0	0	0	0
ENSG00000272298	0	0	0	0	0	0	0	0	0	0
ENSG00000272297	0	0	0	0	0	0	0	0	0	0
ENSG00000272296	0	0	0	0	0	0	0	0	0	0
ENSG00000272294	0	0	0	0	0	0	1	2	0	0
ENSG00000272293	3	12	23	7	5	14	56	30	0	0
ENSG00000272292	0	0	0	0	0	0	0	0	0	0
ENSG00000272291	0	0	0	0	0	0	0	0	0	0
ENSG00000272288	14	10	32	13	4	26	52	66	2	0
ENSG00000272282	0	1	0	0	0	2	60	11	0	0
ENSG00000272281	0	1	0	0	0	0	1	3	0	0
ENSG00000272279	2	1	0	2	2	0	1	12	0	0
ENSG00000272277	3	0	5	1	3	4	13	3	0	0
ENSG00000272275	2	2	5	1	2	6	10	11	0	0
ENSG00000272274	0	1	3	1	0	0	32	0	0	0
ENSG00000272273	0	1	0	0	0	0	13	3	0	0
ENSG00000272269	3	5	19	5	1	9	94	64	0	0
ENSG00000272267	1	0	0	0	1	2	6	0	0	0
ENSG00000272265	0	1	0	0	0	2	0	0	0	0
ENSG00000272264	0	1	2	0	0	0	4	2	0	0
ENSG00000272263	0	1	3	0	0	1	10	7	0	0
ENSG00000272262	0	0	0	0	0	0	0	0	0	0
ENSG00000272259	0	0	0	0	0	0	0	0	0	0
ENSG00000272256	0	1	0	0	0	0	11	14	0	0
ENSG00000272255	0	0	0	0	0	0	1	0	0	0
ENSG00000272254	0	0	0	0	0	0	0	0	0	0
ENSG00000272253	0	0	0	0	0	0	0	0	0	0
ENSG00000272250	0	2	1	0	0	0	3	0	0	0
ENSG00000272249	0	0	0	0	0	0	0	0	0	0
ENSG00000272248	0	0	0	0	0	0	0	0	0	0
ENSG00000272247	0	0	0	0	0	0	0	0	0	0
ENSG00000272243	0	0	0	0	2	0	1	0	0	0
ENSG00000272240	7	13	13	2	6	18	52	76	0	0
ENSG00000272239	0	0	0	0	0	0	1	0	0	0
ENSG00000272237	0	0	0	0	0	0	0	0	0	0
ENSG00000272236	0	0	0	0	0	0	5	3	0	0
ENSG00000272235	0	0	0	0	0	0	45	0	0	0
ENSG00000272234	0	0	0	0	0	0	0	0	0	0
ENSG00000272232	0	0	0	0	0	0	0	0	0	0
ENSG00000272231	0	0	0	0	0	0	0	0	0	0
ENSG00000272230	0	0	0	0	0	0	0	0	0	0
ENSG00000272226	1	1	3	0	1	2	5	0	0	0
ENSG00000272223	0	1	4	3	2	8	1	0	0	0
ENSG00000272221	10	8	18	18	17	38	41	83	0	9
ENSG00000272220	0	0	0	0	0	0	0	0	0	0
ENSG00000272219	0	0	0	0	0	0	6	6	0	0
ENSG00000272218	0	0	0	0	0	0	0	5	0	0
ENSG00000272217	4	4	11	2	1	0	15	3	0	0

ENSG00000272215	0	0	0	0	0	0	0	0	0	0
ENSG00000272213	0	0	0	0	0	0	0	0	0	0
ENSG00000272211	1	1	2	1	2	0	5	6	0	0
ENSG00000272209	0	0	0	0	0	0	1	0	0	0
ENSG00000272205	3	0	1	5	0	2	8	2	0	0
ENSG00000272203	0	0	0	0	0	0	1	1	0	0
ENSG00000272202	0	1	0	1	1	0	3	0	0	0
ENSG00000272201	1	0	0	1	0	3	3	10	0	0
ENSG00000272197	0	0	0	0	0	0	0	0	0	0
ENSG00000272196	199	261	300	386	179	597	1987	654	52	598
ENSG00000272195	0	0	0	1	0	0	0	0	0	0
ENSG00000272192	0	3	0	1	0	4	8	4	0	0
ENSG00000272189	4	9	11	6	10	19	15	3	0	0
ENSG00000272186	2	0	3	3	1	2	12	5	0	0
ENSG00000272183	3	3	9	3	1	10	1	1	0	0
ENSG00000272182	0	0	0	0	0	0	0	0	0	0
ENSG00000272181	0	0	0	0	0	0	16	3	0	0
ENSG00000272180	0	0	0	0	0	1	0	3	0	0
ENSG00000272177	0	0	0	0	0	0	0	0	0	0
ENSG00000272175	0	0	0	0	0	0	1	1	0	0
ENSG00000272174	0	0	0	0	0	0	0	0	0	0
ENSG00000272173	0	1	3	3	1	5	10	19	0	0
ENSG00000272172	2	2	3	0	0	4	2	0	0	0
ENSG00000272170	0	3	4	0	0	1	0	3	0	0
ENSG00000272168	16	11	50	22	21	59	141	115	0	0
ENSG00000272167	0	0	0	0	0	0	0	6	0	0
ENSG00000272166	0	0	0	0	0	0	0	0	0	0
ENSG00000272164	0	0	0	0	0	0	0	0	0	0
ENSG00000272163	0	0	0	0	0	0	1	0	0	0
ENSG00000272162	0	0	0	0	0	0	0	0	0	0
ENSG00000272161	0	0	0	0	0	0	1	0	0	0
ENSG00000272160	0	0	0	0	0	0	1	0	0	0
ENSG00000272159	0	0	0	0	0	1	11	2	0	0
ENSG00000272158	0	0	0	0	0	0	14	5	0	0
ENSG00000272157	0	0	0	0	0	0	0	0	0	0
ENSG00000272156	1	7	9	5	3	16	6	2	0	0
ENSG00000272155	0	0	0	0	0	1	19	5	0	0
ENSG00000272154	0	0	0	0	0	0	0	0	0	0
ENSG00000272153	3	3	4	2	5	2	1	1	0	0
ENSG00000272150	1146	1184	1867	934	1140	2901	1444	457	0	10
ENSG00000272149	0	0	0	0	0	0	0	0	0	0
ENSG00000272148	0	0	2	3	1	5	7	1	0	0
ENSG00000272146	0	1	0	1	1	0	0	0	0	0
ENSG00000272145	5	4	7	4	1	5	15	8	0	0
ENSG00000272144	2	0	0	0	0	0	8	20	0	0
ENSG00000272143	2	7	5	2	1	1	19	144	0	0
ENSG00000272142	17	24	29	19	15	97	5	17	0	2
ENSG00000272141	21	29	22	32	22	65	44	0	0	0
ENSG00000272140	13	13	9	4	5	27	32	14	0	0
ENSG00000272139	0	0	0	0	0	0	0	0	0	0
ENSG00000272138	3	2	0	2	4	0	1	7	0	0
ENSG00000272137	0	0	0	0	0	0	2	4	0	0
ENSG00000272135	0	0	0	0	0	0	0	0	0	0
ENSG00000272134	0	0	0	0	0	0	0	0	0	0
ENSG00000272130	2	0	0	0	0	0	0	2	0	0
ENSG00000272129	3	2	2	4	3	0	1	12	0	0
ENSG00000272128	0	0	1	0	1	0	5	0	0	0

ENSG00000272123	0	5	1	3	0	5	2	10	0	0
ENSG00000272121	9	3	15	24	8	38	0	0	0	0
ENSG00000272115	0	0	0	0	0	0	0	4	0	0
ENSG00000272114	0	0	0	0	0	0	0	0	0	0
ENSG00000272113	0	0	0	0	0	0	0	0	0	0
ENSG00000272112	1	3	2	2	1	0	7	12	0	0
ENSG00000272109	0	1	0	0	0	6	31	9	0	0
ENSG00000272108	0	0	0	0	0	0	1	0	0	0
ENSG00000272106	17	21	38	20	19	44	69	94	2	13
ENSG00000272104	0	0	0	0	0	0	0	0	0	0
ENSG00000272103	0	0	0	0	0	0	3	0	0	0
ENSG00000272102	6	4	9	11	4	11	4	3	0	0
ENSG00000272100	1	1	2	0	3	6	11	2	0	0
ENSG00000272097	4	6	10	6	3	18	5	0	0	0
ENSG00000272096	0	0	0	0	0	0	0	0	0	0
ENSG00000272094	1	0	0	1	0	0	10	5	0	0
ENSG00000272093	67	40	69	38	42	101	31	15	0	0
ENSG00000272092	7	6	19	2	1	10	7	17	0	9
ENSG00000272091	22	11	15	4	12	40	56	102	0	0
ENSG00000272088	1	6	17	4	0	20	1	0	0	0
ENSG00000272087	1	2	10	5	3	8	15	7	0	0
ENSG00000272086	5	5	13	4	6	18	20	35	0	0
ENSG00000272085	0	0	0	0	0	0	0	0	0	0
ENSG00000272084	1	0	12	2	1	6	85	35	0	0
ENSG00000272082	0	0	0	0	0	0	0	0	0	0
ENSG00000272081	4	1	8	7	2	3	3	6	0	0
ENSG00000272080	0	0	0	0	0	0	0	0	0	0
ENSG00000272079	2	2	4	1	1	2	42	6	0	0
ENSG00000272078	8	5	17	4	3	11	202	25	0	1
ENSG00000272077	26	20	54	24	32	104	66	105	0	0
ENSG00000272076	0	0	1	0	0	0	0	0	0	0
ENSG00000272075	0	0	0	0	0	0	0	0	0	0
ENSG00000272072	3	10	2	4	8	11	33	4	0	0
ENSG00000272071	3	0	0	2	0	0	5	0	0	0
ENSG00000272070	1	2	0	0	2	6	9	13	0	0
ENSG00000272068	0	2	9	4	0	3	1	13	0	1
ENSG00000272065	0	0	0	1	0	0	29	16	0	11
ENSG00000272058	86	86	155	92	86	297	224	118	0	18
ENSG00000272057	0	0	0	0	0	0	1	0	0	0
ENSG00000272056	2	1	0	1	0	8	11	12	0	0
ENSG00000272055	0	1	0	0	0	1	16	0	0	4
ENSG00000272054	3	1	8	3	1	16	28	15	0	0
ENSG00000272053	0	0	0	0	0	0	29	6	0	0
ENSG00000272051	0	0	0	0	0	0	0	0	0	0
ENSG00000272050	0	0	0	0	0	0	0	0	0	0
ENSG00000272049	2	0	0	0	0	0	2	3	0	0
ENSG00000272047	415	436	757	363	339	1106	364	421	0	7
ENSG00000272046	0	0	0	0	0	0	0	2	0	0
ENSG00000272045	0	0	0	0	0	0	0	0	0	0
ENSG00000272043	0	0	6	0	1	1	4	0	0	0
ENSG00000272040	9	10	11	5	8	23	30	22	0	0
ENSG00000272037	0	0	0	1	0	2	40	7	0	0
ENSG00000272036	0	0	0	0	0	0	0	0	0	0
ENSG00000272034	3	3	3	3	7	16	86	132	0	0
ENSG00000272033	0	1	3	0	1	0	1	0	0	0
ENSG00000272031	26	34	35	21	12	77	23	29	0	0
ENSG00000272030	0	0	0	0	0	0	2	6	0	0

ENSG00000272028	0	0	0	0	0	0	0	0	0	0
ENSG00000272027	0	0	0	0	0	0	0	3	0	0
ENSG00000272025	0	0	0	0	0	0	0	0	0	0
ENSG00000272024	0	0	0	1	0	0	3	1	0	0
ENSG00000272023	0	0	0	0	0	0	0	3	0	0
ENSG00000272021	0	0	2	0	0	0	2	0	0	0
ENSG00000272020	0	0	0	0	0	0	0	0	0	0
ENSG00000272019	0	0	0	0	0	0	0	0	0	0
ENSG00000272017	1	1	4	1	0	0	1	0	0	0
ENSG00000272016	11	15	25	12	10	30	906	89	2	1
ENSG00000272015	0	0	0	0	0	0	0	0	0	0
ENSG00000272010	0	0	0	0	0	0	1	14	0	0
ENSG00000272009	27	34	35	23	20	61	41	25	0	6
ENSG00000272008	1	2	0	2	0	7	9	6	0	0
ENSG00000272006	0	0	0	0	0	0	0	0	0	0
ENSG00000272004	0	0	0	1	0	0	0	2	0	0
ENSG00000272002	0	0	0	0	0	0	5	8	0	0
ENSG00000271998	0	0	0	0	0	0	0	0	0	0
ENSG00000271997	4	5	4	5	3	10	12	41	0	0
ENSG00000271996	0	0	0	0	0	1	0	0	0	0
ENSG00000271995	0	0	0	0	0	0	0	0	0	0
ENSG00000271993	1	0	0	1	1	0	5	0	0	0
ENSG00000271992	0	0	0	0	1	0	4	0	0	0
ENSG00000271991	0	0	0	0	0	3	3	0	0	0
ENSG00000271989	0	0	0	0	0	0	0	0	0	0
ENSG00000271986	0	0	0	0	0	0	0	5	0	0
ENSG00000271985	0	0	0	0	0	0	0	0	0	0
ENSG00000271984	0	3	4	1	1	4	0	0	0	0
ENSG00000271983	1	0	0	0	0	0	3	8	0	0
ENSG00000271982	0	0	0	0	0	0	0	0	0	0
ENSG00000271981	0	0	0	0	0	0	0	0	0	0
ENSG00000271980	0	0	0	1	0	1	3	0	0	0
ENSG00000271978	2	4	0	0	1	1	5	6	0	0
ENSG00000271977	0	0	0	0	0	0	0	0	0	0
ENSG00000271976	37	51	38	32	22	72	137	28	0	2
ENSG00000271975	13	18	48	13	13	40	103	70	0	0
ENSG00000271974	0	0	0	0	0	0	1	2	0	0
ENSG00000271973	0	0	2	0	1	0	4	6	0	0
ENSG00000271971	0	3	4	1	1	11	26	15	0	0
ENSG00000271970	0	0	0	0	0	0	5	0	0	0
ENSG00000271969	5	0	0	1	2	1	1	0	0	0
ENSG00000271968	0	0	0	0	0	0	0	0	0	0
ENSG00000271967	2	2	2	0	1	0	5	3	0	0
ENSG00000271966	1	3	2	1	3	4	5	1	0	0
ENSG00000271964	1	1	1	1	0	5	15	13	0	0
ENSG00000271963	0	0	0	1	0	0	0	0	0	0
ENSG00000271959	0	0	0	0	0	1	7	87	0	0
ENSG00000271958	0	0	0	0	0	0	0	1	0	0
ENSG00000271955	0	0	0	0	0	0	0	0	0	0
ENSG00000271952	1	0	0	0	0	0	0	0	0	0
ENSG00000271949	0	0	0	0	0	0	0	0	0	0
ENSG00000271947	0	0	0	0	0	0	3	0	0	0
ENSG00000271945	0	0	0	0	0	0	0	0	0	0
ENSG00000271943	0	0	0	0	0	0	2	6	0	0
ENSG00000271941	1	1	3	0	0	7	22	2	0	0
ENSG00000271938	0	0	0	0	0	0	1	21	0	0
ENSG00000271937	0	0	0	0	0	0	0	0	0	0

ENSG00000271936	8	3	12	9	7	7	7	2	0	0
ENSG00000271934	0	0	0	0	0	0	0	0	0	0
ENSG00000271933	10	10	15	8	5	11	26	7	0	0
ENSG00000271932	0	0	0	0	0	0	0	0	0	0
ENSG00000271931	0	1	0	0	0	0	1	0	0	0
ENSG00000271930	0	0	1	0	0	0	0	0	0	0
ENSG00000271927	10	11	16	10	13	25	31	16	0	0
ENSG00000271926	0	1	0	1	3	0	1	0	0	0
ENSG00000271924	1	2	8	1	0	2	1	7	0	0
ENSG00000271923	0	0	0	0	0	0	0	9	0	0
ENSG00000271922	0	0	0	0	0	0	0	0	0	0
ENSG00000271919	0	0	0	0	0	0	1	0	0	0
ENSG00000271918	2	15	14	10	3	32	16	27	0	0
ENSG00000271917	0	0	0	0	0	0	0	0	0	0
ENSG00000271916	0	0	0	0	0	0	2	0	0	0
ENSG00000271914	0	1	0	2	3	3	6	1	0	0
ENSG00000271913	12	4	14	0	2	21	10	11	0	0
ENSG00000271911	0	0	0	0	0	0	2	0	0	0
ENSG00000271907	0	0	0	0	0	0	8	3	0	0
ENSG00000271905	0	0	0	0	0	0	1	0	0	0
ENSG00000271904	0	0	0	0	0	0	0	0	0	0
ENSG00000271901	0	0	0	0	0	0	0	0	0	0
ENSG00000271900	0	0	0	0	0	0	0	0	0	0
ENSG00000271899	0	0	0	0	0	0	0	0	0	0
ENSG00000271898	0	0	0	0	0	0	0	0	0	0
ENSG00000271897	0	0	0	0	0	0	2	0	0	0
ENSG00000271895	4	4	15	2	1	17	25	18	0	0
ENSG00000271894	0	0	0	0	0	0	0	0	0	0
ENSG00000271893	0	0	0	0	0	0	0	0	0	0
ENSG00000271892	0	0	0	0	0	0	2	0	0	0
ENSG00000271890	0	0	0	0	0	0	0	7	0	0
ENSG00000271889	2	0	2	0	2	5	0	0	0	0
ENSG00000271888	1	0	3	3	1	2	9	11	0	0
ENSG00000271886	0	2	5	0	1	0	3	0	0	0
ENSG00000271882	1	0	0	0	2	0	15	14	0	0
ENSG00000271880	0	1	1	0	1	0	2	0	0	0
ENSG00000271876	0	0	0	1	0	0	0	2	0	0
ENSG00000271875	0	0	0	0	0	0	0	0	0	0
ENSG00000271874	0	1	0	1	1	0	2	8	0	0
ENSG00000271871	0	0	0	0	0	4	17	5	0	0
ENSG00000271870	4	3	1	2	2	22	169	64	0	0
ENSG00000271869	3	6	3	2	2	4	50	48	0	0
ENSG00000271868	1	0	1	3	0	14	1	0	0	0
ENSG00000271862	9	12	24	12	24	45	41	15	0	0
ENSG00000271860	4	0	5	1	0	6	0	0	0	0
ENSG00000271858	0	0	0	0	0	0	0	0	0	0
ENSG00000271857	0	1	5	3	2	8	10	0	0	0
ENSG00000271856	0	0	0	0	0	0	145	5	0	13
ENSG00000271855	1	0	5	0	1	3	18	0	0	0
ENSG00000271853	0	0	0	0	0	0	0	0	0	0
ENSG00000271852	0	0	0	0	0	0	0	0	0	0
ENSG00000271851	2	4	2	1	1	8	8	10	0	0
ENSG00000271850	0	0	0	0	0	0	0	0	0	0
ENSG00000271849	2	0	0	0	1	0	3	1	0	0
ENSG00000271848	0	0	0	0	0	0	0	0	0	0
ENSG00000271846	0	0	0	0	0	0	0	6	0	0
ENSG00000271843	0	4	4	4	5	7	6	4	0	0

ENSG00000271841	0	0	0	0	0	0	0	6	0	0
ENSG00000271840	0	0	0	0	0	0	29	6	0	0
ENSG00000271833	5	3	0	1	6	6	10	24	0	0
ENSG00000271831	0	0	0	0	0	0	0	0	0	0
ENSG00000271830	0	0	0	1	1	0	0	0	0	0
ENSG00000271828	4	8	6	3	5	11	12	0	0	0
ENSG00000271826	6	3	5	2	8	15	4	3	0	0
ENSG00000271825	0	0	0	0	2	3	0	9	0	0
ENSG00000271824	0	0	1	0	0	1	0	0	0	0
ENSG00000271821	0	0	0	0	0	0	2	0	0	0
ENSG00000271820	0	0	0	0	0	0	0	0	0	0
ENSG00000271819	0	0	0	0	0	0	6	0	0	0
ENSG00000271818	0	0	0	0	0	0	0	0	0	0
ENSG00000271817	0	1	2	0	1	0	1	0	0	0
ENSG00000271816	3	3	3	1	4	18	10	1	0	0
ENSG00000271815	3	3	7	3	7	17	4	4	0	0
ENSG00000271814	0	0	0	0	0	0	0	0	0	0
ENSG00000271811	0	0	0	0	0	0	210	30	0	0
ENSG00000271810	0	0	0	0	0	0	0	0	0	0
ENSG00000271806	2	2	8	0	3	6	10	5	0	2
ENSG00000271803	0	0	1	0	1	0	0	0	0	0
ENSG00000271798	0	1	0	0	0	0	0	0	0	0
ENSG00000271797	0	3	1	2	1	4	3	7	0	0
ENSG00000271795	43	64	59	35	39	54	14	4	0	0
ENSG00000271793	0	0	0	0	0	0	0	0	0	0
ENSG00000271792	0	0	0	0	0	0	0	0	0	0
ENSG00000271789	1	3	2	1	4	3	3	0	0	0
ENSG00000271788	0	0	4	0	0	11	9	9	0	0
ENSG00000271787	0	0	0	0	0	0	0	2	0	0
ENSG00000271786	0	0	0	0	0	1	0	0	0	0
ENSG00000271784	4	0	0	1	1	0	1	0	0	0
ENSG00000271782	0	0	0	0	0	0	2	2	0	0
ENSG00000271781	1	6	5	4	4	19	5	0	0	0
ENSG00000271780	8	5	15	5	6	14	23	22	0	0
ENSG00000271779	0	0	6	0	0	2	2	0	0	0
ENSG00000271778	0	0	0	0	0	0	15	5	0	0
ENSG00000271776	0	0	0	0	0	0	0	4	0	0
ENSG00000271774	0	0	0	0	0	0	0	0	0	0
ENSG00000271771	0	0	0	0	0	0	0	2	0	0
ENSG00000271766	0	0	0	0	0	0	0	0	0	0
ENSG00000271765	0	0	0	0	0	0	1	2	0	0
ENSG00000271763	2	0	0	0	0	0	3	0	0	0
ENSG00000271762	0	0	0	0	0	0	0	0	0	0
ENSG00000271761	0	0	0	0	0	0	0	0	0	0
ENSG00000271758	0	0	0	0	0	0	0	0	0	0
ENSG00000271757	0	0	0	0	1	0	0	3	0	0
ENSG00000271755	10	3	7	2	1	5	14	38	0	0
ENSG00000271754	1	11	9	6	6	14	5	15	0	0
ENSG00000271752	0	0	0	0	1	0	0	1	0	0
ENSG00000271751	3	0	0	0	1	0	2	0	0	0
ENSG00000271749	0	1	2	0	2	0	3	0	0	0
ENSG00000271748	0	0	0	0	0	0	0	0	0	0
ENSG00000271746	0	0	0	0	0	0	0	0	0	0
ENSG00000271745	0	0	0	0	0	0	3	145	0	0
ENSG00000271743	0	0	0	2	0	0	0	11	0	0
ENSG00000271742	0	3	0	0	1	0	0	0	0	0
ENSG00000271741	0	0	0	0	0	0	0	0	0	0

ENSG00000271739	0	0	0	0	0	0	0	2	0	0
ENSG00000271737	4	0	0	1	1	1	7	9	0	0
ENSG00000271736	0	0	0	0	0	0	2	0	0	0
ENSG00000271734	0	1	0	0	0	0	16	5	0	0
ENSG00000271732	0	0	0	0	0	0	3	2	0	0
ENSG00000271730	0	0	0	0	0	0	1	3	0	0
ENSG00000271727	0	0	0	0	0	2	0	0	0	0
ENSG00000271725	1	1	0	1	1	8	3	3	0	3
ENSG00000271724	0	0	0	0	0	0	0	0	0	0
ENSG00000271723	0	0	0	0	0	0	0	0	0	0
ENSG00000271722	0	0	3	3	2	1	7	3	0	0
ENSG00000271721	2	10	14	3	8	13	4	1	0	0
ENSG00000271717	0	0	0	0	0	0	0	1	0	0
ENSG00000271716	0	0	0	0	0	0	4	0	0	0
ENSG00000271715	0	0	0	0	0	0	0	0	0	0
ENSG00000271714	1	1	4	1	0	0	0	0	0	0
ENSG00000271712	0	0	0	0	0	0	0	0	0	0
ENSG00000271711	0	1	3	0	0	0	3	1	0	0
ENSG00000271710	0	0	0	0	0	0	0	0	0	0
ENSG00000271709	0	0	0	0	0	0	7	4	0	0
ENSG00000271707	0	0	0	1	0	0	0	0	0	0
ENSG00000271705	0	0	0	0	0	0	0	4	0	0
ENSG00000271704	0	0	0	0	0	0	0	0	0	0
ENSG00000271702	0	0	0	0	0	0	0	0	0	0
ENSG00000271701	0	0	0	0	0	0	0	0	0	0
ENSG00000271699	0	0	0	0	0	0	0	0	0	0
ENSG00000271698	2	2	2	0	3	1	0	0	0	0
ENSG00000271697	0	0	0	1	0	0	0	0	0	0
ENSG00000271696	0	0	0	0	0	0	0	0	0	0
ENSG00000271693	0	0	0	0	0	0	0	0	0	0
ENSG00000271691	0	0	0	0	0	0	0	0	0	0
ENSG00000271687	0	0	0	0	0	0	0	0	0	0
ENSG00000271686	0	0	0	0	0	0	0	0	0	0
ENSG00000271682	0	0	0	0	0	0	0	0	0	0
ENSG00000271681	0	0	0	0	0	0	0	0	0	0
ENSG00000271680	0	0	0	0	0	0	8	0	0	0
ENSG00000271679	0	0	0	0	0	0	0	0	0	0
ENSG00000271677	0	0	0	0	0	0	0	0	0	0
ENSG00000271676	12	5	13	1	9	8	7	0	0	0
ENSG00000271672	0	0	0	0	0	0	0	0	0	0
ENSG00000271671	0	0	0	0	0	0	0	0	0	0
ENSG00000271670	3	1	10	1	5	4	0	0	0	0
ENSG00000271667	0	0	0	0	0	0	0	0	0	0
ENSG00000271666	0	0	0	0	0	0	0	0	0	0
ENSG00000271664	5	4	2	2	1	10	0	1	0	0
ENSG00000271662	5	4	8	5	4	17	4	0	0	0
ENSG00000271661	0	0	0	0	0	0	0	0	0	0
ENSG00000271660	0	0	0	0	0	0	0	0	0	0
ENSG00000271659	0	3	2	1	1	0	1	0	0	0
ENSG00000271657	0	0	0	0	0	0	0	0	0	0
ENSG00000271656	0	0	0	0	0	0	0	0	0	0
ENSG00000271655	0	0	0	0	0	0	0	0	0	0
ENSG00000271654	0	0	0	0	0	0	0	0	0	0
ENSG00000271653	0	0	0	0	0	0	3	0	0	0
ENSG00000271652	0	0	0	0	0	0	0	0	0	0
ENSG00000271650	0	0	0	0	0	0	0	0	0	0
ENSG00000271649	0	0	0	0	0	0	0	0	0	0

ENSG00000271647	0	0	0	0	0	0	0	0	0	0
ENSG00000271646	6	10	6	6	5	25	82	75	0	19
ENSG00000271644	2	1	6	0	0	2	2	0	0	0
ENSG00000271643	94	96	143	64	108	213	17	39	0	0
ENSG00000271642	0	0	0	0	0	0	0	0	0	0
ENSG00000271639	0	0	0	0	0	0	0	0	0	0
ENSG00000271638	0	0	0	0	0	0	0	3	0	0
ENSG00000271635	0	0	0	0	0	0	0	0	0	0
ENSG00000271632	0	0	0	0	0	0	0	0	0	0
ENSG00000271631	0	0	1	0	1	5	2	0	0	0
ENSG00000271629	0	0	0	0	0	0	0	0	0	0
ENSG00000271627	0	0	0	0	0	0	0	0	0	0
ENSG00000271626	0	0	0	0	0	0	3	0	0	0
ENSG00000271625	0	0	0	0	0	0	0	0	0	0
ENSG00000271624	0	0	0	0	0	0	1	10	0	0
ENSG00000271623	0	0	0	0	0	0	0	0	0	0
ENSG00000271621	0	0	0	0	0	0	1	0	0	0
ENSG00000271620	0	0	0	0	0	0	0	0	0	0
ENSG00000271618	0	0	0	0	0	0	0	0	0	0
ENSG00000271616	0	0	0	0	0	0	0	0	0	0
ENSG00000271615	0	0	0	0	0	0	0	0	0	0
ENSG00000271614	0	1	4	0	3	1	71	90	0	18
ENSG00000271612	0	0	0	0	0	0	0	0	0	0
ENSG00000271611	0	0	0	0	0	0	0	0	0	0
ENSG00000271609	0	1	0	0	0	0	0	0	0	0
ENSG00000271608	0	0	0	0	0	0	0	0	0	0
ENSG00000271607	0	0	1	0	2	0	0	0	0	0
ENSG00000271606	0	0	0	0	0	0	0	0	0	0
ENSG00000271605	35	30	35	29	32	98	64	38	0	16
ENSG00000271602	0	0	0	0	0	0	0	0	0	0
ENSG00000271601	172	190	311	190	136	498	1033	984	6	39
ENSG00000271600	0	0	0	0	0	0	0	0	0	0
ENSG00000271598	0	0	4	0	0	0	0	2	0	0
ENSG00000271597	0	0	0	0	0	0	0	0	0	0
ENSG00000271596	0	0	0	0	0	0	3	0	0	0
ENSG00000271595	0	0	0	0	0	0	0	0	0	0
ENSG00000271593	0	0	0	0	0	0	0	0	0	0
ENSG00000271590	2	3	0	1	0	2	22	2	0	0
ENSG00000271589	0	0	0	0	0	0	0	0	0	0
ENSG00000271588	0	0	0	0	0	0	0	0	0	0
ENSG00000271587	0	0	0	0	0	0	0	0	0	0
ENSG00000271586	0	0	0	0	0	0	0	0	0	0
ENSG00000271585	0	0	0	0	0	0	0	0	0	0
ENSG00000271584	1	3	2	1	0	2	1	2	0	0
ENSG00000271583	0	0	0	0	0	0	0	0	0	0
ENSG00000271582	0	0	0	0	0	0	0	0	0	0
ENSG00000271581	0	0	0	0	0	0	6	0	0	0
ENSG00000271580	2	0	3	0	1	19	2	4	0	0
ENSG00000271579	0	0	0	0	0	0	54	10	0	0
ENSG00000271578	0	0	0	0	0	0	0	0	0	0
ENSG00000271576	5	3	1	1	0	4	9	12	0	0
ENSG00000271573	0	2	0	1	0	1	0	0	0	0
ENSG00000271569	0	0	0	0	0	0	0	0	0	0
ENSG00000271568	0	0	0	0	0	0	0	0	0	0
ENSG00000271567	0	0	0	0	0	0	6	0	0	0
ENSG00000271565	0	0	0	0	0	0	0	0	0	0
ENSG00000271564	0	0	0	0	0	0	0	1	0	0

ENSG00000271563	0	0	0	0	0	0	0	0	0	0
ENSG00000271560	0	0	0	0	0	0	0	0	0	0
ENSG00000271559	0	0	0	0	0	0	0	0	0	0
ENSG00000271558	0	0	0	0	0	0	0	0	0	0
ENSG00000271557	0	0	0	0	0	2	1	0	0	0
ENSG00000271555	0	0	0	0	0	0	4	0	0	0
ENSG00000271554	1	0	2	0	0	0	8	3	0	0
ENSG00000271553	4	1	4	3	0	1	29	17	0	4
ENSG00000271551	0	0	1	0	3	1	3	0	0	0
ENSG00000271550	4	5	6	1	10	8	7	20	0	7
ENSG00000271547	0	0	0	1	0	0	0	0	0	0
ENSG00000271546	0	0	0	0	0	0	0	0	0	0
ENSG00000271544	0	0	0	0	0	0	0	0	0	0
ENSG00000271543	0	0	0	0	0	0	0	0	0	0
ENSG00000271538	0	0	0	0	1	0	0	0	0	0
ENSG00000271537	0	2	0	0	0	5	0	0	0	0
ENSG00000271536	0	0	0	0	0	0	0	0	0	0
ENSG00000271533	13	12	17	9	10	21	132	19	0	0
ENSG00000271532	0	0	0	0	0	0	0	0	0	0
ENSG00000271530	0	0	0	0	0	0	0	0	0	0
ENSG00000271527	0	0	0	0	0	0	2	0	0	0
ENSG00000271526	0	0	0	0	0	0	0	0	0	0
ENSG00000271525	0	0	0	0	0	0	1	0	0	0
ENSG00000271524	0	0	0	0	1	0	0	0	0	0
ENSG00000271523	0	0	0	0	0	0	0	0	0	0
ENSG00000271522	0	0	0	0	0	1	25	1	0	0
ENSG00000271519	0	0	2	0	0	2	1	5	0	0
ENSG00000271511	0	0	0	0	0	0	0	0	0	0
ENSG00000271509	0	0	0	2	1	6	0	0	0	0
ENSG00000271508	0	0	0	0	0	0	0	0	0	0
ENSG00000271507	0	0	0	0	0	0	0	1	0	0
ENSG00000271503	31	21	26	11	25	30	1942	1125	8	61
ENSG00000271502	0	0	0	0	0	0	0	0	0	0
ENSG00000271500	0	0	0	0	0	0	3	10	0	0
ENSG00000271499	0	0	0	0	0	1	0	0	0	0
ENSG00000271498	0	0	0	0	0	0	0	0	0	0
ENSG00000271496	0	0	0	0	0	0	0	5	0	0
ENSG00000271495	0	0	0	0	0	0	0	0	0	0
ENSG00000271494	0	0	0	0	0	0	0	0	0	0
ENSG00000271492	0	0	0	0	0	0	0	0	0	0
ENSG00000271491	0	0	0	0	0	0	0	0	0	0
ENSG00000271490	0	0	0	0	0	0	0	0	0	0
ENSG00000271488	0	0	0	0	0	0	0	0	0	0
ENSG00000271486	0	0	0	0	0	0	0	0	0	0
ENSG00000271484	0	0	0	0	0	0	3	0	0	0
ENSG00000271483	0	0	0	0	0	0	10	0	0	0
ENSG00000271482	0	0	0	0	0	0	6	0	0	0
ENSG00000271480	0	0	0	0	0	0	0	0	0	0
ENSG00000271477	0	0	0	0	0	0	0	0	0	0
ENSG00000271475	0	0	0	0	0	0	0	0	0	0
ENSG00000271474	0	0	0	0	0	0	4	19	0	0
ENSG00000271468	0	0	0	0	0	0	0	0	0	0
ENSG00000271466	1	1	9	3	2	2	0	0	0	0
ENSG00000271465	0	0	0	0	0	0	0	0	0	0
ENSG00000271464	0	0	0	0	0	0	0	0	0	0
ENSG00000271462	0	0	0	0	0	0	0	0	0	0
ENSG00000271461	0	0	0	0	0	0	0	6	0	0

ENSG00000271459	0	0	0	0	0	0	0	0	0	0
ENSG00000271457	0	0	0	0	0	0	0	0	0	0
ENSG00000271456	0	0	0	0	0	0	0	13	0	0
ENSG00000271455	0	0	0	0	0	0	0	0	0	0
ENSG00000271454	0	0	0	0	0	0	0	6	0	7
ENSG00000271452	2	2	12	2	1	17	7	6	0	0
ENSG00000271449	0	0	0	0	0	0	0	0	0	0
ENSG00000271447	409	465	576	396	414	1002	143	563	0	0
ENSG00000271443	0	0	0	0	0	0	0	0	0	0
ENSG00000271440	0	0	0	0	0	0	0	0	0	0
ENSG00000271439	0	0	0	0	0	0	0	0	0	0
ENSG00000271437	0	0	0	0	0	0	6	0	0	0
ENSG00000271435	0	0	0	0	0	0	0	0	0	0
ENSG00000271434	1	0	1	0	0	2	0	0	0	0
ENSG00000271433	0	0	0	0	0	0	0	0	0	0
ENSG00000271430	66	74	101	48	40	164	102	80	0	11
ENSG00000271429	0	0	0	0	0	0	0	0	0	0
ENSG00000271428	4	6	0	0	1	5	2	0	0	0
ENSG00000271427	0	1	1	0	0	0	2	0	0	0
ENSG00000271426	2	0	0	0	1	0	1	0	0	0
ENSG00000271425	17466	18238	28231	14328	17245	46980	5685	1121	1	55
ENSG00000271424	1	0	0	0	0	0	5	0	0	0
ENSG00000271423	0	0	0	0	0	0	0	0	0	0
ENSG00000271421	0	0	0	0	0	0	0	0	0	0
ENSG00000271420	0	0	0	0	0	0	7	9	1	0
ENSG00000271419	0	0	0	0	0	0	0	0	0	0
ENSG00000271418	0	0	0	0	0	0	0	1	0	0
ENSG00000271417	0	0	4	1	1	2	5	0	0	0
ENSG00000271415	0	0	1	0	0	0	0	0	0	0
ENSG00000271414	0	1	0	0	0	0	0	0	0	0
ENSG00000271413	0	0	0	0	0	0	0	0	0	0
ENSG00000271412	0	0	0	0	0	0	0	0	0	0
ENSG00000271410	0	0	0	0	0	0	11	0	0	0
ENSG00000271409	0	0	0	0	0	0	0	0	0	0
ENSG00000271408	0	0	0	0	0	0	0	0	0	0
ENSG00000271404	0	0	0	0	0	0	4	0	0	0
ENSG00000271402	0	0	0	0	0	0	0	0	0	0
ENSG00000271401	0	0	0	0	1	1	4	0	0	0
ENSG00000271400	2	1	2	0	1	4	1	0	0	0
ENSG00000271399	0	0	0	0	0	0	3	1	0	0
ENSG00000271398	0	2	0	0	1	1	0	0	0	0
ENSG00000271397	0	0	0	0	0	0	0	0	0	0
ENSG00000271396	2	0	0	1	0	1	1	0	0	0
ENSG00000271395	0	0	0	0	0	0	0	0	0	0
ENSG00000271394	0	1	0	0	0	0	38	0	1	19
ENSG00000271392	0	1	1	1	1	1	0	3	0	0
ENSG00000271390	0	0	0	0	0	0	0	7	0	0
ENSG00000271389	0	0	0	0	0	0	0	1	0	0
ENSG00000271387	0	2	0	0	0	0	2	28	0	0
ENSG00000271386	0	0	0	0	0	0	0	0	0	0
ENSG00000271385	0	0	0	0	0	0	0	0	0	0
ENSG00000271384	0	0	0	0	0	4	0	0	0	0
ENSG00000271383	12284	13386	19247	10158	12077	34097	6736	1856	6	36
ENSG00000271382	0	0	0	1	0	0	7	3	0	0
ENSG00000271381	0	0	0	0	0	0	0	0	0	0
ENSG00000271380	0	1	2	2	2	2	0	1	0	0
ENSG00000271379	0	0	0	0	0	0	0	0	0	0

ENSG00000271378	0	0	0	0	0	0	0	0	0	0
ENSG00000271377	0	0	0	0	0	0	3	0	0	0
ENSG00000271375	0	0	0	0	0	0	1	5	0	0
ENSG00000271373	0	0	0	0	0	0	0	8	0	0
ENSG00000271370	0	0	0	0	0	0	2	0	0	0
ENSG00000271369	3	4	0	3	4	10	1	0	0	0
ENSG00000271368	0	0	0	0	0	0	0	0	0	0
ENSG00000271367	0	0	2	0	0	0	3	9	0	0
ENSG00000271366	1	5	6	2	8	7	2	1	0	0
ENSG00000271365	0	0	0	0	0	0	0	0	0	0
ENSG00000271364	0	0	0	0	0	0	0	0	0	0
ENSG00000271362	0	0	0	0	0	0	0	0	0	0
ENSG00000271361	0	0	0	0	0	0	0	0	0	0
ENSG00000271360	0	0	0	0	0	0	0	5	0	0
ENSG00000271359	86	105	118	59	60	233	17	3	0	0
ENSG00000271358	0	0	0	0	0	0	0	0	0	0
ENSG00000271357	0	0	0	0	0	0	0	0	0	0
ENSG00000271356	0	0	0	0	0	0	0	0	0	0
ENSG00000271355	0	0	0	0	0	0	0	0	0	0
ENSG00000271353	0	0	0	0	0	0	0	2	0	0
ENSG00000271351	0	0	0	0	0	0	0	0	0	0
ENSG00000271350	0	0	0	0	0	0	0	0	0	0
ENSG00000271349	0	0	0	0	0	0	0	0	0	0
ENSG00000271347	0	2	0	1	0	5	5	3	0	0
ENSG00000271346	0	0	0	0	0	0	0	0	0	0
ENSG00000271344	13	15	16	19	14	44	19	13	0	0
ENSG00000271343	0	0	0	0	0	0	0	8	0	0
ENSG00000271340	0	0	0	0	0	0	0	0	0	0
ENSG00000271339	0	0	0	0	0	0	0	0	0	0
ENSG00000271338	0	0	0	0	0	0	0	0	0	0
ENSG00000271337	0	0	0	0	0	0	0	0	0	0
ENSG00000271336	0	0	0	0	0	0	0	0	0	0
ENSG00000271335	29	26	46	23	24	66	27	35	0	0
ENSG00000271334	0	0	0	0	0	0	0	4	0	7
ENSG00000271333	0	0	0	0	0	0	0	0	0	0
ENSG00000271332	0	0	0	0	0	0	1	0	0	0
ENSG00000271330	0	0	0	0	0	0	0	0	0	0
ENSG00000271329	0	1	0	0	0	0	2	0	0	0
ENSG00000271328	0	0	0	0	0	0	0	0	0	0
ENSG00000271327	0	0	1	1	1	7	7	10	0	0
ENSG00000271324	0	0	0	0	1	1	3	15	0	0
ENSG00000271322	0	0	0	0	0	0	0	0	0	0
ENSG00000271321	64	65	86	54	54	150	116	26	0	0
ENSG00000271320	0	1	0	0	0	1	0	0	0	0
ENSG00000271318	0	0	0	0	0	0	1	0	0	0
ENSG00000271317	0	0	0	0	0	0	0	0	0	0
ENSG00000271315	0	0	0	0	0	0	0	0	0	0
ENSG00000271314	0	0	0	0	0	0	0	0	0	0
ENSG00000271313	0	0	0	0	0	0	0	0	0	0
ENSG00000271309	0	0	0	0	0	0	0	0	0	0
ENSG00000271307	0	0	0	0	0	0	0	0	0	0
ENSG00000271306	0	0	0	0	0	0	0	0	0	0
ENSG00000271305	0	0	0	0	0	0	0	0	0	0
ENSG00000271304	0	0	0	0	0	0	0	0	0	0
ENSG00000271303	0	0	2	1	1	0	13	43	0	0
ENSG00000271302	0	0	0	0	0	0	0	0	0	0
ENSG00000271299	0	0	0	0	0	0	0	0	0	0

ENSG00000271298	0	0	0	0	0	0	0	0	0	0
ENSG00000271296	0	0	0	0	0	0	0	0	0	0
ENSG00000271291	0	0	0	0	0	0	0	0	0	0
ENSG00000271290	0	0	0	0	0	0	0	0	0	0
ENSG00000271288	0	0	0	0	0	0	0	0	0	0
ENSG00000271287	0	0	0	0	0	0	0	0	0	0
ENSG00000271286	0	0	0	0	0	0	0	0	0	0
ENSG00000271284	0	0	0	0	0	0	0	0	0	0
ENSG00000271283	0	0	1	0	0	0	1	0	0	0
ENSG00000271278	0	0	2	0	0	2	2	0	0	0
ENSG00000271277	0	0	0	0	0	0	0	0	0	0
ENSG00000271275	0	0	0	0	0	0	0	0	0	0
ENSG00000271272	0	0	0	0	0	0	0	0	0	0
ENSG00000271271	0	0	0	0	0	0	0	0	0	0
ENSG00000271270	14	8	9	9	6	32	25	9	0	0
ENSG00000271269	0	0	0	0	0	0	0	0	0	0
ENSG00000271268	0	0	0	0	0	0	1	0	0	0
ENSG00000271267	1	0	2	0	0	2	2	0	0	0
ENSG00000271266	0	0	0	0	0	0	0	0	0	0
ENSG00000271265	0	0	0	0	0	0	1	0	0	0
ENSG00000271264	0	0	0	0	0	0	0	0	0	0
ENSG00000271259	0	0	3	0	0	0	7	4	0	0
ENSG00000271257	0	0	0	0	0	0	0	0	0	0
ENSG00000271255	0	0	0	0	0	0	0	0	0	0
ENSG00000271253	0	0	0	0	0	0	0	0	0	0
ENSG00000271252	1	0	0	1	0	3	0	0	0	0
ENSG00000271251	0	0	0	0	0	0	0	0	0	0
ENSG00000271250	0	0	0	0	0	0	0	0	0	0
ENSG00000271249	0	0	0	0	0	0	0	0	0	0
ENSG00000271248	0	0	0	0	0	0	0	0	0	0
ENSG00000271245	0	0	0	0	0	0	0	0	0	0
ENSG00000271243	0	0	0	0	0	0	0	8	0	0
ENSG00000271242	0	0	0	0	0	0	0	0	0	0
ENSG00000271240	0	0	0	1	0	0	0	0	0	0
ENSG00000271239	1	0	0	0	0	0	0	0	0	0
ENSG00000271238	0	0	0	0	0	0	0	0	0	0
ENSG00000271237	0	0	0	0	0	0	0	0	0	0
ENSG00000271236	0	0	0	0	0	0	0	0	0	0
ENSG00000271235	0	0	0	0	0	0	1	0	0	0
ENSG00000271234	0	0	0	0	0	0	0	0	0	0
ENSG00000271232	0	0	0	0	0	0	0	2	0	0
ENSG00000271231	0	0	0	0	0	0	0	0	0	0
ENSG00000271230	0	0	0	0	0	0	0	0	0	0
ENSG00000271228	2	1	2	0	0	0	0	3	0	0
ENSG00000271227	0	0	0	0	0	0	0	3	0	0
ENSG00000271225	4	3	5	5	2	11	3	0	0	0
ENSG00000271222	0	0	0	0	0	0	0	0	0	0
ENSG00000271220	0	0	0	0	0	0	0	0	0	0
ENSG00000271219	0	0	0	0	0	0	0	4	0	0
ENSG00000271218	1	1	7	2	1	0	3	6	0	0
ENSG00000271216	0	0	0	0	0	0	12	0	0	0
ENSG00000271215	0	0	0	0	0	0	2	9	0	0
ENSG00000271214	0	0	0	0	0	0	0	0	0	0
ENSG00000271211	0	0	0	0	0	0	0	0	0	0
ENSG00000271209	0	0	0	0	0	0	0	0	0	0
ENSG00000271208	0	0	0	0	0	0	0	9	0	0
ENSG00000271207	0	0	1	0	0	0	0	0	0	0

ENSG00000271205	0	0	0	0	0	0	0	0	0	0
ENSG00000271204	0	0	0	0	0	0	53	12	0	5
ENSG00000271202	0	0	0	0	0	0	0	0	0	0
ENSG00000271201	0	0	0	0	0	0	0	0	0	0
ENSG00000271200	0	0	0	0	0	0	13	0	0	0
ENSG00000271199	0	0	0	0	0	0	0	0	0	0
ENSG00000271198	0	0	0	1	0	0	1	0	0	0
ENSG00000271197	0	0	0	0	0	0	0	0	0	0
ENSG00000271196	0	0	0	0	0	0	0	0	0	0
ENSG00000271195	0	0	0	0	0	0	0	0	0	0
ENSG00000271194	0	0	0	0	0	0	0	0	0	0
ENSG00000271192	1	3	2	3	1	9	0	0	0	0
ENSG00000271190	0	0	0	0	0	0	0	0	0	0
ENSG00000271187	0	0	0	0	0	0	0	0	0	0
ENSG00000271185	0	0	0	0	0	0	1	0	0	0
ENSG00000271184	0	0	0	0	0	0	0	0	0	0
ENSG00000271182	0	0	0	0	0	0	0	0	0	0
ENSG00000271181	0	0	0	0	0	0	2	0	0	0
ENSG00000271180	0	0	0	0	0	0	0	0	0	0
ENSG00000271179	0	0	0	0	0	0	0	0	0	0
ENSG00000271178	0	0	0	0	0	0	0	3	0	0
ENSG00000271177	0	0	0	0	0	0	0	4	0	0
ENSG00000271175	0	0	0	0	0	0	0	0	0	0
ENSG00000271174	0	0	0	0	0	0	0	0	0	0
ENSG00000271173	0	0	0	0	0	0	0	0	0	0
ENSG00000271172	0	0	0	0	0	0	0	3	0	0
ENSG00000271171	0	0	0	0	0	0	0	0	0	0
ENSG00000271167	0	0	0	0	0	0	0	0	0	0
ENSG00000271166	0	0	0	0	0	0	0	0	0	0
ENSG00000271164	0	0	0	0	0	0	0	3	0	0
ENSG00000271163	0	0	0	0	0	0	1	0	0	0
ENSG00000271162	0	0	0	0	0	0	0	0	0	0
ENSG00000271161	1	2	5	1	3	5	0	0	0	0
ENSG00000271159	0	0	0	0	0	0	2	0	0	0
ENSG00000271158	0	0	0	0	0	0	0	0	0	0
ENSG00000271157	0	0	0	0	0	0	0	0	0	0
ENSG00000271156	0	0	0	0	0	2	0	0	0	0
ENSG00000271155	0	3	3	0	1	1	3	0	0	0
ENSG00000271154	0	0	0	0	0	0	0	0	0	0
ENSG00000271153	2	4	18	7	9	21	16	2	0	0
ENSG00000271151	3	0	0	2	1	0	5	1	0	3
ENSG00000271150	0	0	0	0	0	0	0	0	0	0
ENSG00000271149	0	0	0	0	0	0	0	0	0	0
ENSG00000271148	0	0	0	0	0	0	0	0	0	0
ENSG00000271147	21	15	23	20	15	49	58	36	0	9
ENSG00000271146	0	0	0	0	0	0	2	0	0	0
ENSG00000271143	0	0	0	0	0	0	0	0	0	0
ENSG00000271142	0	0	0	0	0	0	0	0	0	0
ENSG00000271141	0	0	0	0	0	0	0	0	0	0
ENSG00000271140	0	0	0	0	0	0	0	0	0	0
ENSG00000271138	0	0	0	0	0	0	0	0	0	0
ENSG00000271137	0	0	0	0	0	0	1	0	0	0
ENSG00000271136	0	0	0	0	0	0	0	0	0	0
ENSG00000271134	9	13	6	7	9	38	0	0	0	0
ENSG00000271133	10	5	7	4	4	19	38	38	0	4
ENSG00000271131	0	0	0	0	0	0	0	0	0	0
ENSG00000271130	0	0	0	0	0	0	0	0	0	0

ENSG00000271129	0	0	0	0	0	0	0	0	0	0
ENSG00000271128	0	0	0	0	0	0	1	0	0	0
ENSG00000271127	1	2	1	0	0	0	5	6	0	0
ENSG00000271123	0	0	0	0	0	0	0	0	0	0
ENSG00000271122	105	121	173	81	106	216	45	103	2	0
ENSG00000271121	42	38	74	19	29	102	59	27	0	0
ENSG00000271119	11	7	10	4	6	22	7	6	0	0
ENSG00000271118	0	0	0	0	0	0	1	0	0	0
ENSG00000271117	0	0	0	0	0	0	0	0	0	0
ENSG00000271115	0	0	0	0	0	0	0	0	0	0
ENSG00000271114	0	0	0	0	0	0	0	0	0	0
ENSG00000271113	0	0	0	0	0	0	0	0	0	0
ENSG00000271111	0	0	0	0	0	0	0	10	0	0
ENSG00000271109	3	3	9	4	1	5	19	36	0	0
ENSG00000271108	0	0	0	0	0	0	0	0	0	0
ENSG00000271105	0	2	0	2	0	0	1	0	0	0
ENSG00000271101	0	0	0	0	0	0	1	0	0	0
ENSG00000271100	0	0	0	0	0	0	0	0	0	0
ENSG00000271099	0	0	0	0	0	0	0	0	0	0
ENSG00000271098	0	0	0	0	0	0	2	0	0	0
ENSG00000271097	0	0	0	0	0	0	0	0	0	0
ENSG00000271096	0	0	0	0	0	0	2	0	0	0
ENSG00000271095	0	0	0	0	0	0	3	0	0	0
ENSG00000271094	0	0	0	0	0	0	0	0	0	0
ENSG00000271093	0	0	0	0	0	0	0	3	0	0
ENSG00000271092	0	0	0	1	0	0	0	0	0	0
ENSG00000271088	0	0	0	0	0	0	0	0	0	0
ENSG00000271086	0	0	0	0	0	0	4	8	0	0
ENSG00000271081	0	0	0	0	0	4	0	0	0	0
ENSG00000271079	55	61	80	44	51	126	101	32	0	0
ENSG00000271078	0	0	0	0	0	0	0	11	0	0
ENSG00000271077	0	0	0	0	0	0	0	0	0	0
ENSG00000271075	0	0	0	1	0	0	16	0	0	0
ENSG00000271074	0	0	0	0	0	0	0	0	0	0
ENSG00000271072	0	2	0	0	0	0	0	0	0	0
ENSG00000271071	1	0	0	0	0	0	0	0	4	0
ENSG00000271070	0	0	0	0	0	0	0	0	0	0
ENSG00000271065	0	0	0	0	0	0	3	0	0	0
ENSG00000271064	2	2	4	1	2	0	8	0	0	0
ENSG00000271063	0	0	0	0	0	0	0	0	0	0
ENSG00000271057	0	0	0	0	0	0	2	0	0	0
ENSG00000271056	0	0	0	0	0	0	0	0	0	0
ENSG00000271053	0	0	0	0	0	0	0	0	0	0
ENSG00000271052	0	0	0	0	0	0	0	2	0	0
ENSG00000271049	0	0	0	0	0	0	0	3	0	0
ENSG00000271048	0	0	0	0	0	0	0	0	0	0
ENSG00000271047	0	0	0	0	0	0	0	0	0	0
ENSG00000271046	0	0	2	1	0	0	1	3	0	0
ENSG00000271045	0	0	0	0	0	0	0	0	0	0
ENSG00000271044	0	0	0	0	0	0	0	0	0	0
ENSG00000271043	0	0	0	0	0	0	0	0	0	0
ENSG00000271042	0	0	0	0	0	0	0	4	0	0
ENSG00000271041	0	0	0	0	0	0	0	0	0	0
ENSG00000271040	1	2	3	0	2	13	5	0	0	0
ENSG00000271036	0	0	0	0	0	0	0	0	0	0
ENSG00000271035	0	0	0	0	0	0	0	0	0	0
ENSG00000271034	0	0	0	0	0	0	0	9	0	0

ENSG00000271032	3	4	8	1	3	14	20	29	0	0
ENSG00000271029	0	0	0	0	0	0	0	6	0	0
ENSG00000271028	0	0	0	0	0	0	0	0	0	0
ENSG00000271027	0	0	0	0	0	0	0	5	0	0
ENSG00000271026	0	0	0	0	0	0	1	5	0	0
ENSG00000271025	0	0	0	0	0	0	0	0	0	0
ENSG00000271024	0	0	0	0	0	0	0	0	0	0
ENSG00000271022	0	0	0	0	0	0	0	0	0	0
ENSG00000271021	0	0	3	0	0	1	0	0	0	0
ENSG00000271020	11	18	48	11	19	49	3	10	0	0
ENSG00000271018	0	0	0	0	0	0	0	0	0	0
ENSG00000271015	0	0	0	0	0	0	0	0	0	0
ENSG00000271014	0	0	0	0	0	0	0	15	0	0
ENSG00000271013	1	0	2	0	0	0	0	0	5	0
ENSG00000271011	0	0	0	0	0	0	0	0	0	0
ENSG00000271010	0	0	4	0	0	0	0	3	0	0
ENSG00000271009	0	1	0	0	0	0	6	31	0	0
ENSG00000271005	0	0	0	0	0	0	0	0	0	0
ENSG00000271003	0	0	0	0	0	0	0	0	0	0
ENSG00000271002	0	0	0	0	1	0	0	0	0	0
ENSG00000270999	0	0	0	0	0	0	5	4	0	0
ENSG00000270997	0	0	0	0	0	0	0	0	0	0
ENSG00000270996	0	0	0	0	0	0	0	0	0	0
ENSG00000270995	0	0	0	0	0	0	5	0	0	0
ENSG00000270994	0	0	0	0	0	0	0	0	0	0
ENSG00000270993	0	1	0	1	1	2	2	0	0	0
ENSG00000270992	0	0	0	0	0	0	0	0	0	0
ENSG00000270990	0	0	0	0	0	0	1	0	0	0
ENSG00000270989	0	0	0	0	0	0	0	0	0	0
ENSG00000270988	0	0	0	0	0	0	0	0	0	0
ENSG00000270987	0	0	0	1	0	1	3	0	0	0
ENSG00000270986	0	0	0	0	0	0	5	0	0	0
ENSG00000270983	0	0	0	0	0	0	1	0	0	0
ENSG00000270982	0	0	0	0	0	0	0	0	0	0
ENSG00000270981	0	0	0	0	0	0	1	0	0	0
ENSG00000270980	0	0	0	0	0	0	1	0	0	0
ENSG00000270978	0	0	0	0	0	0	3	0	0	0
ENSG00000270977	0	0	0	1	0	1	4	0	0	0
ENSG00000270976	0	0	0	0	0	0	0	0	0	0
ENSG00000270975	0	3	1	0	2	1	0	0	0	0
ENSG00000270973	0	0	0	0	0	0	0	0	0	0
ENSG00000270972	0	0	0	0	0	0	2	17	2	17
ENSG00000270971	0	0	0	0	0	0	0	0	0	0
ENSG00000270969	0	0	0	0	0	0	0	0	0	0
ENSG00000270966	0	0	0	0	0	0	0	6	0	0
ENSG00000270965	0	0	0	0	0	0	0	3	0	0
ENSG00000270964	7	6	14	3	5	13	2	5	0	0
ENSG00000270962	0	0	1	0	0	1	4	4	0	0
ENSG00000270961	0	0	0	0	0	0	0	0	0	0
ENSG00000270960	0	0	0	0	0	0	0	0	0	0
ENSG00000270959	5	11	7	3	2	10	22	61	0	0
ENSG00000270957	0	0	0	0	0	0	0	0	0	0
ENSG00000270956	7	6	2	3	0	13	0	3	0	0
ENSG00000270955	0	0	0	0	0	0	0	11	0	0
ENSG00000270954	0	0	0	0	0	0	0	0	0	0
ENSG00000270953	0	0	2	2	0	2	3	6	0	0
ENSG00000270951	0	2	0	0	2	3	2	0	0	0

ENSG00000270949	19	43	50	27	35	59	150	50	1	0
ENSG00000270948	1	1	0	0	1	0	0	0	0	0
ENSG00000270947	0	0	0	0	0	0	0	0	0	0
ENSG00000270946	0	0	0	0	0	0	0	0	0	0
ENSG00000270945	0	0	0	0	0	0	0	0	0	0
ENSG00000270941	0	0	0	0	0	0	0	0	0	0
ENSG00000270938	0	0	0	0	0	0	1	0	0	0
ENSG00000270937	0	0	0	0	0	0	0	0	0	0
ENSG00000270936	0	0	0	0	0	0	0	0	0	0
ENSG00000270935	0	0	0	0	0	0	0	0	0	0
ENSG00000270934	0	0	0	0	0	0	0	0	0	0
ENSG00000270933	0	1	2	2	0	1	12	3	0	0
ENSG00000270930	0	0	0	0	0	0	0	0	0	0
ENSG00000270929	0	0	0	0	0	0	0	0	0	0
ENSG00000270927	0	0	0	0	0	0	0	0	0	0
ENSG00000270926	0	0	0	0	0	0	0	0	0	0
ENSG00000270924	0	0	0	0	0	0	0	0	0	0
ENSG00000270923	0	2	3	0	2	4	3	0	0	0
ENSG00000270921	0	11	11	1	3	6	1	0	0	0
ENSG00000270920	0	0	0	0	0	0	0	0	0	0
ENSG00000270919	0	0	2	1	0	2	0	2	0	0
ENSG00000270917	1	1	1	2	2	7	4	7	0	0
ENSG00000270916	0	1	5	0	0	0	0	5	0	1
ENSG00000270915	0	0	0	0	0	0	3	0	0	0
ENSG00000270914	0	0	0	0	0	0	0	0	0	0
ENSG00000270912	0	0	0	0	0	0	0	0	0	0
ENSG00000270911	0	0	0	0	0	0	2	4	0	0
ENSG00000270909	0	0	0	0	0	0	0	10	0	8
ENSG00000270906	0	0	0	0	0	0	0	77	1	2
ENSG00000270904	0	0	0	0	0	0	1	0	0	0
ENSG00000270903	7	18	8	10	7	26	10	0	0	0
ENSG00000270902	0	0	0	0	0	0	0	0	0	0
ENSG00000270900	0	0	0	0	0	0	0	0	0	0
ENSG00000270898	0	0	0	0	0	2	3	0	0	0
ENSG00000270897	0	0	0	0	0	0	0	0	0	0
ENSG00000270896	2	0	0	0	0	6	1	3	0	0
ENSG00000270894	0	0	0	0	1	1	0	0	0	0
ENSG00000270893	0	0	0	0	0	0	0	0	0	0
ENSG00000270892	0	0	0	0	0	0	0	0	0	0
ENSG00000270891	0	0	0	0	0	0	0	0	0	0
ENSG00000270890	9	7	8	7	3	16	19	0	0	0
ENSG00000270889	0	0	0	0	0	0	0	0	0	0
ENSG00000270885	5	2	3	1	0	6	19	27	0	0
ENSG00000270883	0	0	0	0	0	0	1	0	0	0
ENSG00000270882	197	231	458	181	189	508	289	94	0	21
ENSG00000270880	0	0	0	0	0	0	3	0	0	0
ENSG00000270879	0	0	2	0	2	3	1	2	0	0
ENSG00000270878	2	0	7	3	3	1	12	2	0	0
ENSG00000270876	1	0	0	1	0	0	0	0	0	0
ENSG00000270874	0	0	0	0	0	0	0	0	0	0
ENSG00000270872	248	222	387	189	218	585	183	14	0	0
ENSG00000270871	13	15	17	13	10	37	5	2	0	0
ENSG00000270870	0	0	0	0	0	0	0	0	0	0
ENSG00000270868	0	0	0	0	0	0	1	0	0	0
ENSG00000270866	0	0	0	0	0	0	0	0	0	0
ENSG00000270865	0	0	0	0	0	0	0	0	0	0
ENSG00000270864	0	0	0	0	0	0	0	0	0	0

ENSG00000270863	0	0	0	0	0	0	1	0	0	0
ENSG00000270861	0	0	0	0	0	0	0	0	0	0
ENSG00000270859	0	0	0	0	0	0	0	0	0	0
ENSG00000270858	1	0	0	0	0	0	0	0	0	0
ENSG00000270857	0	0	0	0	0	0	2	0	0	0
ENSG00000270856	0	0	0	0	0	0	0	0	0	0
ENSG00000270855	0	0	0	0	0	0	0	0	0	2
ENSG00000270852	0	0	0	0	0	0	0	0	0	0
ENSG00000270850	0	0	0	0	0	0	0	0	0	0
ENSG00000270849	0	0	0	0	0	0	0	0	0	0
ENSG00000270846	0	0	0	0	0	4	4	0	0	0
ENSG00000270842	0	0	0	0	0	0	1	0	0	0
ENSG00000270839	0	0	2	0	0	0	0	0	0	0
ENSG00000270838	0	0	0	0	0	0	0	0	0	0
ENSG00000270837	0	0	1	0	0	0	0	0	0	0
ENSG00000270835	0	0	0	0	0	0	0	0	0	0
ENSG00000270832	0	0	0	0	0	0	12	0	0	0
ENSG00000270831	0	0	0	0	0	0	0	8	0	0
ENSG00000270830	0	0	0	0	0	0	0	0	0	0
ENSG00000270829	1	0	0	0	0	0	0	0	0	0
ENSG00000270828	0	0	0	0	0	0	0	0	0	0
ENSG00000270826	0	0	0	0	0	0	0	0	0	0
ENSG00000270824	0	0	0	0	0	0	0	0	0	0
ENSG00000270823	1	0	0	0	0	0	1	18	0	0
ENSG00000270822	0	0	0	0	1	0	1	0	0	0
ENSG00000270820	2	3	8	3	4	5	28	27	1	0
ENSG00000270818	0	0	0	0	0	0	0	3	0	0
ENSG00000270816	0	0	0	0	0	0	0	0	0	0
ENSG00000270815	0	0	0	0	0	0	0	0	0	0
ENSG00000270814	0	0	0	0	0	0	0	0	0	0
ENSG00000270813	0	2	0	3	0	0	1	3	0	0
ENSG00000270812	0	0	0	0	0	0	0	0	0	0
ENSG00000270811	0	0	0	0	0	0	0	1	0	0
ENSG00000270810	6	5	10	6	3	16	7	7	0	0
ENSG00000270809	1	0	0	0	0	0	1	1	0	0
ENSG00000270808	2	4	0	1	0	6	0	0	0	0
ENSG00000270807	0	0	0	0	0	0	0	0	0	0
ENSG00000270806	0	2	3	1	0	2	0	0	0	0
ENSG00000270804	26	34	32	29	31	91	35	5	1	0
ENSG00000270802	0	0	0	1	0	1	7	7	0	0
ENSG00000270800	0	0	0	0	0	0	0	0	0	0
ENSG00000270799	0	0	0	0	0	0	0	0	0	0
ENSG00000270798	0	0	0	0	0	0	0	0	0	0
ENSG00000270797	0	0	0	0	0	0	0	0	0	0
ENSG00000270794	0	0	0	0	0	0	0	0	0	0
ENSG00000270793	0	0	0	0	0	0	0	0	0	0
ENSG00000270792	0	0	1	3	0	2	0	0	0	0
ENSG00000270791	0	0	0	0	0	0	0	3	0	0
ENSG00000270790	0	0	0	0	0	0	0	0	0	0
ENSG00000270789	0	0	0	1	0	0	0	0	0	0
ENSG00000270788	0	0	0	0	0	0	0	0	0	0
ENSG00000270783	0	0	0	0	0	0	0	0	0	0
ENSG00000270782	0	0	0	0	0	0	0	0	0	0
ENSG00000270781	0	0	0	0	1	0	1	0	0	0
ENSG00000270780	0	0	0	0	0	0	0	0	0	0
[truncated: 467,095 more chars]
